# Multiomics-Based Signaling Pathway Network Alterations in Human Non-functional Pituitary Adenomas

**DOI:** 10.3389/fendo.2019.00835

**Published:** 2019-12-17

**Authors:** Ying Long, Miaolong Lu, Tingting Cheng, Xiaohan Zhan, Xianquan Zhan

**Affiliations:** ^1^Key Laboratory of Cancer Proteomics of Chinese Ministry of Health, Xiangya Hospital, Central South University, Changsha, China; ^2^Hunan Engineering Laboratory for Structural Biology and Drug Design, Xiangya Hospital, Central South University, Changsha, China; ^3^State Local Joint Engineering Laboratory for Anticancer Drugs, Xiangya Hospital, Central South University, Changsha, China; ^4^Department of Oncology, Xiangya Hospital, Central South University, Changsha, China; ^5^National Research Center for Geriatric Disorders, Xiangya Hospital, Central South University, Changsha, China

**Keywords:** non-functional pituitary adenoma, integrative omics data, PTMScan, immunoaffinity, signaling pathways, molecular networks, biomarkers

## Abstract

Non-functional pituitary adenoma (NFPA) seriously affects hypothanamus-pituitary-target organ axis system, with a series of molecule alterations in the multiple levels of genome, transcriptome, proteome, and post-translational modifications, and those molecules mutually interact in a molecular-network system. Meta analysis coupled with IPA pathway-network program was used to comprehensively analyze nine sets of documented NFPA omics data, including NFPA quantitative transcriptomics data [280 differentially expressed genes (DEGs)], NFPA quantitative proteomics data [50 differentially expressed proteins (DEPs)], NFPA mapping protein data (218 proteins), NFPA mapping protein nitration data (9 nitroproteins and 3 non-nitrated proteins), invasive NFPA quantitative transriptomics data (346 DEGs), invasive NFPA quantitative proteomics data (57 DEPs), control mapping protein data (1469 proteins), control mapping protein nitration data (8 nitroproteins), and control mapping phosphorylation data (28 phosphoproteins). A total of 62 molecular-networks with 861 hub-molecules and 519 canonical-pathways including 54 cancer-related canonical pathways were revealed. A total of 42 hub-molecule panels and 9 canonical-pathway panels were identified to significantly associate with tumorigenesis. Four important molecular-network systems, including PI3K/AKT, mTOR, Wnt, and ERK/MAPK pathway-systems, were confirmed in NFPAs by PTMScan experiments with altered expression-patterns and phosphorylations. Nineteen high-frequency hub-molecules were also validated in NFPAs with PTMScan experiment with at least 2.5-fold changes in expression or phosphorylation, including ERK, ERK1/2, Jnk, MAPK, Mek, p38 MAPK, AKT, PI3K complex, p85, PKC, FAK, Rac, Shc, HSP90, NFκB Complex, histone H3, AP1, calmodulin, and PLC. Furthermore, mTOR and Wnt pathway-systems were confirmed in NFPAs by immunoaffinity Western blot analysis, with significantly decreased expression of PRAS40 and increased phosphorylation levels of p-PRAS40 (Thr246) in mTOR pathway in NFPAs compared to controls, and with the decreased protein expressions of GSK-3β and GSK-3β, significantly increased phosphorylation levels of p-GSK3α (Ser21) and p-GSK3β (Ser9), and increased expression level of β-catenin in Wnt pathway in NFPAs compared to controls. Those findings provided a comphrensive and large-scale pathway network data for NFPAs, and offer the scientific evidence for insights into the accurate molecular mechanisms of NFPA and discovery of the effective biomarkers for diagnosis, prognosis, and determination of therapeutic targets.

## Introduction

Human non-functional pituitary adenoma (NFPA) is a common disease that occurs in the central regulatory organ pituitary, and seriously impacts on physiological functions and human health (1). Compared to functional pituitary adenomas (FPAs), NFPA has a very challenging clinical problem in early diagnosis and treatment because of the lack of the corresponding hormone elevation in NFPA patients (2, 3). NFPA is a complex whole-body disease that alters in the levels of gene (genome), RNA (transcriptome), protein (proteome), and metabolite (metabolome), and that involves multi-factors, multi-processes, and multi-consequences (4–6). Individual variations are involved in prediction/prevention, early-stage diagnosis/therapy, and late-stage diagnosis/therapy. Moreover, omics (genomics, transcriptomics, proteomics, peptidomics, metabolomics, and radiomics) and systems biology are promoting one to change paradigms from traditional single-factor strategy to multi-parameter systematic strategy in pituitary adenoma studies and clinical practice (4, 7), in the model of predictive screening and prognostic assessment, which traditionally only depended on the changes of serum single-hormone change and pituitary imaging, and in the therapeutic model of cancer from the general radiotherapy and chemotherapy to personalized strategy (8, 9). From multi-parameter systematic strategy opinion, it is necessary to systematically study the changes in genome, transcriptome, proteome, peptidome, and metabolome in individual pituitary adenoma tissue and body-fluid (cerebrospinal fluid, CSF; serum/plasma) (7). Systems biological technologies are able to integrate all experimental data and clinical information of individuals to identify key molecular networks specific to individual NFPA (10, 11). However, the data from genome, transcriptome, proteome, peptidome, metabolome, and radiome are much different among individual tumors, and between tumors and normals; and molecular networks alter among individuals, and between tumors and normals. Therefore, it is necessary to construct multiple omics data-based molecular networks for clarification of accurate molecular mechanisms of NFPAs, and discovery of tumor-specific biomarker pattern for efficient prediction screening, early diagnosis, prognostic assessment, and individualized prevention and therapy (12).

Molecular-network alterations are the hallmark in complex cancer disease (4, 7, 12). The molecules in the levels of gene (genome), RNA (transcriptome), protein (proteome), and metabolite (metabolome) are mutually regulated and form dynamically associated systems. Each molecule change is associated with the changes of other molecules in a pathway system. One molecule in a signaling pathway system might also trigger the effects of other signaling pathways in a tumor biological system. Thus, if only a single-one molecule is targeted or only a single-level of omics studies is focused on, then it must result in obvious limitations. A globally systematic and comprehensive recognition of molecular networks based on multi-omics data has an important scientific merit to understand the molecular mechanisms of NFPAs and discover really useful biomarkers for NFPAs. However, it is difficulty for a single research team to perform all studies in each level of genome, transcriptome, proteome, and metabolome commonly, due to the limited experimental conditions, expertise, and financial support in a single research team. The single and independent experimental data from different omics studies under a given condition can only explain and represent a certain aspects of a tumor because of tumor heterogeneity and plasticity (13, 14). The experimental subjects are also not same among different research groups. Thus, the experimental results from different research group have their own strengths and limitations. The concept and principle of Meta analysis (15), which is a secondary analysis based on multiple center published experimental data, offers one a new strategy to integrate and analyze different levels of NFPA omics data that were published by different research groups. Moreover, Ingenuity Pathway Analysis (IPA) system is an extensively used and classic pathway analysis system to construct pathway networks with different omics data from large-scale IPA knowledge base database (>6 million scientific findings and >800 canonical pathways) (16, 17). Meta analysis in combination with IPA pathway network analysis can construct integrative molecular networks to in-depth understand NFPA pathogenesis and discover accurate and reliable biomarkers for NFPAs (5).

**Figure 1:**
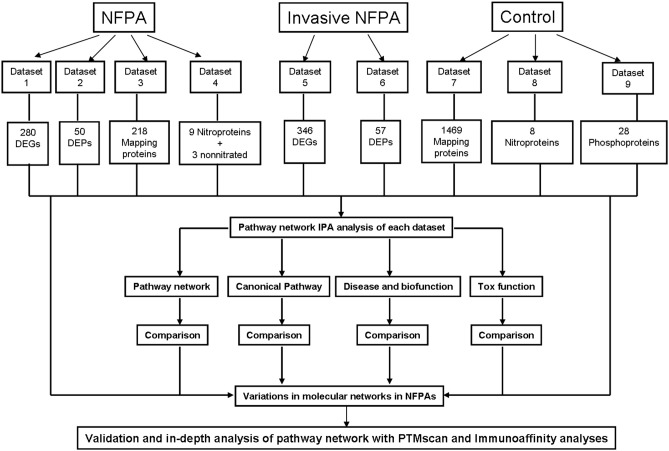
Scheme to construct molecular networks based on an integrative analysis of the documented omics data in NFPAs. DEG, differentially expressed gene; DEP, differentially expressed protein.

The present study collected all published omics data about NFPAs (Supplemental Table 1), including mapping protein data in NFPAs and controls, quantitatively transcriptomic data and proteomic data between NFPAs and controls, mapping protein-nitration data in NFPAs and controls, mapping protein-phosphorylation data in controls, and quantitatively transcriptomic data and proteomic data between invasive and non-invasive NFPAs. IPA pathway analysis program (17) was used to reveal signaling networks, canonical pathways, and biological functions with each set of omics data. The important signaling pathways and the corresponding molecules were confirmed with PTMScan experiments and immunoaffinity Western blot in the real NFPA samples compared to control samples. Then, these data were comprehensively analyzed to reveal integrative molecular networks that function in an NFPA biological system. The experimental flow chart was shown to construct and validate pathway-network systems in NFPAs (Figure 1).

## Materials and Methods

### Omics Datasets

All documented omics data regarding NFPAs were collected from Pubmed and Google Scholar databases. Those omics data from NFPAs were classified into nine datasets (Figure 1 and Supplemental Table 1): (i) *Dataset 1*—NFPA quantitative transcriptomics data, including 280 differentially expressed genes (DEGs) (114 upregulated and 166 downregulated) (Supplemental Materials 1.1). (ii) *Dataset 2*—NFPA quantitative proteomics data: including 50 differentially expressed proteins (DEPs) (21 upregulated and 29 downregulated) (Supplemental Materials 2.1). (iii) *Dataset 3*—NFPA mapping protein data, including 218 proteins (Supplemental Materials 3.1). (iv) *Dataset 4*—NFPA mapping protein nitration data, including 9 nitroproteins and 3 non-nitrated proteins (Supplemental Materials 4.1). (v) *Dataset 5*—Invasive NFPA quantitative transriptomics data, including 346 DEGs (233 upregulated and 113 downregulated) (Supplemental Materials 5.1). (vi) *Dataset 6*—Invasive NFPA quantitative proteomics data, including 57 DEPs (30 upregulated and 27 downregulated) (Supplemental Materials 6.1). (vii) *Dataset 7*—Pituitary control mapping protein data, including 1,469 proteins (Supplemental Materials 7.1). (viii) *Dataset 8*—Pituitary control mapping protein nitration data, including 8 nitroproteins (Supplemental Materials 8.1). (ix) *Dataset 9*—Pituitary control mapping phosphorylation data, including 28 phosphoproteins (Supplemental Materials 9.1).

### IPA Analysis

Each dataset was analyzed with IPA analysis program (http://www.ingenuity.com). Briefly, the ID numbers of genes and proteins in each dataset were used as the identifiers, and input into the IPA analysis program with the Core analysis platform. For DEG and DEP data, the ID numbers and corresponding fold-change values were input simultaneously into the IPA analysis system to automatically search the matched genes/molecules, and generate a two-dimensional table that includes the matched and unmatched genes/proteins. Five subdatasets were automatically generated, including (i) All IDs that contained all input IDs, (ii) Unmapped IDs that were without the matched molecules in the IPA system, which did not enter the next-step pathway analysis, (iii) Mapped IDs that were the matched molecules with duplicated IDs, (iv) Network-eligible IDs that were the mapped IDs without duplicated IDs, and (v) Functions/Pathways**/**List-eligible IDs. For the duplicated IDs for the same gene/protein, the identifier with the highest fold-change was used in the pathway analysis; or, the first appeared gene/protein was used in the pathway analysis without an expression value such as mapping proteomic data, nitroprotein data, and phosphorylation data. The Network-eligible IDs were proceeded into the pathway network analysis with comparison of network-eligible molecules (genes; proteins) with the IPA knowledge base (IPAKB); and IPAKB contains over 6 million scientific findings and over 800 canonical pathways (2, 17). The significances (*p*-values) of the associations between the dataset and the canonical pathways in the IPAKB were measured with comparison of the number of use-specific genes/proteins of interest that participate in a given pathway to the total number of occurrences of these genes in all pathway annotations that are stored in the IPAKB. The Benjamini-Hochberg for multiple testing was used to calculate each *p*-value to determine the probability that the association between genes in the dataset and the canonical pathways in IPAKB was explained only by chance, with a statistical significance of *p* < 0.05. Each IPA analysis generated statistically significant networks, canonical pathways, biofunctions, and tox functions. A toxic pathway is defined as a canonical pathway that is significantly associated with toxicity lists that describe adaptive, defensive, or reparative responses to xenobiotic insult, and could be used to understand biological responses.

### Analysis of Molecular Networks

All IPA data (networks, canonical pathways, biofunctions, and tox functions) from different datasets together with the original gene/protein data were comprehensively analyzed in combination with literature-based bioinformatics and clinical features, to clarify molecular pathway-network alterations in NFPAs. Those common networks, canonical pathways, biofunctions, and tox functions derived from multiple datasets were important molecular events that occurred in NFPAs. Moreover, an important role of network is to find hub-molecules. All of those hub-molecules with at least five linked molecules among those networks identified from nine datasets were further analyzed to find hub-molecule panels. Each hub-molecule panel was further rationalized in NFPAs. Each canonical-pathway panel derived from nine datasets was also rationalized in NFPA biological processes.

### Pituitary Tumor and Control Tissues

Pituitary adenoma tissue samples were obtained from Department of Neurosurgery, Xiangya Hospital, Central South University, and were approved by Xiangya Hospital Medical Ethics Committee of Central South University. Control pituitary glands were post-mortem tissues obtained from the Memphis Regional Medical Center, and were approved by University of Tennessee Health Science Center Internal Review Board (UTHSC-IRB). The written informed consent was obtained from each patient or the family of control pituitary subject, after full explanation of the purpose and nature of all used procedures. The tissues were removed during neurosurgery or autopsy, frozen immediately in liquid nitrogen, and stored (−80°C) until processed.

### PTMScan Direct Multi-Pathway Analysis of Mined Signaling Pathways

Pituitary tissue samples from NFPA patients (*n* = 4) and control pituitaries (*n* = 4) (Supplemental Table 2-1) were analyzed with PTMScan® Direct Test (Cell Signaling Technology Company, Danvers, MA, USA) to experimentally investigate the roles of multiple pathways including PI3K/AKT, mTOR, Wnt, and ERK/MAPK signaling pathways derived from nine sets of omics data in NFPAs.

#### Tissue Lysate Preparation

An amount (100 mg) of pituitary tissue samples were added in a volume (1 ml) of urea lysis buffer (20 mM 2-hydroxyethyl (HEPES), 9 M urea, 2.5 mM sodium pyrophosphate, 1 mM sodium orthovanadate, and 1 mM β-glycerophosphate, pH 8.0), and homogenized with refiner on the ice. The lysates were sonicated (30 s x 3 times at 15 W output, chilled on ice with 1-min intervals), and centrifuged (20,000 g, 4°C, 15 min). The supernatant was collected, and its protein concentration was measured with Bio-Rad 2-D Quant assay using bovine serum albumin (BSA) as standard. Each sample was mixed with the equal protein amount in NFPA group and in control group, respectively.

#### Protein Digestion and Purification

Equal amount (10 mg/sample) of protein mixture (NFPAs; and controls) was reduced (55°C, and 30 min) with a final concentration of 4.5 mM dithiothreitol (DTT) in an incubator. After the solution was cooled on ice to room temperature, an appropriate volume (1 ml) of 100 mM iodoacetamide was added to 40 mg of protein extract, mixed well, and incubated (dark, 15 min, and room temperature). The reduced and alkalized samples were diluted (1:4) with 20 mM HEPES buffer at pH 8.0. The diluted samples were digested (overnight, room temperature, and gentle mixing) with 10 μg/ml trypsin-TPCK (TPCK = tosyl-phenylalanine chloromethyl-ketone) in 1 mM hydrochloric acid (HCl). After digestion, the tryptic peptides were acidified with 1% trifluoroacetic acid (TFA) to reach pH <3, and then stood on ice for 15 min to be precipitated. The acidified peptide solution was centrifuged (1,780 g, 15 min, and room temperature), followed by desalination through a C18 Sep-Pak cartridge (Waters) and elution by 40% acetonitrile in 0.1% TFA. The eluted peptides were lyophilized.

#### Immunoprecipitation Through PTMScan Direct Multi-Pathway Reagents and Purification

PTMScan Direct Multipathway V2.0 (18) antibody mixture was incubated (overnight; 4°C) with 30 μl protein G agarose beads (Roche). The beads with antibodies were washed four times with 1X phosphate buffered saline (PBS). Lyophilized peptides were resuspended in 1.4 ml 1X IAP buffer (50 mM MOPS, 50 mM sodium chloride, 10 mM sodium phosphate, pH 7.2), and centrifuged (10,000 g, 5 min, 4°C). The resupended peptides were added into the beads with PTMScan antibodies, and incubated (4°C, 2 h); and a mixture of tryptic peptides of various cell lysates was used as a positive control (18). After immunoaffinity reaction, the supernatant was removed, and beads with antibody-peptides were washed with 1 ml 1X IAP buffer for three times, then followed by wash with 1 ml high-performance liquid chromatography (HPLC)-grade water for three times. Enriched peptides were eluted (25°C, 10 min, and gentle mixing) from the beads with 50 μl 0.15% TFA, repeat the elution step, and all of the eluents were combined. The combined peptide solution was desalted through Stagetip by laying two layers of C18 Empore^TM^ materials into a 10-μl pipette tip (Cell Signaling Technology), passed with 50 μl 50% acetonitrile in 0.1% TFA (1,500 g, 2 min; 2 x), and followed by rinsing with 50 μl 0.1% TFA by centrifuging the tip (1,500 g, 1 min; 2 x). The peptides were eluted from the Stagetip through passing 10 μl 40% acetonitrile in 0.1% TFA and centrifuging (750 g, 1 min; 2 x). The eluted peptides were vacuum-dried.

#### LC-MS/MS

The PTMScan antibody-enriched peptides were resuspended in 12 μl of 0.125% formic acid for each sample, and separated through a reversed-phase C18 column (75 μm i.d. x 10 cm length) which packed into a PicoTip emitter (~8 μm i.d.) with a Magic C18 AQ (100 Å x 5 μm). Each sample was divided into two equal portions for liquid chromatography-tandem mass spectrometry (LC-MS/MS) analysis to increase the number of identifications and perform analytical reproducibility. Each sample was spiked with a standard peptide mixture [MassPREP^TM^ Protein Digestion Standard Mix 1; an overall quantity of 100 fmol (33 fmol per injection)] ahead of LC-MS/MS analysis on an Easy-nLC 1000 hyphened Q-Exactive^TM^ mass spectrometer. Peptides were separated by a linear gradient from 2 to 32% acetonitrile over 120 min. Both MS and MS/MS data were acquired in centroid mode. For precursor ion scan, resolution was set at 70,000 with an automatic gain control (AGC) target of 1 x 10^6^, and scan range was from *m/z* 300 to 1,500. For product ion scan, resolution was set at 17,500 with AGC target of 1 x 10^5^, and scan range was from *m/z* 200 to 2,000. The top 10 intensive precursor ions in each MS scan were selected for MS/MS analysis with normalized collision energy of 25.

#### Database Searching and Label-Free Quantification

SEQUEST and the core platform from Harvard University were used to evaluate MS/MS spectra. MS/MS data were used to search against Swiss-Prot *homo sapiens* FAST database (updated on April 29, 2015; 42,104 forward and 42,104 reverse sequences, and isoform messages). A mass accuracy of ±0.02 Da was used for product ions and ±5 ppm for precursor ions. Enzyme was selected as trypsin with at least one tryptic (K- or R-containing) terminus required and up to four miscleavages allowed per peptide. Carboxamidomethylation at cysteine residues was set as a fixed modification; and oxidation at methionine residues and the appropriate PTMs were set as variable modifications. All searches included reverse decoy database was used to value false discovery rates (FDR), and the linear discriminant module of core was screened with 5% FDR. Progenesis V4.1 (Waters Cooperation) and Skyline Version 3.1 (MacCoss Lab, University of Washington) were used to generate quantitative data and to extract the whole peal area of the corresponding peptide assignments. Extracted ion chromatograms of peptide ions with abundance variations between samples were manually assessed to make sure the accurate quantification in Skyline.

### West Blot Evaluation of Key Molecules in Signaling Pathways

Equal amount of tissue lysates (20 μg) were mixed (v:v = 1:1) with 2x loading buffer that mixed 50 μl β-mercaptoethanol (β-ME) and 950 μl Laemmli sample buffer (Bio-Rad, Cat#: 1610737), boiled (95–100°C, 5 min), and then chilled on the ice. The boiled protein samples were separated with 10% sodium dodecyl sulfate polyacrylamide gel electrophoresis (SDS-PAGE) (Bio-Rad, Cat#: 4561033), transferred to a polyvinylidene fluoride (PVDF) membrane (Merck Millipore, Cat#: INCP00010), and blocked (1 h, room temperature) with 5% BSA in Tris buffered saline (TBS) containing 0.1% Tween 20 (TBST). The blocked proteins on the PVDF membrane were incubated (4°C, overnight) with primary antibodies, washed in TBST, and incubated (2 h, room temperature) with anti-mouse or anti-rabbit HRP-conjugated secondary antibodies. Each primary or secondary antibody was prepared (Supplemental Table 2-2). The membranes were washed and developed with chemiluminescence reaction (SuperSignal™ West Pico Chemiluminescent Substrate, Thermo Fisher Scientific, Cat#: 34077; or Clarity Max Western ECL Substrate, Bio-Rad, Cat#: 1705062s). The digital images were acquired with a scanner (FLURCHEM FC3, ProteinSimple), and optical density (O.D.) values were quantified with a specific densitometric software (Quantity One, Bio-Rad). Each targeted protein was analyzed with Western blot for at least three times. Student *t*-test with *p* < 0.05 was used to determine statistically significant difference between NFPAs and controls.

### Statistical Analysis

For IPA analysis of multi-omics data, Benjamini-Hochberg for multiple testing with significance level of *p* < 0.05 was used to determine statistically significant molecular-networks, and canonical pathways. For PTMScan experiments, 5% FDR with reverse decoy database search using Progenesis V4.1 (Waters Cooperation) and Skyline Version 3.1 (MacCoss Lab, University of Washington) was used to quantitatively determine a reliable peptide, protein, and phosphorylation. For Western blot analysis, Student *t*-test with *p* < 0.05 and at least repetition three times were used to determine statistically significant difference in each protein or phosphorylation in NFPAs relative to controls.

## Results

### Data Characteristics of Nine Sets of Omics Datasets

Nine sets of omics data (Supplemental Materials 1.1, 2.1, 3.1, 4.1, 5.1, 6.1, 7.1, 8.1, 9.1) were input into the IPA, respectively. Each set of omics data was classified into unmatched, matched, duplicated, and network-eligible IDs (Supplemental Table 3). Only network-eligible IDs were processed into network analysis. Among them, there were two sets of DEG data (Datasets 1 and 5), two sets of DEP data (Datasets 2 and 6), and one set of nitroprotein data (Dataset 4), from NFPAs or invasive NFPAs (Figure 1, Supplemental Table 1). Analyses of those DEG, DEP, and nitroprotein data from NFPAs or invasive NFPAs directly resulted in clarification of molecular profiling variations in NFPAs relative to controls. Four sets of mapping data (proteins, nitroproteins, and phosphoproteins) from controls (Datasets 7, 8, and 9) or NFPAs (Dataset 3) provided the baseline data for NFPA molecular profiling variations. Moreover, the data characteristics of nine sets of omics data were summarized (Supplemental Table 4), of which four types of common molecules were present in NFPA datasets 1 (DEGs) and 2 (DEPs), and invasive NFPA datasets 5 (DEGs) and 6 (DEPs), including growth factors, kinases/enzymes, transcriptional regulators, and transporters.

### Molecular Network Alterations in NFPAs

**Figure 2:**
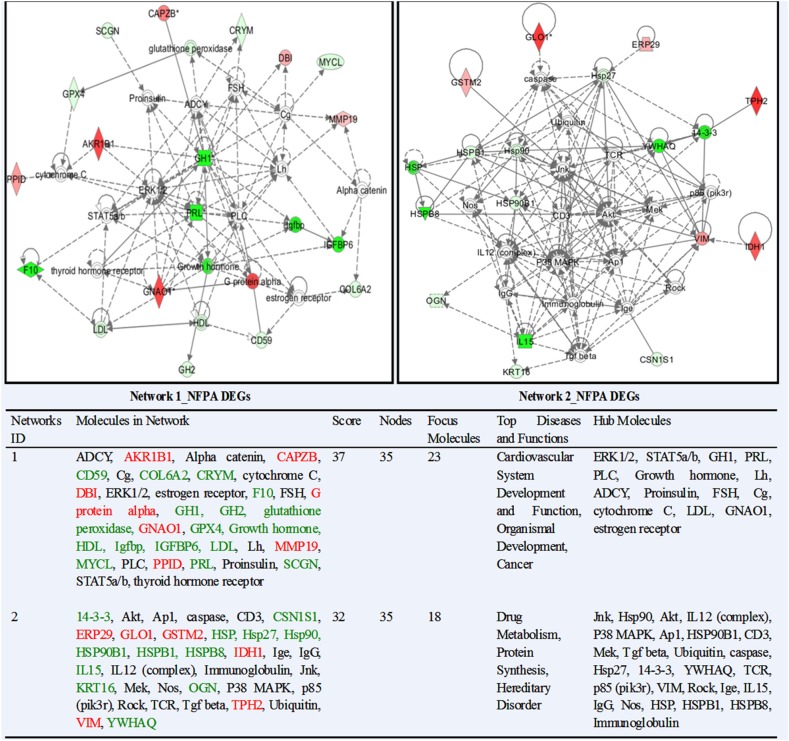
The representative molecular networks derived from NFPA DEG dataset.

IPA system compared and associated the network-eligible molecules (genes; proteins) in each omics dataset with the large-scale IPAKB to link molecules with direct or indirect relationship into a biological function group, and further generate the complicated interactive molecular-network diagrams. This study identified 62 statistically significant molecular-networks from nine sets of omics data, including 15 networks from NFPA DEGs (Supplemental Materials 1.2), 4 from NFPA DEPs (Supplemental Materials 2.2), 12 from NFPA mapping proteins (Supplemental Materials 3.2), 1 from NFPA nitroproteins (Supplemental Materials 4.2), 13 from invasive NFPA DEGs (Supplemental Materials 5.2), 3 from invasive NFPA DEPs (Supplemental Materials 6.2), 10 from control mapping proteins (Supplemental Materials 7.2), 1 from control nitroproteins (Supplemental Materials 8.2), and 3 from control phosphoproteins (Supplemental Materials 9.2). Each network performed the corresponding biological functions. The functions, nodes, molecules, and statistical score of each network were collected in Supplemental Materials 1.2–9.2. Two networks from NFPA DEP data (Dataset 2) were taken as representative examples (Figure 2). Network 1 mainly functioned in cancer, organismal development, and vascular system development and function, which contained 35 nodes (genes; proteins), 23 nodes (66%) were identified in this network, and ERK1/2, STAT5a/b, GH1, PRL, PLC, growth hormone, LH, ADCY, proinsulin, FSH, Cg, cytochrome C, LDL, GNAO1, and estrogen receptor were the key molecules in this network. Network 2 mainly functioned in drug metabolism, protein synthesis, and hereditary disorder, which contained 35 nodes (genes; proteins), 18 nodes (51%) were identified in this network, and Jnk, Hsp90, Akt, IL12 (complex), p38 MAPK, Ap1, HSP90B1, CD3, Mek, Tgf beta, ubiquitin, caspase, Hsp27, 14-3-3, YWHAQ, TCR, p85 (pik3r), VIM, Rock, Ige, IL15, IgG, Nos, HSP, HSPB1, HSPB8, and immunoglobulin were the key molecules in this network. Similarly, the rest 60 networks were present in Supplemental Materials 1.2–9.2. Comprehensive analysis of all 62 networks clearly found that some functional items of networks were present in different networks across nine different omics datasets.

To reveal the significance of overall variations in molecular networks from different datasets in NFPAs relative to controls, the frequencies of top functional items occurred among networks were counted and analyzed. The results revealed that top network-function items from control pituitary group were mainly related to essential biological processes of normal cell life, including RNA-transcriptional modifications, protein synthesis, carbohydrate metabolism, molecular transport, cell morphology, cell cycle, and cell growth and proliferation (Supplemental Figure 1A). Whereas, top network-function items from NFPA group were mainly related to the biological processes of endocrine and nervous system tumors, including cancer, cell signaling and interaction alteration, endocrine and nervous system disorder, inflammatory response, immunological response, cell death and survival, cell growth and proliferation, metabolism abnormalities, free radical and oxidative stress, and protein synthesis and degradation abnormalities (Supplemental Figure 1B). These results demonstrated that much different network-function variations were occurred between NFPAs and controls.

### Molecular Network-Based Hub-Molecule Panels in NFPAs

Each network containing multiple node molecules (genes; proteins) formed a web to participate in the biological functions. However, different node molecules in a given network did not equally contribute to the biological functions of that given network. Some node molecules in a network were in the hub position, namely hub molecule that connected with many other node molecules in a direct or indirect way, and played key roles in that network. Some node molecules were at the boundary position in a network, and interacted with a relatively less number of other node molecules; such a boundary node molecule could only contribute a relatively weak role in a network. Furthermore, some hub molecules were present in multiple networks that were derived from nine different datasets, which clearly revealed that different networks were interacted mutually in an NFPA biological system. Therefore, the detailed analysis of all hub-molecules in 62 networks from nine sets of omics datasets might reveal and discover key molecules, molecule-panels, and corresponding biological functions that operate in an NFPA biological system, which benefits the discovery of biomarkers for NFPAs.

In this study, a hub-molecule was defined as a node molecule that directly or indirectly connected at least five other node molecules in a network. Thus, a total of 861 hub-molecules were identified from 62 networks, and the primary function annotation of each hub-molecule was obtained from UniProt annotation page, NCBI database, and extensive literature analysis (Supplemental Table 5). A total of 42 hub-molecule panels were generated from 861 hub-molecules according to primary functions of hub-molecules. Each hub-molecule panel was displayed with the number of hub-molecules originated from different dataset. Those 42 hub-molecule panels were further grouped into 16 functional categories, and each functional category was described in detail (Supplemental Figure 2). Here, the hub-molecule panel regarding GF, GFR, and related proteins from functional category A (Supplemental Figure 2A) was taken as an example for detailed description (Supplemental Figure 3). In this panel, those hub-molecules were GF, GFR and related molecules, and 9 hub-molecules (ANGPT1, FDF, FGF2, FGFR, FGFR1, PDGF BB, PTN, Tgf beta, and VEGF) from NFPA DEGs (Dataset 1), 6 (FLT4, GFR, PDGF BB, PDGFR, Tgf beta, and VEGF) from NFPA DEPs (Dataset 2), 5 (PDGF complex, PDGF BB, Tgf beta, TGFB1, and VEGF) from NFPA mapping proteins (Dataset 3), 6 (EGFR, FGFR1, PDGF BB, Tgf beta, TGFB1, and VEGF) from invasive NFPA DEGs (Dataset 5), 2 (EGFR, and Tgf beta) from invasive NFPA DEPs (Dataset 6), 3 (ACVRL1, EGFR, and FGFR1) from control mapping proteins (Dataset 7), and 1 (VEGF) from control nitroproteins (Dataset 8).

Comprehensive analysis of those 42 hub-molecule panels revealed 16 hub-molecule functional categories (Supplemental Figure 2). In each category, those hub-molecules derived from NFPA DEGs and DEPs (Datasets 1 and 2), nitroprotein data (Dataset 4), and invasive NFPA DEGs, and DEPs (Datasets 5 and 6) were directly associated with NFPA pathogenesis, compared to hub-molecules from mapping data (datasets 3, 7, 8, and 9). For cell movement, angiogenesis, invasion, and metastasis (Supplemental Figure 2A), the important hub-molecules included actin, F-actin, a-actin, cofilin, EZR, VIM, FLNA, Rar, Rxr, Rock, ANGPT1, FDF, FGF2, FGFR, FGFR1, PDGF BB, PDGFR, PTN, TGF beta, TGFB1, EFGR, VEGF, FLT4, GFR, CDH2, COL2A1, collegens, collegen type I, collegen type IV, integrin, Laminin, SELL, VCAN, ICAM3, and MMP, which were significantly associated with NFPAs. For kinase signaling pathways-related proteins (Supplemental Figure 2B), the important hub-molecules included DUSP4, ERK, ERK1/2, JNK, MAPK, MEK, p38 MAPK, MAP2K1/2, Shc, Rac, Ras, Ras homolog, K-Ras, Rsk, Sos, Akt, PI3K (complex), PI3K (family), p85, p70 S6k, PKA, PKC(s), PKG, PRKAA, PRKACA, PRKAR1A, TK, FAK, FYN, CK2, and PDPK1, which were significantly associated with NFPAs. For protein synthesis and degradation (Supplemental Figure 2C), the important hub-molecules included FBXO6, UBC, ubiquitin, PSMA2, 26s proteasome, CAND1, MDM2, EEF1A1, and KARS, which were significantly associated with NFPAs. For stress response (Supplemental Figure 2D), the important hub-molecules included P4HB, HSP, HSP27, HSP70, HSP90, HSP90B1, HSP90AA1, HSP90AB1, HSPB1, HSPB8, HSPA5, NOS, nitric oxide, SOD, MT1L, and CAT, which were significantly associated with NFPAs. For Notch-Wnt signaling pathway (Supplemental Figure 2E), the important hub-molecules included GSK3, LGR4, CTNNB1, Notch, NOTCH3, and ATXN1, which were significantly associated with NFPAs. For cell-cycle regulation (Supplemental Figure 2F), the important hub-molecules included 14-3-3, YWHAQ, YWHAG, CDC2, cyclin A, CCND1, CDH1, and CDKN1A, which were significantly associated with NFPAs. For transcription and its regulation (Supplemental Figure 2G), the important hub-molecules included C/ebp, CREB, CREM, ETV5, FOXO1, HES1, NFAT (complex), NFAT (family), ARNT2, CEBPA, E2F, XBP1, NFYB, NFkB (complex), NANOG, GATA3, NEUROD1, N-cor, BHLHE40, SAFB, and SMAD3, which were significantly associated with NFPAs. For DNA/RNA regulation and metabolism (Supplemental Figure 2H), the important hub-molecules included ZFP36, TARDBP, HDAC, histone, histone H3, histone H4, CBX5, TIP60, and RNA polymerase II, which were significantly associated with NFPAs. For immune and inflammation-related proteins and cytokines (Supplemental Figure 2I), the important hub-molecules included BCR (complex), BSG, IgE, IgG, IgM, immunoglobulin, LGALS3, CD1, CD3, TCR, Fc gamma receptor, CXCR4, IL1, IL12 (complex), IL12 (family), IL15, interferon-a, IFNG, TNF, TNF (family), and pro-inflammatory cytokines, which were significantly associated with NFPAs. For hormones and related proteins (Supplemental Figure 2J), the important hub-molecules included ADM, β-estradiol, CGA, CYP11A1, estrogen receptor, FSH, GH1, GRB2, GH, IGFBP3, insulin, LH, POMC, PRL, proinsulin, ADRB, ESR1, ESR2, IGFBP5, progesterone, EDNRA, and GAST, which were significantly associated with NFPAs. For energy metabolism (Supplemental Figure 2K), the important hub-molecules included cytochrome C, cytochrome-c oxidase, COX4I1, COX6C, POR, AMPK, ATP5B, and LDH, which were significantly associated with NFPAs. For proteins involved in tumorigenesis (Supplemental Figure 2L), the important hub-molecules included Ap1, FOS, JUN/JUNB/JUND, STAT5a/b, SKI, SNAI2, SRC (family), TP53, and Rb, which were significantly associated with NFPAs. For apoptosis-related proteins (Supplemental Figure 2M), the important hub-molecules included BCL2, caspase, BAX, BBC3, PARP, and CASP1, which were significantly associated with NFPAs. For Ca^2+^-related proteins (Supplemental Figure 2N), the important hub-molecules included calpain, CACNA1B, calmodulin, SLC8A1, S100A1, and TRPC6, which were significantly associated with NFPAs. For G protein-related signaling pathway (Supplemental Figure 2O), the important hub-molecules included ADCY, PLC, PLC gamma, RGS2, GNAO1, and Gpcr, which were significantly associated with NFPAs. Moreover, the hub-molecules, Nr1h, NR4A1, NR4A2, HNRNPU, PP1 protein complex group, PP2A, Ppp2c, LDH, LDL, SREBF1, and APOA1, were also significantly associated with NFPAs (Supplemental Figure 2P). Those NFPA-associated hub-molecules offered an important resource to determine reliable biomarkers for NFPAs.

### High-Frequency Hub-Molecules Among Datasets in NFPAs

Some hub-molecules appeared multiple times in different NFPA dataset groups. For example, integrin, VEGF, PDGF BB, Ras, Mek, p38 MAPK, PKA, FAK, Creb, histone h3, estrogen receptor, growth hormone, cytochrome C, AP1, and ADCY appeared four times; TGF-β, ERK, Jnk, MAPK, Akt, PI3K complex, NFκB complex, immunoglobulin, LH, insulin, and LDL appeared five times; PKC, and UBC appeared six times. In this study, a hub-molecule that appeared at least three times among 42 hub-molecule panels across nine datasets was defined as a high-frequency hub-molecule. A total of 57 high-frequency hub-molecules were obtained (Table 1). Among them, 25 (43.8%) high-frenquency hub-molecules were also found with PTMScan experiment in NFPAs, including ERK, ERK1/2, Jnk, MAPK, Mek, p38 MAPK, AKT, PI3K complex, p85, PKC, FAK, Rac, Shc, HSP90, NFκB Complex, histone H3, AP1, calmodulin, and PLC, which were differentially expressed or modified at least 2.5-fold changes in NFPAs compared to controls; and actin, rock, PKA, creb, STAT5a/b, and caspase, which were differentially expressed with a fold-change of 1~2.5. Most of these high-frequency hub-molecules were kinases and signaling transduction-related molecules, which might contribute to oncogenesis and tumor development. Thereby, high-frequency hub-molecules did play essential roles in the progression of an NFPA. Those high-frequency hub-molecules might be used as targets for NFPA diagnostic indicators.

**Table 1:** High-frequency hub-molecules that were present in multiple datasets of NFPA group.

**Hub molecule**	**Frequency**	**Dataset serial number**	**PTMScan detection**	**Fold change ≥ 2.5 or ≤ −2.5**
PKC	6	1, 2, 3, 4, 5, 6	Y	Y
UBC	6	1, 2, 3, 4, 5, 6		
TGF-β	5	1, 2, 3, 5, 6		
ERK	5	1, 2, 3, 5, 6	Y	Y
ERK1/2	5	1, 2, 3, 5, 6	Y	Y
Jnk	5	1, 2, 3, 5, 6	Y	Y
MAPK	5	1, 2, 3, 5, 6	Y	Y
Akt	5	1, 2, 3, 5, 6	Y	Y
PI3K complex	5	1, 2, 3, 5, 6	Y	Y
NFκB Complex	5	1, 2, 3, 5, 6	Y	Y
Immunoglobulin	5	1, 2, 3, 5, 6		
Lh	5	1, 2, 3, 5, 6		
Insulin	5	1, 2, 3, 5, 6		
LDL	5	1, 2, 3, 5, 6		
Integrin	4	1, 2, 3, 5		
VEGF	4	1, 2, 3, 5		
PDGF BB	4	1, 2, 3, 5		
Ras	4	1, 2, 3, 5		
Mek	4	1, 2, 3, 5	Y	Y
p38 MAPK	4	2, 3, 5, 6	Y	Y
PKA	4	2, 3, 5, 6	Y	N
FAK	4	2, 3, 5, 6	Y	Y
Creb	4	1, 2, 3, 5	Y	N
Histone h3	4	2, 3, 4, 5	Y	Y
Estrogen receptor	4	1, 2, 3, 5		
GH1	4	1, 2, 3, 6		
Growth hormone	4	1, 2, 3, 5		
Cytochrome C	4	1, 2, 3, 5		
AP1	4	1, 2, 3, 5	Y	Y
ADCY	4	1, 2, 3, 6		
Actin	3	1, 3, 5	Y	N
F-Actin	3	1, 3, 5		
Rock	3	1, 2, 3	Y	N
Collagens	3	1, 3, 5		
Collagen type I	3	2, 3, 5		
Laminin	3	1, 3, 5		
p85	3	2, 3, 5	Y	Y
p70S6K	3	1, 3, 5		
Rac	3	1, 5, 6	Y	Y
Shc	3	2, 3, 5	Y	Y
Ubiquitin	3	2, 3, 5		
HSP90	3	2, 3, 5	Y	Y
Cyclin A	3	1, 3, 5		
IgG	3	2, 3, 6		
TCR	3	2, 3, 5		
IgE	3	1, 2, 3		
IFNG	3	3, 5, 6		
IFN-α	3	3, 4, 5		
B-estradiol	3	1, 3, 5		
FSH	3	1, 2, 5		
Proinsulin	3	1, 2, 3		
AMPK	3	1, 3, 5		
STAT5a/b	3	1, 2, 3	Y	N
Caspase	3	2, 3, 5	Y	N
Calmodulin	3	3, 5, 6	Y	Y
Calpain	3	1, 3, 5		
PLC	3	2, 3, 6	Y	Y

*Among 57 hub-molecules that were present in at least 3 datasets, a total of 25 hub-molecules (25/57 = 43.9%) were detected by PTMScan experiments, 19(19/57 = 33.3%) of which were changed more than 2.5 times in NFPAs compared to controls. Y, yes; N, no*.

### Canonical Pathway Alterations in NFPAs

A total of 519 statistically significant canonical pathways were mined from nine datasets, including 68 canonical pathways from NFPA DEGs (Dataset 1), 25 from NFPA DEPs (Dataset 2), 89 from NFPA mapping proteins (Dataset 3), 29 from NFPA nitroproteins (Dataset 4), 30 from invasive NFPA DEGs (Dataset 5), 28 from invasive NFPA DEPs (Dataset 6), 174 form control mapping proteins (Dataset 7), 33 from control nitroproteins (Dataset 8), and 43 from control phosphoproteins (Dataset 9) (Supplemental Materials 1.3–9.3). Of them, some statistically significantly canonical pathways were mined from only one dataset, such a type of canonical pathways had 30 canonical pathways from dataset 1, 4 from dataset 2, 8 from dataset 3, 15 from dataset 4, 5 from dataset 5, 3 from dataset 6, 49 from dataset 7, 1 from dataset 8, and 3 from dataset 9 (Supplemental Table 6). Meanwhile, a total of 139 statistically significantly canonical pathways were mined from at least two datasets (Supplemental Table 7). After extensive literature analysis of these 139 canonical pathways, a total of 68 canonical pathways were found to obviously associate with the occurrence and development of a tumor in direct and indirect ways (Supplemental Table 8). Moreover, for those 68 cancer-related canonical pathways, 14 canonical pathways were not mined from any DEGs or DEPs datasets, and 54 canonical pathways involved in any DEGs or DEPs were divided into nine canonical-pathway panels according to the similar cellular functions and biological processes (Supplemental Table 8; Supplemental Figure 4). Nine canonical-pathway panels associated significantly with NFPA pathophysiological processes were addressed in detail (Supplemental Figure 4), and differentially expressed hub-molecules (DEGs, or DEPs) in those 54 significantly cancer-related canonical pathways among nine canonical-pathway panels were summarized (Table 2). Those important canonical-pathway panels with differentially expressed hub-molecules (DEGs; DEPs) in NFPAs benefited for in-depth understanding of NFPA molecular mechanisms and discovery of reliable biomarkers for NFPAs. For example, (i) Gao and CDK5 were upregulated in CDK5 signaling. Nectin and myosin were upregulated, and CLDN was downregulated in tight junction signaling. Notch, N-cadherin, and FGFR1 were upregulated in epithelial adherens junction signaling. Dysregulation of these molecules in those pathways might promote cytoskeleton, cell adhesion, and movement imbalance in pituitary cells (Table 2: Panel A). (ii) NDUFS8, COX6B, ATP5B, CAT, and β-secret 2 were upregulated in mitochondrial dysfunction pathway, which might cause mitochondrial dysfunction and energy metabolism abnormality in NFPAs (Table 2: Panel B). (iii) ESR1 was upreguated in eNOS signaling. CaM, IP3R, and SERCA were upregulated in nitric oxide signaling in the vascular system. EPHB, EFNE, and Gβ were upreguated in ephrin b/ephrin receptor signaling. The expression abnormalities of these molecules facilitated angiogenesis, and invasion abilities of NFPAs (Table 2: Panel C). (iv) AKR1B1 was upregulated in methylglyoxal degradation III pathway. GST (GSTM2) was upregulated in nrf2-mediated oxidative stress response pathway. This situation might convert toxin metabolism and oxidative stress response in pituitary to benefit the tumor progression (Table 2: Panel D). (v) VIM was upregulated, and 14-3-3 was downregulated, in 14-3-3-mediated signaling. CALM, NCX (SLC8A2), and tropomyosin (TPM3, TPM4) were upregulated in calcium signaling. Talin, FYN, and PPM1K were upregulated, and MKP2, PPM1A, and ESR2 were downregulated, in ERK/MAPK signaling. Various IGFBPs (IGFBP3, IGFBP5, and IGFBP6) were differentially expressed in IGF-1 signaling from multiple datasets (Table 2: Panel F). These changed molecules and pathways might cause the imbalance of many important processes such as cell cycle, and proliferation, and apoptosis to promote NFPA progression.

**Table 2:** Differentially expressed hub-molecules (DEGs, or DEPs) in 54 significantly cancer-related canonical pathways among nine canonical-pathway panels in NFPAs.

**Canonical-pathway panel**	**Pathway name**		**Upregulated hub-molecules (DEGs; DEPs)**	**Downregulated hub-molecules (DEGs; DEPs)**	**Nitroproteins and nitroprotein-related proteins**
Panel A:Cytoskeleton, cell adhesion and movement pathways	1.	Actin Cytoskeleton Signaling	Dataset 1: PI3K, Talin, and Myosin	Dataset 1: TIAM, PIR121, TMSB4 and ERM	_
	2.	CDK5 Signaling	Dataset 6: Gao and CDK5	_	_
	3.	ILK Signaling	Dataset 5: FILAMIN (FLNA) and SLUG	Dataset 5: PI3K, PDK1 and MSK1/2 (RPS6KA5)	_
	4.	Inhibition of Matrix Metalloproteases	Dataset 6: ADAM	Dataset 6: MMP19	_
	5.	RhoA Signaling	_	_	Dataset 4: RHOGAP and Rhophilin are nitrated
	6.	Tight Junction Signaling	Dataset 1: NECTIN and MYOSIN	Dataset 1: TIAM1, CLDN and AP-1	_
	7.	Epithelial Adherens Junction Signaling	Dataset 1: Nectin, NOTCH, N-cadherin, FGFR1 and Myosin	_	_
Panel B:Mitochondrial dysfunction and energy metabolism related pathways	1.	Mitochondrial Dysfunction	Dataset 2: NDUFS8, COX6B and ATP5B; Dataset 6: CAT and β-secret2, ATP5B	Dataset 2: GPX4; Dataset 6: ATP5A1	_
	2.	Oxidative Phosphorylation	Dataset 2: NDUFS8, COX6B, ATP5B	_	_
	3.	AMPK Signaling	Dataset 5: PP2C and PFK	Dataset 5: PI3K, PKA and PDK1	_
Panel C:Angiogenesis, invasion, and metastasis related pathways	1.	CXCR4 Signaling	Dataset 1: Gβ, PI3K and IP3R	Dataset 1: CXCR4 and c-FOS	_
	2.	eNOS Signaling	Dataset 5: ESR1 and HSP90 (HSPCA and HSPCB)	Dataset 5: PI3K, PDK1, PKA and ESR2; Dataset 6: HSP70	_
	3.	Nitric oxide signaling in the cardiovascular system	Dataset 1: PI3K, CaM, IP3R and SERCA; Dataset 5: HSP90 (HSPCA and HSPCB)	Dataset 5: PI3K and PKA	_
	4.	Ephrin B Signaling	Dataset 1: EPHB, EFNE and Gβ	Dataset 1: CXCR4	_
	5.	Ephrin Receptor Signaling	Dataset 1: EPHB, EFNE and Gβ	Dataset 1: CXCR4 and ANGPT1	_
	6.	Hypoxia signaling in the cardiovascular system	Dataset 5: HSP90 (HSPCA and HSPCB)	Dataset 5: UBE2	_
	7.	Role of Tissue Factor Cancer	Dataset 2: Src	Dataset 2: FX (FXα)	_
Panel D:Toxin metabolism and oxidative stress related pathways	1.	Aryl hydrocarbon receptor signaling	Dataset 2: GST (GSTM2); Dataset 5: HSP90 (HSPCA and HSPCB), ESR1 and Bax	Dataset 2: HSP27, HSP90 and TGM2; Dataset 5: ESR2	_
	2.	Corticotropin Releasing Hormone Signaling	Dataset 1: CALM and IP3R	Dataset 1: ACTH, Nur77 and c-FOS	_
	3.	Glucocorticoid Receptor Signaling	Dataset 1: PI3K	Dataset 1: HSP70, c-Fos, CCL2, BCL2, PRL and POMC	_
	4.	Glutathione redox reactions I	_	Dataset 2: GPX4	_
	5.	Melatonin signaling	_	_	Dataset 4: PKA are nitrated
	6.	Methylglyoxal Degradation III	Dataset 2: AKR1B1	_	_
	7.	NRF2-mediated Oxidative Stress Response	Dataset 2: GST (GSTM2) and ERP29	Dataset 2: HSP22, HSP27 and HSP90	_
	8.	Superoxide Radicals Degradation	Dataset 6: CAT	_	_
Panel E:Protein synthesis, degradation and amino acid metabolism related pathways	1.	EIF2 signaling	Dataset 5: 60S ribosomal subunit (RPL10 and RPL32)	Dataset 5: PI3K, PDK1, 40S ribosomal subunit (RPS2 and RPS2) and 60S ribosomal subunit (RPL18A)	_
	2.	Polyamine Regulation in Colon Cancer	Dataset 1: ODC1 and SSAT (SAT1)	_	_
	3.	Putrescine Degradation III	Dataset 1: MAOB	Dataset 1: ALDH2 and SSAT (SAT1)	_
	4.	Protein Ubiquitination Pathway	Dataset 5: HSP (HSPCA and HSPCB)	Dataset 2: HSP (HSPB8, GRP94 and HSPB1); Dataset 5: E2	Dataset 4: PSMA2 is nitrated, Ub is nitroprotein-interacted protein
Panel F:Cell cycle, proliferation and apoptosis related pathways	1.	14-3-3-mediated Signaling	Dataset 2: VIM	Dataset 2: 14-3-3	_
	2.	Calcium Signaling	Dataset 1: CALM, IP3R, PMCA, NCX (SLC8A2), SERCA and Myosin; Dataset 5: nACHR, NCX (SLC8A1) and Tropomyosin (TPM3, TPM4)	Dataset 1: DSCR1; Dataset 5: PKA	_
	3.	Cardiac β-adrenergic Signaling	Dataset 5: PPM1K, PPP1R11 and NCX	Dataset 5: IPKA, AKAP, PKA and PKI(PKIG)	_
	4.	ERK/MAPK Signaling	Dataset 1: PI3K, Talin and cPLA2; Dataset 2: FYN; Dataset 5: 14-3-3(YWHAG), PPM1K, PPP1R11 and ESR1	Dataset 1: MKP2; Dataset 2: 14-3-3(YWHAQ) and HSP27; Dataset 5: PI3K, PKA, PPM1A, ESR2 and RPS6KA5	_
	5.	IGF-1 Signaling	Dataset 1: PI3K; Dataset 5: IGFBP (IGFBP5) and 14-3-3 (YWHAG)	Dataset 1: IGFBP (IGFBP3), FKHR and c-FOS; Dataset 2: IGFBP (IGFBP6) and 14-3-3 (YWHAQ); Dataset 5: PI3K, PDK1 and PKA	_
	6.	mTOR Signaling	Dataset 5: PROTOR (PRR5)	Dataset 5: PI3K, PDK1, RSK (RPS6KA5) and 40S Ribosome(RPS2 and RPS2)	_
	7.	p53 signaling	Dataset 1: PI3K; Slug, Dataset 5: PUMA(BBC3) and BAX	Dataset 1: GADD45, NOXA, Bcl-2 and ZAC1; Dataset 5: PI3K	_
	8.	PEDF signaling	Dataset 1: PI3K and DOCK3; Dataset 5: GDNF	Dataset 1: BCL-2; Dataset 5: PI3K, TCF	_
	9.	PI3K/Akt signaling	Dataset 5: HSP90 (HSPCA and HSPCB) and 14-3-3 (YWHAG)	Dataset 2: HSP90 (GRP94) and 14-3-3 (YWHAQ); Dataset 5: PI3K p110 and PDK1	_
	10.	Sonic Hedgehog Signaling	_	_	Dataset 4: PKA is nitrated
	11.	Tec kinase signaling	Dataset 2: Gα and SRC(FYN)	_	_
	12.	Telomerase Signaling	Dataset 5: HSP90 (HSPCA, HSPCB)	Dataset 5: PI3K and PDK1	_
	13.	β-Adrenergic Signaling	Dataset 1: Gβ, Calm, IP3R and NCX	_	_
Panel G:Immunity related pathways	1.	IL-1 Signaling	_	_	Dataset 4: IRAK-2 and PKA are nitrated
	2.	Role of NFATRegulation of the Immune Response	Dataset 1: PI3K, Gβ, CALM, CSP (CSPG5) and IP3R	Dataset 1: c-FOS	_
Panel H:ER stress related pathways	1.	Endoplasmic Reticulum Stress Pathway	_	Dataset 2: GRP94; Dataset 6: BIP (HSPA5 and HSPA6)	_
	2.	Unfolded protein response	Dataset 1: SREBP (SREBF1)	Dataset 1: PDI (P4HB), c/EBP, BCL2 and HSP70 (HSPA2)	_
Panel I:Others	1.	Aldosterone Signaling Epithelial Cells	Dataset 5: HSPCA and HSPCB	Dataset 2: HSPB8, HSP90B1 (GRP94) and HSPB1; Dataset 5: DNAJB6, PI3K and PDK1	_
	2.	Docosahexaenoic acid (DHA) signaling	Dataset 1: PI3K; Dataset 5: BAX	Dataset 1: FKHR and BCL2; Dataset 5: PI3K and PDK1	_
	3.	Endometrial Cancer Signaling	_	Dataset 5: PI3K, PDK1 and E-cadherin	_
	4.	Growth Hormone Signaling	Dataset 1: PI3K; Dataset 5: CEBPA	Dataset 1: GH, c-FOS and IGFBP3; Dataset 2: GH; Dataset 5: PI3K and PDK1	_
	5.	Hereditary Breast Cancer Signaling	Dataset 1: PI3K	Dataset 1: BLM, Wee1 and GADD45	Dataset 4: Ub is nitroprotein-interacted protein
	6.	PPARα/RXRα Activation	_	Dataset 2: GH, HSP90 (GRP94) and APOA1	_
	7.	PXR/RXR Activation	_	_	Dataset 4: PKA is nitrated
	8.	TR/RXR Activation	Dataset 1: PI3K, ZAKI4 and SREBP; Dataset 6: F10	Dataset 1: GH1 and FASN; Dataset 2: F10 and GH1; Dataset 6: GH1	_

*Dataset 1: NFPA DEGs. Dataset 2: NFPA DEPs. Dataset 5: invasive NFPA DEGs. Dataset 6: NFPA DEPs. Dataset 4: NFPA nitroproteins*.

### Validations of Networks and Canonical Pathways With PTMScan® Direct Test

**Figure 3:**
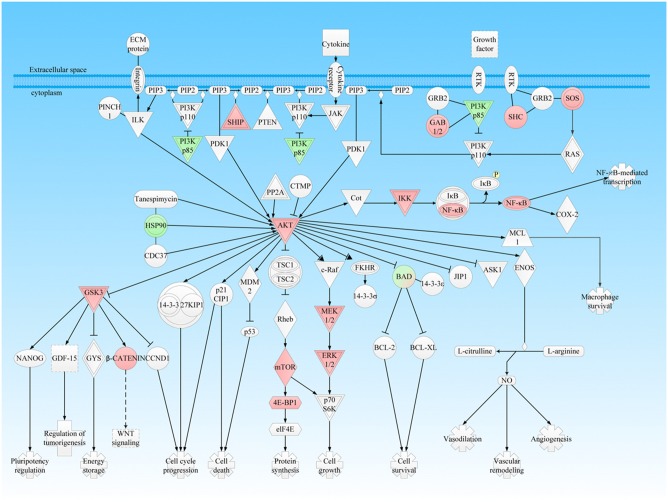
PI3K-AKT signaling pathway was involved in NFPA. This pathway was derived from the IPA analysis results of multi-omics, and then it was modified and verified according to the PTMScan results of NFPAs compared to controls. The red color means upregulation of a molecule in NFPAs, and the green color means downregulation of a molecule in NFPAs. The gradient color degree means slightly different expression tendency of that molecule.

**Figure 4:**
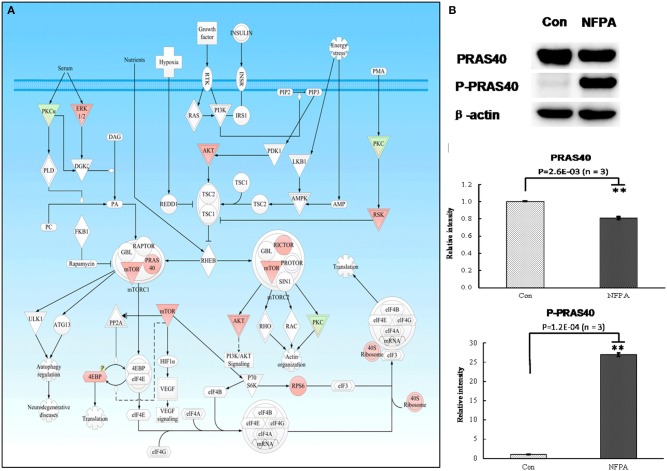
mTOR signaling pathway was involved in NFPA. **(A)** mTOR signaling pathway was derived from the IPA analysis results of multi-omics, and then it was modified and verified according to the PTMScan results of NFPAs compared to controls. The red color means upregulation of a molecule in NFPAs, and the green color means downregulation of a molecule in NFPAs. The gradient color degree means slightly different expression tendency of that molecule. **(B)** Immunoaffinity Western blot analyses of PRAS40 and p-PRAS40 (Thr246) confirmed. mTORC1 signaling was involved in NFPAs (*n* ≥ 3). The relative intensity was the ratio of absorbance units of NFPAs compared to controls. **p < 0.01.

**Figure 5:**
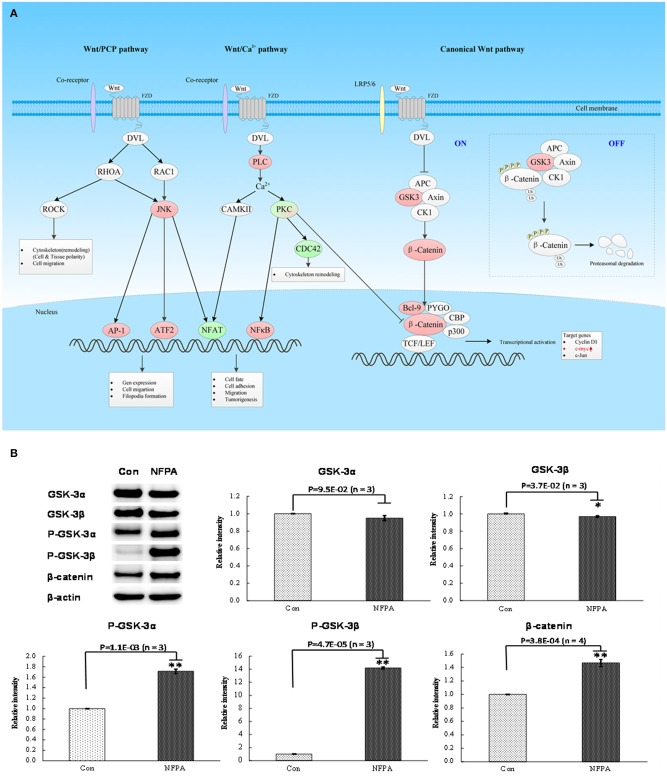
Wnt signaling pathway was involved in NFPA. **(A)** Wnt signaling pathway was derived from PTMScan results of NFPAs compared to controls. The red color means upregulation of a molecule in NFPAs, and the green color means downregulation of a molecule in NFPAs. The gradient color degree means slightly different expression tendency of that molecule. **(B)** Activation of canonical Wnt pathway in NFPAs was verified by Western blot results of GSK-3α, GSK-3β, p-GSK-3β (Ser9), p-GSK-3α/β (Ser21/9), β-catenin and β-actin in control pituitaries (Con) and NFPAs (*n* ≥ 3). The relative intensity was the ratio of absorbance units of NFPAs compared to Con. *p < 0.05, **p < 0.01.

**Figure 6:**
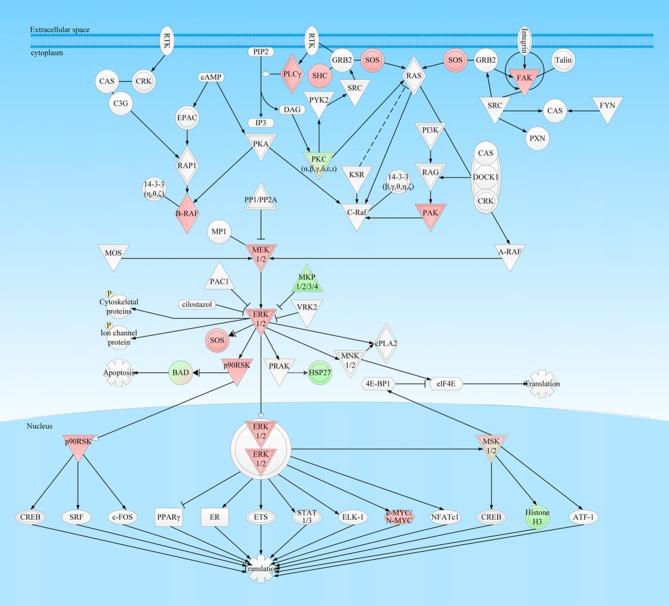
ERK-MAPK signaling pathway was involved in NFPA. This pathway was derived from the IPA analysis results of multi-omics, and then it was modified and verified according to the PTMScan results of NFPAs compared to controls. The red color means upregulation of a molecule in NFPAs, and the green color means downregulation of a molecule in NFPAs. The gradient color degree means slightly different expression tendency of that molecule.

PTMScan® Direct test that combined immunoaffinity enrichment and LC-MS/MS was used to identify and quantify phosphorylated peptides/proteins within multiple key canonical pathways (18). This study analyzed a total of 1006 unique phosphorylated-sites within 409 proteins that participated in more than 19 pathways. Moreover, many hub-molecules in multiple important canonical pathways including PI3K/Akt, mTOR, Wnt, NFκB, ERK/MAPK, p38, and JNK signaling pathways in NFPAs were identified with PTMScan® Direct test; and PI3K/AKT, mTOR, Wnt, NF-κB, ERK/MAPK, p38, and JNK signaling pathways were confirmed excessively activated in NFPAs with PTMScan experiments-based phosphorylation analysis (Table 3). From PTMScan® Direct results in NFPAs compared to controls, the phosphorylated sites and levels were identified and quantantified for proteins PI3K, SHIP, GAB2, SHC, SOS, HSP90, AKT, IKK, NFκB, GSK3, β-CATENIN, BAD, MEK1/2, and ERK1/2 in PI3K/Akt signaling pathway (Figure 3); for proteins PKC, p90RSK, mTOR, PRAS40, RICTOR, 4EBP, and RPS6 in the downstream mTOR signaling (Figure 4A); for proteins GSK3, β-catenin, Bcl-9, PLC, NFkB, JNK, AP-1, and ATF2 in Wnt pathways (Figure 5A); and for proteins SHC, SOS, PI3K, PKC, MEK1/2, ERK1/2, BAD, p90RSK, 4EBP1, PLC, PAK, B-RAF, FAK, MKP, HSP27, MSK1/2, c-Myc, NFATc, CREB, Histone H3, and Jun in ERK/MAPK signaling (Figure 6), and some of these proteins were also found in PI3K/Akt/mTOR signaling (Table 3). PTMScan experiments revealed the significantly increased phosphorylation levels at residues Thr308/309/305 in Akt1/2/3, Ser472 in Akt3, Ser2448 in mTOR, Ser246 in PRAS40; Thr37 and Thr46 in both 4E-BP1 and 4E-BP2, Thr23 in 4E-BP3; Ser235, Ser236, Ser240, Thr241 and Ser244 in S6, Ser376 in IKKγ, Ser21 in GSK3α, and Ser552 and Ser675 in β-Catenin (Table 3), which might stimulate and magnify PI3K/Akt signaling, and its downstream mTOR, NFκB, and canonical Wnt pathways to contribute to tumor progression. Moreover, the significantly increased phosphorylation levels were also found at residues Ser446 or Ser447 in B-Raf, Ser218 or Ser222 in MEK1, Thr202 and Tyr187 in both ERK1 and ERK2, Tyr220, Ser221, and Thr225 in RSK1, Tyr226, Ser227, and Thr231 in RSK2, Tyr231, Ser232, and Thr236 in RSK4, Ser376 in MSK1, Ser360 in MSK2, Thr575 and Tyr577 (Thr575 and/or Tyr576) in FAK, Thr180 and Tyr182 (Tyr182 and Thr185) in p38a, Thr183 and Tyr185 in JNK1, Thr221 and Tyr223 in JNK3, Thr183 and Tyr185 (Thr175 and Tyr185) in JNK2, and Thr69 and Thr71 in ATF2; whereas the decreased phosphorylation level was found at residue Ser359 in MKP1, and Ser71, Ser74, Ser75, and Tyr76 in BAD (Figure 6 and Table 3). Those findings demonstrated that ERK/MAPK and its related p38 and Jnk pathways were activated to significantly affect NFPA development. Thereby, PTMScan® experiment confirmed clearly PI3K/Akt, mTOR, Wnt, ERK/MAPK, p38, and Jnk pathways derived from IPA pathway network and bioinformatic analyses of multi-omics data, and further revealed the functions of those pathway-networks in NFPA tumorigenesis.

**Table 3:** PI3K/AKT, mTOR, Wnt, and ERK/MAPK signaling pathways confirmed with PTMScan experiments and phosphorylation sites.

**Pathway**	**Symbol**	**Hub molecules**	**Gene name**	**Protein name**	**Phospho-site**	**Peptide**	**Tumor: normal ratio**	**Reported reference**
PI3K/AKT pathway	HSP90	Y	HSP90B1	GRP94		EAESSPFVER	−2.5	
						FAFQAEVNR	−2.5	
						FQSSHHPTDITSLDQYVER	−3.0	
						GTTITLVLK	−2.6	
						GVVDSDDLPLNVSR	−4.3	
						IKEDEDDKTVLDLAVVLFETATLR	−3.0	
						NLLHVTDTGVGM#TR	−2.7	
						TVWDWELM#NDIKPIWQRPSK	−2.5	
			HSP90B2P; HSP90B1	HSP90B2P; GRP94		GLFDEYGSKK	−2.8	
	IKK	Y	IKBKG	IKKG; IKKG iso 2; IKKG iso 3	§374; 442; 275	HVEVSQAPLPPAPAY^*^LSSPLALPSQR	2.8	
					§376; 444; §277	HVEVSQAPLPPAPAYLS^*^SPLALPSQR	2.8	
					§377; 445; 278	HVEVSQAPLPPAPAYLSS^*^PLALPSQR	5.2	
	NFKB	Y	NFKB2	NFkB-p100; NFkB-p100 iso4 iso 4		DSGEEAAEPSAPSR	6.1	
	SHIP	N	INPPL1	SHIP-2; SHIP-2 iso 2	§886; 644	ERLY^*^EWISIDKDEAGAK	33.7	
	GAB2	N	GAB2	GAB2; GAB2 iso2 iso 2	§476; 438	AGDNSQSVY^*^IPM#SPGAHHFDSLGYPSTTLPVHR	4.8	
PI3K/AKT pathway, mTOR pathway	PI3K (P85)	Y	PIK3R2	PIK3R2		VYHQQYQDK	−2.9	
			PIK3R4	PIK3R4	§926, 932	KPVIPVLSS^*^TILPST^*^YQIR	−5.8	
	AKT	Y	AKT1; AKT2; AKT1; AKT3; AKT3; RPS6KB1; RPS6KB1; RPS6KB1; RPS6KB1; RPS6KB1; RPS6KB2; SGK1; SGK2; SGK3; SGK1; SGK1; SGK1; SGK1; SGK2; SGK2; SGK3	Akt1; Akt2; Akt1 iso 2; Akt3; Akt3 iso 2; p70S6K; p70S6K iso2 iso 2; p70S6K iso2 iso 3; p70S6K iso2 iso 5; p70S6K iso2 iso 4; P70S6KB; SGK1; SGK2; SGK3; SGK1 iso 2; SGK1 iso3 iso 3; SGK1 iso 4; SGK1 iso 5; SGK2 iso2 iso 2; SGK2 iso 3; SGK3 iso 2		ITDFGLCK	3.3	Phospho-Akt (Ser473), and Akt (Total), increased (19).
			AKT1; AKT2; AKT1; AKT3; AKT3	Akt1; Akt2; Akt1 iso 2; Akt3; Akt3 iso 2	§308; §309; §246; §305; §305	T^*^FCGTPEYLAPEVLEDNDYGR	3.1	
					§312; §313; 250; §309; §309	TFCGT^*^PEYLAPEVLEDNDYGR	3.1	
			AKT2	Akt2	§313	EGISDGATM#KTFCGT^*^PEYLAPEVLEDNDYGR	34.0	
			AKT2	Akt2; Akt2 iso 2	§475; 432	THFPQFSY^*^SASIRE	3.7	
			AKT3	Akt3	§472	RPHFPQFS^*^YSASGR	6.6	
			AKT3	Akt3	§472, §476	RPHFPQFS^*^YSAS^*^GRE	9.9	
			AKT3	Akt3	§474, §476	RPHFPQFSYS^*^AS^*^GRE	9.9	
			AKT3	Akt3; Akt3 iso 2		EGITDAATM#K	4.3	
	mTOR	Y	mTOR	mTOR	§2444	T^*^RTDSYSAGQSVEILDGVELGEPAHKK	2.6	Phospho-mTOR (Ser2448), and mTOR (Total), no significant change (20).
					§2446	TRT^*^DSYSAGQSVEILDGVELGEPAHK	3.0	
					§2446, §2449	T^*^DSY^*^SAGQSVEILDGVELGEPAHK	5.8	
					§2448	TRTDS^*^YSAGQSVEILDGVELGEPAHK	2.5	
					§2449	TRTDSY^*^SAGQSVEILDGVELGEPAHKK	2.6	
					§2450	TRTDSYS^*^AGQSVEILDGVELGEPAHKK	2.6	
					§2454	TRTDSYSAGQS^*^VEILDGVELGEPAHKK	5.1	
					§2471	T^*^GTTVPESIHSFIGDGLVKPEALNK	23.3	
	PRAS40	N	AKT1S1	PRAS40; PRAS40 iso3 iso 2; PRAS40 iso3 iso 3	§246; 116; 266	LNT^*^SDFQK	22.7	
	S6	N	RPS6	S6	§235, §236, §240	RLS^*^S^*^LRAS^*^TSKSESSQK	55.8	
					§235, §241, §244	LS^*^SLRAST^*^SKS^*^ESSQK	34.2	
					§236, §240	RLSS^*^LRAS^*^TSK	10.1	
					§236, §241, §242	RLSS^*^LRAST^*^S^*^KSESSQK	7.8	
					§236, §241, §244	RLSS^*^LRAST^*^SKS^*^ESSQK	55.8	
					§236, §242, §244	RLSS^*^LRASTS^*^KS^*^ESSQK	7.8	
	RICTOR	Y	RICTOR	RICTOR; RICTOR iso3 iso 3		DAFGYATLK	3.9	
	VEGFR	N	FLT1	VEGFR1; VEGFR1 iso2 iso 5; VEGFR1 iso2 iso 6; VEGFR1 iso2 iso 7; VEGFR1 iso2 iso 8	1295; 513; 420; 300; 318	ESGLSDVSRPSFCHS^*^SCGHVSEGK	2.8	
PI3K/AKT pathway, mTOR pathway, ERK/ MAPK signaling	ERK	Y	MAPK1	ERK2; ERK2 iso 2	§185, §187; §185, §187	VADPDHDHTGFLT^*^EY^*^VATR	90.6	Phospho-ERK1/2(Thr183), increased; ERK1/2(Total), no significant change (20).
					§185; §185	VADPDHDHTGFLT^*^EYVATR	114.5	
					§187; §187	VADPDHDHTGFLTEY^*^VATR	114.5	
						VADPDHDHTGFLTEYVATR	4.9	
			MAPK1; MAPK1; MAPK3; MAPK3; MAPK3	ERK2; ERK2 iso 2; ERK1; ERK1 iso2 iso 2; ERK1 iso2 iso 3		APEIM#LNSK	6.5	
			MAPK3	ERK1; ERK1 iso2 iso 2; ERK1 iso2 iso 3	§198, §204; 198, 204; 198, 204	IADPEHDHT^*^GFLTEY^*^VATR	385.0	
					§202, §204; 202, 204; 202, 204	IADPEHDHTGFLT^*^EY^*^VATR	24.9	
					§202, §207; 202, 207; 202, 207	IADPEHDHTGFLT^*^EYVAT^*^R	24.9	
					§202; 202; 202	IADPEHDHTGFLT^*^EYVATR	62.4	
					§204; 204; 204	IADPEHDHTGFLTEY^*^VATR	62.4	
	RSK	Y	RPS6KA1; RPS6KA3; RPS6KA6; RPS6KA1; RPS6KA1; RPS6KA1; RPS6KA6	p90RSK; RSK2; RSK4; p90RSK iso2 iso 2; p90RSK iso 3; p90RSK iso 4; RSK4 iso 2	§220; §226; §231; 229; 128; 204; §231	KAY^*^SFCGTVEYM#APEVVNR	2.8	
					§221; §227; §232; 230; 129; 205; §232	KAYS^*^FCGTVEYM#APEVVNR	2.8	
					§225; §231; §236; 234; 133; §209; §236	KAYSFCGT^*^VEYMAPEVVNR	7.7	
			RPS6KA4	MSK2; MSK2 iso2 iso 2	§360, §365; 360, 365	IFQGYS^*^FVAPS^*^ILFDHNNAVM#TDGLEAPGAGDRPGR	−3.0	
					§360; 360	IFQGYS^*^FVAPSILFDHNNAVM#TDGLEAPGAGDRPGR	12.1	
			RPS6KA5	MSK1; MSK1 iso 2; MSK1 iso 3	§376; §376; 297	LFQGYS^*^FVAPSILFK	184.0	
						EFVADETER	7.5	
	4EBP	N	EIF4EBP1	4E-BP1	§34, §37	VVLGDGVQLPPGDY^*^STT^*^PGGTLFSTTPGGTR	61.1	
					§34, §41	VVLGDGVQLPPGDY^*^STTPGGT^*^LFSTTPGGTR	61.1	
					§35, §41	VVLGDGVQLPPGDYS^*^TTPGGT^*^LFSTTPGGTR	61.1	
					§35, §44	VVLGDGVQLPPGDYS^*^TTPGGTLFS^*^TTPGGTR	61.1	
					§35, §46	RVVLGDGVQLPPGDYS^*^TTPGGTLFSTT^*^PGGTR	3.3	
					§36	VVLGDGVQLPPGDYST^*^TPGGTLFSTTPGGTR	9.7	
					§37, §45	VVLGDGVQLPPGDYSTT^*^PGGTLFST^*^TPGGTR	61.1	
					§41	VVLGDGVQLPPGDYSTTPGGT^*^LFSTTPGGTR	9.7	
					§41, §44	VVLGDGVQLPPGDYSTTPGGT^*^LFS^*^TTPGGTR	61.1	
					§44	VVLGDGVQLPPGDYSTTPGGTLFS^*^TTPGGTR	9.7	
					§46	VVLGDGVQLPPGDYSTTPGGTLFSTT^*^PGGTR	7.1	
						DLPTIPGVTSPSSDEPPM#EASQSHLR	35.2	
						FLM#ECR	20.5	
			EIF4EBP1; EIF4EBP2	4E-BP1; 4E-BP2	§44; §44	TPGGTLFS^*^TTPGGTR	94.0	
					§45; §45	TPGGTLFST^*^TPGGTR	94.0	
					§46; §46	TLFSTT^*^PGGTR	2.9	
			EIF4EBP2	4E-BP2	§25, §44	TVAIS^*^DAAQLPHDYCTTPGGTLFS^*^TTPGGTR	61.3	
					§25, §45	TVAIS^*^DAAQLPHDYCTTPGGTLFST^*^TPGGTR	61.3	
					§25, §46	TVAIS^*^DAAQLPHDYCTTPGGTLFSTT^*^PGGTR	41.7	
					§34, §45	TVAISDAAQLPHDY^*^CTTPGGTLFST^*^TPGGTR	61.3	
					§34, §46	TVAISDAAQLPHDY^*^CTTPGGTLFSTT^*^PGGTR	61.3	
					§36	TVAISDAAQLPHDYCT^*^TPGGTLFSTTPGGTR	6.9	
					§36, §46	TVAISDAAQLPHDYCT^*^TPGGTLFSTT^*^PGGTR	61.3	
					§37	TVAISDAAQLPHDYCTT^*^PGGTLFSTTPGGTR	6.9	
					§37, §45	TVAISDAAQLPHDYCTT^*^PGGTLFST^*^TPGGTR	61.3	
					§37, §46	TVAISDAAQLPHDYCTT^*^PGGTLFSTT^*^PGGTR	61.3	
					§44	TVAISDAAQLPHDYCTTPGGTLFS^*^TTPGGTR	6.9	
					§45	TVAISDAAQLPHDYCTTPGGTLFST^*^TPGGTR	6.9	
					§46	TVAISDAAQLPHDYCTTPGGTLFSTT^*^PGGTR	6.9	
						TVAISDAAQLPH	74.7	
						VEVNNLNNLNNHDR	33.4	
			EIF4EBP3	4E-BP3	§23	DQLPDCYSTT^*^PGGTLYATTPGGTR	19.3	
					§23, §32	DQLPDCYSTT^*^PGGTLYATT^*^PGGTR	84.7	
					§27	DQLPDCYSTTPGGT^*^LYATTPGGTR	67.4	
					§31	DQLPDCYSTTPGGTLYAT^*^TPGGTR	67.4	
					§32	DQLPDCYSTTPGGTLYATT^*^PGGTR	67.4	
					22	DQLPDCYST^*^TPGGTLYATTPGGTR	67.4	
PI3K/AKT pathway, mTOR pathway, ERK/ MAPK signaling, Noncanonical Wnt pathway	PKC	Y	PRKCA	PKCA	§651	IANIDQS^*^DFEGFSYVNPQFVHPILQSAV	24.4	
			PRKCA; PRKCB; PRKCB	PKCA; PKCB; PKCB iso2 iso 2	§48; §48; 48	QPT^*^FCSHCTDFIWGFGK	−33.1	
			PRKCA; PRKCB; PRKCB; PRKCG; PRKCG	PKCA; PKCB; PKCB iso2 iso 2; PKCG; PKCG iso 2	§497; §500; 500; §514; 401	T^*^FCGTPDYIAPEIIAYQPYGK	−3.1	
					§501; §504; 504; §518; 405	TFCGT^*^PDYIAPEIIAYQPYGK	−3.6	
					§504; §507; 507; §521; 408	TFCGTPDY^*^IAPEIIAYQPYGK	−3.1	
			PRKCD	PKCD; PKCD iso2 iso 2	§664; 695	NLIDSM#DQSAFAGFS^*^FVNPK	−3.7	
						FEHLLED	−4.5	
PI3K/AKT pathway, Wnt pathway	GSK3	Y	GSK3A	GSK3A	§19	T^*^SSFAEPGGGGGGGGGGPGGSASGPGGTGGGK	17.1	
					§19, §39	T^*^SSFAEPGGGGGGGGGGPGGS^*^ASGPGGTGGGK	17.8	
					§21	TSS^*^FAEPGGGGGGGGGGPGGSASGPGGTGGGK	17.1	
					§21, §39	TSS^*^FAEPGGGGGGGGGGPGGS^*^ASGPGGTGGGK	22.1	
	CTNNB1	Y	CTNNB1	CTNNB1	§551	T^*^SMGGTQQQFVEGVR	5.0	No significant change of total β-catenin (21), nuclear accumulation of β-catenin (22).
					§552	RTS^*^M#GGTQQQFVEGVR	5.0	
					§556	TSM#GGT^*^QQQFVEGVR	4.7	
					§675, §679	RLS^*^VELT^*^SSLFR	4.7	
					§675, §680	KRLS^*^VELTS^*^SLFR	7.7	
					§675, 681	RLS^*^VELTSS^*^LFR	10.0	
					§718	S^*^FHSGGYGQDAL	19.1	
					§721	SFHS^*^GGYGQDALGM#DPM#	28.4	
						GGTQQQFVEGVR	17.8	
						GTQQQFVEGVR	49.5	
						QDDPSYR	4.0	
						RTSM#GGTQQQFVEGVR	67.3	
						SFHSGGYGQD	4.2	
						SFHSGGYGQDA	6.8	
						SFHSGGYGQDAL	3.2	
						TQQQFVEGVR	23.9	
						TSM#GGTQQQFVEGVR	5.3	
						TSMGGTQQQFVEGVR	18.0	
			CTNNB1; JUP; CTNNB1	CTNNB1; CTNNG; CTNNB1 iso 2		M#EEIVEGCTGALH	2.5	
						M#EEIVEGCTGALHI	41.9	
						MEEIVEGCTGALH	52.8	
Wnt pathway	BCL9	N	BCL9	Bcl-9		TVASSDDDSPPAR	2.9	
Noncanonical Wnt pathway	JNK	Y	MAPK8; MAPK10; MAPK8; MAPK8; MAPK8;	JNK1; JNK3; JNK1 iso2 iso 2; JNK1 iso2 iso 3; JNK1 iso2 iso 4; JNK1 iso2 iso 5; JNK3 iso2 iso 2; JNK3	§183, §185; §221, §223; §183, §185; §183, §185; §183, §185; §183, §185; §221, §223;	TAGTSFM#M#T^*^PY^*^VVTR	12.2	
			MAPK8; MAPK10; MAPK10; MAPK10	iso2 iso 3; JNK3 iso2 iso 4	183, 185; 76, 78			
			MAPK9	JNK2; JNK2 iso2 iso 2; JNK2 iso3 iso 3; JNK2 iso3 iso 4; JNK2 iso3 iso 5	§183, §185; 183, §185; 183, 185; 183, 185; 183, 185	TACTNFM#M#T^*^PY^*^VVTR	8.3	
					175, §185; 175, §185; 175, 185; 175, 185; 175, 185	T^*^ACTNFM#M#TPY^*^VVTR	8.3	
	CDC42	N	CDC42	CDC42; CDC42 iso1 iso 1		IGGEPYTLGLFDTAGQEDYDR	−28.6	
ERK/ MAPK signaling, Noncanonical Wnt pathway	PLC	Y	PLCG1	PLCG1; PLCG1 iso2 iso 2	§1248; 1249	AREGS^*^FESR	22.4	
PI3K/AKT pathway, ERK/ MAPK signaling	SHC	Y	SHC4	SHC4; SHC4 iso 2	§424; 181	CSSVY^*^ENCLEQSR	21.2	
	SOS	Y	SOS2	SOS2; SOS2 iso 2	§1132; 1099	SFFSS^*^CGSLHK	11.9	
	BAD	N	BAD	BAD	§71	S^*^RHSSYPAGTEDDEGM#GEEPSPFR	−26.9	
					§74	SRHS^*^SYPAGTEDDEGM#GEEPSPFR	−26.9	
					§75	SRHSS^*^YPAGTEDDEGM#GEEPSPFR	−26.9	
					§76	SRHSSY^*^PAGTEDDEGM#GEEPSPFR	−332.1	
					§80	HSSYPAGT^*^EDDEGMGEEPSPFR	7.5	
	MEK	Y	MAP2K1; MAP2K2; MAP2K1	MEK1; MEK2; MEK1 iso 2 iso 2	§218; §222; 192	LCDFGVSGQLIDS^*^M#ANSFVGTR	339.6	Phospho-MEK1/2 (Ser217/221), increased; MEK1/2 (Total), no significant change (20).
					§222; §226; 196	LCDFGVSGQLIDSM#ANS^*^FVGTR	339.6	
ERK/ MAPK signaling	FAK	Y	PTK2	FAK; FAK iso2 iso 2; FAK iso2 iso 3; FAK iso2 iso 4; FAK iso5 iso 5; FAK iso2 iso 7	§575, §577; 423, 425; 423, 425; 423, 425; 575, 577; 575, 577	YMEDST^*^YY^*^K	16.3	
					§575; 423; 423; 423; 575; 575	YMEDST^*^YYK	233.4	
					§576; 424; 424; 424; 576; 576	YMEDSTY^*^YK	233.4	
			PTK2; PTK2; PTK2; PTK2; PTK2; PTK2; PTK2B; PTK2B	FAK; FAK iso2 iso 2; FAK iso2 iso 3; FAK iso2 iso 4; FAK iso5 iso 5; FAK iso2 iso 7; Pyk2; Pyk2 iso2 iso 2		LGDFGLSR	7.1	
	PAK	N	PAK6	PAK6; PAK6 iso 2	§132; §132	AQSLGLLGDEHWATDPDM#YLQS^*^PQSER	27.1	
	RAF	Y	BRAF	BRAF	§446	RDS^*^SDDWEIPDGQITVGQR	1193.2	B-Raf mRNA, increased, B-Raf protein (Total), variable expression, increased, decreased or no significant change (23).
					§447	RDSS^*^DDWEIPDGQITVGQR	1193.2	
	MKP	Y	DUSP1	MKP-1	§359	GTSTTTVFNFPVSIPVHSTNSALSYLQS^*^PITTSPSC	−2.8	
	HSP27	Y	HSPB1	HSP27		GPSWDPFR	−5.6	
						LFDQAFGLPR	−18.1	
	MYC	N	MYC	Myc; Myc iso2 iso 2		NYDLDYDSVQPY	4.6	Phospho-c-myc (Thr58/Ser62), decreased; Phospho-c-myc (Ser62), no significant change; c-myc (total), no significant change (20) or increased (21, 22).
	MYCT1		MYCT1	MYCT1	112, 114	S^*^RS^*^SYTHGLNR	590.0	
					114	S^*^SYTHGLNR	12.5	
					115	SRSS^*^YTHGLN	189.3	
	NFAT	Y	NFATC3	NFAT4;NFAT4 iso 2;NFAT4 iso 3;NFAT4 iso 4;NFAT4 iso 5;NFAT4 iso 6		LVFGEDGAPAPPPPGSR	−2.8	
	Histone h3	Y	H3F3A	H3F3A		FQSAAIGALQEASEAYLVGLFEDTNLCAIHAK	−23.2	
			HIST3H3; HIST1H3A; HIST2H3C; H3F3A; H3F3C	HIST3H3; H3; HIST2H3A/C/D; H3F3A; H3F3C		YRPGTVALR	−6.4	
	Jun	Y	JUN	Jun	§58, §63	AKNS^*^DLLTS^*^PDVGLLK	14.4	
					§63	NSDLLTS^*^PDVGLLK	16.1	
						NSDLLTSPDVGLLK	19.1	
			JUND	JunD	§90	ADGAPSAAPPDGLLAS^*^PDLGLLK	56.7	
						AAALKPAAAPPPTPLR	16.8	
						ADGAPSAAPPDGLLASPDLGLLK	182.4	
						KDALTLSLSEQVAAALKPAAAPPPTPLR	40.8	
			JUND; JUN	JunD; Jun	§100; §73	LAS^*^PELER	14.4	
	ATF	N	ATF2	ATF-2; ATF-2 iso 3; ATF-2 iso 5; ATF-2 iso 7	§69, §71; §69, §71; 51, 53; 51, 53	NDSVIVADQT^*^PT^*^PTR	8.1	
					§71; §71; 53; 53	NDSVIVADQTPT^*^PTR	23.5	
			ATF7	ATF7; ATF7 iso2 iso 2; ATF7 iso6 iso 3; ATF7 iso6 iso 4; ATF7 iso6 iso 6	§424, §434; 392, 402; 403, 413; 237, 247; 413, 423	TQGYLES^*^PKESSEPTGS^*^PAPVIQHSSATAPSNGLSVR	20.9	
					§424; 392; 403; 237; 413	TQGYLES^*^PKESSEPTGSPAPVIQH	7.1	
					§428, §434; 396, 402; 407, 413; 241, 247; 417, 423	TQGYLESPKES^*^SEPTGS^*^PAPVIQH	25.1	
					§429; 397; 408; 242; 418	TQGYLESPKESS^*^EPTGSPAPVIQH	7.1	
					§432; 400; 411; 245; 421	ESSEPT^*^GSPAPVIQHSSATAPSNGLSVR	213.7	
					§434; 402; 413; 247; 423	ESSEPTGS^*^PAPVIQHSSATAPSNGLSVR	213.7	
						ESSEPTGSPAPVIQH	20.8	
						ESSEPTGSPAPVIQHSSATAPSNGLSVR	2.5	
						SSATAPSNGLSVR	6.9	
				ATF7;ATF7 iso2 iso 2;ATF7 iso6 iso 3;ATF7 iso6 iso 4;ATF7 iso6 iso 6;ATF7 iso5 iso 5	§53; 53; 53; 53; 53; 53	TDSVIIADQTPT^*^PTR	80.0	
P38 MAPK signaling	P38 MAPK	Y	MAPK14	P38A; P38A iso2 iso 2; P38A iso2 iso 3; P38A iso2 iso 4; P38A iso2 iso 5	§180, §182; 180, §182; 180, §182; 180, §182; 180, §182	HTDDEM#T^*^GY^*^VATR	3.0	Phospho-p38 MAPK (Thr180/Tyr182) and p38 MAPK (total), no significant change (20).
					§182, §185; §182, 185; §182, 185; §182, 185; §182, 185	HTDDEM#TGY^*^VAT^*^R	3.0	

*N, Not exist in this category; Y, Exist in this category. §, the phospho site was confirmed by literature*.

*, *phosphorylation*,

*#, oxidized methionine*.

### mTORC1 Signaling and Canonical Wnt Pathway Are Activated in an NFPA

For mTOR signaling pathway (Figure 4A), PRAS40 was the pivotal modulator of the mTOR complex 1 (mTORC1**)**. The total protein expression level of PRAS40 and its phosphorylation level at residue Thr246 in PRAS40 were the switch of mTORC1 pathway to decide the initiation of the downstream protein synthesis and metabolism enhancement (24). Western blot analysis found that PRAS40 was significantly downregulated in NFPAs (Figure 4B), while the phosphorylation level at residue Thr246 in p-PRAS40 was significantly increased in NFPAs relative to controls. For canonical Wnt pathway (Figure 5A), GSK3α and GSK3β were key inhibitors of canonical Wnt pathway. The enhanced phosphorylation level at residue Ser21/9 in GSK3α/β would remove the inhibition of Wnt pathway. The total protein expression levels of GSK3α and GSK3β were decreased slightly in NFPAs relative to controls (Figure 5B). Whereas, the phosphorylation levels at residues Ser21 in p-GSK3α and Ser9 in p-GSK3β were significantly increased in NFPAs relative to controls. In addition, the main effector β-catenin in Wnt pathway was significantly upregulated in NFPAs compared to controls. Therefore, the expression and phosphorylation levels of GSK3α/β could reflect the state of canonical Wnt pathway. These Western blot experiments further validated IPA pathway-network analysis results and PTMScan experimental results; namely, the canonical Wnt pathway and mTORC1 signaling were activated in NFPAs, and contributed to NFPA pathogenesis.

## Discussion

Molecular network changes are the hallmark in human pituitary adenoma pathogenesis, which is involved in multiple molecule alterations in different levels of genes (genome), RNAs (transcriptome), proteins (proteome), metabolites (metabolome), and imaging features (radiome), and those different levels of molecules are mutually interacted. Multiomics data-based pathway network analysis benefits for the complete and comprehensive understanding of molecular mechanisms and discovery of reliable pathway-network-based biomarkers for pituitary adenomas. This study collected nine sets of omics data from different research groups in the world, including DEG and DEP data in NFPAs compared to controls, DEG and DEP data in invasive NFPAs compared to non-invasive NFPAs, nitroproteins in NFPAs, mapping proteins in NFPAs, and mapping proteins/nitroproteins/phosphoproteins in control pituitaries. Each omics data was performed by pathway-network analysis and related bioinformatics analysis, and hub-molecules were identified from pathway-networks. Those pathway networks derived from mapping proteins in NFPAs and mapping proteins/nitroproteins/phosphoproteins in controls were the base line data, which provided the reference for determination of reliable NFPA-related pathway-networks. Those pathway networks derived from DEGs and DEPs in NFPAs and invasive NFPAs, and nitroproteins in NFPAs, were directly associated with NFPAs pathophysiological changes. Thus, a total of 62 molecular networks and 861 hub-molecules were identified. According to the primary functions of 861 hub-molecules, 42 hub-molecule panels that were grouped into 16 functional categories were identified (Supplemental Figure 2); and 57 high-frequency hub-molecules were identified. Moreover, among 519 statistically significant canonical pathways derived from nine sets of omics data, 54 significantly cancer-related canonical pathways were identified to involve differentially expressed hub-molecules (DEGs, or DEPs), and were further grouped into 9 canonical-pathway panels (Table 2). Many of these altered canonical pathways interact with each other through hub-molecules to form pathway networks. Comprehensive analysis of all these networks, hub-molecule panels, cancer-related canonical pathways, and canonical pathway panels, the PTMScan experiment (multiple antibodies-based enrichment and LC-MS/MS) that contained 1006 phosphorylated sites with 409 proteins within 19 important signaling pathways were carried out in NFPAs compared to control pituitaries, which confirmed the important pathway-networks and the corresponding hub-molecules in NFPAs and further investigate the functional roles of those pathway network and hub-molecules, including PI3K/Akt signaling, mTOR signaling, Wnt pathway, NFκB signaling, apoptosis-regulated pathways, ERK/MAPK signaling, p38 MAPK, and JNK pathways, and the corresponding hub-molecule changes (Table 3). Furthermore, differentially expressed hub-molecules and differentially phosphorylated hub-molecules in mTOR pathway and Wnt pathway were confirmed and validated with immunoaffinity Western blot in NFPAs compared to control pituitaries (Figures 4B, 5B). One might note that those important pathway-systems and hub-molecules were not directly further confirmed and validated in invasive NFPAs compared to non-invasive NFPAs to identify NFPA invasiveness-related pathways and hub-molecules, because there were not enough clinical information in our laboratory to distinguish those NFPA samples used for PTMScan experiment into invasive and non-invasive groups. However, NFPA samples used for PTMScan experiment included invasive and non-invasive NFPA tissues. Those solid data including 42 hub-molecule panels, 57 high-frequency hub-molecules, 9 canonical-pathway panels, and PTMScan and Western blot-valued pathway-systems and hub-molecules in NFPAs provided overall and large-scale pathway-network alteration profiles for NFPAs, which benefited for discovery of novel drug targets and tumor molecular biomarkers, and development of more specific and comprehensive diagnosis and target treatment strategies for NFPAs.

Based on multi-omics data, PTMScan experimental data, and immunoaffinity Western blot data of key hub-molecules within important signaling pathway systems, PI3K/Akt signaling pathways (mTOR signaling, Wnt pathway, NFκB signaling, and apoptosis-regulated pathways), and MAPK signaling pathways (ERK/MAPK signaling, and p38 and JNK pathways), were significantly associated with NFPA pathogenesis, and were further discussed in details below.

### PI3K/Akt Signaling Pathways Were Activated in NFPAs

The PI3K/Akt cascade is an essential downstream effector of many protein**-**kinase signaling pathways, including receptor tyrosine kinases (RTKs) and G protein-coupled receptors (GPCRs), and its excessive activation often leads to genomic instability, tumor formation, progression, angiogenesis, and multidrug resistance (25). This study found that many abnormal protein expressions in the PI3K/Akt canonical pathway and mTOR pathway, including upregulated SHIP, GAB1/2, SHC, SOS, AKT, IKK, NFkB, MEK1/2, ERK1/2, mTOR, 4E-BP1, GSK3, and β-catenin, and downregulated PI3K p85, HSP90, and BAD, in PI3K/Akt pathway (Figure 3); upregualted ERK1/2, AKT, RSK, mTOR, PRAS40, RICTOR, 4EBP, RPS6, and 40S ribosome, and downregulated PKC, PKCa, and p-4EBP in mTOR pathway (Figure 4). These data indicated multi-level dysfunctions in PI3K/Akt signaling pathway in NFPA. Moreover, mTOR signaling abnormality was found with abnormally expressed proteins RSK, mTORC1 that regulated ATG13 and 40S ribosomes, and RICTOR in mTORC2 (Figure 4A). These findings demonstrated that mTOR pathway as the downstream signaling of PI3K/Akt was also dysregulated. Furthermore, the abnormally expressed 14-3-3 and HSP90 in PI3K/Akt signaling pathway played an important role in the aberrantly activated PI3K/Akt signaling.

Akt is also known as PKB, a serine/threonine kinase, and belongs to protein kinase A/G/C family. Akt modulates many important cellular processes. Aberrant activation of Akt often leads to multiple diseases including cancers (26). Three Akt isoforms (Akt1, Akt2, and Akt3) exist in mammals, and phosphorylation at two residues Thr308 and Ser473 in Akt1, Thr309 and Ser474 in Akt2, and Thr304 and Ser472 in Akt3 are needed for their full activation. The residues Thr308 in Akt1, Thr309 in Akt2, and Thr304 in Akt3 were located in the activation loop, and were phosphorylated by PDK1. Whereas, the residues Ser473 in Akt1, Ser474 in Akt2, and Ser472 in Akt3 were located in C-terminal regulatory domain, and were regulated by mTORC2, which were critical to stabilize their active conformation (27). This study found that Akt1, Akt2, and Akt3 were upregulated in NFPAs relative to controls, and phosphorylation levels at residues Thr308 in Akt1, Thr309 in Akt2, and Thr305 and Ser472 in Akt3 were upregulated in NFPAs relative to controls, which demonstrated Akt was overexpressed and fully activated in NFPAs. Furthermore, Akt can regulate the functions of more than 100 substrates in a cell, which suggests a possibility for PI3K/Akt to build crosstalk with other pathways, including ERK, JNK, p38, NFκB, and Wnt signaling (26). This study demonstrated that the activated PI3K/Akt signaling was substantially involved in stimulation of multiple cascades in NFPAs through the following pathways (Figure 3): (i) The activated Akt phosphorylated TSC2 and residue Thr246 in PRAS40 to inactivate these proteins and subsequently stimulated mTORC1 signaling; (ii) The functional loss of Akt consequentially activated canonical Wnt pathway by phosphorylation at residue Ser21 in GSK3α, and Ser9 in GSK3β; (iii) Akt activated NF-κB signaling through phosphorylating the upstream IκB kinase α; (iv) Akt blocked pro-apoptotic activity of BAD through phosphorylation and promoted cell survival; (v) PI3K/Akt signaling could interact with ERK, JNK, and p38 signaling from multiple levels; and (vi) Akt lead to degradation of the transcription factors NFAT and inhibited migration and invasion of a cancer.

### The mTOR Signaling Pathway Was Activated in NFPAs

The mTOR was a conserved serine/threonine kinase, and belongs to the PI3K superfamily, which can integrate multiple signals such as pressure, oxygen content, nutrient availability, and mitogenic signals to regulate growth and homeostasis. The mTOR functioned in two functionally distinct complexes, including mTORC1 (mTORC1), and mTOR complex 2 (mTORC2) (Figure 4A). The core components mainly included mTOR, Raptor, PRAS40, and mLST8 in mTORC1, and mTOR, RICTOR, mSIN1, and mLST8 in mTORC2 (24). When stimulated by stress, oxygen, nutrition, energy and growth factors, mTORC1 regulated protein translation, autophagy and metabolism including adipogenesis, ketone formation and glucose homeostasis. The mTORC2 that can be activated by extracellular growth factors phosphorylated downstream kinases such as Akt, PKC and SGK to enhance signal cascade and regulate biological effects including cell survival, cytoskeleton, and metabolism (28). The activated mTORC1 phosphorylated S6K and eIF-4E binding proteins (4E-BPs), and mTORC2 was necessary for the maximal activation of Akt through phosphorylation of its residue Ser473 (29). Upon stimulation, mTOR was commonly phosphorylated at its residues Thr2446, Ser2448, and Ser2481, and the region of its residues 2430–2450 was important for function regulation of mTOR (29). The residue Ser2448 in mTOR was phosphorylated by S6K, and this phosphorylation increased associations of mTOR with Raptor in mTORC1 and Rictor in mTORC2. The phosphorylation status of mTOR Ser2448 was correlated with mTORC1 activity (30). In this study, PTMScan results demonstrated that phosphorylation levels at residues Thr2446 or Ser2448 in mTOR were significantly increased, which demonstrated that mTOR was activated in NFPAs, and contributed to the initiation and development of NFPAs. As a substrate, AKT-phosphorylated PRAS40 negatively regulated mTORC1 Rheb-GTP-dependent activation in normal state. When residue Thr246 in PRAS40 was phosphorylated by Akt, PRAS40 could not inhibit the activity of mTORC1 (24), thus activated mTORC1 phosphorylated downstream eIF4E-binding proteins (4E-BPs) and ribosomal S6 kinase (p70RSK) to enhance protein synthesis and regulate energy metabolism (31). Moreover, this study found that phosphorylation of residue Thr246 in PRAS40 was significantly increased in NFPAs with Western blot and PTMScan experiment, while the overall expression of PRAS40 was significantly decreased in NFPAs. These results demonstrated that the suppressions of PRAS40 on mTORC1 were dramatically relieved in NFPAs.

There are three kinds of 4E-BPs, including 4E-BP1, 4E-BP2, and 4E-BP3 in mammals. In quiescent state, 4E-BPs tightly interact eIF4E to prevent the initiation of translation (24, 32). Phosphorylation at residues Thr37 and Thr46 in 4E-BP2 by mTOR significantly decreased the affinity capability between 4E-BP2 and eIF4E by100 folds (33). 4E-BP3 had similar functional characteristics with 4E-BP1 and 4E-BP2 (34). In this study, PTMScan experiment found that many peptides derived from 4E-BPs were significantly increased in NFPAs, and phosphorylations at residues Thr37 and Thr46 in both 4E-BP1 and 4E-BP2, and Thr23 in 4E-BP3 were increased in NFPAs. The increased phosphorylation in 4E-BPs benefited for disassociation of 4E-BPs from eIF4E to promote the initiation of protein translation in NFPAs. Moreover, the highly conserved phosphorylation at residues Ser235, Ser236, Ser240, Ser244, and Ser247 in 40S ribosomal protein S6 played an important role in protein translation initiation. These phosphorylations especially at residues Ser235 and Ser236 in 40S ribosomal protein S6 could effectively facilitate the assembly of translational pre-initiation complex (35). In this study, PTMScan experiments found that phosphorylations at residues Ser235, Ser236, Ser240, Thr241 and Ser244 in 40S ribosomal protein S6 were obviously increased in NFPAs. In addition, Rictor was a scaffolding protein to regulate the localization, assembly, and substrate binding in mTORC2, and its overexpression was highly related to metastatic process (36). PTMScan experiment found the upregulated Rictor in NFPAs and increased phosphorylations at residues Ser472 in Akt3, which suggested that activation of mTORC2 contributed to the tumor progression in NFPAs.

Those findings clearly demonstrated that mTOR signaling, including mTORC1 and mTORC2 complexes, was involved in NFPA pathogenesis, including cell survival, protein synthesis, and metabolism.

### Wnt Pathways Were Involved in NFPAs

Wnt pathways are pivotal to modulate many cellular and physiological processes such as cell polarity, motility, adhesion, proliferation, survival, stem cells self-renewal, and tissue homeostasis, and include canonical and non-canonical Wnt pathways according to different ligands and downstream effectors (37) (Figure 5A).

#### Canonical Wnt Pathway Was Activated in NFPAs

Canonical Wnt pathway mainly regulated the stability of β-catenin to modulate transcriptions of Wnt-targeted genes, and control cell fate, growth and differentiation (38). (i) β-catenin was the key effector in canonical Wnt pathway. There was a destruction complex containing two kinases [glycogen synthase kinase 3 (GSK3) and casein kinase I (CKI)], and two scaffold proteins [adenomatous polyposis coli (APC) and Axin 1/2] that controlled the stability of cytoplasmic β-catenin through phosphorylation and ubiquitylation on specific sites and then degraded it through proteasome. Without Wnt ligand stimulation, the destruction complex could keep cytoplasmic β-catenin in a low level (37). When canonical Wnt pathway was activated by Wnt ligand, the destruction complex was inhibited by the activated disheveled-protein (DVL or DSH), which lead to the cellular accumulation and nuclear import of β-catenin (39). In the nucleus, β-catenin acted as a co-activator for T-cell factor/lymphoid enhancer factor (TCF/LEF), and then recruited various transcriptional cofactors including B-cell CLL/lymphoma 9 protein (Bcl9), Pygopus (PYGO), CREB-binding protein (CBP), and histone acetyltransferase p300 (p300) to activate transcription of target genes such as FGF20, JUN, MYC, and CCND1. In order to degrade β-catenin, β-catenin should be initially phosphorylated at its residue Ser45 by CK1, and subsequently be phosphorylated at its residues Ser33, Ser37, and Thr41 by GSK3. The E3-ubiquitin ligase β-TrCP could bind to a short region containing phosphorylated residues Ser33 and Ser37 in β-catenin to result in its followed ubiquitylation and degradation (40); and phosphorylation at N-terminal in β-catenin regulated its degradation, while phosphorylation at C-terminal in β-catenin regulated its function. Phosphorylation at residues Ser552 and Ser675 in β-catenin, which was regulated by PI3K/Akt, Camp/PKA and/or GPCR/PKD, caused β-catenin to be accumulated in nucleus for stimulating its transcriptional activity to increase expressions of cyclin D1 and c-Myc (41, 42), and enhanced the ability of β-catenin to recruit many transcriptional coactivators such as CBP or TBP (TATA binding protein) to bind to its C-terminal tail (43). In this study, PTMScan experiment found that all detected peptides derived from β-catenin were dramatically elevated about 2.5 to 65.3-fold. No phosphorylation was found at residues Ser33, Ser37, Th41, and Ser45 in β-Catenin, which indicated that N-terminal of β-catenin was barely phosphorylated, thus degradation complex might be inhibited. However, PTMScan found that phosphorylations at residues S552 and S675 in β-catenin C-terminal were significantly increased, which might improve stability and transcriptional functions of β-catenin. These results were also confirmed by Western blot in its overall expression level of β-catenin (Figure 5). (ii) GSK3 was a highly conserved and multifunctional serine/threonine kinase to participate in various cellular processes (44), which was inactivated through phosphorylating residues Ser21 in GSK-3α and Ser9 in GSK-3β. In this study, Western blot found slightly decreased expression levels of GSK3α and GSK3β, and increased phosphorylation levels at residues Ser21 in GSK3α and Ser9 in GSK3β in NFPAs compared to controls; and PTMScan experiment also found increased phosphorylation at residue Ser21 in GSK3α. These findings clearly demonstrated that GSK3α was inhibited in NFPAs, which might cause differential expressions of many target genes that regulate apoptosis, proliferation, differentiation, and motility. (iii) Bcl9 was an important downstream transcriptional co-activator, which can augment and diversify the transcriptional output of canonical Wnt pathway. Bcl9 could also modulate interactions of β-catenin to improve EMT and invasion, and was associated with poor outcome in cancer (45). In this study, PTMScan experiment found the increased level of Bcl9 in NFPAs, and upregulations of many target genes such as c-Myc and c-Jun in canonical Wnt pathway. Moreover, some studies clearly demonstrated that β-catenin was accumulated in the nucleus of NFPAs (22), several Wnt target genes such as Cyclin D1 and c-MY were upregulated in NFPAs (21, 22), and the inhibitor of Wnt pathway including Wnt inhibitory factor 1 (WIF1) and secreted frizzled related protein (sFRP) were significantly decreased in NFPAs (22, 46). All these data revealed that canonical Wnt pathway was activated in NFPAs to participate in cell apoptosis, proliferation, differentiation, and motility.

#### Non-canonical Wnt Pathway Might Be Involved in Regulation of NFPAs

Non-canonical Wnt pathways were independent of β-Catenin, and involved many downstream signal transduction effectors, including multiple small GTPases (RAC, RHOA, and CDC42), G proteins, calmodulin/calcium, PKC, Src, and JNK (37). Among them, Wnt/planar cell polarity (Wnt/PCP) pathway and Wnt/Ca^2+^ pathway had been characterized (47) (Figure 5A). Wnt/PCP pathway mainly engaged GTPases to activate downstream targets such as JNK or rho-related kinase ROCK, which played important roles in reconfiguration of cytoskeleton, cell movement, polarity, and patterning in tissue (37, 38). When activated by a Wnt ligand, Wnt/Ca^2+^ pathway exerted its function by activating G proteins, phospholipase C (PLC) and phosphodiesterase (PDE), then invoked calcium-sensitive enzymes such as calcium–calmodulin-dependent kinase II (CaMK II) and PKC, which subsequently activated the corresponding transcription factor NFAT and CDC42 to cause a wide range of cellular effects, including cell adhesion, migration, inflammation, and tumorigenesis (47). In addition, non-canonical Wnt pathways could regulate canonical Wnt pathway through GTPases, PKC or other mechanisms. PKC especially PKCa could negatively regulate Wnt/β-catenin pathway through directly phosphorylating residues Ser33, Ser37, and Ser45 in β-catenin (48). This study found that JNK, Jun, ATF2, NFAT, PLC, PKC, CDC42, and NFκB were differentially expressed in non-canonical Wnt pathway in NFPAs compared to controls, which demonstrated non-canonical Wnt pathways were closely related to NFPAs. Moreover, this study also found that Rho/Rac-related proteins such as ARHGAP18 and ARHGEF17 were upregulated in NFPAs, and that canonical pathways were involved in cytoskeleton rearrangement and cell mobility, including Gaq signaling, RhoGDI signaling, Rho family GTPase signaling, and RhoA signaling, and in calcium modulation-related signaling, including calcium signaling, and calcium-induced T lymphocyte apoptosis. Those findings indicated that PCP signaling and calcium-related signaling might be involved in NFPA tumorgenesis. Also, Ephs and Ephrins signaling regulated activation of Rho GTPases such as RAC, RHO, CDC42, and JNK through PCP signaling (37). Ephrin B and Ephrin receptor signaling were identified as canonical pathway in this study, with upregulations of Ephrin B and Ephrin receptor in NFPAs, which means Ephrin B and Ephrin receptor signaling were activated in NFPAs. In this study, PTMscan experiment found most (80%) peptides derived from cPKCs were decreased in NFPAs. Only one peptide derived from PKCα was increased and its phosphorylation at residue Ser651 in PKCα was not involved in its own activation. All peptides derived from PKCδ are decreased in NFPAs, which means the decreased function of PKC to attenuate inhibition of β-catenin and improve apoptosis-resistance in NFPAs. Therefore, dysregulated non-canonical Wnt pathway might reduce inhibition of canonical Wnt pathway, and be involved in NFPA pathogenesis.

### NFκB Signaling Was Provoked in NFPAs

Nuclear factor kappa B (NFκB) family members were critical transcription factors, and were involved in numerous cellular processes including cancer (49). After cellular stimulation, phosphorylation of IkBs by IκB kinase (IKK) complex resulted in ubiquitination and subsequent degradation to cause activation of NFκB signaling. The IKK complex was composed of two catalytic IκB kinases (IKKα and IKKβ) and one scaffold/adaptor protein IKKγ (49). Phosphorylation at residue Ser376 in IKKγ was required for signal dependent activation of IKKβ and regulated NFκB signaling. In this study, PTMScan experiment found the increased phosphorylation at residue Ser376 in IKKγ and the upregulated NFκB in NFPAs compared to controls, which indicated the activation of NFκB signaling in NFPAs. Moreover, NFκB signaling and Wnt pathway had crosstalk in multiple levels and modulated the activities and functions of other signaling pathways (50), such as the activation of NFκB signaling could infect Wnt pathway in NFPAs.

### Apoptosis-Regulated Pathway Was Involved in NFPAs

BAD was a proapoptotic member in Bcl-2 family, and was phosphorylated by many upstream kinases to control apoptosis. BAD could inhibit Ras/MEK/ERK and JNK signaling, and EMT. Studies found that downregulated p-BAD and BAD in cancer cells promoted tumor invasion and migration (26, 51). The dysregulated expressions and phosphorylation of BAD might lead to imbalance of programmed cell death and immortalized cancer cells. In this study, PTMScan experiment found most of peptides derived from BAD were dramatically decreased to cause significantly reduced binding among BAD, Bcl-2, and Bcl-xL, and promote cell survival and invasion in NFPAs. Thus, PI3K/Akt signaling and its related pathways including *mTOR*, Wnt, and NFκB signalings were dysregulated in NFPAs, and multiple molecules in these signaling pathways were further abnormally modulated in invasive relative to non-invasive NFPAs.

### MAPK Pathways Were Dysregulated in NFPAs

MAPKs can regulate many biological processes, including proliferation, apoptosis, stress responses, and immune defense, and include four independent MAPK cascades: Extracellular signal-regulated kinase1/2 (ERK1/2) pathway (canonical ERK/MAPK pathway), c-Jun N-terminal kinase (JNK) pathway, p38 pathway, and ERK5 pathway (52) (Figure 6). In this study, mRNA expressions of PI3K, Talin, and cPLA2 in NFPA DEG data were significantly upregulated in ERK/MAPK signaling (PI3K: 2.26-fold; Talin: 2.9-fold; and cPLA2: 2.44-fold), while MKP1/2/3/4 was significantly downregulated (MKP2: −2.52 fold). For NFPA DEP data, FYN was significantly upregulated (3.9-fold), and 14-3-3 (β, γ, θ, η, ζ) and HSP27 were downregulated (14-3-3: −1,000-fold; HSP27: −4.7-fold). For invasive NFPA DEG data, PI3K, PKA, PP1/PP2A, 14-3-3 (β, γ, θ, η, ζ), MSK1/2 and ER were expressed abnormally [PI3K: −1.85-fold; PKA: −1.56-fold; PP1/PP2A (PPM1A −1.85-fold; PPM1K 1.66-fold; PPP1R11 1.59-fold); 14-3-3 (2.06-fold); MSK1 (−1.92-fold); and ER (ERα 2.12-fold; ERβ −3.7-fold)]. The abnormal expressions of key molecules in ERK/MAPK signaling pathway in NFPAs clearly demonstrated that ERK/MAPK signaling were dysregulated in NFPAs, and could intensely promote occurrence and development of an NFPA.

#### ERK/MAPK Signaling Was Stimulated at Multiple Levels in NFPAs

Extracellular signals were transducted to intracellular targets through phosphorylation cascade reactions in Ras-Raf-Mek-ERK1/2 activation pattern. The ectopic activation of this signaling could result in tumorigenesis, progression, and metastasis; and Ras-Raf-Mek-ERK1/2 signaling can interact with Ras-PI3K-Akt signaling to amplify signal regulation range and mutually modulate tumorigenesis (53). (i) In mammalian cells, Raf family includes A-Raf, B-Raf, and C-Raf (Raf-1). The constitutive phosphorylation at residues Ser446 and/or Ser447 and the presence of two aspartates acids at residues 448/449 in B-Raf promoted its activation, and activated ERK/MAPK pathway. In this study, PTMScan experiment found that phosphorylations at residues Ser446 or Ser447 in B-Raf were dramatically increased over 1,000-fold in NFPAs, which clearly demonstrated that B-Raf was utmostly invoked in NFPAs to constantly abnormally activate ERK/MAPK pathway. Thus, B-Raf might act as one of potential biomarkers or therapy targets for NFPA patients. (ii) Phosphorylations at residue Ser218 and Ser222 in human MEK1 were needed for its full activation. MEKs modulated the activation of ERKs through phosphorylations at residues Thr202 & Tyr204 and Thr185 & Tyr187 in human ERK1 and ERK2 in their activation loop Thr-Glu-Tyr motif (54). In this study, PTMScan experiment found phosphorylations at residues Ser218 or Ser222 in MEK1 were significantly increased over 300-fold in NFPAs, and phosphorylations at residues Thr202 in ERK1 and Tyr187 in ERK2 were also significantly increased in NFPAs. These data revealed that the phosphorylation cascade in ERK/MAPK pathway was full stimulated in NFPAs. (iii) As bispecific protein phosphatases, MKP proteins can inactivate the phosphorylated MAPK through dephosphorylation at residues Ser and Tyr of TXY motif. Among them, MKP1 and MKP2 can dephosphorylate ERK, JNK, and p38, which negatively regulated MAPK cascades (55). Phosphorylation at residues Ser359 and Ser364 in MKP1 by ERK1 and ERK2 enhanced MKP1 stability and protected it from proteasome-mediated degradation (56). Therefore, downregulated expressions of MKP1 and MKP2 might attenuate their inhibitory effect to promote tumorigenesis. Moreover, MKP1 with pSer359 was reduced, and MKP2 was decreased in NFPA DEG data, these findings demonstrated that the inhibition of MKPs to ERK1/2, JNK and p38 are decreased to further augment the MAPK signaling in NFPAs. (iv) PPM1A was a member of the protein phosphatase 2C family and an important tumor suppressor, which was involved in regulation of multiple pathways, such as TGFβ/Smad, JNK/p38, Cdk2, Cdk6, and Akt/ERK signaling (57). Its low expression can enhance NF-κB-dependent tumor invasion, tumor poor differentiation and prognosis. The invasive NFPA DEG data demonstrated that the downregulation of PPM1A might contribute to tumor invasion of NFPAs. Moreover, PPM1K was a highly conserved serine/threonine protein phosphatise, and mainly targeted mitochondrial matrix to regulate mitochondrial permeability transition pore (MPTP), which played important roles in cell survival, and nervous system development (58). The invasive NFPA DEG data found that PPM1K was increased in NFPAs. (v) Human p90 ribosomal S6 kinases (RSKs) are a family of Ser/Thr kinases that comprises of four isoforms (RSK1–4) and two structurally related homologs of RLPK (MSK1) and RSKB (MSK2) (59). RSKs are directly phosphorylated by ERK1/2 and PDK1, and RSKs are potently activated by ERK1/2 and p38 pathways to regulate various biological processes through phosphorylating numerous transcription factors, such as CREB, CBP, p300, SRF, c-Fos, ETV1, estrogen receptor-α (ERα), NF-κB, and NFATc4. Moreover, RSKs inhibit the tumor-suppressor TSC2 through phosphorylation at its residue Ser1798. MSKs predominantly resided in the nucleus and were required for phosphorylating histone H3, CREB, ATF1, and HMG-14. Phosphorylations at residues Ser221 in RSK1-3 and Ser376 in MSK1 were required for its catalytic activity (60). In this study, PTMScan experiment found phosphorylations at residues Tyr220, Ser221 and Thr225 in RSK1, Tyr226, Ser227, and Thr231 in RSK2, and Tyr231, Ser232, and Thr236 in RSK4 were significantly increased in NFPAs. Moreover, phosphorylations at resides Ser376 in MSK1 and Ser360 in MSK2 were also increased in NFPAs. Those data clearly demonstrated most of RSK family members were overexpressed and activated in NFPAs. (vi) Focal adhesion kinase (FAK) was a cytoplasmic non-receptor tyrosine kinase (61). When FAK was stimulated by integrin, FAK was autophosphorylated at its residue Tyr397 to result in subsequent phosphorylations at residues Tyr576 and Tyr577, and Tyr861 and Tyr925 in the C-terminal domain of FAK by Src. The activations of FAK and Src can exert their catalytic activities through promoting gene expressions of VEGF and MMPs (61). In this study, PTMScan experiment found that phosphorylations at residues Thr575, Tyr576, and Tyr577 in FAK are significantly increased in NFPAs (Table 3), which clearly demonstrated that FAK was strongly activated in NFPAs to further stimulate multiple cascades including Akt, MAPKs, p53, VEGF, and IGF-1 pathways in NFPAs. (vii) Many proteins associated with modulation of MAPK signaling were also downregulated in NFPAs, including SHC, SOS, PLC, PAK, HSP27, c-Myc, NFATc, histone H3, and Jun. In this study, PTMScan experiment found that peptides derived from SHC, SOS, PLC, PAK, c-Myc, Jun, ATF2, ATF7, GRB2-associated-binding protein 2 (GAB2), and Myc target protein 1 (MYCT1) were significantly increased in NFPAs, whereas derived from HSP27, NFATc, and Histone H3 were downregulated in NFPAs. Therefore, those findings clearly demonstrated that ERK/MAPk signaling pathway was changed in multiple levels in NFPAs.

#### The p38 and JNK Pathways Were Involved in NFPAs

p38 signaling primarily participated in responses to environmental stress, and also answers to immune response and inflammation. p38 MAPKs included four homologous members p38α, p38β, p38γ, and p38δ. p38α and p38β were ubiquitously expressed in human body, whereas p38γ and p38δ were restricted expressed in muscle, and lung and kidney, respectively (62). Upon stimulation, p38 MAPKs controlled cell fate by regulating activities of heat shock proteins and transcription factors (ATF2, CHOP, ELK1, and MEF2C). In addition, p38 signaling was also involved in modulation of eIFs function and protein synthesis (62). p38a was activated through dual phosphorylations at its residues Thr180 and Tyr182 in the activation loop Thr-Gly-Tyr motif (54). Another important stress-activated MAPK cascade was JNK signaling that has been involved in cell fate decisions responding to various stress stimulations. JNK regulated and activated the functions of its targets including transcription factors (Elk1, c-Myc, c-Jun, JunB, ATF2, and p53), and factors related to cell death such as the members of Bcl-2 family to modulate many cellular processes (54). JNK family included JNK1, JNK2, and JNK3. JNK1 and JNK2 were ubiquitously expressed in human body, and JNK3 was mainly expressed in the brain (63). JNKs were activated by phosphorylations at residues Thr183 and Tyr185 in JNK1 and JNK2, and Thr221 and Tyr223 in JNK3. Moreover, JNK and p38 cascades shared synergistically many components and functions. JNK and p38 contributed to AP-1 activities through phosphorylations at residues Thr69 and Thr71 in ATF2, and these two phosphorylations in ATF2 enhanced its histone acetyltransferase (HAT) activity (64). JNK and p38 played pivotal roles in coordination of immune and inflammatory responses through various cytokines including interleukin-1 (IL-1), IL-10, IL-12, and tumor necrosis factor α (TNFα). Cytokines, especially TNFα, contributed to the generation of reactive oxygen species/reactive nitrogen species (ROS/RNS) (65) to activate JNK and p38 signaling, thus continuously activated JNK and p38 signaling facilitated abnormal synthesis of ROS and chronic inflammation (63). In this study, PTMScan experiment found that phosphorylations at residues Thr180 & Tyr182 and/or Tyr182 & Thr185 in p38a, Thr183 & Tyr185 in JNK1, Thr221 & Tyr223 in JNK3, Thr183 & Tyr185 and/or Thr175 & Tyr185 in JNK2, and Thr69 & Thr71 in ATF2 were significantly increased in NFPAs. Those results strongly supported that activations of JNK and p38 signaling were involved in development of NFPAs, and were most likely responsible for the high oxidative stress state, immune and inflammatory disorders in NFPAs.

This present study used the systems biology opinion to find out the key pathological mechanisms commonly existing in NFPA through Meta analysis. The IPA system was used to further analyze different omics data of NFPA and extract important data such as molecular networks, canonical signaling pathways, and high-frequency hub molecules closely related to the occurrence and development of NFPAs, providing effective data to promote individualized precise treatment. Many studies have shown that targeting some high-frequency hub-molecules in Table 1 has achieved good results in other types of pituitary adenomas. For example, TGFβ1 is used as a novel therapeutic target to treat resistant prolactinomas (66); the microRNA-145 inhibits the activation of the mTOR signaling by targeting AKT3 to suppress the proliferation and invasion of invasive pituitary adenoma cells (67); lncRNA H19 inhibits mTORC1 by disrupting 4E-BP1/Raptor interaction in pituitary tumors (68); and MAPK Pathways act as therapeutic targets in pituitary tumors (69). Because PI3K/Akt/mTOR and ERK/MAPK signaling pathways are significantly dysregulated in NFPA, and these two essential signaling pathways are not only related to rapid proliferation and apoptosis resistance of tumor cells, but also can regulate the activities of many other pathways and then regulate tumor growth in the multiple levels. Therefore, these two pathways can act as the most convenient and effective therapeutic target in pituitary adenomas. Moreover, since NFPA is a multifactorial and multifaceted disease, it is reasonable to infer that the combination therapy targeting multiple pathways and hub-molecules based on the patient's tumor molecular subtype can achieve better therapeutic results. Researchers have shown that the combination treatment targeting AMPK and PI3K/Akt/mTOR pathway at the same time in GH-secreting pituitary tumors achieved a better treatment effect than the single medication alone (70). This study found that AMPK was a high-frequency hub-molecule in NFPA, and PI3K/Akt/mTOR signaling pathway was highly maladjusted in NFPA. Thus, has laterally proved that this kind of drug combination also achieved a good therapeutic effect in NFPA. In future studies, multiple combinations of drugs targeting different high-frequency hub-molecules and important signaling pathways would provide new hope for improving the therapeutic effects of NFPAs.

## Conclusions

Pituitary adenoma is a common pituitary disease, with a series of molecule alterations in DNAs (genome), RNAs (transcriptome), proteins (proteome), metabolites (metabolome), and imaging characteristics (radiome) that resulted from exogenous and endogenous carcinogens, and those molecules associate mutually and function in a molecular network system. Molecular network-based molecule pattern has important scientific merits in clarification of molecular mechanisms and discovery of effective biomarkers and therapeutic targets for pituitary adenomas. This study used the Meta-analysis strategy and integratively analyzed all documented NFPA omics data (a total of nine sets of omics data) with IPA pathway network program. A total of 62 molecular-networks and 519 canonical-pathways were revealed with statistical significance from nine sets of NFPA omics data. A total of 861 hub-molecules were derived from those molecular-networks and were classified into 42 hub-molecule panels to associate with pituitary tumorigenesis. A total of 139 canonical-pathways were found from at least two sets of omics data, which generated 68 cancer-related canonical-pathways to obviously associate with the occurrence and development of tumor, and of them 54 canonical pathways involved in any DEGs or DEPs were divided into 9 canonical-pathway panels according to the similar cellular functions and biological processes. Those molecular networks, hub molecules, hub-molecule panels, canonical pathways, and canonical-pathway panels formed the overall pathway-network characterization of NFPAs. The important pathway-networks and hub-molecules were further validated and in-depth studied with PTMScan experiments and immunoaffinity Western blot analysis to quantify the alterations in the protein expressions and specific phosphorylation status. Comprehensive analysis of all data including multi-omics data, PTMScan experimental data, and immunoaffinity Western blot data revealed several important signaling pathway systems that operate in NFPA biological system, including PI3K/Akt signaling pathways (mTOR signaling, Wnt pathway, NFκB signaling, and apoptosis-regulated pathways), and MAPK signaling pathways (ERK/MAPK signaling, and p38 and JNK pathways). These findings are the solid scientific evidence and molecular targets to discover molecular-network-based biomarkers and effective therapeutic targets for the accurate diagnosis and treatment of the different types and different development stages of NFPAs. Here we specially emphasize that, NFPAs are very complex diseases, involving a series of molecule changes and pathway-network changes. One should change our thinking and working models from mono-targeting pharmacological treatment concept to the multi-targeting pharmacological treatment concept, from mono-molecule biomarker to multi-molecule-panel biomarker for insights into its molecular mechanism, patient stratification, diagnosis, and prognostic assessment. This present multi-omics data exactly offer the scientific data for multi-targeting pharmacological treatment concept and multi-molecule-panel biomarker for pituitary adenomas including NFPAs.

## Data Availability Statement

All datasets generated for this study are included in the article/Supplementary Material.

## Ethics Statement

Pituitary adenoma tissue samples were obtained from Department of Neurosurgery, Xiangya Hospital, Central South University, and were approved by Xiangya Hospital Medical Ethics Committee of Central South University. Control pituitary glands were post-mortem tissues obtained from the Memphis Regional Medical Center, and were approved by University of Tennessee Health Science Center Internal Review Board (UTHSC-IRB). The written informed consent was obtained from each patient or the family of control pituitary subject, after full explanation of the purpose and nature of all used procedures.

## Author Contributions

YL analyzed the documented omics data, IPA-mined result data and PTMScan experimental result data, performed Western blot experiments, and wrote partial manuscript draft. ML and TC participated in Western blot experiments. XiaoZ participated in the critical revision. XianZ conceived the concept, designed the entire project, collected the documented omics data, performed IPA analysis, designed and coordinated the entire experiments, designed and wrote manuscript, critically revised the manuscript, trained YL regarding omics, systems biology, molecular networks, and pathway analysis, and was responsible for its financial supports and the corresponding works. All authors approved the final manuscript.

### Conflict of Interest

The authors declare that the research was conducted in the absence of any commercial or financial relationships that could be construed as a potential conflict of interest.
