# Supplementary material for: Multiomics-Based Signaling Pathway Network Alterations in Human Non-functional Pituitary Adenomas
Source: Front Endocrinol (Lausanne). 2019 Dec 17;10:835. doi: 10.3389/fendo.2019.00835 (PMC6928143; doi:10.3389/fendo.2019.00835)
Supplement: Supplementary file 1 [file Presentation_1.zip › Supplemental Figure 2_v1.pdf]

Supplemental Figure 2

Functional categories based on 42 hub-molecule panels derived from molecular networks in NFPA

Supplemental figure 2A
Category A includes 5 hub-molecule panels, and mainly functions in cell movement, angiogenesis, invasion, and metastasis.

(i) Actin and F-actin were present in NFPA DEGs (Dataset 1) and invasive DEGs (Dataset 5) groups in the Cytoskeleton associated proteins classification. Both of them appear 3 times in the NFPA groups (includes 6 Datasets in NFPA, if no special instructions), indicating that expression of molecules involved in cytoskeletal organization in NFPA is deregulated, leading to transforms in cell morphology and exercise capacity. (ii) In Rock (Rho-associated coiled-coil kinase) classification, it is shows that Rock appears in differentially expressed proteins (DEP) and differentially expressed genes (DEG) dataset. (iii) In the extracellular matrix and cell junction related proteins classification, certain molecules appear multiple times in in NFPA groups, indicating that these molecules play a relatively important role in the process of In the development of NFPA, they are: Integrin (4 times); Collagens (3 times); Collagen type I (3 times); Laminin (3 times). Changes in the expression of these molecules can cause connection alteration between cell and matrix, thereby changing the migration ability of cells and mediating a series of biological effects. In addition, MMP is a matrix metalloproteinase which presents in Dataset 5 can degrade a variety of extracellular matrix components, weaken the adhesion between cell and matrix and contribute to the distal migration of tumor cells. (vi) In the GF, GFR and related proteins classification, there are several molecules appear several times: TGF-β 5 times, VEGF 4 times, PDGF BB 4 times. And EGFR is found in both the DEG and DEP groups in invasive NFPA, but it is not shown in the noninvasive group, suggesting that the expression of EGFR increase malignancy during the development of nonfunctioning pituitary adenomas. (v) In the Retinoic acid receptors classification, Rxr appears twice. And its abnormal regulation may be related to the occurrence of non-functional pituitary adenomas.

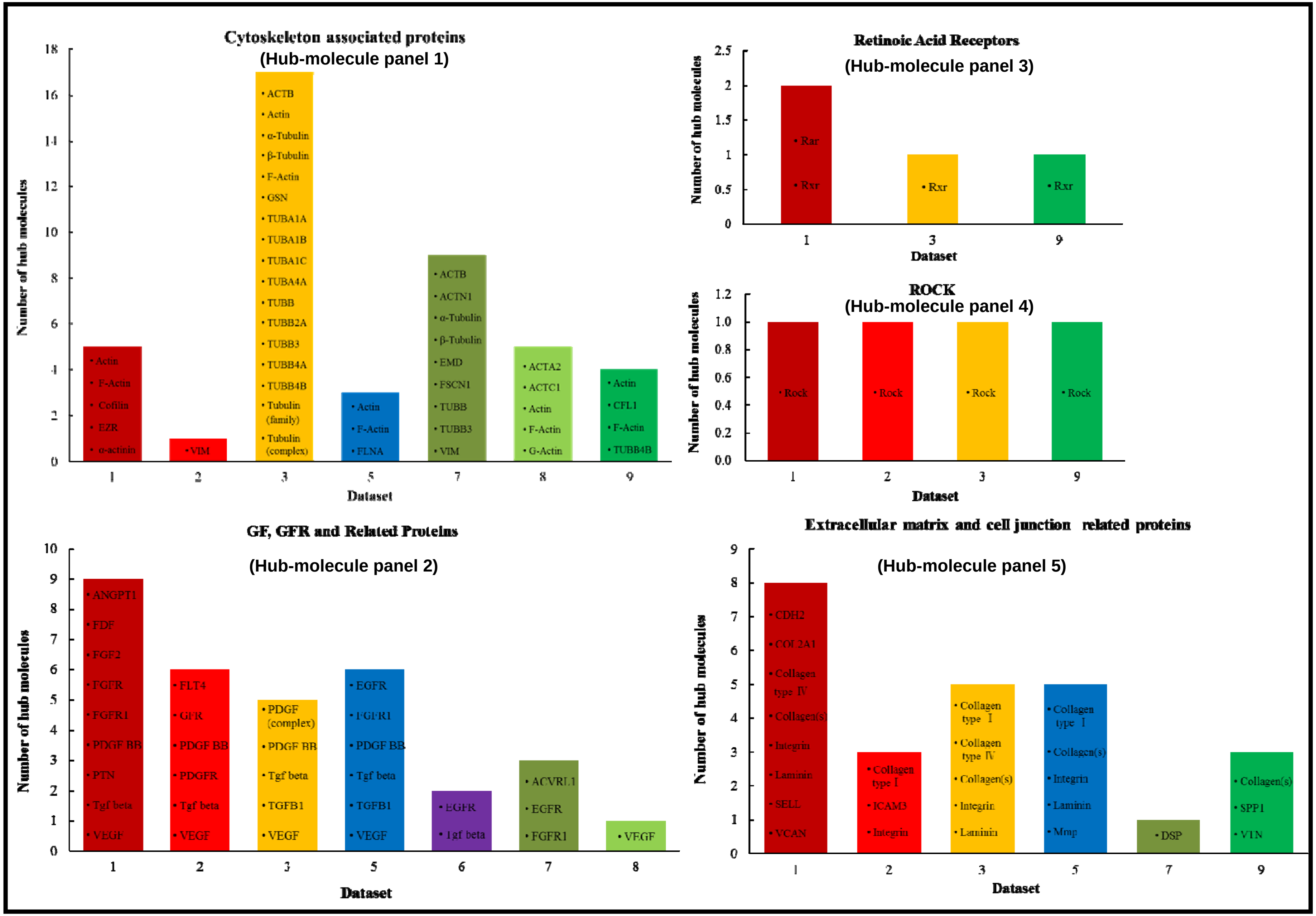

Supplemental figure 2A

Supplemental figure 2B

Category B includes 3 hub-molecule panels, and mainly functions in kinase signaling pathways related proteins.

The number of kinases in the invasive DEG group (Dataset 5) in three kinase-related classifications is all at the forefront, indicating that the dysfunction of the kinase and its associated proteins contributed to the malignancy progress of NFPA. (i) In the MAPK signaling pathway related proteins classification, various kinases shown several times in NFPA groups: ERK 5 times; ERK1/2 5 times; Jnk 5 times; MAPK 5 times; Ras 5 times; Mek 4 times; p38 MAPK 3 times. (ii) In the PI3K signaling pathway related proteins classification, many kinases appear several times: Akt 5 times; PI3K complex 5 times; p85 3 times in NFPA groups. (iii) In other protein kinases classification, the duplicated appeared kinases are: Pkc 6 times; Pka 4 times; FAK 4 times; p70S6K 3 times in the NFPA groups.

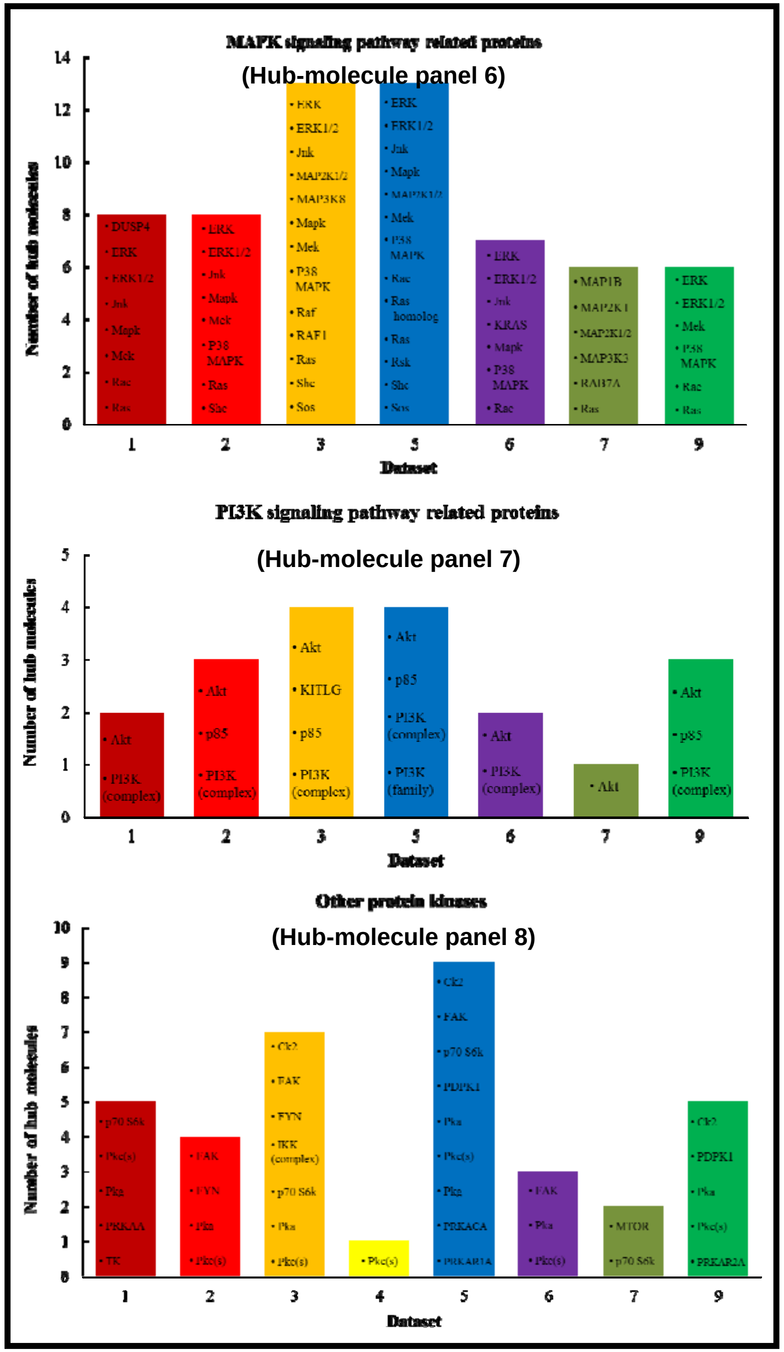

Supplemental figure 2B

Supplemental figure 2C
Category C includes 2 hub-molecule panels, and mainly functions in protein synthesis and degradation.

Normal pituitary groups have a high number of hub molecules, consistent with normal physiologic processes. (i) Protein synthesis related proteins are involved in protein synthesis and metabolism, is the key to maintain the basis activities of life in cells, therefore molecules involved in the regulation of these processes should also account for a large part of the cell proportion. (ii) In the Ubiquitin protein and protein degradation pathway related proteins classification, UBC occurred six times in the NFPA groups suggesting that the ubiquitination pathway has been changed in nonfunctioning pituitary adenomas and would affecting the occurrence and development of the tumor.

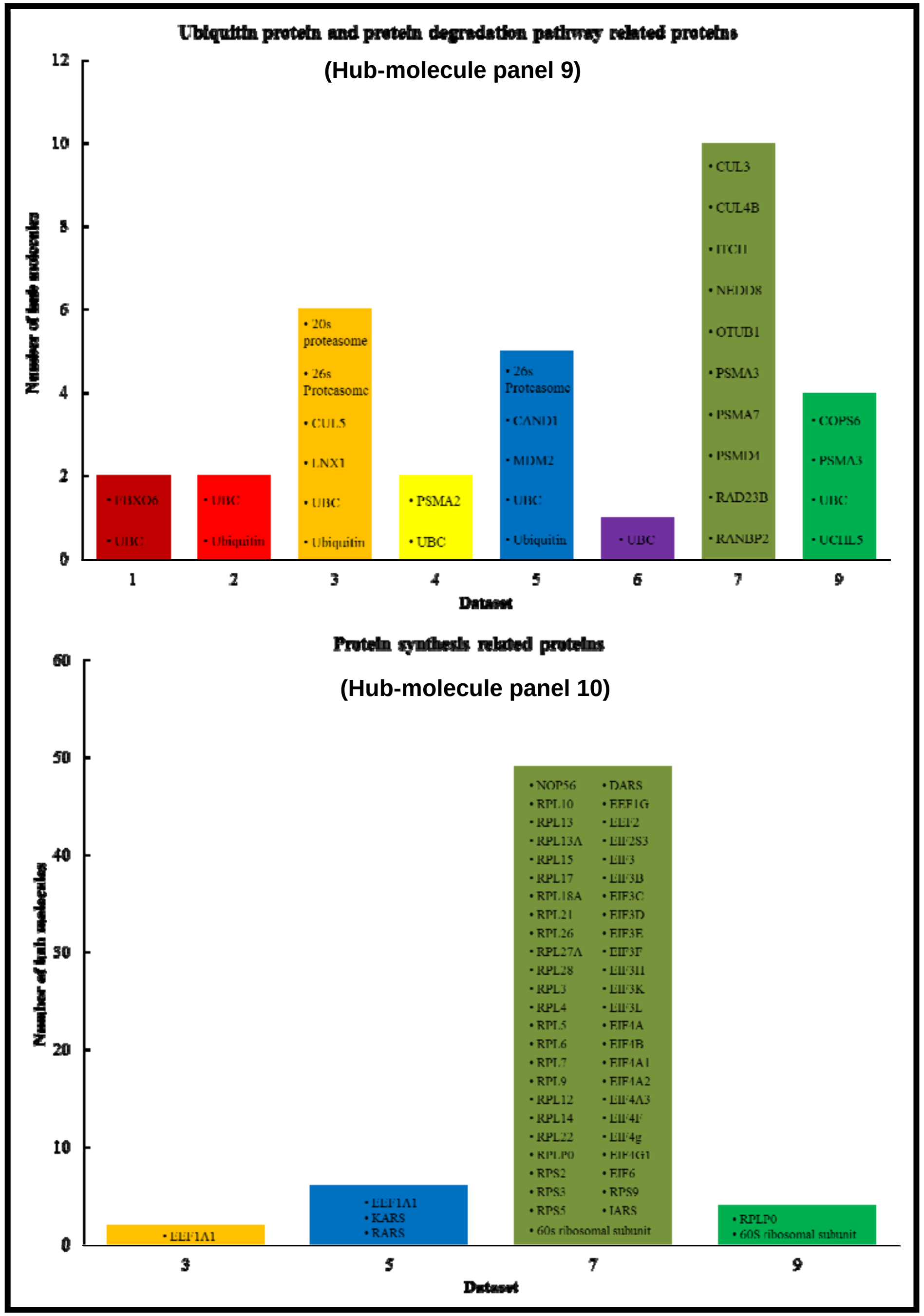

Supplemental figure 2C

Supplemental figure 2D
Category D includes 3 hub-molecule panels, and mainly functions in stress response.

(i) In the heat shock and molecular partner classification, the number of hub molecules is higher in the normal pituitary group. Thus this case is reasonable for heat shock protein plays an important role in protecting the normal physiological activities of cells when encounter stress such as endoplasmic reticulum stress and heat stress. In the NFPA groups HSP90 appears three times, suggesting that this molecule exert an essential role in NFPA. (ii) In the Proteins against oxidative stress classification, the number of hub molecules is still higher in the normal pituitary group, but in the NFPA groups, SOD appears three times, suggesting that in NFPA the expression of molecules that encountering oxidative stress have changed, leading to different biological results in response to oxidative stress compared to normal pituitary. (iii) In addition, in the NO, NOS classification, molecules only show in the NFPA groups, indicating that the way of application of NO for signal transduction or exerting other biological function in NFPA is quite different from that in the normal pituitary. Non-functional pituitary adenomas may superfluously produce NO to promote the hemangiectasis and facilitate the growth of tumor tissues.

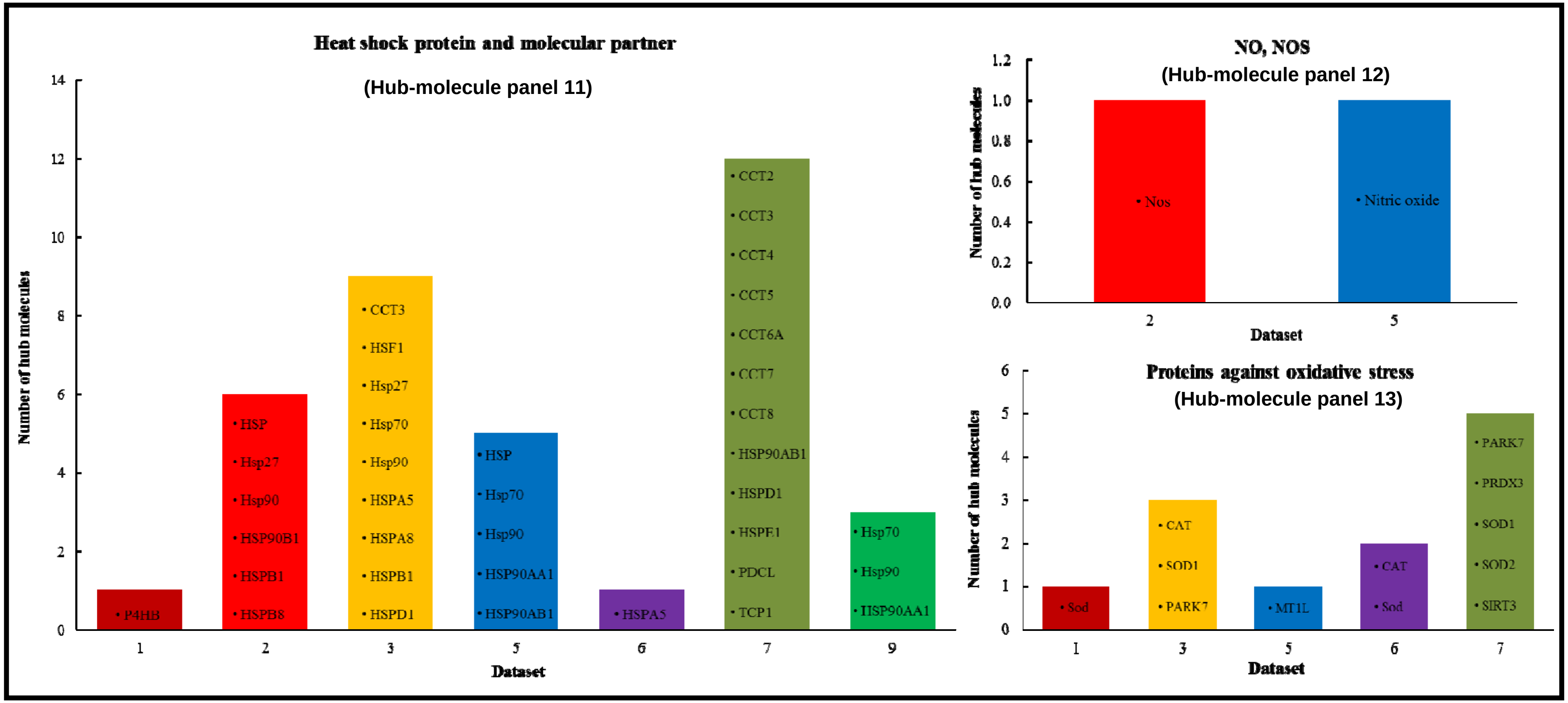

Supplemental figure 2D

Supplemental figure 2E
Category E includes 2 hub-molecule panels, and mainly functions in Notch-Wnt signaling pathway.

The hub molecules are both higher in the NFPA groups: (i) In the Wnt signaling pathway related proteins classification, hub molecules in invasive group account for the majority, indicating that Wnt pathway play an important role in the malignant transforms in non-functional pituitary adenoma. (ii) In the Notch signaling pathway related proteins classification, hub molecules both show in the non-invasive group and invasive group, and non-invasive group of has the majority of the hub molecules, indicating that the Notch pathway performs a pivotal role in the development and progression of NFPA.

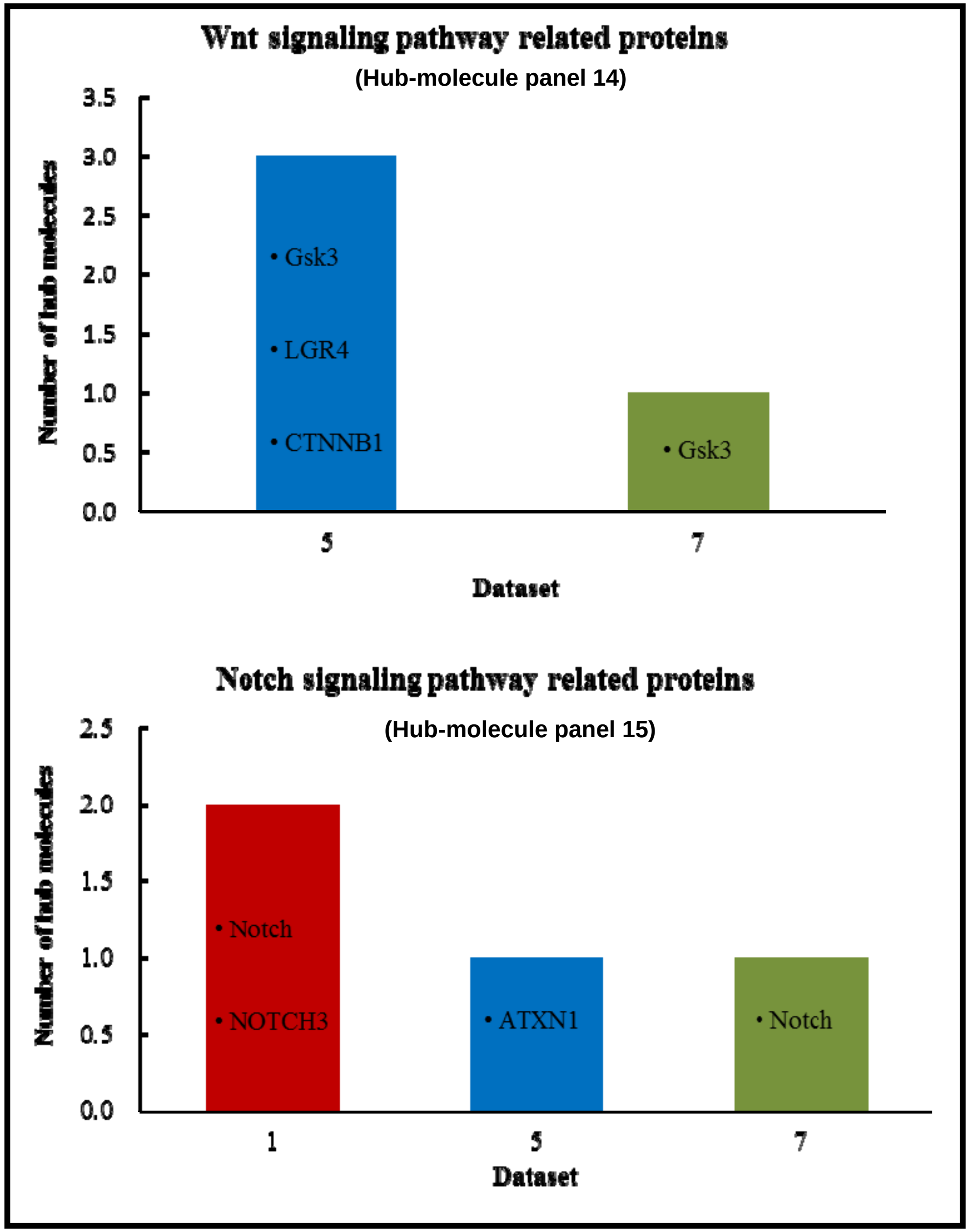

Supplemental figure 2E

Supplemental figure 2F

Category F includes 2 hub-molecule panels, and mainly functions in cell-cycle regulation.

(i) In 14-3-3 and related proteins classification, the number of hub molecules in the normal pituitary group is higher, but in the NFPA groups YWHAQ appears three times. (ii) In the cell cycle related proteins classification, Cyclin A appears 3 times in the NFPA groups, indicating that non-functional pituitary adenomas may exist cell cycle disorders.

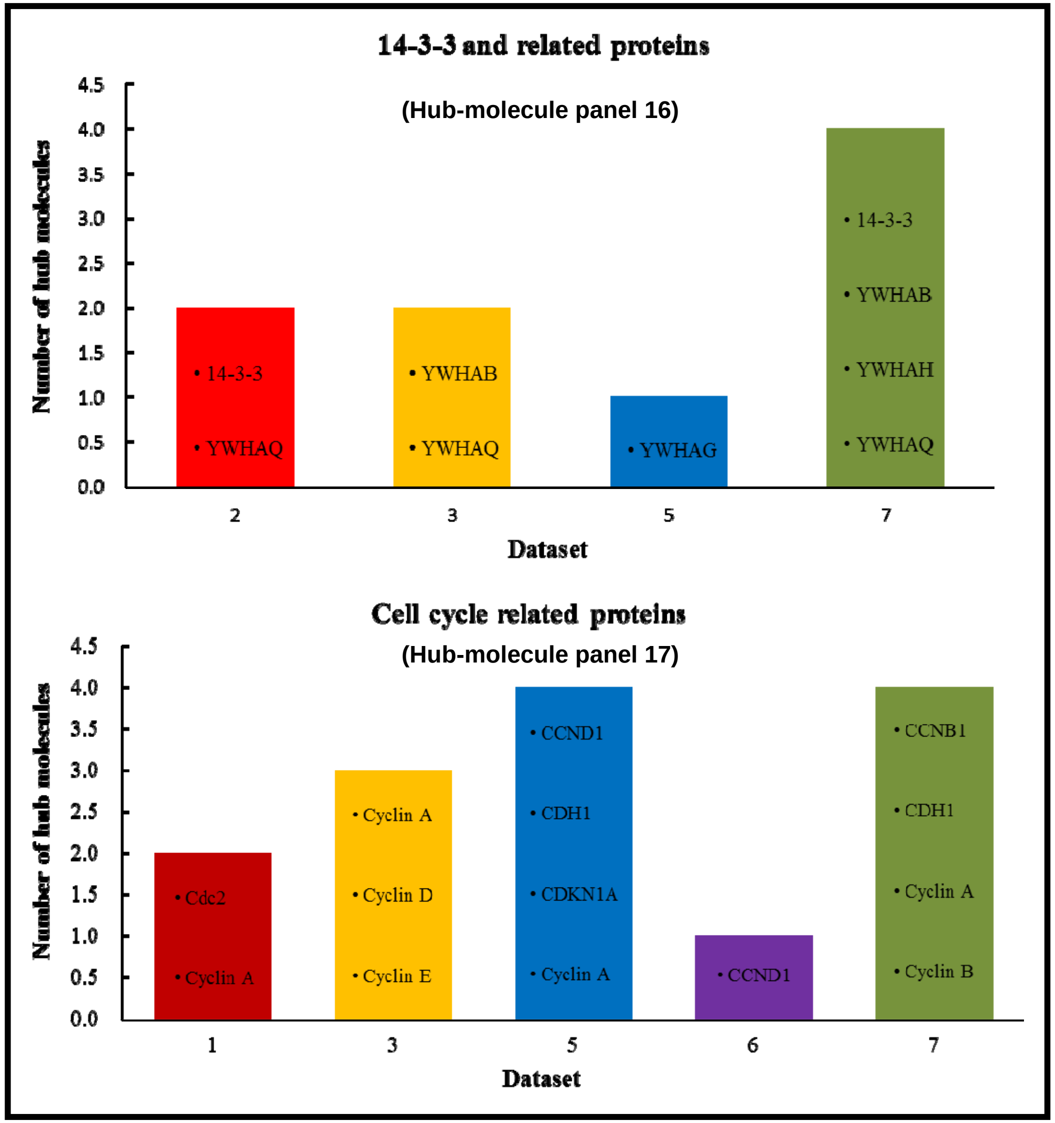

Supplemental figure 2F

Supplemental figure 2G
Category G includes 5 hub-molecule panels, and mainly functions in transcription and its regulation.

The number of hub molecules in NFPA groups is higher than that in the normal pituitary in all of the five classifications, indicating abnormalities exist in all aspects of transcriptional regulation in nonfunctioning pituitary adenomas, and these changes is conducive to tumor development. In the Transcription related proteins classification, NF κ B Complex appears five times and Creb appears four times. These two molecules occur repeatedly, indicating that the transcriptional regulation in non-functional pituitary adenomas is different from that in normal pituitary, which is favorable to the NFPA to transcript large amounts of cancer related mRNA which is conducive to survival and growth of tumor.

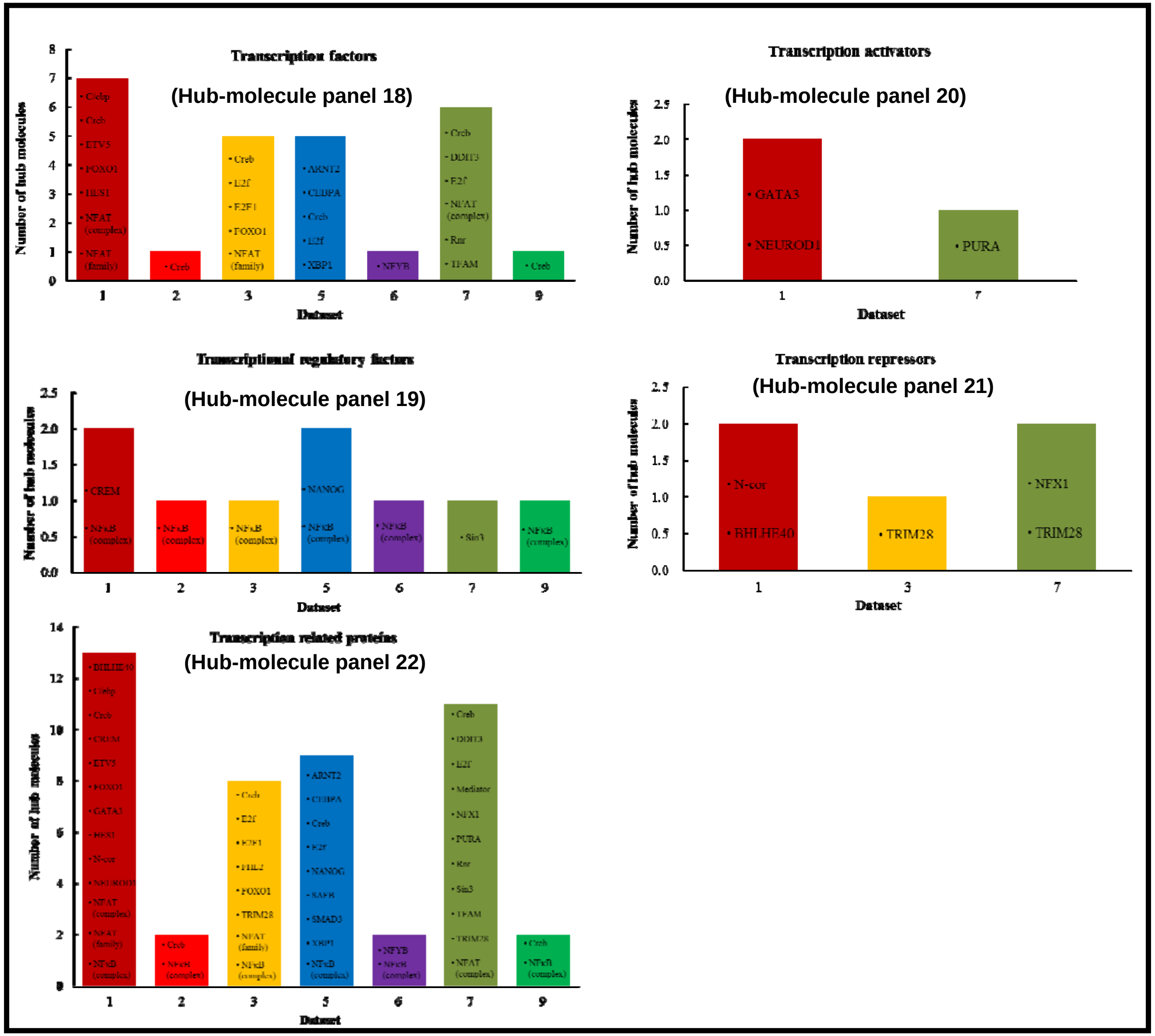

Note: The classification "Transcription related proteins" is a summary classification, which contains not only the other four related sub-categories (the four sub-categories are: "Transcription factors", "Transcriptional regulatory factors", "Transcription activators" and "Transcription repressors"), but also four additional hub molecules that associated with transcriptional regulation, they are FHL2 (from Dataset 3); SAFB, SMAD3 (from Dataset 5) and Mediator (from Dataset 7).

Supplemental figure 2G

Supplemental figure 2H
Category H includes 5 hub-molecule panels, and mainly functions in DNA/RNA regulation and metabolism.

The number of hub molecules in the normal pituitary group is higher than that in the NFPA groups in all of the five classifications, but in the histones and related proteins classification, Histone 3 appears 4 times in the NFPA groups, indicating that Histone 3 often involved in the process of this kind of cancer. Histone 3 dysfunction often leads to chromosomal structural instability, which is conducive to the initiation of target gene transcription and genome replication. What's more, the chromosome structure instability is likely to trigger mutations, so that accumulation of more types of mutations in tumor tissue and contribute to the survival and rapid growth.

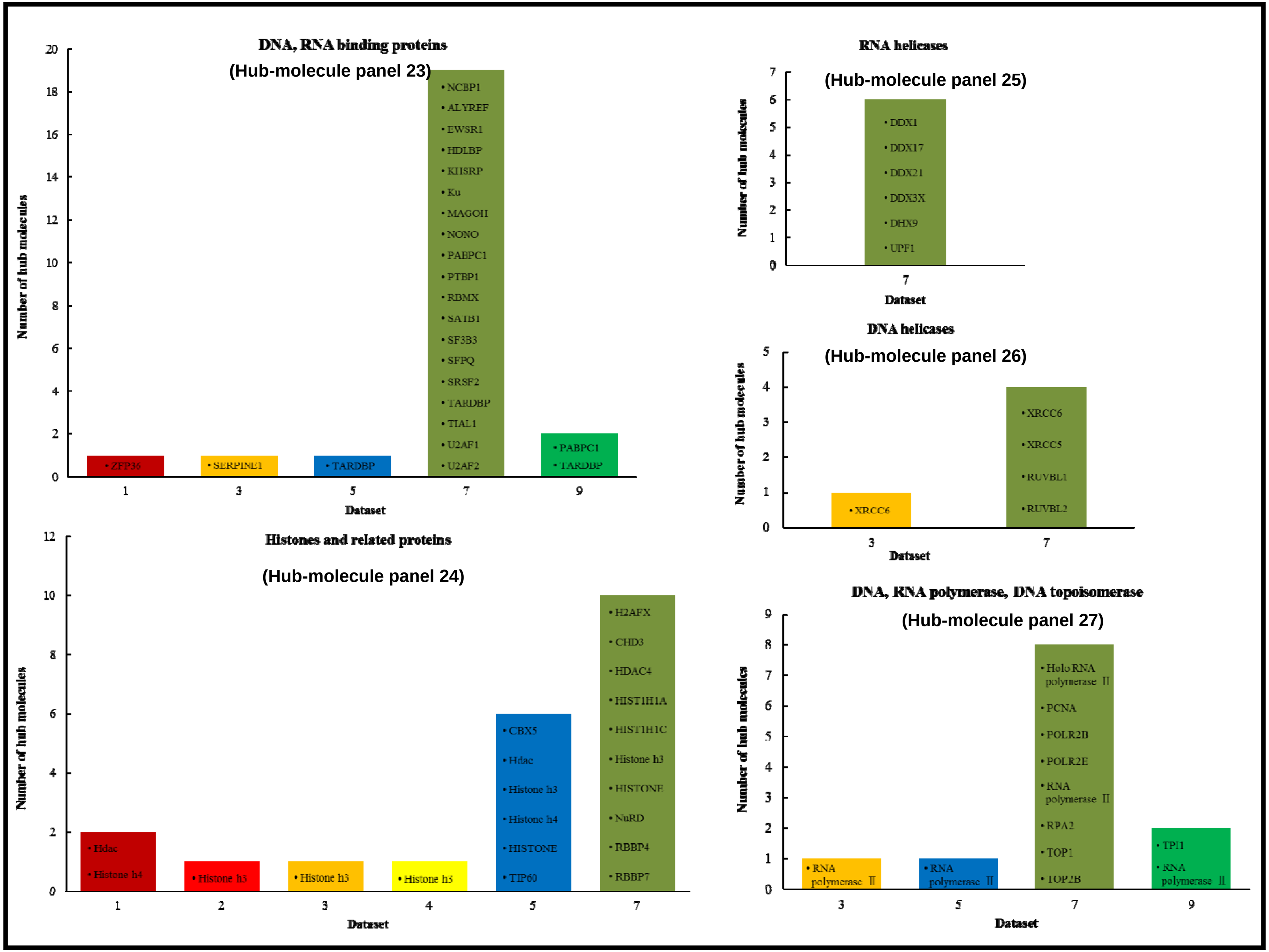

Supplemental figure 2H

Supplemental figure 2I
Category I includes 2 hub-molecule panels, and mainly functions in immune and inflammation related proteins and cytokines.

The number of hub molecules in the NFPA groups is higher in both classifications. (i) In immun related proteins classification, immunoglobulins appears five times; IgG appears three times; TCR appears three times; IgE appears three times in NFPA groups. (ii) IFNG appeared three times and IFN-α shows 3 times in the NFPA groups within the Inflammation related proteins classification, and the number of inflammatory-related molecules is higher in the invasive group than in the non-invasive group. These data indicates that the immunoreaction and inflammatory response are both exist in NFPA, and the inflammatory response is more severe in the invasive group than in the non-invasive group.

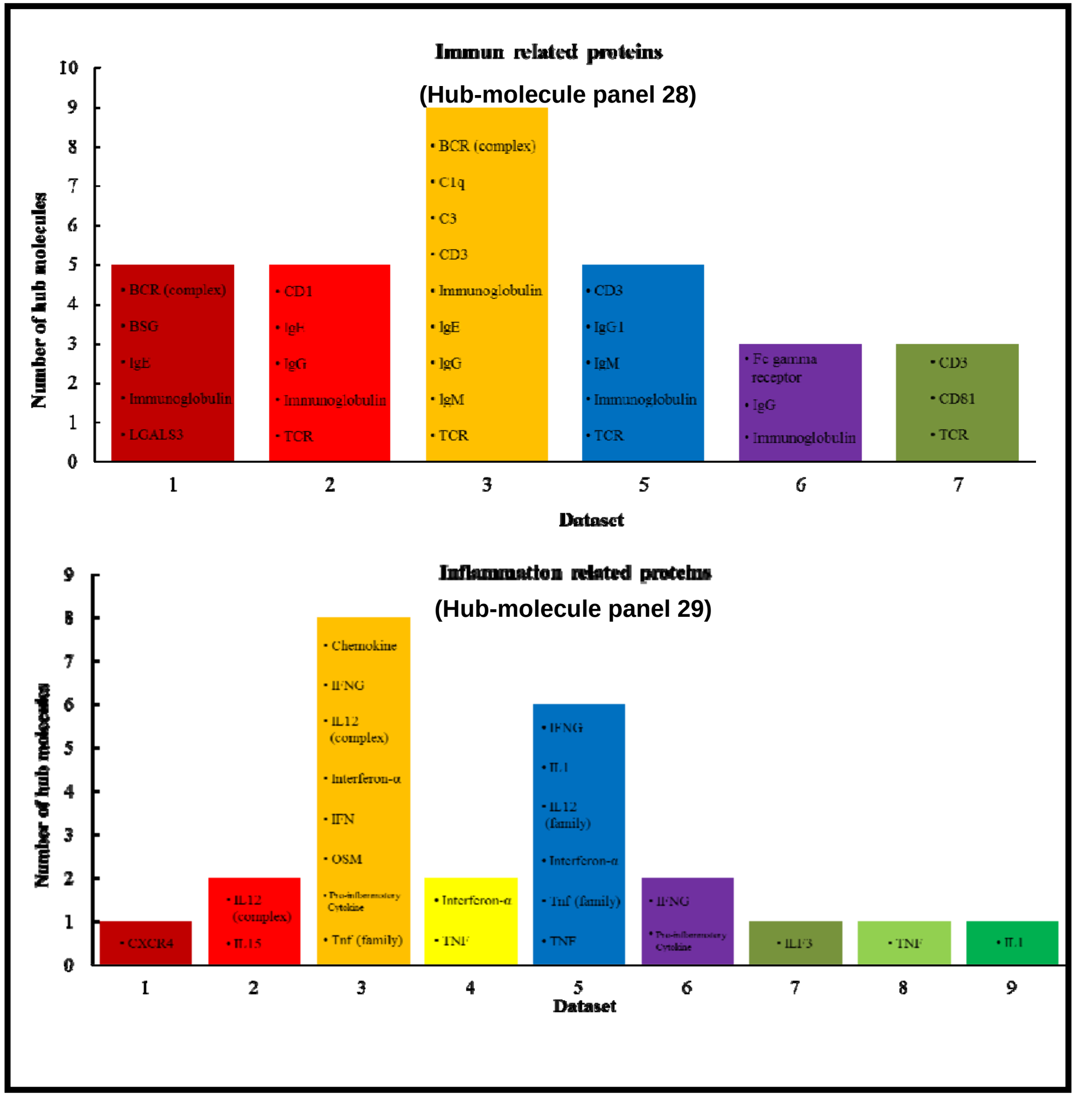

Supplemental figure 2I

Supplemental figure 2J
Category J includes 1 hub-molecule panel, and mainly functions in hormones.

The number of hub molecules in the NFPA groups is higher than in the normal pituitary group in Hormones and related proteins classification, in which the Lh appears 5 times; Insulin appears 4 times; GH appears 4; GH1 appears 4 times; Estrogen receptor appears 4 times; β -estradiol appears 3 times and FSH appears 3 times. There are differences about some hormones and related molecules between the non-invasive group and the invasive group: ESR1 only appears in the invasive group and occurs twice; while Proinsulin and PRL only appears in the non-invasive group, in which Proinsulin appears 3 times and PRL appears 2 times. This phenomenon shows that in the non-functional pituitary adenoma invasion and non-invasive groups there are expression disorders about hormones and their associated protein, which is benefit to the regulation of many physiological functions such as tumor growth and metabolism. In addition, there are also differences in the expression of some hormones and their associated proteins between the two groups, suggesting that different types of hormones are required in different stages of NFPA development, and this phenomenon could contribute to the establishment of clinical classification for nonfunctional pituitary adenomas.

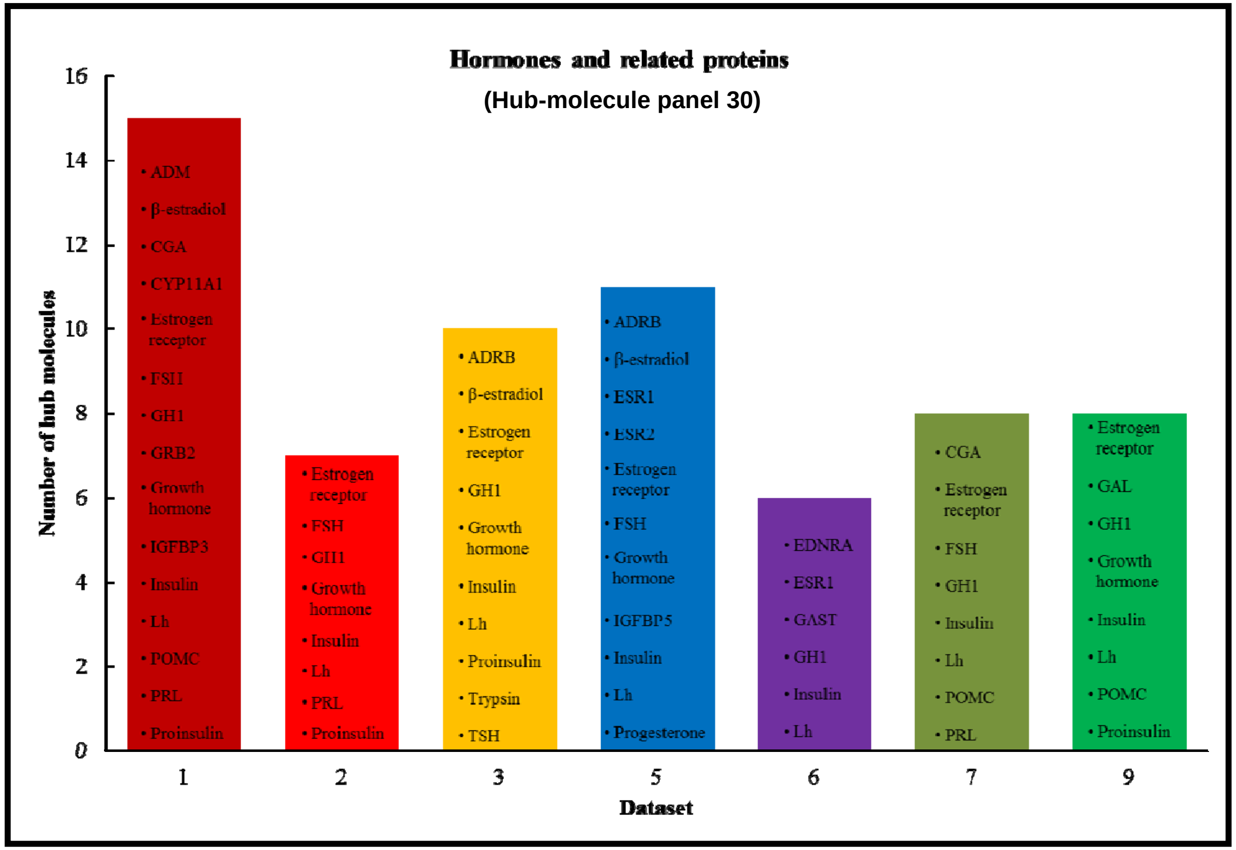

Supplemental figure 2J

Supplemental figure 2K
Category K includes 3 hub-molecule panels, and mainly functions in energy metabolism.

(i) The number of hub molecules in the Mitochondrial electron transport related proteins classification in normal pituitary group is much higher than in the NFPA groups, probably due to the mitochondrial dysfunction in non-functional pituitary adenomas, leading to the loss of mitochondrial-associated proteins and especially the deficiency of electron transport chain related proteins. In this context, cytochrome c appears 4 times in NFPA groups, indicating that the expression disorders of this hub molecule play a vital role in the non-functional pituitary adenoma carcinogenesis. (ii) In addition, in the ATP synthesis related proteins classification, hub molecules only appear in the NFPA groups, indicating that although nonfunctional pituitary adenomas exist mitochondrial dysfunction, it does not injury the ATP synthesis, but by abnormal regulation of the proteins related to ATP synthesis to increase ATP production and meet the rapid growth needs of NFPA.

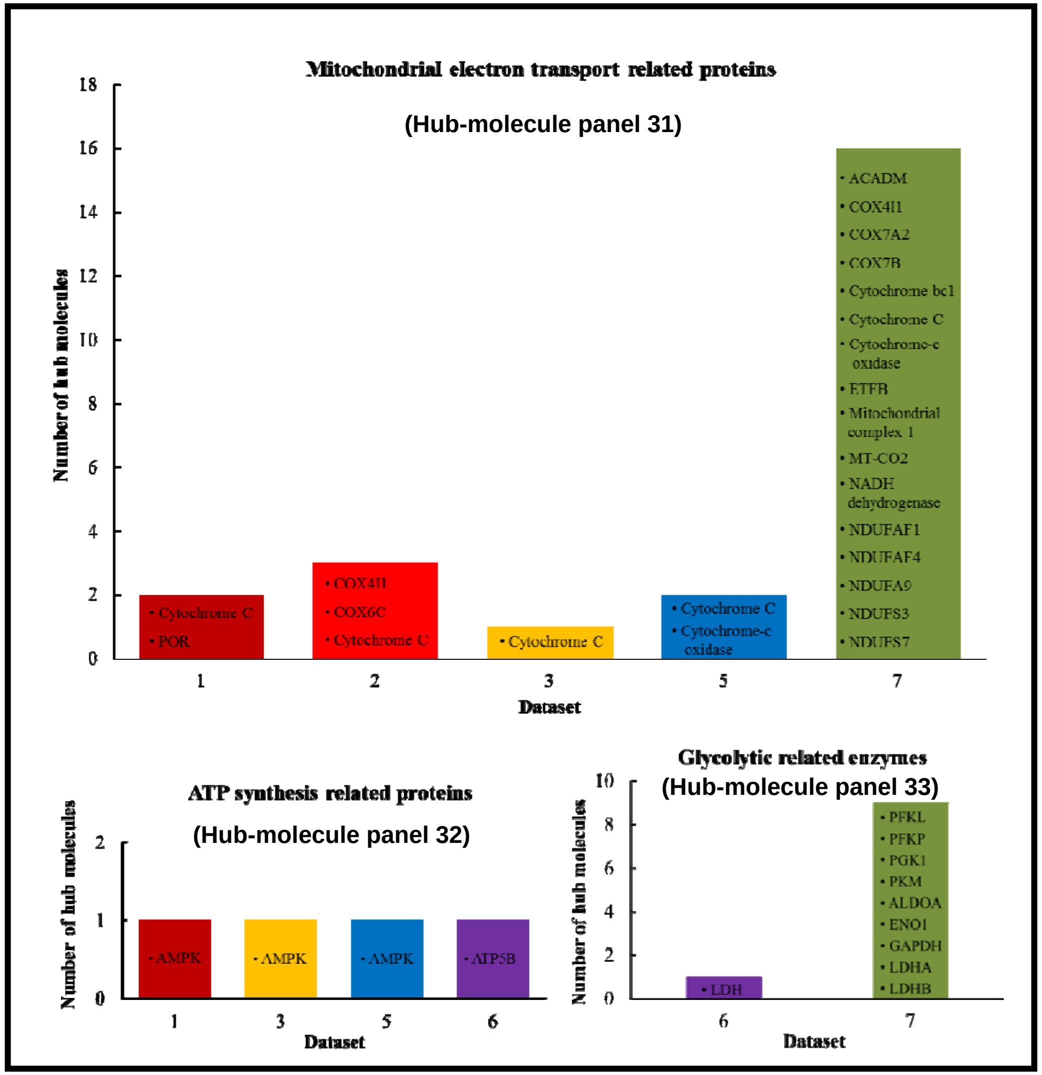

Supplemental figure 2K

Supplemental figure 2L
Category L includes 2 hub-molecule panels, and mainly functions in proteins involved in tumorigenesis.

(i) The number of hub molecules in the NFPA groups is significantly higher than that in the normal pituitary group in the Oncogene proteins and proteins involved in tumorigenesis classification. In the NFPA groups AP1 occurs 4 times and STAT5a/b appears 3 times, indicating that these two molecules play an important role in the development of non-functional pituitary adenomas. (ii) In the Tumor Suppressor classification, both non-invasive and invasive DEG group have one hub molecule with tumor suppressor function, suggesting that there is abnormal regulation of tumor suppressor in non-functional pituitary adenomas benefit its tumorigenicity.

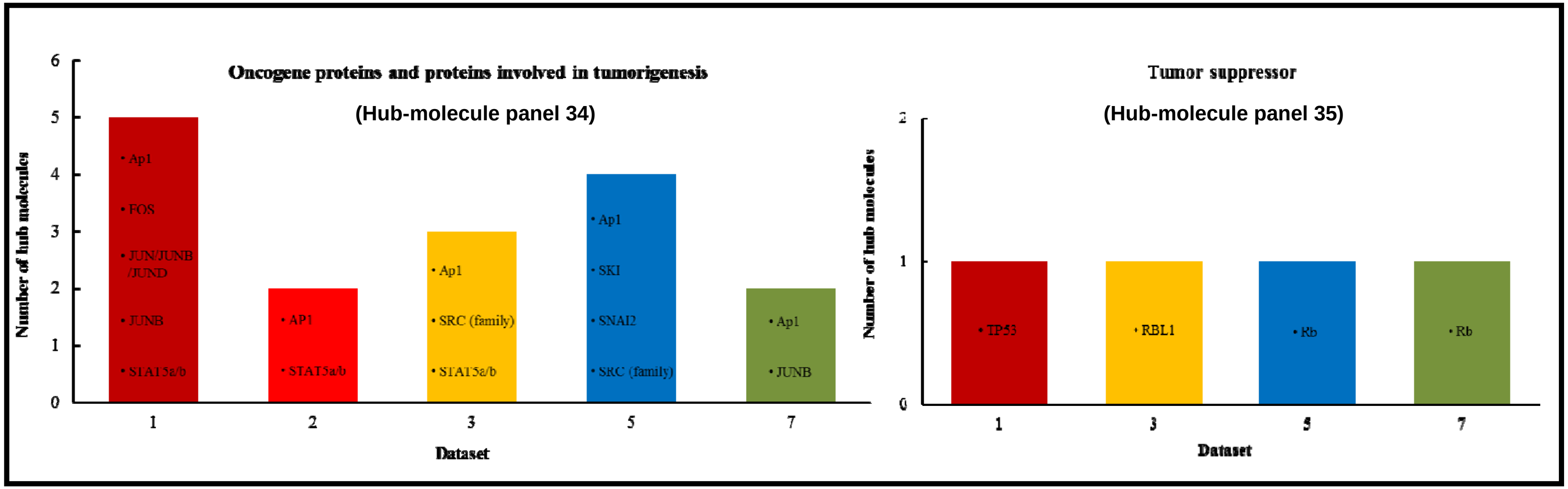

Supplemental figure 2L

Supplemental figure 2M
Category M includes 1 hub-molecule panel, and mainly functions in Apoptosis related proteins.

Caspase appears 3 times in the NFPA groups within the Apoptosis related proteins classification. This suggests that the programmed death pathway in nonfunctioning pituitary adenomas may be altered, which lead to the cancer cells become not subject to normal death procedures, and resistant to a range of stimuli and chemotherapeutic agents, resulting in the unlimited proliferation and immortalization of tumor cells.

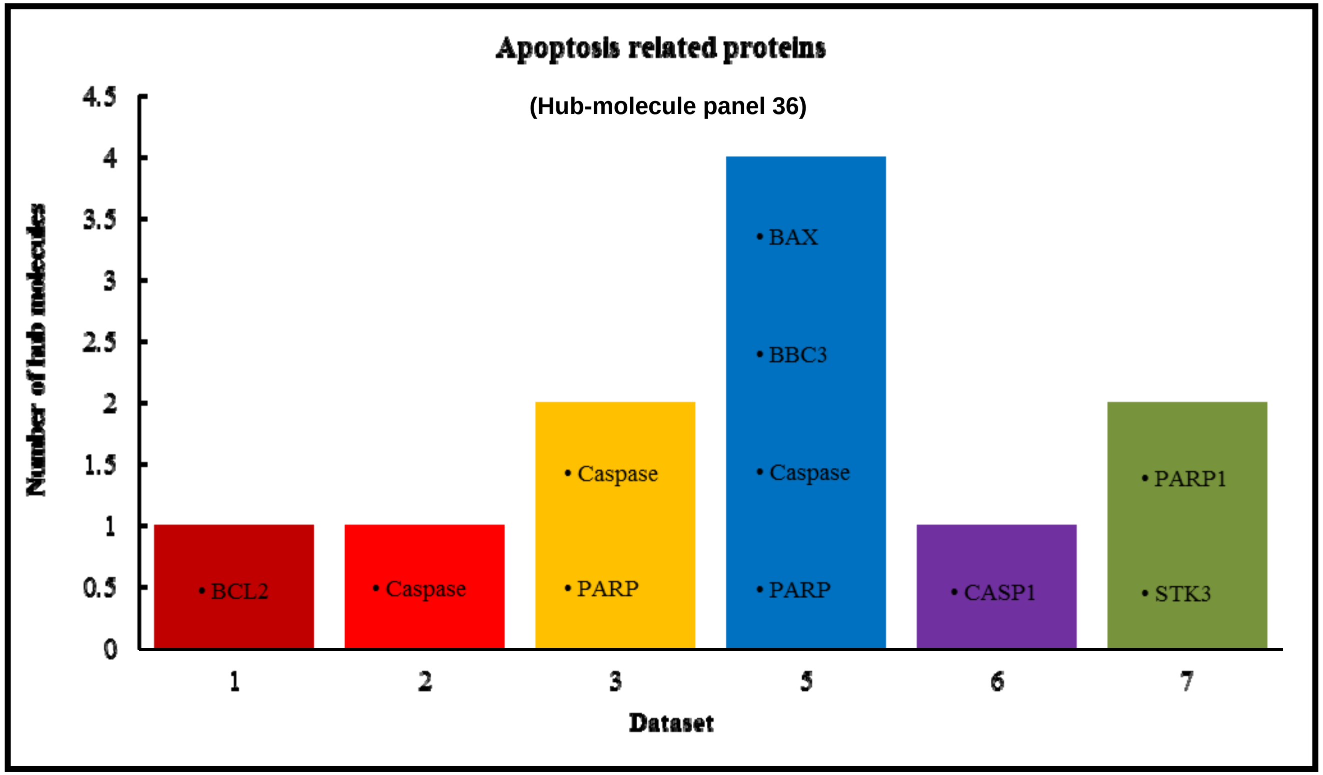

Supplemental figure 2M

Supplemental figure 2N
Category N includes 1 hub-molecule panels, and mainly functions in Ca2+ related proteins.

Both Calmodulin and Calpain appear 3 times in the NFPA groups within the Ca2+ related proteins classification. The number of Ca2+ -related proteins in the invasive groups is higher than that in the noninvasive groups, suggesting that the regulation of Ca2+ in non-functional pituitary adenomas is abnormal, and the abnormality is getting more serious with the malignant development of the tumor. Dysregulation of Ca2+ results in electrolyte imbalance, disorders in related signal and abnormal cell connections, these changes are conducive to tumor cell growth and movement, facilitate invasiveness and metastasis.

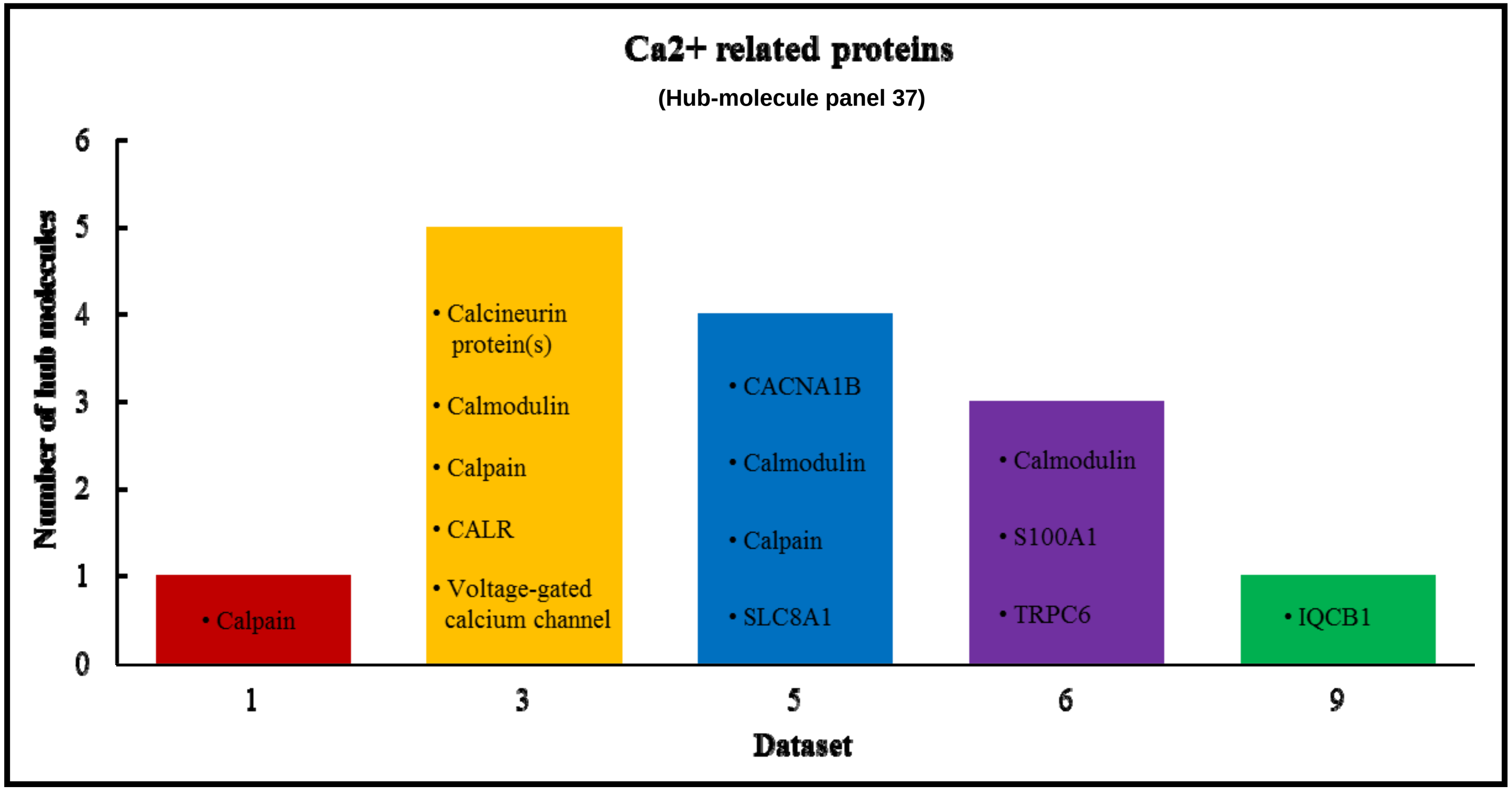

Supplemental figure 2N

Supplemental figure 2O
Category O includes 1 hub-molecule panel, and mainly functions in G protein-related signaling pathway.

ADCY appears 4 times and PLC appears 3 times in the NFPA groups within the Proteins associated with G protein and its signal transduction pathway classification. The results suggesting that there are disorders in the G protein signal transduction pathway in NFPA. Because the G protein signal transduction pathway participates in the control of various important intracellular physiological processes, disorders in this signaling would induce a series of dysfunction in cells, and that is conducive to survival for cancer cells.

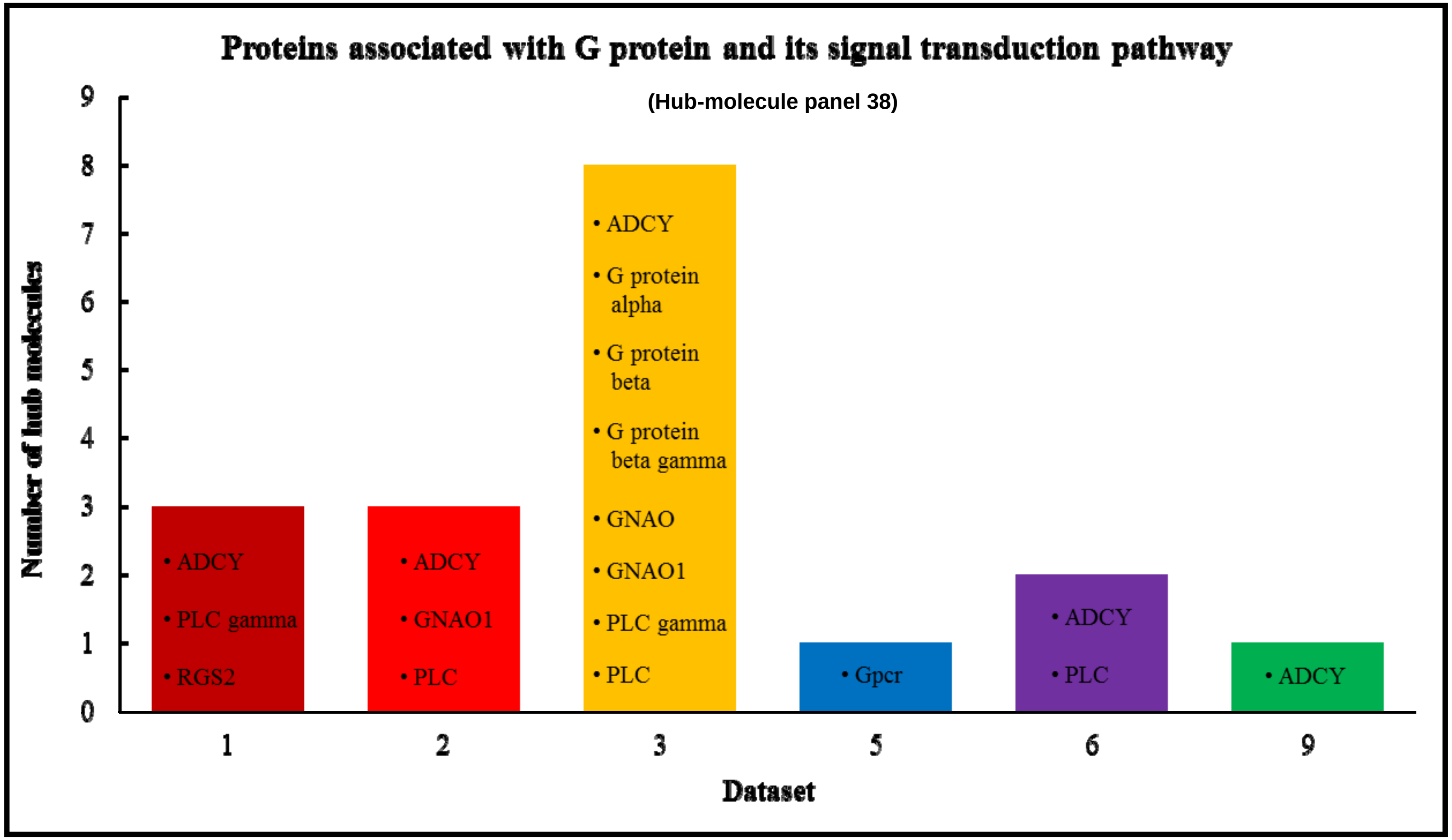

Supplemental figure 2O

Supplemental figure 2P
Category P includes 4 hub-molecule panels that were grouped as others.

(i) Both PP2A and PP1 protein complex group appear twice in the NFPA groups within the Phosphatase and phosphodiesterases classification. These two enzymes can regulate the activity of phosphorylated proteins, thereby controlling the related kinase signal transduction pathways, the expression disorders contribute to the excessive activation of certain signal pathways or signal blockage, and regulate cancer cell growth. (ii) LDL appears 5 times in the NFPA groups within the LDL, HDL and its related proteins classification, suggesting the existence of abnormal lipid metabolism in nonfunctioning pituitary adenomas.

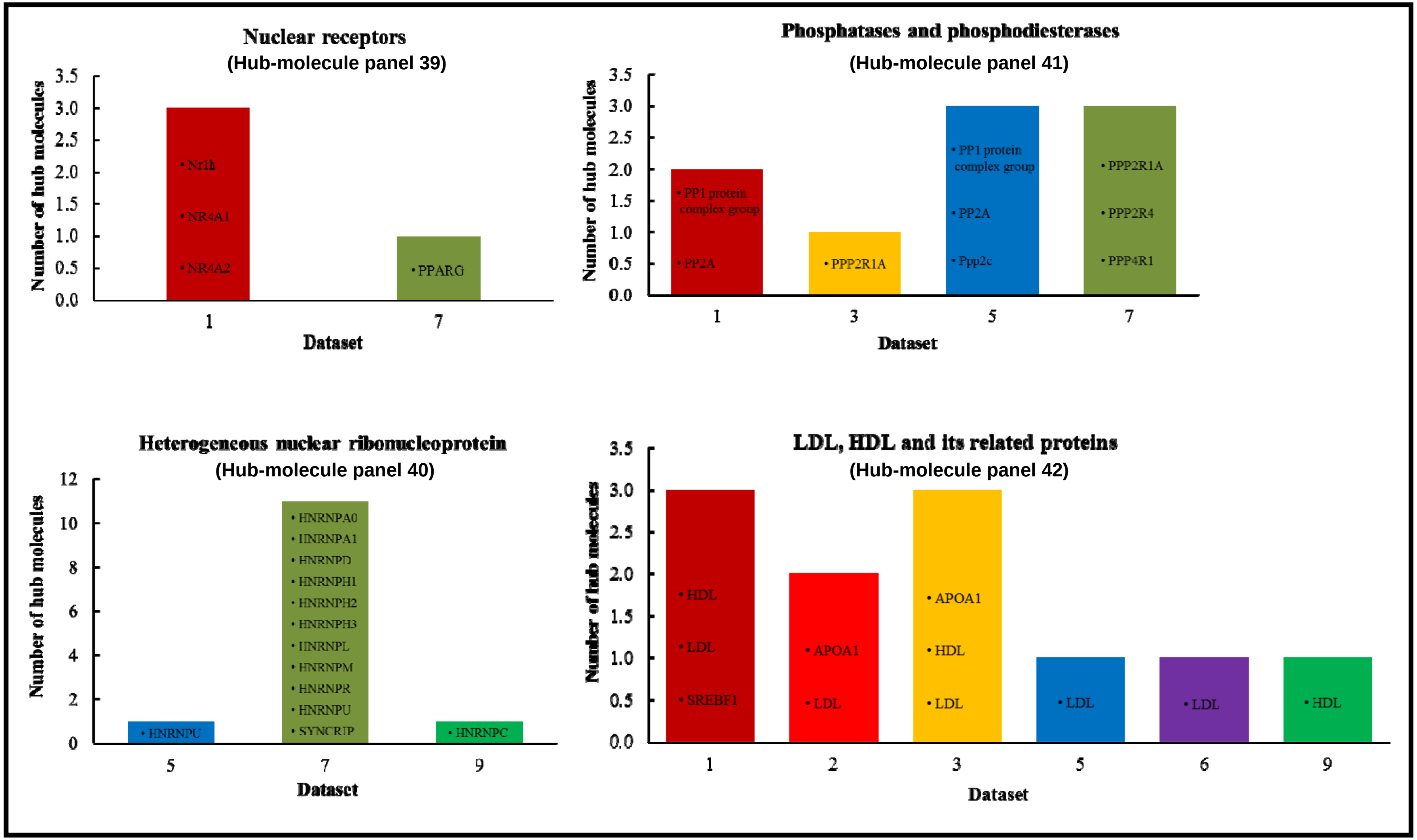

Supplemental figure 2P
