# Supplementary material for: Multiomics-Based Signaling Pathway Network Alterations in Human Non-functional Pituitary Adenomas
Source: Front Endocrinol (Lausanne). 2019 Dec 17;10:835. doi: 10.3389/fendo.2019.00835 (PMC6928143; doi:10.3389/fendo.2019.00835)

Supplemental Figure 3. Hub-molecule panel regarding GF, GFR and related proteins that were derived from different datasets.

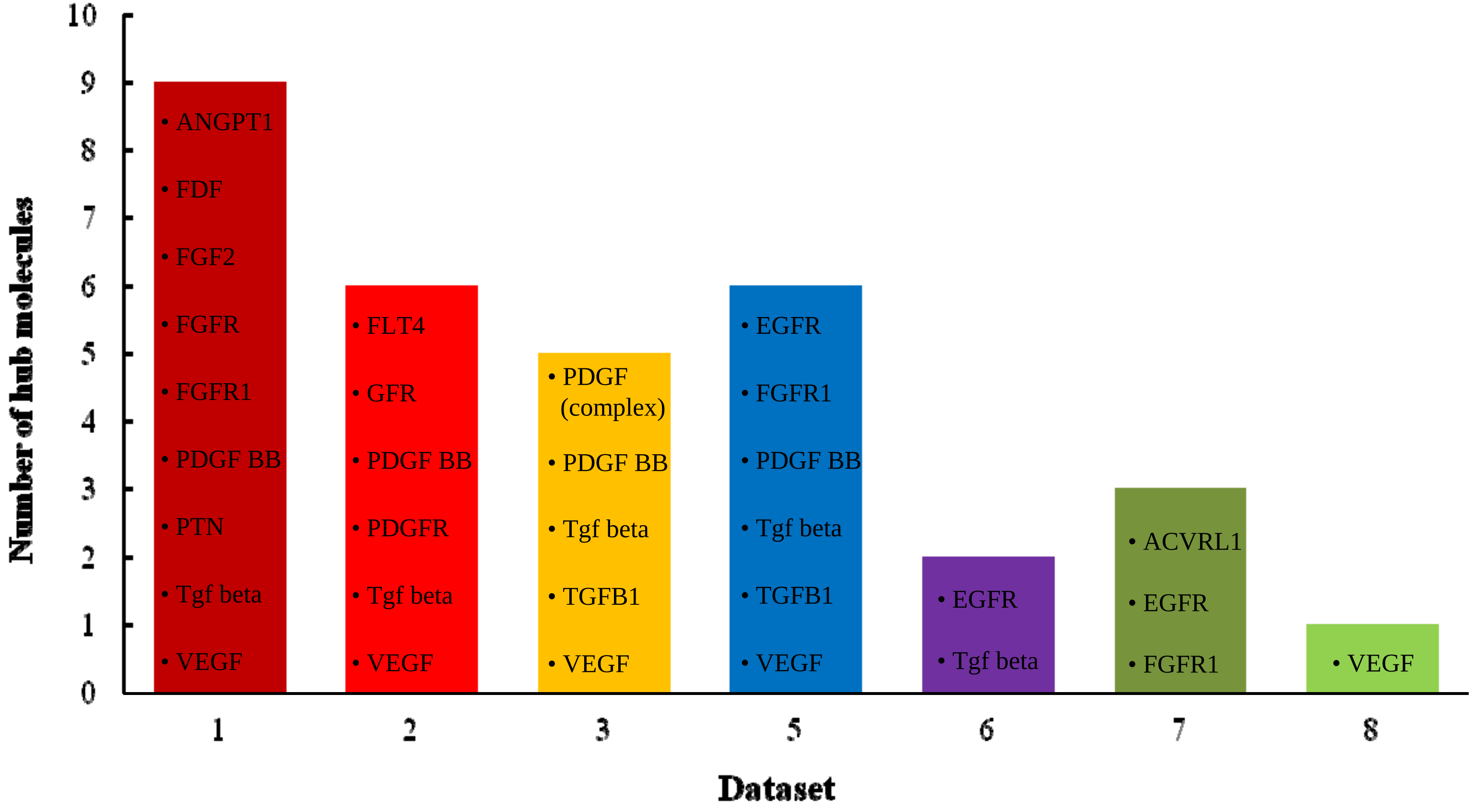

Supplement: Supplementary file 1 [file Presentation_1.zip › Supplemental Figure 3_v1.pdf]
