# Supplementary material for: Multiomics-Based Signaling Pathway Network Alterations in Human Non-functional Pituitary Adenomas
Source: Front Endocrinol (Lausanne). 2019 Dec 17;10:835. doi: 10.3389/fendo.2019.00835 (PMC6928143; doi:10.3389/fendo.2019.00835)
Supplement: Supplementary file 1 [file Presentation_1.zip › Supplemental Figure 4_v1.pdf]

Supplemental Figure 4

Nine canonical-pathway panels that were significantly associated with NFPA pathophysiological processes

Supplemental Figure 4A
Panel A: cytoskeleton, cell adhesion and movement pathways.

This panel includes 7 canonical pathways: (i). Actin Cytoskeleton Signaling existed in the normal pituitary (Dataset 7 and Dataset 8), however, this pathway is significantly changed in the NFPA with evidence in the upregulation of the key molecules (PI3K, Talin, and Myosin), and downregulation of the key molecules (TIAM, PIR121, TMSB4 and ERM) with the analysis of NFPA DEG data (Dataset 1). (ii). CDK5 Signaling appeared in the normal pituitary (Dataset 7), however, this pathway is also significantly altered in the NFPA with evidence in the overexpression of the key molecules (Gao and CDK5) in invasive NFPA DEP data (Dataset 6). (iii). ILK Signaling emerged in the normal pituitary (Dataset 7 and Dataset 8), yet this pathway is significantly changed in the NFPA with evidence in the upregulation of the key molecules (FILAMIN (FLNA) and SLUG), and downregulation of the key molecules (PI3K, PDK1 and MSK1/2 (RPS6KA5)) with the analysis of invasive NFPA DEG data (Dataset 5). (iv). Inhibition of Matrix Metalloproteases pathway showed in the normal pituitary (Dataset 7), while this pathway is significantly changed in the NFPA with evidence in the upregulation of the key molecule (ADAM) and downregulation of the key molecule (MMP19) with the analysis of invasive NFPA DEP data (Dataset 6). (In dataset 2 MMP19 is increased, but not have this pathway in the dataset). (v). RhoA Signaling existed in the normal pituitary (Dataset 7 and Dataset 8), however, this pathway is significantly changed in the NFPA with evidence in the nitration of the key molecules (RHOGAP (ARHGAP5) and Rhophilin (Rhophilin2)) with the analysis of NFPA nitroproteins and nitroprotein related proteins data (Dataset 4). (vi). Tight Junction Signaling existed in the normal pituitary (Dataset 7, Dataset 8 and Dataset 9) and the pituitary mapping proteins dataset (Dataset 3), as well. However, this pathway is significantly changed in the NFPA with evidence in the upregulation of the key molecules (NECTIN and MYOSIN), and downregulation of the key molecules (TIAM1, CLDN and AP-1) with the analysis of NFPA DEG data (Dataset 1). (vii). Epithelial Adherens Junction Signaling existed in the normal pituitary (Dataset 7and Dataset 8) and the pituitary mapping proteins dataset (Dataset 3), as well. However, this pathway is significantly changed in the NFPA with evidence in the upregulation of the key molecules (Nectin, NOTCH, N-cadherin, FGFR1 and Myosin) with the analysis of NFPA DEG data (Dataset 1).

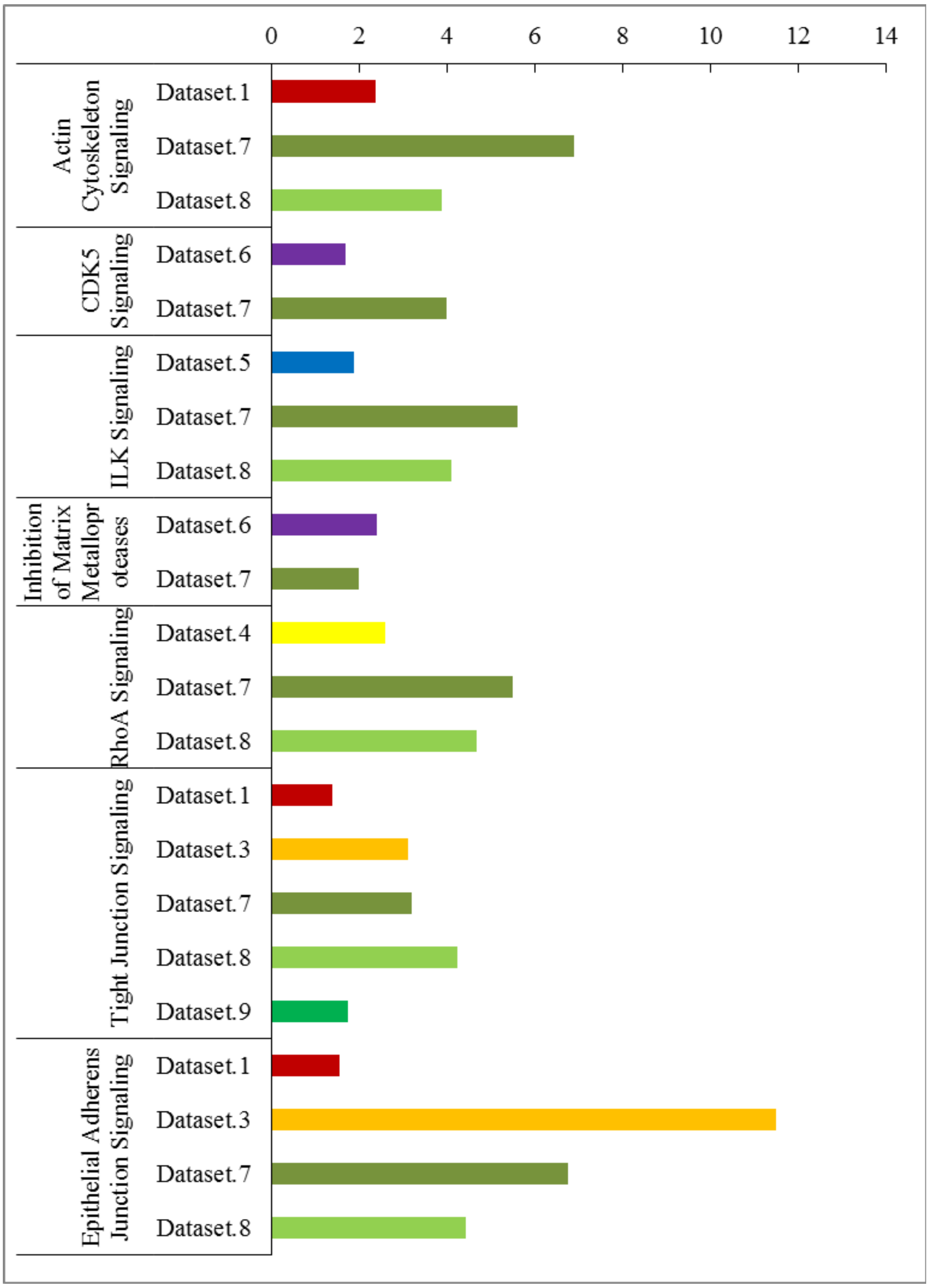

Supplemental Figure 4A

Supplemental Figure 4B
Panel B: mitochondrial dysfunction and energy metabolism related pathways.

This panel includes 3 canonical pathways: (i). Mitochondrial Dysfunction pathway existed in the normal pituitary (Dataset 7) and the pituitary mapping proteins dataset (Dataset 3), as well. However, this pathway issignificantly changed in the NFPA with evidence in the upregulation of the key molecules (NDUFS8, COX6B and ATP5B in Dataset 2; CAT, β-secret2 and ATP5B in Dataset 6), and downregulation of the key molecules (GPX4 in Dataset 2; ATP5A1 in Dataset 6) with the analysis of NFPA DEP data (Dataset 2) and invasive NFPA DEP data (Dataset 6). (ii). Oxidative Phosphorylation pathway existed in the normal pituitary (Dataset 7) andthe pituitary mapping proteins dataset (Dataset 3), as well. However, this pathway is significantly changed in the NFPA with evidence in the upregulation of the key molecules (NDUFS8, COX6B, ATP5B) with the analysis of NFPA DEP data (Dataset 2). (iii). AMPK Signaling existed in the normal pituitary (Dataset 7 and Dataset 9), however, this pathway is significantly changed in the NFPA with evidence in the upregulation of the key molecules (PP2C and PFK), and downregulation of the key molecules (PI3K, PKA and PDK1) with the analysis of invasive NFPA DEG data (Dataset 5).

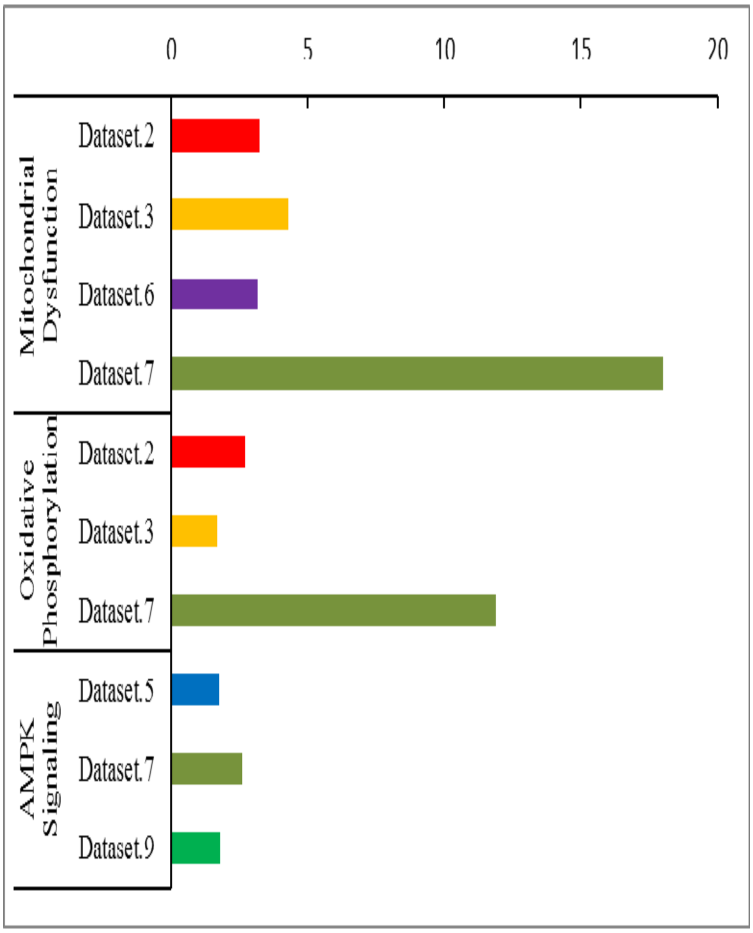

Supplemental Figure 4B

Supplemental Figure 4C
Panel C: angiogenesis, invasion, and metastasis related pathways

This panel includes 7 canonical pathways: (i). CXCR4 Signaling existed in the normal pituitary (Dataset 7) and the pituitary mapping proteins dataset (Dataset 3), as well. However, this pathway is significantly changed in the NFPA with evidence in the upregulation of the key molecules (G β , PI3K, IP3R), and downregulation of the key molecules (CXCR4 and c-FOS) with the analysis of NFPA DEG data (Dataset 1). (ii). eNOS Signaling existed in the normal pituitary (Dataset 7 and Dataset 9) and the pituitary mapping proteins dataset (Dataset 3), as well. However, this pathway is significantly changed in the NFPA with evidence in the upregulation of the key molecules (ESR1, HSPCA and HSPCB in Dataset 5), and downregulation of the key molecules (PI3K, PDK1, PKA and ESR2 in Dataset 5; HSP70 in Dataset 6) with the analysis of invasive NFPA DEG and DEP data (Dataset 5 and Dataset 6). (iii). Nitric Oxide Signaling in the Cardiovascular System existed in the normal pituitary (Dataset 7, Dataset 8 and Dataset 9), however, this pathway is significantly changed in the NFPA with evidence in the upregulation of the key molecules (PI3K, CaM, IP3R and SERCA in Dataset 1, and HSPCA, HSPCB, CACNA1I (T-Type calcium channel) and CACNA1E (R-Type calcium channel) in Dataset 5), and downregulation of the key molecules (PI3K and PKA in Dataset 5) with the analysis of NFPA DEG data and invasive NFPA DEG data (Dataset 1 and Dataset 5). (iv). Ephrin B Signaling existed in the normal pituitary (Dataset 7) and the pituitary mapping proteins dataset (Dataset 3), as well. However, this pathway is significantly changed in the NFPA with evidence in the upregulation of the key molecules (EPHB, EFNE and G β), and downregulation of the key molecule (CXCR4) with the analysis of NFPA DEG data (Dataset 1). (v). Ephrin Receptor Signaling existed in the normal pituitary (Dataset 7) and the pituitary mapping proteins dataset (Dataset 3), as well. However, this pathway is significantly changed in the NFPA with evidence in the upregulation of the key molecules (EPHB, EFNE and G β), and downregulation of the key molecules (CXCR4 and ANGPT1) with the analysis of NFPA DEG data (Dataset 1). (vi). Hypoxia Signaling in the Cardiovascular System existed in the normal pituitary (Dataset 7), however, this pathway is significantly changed in the NFPA with evidence in the upregulation of the key molecule (HSP90), and downregulation of the key molecule (UBE2) with the analysis of invasive NFPA DEG data (Dataset 5). (vii). Role of Tissue Factor in Cancer pathway existed in the normal pituitary (Dataset 7) and the pituitary mapping proteins dataset (Dataset 3), as well. However, this pathway is significantly changed in the NFPA with evidence in the upregulation of the key molecule (Src), and downregulation of the key molecule (FX (FX α)) with the analysis of NFPA DEP data (Dataset 2).

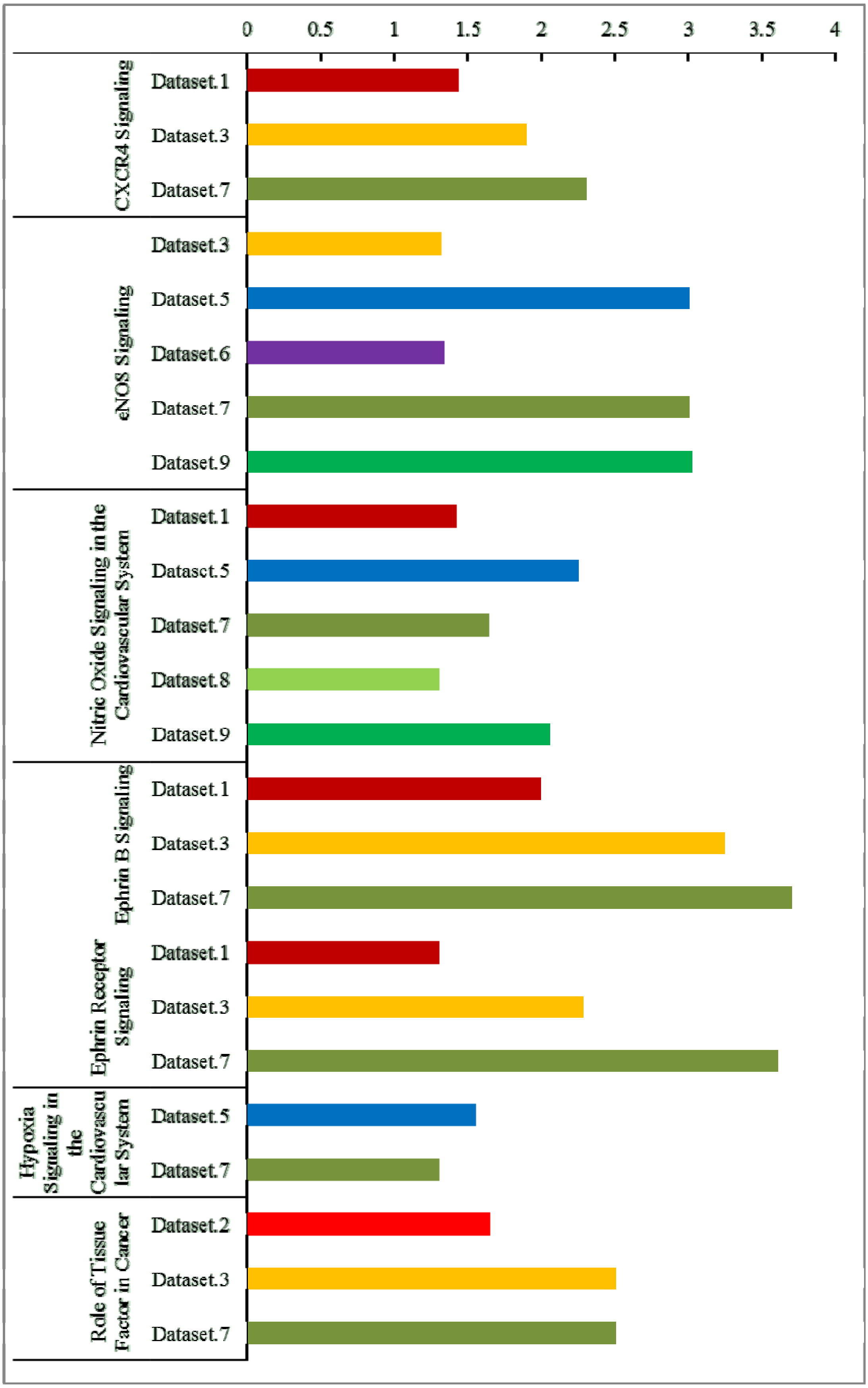

Supplemental Figure 4C

Supplemental Figure 4D

Panel D: toxin metabolism and oxidative stress related pathways

This panel includes 8 canonical pathways: (i). Aryl Hydrocarbon Resceptor Signaling existed in the normal pituitary (Dataset 7 and Dataset 9) and the pituitary mapping proteins dataset (Dataset 3), as well. However, this pathway is significantly changed in the NFPA with evidence in the upregulation of the key molecules (GST in Dataset 2; HSPCA, HSPCB, ESR1 and Bax in Dataset 5), and downregulation of the key molecules (HSP27, HSP90 (GRP94) and TGM2 in Dataset 2; ESR2 in Dataset 5) with the analysis of NFPA DEP data and invasive NFPA DEG (Dataset 2 and Dataset 5). (ii). Corticotropin Releasing Hormone Signaling existed in the normal pituitary (Dataset 7 and Dataset 9), however, this pathway is significantly changed in the NFPA with evidence in the upregulation of the key molecules (CALM, IP3R), and downregulation of the key molecules (ACTH, Nur77 and c-FOS) with the analysis of NFPA DEG data (Dataset 1). (iii). Glucocorticoid Receptor Signaling existed in the normal pituitary (Dataset 7 and Dataset 9) and the pituitary mapping proteins dataset (Dataset 3), as well. However this pathway is significantly changed in the NFPA with evidence in the upregulation of the key molecule (PI3K), and downregulation of the key molecules (HSP70, c-Fos, CCL2, BCL2, PRL, POMC) with the analysis of NFPA DEG data (Dataset 1). (iv). Glutathione Redox Reactions I pathway existed in the normal pituitary (Dataset 7), however, this pathway is significantly changed in the NFPA with evidence in the downregulation of the key molecule (GPX4) with the analysis of NFPA DEP data (Dataset 2). (v). Melatonin Signaling existed in the normal pituitary (Dataset 7) and the pituitary mapping proteins dataset (Dataset 3), as well. However, this pathway is significantly changed in the NFPA with evidence in the nitration of the key molecule (PKA), with the analysis of NFPA nitroproteins and their related proteins data (Dataset 4). (vi). Methylglyoxal Degradation III pathway existed in the normal pituitary (Dataset 7) and the pituitary mapping proteins dataset (Dataset 3), as well. However, this pathway is significantly changed in the NFPA with evidence in the upregulation of the key molecules (Aldose reductase (AKR1B1)) with the analysis of NFPA DEP data (Dataset 2). (vii). NRF2-mediated Oxidative Stress Response pathway existed in the normal pituitary (Dataset 7 and Dataset 8) and the pituitary mapping proteins dataset (Dataset 3), as well. However, this pathway is significantly changed in the NFPA with evidence in the upregulation of the key molecules (GST (GSTM2) and ERP29), and downregulation of the key molecules (HSP22, HSP27 and HSP90 (GRP94)) with the analysis of NFPA DEP data (Dataset 2). (viii). Superoxide Radicals Degradation pathway existed in the normal pituitary (Dataset 7) and the pituitary mapping proteins dataset (Dataset 3), as well. However this pathway is significantly changed in the NFPA with evidence in the upregulation of the key molecule (CAT) with the analysis of invasive NFPA DEP data (Dataset 6).

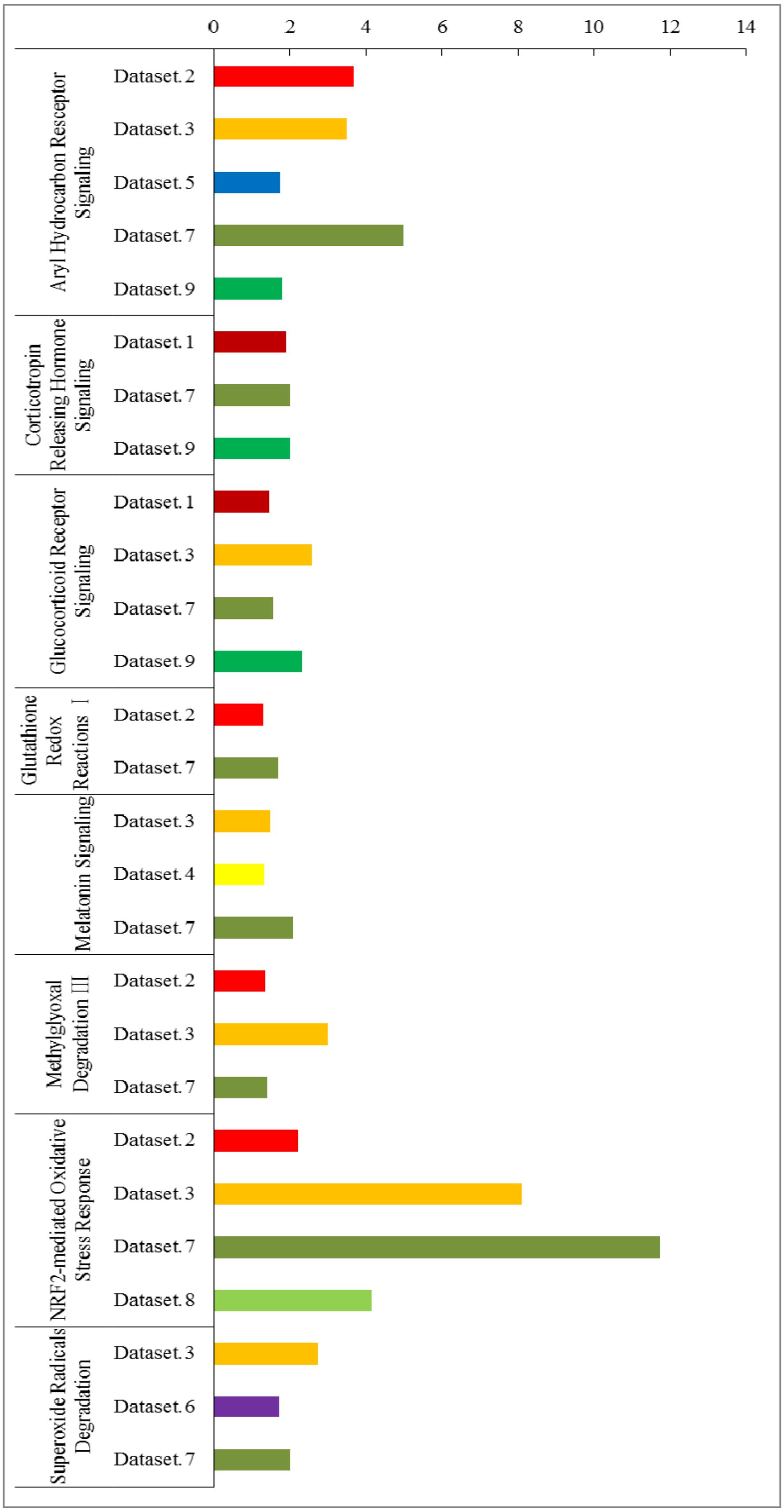

Supplemental Figure 4D

Supplemental Figure 4E
Panel E: Protein synthesis, degradation and amino acid metabolism related pathways

This panel includes 4 canonical pathways: (i). EIF2 Signaling existed in the normal pituitary (Dataset 7 and Dataset 9), however, this pathway is significantly changed in the NFPA with evidence in the upregulation of the key molecules (60S ribosomal subunit (RPL10 and RPL32), and downregulation of the key molecules (PI3K, PDK1, 40S ribosomal subunit (RPS2 and RPS2) and 60S ribosomal subunit (RPL18A)) with the analysis of invasive NFPA DEG data (Dataset 5). (ii). Polyamine Regulation in Colon Cancer pathway existed in the pituitary mapping proteins dataset (Dataset 3) and also significantly changed in the NFPA with evidence in the downregulation of the key molecules (ODC1 and SSAT (SAT1)) with the analysis of NFPA DEG data (Dataset 1). (iii). Putrescine Degradation III pathway existed in the normal pituitary (Dataset 7), however, this pathway is significantly changed in the NFPA with evidence in the upregulation of the key molecule (MAOB), and downregulation of the key molecules (ALDH2 and SSAT (SAT1)) with the analysis of NFPA DEG data (Dataset 1). (iv). Protein Ubiquitination Pathway existed in the normal pituitary (Dataset 7, Dataset 8 and Dataset 9) and the pituitary mapping proteins dataset (Dataset 3), as well. However, this pathway is significantly changed in the NFPA with evidence in the upregulation of the key molecules (HSPCA and HSPCB in Dataset 5), downregulation of the key molecules (HSPB8, GRP94 and HSPB1 in Dataset 2; E2 in Dataset 5), and nitration and nitration related key molecules (PSMA2 is nitrated, Ub is nitroprotein-interacted protein) with the analysis of NFPA DEP data, NFPA nitroproteins and their related proteins data and invasive NFPA DEG data (Dataset 2, Dataset 4 and Dataset 5).

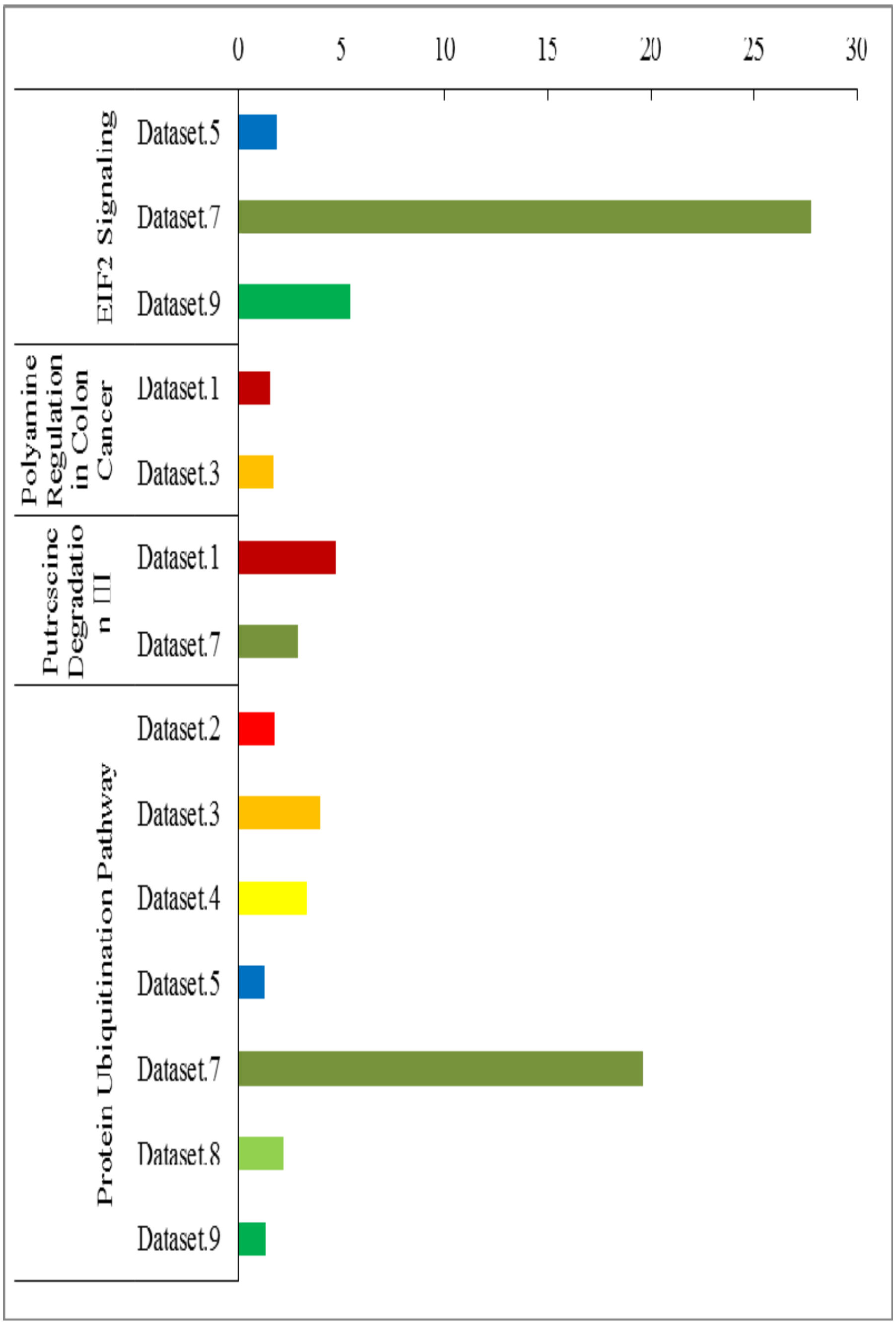

Supplemental Figure 4E

Supplemental Figure 4F
Panel F: Cell cycle, proliferation and apoptosis related pathways

This panel includes 13 canonical pathways: (i). 14-3-3-mediated Signaling existed in the normal pituitary (Dataset 7) and the pituitary mapping proteins dataset (Dataset 3). However, this pathway is significantly changed in the NFPA with evidence in the upregulation of the key molecule (VIM), and downregulation of the key molecule (14-3-3) with the analysis of NFPA DEP data (Dataset 2). (ii). Calcium Signaling existed in the normal pituitary (Dataset 7 and Dataset 8 –, however, this pathway is significantly changed in the NFPA with evidence in the upregulation of the key molecules (CALM, IP3R, PMCA, NCX (SLC8A2), SERCA and Myosin in Dataset 1; nACHR, NCX (SLC8A1) and Tropomyosin (TPM3, TPM4) in Dataset 5), and downregulation of the key molecules (DSCR1in Dataset 1; PKA in Dataset 5) with the analysis of NFPA DEG data and invasive NFPA DEG data (Dataset 1 and Dataset 5). (iii). Cardiac β-adrenergic Signaling existed in the normal pituitary (Dataset 7), however, this pathway is significantly changed in the NFPA with evidence in the upregulation of the key molecules (CACNA1I(T-Type calcium channel), CACNA1E(R-Type calcium channel), PPM1K, PPP1R11 and NCX), and downregulation of the key molecules (IPKA, AKAP, PKA and PKI(PKIG)) with the analysis of invasive NFPA DEG data (Dataset 5). (iv). ERK/MAPK Signaling existed in the normal pituitary (Dataset 7) and the pituitary mapping proteins dataset (Dataset 3), as well. However, this pathway is significantly changed in the NFPA with evidence in the upregulation of the key molecules (PI3K, Talin and cPLA2 in Dataset 1; FYN in Dataset 2; 14-3-3(YWHAG), PPM1K, PPP1R11 and ESR1 in Dataset 5), and downregulation of the key molecules (MKP2 in Dataset 1; 14-3-3(YWHAQ) and HSP27 in Dataset 2; PI3K, PKA, PPM1A, ESR2 and RPS6KA5 in Dataset 5) with the analysis of NFPA DEG data, NFPA DEP data and invasive NFPA DEG data (Dataset 1, Dataset 2 and Dataset 5). (v). IGF-1 Signaling existed in the normal pituitary (Dataset 7 and Dataset 9), however, this pathway is significantly changed in the NFPA with evidence in the upregulation of the key molecules (PI3K in Dataset 1; IGFBP (IGFBP5) and 14-3-3 (YWHAG) in Dataset 5), and downregulation of the key molecules (IGFBP (IGFBP3), FKHR and c-FOS in Dataset 1; IGFBP (IGFBP6) and 14-3-3 (YWHAQ) in Dataset 2; PI3K, PDK1 and PKA in Dataset 5) with the analysis of NFPA DEG data, NFPA DEP data and invasive NFPA DEG data (Dataset 1, Dataset 2 and Dataset 5). (vi). mTOR Signaling existed in the normal pituitary (Dataset 7 and Dataset 9), however, this pathway is significantly changed in the NFPA with evidence in the upregulation of the key molecule (PROTOR (PRR5)), and downregulation of the key molecules (PI3K, PDK1, RSK (RPS6KA5) and 40S Ribosome(RPS2 and RPS2)) with the analysis of invasive NFPA DEG data (Dataset 5). (vii). p53 Signaling is significantly changed in the NFPA with evidence in the upregulation of the key molecules (PI3K in Dataset 1; Slug, PUMA (BBC3) and BAX in Dataset 5), and downregulation of the key molecules (GADD45, NOXA, Bcl-2 and ZAC1 in Dataset 1; PI3K in Dataset 5) with the analysis of NFPA DEG data and invasive NFPA DEG data (Dataset 1 and Dataset 5). (viii). PEDF Signaling is significantly changed in the NFPA with evidence in the upregulation of the key molecules (PI3K and DOCK3 in Dataset 1; GDNF in Dataset 5), and downregulation of the key molecules (BCL-2 in Dataset 1; PI3K, TCF in Dataset 5) with the analysis of NFPA DEG data and invasive NFPA DEG data (Dataset 1 and Dataset 5). (ix). PI3K/AKT Signaling existed in the normal pituitary (Dataset 7 and Dataset 9) and the pituitary mapping proteins dataset (Dataset 3), as well. However, this pathway is significantly changed in the NFPA with evidence in the upregulation of the key molecules (HSP90 (HSPCA and HSPCB) and 14-3-3 (YWHAG) in Dataset 5), and downregulation of the key molecules (HSP90 (GRP94) and 14-3-3 (YWHAQ) in Dataset 2; PI3K p110 and PDK1 in Dataset 5) with the analysis of NFPA DEP data and invasive NFPA DEG data (Dataset 2 and Dataset 5). (x). Sonic Hedgehog Signaling existed in the normal pituitary (Dataset 7 and Dataset 9), however, this pathway is significantly changed in the NFPA with evidence in the nitration of the key molecule (PKA) with the analysis of NFPA nitroproteins and their related proteins data (Dataset 4). (xi). Tec Kinase Signaling existed in the normal pituitary (Dataset 8) and the pituitary mapping proteins dataset (Dataset 3), as well. However, this pathway is significantly changed in the NFPA with evidence in the upregulation of the key molecules (Gα and SRC(FYN)) with the analysis of NFPA DEP data (Dataset 2). (xii). Telomerase Signaling existed in the normal pituitary (Dataset 7 and Dataset 9), however, this pathway is significantly changed in the NFPA with evidence in the upregulation of the key molecules (HSP90 (HSPCA, HSPCB)), and downregulation of the key molecules (PI3K and PDK1) with the analysis of invasive NFPA DEG data (Dataset 5). (xiii). α-Adrenergic Signaling existed in the normal pituitary (Dataset 7) and the pituitary mapping proteins dataset (Dataset 3), as well. However, this pathway is significantly changed in the NFPA with evidence in the upregulation of the key molecules (Gβ, Calm, IP3R and NCX) with the analysis of NFPA DEG data (Dataset 1).

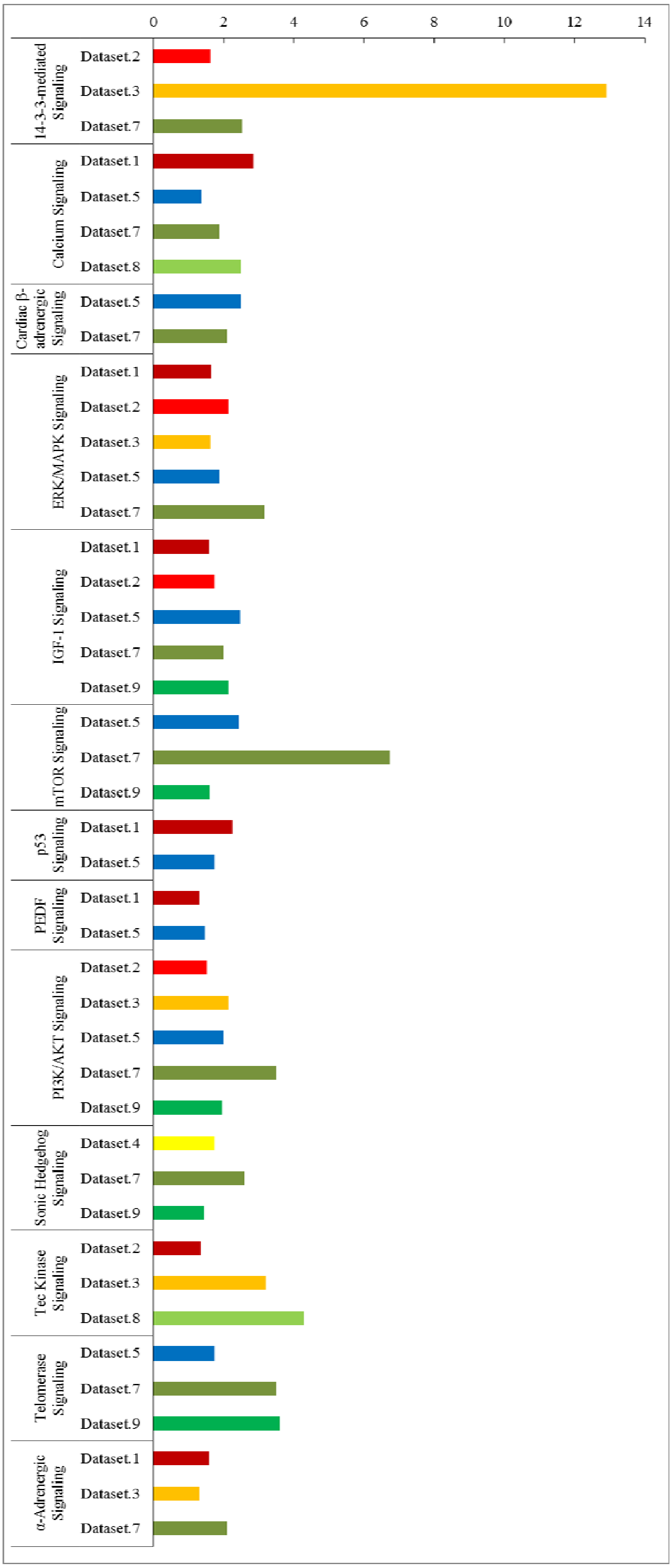

Supplemental Figure 4F

Supplemental Figure 4G
Panel G: Immunity related pathways

This panel includes 2 canonical pathways: (i). IL-1 Signaling existed in the normal pituitary (Dataset 7) and NFPA mapping proteins (Dataset 3), as well. However, significant nitration and nitration related key molecules (PKA is nitrated, IRAK2 is nitroprotein-interacted protein) appeared in IL-1 Signaling of NFPA with the analysis of NFPA nitroproteins and their related proteins data (Dataset 4). (ii). Role of NFAT in the Regulation of the Immune Response pathway, which only existed in the pituitary mapping proteins dataset (Dataset 3), showed significantly the upregulation of the key molecules (PI3K, G β , CALM, CSP (CSPG5) and IP3R), downregulation of the key molecule (c-FOS) with the analysis of NFPA DEG data (Dataset 1).

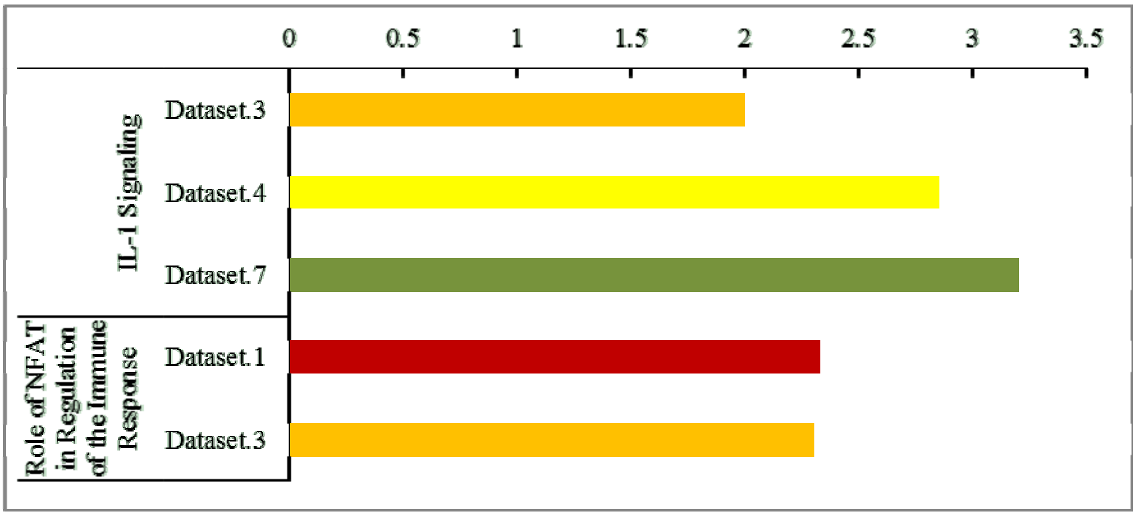

Supplemental Figure 4G

Supplemental Figure 4H

Panel H: ER stress related pathways

This panel includes 2 canonical pathways: (i). Endoplasmic Reticulum Stress Pathway existed in the normal pituitary (Dataset 7),and the pituitary mapping proteins dataset (Dataset 3), as well. However, the key molecules (GRP94 in Dataset 2; BIP (HSPA5 and HSPA6) in Dataset 6) showed significantly downregulation in the NFPA with the analysis of NFPA DEP data and invasive NFPA DEP data (Dataset 2 and Dataset 6). (ii). Unfolded protein response pathway existed in the normal pituitary (Dataset 7), and the pituitary mapping proteins dataset (Dataset 3), as well. However, the upregulation of the key molecule SREBP (SREBF1) and downregulation of the key molecules (PDI (P4HB), c/EBP, BCL2 and HSP70 (HSPA2)) was found in the NFPA with the analysis of NFPA DEG data (Dataset 1).

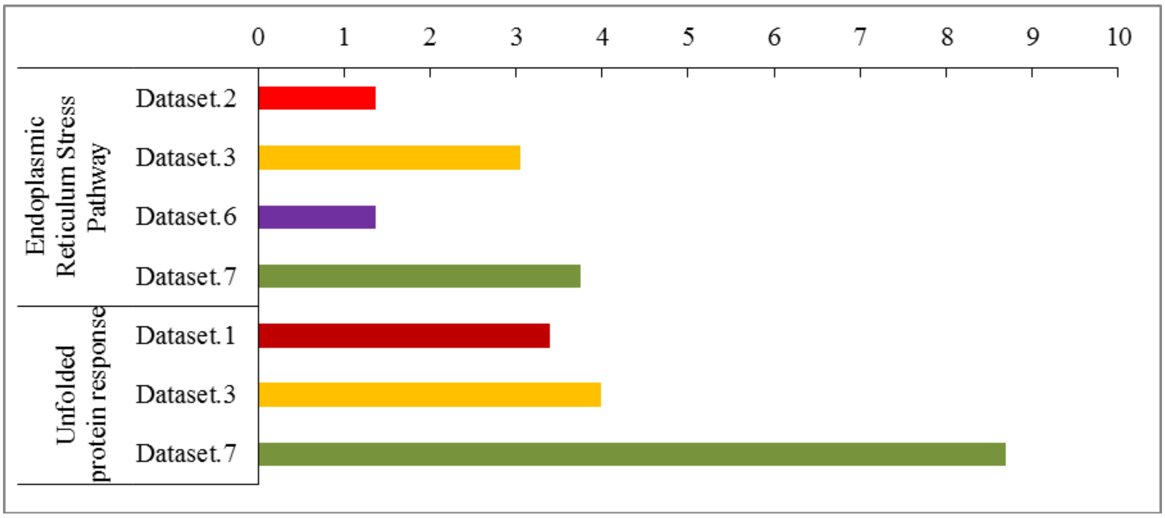

Supplemental Figure 4H

Supplemental Figure 4I
Panel I: others.

This panel includes 8 canonical pathways: (i). Aldosterone Signaling in Epithelial Cells pathway existed in the normal pituitary (Dataset 7 and Dataset 9) and the pituitary mapping proteins dataset (Dataset 3), as well. However the upregulation of the key molecules (HSPCA and HSPCB in Dataset 5) and downregulation of the key molecules (HSPB8, HSP90B1 (GRP94) and HSPB1 in Dataset 2; DNAJB6, PI3K and PDK1 in Dataset 5) was found significantly changed in the NFPA with the analysis of NFPA DEP data and invasive NFPA DEG data (Dataset 2 and Dataset 5). (ii). Docosahexaenoic acid (DHA) signaling is significantly changed in the NFPA with evidence in the upregulation of of the key molecules (PI3K in Dataset 1; BAX in Dataset 5), downregulation of the key molecules (FKHR and BCL2 in Dataset 1; PI3K and PDK1in Dataset 5) with the analysis of NFPA DEG data and invasive NFPA DEG data (Dataset 1 and Dataset 5). (iii). Endometrial Cancer Signaling existed in the normal pituitary (Dataset 7 and Dataset 9), however, this pathway is significantly changed in the NFPA with evidence in the downregulation of the key molecules (PI3K, PDK1 and E-cadherin) with the analysis of invasive NFPA DEG data (Dataset 5). (iv). Growth Hormone Signaling existed in the normal pituitary (Dataset 9), however, this pathway is significantly changed in the NFPA with evidence in the upregulation of the key molecules (PI3K in Dataset 1; CEBPA in Dataset 5), downregulation of the key molecules (GH, c-FOS and IGFBP3 in Dataset 1; GH in Dataset 2; PI3K and PDK1 in Dataset 5) with the analysis of NFPA DEP data and invasive NFPA DEG data (Dataset 2 and Dataset 5). (v). Hereditary Breast Cancer Signaling is significantly changed in the NFPA with evidence in the upregulation of the key molecules (PI3K in Dataset 1), downregulation of the key molecules (BLM, Wee1 and GADD45 in Dataset 1), and nitration and nitration related key molecules (Ub is nitroprotein-interacted protein) with the analysis of NFPA DEG data and NFPA nitroproteins and their related proteins data (Dataset 1 and Dataset 4). (vi). PPARα/RXRα Activation pathway existed in the normal pituitary (Dataset 7 and Dataset 9) and the pituitary mapping proteins dataset (Dataset 3), as well. However, this pathway is significantly changed in the NFPA with evidence in the downregulation of the key molecules (GH, HSP90 (GRP94) and APOA1) with the analysis of NFPA DEP data (Dataset 2). (vii). PXR/RXR Activation pathway existed in the normal pituitary (Dataset 7), however, this pathway is significantly changed in the NFPA with evidence in the nitration and nitration related key molecules (PKA is nitrated) with the analysis of NFPA nitroproteins and their related proteins data (Dataset 4). (viii). TR/RXR Activation pathway existed in the normal pituitary (Dataset 7 and Dataset 9), and the pituitary mapping proteins dataset (Dataset 3), as well. However, this pathway issignificantly changed in the NFPA with evidence in the upregulation of the key molecules (PI3K, ZAKI4 and SREBP in Dataset 1; F10 in Dataset 6), downregulation of the key molecules (GH1 and FASN in Dataset 1; F10 and GH1 in Dataset 2; GH1 in Dataset 6) with the analysis of NFPA DEG data, NFPA DEP data and invasive NFPA DEP data (Dataset 1, Dataset 2 and Dataset 6).

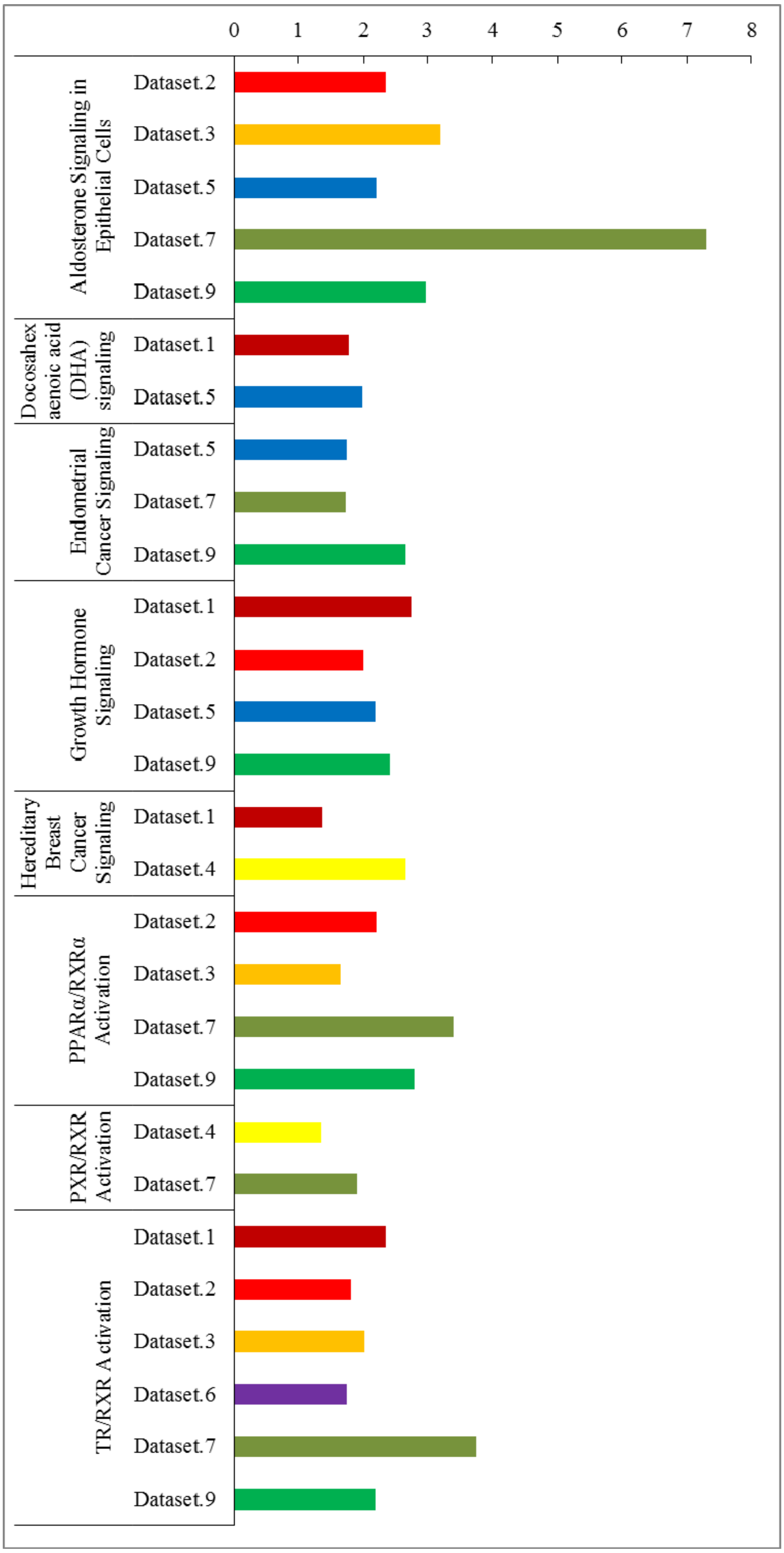

Supplemental Figure 4I
