# Supplementary material for: Multiomics-Based Signaling Pathway Network Alterations in Human Non-functional Pituitary Adenomas
Source: Front Endocrinol (Lausanne). 2019 Dec 17;10:835. doi: 10.3389/fendo.2019.00835 (PMC6928143; doi:10.3389/fendo.2019.00835)
Supplement: Supplementary file 1 [file Presentation_1.zip › Supplemental Table 1_v2.pdf]

Supplemental Table 1. Documented omics data that were used to construct molecular networks in human pituitary adenomas

Category	Data type	Reference	Sample and size	Methods	Genes/Proteins
NFPA	DEGs	Moreno CS, *et al*. Cancer Res. 2005, 65(22):10214-10222. [**1**]	3 controls; 11 NFPAs	Microarray	280 DEGs (297 probe sets)
	DEPs	Moreno CS, et al. Cancer Res. 2005, 65(22):10214-10222. [**1**]	8 controls; 11 NFPAs	2DGE, MS	50 DEPs (93 differential protein spots)
	Mapping proteins	Zhan X, *et al*. Proteomics. 2003, 3(5): 699-713. [**2**]	1 NFPA	2DGE, MS	111 proteins
		Wang XW, et al. Electrophoresis. 2015, 36: 1289-1304. [**3**]	1 NFPA	2DGE, MS	107 proteins
	Mapping nitroproteins	Zhan X, *et al*. Anal Biochem. 2006, 354(2): 279-289. [**4**]	1 NFPA	NTAC, MS	9 nitrated proteins, 3 nitroprotein-interacted protein
Invasive vs noninvasive NFPAs	DEGs	Galland F, *et al*. Endocr Relat Cancer. 2010, 17(2): 361-371. [**5**]	40 NFPAs (22 invasive, 18 non-invasive)	Microarray	346 DEGs
		Zhou W, *et al*. J Clin Endocrinol Metab. 2011, 96(8): E1237-E1245. [**6**]	41 NFPAs (15 invasive, 26 non-invasive)	IHC, PCR, WB	4 DEGs
	DEPs	Zhan X, *et al*. Electrophoresis. 2014, 35(15): 2184-2194. [**7**]	8 NFPAs (4 invasive, 4 non-invasive)	2DGE, MS	57 DEPs

Pituitary control	Mapping proteins	Beranova-Giorgianni S, *et al*. Proteomics. 2002, 2(5): 534-542. [**8**]	1 control	2DGE, MS	38 proteins
		Giorgianni F, *et al*. Electrophoresis. 2003, 24(1-2): 253-259. [**9**]	1 control	2DGE, MS	127 proteins
		Zhao Y, *et al*. Anal Chem. 2005, 77(16): 5324-5331. [**10**]	1 control	IEF, SDS-PAGE, Nano-HPLC-MS/MS	1449 proteins
	Mapping nitroproteins	Zhan X, *et al*. INT J MASS SPECTROM. 2007, 259(1): 96-104. [**11**]	1 control	2DGE, WB, MS	4 nitrated proteins
		Zhan X, *et al*. Biochem Biophys Res Commun. 2004, 325(4): 1180-1186. [**12**]	1 control	2DGE, WB, MS	4 nitrated proteins
	Mapping phosphoproteins	Giorgianni F, *et al*. Proteomics. 2004, 4(3): 587-598. [**13**]	1 control	IMAC-based enrichment, LC-MS/MS	6 phosphorylated proteins
		Beranova-Giorgianni S, *et al*. Pituitary. 2006, 9(2): 109-120. [**14**]	1 control	IMAC-based enrichment, IEF-LC-MS/MS	26 phosphorylated proteins

Note: NFPA = nonfunctional pituitary adenoma.

References:

[1] Moreno CS, Evans CO, Zhan X, Okor M, Desiderio DM, Oyesiku NM. Novel molecular signaling and classification of human clinically nonfunctional pituitary adenomas identified by gene expression profiling and proteomic analyses. Cancer Res 2005; 65: 10214-22.

[2] Zhan X, Desiderio DM. A reference map of a human pituitary adenoma proteome. Proteomics 2003; 3: 699-713.

[3] Wang X, Guo T, Peng F, Long Y, Mu Y, Yang H, et al. Proteomic and functional profiles of a follicle-stimulating hormone positive human nonfunctional pituitary adenoma. Electrophoresis 2015; 36: 1289-304.

[4] Zhan X, Desiderio DM. Nitroproteins from a human pituitary adenoma tissue discovered with a nitrotyrosine affinity column and tandem mass spectrometry. Anal Biochem 2006; 354: 279-89.

[5] Galland F, Lacroix L, Saulnier P, Dessen P, Meduri G, Bernier M, et al. Differential gene expression profiles of invasive and non-invasive non-functioning pituitary adenomas based on microarray analysis. Endocr Relat Cancer 2010; 17: 361-71.

[6] Zhou W, Song Y, Xu H, Zhou K, Zhang W, Chen J, et al. In nonfunctional pituitary adenomas, estrogen receptors and slug contribute to development of invasiveness. J Clin Endocrinol Metab 2011; 96: E1237-45.

[7] Zhan X, Desiderio DM, Wang X, Zhan X, Guo T, Li M, et al. Identification of the proteomic variations of invasive relative to non-invasive non-functional pituitary adenomas. Electrophoresis 2014; 35: 2184-94.

[8] Beranova-Giorgianni S, Giorgianni F, Desiderio DM. Analysis of the proteome in the human pituitary. Proteomics 2002; 2: 534-42.

[9] Giorgianni F, Desiderio DM, Beranova-Giorgianni S. Proteome analysis using isoelectric focusing in immobilized pH gradient gels followed by mass spectrometry. Electrophoresis 2003; 24: 253-9.

[10] Zhao Y, Giorgianni F, Desiderio DM, Fang B, Beranova-Giorgianni S. Toward a global analysis of the human pituitary proteome by multiple gel-based technology. Anal Chem 2005; 77: 5324-31.

[11] Zhan X, Desiderio DM. Linear ion-trap mass spectrometric characterization of human pituitary nitrotyrosine-containing proteins. Int J Mass Spectrom 2007; 259: 96-104.

[12] Zhan X, Desiderio DM. The human pituitary nitroproteome: detection of nitrotyrosyl-proteins with two-dimensional Western blotting, and amino acid sequence determination with mass spectrometry. Biochem Biophys Res Commun 2004; 325: 1180-6.

[13] Giorgianni F, Beranova-Giorgianni S, Desiderio DM. Identification and characterization of phosphorylated proteins in the human pituitary. Proteomics 2004; 4: 587-98.

[14] Beranova-Giorgianni S, Zhao Y, Desiderio DM, Giorgianni F. Phosphoproteomic analysis of the human pituitary. Pituitary 2006; 9: 109-120.
