# Supplementary material for: Multiomics-Based Signaling Pathway Network Alterations in Human Non-functional Pituitary Adenomas
Source: Front Endocrinol (Lausanne). 2019 Dec 17;10:835. doi: 10.3389/fendo.2019.00835 (PMC6928143; doi:10.3389/fendo.2019.00835)
Supplement: Supplementary file 1 [file Presentation_1.zip › Supplemental Table 2-1.pdf]

Supplemental Table 2-1. Clinical characteristic of NFPA and control tissue samples. DNT = do not test.

Group	Sex	Age	Clinicla information	Immunohistochemistry	Experiments
Control	Female	40	White, Multiple toxic compounds. Blood: HepB (+), HepC (+), HIV(−).	DNT	PTMScan; Western blot
	Male	36	White, Multiple toxic materials. Blood alcohol = 0.5 g/L. Blood: HepB (+), HepC (−), HIV (−).	DNT	PTMScan; Western blot
	Female	34	Black, Gunshot wound to chest. Blood alcohol = 0.3 g/L; no drugs. Blood: HepB (+), HepC (−), HIV (−).	DNT	PTMScan; Western blot
	Female		White, 15 h gunshot wound to head. No drugs or alcohol. Blood: HepB (−), HepC (−), HIV (−).	DNT	PTMScan
	Male	45	White, Drowning. Blood alcohol =3.1 g/L; no other drugs detected. Blood: HepB (+), HepC (+), HIV (-).	DNT	Western blot
NFPA	Female	43	NFPA in sellar region. Sellar floor bone thinning, enriched blood supply in tumor, and tumor size: 4 x 3 x 3 cm^3	ACTH (-), hGH (-), PRL (-), FSH (+), LH (-), TSH (-)	PTMScan; Western blot
	Male	53	NFPA in sellar region. Sellar floor bone thinning, and tumor size: 3 x 3 x 2.5 cm^3	ACTH (-), hGH (-), PRL (-), FSH (-), LH (-), TSH (-)	PTMScan; Western blot
	Female	43	NFPA in sellar region. Adehesion of surrounding tissues, and tumoer size: 4.5 x 4 x6 cm^3	ACTH (-), hGH (-), PRL (-), FSH (+), LH (-), TSH (-)	PTMScan; Western blot
	Male	58	NFPA in sellar region. Sellar floor bone destruction, enriched blood supply in tumor, and tumor size: 4.5 x 3 x 3 cm^3	ACTH (-), hGH (-), PRL (-), FSH (-), LH (-), TSH (-)	PTMScan; Western blot
