# Supplementary material for: Multiomics-Based Signaling Pathway Network Alterations in Human Non-functional Pituitary Adenomas
Source: Front Endocrinol (Lausanne). 2019 Dec 17;10:835. doi: 10.3389/fendo.2019.00835 (PMC6928143; doi:10.3389/fendo.2019.00835)
Supplement: Supplementary file 1 [file Presentation_1.zip › Supplemental Table 2-2.pdf]

Supplemental Table 2-2. The primary and secondary antibodies used for Western blot.

Target protein	Origin	Antibody dilution	Company, Cat#
GSK-3α	Rabbit	1: 1000, 5% BSA, 0.1% Tween, 1×TBS	CST, 4337
GSK-3β	Rabbit	1: 1000, 5% BSA, 0.1% Tween, 1×TBS	CST, 12456
P-GSK-3β (Ser9)	Rabbit	1: 1000, 5% BSA, 0.1% Tween, 1×TBS	CST, 5558
P-GSK-3α/β (Ser21/9)	Rabbit	1: 1000, 5% BSA, 0.1% Tween, 1×TBS	CST, 8566
β-Catenin	Rabbit	1: 1000, 5% non-fat milk, 0.1% Tween, 1×TBS	CST, 8480T
PRAS40	Rabbit	1: 1000, 5% BSA, 0.1% Tween, 1×TBS	CST, 2691T
P-PRAS40 (Thr246)	Rabbit	1: 1000, 5% non-fat milk, 0.1% Tween, 1×TBS	CST, 13175S
β-actin	Mouse	1: 1200, 5% non-fat milk, 0.1% Tween, 1×TBS	ABGENT, AM1021B
Anti-rabbit IgG	Goat	1: 2000, 5% non-fat milk, 0.1% Tween, 1×TBS	CST, 7074
Anti-mouse Ig	Goat	1: 5000, 0.1% Tween, 1×TBS	ABGENT, ASS1027
