# Supplementary material for: Multiomics-Based Signaling Pathway Network Alterations in Human Non-functional Pituitary Adenomas
Source: Front Endocrinol (Lausanne). 2019 Dec 17;10:835. doi: 10.3389/fendo.2019.00835 (PMC6928143; doi:10.3389/fendo.2019.00835)
Supplement: Supplementary file 1 [file Presentation_1.zip › Supplemental Table 3.pdf]

Supplemental Table 3. The network-eligible IDs of each dataset for IPA analysis

Dataset	Total IDs	Unmatched IDs	Matched IDs	Duplicated IDs	Network-eligible IDs
1	297	31	266	19	247
2	76	7	69	25	44
3	218	16	202	12	190
4	13	0	13	0	13
5	346	101	245	0	245
6	59	3	56	2	54
7	1469	113	1356	4	1352
8	10	0	10	0	10
9	29	1	28	1	27
