# Supplementary material for: Multiomics-Based Signaling Pathway Network Alterations in Human Non-functional Pituitary Adenomas
Source: Front Endocrinol (Lausanne). 2019 Dec 17;10:835. doi: 10.3389/fendo.2019.00835 (PMC6928143; doi:10.3389/fendo.2019.00835)
Supplement: Supplementary file 1 [file Presentation_1.zip › Supplemental Table 4.pdf]

Supplemental Table 4. Differences in the types of molecules between omics datasets.

Type(s)	Dataset 1		Dataset 2		Dataset 3		Dataset 4		Dataset 5		Dataset 6		Dataset 7		Dataset 8		Dataset 9	
	**n**	**%**	**n**	**%**	**n**	**%**	**n**	**%**	**n**	**%**	**n**	**%**	**n**	**%**	**n**	**%**	**n**	**%**
Chemical - endogenous mammalian	1	0.4%	_	_	_	_	_	_	_	_	_	_	_	_	_	_	_	_
Chemical-endogenous non-mammalian	_	_	_	_	_	_	_	_	1	0.4%	_	_	_	_	_	_	_	_
Cytokine	3	1.1%	7	10.1%	_	_	1	7.7%	1	0.4%	_	_	9	0.7%	_	_	2	7.1%
G-protein coupled receptor	3	1.1%	_	_	_	_	_	_	2	0.8%	_	_	12	0.9%	_	_	_	_
Growth factor	5	1.9%	19	27.5%	1	0.5%	_	_	1	0.4%	3	5.4%	3	0.2%	_	_	2	7.1%
Ion channel	5	1.9%	_	_	4	2.0%	_	_	2	0.8%	1	1.8%	17	1.3%	_	_	_	_
Ligand-dependent nuclear receptor	2	0.8%	_	_	1	0.5%	_	_	2	0.8%	_	_	2	0.1%	_	_	_	_
Kinase	11	4.1%	2	2.9%	7	3.5%	2	15.4%	14	5.7%	3	5.4%	63	4.6%	2	20.0%	2	7.1%
Peptidase	6	2.3%	3	4.3%	10	5.0%	1	7.7%	3	1.2%	7	12.5%	71	5.2%	1	10.0%	_	_
Phosphatase	5	1.9%	_	_	3	1.5%	_	_	2	0.8%	_	_	17	1.3%	_	_	_	_
Enzyme	40	15.0%	16	23.2%	60	29.7%	4	30.8%	35	14.3%	10	17.9%	403	29.7%	1	10.0%	5	17.9%

Transmembrane receptor	8	3.0%	_	_	_	_	_	_	4	1.6%	1	1.8%	23	1.7%	_	_	_	_
Transporter	20	7.5%	3	4.3%	13	6.4%	_	_	12	4.9%	5	8.9%	111	8.2%	_	_	_	_
Transcription regulator	45	16.9%	1	1.4%	17	8.4%	_	_	26	10.6%	1	1.8%	84	6.2%	_	_	_	_
Translation regulator	_	_	_	_	4	2.0%	_	_	2	0.8%	_	_	26	1.9%	_	_	1	3.6%
Other	112	42.1%	18	26.1%	82	40.6%	5	38.5%	138	56.3%	25	44.6%	515	38.0%	6	60.0%	16	57.1%
**Total**	266	100.0%	69	100.0%	202	100.0%	13	100.0%	245	100.0%	56	100.0%	1356	100.0%	10	100.0%	28	100.0%
