# Supplementary material for: Multiomics-Based Signaling Pathway Network Alterations in Human Non-functional Pituitary Adenomas
Source: Front Endocrinol (Lausanne). 2019 Dec 17;10:835. doi: 10.3389/fendo.2019.00835 (PMC6928143; doi:10.3389/fendo.2019.00835)
Supplement: Supplementary file 1 [file Presentation_1.zip › Supplemental Table 5_v1.pdf]

Supplemental Table 5. A total of 861 hub-molecules that were extracted from 62 networks from 9 datasets.

Hub-molecule panel	Dataset number	Hub molecule serial number	Hub-mulecule	Note	Hub-molecule full name	Function description
**Cytoskeleton associated proteins**		C.N.N.N.10.001	ACTA2		Alpha-actin-2	Actins are highly conserved proteins that are involved in various types of cell motility and are ubiquitously expressed in all eukaryotic cells.
		C.N.M.N.9.004	ACTB		Beta-actin	
		C.N.N.N.10.002	ACTC1		Alpha-cardiac actin	
		C.N.P.N.11.002	Actin		Actin	
		N.N.DG.N.1.001	Actin		Actin	
		C.N.N.N.10.003	Actin		Actin	
		N.I.DG.N.12.002	Actin		Actin	
		C.N.M.N.9.005	ACTN1		Alpha-actinin-1	F-actin cross-linking protein which is thought to anchor actin to a variety of intracellular structures. This is a bundling protein.
		C.N.P.N.11.013	F Actin		F Actin	
		N.I.DG.N.12.042	F Actin		F Actin	
		N.N.DG.N.1.035	F Actin		F Actin	
		C.N.N.N.10.005	F Actin		F Actin	
		C.N.N.N.10.006	G-Actin		G-Actin	
		N.N.DG.N.1.006	Alpha actinin		Alpha actinin	
		N.I.DG.N.12.044	FLNA		Filamin-A	Actin crosslink formation, epithelial to mesenchymal transition
		C.N.P.N.11.005	CFL1		Cofilin-1	Binds to F-actin and exhibits pH-sensitive F-actin depolymerizing activity. Regulates actin cytoskeleton dynamics. Important for normal progress through mitosis and normal cytokinesis. Plays a role in the regulation of cell morphology and cytoskeletal organization.

C.N.M.N.9.088	EMD	Emerin	Stabilizes and promotes the formation of a nuclear actin cortical network.
N.N.DG.N.1.034	EZR	Ezrin	Extrinsic component of membrane, cytoskeleton, plasma membrane
C.N.M.N.9.096	FSCN1	Fascin	Organizes filamentous actin into bundles with a minimum of 4.1:1 actin/fascin ratio.
N.N.DP.N.2.068	VIM	Vimentin	Vimentins are class-III intermediate filaments found in various non-epithelial cells, especially mesenchymal cells. Vimentin is attached to the nucleus, endoplasmic reticulum, and mitochondria, either laterally or terminally.
C.N.M.N.9.253	VIM	Vimentin	
C.N.M.N.9.248	TUBB	Tubulin beta chain	Tubulin is the major constituent of microtubules. It binds two moles of GTP, one at an exchangeable site on the beta chain and one at a non-exchangeable site on the alpha chain.
C.N.M.N.9.249	TUBB3	Tubulin beta-3 chain	
C.N.P.N.11.053	TUBB4B	Tubulin beta-4B chain	
C.N.M.N.9.013	Alpha tubulin	Alpha tubulin	
C.N.M.N.9.022	Beta Tubulin	Beta Tubulin	
N.N.M.N.3.003	ACTB	Beta-actin	
N.N.M.N.3.004	Actin	Actin	
N.N.M.N.3.011	Alpha tubulin	Alpha tubulin	
N.N.M.N.3.020	Beta Tubulin	Beta Tubulin	
N.N.M.N.3.051	F Actin	F Actin	
N.N.M.N.3.145	TUBA1A	Tubulin alpha-1A chain	
N.N.M.N.3.146	TUBA1B		
N.N.M.N.3.147	TUBA1C		
N.N.M.N.3.148	TUBA4A		
N.N.M.N.3.149	TUBB		
N.N.M.N.3.150	TUBB2A		
N.N.M.N.3.151	TUBB3		
N.N.M.N.3.152	TUBB4A		

	N.N.M.N.3.153	TUBB4B		
	N.N.M.N.3.154	tubulin (complex)		
	N.N.M.N.3.155	tubulin (family)		
	N.N.M.N.3.064	GSN	Gelsolin	Calcium-regulated, actin-modulating protein that binds to the plus (or barbed) ends of actin monomers or filaments, preventing monomer exchange (end-blocking or capping). It can promote the assembly of monomers into filaments (nucleation) as well as sever filaments already formed. Plays a role in ciliogenesis.
	N.N.DG.N.1.116	Cofilin		Binds to F-actin and exhibits pH-sensitive F-actin depolymerizing activity. Regulates actin cytoskeleton dynamics. Important for normal progress through mitosis and normal cytokinesis. Plays a role in the regulation of cell morphology and cytoskeletal organization.
**Extracellular matrix and cell junction related proteins**	N.I.DG.N.12.027	Collagen type Ⅰ	Collagen type Ⅰ	Type I collagen is a member of group I collagen (fibrillar forming collagen).
	N.N.DP.N.2.010	Collagen type Ⅰ	Collagen type Ⅰ	
	N.N.DG.N.1.021	Collagen type Ⅳ	Collagen type Ⅳ	Type IV collagen is the major structural component of glomerular basement membranes (GBM), forming a 'chicken-wire' meshwork together with laminins, proteoglycans and entactin/nidogen
	N.N.DG.N.1.022	Collagen(s)	Collagen(s)	
	C.N.P.N.11.007	Collagen(s)	Collagen(s)	
	N.I.DG.N.12.028	Collagen(s)	Collagen(s)	
	N.N.DG.N.1.020	COL2A1	Collagen alpha-1(II) chain	
	N.N.DG.N.1.065	Laminin	Laminin	Binding to cells via a high affinity receptor, laminin is thought to mediate the attachment, migration and organization of cells into tissues during embryonic development by interacting with other extracellular matrix components.
	N.I.DG.N.12.073	Laminin	Laminin	
	N.N.DG.N.1.060	Integrin	Integrin	
	N.N.DP.N.2.040	Integrin	Integrin	
	N.I.DG.N.12.069	Integrin	Integrin	

N.N.DP.N.2.034	ICAM3	Intercellular adhesion molecule 3	ICAM proteins are ligands for the leukocyte adhesion protein LFA-1 (integrin alpha-L/beta-2). ICAM3 is also a ligand for integrin alpha-D/beta-2.
C.N.P.N.11.058	VTN	Vitronectin	Vitronectin is a cell adhesion and spreading factor found in serum and tissues. Vitronectin interact with glycosaminoglycans and proteoglycans. Is recognized by certain members of the integrin family and serves as a cell-to-substrate adhesion molecule
N.I.DG.N.12.081	Mmp	Matrix metalloproteinase	
N.N.DG.N.1.104	SELL	L-selectin	Cell surface adhesion protein. Mediates the adherence of lymphocytes to endothelial cells of high endothelial venules in peripheral lymph nodes. Promotes initial tethering and rolling of leukocytes in endothelia.
C.N.M.N.9.065	DSP	Desmoplakin	Major high molecular weight protein of desmosomes.
N.N.DG.N.1.017	CDH2	Cadherin-2	Cadherins are calcium-dependent cell adhesion proteins. They preferentially interact with themselves in a homophilic manner in connecting cells; cadherins may thus contribute to the sorting of heterogeneous cell types.
C.N.P.N.11.049	SPP1	Secreted phosphoprotein 1	Extracellular matrix binding
N.N.M.N.3.034	Collagen type Ⅰ		
N.N.M.N.3.035	Collagen type Ⅳ		
N.N.M.N.3.036	Collagen(s)		
N.N.M.N.3.082	Integrin		
N.N.M.N.3.086	Laminin		
N.N.DG.N.1.115	VCAN	Versican core protein	May play a role in intercellular signaling and in connecting cells with the extracellular matrix. May take part in the regulation of cell motility, growth and differentiation. Binds hyaluronic acid.

**ROCK**	N.N.DG.N.1.102	Rock	Rho-associated protein kinase	Protein kinase which is a key regulator of actin cytoskeleton and cell polarity. Involved in regulation of smooth muscle contraction, actin cytoskeleton organization, stress fiber and focal adhesion formation, neurite retraction, cell adhesion and motility via phosphorylation of DAPK3, GFAP, LIMK1, LIMK2, MYL9/MLC2, PFN1 and PPP1R12A.
	N.N.DP.N.2.059	Rock	Rho-associated protein kinase	
	C.N.P.N.11.044	Rock	Rho-associated protein kinase	
	N.I.DG.N.12.110	Rock	Rho-associated protein kinase	
	N.N.M.N.3.129	Rock		
**MAPK signaling pathway related proteins**	N.N.DG.N.1.030	ERK (MAPK1)	extracellular signal-regulated kinases/mitogen-activated protein kinase 1	It acts as an essential component of the MAP kinase signal transduction pathway. MAPK1/ERK2 and MAPK3/ERK1 are the 2 MAPKs which play an important role in the MAPK/ERK cascade.the MAPK/ERK cascade mediates diverse biological functions such as cell growth, adhesion, survival and differentiation through the regulation of transcription, translation, cytoskeletal rearrangements.
	N.I.DP.N.13.014	ERK	extracellular signal-regulated kinases/mitogen-activated	
	N.N.DP.N.2.015	ERK	extracellular signal-regulated kinases/mitogen-activated	
	C.N.P.N.11.010	ERK	extracellular signal-regulated kinases/mitogen-activated	

N.I.DG.N.12.037	ERK	extracellular signal-regulated kinases/mitogen-activated	
N.N.DP.N.2.016	ERK1/2	extracellular signal-regulated kinases/mitogen-activated	
N.I.DG.N.12.038	ERK1/2	extracellular signal-regulated kinases/mitogen-activated	
N.I.DP.N.13.015	ERK1/2	extracellular signal-regulated kinases/mitogen-activated	
N.N.DG.N.1.031	ERK1/2	extracellular signal-regulated kinases/mitogen-activated	
C.N.P.N.11.011	ERK1/2	extracellular signal-regulated kinases/mitogen-activated	
N.N.DG.N.1.062	Jnk	mitogen-activated protein kinase 8	Serine/threonine-protein kinase involved in various processes such as cell proliferation, differentiation, migration, transformation and programmed cell death.
N.I.DG.N.12.071	Jnk	mitogen-activated protein kinase 8	
N.N.DP.N.2.041	Jnk	mitogen-activated protein kinase 8	
N.I.DP.N.13.025	Jnk	mitogen-activated protein kinase 8	
C.N.M.N.9.140	MAP1B	Microtubule-associated protein 1B	Facilitates tyrosination of alpha-tubulin in neuronal microtubules (By similarity). Phosphorylated MAP1B may play a role in the cytoskeletal changes that accompany neurite extension.

C.N.M.N.9.141	MAP2K1	Mitogen-activated protein kinase kinase 1	It acts as an essential component of the MAP kinase signal transduction pathway. Binding of extracellular ligands such as growth factors, cytokines and hormones to their cell-surface receptors activates RAS and this initiates RAF1 activation.
C.N.M.N.9.142	MAP2K1/2	Mitogen-activated protein kinase kinase 1	
C.N.M.N.9.143	MAP3K3	Mitogen-activated protein kinase kinase kinase 3	Component of a protein kinase signal transduction cascade. Mediates activation of the NF-kappa-B, AP1 and DDIT3 transcriptional regulators.
N.N.DG.N.1.069	Mapk	Mitogen-activated protein kinase 1	Serine/threonine kinase which acts as an essential component of the MAP kinase signal transduction pathway. MAPK1/ERK2 and MAPK3/ERK1 are the 2 MAPKs which play an important role in the MAPK/ERK cascade.
N.N.DP.N.2.044	Mapk	Mitogen-activated protein kinase 1	
N.I.DG.N.12.077	Mapk	Mitogen-activated protein kinase 1	
N.I.DP.N.13.030	Mapk	Mitogen-activated protein kinase 1	
N.I.DG.N.12.078	MAP2K1/2	Mitogen-activated protein kinase 1	
N.N.DP.N.2.048	P38 MAPK	MAPK14 / MAPK1	
C.N.P.N.11.028	P38 MAPK	MAPK14 / MAPK1	
N.I.DG.N.12.086	P38 MAPK	MAPK14 / MAPK1	
N.I.DP.N.13.035	P38 MAPK	MAPK14 / MAPK1	
N.N.DG.N.1.070	Mek (MAP2K7)	Dual specificity mitogen-activated protein kinase kinase 7	This kinase is involved in the signal transduction mediating the cell responses to proinflammatory cytokines, and environmental stresses.

N.N.DP.N.2.045	Mek	Dual specificity mitogen-activated protein kinase kinase 7	
C.N.P.N.11.026	Mek	Dual specificity mitogen-activated protein kinase kinase 7	
N.I.DG.N.12.080	Mek	Dual specificity mitogen-activated protein kinase kinase 7	
N.N.DG.N.1.098	Rac (AKT1)	RAC-alpha serine/threonine-protein kinase	Regulate many processes including metabolism, proliferation, cell survival, growth and angiogenesis.cellular response to insulin stimulus
C.N.P.N.11.041	Rac	RAC-alpha serine/threonine-protein	
N.I.DG.N.12.104	Rac	RAC-alpha serine/threonine-protein	
N.I.DP.N.13.041	Rac	RAC-alpha serine/threonine-protein	
C.N.P.N.11.042	Ras	Ras-like protein	
N.N.DG.N.1.100	Ras	Ras-like protein	
N.N.DP.N.2.058	Ras	Ras-like protein	
C.N.M.N.9.201	Ras	Ras-like protein	
N.I.DG.N.12.107	Ras	Ras-like protein	
N.I.DG.N.12.106	Ras homolog	Ras-like protein	

N.I.DG.N.12.113	Shc	SHC-transforming protein 1	Signaling adapter that couples activated growth factor receptors to signaling pathways.once phosphorylated, couple activated receptor tyrosine kinases to Ras via the recruitment of the GRB2/SOS complex and are implicated in the cytoplasmic propagation of mitogenic signals. Isoform p66Shc is involved in signal transduction pathways that regulate the cellular response to oxidative stress and life span, and plays a role in the regulation of endothelial cell migration and sprouting angiogenesis.
N.N.DP.N.2.060	Shc	SHC-transforming protein	
N.N.DG.N.1.029	DUSP4	Dual specificity protein phosphatase 4	Regulates mitogenic signal transduction by dephosphorylating both Thr and Tyr residues on MAP kinases ERK1 and ERK2.
N.I.DG.N.12.111	Rsk	Ribosomal protein S6 kinase alpha	Serine/threonine-protein kinase that acts downstream of ERK (MAPK1/ERK2 and MAPK3/ERK1) signaling and mediates mitogenic and stress-induced activation of the transcription factors CREB1, ETV1/ER81 and NR4A1/NUR77, regulates translation through RPS6 and EIF4B phosphorylation, and mediates cellular proliferation, survival, and differentiation by modulating mTOR signaling and repressing pro-apoptotic function of BAD and DAPK1.
C.N.M.N.9.198	RAB7A	Ras-related protein Rab-7a	Key regulator in endo-lysosomal trafficking.
N.I.DG.N.12.118	Sos	(sos1) son of sevenless homolog 1	Promotes the exchange of Ras-bound GDP by GTP.
N.N.M.N.3.048	ERK		
N.N.M.N.3.049	ERK1/2		
N.N.M.N.3.084	Jnk		
N.N.M.N.3.096	MAP2K1/2		
N.N.M.N.3.097	MAP3K8		
N.N.M.N.3.098	Mapk		

	N.N.M.N.3.099 Mek		
	N.N.M.N.3.106 P38 MAPK		
	N.N.M.N.3.123 Raf	RAF proto-oncogene serine/threonine-protein kinase	Serine/threonine-protein kinase that acts as a regulatory link between the membrane-associated Ras GTPases and the MAPK/ERK cascade,
	N.N.M.N.3.124 RAF1		
	N.N.M.N.3.125 Ras		
	N.N.M.N.3.132 Shc		
	N.N.M.N.3.134 Sos		
	N.I.DP.N.13.026 KRAS	GTPase KRas	
**PI3K signaling pathway related proteins**	N.I.DP.N.13.036 PI3K (complex)	phosphatidylinositol-4,5-bisphosphate 3-kinase	Phosphoinositide-3-kinase (PI3K) that phosphorylates PtdIns (Phosphatidylinositol), PtdIns4P (Phosphatidylinositol 4-phosphate) and PtdIns(4,5)P2 (Phosphatidylinositol 4,5-bisphosphate) to generate phosphatidylinositol 3,4,5-trisphosphate (PIP3). PIP3 plays a key role by recruiting PH domain-containing proteins to the membrane, including AKT1 and PDPK1, activating signaling cascades involved in cell growth, survival, proliferation, motility and morphology.
	N.N.DG.N.1.086 PI3K (complex)	phosphatidylinositol-4,5-bisphosphate 3-kinase	
	N.N.DP.N.2.052 PI3K (complex)	phosphatidylinositol-4,5-bisphosphate 3-kinase	
	C.N.P.N.11.032 PI3K (complex)	phosphatidylinositol-4,5-bisphosphate 3-kinase	
	N.I.DG.N.12.092 PI3K (complex)	phosphatidylinositol-4,5-bisphosphate 3-kinase	
	N.I.DG.N.12.093 PI3K (family)	phosphatidylinositol-4,5-bisphosphate 3-kinase	

N.N.DG.N.1.004	Akt (PKB)	RAC-beta serine/threonine-protein kinase	AKT2 is one of 3 closely related serine/threonine-protein kinases (AKT1, AKT2 and AKT3) called the AKT kinase, and which regulate many processes including metabolism, proliferation, cell survival, growth and angiogenesis. This is mediated through serine and/or threonine phosphorylation of a range of downstream substrates.
C.N.P.N.11.004	Akt	RAC-beta serine/threonine-protein	
N.I.DG.N.12.004	Akt	RAC-beta serine/threonine-protein	
N.N.DP.N.2.003	Akt	RAC-beta serine/threonine-protein	
C.N.M.N.9.008	Akt	RAC-beta serine/threonine-protein	
N.I.DP.N.13.001	Akt	RAC-beta serine/threonine-protein	
C.N.P.N.11.029	p85 (pik3r) /PIK3R2	Phosphatidylinositol 3-kinase regulatory subunit beta	Regulatory subunit of phosphoinositide-3-kinase (PI3K), a kinase that phosphorylates PtdIns(4,5)P2 (Phosphatidylinositol 4,5-bisphosphate) to generate phosphatidylinositol 3,4,5-trisphosphate (PIP3). PIP3 plays a key role by recruiting PH domain-containing proteins to the membrane, including AKT1 and PDPK1, activating signaling cascades involved in cell growth, survival, proliferation, motility and morphology.
N.N.DP.N.2.049	p85 (pik3r)	Phosphatidylinositol 3-kinase regulatory subunit	
N.I.DG.N.12.088	p85 (pik3r)	Phosphatidylinositol 3-kinase regulatory subunit	
N.N.M.N.3.007	Akt		
N.N.M.N.3.108	p85(pik3r)		
N.N.M.N.3.114	PI3K (complex)		

	N.N.M.N.3.085	KITLG	Kit ligand	Ligand for the receptor-type protein-tyrosine kinase KIT. Plays an essential role in the regulation of cell survival and proliferation, hematopoiesis, stem cell maintenance, gametogenesis, mast cell development, migration and function, and in melanogenesis. KITLG/SCF binding can activate several signaling pathways. Promotes phosphorylation of PIK3R1, the regulatory subunit of phosphatidylinositol 3-kinase, and subsequent activation of the kinase AKT1. KITLG/SCF and KIT also transmit signals via GRB2 and activation of RAS, RAF1 and the MAP kinases MAPK1/ERK2 and/or MAPK3/ERK1. KITLG/SCF and KIT promote activation of STAT family members STAT1, STAT3 and STAT5. KITLG/SCF and KIT promote activation of PLCG1, leading to the production of the cellular signaling molecules diacylglycerol and inositol 1,4,5-trisphosphate. KITLG/SCF acts synergistically with other cytokines, probably interleukins.
**Other protein kinase**	N.N.DP.N.2.053	Pka	protein kinase A	
	C.N.P.N.11.033	Pka	protein kinase A	
	N.I.DG.N.12.094	Pka	protein kinase A	
	N.I.DP.N.13.037	Pka	protein kinase A	
	N.N.DG.N.1.087	Pkc(s)	Protein kinase C	Calcium-activated, phospholipid- and diacylglycerol (DAG)-dependent serine/threonine-protein kinase involved in various cellular processes such as regulation of the B-cell receptor (BCR) signalosome, oxidative stress-induced apoptosis, androgen receptor-dependent transcription regulation, insulin signaling and endothelial cells proliferation.
	N.N.DP.N.2.054	Pkc(s)	Protein kinase C	
	C.N.P.N.11.034	Pkc(s)	Protein kinase C	
	N.I.DG.N.12.095	Pkc(s)	Protein kinase C	
	N.I.DP.N.13.038	Pkc(s)	Protein kinase C	

N.I.DG.N.12.096	Pkg	protein kinase	PRKG isoforms act as key mediators of the nitric oxide/cGMP signaling pathway and are important components of many signal transduction processes in diverse cell types.
N.N.DP.N.2.019	Focal adhesion kinase	Focal adhesion kinase	
N.I.DP.N.13.017	Focal adhesion kinase	Focal adhesion kinase	
N.I.DG.N.12.045	Focal adhesion kinase	Focal adhesion kinase	
N.N.DP.N.2.021	FYN	Tyrosine-protein kinase Fyn	It plays a role in many biological processes including regulation of cell growth and survival, cell adhesion, integrin-mediated signaling, cytoskeletal remodeling, cell motility, immune response and axon guidance.
N.N.DG.N.1.083	p70 S6k	Ribosomal protein S6 kinase beta-1	Serine/threonine-protein kinase that acts downstream of mTOR signaling in response to growth factors and nutrients to promote cell proliferation, cell growth and cell cycle progression. Regulates protein synthesis through phosphorylation of EIF4B, RPS6 and EEF2K, and contributes to cell survival by repressing the pro-apoptotic function of BAD.
C.N.M.N.9.170	p70 S6k	Ribosomal protein S6 kinase beta-1	
N.I.DG.N.12.087	p70 S6k	Ribosomal protein S6 kinase beta-1	
N.I.DG.N.12.091	PDPK1	3-phosphoinositide-dependent protein kinase 1	Serine/threonine kinase which acts as a master kinase, phosphorylating and activating a subgroup of the AGC family of protein kinases.Plays an important role during thymocyte development by regulating the expression of key nutrient receptors on the surface of pre-T cells and mediating Notch-induced cell growth and proliferative responses. Provides negative feedback inhibition to toll-like receptor-mediated NF-kappa-B activation in macrophages.
C.N.P.N.11.031	PDPK1	3-phosphoinositide-dependent protein kinase	

N.N.DG.N.1.109	thymidine kinase （T	thymidine kinase	In latent infection, may allow the virus to be reactivated and to grow in cells lacking a high concentration of phosphorylated nucleic acid precursors.
C.N.M.N.9.250	tyrosine kinase	tyrosine kinase	
C.N.P.N.11.006	Ck2	Casein kinase II	
N.I.DG.N.12.026	Ck2	Casein kinase II	
N.N.DG.N.1.094	PRKAA	5'-AMP-activated protein kinase catalytic subunit alpha-2	Catalytic subunit of AMP-activated protein kinase (AMPK), an energy sensor protein kinase that plays a key role in regulating cellular energy metabolism.
N.I.DG.N.12.101	PRKACA	cAMP-dependent protein kinase catalytic subunit alpha	Phosphorylates a large number of substrates in the cytoplasm and the nucleus. Regulates the abundance of compartmentalized pools of its regulatory subunits through phosphorylation of PJA2 which binds and ubiquitinates these subunits, leading to their subsequent proteolysis.
N.I.DG.N.12.102	PRKAR1A	cAMP-dependent protein kinase type I-alpha regulatory subunit	Regulatory subunit of the cAMP-dependent protein kinases involved in cAMP signaling in cells.
C.N.P.N.11.038	PRKAR2A	cAMP-dependent protein kinase type II-alpha regulatory subunit	Regulatory subunit of the cAMP-dependent protein kinases involved in cAMP signaling in cells. Type II regulatory chains mediate membrane association by binding to anchoring proteins, including the MAP2 kinase.
C.N.M.N.9.150	MTOR	Serine/threonine-protein kinase mTOR	Serine/threonine protein kinase which is a central regulator of cellular metabolism, growth and survival in response to hormones, growth factors, nutrients, energy and stress signals. MTOR directly or indirectly regulates the phosphorylation of at least 800 proteins.
N.N.M.N.3.033	Ck2		
N.N.M.N.3.054	Focal adhesion kinase		
N.N.M.N.3.056	FYN		

	N.N.M.N.3.077	IKK (complex)			
	N.N.M.N.3.107	p70 S6k			
	N.N.M.N.3.115	Pka			
	N.N.M.N.3.116	Pkc(s)			
	N.N.N.N.4.003	Pkc(s),			
	N.N.DG.N.1.117	Pkg			
**Ubiquitin protein and protein degradation pathway related proteins**	N.N.DP.N.2.065	UBC		ubiquitin C	It is a polyubiquitin precursor.Ubiquitination has been associated with protein degradation, DNA repair, cell cycle regulation, kinase modification, endocytosis, and regulation of other cell signaling pathways.
	N.N.DG.N.1.111	UBC	D	ubiquitin C	
	N.N.DG.N.1.112	UBC	D	ubiquitin C	
	N.I.DG.N.12.128	UBC	D	ubiquitin C	
	N.I.DG.N.12.129	UBC	D	ubiquitin C	
	C.N.P.N.11.054	UBC		ubiquitin C	
	N.I.DP.N.13.045	UBC		ubiquitin C	
	N.N.DP.N.2.066	Ubiquitin		Ubiquitin	
	N.I.DG.N.12.130	Ubiquitin		Ubiquitin	
	N.N.DG.N.1.038	FBXO6		F-box only protein 6	Substrate-recognition component of some SCF (SKP1-CUL1-F-box protein)-type E3 ubiquitin ligase complexes.
	C.N.M.N.9.158	NEDD8		Neural precursor cell expressed developmentally down-regulated protein 8	Ubiquitin-like protein which plays an important role in cell cycle control and embryogenesis.
	C.N.M.N.9.169	OTUB1		Ubiquitin thioesterase OTUB1	Hydrolase that can specifically remove 'Lys-48'-linked conjugated ubiquitin from proteins and plays an important regulatory role at the level of protein turnover by preventing degradation.

C.N.P.N.11.055	UBQLN1	Ubiquilin-1	Plays an important role in the regulation of different protein degradation mechanisms and pathways including ubiquitin-proteasome system (UPS), autophagy and endoplasmic reticulum-associated protein degradation (ERAD) pathway. Mediates the proteasomal targeting of misfolded or accumulated proteins for degradation by binding (via UBA domain) to their polyubiquitin chains and by interacting (via ubiquitin-like domain) with the subunits of the proteasome
C.N.P.N.11.056	UCHL5	Ubiquitin carboxyl-terminal hydrolase isozyme L5	Protease that specifically cleaves 'Lys-48'-linked polyubiquitin chains. Deubiquitinating enzyme associated with the 19S regulatory subunit of the 26S proteasome.
N.I.DG.N.12.017	CAND1	Cullin-associated NEDD8-dissociated protein 1	Key assembly factor of SCF (SKP1-CUL1-F-box protein) E3 ubiquitin ligase complexes that promotes the exchange of the substrate-recognition F-box subunit in SCF complexes
C.N.P.N.11.008	COPS6	COP9 signalosome complex subunit 6	Component of the COP9 signalosome complex (CSN), a complex involved in various cellular and developmental processes. The CSN complex is an essential regulator of the ubiquitin (Ubl) conjugation pathway
C.N.M.N.9.047	CUL3	Cullin-3	Core component of multiple cullin-RING-based BCR (BTB-CUL3-RBX1) E3 ubiquitin-protein ligase complexes which mediate the ubiquitination and subsequent proteasomal degradation of target proteins.
C.N.M.N.9.048	CUL4B	Cullin-4B	Ubiquitin-dependent protein catabolic process, positive regulation of G1/S transition of mitotic cell cycle

C.N.M.N.9.132	ITCH	E3 ubiquitin-protein ligase Itchy homolog	Acts as an E3 ubiquitin-protein ligase which accepts ubiquitin from an E2 ubiquitin-conjugating enzyme in the form of a thioester and then directly transfers the ubiquitin to targeted substrates.
N.I.DG.N.12.079	MDM2	E3 ubiquitin-protein ligase Mdm2	E3 ubiquitin-protein ligase that mediates ubiquitination of p53/TP53, leading to its degradation by the proteasome. Inhibits p53/TP53- and p73/TP73-mediated cell cycle arrest and apoptosis by binding its transcriptional activation domain.
C.N.M.N.9.199	RAD23B	UV excision repair protein RAD23 homolog B	Multiubiquitin chain receptor involved in modulation of proteasomal degradation. Binds to polyubiquitin chains.
C.N.M.N.9.200	RANBP2	E3 SUMO-protein ligase RanBP2	E3 SUMO-protein ligase which facilitates SUMO1 and SUMO2 conjugation by UBE2I.Component of the nuclear
N.I.DG.N.12.001	26s Proteasome	26s Proteasome	
C.N.M.N.9.193	PSMA3	Proteasome subunit alpha type-3	The proteasome is a multicatalytic proteinase complex which is characterized by its ability to cleave peptides with Arg, Phe, Tyr, Leu, and Glu adjacent to the leaving group at neutral or slightly basic pH.
C.N.P.N.11.040	PSMA3	Proteasome subunit alpha type-3	
C.N.M.N.9.194	PSMA7	Proteasome subunit alpha type-7	Threonine-type endopeptidase activity
C.N.M.N.9.195	PSMD4	26S proteasome non-ATPase regulatory subunit 4	Binds and presumably selects ubiquitin-conjugates for destruction. Displays selectivity for longer polyubiquitin chains. Modulates intestinal fluid secretion.
N.N.M.N.3.001	20s proteasome		
N.N.M.N.3.002	26s Proteasome		
N.N.M.N.3.038	CUL5		
N.N.M.N.3.095	LNX1	E3 ubiquitin-protein ligase LNX	

	N.N.N.N.4.004	PSMA2,		Proteasome subunit alpha type-2	
	N.N.M.N.3.156	UBC	D		
	N.N.M.N.3.157	UBC	D		
	N.N.N.N.4.006	UBC,			
	N.N.M.N.3.158	Ubiquitin			
**14-3-3 and related proteins**	N.N.DP.N.2.001	14-3-3		tyrosine 3-monooxygenase/tryptophan 5-monooxygenase activation protein	Adapter protein implicated in the regulation of a large spectrum of both general and specialized signaling pathways. Binds to a large number of partners, usually by recognition of a phosphoserine or phosphothreonine motif.it may play a role in linking mitogenic signaling and the cell cycle machinery.
	C.N.M.N.9.001	14-3-3		tyrosine 3-monooxygenase/tryptophan 5-monooxygenase activation protein	
	C.N.M.N.9.258	YWHAB		14-3-3 protein beta/alpha	
	N.I.DG.N.12.133	YWHAG		14-3-3 protein gamma	
	C.N.M.N.9.259	YWHAH		14-3-3 protein eta	
	C.N.M.N.9.260	YWHAQ		14-3-3 protein theta	
	N.N.DP.N.2.069	YWHAQ		14-3-3 protein theta	
	N.N.M.N.3.162	YWHAB			
	N.N.M.N.3.163	YWHAQ			
**Cell cycle related proteins**	N.N.DG.N.1.016	Cdc2		Cyclin-dependent kinase 1	Plays a key role in the control of the eukaryotic cell cycle by modulating the centrosome cycle as well as mitotic onset; promotes G2-M transition, and regulates G1 progress and G1-S transition via association with multiple interphase cyclins.
	C.N.M.N.9.037	CDH1		Fizzy-related protein homolog (CDC20-like protein 1)	Key regulator of ligase activity of the anaphase promoting complex/cyclosome (APC/C), which confers substrate specificity upon the complex.

	N.I.DG.N.12.022	CDH1	Fizzy-related protein homolog (CDC20-like protein 1)	
	N.I.DG.N.12.023	CDKN1A	Cyclin-dependent kinase inhibitor 1	May be the important intermediate by which p53/TP53 mediates its role as an inhibitor of cellular proliferation in response to DNA damage. Binds to and inhibits cyclin-dependent kinase activity, preventing phosphorylation of critical cyclin-dependent kinase substrates and blocking cell cycle progression.
	N.N.DG.N.1.026	Cyclin A	Cyclin-A (Cyclin-A2)	Essential for the control of the cell cycle at the G1/S (start) and the G2/M (mitosis) transitions.
	N.I.DG.N.12.031	Cyclin A	Cyclin-A (Cyclin-A2)	
	C.N.M.N.9.049	Cyclin B	Cyclin B	
	C.N.M.N.9.027	CCNB1	G2/mitotic-specific cyclin-B1	Essential for the control of the cell cycle at the G2/M (mitosis) transition.
	N.I.DP.N.13.007	CCND1	G1/S-specific cyclin-D1	Cyclin-dependent protein serine/threonine kinase regulator
	N.I.DG.N.12.020	CCND1	G1/S-specific cyclin-D1	
	N.N.M.N.3.039	Cyclin A		
	N.N.M.N.3.040	Cyclin D		
	N.N.M.N.3.041	Cyclin E		
	C.N.M.N.9.275	Cyclin A		
**Mitochondrial electron transport related proteins**	N.N.DP.N.2.011	COX4I1	Cytochrome c oxidase subunit 4 isoform 1,	Cytochrome-c oxidase activity
	C.N.M.N.9.041	COX4I1	Cytochrome c oxidase subunit 4 isoform 1, mitochondrial	
	N.N.DP.N.2.012	COX6C	Cytochrome c oxidase subunit 6C	This protein is one of the nuclear-coded polypeptide chains of cytochrome c oxidase, the terminal oxidase in mitochondrial electron transport
	C.N.M.N.9.042	COX7A2	Cytochrome c oxidase subunit 7A2,	Cytochrome-c oxidase activity

C.N.M.N.9.044	COX7B	Cytochrome c oxidase subunit 7B, mitochondrial	
C.N.M.N.9.050	Cytochrome bc1 (UQ	Cytochrome b-c1 complex subunit Rieske,	
N.N.DP.N.2.014	cytochrome C	cytochrome C	
N.I.DG.N.12.032	cytochrome C	cytochrome C	
N.N.DG.N.1.028	cytochrome C	cytochrome C	
C.N.M.N.9.051	cytochrome C	cytochrome C	
N.I.DG.N.12.033	cytochrome-c oxidase	cytochrome-c oxidase	Cytochrome c oxidase (COX), the terminal enzyme of the mitochondrial respiratory chain, catalyzes the electron transfer from reduced cytochrome c to oxygen.
C.N.M.N.9.052	cytochrome-c oxidase	cytochrome-c oxidase	
C.N.M.N.9.149	MT-CO2	Cytochrome c oxidase subunit 2	Cytochrome c oxidase is the component of the respiratory chain that catalyzes the reduction of oxygen to water.
N.N.DG.N.1.091	POR	NADPH--cytochrome P450 reductase	This enzyme is required for electron transfer from NADP to cytochrome P450 in microsomes. It can also provide electron transfer to heme oxygenase and cytochrome B5.
N.N.M.N.3.042	cytochrome C		
C.N.M.N.9.148	Mitochondrial comple	Mitochondrial complex 1	
C.N.M.N.9.152	NADH dehydrogenas	NADH dehydrogenase	
C.N.M.N.9.155	NDUFAF1	Complex I intermediate-associated protein 30, mitochondrial(NADH dehydrogenase [ubiquinone] 1 alpha subcomplex assembly factor 1)	Chaperone protein involved in the assembly of the mitochondrial NADH:ubiquinone oxidoreductase complex (complex I).
C.N.M.N.9.156	NDUFAF4	NADH dehydrogenase [ubiquinone] 1 alpha subcomplex assembly factor 4	Involved in the assembly of mitochondrial NADH:ubiquinone oxidoreductase complex (complex I). May be involved in cell proliferation and survival of hormone-dependent tumor cells. May be a regulator of breast tumor cell invasion.

	C.N.M.N.9.157	NDUFS7		NADH dehydrogenase [ubiquinone] iron-sulfur protein 7, mitochondrial	Core subunit of the mitochondrial membrane respiratory chain NADH dehydrogenase (Complex I) that is believed to belong to the minimal assembly required for catalysis.
	C.N.M.N.9.003	ACADM		acyl-CoA dehydrogenase, mitochondrial	
	C.N.M.N.9.271	NDUFA9		NADH dehydrogenase [ubiquinone] 1 alpha subcomplex subunit 9, mitochondrial	Accessory subunit of the mitochondrial membrane respiratory chain NADH dehydrogenase (Complex I), that is believed not to be involved in catalysis. Complex I functions in the transfer of electrons from NADH to the respiratory chain. The immediate electron acceptor for the enzyme is believed to be ubiquinone.
	C.N.M.N.9.270	NDUFS3,		NADH dehydrogenase [ubiquinone] iron-sulfur protein 3, mitochondrial	Core subunit of the mitochondrial membrane respiratory chain NADH dehydrogenase (Complex I) that is believed to belong to the minimal assembly required for catalysis. Complex I functions in the transfer of electrons from NADH to the respiratory chain. The immediate electron acceptor for the enzyme is believed to be ubiquinone (By similarity).
	C.N.M.N.9.091	ETFB		Electron transfer flavoprotein subunit beta	
**Heat shock protein and molecular chaperone**	N.I.DG.N.12.056	HSP		Heat Shock Protein	
	N.N.DP.N.2.028	HSP		Heat Shock Protein	
	N.N.DP.N.2.029	Hsp27	D	Heat shock protein beta-1	Involved in stress resistance and actin organization.
	N.I.DG.N.12.057	Hsp70		heat shock 70kDa protein 4	ATP binding, chaperone-mediated protein complex assembly, protein import into mitochondrial outer membrane, response to unfolded protein
	C.N.P.N.11.019	Hsp70		heat shock 70kDa protein	

N.I.DG.N.12.058	Hsp90 (HSP90AA1)	heat shock protein 90kDa alpha	Molecular chaperone that promotes the maturation, structural maintenance and proper regulation of specific target proteins involved for instance in cell cycle control and signal transduction. Undergoes a functional cycle that is linked to its ATPase activity. This cycle probably induces conformational changes in the client proteins, thereby causing their activation.
N.N.DP.N.2.030	Hsp90	heat shock protein 90kDa alpha	
C.N.P.N.11.020	Hsp90	heat shock protein 90kDa alpha	
C.N.P.N.11.021	HSP90AA1	heat shock protein 90kDa alpha	
N.I.DG.N.12.059	HSP90AA1	heat shock protein 90kDa alpha	
C.N.M.N.9.122	HSP90AB1	Heat shock protein HSP 90-beta	Molecular chaperone that promotes the maturation, structural maintenance and proper regulation of specific target proteins involved for instance in cell cycle control and signal transduction. Undergoes a functional cycle that is linked to its ATPase activity.
N.I.DG.N.12.060	HSP90AB1	Heat shock protein HSP 90-beta	
N.N.DP.N.2.031	HSP90B1	Endoplasmin(Heat shock protein 90 kDa beta member 1)	Molecular chaperone that functions in the processing and transport of secreted proteins. When associated with CNPY3, required for proper folding of Toll-like receptors (By similarity). Functions in endoplasmic reticulum associated degradation (ERAD). Has ATPase activity.

N.I.DP.N.13.020	HSPA5		78 kDa glucose-regulated protein	Probably plays a role in facilitating the assembly of multimeric protein complexes inside the endoplasmic reticulum. Involved in the correct folding of proteins and degradation of misfolded proteins via its interaction with DNAJC10, probably to facilitate the release of DNAJC10 from its substrate.
N.N.DP.N.2.032	HSPB1	D	Heat shock protein beta-1	Involved in stress resistance and actin organization.
N.N.DP.N.2.033	HSPB8		Heat shock protein beta-8	Displays temperature-dependent chaperone activity.
C.N.M.N.9.123	HSPD1		60 kDa heat shock protein, mitochondrial	Implicated in mitochondrial protein import and macromolecular assembly.
C.N.M.N.9.124	HSPE1		10 kDa heat shock protein, mitochondrial	Eukaryotic CPN10 homolog which is essential for mitochondrial protein biogenesis, together with CPN60. Binds to CPN60 in the presence of Mg-ATP and suppresses the ATPase activity of the latter.
N.N.DG.N.1.082	P4HB		Protein disulfide-isomerase	This multifunctional protein catalyzes the formation, breakage and rearrangement of disulfide bonds.May be involved with other chaperones in the structural modification of the TG precursor in hormone biogenesis.
C.N.M.N.9.176	PDCL		Phosducin-like protein	Functions as a co-chaperone for CCT in the assembly of heterotrimeric G protein complexes, facilitates the assembly of both Gbeta-Ggamma and RGS-Gbeta5 heterodimers.
C.N.M.N.9.240	TCP1		T-complex protein 1 subunit alpha	Molecular chaperone; assists the folding of proteins upon ATP hydrolysis.
C.N.M.N.9.028	CCT2		T-complex protein 1 subunit beta	Molecular chaperone; assists the folding of proteins upon ATP hydrolysis. As part of the BBS/CCT complex may play a role in the assembly of BBSome, a complex involved in ciliogenesis regulating transports vesicles to the cilia. Known to play a role, in vitro, in the folding of actin and tubulin.

	C.N.M.N.9.029	CCT3		T-complex protein 1 subunit gamma	
	C.N.M.N.9.030	CCT4		T-complex protein 1 subunit delta	
	C.N.M.N.9.031	CCT5		T-complex protein 1 subunit epsilon	
	C.N.M.N.9.032	CCT6A		T-complex protein 1 subunit zeta/Chaperonin	
	C.N.M.N.9.033	CCT7		T-complex protein 1 subunit eta	
	C.N.M.N.9.034	CCT8		T-complex protein 1 subunit theta	
	N.N.M.N.3.030	CCT3			
	N.N.M.N.3.068	Hsp27	D		
	N.N.M.N.3.069	Hsp70			
	N.N.M.N.3.070	Hsp90			
	N.N.M.N.3.071	HSPA5			
	N.N.M.N.3.072	HSPA8			
	N.N.M.N.3.073	HSPB1	D		
	N.N.M.N.3.074	HSPD1			
	N.N.M.N.3.067	HSF1		Heat shock factor protein	
**GF, GFR and Related Proteins**	N.N.DG.N.1.113	Vegf		Vascular endothelial growth factor A	Growth factor active in angiogenesis, vasculogenesis and endothelial cell growth. Induces endothelial cell proliferation, promotes cell migration, inhibits apoptosis and induces permeabilization of blood vessels.
	C.N.N.N.10.009	Vegf		Vascular endothelial growth factor A	
	N.I.DG.N.12.131	Vegf		Vascular endothelial growth factor A	
	N.N.DP.N.2.067	Vegf		Vascular endothelial growth factor A	

N.N.DP.N.2.018	FLT4	Vascular endothelial growth factor receptor 3	Tyrosine-protein kinase that acts as a cell-surface receptor for VEGFC and VEGFD, and plays an essential role in adult lymphangiogenesis and in the development of the vascular network and the cardiovascular system during embryonic development. Promotes proliferation, survival and migration of endothelial cells, and regulates angiogenic sprouting.
N.N.DG.N.1.084	PDGF BB	Platelet-derived growth factor subunit B	Growth factor that plays an essential role in the regulation of embryonic development, cell proliferation, cell migration, survival and chemotaxis. Potent mitogen for cells of mesenchymal origin. Required for normal proliferation and recruitment of pericytes and vascular smooth muscle cells in the central nervous system, skin, lung, heart and placenta. Required for normal blood vessel development, and for normal development of kidney glomeruli. Plays an important role in wound healing. Signaling is modulated by the formation of heterodimers with PDGFA (By similarity).
N.N.DP.N.2.050	PDGF BB	Platelet-derived growth factor subunit B	
N.I.DG.N.12.090	PDGF BB	Platelet-derived growth factor subunit B	
N.N.DP.N.2.051	Pdgfr	Platelet-derived growth factor receptor beta	Tyrosine-protein kinase that acts as cell-surface receptor for homodimeric PDGFB and PDGFD and for heterodimers formed by PDGFA and PDGFB, and plays an essential role in the regulation of embryonic development, cell proliferation, survival, differentiation, chemotaxis and migration. Plays an essential role in blood vessel development by promoting proliferation, migration and recruitment of pericytes and smooth muscle cells to endothelial cells.
N.N.DG.N.1.008	ANGPT1	Angiopoietin-1	
C.N.M.N.9.070	EGFR	Epidermal growth factor receptor	Transmembrane receptor protein tyrosine kinase activity,ATP binding

N.I.DG.N.12.036	EGFR	Epidermal growth factor receptor	
N.I.DP.N.13.013	EGFR	Epidermal growth factor receptor	
N.N.DG.N.1.039	Fgf	Fibroblast growth factor	
N.N.DG.N.1.040	FGF2	Fibroblast growth factor 2	Plays an important role in the regulation of cell survival, cell division, angiogenesis, cell differentiation and cell migration. Functions as potent mitogen in vitro.
N.N.DG.N.1.041	Fgfr	Fibroblast growth factor receptor	
N.N.DG.N.1.042	FGFR1	Fibroblast growth factor receptor 1	Tyrosine-protein kinase that acts as cell-surface receptor for fibroblast growth factors and plays an essential role in the regulation of embryonic development, cell proliferation, differentiation and migration.
C.N.M.N.9.094	FGFR1	Fibroblast growth factor receptor 1	
N.I.DG.N.12.043	FGFR1	Fibroblast growth factor receptor 1	
N.N.M.N.3.111	Pdgf (complex)		
N.N.M.N.3.112	PDGF BB		
N.N.M.N.3.160	Vegf		
N.N.DP.N.2.024	growth factor receptor	growth factor receptor	
N.N.DG.N.1.108	Tgf beta	Transforming growth factor beta-1	This gene encodes a member of the transforming growth factor beta (TGFB) family of cytokines, which are multifunctional peptides that regulate proliferation, differentiation, adhesion, migration, and other functions in many cell types. Many cells have TGFB receptors, and the protein positively and negatively regulates many other growth factors.
N.N.DP.N.2.063	Tgf beta	Transforming growth factor beta-1	

	N.I.DP.N.13.044	Tgf beta	Transforming growth factor beta-1	
	N.I.DG.N.12.123	TGFB1	Transforming growth factor beta-1	Multifunctional protein that controls proliferation, differentiation and other functions in many cell types. Many cells synthesize TGFB1 and have specific receptors for it. It positively and negatively regulates many other growth factors. It plays an important role in bone remodeling as it is a potent stimulator of osteoblastic bone formation, causing chemotaxis, proliferation and differentiation in committed osteoblasts.
	C.N.M.N.9.006	ACVRL1	Serine/threonine-protein kinase receptor R3	Type I receptor for TGF-beta family ligands
	N.N.M.N.3.138	Tgf beta		
	N.N.M.N.3.139	TGFB1		
	N.N.DG.N.1.119	PTN	Pleiotrophin	Secreted growth factor that induces neurite outgrowth and which is mitogenic for fibroblasts, epithelial, and endothelial cells. Binds anaplastic lymphoma kinase (ALK) which induces MAPK pathway activation, an important step in the anti-apoptotic signaling of PTN and regulation of cell proliferation
	N.I.DG.N.12.135	Tgf beta		
**Hormones and related proteins**	C.N.M.N.9.102	GH1	Somatotropin (Growth hormone 1)	Plays an important role in growth control. Its major role in stimulating body growth is to stimulate the liver and other tissues to secrete IGF-1. It stimulates both the differentiation and proliferation of myoblasts. It also stimulates amino acid uptake and protein synthesis in muscle and other tissues.
	N.N.DG.N.1.048	GH1	Somatotropin (Growth hormone 1)	
	N.N.DP.N.2.022	GH1	Somatotropin (Growth hormone 1)	
	C.N.P.N.11.015	GH1	Somatotropin (Growth hormone 1)	

N.I.DP.N.13.018	GH1	Somatotropin (Growth hormone 1)	
N.N.DG.N.1.050	Growth hormone	Growth hormone	
N.I.DG.N.12.049	Growth hormone	Growth hormone	
N.N.DP.N.2.025	Growth hormone	Growth hormone	
C.N.P.N.11.016	Growth hormone	Growth hormone	
N.N.DG.N.1.012	beta estradiol	beta estradiol	
N.I.DG.N.12.013	beta estradiol	beta estradiol	
N.N.DP.N.2.017	estrogen receptor	estrogen receptor	
N.I.DG.N.12.041	estrogen receptor	estrogen receptor	
N.N.DG.N.1.032	estrogen receptor	estrogen receptor	
C.N.M.N.9.090	estrogen receptor	estrogen receptor	
C.N.P.N.11.012	estrogen receptor	estrogen receptor	
N.I.DG.N.12.039	ESR1	Estrogen receptor	Nuclear hormone receptor. The steroid hormones and their receptors are involved in the regulation of eukaryotic gene expression and affect cellular proliferation and differentiation in target tissues.
N.I.DP.N.13.016	ESR1	Estrogen receptor	
N.I.DG.N.12.040	ESR2	Estrogen receptor beta	Nuclear hormone receptor. Binds estrogens with an affinity similar to that of ESR1, and activates expression of reporter genes containing estrogen response elements (ERE) in an estrogen-dependent manner.
C.N.M.N.9.097	FSH	Follitropin subunit	
N.N.DG.N.1.046	FSH	Follitropin subunit	
N.N.DP.N.2.020	FSH	Follitropin subunit	
N.I.DG.N.12.046	FSH	Follitropin subunit	
N.N.DP.N.2.057	Proinsulin	Proinsulin	
N.N.DG.N.1.096	Proinsulin	Proinsulin	
C.N.P.N.11.039	Proinsulin	Proinsulin	
N.N.DG.N.1.059	Insulin	Insulin	
N.I.DG.N.12.068	Insulin	Insulin	
N.N.DP.N.2.039	Insulin	Insulin	

C.N.M.N.9.130	Insulin	Insulin	
C.N.P.N.11.023	Insulin	Insulin	
N.I.DP.N.13.023	Insulin	Insulin	
C.N.P.N.11.025	Lh	Luteinizing Hormone	
N.N.DG.N.1.068	Lh	Luteinizing Hormone	
N.N.DP.N.2.043	Lh	Luteinizing Hormone	
C.N.M.N.9.138	Lh	Luteinizing Hormone	
N.I.DG.N.12.076	Lh	Luteinizing Hormone	
N.I.DP.N.13.027	Lh	Luteinizing Hormone	
N.N.DG.N.1.095	PRL	Prolactin	Prolactin acts primarily on the mammary gland by promoting lactation.
N.N.DP.N.2.056	PRL	Prolactin	
C.N.M.N.9.192	PRL	Prolactin	
N.I.DG.N.12.103	progesterone	progesterone	
N.N.DG.N.1.057	IGFBP3	Insulin-like growth factor-binding protein 3	IGF-binding proteins prolong the half-life of the IGFs and have been shown to either inhibit or stimulate the growth promoting effects of the IGFs on cell culture. They alter the interaction of IGFs with their cell surface receptors. Also exhibits IGF-independent antiproliferative and apoptotic effects mediated by its receptor TMEM219/IGFBP-3R.
N.I.DG.N.12.062	IGFBP5	Insulin-like growth factor-binding protein 5	IGF-binding proteins prolong the half-life of the IGFs and have been shown to either inhibit or stimulate the growth promoting effects of the IGFs on cell culture. They alter the interaction of IGFs with their cell surface receptors.
N.N.DG.N.1.003	ADM	adrenomedullin	
N.I.DG.N.12.003	ADRB	adrenergic receptor	
N.N.DG.N.1.019	CGA	Glycoprotein hormones alpha chain	Hormone activity
C.N.M.N.9.039	CGA	Glycoprotein hormones alpha chain	

	C.N.P.N.11.014	GAL	Galanin peptides	Contracts smooth muscle of the gastrointestinal and genitourinary tract, regulates growth hormone release, modulates insulin release, and may be involved in the control of adrenal secretion.
	N.N.DG.N.1.049	GRB2	Growth factor receptor-bound protein 2	Adapter protein which acts downstream of several membrane receptors including cytokine, antigen, hormone, cell matrix and growth factor receptors to regulate multiple signaling pathways.
	C.N.M.N.9.186	POMC	Pro-opiomelanocortin	ACTH stimulates the adrenal glands to release cortisol.hormone activity
	C.N.P.N.11.036	POMC	Pro-opiomelanocortin	
	N.N.DG.N.1.090	POMC	Pro-opiomelanocortin	
	N.N.DG.N.1.027	CYP11A1	Cholesterol side-chain cleavage enzyme, mitochondrial	Catalyzes the side-chain cleavage reaction of cholesterol to pregnenolone.
	C.N.M.N.9.180	PGRMC1	Membrane-associated progesterone receptor component 1	Heme binding, steroid binding
	N.N.M.N.3.006	ADRB		
	N.N.M.N.3.019	beta estradiol		
	N.N.M.N.3.050	estrogen receptor		
	N.N.M.N.3.060	GH1		
	N.N.M.N.3.063	Growth hormone		
	N.N.M.N.3.081	Insulin		
	N.N.M.N.3.092	Lh		
	N.N.M.N.3.122	Proinsulin		
	N.N.M.N.3.143	trypsin		
	N.N.M.N.3.144	TSH		
	N.I.DP.N.13.010	EDNRA	Endothelin-1 receptor	
	N.I.DP.N.13.017	GAST	Gastrin	
**Glycolytic related enzymes**	C.N.M.N.9.177	PFKL	ATP-dependent 6-phosphofructokinase, liver type	Catalyzes the phosphorylation of D-fructose 6-phosphate to fructose 1,6-bisphosphate by ATP, the first committing step of glycolysis.

	C.N.M.N.9.178	PFKP	ATP-dependent 6-phosphofructokinase, platelet type	
	C.N.M.N.9.179	PGK1	Phosphoglycerate kinase 1	In addition to its role as a glycolytic enzyme, it seems that PGK-1 acts as a polymerase alpha cofactor protein
	C.N.M.N.9.182	PKM	Pyruvate kinase PKM	Glycolytic enzyme that catalyzes the transfer of a phosphoryl group from phosphoenolpyruvate (PEP) to ADP, generating ATP.
	C.N.M.N.9.012	ALDOA	Fructose-bisphosphate aldolase A	Plays a key role in glycolysis and gluconeogenesis. In addition, may also function as scaffolding protein (By similarity)
	C.N.M.N.9.089	ENO1	Alpha-enolase	Multifunctional enzyme that, as well as its role in glycolysis, plays a part in various processes such as growth control, hypoxia tolerance and allergic responses.
	C.N.M.N.9.099	GAPDH	Glyceraldehyde-3-phosphate dehydrogenase	participates in glycolysis
	C.N.M.N.9.136	LDHA	L-lactate dehydrogenase A chain	The key enzyme in the glycolysis
	C.N.M.N.9.137	LDHB	L-lactate dehydrogenase B chain	
	N.I.DP.N.13.027	Ldh	L-lactate dehydrogenase	
**Nuclear receptors**	N.N.DG.N.1.079	Nr1h	Oxysterols receptor LXR	Nuclear receptor.
	N.N.DG.N.1.080	NR4A1	Nuclear receptor subfamily 4 group A member 1	Orphan nuclear receptor.Participates in energy homeostasis by sequestrating the kinase STK11 in the nucleus, thereby attenuating cytoplasmic AMPK activation.
	N.N.DG.N.1.081	NR4A2	Nuclear receptor subfamily 4 group A member 2	Transcriptional regulator which is important for the differentiation and maintenance of meso-diencephalic dopaminergic (mdDA) neurons during development. It is crucial for expression of a set of genes such as SLC6A3, SLC18A2, TH and DRD2 which are essential for development of mdDA neurons

	C.N.M.N.9.187	PPARG	Peroxisome proliferator-activated receptor gamma	Nuclear receptor that binds peroxisome proliferators such as hypolipidemic drugs and fatty acids.Acts as a critical regulator of gut homeostasis by suppressing NF-kappa-B-mediated proinflammatory responses.
**Inflammation related proteins**	N.I.DP.N.13.040	Pro-inflammatory Cyt	Pro-inflammatory Cytokine	
	N.I.DG.N.12.065	IL1	interleukin 1	This cytokine is an important mediator of the inflammatory response, and is involved in a variety of cellular activities, including cell proliferation, differentiation, and apoptosis.
	C.N.P.N.11.022	IL1	interleukin 1	
	N.N.DP.N.2.037	IL12 (complex)	interleukin 12	A cytokine that acts on T and natural killer cells, and has a broad array of biological activities. This cytokine has been found to be important for sustaining a sufficient number of memory/effector Th1 cells to mediate long-term protection to an intracellular pathogen. Overexpression of this gene was observed in the central nervous system of patients with multiple sclerosis (MS), suggesting a role of this cytokine in the pathogenesis of the disease.
	N.I.DG.N.12.066	IL12 (family)	interleukin 12	
	N.N.DP.N.2.038	IL15	Interleukin-15	Cytokine that stimulates the proliferation of T-lymphocytes. Stimulation by IL-15 requires interaction of IL-15 with components of IL-2R, including IL-2R beta and probably IL-2R gamma but not IL-2R alpha.
	C.N.M.N.9.127	ILF3	Interleukin enhancer-binding factor 3	This gene encodes a double-stranded RNA (dsRNA) binding protein that complexes with other proteins, dsRNAs, small noncoding RNAs, and mRNAs to regulate gene expression and stabilize mRNAs.Can act as a translation inhibitory ,Can regulate protein arginine N-methyltransferase 1 activity. May regulate transcription of the IL2 gene during T-cell activation. Can promote the formation of stable DNA-dependent protein kinase holoenzyme complexes on DNA.

	C.N.N.N.10.007	TNF	Tumor necrosis factor	It is mainly secreted by macrophages and can induce cell death of certain tumor cell lines. It is potent pyrogen causing fever by direct action or by stimulation of interleukin-1 secretion and is implicated in the induction of cachexia, Under certain conditions it can stimulate cell proliferation and [illegible]
	N.I.DG.N.12.126	TNF	Tumor necrosis factor	
	N.I.DG.N.12.125	Tnf (family)	Tumor necrosis factor	
	N.I.DG.N.12.070	Interferon alpha	Interferon alpha	
	N.I.DG.N.12.061	Ifn gamma (IFNG)	Ifn gamma (IFNG)	
	N.N.M.N.3.032	chemokine		
	N.N.M.N.3.076	IFNG		
	N.N.M.N.3.079	IL12 (complex)		
	N.N.M.N.3.083	Interferon alpha		
	N.N.N.N.4.002	Interferon alpha,		
	N.N.M.N.3.088	lfn		
	N.N.M.N.3.105	OSM	Oncostatin-M	Cytokine activity， growth factor activity， oncostatin-M receptor binding.Growth regulator. Inhibits the proliferation of a number of tumor cell lines. Stimulates proliferation of AIDS-KS cells. It regulates cytokine production, including IL-6, G-CSF and GM-CSF from endothelial cells.
	N.N.M.N.3.121	Pro-inflammatory Cytokine		
	N.N.M.N.3.140	Tnf (family)		
	N.N.N.N.4.005	TNF,		
	N.N.DG.N.1.025	CXCR4	C-X-C chemokine receptor type 4	Receptor for the C-X-C chemokine CXCL12/SDF-1 that transduces a signal by increasing intracellular calcium ion levels and enhancing MAPK1/MAPK3 activation. Acts as a receptor for extracellular ubiquitin; leading to enhanced intracellular calcium ions and reduced cellular cAMP levels.
	N.I.DP.N.13.021	IFNG	Interferon gamma	
**Immun related proteins**	N.N.DP.N.2.008	CD1	Antigen-presenting glycoprotein	

C.N.M.N.9.035	CD3	T-cell surface glycoprotein CD3	
N.I.DG.N.12.021	CD3	T-cell surface glycoprotein CD3	
C.N.M.N.9.036	CD81	CD81 antigen	May play an important role in the regulation of lymphoma cell growth.
N.I.DP.N.13.021	IgG	immunoglobulin G	
N.N.DP.N.2.036	IgG	immunoglobulin G	
N.I.DG.N.12.063	IgG1	immunoglobulin G	
N.I.DG.N.12.064	Igm	VH4 heavy chain variable region	The protein encoded by this gene is expressed on the surface of T cells. It regulates B cell function by engaging CD40 on the B cell surface. A defect in this gene results in an inability to undergo immunoglobulin class switch and is associated with hyper-IgM syndrome.
N.N.DG.N.1.058	Immunoglobulin	Immunoglobulin	Immunoglobulins recognize foreign antigens and initiate immune responses such as phagocytosis and the complement
N.I.DG.N.12.067	Immunoglobulin	Immunoglobulin	
N.I.DP.N.13.022	Immunoglobulin	Immunoglobulin	
N.N.DP.N.2.035	Ige	Ig epsilon chain C region	Antigen binding, Fc-epsilon receptor signaling pathway, immune response, innate immune response
N.N.DG.N.1.056	Ige （IGHE）	Ig epsilon chain C region	
N.N.DG.N.1.067	LGALS3	Galectin-3	Galactose-specific lectin which binds IgE. May mediate with the alpha-3, beta-1 integrin the stimulation by CSPG4 of endothelial cells migration.
N.I.DG.N.12.122	TCR	Tcell alpha chain	
N.N.DP.N.2.062	TCR	Tcell alpha chain	
C.N.M.N.9.241	TCR	Tcell alpha chain	
N.N.DG.N.1.011	BCR (complex)	B-cell antigen receptor complex	
N.N.M.N.3.018	BCR (complex)		
N.N.M.N.3.031	CD3		
N.N.M.N.3.080	Immunoglobulin		

	N.N.M.N.3.089	lge		
	N.N.M.N.3.090	lgG		
	N.N.M.N.3.091	lgm		
	N.N.M.N.3.137	TCR		
	N.N.M.N.3.021	C1q	Complement	C1q associates with the proenzymes C1r and C1s to yield C1, the first component of the serum complement system. The collagen-like regions of C1q interact with the Ca2+-dependent C1r2C1s2 proenzyme complex, and efficient activation of C1 takes place on interaction of the globular heads of C1q with the Fc regions of IgG or IgM antibody present in immune [illegible]
	N.N.M.N.3.022	C3	Complement C3	C3 plays a central role in the activation of the complement system. Its processing by C3 convertase is the central reaction in both classical and alternative complement pathways. After activation C3b can bind covalently, via its reactive thioester, to cell surface carbohydrates or immune aggregates.
	N.N.DG.N.1.118	BSG		Plays an important role in targeting the monocarboxylate transporters SLC16A1, SLC16A3 and SLC16A8 to the plasma membrane. Plays pivotal roles in spermatogenesis, embryo implantation, neural network formation and tumor progression. Stimulates adjacent fibroblasts to produce matrix metalloproteinases (MMPS). Seems to be a receptor for oligomannosidic glycans. In vitro, promotes outgrowth of [illegible]
	N.N.DP.N.2.070	Immunoglobulin		
	N.I.DP.N.13.015	Fc gamma receptor		Receptor for the Fc region of IgG. Binds complexed or aggregated IgG and also monomeric IgG. Mediates antibody-dependent cellular cytotoxicity (ADCC) and other antibody-dependent responses, such as phagocytosis.
**LDL, HDL and its related proteins**	N.N.DG.N.1.066	LDL	Low-density lipoprotein	LDL is normally bound at the cell membrane and taken into the cell ending up in lysosomes where the protein is degraded and the cholesterol is made available for repression of microsomal enzyme 3-hydroxy-3-methylglutaryl coenzyme A (HMG CoA) reductase, the rate-limiting step in cholesterol [illegible]

	N.N.DP.N.2.042	LDL	Low-density lipoprotein	
	N.I.DG.N.12.074	LDL	Low-density lipoprotein	
	N.I.DP.N.13.026	LDL	Low-density lipoprotein	
	C.N.P.N.11.017	HDL		
	N.N.DG.N.1.052	HDL		
	N.N.DG.N.1.106	SREBF1	Sterol regulatory element-	Transcriptional activator required for lipid homeostasis.
	N.N.DP.N.2.006	APOA1	Apolipoprotein A-I	
	N.N.M.N.3.017	APOA1		
	N.N.M.N.3.065	HDL		
	N.N.M.N.3.087	LDL		
**Apoptosis related proteins**	N.I.DP.N.13.005	CASP1	Caspase-1	Thiol protease that cleaves IL-1, releasing the mature cytokine which is involved in a variety of inflammatory processes. Can also promote apoptosis.
	N.N.DP.N.2.007	caspase	caspase	
	N.I.DG.N.12.018	caspase	caspase	
	N.N.DG.N.1.089	PMAIP1	Phorbol-12-myristate-13-acetate-induced protein 1	Promotes activation of caspases and apoptosis. Promotes mitochondrial membrane changes and efflux of apoptogenic proteins from the mitochondria.
	N.I.DG.N.12.097	PMAIP1	Phorbol-12-myristate-13-acetate-induced protein 1	
	C.N.M.N.9.237	STK3	Serine/threonine-protein kinase 3	Stress-activated, pro-apoptotic kinase which, following caspase-cleavage, enters the nucleus and induces chromatin condensation followed by internucleosomal DNA fragmentation. Key component of the Hippo signaling pathway which plays a pivotal role in organ size control and tumor suppression by restricting proliferation and promoting [illegible]
	N.I.DG.N.12.011	BAX	Apoptosis regulator BAX	
	N.I.DG.N.12.012	BBC3	Bcl-2-binding component 3	Essential mediator of p53/TP53-dependent and p53/TP53-independent apoptosis.
	N.N.DG.N.1.010	BCL2	Apoptosis regulator Bcl-2	

	N.I.DG.N.12.089	PARP	poly ADP-ribose polymerase	It is a chromatin-associated enzyme,which modifies various nuclear proteins. The modification is dependent on DNA and is involved in the regulation of various important cellular processes such as differentiation, proliferation, and tumor transformation and also in the regulation of the molecular events involved in the recovery of cell from DNA damage.
	C.N.M.N.9.173	PARP1	Poly [ADP-ribose] polymerase 1	Involved in the base excision repair (BER) pathway, by catalyzing the poly(ADP-ribosyl)ation of a limited number of acceptor proteins involved in chromatin architecture and in
	N.N.M.N.3.027	caspase		
	N.N.M.N.3.110	PARP		
**Oncogene proteins and proteins involved in tumorigenesis**	N.N.DG.N.1.063	JUN/JUNB/JUND	Transcription factor AP-1 / Transcription factor jun-B/ Transcription factor jun-D	Transcription factor that recognizes and binds to the enhancer heptamer motif 5'-TGA[CG]TCA-3'. Promotes activity of NR5A1 when phosphorylated by HIPK3 leading to increased steroidogenic gene expression upon cAMP signaling pathway
	N.N.DG.N.1.064	JUNB	Transcription factor jun-B	Transcription factor involved in regulating gene activity following the primary growth factor response. Binds to the DNA sequence 5'-TGA[CG]TCA-3'.
	C.N.M.N.9.133	JUNB	Transcription factor jun-B	
	N.N.DG.N.1.044	FOS	Proto-oncogene c-Fos	Nuclear phosphoprotein which forms a tight but non-covalently linked complex with the JUN/AP-1 transcription factor. In the heterodimer, FOS and JUN/AP-1 basic regions each seems to interact with symmetrical DNA half sites.
	N.I.DG.N.12.007	Ap1	Activator protein 1	Transcription factor that recognizes and binds to the enhancer heptamer motif 5'-TGA[CG]TCA-3'. Promotes activity of NR5A1 when phosphorylated by HIPK3 leading to increased steroidogenic gene expression upon cAMP signaling pathway [illegible]
	N.N.DG.N.1.009	Ap1	Activator protein 1	
	N.N.DP.N.2.005	AP1	Activator protein 1	
	C.N.M.N.9.019	Ap1	Activator protein 1	

	N.I.DG.N.12.114	SKI	Ski oncogene	May play a role in terminal differentiation of skeletal muscle cells but not in the determination of cells to the myogenic lineage. Functions as a repressor of TGF-beta signaling.
	N.I.DG.N.12.119	SRC (family)	Proto-oncogene tyrosine-protein kinase Src	Non-receptor protein tyrosine kinase which is activated following engagement of many different classes of cellular receptors including immune response receptors, integrins and other adhesion receptors, receptor protein tyrosine kinases, G protein-coupled receptors as well as cytokine receptors.
	N.N.DG.N.1.107	STAT5a/b	Signal transducer and activator of transcription 5A/B	In response to cytokines and growth factors, Activation of this protein has been shown to be essential for tumorigenesis.It has been shown to be involved in diverse biological processes, such as TCR signaling, apoptosis, adult mammary gland development, and sexual dimorphism of liver gene expression. This gene was found to fuse to retinoic acid receptor-alpha (RARA) gene in a small subset of acute promyelocytic [illegible]
	N.N.DP.N.2.061	STAT5a/b	Signal transducer and activator of transcription 5A/B	
	N.I.DG.N.12.117	SNAI2	Zinc finger protein SNAI2	Transcriptional repressor that modulates both activator-dependent and basal transcription.Plays a role in mediating RAF1-induced transcriptional repression of the TJ protein, occludin (OCLN) and subsequent oncogenic transformation of epithelial cells ,Plays an essential role in TWIST1-induced EMT and its ability to promote invasion and metastasis.
	N.N.M.N.3.015	Ap1		
	N.N.M.N.3.135	SRC (family)		
	N.N.M.N.3.136	STAT5a/b		
**Tumor suppressor**	C.N.M.N.9.246	TP53BP1	Tumor suppressor p53-binding protein 1	Plays a key role in the response to DNA damage. May have a role in checkpoint signaling during mitosis. Enhances TP53-mediated transcriptional activation.

	N.N.DG.N.1.110	TP53	Cellular tumor antigen p53	Acts as a tumor suppressor in many tumor types; induces growth arrest or apoptosis depending on the physiological circumstances and cell type. Involved in cell cycle regulation as a trans-activator that acts to negatively regulate cell division by controlling a set of genes required for this process.
	C.N.M.N.9.202	Rb	Retinoblastoma-associated protein	Key regulator of entry into cell division that acts as a tumor suppressor. Promotes G0-G1 transition when phosphorylated by CDK3/cyclin-C. Acts as a transcription repressor of E2F1 target genes.
	N.I.DG.N.12.108	Rb	Retinoblastoma-associated protein	
	N.N.M.N.3.126	RBL1	Retinoblastoma-like protein 1	Key regulator of entry into cell division. Directly involved in heterochromatin formation by maintaining overall chromatin structure and, in particular, that of constitutive heterochromatin by stabilizing histone methylation. Recruits and targets histone methyltransferases SUV420H1 and SUV420H2, leading to epigenetic transcriptional repression. Controls histone H4 'Lys-20' trimethylation. Probably acts as a transcription repressor by recruiting chromatin-modifying enzymes to promoters. Potent inhibitor of E2F-mediated trans-activation. Forms a complex with adenovirus E1A and with SV40 large T antigen. May bind and modulate functionally certain cellular proteins with which T and E1A compete for pocket binding. May act as a tumor suppressor.
**Notch signaling pathway related proteins**	N.N.DG.N.1.077	Notch	Neurogenic locus notch homolog protein	Functions as a receptor for membrane-bound ligands to regulate cell-fate determination. Upon ligand activation through the released notch intracellular domain (NICD) it forms a transcriptional activator complex with RBPJ/RBPSUH and activates genes of the enhancer of split locus. Affects the implementation of differentiation, proliferation and apoptotic programs.
	C.N.M.N.9.164	Notch	Neurogenic locus notch homolog protein	

	N.N.DG.N.1.078	NOTCH3	Neurogenic locus notch homolog protein 3	
	N.I.DG.N.12.010	ATXN1	Ataxin-1	Chromatin-binding factor that repress Notch signaling in the absence of Notch intracellular domain by acting as a CBF1
**Wnt signaling pathway related proteins**	N.I.DG.N.12.030	CTNNB1	Catenin beta-1	Key downstream component of the canonical Wnt signaling
	C.N.M.N.9.104	Gsk3	Glycogen synthase kinase-3	Constitutively active protein kinase that acts as a negative regulator in the hormonal control of glucose homeostasis, Wnt signaling and regulation of transcription factors and
	N.I.DG.N.12.050	Gsk3	Glycogen synthase kinase-	
	N.I.DG.N.12.075	LGR4	Leucine-rich repeat-containing G-protein coupled receptor 4	Receptor for R-spondins that potentiates the canonical Wnt signaling pathway and is involved in the formation of various organs.
**Phosphatases and phosphodiesterases**	N.N.DG.N.1.092	PP1 protein complex g	(PPA1) Inorganic pyrophosphatase	
	N.I.DG.N.12.098	PP1 protein complex g	(PPA1) Inorganic pyrophosphatase	
	N.N.DG.N.1.093	PP2A	(PPP2R4) Serine/threonine-protein phosphatase 2A activator	PPIases accelerate the folding of proteins. It catalyzes the cis-trans isomerization of proline imidic peptide bonds in oligopeptides.
	N.I.DG.N.12.099	PP2A	(PPP2R4) Serine/threonine-protein phosphatase 2A activator	
	N.I.DG.N.12.100	Ppp2c	protein phosphatase 2, catalytic subunit,	Protein phosphatase 2A is one of the four major Ser/Thr phosphatases, and it is implicated in the negative control of cell growth and division.
	C.N.M.N.9.188	PPP2R1A	Serine/threonine-protein phosphatase 2A 65 kDa regulatory subunit A alpha isoform	The PR65 subunit of protein phosphatase 2A serves as a scaffolding molecule to coordinate the assembly of the catalytic subunit and a variable regulatory B subunit.
	C.N.M.N.9.189	PPP2R4	Serine/threonine-protein phosphatase 2A activator	

	C.N.M.N.9.190	PPP4R1	Serine/threonine-protein phosphatase 4 regulatory subunit 1	Regulatory subunit of serine/threonine-protein phosphatase 4.
	N.N.M.N.3.120	PPP2R1A		
**Ca2+ related proteins**	N.I.DP.N.13.043	TRPC6	Short transient receptor potential channel 6	
	N.I.DG.N.12.015	Calmodulin	Calmodulin	
	N.I.DP.N.13.004	Calmodulin	Calmodulin	
	N.I.DG.N.12.014	CACNA1B	Voltage-dependent N-type calcium channel subunit alpha-1B	It is involved in a variety of calcium-dependent processes, including muscle contraction, hormone or neurotransmitter release, gene expression, cell motility, cell division and cell
	C.N.P.N.11.024	IQCB1	IQ calmodulin-binding motif-containing protein 1	Calmodulin binding, enzyme binding
	N.I.DP.N.13.042	S100A1	Protein S100-A1	Weakly binds calcium but binds zinc very tightly-distinct binding sites with different affinities exist for both ions on
	N.I.DG.N.12.115	SLC8A1	Sodium/calcium exchanger 1	Rapidly transports Ca2+ during excitation-contraction coupling. Ca2+ is extruded from the cell during relaxation so as to prevent overloading of intracellular stores.
	N.N.DG.N.1.015	calpain	calpain	
	N.I.DG.N.12.016	calpain	calpain	
	N.N.M.N.3.023	Calcineurin protein(s)		
	N.N.M.N.3.024	Calmodulin		
	N.N.M.N.3.025	calpain		
	N.N.M.N.3.026	CALR		
	N.N.M.N.3.167	voltage-gated calcium channel		
**Protein synthesis related proteins**	N.I.DG.N.12.105	RARS	Arginine--tRNA ligase, cytoplasmic	Forms part of a macromolecular complex that catalyzes the attachment of specific amino acids to cognate tRNAs during protein synthesis. Modulates the secretion of AIMP1 and may be involved in generation of the inflammatory cytokine [illegible]
	C.N.M.N.9.053	DARS	Aspartate--tRNA ligase, cytoplasmic	

C.N.M.N.9.126	IARS	Isoleucine--tRNA ligase, cytoplasmic	
N.I.DG.N.12.072	KARS	Lysine--tRNA ligase	
N.I.DG.N.12.035	EEF1A1	Elongation factor 1-alpha 1	This protein promotes the GTP-dependent binding of aminoacyl-tRNA to the A-site of ribosomes during protein
C.N.M.N.9.068	EEF1G	Elongation factor 1-	
C.N.M.N.9.069	EEF2	Elongation factor 2	Catalyzes the GTP-dependent ribosomal translocation step during translation elongation.
C.N.P.N.11.001	60S ribosomal subuni	60S ribosomal subunit	
C.N.M.N.9.002	60s ribosomal subunit	60s ribosomal subunit	
C.N.M.N.9.163	NOP56	Nucleolar protein 56	Involved in the early to middle stages of 60S ribosomal subunit biogenesis.
C.N.M.N.9.209	RPL10	60S ribosomal protein	
C.N.M.N.9.210	RPL13	60S ribosomal protein	
C.N.M.N.9.211	RPL13A	60S ribosomal protein	
C.N.M.N.9.212	RPL15	60S ribosomal protein	
C.N.M.N.9.213	RPL17	60S ribosomal protein	
C.N.M.N.9.214	RPL18A	60S ribosomal protein	
C.N.M.N.9.215	RPL21	60S ribosomal protein	
C.N.M.N.9.216	RPL26	60S ribosomal protein	
C.N.M.N.9.217	RPL27A	60S ribosomal protein	
C.N.M.N.9.218	RPL28	60S ribosomal protein	
C.N.M.N.9.219	RPL3	60S ribosomal protein L3	
C.N.M.N.9.220	RPL4	60S ribosomal protein L4	
C.N.M.N.9.221	RPL5	60S ribosomal protein L5	
C.N.M.N.9.222	RPL6	60S ribosomal protein L6	
C.N.M.N.9.223	RPL7	60S ribosomal protein L7	
C.N.P.N.11.045	RPLP0	60S acidic ribosomal protein P0	Structural constituent of ribosome,poly(A) RNA binding,
C.N.M.N.9.224	RPS10	40S ribosomal protein S10	
C.N.M.N.9.109.2	RPS9	40S ribosomal protein S9	
C.N.M.N.9.225	RPS2	40S ribosomal protein S2	

C.N.P.N.11.046	RPS3	40S ribosomal protein S3	
C.N.M.N.9.226	RPS3	40S ribosomal protein S3	
C.N.M.N.9.227	RPS5	40S ribosomal protein S5	
N.N.M.N.3.046	EEF1A1		
C.N.M.N.9.071	EIF2S3	Eukaryotic translation initiation factor 2 subunit	eIF-2 functions in the early steps of protein synthesis by forming a ternary complex with GTP and initiator tRNA.
C.N.M.N.9.072	EIF3	Eukaryotic translation initiation factor 3	
C.N.M.N.9.073	EIF3B	Eukaryotic translation initiation factor 3 subunit	
C.N.M.N.9.074	EIF3C	Eukaryotic translation initiation factor 3 subunit	
C.N.M.N.9.075	EIF3D	Eukaryotic translation initiation factor 3 subunit	
C.N.M.N.9.076	EIF3E	Eukaryotic translation initiation factor 3 subunit	
C.N.M.N.9.077	EIF3F	Eukaryotic translation initiation factor 3 subunit	
C.N.M.N.9.078	EIF3H	Eukaryotic translation initiation factor 3 subunit	
C.N.M.N.9.079	EIF3K	Eukaryotic translation initiation factor 3 subunit	
C.N.M.N.9.080	EIF3L	Eukaryotic translation initiation factor 3 subunit	
C.N.M.N.9.081	EIF4A	Eukaryotic initiation factor 4A	
C.N.M.N.9.082	EIF4A1	Eukaryotic initiation factor 4A-I	
C.N.M.N.9.083	EIF4A2	Eukaryotic initiation factor 4A-II	
C.N.M.N.9.084	EIF4A3	Eukaryotic initiation factor 4A-III	

		C.N.M.N.9.085	EIF4F	Eukaryotic translation initiation factor 4E	
		C.N.M.N.9.086	EIF4g	Eukaryotic translation initiation factor 4 gamma	
		C.N.M.N.9.087	EIF4G1	Eukaryotic translation initiation factor 4 gamma	
		C.N.M.N.9.273	EIF4B		
		C.N.M.N.9.274	EIF6		
		C.N.M.N.9.264	RPL12,		
		C.N.M.N.9.263	RPL14,		
		C.N.M.N.9.267	RPL22,		
		C.N.M.N.9.265	RPL9,		
		C.N.M.N.9.266	RPLP0,		
**Transcription related proteins**	**Transcription factors**	N.I.DG.N.12.009	ARNT2	Aryl hydrocarbon receptor nuclear translocator 2	Transcription factor that plays a role in the development of the hypothalamo-pituitary axis, postnatal brain growth, and visual and renal function
		C.N.M.N.9.055	DDIT3	DNA damage-inducible transcript 3 protein	Multifunctional transcription factor in ER stress response. Plays an essential role in the response to a wide variety of cell stresses and induces cell cycle arrest and apoptosis in
		C.N.M.N.9.067	E2f	Transcription factor E2F	The E2F family plays a crucial role in the control of cell cycle and action of tumor suppressor proteins and is also a target of the transforming proteins of small DNA tumor viruses. It can mediate both cell proliferation and p53- [illegible]
		N.I.DG.N.12.034	E2f	Transcription factor E2F	
		N.N.DG.N.1.045	FOXO1	Forkhead box protein O1	Transcription factor that is the main target of insulin signaling and regulates metabolic homeostasis in response to oxidative stress. Binds to the insulin response element (IRE) with consensus sequence 5'-TT[G/A]TTTTG-3' and the related Daf-16 family binding element (DBE) with consensus sequence 5'-TT[G/A]TTTAC-3'. Activity suppressed by [illegible]

N.N.DG.N.1.053	HES1	Transcription factor HES-1	DNA binding, histone deacetylase binding, RNA polymerase II core promoter proximal region sequence-specific DNA binding transcription factor activity involved in negative regulation of transcription, cell adhesion, cell maturation, cell [illegible]
C.N.M.N.9.207	Rnr (NR2E3)	Photoreceptor-specific nuclear recepto	Orphan nuclear receptor of retinal photoreceptor cells. Transcriptional factor that is an activator of rod development and repressor of cone development.
C.N.M.N.9.242	TFAM	Transcription factor A, mitochondrial	Binds to the mitochondrial light strand promoter and functions in mitochondrial transcription regulation. Required for accurate and efficient promoter recognition by the mitochondrial RNA polymerase.
N.I.DG.N.12.132	XBP1	X-box-binding protein 1	Transcription factor essential for hepatocyte growth, the differentiation of plasma cells, the immunoglobulin secretion, and the unfolded protein response (UPR). Acts during endoplasmic reticulum stress (ER) by activating unfolded protein response (UPR) target genes via direct binding to the [illegible]
N.N.DG.N.1.014	C/ebp	CCAAT/enhancer-binding protein	Important transcription factor regulating the expression of genes involved in immune and inflammatory responses.
N.I.DG.N.12.024	CEBPA	CCAAT/enhancer-binding protein alpha	C/EBP is a DNA-binding protein that recognizes two different motifs: the CCAAT homology common to many promoters and the enhanced core homology common to many enhancers.
C.N.P.N.11.009	Creb	cAMP responsive element binding protein 1	This gene encodes a transcription factor that is a member of the leucine zipper family of DNA binding proteins. This protein binds as a homodimer to the cAMP-responsive element, an octameric palindrome. The protein is phosphorylated by several protein kinases, and induces transcription of genes in response to hormonal stimulation of [illegible]
N.I.DG.N.12.029	Creb	cAMP responsive element binding protein 2	
N.N.DG.N.1.023	Creb	cAMP responsive element binding protein 3	

	N.N.DP.N.2.013	Creb	cAMP responsive element binding protein 4	
	C.N.M.N.9.045	Creb	cAMP responsive element binding protein 5	
	N.N.DG.N.1.074	NFAT (complex)	Nuclear factor of activated T-cells	Transcription factor involved in the transcriptional regulation of osmoprotective and inflammatory genes. Regulates hypertonicity-induced cellular accumulation of osmolytes.
	C.N.M.N.9.159	NFAT (complex)	Nuclear factor of activated T-cells	
	N.N.DG.N.1.075	Nfat (family)	Nuclear factor of activated T-cells	
	N.N.DG.N.1.033	ETV5	ETS translocation variant 5	Binds to DNA sequences containing the consensus nucleotide core sequence GGAA.RNA polymerase II transcription regulatory region sequence-specific DNA binding transcription factor activity involved in positive regulation of [illegible]
	N.N.M.N.3.037	Creb		
	N.N.M.N.3.044	E2f		
	N.N.M.N.3.045	E2F1		
	N.N.M.N.3.055	FOXO1		
	N.N.M.N.3.102	Nfat (family)		
	N.I.DP.N.13.031	NFYB	Nuclear transcription factor Y subunit beta	
**Transcriptional regulatory factors**	N.N.DG.N.1.024	CREM	cAMP-responsive element modulator	Transcriptional regulator that binds the cAMP response element (CRE),
	N.I.DG.N.12.083	NANOG	Homeobox protein NANOG	Transcription regulator involved in inner cell mass and embryonic stem (ES) cells proliferation and self-renewal. When overexpressed, promotes cells to enter into S phase and
	C.N.M.N.9.233	Sin3	SIN3 transcription regulator family member	It is a transcriptional regulatory protein. It contains paired amphipathic helix (PAH) domains, which are important for protein-protein interactions and may mediate repression by the Mad-Max complex.

	N.N.DG.N.1.076	NFκB (complex)	Nuclear factor NF-kappa-B p105 subunit	NFKB is a transcription regulator that is activated by various intra- and extra-cellular stimuli such as cytokines, oxidant-free radicals, ultraviolet irradiation, and bacterial or viral products. Activated NFKB translocates into the nucleus and stimulates the expression of genes involved in a wide variety of biological functions. Inappropriate activation of NFKB has been associated with a number of inflammatory diseases while persistent inhibition of NFKB leads to inappropriate immune cell development or delayed cell growth.
	C.N.P.N.11.027	NFκB (complex)	Nuclear factor NF-kappa-B p105 subunit	
	N.I.DG.N.12.084	NFκB (complex)	Nuclear factor NF-kappa-B p105 subunit	
	N.N.DP.N.2.046	NFκB (complex)	Nuclear factor NF-kappa-B p105 subunit	
	N.I.DP.N.13.033	NFκB (complex)	Nuclear factor NF-kappa-B p105 subunit	
	N.N.M.N.3.103	NFκB (complex)		
**Transcription activators**	N.N.DG.N.1.047	GATA3	Trans-acting T-cell-specific transcription factor GATA-3	Transcriptional activator which binds to the enhancer of the T-cell receptor alpha and delta genes. Binds to the consensus sequence 5'-AGATAG-3'.
	N.N.DG.N.1.073	NEUROD1	Neurogenic differentiation factor 1	Acts as a transcriptional activator，Associates with the p300/CBP transcription coactivator complex to stimulate transcription of the secretin gene as well as the gene encoding the cyclin-dependent kinase inhibitor CDKN1A.
	C.N.M.N.9.197	PURA	Transcriptional activator protein Pur-alpha	This is a probable transcription activator that specifically binds the purine-rich single strand of the PUR element located upstream of the MYC gene. May play a role in the initiation of DNA replication and in recombination.
**Transcriptio**	N.N.DG.N.1.013	BHLHE40	Class E basic helix-loop-helix protein 40	Transcriptional repressor involved in the regulation of the circadian rhythm by negatively regulating the activity of the clock genes and clock-controlled genes.

**n repr esso rs**	C.N.M.N.9.160	NFX1	Transcriptional repressor NF-X1	Binds to the X-box motif of MHC class II genes and represses their expression. May play an important role in regulating the duration of an inflammatory response by limiting the period in which MHC class II molecules are induced by interferon-
	N.N.DG.N.1.072	N-cor	Nuclear receptor corepressor 1	Mediates transcriptional repression by certain nuclear receptors. Part of a complex which promotes histone deacetylation and the formation of repressive chromatin structures which may impede the access of basal transcription factors. Participates in the transcriptional repressor activity [illegible]
	C.N.M.N.9.247	TRIM28	Transcription intermediary factor 1-beta	Nuclear corepressor for KRAB domain-containing zinc finger proteins (KRAB-ZFPs).
	N.N.M.N.3.141	TRIM28		
**Othe**	N.I.DG.N.12.112	SAFB	Scaffold attachment factor B1	Can inhibit cell proliferation.Binds to scaffold/matrix attachment region (S/MAR) DNA and forms a molecular assembly point to allow the formation of a 'transcriptosomal' complex (consisting of SR proteins and RNA polymerase II) coupling transcription and RNA processing (By similarity).
	C.N.M.N.9.147	mediator	mediator	Mediator functions as a bridge to convey information from gene-specific regulatory proteins to the basal RNA polymerase II transcription machinery. Mediator is recruited to promoters by direct interactions with regulatory proteins and serves as a scaffold for the assembly of a functional preinitiation complex with RNA polymerase II and the general [illegible]
	N.I.DG.N.12.116	SMAD3	Mothers against decapentaplegic homolog 3	Receptor-regulated SMAD (R-SMAD) that is an intracellular signal transducer and transcriptional modulator activated by TGF-beta (transforming growth factor) and activin type 1
	N.N.M.N.3.165	FHL2		May function as a molecular transmitter linking various signaling pathways to transcriptional regulation. Negatively regulates the transcriptional repressor E4F1 and may function in cell growth. Inhibits the transcriptional activity of FOXO1 and its apoptotic function by enhancing the interaction of FOXO1 with SIRT1 and FOXO1 deacetylation.

**Histones and related proteins**	C.N.M.N.9.105	H2AFX		Histone H2AX	
	N.N.DG.N.1.051	Hdac	D	Histone deacetylase	Histone deacetylation gives a tag for epigenetic repression and Histones thereby play a central role in transcription regulation, DNA repair, DNA replication and chromosomal stability. DNA accessibility is regulated via a complex set of post-translational modifications of histones, also called histone code, and nucleosome remodeling.
	N.N.DG.N.1.054	histone deace	D	histone deacetylase (HDAC)	
	N.I.DG.N.12.051	Hdac		Histone deacetylase	
	C.N.M.N.9.106	HDAC4		Histone deacetylase 4	
	C.N.M.N.9.108	HIST1H1A		Histone H1.1	Histone H1 protein binds to linker DNA between nucleosomes forming the macromolecular structure known as the chromatin fiber.
	C.N.M.N.9.109	HIST1H1C		Histone H1.2	
	C.N.M.N.9.111	HISTONE		HISTONE	
	N.I.DG.N.12.054	HISTONE		HISTONE	Histone deacetylation gives a tag for epigenetic repression and plays an important role in transcriptional regulation, cell cycle progression and developmental events.
	N.N.DP.N.2.027	Histone h3		Histone h3	
	C.N.M.N.9.110	Histone h3		Histone h3	
	N.I.DG.N.12.052	Histone h3		Histone h3	
	N.N.DG.N.1.055	Histone h4		Histone h4	
	N.I.DG.N.12.053	Histone h4		Histone h4	
	C.N.M.N.9.203	RBBP4		Histone-binding protein RBBP4	Core histone-binding subunit that may target chromatin assembly factors, chromatin remodeling factors and histone deacetylases to their histone substrates in a manner that is regulated by nucleosomal DNA.
	C.N.M.N.9.204	RBBP7		Histone-binding protein RBBP7	Core histone-binding subunit that may target chromatin remodeling factors, histone acetyltransferases and histone deacetylases to their histone substrates in a manner that is regulated by nucleosomal DNA.

	N.I.DG.N.12.124	TIP60	Histone acetyltransferase KAT5	Catalytic subunit of the NuA4 histone acetyltransferase complex which is involved in transcriptional activation of select genes principally by acetylation of nucleosomal
	C.N.M.N.9.040	CHD3	Chromodomain-helicase-DNA-binding protein 3	Component of the histone deacetylase NuRD complex which participates in the remodeling of chromatin by deacetylating histones.
	N.I.DG.N.12.019	CBX5	Chromobox protein homolog 5	Component of heterochromatin that recognizes and binds histone H3 tails methylated at 'Lys-9' (H3K9me), leading to epigenetic repression.
	C.N.M.N.9.168<br>N.N.M.N.3.066<br>N.N.N.N.4.001<br>N.I.DG.N.12.134	NuRD<br>Histone h3<br>Histone h3，<br>histone deacetylase	The chromatin remodeling	核小体重组和脱乙酰基酶复合物（NuRD）是介导组蛋白
**RNA helicases**	C.N.M.N.9.056	DDX1	ATP-dependent RNA helicase DDX1	Acts as an ATP-dependent RNA helicase, able to unwind both RNA-RNA and RNA-DNA duplexes.
	C.N.M.N.9.057	DDX17	Probable ATP-dependent RNA helicase DDX17	
	C.N.M.N.9.058	DDX21	Nucleolar RNA helicase 2	
	C.N.M.N.9.059	DDX3X	ATP-dependent RNA helicase DDX3X	
	C.N.M.N.9.062	DHX9	ATP-dependent RNA helicase A	
	C.N.M.N.9.252	UPF1	Regulator of nonsense transcripts 1	RNA-dependent helicase and ATPase required for nonsense-mediated decay (NMD) of mRNAs containing premature stop codons.
**DNA helicases**	C.N.M.N.9.229	RUVBL1	RuvB-like 1	Possesses single-stranded DNA-stimulated ATPase and ATP-dependent DNA helicase (3' to 5') activity; hexamerization is thought to be critical for ATP hydrolysis and adjacent subunits in the ring-like structure contribute to the ATPase [illegible]

	C.N.M.N.9.230	RUVBL2	RuvB-like 2	Possesses single-stranded DNA-stimulated ATPase and ATP-dependent DNA helicase (5' to 3') activity; hexamerization is thought to be critical for ATP hydrolysis and adjacent subunits in the ring-like structure contribute to the ATPase activity
	C.N.M.N.9.256	XRCC5	X-ray repair cross-complementing protein 5	Single-stranded DNA-dependent ATP-dependent helicase. Has a role in chromosome translocation. The DNA helicase II complex binds preferentially to fork-like ends of double-stranded DNA in a cell cycle-dependent manner.
	C.N.M.N.9.257	XRCC6	X-ray repair cross-complementing protein 6	Single-stranded DNA-dependent ATP-dependent helicase. Has a role in chromosome translocation. The DNA helicase II complex binds preferentially to fork-like ends of double-stranded DNA in a cell cycle-dependent manner.
	N.N.M.N.3.161	XRCC6	X-ray repair cross-complementing protein 6	Single-stranded DNA-dependent ATP-dependent helicase. Has a role in chromosome translocation. The DNA helicase II complex binds preferentially to fork-like ends of double-stranded DNA in a cell cycle-dependent manner.
**DNA, RNA binding proteins**	C.N.M.N.9.153	NCBP1	Nuclear cap-binding protein subunit 1	RNA cap binding, mRNA cis splicing, via spliceosome, mRNA transport。Component of the cap-binding complex (CBC), which binds cotranscriptionally to the 5'-cap of pre-mRNAs and is involved in various processes such as pre-mRNA splicing, translation regulation, nonsense-mediated mRNA decay, RNA-mediated gene silencing (RNAi) by microRNAs (miRNAs) and mRNA export.
	C.N.M.N.9.162	NONO	Non-POU domain-containing octamer-binding protein	DNA- and RNA binding protein, involved in several nuclear processes. Involved in pre-mRNA splicing, probably as a heterodimer with SFPQ.
	C.N.P.N.11.030	PABPC1	Polyadenylate-binding protein 1	Binds the poly(A) tail of mRNA, including that of its own transcript. May be involved in cytoplasmic regulatory processes of mRNA metabolism such as pre-mRNA splicing.

C.N.M.N.9.171	PABPC1	Polyadenylate-binding protein 1	
C.N.M.N.9.174	PCBP1	Poly(rC)-binding protein 1	Single-stranded nucleic acid binding protein that binds preferentially to oligo dC.
C.N.M.N.9.205	RBMX	RNA-binding motif protein, X chromosome	RNA-binding protein that plays several role in the regulation of pre- and post-transcriptional processes. Implicated in tissue-specific regulation of gene transcription and alternative splicing of several pre-mRNAs. Binds to and stimulates transcription from the tumor suppressor TXNIP gene promoter; may thus be involved in tumor suppression.
C.N.M.N.9.232	SFPQ	Splicing factor, proline- and glutamine-rich	DNA- and RNA binding protein, involved in several nuclear processes. Essential pre-mRNA splicing factor required early in spliceosome formation and for splicing catalytic step II, probably as a heteromer with NONO.
C.N.M.N.9.236	SRSF2	Serine/arginine-rich splicing factor 2	Necessary for the splicing of pre-mRNA. It is required for formation of the earliest ATP-dependent splicing complex and interacts with spliceosomal components bound to both the 5'- and 3'-splice sites during spliceosome assembly.
N.I.DG.N.12.121	TARDBP	TAR DNA-binding protein 43	DNA and RNA-binding protein which regulates transcription and splicing. Involved in the regulation of CFTR splicing.Stabilizes the low molecular weight neurofilament (NFL) mRNA through a direct interaction with the 3' UTR.
C.N.M.N.9.239	TARDBP	TAR DNA-binding protein 43	
C.N.P.N.11.050	TARDBP	TAR DNA-binding protein 43	
C.N.M.N.9.251	U2AF2	Splicing factor U2AF 65 kDa subunit	Necessary for the splicing of pre-mRNA.

C.N.M.N.9.196	PTBP1	Polypyrimidine tract-binding protein 1	Plays a role in pre-mRNA splicing and in the regulation of alternative splicing events.
C.N.M.N.9.014	ALYREF	THO complex subunit 4	Nucleic acid binding
C.N.M.N.9.134	KHSRP	Far upstream element-binding protein 2	Binds to the dendritic targeting element and may play a role in mRNA trafficking (By similarity).
C.N.M.N.9.139	MAGOH	Protein mago nashi homolog	Core component of the splicing-dependent multiprotein exon junction complex (EJC) deposited at splice junctions on mRNAs
C.N.M.N.9.107	HDLBP	Vigilin	Poly(A) RNA binding, lipid binding，play a role in cell sterol metabolism. It may function to protect cells from over-accumulation of cholesterol.
C.N.M.N.9.231	SATB1	DNA-binding protein SATB1	Crucial silencing factor contributing to the initiation of X inactivation mediated by Xist RNA that occurs during embryogenesis and in lymphoma (By similarity).Reprograms chromatin organization and the transcription profiles of breast tumors to promote growth and metastasis.Delineates specific epigenetic modifications at target gene loci, directly up-regulating metastasis-associated genes while down-regulating tumor-suppressor genes. Reprograms chromatin organization and the transcription profiles of breast tumors to promote growth and metastasis.
C.N.M.N.9.092	EWSR1	RNA-binding protein EWS	Might normally function as a transcriptional repressor.They may disturb gene expression by mimicking, or interfering with the normal function of CTD-POLII within the transcription initiation complex. They may also contribute to an aberrant activation of the fusion protein target genes.
C.N.M.N.9.243	TIAL1	Nucleolysin TIAR	RNA-binding protein. Possesses nucleolytic activity against cytotoxic lymphocyte target cells. May be involved in apoptosis

	N.N.M.N.3.131	SERPINE1	May play a role in the regulation of mRNA stability. Binds to the 3'-most 134 nt of the SERPINE1/PAI1 mRNA, a region which confers cyclic nucleotide regulation of message decay	
	N.N.DG.N.1.114	ZFP36	Tristetraprolin	mRNA-binding protein involved in post-transcriptional regulation of AU-rich element (ARE)-containing mRNAs. Acts by specifically binding ARE-containing mRNAs and promoting their degradation. Recruits deadenylase CNOT7 (and probably the CCR4-NOT complex) via association with CNOT1. Plays a key role in the post-transcriptional regulation of tumor necrosis factor (TNF).
	C.N.M.N.9.272	Ku		
	C.N.M.N.9.276	SF3B3	Splicing factor 3B subunit 3	Subunit of the splicing factor SF3B required for 'A' complex assembly formed by the stable binding of U2 snRNP to the branchpoint sequence (BPS) in pre-mRNA. Sequence independent binding of SF3A/SF3B complex upstream of the branch site is essential, it may anchor U2 snRNP to the pre-mRNA. May also be involved in the assembly of the 'E' complex. Belongs also to the minor U12-dependent spliceosome, which is involved in the splicing of rare class of nuclear pre-mRNA intron.
**DNA, RNA polymerase, DNA topoisomerase**	C.N.M.N.9.183	POLE	DNA polymerase epsilon catalytic subunit A	Participates in DNA repair and in chromosomal DNA replication.
	C.N.M.N.9.184	POLR2B	DNA-directed RNA polymerase II subunit RPB2	DNA-dependent RNA polymerase catalyzes the transcription of DNA into RNA using the four ribonucleoside triphosphates as substrates.

	C.N.M.N.9.185	POLR2E	DNA-directed RNA polymerases I, II, and III subunit RPABC1	DNA-dependent RNA polymerase catalyzes the transcription of DNA into RNA using the four ribonucleoside triphosphates as substrates.
	C.N.M.N.9.121	Holo RNA polymeras	Holo RNA polymerase Ⅱ	
	C.N.M.N.9.206	RNA polymerase Ⅱ	RNA polymerase Ⅱ	
	C.N.P.N.11.043	RNA polymerase Ⅱ	RNA polymerase Ⅱ	
	N.I.DG.N.12.109	RNA polymerase Ⅱ	RNA polymerase Ⅱ	
	C.N.M.N.9.244	TOP1	DNA topoisomerase 1	Releases the supercoiling and torsional tension of DNA introduced during the DNA replication and transcription by transiently cleaving and rejoining one strand of the DNA duplex. Introduces a single-strand break via transesterification at a target site in duplex DNA.
	C.N.M.N.9.245	TOP2B	DNA topoisomerase 2-beta	Control of topological states of DNA by transient breakage and subsequent rejoining of DNA strands. Topoisomerase II makes double-strand breaks.
	C.N.P.N.11.052	TPI1	Triosephosphate	
	C.N.M.N.9.175	PCNA	Proliferating cell nuclear antigen	This protein is an auxiliary protein of DNA polymerase delta and is involved in the control of eukaryotic DNA replication by increasing the polymerase's processibility during elongation of the leading strand.
	C.N.M.N.9.208	RPA2	Replication protein A 32 kDa subunit	As part of the heterotrimeric replication protein A complex (RPA/RP-A), binds and stabilizes single-stranded DNA intermediates, that form during DNA replication or upon DNA stress.
	N.N.M.N.3.128	RNA polymerase Ⅱ		
**Proteins against oxidative stress**	N.N.DG.N.1.105	Sod	Superoxide dismutase [Cu-Zn]	Destroys radicals which are normally produced within the cells and which are toxic to biological systems.
	C.N.M.N.9.172.2	SOD1	Superoxide dismutase [Cu-Zn]	
	C.N.M.N.9.235	SOD2	Superoxide dismutase [Mn], mitochondrial	Destroys superoxide anion radicals which are normally produced within the cells and which are toxic to biological systems.

	C.N.M.N.9.172	PARK7	Protein DJ-1	Protects cells against oxidative stress and cell death.
	N.I.DG.N.12.082	MT1L	Metallothionein-1L	Metallothioneins have a high content of cysteine residues that bind various heavy metals; these proteins are transcriptionally regulated by both heavy metals and glucocorticoids.
	C.N.M.N.9.234	SIRT3	NAD-dependent protein deacetylase sirtuin-3, mitochondrial	Activates or deactivates mitochondrial target proteins by deacetylating key lysine residues. Important for regulating tissue-specific ATP levels.
	N.N.M.N.3.109	PARK7	Protein deglycase DJ-1	Protein deglycase that repairs methylglyoxal- and glyoxal-glycated amino acids and proteins, and releases repaired proteins and lactate or glycolate, respectively. Deglycates cysteines, arginines and lysines residues in proteins, and thus reactivates these proteins by reversing glycation by glyoxals. Acts on early glycation intermediates (hemithioacetals and aminocarbinols), preventing the formation of advanced glycation endproducts (AGE) (PubMed:25416785). Plays an important role in cell protection against oxidative stress and cell death acting as oxidative stress sensor and redox-sensitive chaperone and protease; functions probably related to its primary function
	N.N.M.N.3.133	SOD1		
	C.N.M.N.9.269	PRDX3,	Thioredoxin-dependent peroxide reductase, mitochondrial	Involved in redox regulation of the cell. Protects radical-sensitive enzymes from oxidative damage by a radical-generating system. Acts synergistically with MAP3K13 to regulate the activation of NF-kappa-B in the cytosol.
	N.I.DP.N.13.041	Sod	Superoxide dismutase	
	N.I.DP.N.13.006	CAT	catalase	
	N.N.M.N.3.028	CAT	catalase	
**NO, NOS**	N.I.DG.N.12.085	nitric oxide	nitric oxide	

	N.N.DP.N.2.047	Nos	Nitric oxide synthase, brain	Produces nitric oxide (NO) which is a messenger molecule with diverse functions throughout the body. In the brain and peripheral nervous system, NO displays many properties of a neurotransmitter. Probably has nitrosylase activity and mediates cysteine S-nitrosylation of cytoplasmic target proteins such SRR
**Retinoic Acid Receptors**	N.N.DG.N.1.099	Rar (RARA)	Retinoic acid receptor alpha	RARA plays an essential role in the regulation of retinoic acid-induced germ cell development during spermatogenesis. Has a role in the survival of early spermatocytes at the beginning prophase of meiosis. In Sertoli cells, may promote the survival and development of early meiotic prophase spermatocytes.
	N.N.DG.N.1.103	Rxr	retinoid X receptor	Receptor for retinoic acid. Retinoic acid receptors bind as heterodimers to their target response elements in response to their ligands, all-trans or 9-cis retinoic acid, and regulate gene expression in various biological processes.
	C.N.P.N.11.047	Rxr	retinoid X receptor	
	N.N.M.N.3.130	Rxr		
**Proteins associated with G protein and its signal transduction pathway**	N.N.DG.N.1.101	RGS2	Regulator of G-protein signaling 2	Inhibits signal transduction by increasing the GTPase activity of G protein alpha subunits thereby driving them into their inactive GDP-bound form. May play a role in leukemogenesis. Plays a role in negative feedback control pathway for adenylyl cyclase signaling. Binds EIF2B5 and blocks its activity, thereby inhibiting the translation of mRNA [illegible]
	N.N.DG.N.1.002	ADCY	Adenylate cyclase	
	N.N.DP.N.2.002	ADCY	Adenylate cyclase	
	C.N.P.N.11.003	ADCY	Adenylate cyclase	
	N.I.DG.N.12.048	Gpcr	Probable G-protein coupled receptor	
	N.N.DP.N.2.023	GNAO1	Guanine nucleotide-binding protein G(o)	GTPase activity, GTP binding, signal transducer activity
	N.N.M.N.3.005	ADCY		

	N.N.M.N.3.057	G protein alpha		
	N.N.M.N.3.058	G protein beta		
	N.N.M.N.3.059	G protein beta gamma		
	N.N.M.N.3.061	GNAO		
	N.N.M.N.3.062	GNAO1		
	N.N.M.N.3.118	PLC		
	N.N.M.N.3.117	PLC gamma		
	N.N.DP.N.2.055	PLC	1-phosphatidylinositol phosphodiesterase	
	N.N.DG.N.1.088	PLC gamma	1-phosphatidylinositol 4,5-bisphosphate phosphodiesterase gamma-1	Mediates the production of the second messenger molecules diacylglycerol (DAG) and inositol 1,4,5-trisphosphate (IP3). Plays an important role in the regulation of intracellular signaling cascades
	C.N.P.N.11.035	PLCB1	1-phosphatidylinositol 4,5-bisphosphate phosphodiesterase beta-1	The production of the second messenger molecules diacylglycerol (DAG) and inositol 1,4,5-trisphosphate (IP3) is mediated by activated phosphatidylinositol-specific phospholipase C enzymes
	N.I.DP.N.13.001	ADCY		
	N.I.DP.N.13.037	PLC		
**ATP synthesis related proteins**	N.I.DP.N.13.002	ATP5B	ATP synthase subunit beta	
	N.N.DG.N.1.007	AMPK	5'-AMP-activated protein kinase catalytic subunit alpha	Catalytic subunit of AMP-activated protein kinase (AMPK), an energy sensor protein kinase that plays a key role in regulating cellular energy metabolism. In response to reduction of intracellular ATP levels, AMPK activates energy-producing pathways and inhibits energy-consuming processes: inhibits protein, carbohydrate and lipid biosynthesis, as well as cell growth and proliferation.
	N.I.DG.N.12.006	AMPK	5'-AMP-activated protein kinase catalytic subunit	
	N.N.M.N.3.012	AMPK		
**Alkaline phosphatase**	N.I.DG.N.12.005	Alp	Alkaline phosphatase	
	N.N.DP.N.2.004	Alp		
	N.N.M.N.3.010	Alp		

**Heterogeneous nuclear ribonucleoprotein**	C.N.M.N.9.112	HNRNPA1	Heterogeneous nuclear ribonucleoprotein A1	Involved in the packaging of pre-mRNA into hnRNP particles, transport of poly(A) mRNA from the nucleus to the cytoplasm and may modulate splice site selection.
	C.N.P.N.11.018	HNRNPC	Heterogeneous nuclear ribonucleoproteins C1/C2	Binds pre-mRNA and nucleates the assembly of 40S hnRNP particles. Single HNRNPC tetramers bind 230-240 nucleotides.
	C.N.M.N.9.113	HNRNPD	Heterogeneous nuclear ribonucleoprotein D0	Binds with high affinity to RNA molecules that contain AU-rich elements (AREs) found within the 3'-UTR of many proto-oncogenes and cytokine mRNAs.
	C.N.M.N.9.114	HNRNPH1	Heterogeneous nuclear ribonucleoprotein H	This protein is a component of the heterogeneous nuclear ribonucleoprotein (hnRNP) complexes which provide the substrate for the processing events that pre-mRNAs undergo before becoming functional, translatable mRNAs in the [illegible]
	C.N.M.N.9.115	HNRNPH2	Heterogeneous nuclear ribonucleoprotein H2	
	C.N.M.N.9.116	HNRNPH3	Heterogeneous nuclear ribonucleoprotein H3	
	C.N.M.N.9.117	HNRNPL	Heterogeneous nuclear ribonucleoprotein L	Splicing factor binding to exonic or intronic sites and acting as either an activator or repressor of exon inclusion.
	C.N.M.N.9.118	HNRNPM	Heterogeneous nuclear ribonucleoprotein M	
	C.N.M.N.9.119	HNRNPR	Heterogeneous nuclear ribonucleoprotein R	Component of ribonucleosomes, which are complexes of at least 20 other different heterogenious nuclear ribonucleoproteins (hnRNP). hnRNP play an important role in processing of precursor mRNA in the nucleus.
	C.N.M.N.9.120	HNRNPU	Heterogeneous nuclear ribonucleoprotein U	Component of the CRD-mediated complex that promotes MYC mRNA stabilization. Binds to pre-mRNA. Has high affinity for scaffold-attached region (SAR) DNA.

		N.I.DG.N.12.055	HNRNPU	Heterogeneous nuclear ribonucleoprotein U	
		C.N.M.N.9.238	SYNCRIP	Heterogeneous nuclear ribonucleoprotein Q	Heterogenous nuclear ribonucleoprotein (hnRNP) implicated in mRNA processing mechanisms. Component of the CRD-mediated complex that promotes MYC mRNA stability.
		C.N.M.N.9.261	HNRNPA0,		
**Other types of protein classification**	**1**	N.N.DG.N.1.018	Cg	Cathepsin G (CTSG)?	The protein is a member of the peptidase S1 protein family,it has a specificity similar to that of chymotrypsin C, and may participate in the killing and digestion of engulfed pathogens, and in connective tissue remodeling at sites of inflammation.
		C.N.N.N.10.004	Cg		
		N.I.DP.N.13.012	Cg		
		N.N.DP.N.2.009	Cg		
		C.N.M.N.9.038	Cg		
		N.I.DG.N.12.025	Cg		
	**2**	C.N.P.N.11.037	POT1	Protection of telomeres protein 1	Component of the telomerase ribonucleoprotein (RNP) complex that is essential for the replication of chromosome termini
		C.N.P.N.11.051	TERF1	Telomeric repeat-binding factor 1	Binds the telomeric double-stranded 5'-TTAGGG-3' repeat and negatively regulates telomere length. Involved in the regulation of the mitotic spindle.
	**3**	N.I.DG.N.12.047	GDNF	Glial cell line-derived neurotrophic factor	Neurotrophic factor that enhances survival and morphological differentiation of dopaminergic neurons and increases their high-affinity dopamine uptake.
		C.N.M.N.9.166	NTF3	Neurotrophin-3	Seems to promote the survival of visceral and proprioceptive sensory neurons.
	**4**	C.N.M.N.9.021	APP	Amyloid beta A4 protein	Unctions as a cell surface receptor and performs on the surface of neurons relevant to neurite growth, neuronal adhesion and axonogenesis. Involved in cell mobility and transcription regulation through protein-protein interactions.

	N.I.DG.N.12.008	APP	Amyloid beta A4 protein	
**5**	C.N.M.N.9.009	ALDH	Aldehyde dehydrogenase, mitochondrial	
	C.N.M.N.9.011	ALDH2	Aldehyde dehydrogenase, mitochondrial	
	C.N.M.N.9.010	ALDH1A1	Retinal dehydrogenase 1	
	N.N.DG.N.1.005	ALDH1A1	Retinal dehydrogenase 1	
**6**	C.N.N.N.10.008	tretinoin	tretinoin	
	N.I.DG.N.12.127	tretinoin	tretinoin	
**7**	C.N.M.N.9.181	PHB	Prohibitin, isoform CRA_a	Prohibitin inhibits DNA synthesis. It has a role in regulating proliferation.
	N.N.M.N.3.113	PHB		
**8**	N.N.DG.N.1.071	Mlc	Membrane protein MLC	Myosin light chain，MLC
	N.N.M.N.3.100	Mlc	Membrane protein MLC	
**9**	C.N.M.N.9.017	ANXA1	Annexin A1	Calcium/phospholipid-binding protein which promotes membrane fusion and is involved in exocytosis.
	C.N.M.N.9.018	ANXA2	Annexin A2	
	N.N.M.N.3.013	ANXA4		
	N.N.M.N.3.014	ANXA6		
**10**	C.N.M.N.9.151	MYH9	Myosin-9	Cellular myosin that appears to play a role in cytokinesis, cell shape, and specialized functions such as secretion and
	N.N.M.N.3.101	Myosin		
	N.N.M.N.3.142	Tropomyosin		
	C.N.M.N.9.054	DCTN1	Dynactin subunit 1	Required for the cytoplasmic dynein-driven retrograde movement of vesicles and organelles along microtubules.
	N.N.M.N.3.043	Dynamin		
	C.N.M.N.9.066	DYNC1H1	Cytoplasmic dynein 1 heavy chain 1	Cytoplasmic dynein 1 acts as a motor for the intracellular retrograde motility of vesicles and organelles along
**11**	C.N.M.N.9.125	HTT	Huntingtin	May play a role in microtubule-mediated transport or vesicle function.
	N.N.M.N.3.075	HTT	Huntingtin	
	N.I.DP.N.13.020	HTT	Huntingtin	

	**12**	C.N.M.N.9.165	NPM1	Nucleophosmin	Involved in diverse cellular processes such as ribosome biogenesis, centrosome duplication, protein chaperoning, histone assembly, cell proliferation, and regulation of tumor suppressors p53/TP53 and ARF.
		N.N.M.N.3.104	NPM1		
**Unclassified molecules in each dataset**	**1**	N.N.DG.N.1.043	Fibrinogen (FGL2)	Fibrinogen	May play a role in physiologic lymphocyte functions at mucosal sites.
		N.N.DG.N.1.097	PTPase	protein tyrosine phosphatases	
		N.N.DG.N.1.036	F3	Tissue factor	Initiates blood coagulation by forming a complex with circulating factor VII or VIIa.
		N.N.DG.N.1.085	PEPCK	Phosphoenolpyruvate carboxykinase [GTP], mitochondrial	Catalyzes the conversion of oxaloacetate (OAA) to phosphoenolpyruvate (PEP), the rate-limiting step in the metabolic pathway that produces glucose from lactate and other precursors derived from the citric acid cycle.
		N.N.DG.N.1.037	FASN	Fatty acid synthase	Oxidoreductase activity, zinc ion binding
		N.N.DG.N.1.061	JINK1/2	?	
	**2**	N.N.DP.N.2.064	TGM2	Protein-glutamine gamma-glutamyltransferase 2	Catalyzes the cross-linking of proteins and the conjugation of polyamines to proteins.
	**3**	N.N.M.N.3.119	Pld	Phospholipase D	May have a role in signal-induced cytoskeletal regulation and/or endocytosis.
		N.N.M.N.3.159	VDAC1	Voltage-dependent anion-selective channel protein 1	Forms a channel through the mitochondrial outer membrane and also the plasma membrane. The channel at the outer mitochondrial membrane allows diffusion of small hydrophilic molecules; in the plasma membrane it is involved in cell volume regulation and apoptosis.
		N.N.M.N.3.008	ALB		

N.N.M.N.3.009	Aldose Reductase		Catalyzes the NADPH-dependent reduction of a wide variety of carbonyl-containing compounds to their corresponding alcohols with a broad range of catalytic efficiencies.
N.N.M.N.3.016	APCS	Serum amyloid P-component	Can interact with DNA and histones and may scavenge nuclear material released from damaged circulating cells. May also function as a calcium-dependent lectin.
N.N.M.N.3.029	Caveolin		
N.N.M.N.3.047	EPB41	Protein 4.1	Protein 4.1 is a major structural element of the erythrocyte membrane skeleton. It plays a key role in regulating membrane physical properties of mechanical stability and deformability by stabilizing spectrin-actin interaction. [illegible]
N.N.M.N.3.052	Ferritin	Stores iron in a soluble, non-toxic, readily available form. Important for iron homeostasis. Has ferroxidase activity. Iron is taken up in the ferrous form and deposited as ferric hydroxides after oxidation	
N.N.M.N.3.053	Fibrin		
N.N.M.N.3.093	Ikb		Inhibits NF-kappa-B by complexing with and trapping it in the cytoplasm. However, the unphosphorylated form resynthesized after cell stimulation is able to bind NF-kappa-B allowing its transport to the nucleus and protecting it to further NFKBIA-dependent inactivation.
N.N.M.N.3.094	LMNA	lamin	

	N.N.M.N.3.127	RICTOR	Rapamycin-insensitive companion of mTOR	Subunit of mTORC2, which regulates cell growth and survival in response to hormonal signals. mTORC2 is activated by growth factors, mTORC2 seems to function upstream of Rho GTPases to regulate the actin cytoskeleton, probably by activating one or more Rho-type guanine nucleotide exchange factors. mTORC2 promotes the serum-[illegible]
	N.N.M.N.3.164	HPX	Hemopexin	
	N.N.M.N.3.168	NADPH oxidase		
**5**	N.I.DG.N.12.120	SUPT16H	FACT complex subunit SPT16	Component of the FACT complex, a general chromatin factor that acts to reorganize nucleosomes. The FACT complex is involved in multiple processes that require DNA as a template such as mRNA elongation, DNA replication and DNA repair.The FACT complex is probably also involved in phosphorylation of 'Ser-392' of p53/TP53 via its association [illegible]
	N.I.DG.N.12.136	hemoglobin		
**6**	N.I.DP.N.13.049	DBI	Acyl-CoA-binding protein	
**7**	C.N.M.N.9.015	ANP32B	Acidic leucine-rich nuclear phosphoprotein 32 family member B	Multifunctional protein working as a cell cycle progression factor as well as a cell survival factor.
	C.N.M.N.9.023	BRCA1	Breast cancer type 1 susceptibility protein	
	C.N.M.N.9.024	BRCA2	Breast cancer type 2 susceptibility protein	
	C.N.M.N.9.128	Importin alpha	Importin alpha	
	C.N.M.N.9.129	Importin beta	Importin beta	
	C.N.M.N.9.135	KPNB1	Importin subunit beta-1	
	C.N.M.N.9.131	IPO5	Importin-5	Protein transporter activity
	C.N.M.N.9.046	CSE1L	Exportin-2	Export receptor for importin-alpha.Mediates importin-alpha re-export from the nucleus to the cytoplasm after import substrates (cargos) have been released into the nucleoplasm.

C.N.M.N.9.100	GBAS	Protein NipSnap homolog 2	ATP biosynthetic process, negative regulation of ATP citrate synthase activity,oxidative phosphorylation
C.N.M.N.9.145	MDH2	Malate dehydrogenase, mitochondrial	
C.N.M.N.9.254	VPS26A	Vacuolar protein sorting-associated protein 26A	Acts as component of the retromer cargo-selective complex (CSC). The CSC is believed to be the core functional component of retromer or respective retromer complex variants acting to prevent missorting of selected transmembrane cargo proteins into the lysosomal degradation pathway.
C.N.M.N.9.255	VPS35	Vacuolar protein sorting-associated protein 35	Act as component of the retromer cargo-selective complex (CSC). The CSC is believed to be the core functional component of retromer or respective retromer complex variants acting to prevent missorting of selected transmembrane cargo proteins into the lysosomal degradation pathway.
C.N.M.N.9.020	APEX1	DNA-(apurinic or apyrimidinic site) lyase/APEX nuclease 1	
C.N.M.N.9.063	DNMT3B	DNA (cytosine-5)-methyltransferase 3B	
C.N.M.N.9.191	PRKDC	DNA-dependent protein kinase catalytic subunit	Phosphotransferase activity, alcohol group as acceptor
C.N.M.N.9.146	MECP2	Methyl-CpG-binding protein 2	Chromosomal protein that binds to methylated DNA.
C.N.M.N.9.060	DGCR8	Microprocessor complex subunit DGCR8	Component of the microprocessor complex that acts as a RNA- and heme-binding protein that is involved in the initial step of microRNA (miRNA) biogenesis.
C.N.M.N.9.007	ADAR	Double-stranded RNA-specific adenosine deaminase	

C.N.M.N.9.025	C1QBP	Complement component 1 Q subcomponent-binding protein, mitochondrial	Is believed to be a multifunctional and multicompartmental protein involved in inflammation and infection processes, ribosome biogenesis, regulation of apoptosis, transcriptional regulation and pre-mRNA splicing.
C.N.M.N.9.098	GABARAPL2	Gamma-aminobutyric acid receptor-associated protein-like 2	Ubiquitin-like modifier involved in intra-Golgi traffic. Modulates intra-Golgi transport through coupling between NSF activity and SNAREs activation.Involved in autophagy. Plays a role in mitophagy which contributes to regulate mitochondrial quantity and quality by eliminating the mitochondria to a basal level to fulfill cellular energy requirements and preventing excess ROS production.
C.N.M.N.9.154	NCL	Nucleolin	Nucleolin is the major nucleolar protein of growing eukaryotic cells. It is found associated with intranucleolar chromatin and pre-ribosomal particles.
C.N.M.N.9.167	NUP214	Nuclear pore complex protein Nup214	May serve as a docking site in the receptor-mediated import of substrates across the nuclear pore complex.
C.N.M.N.9.026	CAV1	Caveolin-1	May act as a scaffolding protein within caveolar membranes.
C.N.M.N.9.064	DPYSL2	Dihydropyrimidinase-related protein 2	Plays a role in neuronal development and polarity, as well as in axon growth and guidance, neuronal growth cone collapse and cell migration.
C.N.M.N.9.093	FBL	rRNA 2'-O-methyltransferase fibrillarin	methyltransferase activity，RNA binding
C.N.M.N.9.095	FHIT	Diadenosine triphosphate hydrolase	Cleaves P(1)-P(3)-bis(5'-adenosyl) triphosphate (Ap3A) to yield AMP and ADP.

	C.N.M.N.9.103	GRIN2A	Glutamate receptor ionotropic, NMDA 2A	NMDA receptor subtype of glutamate-gated ion channels possesses high calcium permeability and voltage-dependent sensitivity to magnesium.
	C.N.M.N.9.144	MATR3	Matrin-3	May play a role in transcription or may interact with other nuclear matrix proteins to form the internal fibrogranular network. nucleotide binding，poly(A) RNA binding，structural molecule activity，zinc ion binding
	C.N.M.N.9.228	RTN4	Reticulon-4	Developmental neurite growth regulatory factor with a role as a negative regulator of axon-axon adhesion and growth, and as a facilitator of neurite branching. Regulates neurite fasciculation, branching and extension in the developing nervous system.
	C.N.M.N.9.161	NIPSNAP1	Protein NipSnap homolog 1	neurotransmitter binding, sensory perception of pain
	C.N.M.N.9.268	aldehyde dehydrogenase (NAD)		
**9**	C.N.P.N.11.048	SCG2	Secretogranin-2	Secretogranin-2 is a neuroendocrine secretory granule protein, which is the precursor for biologically active peptides.

D: The same protein shown in a dataset in two different molecular networks, considering the same proteins involved in the different network function, and count it only one time.
