# Supplementary material for: Multiomics-Based Signaling Pathway Network Alterations in Human Non-functional Pituitary Adenomas
Source: Front Endocrinol (Lausanne). 2019 Dec 17;10:835. doi: 10.3389/fendo.2019.00835 (PMC6928143; doi:10.3389/fendo.2019.00835)
Supplement: Supplementary file 1 [file Presentation_1.zip › Supplemental Table 6_v1.pdf]

Supplemental Table 6. Statically significantly canonical pathways that were mined from only one dataset

Canonical pathway seriel number	Canonical pathway name
Dataset 1 only	
N.N.DG.C.1.015	Adipogenesis pathway
N.N.DG.C.1.056	Angiopoietin Signaling
N.N.DG.C.1.029	Calcium Transport Ⅰ
N.N.DG.C.1.025	Calcium-induced T Lymphocyte Apoptosis
N.N.DG.C.1.013	CCR3 Signaling in Eosinophils
N.N.DG.C.1.068	CCRS Signaling in Macrophages
N.N.DG.C.1.033	CD28 Signaling in T Helper Cells
N.N.DG.C.1.031	Chemokine Signaling
N.N.DG.C.1.040	D-myo-inositol(1,4,5)-trisphosphate Degradation
N.N.DG.C.1.047	EGF Signaling
N.N.DG.C.1.054	Fc Epsilon RI Signaling
N.N.DG.C.1.062	GDNF Family Ligand-Receptor Interactions
N.N.DG.C.1.027	Gaq Signaling
N.N.DG.C.1.007	Granulocyte Adhesion and Diapedesis
N.N.DG.C.1.052	iCOS-iCOSL Signaling in T Helper Cells
N.N.DG.C.1.024	IL-17A Signaling in Fibroblasts
N.N.DG.C.1.061	IL-3 Signaling
N.N.DG.C.1.034	MIF Regulation of Innate Immunity
N.N.DG.C.1.044	Nur77 Signaling in T Lymphocytes
N.N.DG.C.1.064	PTEN Signaling
N.N.DG.C.1.059	Putrescine Biosynthesis Ⅲ
N.N.DG.C.1.037	Regulation of the Epithelial-Mesenchymal Transition Pathway
N.N.DG.C.1.060	Renin-Angiotensin Signaling
N.N.DG.C.1.028	Role of MAPK Signaling in the Pathogenesis of Influenza
N.N.DG.C.1.023	Role of NFAT in Cardiac Hypertrophy
N.N.DG.C.1.057	Role of Osteoblasts, Osteoclasts and Chondrocytes in Rheumatoid Arthritis
N.N.DG.C.1.065	Superpathway of D-myo-inositol(1,4,5)-trisphosphate Metabolism
N.N.DG.C.1.045	T Cell Receptor Signaling
N.N.DG.C.1.035	VEGF Family Ligand-Receptor Interaction
N.N.DG.C.1.048	Cholecystokinin-Gastrin-mediated Signaling

Dataset 2 only

N.N.DP.C.2.018	Acyl-CoA Hydrolysis
N.N.DP.C.2.013	Methylglyoxal Degradation Ⅰ
N.N.DP.C.2.010	Prolactin Signaling
N.N.DP.C.2.020	Serotonin and melatonin Biosynthess

Dataset 3 only

N.N.M.C.3.049	Clathrin-mediated Endocytosis Signaling
N.N.M.C.3.074	Cysteine Biosynthesis Ⅲ(mammalia)
N.N.M.C.3.041	D-glucuronate Degradation Ⅰ
N.N.M.C.3.040	G Protein Signaling Mediated by Tubby
N.N.M.C.3.054	Parkinson’ s Signalng
N.N.M.C.3.083	Pyrimidine Deoxyribonucleotides De Novo Biosynthesis Ⅰ
N.N.M.C.3.065	Retinoic acid Mediated Apoptosis Signaling
N.N.M.C.3.089	Tryptophan Degradation Ⅲ(Eukaryotic)

Dataset 4 only

N.N.N.C.4.029	Altered T cell and B cell signaling in Rheumatoid arthritis
N.N.N.C.4.026	BMP signaling pathway
N.N.N.C.4.019	Gaft-versus-Host Disease Signaling
N.N.N.C.4.002	Hepatic Cholestasis
N.N.N.C.4.024	IL-10 signaling
N.N.N.C.4.018	iNOS Signaling
N.N.N.C.4.015	Netrin Signaling
N.N.N.C.4.008	p38 MAPK Signaling
N.N.N.C.4.022	Phototransduction Pathway
N.N.N.C.4.004	Renal Cell Carcinoma Signaling
N.N.N.C.4.021	Role of Cytokines in Mediating Communication between Immune Cells
N.N.N.C.4.016	Role of Hypercytokinemia/hyperchemokinemia in the Pathogenesis of Influenza
N.N.N.C.4.013	Role of Macrophage, Fibroblasts and Endothelial Cells inRheumatoid Arthritis
N.N.N.C.4.001	Toll-like Receptor Signaling
N.N.N.C.4.010	Wint/β-catenin Signaling

Dataset 5 only

N.I.DG.C.12.029	Acute myeloid leukemia signaling

N.I.DG.C.12.023	Estrogen-mediate S-phase entry
N.I.DG.C.12.008	FLT3 signaling hematopoietic progenitor cells
N.I.DG.C.12.021	Myc mediated apoptosis signaling
N.I.DG.C.12.024	NGF signaling

Dataset 6 only

N.I.DP.C.13.009	Endothelin-1 signaling
N.I.DP.C.13.024	Ketogenesis
N.I.DP.C.13.006	Semaphorin signaling in neurons

Dataset 7 only

C.N.M.C.9.125	2-ketoglutarate Dehydrogenase Complex
C.N.M.C.9.172	3-phosphoinositide Biosynthesis
C.N.M.C.9.096	Acetyl-CoA Biosynthesis Ⅰ(Pyruvate Dehydrogenase Complex)
C.N.M.C.9.085	Actin Nucleation by ARP-WASP Complex
C.N.M.C.9.174	Aspartate Biosynthesis
C.N.M.C.9.104	BER Pathway
C.N.M.C.9.147	Cell Cycle: G2/M DNA Damage Checkpoint Regulation
C.N.M.C.9.048	Colanic Acid Building Blocks Bisynthesis
C.N.M.C.9.145	Coloretal Cancer Metastasis Signaling
C.N.M.C.9.127	Complement System
C.N.M.C.9.072	DNA Double-Strand Break Repair by Non-Homologous End Joining
C.N.M.C.9.103	DNA Methylation and Transcriptional Repression Signaling
C.N.M.C.9.080	Dopamine Receptor Signaling
C.N.M.C.9.149	Fatty Acid β-oxidation Ⅲ(Unsaturated, Odd Number)
C.N.M.C.9.158	Gai Signaling
C.N.M.C.9.168	Galactose Degradation Ⅰ(Leloir Pathway)
C.N.M.C.9.157	GDP-mannose Biosynthesis
C.N.M.C.9.167	Gliblastma Multiforme Signaling
C.N.M.C.9.047	GNRH Signaling
C.N.M.C.9.014	Granzyme A Signling
C.N.M.C.9.123	Granzyme B Signaling
C.N.M.C.9.150	HGF Signaling
C.N.M.C.9.101	IL-12 Signaling and Production in Macrophages
C.N.M.C.9.141	L-cysteine Degradation Ⅰ
C.N.M.C.9.105	LPS/IL-1 Mediated Inhibition of RXR Function

C.N.M.C.9.165	Lserine Degradation
C.N.M.C.9.111	Mitotic Roles of Polo-like Kinase
C.N.M.C.9.074	Molecular Mechanisms of Cancer
C.N.M.C.9.130	Nucleotide Excision Repair Pathway
C.N.M.C.9.038	Ovarian Cancer Signaling
C.N.M.C.9.139	PAK Signaling
C.N.M.C.9.154	PCP Pathway
C.N.M.C.9.119	Pentose Phosphate Pathway
C.N.M.C.9.140	Pentose Phosphate Pathway (Oxidative Branch)
C.N.M.C.9.132	Phospholipase C Signaling
C.N.M.C.9.040	Protein Kinase A Signaling
C.N.M.C.9.160	Purine Nucleotides De Novo Biosynthesis Ⅱ
C.N.M.C.9.152	Pyrimidine Ribonucleotides De Novo Biosynthesis
C.N.M.C.9.144	Pyrimidine Ribonucleotides Interconversion
C.N.M.C.9.088	Rac Signaling
C.N.M.C.9.097	RAN Signaling
C.N.M.C.9.066	Regulaion of Cellular Mechanics by Calpain Protease
C.N.M.C.9.092	Spliceosomal Cycle
C.N.M.C.9.170	Synaptic Long Term Potentiation
C.N.M.C.9.156	Systemic Lupus Erythematosus Signaling
C.N.M.C.9.065	Thyroid Cancer Signaling
C.N.M.C.9.078	tRNA Charging
C.N.M.C.9.131	Tryptophan Degradation Ⅲ (Eukaryotic)
C.N.M.C.9.171	UDP-N-actyl-D-galactosamine Biosynthesis Ⅱ
**Dataset 8 only**	
C.N.N.C.10.006	MSP-RON signaling pathway
**Dataset 9 only**	
C.N.P.C.11.041	G-protein coupled receptor signaling
C.N.P.C.11.022	Insulin receptor signaling
C.N.P.C.11.027	Prostanoid biosynthesis
