# Supplementary material for: Multiomics-Based Signaling Pathway Network Alterations in Human Non-functional Pituitary Adenomas
Source: Front Endocrinol (Lausanne). 2019 Dec 17;10:835. doi: 10.3389/fendo.2019.00835 (PMC6928143; doi:10.3389/fendo.2019.00835)
Supplement: Supplementary file 1 [file Presentation_1.zip › Supplemental Table 7_v1.pdf]

Supplemental Table 7. Statistical cannonical pathways that were mined from at least two datasests

Code	Dataset.pathway	Canonical pathway name	-log(p-value)
1	2.017	14-3-3-mediated Signaling	1.63
	3.002	14-3-3-mediated signaling	12.9
	9.090	14-3-3-mediated Signaling	2.53
2	1.017	Actin Cytoskeleton Signaling	2.37
	9.016	Actin Cytoskeleton Signaling	6.9
	10.029	Actin cytoskeleton signaling	3.9
3	3.010	Acute Phase Response Signaling	7.4
	9.026	Acute Phase Response Signaling	5.5
	11.029	Acute Phasee response signaling	1.73
4	1.004	Agranulocyte Adhesion and Diapedesis	3.45
	9.169	Agranulocyte Adhesion and Diapedesis	1.3
	10.026	Agranulocyte adhesion and diapedesis	4.1
5	9.073	Agrin Interactions at Neuromuscular Junction	3.13
	10.008	Agrin interactions at neuromuscular junction	5.4
6	2.005	Aldosterone Signaling in Epithelial Cell	2.35
	3.026	Aldosterone Signaling in Epithelial Cells	3.18
	9.013	Aldosterone Signaling in Epithelial Cells	7.3
	11.004	Aldosterone signaling in epithelial cells	2.98
	12.006	Aldosterone signaling in epithelial cells	2.21
7	9.086	AMPK Signaling	2.6
	11.024	AMPK signaling	1.8
	12.019	AMPK signaling	1.75
8	4.020	Amyloid Processing	1.5
	9.122	Amyloid Processing	1.85

	13.008	Amyloid processing	2.2
9	9.126	Amyotrophic Lateral Sclerosis Signaling	1.85
	12.022	Amyotrophic lateral sclerosis signaling	1.63
	13.018	Amyotrophic lateral sclerosis signaling	1.6
10	3.023	Androgen Signaling	3.25
	9.061	Androgen Signaling	3.2
	11.017	Androgen signaling	2.02
11	3.018	Antigen Presentation Pathway	3.55
	9.137	Antigen Presentation Pathway	1.61
12	2.001	AryI hydrocarbon Receptor Signaling	3.7
	9.031	AryI Hydrocarbon Receptor Signaling	5
	3.019	AryI Hydrocarbon Resceptor Signaling	3.5
	11.023	Aryl hydrocarbon receptor signaling	1.82
	12.016	Aryl hydrocarbon receptor signaling	1.77
13	3.050	Aspartate Degradation Ⅱ	2.13
	9.115	Aspartate Degradation Ⅱ	2
14	1.014	Atherosclerosis Signaling	2.51
	9.045	Atherosclerosis Signaling	3.65
15	3.008	Axonal Guidance Signaling	8.8
	9.036	Axonal Guidance Signaling	4
	13.002	Axonal guidance signaling	3.3
16	3.007	Breast Cancer Regulation by Stathmin 1	10.8
	9.089	Breast Cancer Regulation by Stathmin1	2.53
17	1.009	Calcium Signaling	2.85
	9.120	Calcium Signaling	1.9

	10.031	Calcium signaling	2.5
	12.028	Calcium signaling	1.38
18	3.030	Cardiac Hypertrophy Signaling	2.99
	9.059	Cardiac Hypertrophy Signaling	3.3
19	9.106	Cardiac β-adrenergic Signaling	2.1
	12.002	Cardiar beta-adrenergic signaling	2.49
20	3.022	Caveolar-mediated Endocytosis Signaling	3.27
	9.015	Caveolar-mediated Endocytosis Signaling	7
	10.009	Caveolar-mediated endocytosis signaling	5.3
21	9.035	CDK5 Signaling	4
	13.013	CDK5 signaling	1.7
22	3.076	Cellular Effects of Sildenafil (Viagra)	1.46
	9.148	Cellular Effects of Sildenafil (Viagra)	1.5
	10.001	Cellular effects of sildenafil (Viagra)	6.4
23	4.011	Clathrin-mediated Endocytosis Signaling	2.25
	10.005	Clathrin-mediated endocytosis signaling	5.95
	9.010	Clathrin-mediated Endocytosis Signling	8.25
24	3.039	Coagulation System	2.4
	9.173	Coagulation System	1.3
	11.039	Coagulation system	1.36
25	1.031	Corticotropin Releasing Hormone Signaling	1.9
	9.113	Corticotropin Releasing Hormone Signaling	2
	11.018	Corticotropin releasing hormone signaling	2
26	3.064	CREB Signaling in Neurons	1.7
	9.053	CREB Signaling in Neurons	3.6

27	3.081	Crosstalk between Dendritic Cells and Natural Killer Cel	1.35
	10.010	Crosstalk between dentritic cells and natrual killer cel	5.05
28	3.080	CTLA4 Signaling in Cytotoxic T Lymphocytes	1.38
	9.164	CTLA4 Signaling in Cytotoxic T Lymphocytes	1.3
29	1.049	CXCR4 Signaling	1.43
	3.061	CXCR4 Signaling	1.9
	9.099	CXCR4 Signaling	2.3
30	3.031	Death Receptor Signaling	2.9
	10.012	Death receptor signaling	5.01
31	12.010	Docosahexaenoic acid(DHA) signaling	1.98
	1.035	Docosahexaenoic Acid(DHA) Signaling	1.78
32	1.006	Dopamine Degradation	3.1
	9.083	Dopamine Degradation	2.85
33	9.001	EIF2 Signaling	27.8
	11.001	EIF2 signaling	5.45
	12.013	EIF2 signaling	1.9
34	9.129	Endometrial Cancer Signaling	1.72
	11.006	Endometrial cancer signaling	2.65
	12.017	Endometrial cancer signaling	1.75
35	2.022	Endoplasmic Reticulum Stress Pathway	1.37
	3.028	Endoplasmic Reticulum Stress Pathway	3.05
	9.042	Endoplasmic Reticulum Stress Pathway	3.75
	13.023	Endoplasmic reticulum stress pathway	1.37
36	3.082	eNOS Signaling	1.32
	9.076	eNOS Signaling	3

	11.003	eNOS signaling	3.02
	12.001	eNOS signaling	3
	13.026	eNOS signaling	1.34
37	1.029	Ephrin B Signaling	1.99
	3.024	Ephrin B Signaling	3.24
	9.043	Ephrin B Signaling	3.7
38	1.066	Ephrin Receptor Signaling	1.3
	3.043	Ephrin Receptor Signaling	2.28
	9.049	Ephrin Receptor Signaling	3.6
39	1.041	Epithelial Adherens Junction Signaling	1.57
	3.005	Epithelial Adherens Junction Signaling	11.5
	9.017	Epithelial Adherens Junction Signaling	6.75
	10.019	Epthelial adherens junction signaling	4.43
40	1.037	ERK/MAPK Signaling	1.65
	3.070	ERK/MAPK Signaling	1.63
	9.069	ERK/MAPK Signaling	3.15
	2.008	ERK/MAPK Signaling	2.15
	12.014	ERK/MAPK signaling	1.88
41	3.035	ERK5 Signaling	2.6
	9.162	ERK5 Signaling	1.3
42	1.010	Ethanol Degradation Ⅱ	2.8
	3.087	Ethanol Degradation Ⅱ	1.3
	9.030	Ethanol Degradation Ⅱ	5
43	1.020	Ethanol Degradation Ⅳ	2.3
	9.051	Ethanol Degradation Ⅳ	3.6
44	2.021	Extrinsic Prothrombin Activation Pathway	1.44

	3.059	Extrinsic Prothrombin Activation Pathway	1.99
	11.031	Extrinsic prothrombin activation pathway	1.7
	13.022	Extrinsic Prothrombin activation pathway	1.41
45	3.053	FAK Signaling	2.04
	9.093	FAK Signaling	2.5
	10.011	FAK signaling	5.03
46	1.008	Fatty Acid α-oxidation	2.9
	9.063	Fatty Acid α-oxidation	3.18
47	3.048	Fatty Acid β-oxidation Ⅰ	2.14
	9.011	Fatty Acid β-oxidation Ⅰ	7.5
48	3.088	Fcy Receptor-mediated Phagocytosis in Macrophages and Mc	1.3
	10.016	Fcy receptor-mediated phagocytosis in macrophages and mc	4.95
49	1.054	fMLP Signaling in Neutrophils	1.38
	9.143	fMLP Signaling in Neutrophils	1.58
50	3.032	FXR/RXR Activation	2.87
	9.028	FXR/RXR Activation	5.4
	11.021	FXR_RXR activation	1.85
51	3.060	G Beta Gamma Signaling	1.99
	9.054	G Beta Gamma Signaling	3.5
	11.014	G beta gamma signaling	2.15
52	4.005	GABA Receptor Signaling	3.08
	9.151	GABA Receptor Signaling	1.4
53	3.004	Gap Junction Signaling	11.8
	9.067	Gap Junction Signaling	3.15
	10.002	Gap junction signaling	6.15

54	3.003	Germ Cell-Sertoli Cell Junction Signaling	12
	9.039	Germ Cell-Sertoli Cell Junction Signaling	4
	10.021	Germ cell-sertoli cell junction signaling	4.3
	11.026	Germ cell-sertoli cell junction signaling	1.75
55	1.048	Glucocorticoid Receptor Signaling	1.48
	3.036	Glucocorticoid Receptor Signaling	2.59
	9.146	Glucocorticoid Receptor Signaling	1.58
	11.009	Glucocorticoid receptor signaling	2.34
56	9.012	Gluconeogenesis Ⅰ	7.4
	3.015	Gluconeogenesis I	4.28
57	3.062	Glutaryl-CoA Degradation	1.8
	9.046	Glutaryl-CoA Degradation	3.65
58	2.025	Glutathione Redox Reacions Ⅰ	1.3
	9.128	Glutathione Redox Reactions Ⅰ	1.72
59	3.044	Glutathione-mediated Detoxification	2.25
	9.116	Glutathione-mediated Detoxification	2
60	3.021	Glycolysis Ⅰ	3.28
	9.008	Glycolysis Ⅰ	10
	11.043	Glycolysis I	1.3
61	1.011	Growth Hormone Signaling	2.75
	2.009	Growth Hormone Signaling	2
	11.007	Growth hormone signaling	2.42
	12.007	Growth hormone signaling	2.2
62	2.016	Hematopoiesis from Multipotent Stem Cells	1.64
	13.005	Hematopoiesis from pluripotent stem cells	2.2
63	1.025	Hepatic Fibrosis/Hepatic Stellate Cell Activation	2.1

	9.112	Hepatic Fibrosis/Hepatic Stellate Cell Activation	2.1
64	1.057	Hereditary Breast Cancer Signaling	1.36
	4.007	Hereditary Breast Cancer Signaling	2.65
65	3.079	HIPPO Signaling	1.38
	9.161	HIPPO Signaling	1.3
66	1.015	Histamine Degradation	2.45
	9.100	Histamine Degradation	2.3
67	4.012	Huntington’s Disease Signaling	2.06
	9.022	Huntington’s Disease Signaling	5.75
	3.011	Huntington's Disease Signaling	5.1
	11.035	Huntington's disease signaling	1.47
	12.026	Huntington's disease signaling	1.49
	13.001	Huntington's disease signaling	3.75
	12.025	Hypoxia signaling in the cardiavascular system	1.55
68	12.025	Hypoxia signaling in the cardiavascular system	1.55
	9.166	Hypoxia Signaling in the Cardiovascular System	1.3
69	1.040	IGF-1 Signaling	1.58
	2.014	IGF-1 Signaling	1.75
	11.015	IGF-1 signaling	2.15
	12.003	IGF-1 signaling	2.47
	9.114	IGF-1 Signaling	2
70	3.056	IL-1 Signaling	2
	4.006	IL-1 Signaling	2.85
	9.060	IL-1 Signaling	3.2
71	9.023	ILK Signaling	5.6
	10.025	ILK signaling	4.1

	12.012	ILK signaling	1.9
72	9.118	Inhibition of Matrix Metalloproteases	2
	13.004	Inhibition of matrix metalloproteases	2.4
73	3.075	Integrin Signaling	1.47
	9.020	Integrin Signaling	6.15
	10.027	Integrin signaling	4
74	3.072	Intrinsic Prothrombin Activation Pathway	1.58
	9.084	Intrinsic Prothrombin Activation Pathway	2.85
	11.036	Intrinsic prothrombin activation pathway	1.45
75	9.050	Isoleucine Degradation Ⅰ	3.6
	3.033	Isoleucne Degradation Ⅰ	2.8
76	9.133	Ketolysis	1.64
	13.025	Ketolysis	1.35
77	11.008	Leptin signaling in obestiy	2.35
	4.027	Leptin signaling in obesty	1.32
	9.091	Leptin Signaling Obesity	2.5
78	1.045	Leukocyte Extravasation Signaling	1.51
	9.071	Leukocyte Extravasation Signaling	3.14
	10.028	Leukocyte extravasation signaling	4
79	3.067	Lipid Antigen Presentation by CD1	1.68
	9.068	Lipid Antigen Presentation by CD1	3.15
80	3.012	LXR/RXR Activation	4.8
	9.021	LXR/RXR Activation	6
	11.019	LXR_RXR activation	1.96
81	3.085	Mechanisms of Viral Exit from Host Cells	1.3

	9.153	Mechanisms of Viral Exit from Host Cells	1.4
	10.003	Mechanisms of viral exit from host cells	6.13
82	4.028	Melanocyte development and pigmentation signaling	1.3
	9.107	Melanocyte Development and Pigmentation Signaling	2.1
	11.010	Melanocyte development and pigmentation signaling	2.25
83	3.073	Melatonin Signaling	1.5
	4.025	Melatonin signaling	1.33
	9.110	Melatonin Signaling	2.1
84	3.029	Methylglyoxal Degradation Ⅲ	3.01
	9.159	Methylglyoxal Degradation Ⅲ	1.4
	2.024	Methylglyoxal Degradation Ⅲ	1.35
85	2.002	Mitochondrial Dysfunction	3.22
	3.014	Mitochondrial Dysfunction	4.3
	9.003	Mitochondrial Dysfunction	18
	13.003	Mitochondrial Dysfunction	3.15
86	9.018	mTOR Signaling	6.75
	11.034	mTOR signaling	1.62
	12.004	mTOR signaling	2.42
87	9.082	Neuregulin Signaling	2.85
	13.014	Neuregulin signaling	1.7
	11.013	Neurigulin signaling	2.2
88	4.017	Neuroprotective Role of THOP1 in Alzheimer’s Disease	1.55
	9.136	Neuroprotective Role of THOP1 in Alzheimer’s Disease	1.64
89	12.005	Nitric oxide signaling in cardiavascular system	2.25
	11.016	Nitric oxide signaling in cardiovascular system	2.05
	1.050	Nitric Oxide Signaling in the Cardiovascular System	1.42
	9.135	Nitric Oxide Signaling in the Cardiovascular System	1.64

	10.033	Nitric oxide signaling in the cardiovascular system	1.3
90	1.003	Noradrenaline and Adrenaline Degradation	3.5
	9.037	Noradrenaline and Adrenaline Degradation	4
91	2.006	NRF2-mediated Oxidative Stress Response	2.22
	3.009	NRF2-mediated Oxidative Stress Response	8.1
	9.006	NRF2-mediated Oxidative Stress Response	11.75
	10.024	NRF2-mediated oxidative stress response	4.15
92	1.016	Oxidative Ethanol Degradation Ⅲ	2.4
	9.075	Oxidative Ethanol Degradation Ⅲ	3.13
93	2.003	Oxidative Phosphorylation	2.72
	3.066	Oxidative Phosphorylation	1.69
	9.005	Oxidative Phosphorylation	11.9
94	9.124	P2Y Purigenic Receptor Signaling Pahway	1.85
	3.071	P2Y Purigenic Receptor Signaling Pathway	1.6
95	1.021	p53 Signaling	2.25
	12.018	p53 signaling	1.75
96	3.046	p70S6K Signaling	2.19
	9.064	p70S6K Signaling	3.15
97	9.095	Paxillin Signaling	2.4
	10.017	Paxillin signaling	4.9
98	1.062	PEDF Signaling	1.32
	12.027	PEDF signaling	1.45
99	1.065	Phenylalanine Degradation Ⅳ (Mammalian, via Side Chain)	1.3
	9.163	Phenylalanine Degradation Ⅳ(Mammalian, via Side Chain)	1.3

100	3.047	PI3K/AKT Signaling	2.15
	9.055	PI3K/AKT Signaling	3.5
	2.019	PI3K/AKT Sigaling	1.52
	11.020	PI3K_AKT signaling	1.95
	12.009	PI3K-AKT signaling	2
101	1.042	Polyamine Regulation in Colon Cancer	1.56
	3.063	Polyamine Regulation in Colon Cancer	1.75
102	11.005	PPARa/RXRa activation	2.8
	3.068	PPARα/RXRα Activation	1.65
	9.057	PPARα/RXRα Activation	3.4
	2.007	PPARα/RXRα Activation	2.21
103	9.155	Primary Immunodeficiency Signaling	1.4
	13.007	Primary immunodeficiency signaling	2.2
104	3.069	Production of Nitric Oxide and Reactive Oxygen Species i	1.64
	9.102	Production of Nitric Oxide and Reactive Oxygen Species i	2.2
105	9.108	Prostate Cancer Signaling	2.1
	11.011	Prostate cancer signaling	2.25
	12.011	Prostate cancer signaling	1.95
106	2.012	Protein Ubiquitination Pathay	1.78
	3.016	Protein Ubiquitination Pathway	4
	4.003	Protein Ubiquitination Pathway	3.35
	9.002	Protein Ubiquitination Pathway	19.65
	10.032	Protein ubiquitination pathway	2.2
	11.038	Protein ubiquitination pathway	1.39
	12.030	Protein ubiquitination pathway	1.3
107	1.001	Putrescine Degradation Ⅲ	4.75
	9.079	Putrescine Degradation Ⅲ	2.9

108	9.121	PXR/RXR Activation	1.9
	4.023	PXR/RXR activation	1.35
109	9.098	RAR Activation	2.3
	11.030	RAR activation	1.7
110	3.084	Regulation of Actin-based Motility by Rho	1.31
	9.027	Regulation of Actin-based Motility by Rho	5.5
	10.013	Regulation of actin-based motility by Rho	5
111	9.004	Regulation of eIF4 and p70S6K Signaling	13.75
	11.025	Regulation of eIF4 and p70S6K signaling	1.8
112	3.078	Relaxin Signaling	1.43
	9.081	Relaxin Signaling	2.85
113	9.007	Remodeling of Epithelial Adherences Junctions	11
	3.001	Remodeling of epithelial adherens junctions	15.1
	10.007	Remodeling of epthelial adherens junctions	5.45
114	4.009	RhoA Signaling	2.6
	9.025	RhoA Signaling	5.5
	10.018	RhoA signaling	4.67
115	3.013	RhoGDI Signaling	4.5
	9.024	RhoGDI Signaling	5.6
	10.023	RhoGDI signaling	4.15
116	2.004	Role of JAK2 in Hormone-like Cytokine Signaling	2.65
	11.040	Role of JAK2 in hormone-like cytokine signaling	1.35
117	1.019	Role of NFAT in Regulation of the Immune Response	2.33
	3.042	Role of NFAT in Regulation of the Immune Response	2.3

118	3.038	Role of Tissue Factor in Cancer	2.5
	9.094	Role of Tissue Factor in Cancer	2.5
	2.015	Role of Tissue Factor in Cancer	1.65
119	1.012	Serotonin Degradation	2.6
	9.070	Serotonin Degradation	3.15
120	3.006	Sertoli Cell-Sertoli Cell Junction Signaling	11.1
	9.058	Sertoli Cell-Sertoli Cell Junction Signaling	3.4
	10.004	Sertoli cell-sertoli cell junction signaling	5.97
	11.032	Sertoli cell-sertoli cell junction signaling	1.7
121	3.045	Signaling by Rho Family GTPases	2.2
	9.029	Signaling by Rho Family GTPases	5.3
	10.030	Signaling by Rho family GTPases	3.8
122	4.014	Sonic Hedgehog Signaling	1.75
	9.087	Sonic Hedgehog Signaling	2.6
	11.037	Sonic hedgehog signaling	1.43
123	9.142	Sucrose Degradation Ⅴ(Mammalian)	1.58
	11.033	Sucrose degradation V (mammalian)	1.65
124	3.034	Superoxide Radicals Degradation	2.75
	9.117	Superoxide Radicals Degradation	2
	13.011	Superoxide radicals degradation	1.75
125	3.058	Synaptic Long Term Depression	1.99
	9.138	Synaptic Long Term Depression	1.58
126	9.019	TCA Cycle Ⅱ(Eukaryotic)	6.2
	3.020	TCA CycleⅡ（Eukaryotic）	3.3

127	2.023	Tec Kinase Signaling	1.36
	3.025	Tec Kinase Signaling	3.2
	10.020	Tec kinase signaling	4.3
128	9.056	Telomerase Signaling	3.5
	11.002	Telomerase signaling	3.6
	12.020	Telomerase signaling	1.74
129	3.051	Telomere Extension by Telomerase	2.07
	9.077	Telomere Extension by Telomerase	3
130	3.052	Thrombin Signaling	2.05
	9.134	Thrombin Signaling	1.64
131	1.052	Tight Junction Signaling	1.39
	3.027	Tight Junction Signaling	3.12
	9.062	Tight Junction Signaling	3.2
	10.022	Tight junction signaling	4.25
	11.028	Tight junction signaling	1.75
132	13.012	TR RXR activation	1.74
	9.041	TR/RXR Actiation	3.75
	1.018	TR/RXR Activation	2.35
	3.055	TR/RXR Activation	2.01
	11.012	TR/RXR activation	2.2
	2.011	TR/RXR Activation	1.8
133	9.044	Tryptophan Degradation X(Mammalian, via Tryptamine)	3.7
	1.002	Tryptophan Degradation X (Mammalian via Tryptamine)	3.6
134	1.005	Unfolded protein response	3.4
	3.017	Unfolded Protein Response	3.99
	9.009	Unfolded protein response	8.7
135	3.077	Valine Degradation Ⅰ	1.45

	9.034	Valine Degradation Ⅰ	4.35
136	9.052	VEGF Signaling	3.6
	10.015	VEGF signaling	4.98
137	3.057	Virus Entry via Endocytic Pathways	2
	9.033	Virus Entry via Endocytic Pathways	4.75
	10.014	Virus entry via endocytic pathways	5
	12.015	Virus entry via endocytic pathways	1.8
138	3.037	Xenobiotic Metabolism Signaling	2.55
	9.032	Xenobiotic Metabolism Signaling	4.75
	11.042	Xenobiotic metabolism signaling	1.32
139	1.038	α-Adrenergic Signaling	1.6
	3.086	α-Adrenergic Signaling	1.3
	9.109	α-Adrenergic Signaling	2.1
