# Supplementary material for: Multiomics-Based Signaling Pathway Network Alterations in Human Non-functional Pituitary Adenomas
Source: Front Endocrinol (Lausanne). 2019 Dec 17;10:835. doi: 10.3389/fendo.2019.00835 (PMC6928143; doi:10.3389/fendo.2019.00835)
Supplement: Supplementary file 1 [file Presentation_1.zip › Supplemental materials 1.1.pdf]

Supplemental materials 1.1 Differentially expressed genes between NFPAs and controls for IPA analysis (Dataset 1).

Fold-Change	ID	Notes	Molecules	Description	Location	Function
3.270	AB028985		ABCA2	ATP-binding cassette, sub-family A (ABC1), member 2	Plasma Membrane	transporter
2.180	AB020650		ABLIM3	actin binding LIM protein family, member 3	Cytoplasm	other
2.130	N48190		ADAM22	ADAM metallopeptidase domain 22	Plasma Membrane	peptidase
2.510	AF052115		ADAM23	ADAM metallopeptidase domain 23	Plasma Membrane	peptidase
-7.420	D14874		ADM	adrenomedullin	Extracellular Space	other
-2.700	AF038451		AGR2	anterior gradient 2	Extracellular Space	other
5.070	AF052143		AJAP1	adherens junctions associated protein 1	Plasma Membrane	other
-4.830	K03000		ALDH1A1	aldehyde dehydrogenase 1 family, member A1	Cytoplasm	enzyme
-3.940	AB015228		ALDH1A2	aldehyde dehydrogenase 1 family, member A2	Cytoplasm	enzyme
-3.200	X05409		ALDH2	aldehyde dehydrogenase 2 family (mitochondrial)	Cytoplasm	enzyme
-2.360	AB028994		AMOT	angiomotin	Plasma Membrane	other
-5.250	D13628		ANGPT1	angiopoietin 1	Extracellular Space	growth factor
2.760	X81896		ANO3	anoctamin 3	Plasma Membrane	transporter

-2.600	M62895	ANXA2P3	annexin A2 pseudogene 3	Other	other
-3.460	U81504	AP3B1	adaptor-related protein complex 3, beta 1 subunit	Plasma Membrane	transporter
-6.630	AL049471	ARID5B	AT rich interactive domain 5B (MRF1-like)	Nucleus	transcription regulator
-116.820	L08424	ASCL1	achaete-scute family bHLH transcription factor 1	Nucleus	transcription regulator
2.810	AB014534	ASTN2	astrotactin 2	Cytoplasm	other
-6.840	U51478	ATP1B3	ATPase, Na+/K+ transporting, beta 3 polypeptide	Plasma Membrane	transporter
2.680	M23115	ATP2A2	ATPase, Ca++ transporting, cardiac muscle, slow twitch 2	Cytoplasm	transporter
2.610	AF070606	ATP2B1	ATPase, Ca++ transporting, plasma membrane 1	Plasma Membrane	transporter
2.110	AF035315	ATP8A1	ATPase, aminophospholipid transporter (APLT), class I, type 8A, member 1	Cytoplasm	transporter
2.230	AB005298	BAI2	brain-specific angiogenesis inhibitor 2	Plasma Membrane	G-protein coupled receptor
-3.070	AF082868	BBOX1	butyrobetaine (gamma), 2-oxoglutarate dioxygenase (gamma-butyrobetaine hydroxylase) 1	Cytoplasm	enzyme
-2.710	M14745	BCL2	B-cell CLL/lymphoma 2	Cytoplasm	transporter
-5.210	AB004066	BHLHE40	basic helix-loop-helix family, member e40	Nucleus	transcription regulator
-2.100	U39817	BLM	Bloom syndrome, RecQ helicase-like	Nucleus	enzyme
-3.850	AF002697	BNIP3	BCL2/adenovirus E1B 19kDa interacting protein 3	Cytoplasm	other

2.290	AF052149		BSCL2	Berardinelli-Seip congenital lipodystrophy 2 (seipin)	Cytoplasm	other
-12.780	U72649		BTG2	BTG family, member 2	Nucleus	transcription regulator
-2.870	AL049382		C12orf29	chromosome 12 open reading frame 29	Other	other
3.270	AI951798		CADM3	cell adhesion molecule 3	Plasma Membrane	other
-4.050	M64110		CALD1	caldesmon 1	Cytoplasm	other
4.850	W28510		CALM1 (includes others)	calmodulin 1 (phosphorylase kinase, delta)	Cytoplasm	other
4.090	AL080169		CCDC69	coiled-coil domain containing 69	Other	other
-18.670	M26683		CCL2	chemokine (C-C motif) ligand 2	Extracellular Space	cytokine
2.220	X05323		CD200	CD200 molecule	Plasma Membrane	other
-3.150	M31516		CD55	CD55 molecule, decay accelerating factor for complement (Cromer blood group)	Plasma Membrane	other
-4.830	M16279		CD99	CD99 molecule	Plasma Membrane	other
3.230	M34064	D	CDH2	cadherin 2, type 1, N-cadherin (neuronal)	Plasma Membrane	other
2.330	M34064	D	CDH2	cadherin 2, type 1, N-cadherin (neuronal)	Plasma Membrane	other
14.070	AA535884		CDK18	cyclin-dependent kinase 18	Cytoplasm	kinase
-2.530	M63256		CDR2	cerebellar degeneration-related protein 2, 62kDa	Cytoplasm	other

-14.100	M83667	CEBPD	CCAAT/enhancer binding protein (C/EBP), delta	Nucleus	transcription regulator
-13.640	L14813	CELP	carboxyl ester lipase pseudogene	Other	other
-7.530	S70585	CGA	glycoprotein hormones, alpha polypeptide	Extracellular Space	other
-5.080	J00117	CGB (includes others)	chorionic gonadotropin, beta polypeptide	Extracellular Space	other
-6.270	K03183	CGB7	chorionic gonadotropin, beta polypeptide 7	Extracellular Space	other
-2.880	X15334	CKB	creatine kinase, brain	Cytoplasm	kinase
6.880	Z30643	CLCNKA	chloride channel, voltage-sensitive Ka	Plasma Membrane	ion channel
-2.470	AB000714	CLDN3	claudin 3	Plasma Membrane	transmembrane receptor
-2.930	D86322	CLGN	calmegin	Cytoplasm	peptidase
2.690	N99340	CLIP3	CAP-GLY domain containing linker protein 3	Plasma Membrane	other
-2.140	AF104398	CNIH1	cornichon family AMPA receptor auxiliary protein 1	Plasma Membrane	other
-3.740	S80562	CNN3	calponin 3, acidic	Cytoplasm	other
13.820	L10347	COL2A1	collagen, type II, alpha 1	Extracellular Space	other
-2.170	M58526	COL4A5	collagen, type IV, alpha 5	Extracellular Space	other
4.150	AB011156	COLGALT2	collagen beta(1-O)galactosyltransferase 2	Cytoplasm	other

3.040	S74445		CRABP1	cellular retinoic acid binding protein 1	Cytoplasm	transporter
-2.920	AL080209		CREB3L2	cAMP responsive element binding protein 3-like 2	Nucleus	other
-7.290	S68134	D	CREM	cAMP responsive element modulator	Nucleus	transcription regulator
-8.920	S68134	D	CREM	cAMP responsive element modulator	Nucleus	transcription regulator
-20.330	S68271	D	CREM	cAMP responsive element modulator	Nucleus	transcription regulator
2.850	AF059274		CSPG5	chondroitin sulfate proteoglycan 5 (neuroglycan C)	Extracellular Space	growth factor
2.150	AB001928		CTSV	cathepsin V	Cytoplasm	peptidase
2.240	U84487		CX3CL1	chemokine (C-X3-C motif) ligand 1	Extracellular Space	cytokine
-16.360	L06797		CXCR4	chemokine (C-X-C motif) receptor 4	Plasma Membrane	G-protein coupled receptor
-2.420	U66042		CXorf40A/CXorf40B	chromosome X open reading frame 40A	Other	other
-2.330	AF091084		CYB5R1	cytochrome b5 reductase 1	Cytoplasm	enzyme
-2.360	L47738		CYFIP2	cytoplasmic FMR1 interacting protein 2	Cytoplasm	other
4.120	M14565		CYP11A1	cytochrome P450, family 11, subfamily A, polypeptide 1	Cytoplasm	enzyme
-4.560	X76105		DAP	death-associated protein	Cytoplasm	transcription regulator
-4.880	AB002367		DCLK1	doublecortin-like kinase 1	Other	kinase

6.270	AJ003112		DCX	doublecortin	Cytoplasm	other
-3.130	AA522530		DDIT4	DNA-damage-inducible transcript 4	Cytoplasm	other
2.190	AB014545		DEPDC5	DEP domain containing 5	Cytoplasm	other
2.840	U31875		DHRS2	dehydrogenase/reductase (SDR family) member 2	Nucleus	enzyme
-3.310	AB011155		DLG5	discs, large homolog 5 (Drosophila)	Plasma Membrane	other
-917.430	U15979		DLK1	delta-like 1 homolog (Drosophila)	Extracellular Space	other
2.320	AB020627		DNM3	dynamin 3	Cytoplasm	enzyme
2.040	AB002297		DOCK3	dedicator of cytokinesis 3	Cytoplasm	other
4.610	D78014		DPYSL3	dihydropyrimidinase-like 3	Cytoplasm	enzyme
-2.520	U48807		DUSP4	dual specificity phosphatase 4	Nucleus	phosphatase
2.420	U66406		EFNB3	ephrin-B3	Plasma Membrane	kinase
4.520	X51956		ENO2	enolase 2 (gamma, neuronal)	Cytoplasm	enzyme
-2.820	X89602	D	ENOSF1	enolase superfamily member 1	Other	enzyme
-4.690	X67098	D	ENOSF1	enolase superfamily member 1	Other	enzyme
15.090	D83492		EPHB6	EPH receptor B6	Plasma Membrane	kinase

3.730	U12535		EPS8	epidermal growth factor receptor pathway substrate 8	Plasma Membrane	peptidase
-4.070	AF070641		ETV1	ets variant 1	Nucleus	transcription regulator
-2.590	X96381		ETV5	ets variant 5	Nucleus	transcription regulator
-9.150	X51521		EZR	ezrin	Plasma Membrane	other
-7.330	J02931		F3	coagulation factor III (thromboplastin, tissue factor)	Plasma Membrane	transmembrane receptor
3.070	AL050118		FADS2	fatty acid desaturase 2	Plasma Membrane	enzyme
2.630	AB023167		FAIM2	Fas apoptotic inhibitory molecule 2	Plasma Membrane	other
-5.180	W87466		FAM114A1	family with sequence similarity 114, member A1	Extracellular Space	other
5.470	AL050367		FAM171A1	family with sequence similarity 171, member A1	Cytoplasm	other
-9.480	U29344		FASN	fatty acid synthase	Cytoplasm	enzyme
2.430	X63556		FBN1	fibrillin 1	Extracellular Space	other
4.270	X66945	D	FGFR1	fibroblast growth factor receptor 1	Plasma Membrane	kinase
2.640	M34641	D	FGFR1	fibroblast growth factor receptor 1	Plasma Membrane	kinase
2.580	M34641	D	FGFR1	fibroblast growth factor receptor 1	Plasma Membrane	kinase
2.770	U53445		FILIP1L	filamin A interacting protein 1-like	Nucleus	other

5.660	U78793		FOLR1	folate receptor 1 (adult)	Plasma Membrane	transporter
-2.850	D00017		formaldehyde		Other	chemical - endogenous mammalian
-15.900	V01512	D	FOS	FBJ murine osteosarcoma viral oncogene homolog	Nucleus	transcription regulator
-18.460	V01512	D	FOS	FBJ murine osteosarcoma viral oncogene homolog	Nucleus	transcription regulator
-5.790	AF032885		FOXO1	forkhead box O1	Nucleus	transcription regulator
3.510	AB023230		FRMD4B	FERM domain containing 4B	Cytoplasm	other
6.590	AL080093		FUT9	fucosyltransferase 9 (alpha (1,3) fucosyltransferase)	Cytoplasm	enzyme
-6.240	AF078077		GADD45B	growth arrest and DNA-damage-inducible, beta	Cytoplasm	other
-487.800	M77140		GAL	galanin/GMAP prepropeptide	Extracellular Space	other
-3.620	L13698		GAS1	growth arrest-specific 1	Plasma Membrane	other
-9.040	Y07846		GAS2L1	growth arrest-specific 2 like 1	Cytoplasm	other
14.550	M68891	D	GATA2	GATA binding protein 2	Nucleus	transcription regulator
9.020	M68891	D	GATA2	GATA binding protein 2	Nucleus	transcription regulator
12.760	X58072		GATA3	GATA binding protein 3	Nucleus	transcription regulator
-5000.000	V00520		GH1	growth hormone 1	Extracellular Space	growth factor

-307.690	K00470	D	GH2	growth hormone 2	Extracellular Space	other
-578.030	J03756	D	GH2	growth hormone 2	Extracellular Space	other
2.580	AB020643		GLCE	glucuronic acid epimerase	Cytoplasm	enzyme
3.860	M31328	D	GNB3	guanine nucleotide binding protein (G protein), beta polypeptide 3	Plasma Membrane	enzyme
3.210	M31328	D	GNB3	guanine nucleotide binding protein (G protein), beta polypeptide 3	Plasma Membrane	enzyme
-2.410	AF030186		GPC4	glypican 4	Plasma Membrane	transmembrane receptor
-3.310	D32257		GTF3A	general transcription factor IIIA	Nucleus	transcription regulator
-15.630	V00505		HBD	hemoglobin, delta	Other	transporter
-3.830	W27949		HEBP2	heme binding protein 2	Cytoplasm	other
-7.660	L19314		HES1	hes family bHLH transcription factor 1	Nucleus	transcription regulator
-3.540	AB014555		HIP1R	huntingtin interacting protein 1 related	Cytoplasm	other
2.340	M95585		HLF	hepatic leukemia factor	Nucleus	transcription regulator
-5.070	L26336		HSPA2	heat shock 70kDa protein 2	Cytoplasm	other
-9.730	X77956		ID1	inhibitor of DNA binding 1, dominant negative helix-loop-helix protein	Nucleus	transcription regulator
-6.300	D13891		ID2	inhibitor of DNA binding 2, dominant negative helix-loop-helix protein	Nucleus	transcription regulator

13.100	AF020038		IDH1	isocitrate dehydrogenase 1 (NADP+), soluble	Cytoplasm	enzyme
-2.800	X17025		IDI1	isopentenyl-diphosphate delta isomerase 1	Cytoplasm	enzyme
-5.530	M62831		IER2	immediate early response 2	Cytoplasm	other
2.750	D28915		IFI44	interferon-induced protein 44	Cytoplasm	other
-2.590	M35878	D	IGFBP3	insulin-like growth factor binding protein 3	Extracellular Space	other
-3.350	M35878	D	IGFBP3	insulin-like growth factor binding protein 3	Extracellular Space	other
-5.020	AF014398		IMPA2	inositol(myo)-1(or 4)-monophosphatase 2	Cytoplasm	phosphatase
-5.590	AB023183		INPP5F	inositol polyphosphate-5-phosphatase F	Other	phosphatase
-2.250	AI674208		IQCK	IQ motif containing K	Other	other
2.910	D26350	D	ITPR2	inositol 1,4,5-trisphosphate receptor, type 2	Cytoplasm	ion channel
2.850	D26350	D	ITPR2	inositol 1,4,5-trisphosphate receptor, type 2	Cytoplasm	ion channel
-6.120	X51345		JUNB	jun B proto-oncogene	Nucleus	transcription regulator
5.870	M55513		KCNA5	potassium voltage-gated channel, shaker-related subfamily, member 5	Plasma Membrane	ion channel
5.080	AF006823		KCNK3	potassium channel, subfamily K, member 3	Plasma Membrane	ion channel
-2.330	AL035081		KDELR3	KDEL (Lys-Asp-Glu-Leu) endoplasmic reticulum protein retention receptor 3	Cytoplasm	transporter

2.150	U10991	KIAA1549L	KIAA1549-like	Cytoplasm	other
-3.340	AB011103	KIF5C	kinesin family member 5C	Cytoplasm	other
-2.370	AB012917	KLK11	kallikrein-related peptidase 11	Extracellular Space	peptidase
-6.230	AB006780	LGALS3	lectin, galactoside-binding, soluble, 3	Extracellular Space	other
-12.960	AI051683	LHB	luteinizing hormone beta polypeptide	Extracellular Space	other
-3.390	AL120815	LITAF	lipopolysaccharide-induced TNF factor	Nucleus	transcription regulator
-3.770	U24576	LMO4	LIM domain only 4	Nucleus	transcription regulator
3.060	U24389	LOXL1	lysyl oxidase-like 1	Extracellular Space	enzyme
3.750	AB018311	LPHN3	latrophilin 3	Plasma Membrane	G-protein coupled receptor
-3.700	AL039458	LRIG1	leucine-rich repeats and immunoglobulin-like domains 1	Extracellular Space	other
3.130	M69177	MAOB	monoamine oxidase B	Cytoplasm	enzyme
2.110	Y09836	MAP1B	microtubule-associated protein 1B	Cytoplasm	other
3.880	D85131	MAZ	MYC-associated zinc finger protein (purine-binding transcription factor)	Nucleus	transcription regulator
-6.660	AF052114	MEG3	maternally expressed 3 (non-protein coding)	Other	other
-8.660	AL050159	METTL7A	methyltransferase like 7A	Cytoplasm	other

-10.660	AB014526		MFAP3L	microfibrillar-associated protein 3-like	Other	other
-6.080	D28124		MINOS1-NBL1/NBL1	neuroblastoma 1, DAN family BMP antagonist	Nucleus	other
3.300	U16954		MLLT11	myeloid/lymphoid or mixed-lineage leukemia (trithorax homolog, Drosophila); translocated to, 11	Cytoplasm	other
-4.510	Z98946		MSN	moesin	Plasma Membrane	other
-15.320	X16396		MTHFD2	methylenetetrahydrofolate dehydrogenase (NADP+ dependent) 2, methenyltetrahydrofolate cyclohydrolase	Cytoplasm	enzyme
4.320	AF013570		MYH11	myosin, heavy chain 11, smooth muscle	Cytoplasm	other
-7.460	AB018342		MYO10	myosin X	Cytoplasm	other
2.110	AB002361		NACAD	NAC alpha domain containing	Other	other
3.130	AB011179	D	NCDN	neurochondrin	Cytoplasm	other
2.240	AB011179	D	NCDN	neurochondrin	Cytoplasm	other
3.810	D82347		NEUROD1	neuronal differentiation 1	Nucleus	transcription regulator
2.250	AF054995		NFASC	neurofascin	Plasma Membrane	other
-9.730	X64318		NFIL3	nuclear factor, interleukin 3 regulated	Nucleus	transcription regulator
2.280	AB028993		NLGN1	neuroligin 1	Plasma Membrane	enzyme
-5.640	U31767		NNAT	neuronatin	Plasma Membrane	transporter

4.260	U97669		NOTCH3	notch 3	Plasma Membrane	transcription regulator
-2.680	AI743090		NPAS2	neuronal PAS domain protein 2	Nucleus	transcription regulator
-40.100	U29195		NPTX2	neuronal pentraxin II	Extracellular Space	other
-2.740	L13740		NR4A1	nuclear receptor subfamily 4, group A, member 1	Nucleus	ligand-dependent nuclear receptor
-10.620	X75918		NR4A2	nuclear receptor subfamily 4, group A, member 2	Nucleus	ligand-dependent nuclear receptor
-6.580	X16277	D	ODC1	ornithine decarboxylase 1	Cytoplasm	enzyme
-7.630	M33764	D	ODC1	ornithine decarboxylase 1	Cytoplasm	enzyme
-3.690	D82343	D	OLFM1	olfactomedin 1	Cytoplasm	other
-4.400	U79299	D	OLFM1	olfactomedin 1	Cytoplasm	other
-2.550	J02783	D	P4HB	prolyl 4-hydroxylase, beta polypeptide	Cytoplasm	enzyme
-2.780	M22806	D	P4HB	prolyl 4-hydroxylase, beta polypeptide	Cytoplasm	enzyme
2.010	X59841		PBX3	pre-B-cell leukemia homeobox 3	Nucleus	transcription regulator
-2.930	D25547		PCMT1	protein-L-isoaspartate (D-aspartate) O-methyltransferase	Cytoplasm	enzyme
2.260	Y11312		PIK3C2B	phosphatidylinositol-4-phosphate 3-kinase, catalytic subunit type 2 beta	Cytoplasm	kinase
2.920	U70370		PITX1	paired-like homeodomain 1	Nucleus	transcription regulator

12.440	AF048722		PITX2	paired-like homeodomain 2	Nucleus	transcription regulator
5.730	M21056		PLA2G1B	phospholipase A2, group IB (pancreas)	Extracellular Space	enzyme
2.440	AF058921		PLA2G4C	phospholipase A2, group IVC (cytosolic, calcium-independent)	Plasma Membrane	enzyme
-29.750	U81992		PLAGL1	pleiomorphic adenoma gene-like 1	Nucleus	transcription regulator
-5.710	D90070		PMAIP1	phorbol-12-myristate-13-acetate-induced protein 1	Cytoplasm	other
-1666.670	V01510		POMC	proopiomelanocortin	Extracellular Space	other
-12.500	L48516		PON3	paraoxonase 3	Extracellular Space	enzyme
-3.010	S90469		POR	P450 (cytochrome) oxidoreductase	Cytoplasm	enzyme
-2.970	U25182		PRDX4	peroxiredoxin 4	Cytoplasm	enzyme
-100.810	M29386		PRL	prolactin	Extracellular Space	cytokine
-2.600	M57399	D	PTN	pleiotrophin	Extracellular Space	growth factor
-3.710	M57399	D	PTN	pleiotrophin	Extracellular Space	growth factor
6.330	Y00062		PTPRC	protein tyrosine phosphatase, receptor type, C	Plasma Membrane	phosphatase
-13.810	L77886		PTPRK	protein tyrosine phosphatase, receptor type, K	Plasma Membrane	phosphatase
10.160	AL050071		PVRL3	poliovirus receptor-related 3	Plasma Membrane	other

-2.990	U77594	RARRES2	retinoic acid receptor responder (tazarotene induced) 2	Plasma Membrane	transmembrane receptor
2.000	AB011110	RASA4	RAS p21 protein activator 4	Cytoplasm	other
-2.220	U85267	RCAN1	regulator of calcineurin 1	Nucleus	transcription regulator
3.300	D83407	RCAN2	regulator of calcineurin 2	Other	other
-10.200	U70426	RGS16	regulator of G-protein signaling 16	Cytoplasm	other
-2.280	L13463	RGS2	regulator of G-protein signaling 2	Nucleus	other
3.630	AB008109	RGS5	regulator of G-protein signaling 5	Plasma Membrane	other
-2.120	D23660	RPL4	ribosomal protein L4	Cytoplasm	enzyme
-2.260	M17885	RPLP0	ribosomal protein, large, P0	Cytoplasm	other
-2.880	M81757	RPS19	ribosomal protein S19	Cytoplasm	other
-2.780	AF006751	RRBP1	ribosome binding protein 1	Cytoplasm	other
2.020	AI825798	RUNDC3B	RUN domain containing 3B	Other	other
-5.560	AL050290	SAT1	spermidine/spermine N1-acetyltransferase 1	Cytoplasm	enzyme
-2.290	AF047442	SEC22B	SEC22 vesicle trafficking protein homolog B (S. cerevisiae) (gene/pseudogene)	Cytoplasm	other
-2.450	AF054184	SEC61G	Sec61 gamma subunit	Plasma Membrane	transporter

-2.790	M25280		SELL	selectin L	Plasma Membrane	transmembrane receptor
-8.140	AF009314		SEMA5A	sema domain, seven thrombospondin repeats (type 1 and type 1-like), transmembrane domain (TM) and short cytoplasmic domain, (semaphorin) 5A	Plasma Membrane	transmembrane receptor
3.600	U59632		SEPT5-GP1BB	SEPT5-GP1BB readthrough	Other	other
-3.380	AI557272		SERP1	stress-associated endoplasmic reticulum protein 1	Cytoplasm	other
7.620	AL050253	D	SEZ6L	seizure related 6 homolog (mouse)-like	Plasma Membrane	other
5.540	AB023144	D	SEZ6L	seizure related 6 homolog (mouse)-like	Plasma Membrane	other
6.160	AF056087		SFRP1	secreted frizzled-related protein 1	Plasma Membrane	transmembrane receptor
5.720	AF036271		SH3GL3	SH3-domain GRB2-like 3	Cytoplasm	other
-4.670	AJ012611		SIX3	SIX homeobox 3	Nucleus	transcription regulator
3.290	U79245		SLC12A5	solute carrier family 12 (potassium/chloride transporter), member 5	Plasma Membrane	transporter
2.280	AB007448		SLC22A4	solute carrier family 22 (organic cation/zwitterion transporter), member 4	Plasma Membrane	transporter
2.340	D87075		SLC23A2	solute carrier family 23 (ascorbic acid transporter), member 2	Plasma Membrane	transporter
-4.530	AL049963		SLC39A8	solute carrier family 39 (zinc transporter), member 8	Extracellular Space	transporter
3.040	AB029010		SLC8A2	solute carrier family 8 (sodium/calcium exchanger), member 2	Cytoplasm	transporter
3.000	U66619	D	SMARCD3	SWI/SNF related, matrix associated, actin dependent regulator of chromatin, subfamily d, member 3	Nucleus	transcription regulator

2.560	U66619	D	SMARCD3	SWI/SNF related, matrix associated, actin dependent regulator of chromatin, subfamily d, member 3	Nucleus	transcription regulator
-2.130	Y08136		SMPDL3A	sphingomyelin phosphodiesterase, acid-like 3A	Extracellular Space	enzyme
-2.800	U44754		SNAPC1	small nuclear RNA activating complex, polypeptide 1, 43kDa	Nucleus	other
5.410	AF053136		SNCB	synuclein, beta	Cytoplasm	other
-2.810	AF049884		SORBS2	sorbin and SH3 domain containing 2	Plasma Membrane	other
-2.750	U90916		SORL1	sortilin-related receptor, L(DLR class) A repeats containing	Cytoplasm	transporter
2.010	AJ001454		SPOCK3	sparc/osteonectin, cwcv and kazal-like domains proteoglycan (testican) 3	Extracellular Space	other
3.210	U00968		SREBF1	sterol regulatory element binding transcription factor 1	Nucleus	transcription regulator
-4.670	U25997		STC1	stanniocalcin 1	Extracellular Space	kinase
-3.090	AB012124		TCFL5	transcription factor-like 5 (basic helix-loop-helix)	Nucleus	transcription regulator
-3.810	L12350		THBS2	thrombospondin 2	Extracellular Space	other
-4.090	Z19585		THBS4	thrombospondin 4	Extracellular Space	other
-7.940	U90902		TIAM1	T-cell lymphoma invasion and metastasis 1	Cytoplasm	other
2.030	M99436		TLE2	transducin-like enhancer of split 2	Nucleus	transcription regulator
2.900	AB028950		TLN1	talin 1	Plasma Membrane	other

2.490	AF023676		TM7SF2	transmembrane 7 superfamily member 2	Cytoplasm	enzyme
-5.040	AL080235		TMEM158	transmembrane protein 158 (gene/pseudogene)	Plasma Membrane	other
-4.090	M92383		TMSB10/TMSB4X	thymosin beta 10	Cytoplasm	other
2.710	AF096870		TRIM16	tripartite motif containing 16	Cytoplasm	transcription regulator
-3.860	M36035		TSPO	translocator protein (18kDa)	Cytoplasm	transmembrane receptor
2.150	AB017644		UBE2E3	ubiquitin-conjugating enzyme E2E 3	Nucleus	enzyme
-2.680	X15998		VCAN	versican	Extracellular Space	other
4.230	AF039555		VSNL1	visinin-like 1	Cytoplasm	other
-3.330	X62048		WEE1	WEE1 G2 checkpoint kinase	Nucleus	kinase
-26.980	M92843		ZFP36	ZFP36 ring finger protein	Nucleus	transcription regulator
-4.550	X79067		ZFP36L1	ZFP36 ring finger protein-like 1	Nucleus	transcription regulator
-17.220	X78992		ZFP36L2	ZFP36 ring finger protein-like 2	Nucleus	transcription regulator
6.190	AF052145		ZNF804A	zinc finger protein 804A	Other	other
19.870	R61362	unmapped				
5.890	AL022326	unmapped				

5.430	X57985	unmapped
3.430	AC004010	unmapped
3.010	AC004142	unmapped
2.990	HG4724-HT51	unmapped
2.920	AI885852	unmapped
2.900	W26466	unmapped
2.820	W28508	unmapped
2.690	X94700	unmapped
2.100	AL049988	unmapped
2.000	AL031652	unmapped
-2.010	AC003003	unmapped
-2.090	AF037643	unmapped
-2.180	AL022726	unmapped
-2.620	U52153	unmapped
-3.770	AL031228	unmapped

-3.810	AL021786	unmapped
-4.100	AL021154	unmapped
-4.960	Z97632	unmapped
-5.180	AB002362	unmapped
-5.270	D15050	unmapped
-5.710	AF004230	unmapped
-7.410	HG172-HT392	unmapped
-9.580	AL038340	unmapped
-9.910	HG3527-HT37	unmapped
-19.550	J03071	unmapped
-36.910	HG1751-HT17	unmapped
-39.840	M54994	unmapped
-85.030	J03071	unmapped
-255.100	M54994	unmapped
