# Supplementary material for: Multiomics-Based Signaling Pathway Network Alterations in Human Non-functional Pituitary Adenomas
Source: Front Endocrinol (Lausanne). 2019 Dec 17;10:835. doi: 10.3389/fendo.2019.00835 (PMC6928143; doi:10.3389/fendo.2019.00835)

Supplemental materials 1.3

Statistically significant canonical pathways derived from differentially expressed genes between NFPAs and controls (Dataset 1)

1. Putrescine Degradation Ⅲ
2. Tryptophan Degradation X (Mammalian via Tryptamine)
3. Noradrenaline and Adrenaline Degradation
4. Agranulocyte Adhesion and Diapedesis
5. Unfolded protein response
6. Dopamine Degradation
7. Granulocyte Adhesion and Diapedesis
8. Fatty Acid α-oxidation
9. Calcium Signaling
10. Ethanol Degradation Ⅱ
11. Growth Hormine Signaling
12. Serotonin Degradation
13. CCR3 Signaling in Eosinophils
14. Atherosclerosis Signaling
15. Adipogenesis pathway
16. Histamine Degradation
17. Oxidative Ethanol Degradation Ⅲ
18. Actin Cytoskeleton Signaling
19. TR/RXR Activation
20. Role of NFAT in Regulation of the Immune Response
21. Ethanol Degradation Ⅳ
22. p53 Signaling
23. Role of NFAT in Cardiac Hypertrophy
24. IL-17A Signaling in Fibroblasts
25. Calcium-induced T Lymphocyte Apoptosis
26. Hepatic Fibrosis/Hepatic Stellate Cell Activation
27. Gaq Signaling
28. Role of MAPK Signaling in the Pathogenesis of Influenza
29. Calcium Transport Ⅰ
30. Ephrin B Signaling
31. Chemokine Signaling
32. Corticotropin Releasing Hormone Signaling
33. CD28 Signaling in T Helper Cells
34. MIF Regulation of Innate Immunity
35. VEGF Family Ligand-Receptor Interaction
36. Docosahexaenoic Acid(DHA) Signaling
37. Regulation of the Epithelial-Mesenchymal Transition Pathway
38. ERK/MAPK Signaling
39. α-Adrenergic Signaling
40. D-myo-inositol(1,4,5)-trisphosphate Degradation
41. IGF-1 Signaling
42. Epithelial Adherens Junction Signaling
43. Polyamine Regulation in Colon Cancer
44. Nur77 Signaling in T Lymphocytes
45. T Cell Receptor Signaling
46. Leukocyte Extravasation Signaling
47. EGF Signaling
48. Cholecystokinin-Gastrin-mediated Signaling
49. Glucocorticoid Receptor Signaling
50. CXCR4 Signaling
51. Nitric Oxide Signaling in the Cardiovascular System
52. iCOS-iCOSL Signaling in T Helper Cells
53. Tight Junction Signaling
54. Fc Epsilon RI Signaling
55. fMLP Signaling in Neutrophils
56. Angiopoietin Signaling
57. Role of Osteoblasts, Osteoclasts and Chondrocytes in Rheumatoid Arthritis
58. Hereditary Breast Cancer Signaling
59. Putrescine Biosynthesis Ⅲ
60. Renin-Angiotensin Signaling
61. IL-3 Signaling
62. GDNF Family Ligand-Receptor Interactions
63. PEDF Signaling
64. PTEN Signaling
65. Superpathway of D-myo-inositol(1,4,5)-trisphosphate Metabolism
66. Phenylalanine Degradation Ⅳ (Mammalian, via Side Chain)
67. Ephrin Receptor Signaling
68. CCRS Signaling in Macrophages

Dataset 1-Cannonical Pathway Chart

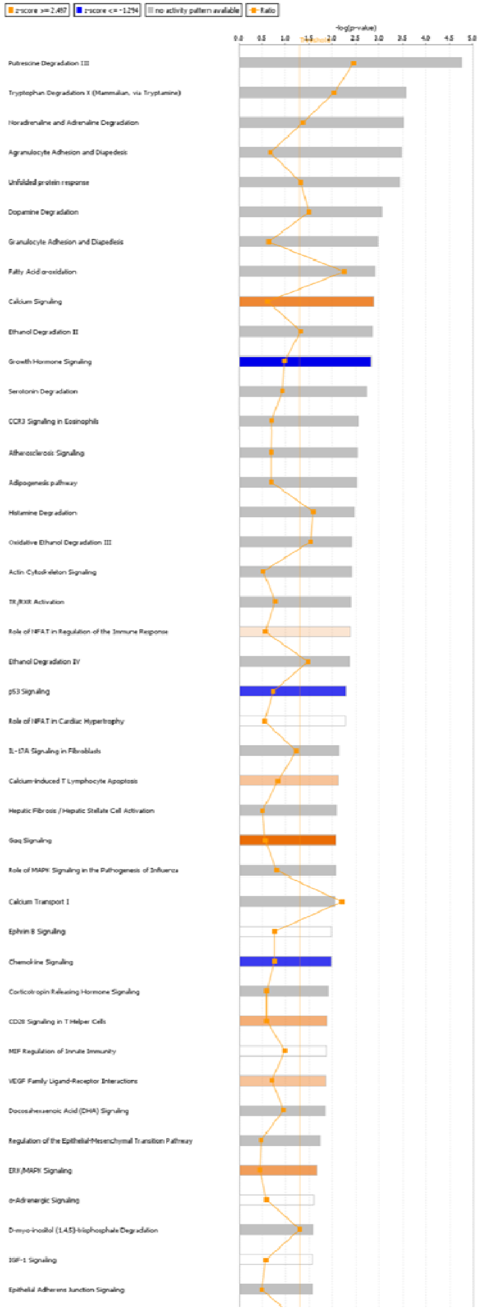

1-Putrescine Degradation III

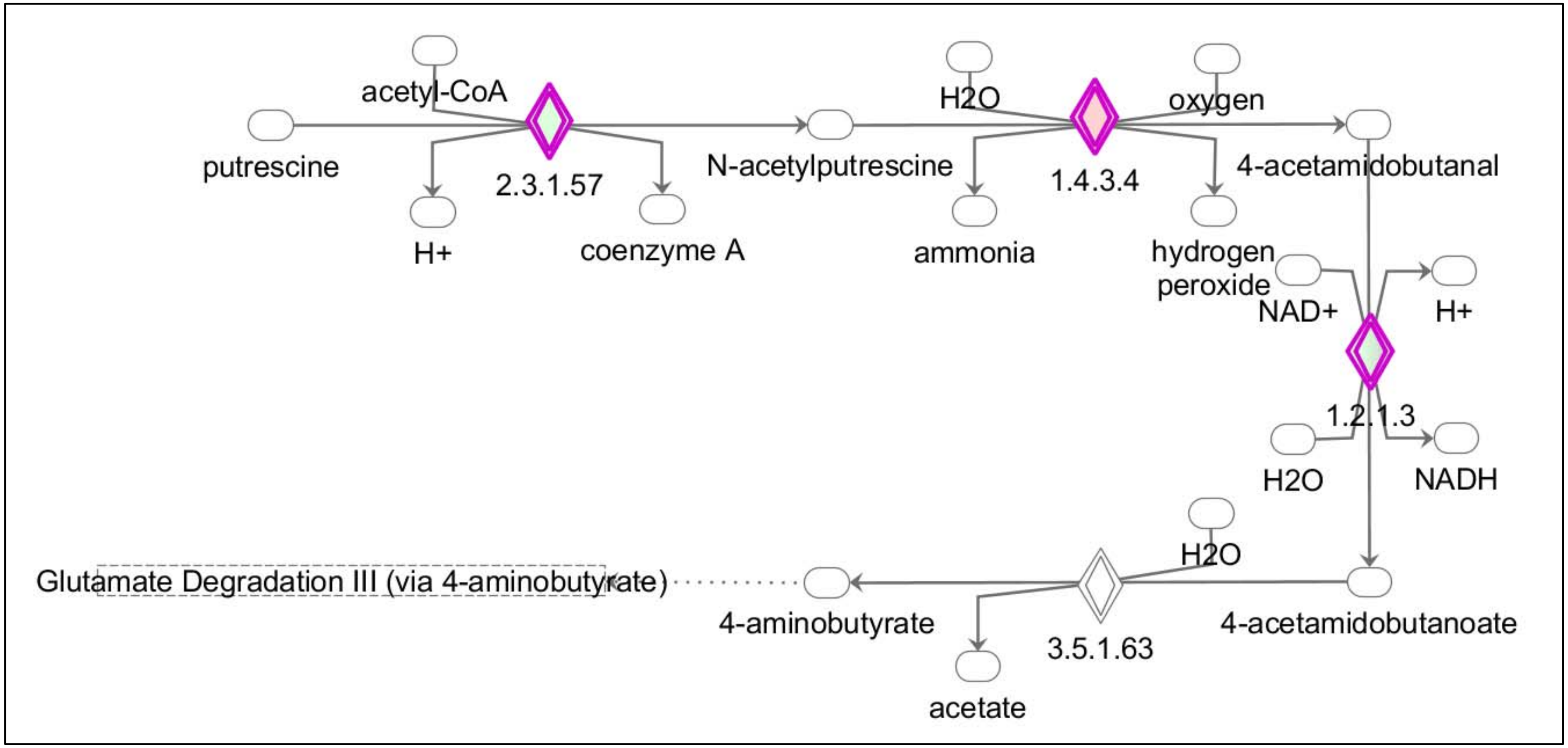

2-Tryptophan Degradation X (Mammalian via Tryptamine)

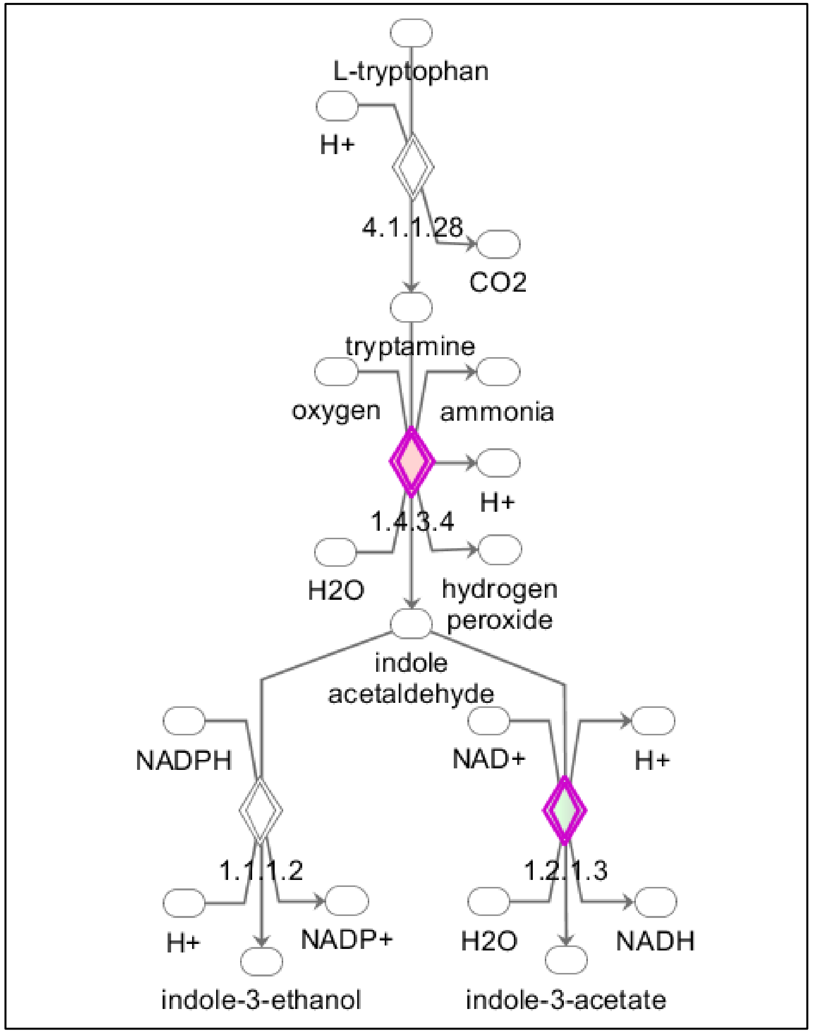

3-Noradrenaline and Adrenaline Degradation

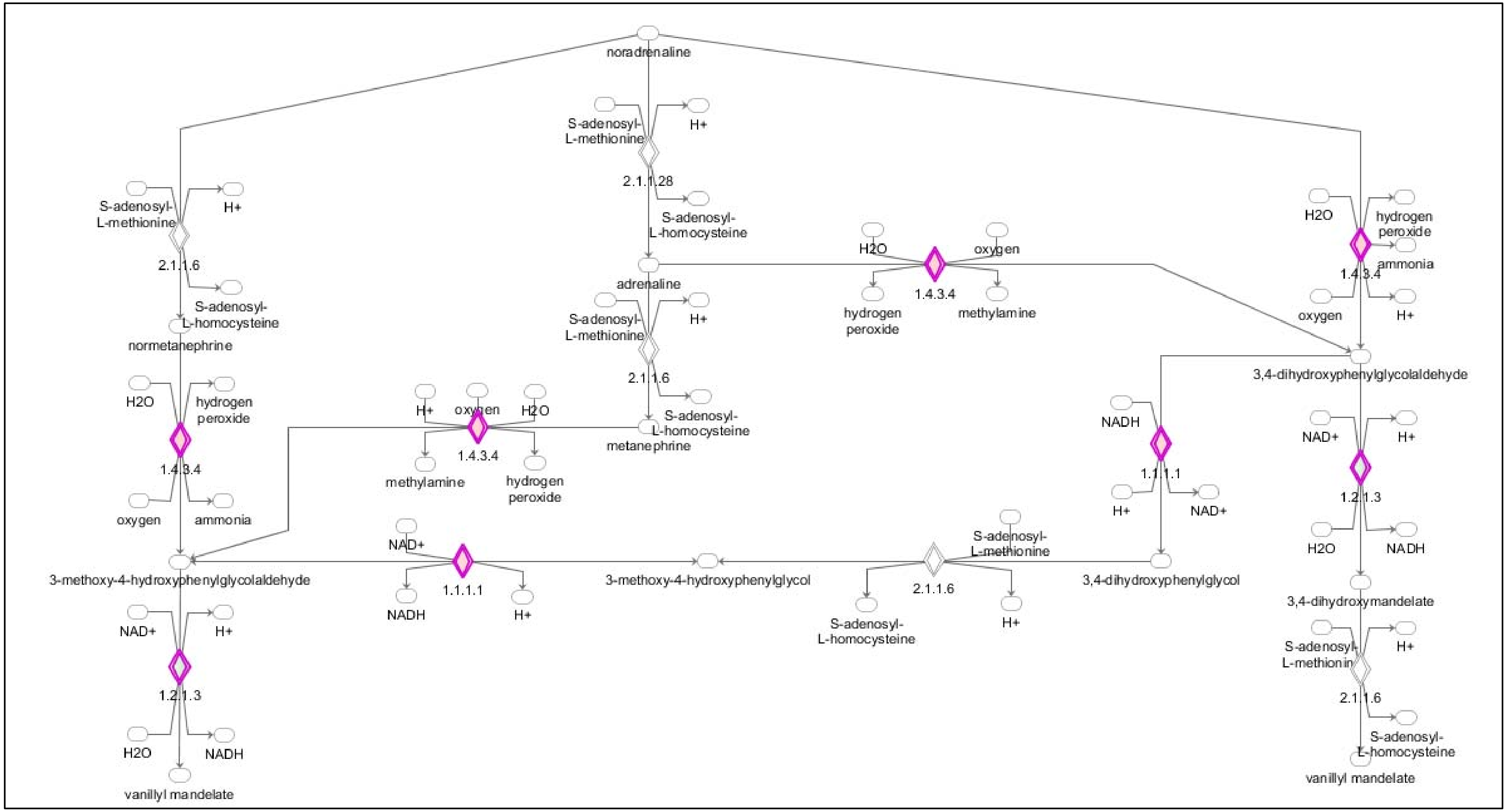

4-Agranulocyte Adhesion and Diapedesis

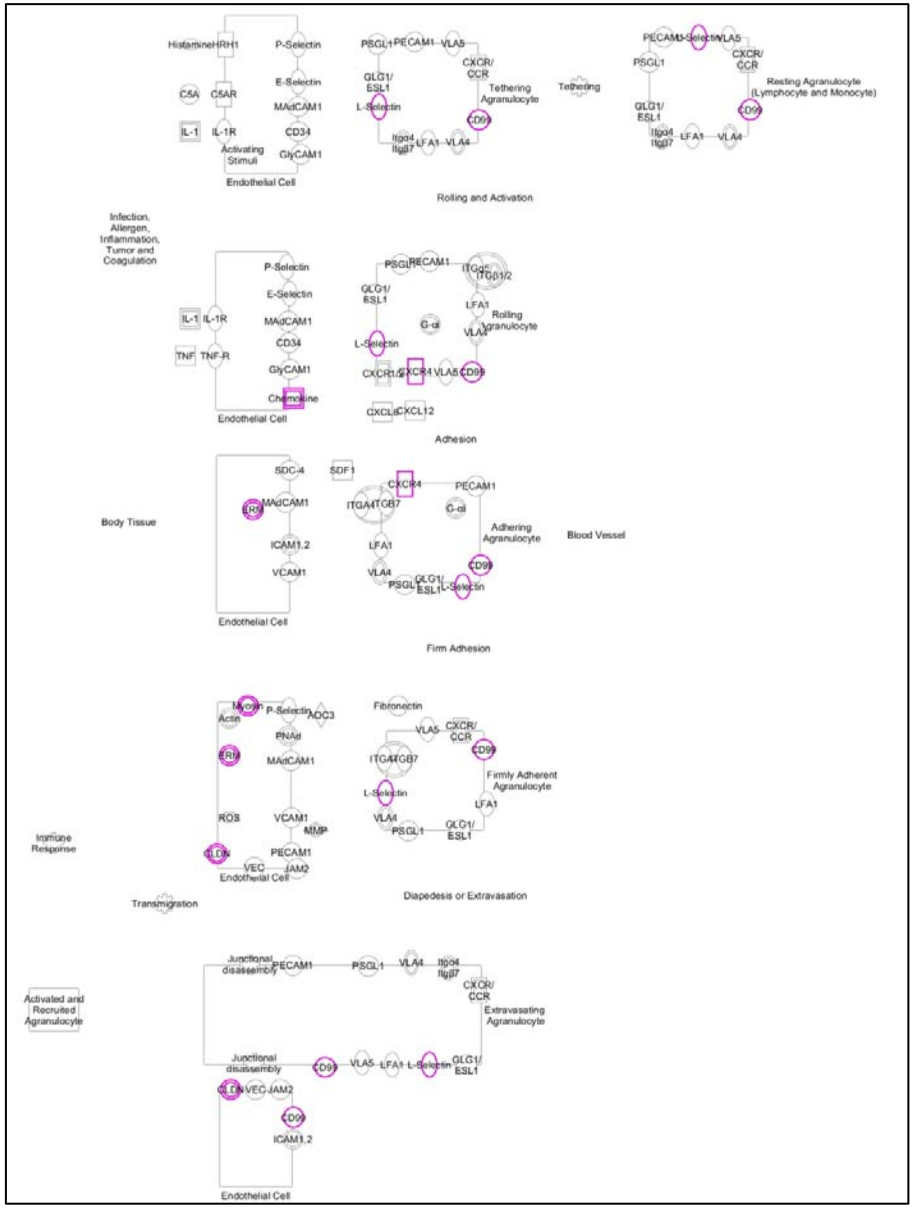

5-Unfolded protein response

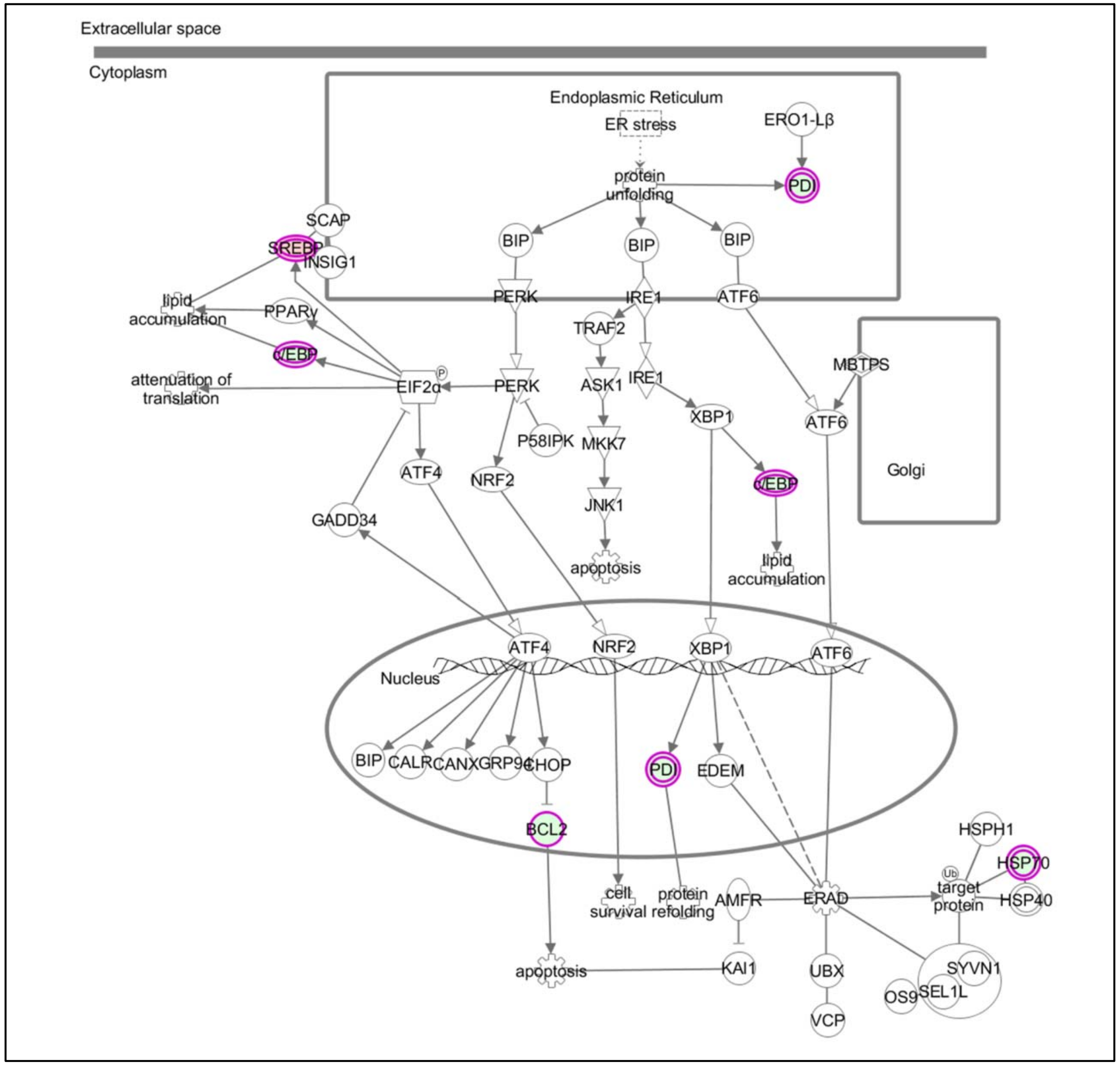

6-Dopamine Degradation

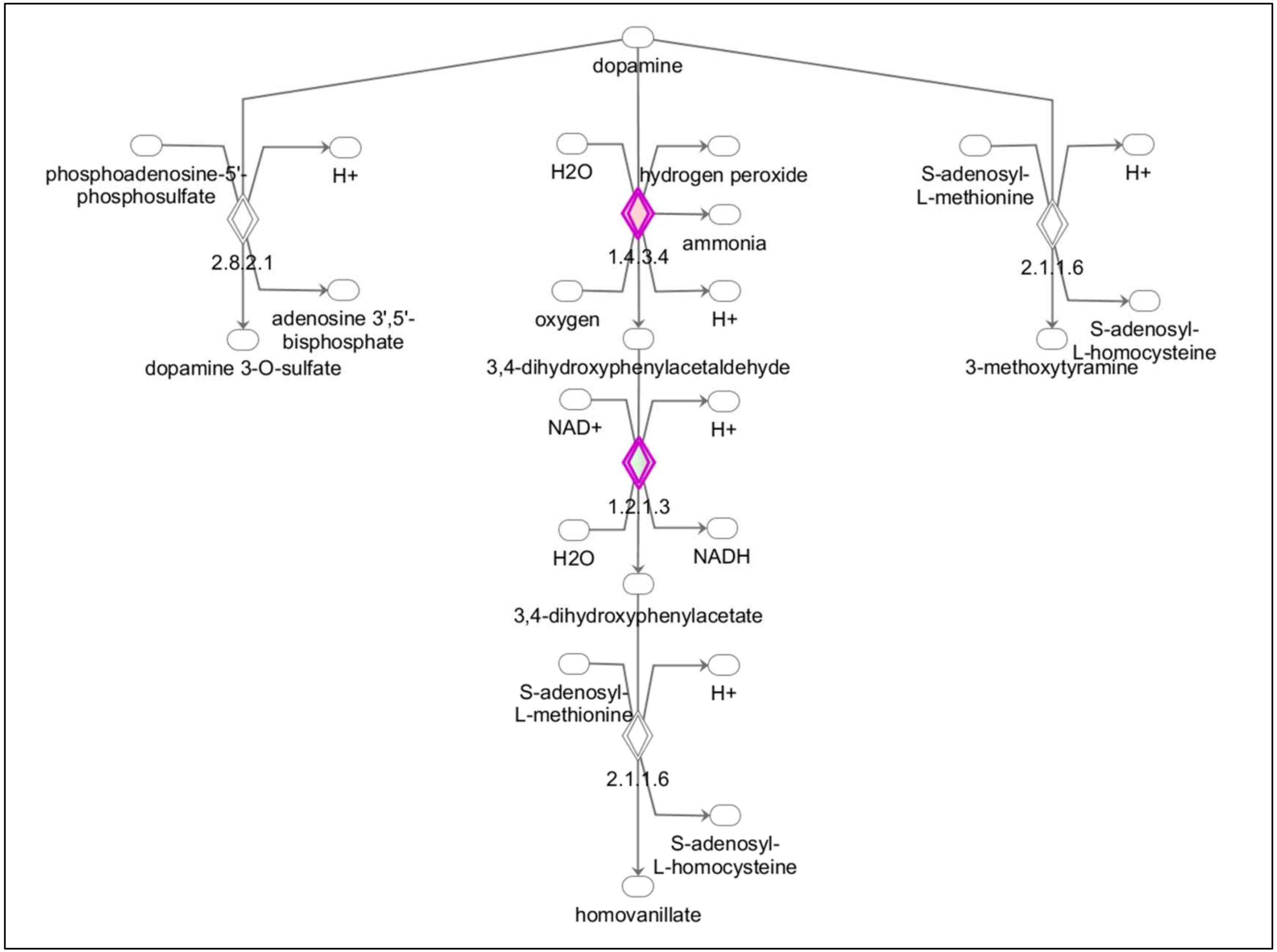

7-Granulocyte Adhesion and Diapedesis

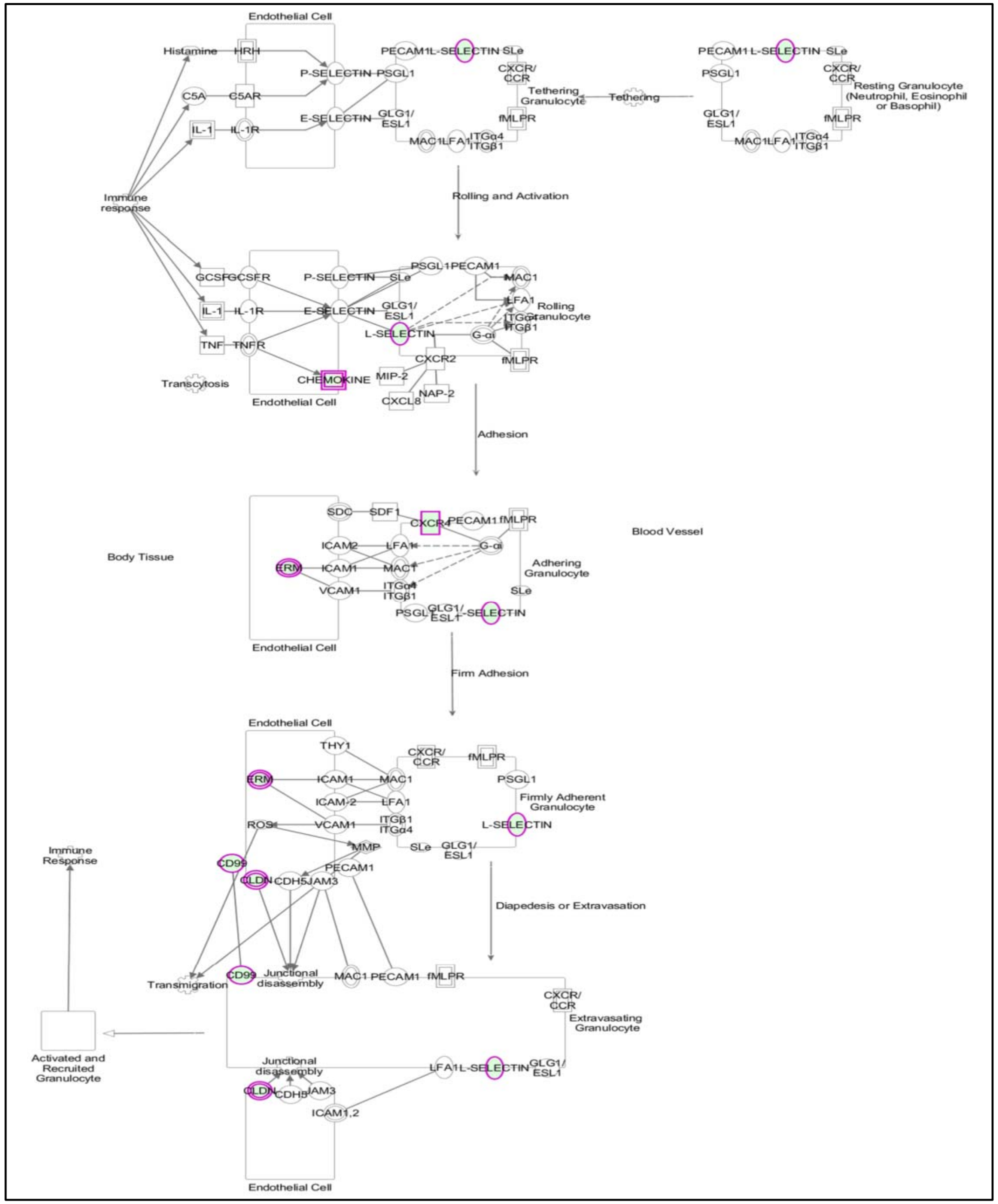

8-Fatty Acid α -oxidation

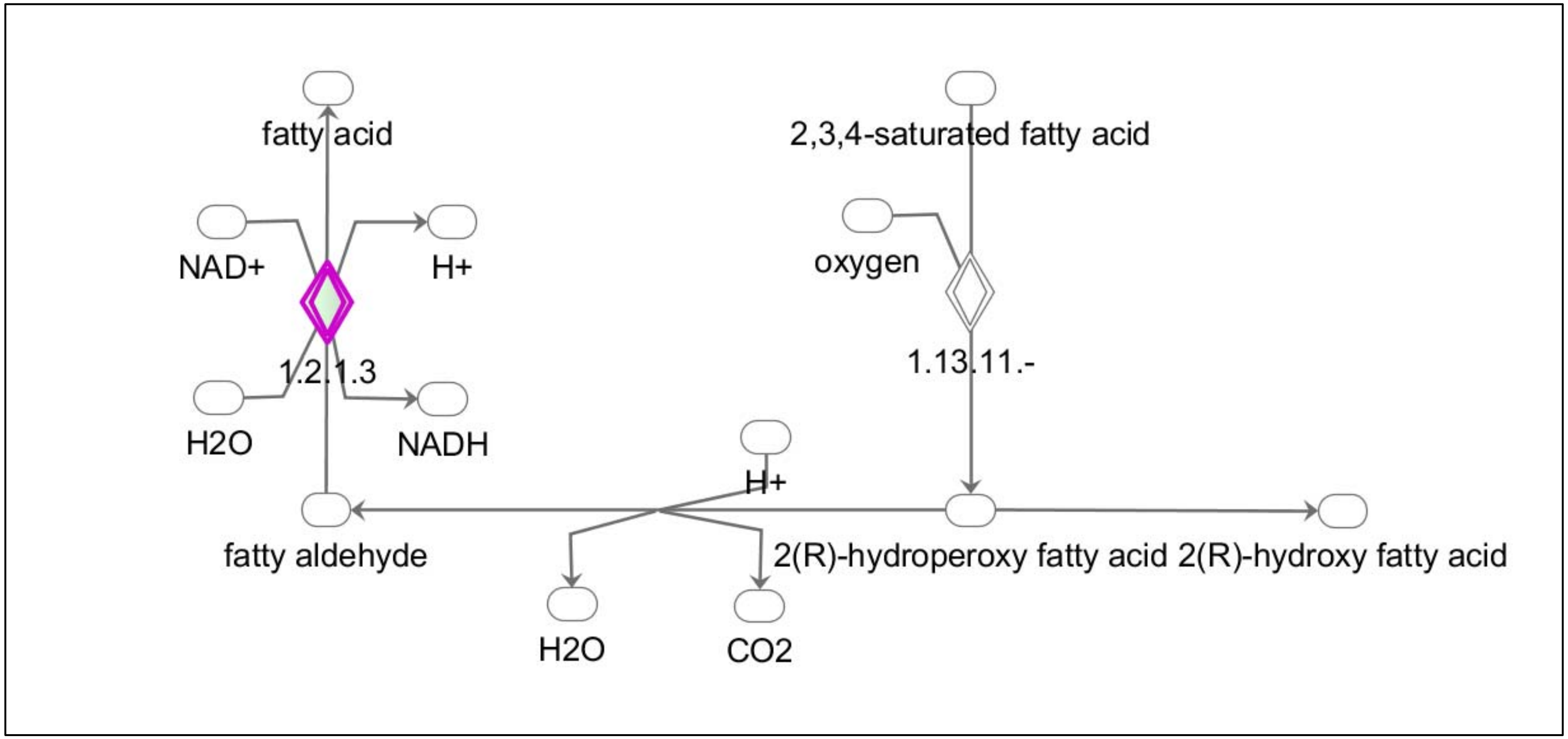

9-Calcium Signaling

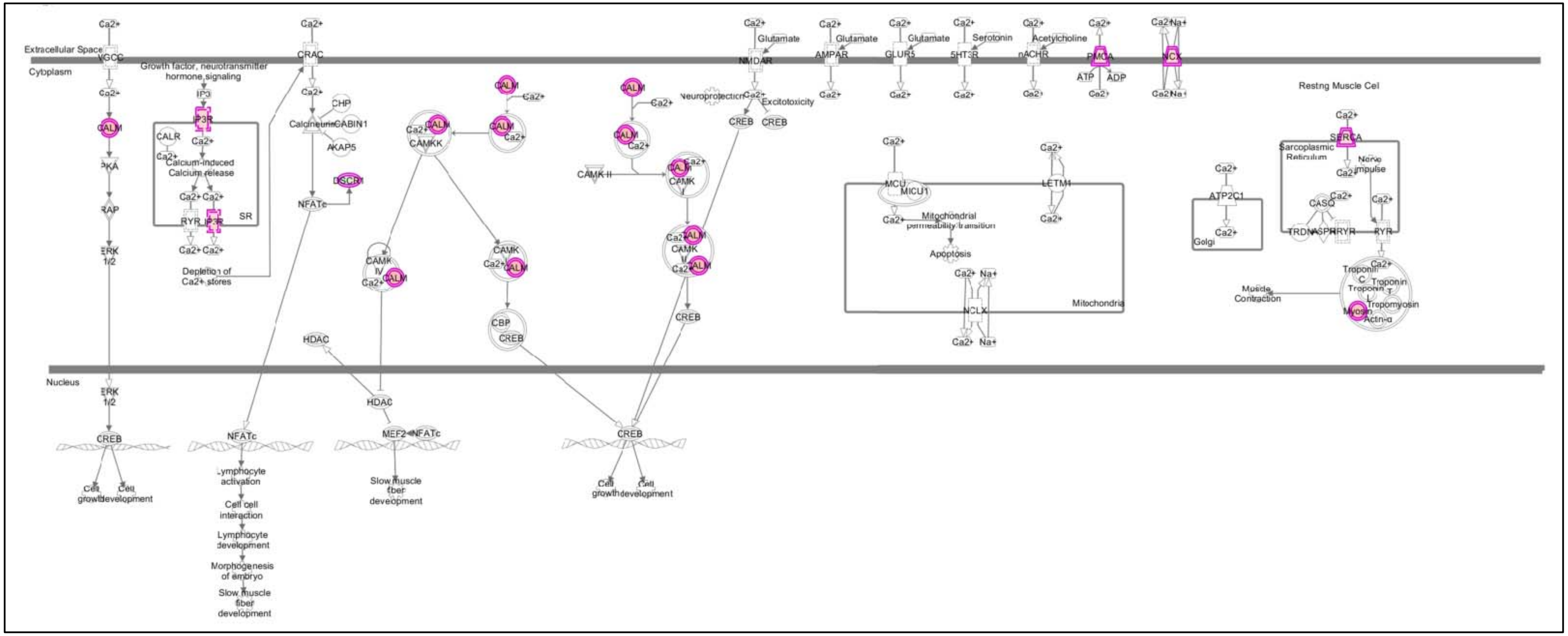

10-Ethanol Degradation Ⅱ

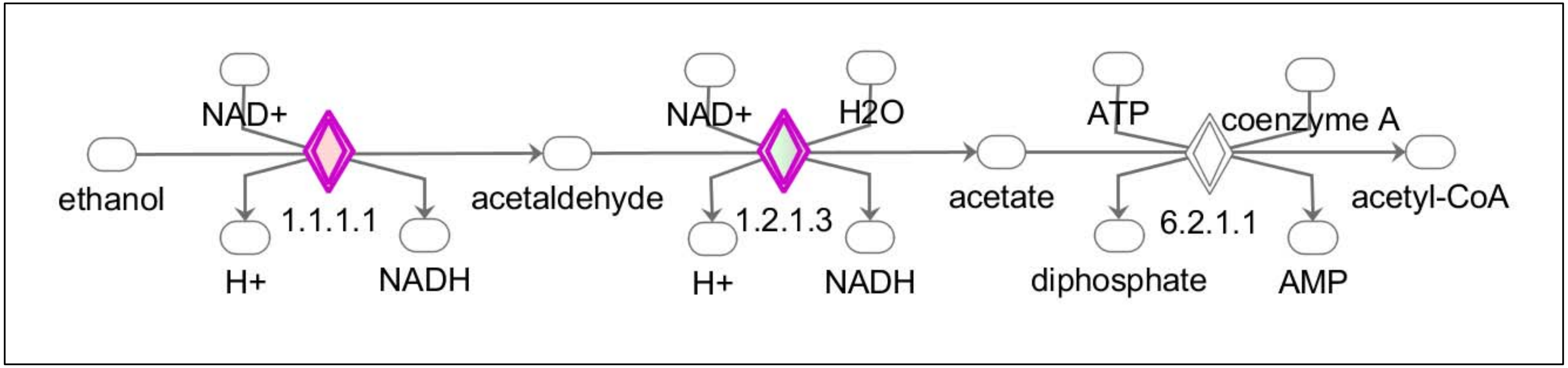

11-Growth Hormine Signaling

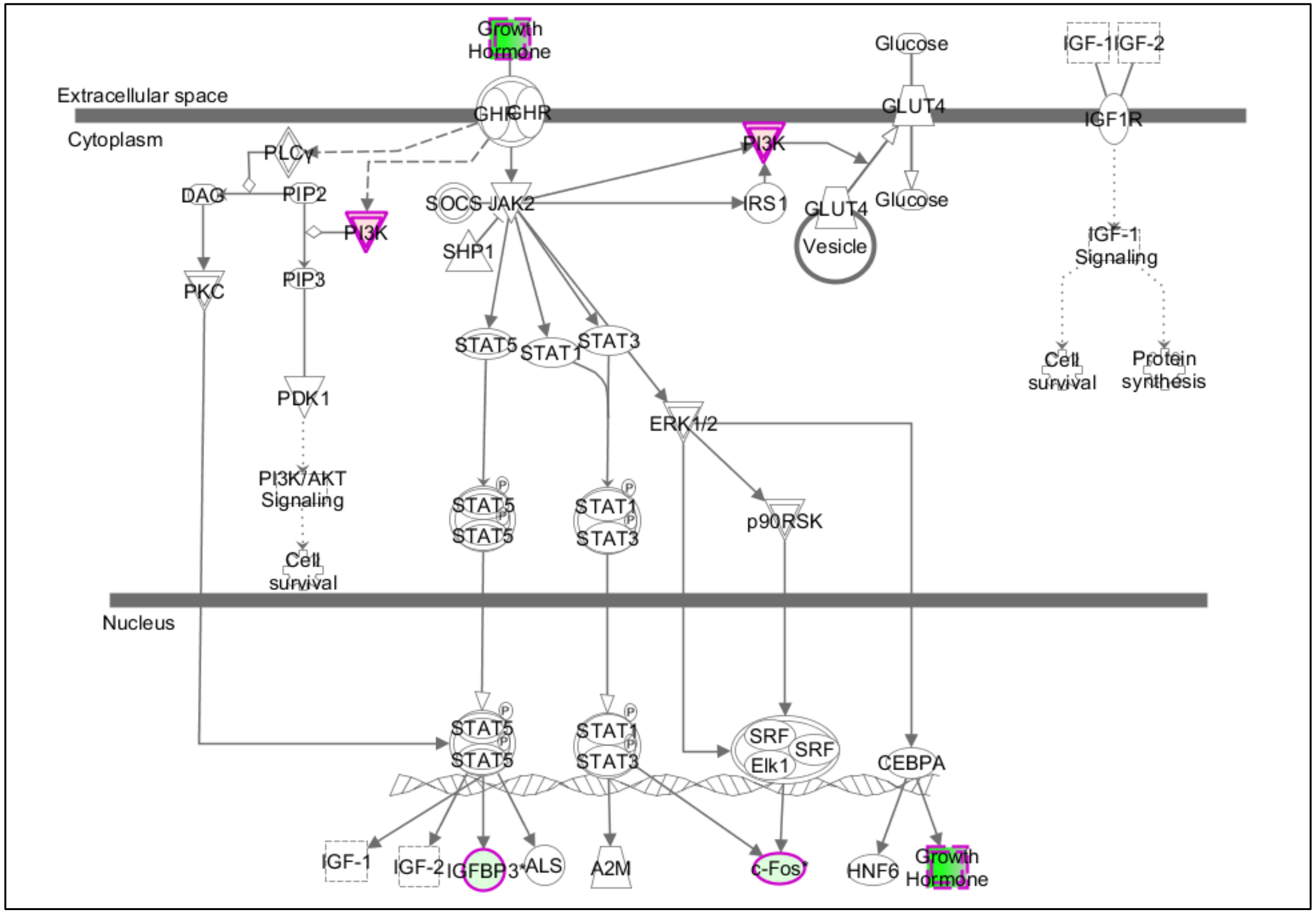

12-Serotonin Degradation

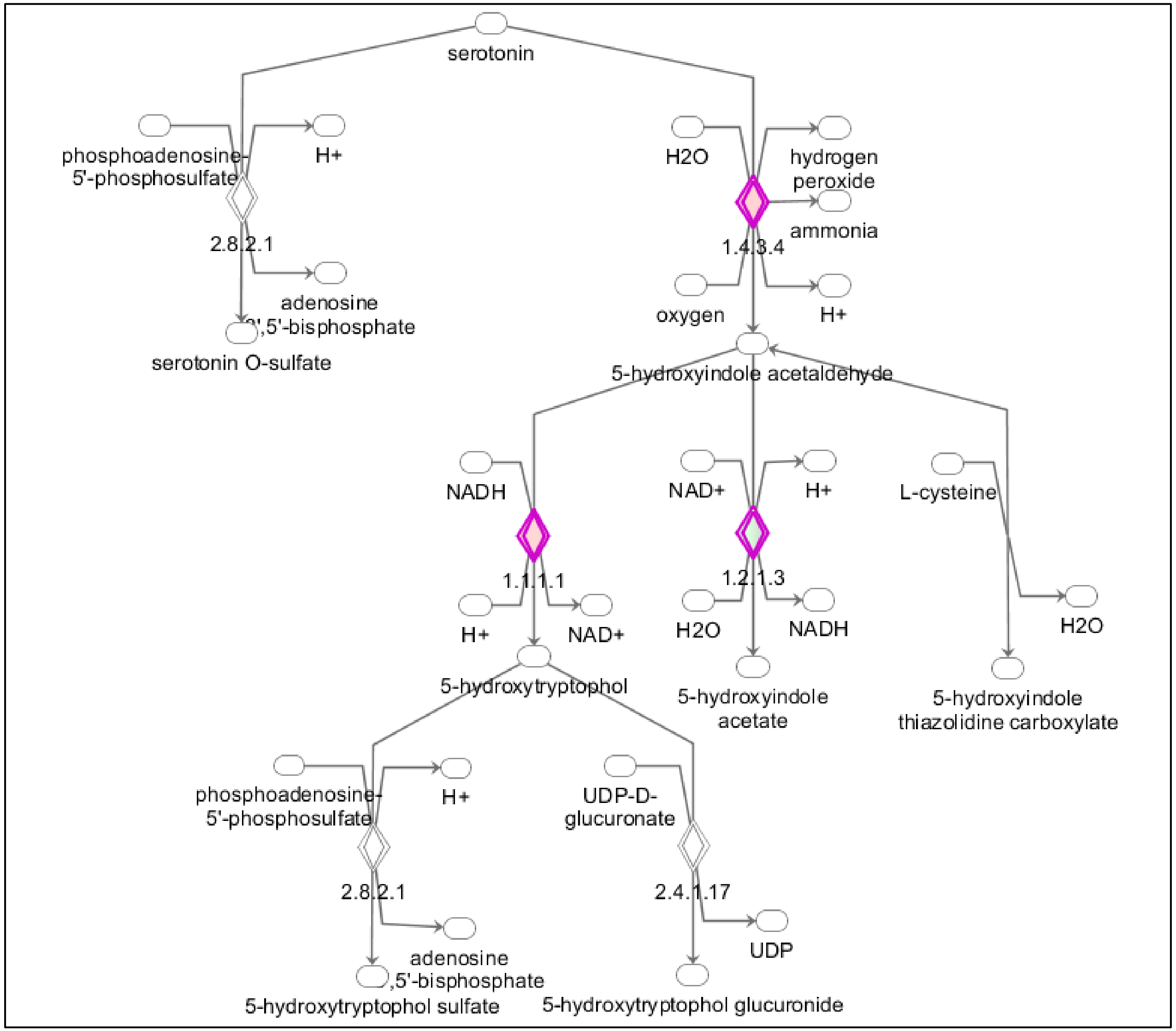

13-CCR3 Signaling in Eosinophils

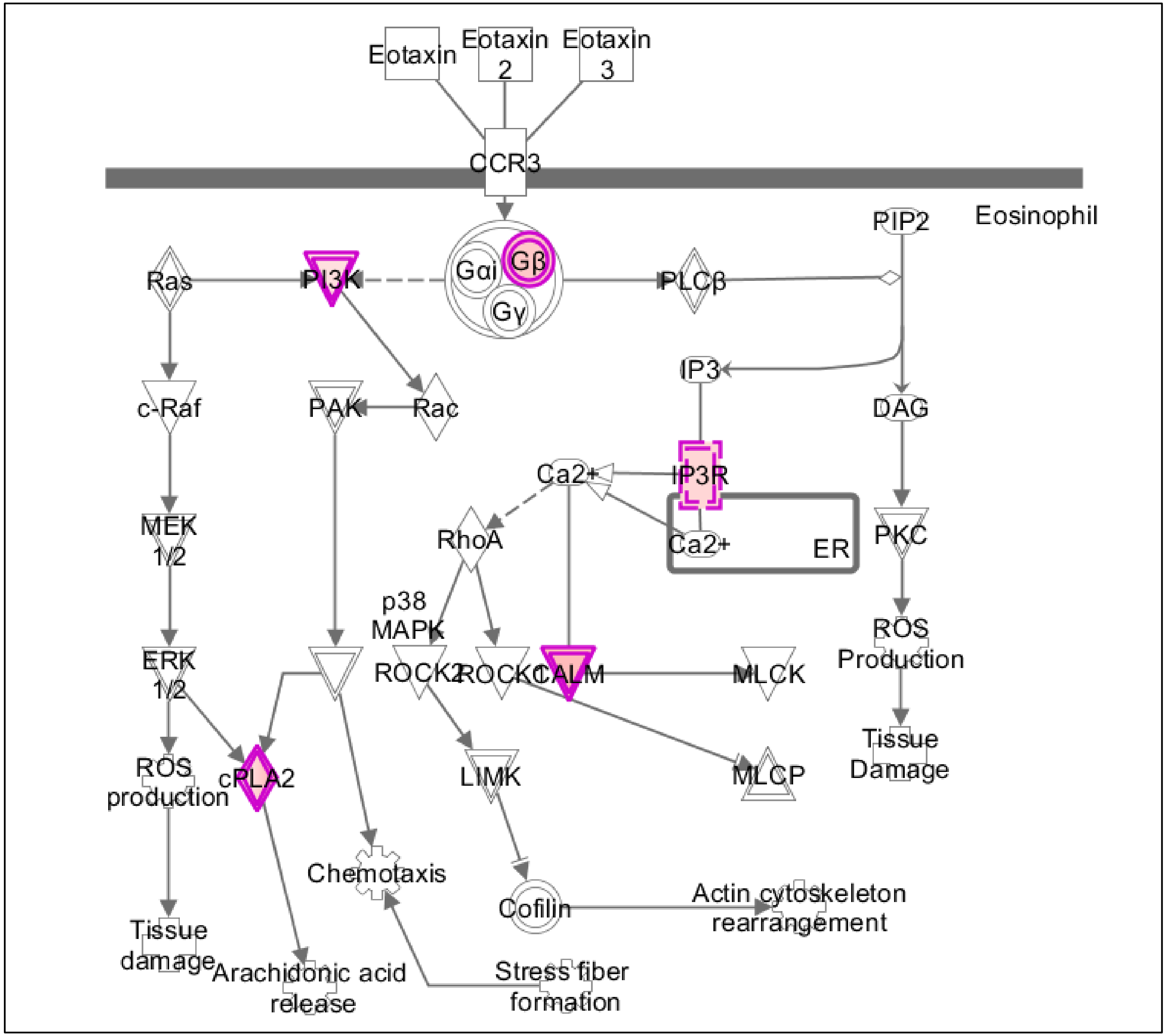

14-Atherosclerosis Signaling

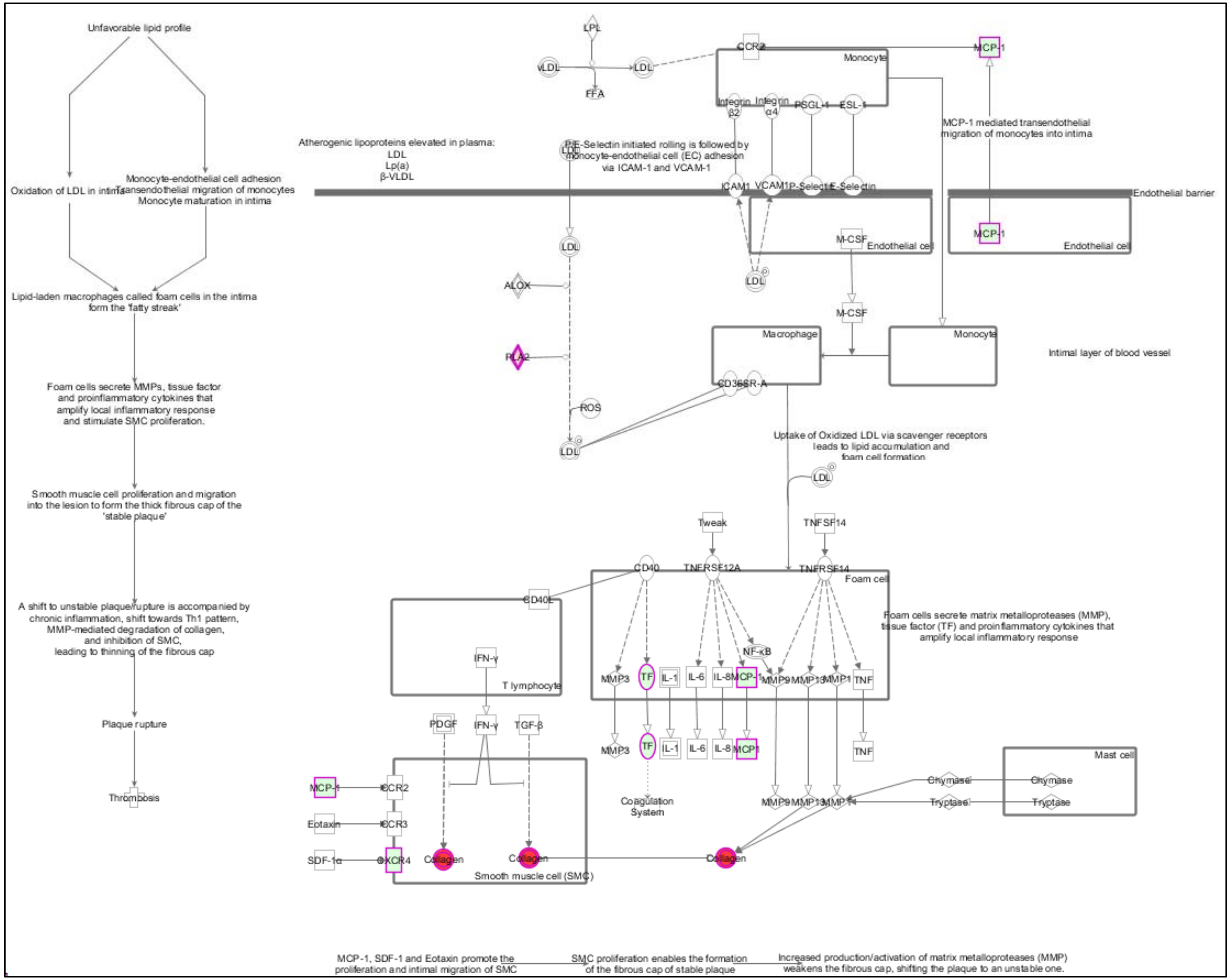

15-Adipogenesis pathway

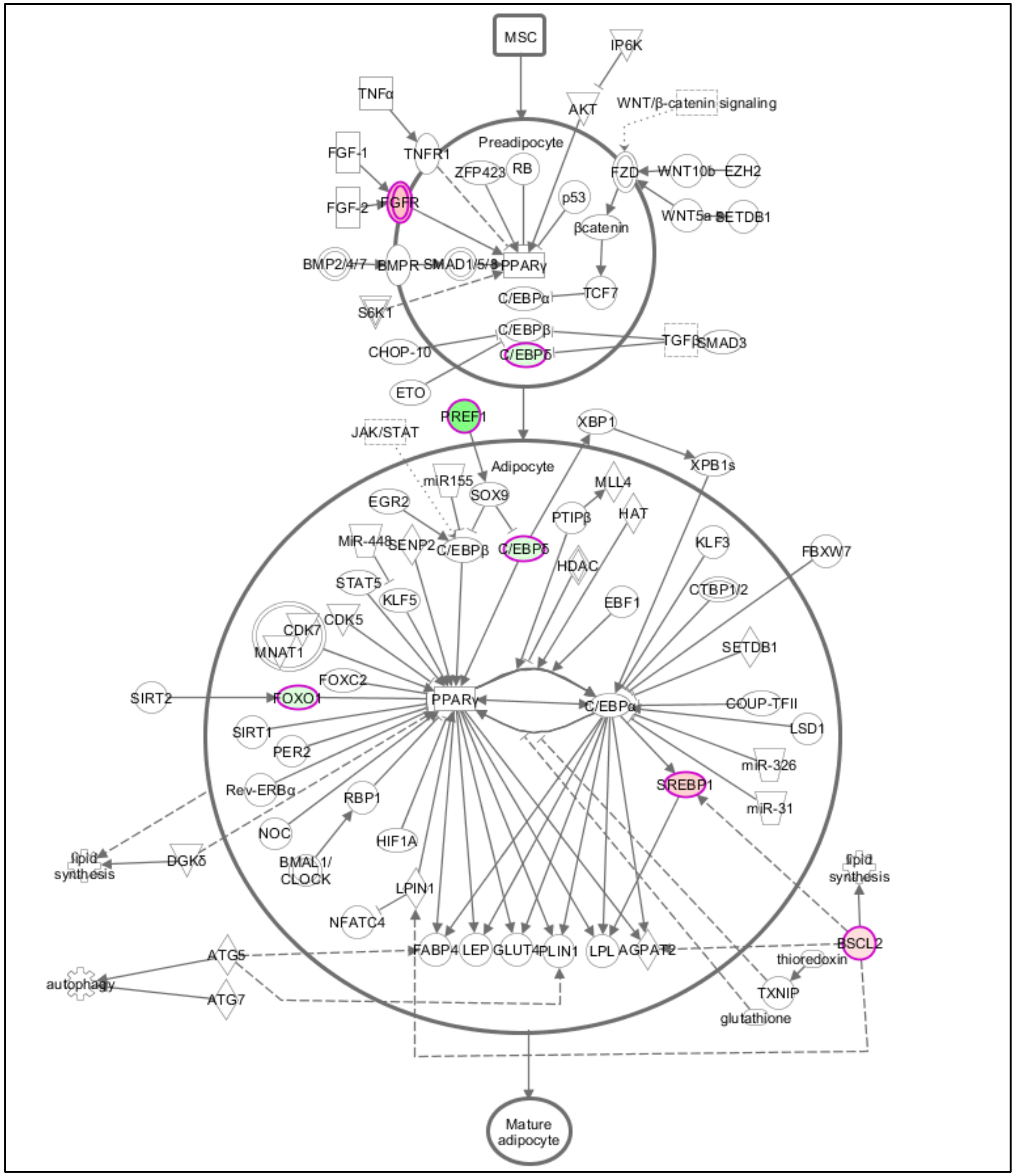

16-Histamine Degradation

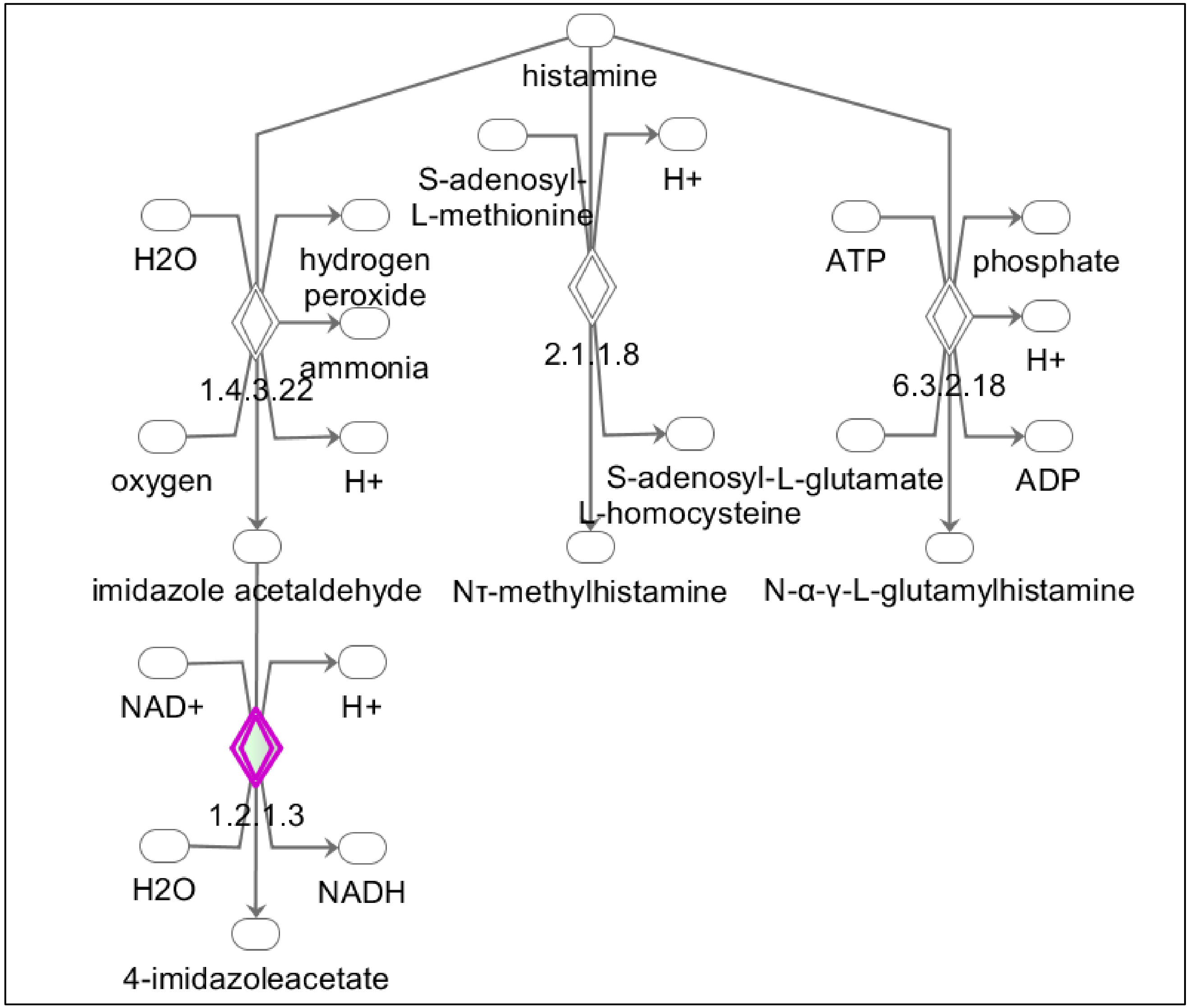

17-Oxidative Ethanol Degradation III

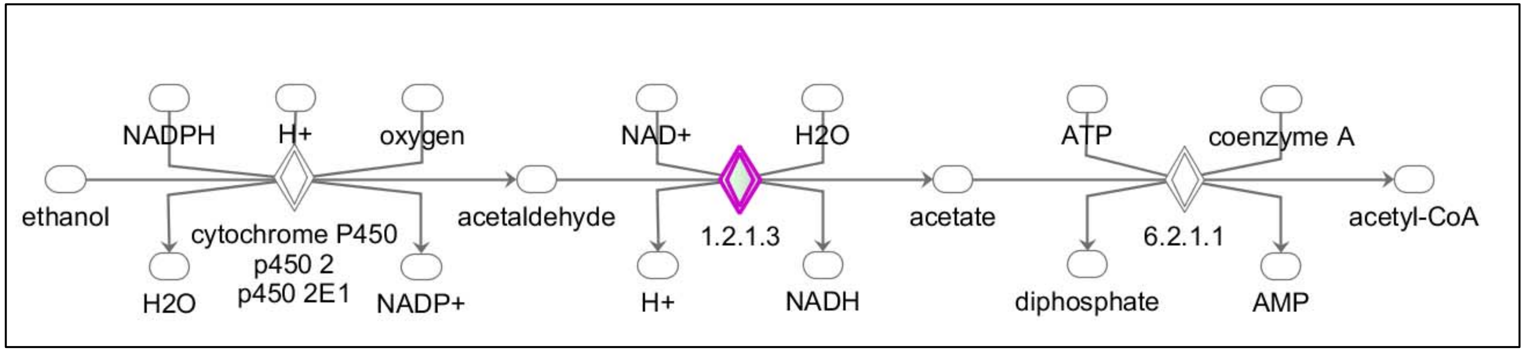

18-Actin Cytoskeleton Signaling

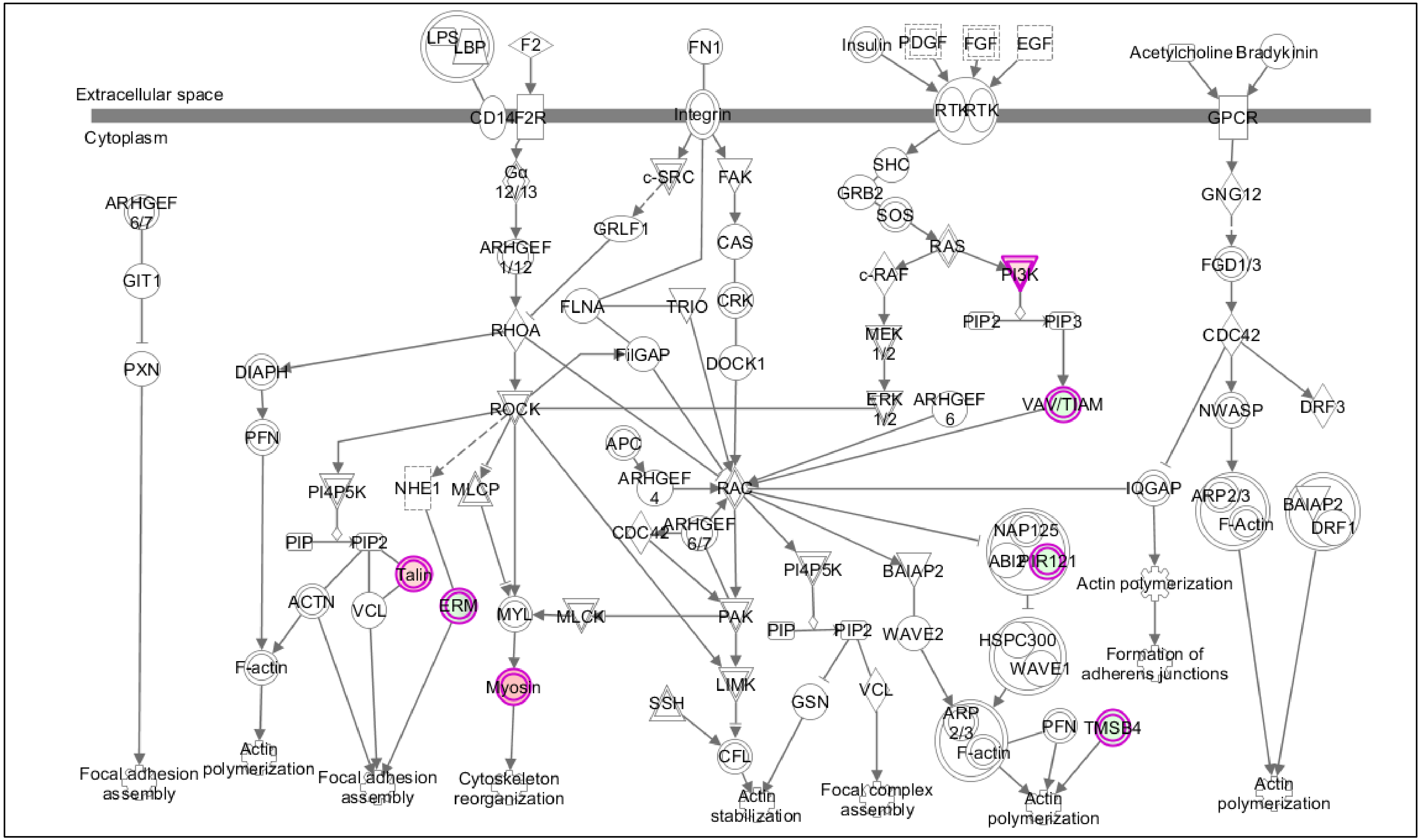

19-TR/RXR Activation

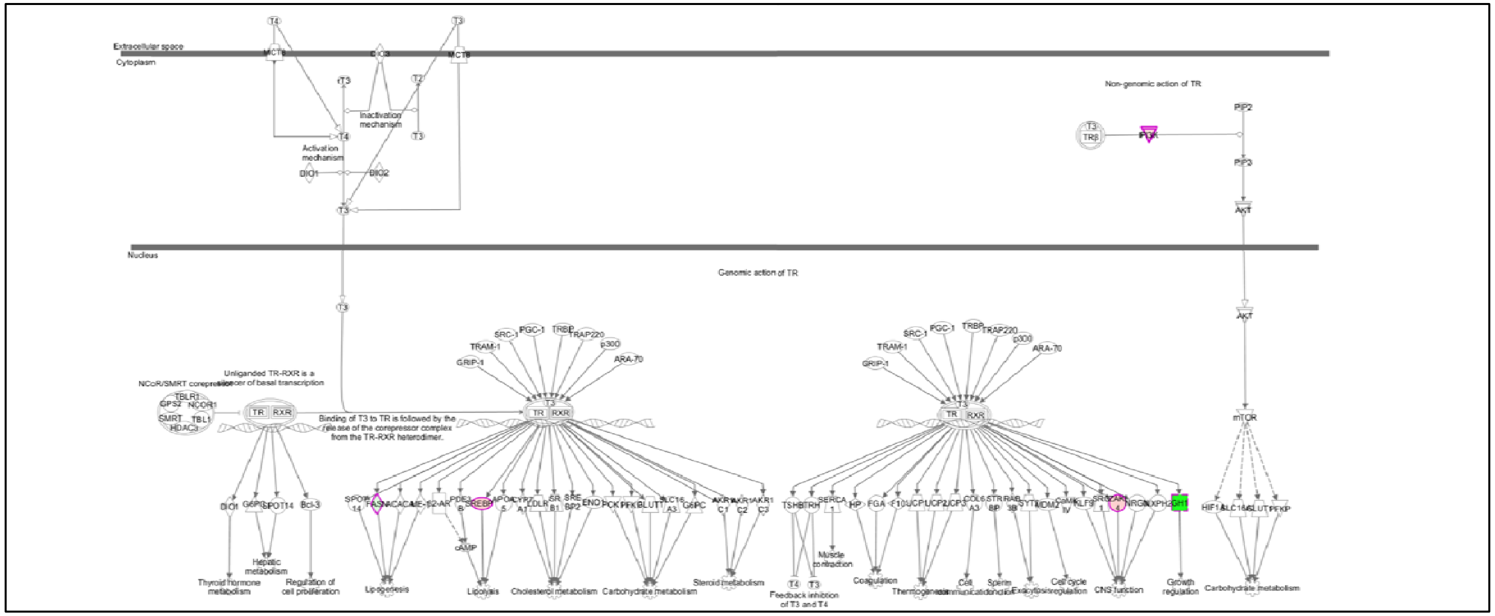

20-Role of NFAT in Regulation of the Immune Response

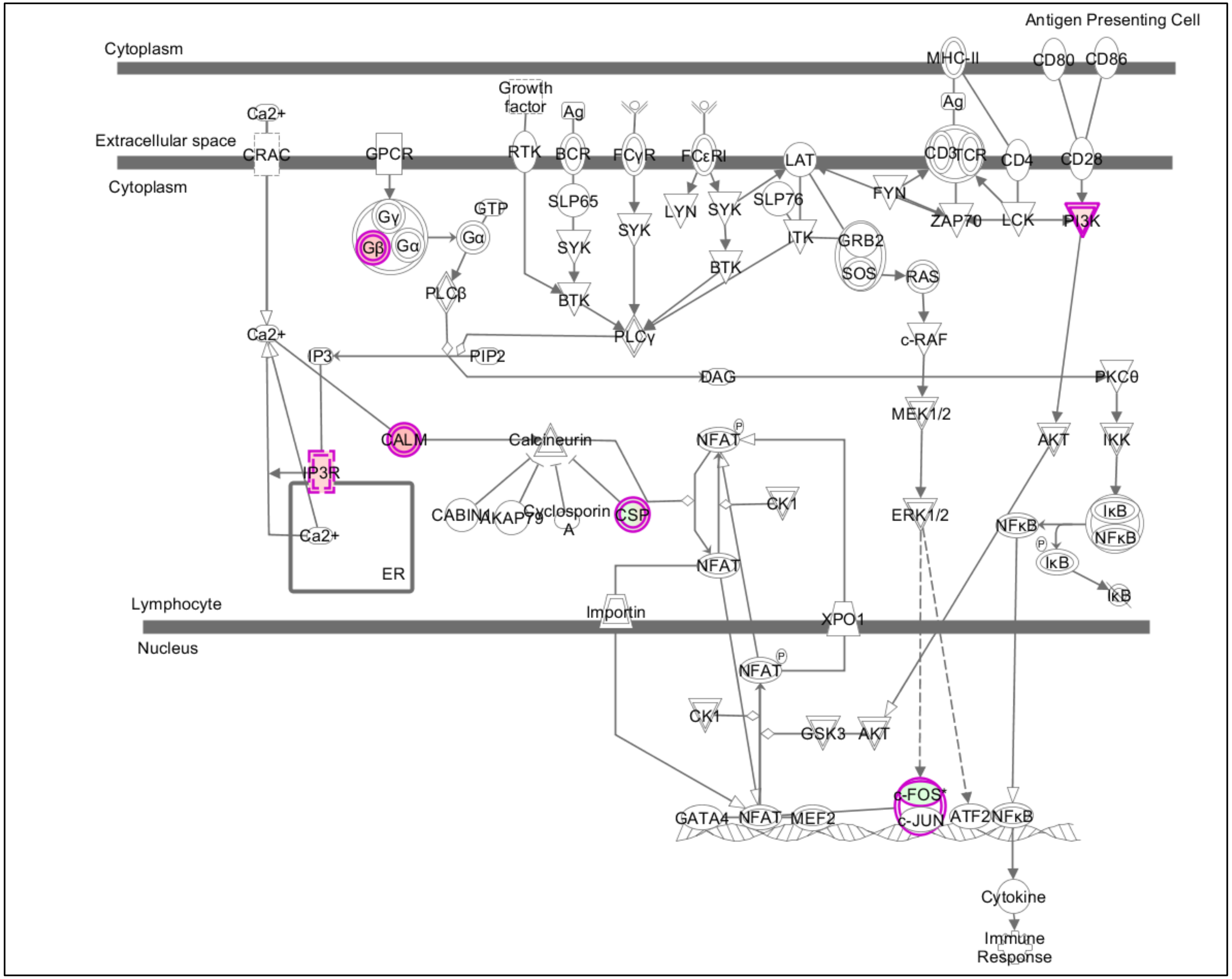

21-Ethanol Degradation Ⅳ

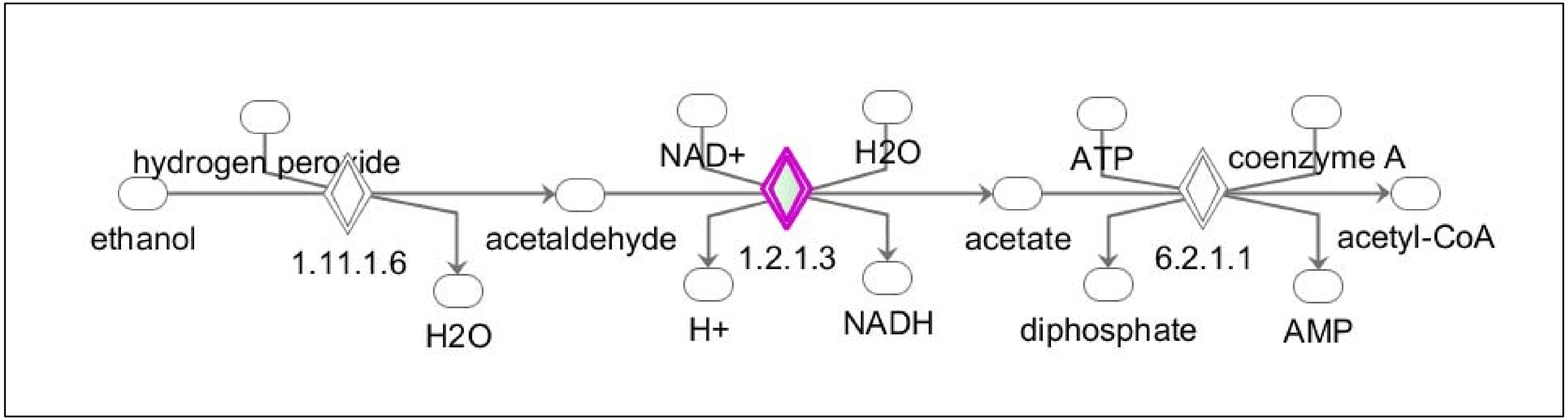

22-p53 Signaling

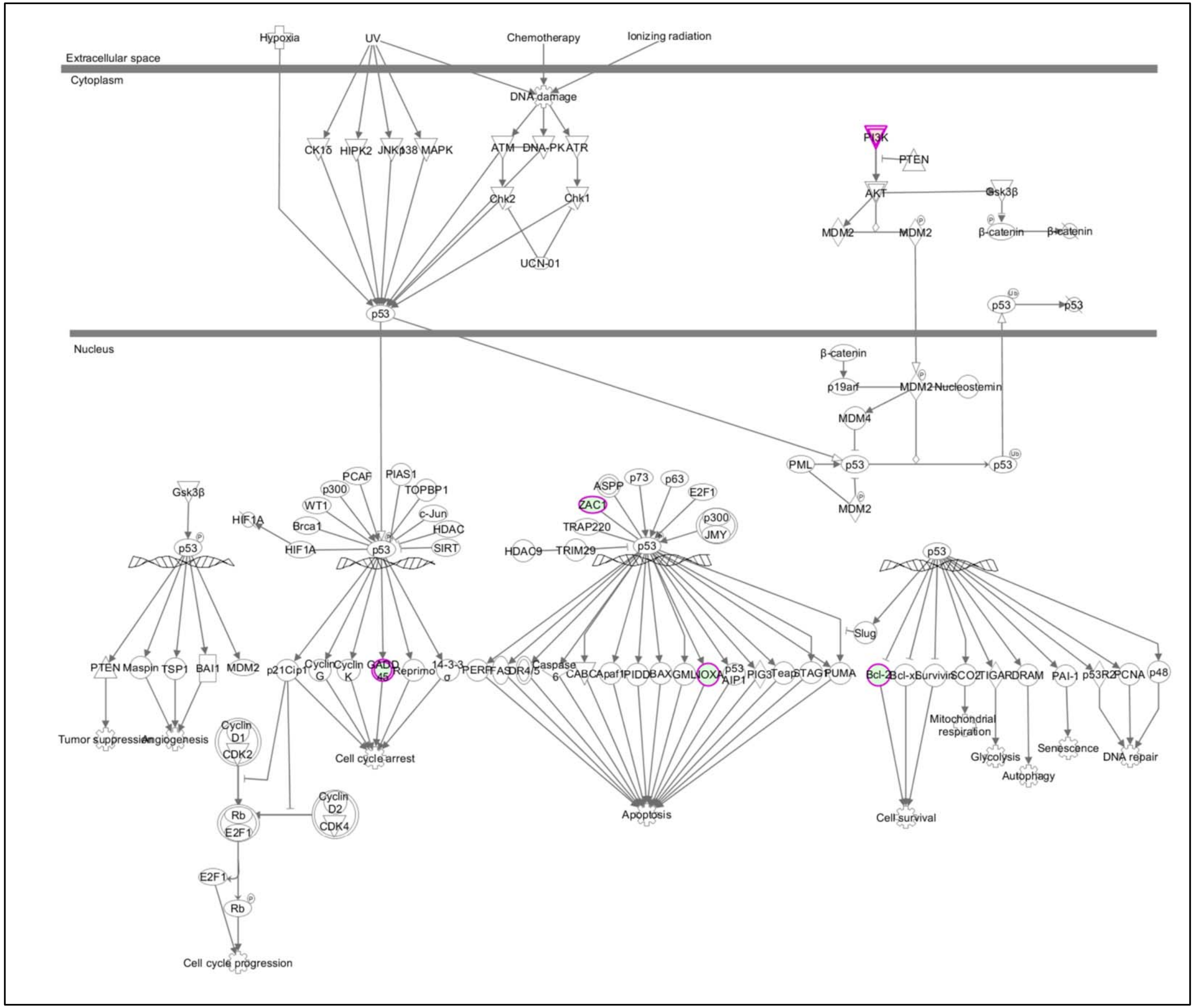

23-Role of NFAT in Cardiac Hypertrophy

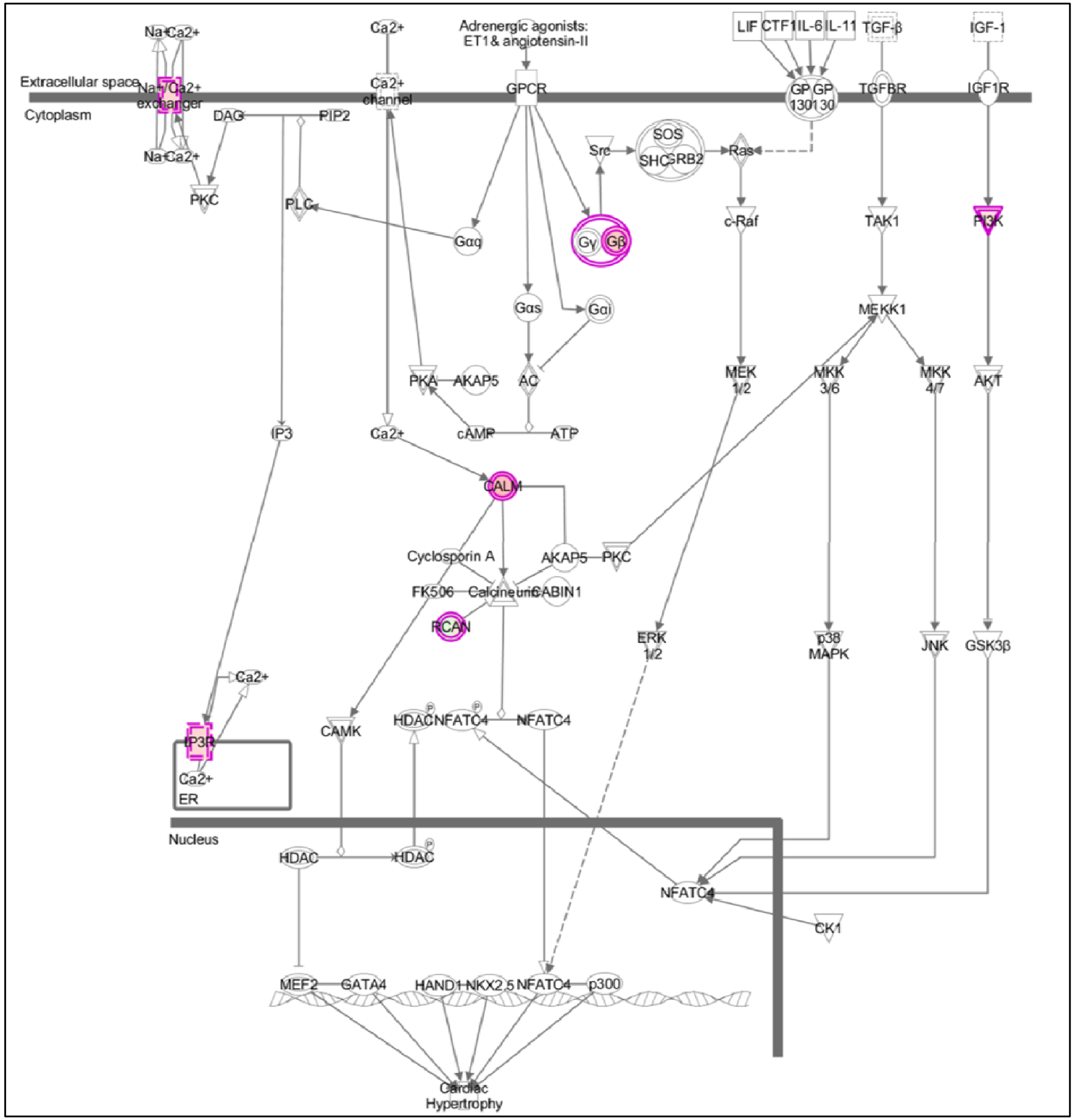

24-IL-17A Signaling in Fibroblasts

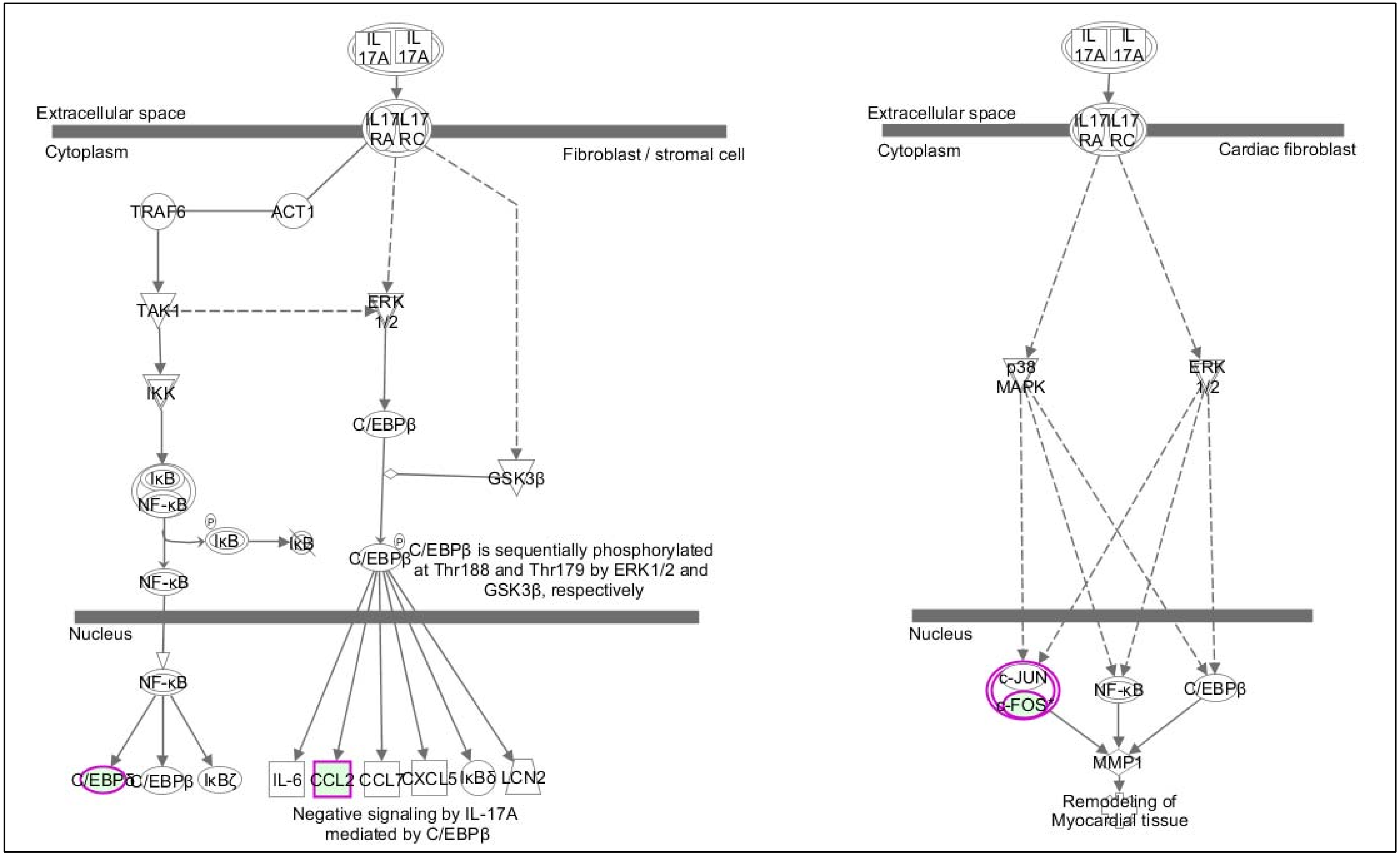

25-Calcium-induced T Lymphocyte Apoptosis

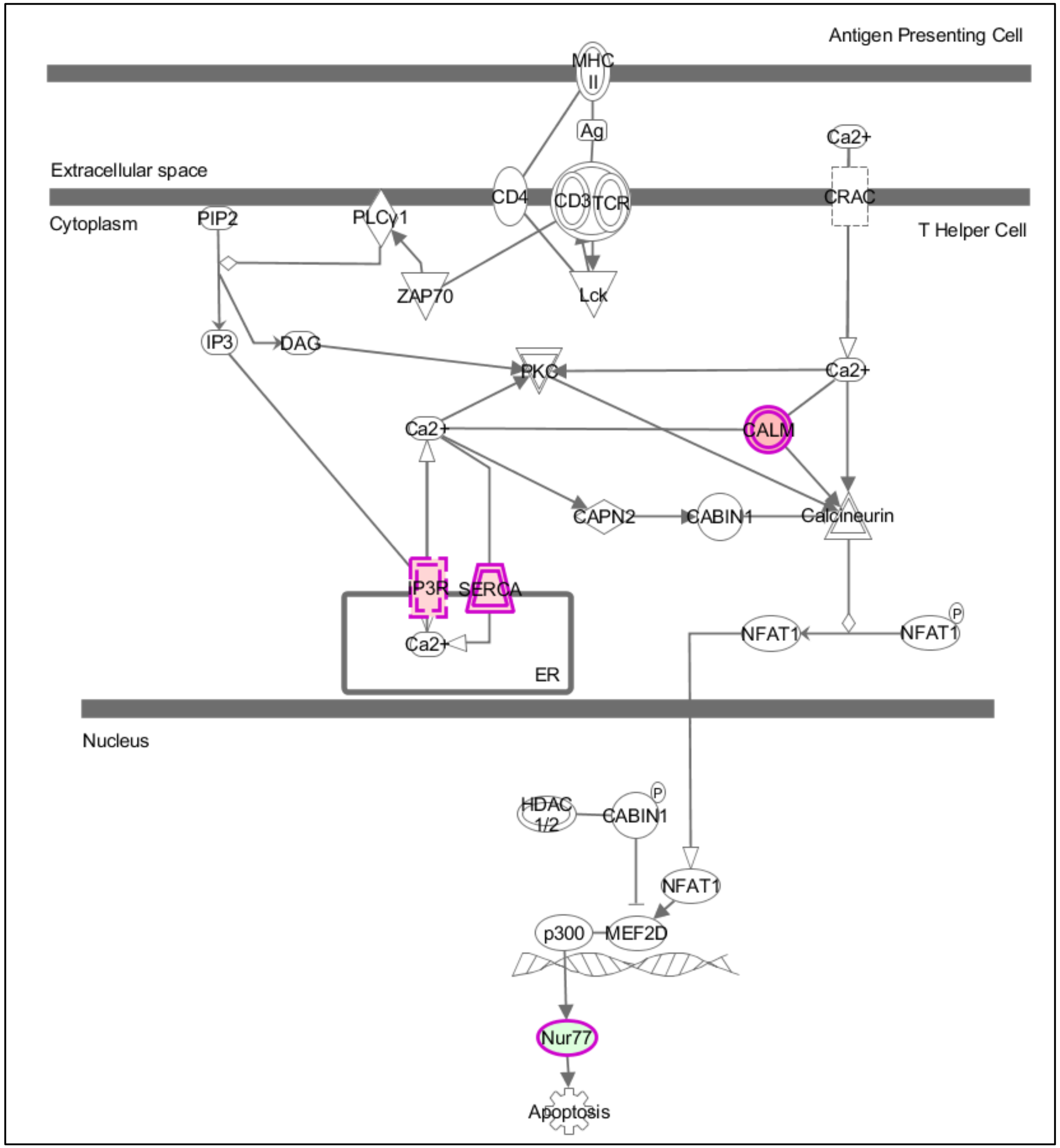

26-Hepatic Fibrosis/Hepatic Stellate Cell Activation

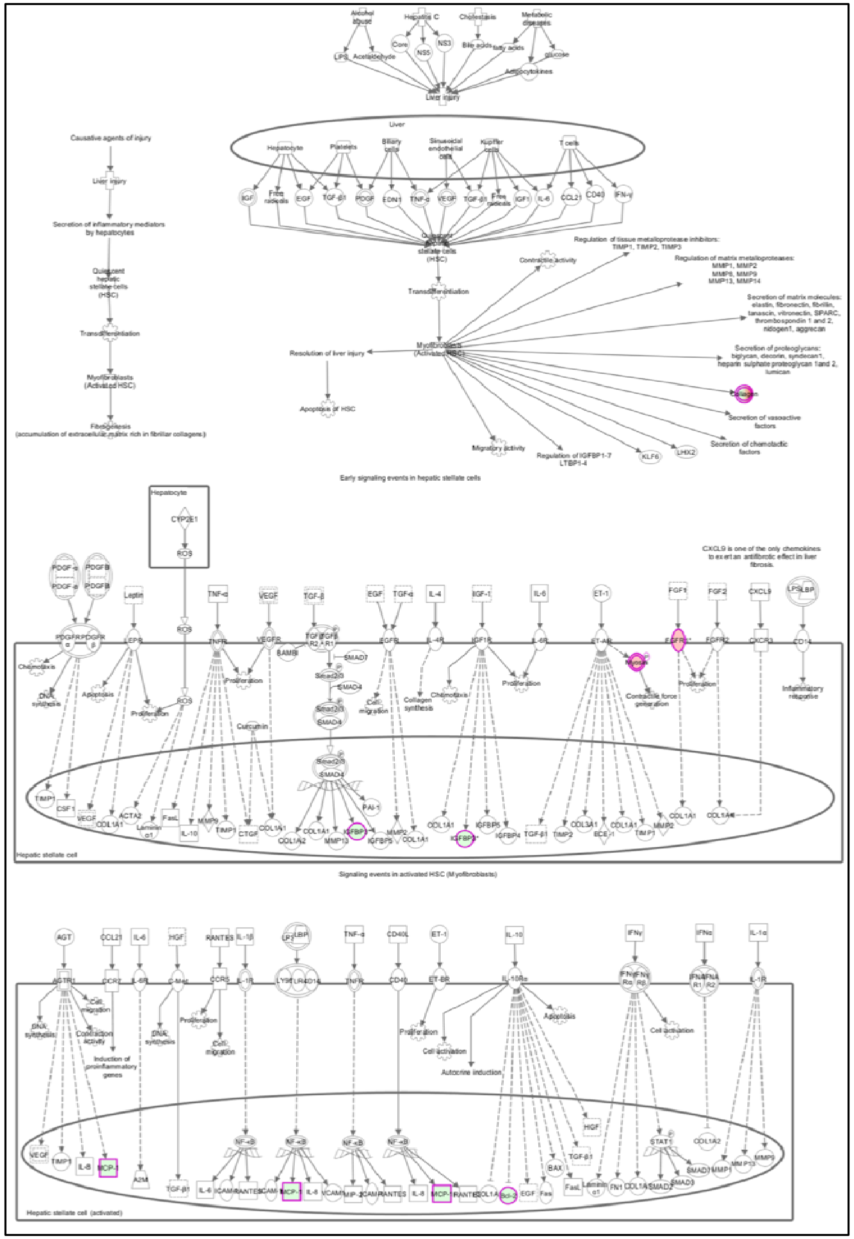

27-Gaq Signaling

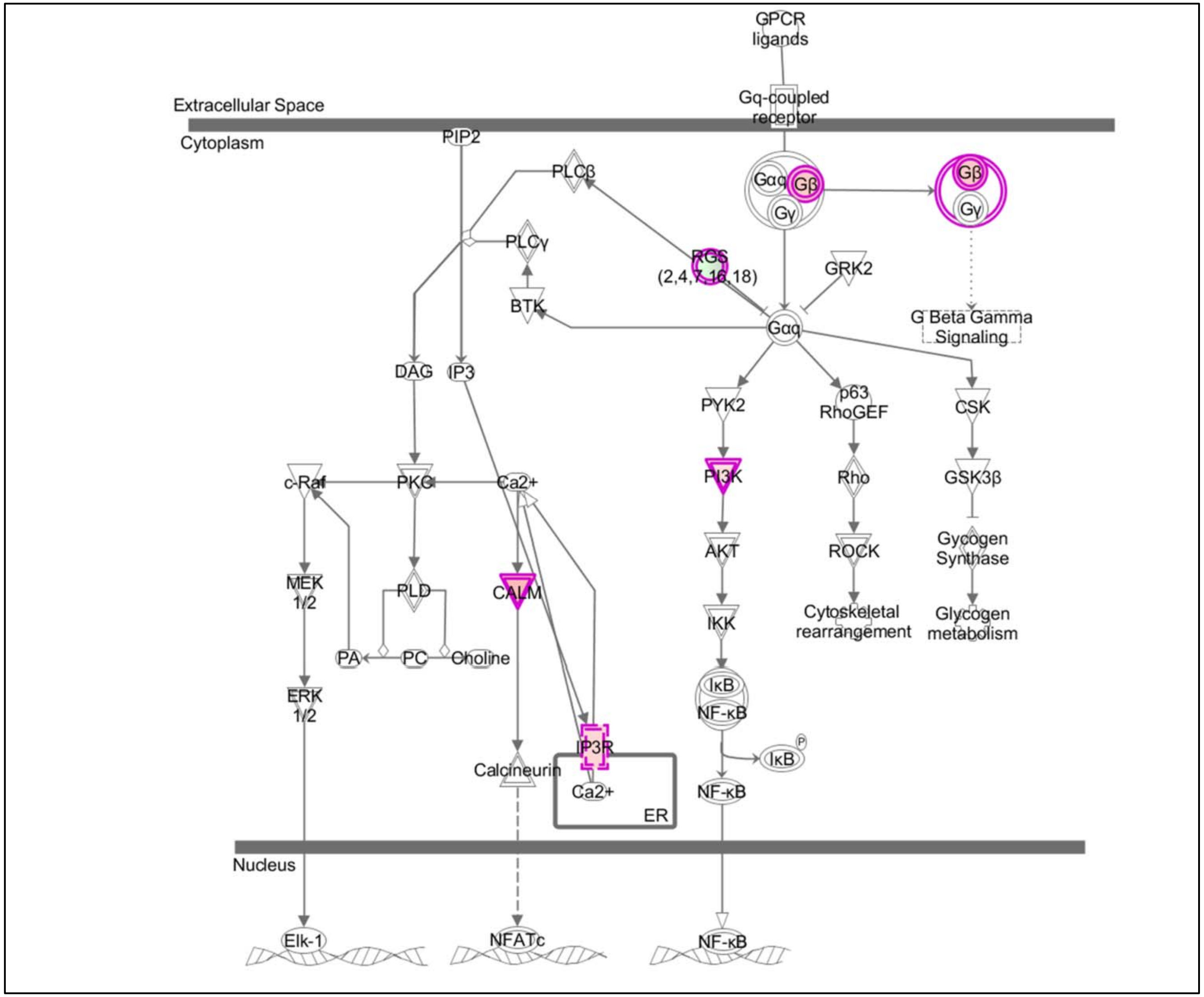

28-Role of MAPK Signaling in the Pathogenesis of Influenza

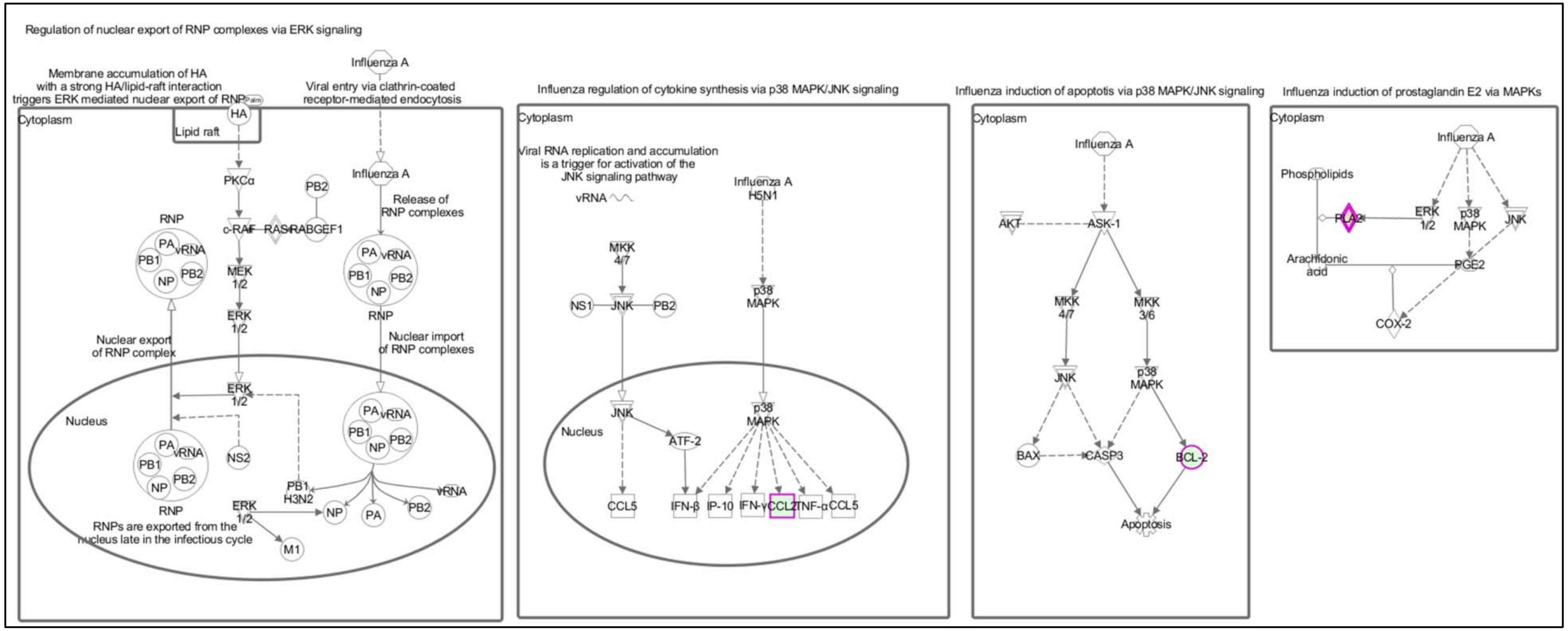

29-Calcium Transport Ⅰ

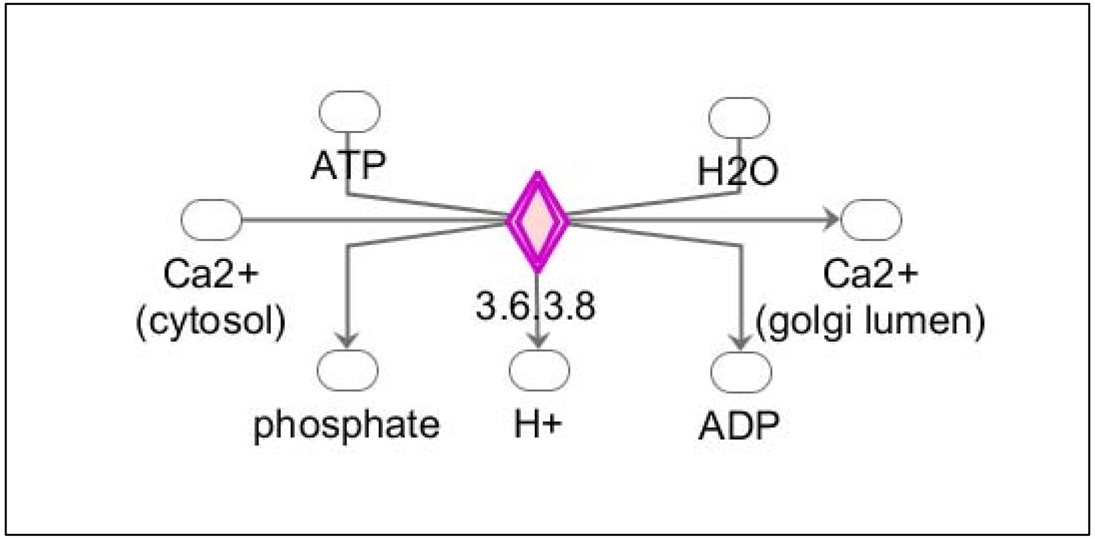

30-Ephrin B Signaling

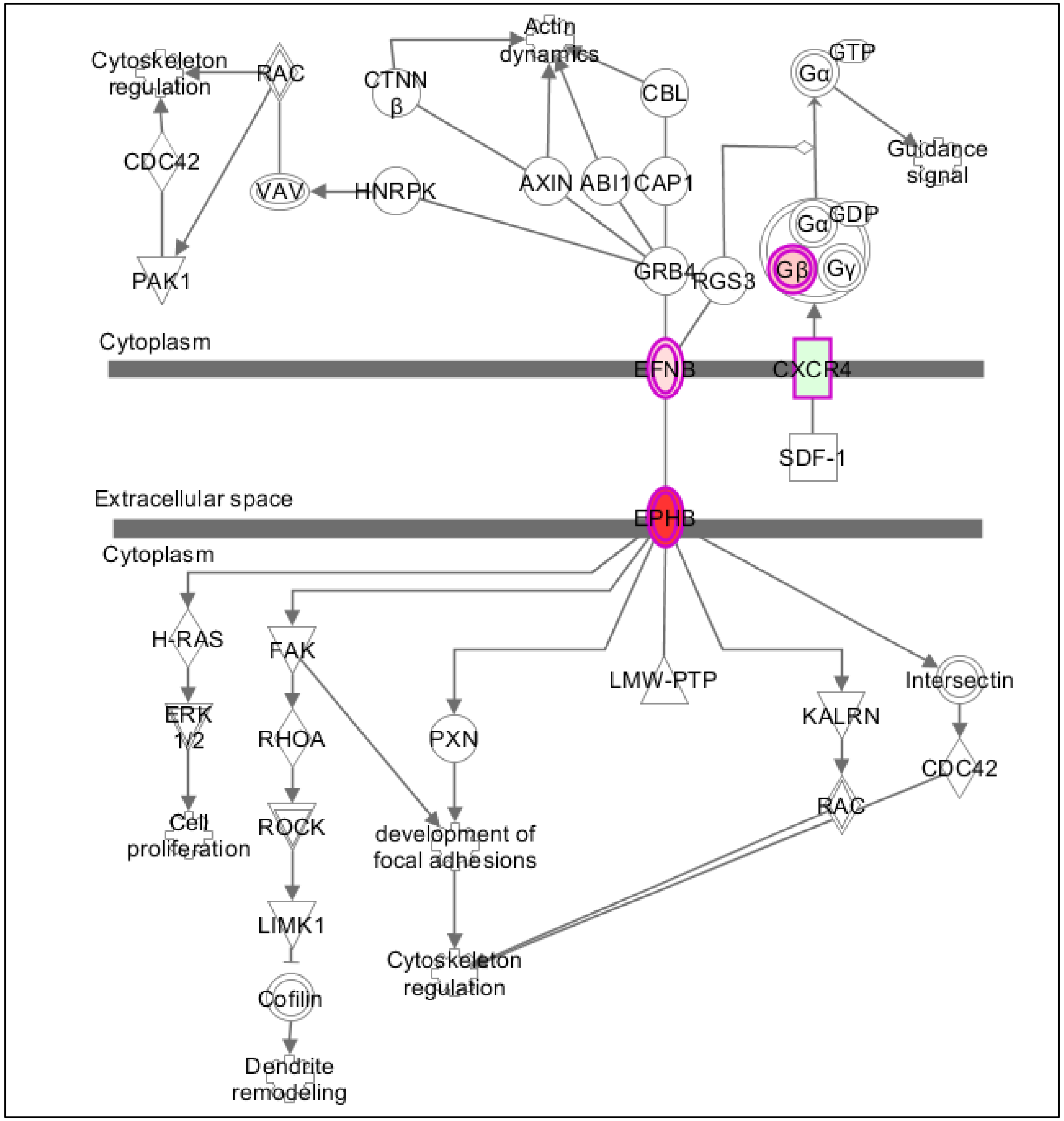

31-Chemokine Signaling

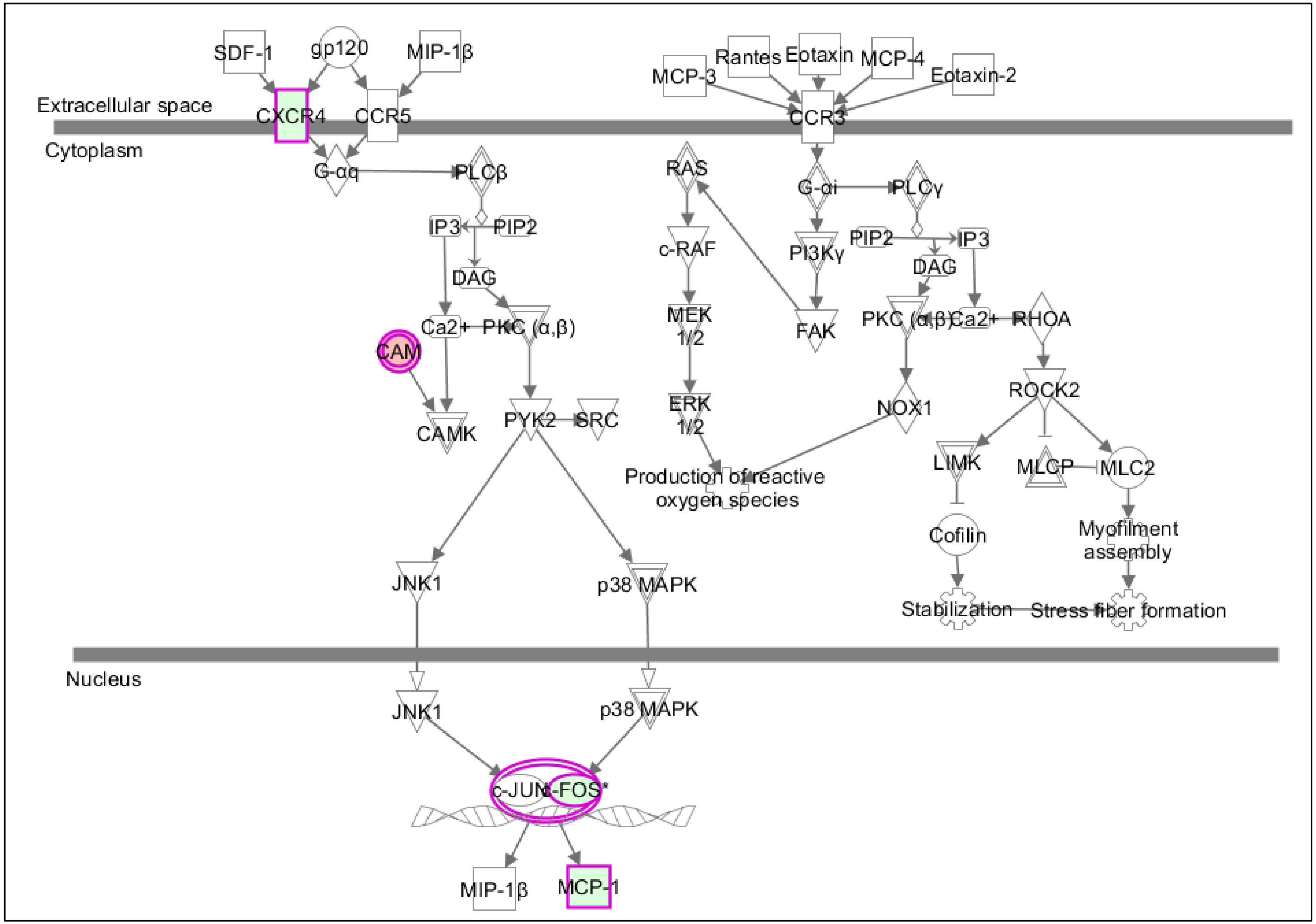

32-Corticotropin Releasing Hormone Signaling

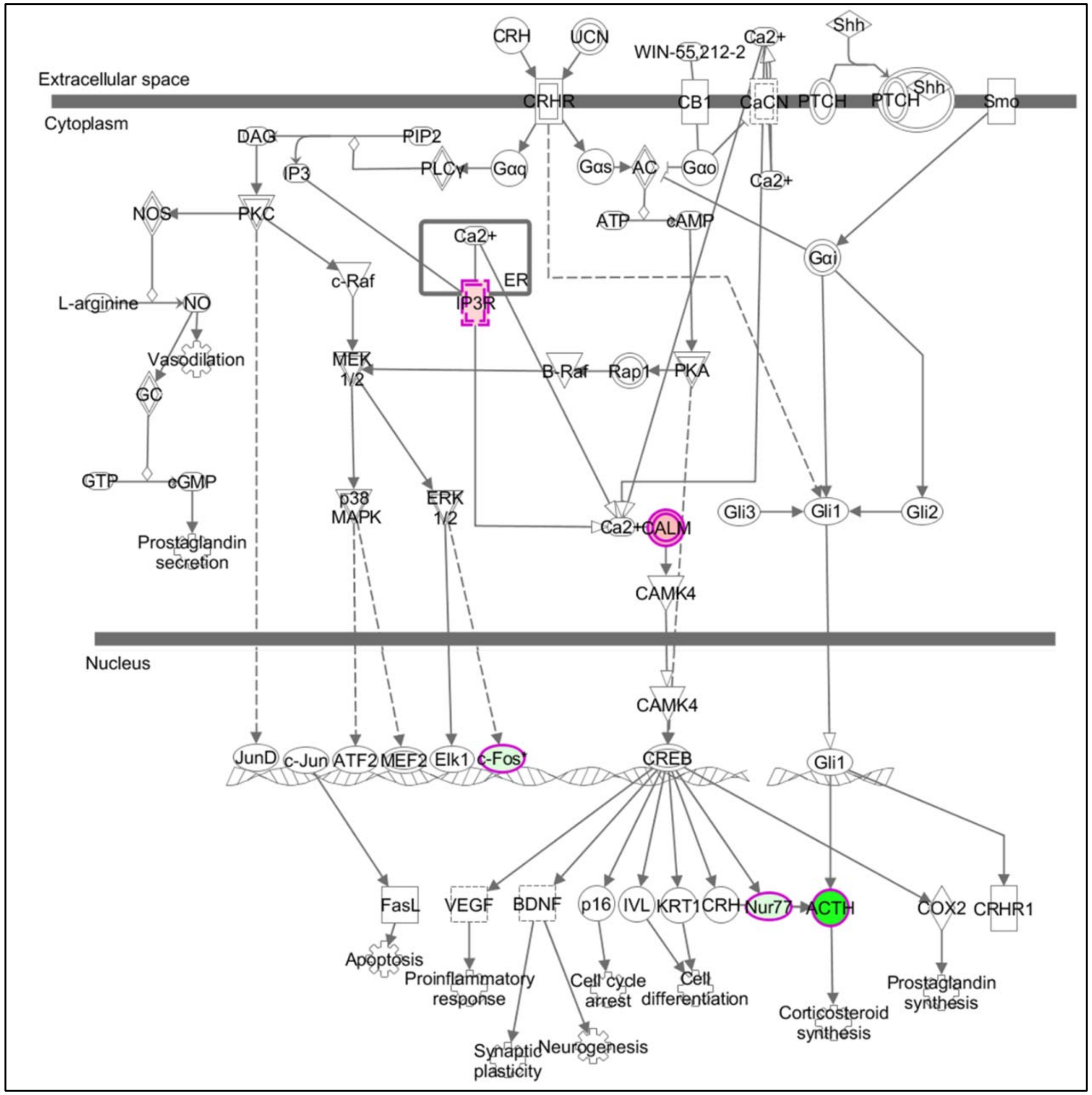

33-CD28 Signaling in T Helper Cells

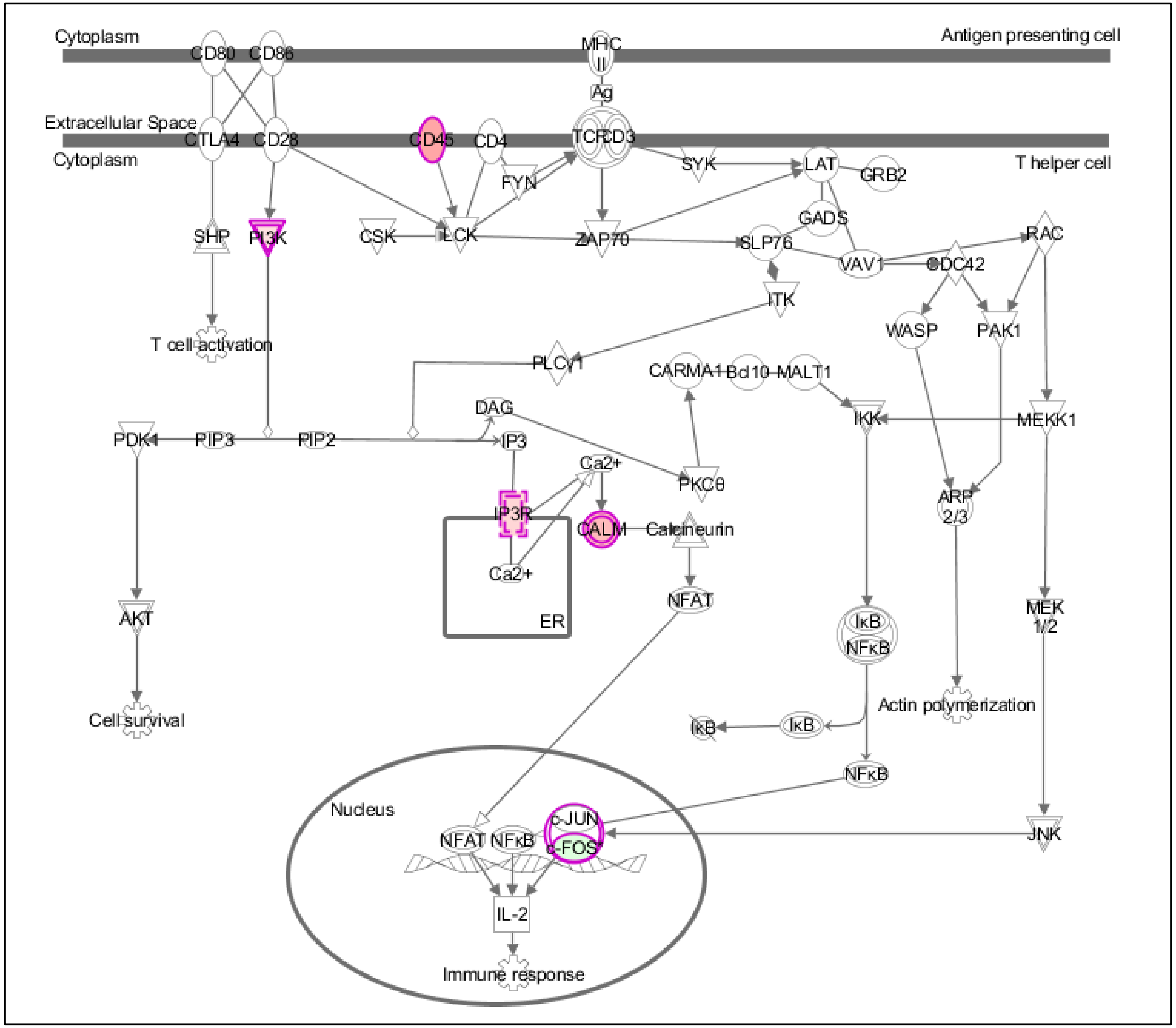

34-MIF Regulation of Innate Immunity

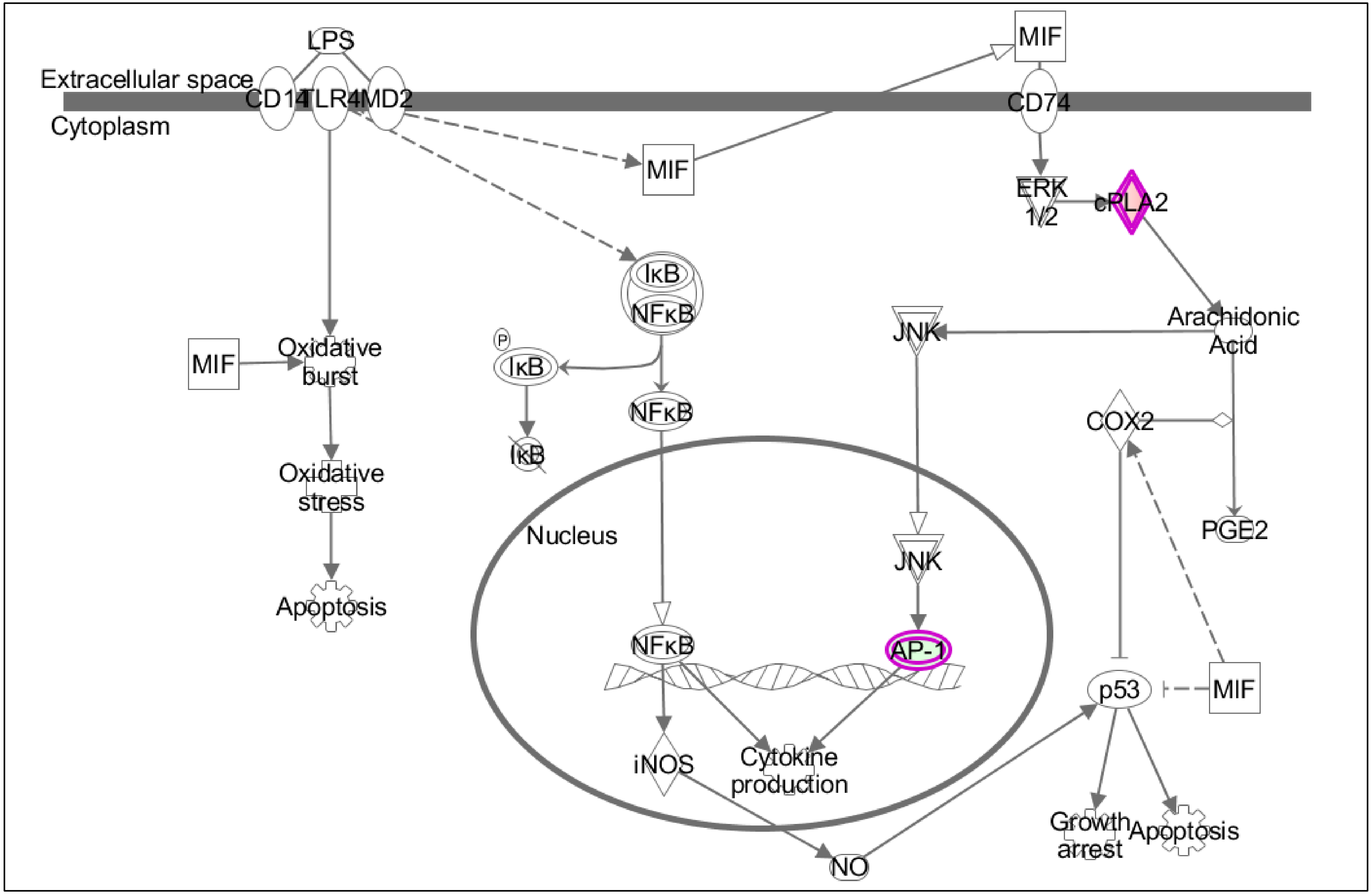

35-VEGF Family Ligand-Receptor Interaction

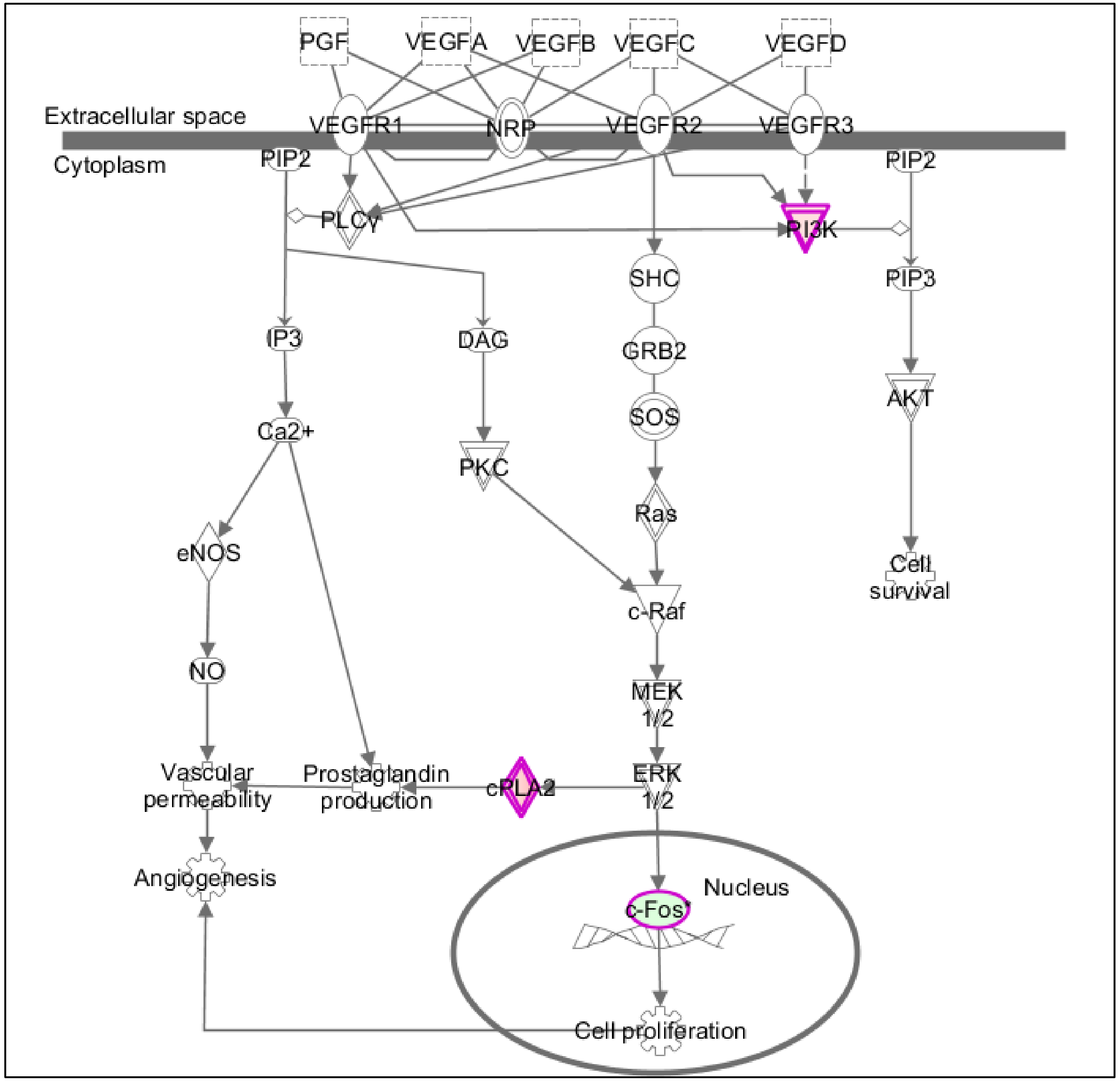

36-Docosahexaenoic Acid(DHA) Signaling

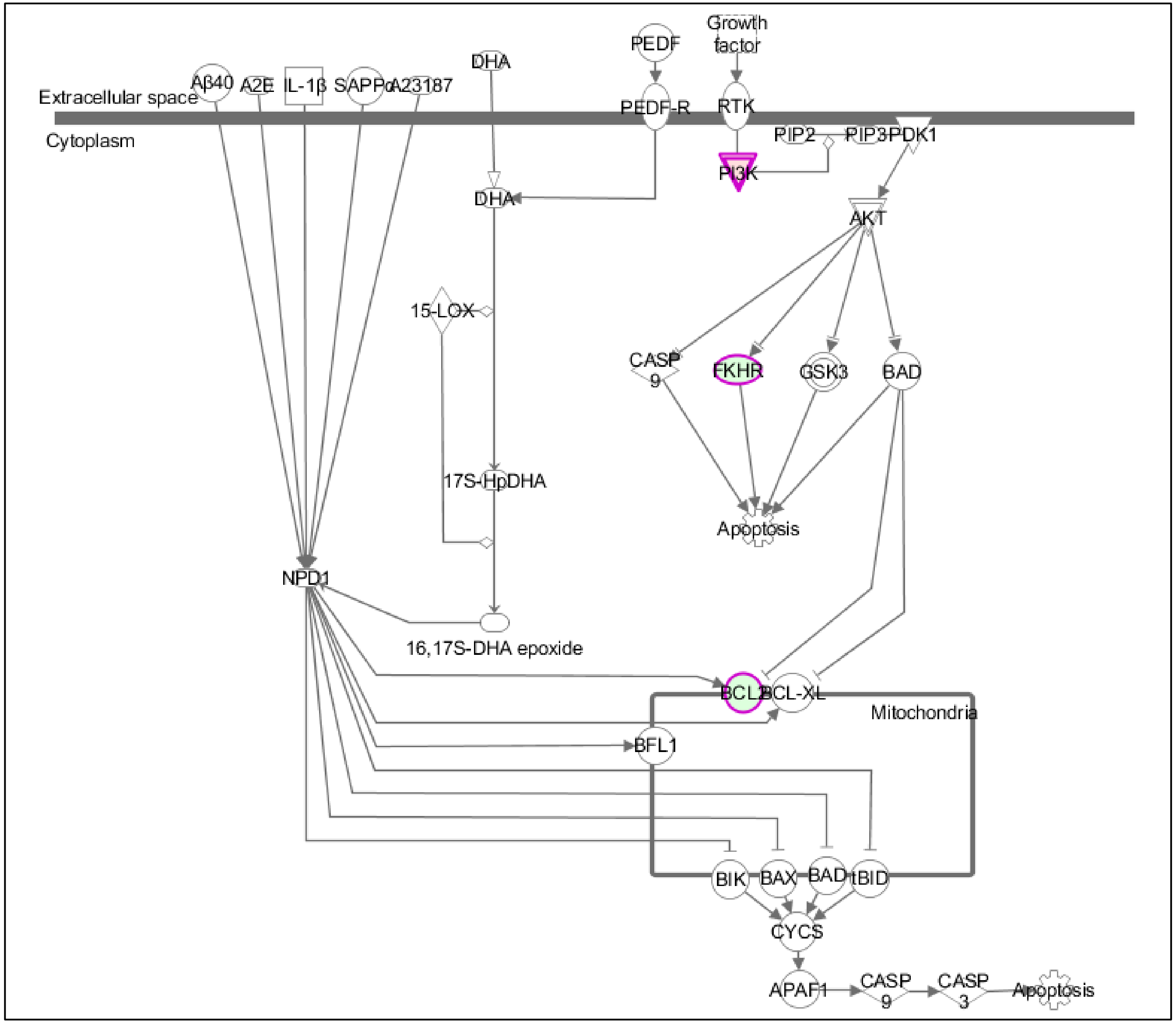

37-Regulation of the Epithelial-Mesenchymal Transition Pathway

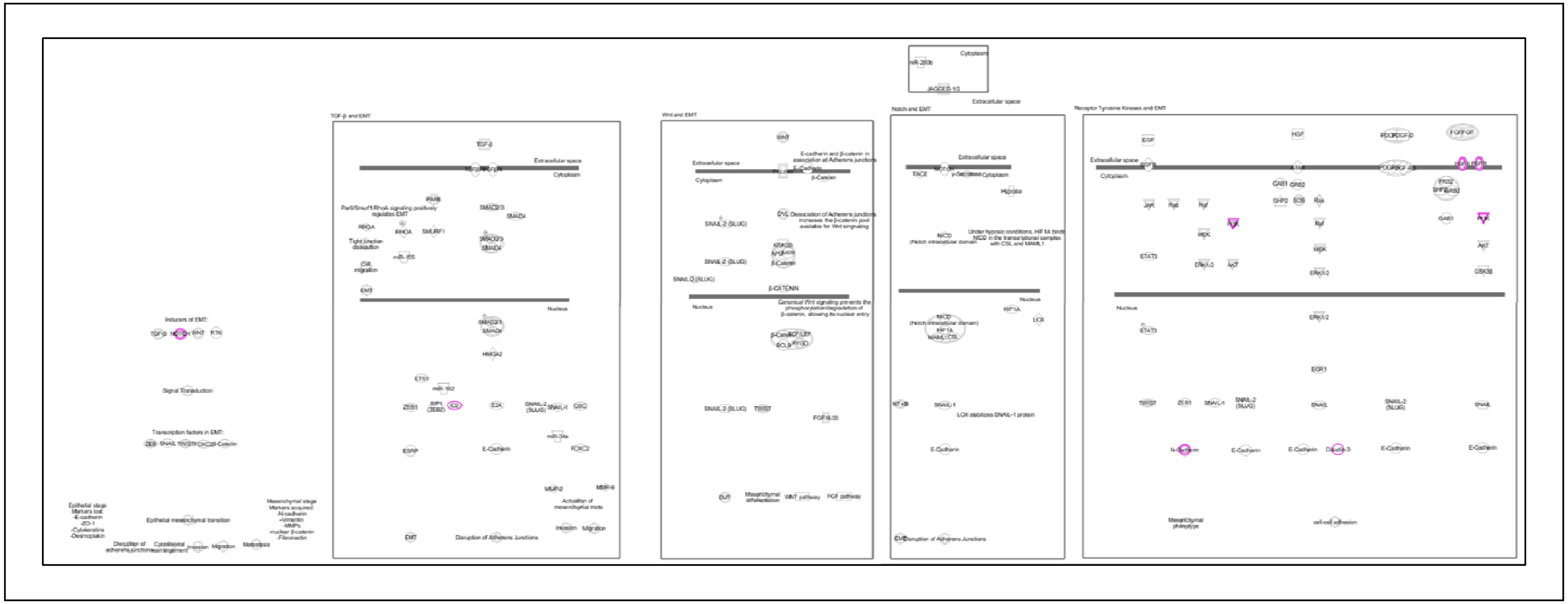

38-ERK/MAPK Signaling

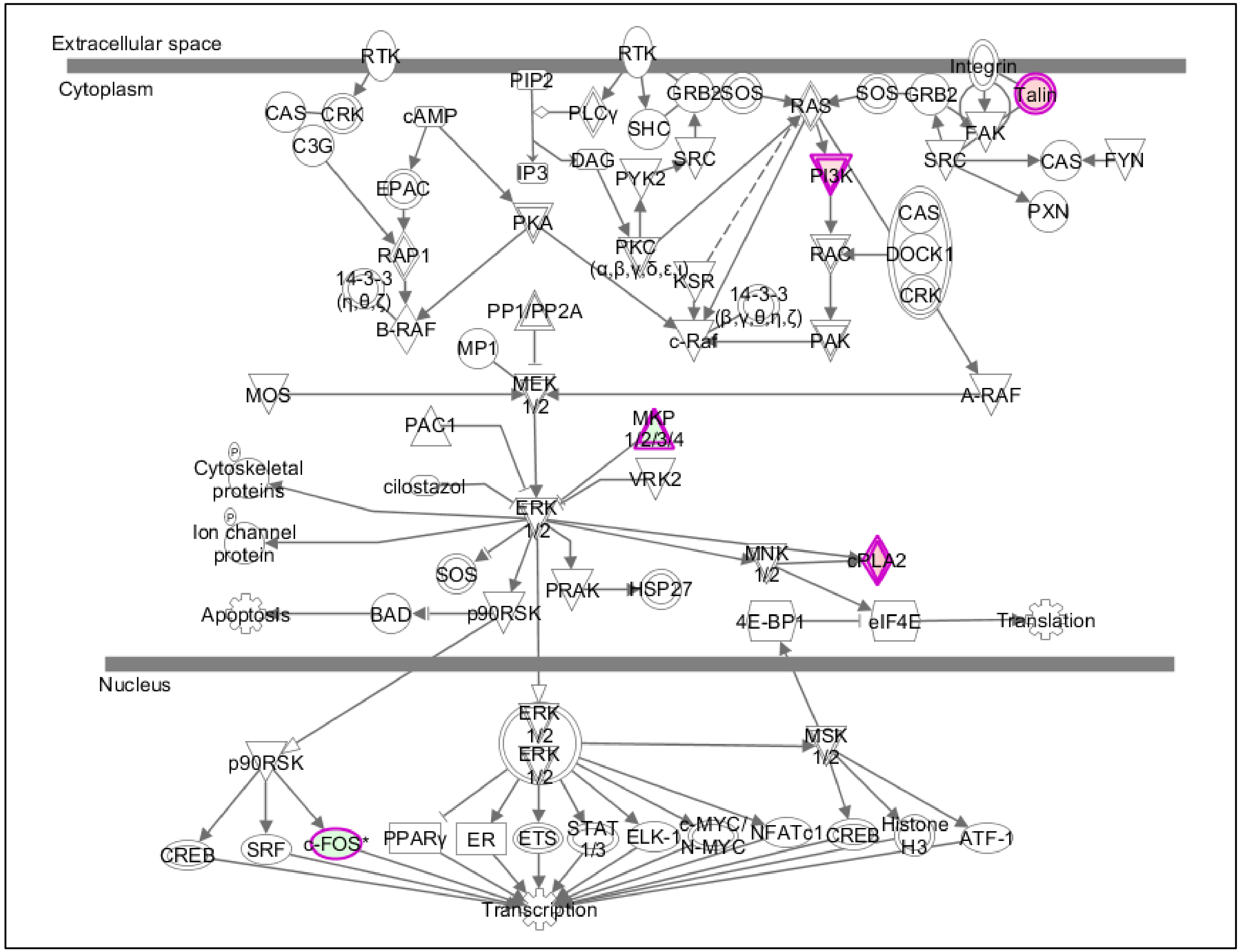

39- α -Adrenergic Signaling

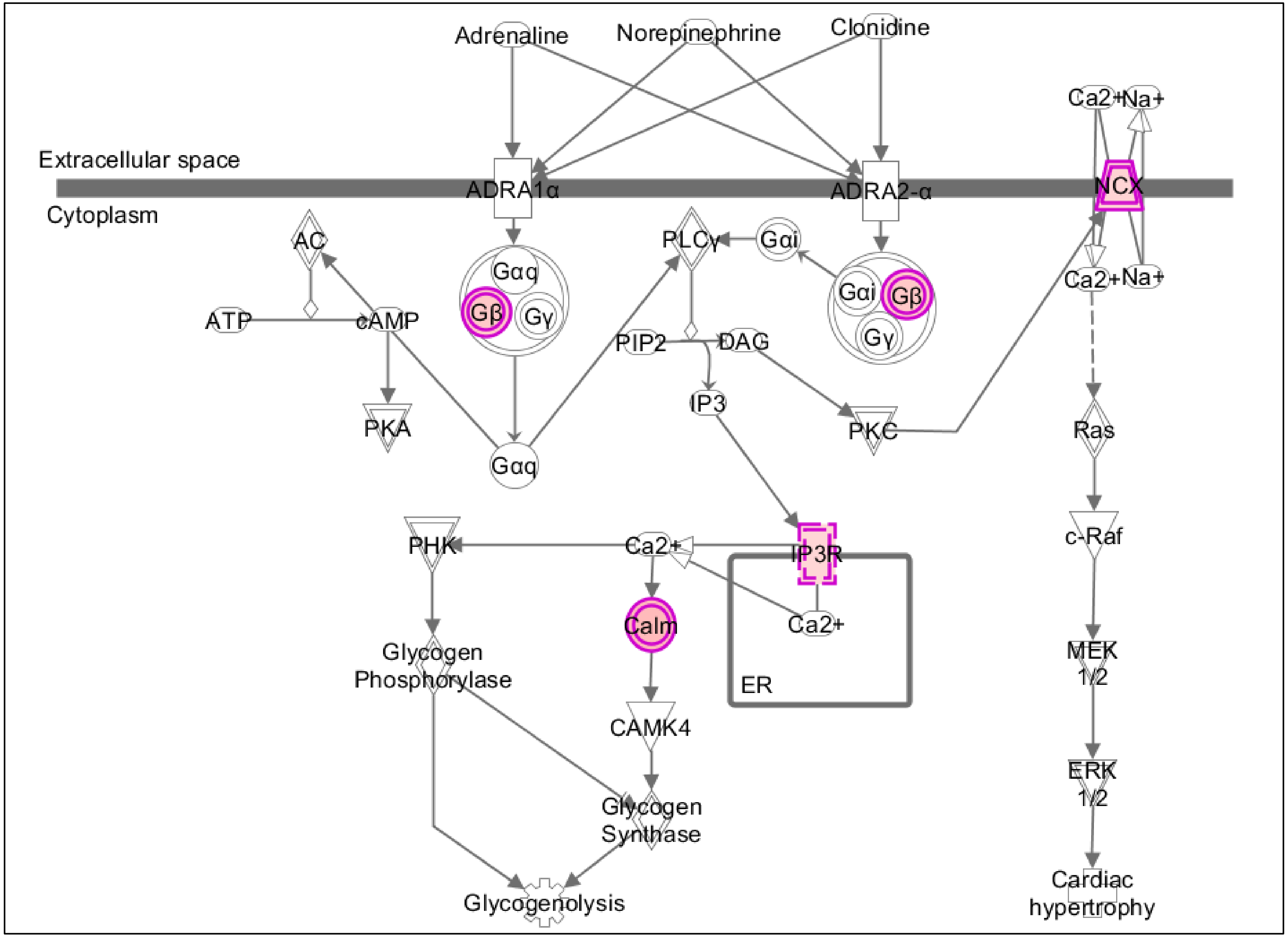

40-D-myo-inositol(1,4,5)-trisphosphate Degradation

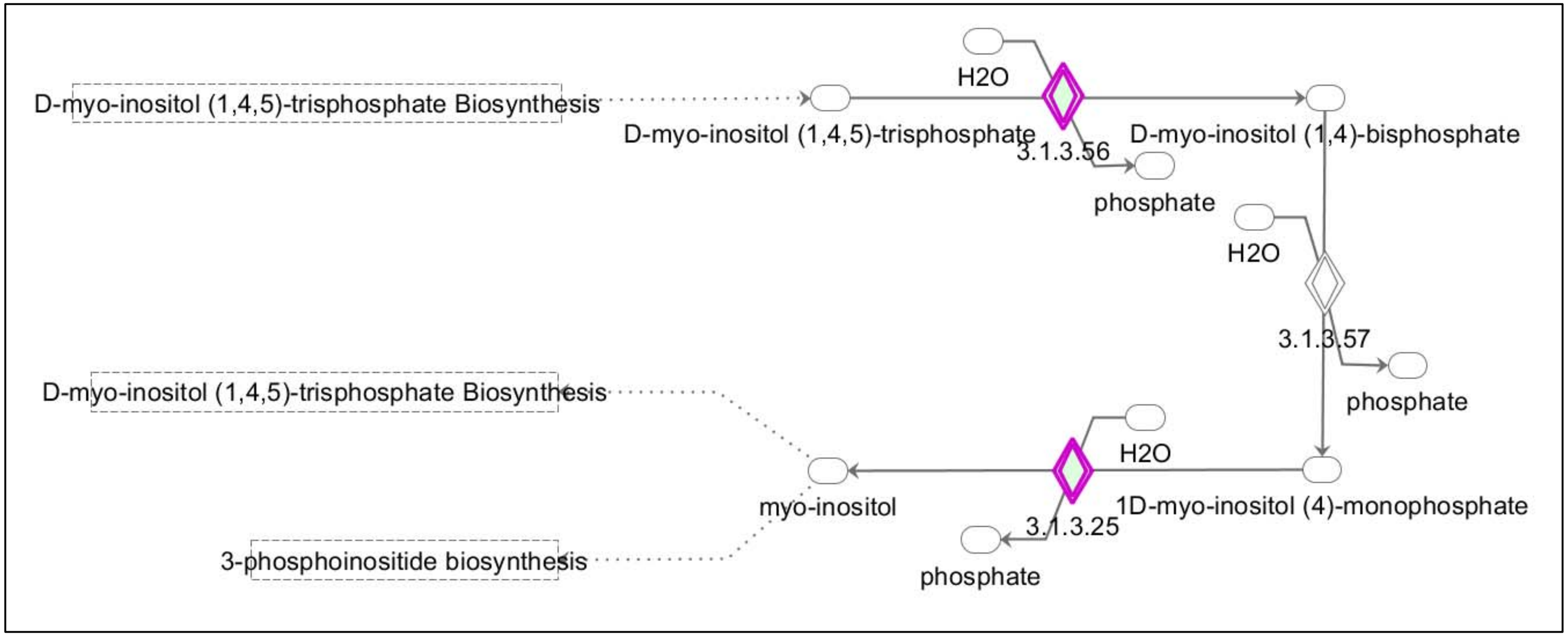

41-IGF-1 Signaling

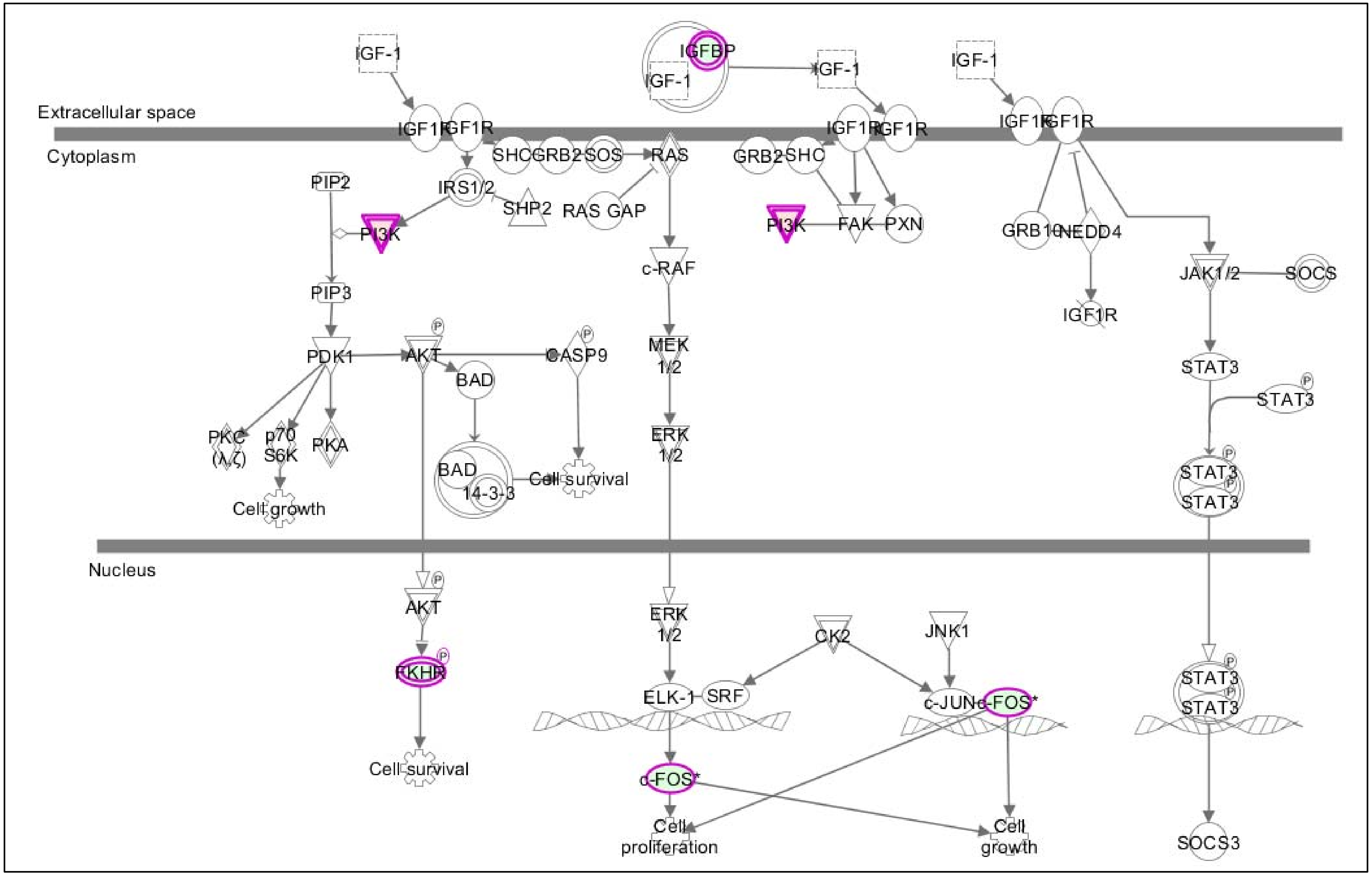

42-Epithelial Adherens Junction Signaling

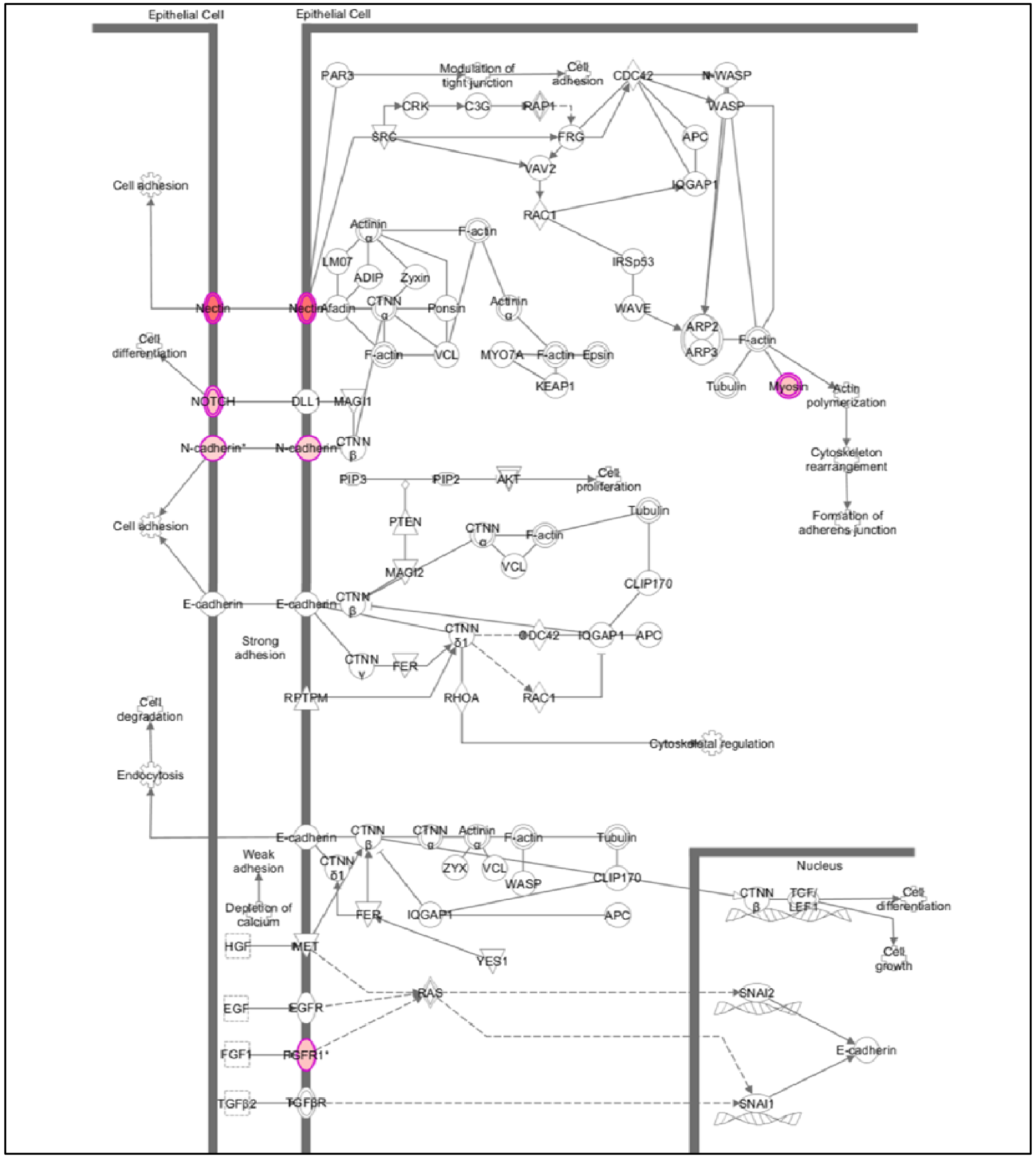

43-Polyamine Regulation in Colon Cancer

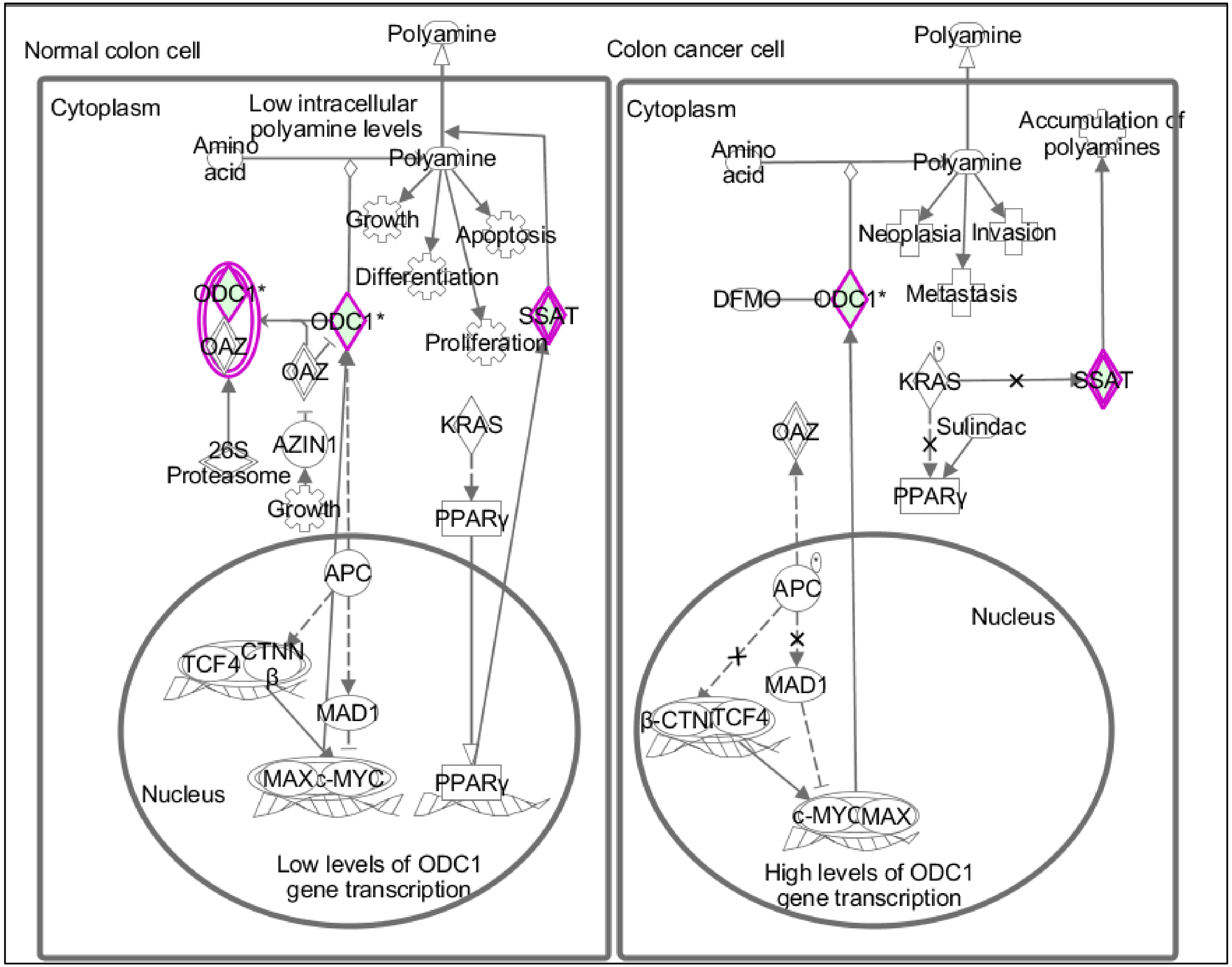

44-Nur77 Signaling in T Lymphocytes

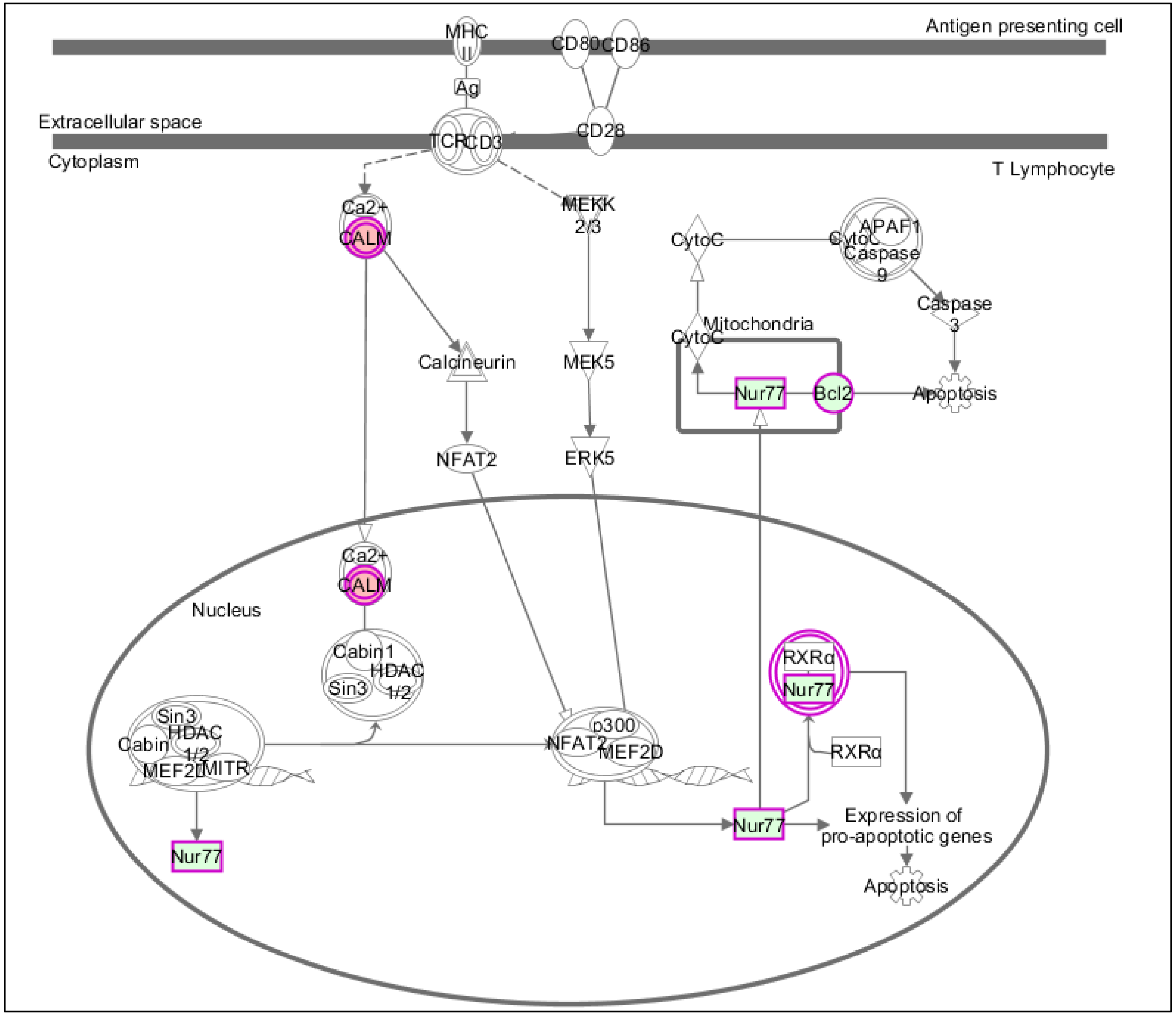

45-T Cell Receptor Signaling

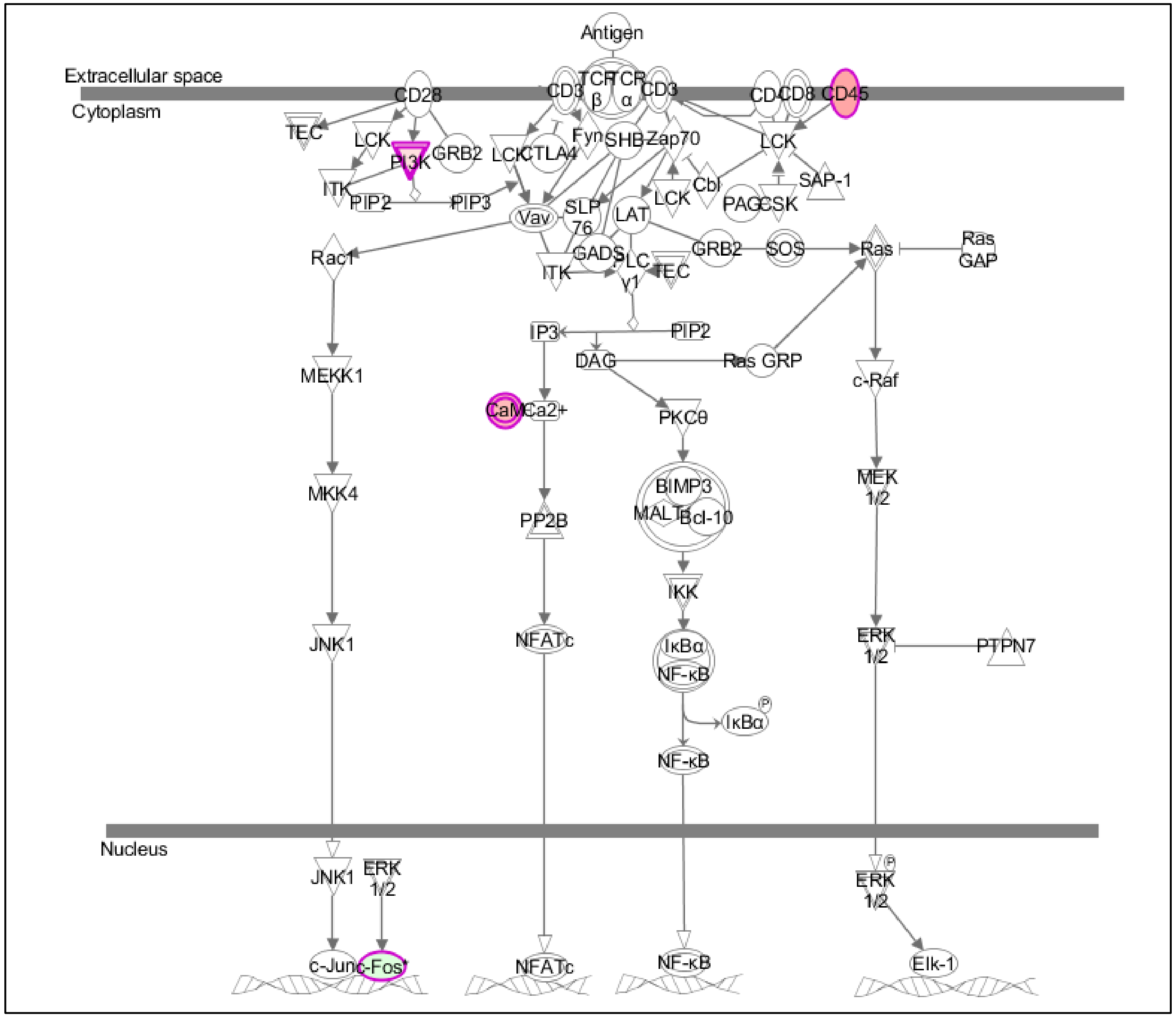

46-Leukocyte Extravasation Signaling

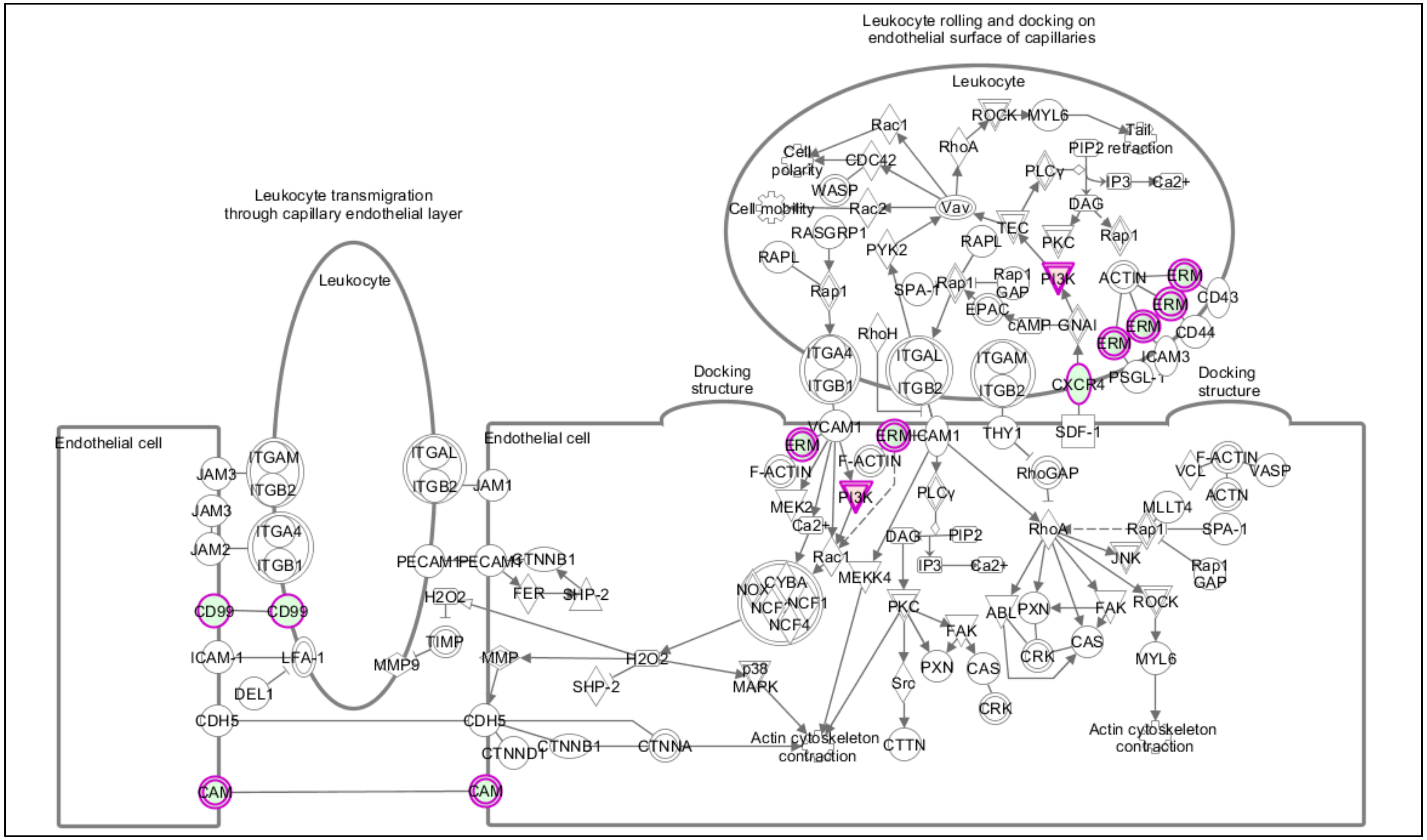

47-EGF Signaling

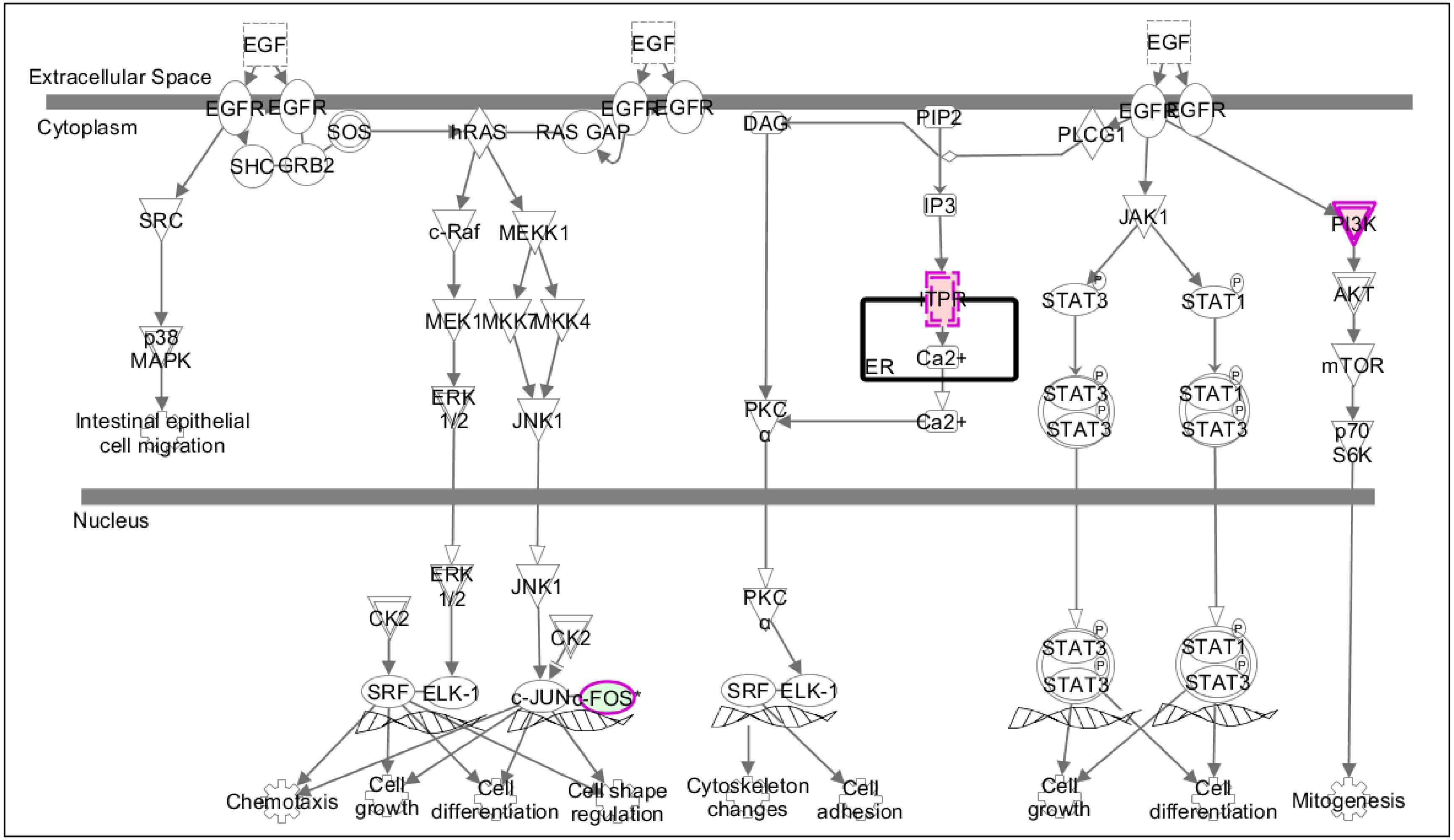

48-Cholecystokinin-Gastrin-mediated Signaling

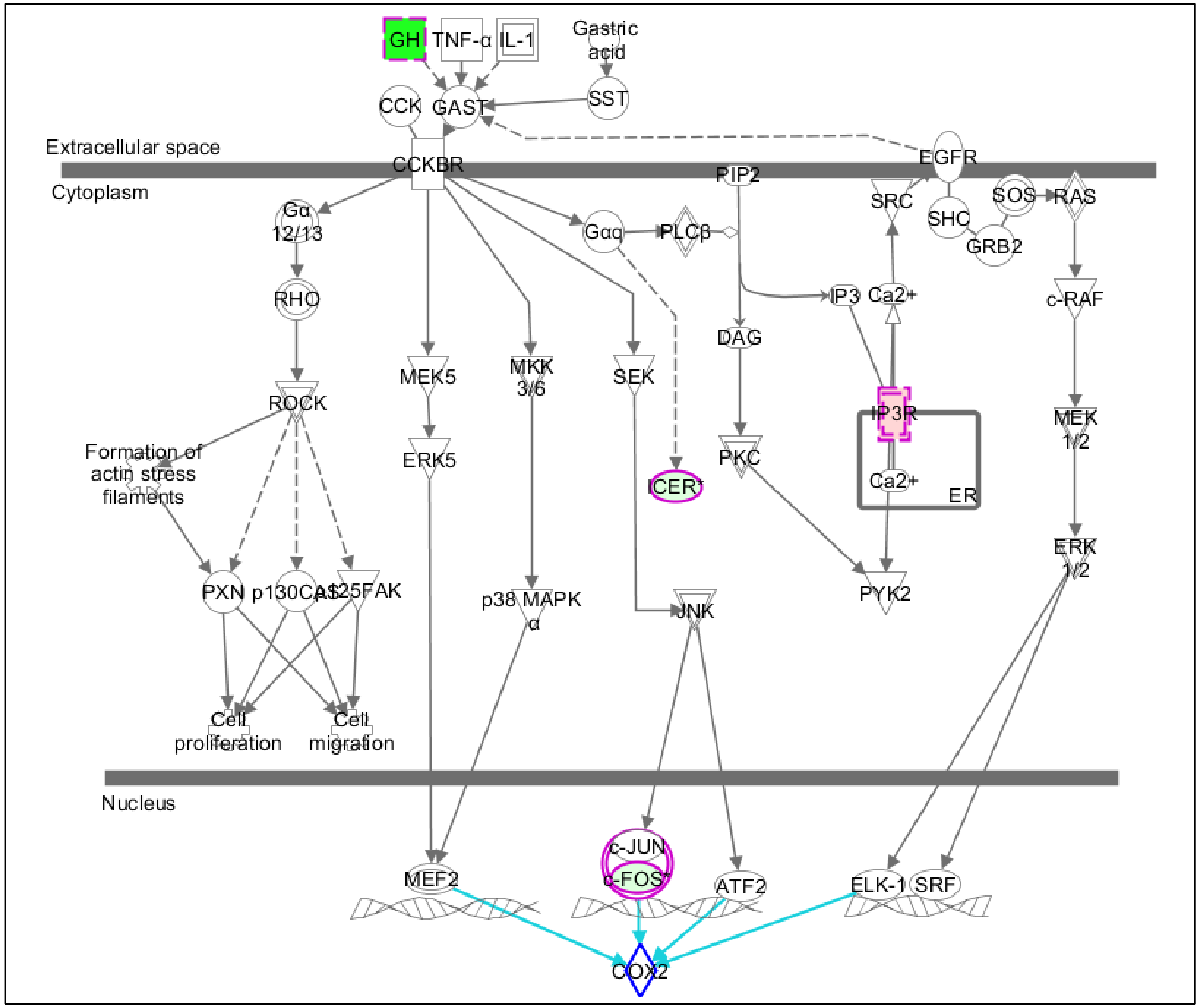

49-Glucocorticoid Receptor Signaling

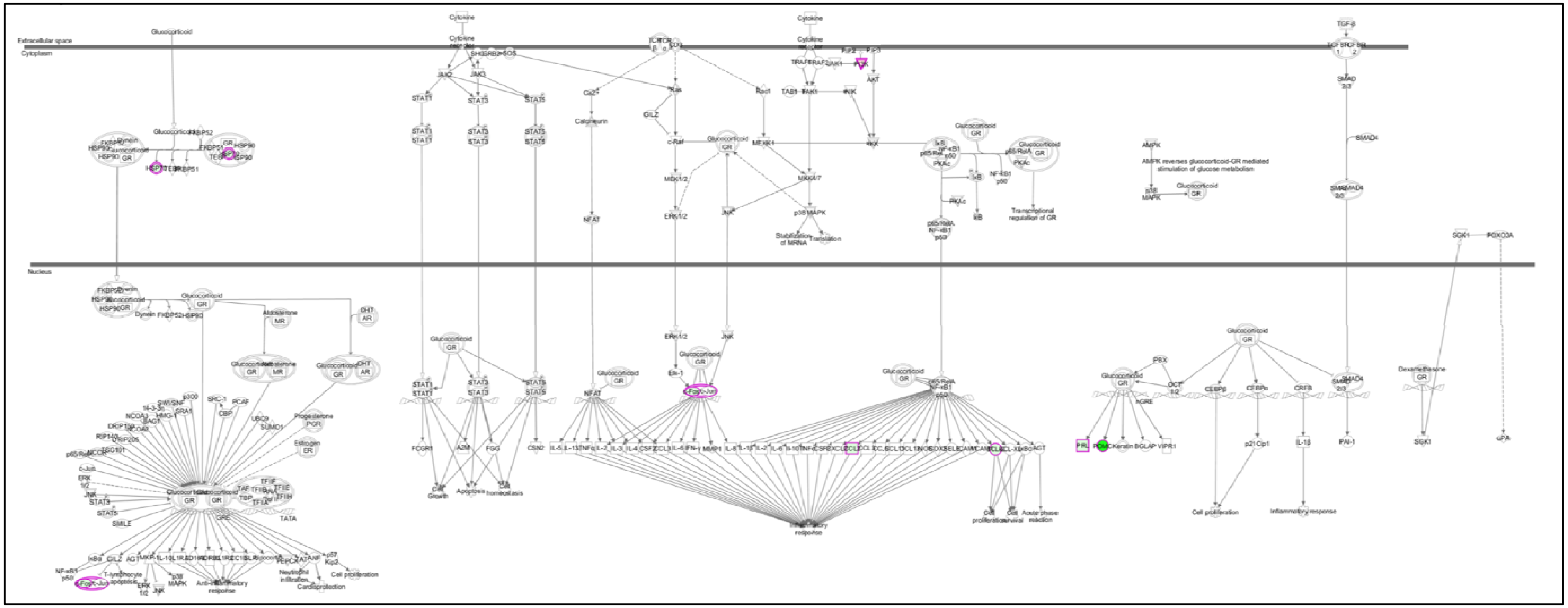

50-CXCR4 Signaling

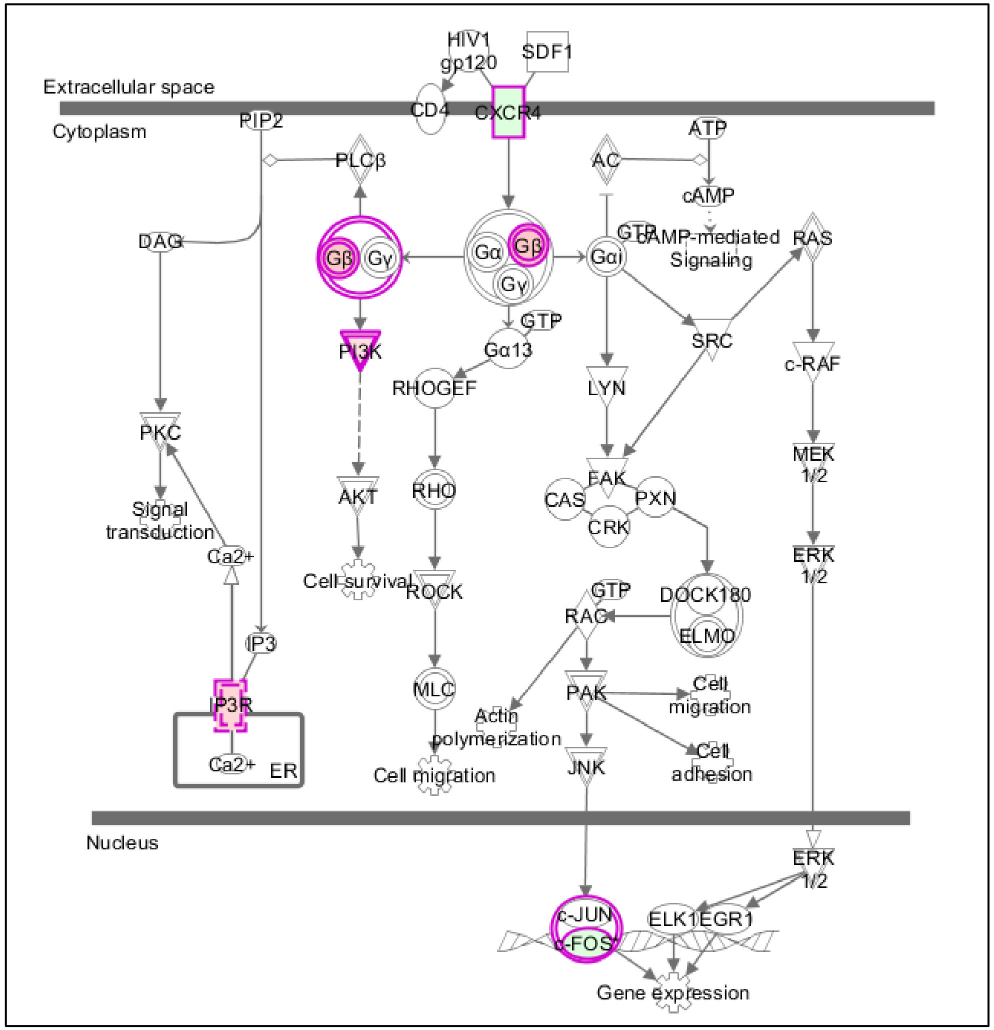

51-Nitric Oxide Signaling in the Cardiovascular System

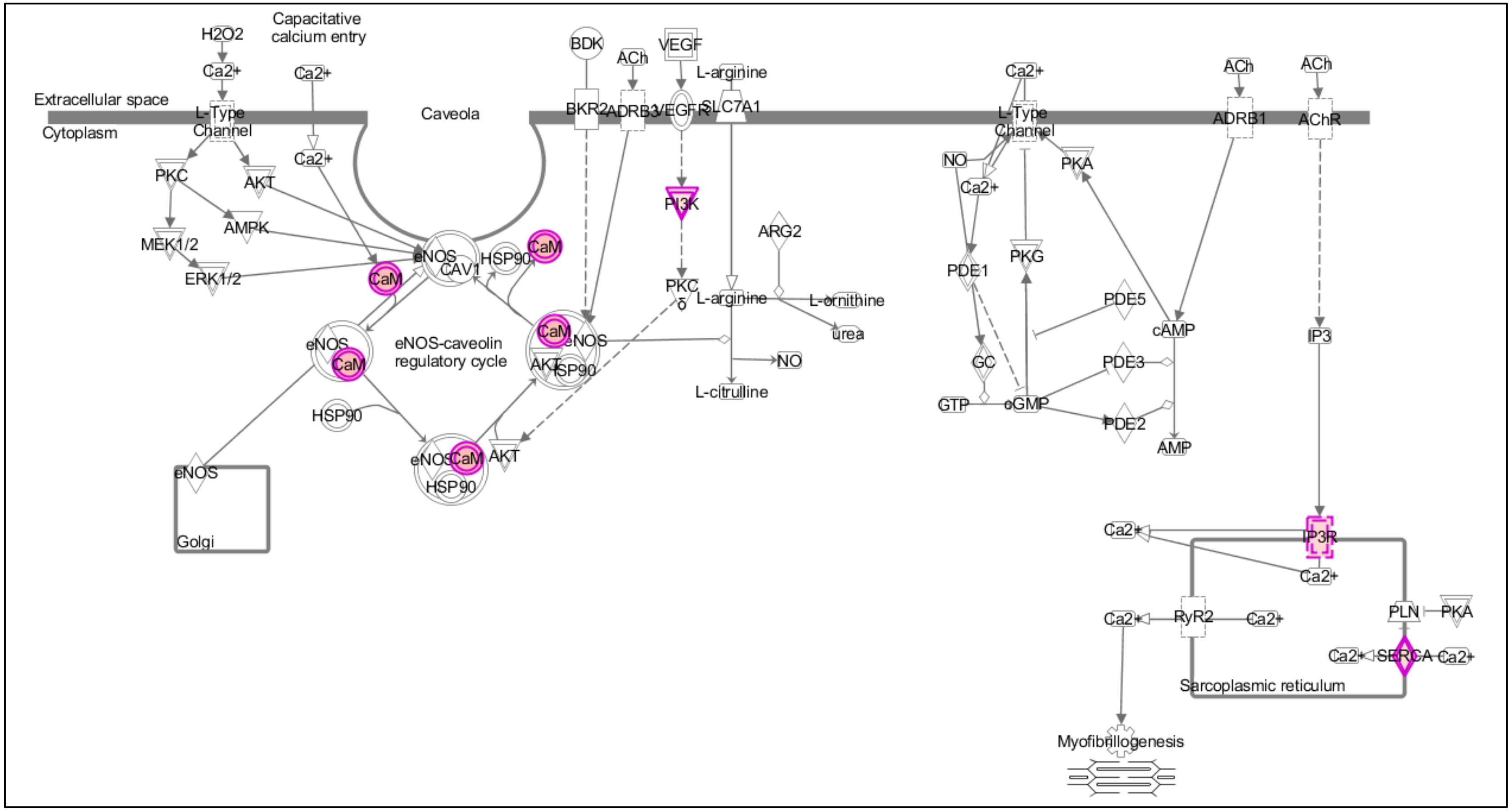

52-iCOS-iCOSL Signaling in T Helper Cells

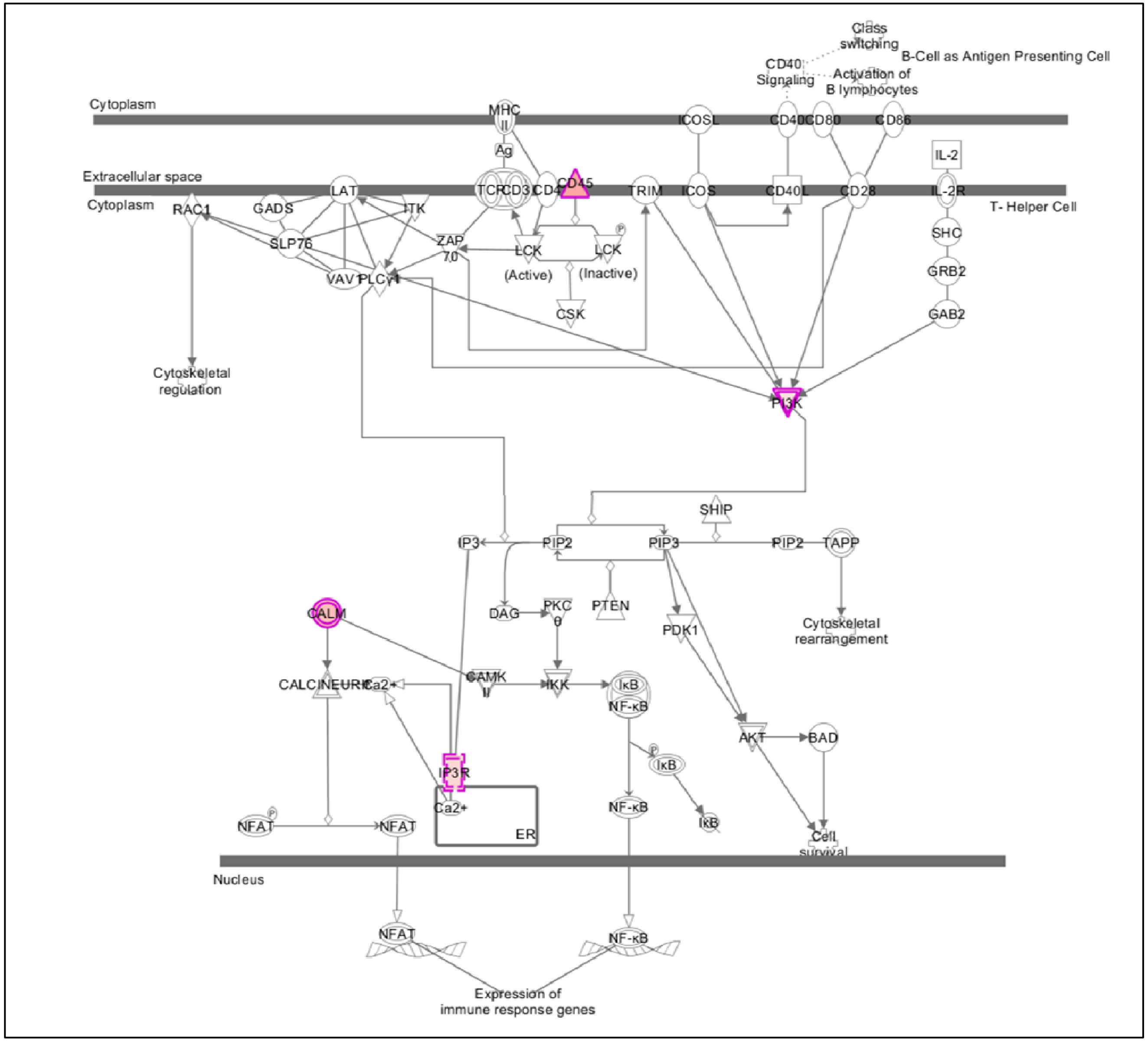

53-Tight Junction Signaling

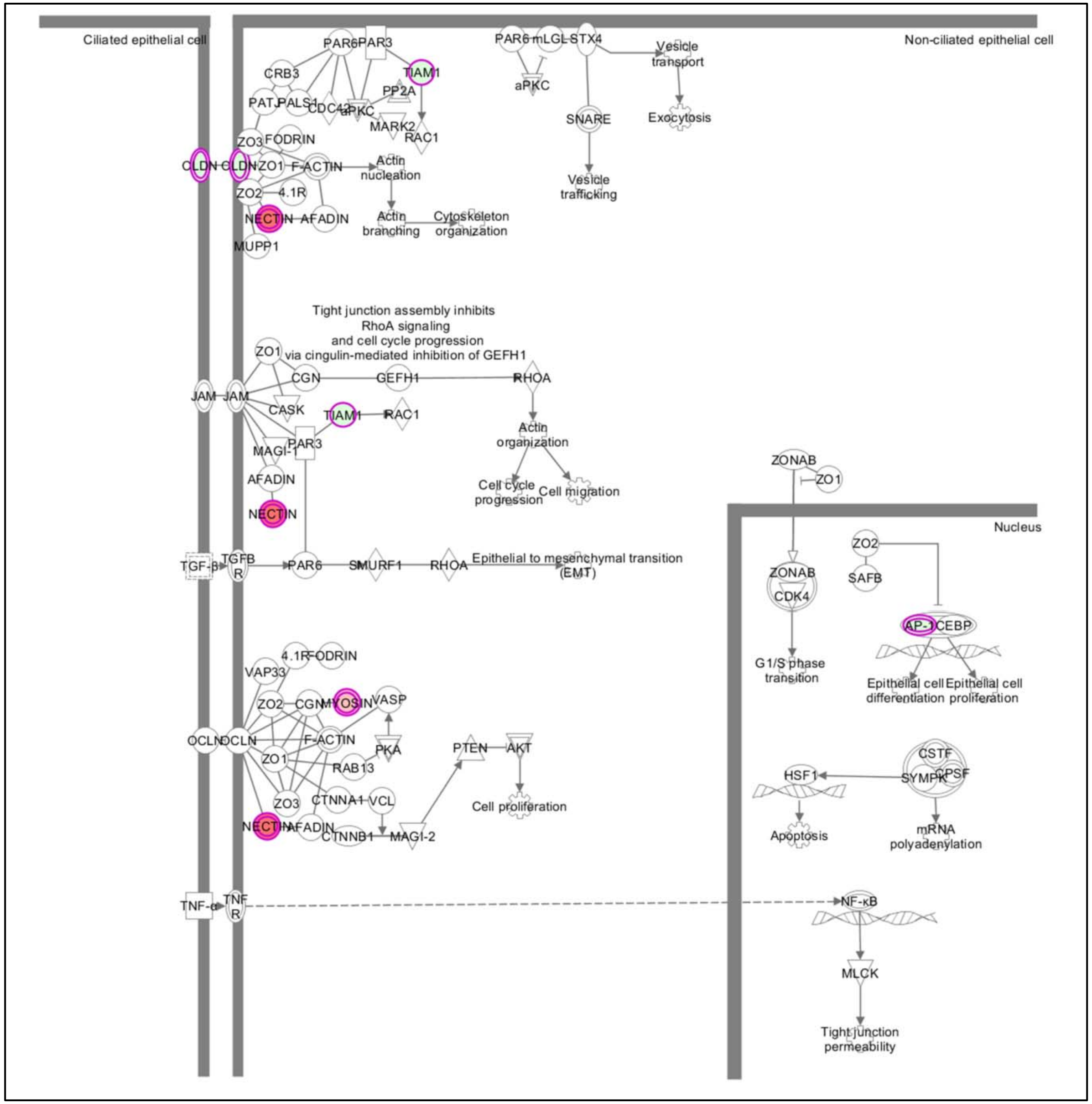

54-Fc Epsilon RI Signaling

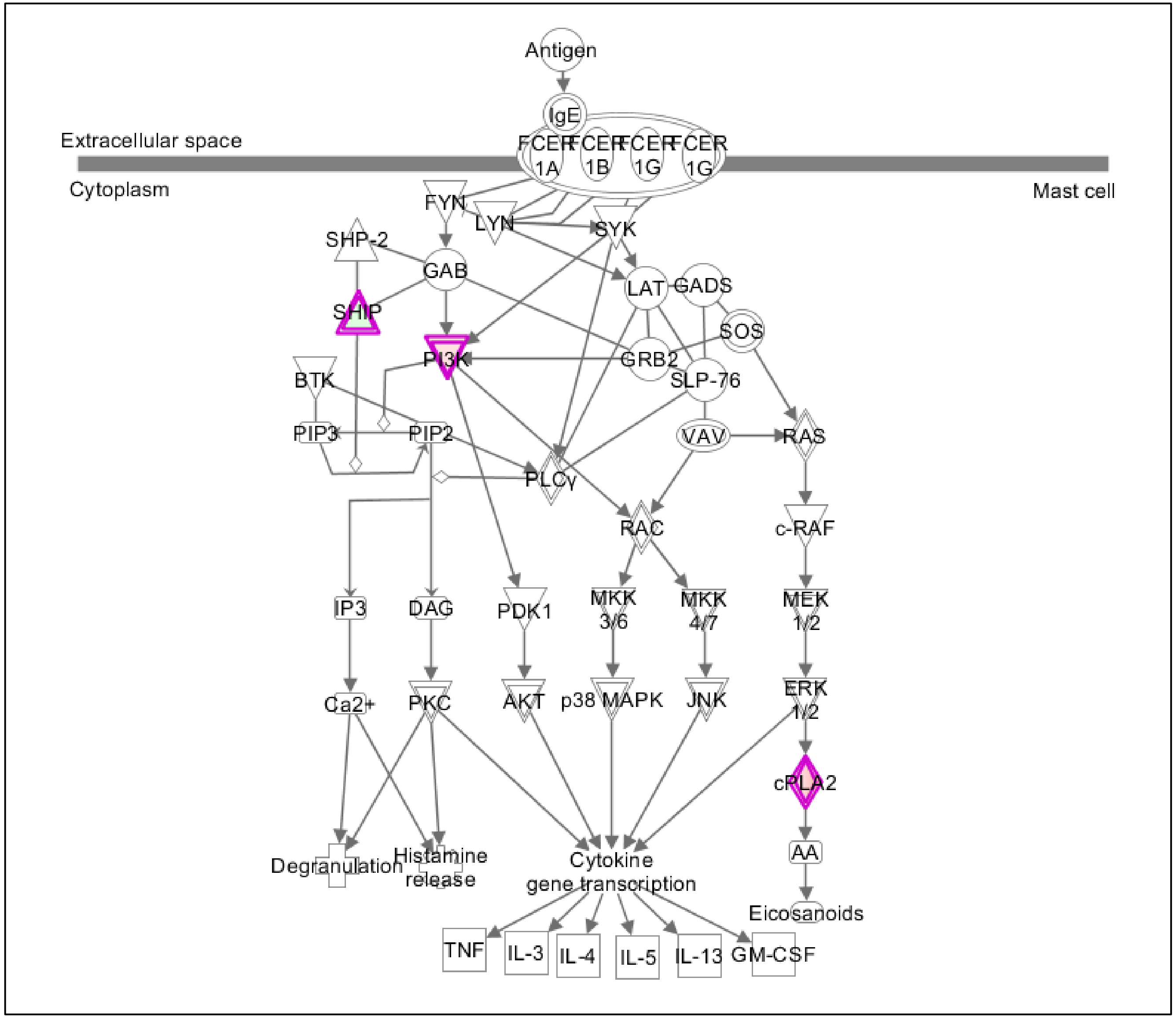

55-fMLP Signaling in Neutrophils

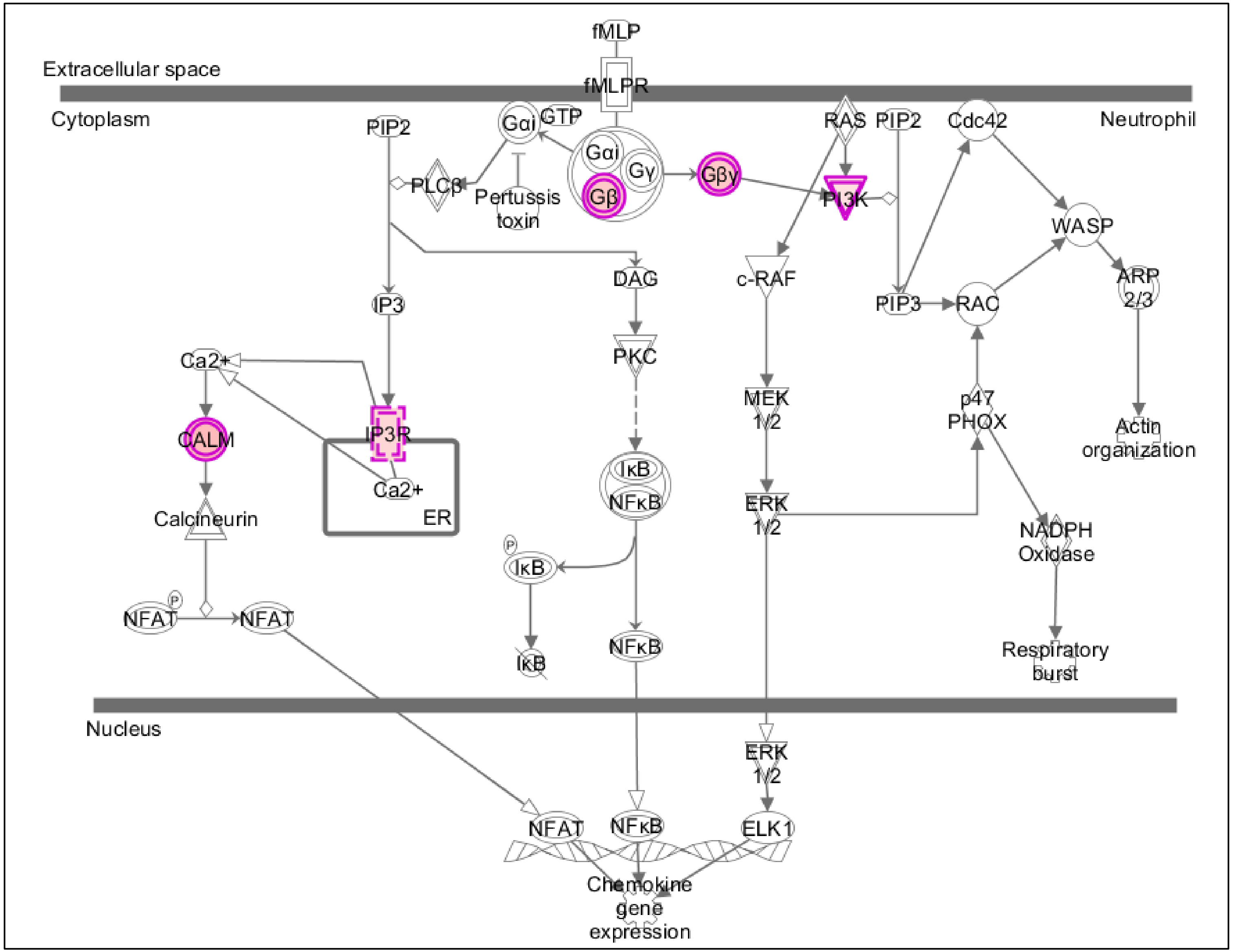

56-Angiopoietin Signaling

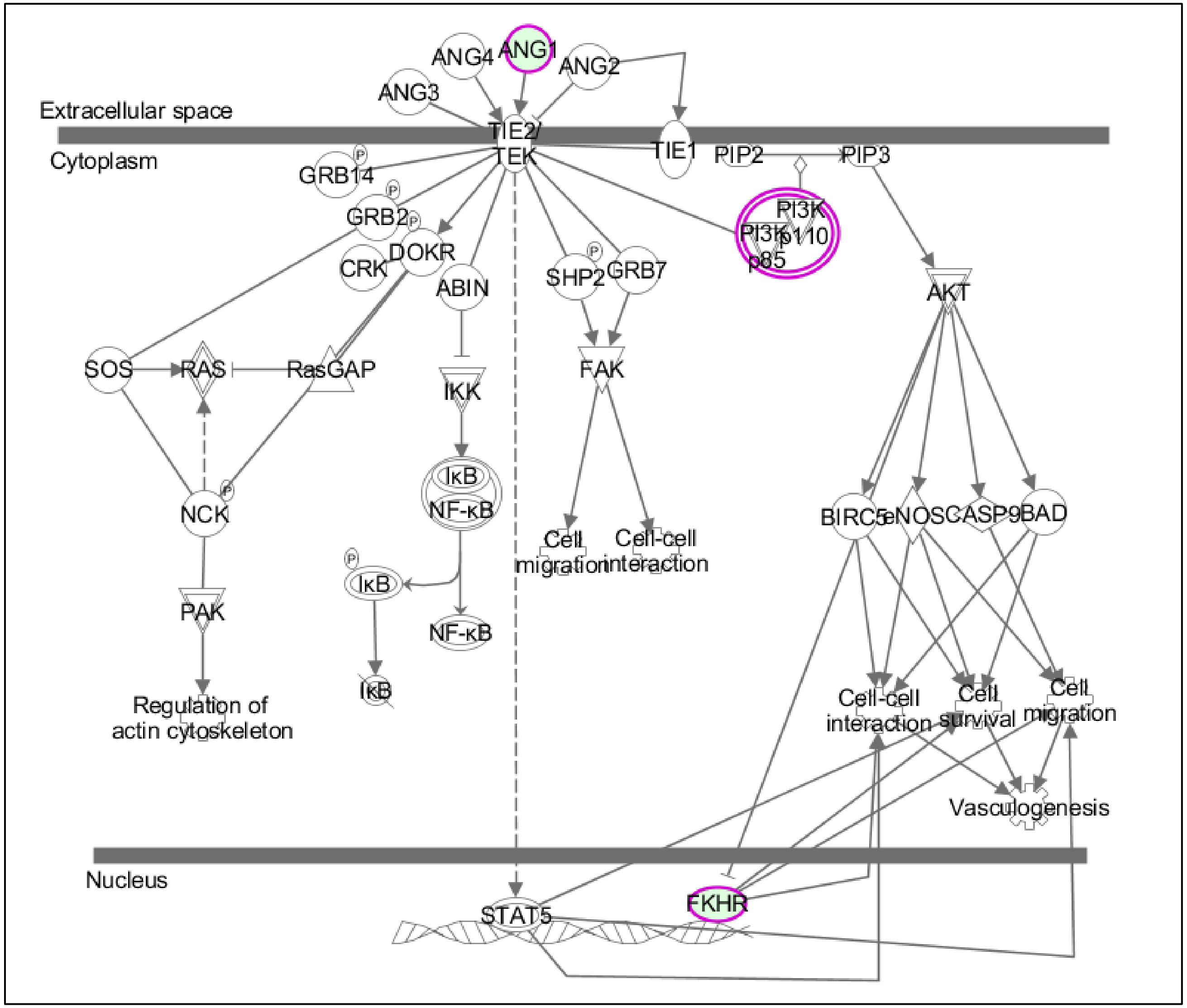

57-Role of Osteoblasts, Osteoclasts and Chondrocytes in Rheumatoid Arthritis

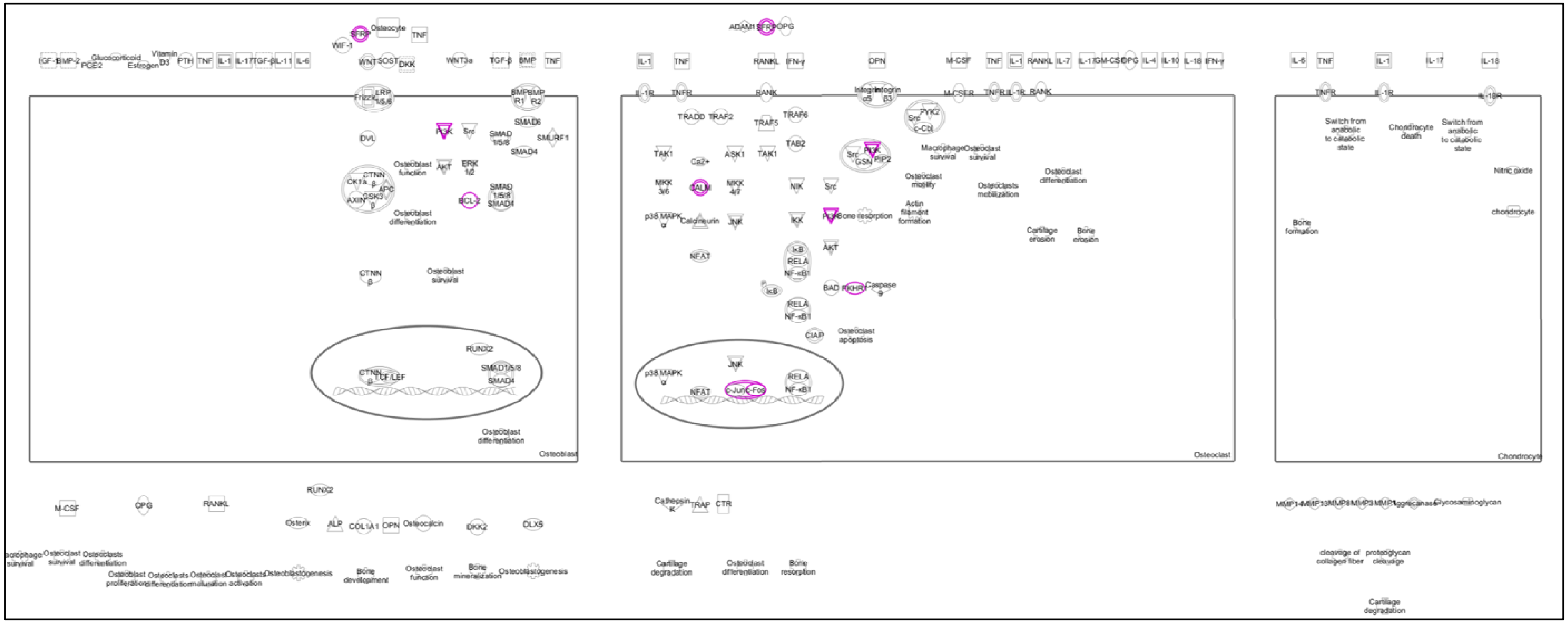

58-Hereditary Breast Cancer Signaling

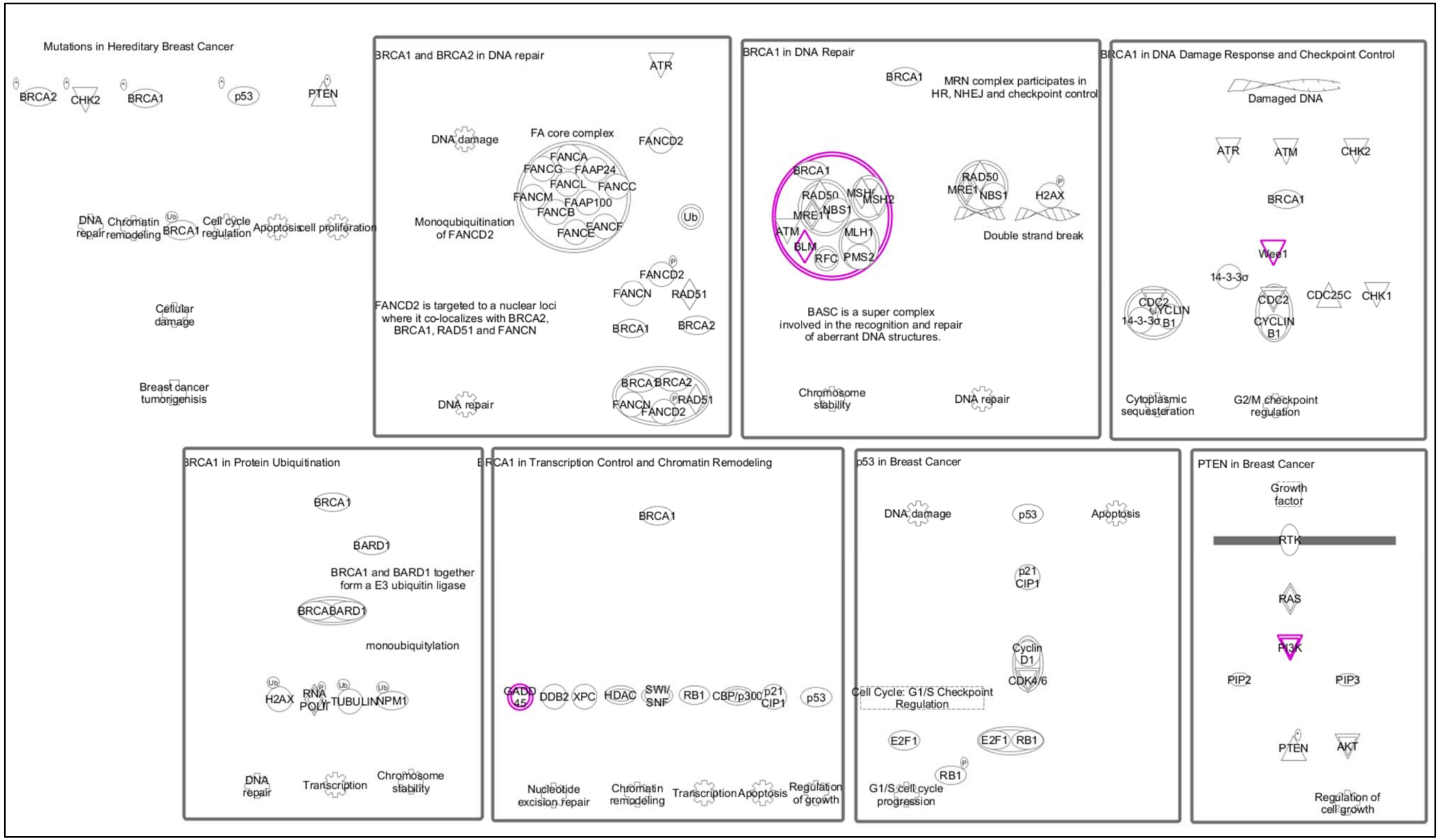

59-Putrescine Biosynthesis III

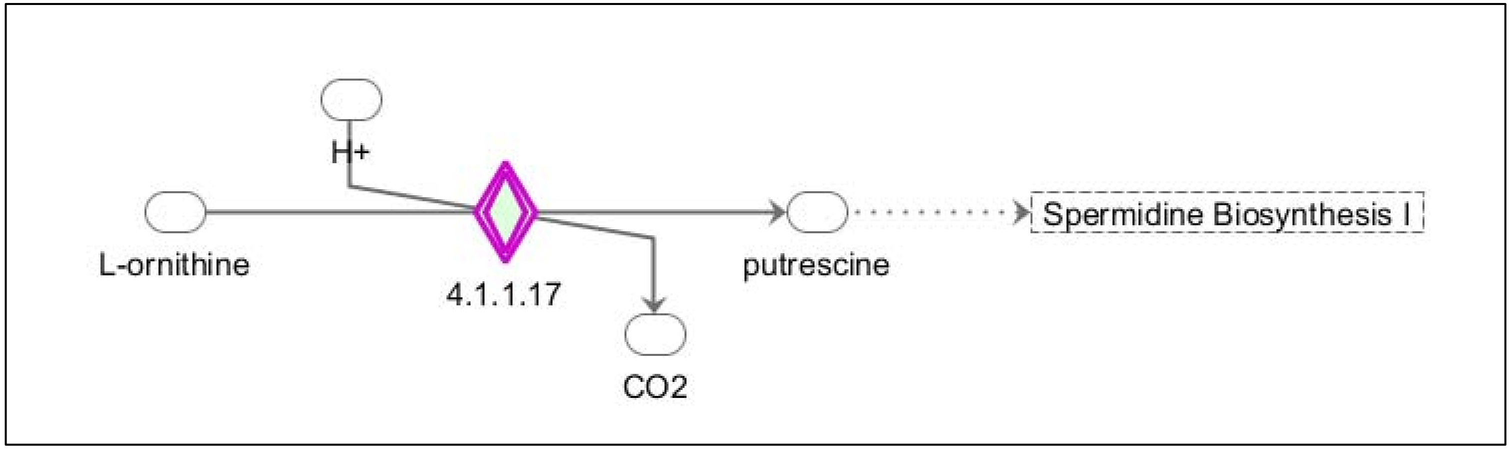

60-Renin-Angiotensin Signaling

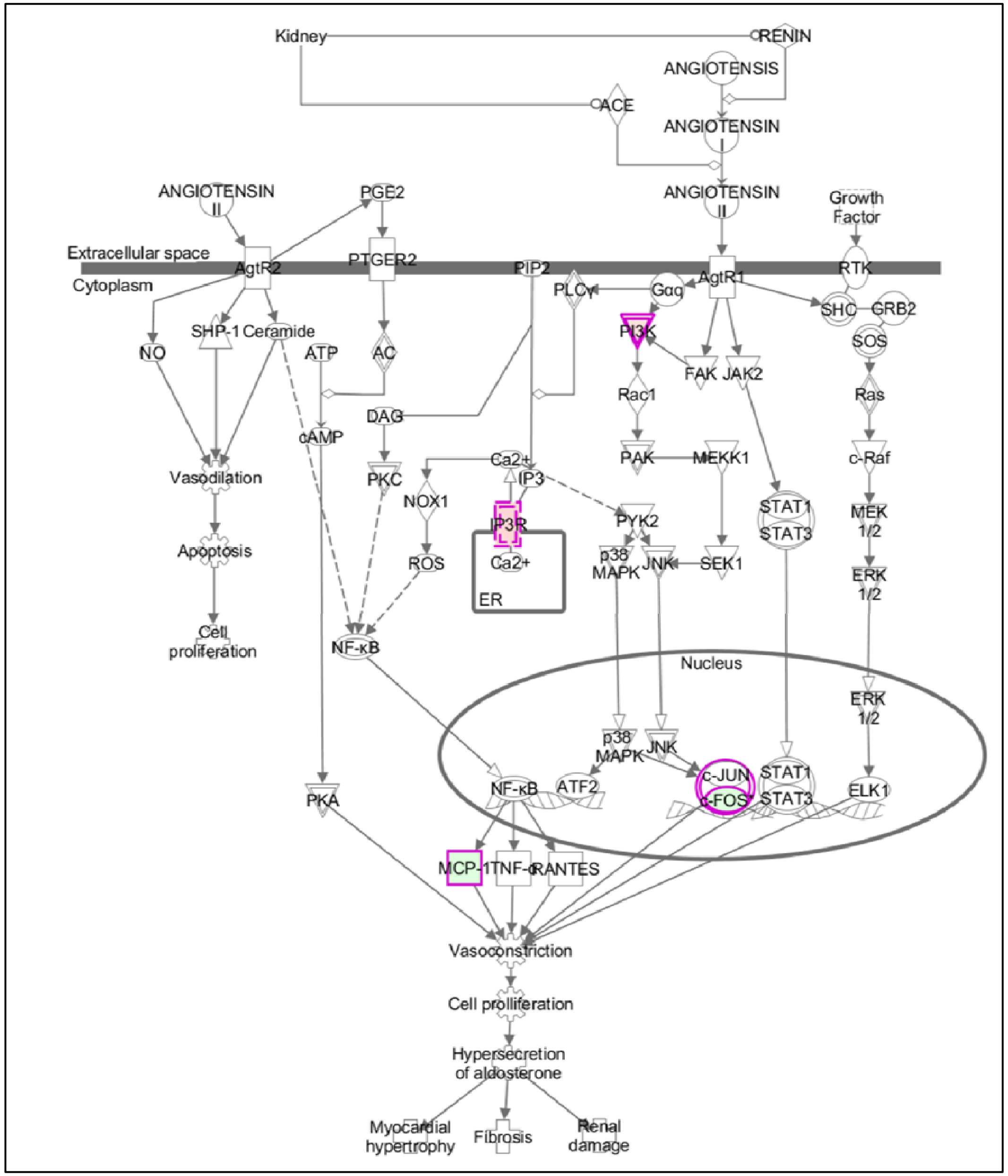

61-IL-3 Signaling

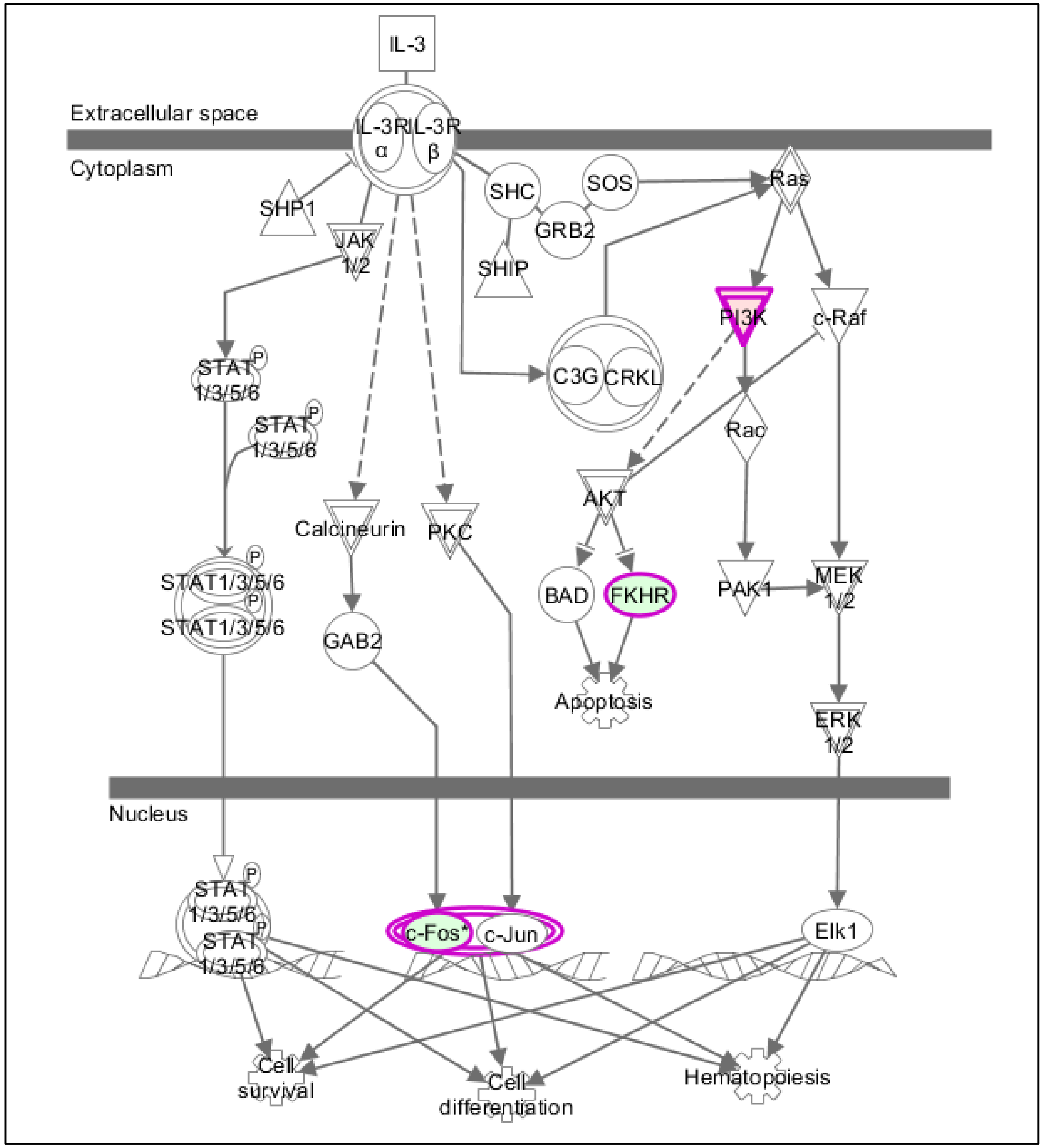

62-GDNF Family Ligand-Receptor Interactions

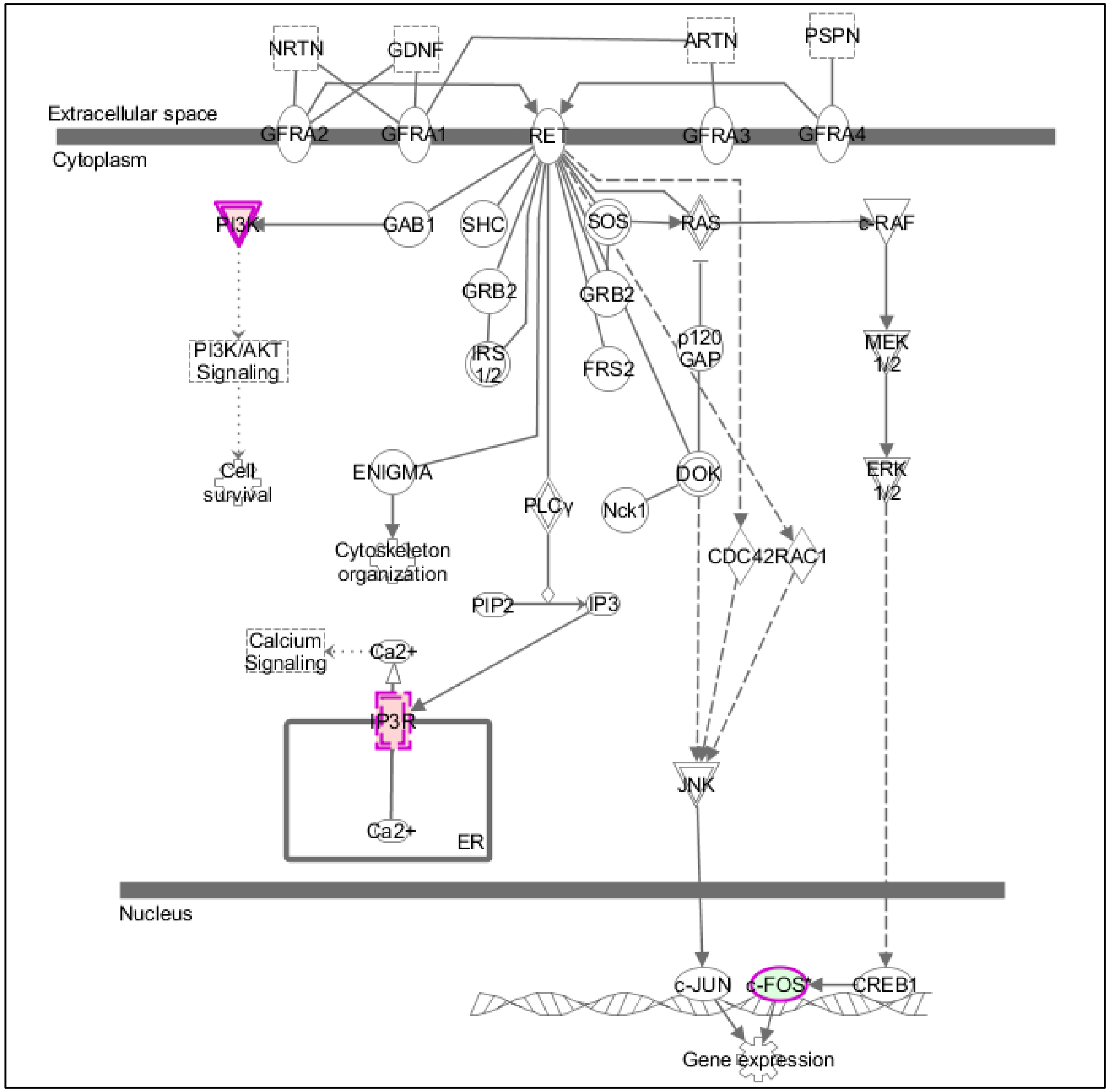

63-PEDF Signaling

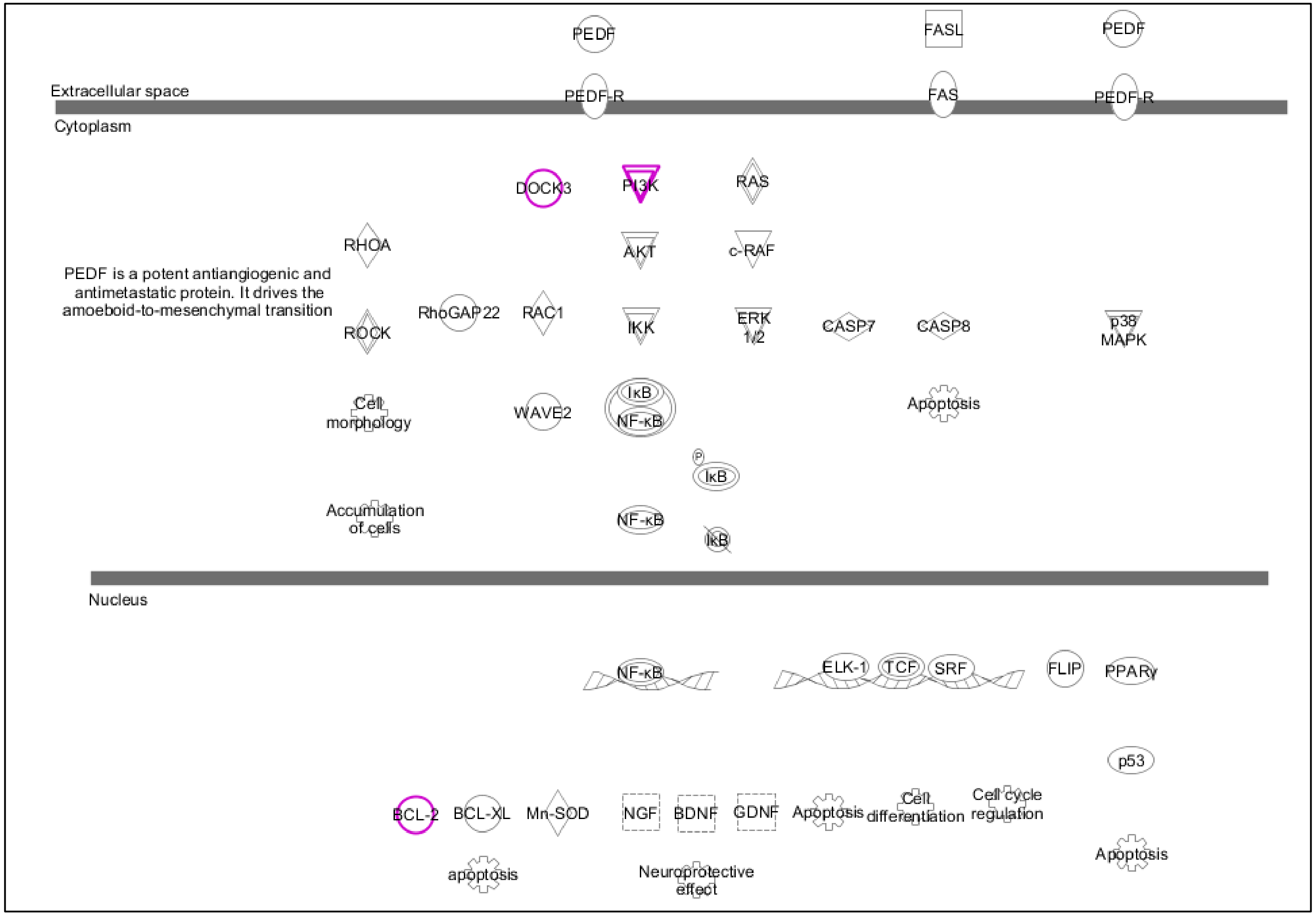

64-PTEN Signaling

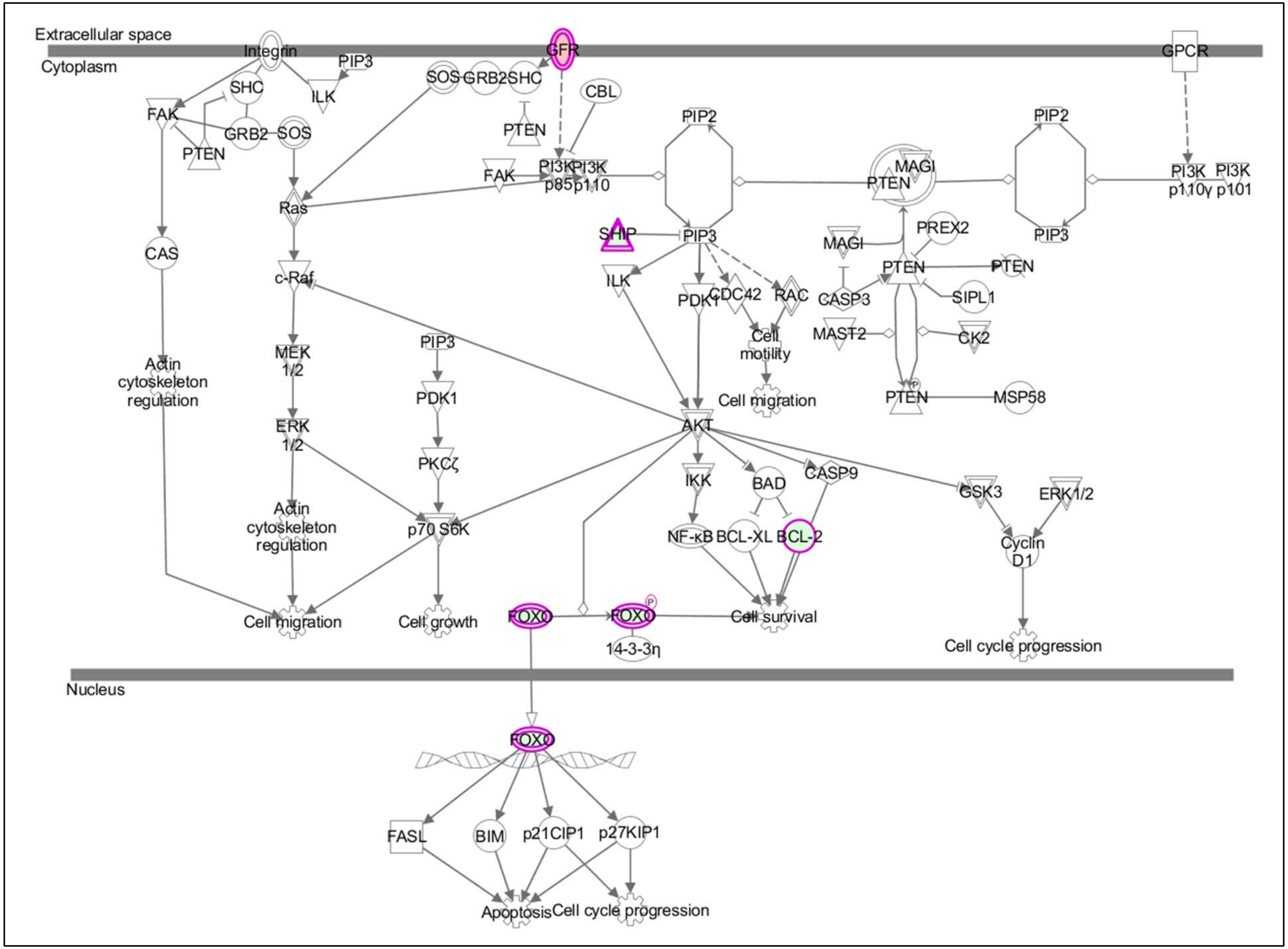

65-Superpathway of D-myo-inositol(1,4,5)-trisphosphate Metabolism

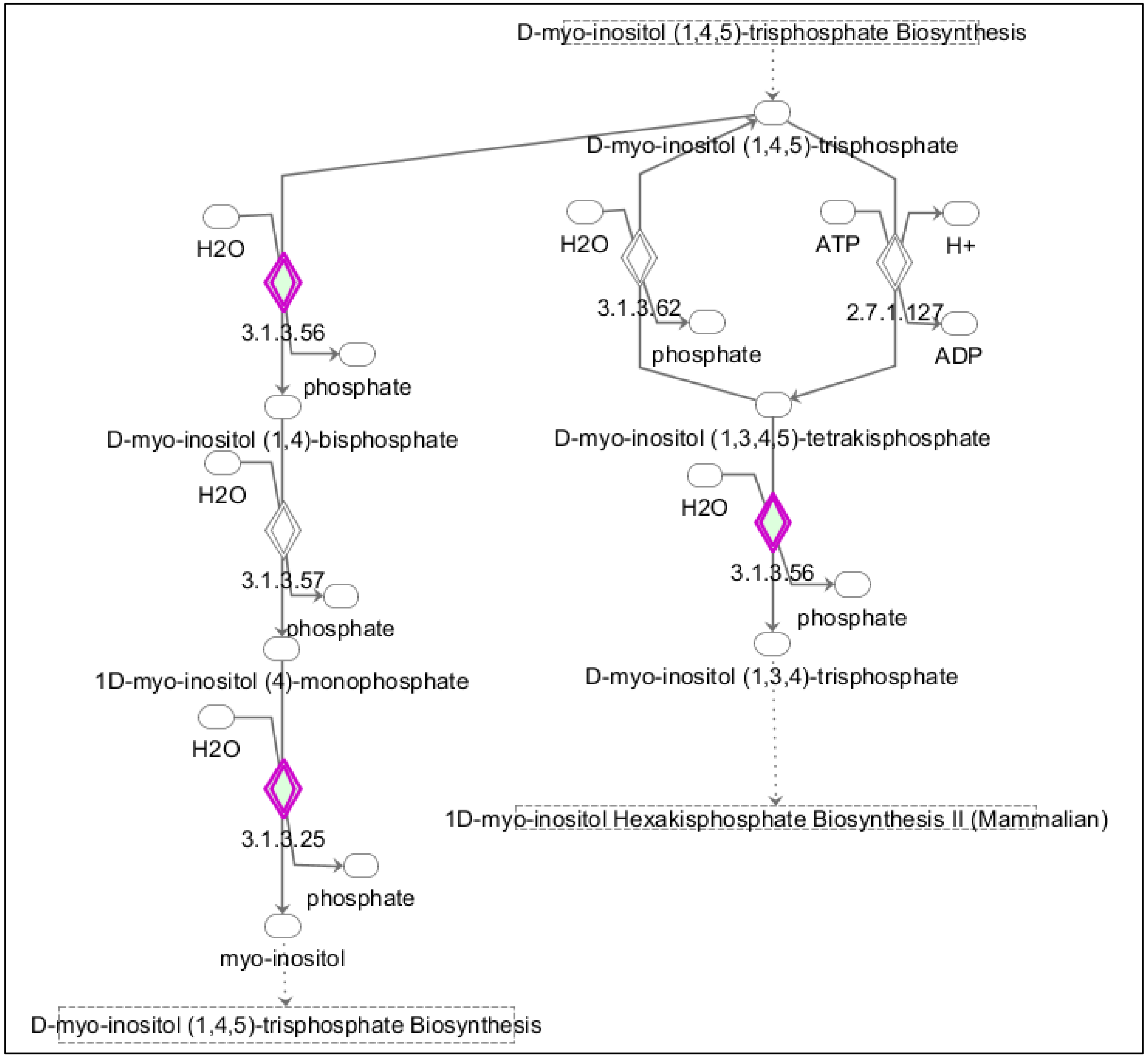

66-Phenylalanine Degradation Ⅳ (Mammalian, via Side Chain)

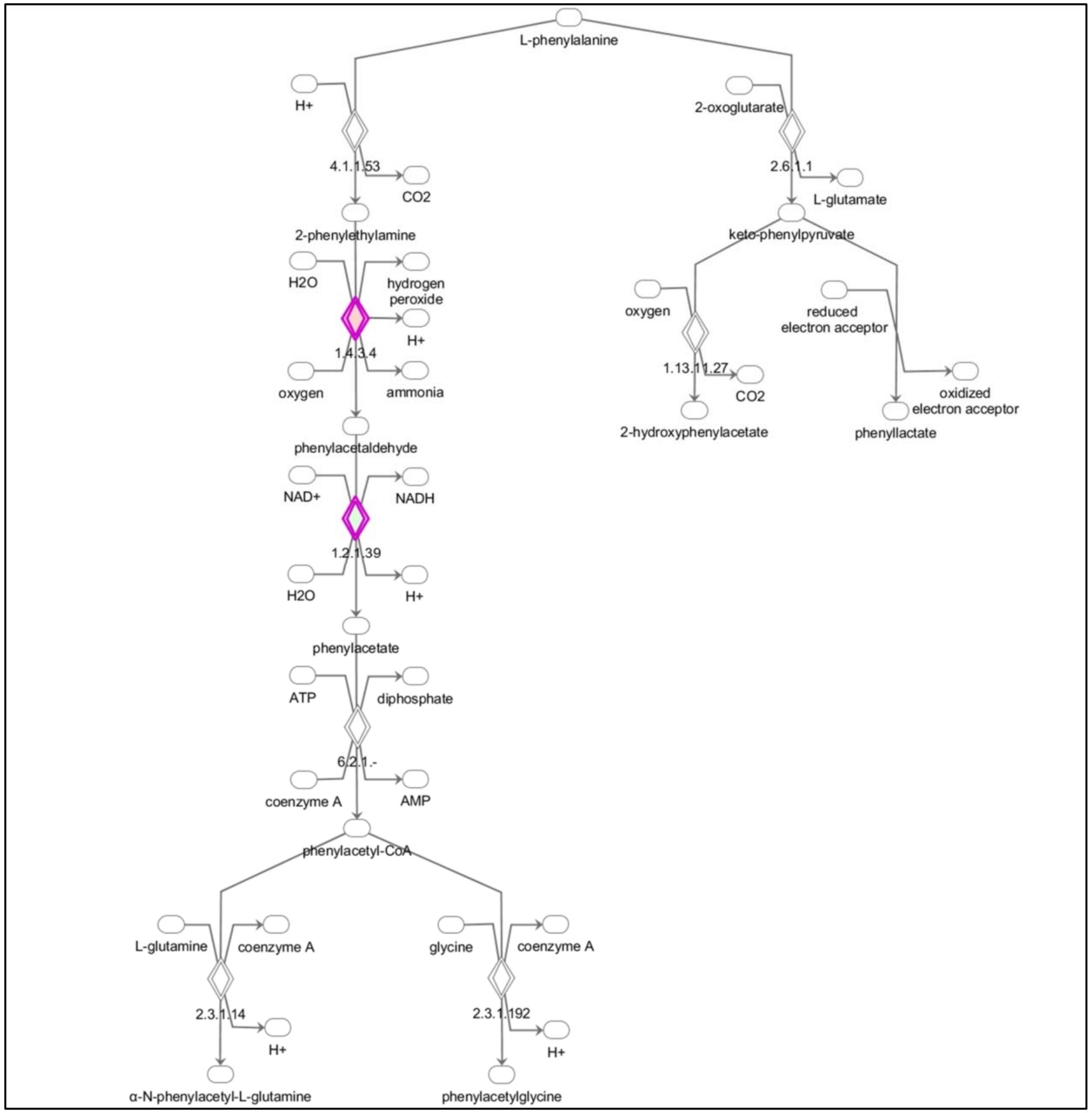

67-Ephrin Receptor Signaling

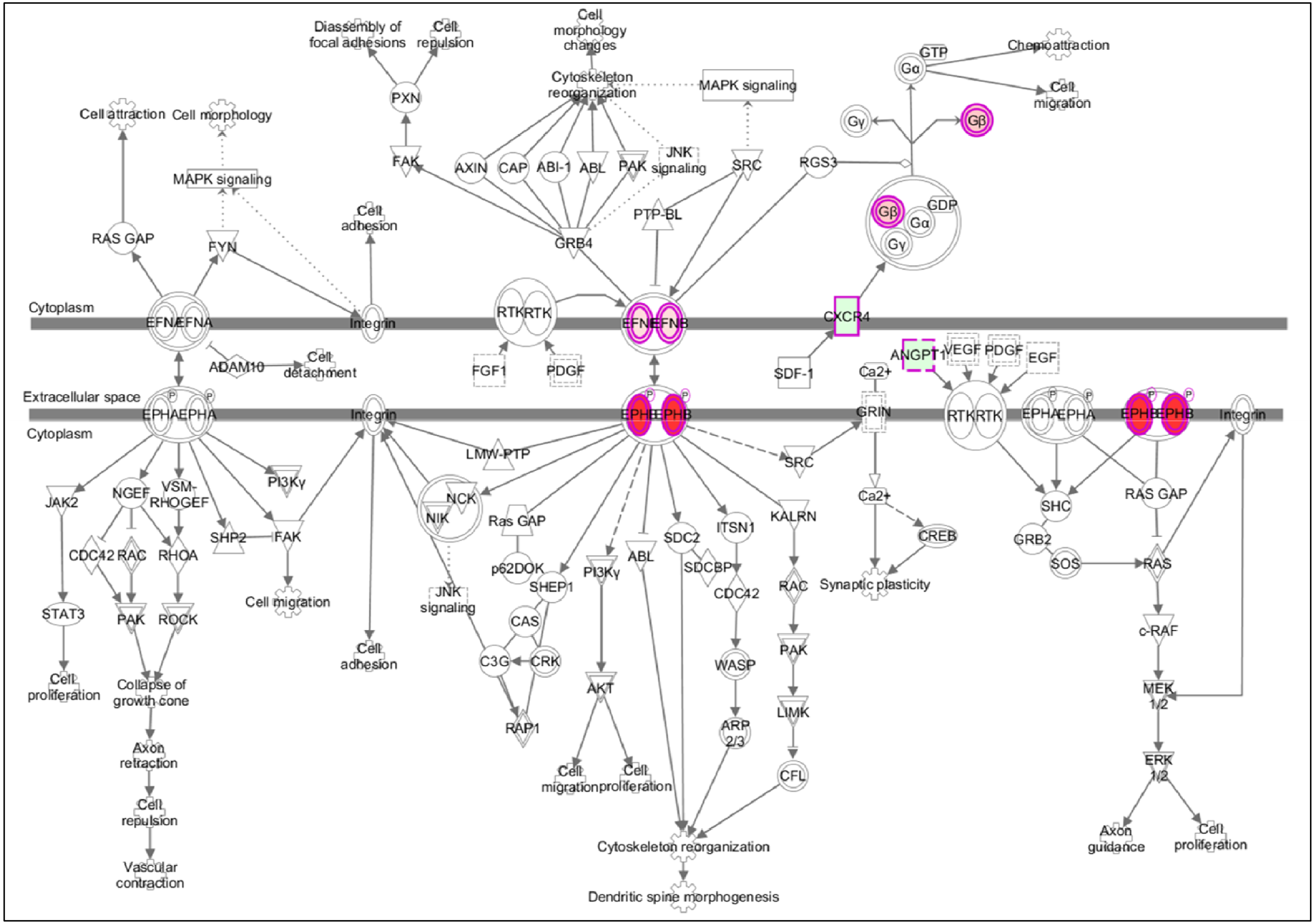

68-CCRS Signaling in Macrophages

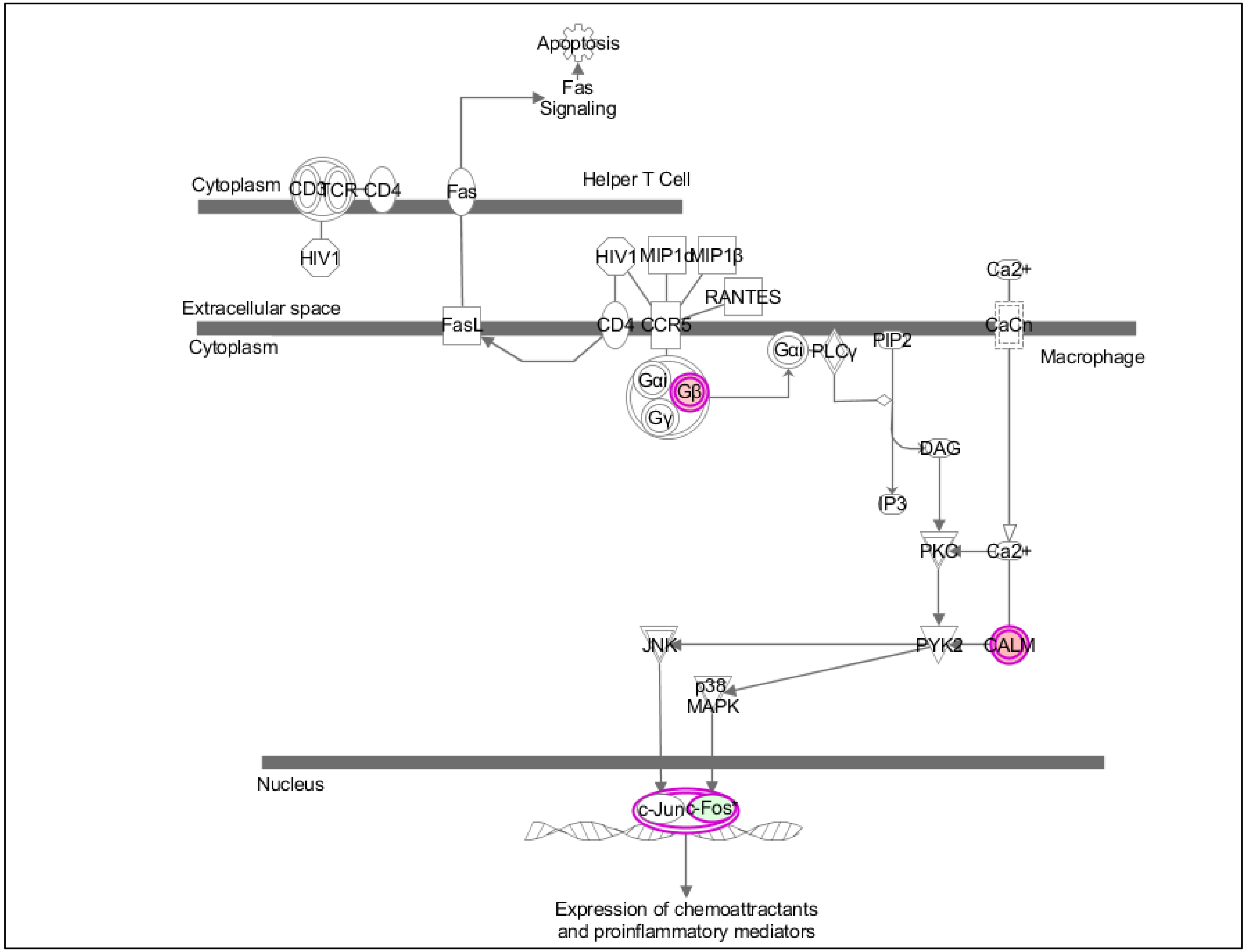

Supplement: Supplementary file 1 [file Presentation_1.zip › Supplemental materials 1.3.pdf]
