# Supplementary material for: Multiomics-Based Signaling Pathway Network Alterations in Human Non-functional Pituitary Adenomas
Source: Front Endocrinol (Lausanne). 2019 Dec 17;10:835. doi: 10.3389/fendo.2019.00835 (PMC6928143; doi:10.3389/fendo.2019.00835)

Supplemental materials 2.3
Statistically significant canonical pathways derived from differentially expressed proteins between NFPAs and controls for IPA analysis (Dataset 2)

1. Aryl hydrocarbon Receptor Signaling
2. Mitochondrial Dysfunction
3. Oxidative Phosphorylation
4. Role of JAK2 in Hormone-like Cytokine Signaling
5. Aldosterone Signaling in Epithelial Cell
6. NRF2-mediated Oxidative Stress Response
7. PPARα-RXRα Activation
8. ERK-MAPK Signaling
9. Growth Hormone Signaling
10. Prolactin Signaling
11. TR-RXR Activation
12. Protein Ubiquitination Pathay
13. Methylglyoxal Degradation Ⅰ
14. IGF-1 Signaling
15. Role of Tissue Factor in Cancer
16. Hematopoiesis from Multipotent Stem Cells
17. 14-3-3-mediated Signaling
18. Acyl-CoA Hydrolysis
19. PI3K/AKT Sigaling
20. Serotonin and melatonin Biosynthess
21. Extrinsic Prothrombin Activation Pathway
22. Endoplasmic Reticulum Stress Pathway
23. Tec Kinase Signaling
24. Methylglyoxal Degradation Ⅲ
25. Glutathione Redox Reacions Ⅰ

Dataset 2-Cannonical Pathway Chart

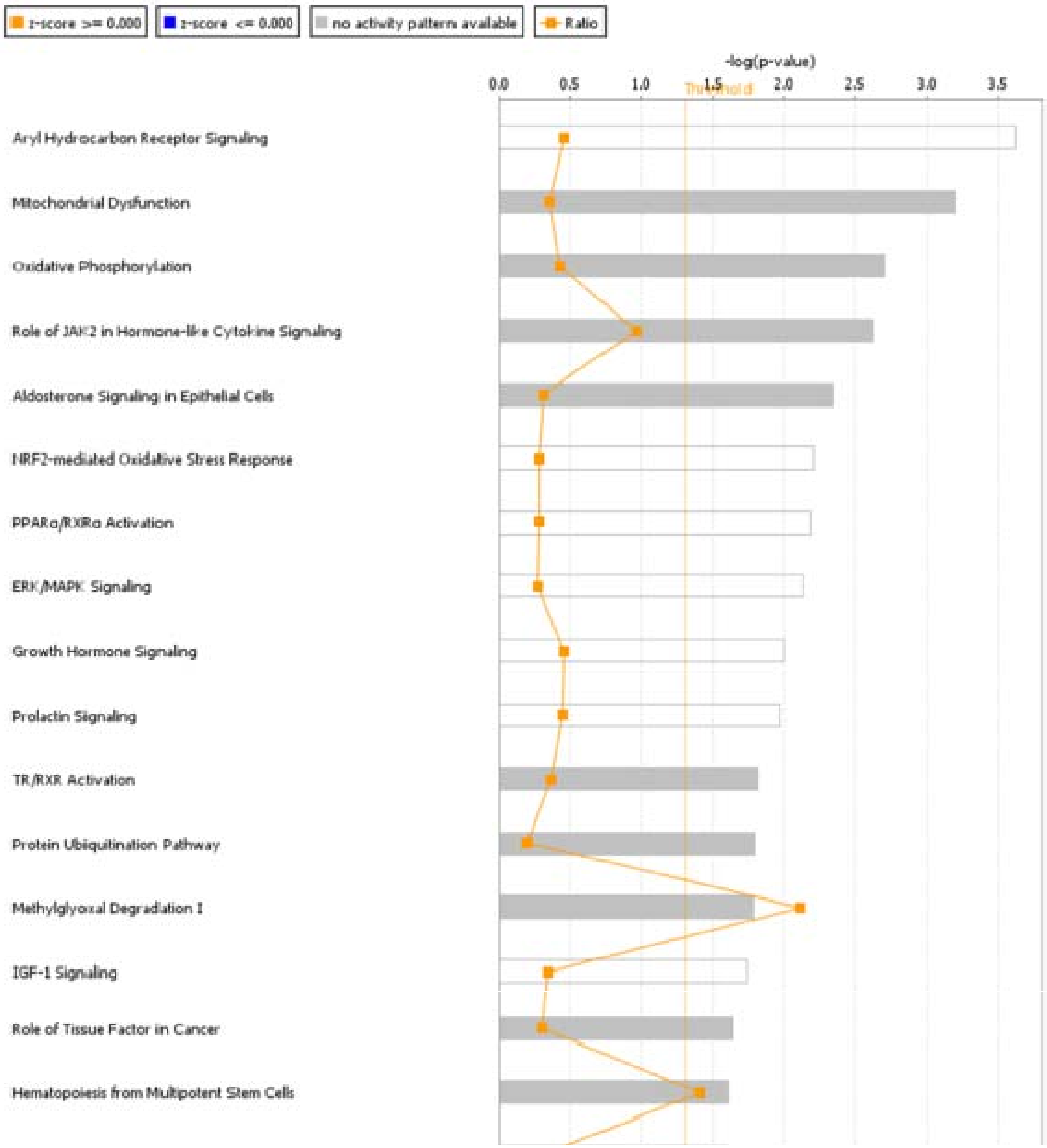

1-Aryhydrocarbon Receptor Signaling

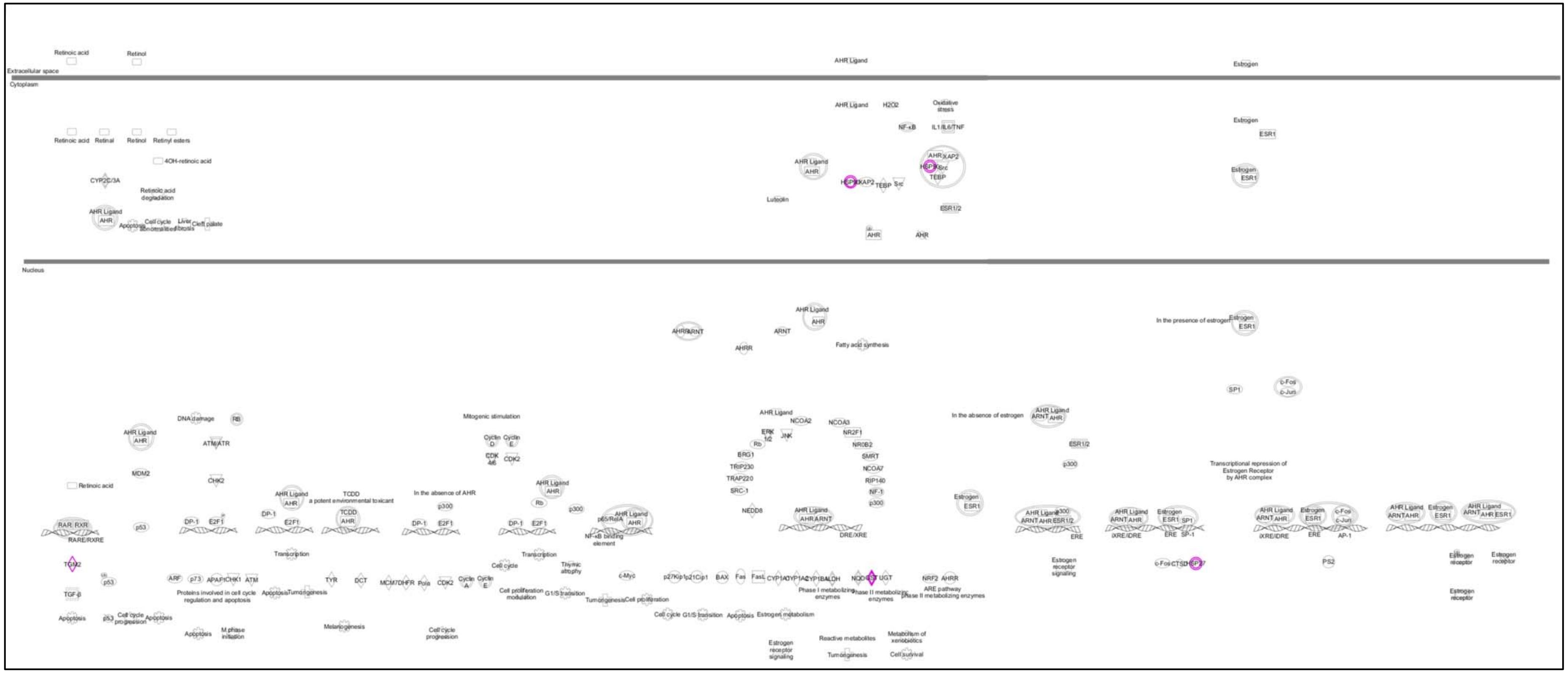

2-Mitochondrial Dysfunction

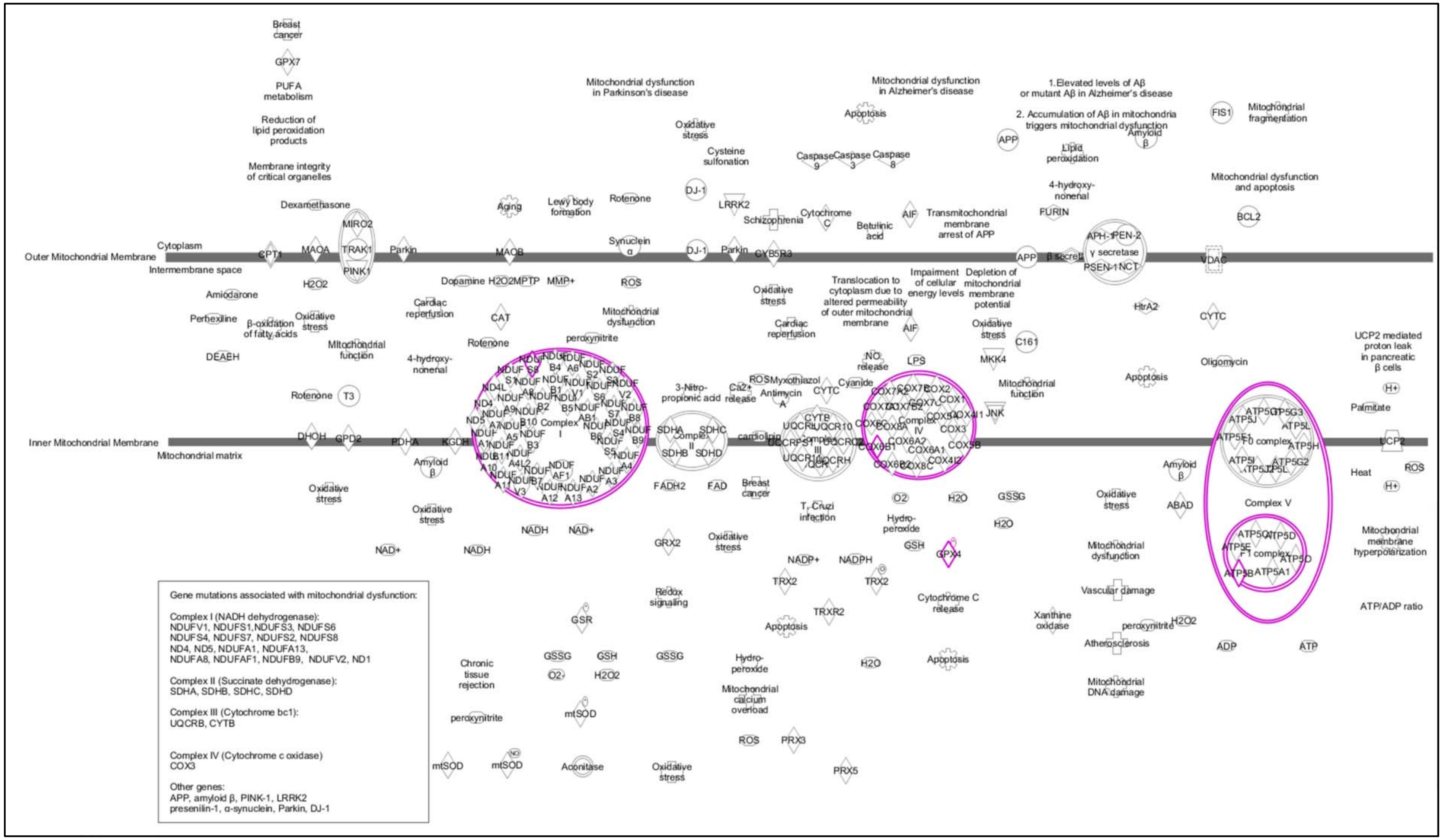

3-Oxidative Phosphorylation

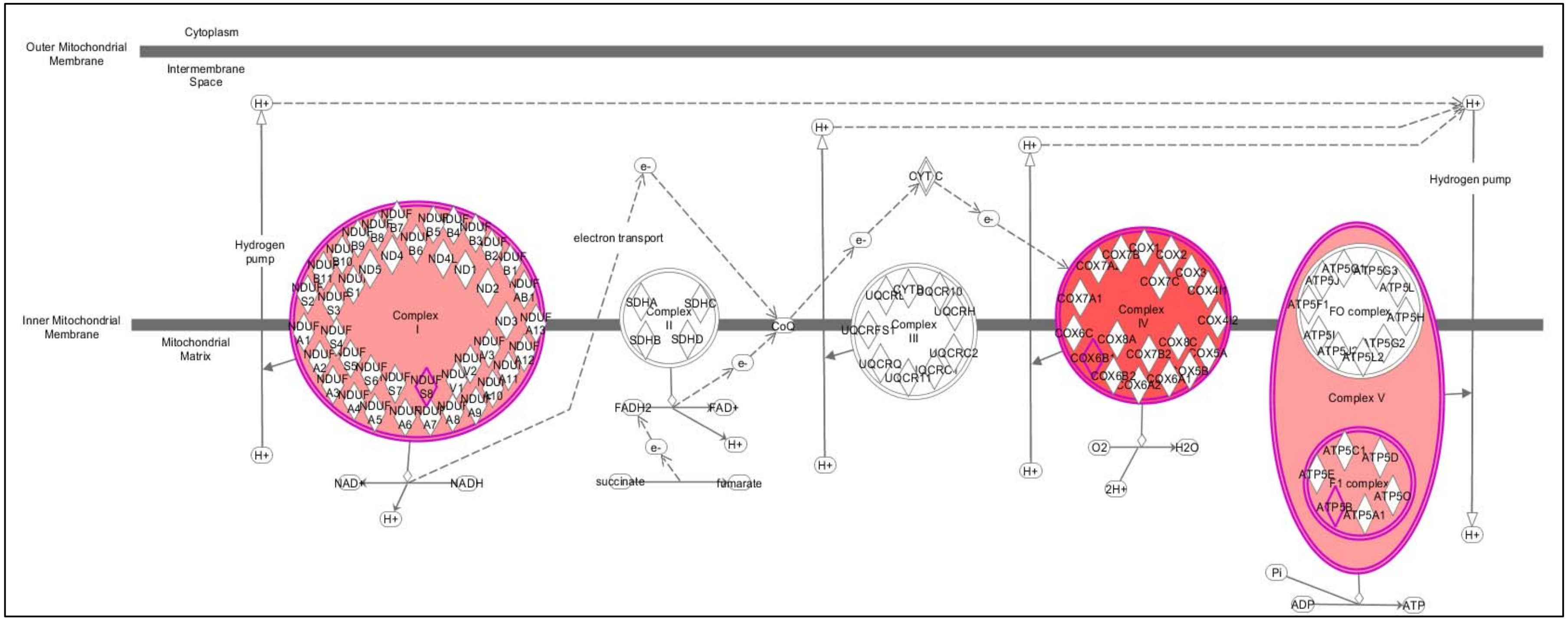

4-Role of JAK2 in Hormone-like Cytokine Signaling

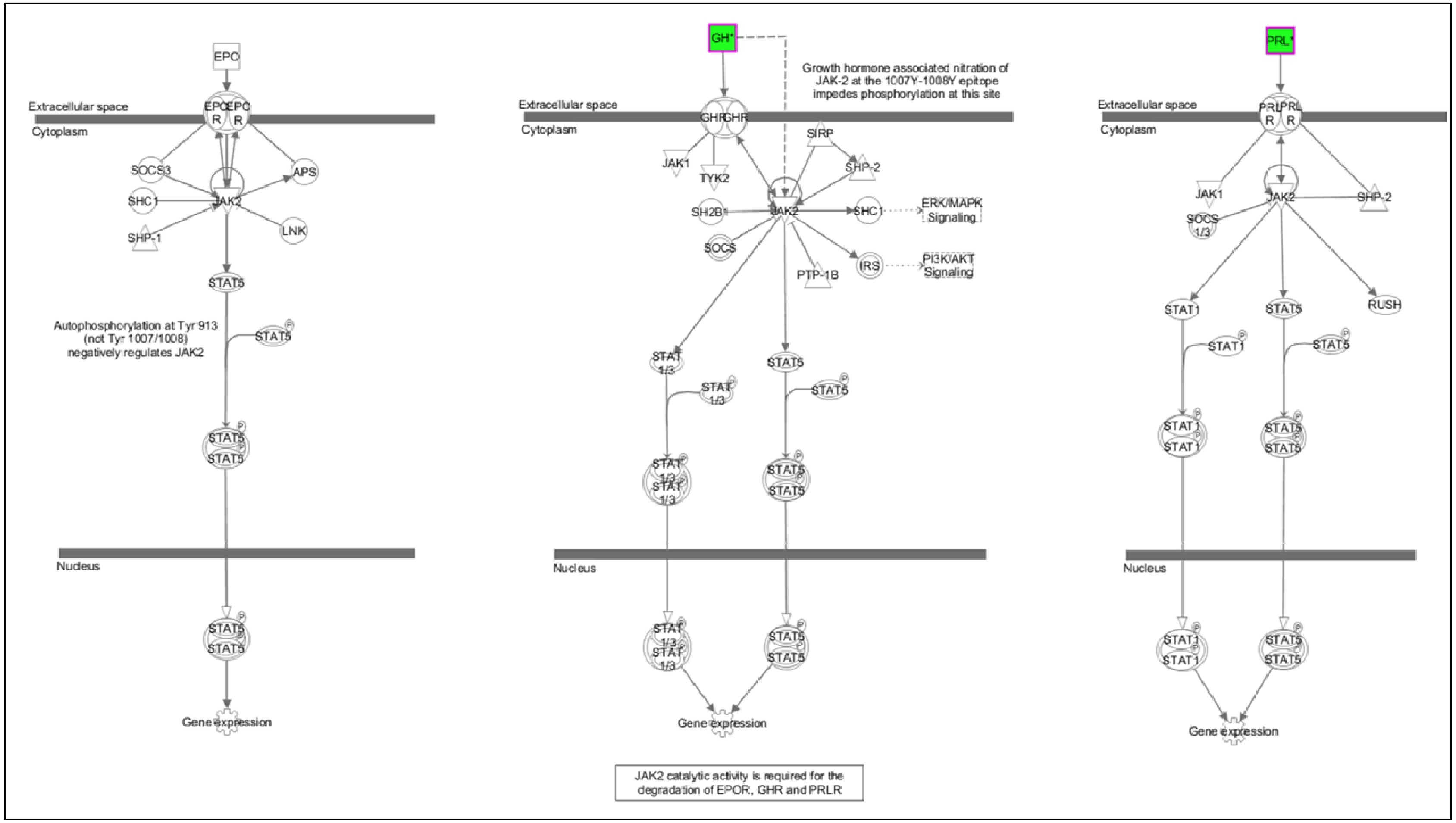

5-Aldosterone Signaling in Epithelial Cell

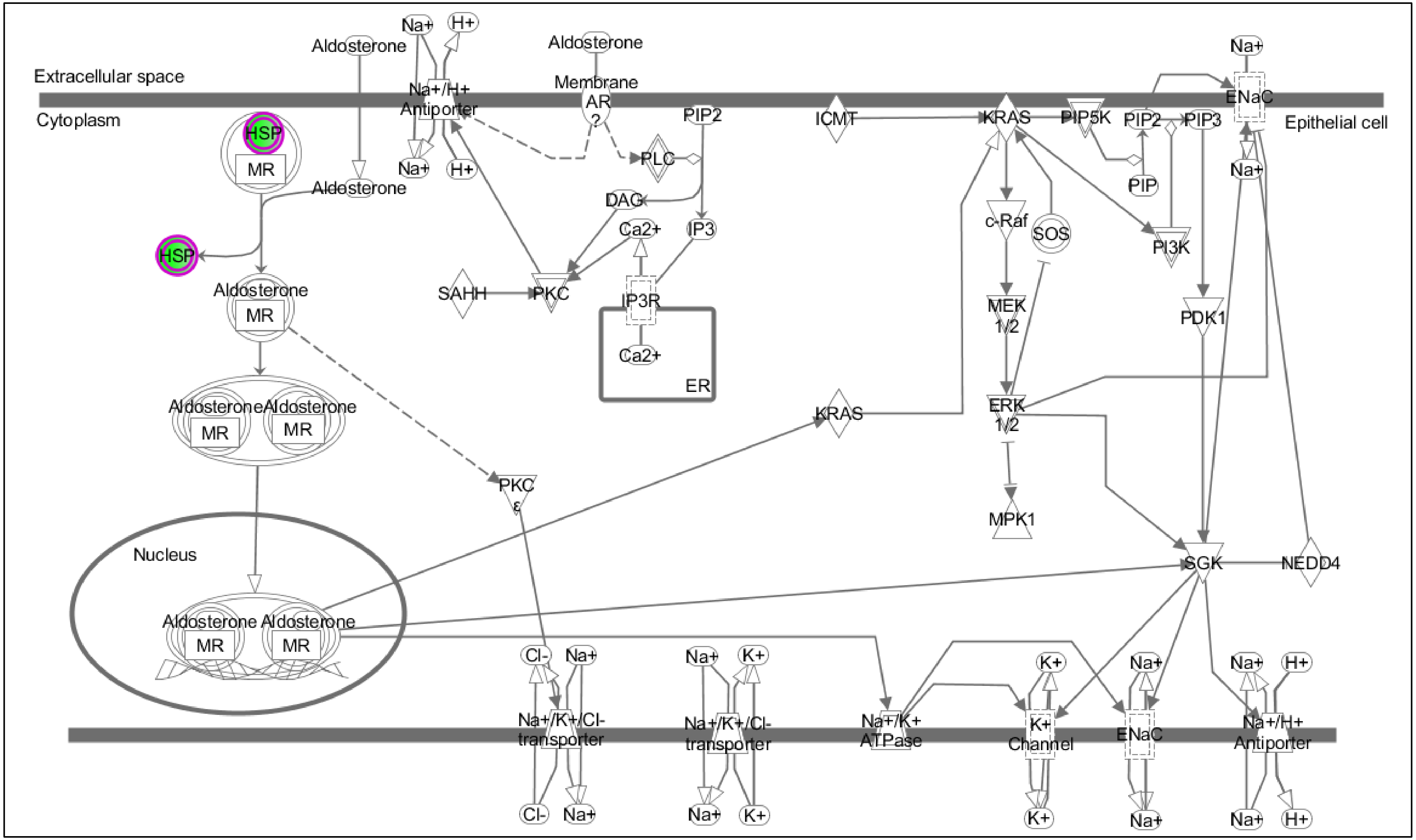

6-NRF2-mediated Oxidative Stress Response

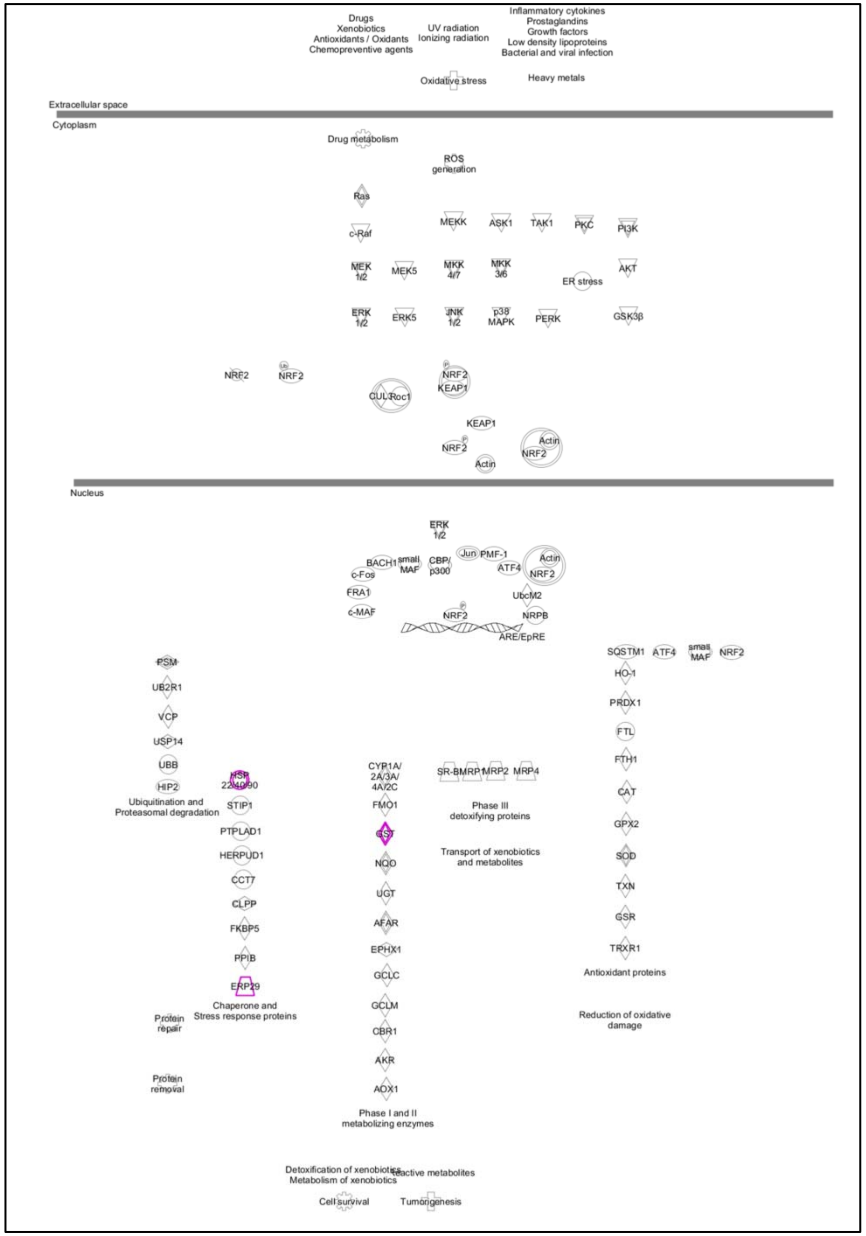

7-PPARα-RXRα Activation

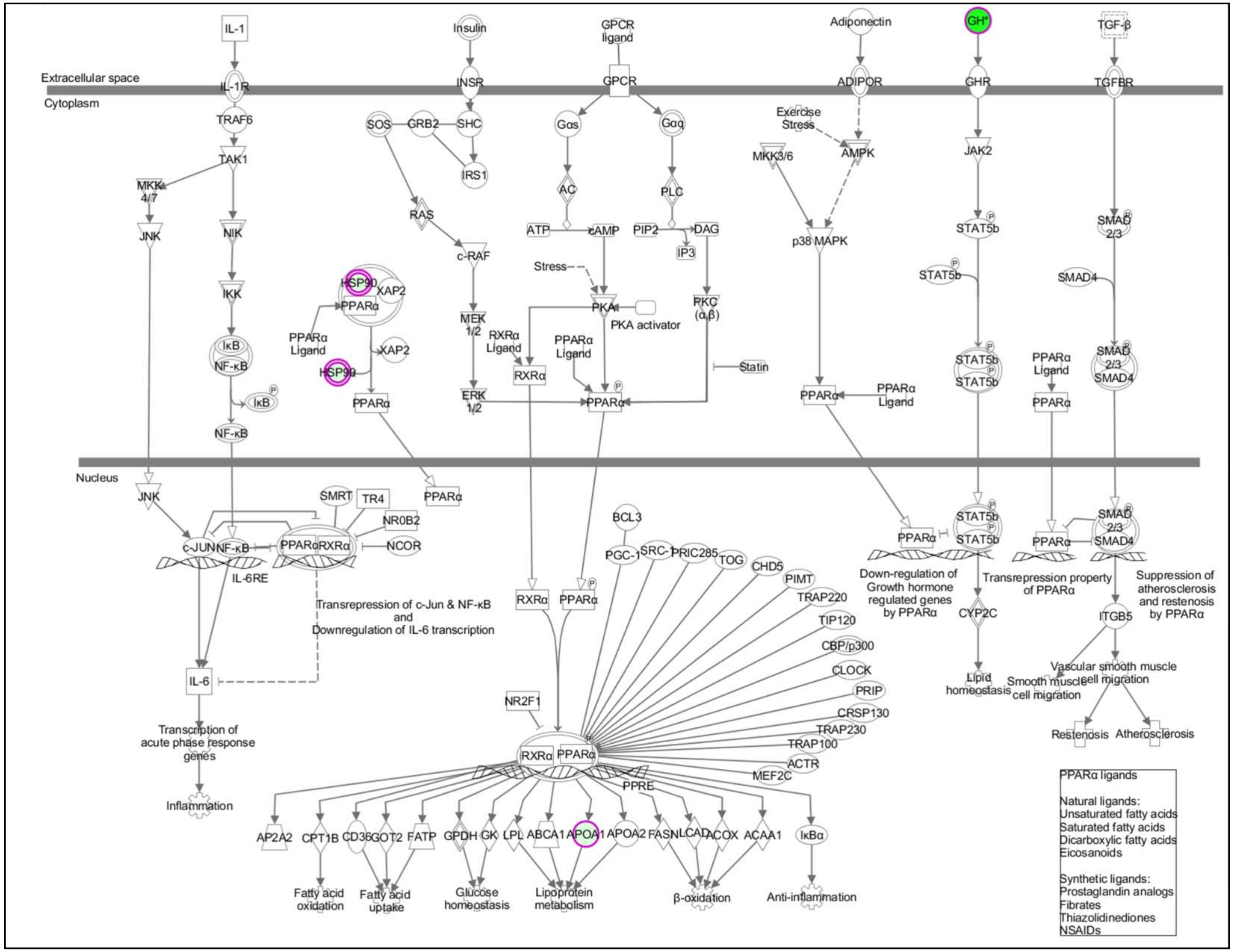

8-ERK-MAPK Signaling

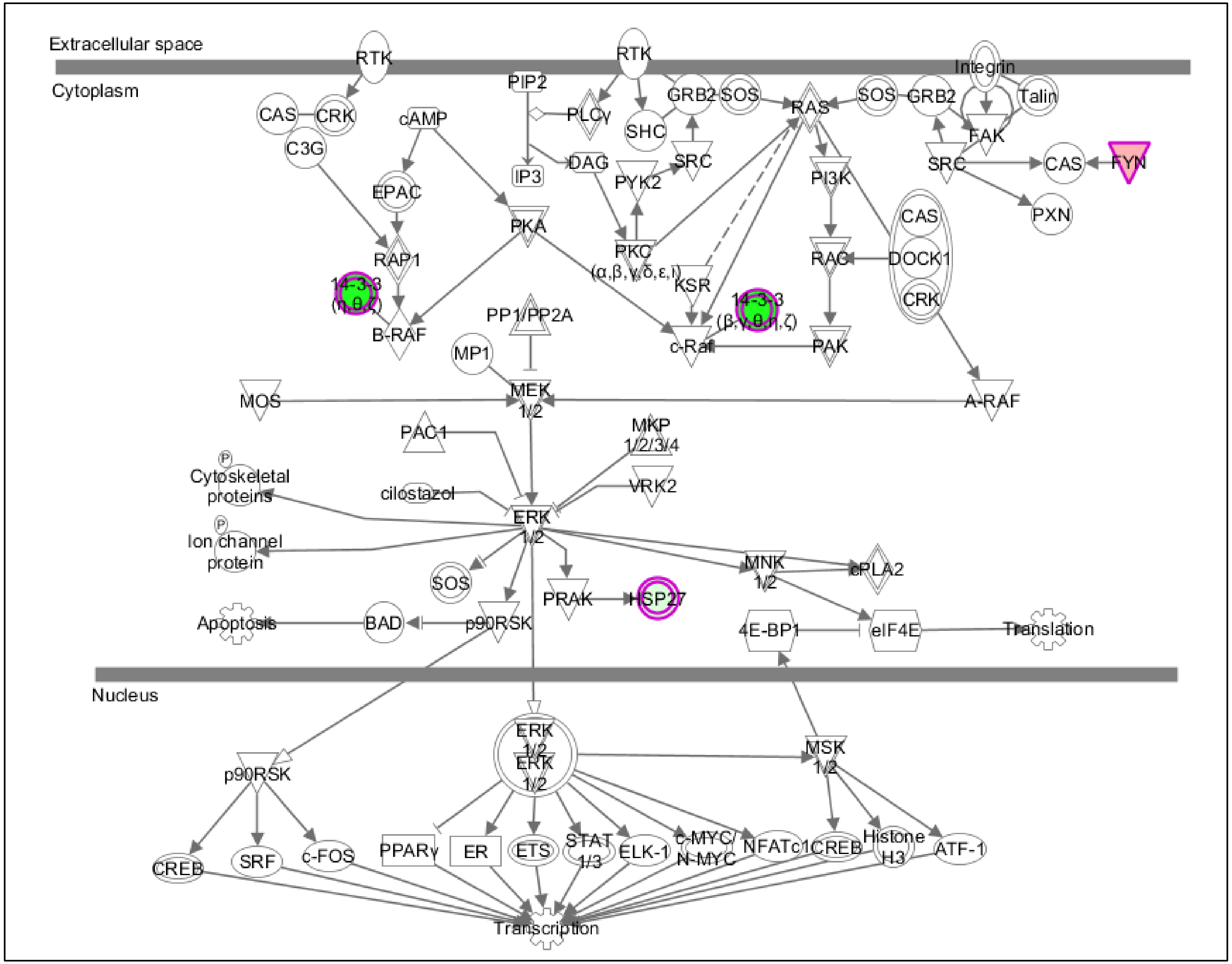

9-Growth Hormone Signaling

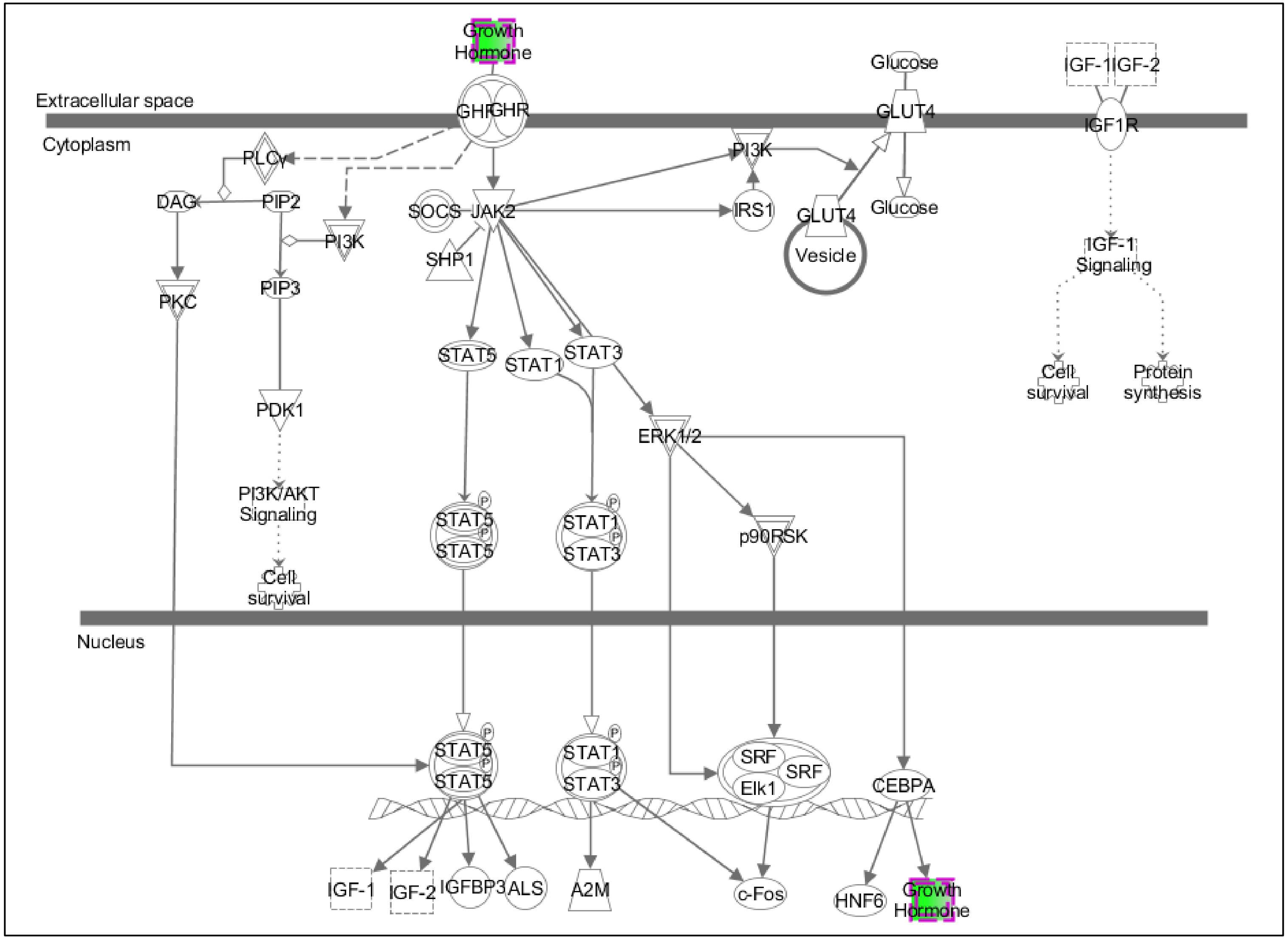

10-Prolactin Signaling

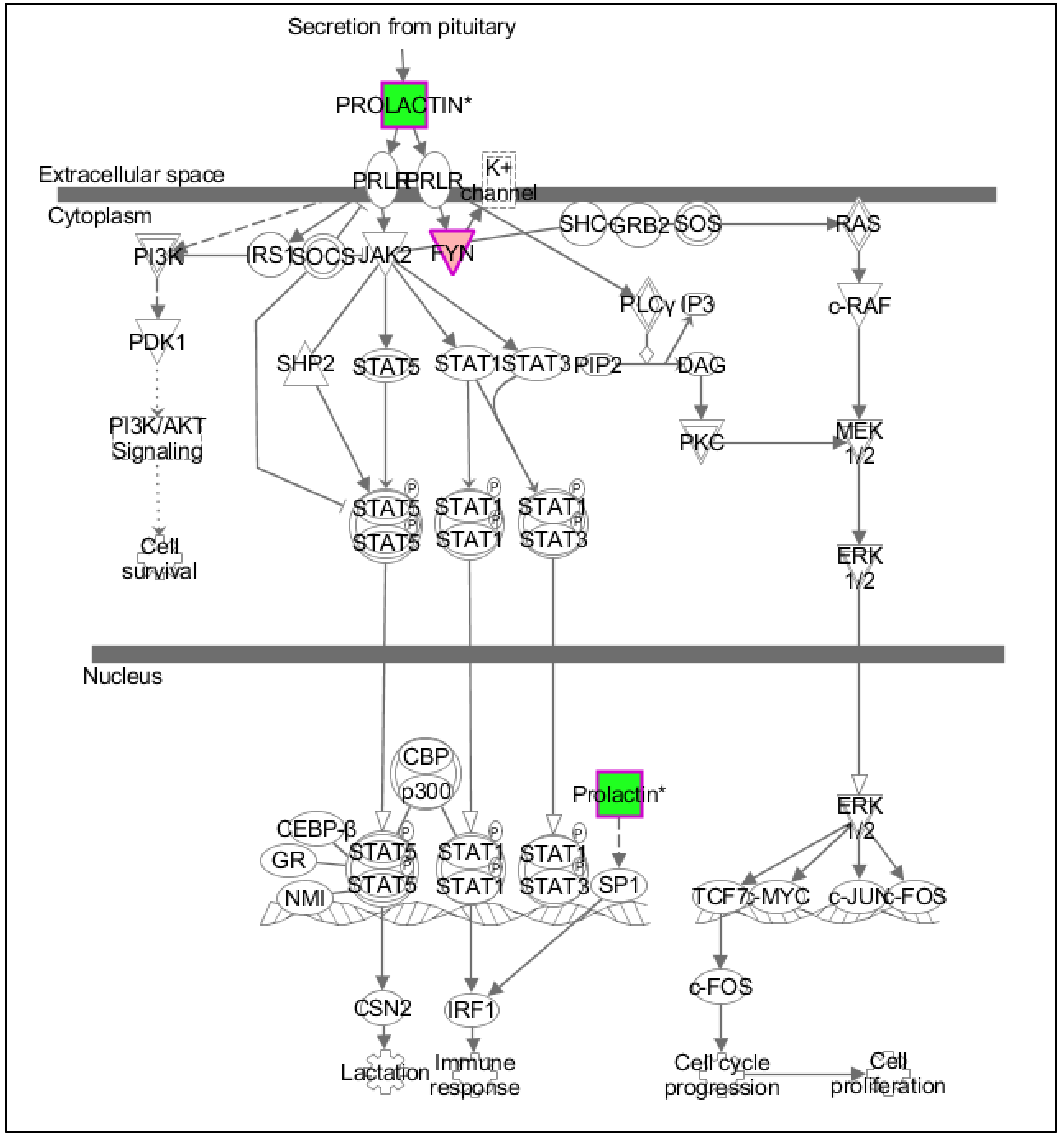

11-TR-RXR Activation

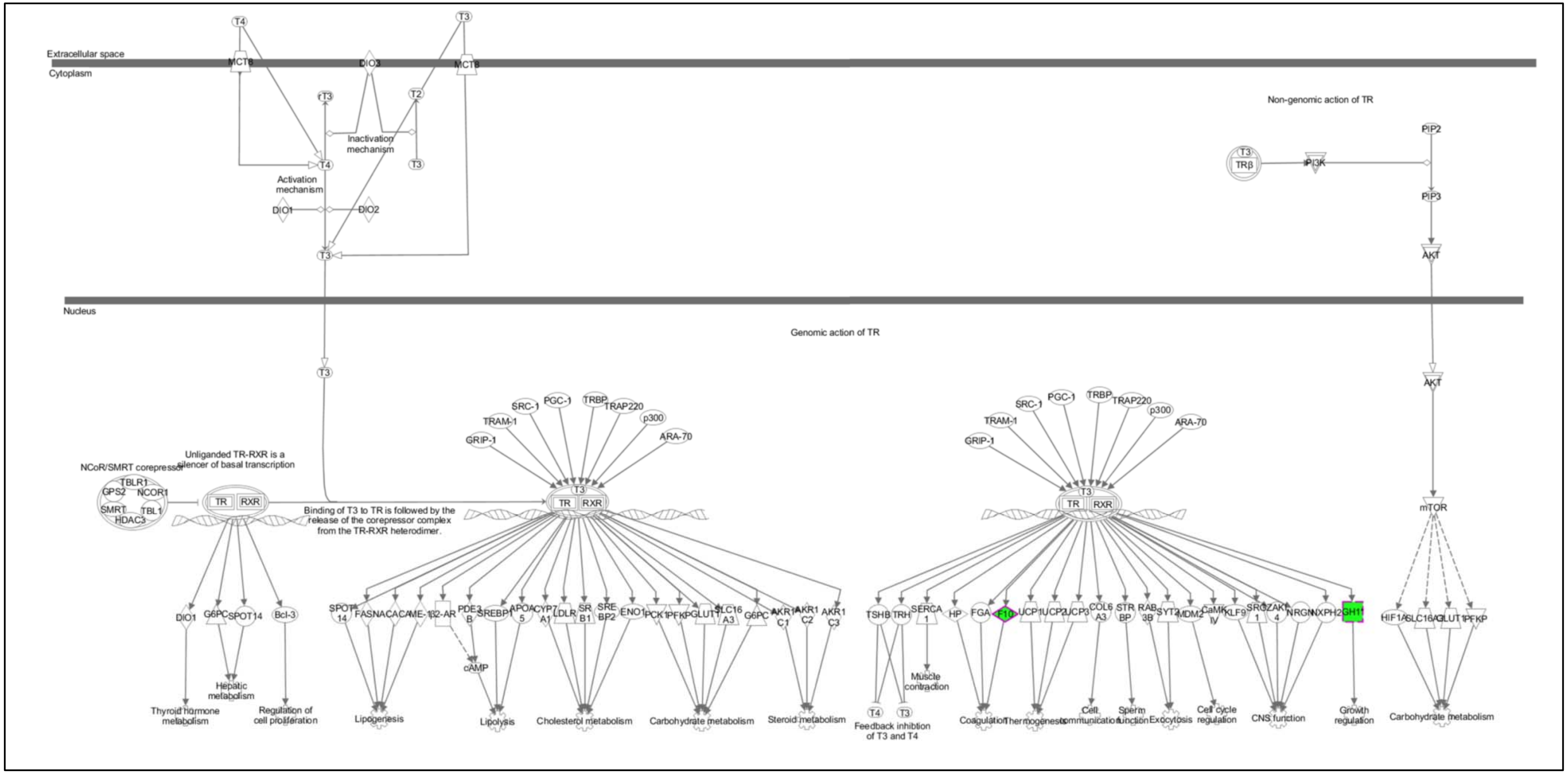

12-Protein Ubiquitination Pathay

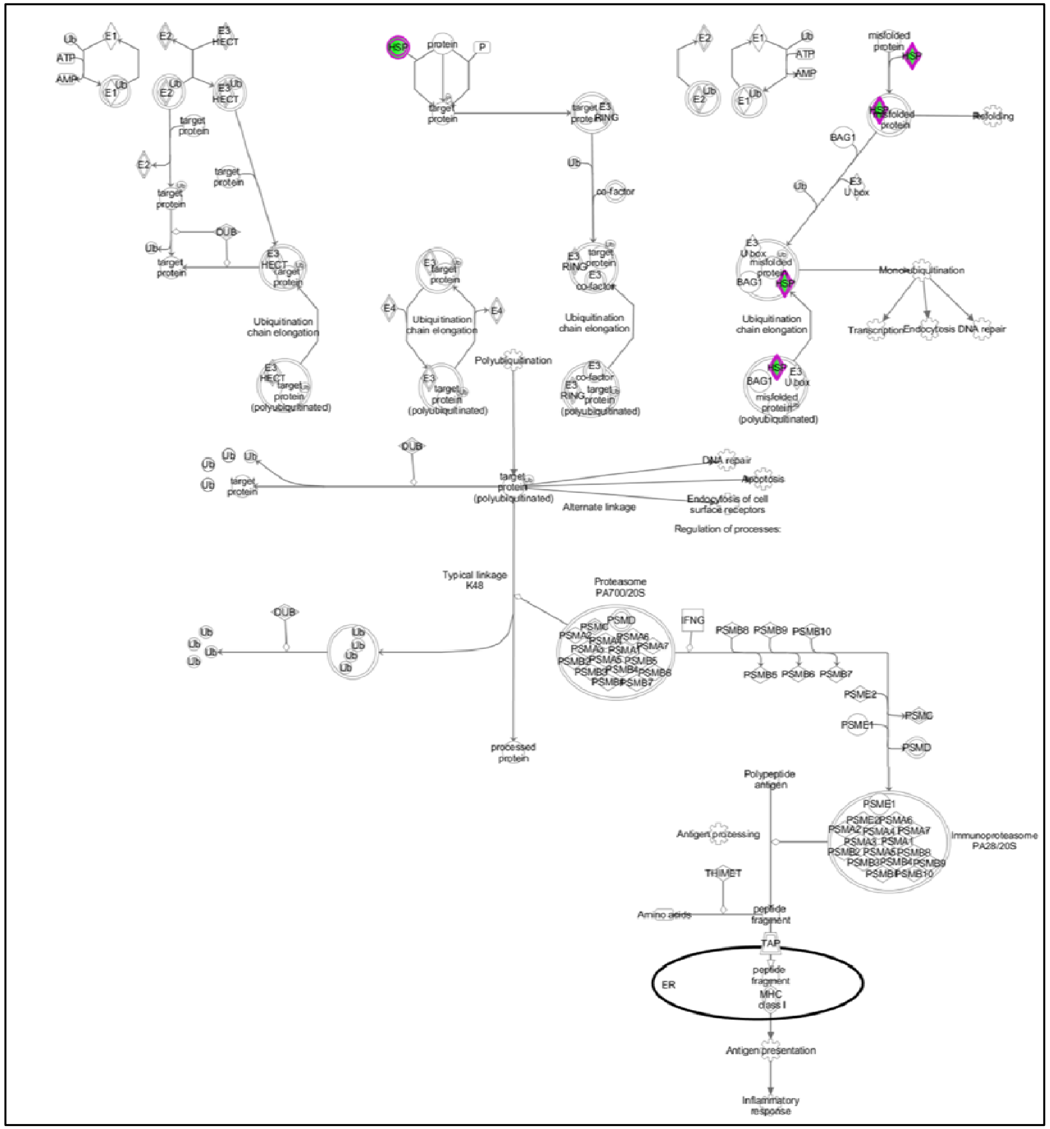

13-Methylglyoxal Degradation Ⅰ

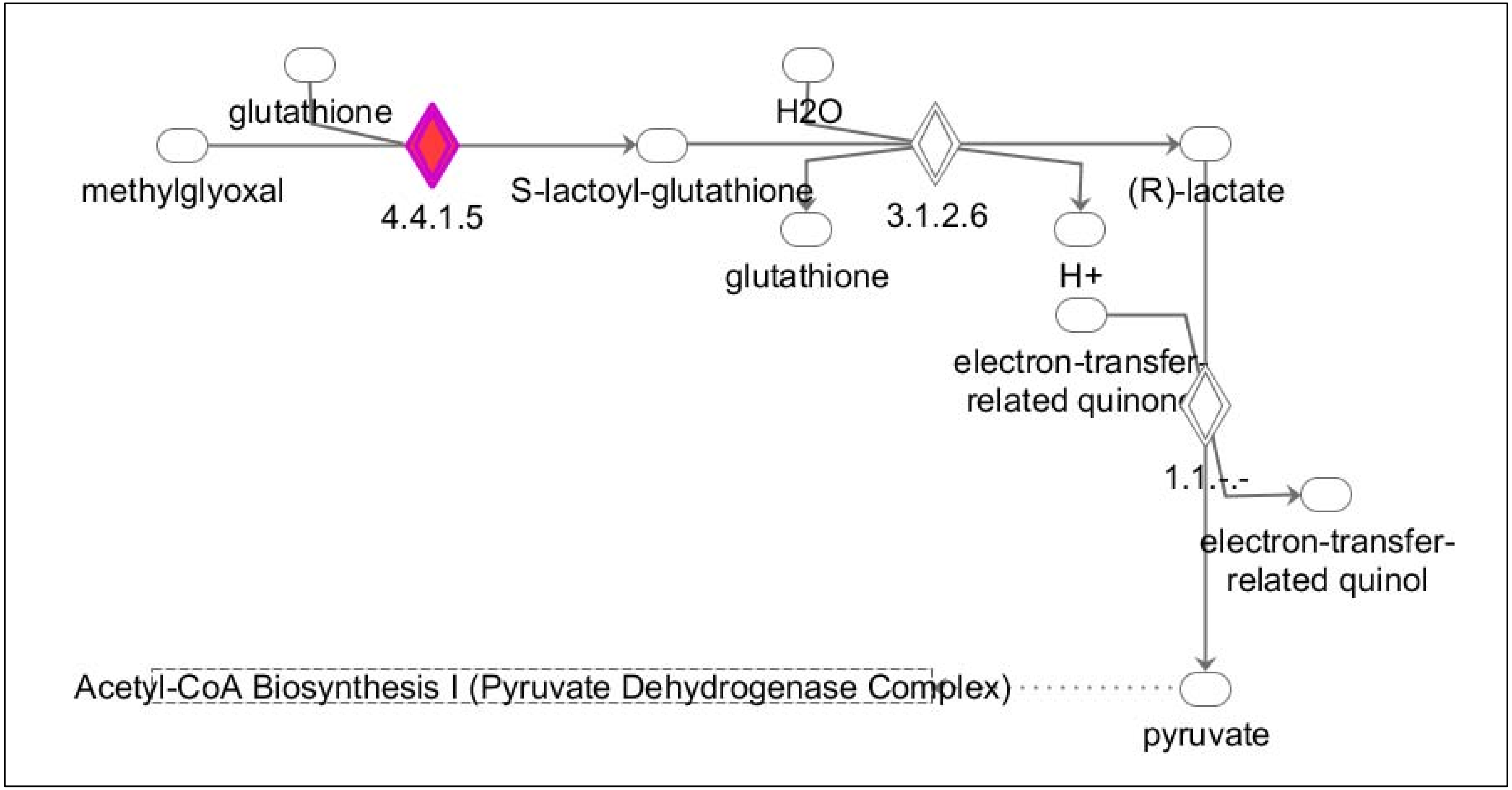

14-IGF-1 Signaling

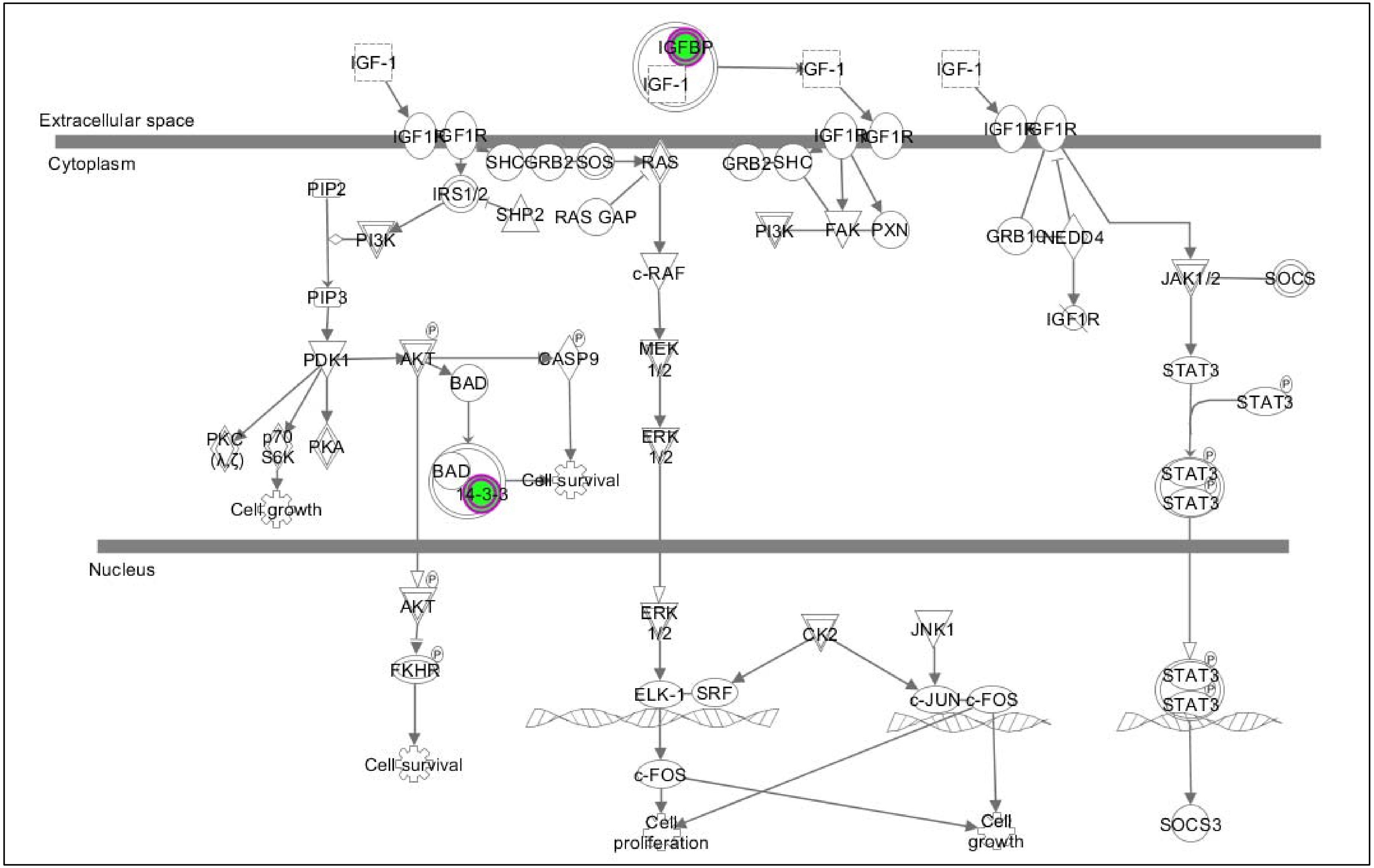

15-Role of Tissue Factor in Cancer

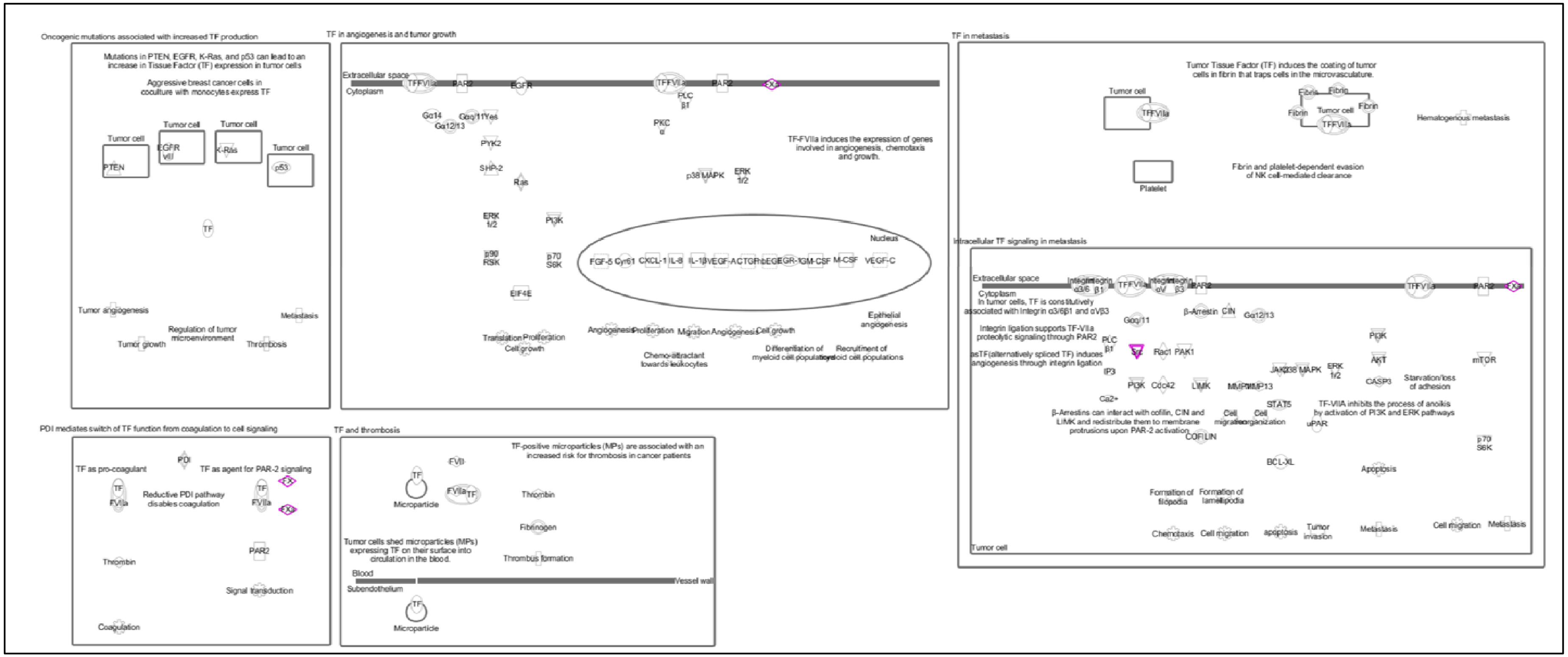

16-Hematopoiesis from Multipotent Stem Cells

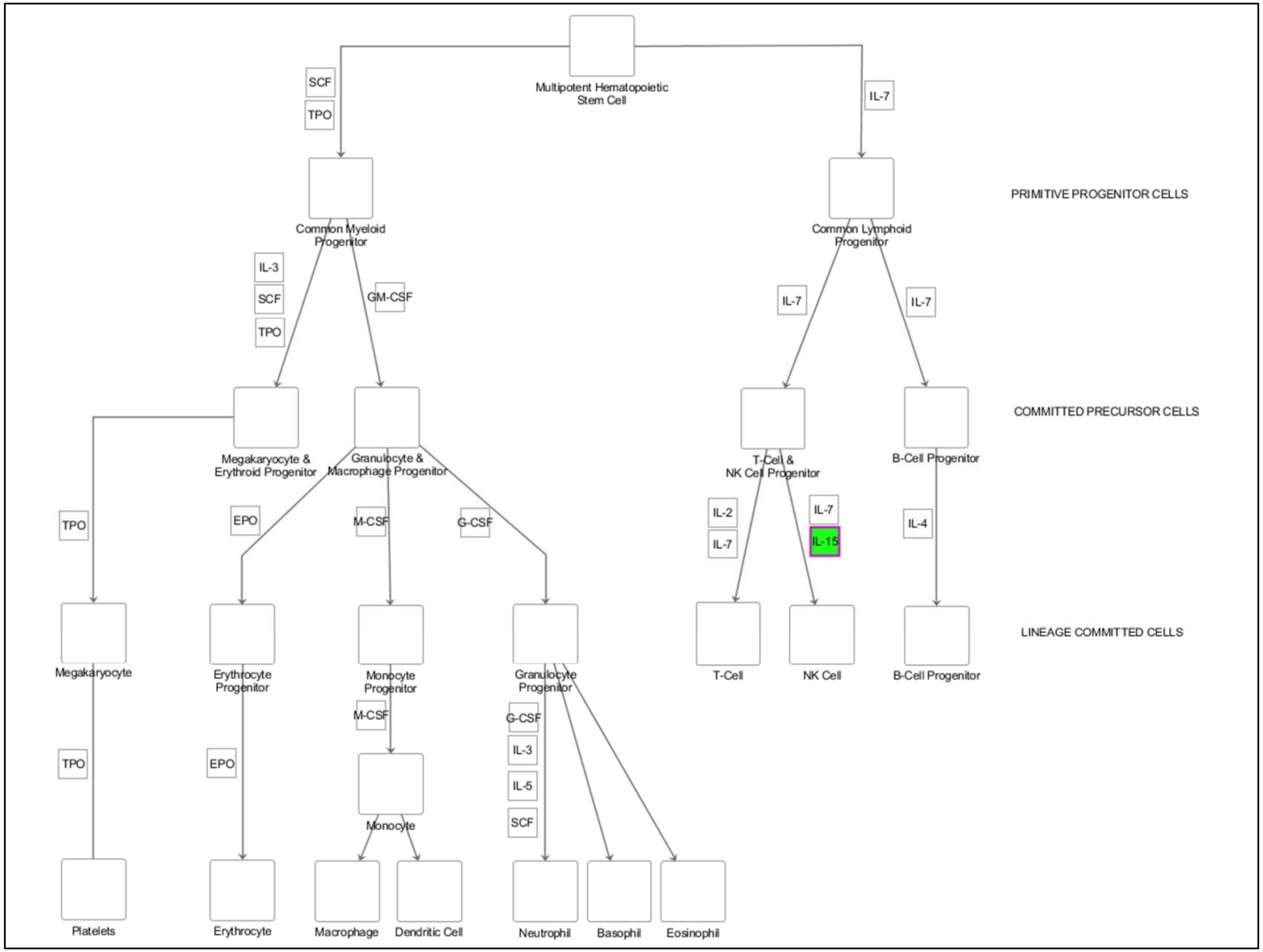

17-14-3-3-mediated Signaling

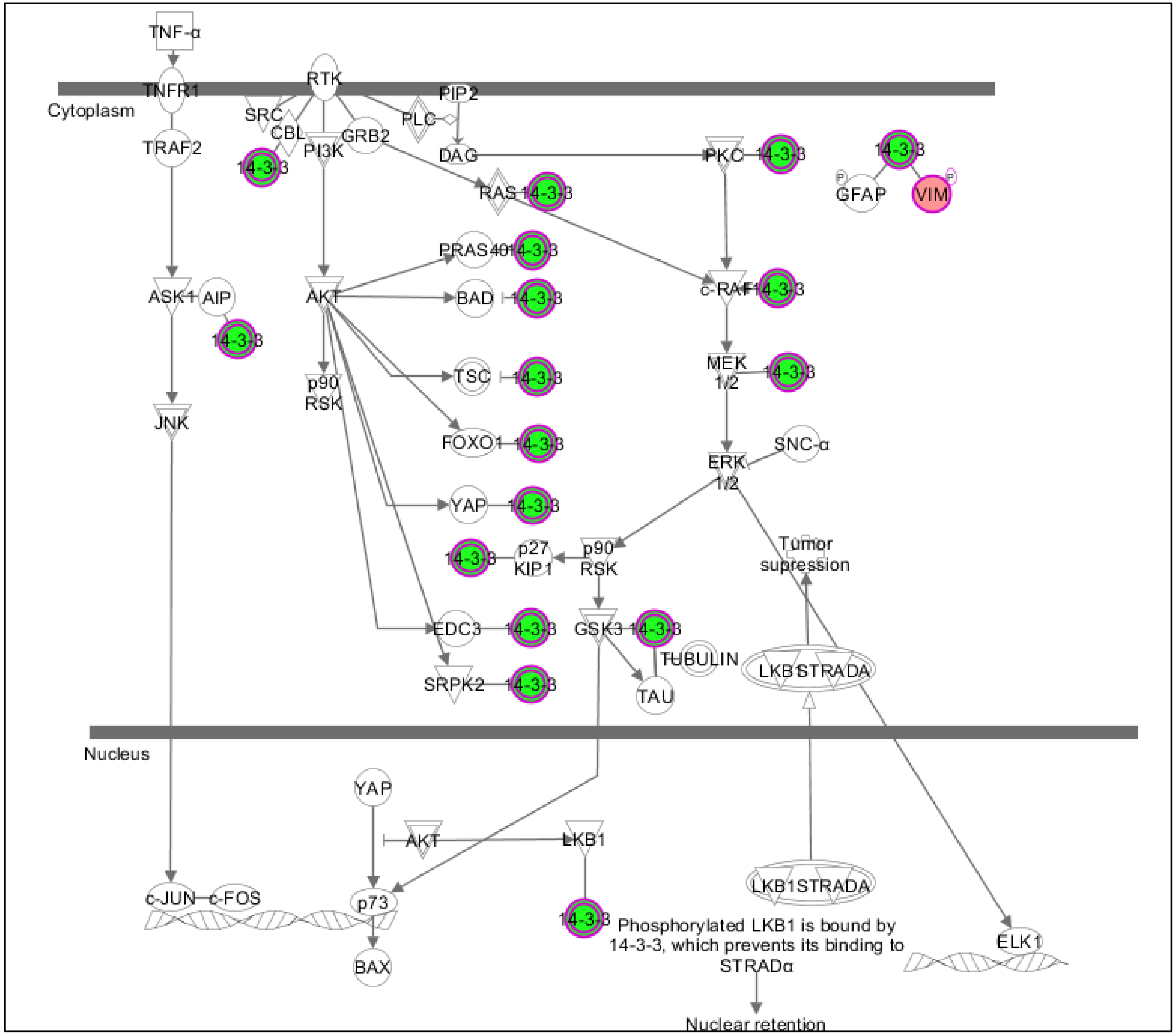

18-Acyl-CoA Hydrolysis

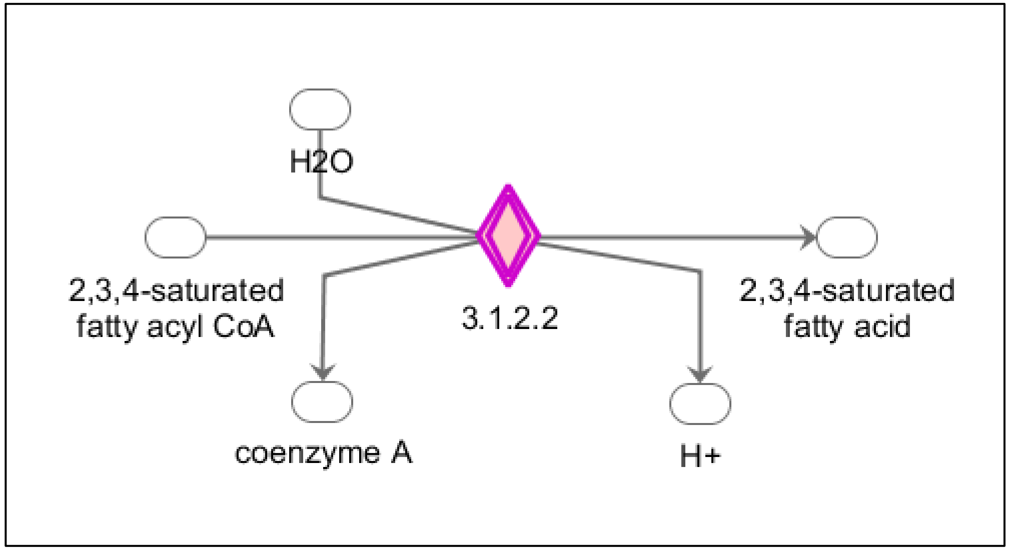

19-PI3K/AKT Sigaling

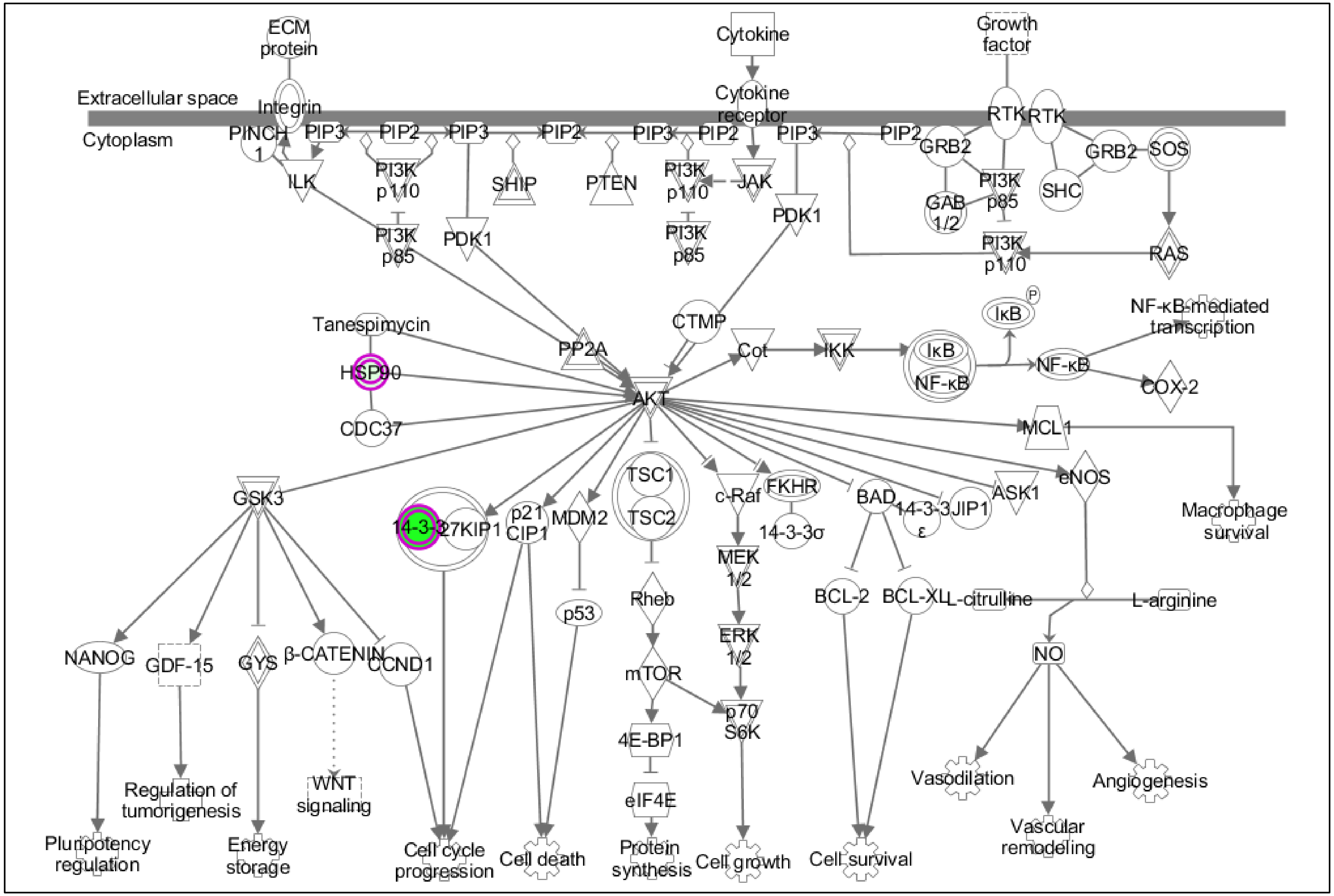

20-Serotonin and melatonin Biosynthess

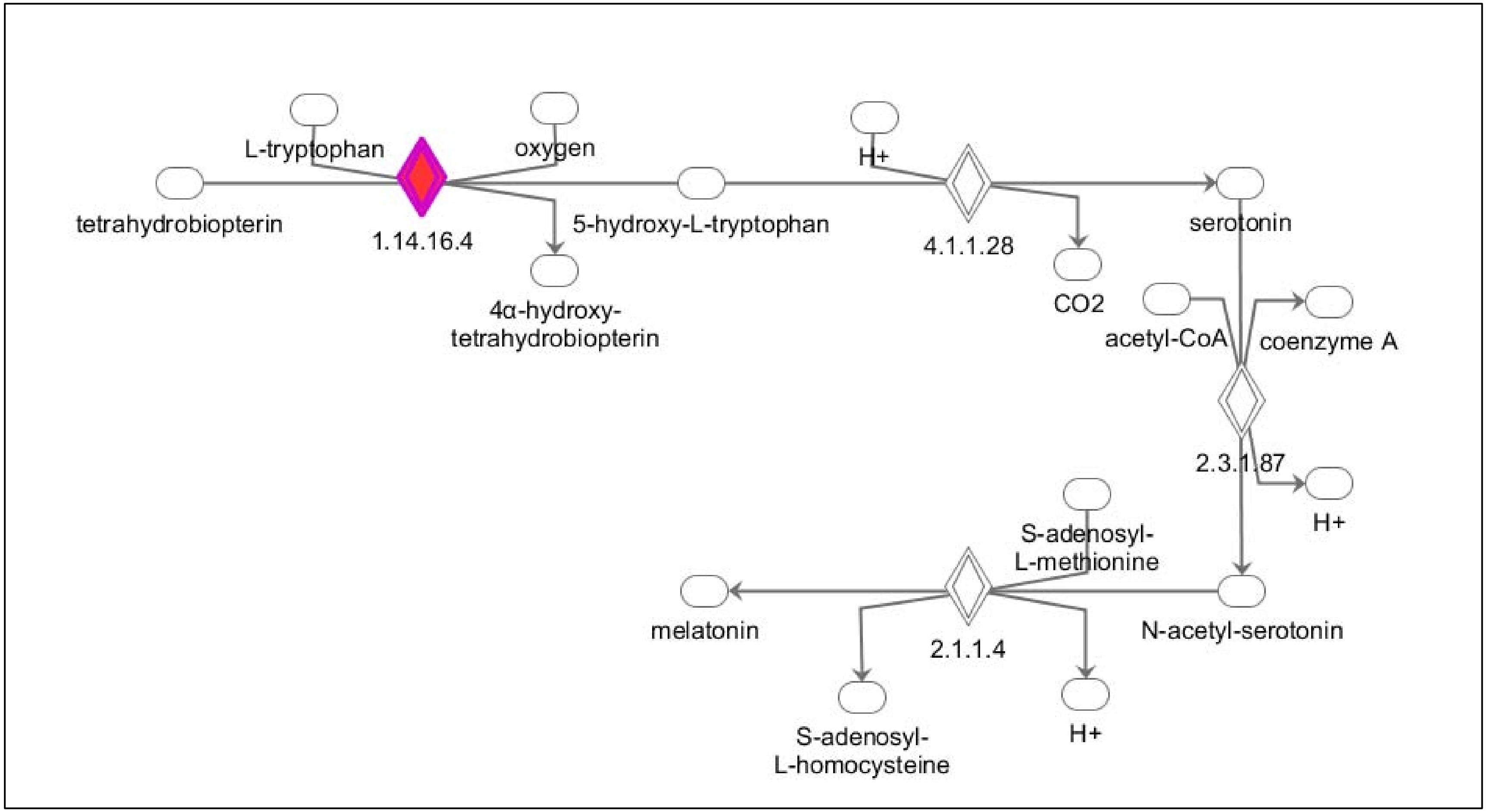

21-Extrinsic Prothrombin Activation Pathway

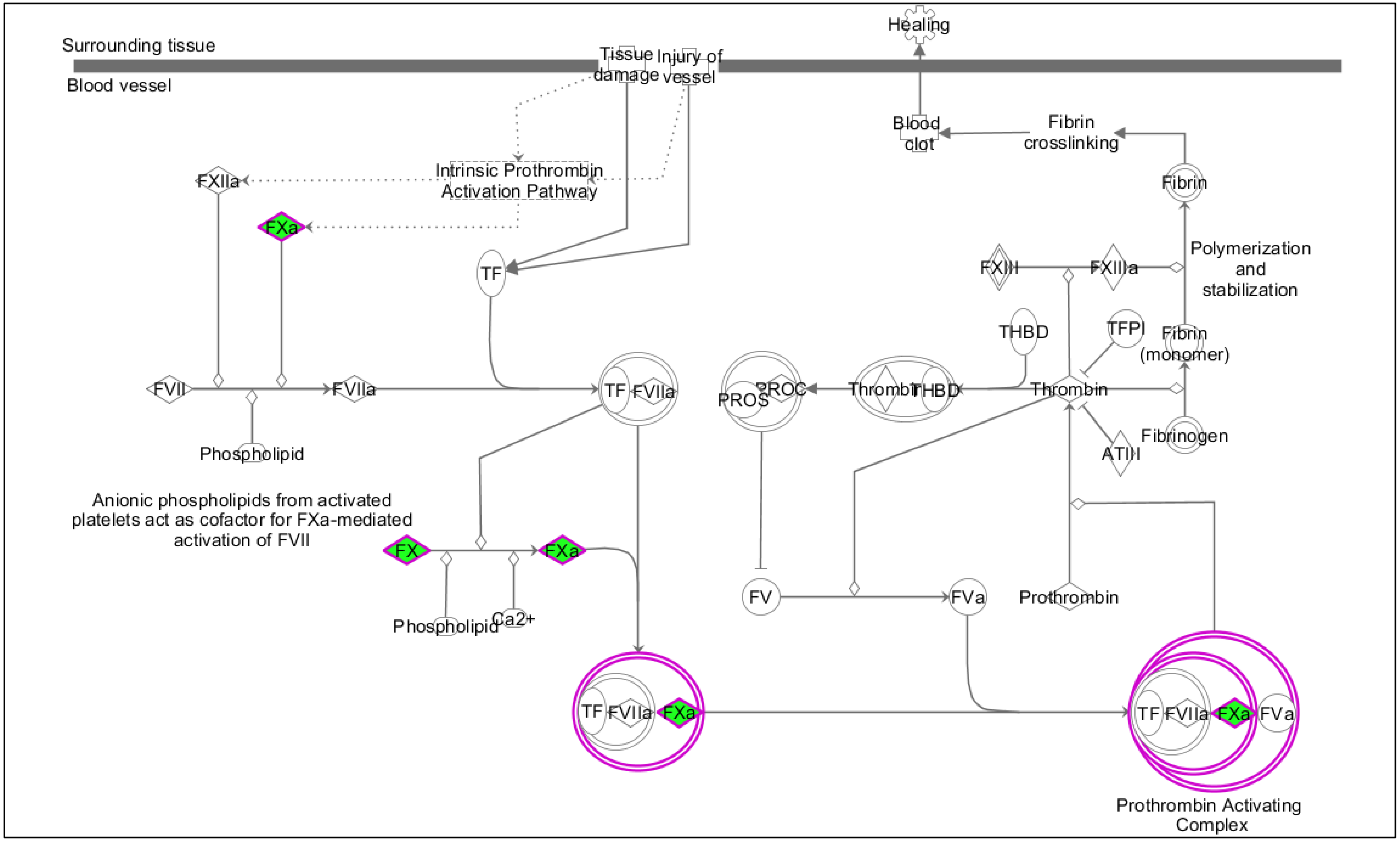

22-Endoplasmic Reticulum Stress Pathway

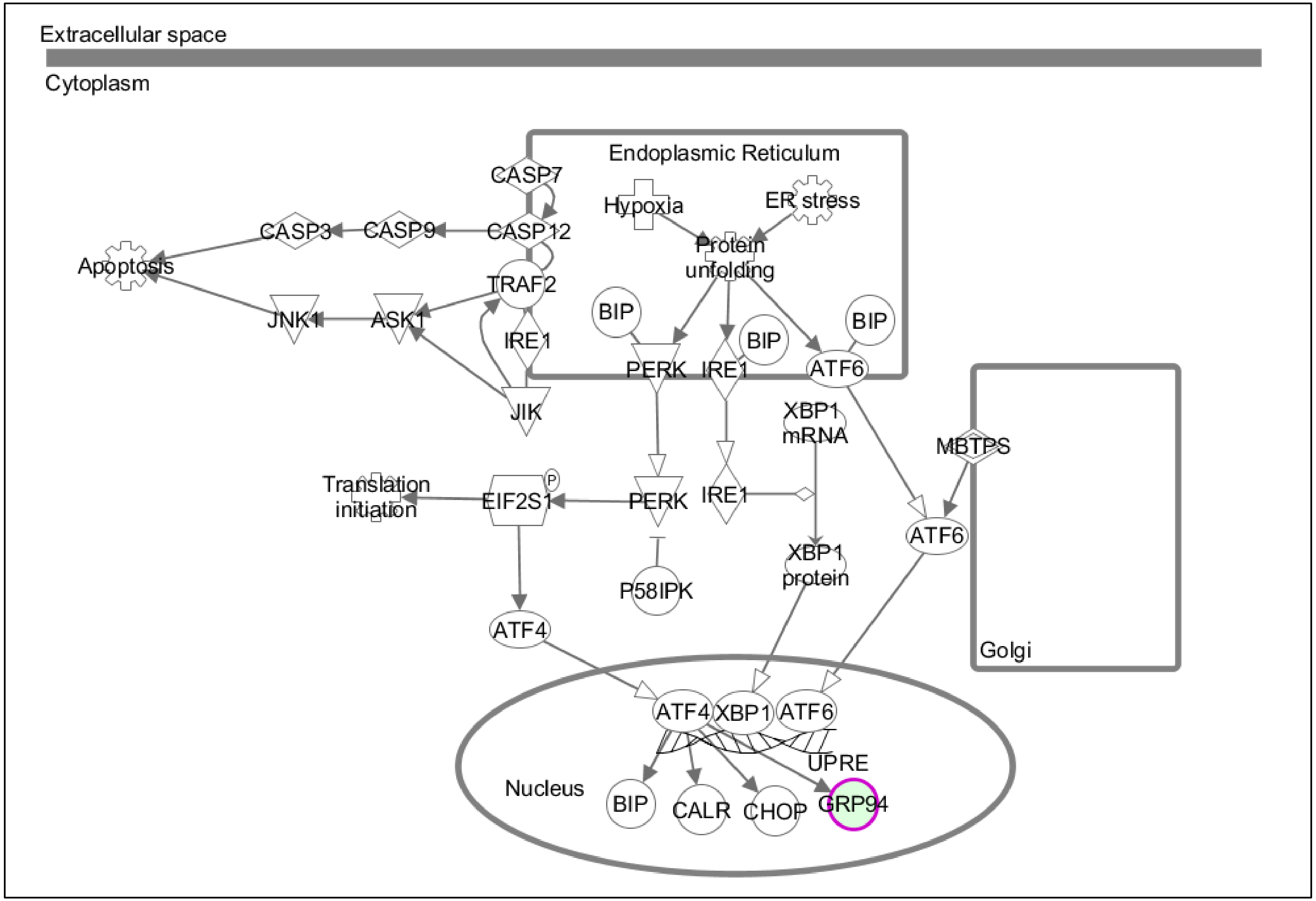

23-Tec Kinase Signaling

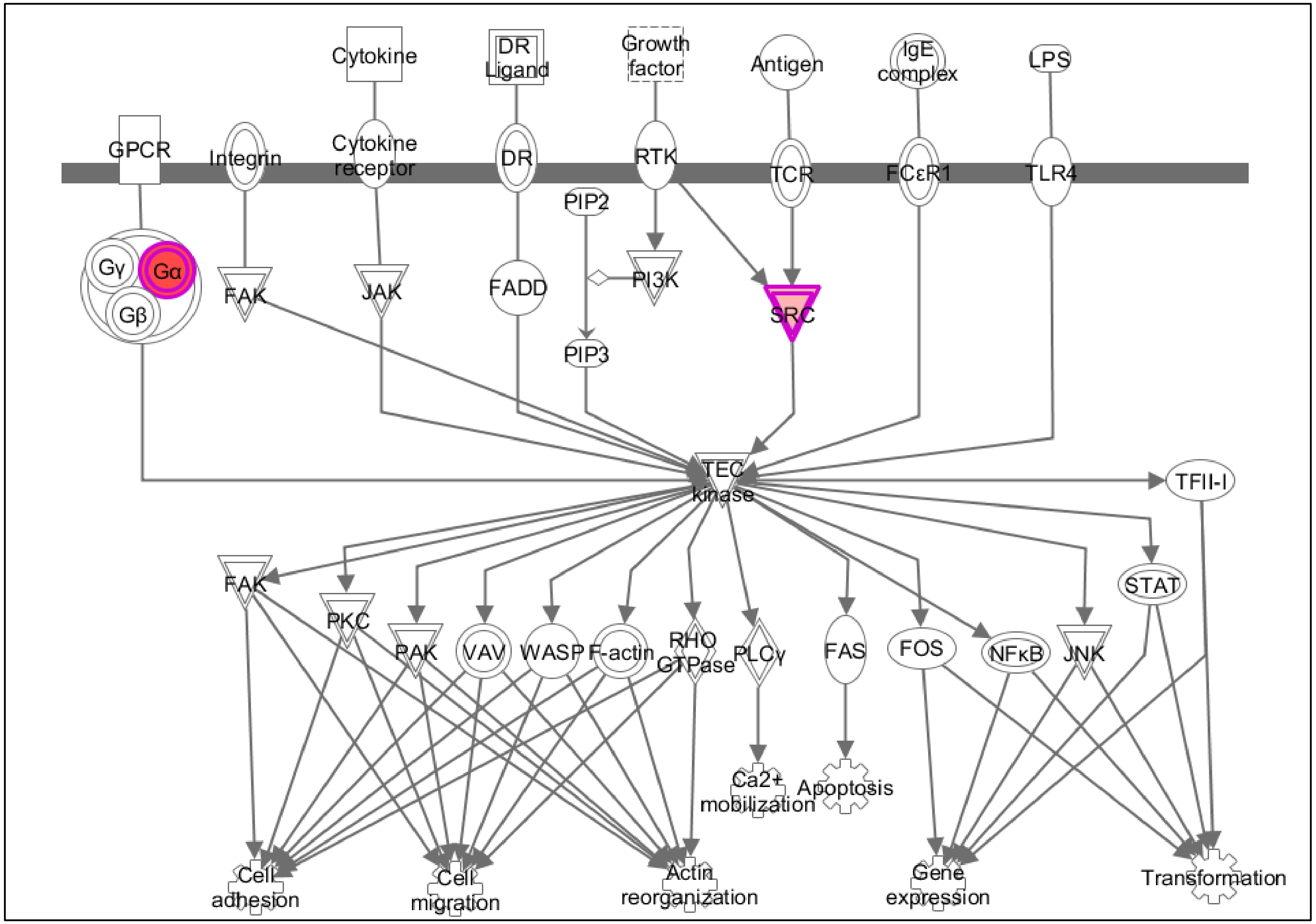

24-Methylglyoxal Degradation III

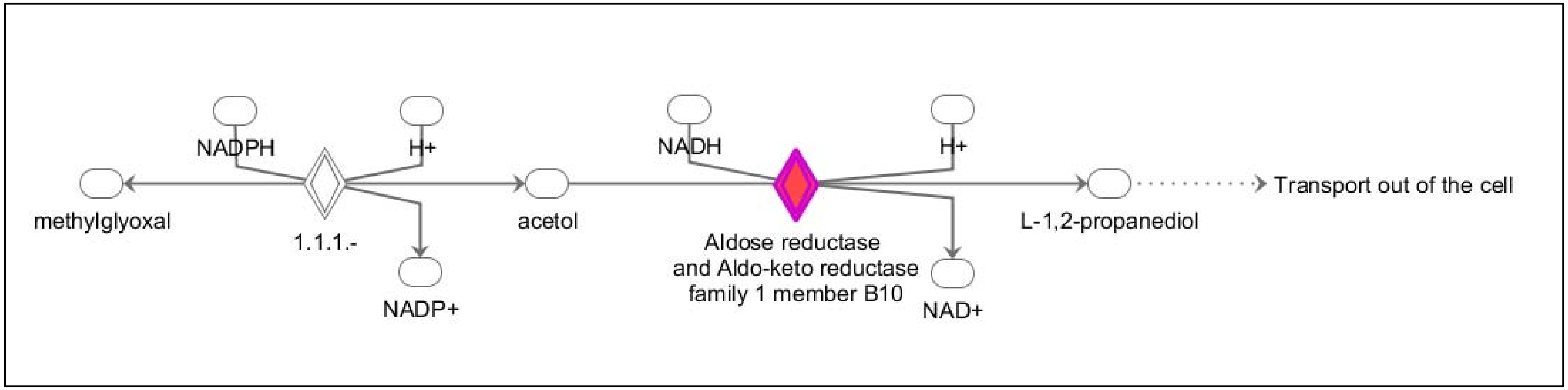

25-Glutathione Redox Reacions Ⅰ

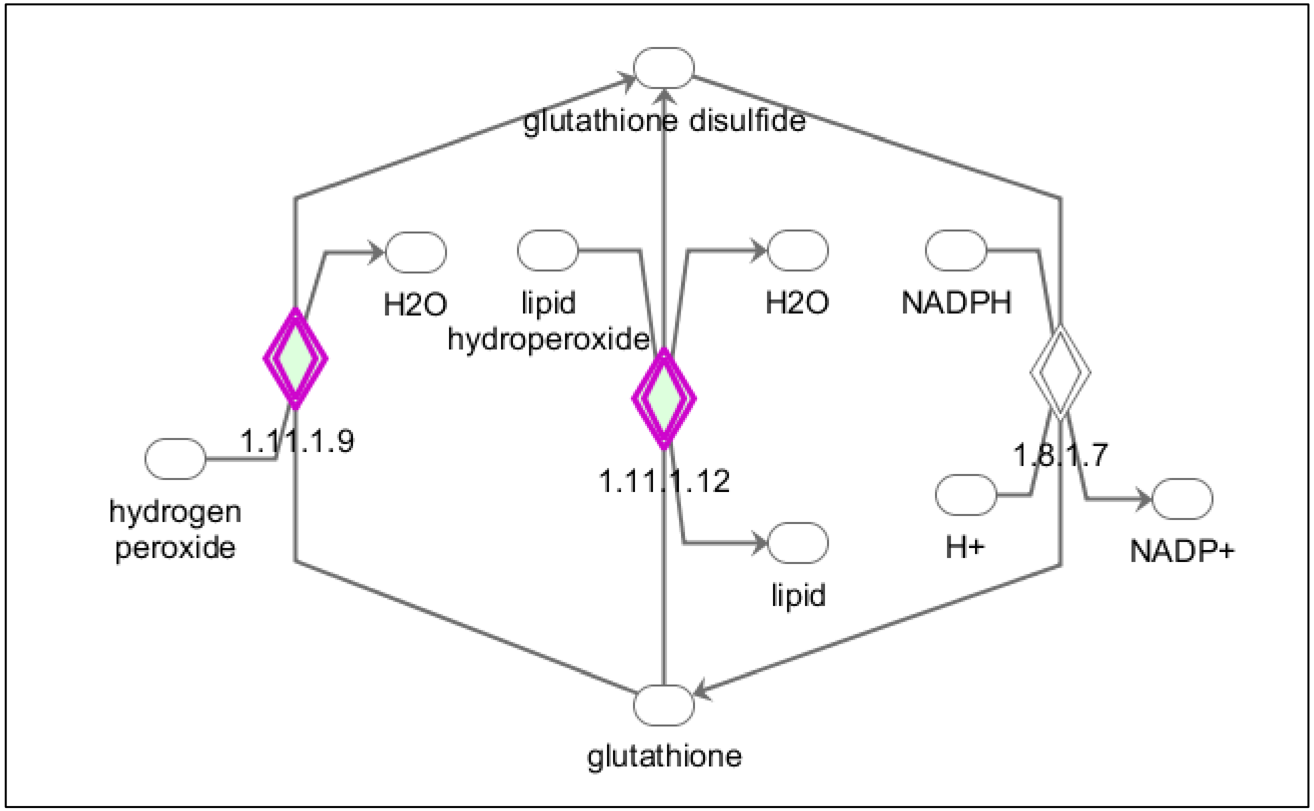

Supplement: Supplementary file 2 [file Presentation_2.zip › Supplemental materials 2.3.pdf]
