# Supplementary material for: Multiomics-Based Signaling Pathway Network Alterations in Human Non-functional Pituitary Adenomas
Source: Front Endocrinol (Lausanne). 2019 Dec 17;10:835. doi: 10.3389/fendo.2019.00835 (PMC6928143; doi:10.3389/fendo.2019.00835)
Supplement: Supplementary file 2 [file Presentation_2.zip › Supplemental materials 3.1.pdf]

Supplemental materials 3.1 Mapping proteins from NFPAs for IPA analysis (Dataset 3)

ID	Notes	Protein Name
gi12805429		Similar to DJ-1 protein [Mus musculus]
gi14249382		Hypothetical protein MGC15429
gi2460318		RNA-binding protein regulatory subunit
O00559		Receptor-binding cancer antigen expressed on SiSo cells
O14753		Putative transcription factor Ovo-like 1 (hOvo 1) (Fragment)
O15266		Short stature homeobox protein
O43422		52 kDa repressor of the inhibitor of the protein kinase
O43488	D	aldo-keto reductase family 1, member A2 (aELatoxin aldehyde reductase)
O43488	D	aldo-keto reductase family 1, member A2 (aELatoxin aldehyde reductase)
O60271		C-Jun-amino-terminal kinase-interacting protein 4
O75489		Chain 1: NADH-ubiquinone oxidoreductase 30 kDa
O75665 (Isoform)		Splice isoform 2 of oral-facial-digital syndrome 1 protein
O75874		Isocitrate dehydrogenase[NADP] cytoplasmic
O75947		ATP synthase D chain, mitochondrial
O76038		Secretagogin
O95336		6-phosphogluconolactonase
O95613		Pericentrin
O95876		WD repeat-containing and planar cell polarity effector protein fritz homolog
O95932		Protein-glutamine gamma-glutamyltransferase 6
P00441		Superoxide dismutase [Cu-Zn]
P00742		Chain 1: Factor X light chain
P00915		Carbonic anhydrase I
P01024		Chain 2: complement C3, beta chain
P01241		Somatotropin precursor

P02545		Prelamin-A/C
P02647		Chain 1: apolipoprotein A-I
P02679	D	Chain 1: Fibrinogen GAMMA chanin
P02679 (isoform)	D	Splice isoform GAMMA-A of fibrinogen gamma chain precursor (PRO2061)
P02743		Chain 1: Serum amyloid P-component
P02768		Serum albumin precursor
P02787		Serotransferrin precursor
P02790		Chain 1: Hemopexin
P02792		Ferritin light chain
P02794		Ferritin heavy chain
P04040		Catalase
P04083		Annexin I
P04181		Ornithine aminotransferase, mitochondrial
P04217		Alpha-1B-glycoprotein
P04350		Tubulin beta-4A chain
P04632		Calcium-dependent protease, small subunit
P04792		Heat shock 27 kDa protein
P05121		Chain 1: Plasminogen activator inhibitor-1
P06241		Proto-oncogene tyrosine-protein kinase FYN
P06396	D	Chain 1: Gelsolin, plasma isoform
P06396	D	Chain 2: Gelsolin, cytoplasmic isoform
P06576		ATP synthase subunit beta, mitochondrial
P06733		Alpha-enolase
P06748		Nucleophosmin
P06753 (Isoform)	D	Splice isoform 2 of tropomyosin alpha 3 chain
P06753 (Isoform)	D	Splice isoform 3 of tropomyosin alpha 3 chain
P07195		L-lactate dehydrogenase B chain

P07237		Chain 1: Protein disulfide isomerase
P07320		Gamma-crystallin D
P07339		Cathepsin D
P07355		Annexin II
P07437		Tubulin beta chain
P07910 (Isoform)		SPLICE isoform C1 of heterogeneous nuclear ribonucleoproteins C1/C2
P07951		Tropomyosin beta chain
P08133		Annexin A6
P08727		Cytokeratin 19
P08758		Annexin V
P09104		Gamma enolase
P09211		Glutathione S-transferase P
P09455		Retinol-binding protein 1
P09471	D	guanine nucleotide binding protein (G protein), alpha activating activity polypeptide 0
P29777	D	guanine nucleotide binding protein (G protein), alpha activating activity polypeptide 0
P09525		Annexin A4
P09936		Ubiquitin carboxyl-terminal hydrolase isozyme L1
P10126		Elongation factor 1-alpha 1
P10809		60 kDa heat shock protein, mitochondrial
P11021		78 kDa glucose-regulated protein precursor
P11142		Heat shock cognate 71 kDa protein
P11171		Protein 4.1
P12270		Nucleoprotein TPR
P12955		Xaa-Pro dipeptidase
P12956		ATP-dependent DNA helicase II,70 kDa subunit
P13765		HLA class II histocompatibility antigen, DO beta chai 门
P14550		Alcohol dehydrogenase [NADP(+)]

P14625		Endoplasmin
P15121		Aldose reductase
P16520		Guanine nucleotide-binding protein G(I)/G(S)/G(T) subunit beta-3
P18669		Phosphoglycerate mutase 1
P20382		Pro-MCH
P21281		V-type proton ATPase subunit B, brain isoforn
P21796		Voltage-dependent anion-selective channel protein 1
P22392		Nucleoside diphosphate kinase B
P22626		Heterogeneous nuclear ribonucleoproteins A2/B1
P23526		Adenosylhomocysteinase
P24844		Myosin regulatory light chain 2, smooth muscle isoform
P26641		Elongation factor 1-gamma
P27348		14-3-3 protein theta
P27797		Chain 1: Calreticulin
P28161		Glutathione S-transferase Mu 2
P29218		Inositol monophosphatase 1
P29320		Ephrin type-A receptor 3
P29373		Retinoic acid-binding protein II, cellular
P30040		Chain 1: Endoplasmic reticulum protein ERP29
P30041		Antioxidant protein 2
P30043		Flavin reductase
P30084		Chain 1: ENOYL-COA hydratase
P30101		Chain 1: Protein disulfide isomerase A3
P30153		Serine/threonine-protein phosphatase 2A 65 kDa regulatory subunit A alpha isoform
P30443	D	Chain 1: HLA class I histocompatibility antigen
P30455	D	Chain 1: HLA class I histocompatibility antigen
P30566	D	Adenylosuccinate lyase

P30566 (Isoform)	D	SPLICE Isoform 2 of Adenylosuccinate lyase
P30626		Sorcin (22 kD protein)
P30740		Leukocyte elastase inhibitor
P31150		Rab GDP dissociation inhibitor alpha
P31350		Ribonucleoside-diphosphate reductase subunit M2
P31930		Cytochrome b-c1 complex subunit 1， mitochondrial
P31946	D	Chain 1: 14-3-3 protein beta/alpha, long isoform
P31946	D	Chain 2: 14-3-3 protein beta/alpha, short isoform
P32119		Peroxiredoxin-2
P32321		Deoxycytidylate deaminase
P35232		Prohibitin
P35520		Cystathionine beta-synthase
P35527		Keratin, type I cytoskeletal 9
P35908		Keratin, type II cytoskeletal 2 epidermal
P35998		26S protease regulatory subunit 7
P38606		V-type proton ATPase catalytic subunit A
P40925		Malate dehydrogenase, cytoplasmic
P40926		Malate dehydrogenase, mitochondrial precursor
P41279	D	Chain 1: Mitogen activated protein kinase kinase kinase8
P41279	D	Chain 2: Mitogen activated protein kinase kinase kinase8
P45880		Voltage-dependent anion-selective channel protein 2
P48443		Retinoic acid receptor RXR-gamma
P49368		T-complex protein 1, gamma subunit
P49411		Elongation factor Tu, mitochondrial
P50148		Guanine nucleotide-binding protein G(q) subunit alpha
P50213		Chain 1: Isocitrate dehydrogenase [NAD] subunit
P50395		Rab GDP dissociation inhibitor beta

P51946		Cyclin H
P54920		Alpha-soluble NSF attachment protein
P55084		Trifunctional enzyme beta subunit, mitochondrial precursor
P60174		Triosephosphate iso me rase
P60228		Eukaryotic translation initiation factor 3 subunit E
P60709		Actin, cytoplasmic 1
P61019		Ras-related protein Rab-2A
P61163		Alpha-centractin
P61978		Heterogeneous nuclear ribonucleoprotein K
P63244		Guanine nucleotide-binding protein subunit beta-2-like 1
P63261		Actin, cytoplasmic 2
P68363		Tubulin alpha-1 B chain
P68366		Tubulin alpha-4A chain
P68371		Tubulin beta-4B chain
P78417		Glutathione transferase omega 1
Q00169		Phosphatidylinositol transfer protein alpha isoform
Q00613	D	Heat shock protein 1
Q00613 (isoform)	D	Splice short isoform of heat shock factor protein 1
Q06323		Proteasome activator complex subunit 1
Q12765		Secernin-1
Q13435		Splicing factor 3B subunit 2
Q13451		51 kDa FK506-binding protein
Q13509		Tubulin beta-3 chain
Q13885		Tubulin beta-2A chain
Q14192		Skeletal muscle LIM-protein 3
Q14204		Cytoplasmic dynein 1 heavy chain 1
Q15120		[Pyruvate dehydrogenase (acetyl-transferring)] kinase isozyme 3， mitochondrial

Q15365	Poly(Rc)-binding protein 1
Q16352	Alpha-internexin
Q499Z4	Zinc finger protein 672
Q4V348	Zinc finger protein 658B
Q5JSL3	Dedicator of cytokinesis protein 11
Q5SSJ5	Heterochromatin protein 1-binding protein 3
Q5T1B0	Axonemal dynein light chain domain-containing protein 1
Q5TYW1	Zinc finger protein 658
Q6NSZ9	Zinc finger and SCAN domain-containing protein 25
Q6PEY2	Tubulin alpha-3E chain
Q6ZN84	Coiled-coil domain-containing protein 81
Q6ZV56	Uncharacterized protein C22orf34
Q71U36	Tubulin alpha-1A chain
Q7Z7B0	Filamin-A-interacting protein 1
Q86V25	Vasohibin-2
Q86Y79	Probable peptidyl-tRNA hydrolase
Q86YM7	Homer protein homolog 1
Q8N0U8	Vitamin K epoxide reductase complex subunit 1-like protein 1
Q8N5Y8	Mono [ADP-ribose] polymerase PARP16
Q8NCM8	Cytoplasmic dynein 2 heavy chain 1
Q8NEV4	Myosin-llla
Q93034	Cullin-5
Q96K21	Zinc finger FYVE domain-containing protein 19
Q96KD3	Protein FAM71F1
Q96P16	Regulation of nuclear pre-mRNA domain-containing protein 1A
Q99536	Synaptic vesicle membrane protein VAT-1 homolog
Q99714	3-hydroxyacyl-CoA dehydrogenase type II

Q9BQE3		Tubulin alpha-1C chain
Q9BTV4		Transmembrane protein 43
Q9BVA1		Tubulin beta-2B chain
Q9NRD0		F-box only protein 8
Q9NTG1		Chain 1: Polycystic kidney disease and receptor
Q9NY65		Tubulin alpha-8 chain
Q9NZL3		Zinc finger protein 224
Q9P0V2		Mitofilin (fragment)
Q9P2R7	D	Chain 12: succinyl-CoA ligase[ADP-forming] beta A
Q9P2R7 (Isoform)	D	SPLICE isoform 2 of succinyl-CoA ligase [ADP-forming] beta-chain, mitochondrial precursor
Q9UBX7 (Isoform)		SPLICE Isoform 2 of Kallikrein 11 precursor
Q9UFN0		Protein NipSnap homolog 3A
Q9UJA3		DNA helicase MCM8
Q9UL46		Proteasome activator complex subunit 2
Q9Y2S2		Lambda-crystallin homolog
Q9Y4A5		Transformation/transcription domain-associated protein
O75198	unmapped	
P00938	unmapped	
P02023	unmapped	
P05092	unmapped	
P07226	unmapped	
P08107	unmapped	
P10990	unmapped	
P11016	unmapped	
P16475	unmapped	
P24572	unmapped	
P32391	unmapped	

P38607	unmapped
P42655	unmapped
Q03527	unmapped
Q13748	unmapped
Q29459	unmapped
