# Supplementary material for: Multiomics-Based Signaling Pathway Network Alterations in Human Non-functional Pituitary Adenomas
Source: Front Endocrinol (Lausanne). 2019 Dec 17;10:835. doi: 10.3389/fendo.2019.00835 (PMC6928143; doi:10.3389/fendo.2019.00835)

Supplemental materials 3.3
Statistically significant canonical pathways derived from mapping proteins from NFPAs for IPA analysis (Dataset 3)

1. Remodeling of epithelial adherens junctions
2. 14-3-3-mediated signaling
3. Germ Cell-Sertoli Cell Junction Signaling
4. Gap Junction Signaling
5. Epithelial Adherens Junction Signaling
6. Sertoli Cell-Sertoli Cell Junction Signaling
7. Breast Cancer Regulation by Stathmin 1
8. Axonal Guidance Signaling
9. NRF2-mediated Oxidative Stress Response
10. Acute Phase Response Signaling
11. Huntington's Disease Signaling
12. LXR/RXR Activation
13. RhoGDI Signaling
14. Mitochondrial Dysfunction
15. Gluconeogenesis I
16. Protein Ubiquitination Pathway
17. Unfolded Protein Response
18. Antigen Presentation Pathway
19. Aryl Hydrocarbon Resceptor Signaling
20. TCA Cycle Ⅱ（Eukaryotic）
21. Glycolysis Ⅰ
22. Caveolar-mediated Endocytosis Signaling
23. Androgen Signaling
24. Ephrin B Signaling
25. Tec Kinase Signaling
26. Aldosterone Signaling in Epithelial Cells
27. Tight Junction Signaling
28. Endoplasmic Reticulum Stress Pathway
29. Methylglyoxal Degradation Ⅲ
30. Cardiac Hypertrophy Signaling
31. Death Receptor Signaling
32. FXR/RXR Activation
33. Isoleucne Degradation Ⅰ
34. Superoxide Radicals Degradation
35. ERK5 Signaling
36. Glucocorticoid Receptor Signaling
37. Xenobiotic Metabolism Signaling
38. Role of Tissue Factor in Cancer
39. Coagulation System
40. G Protein Signaling Mediated by Tubby
41. D-glucuronate Degradation Ⅰ
42. Role of NFAT in Regulation of the Immune Response
43. Ephrin Receptor Signaling
44. Glutathionemediated Detoxification
45. Signaling by Rho Family GTPases
46. p70S6K Signaling
47. PI3K/AKT Signaling
48. Fatty Acid β-oxidation Ⅰ
49. Clathrin-mediated Endocytosis Signaling
50. Aspartate Degradation Ⅱ
51. Telomere Extension by Telomerase
52. Thrombin Signaling
53. FAK Signaling
54. Parkinson’s Signalng
55. TR/RXR Activation
56. IL-1 Signaling
57. Virus Entry via Endocytic Pathways
58. Synaptic Long Term Depression
59. Extrinsic Prothrombin Activation Pathway
60. G Beta Gamma Signaling
61. CXCR4 Signaling
62. Glutaryl-CoA Degradation
63. Polyamine Regulation in Colon Cancer
64. CREB Signaling in Neurons
65. Retinoic acid Mediated Apoptosis Signaling
66. Oxidative Phosphorylation
67. Lipid Antigen Presentation by CD1
68. PPARα/RXRα Activation
69. Production of Nitric Oxide and Reactive Oxygen Species in Macrophages
70. ERK/MAPK Signaling
71. P2Y Purigenic Receptor Signaling Pathway
72. Intrinsic Prothrombin Activation Pathway
73. Melatonin Signaling
74. Cysteine Biosynthesis Ⅲ(mammalia)
75. Integrin Signaling
76. Cellular Effects of Sildenafil (Viagra)
77. Valine Degradation Ⅰ
78. Relaxin Signaling
79. HIPPO Signaling
80. CTLA4 Signaling in Cytotoxic T Lymphocytes
81. Crosstalk between Dendritic Cells and Natural Killer Cells
82. eNOS Signaling
83. Pyrimidine Deoxyribonucleotides De Novo Biosynthesis Ⅰ
84. Regulation of Actin-based Motility by Rho
85. Mechanisms of Viral Exit from Host Cells
86. α-Adrenergic Signaling
87. Ethanol Degradation Ⅱ
88. Fcγ Receptor-mediated Phagocytosis in Macrophages and Monocytes
89. Tryptophan Degradation Ⅲ(Eukaryotic)

Dataset 3-Canonical Pathway Chart

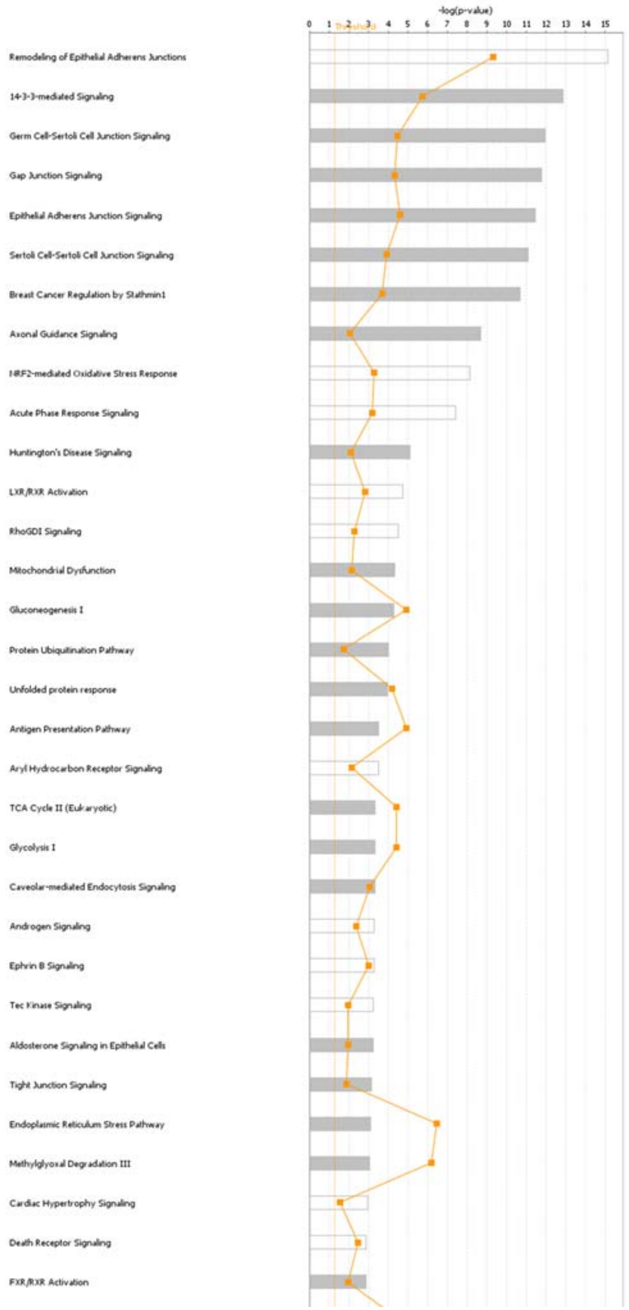

1-Remodeling of epithelial adherens junctions

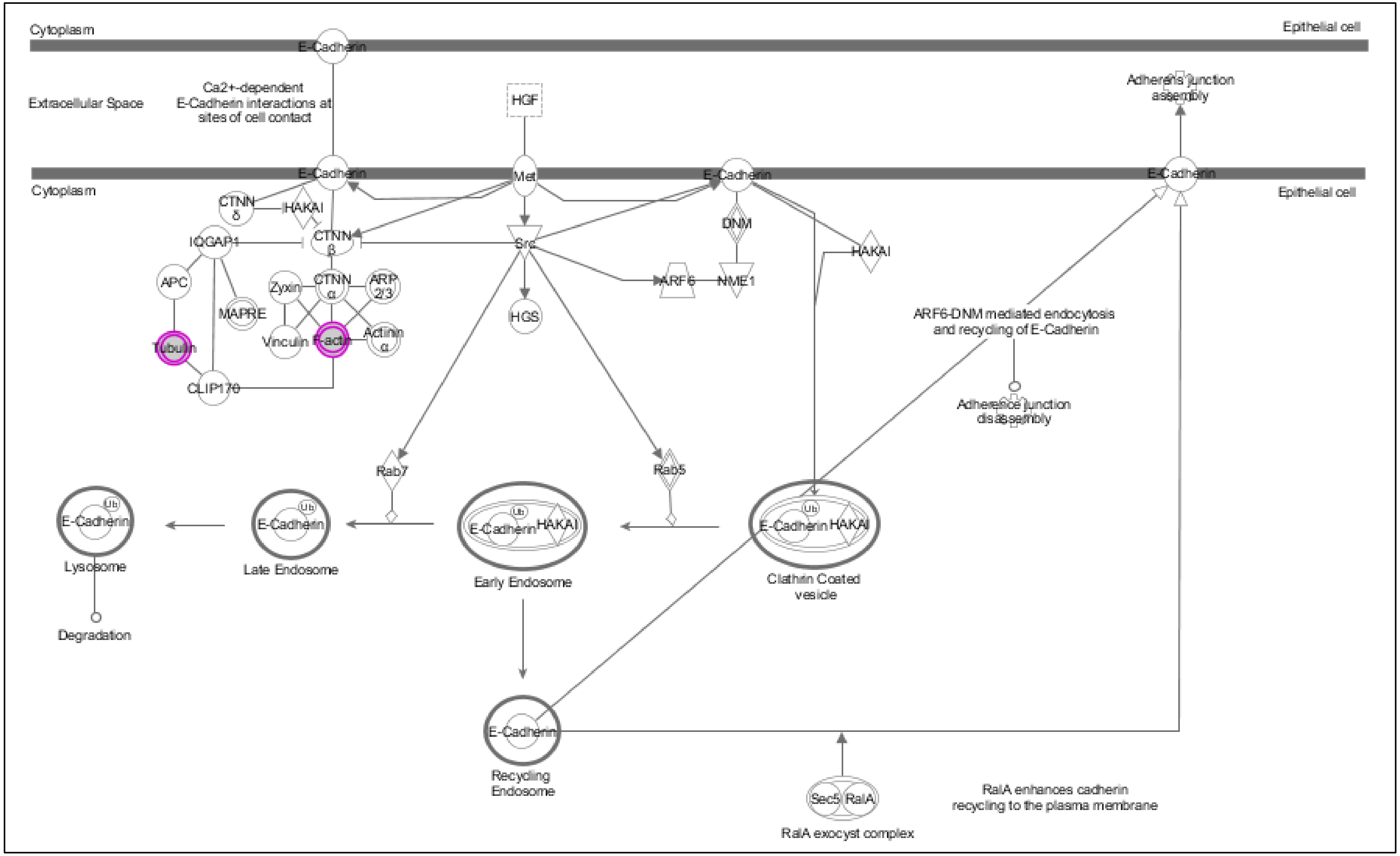

2-14-3-3-mediated signaling

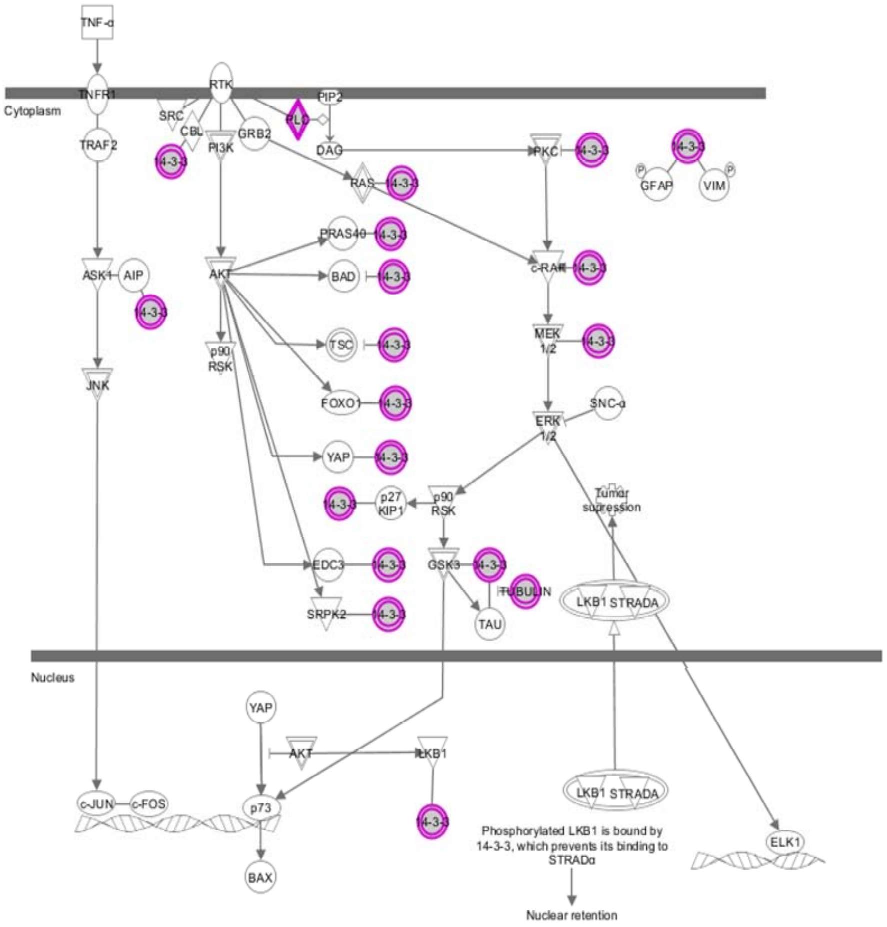

3-Germ Cell-Sertoli Cell Junction Signaling

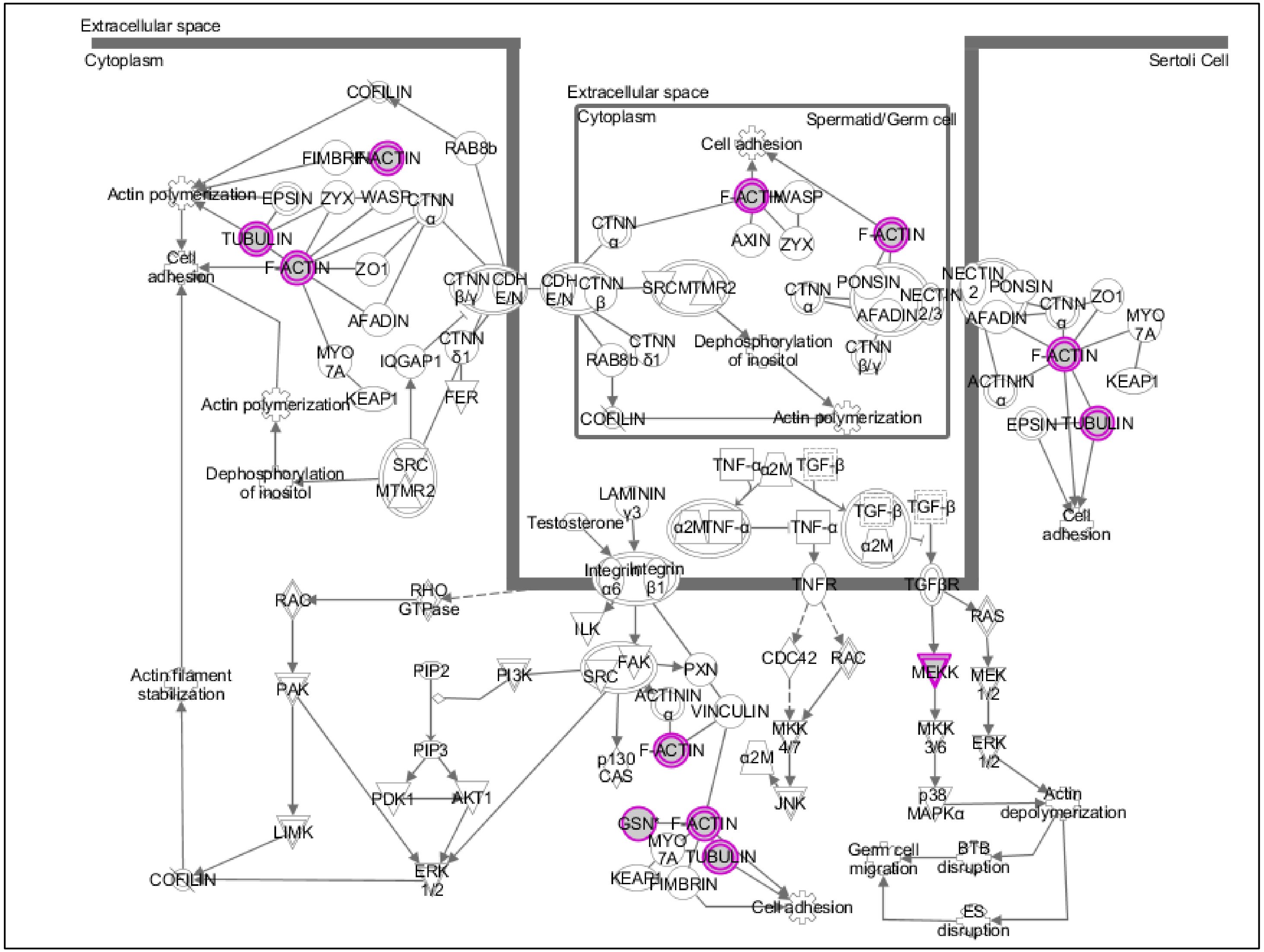

4-Gap Junction Signaling

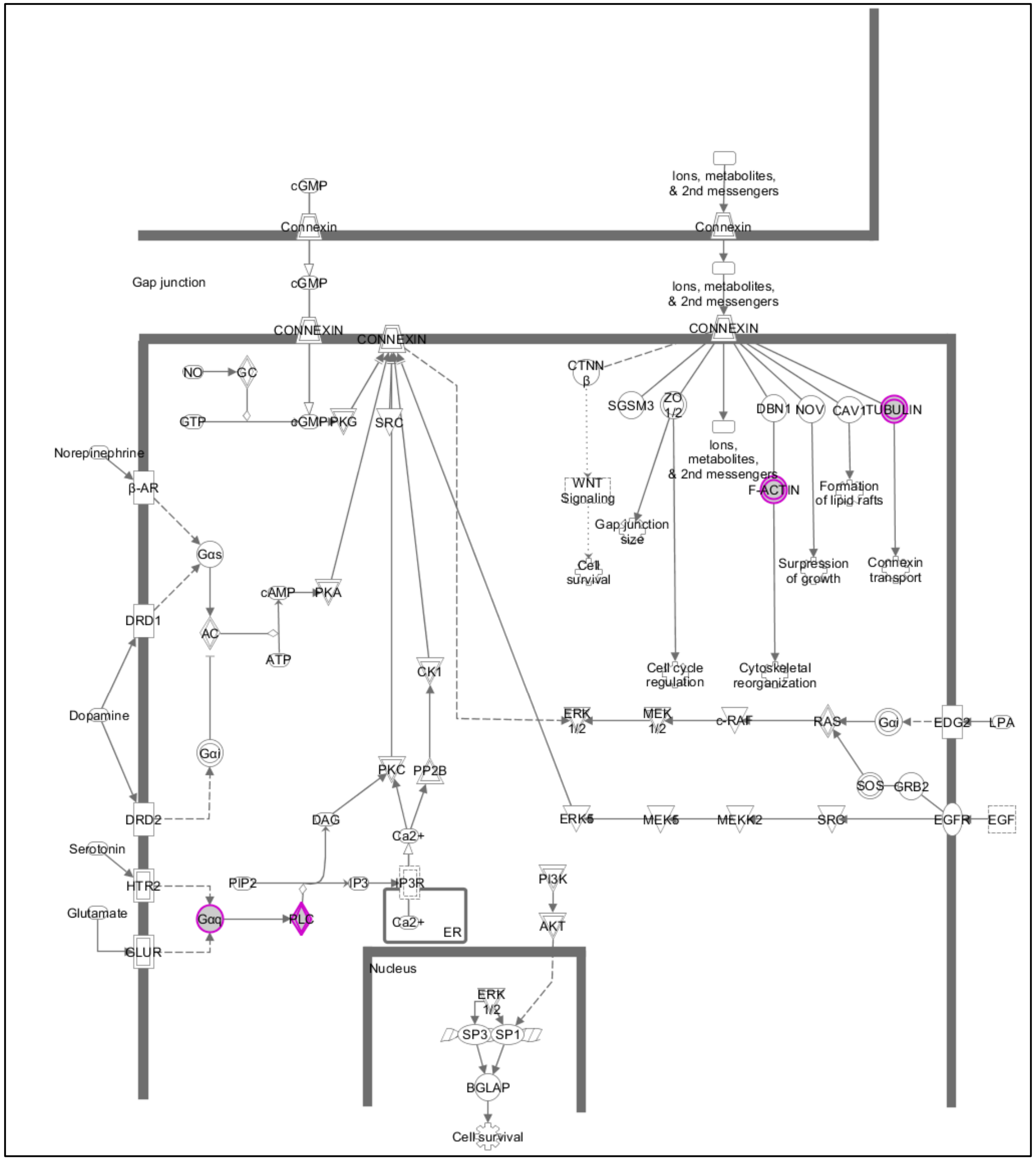

5-Epithelial Adhereences Junction Signaling

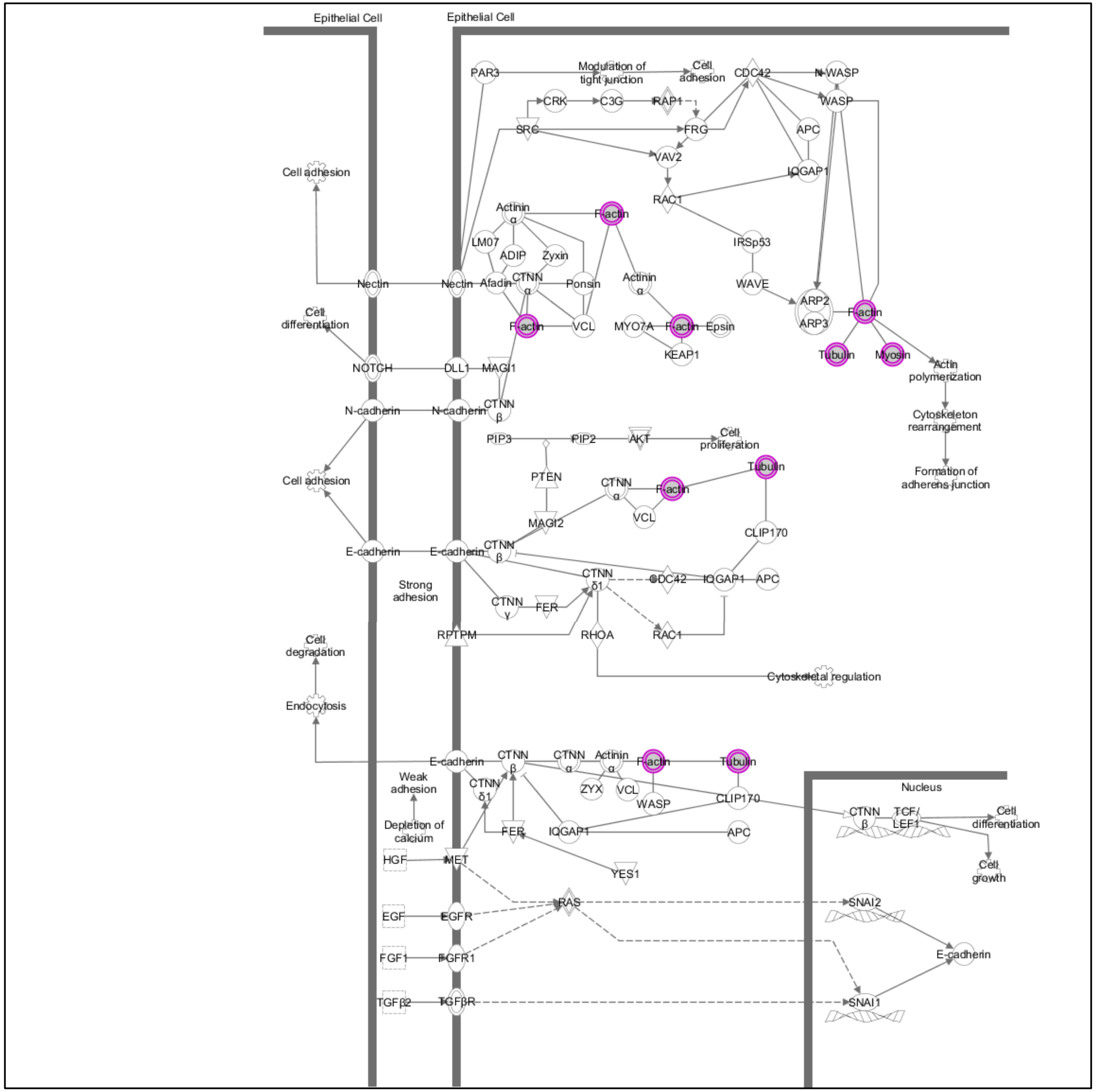

6-Sertoli Cell-Sertoli Cell Junction Signaling

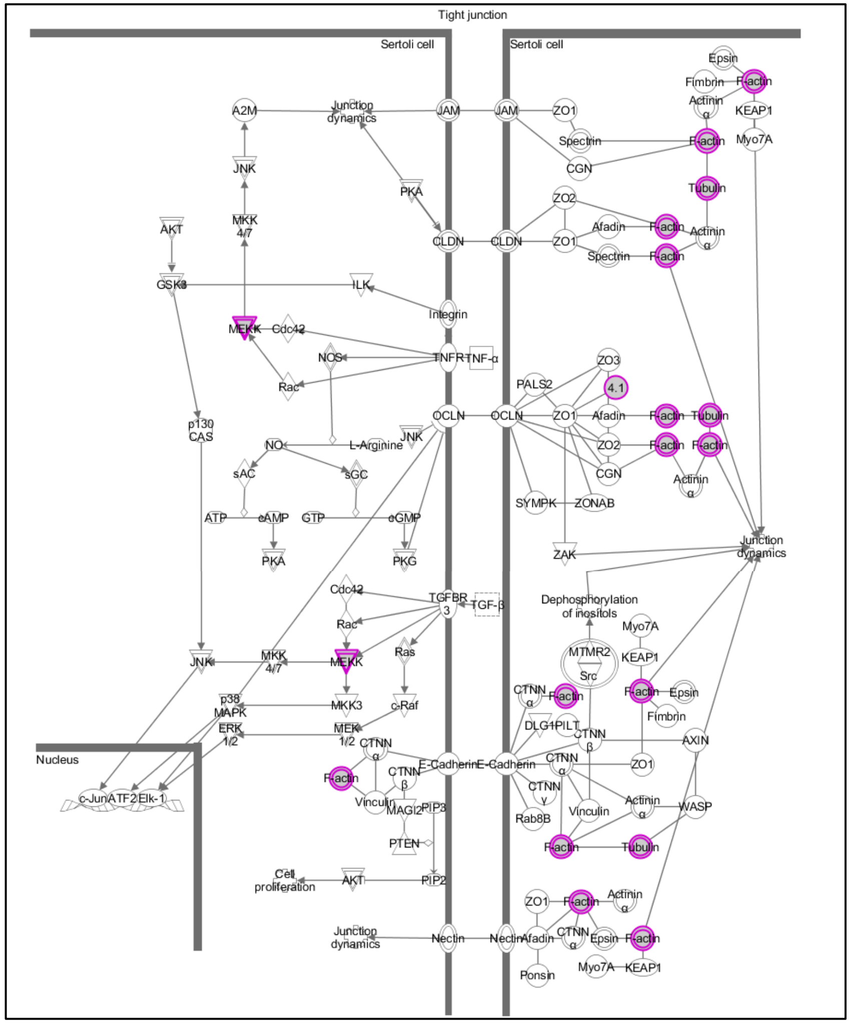

7-Breast Cancer Regulation by Stathmin 1

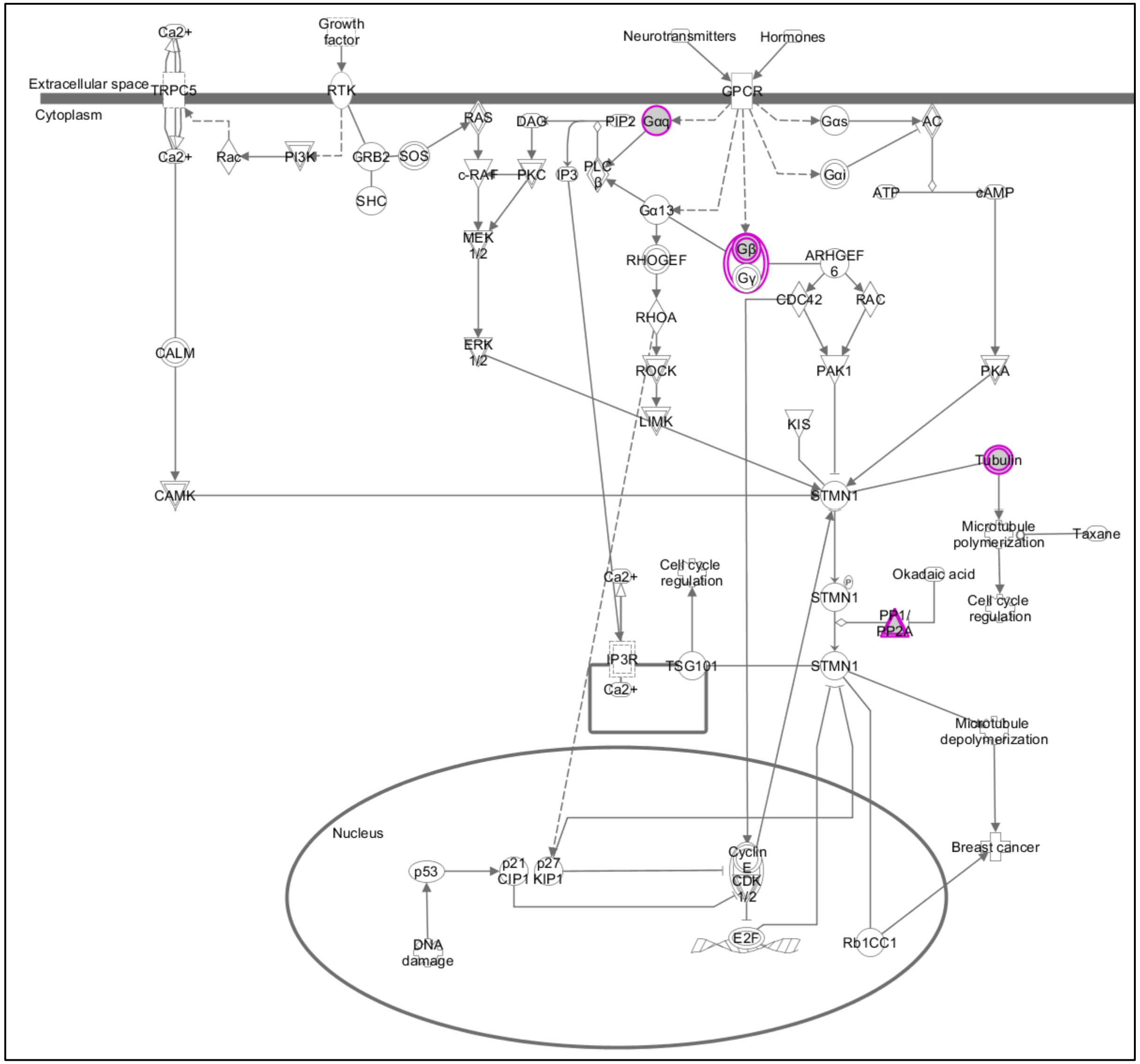

8-Axonal Guidance Signaling

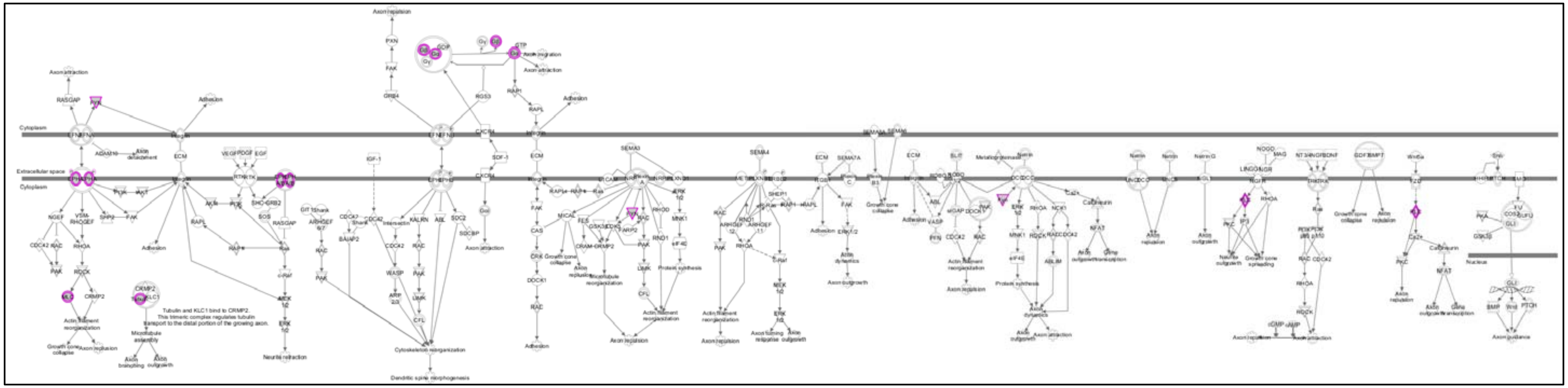

9-NRF2-mediated Oxidative Stress Response

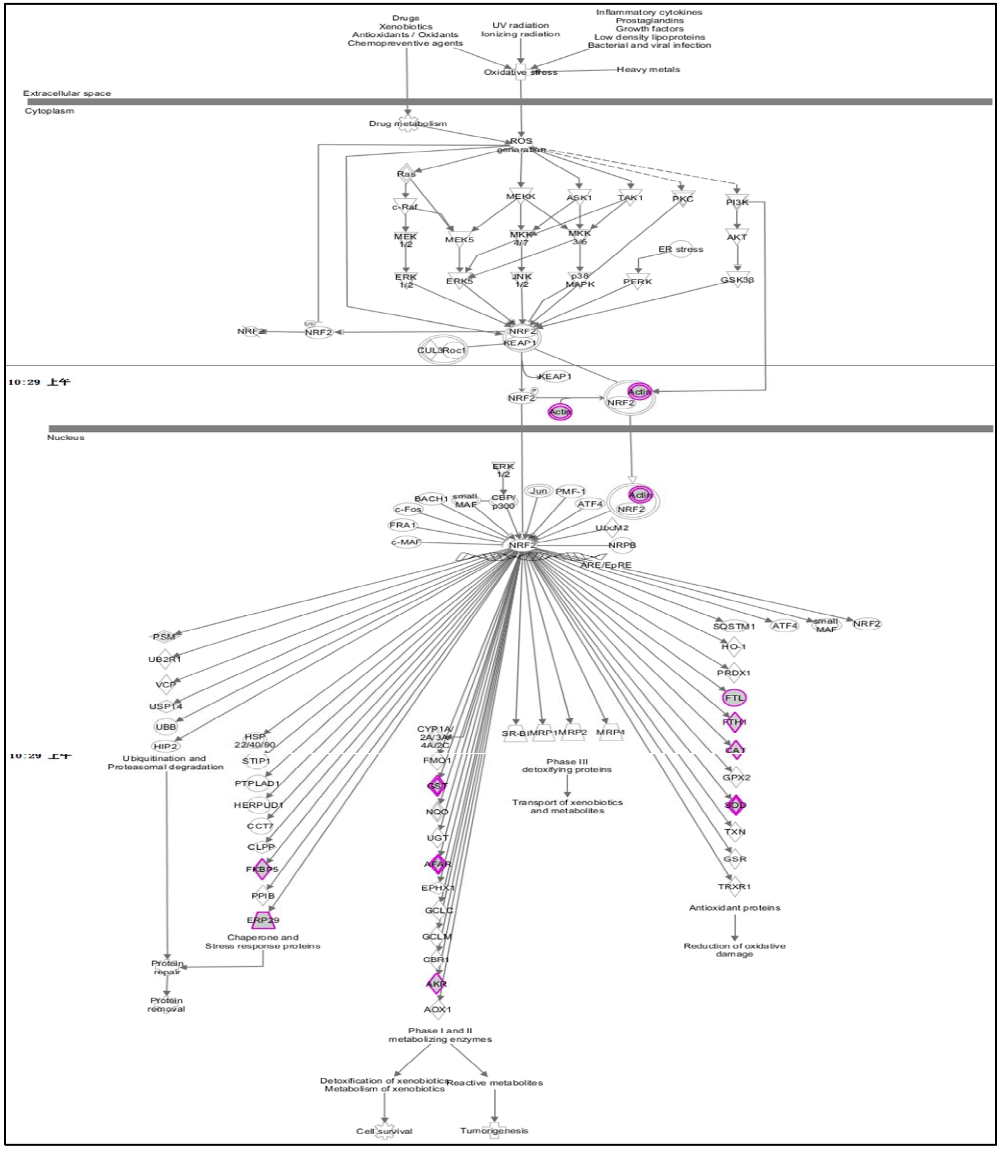

10-Acute Phase Response Signaling

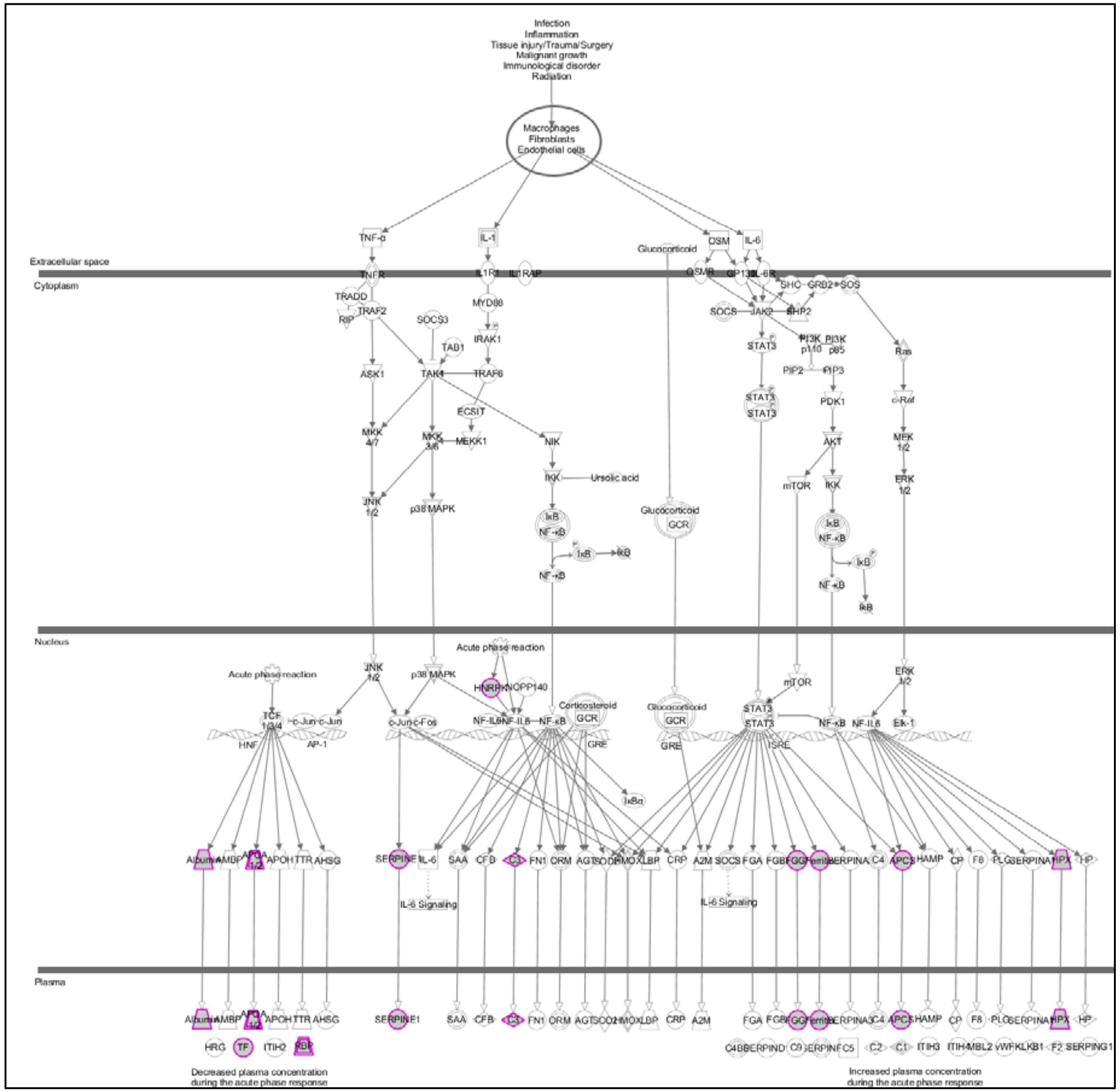

11-Huntington's Disease Signaling

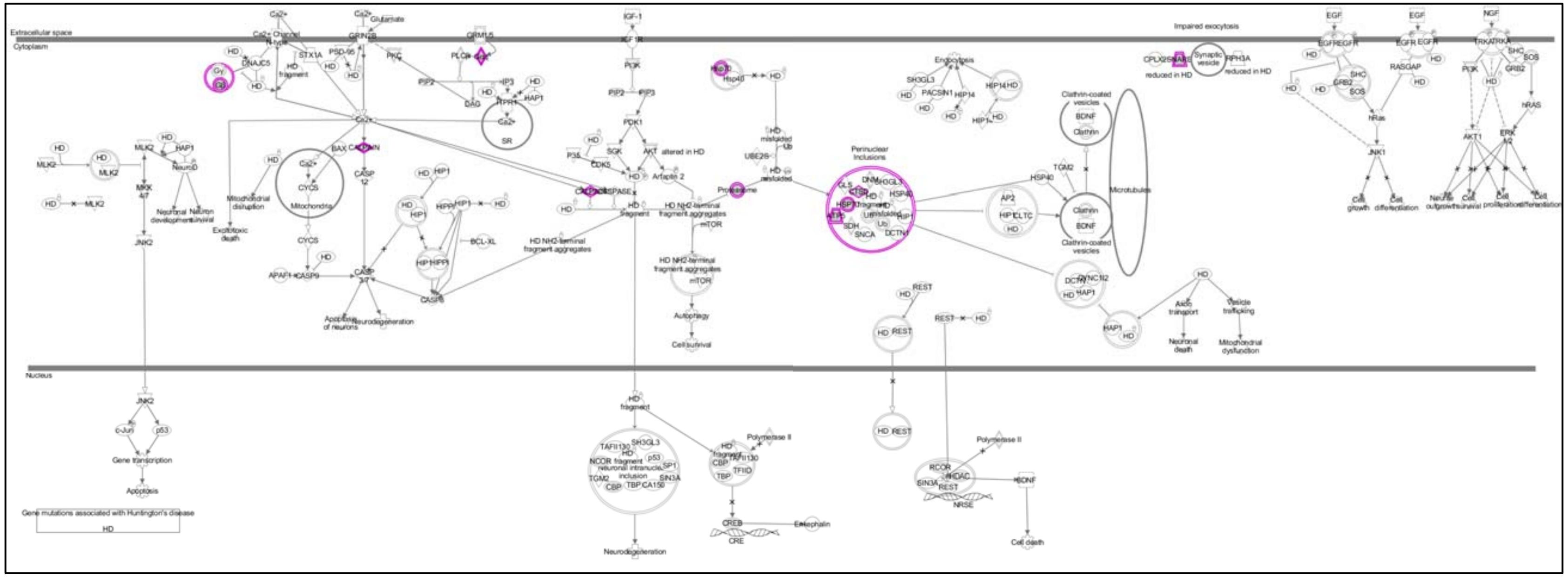

12-LXR-RXR Activation

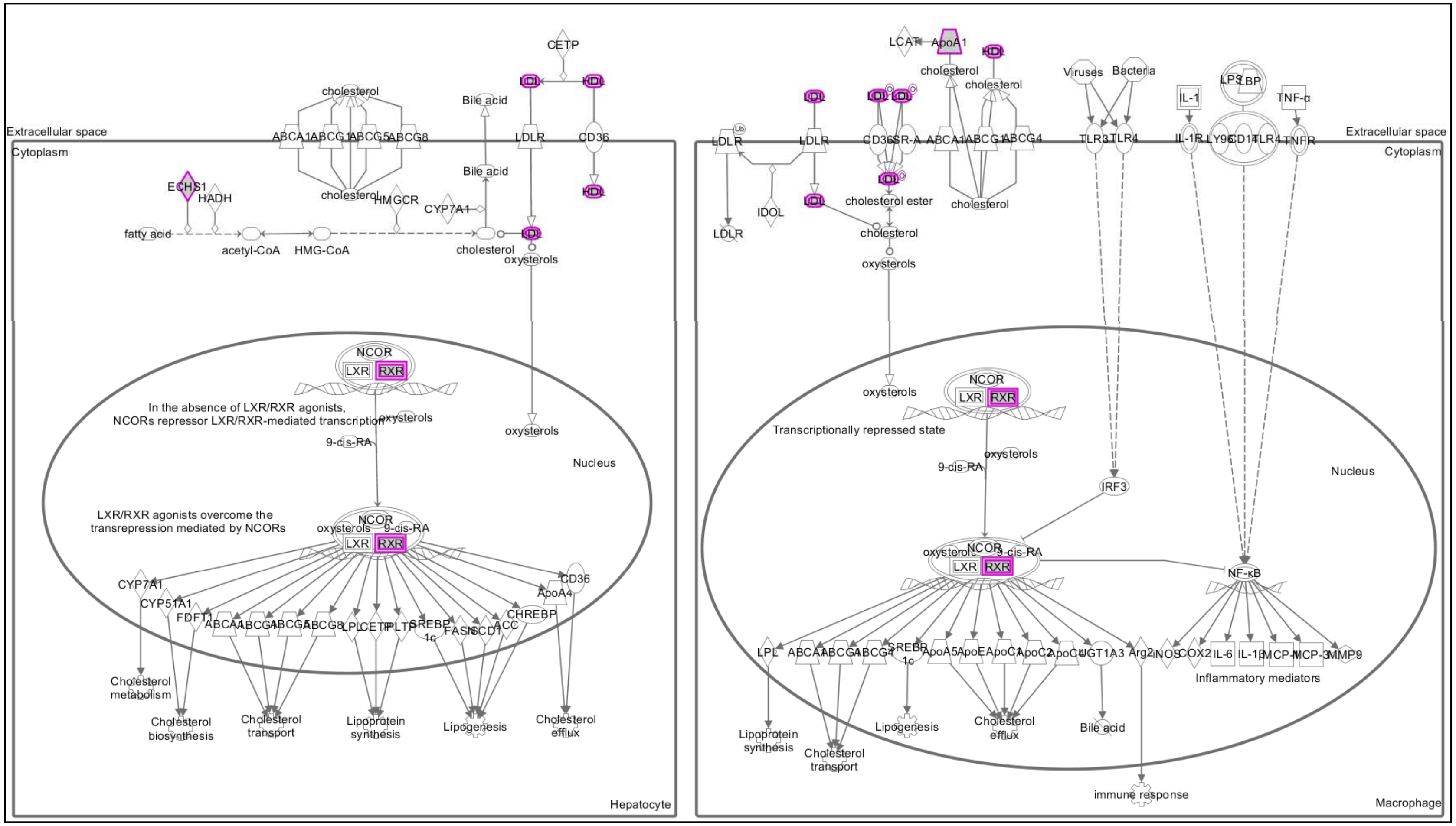

13-RhoGDI Signaling

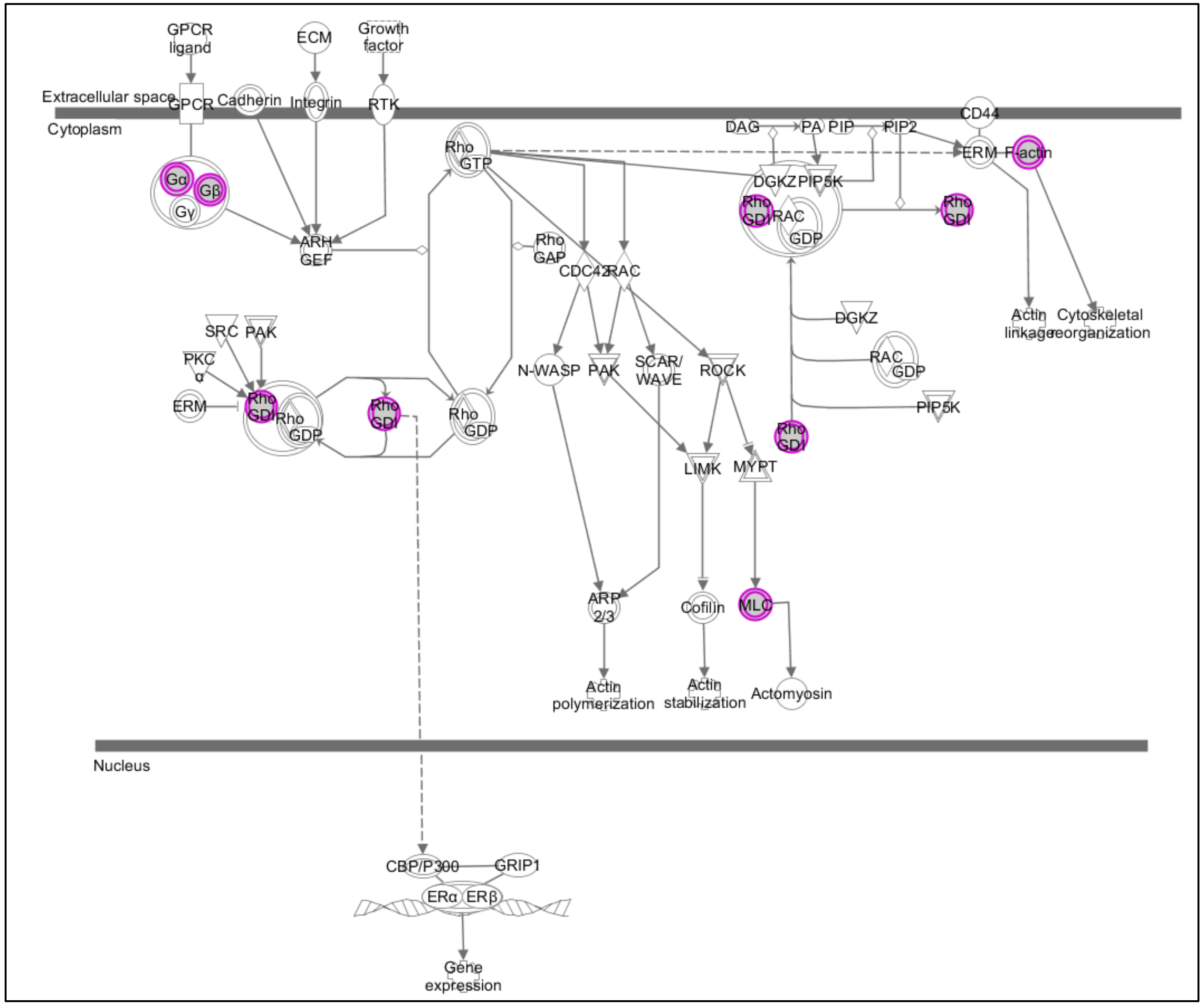

14-Mitochondrial Dysfunction

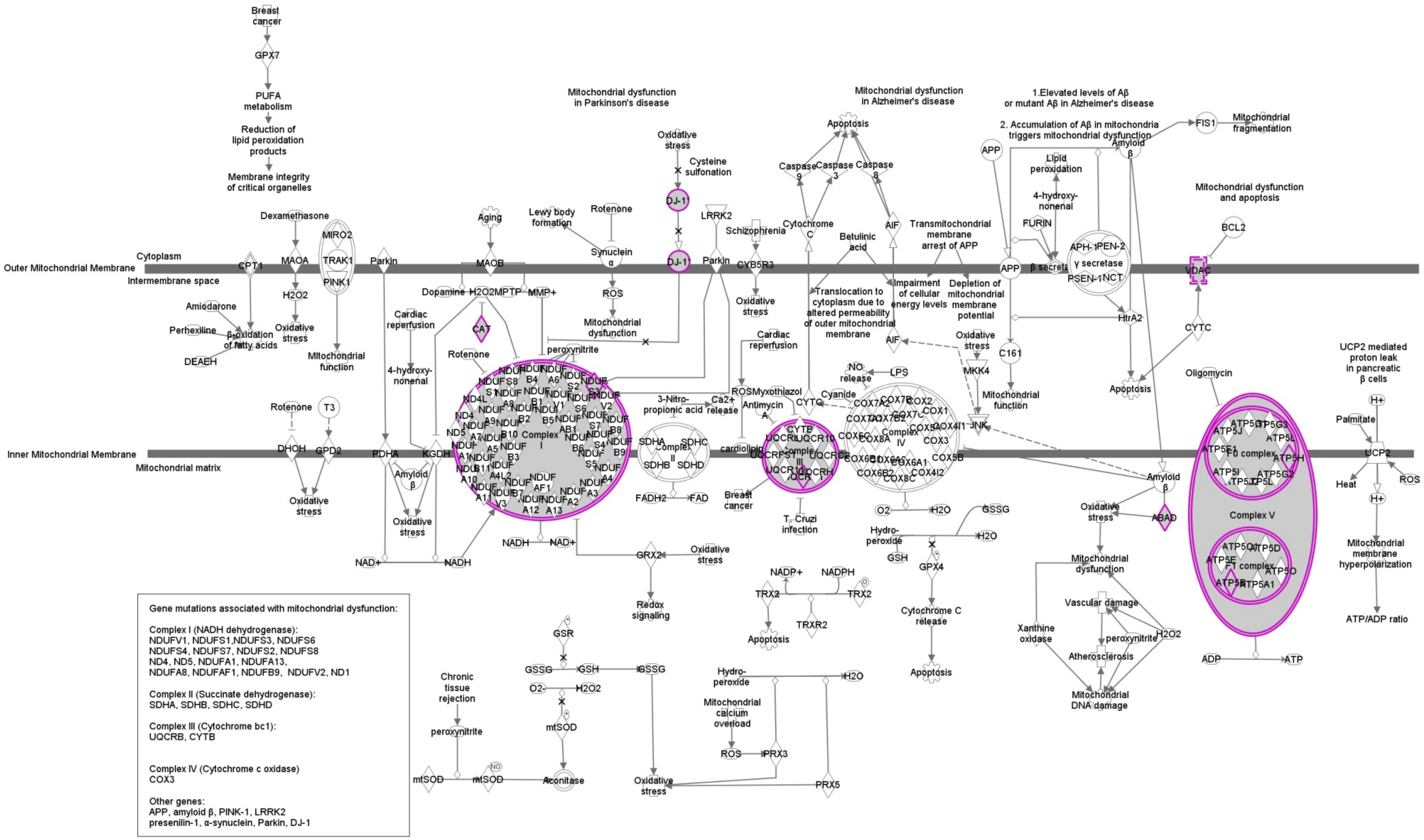

Gene mutations associated with mitochondrial dysfunction:

Complex I (NADH dehydrogenase):
NDUFV1, NDUFS1,NDUFS3, NDUFS6
NDUFS4, NDUFS7, NDUFS2, NDUFS8
ND4, ND5, NDUFA1, NDUFA13,
NDUFA8, NDUFAF1, NDUFB9, NDUFV2, ND1

Complex II (Succinate dehydrogenase):
SDHA, SDHB, SDHC, SDHD

Complex III (Cytochrome bc1):
UQCRB, CYTB

Complex IV (Cytochrome c oxidase)
COX3

Other genes:
APP, amyloid β, PINK-1, LRRK2
presenilin-1, α-synuclein, Parkin, DJ-1

15-Gluconeogenesis I

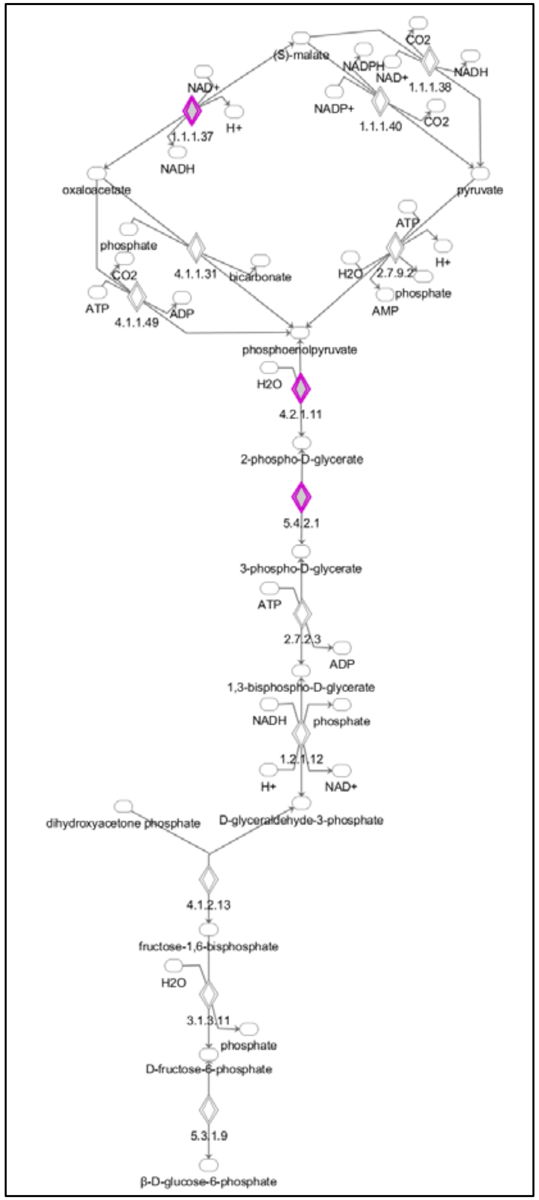

16-Protein Ubiquitination Pathway

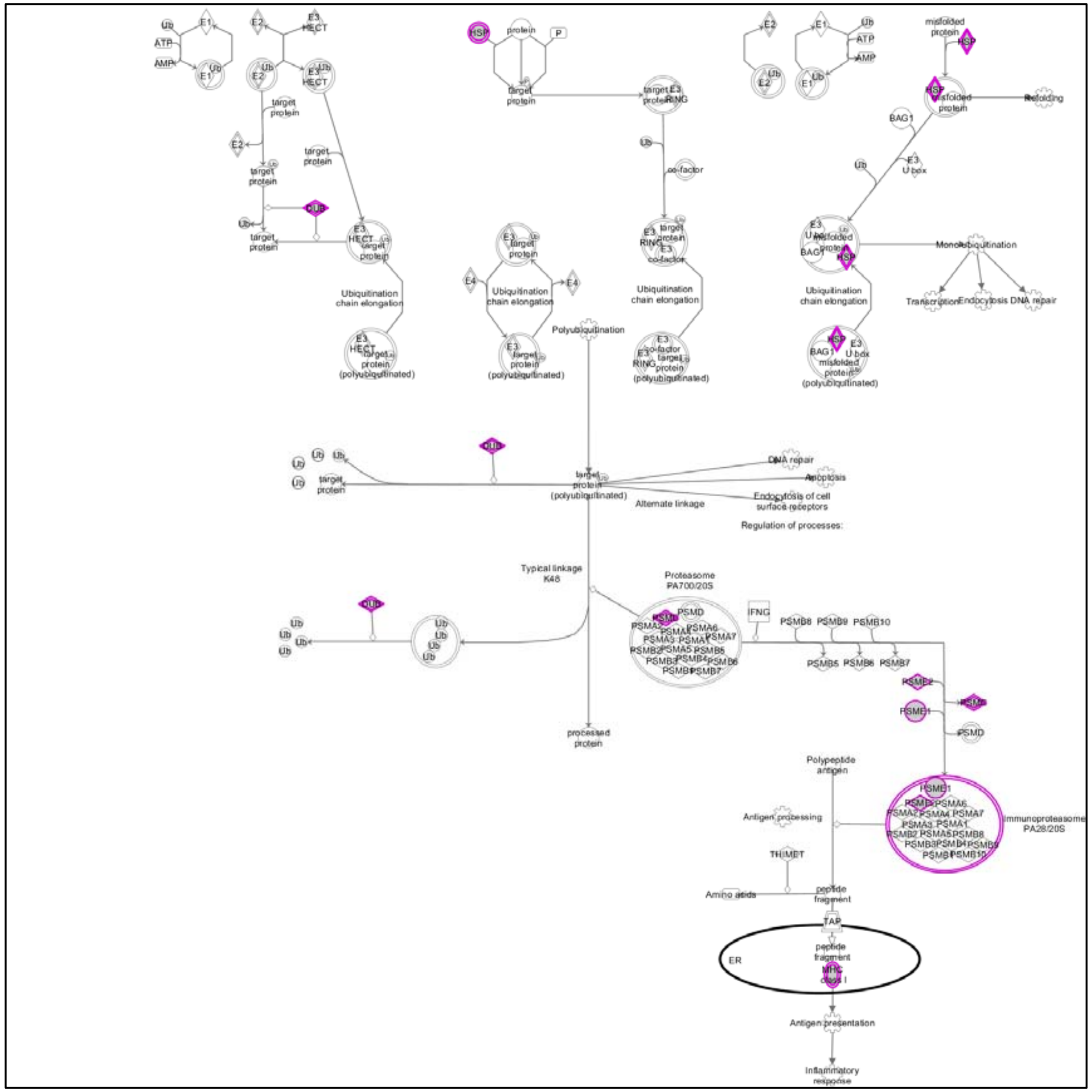

17-Unfolded Protein Response

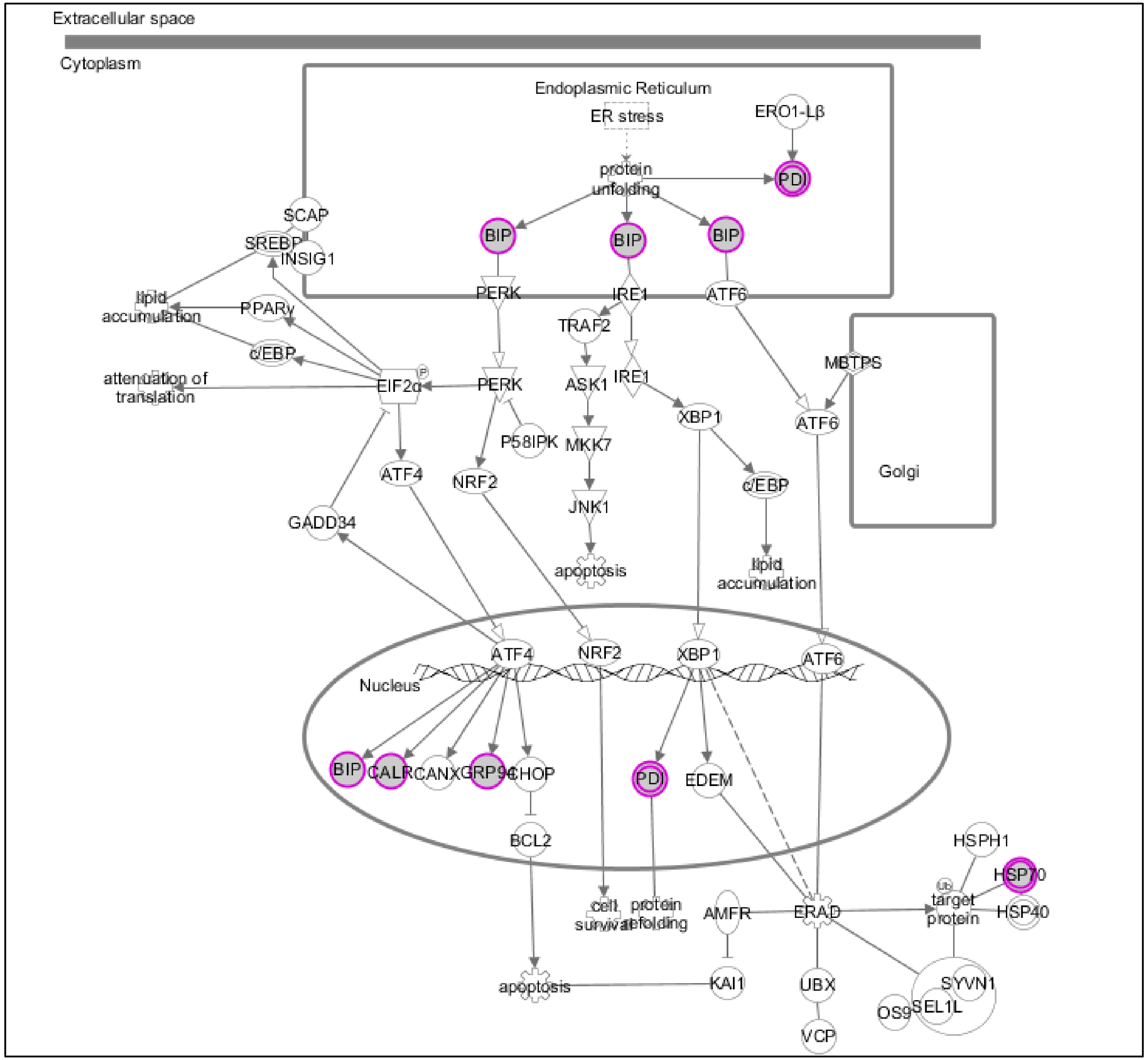

18-Antigen Presentation Pathway

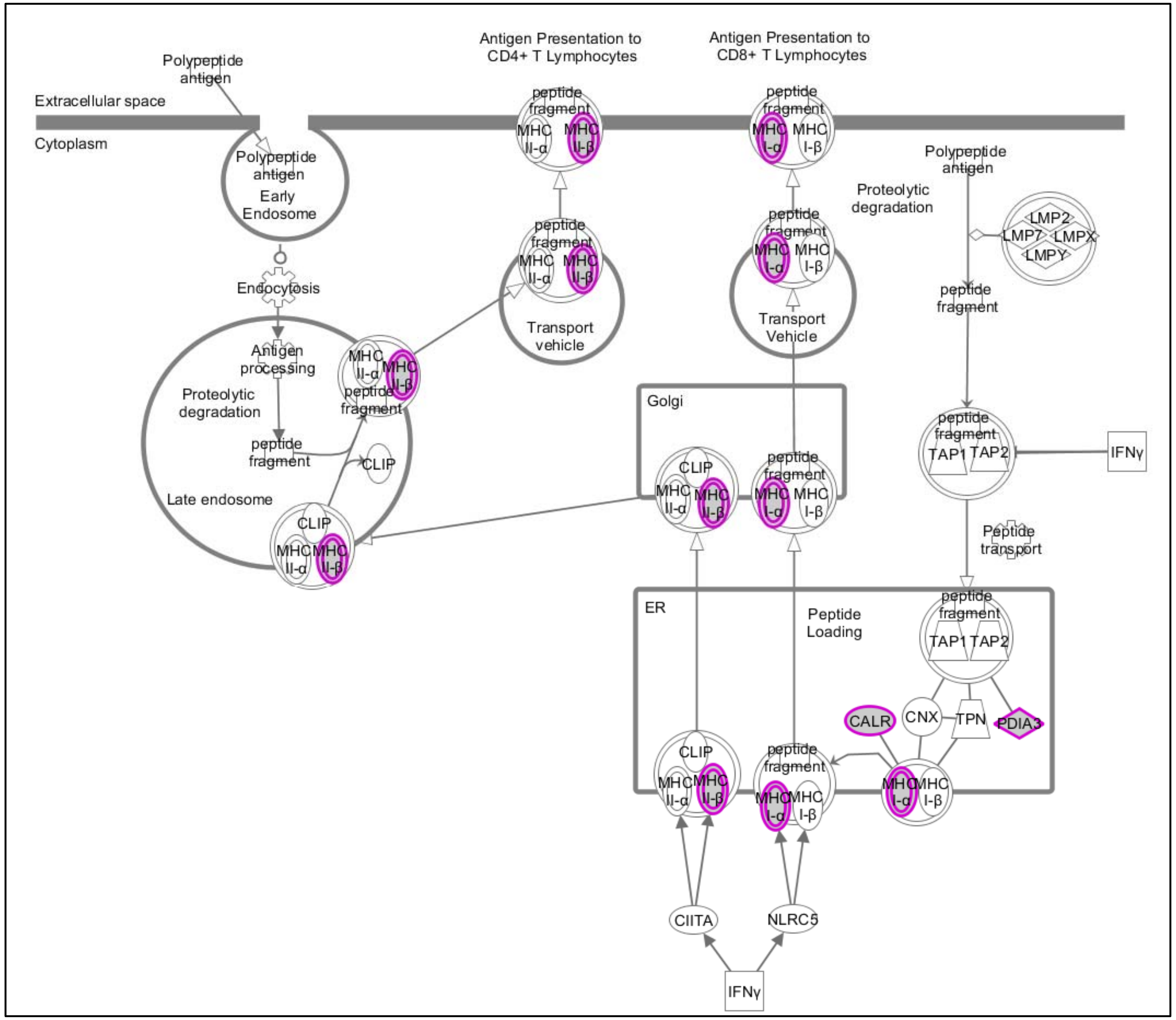

19-Aryl Hydrocarbon Resceptor Signaling

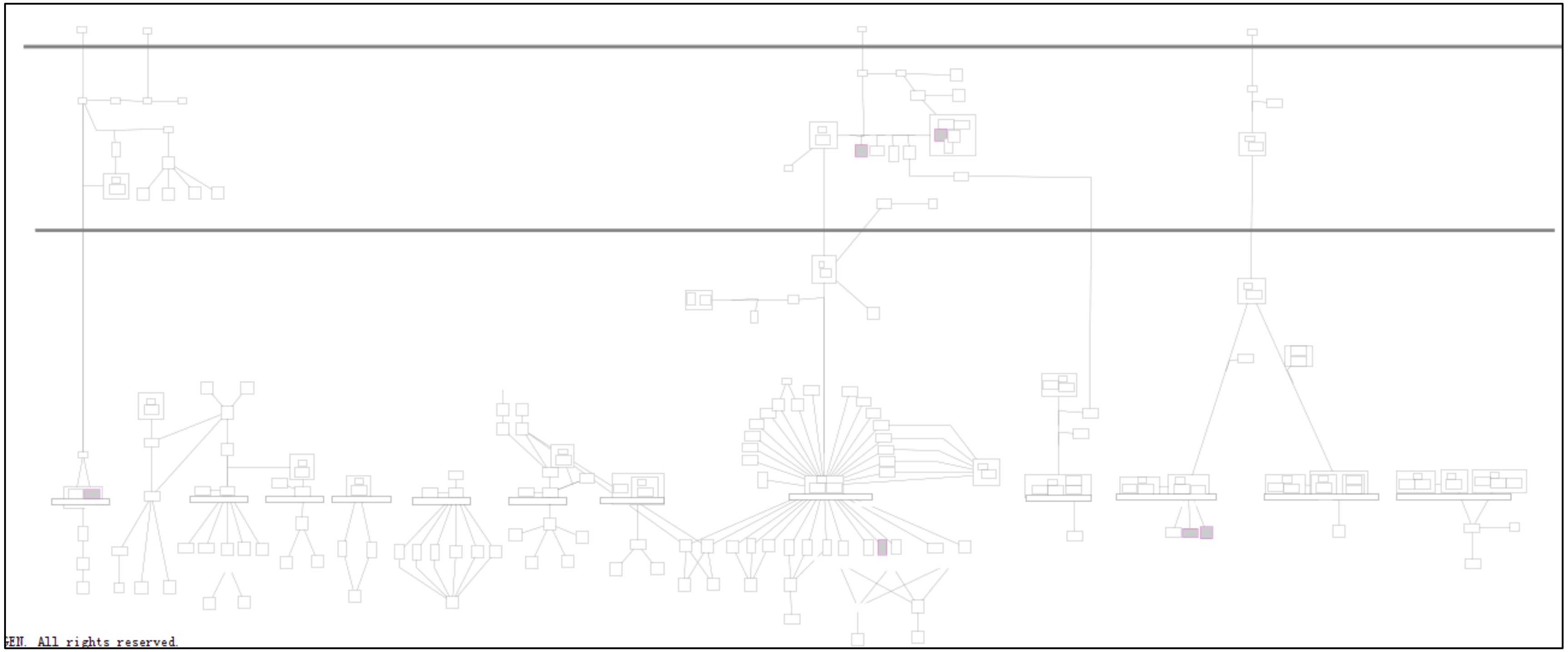

20-TCA Cycle II（Eukaryotic）

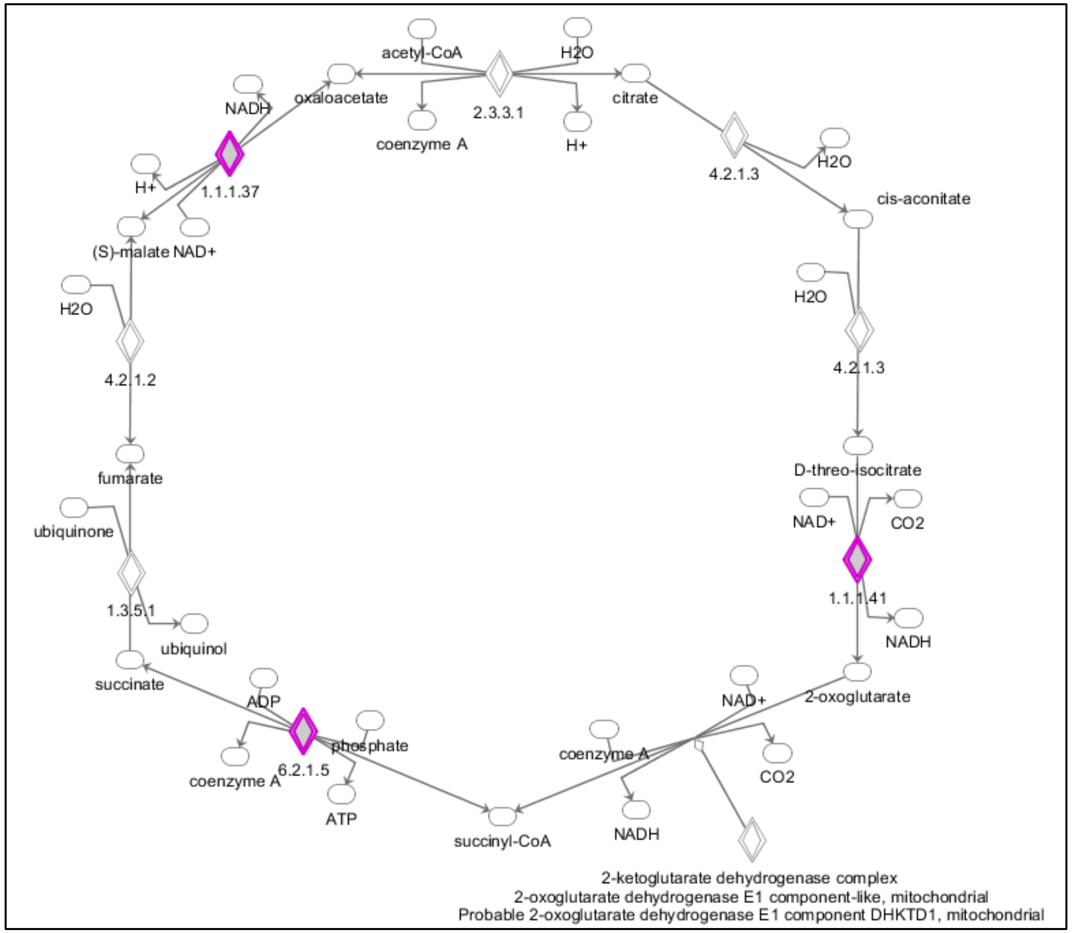

21-Glycolysis Ⅰ

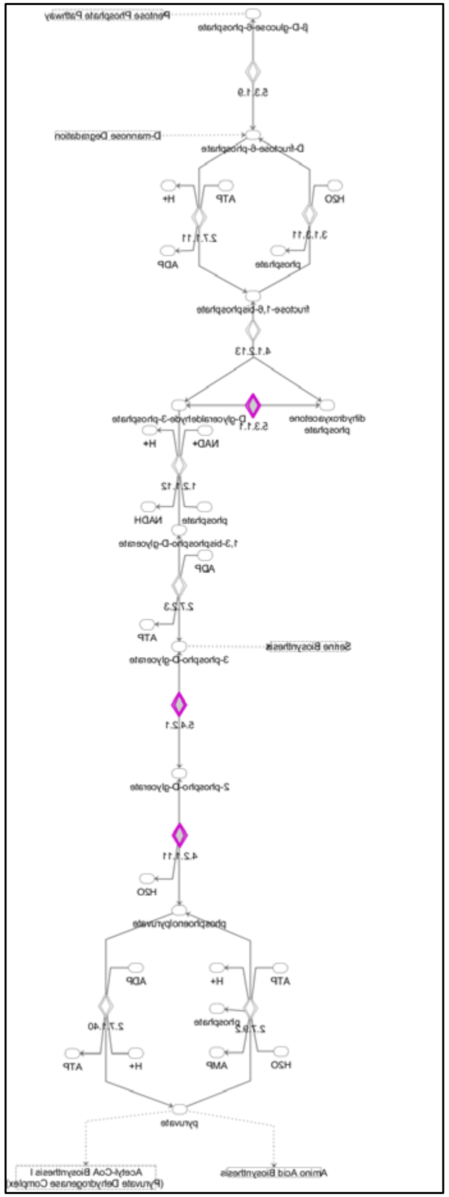

22-Caveolar-mediated Endocytosis Signaling

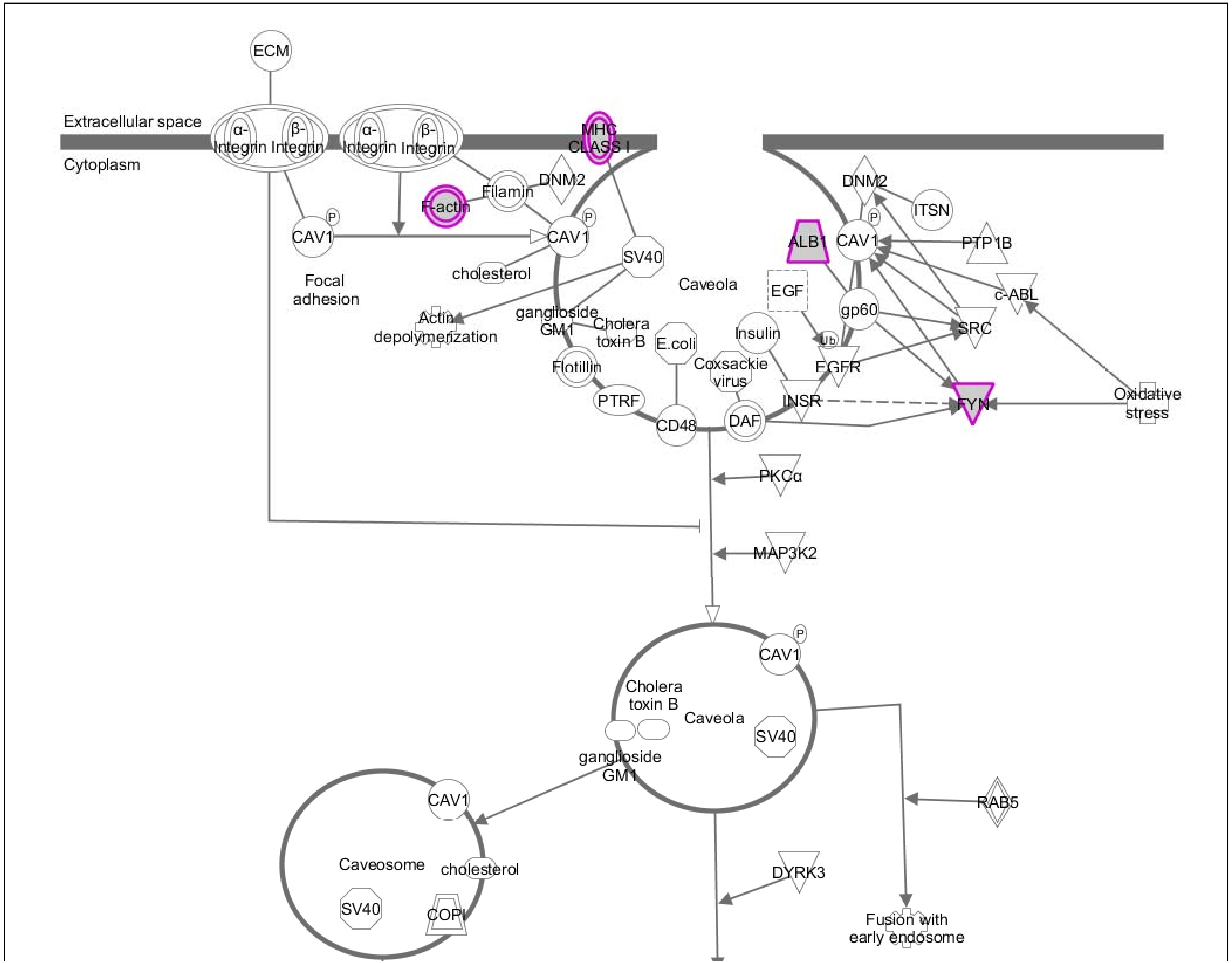

23-Androgen Signaling

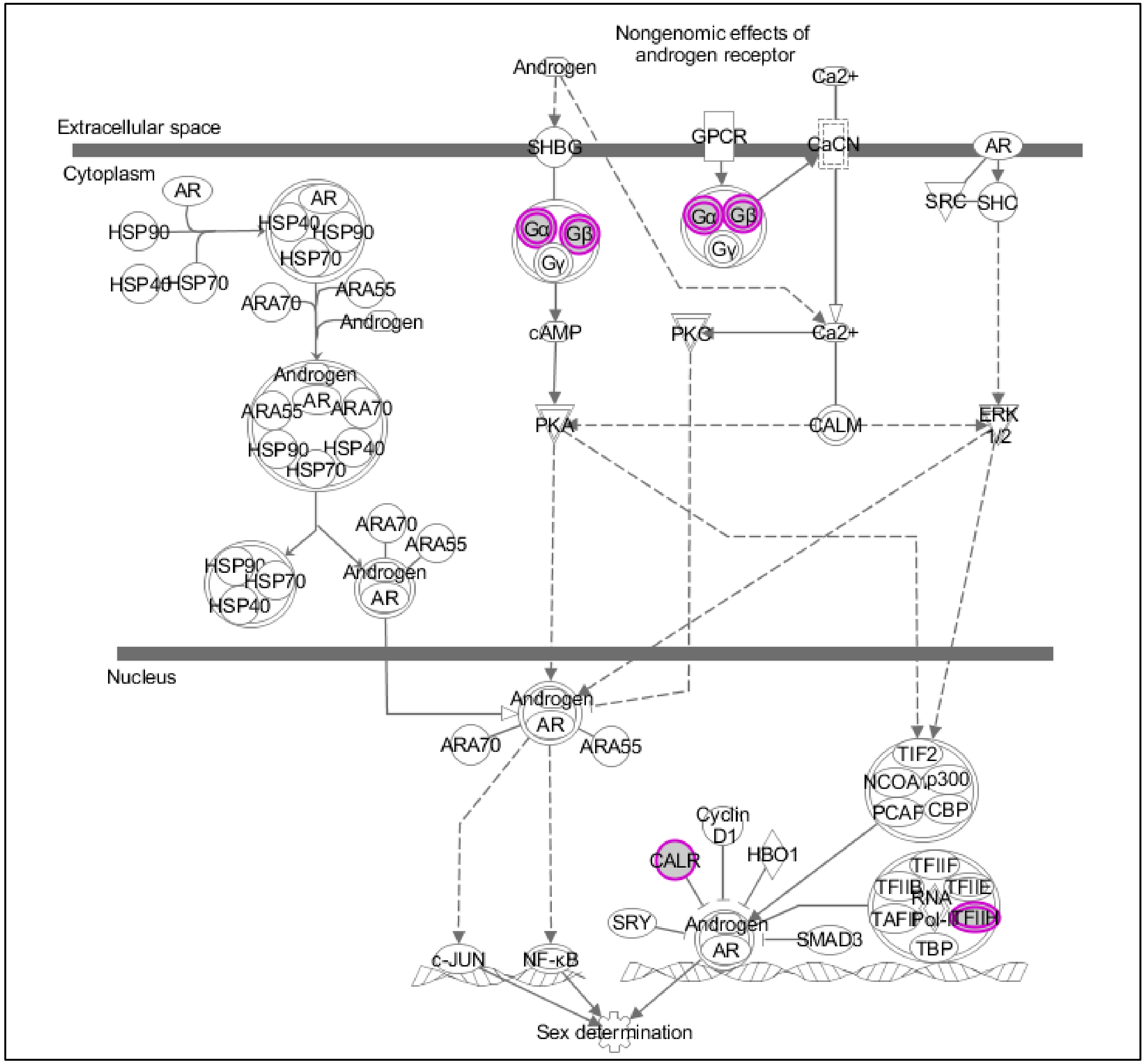

24-Ephrin B Signaling

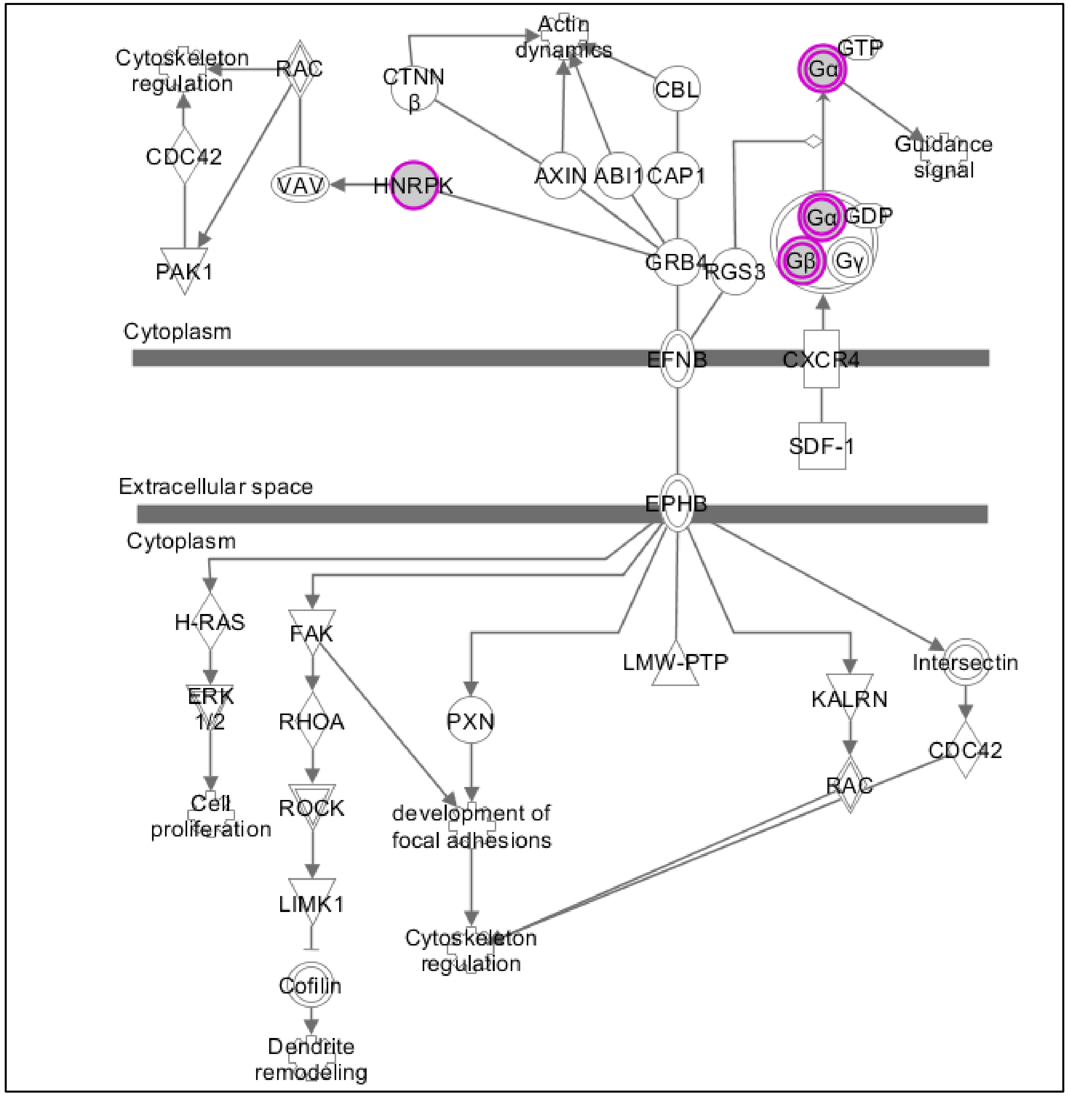

25-Tec Kinase Signaling

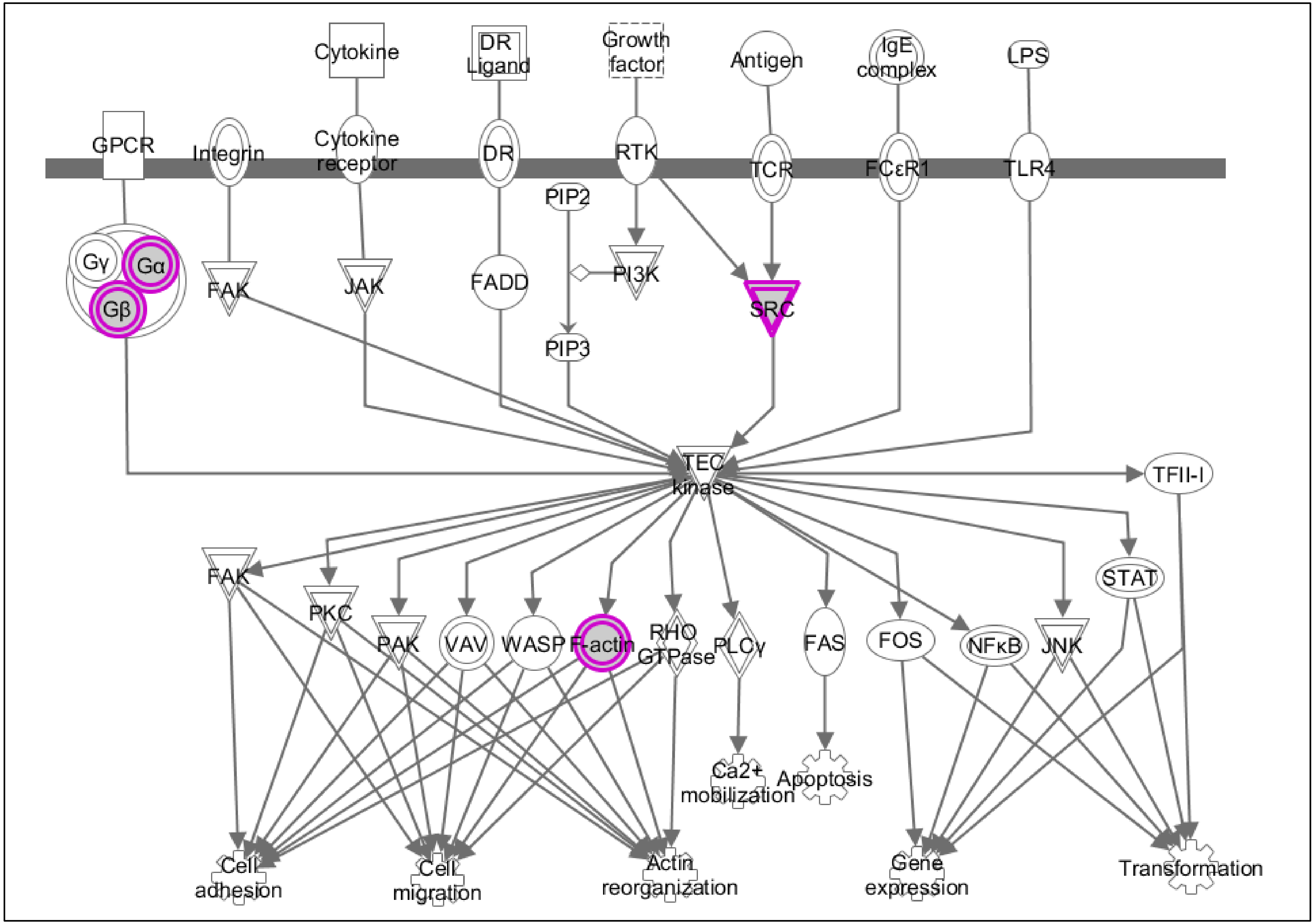

26-Aldosterone Signaling in Epithelial Cells

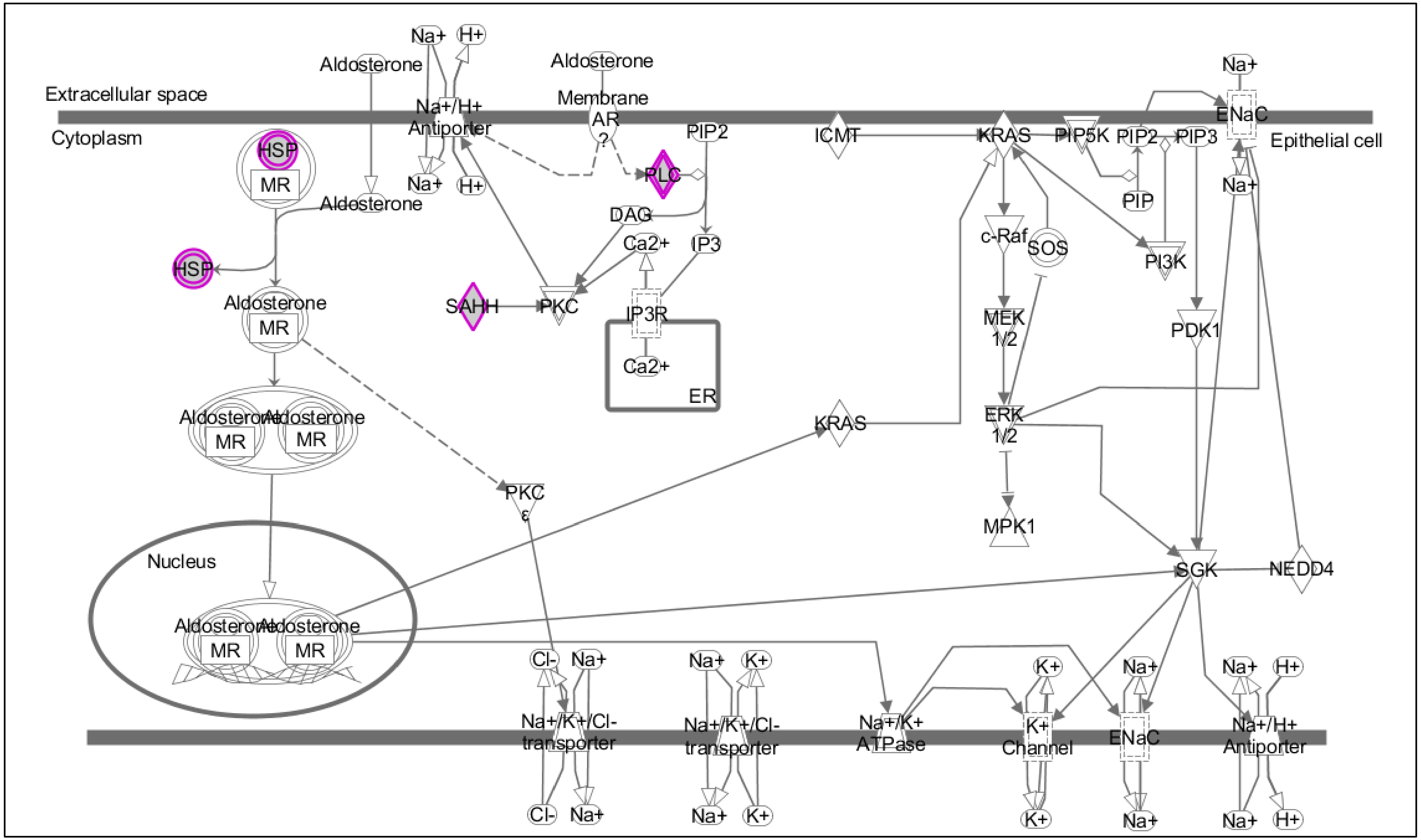

27-Tight Junction Signaling

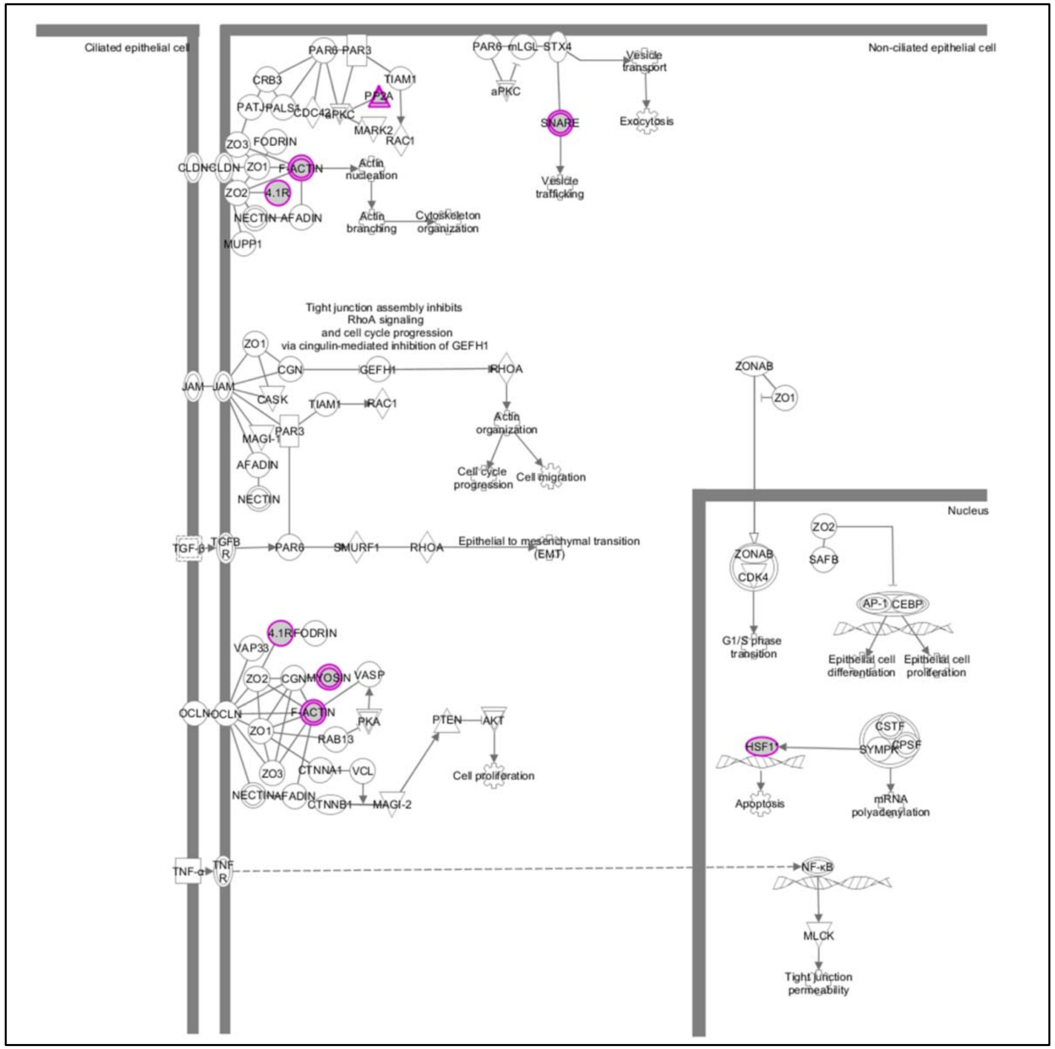

28-Endoplasmic Reticulum Stress Pathway

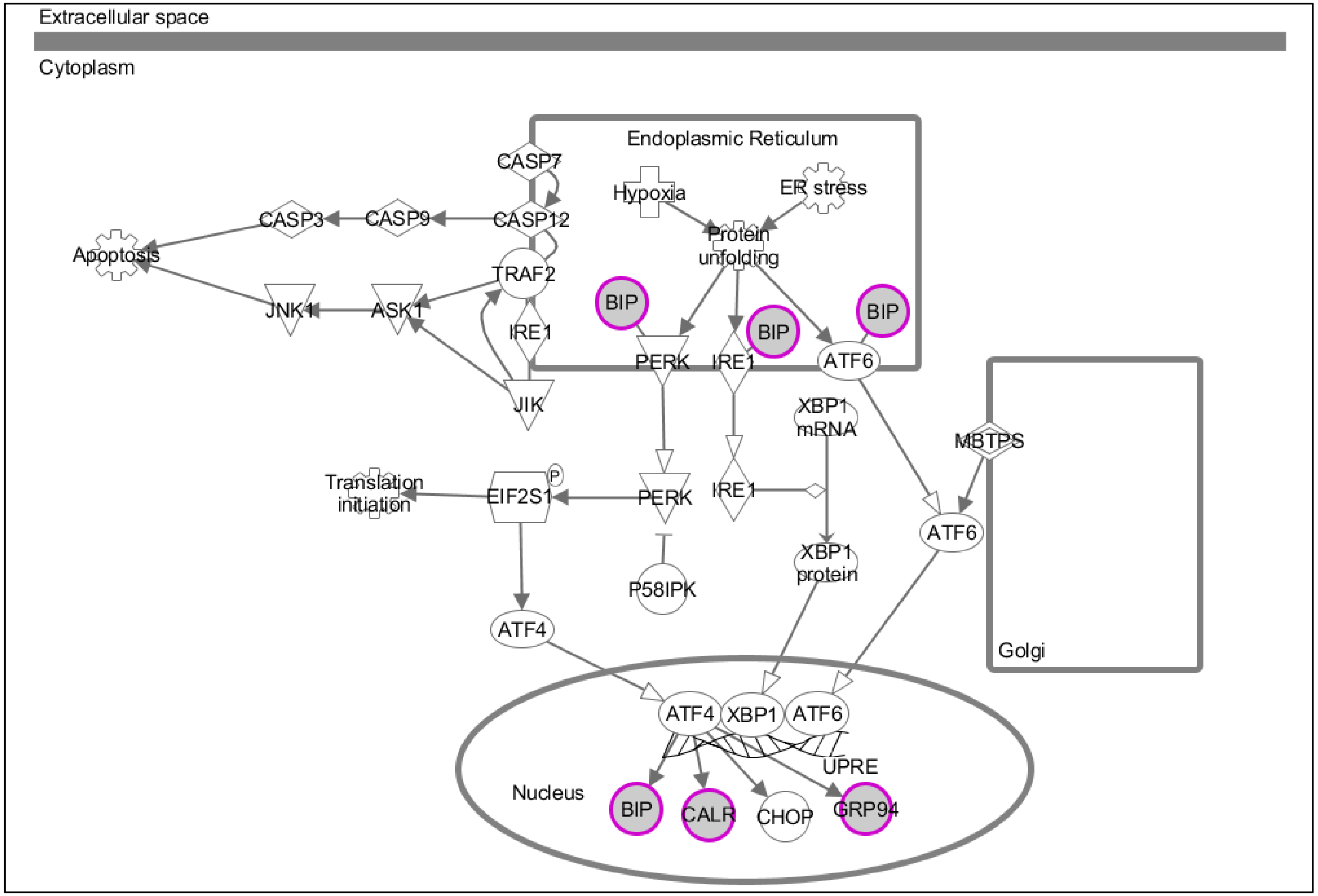

29-Methylglyoxal Degradation III

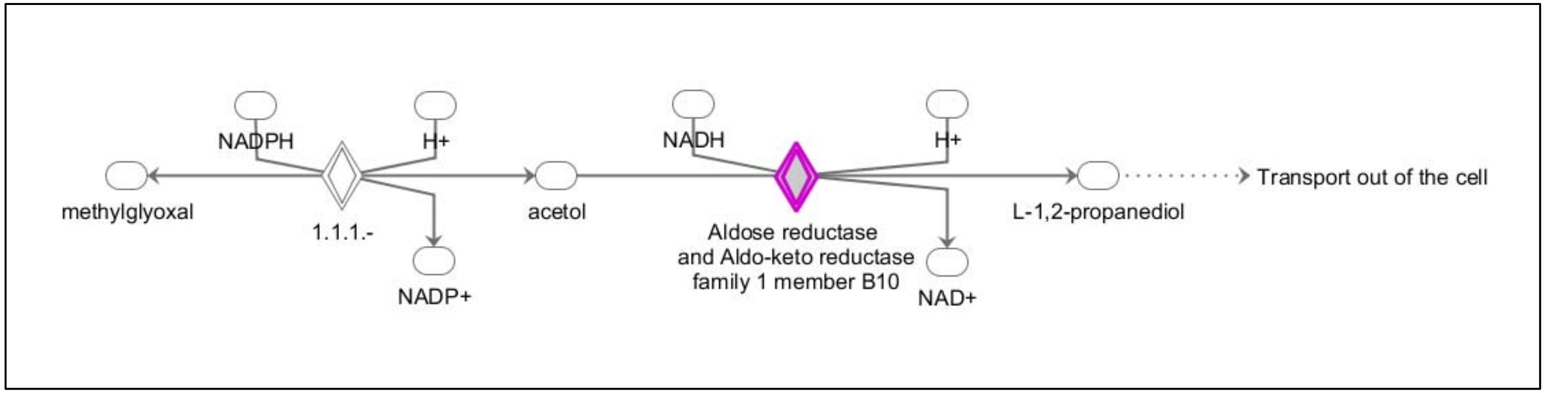

30-Cardiac Hypertrophy Signaling

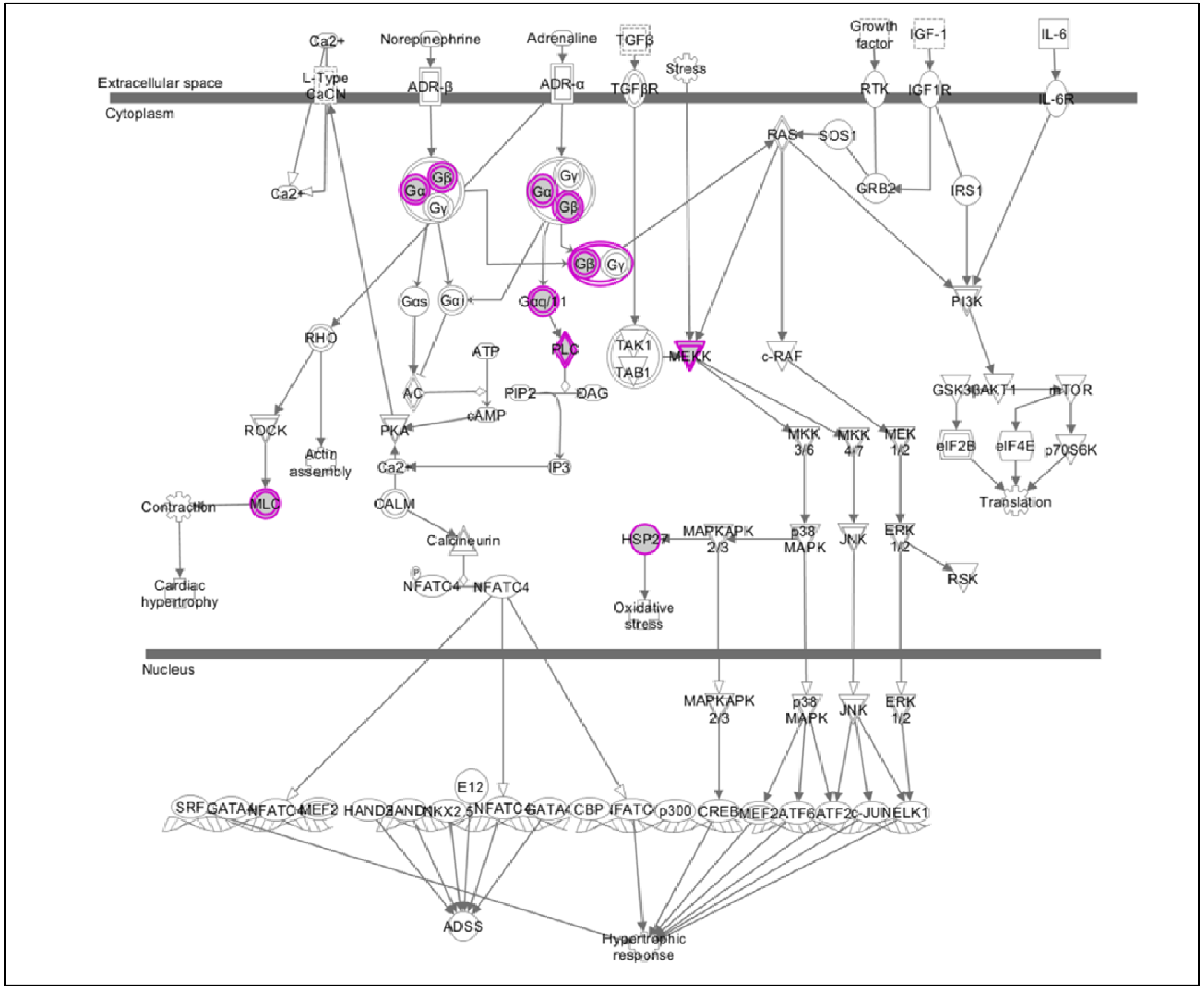

31-Death Receptor Signaling

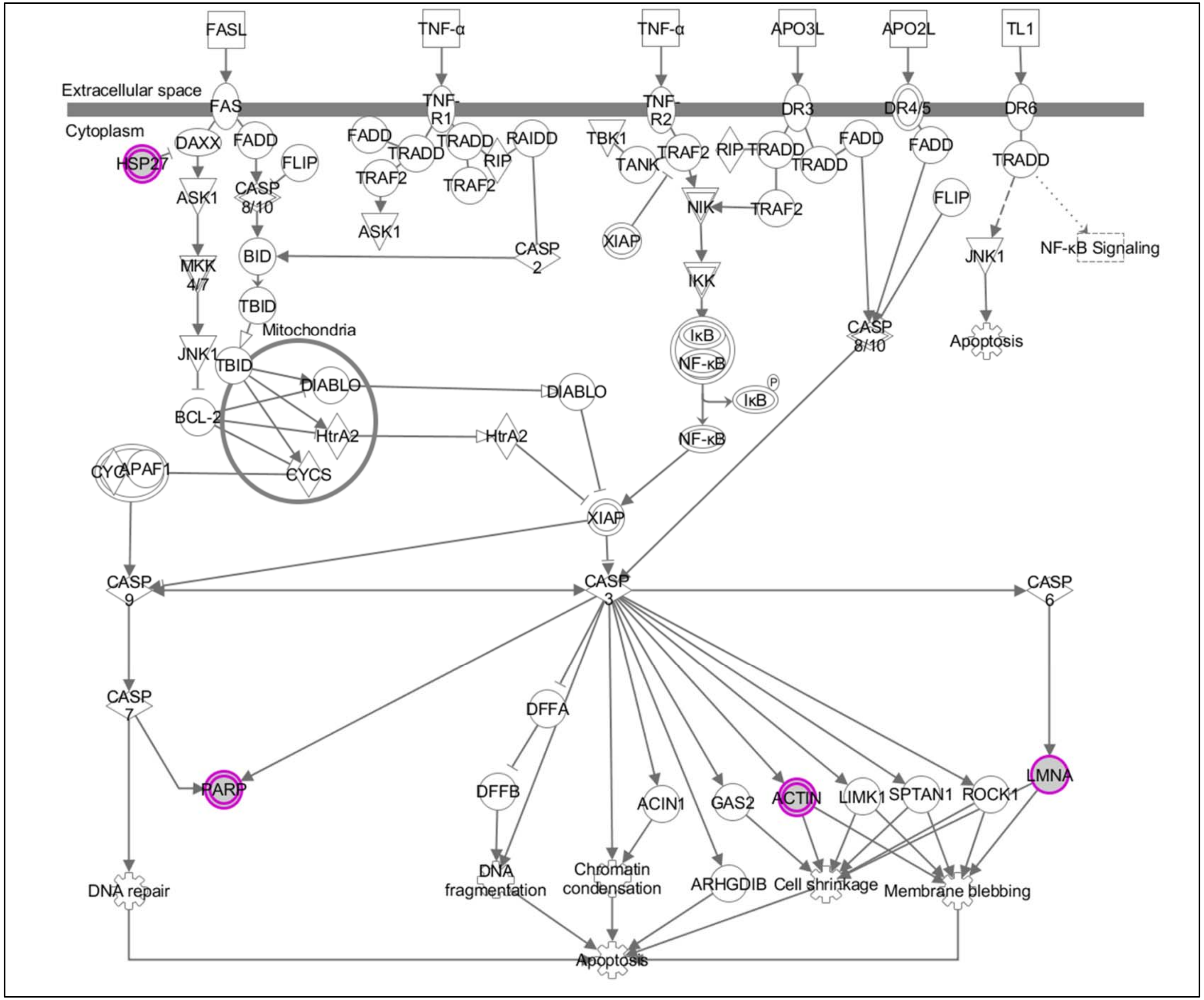

32-FXR/RXR Activation

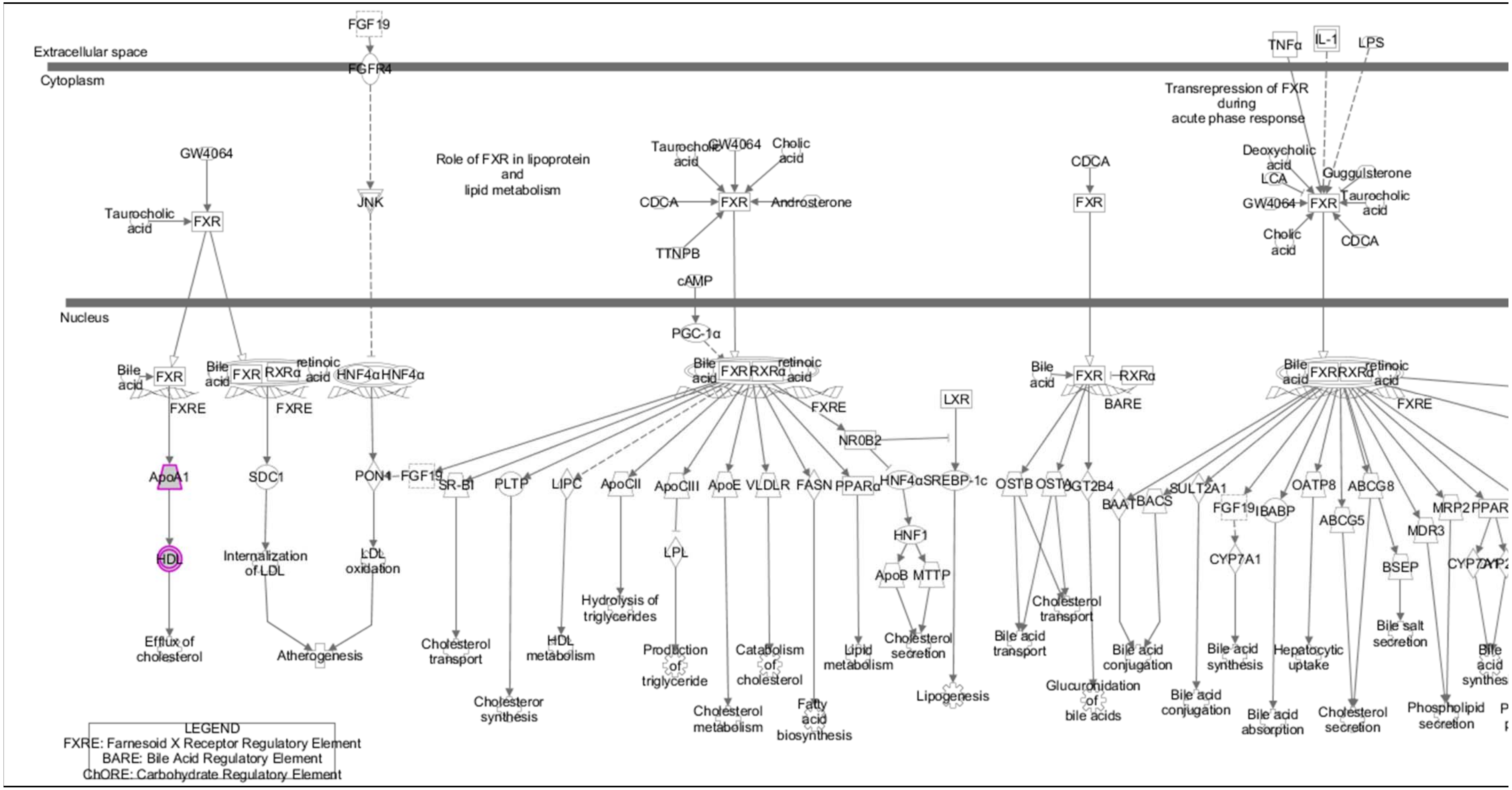

33-Isoleucne Degradation Ⅰ

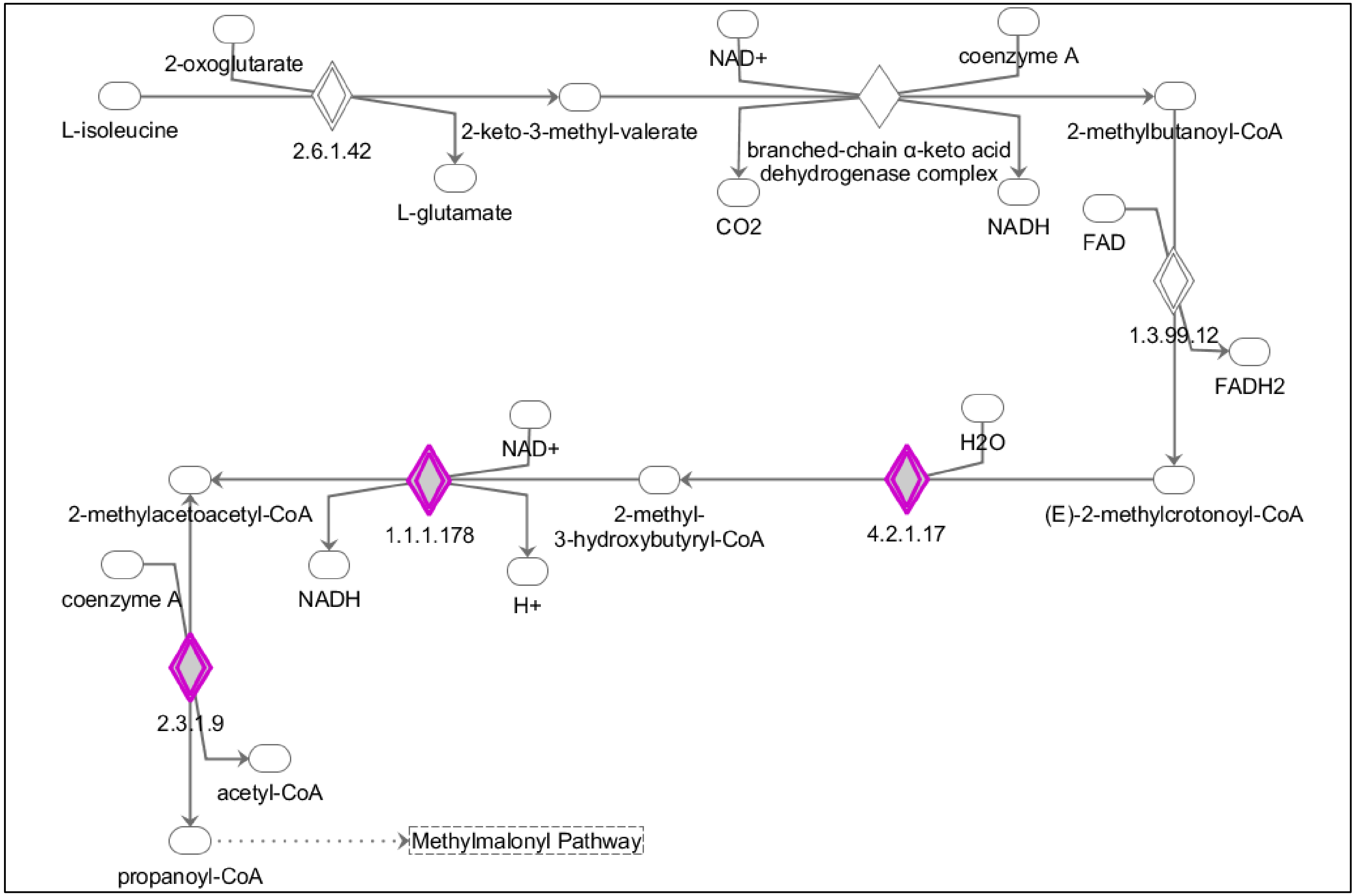

34-Superoxide Radicals Degradation

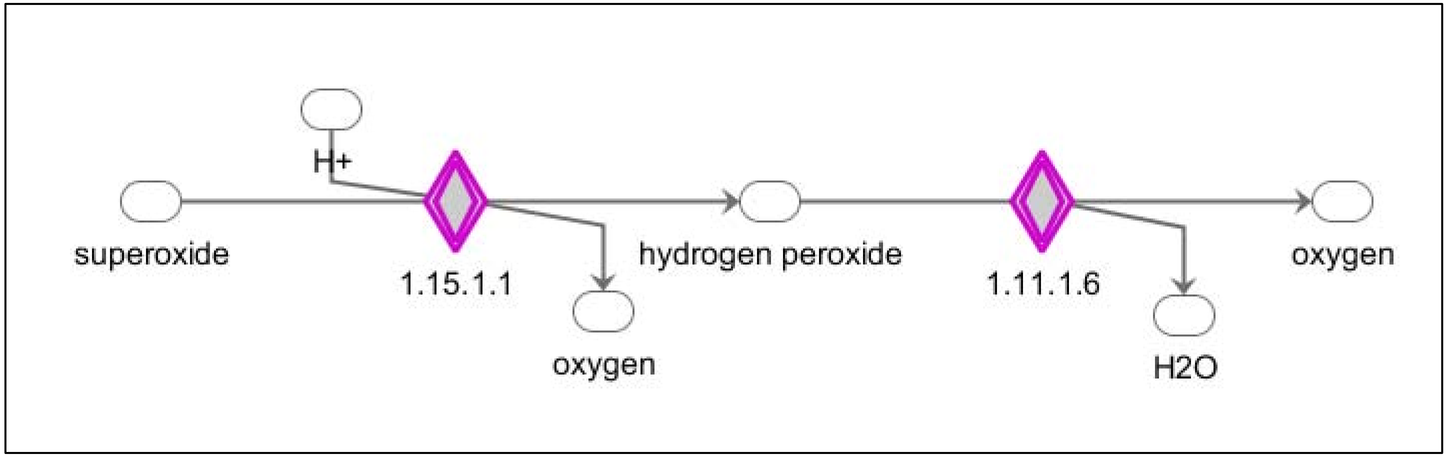

35-ERK5 Signaling

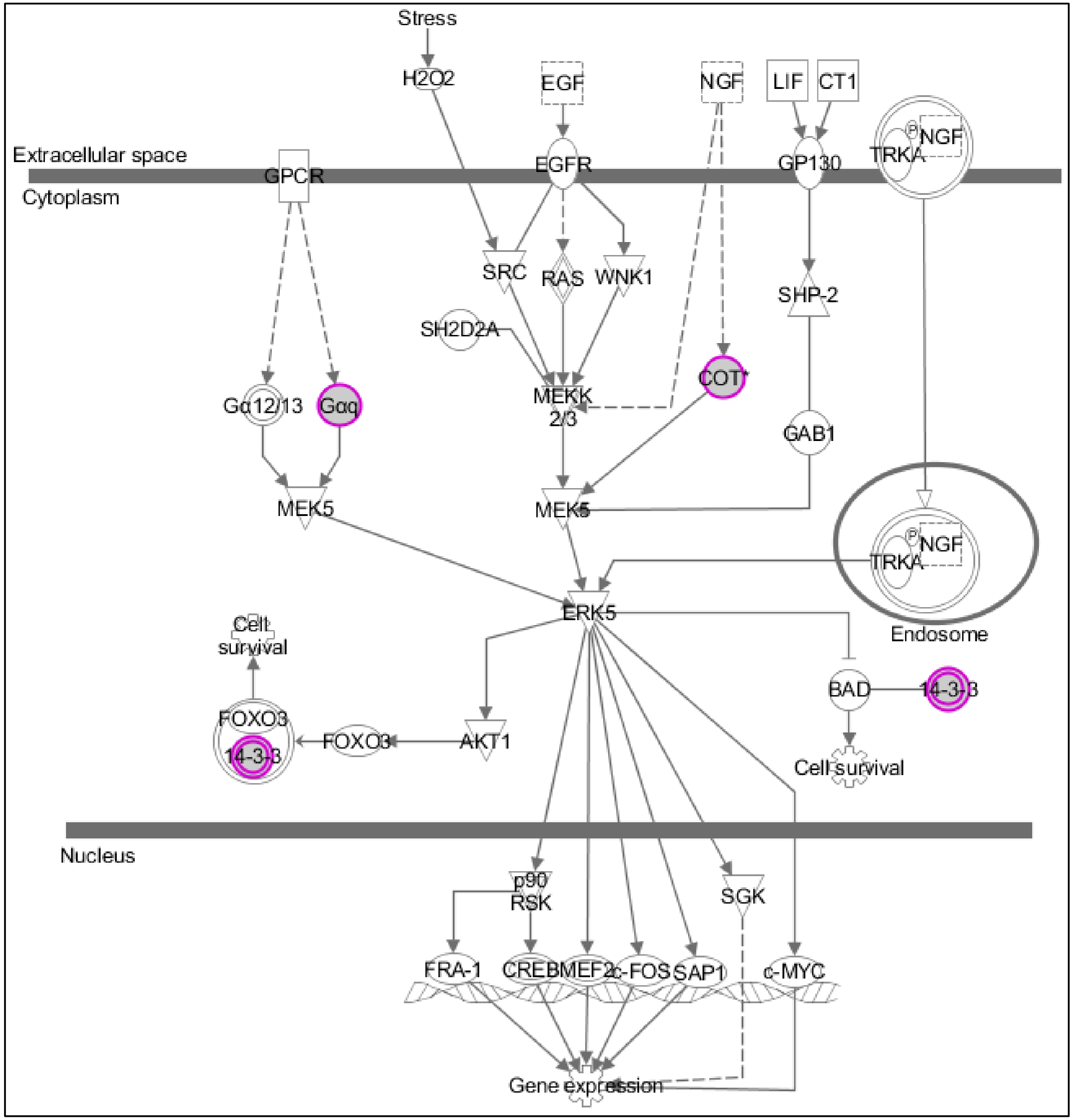

36-Glucocorticoid Receptor Signaling

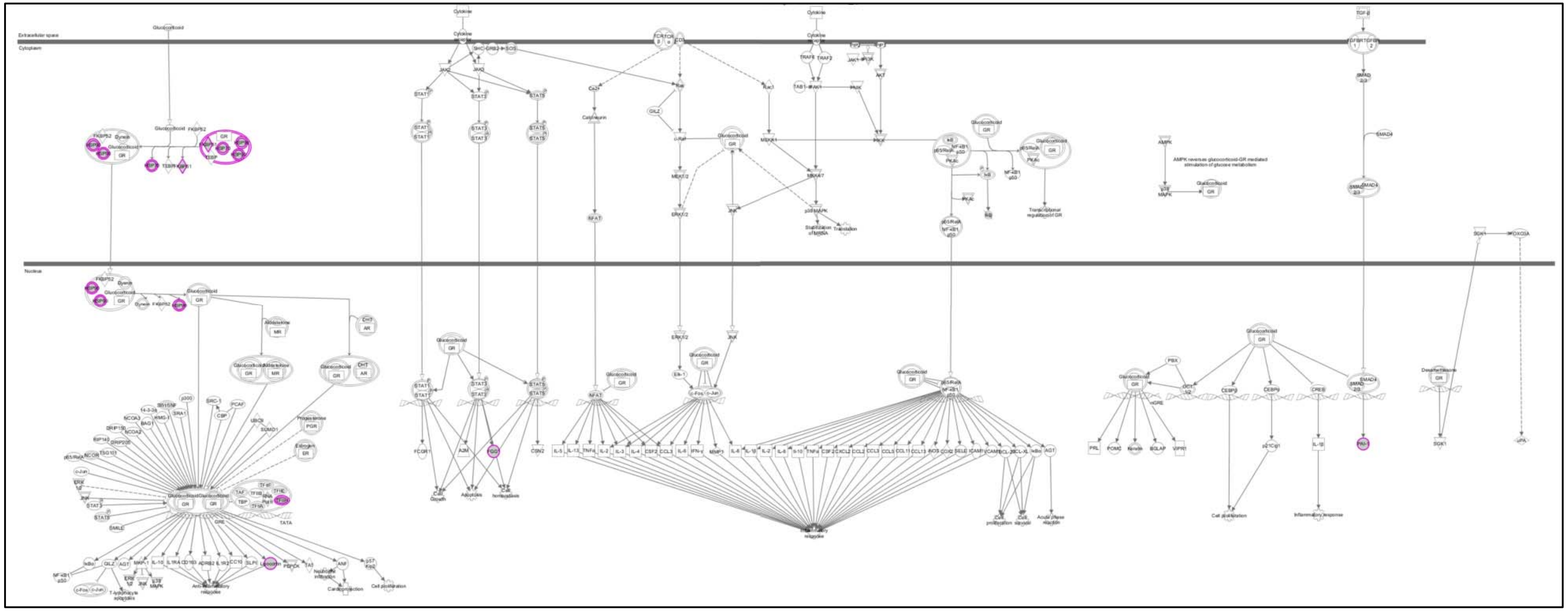

37-Xenobiotic Metabolism Signaling

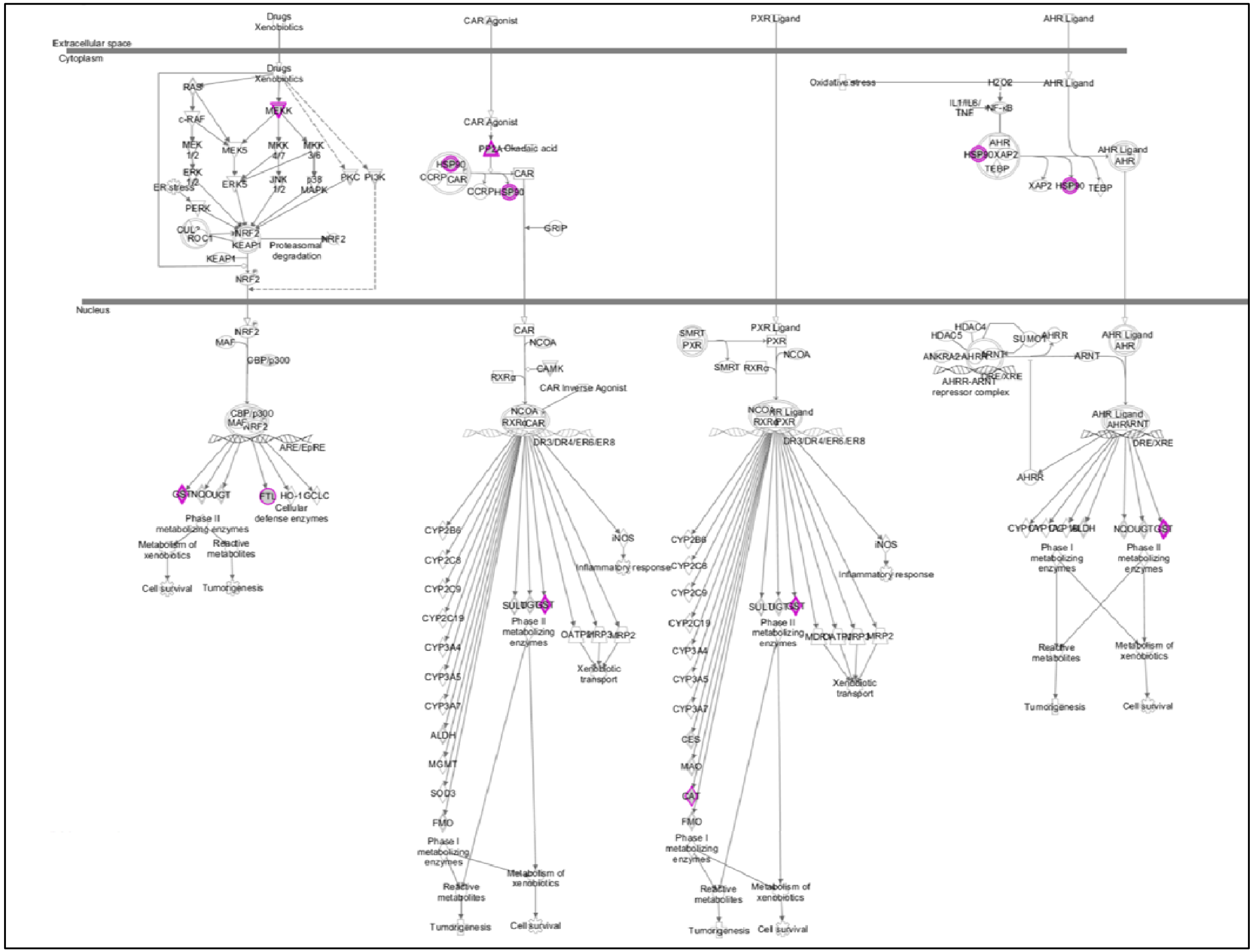

38-Role of Tissue Factor in Cancer

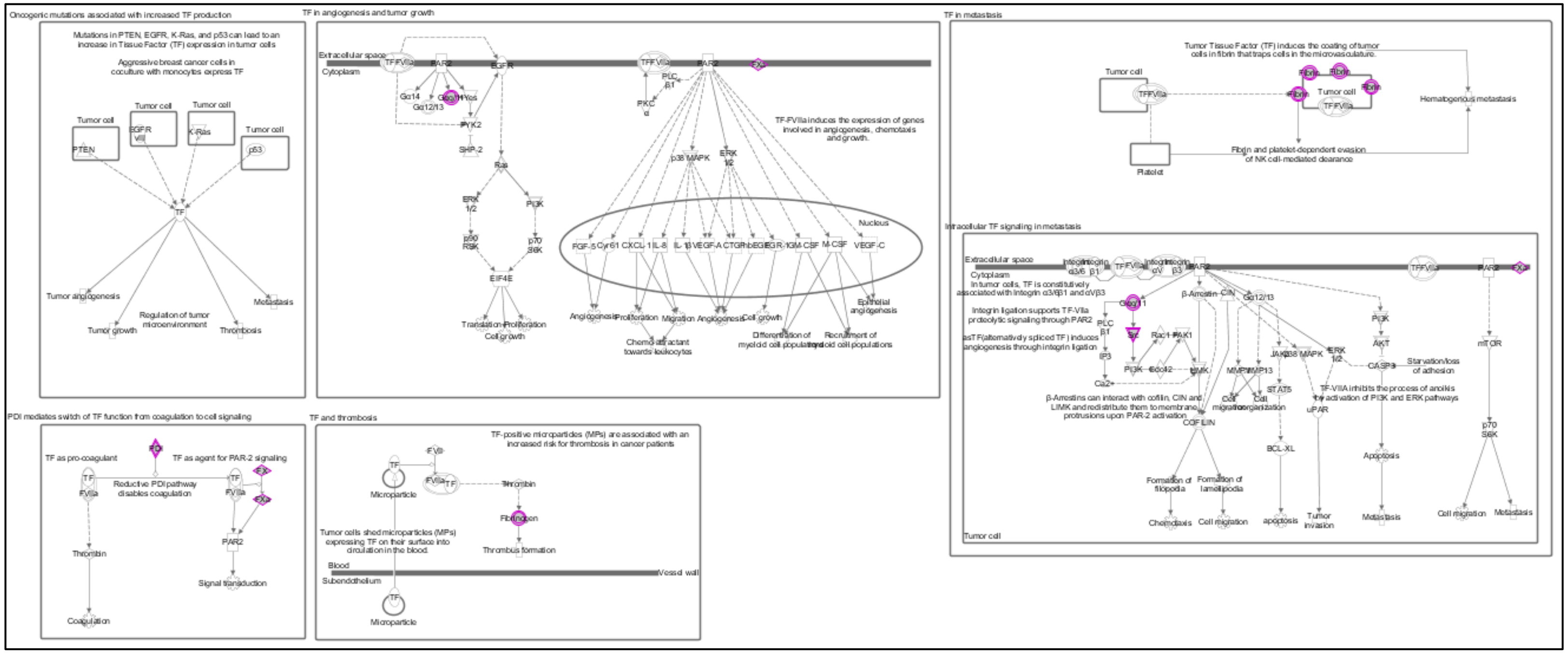

39-Coagulation System

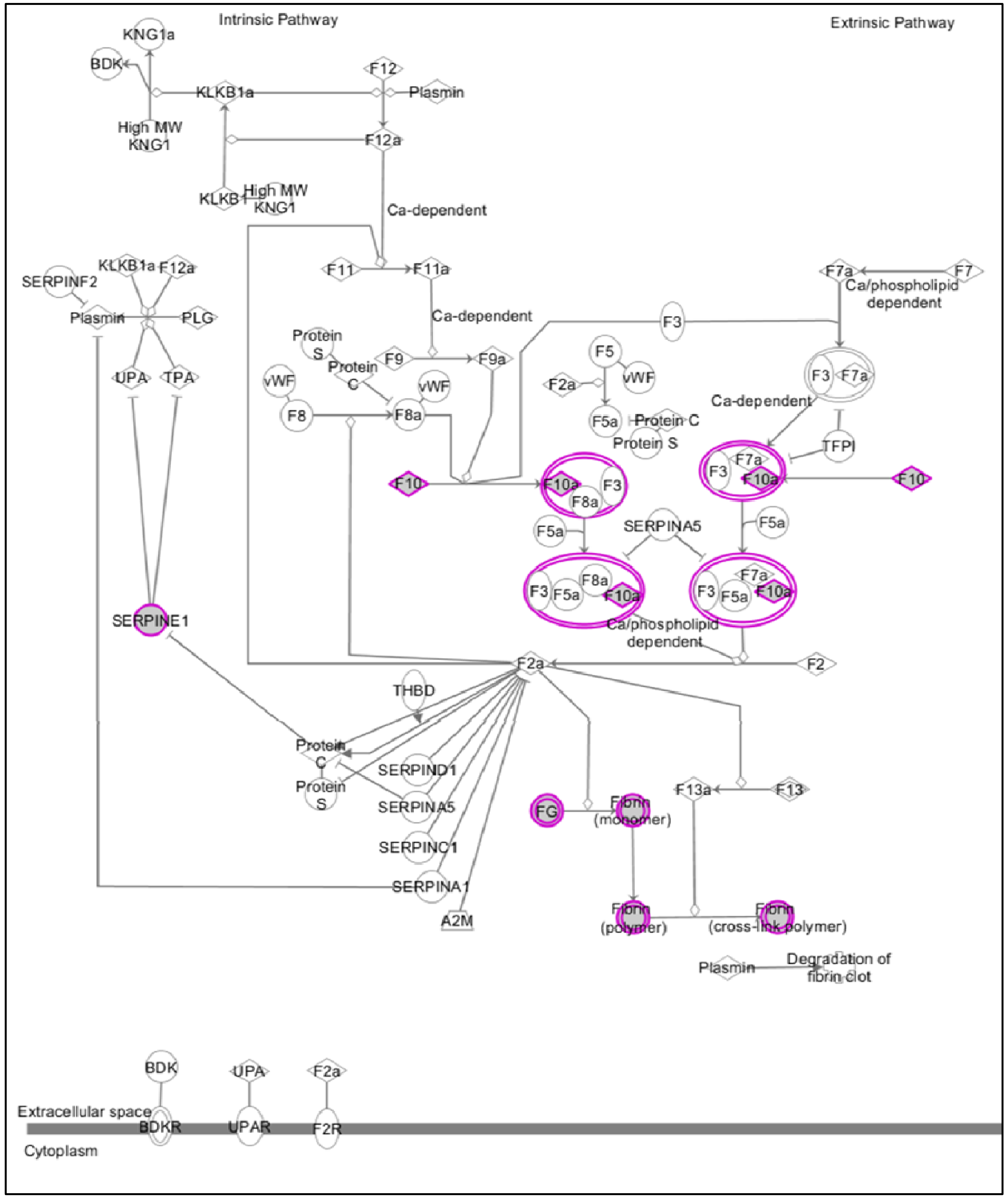

40-G Protein Signaling Mediated by Tubby

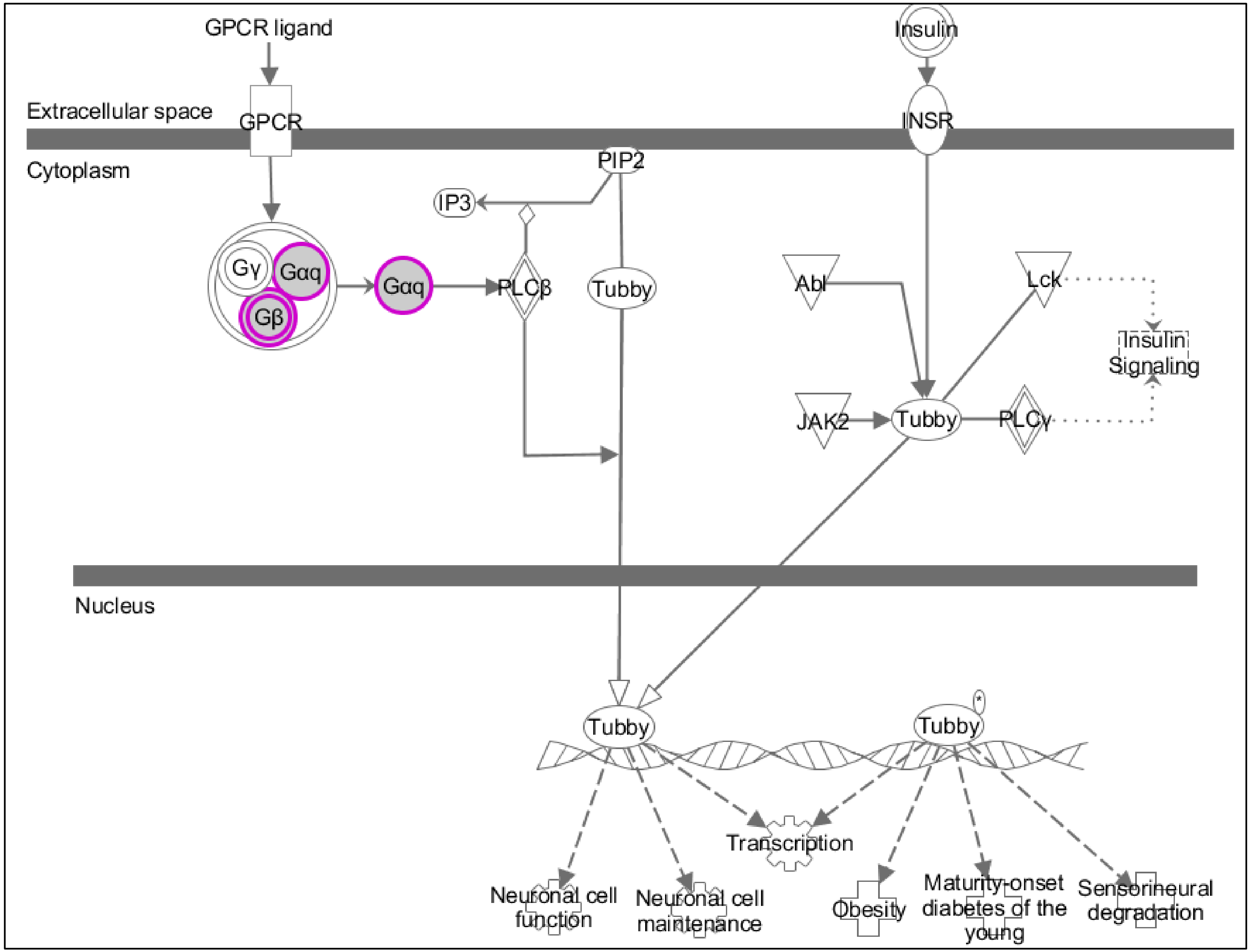

41-D-glucuronate Degradation Ⅰ

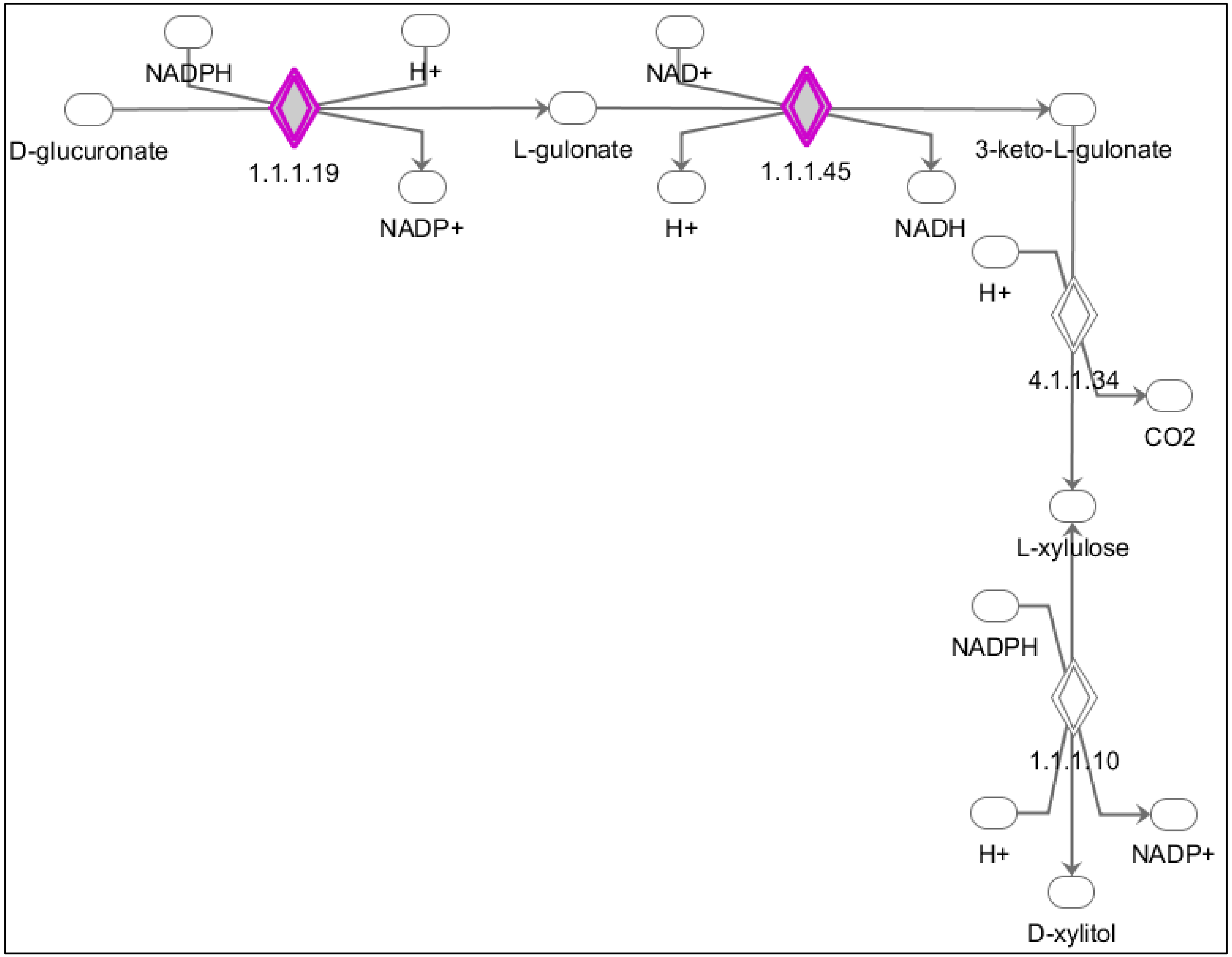

42-Role of NFAT in Regulation of the Immune Response

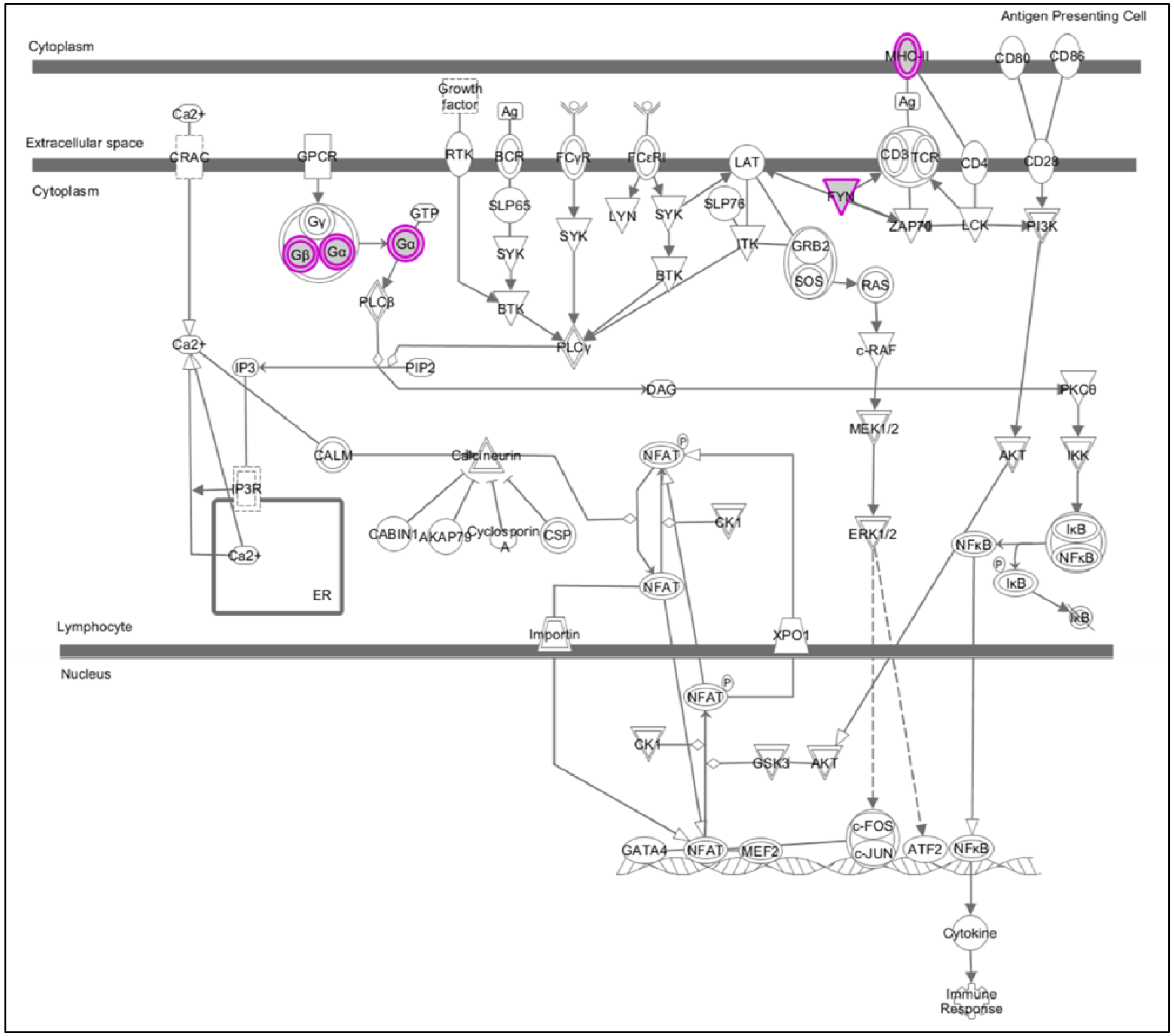

43-Ephrin Receptor Signaling

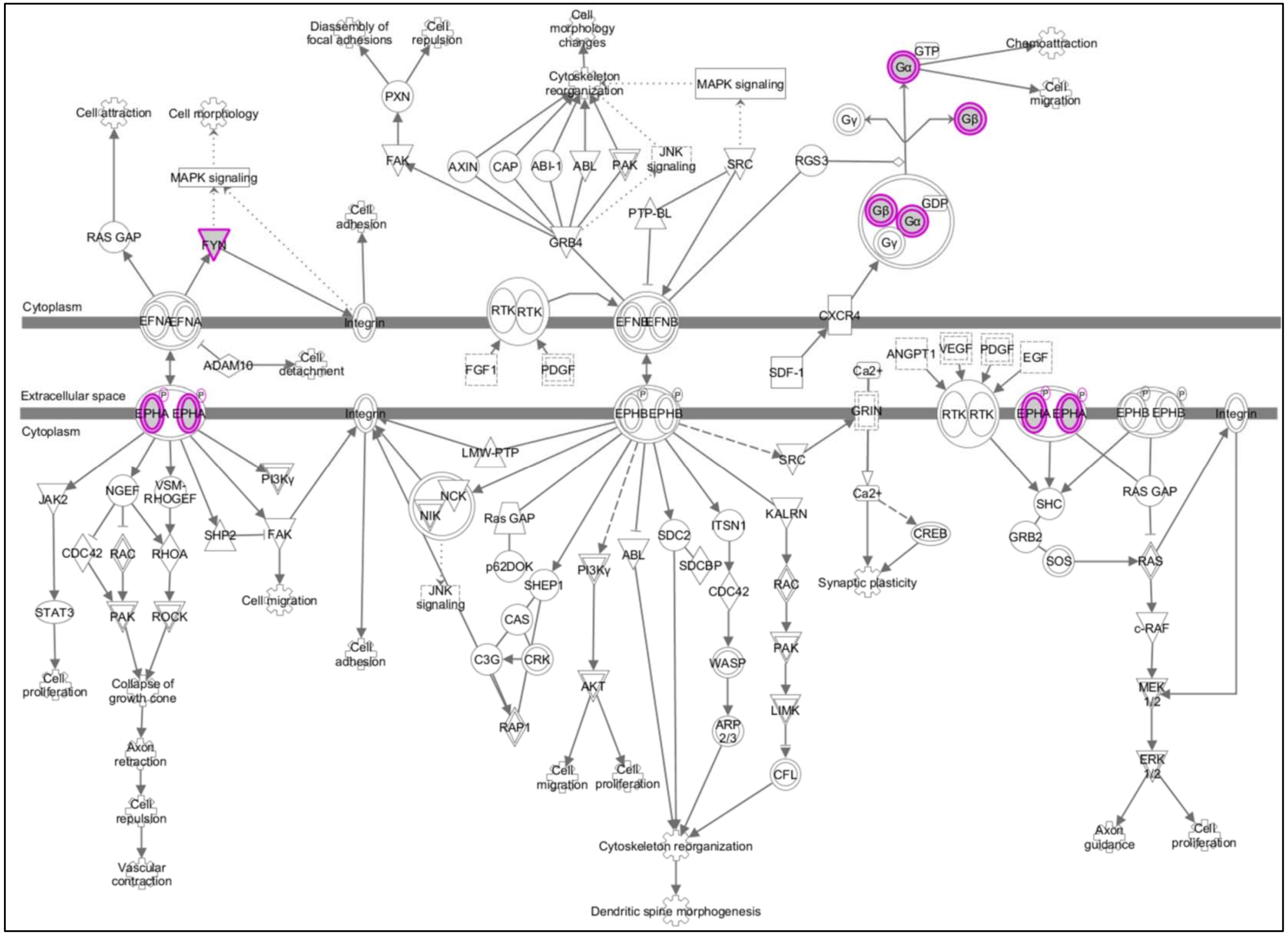

44-Glutathionemediated Detoxification

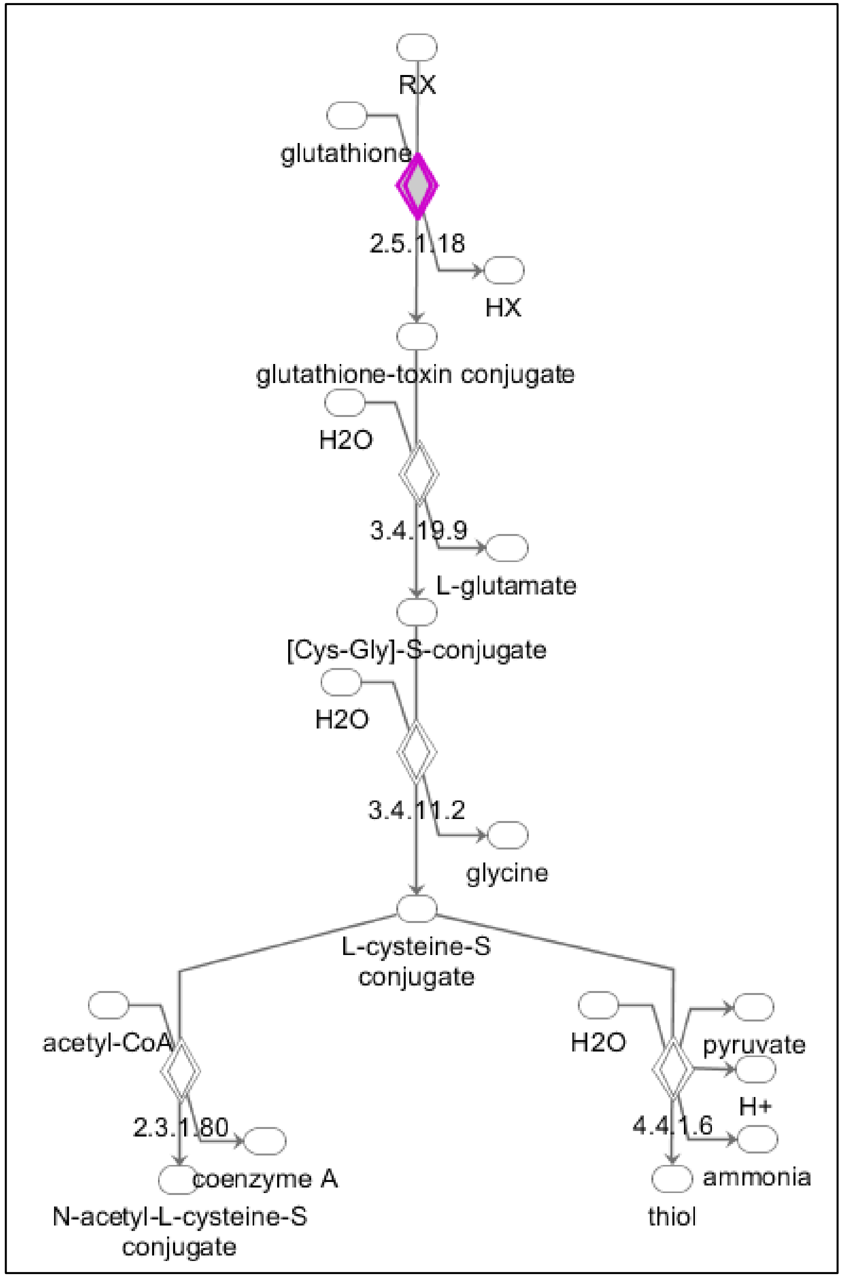

45-Signaling by Rho Family GTPases

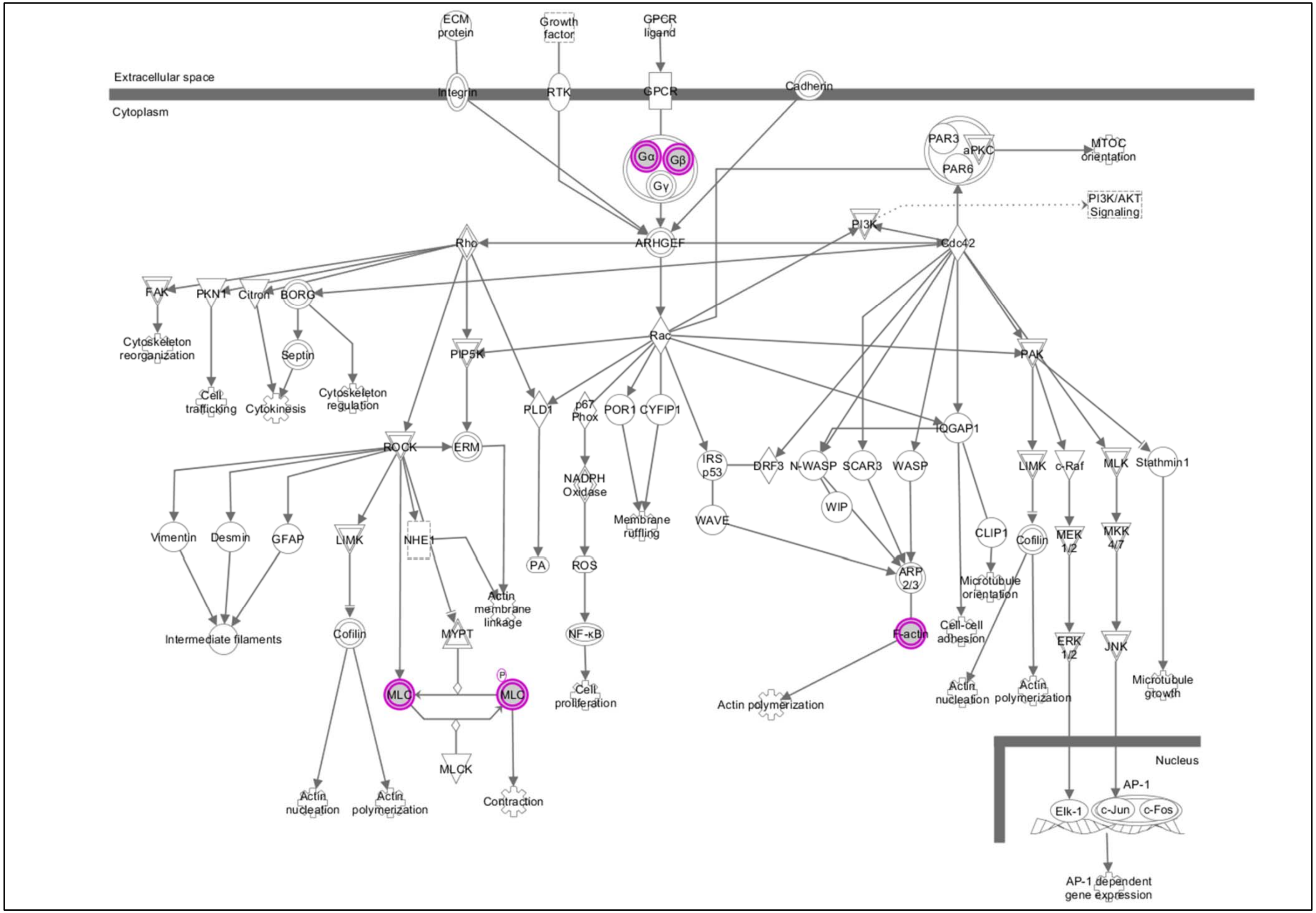

46-p70S6K Signaling

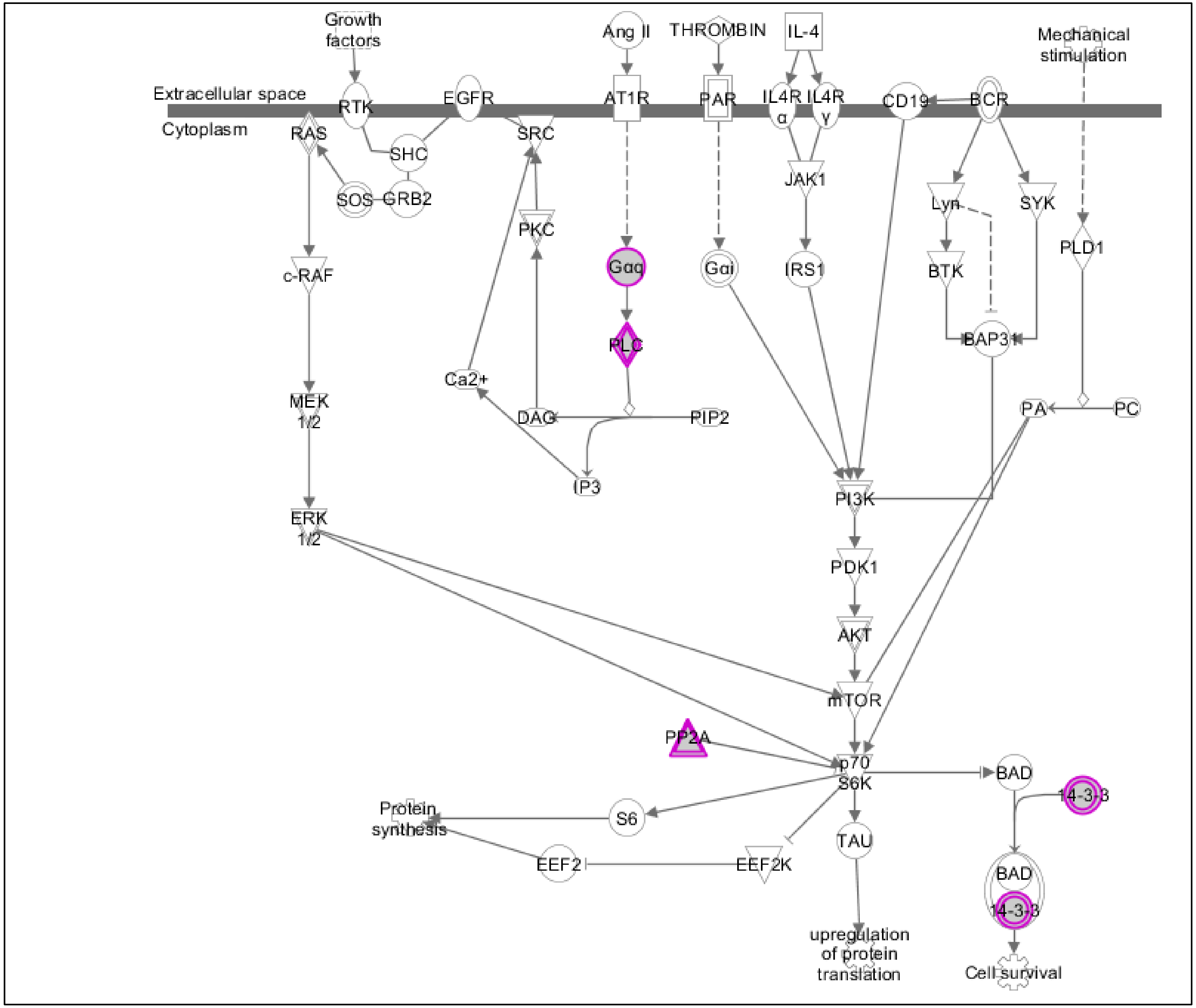

47-PI3K/AKT Signaling

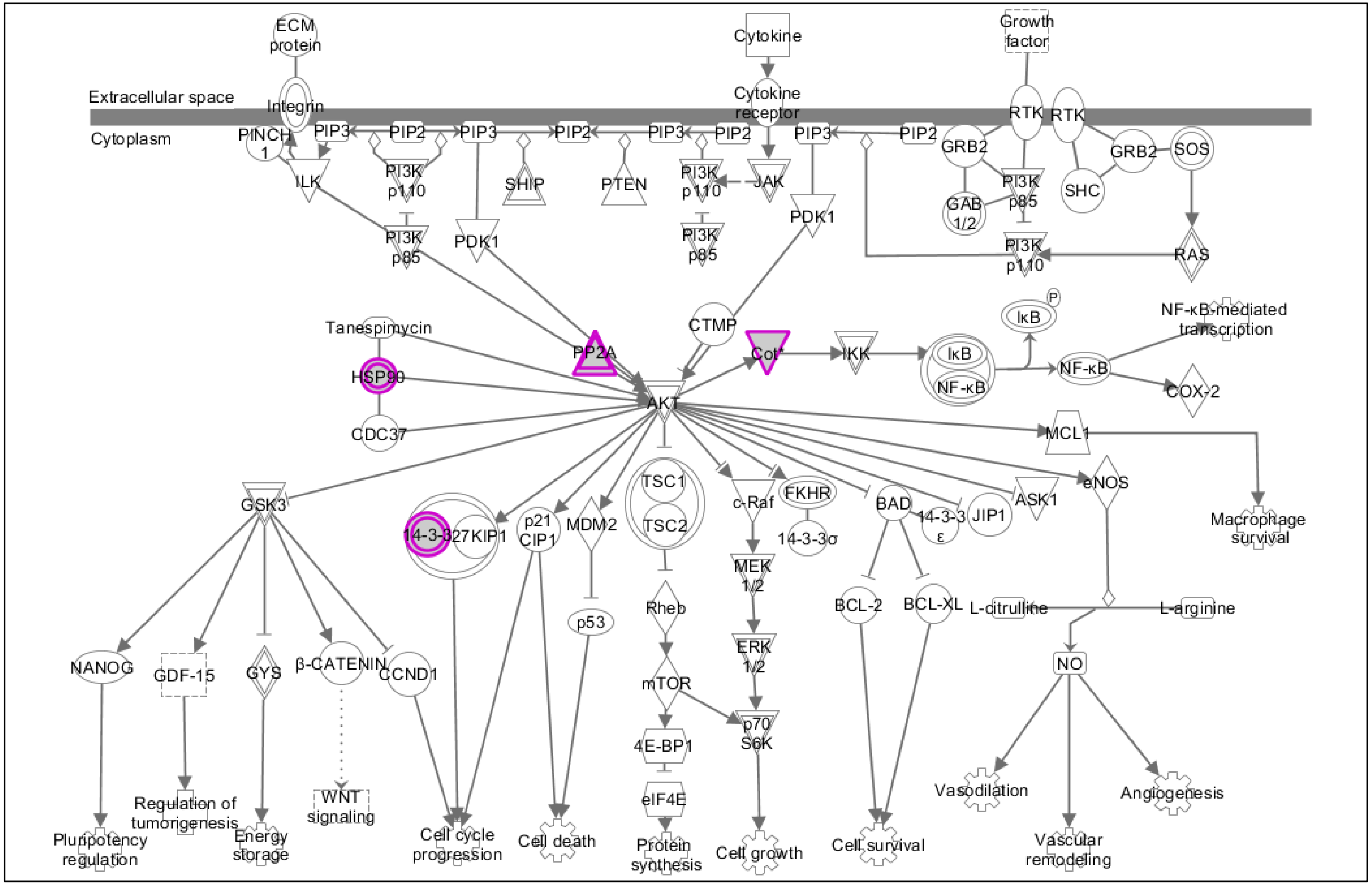

48-Fatty Acid β-oxidation Ⅰ

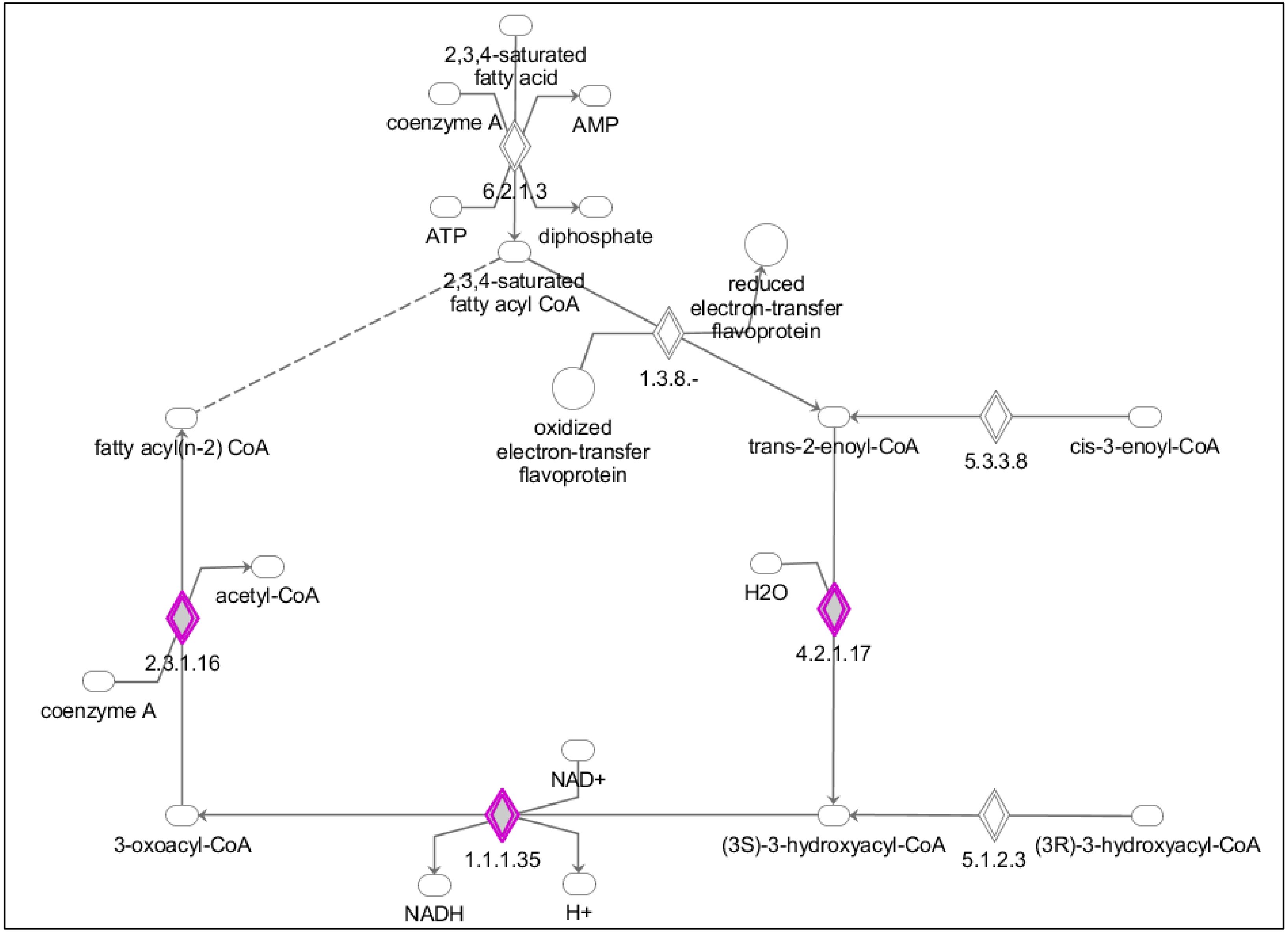

49-Clathrin-mediated Endocytosis Signaling

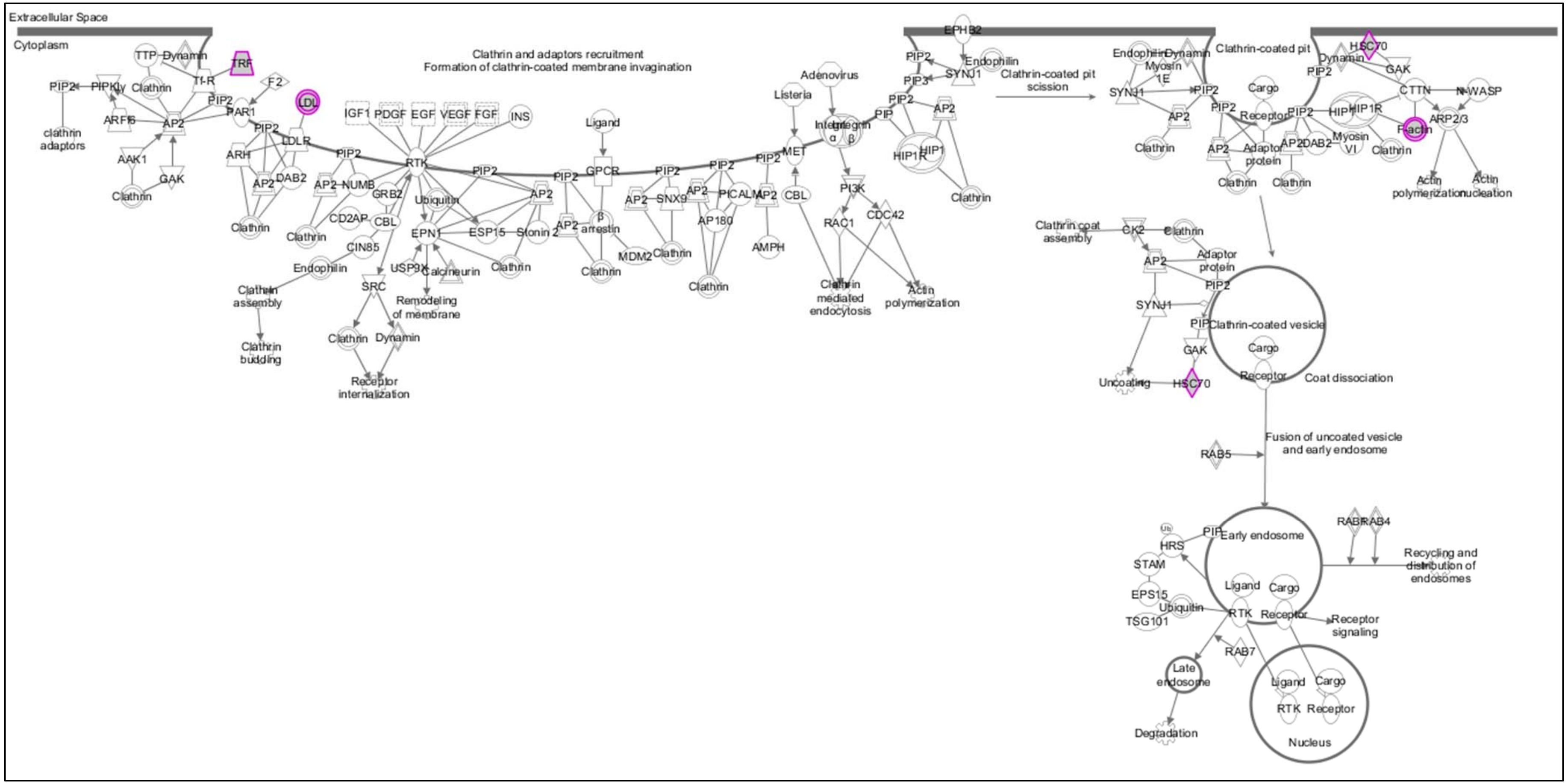

50-Aspartat Degradation II

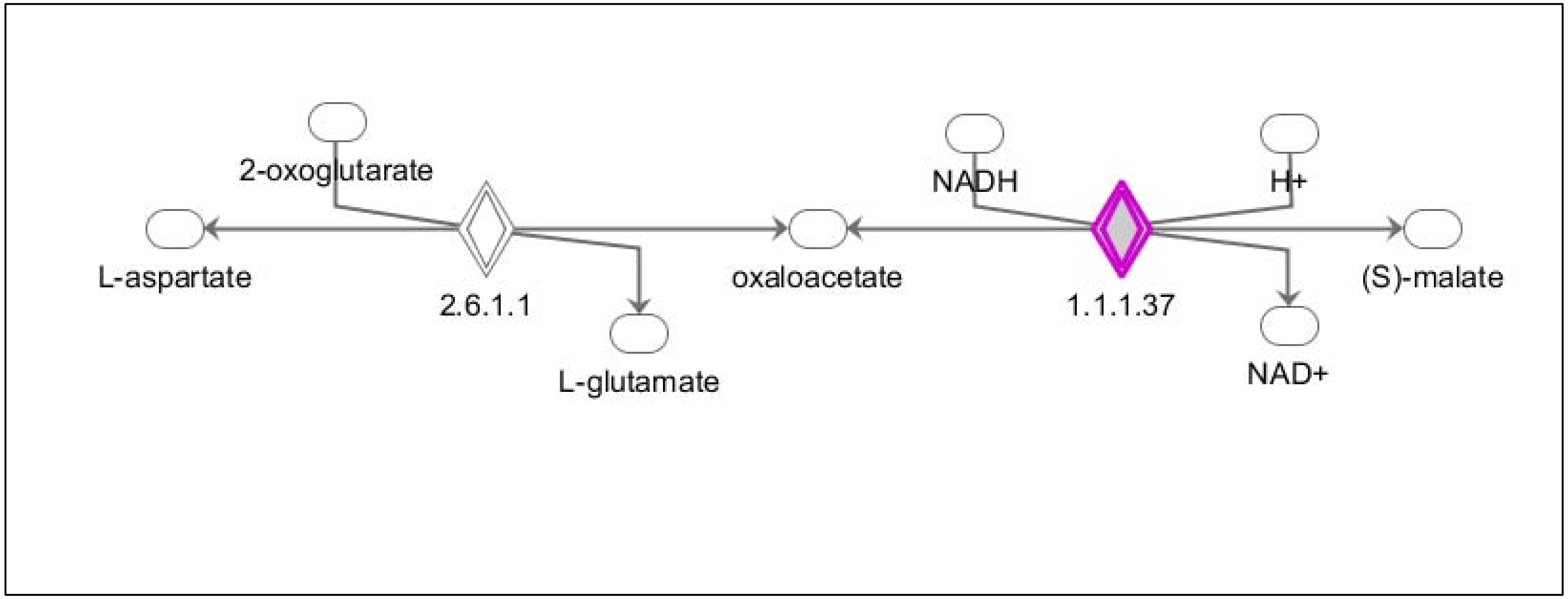

51-Telomere Extension by Telomerase

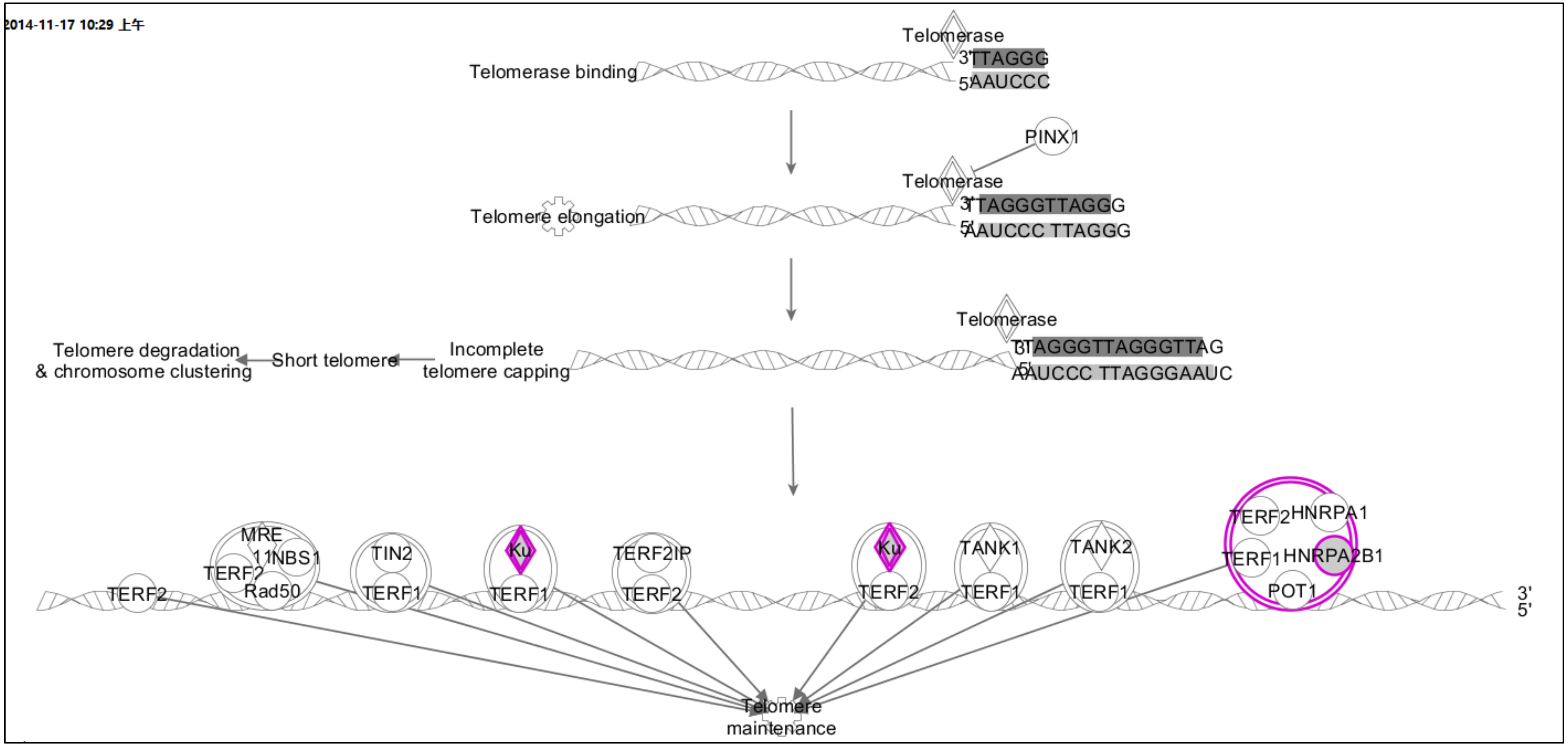

52-Thrombin Signaling

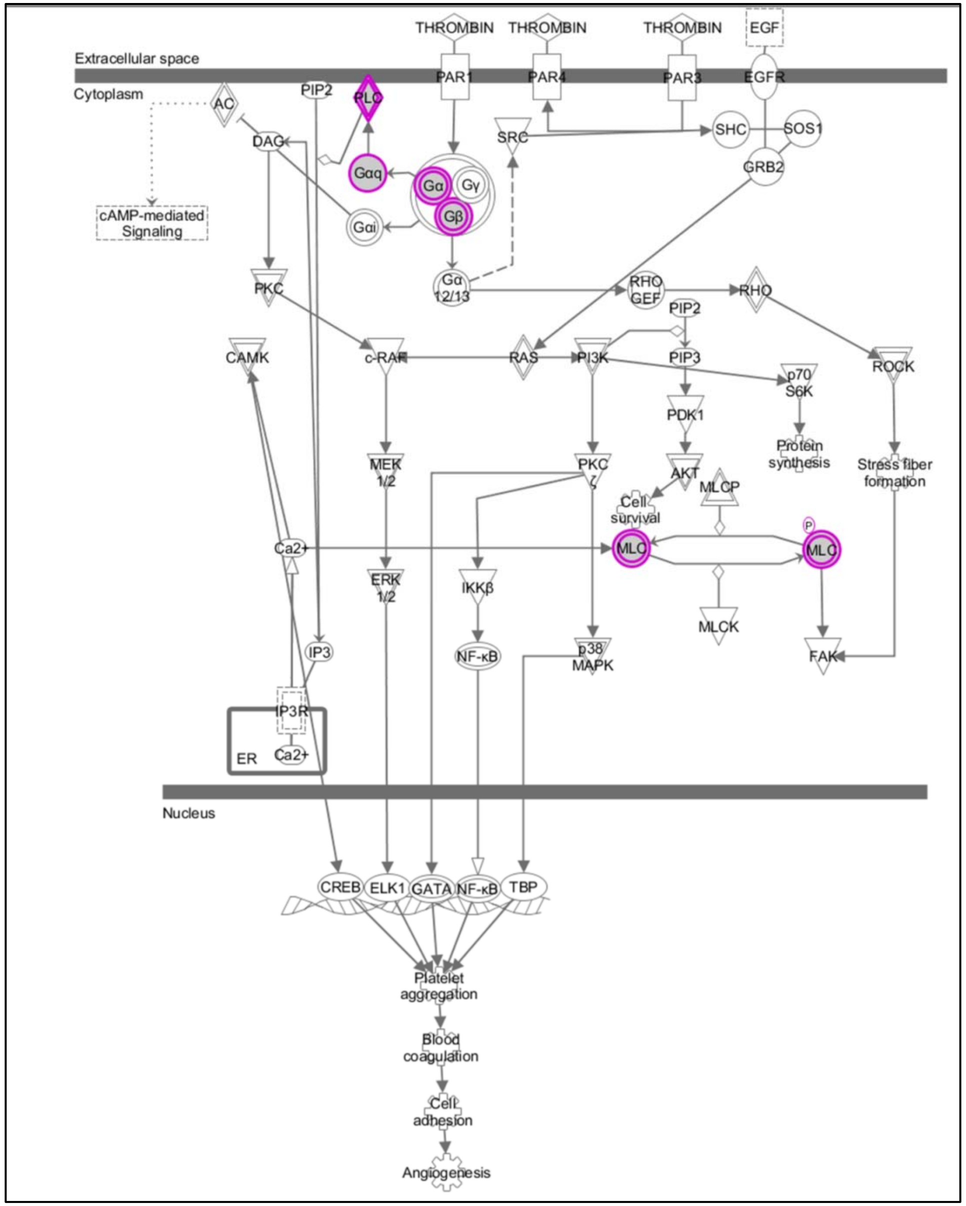

53-FAK Signaling

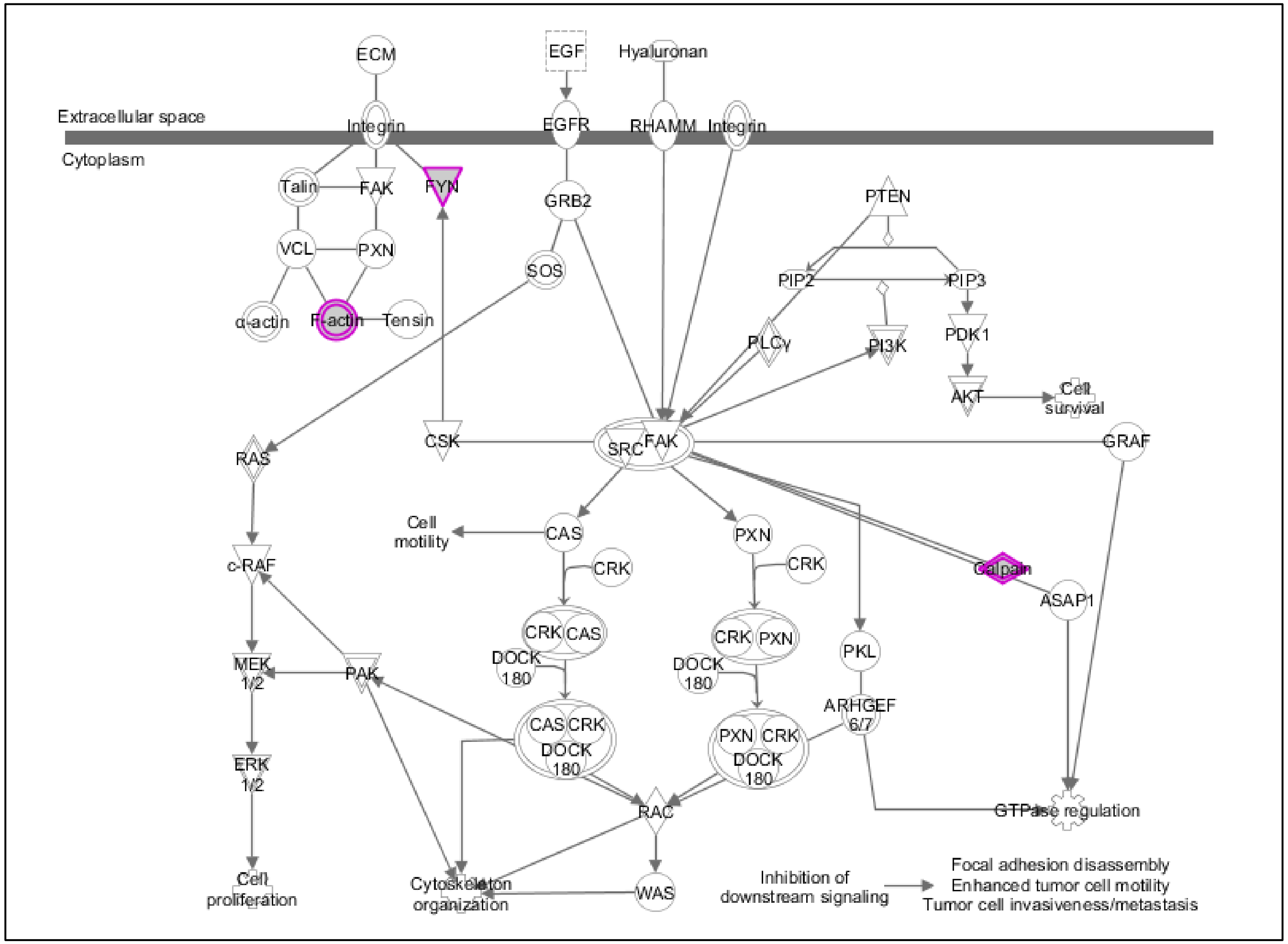

54-Parkinson’s Signalng

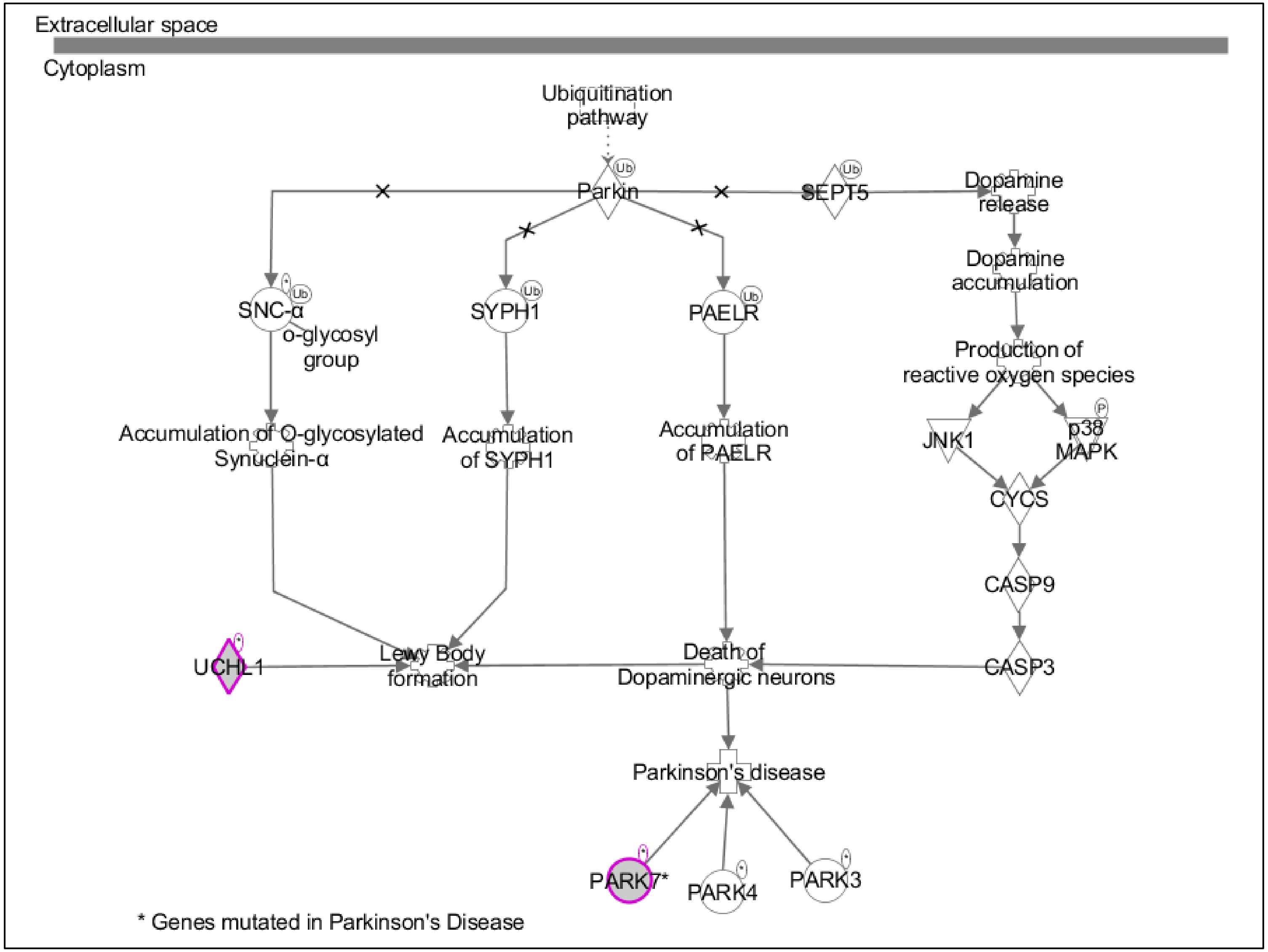

55-TR/RXR Activation

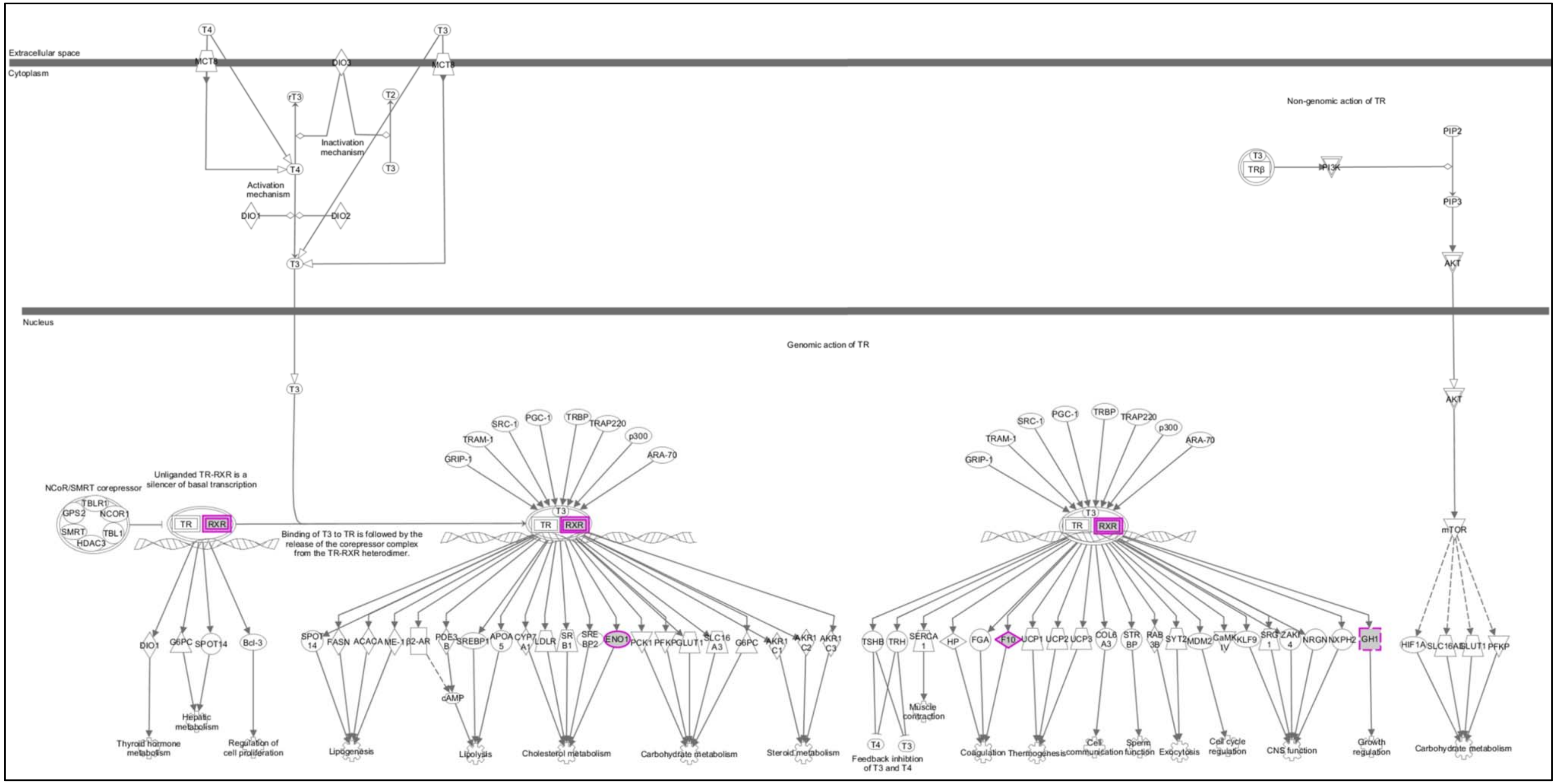

56-IL-1 Signaling

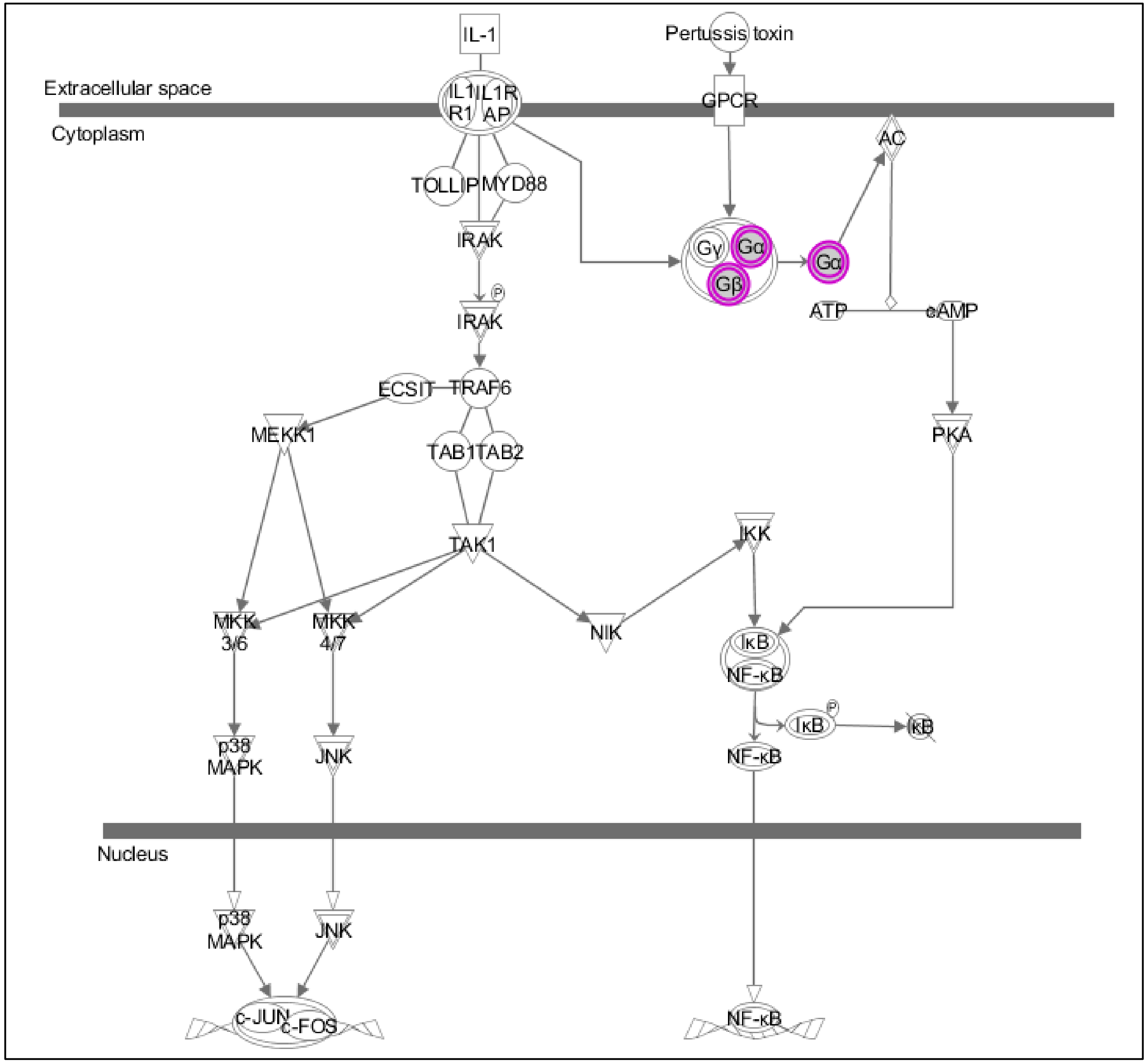

57-Virus Entry via Endocytic Pathways

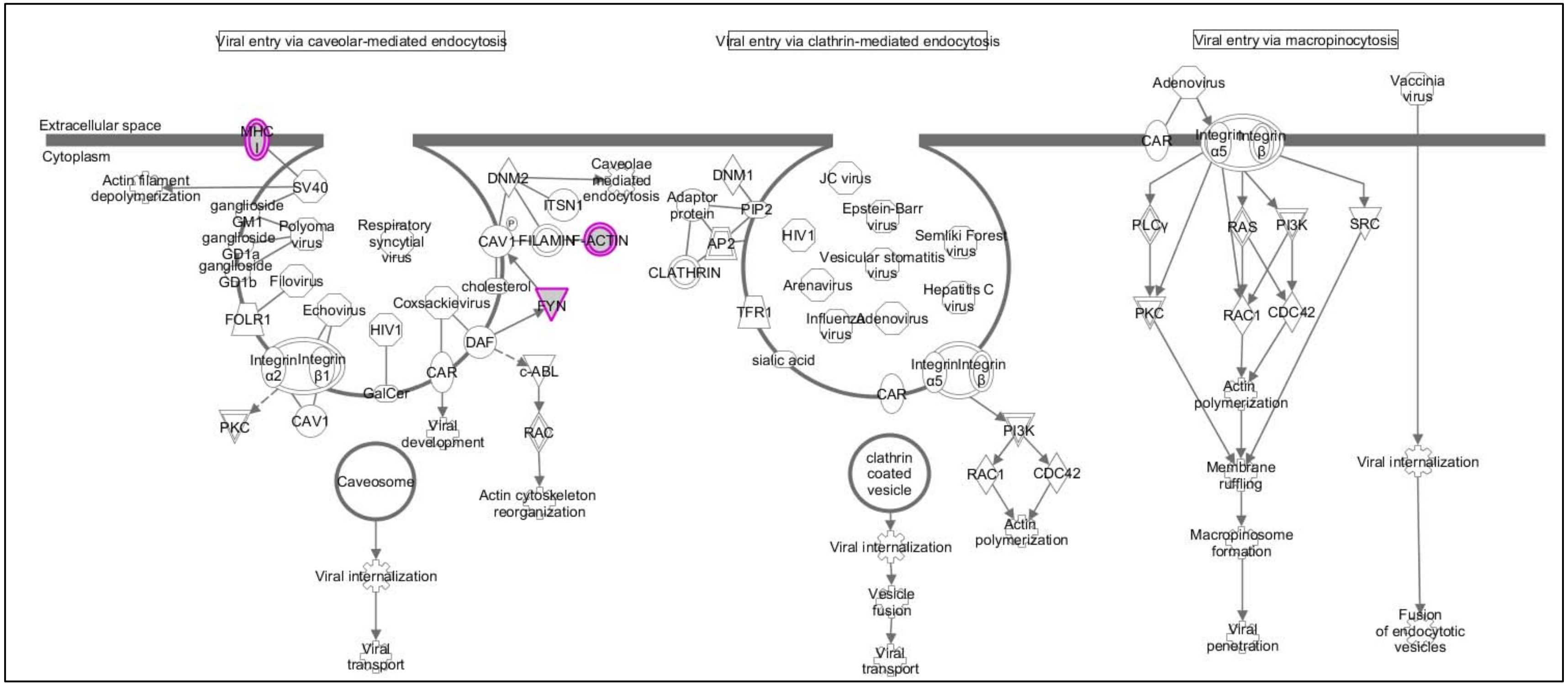

58-Synaptic Long Term Depression

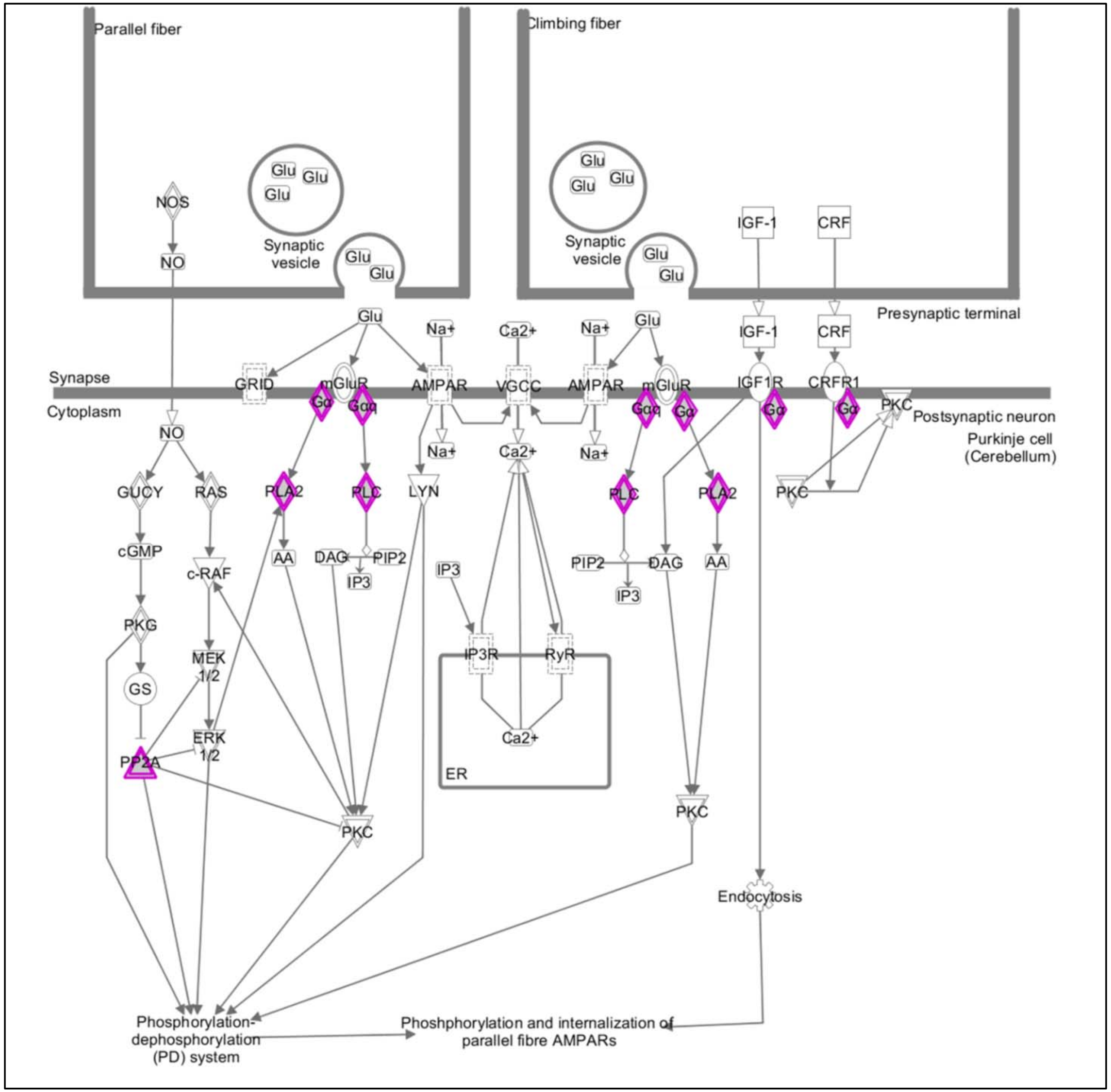

59-Extrinsic Prothrombin Activation Pathway

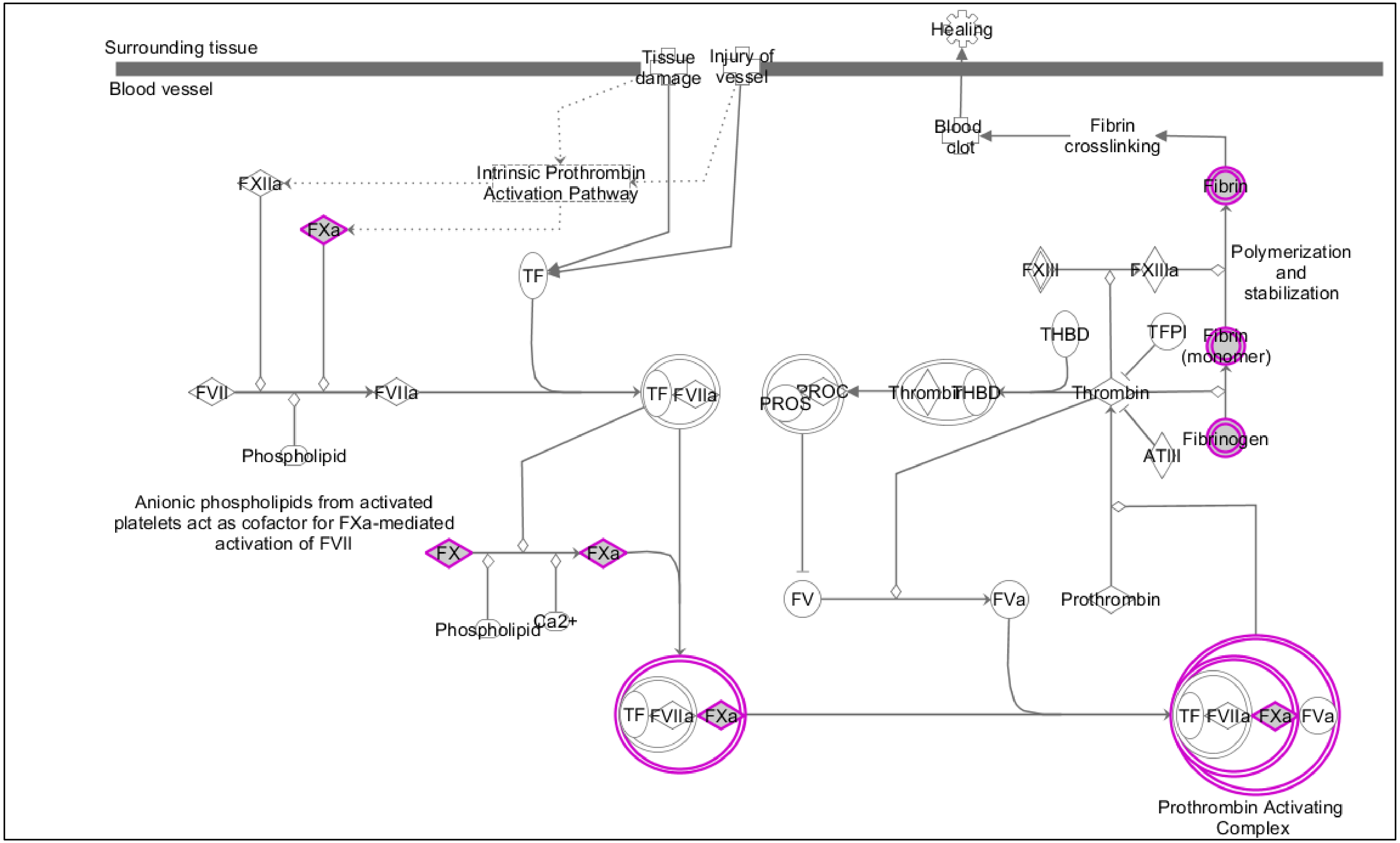

60-G Beta Gamma Signaling

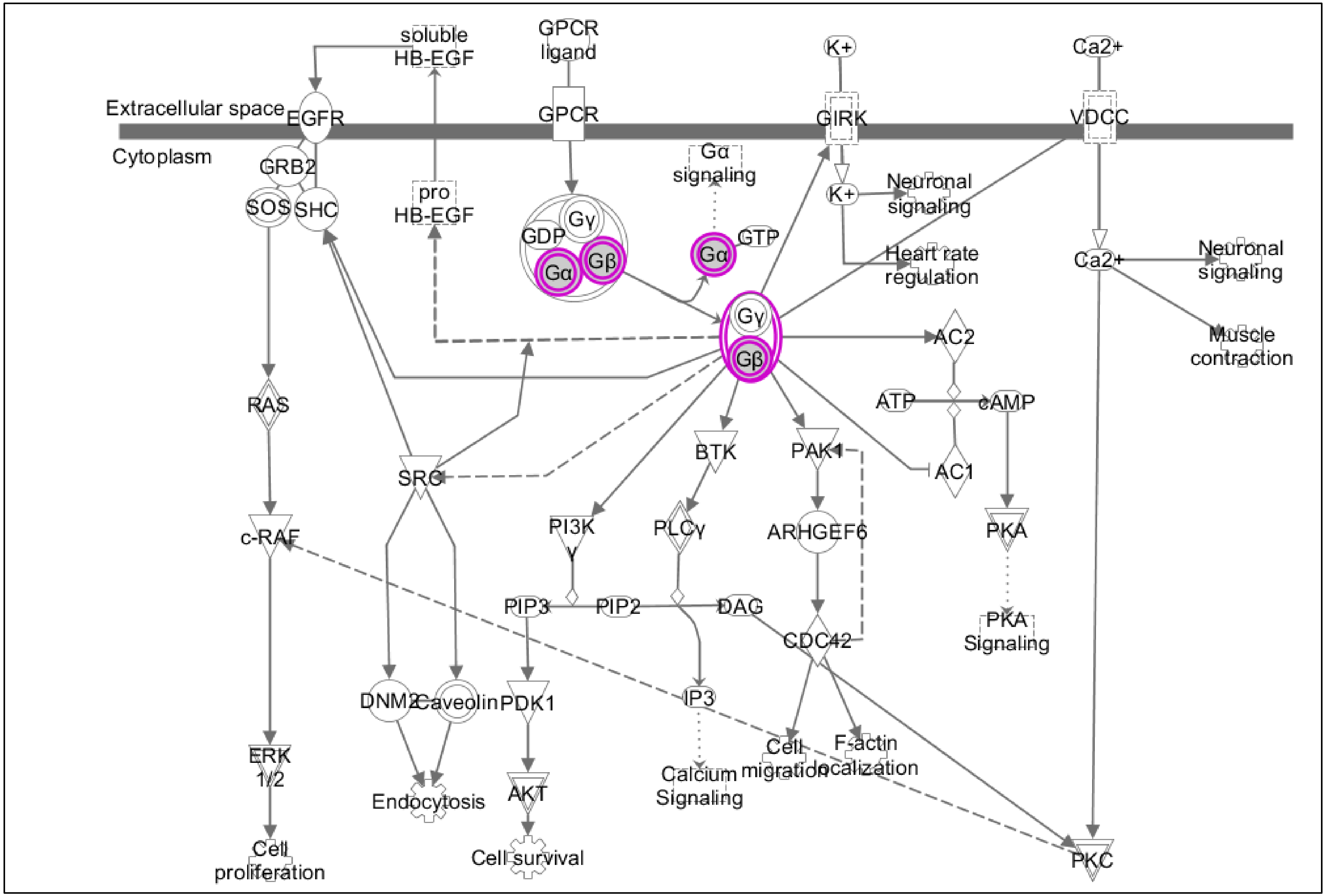

61-CXCR4 Signaling

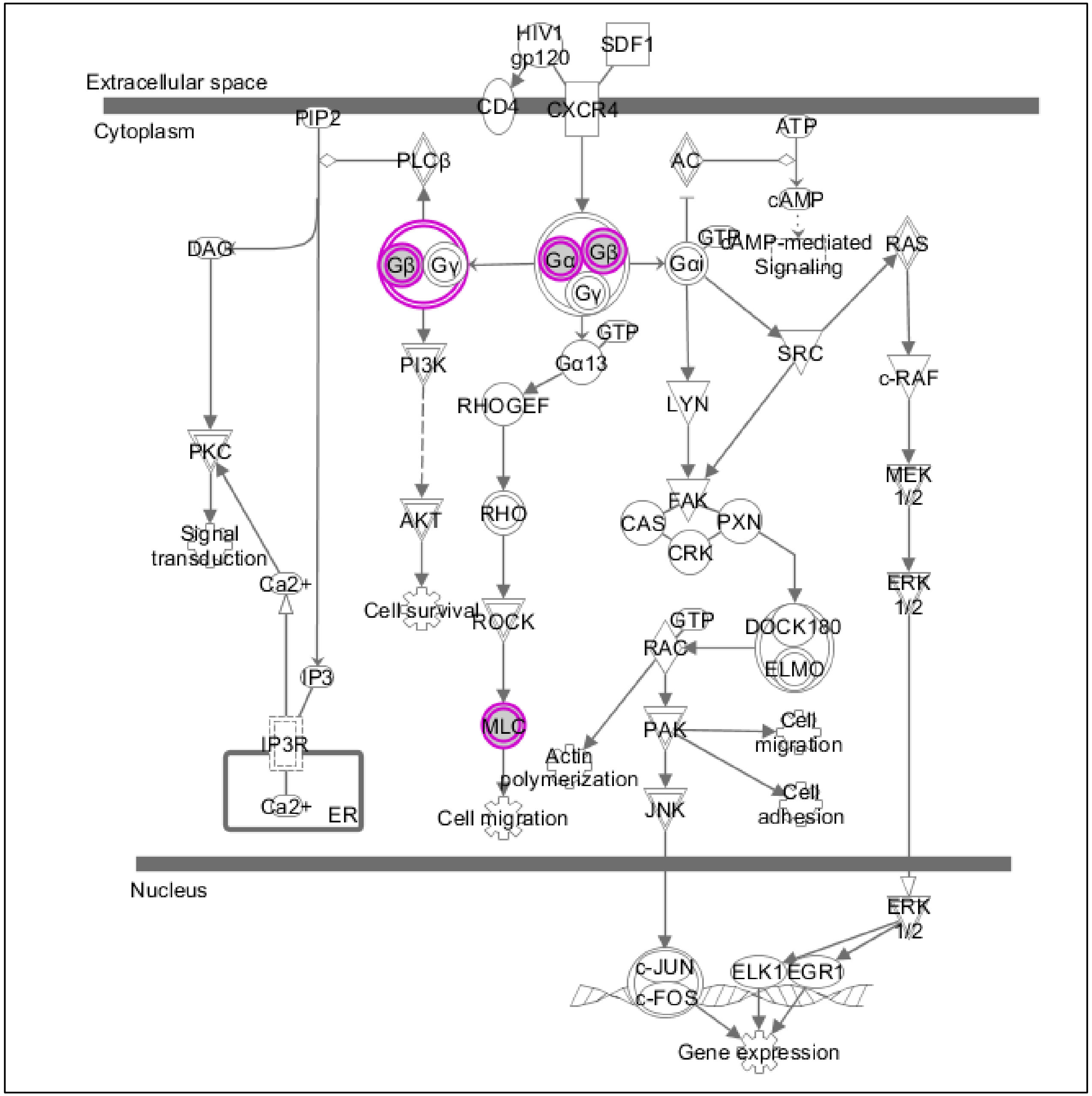

62-Glutaryl-CoA Degradation

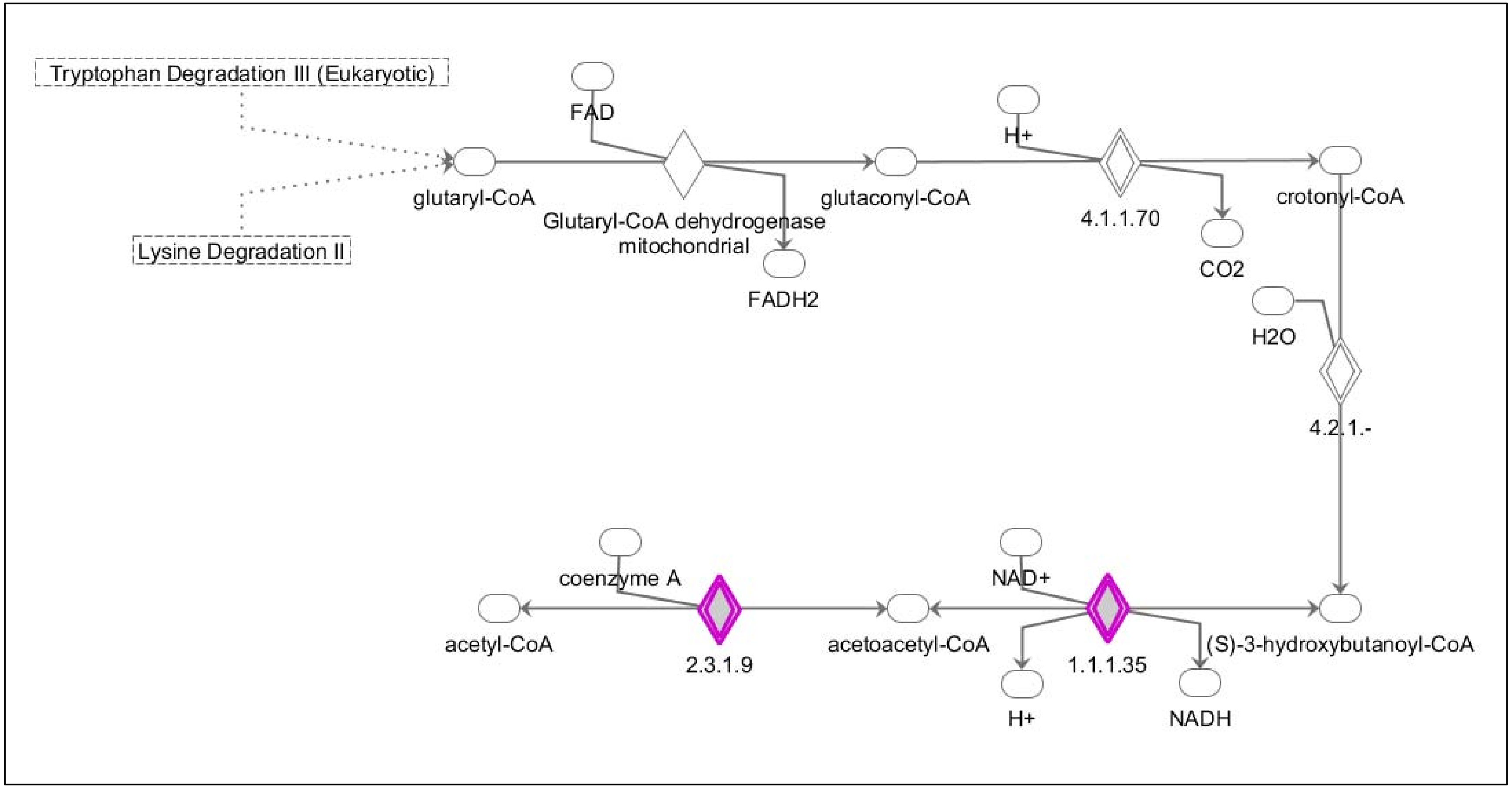

63-Polyamine Regulation in Colon Cancer

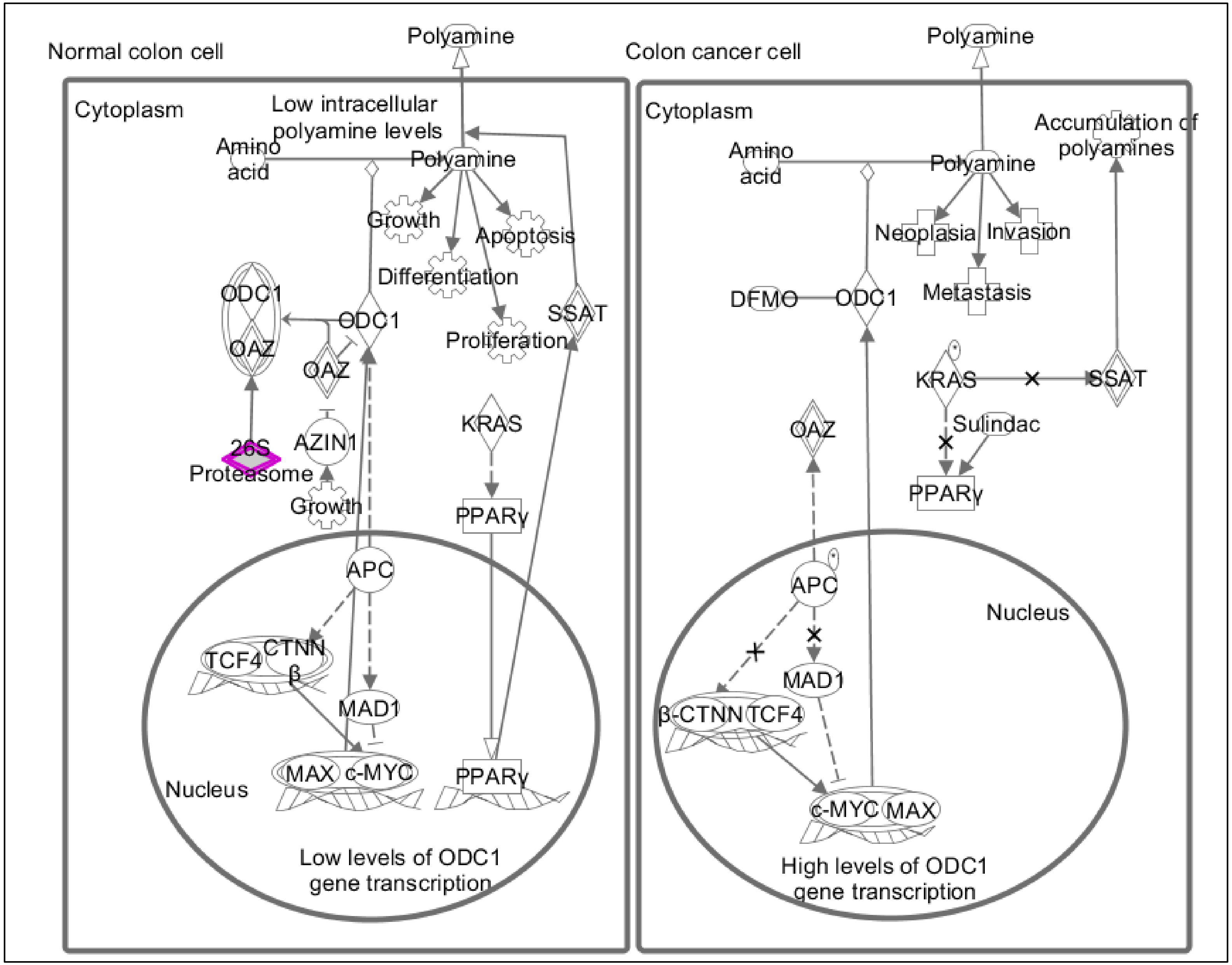

64-CREB Signaling in Neurons

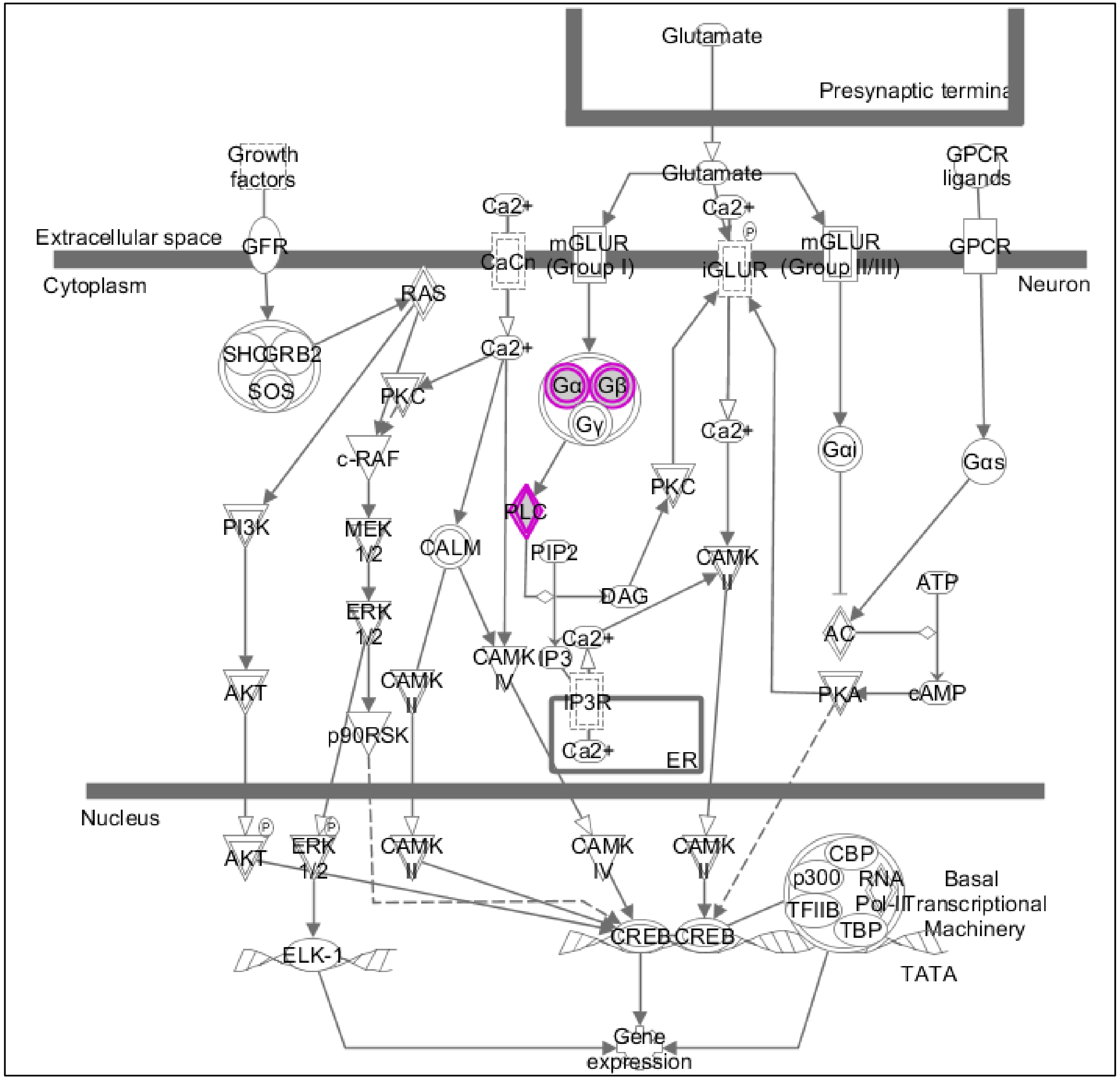

65-Retinoic acid Mediated Apoptosis Signaling

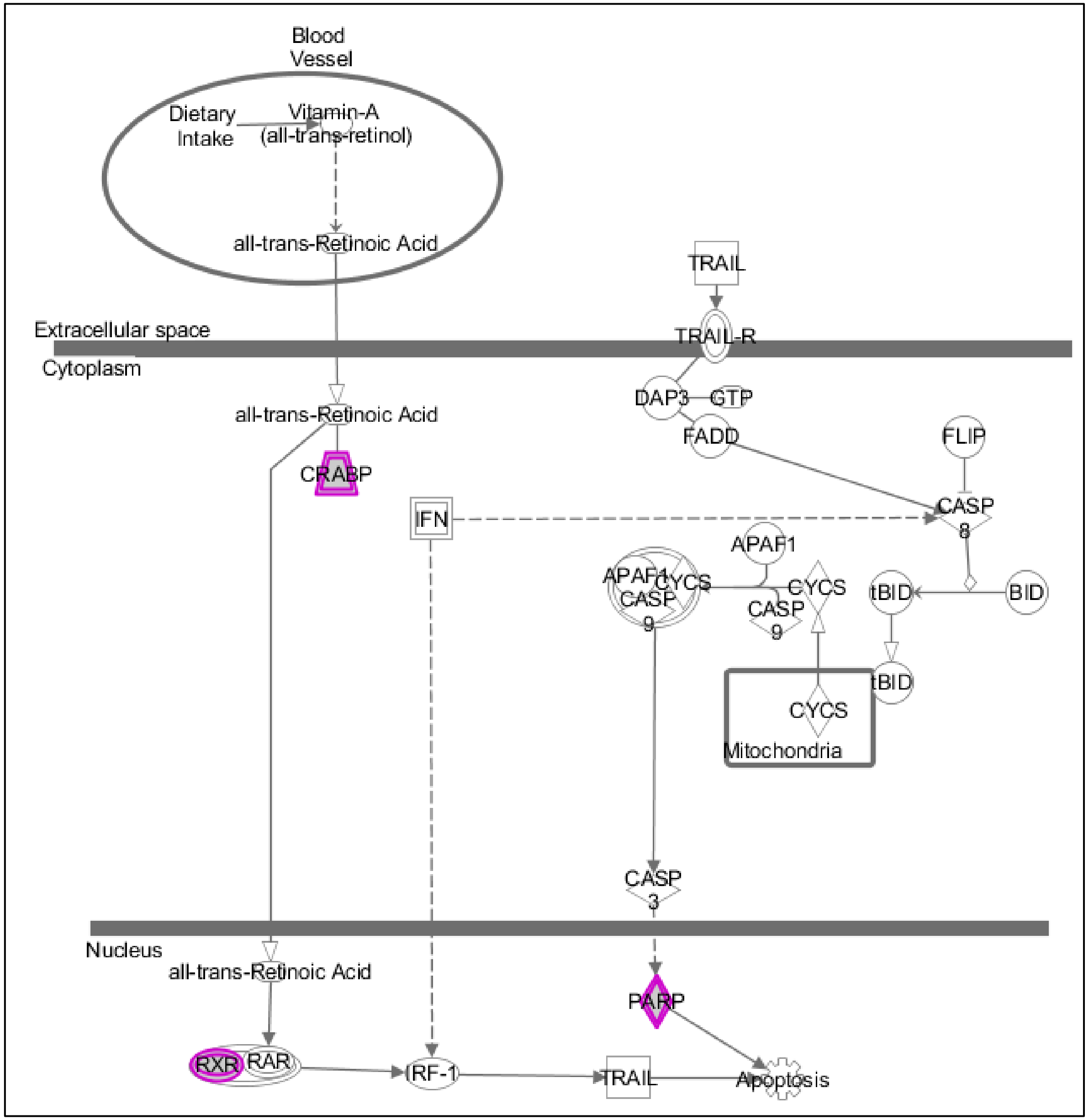

66-Oxidative Phosphorylation

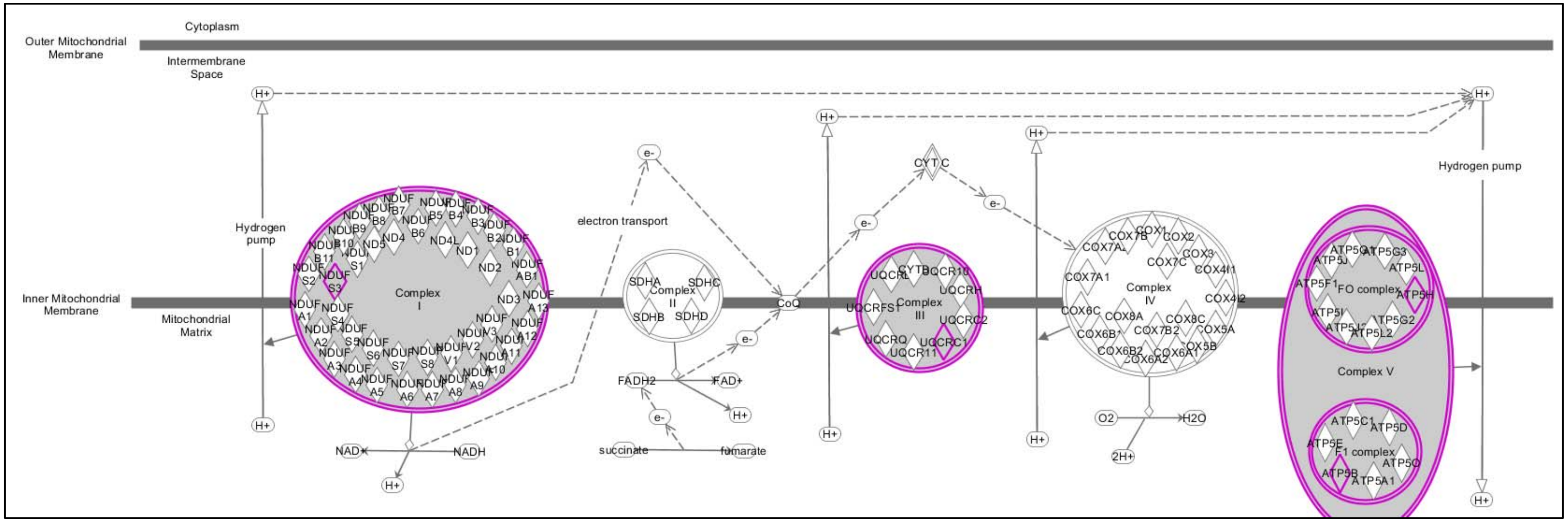

67-Lipid Antigen Presentation by CD1

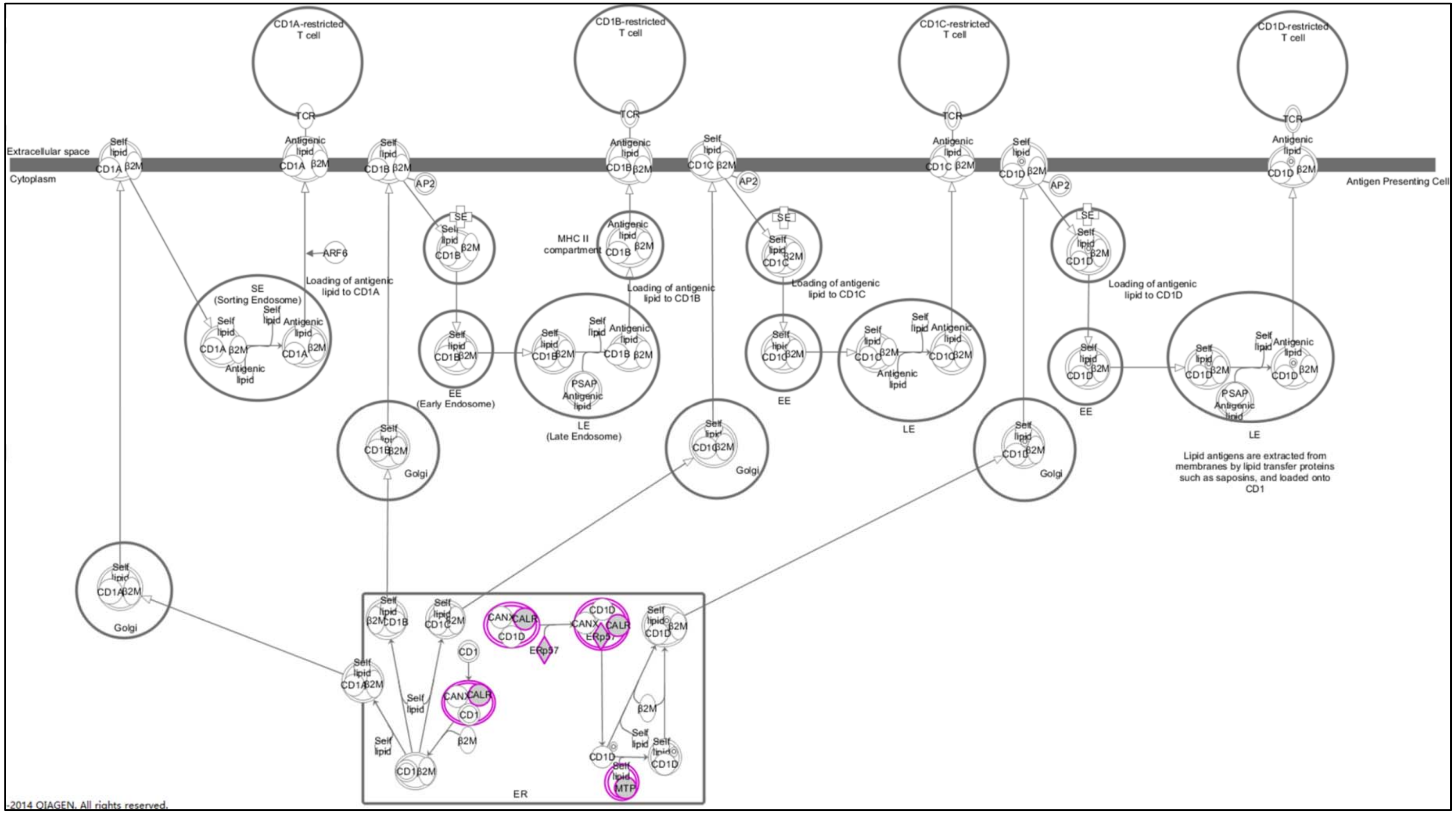

-2014 QIAGEN. All rights reserved.

68-PPARα/RXRα Activation

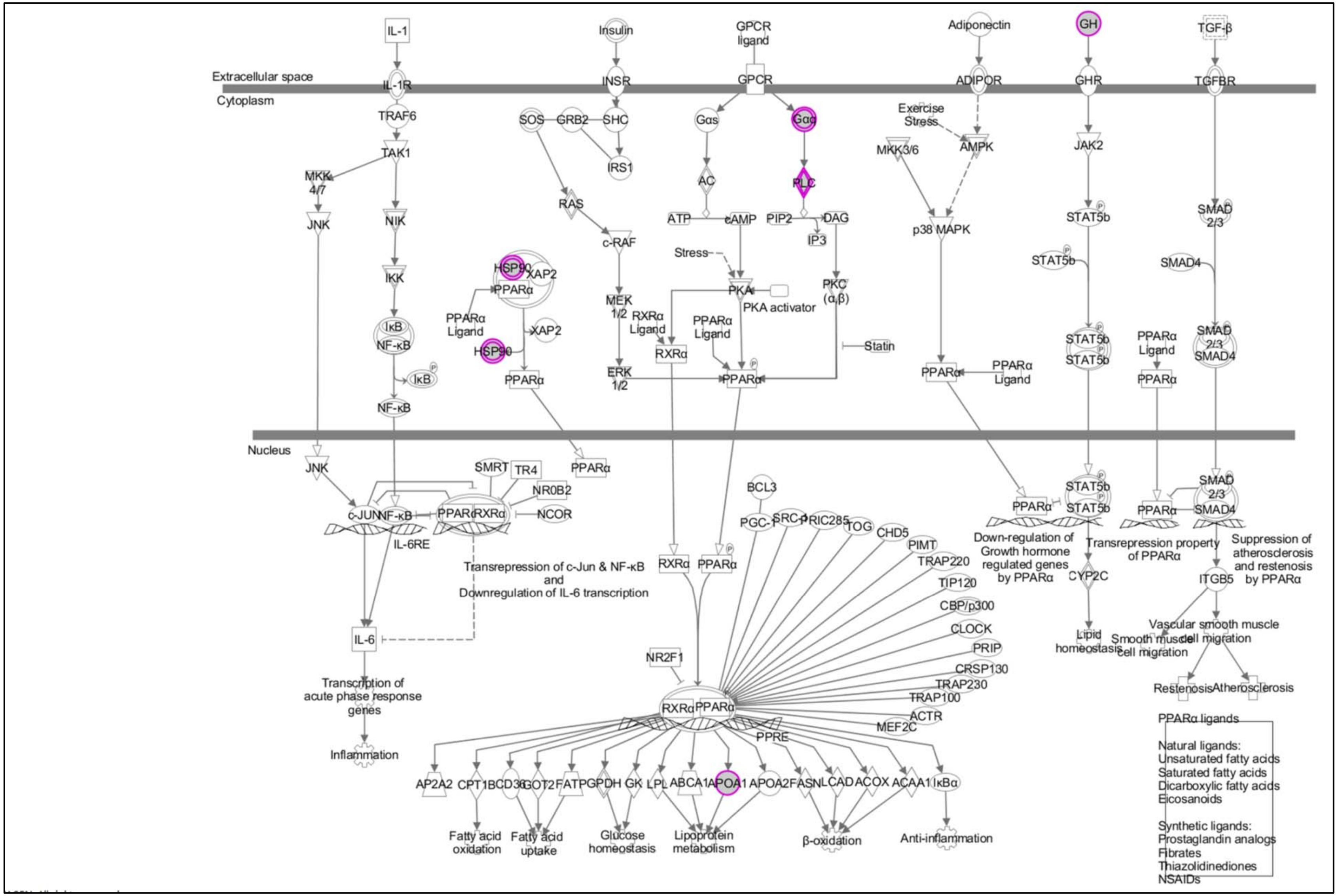

69-Production of Nitric Oxide and Reactive Oxygen Species in Macrophages

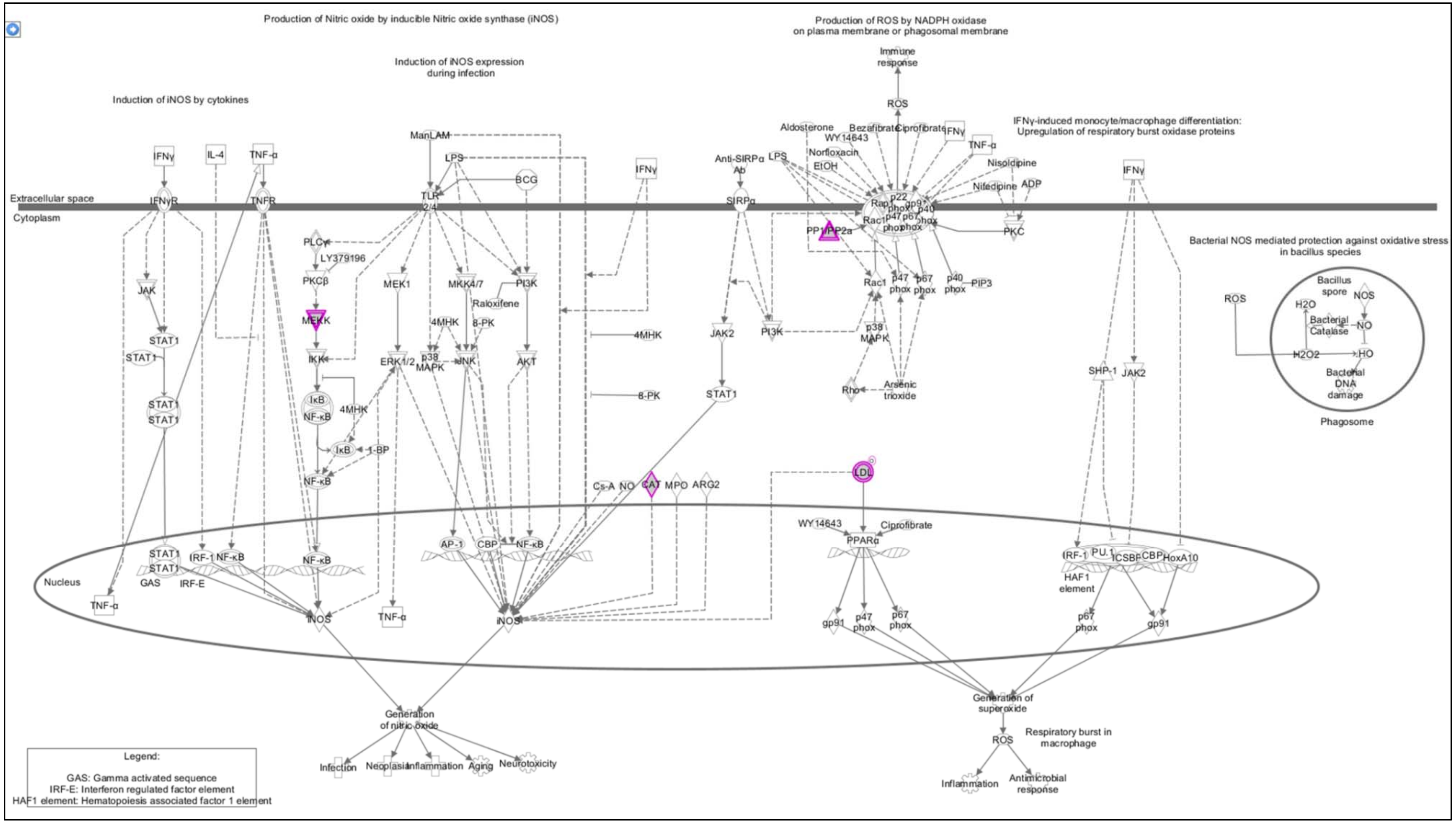

70-ERK/MAPK Signaling

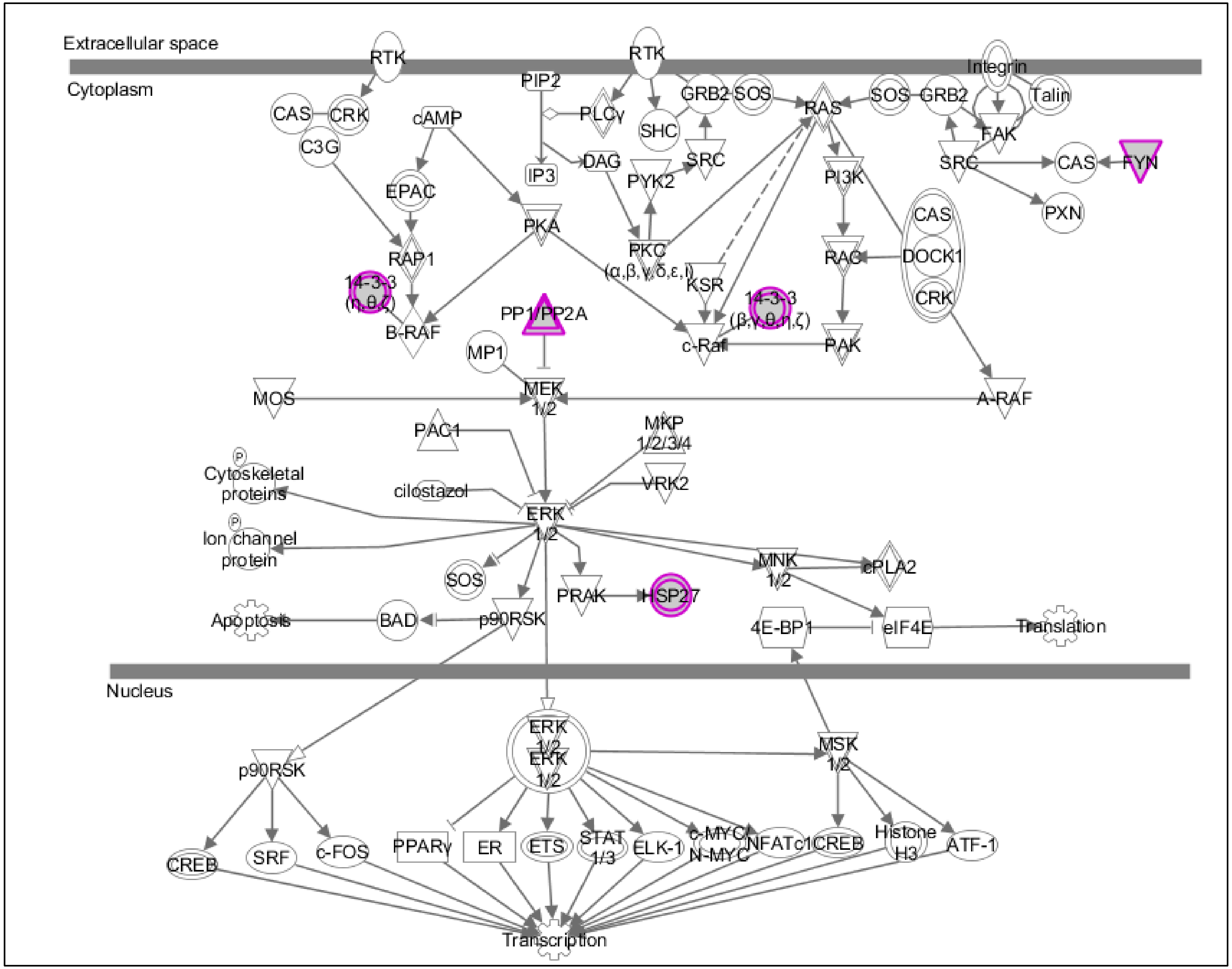

71-P2Y Purigenic Receptor Signaling Pathway

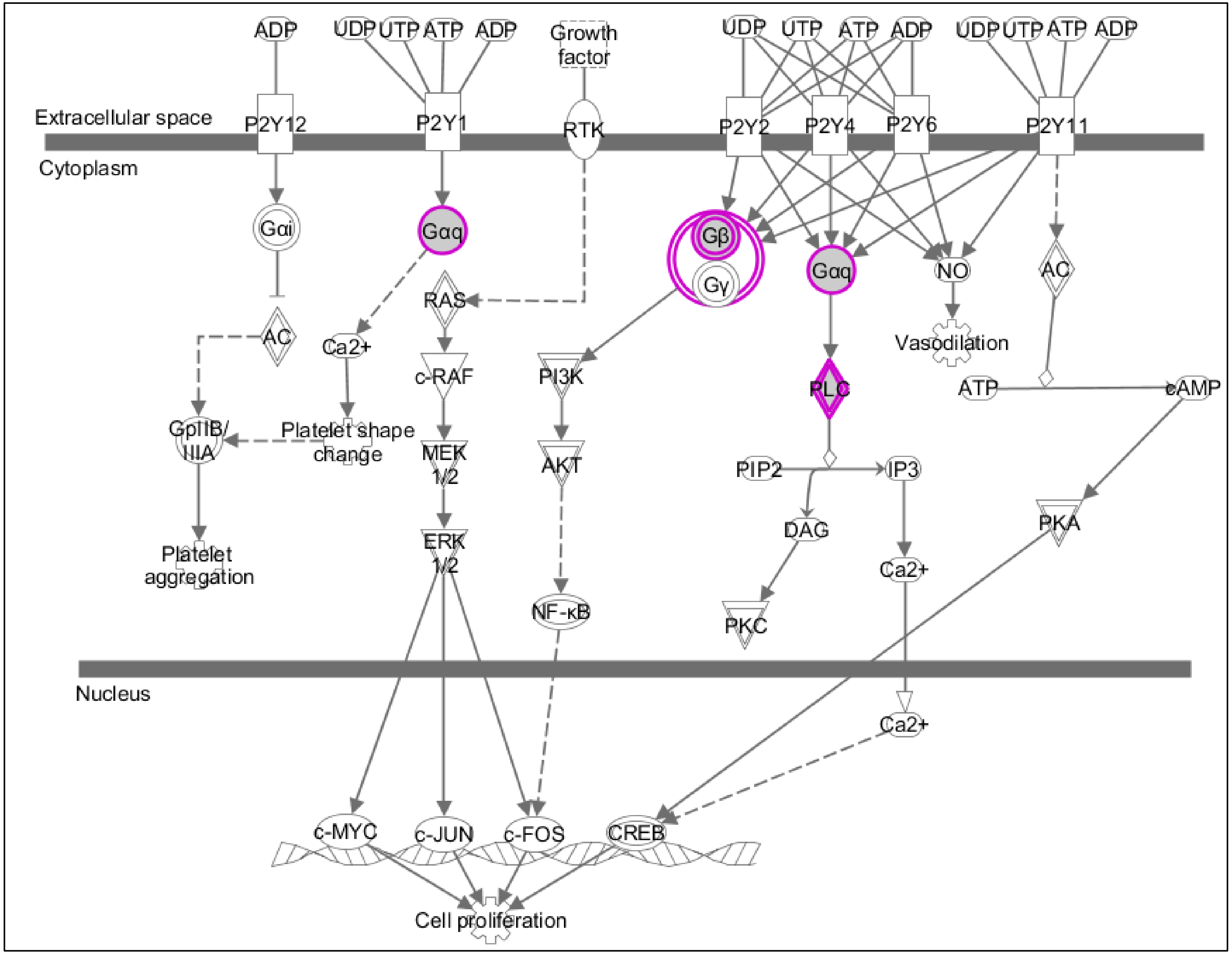

72-Intrinsic Prothrombin Activation Pathway

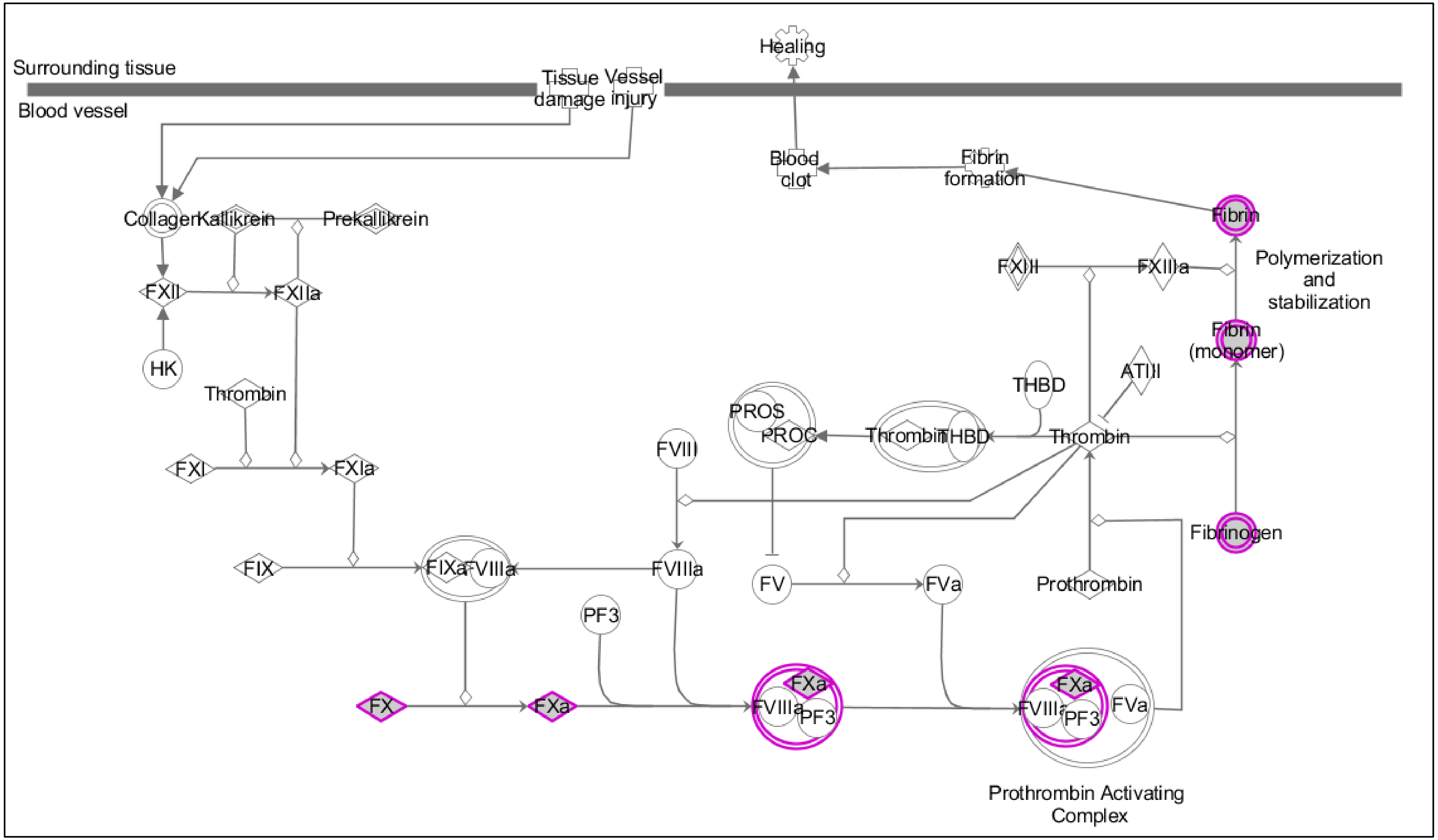

73-Melatonin Signaling

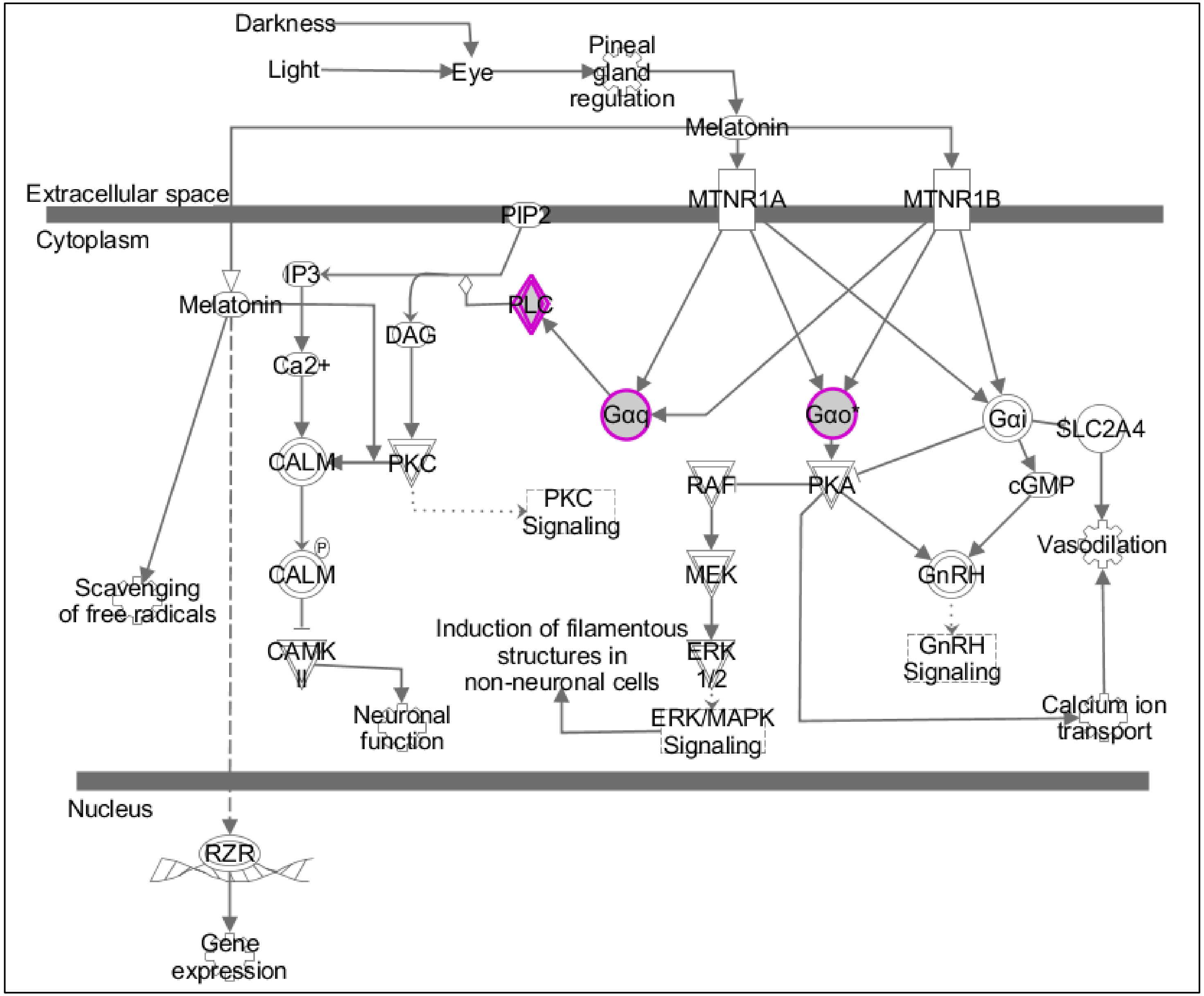

74-Cysteine Biosynthesis III(mammalia)

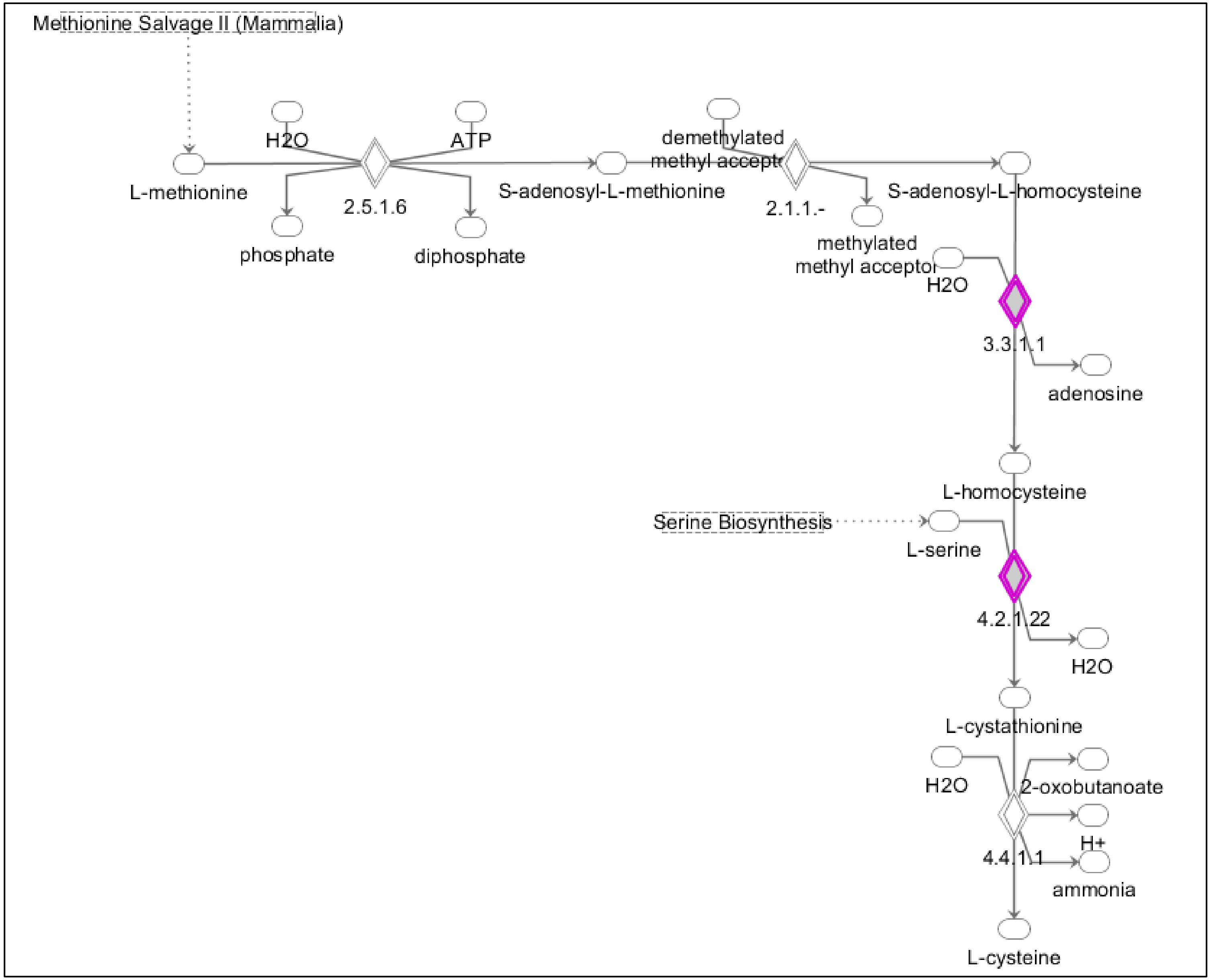

75-Integrin Signaling

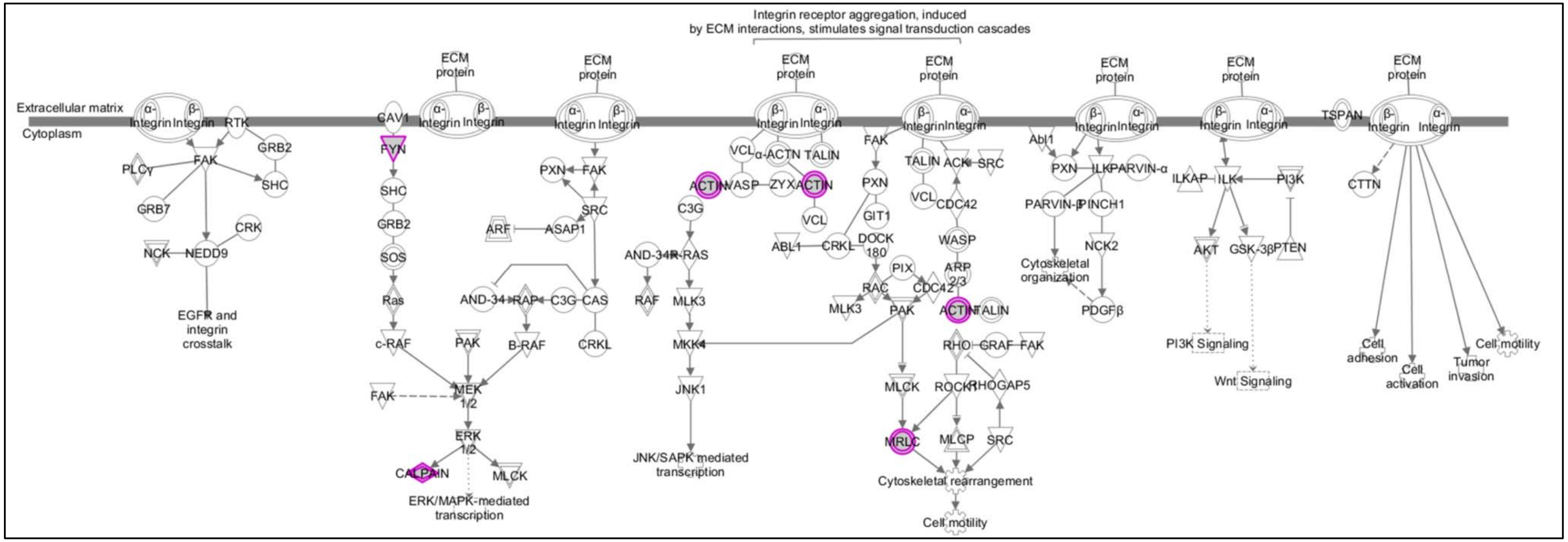

76-Cellular Effects of Sildenafil (Viagra)

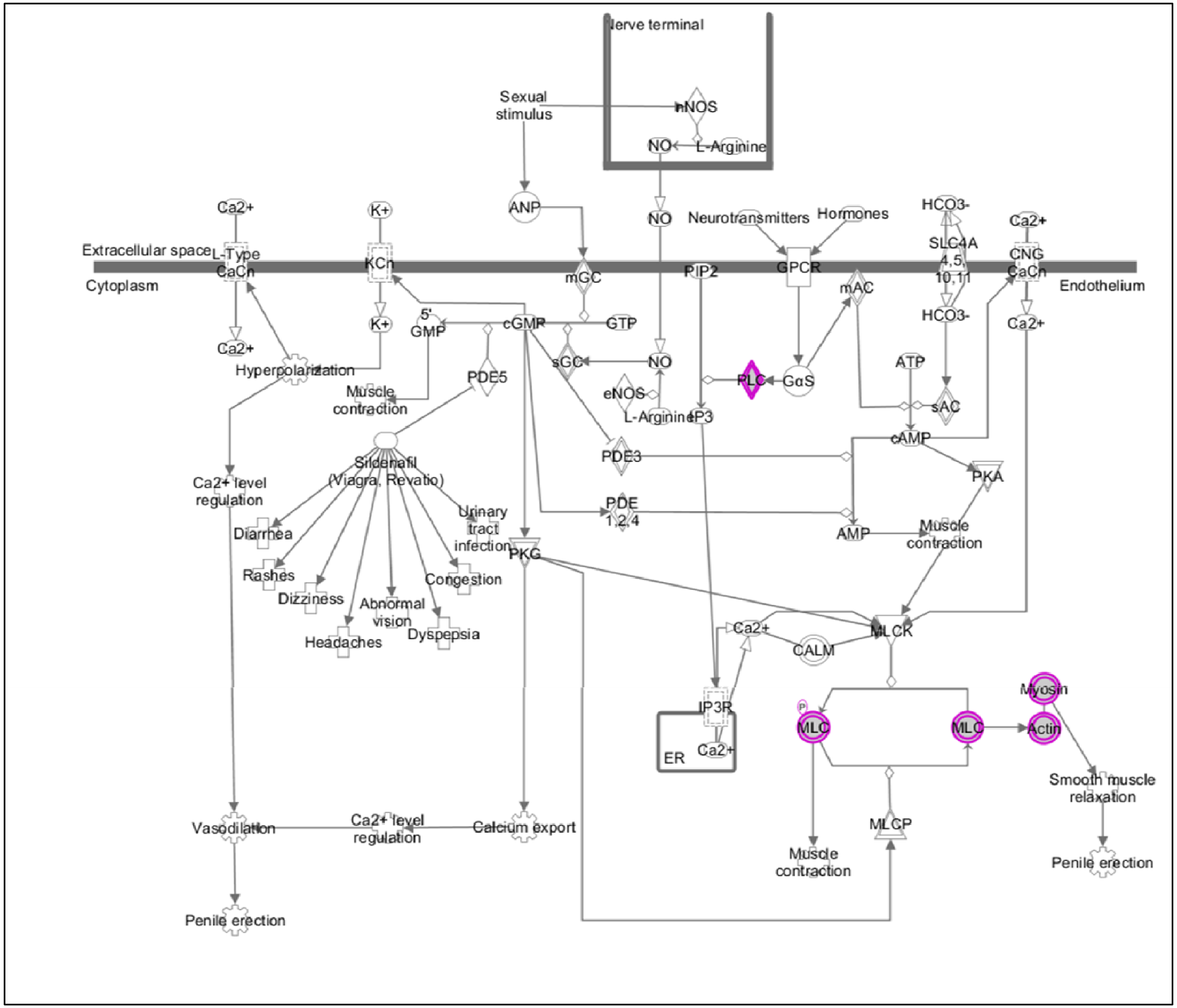

77-Valine Degradation Ⅰ

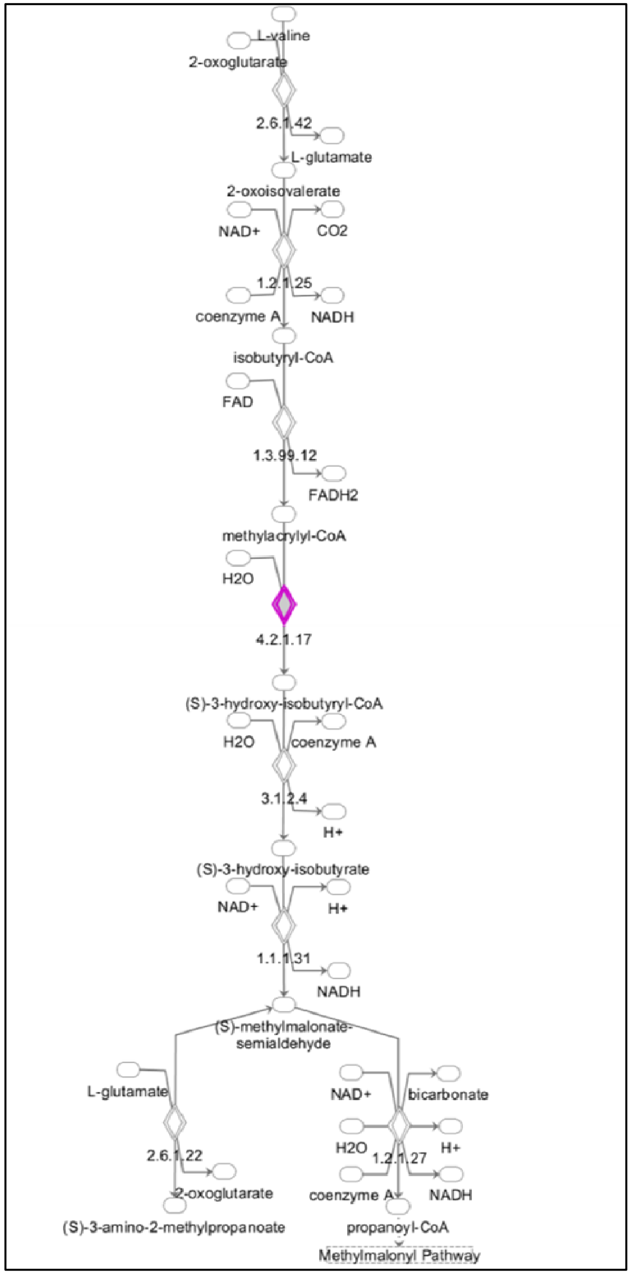

78-Relaxin Signaling

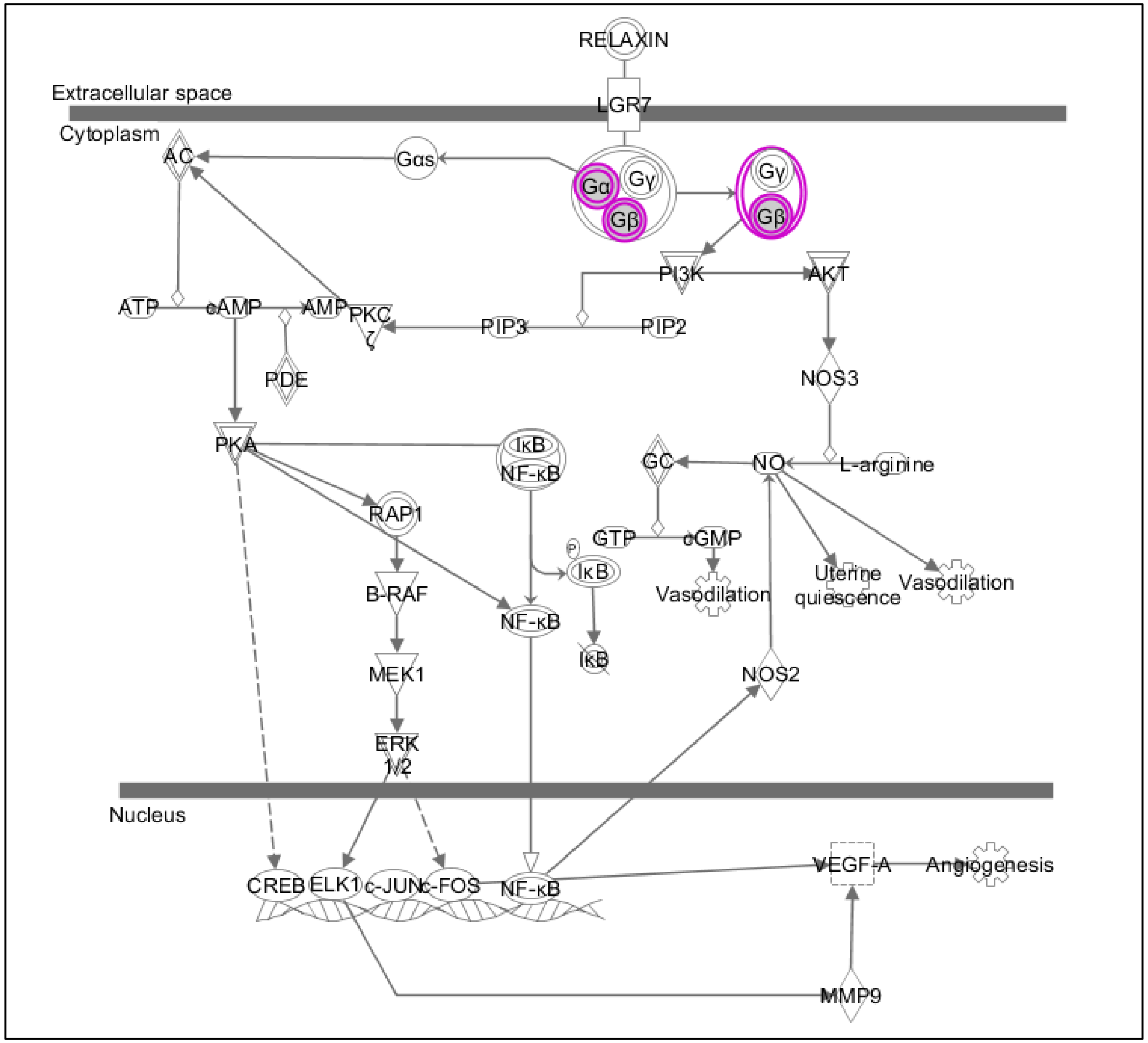

79-HIPPO Signaling

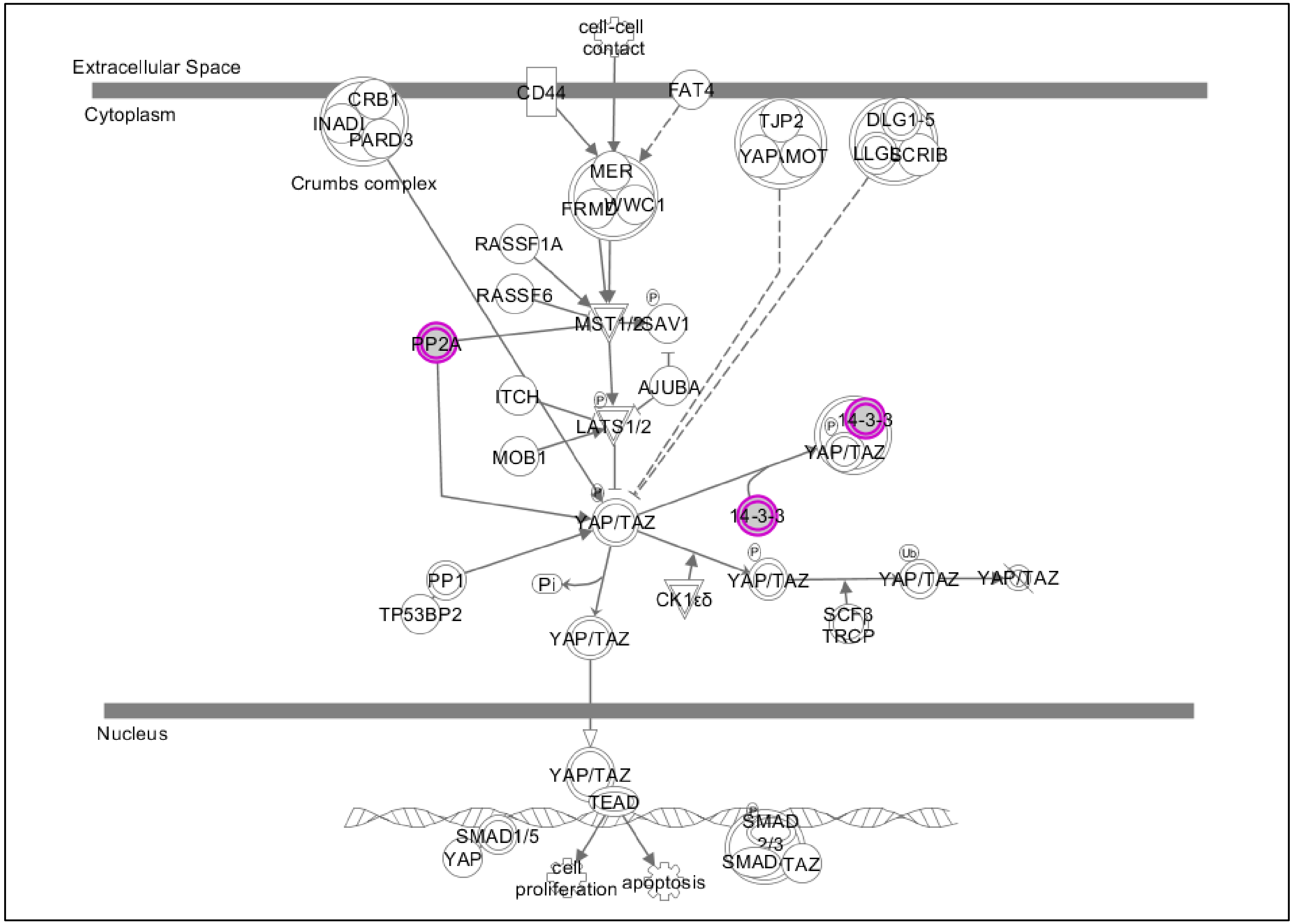

80-CTLA4 Signaling in Cytotoxic T Lymphocytes

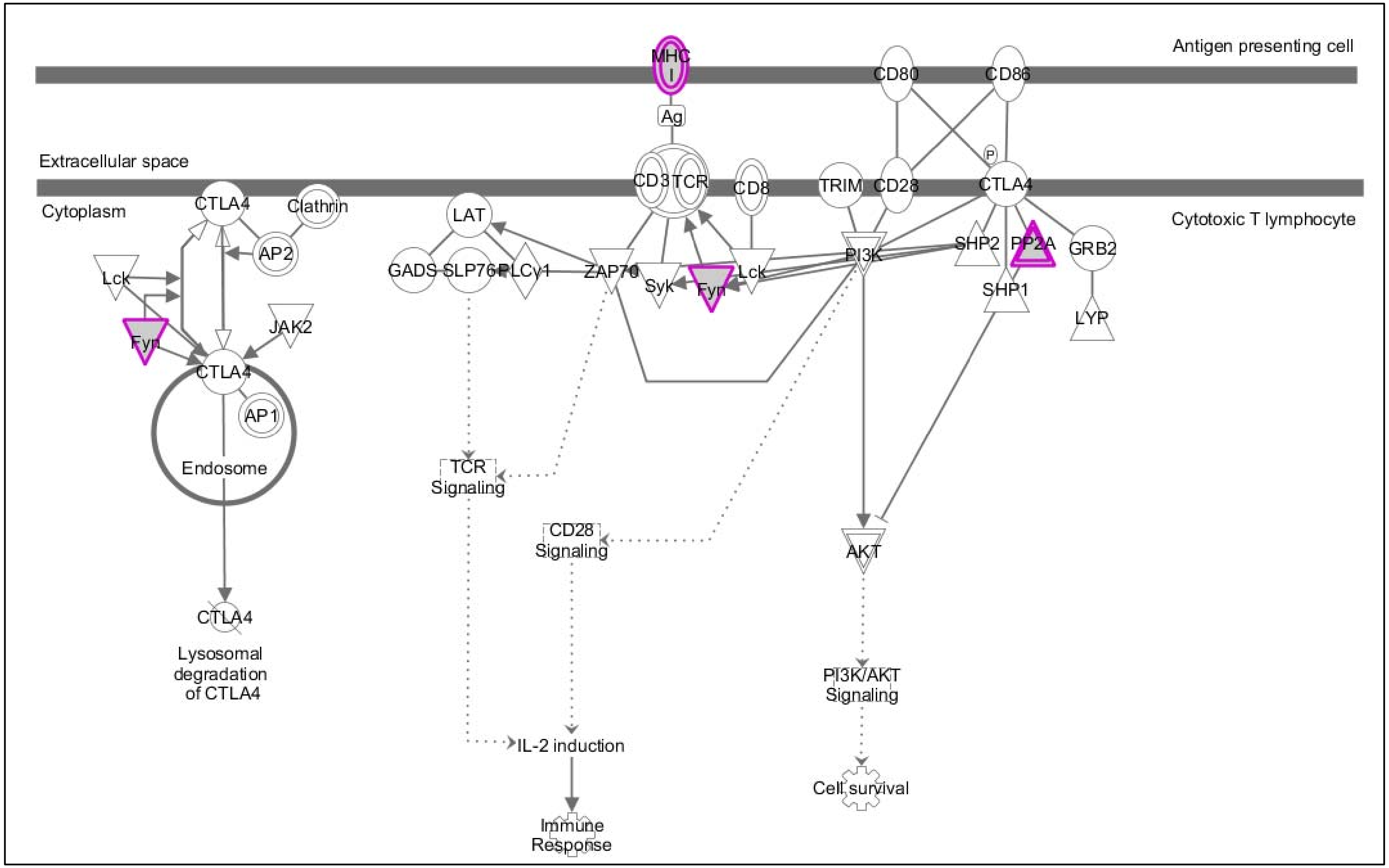

81-Crosstalk between Dendritic Cells and Natural Killer Cells

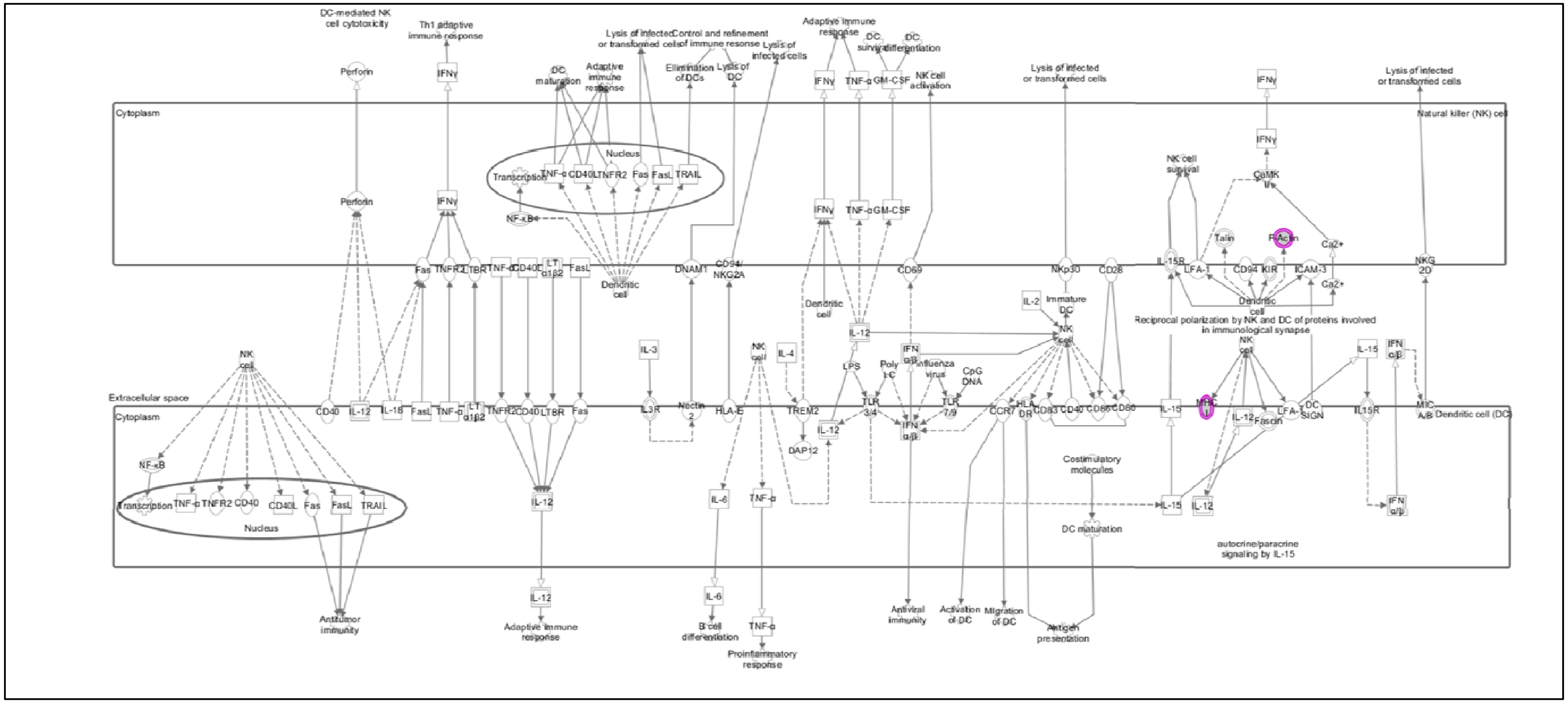

82-eNOS Signaling

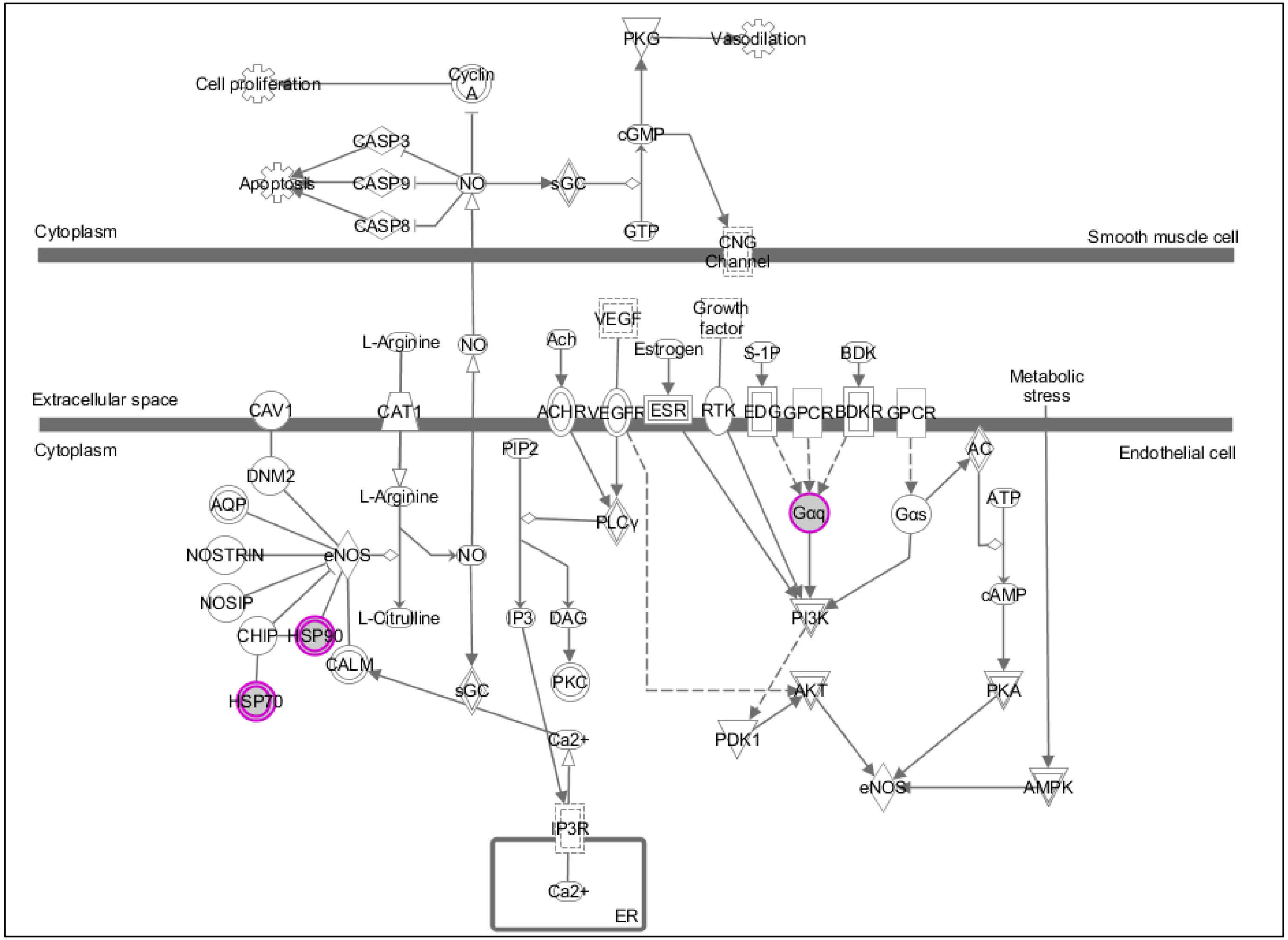

83-Pyrimidine Deoxyribonucleotides De Novo Biosynthesis Ⅰ

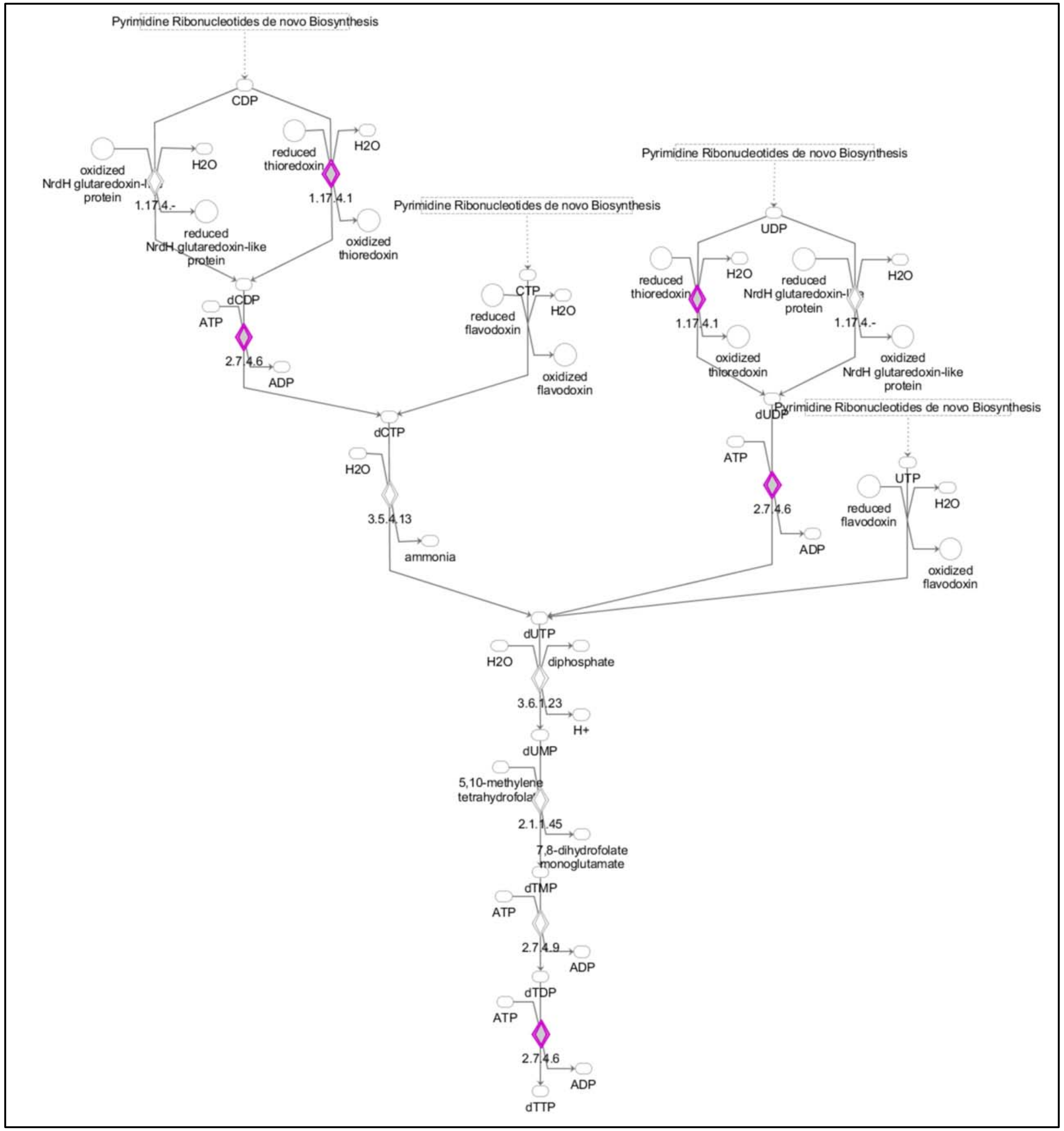

84-Regulation of Actin-based Motility by Rho

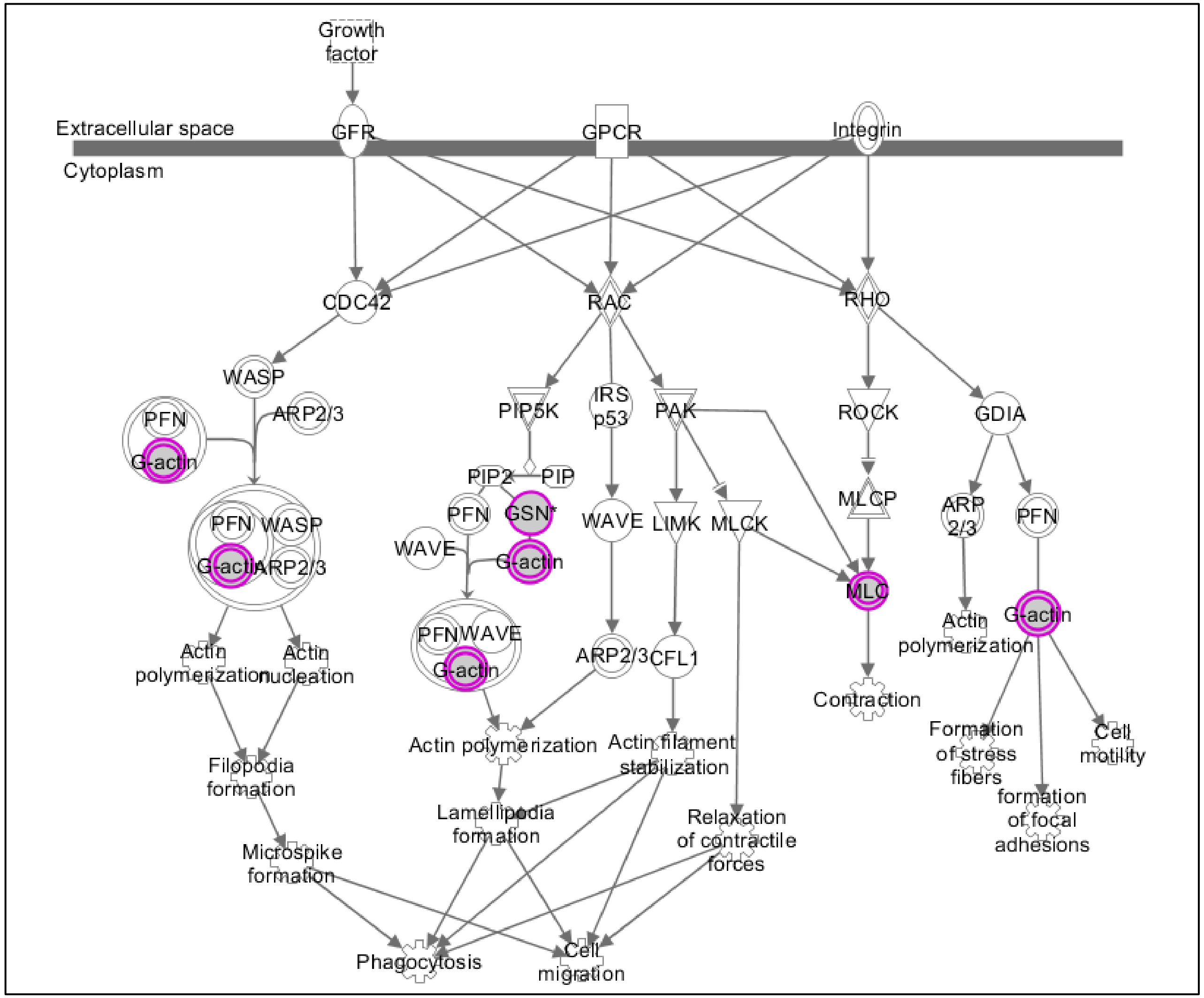

85-Mechanisms of Viral Exit from Host Cells

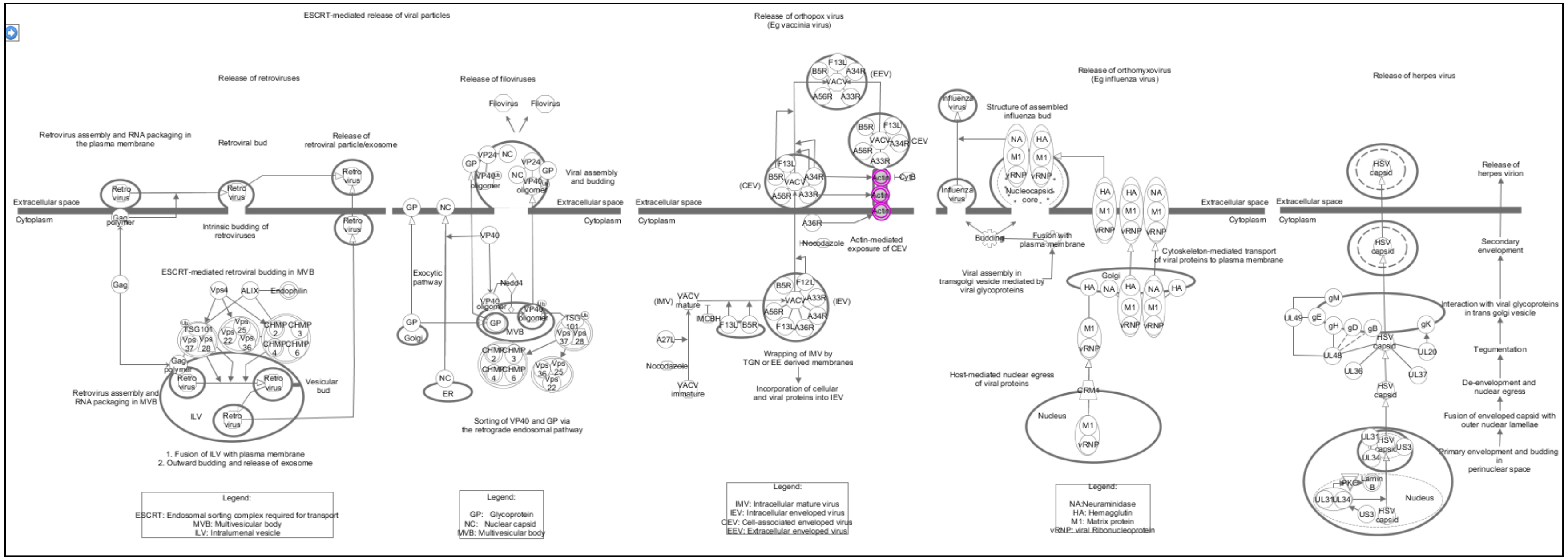

86- α -Adrenergic Signaling

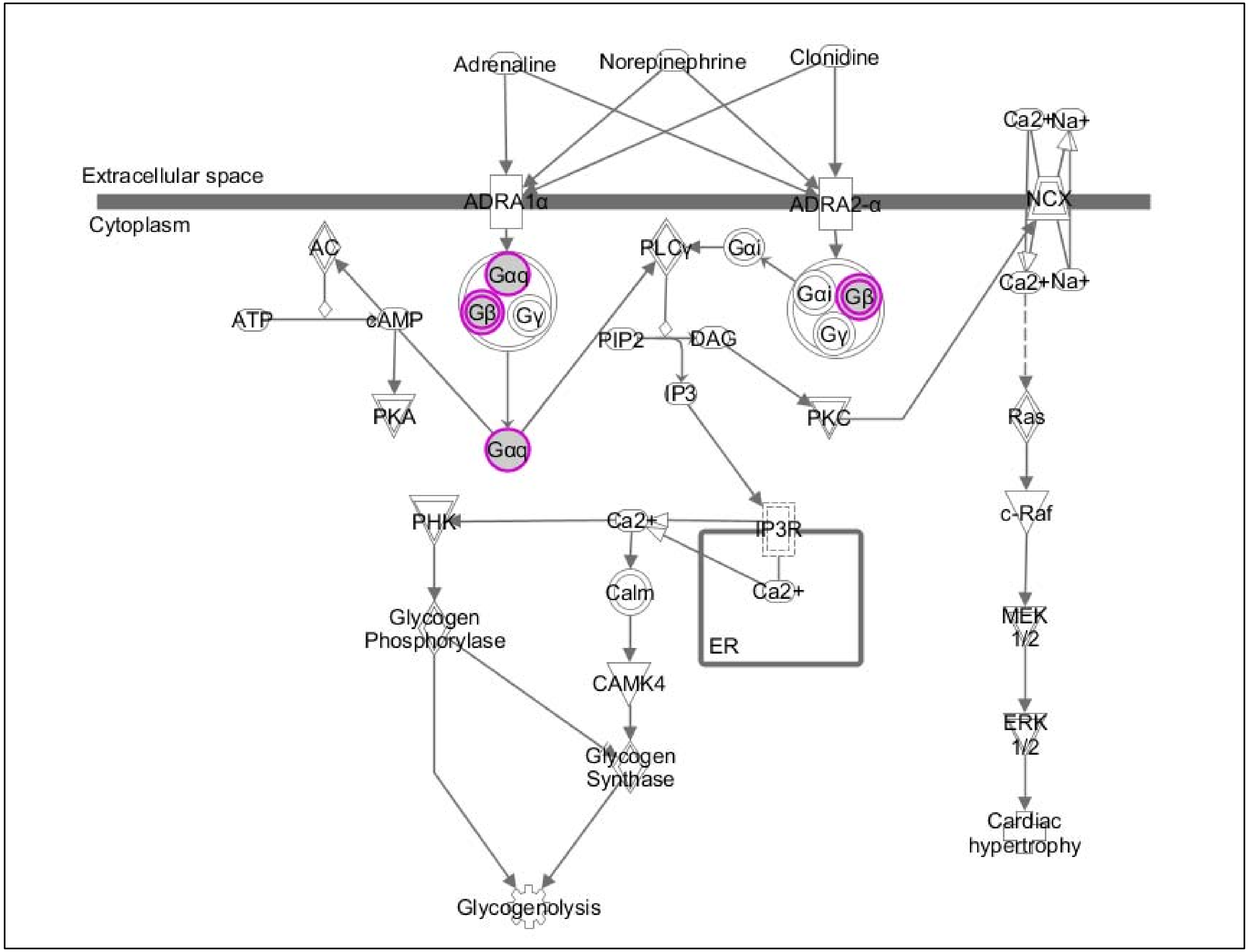

87-Ethanol Degradation Ⅱ

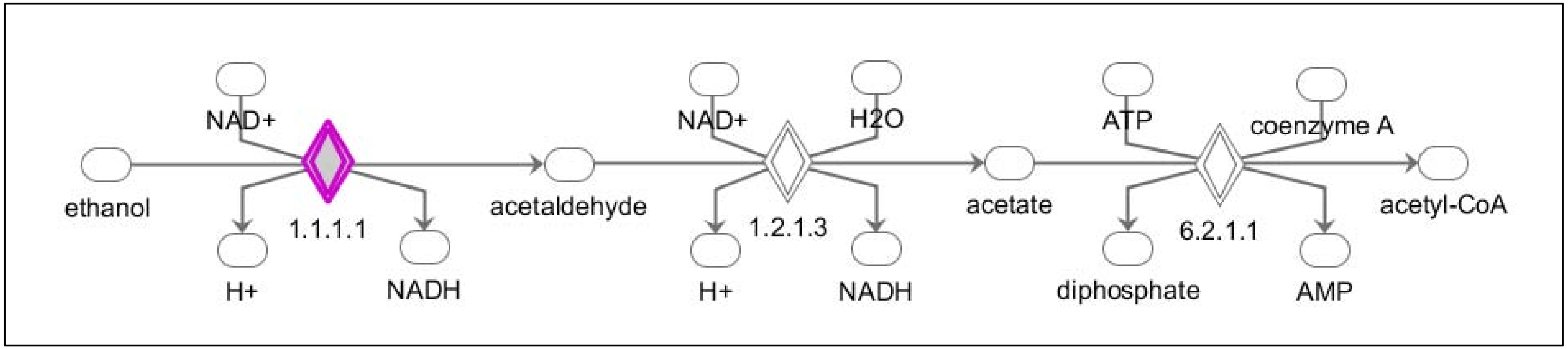

88-Fcy Receptor-mediated Phagocytosis in Macrophages and Monocytes

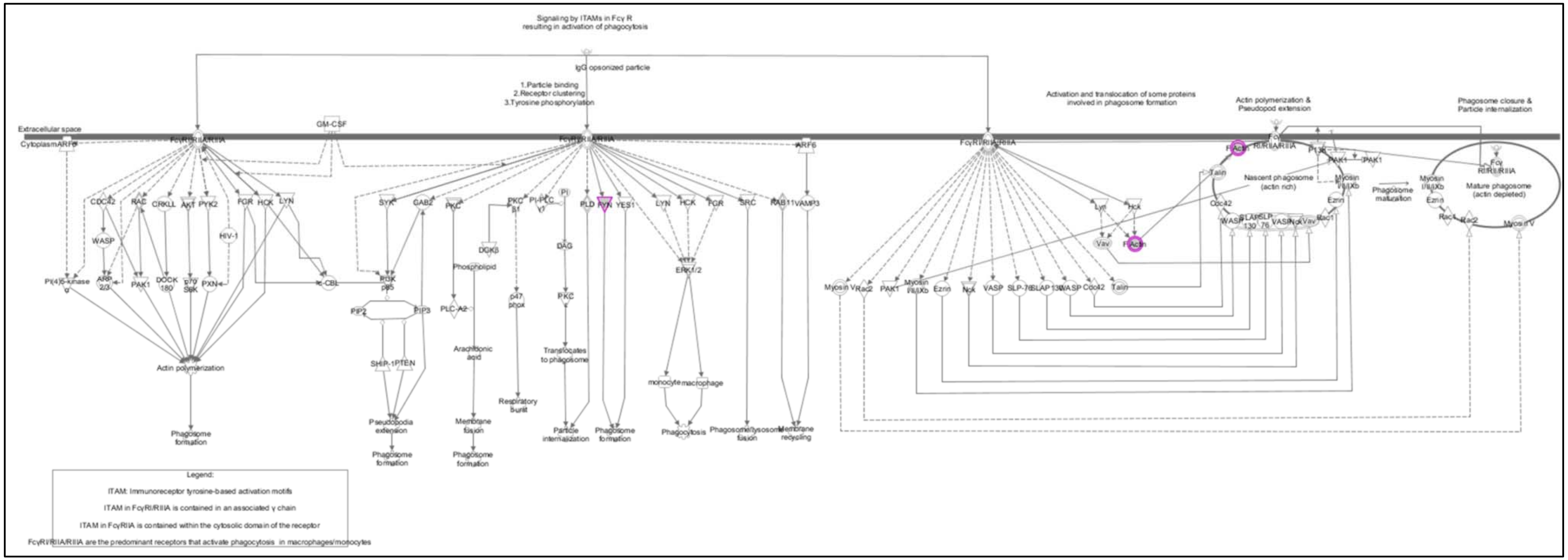

89-Thyptophan Degradation Ⅲ(Eukaryotic)

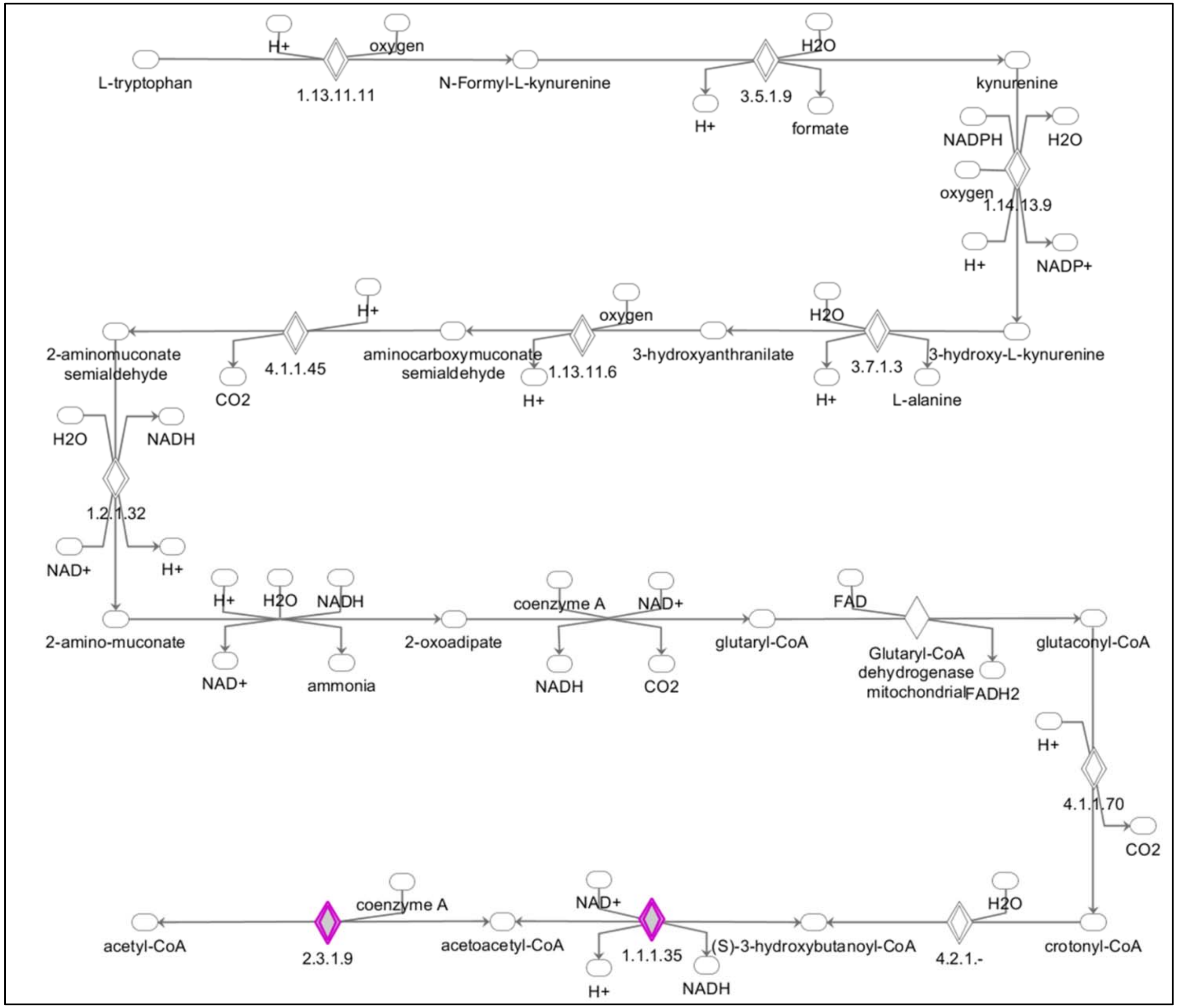

Supplement: Supplementary file 2 [file Presentation_2.zip › Supplemental materials 3.3.pdf]
