# Supplementary material for: Multiomics-Based Signaling Pathway Network Alterations in Human Non-functional Pituitary Adenomas
Source: Front Endocrinol (Lausanne). 2019 Dec 17;10:835. doi: 10.3389/fendo.2019.00835 (PMC6928143; doi:10.3389/fendo.2019.00835)
Supplement: Supplementary file 2 [file Presentation_2.zip › Supplemental materials 2.1.pdf]

Supplemental materials 2.1 Differentially expressed proteins between NFPAs and controls for IPA analysis (Dataset 2)

Fold-Change	ID	Notes	Molecules	Location	Function
2.8	P49753		ACOT2	Cytoplasm	enzyme
9. 6	P15121		AKR1B1	Cytoplasm	enzyme
-2.1	P02647		APOA1	Extracellular Space	transporter
5. 0	P06576		ATP5B	Cytoplasm	transporter
6. 5	P47756	D	CAPZB*	Cytoplasm	other
6.5	P47756-2	D	CAPZB*	Cytoplasm	other
-9.5	P13987		CD59	Plasma Membrane	other
-14.7	P12110		COL6A2	Extracellular Space	other
8.7	P14854		COI6B1	Cytoplasm	enzyme
-16.3	Q14894		CRYM	Cytoplasm	enzyme
-17.8	P47710-3		CSN1S1	Extracellular Space	other
4. 3	P07106		DBI	Cytoplasm	other
-9.3	Q9UHL4		DPP7	Cytoplasm	peptidase
4.1	P30040		ERP29	Cytoplasm	transporter
-1000.0	P00742		F10	Extracellular Space	peptidase
3.9	P06241		FYN	Plasma Membrane	kinase
9. 4	P31150		GDI1	Cytoplasm	other
-180.6	P01241	D	GH1*	Extracellular Space	growth factor
-13.3	P01241	D	GH1*	Extracellular Space	growth factor

-13.3	P01241	D	GH1*	Extracellular Space	growth factor
-20.2	P01241	D	GH1*	Extracellular Space	growth factor
-14.1	P01241	D	GH1*	Extracellular Space	growth factor
-1000.0	P01241	D	GH1*	Extracellular Space	growth factor
-6.2	P01241	D	GH1*	Extracellular Space	growth factor
-1000.0	P01241	D	GH1*	Extracellular Space	growth factor
-1000.0	P01241	D	GH1*	Extracellular Space	growth factor
-9.7	P01241	D	GH1*	Extracellular Space	growth factor
-1000.0	P01241	D	GH1*	Extracellular Space	growth factor
-1000.0	P01241	D	GH1*	Extracellular Space	growth factor
-1000.0	P01241	D	GH1*	Extracellular Space	growth factor
-1000.0	P01241	D	GH1*	Extracellular Space	growth factor
-1000.0	P01241	D	GH1*	Extracellular Space	growth factor
-13.4	P01241	D	GH1*	Extracellular Space	growth factor
-42.0	P01241	D	GH1*	Extracellular Space	growth factor
-1000.0	P01241	D	GH1*	Extracellular Space	growth factor
-14.1	P01242		GH2	Extracellular Space	other
10. 2	Q04760	D	GL01*	Cytoplasm	enzyme
8. 3	Q04760	D	GL01*	Cytoplasm	enzyme
9.5	P09471	D	GNA01*	Plasma Membrane	enzyme
9. 5	P29777	D	GNA01*	Plasma Membrane	enzyme

-14.4	P36969		GPX4	Cytoplasm	enzyme
4.2	P28161		GSTM2	Cytoplasm	enzyme
-11.0	P14625		HSP90B1	Cytoplasm	other
-4.9	P04792		HSPB1	Cytoplasm	other
-1000.0	Q9UJY1		HSPB8	Cytoplasm	kinase
8. 7	O75874		IDH1	Cytoplasm	enzyme
-1000.0	P24592		IGFBP6	Extracellular Space	other
-1000.0	P40933-2		IL15	Extracellular Space	cytokine
-28.8	P03779		KRT16	Cytoplasm	other
-1000.0	Q86U44		METTL3	Nucleus	enzyme
3. 1	Q99542		MMP19	Extracellular Space	peptidase
-7.4	P12524		MYCL	Nucleus	transcription regulator
5. 2	O00217		NDUFS8	Cytoplasm	enzyme
-38.0	P20774		OGN	Extracellular	growth factor
5. 1	Q08752		PPID	Cytoplasm	enzyme
-46.2	P01236	D	PRL*	Extracellular Space	cytokine
-4.9	P01236	D	PRL*	Extracellular Space	cytokine
-26.2	P01236	D	PRL*	Extracellular Space	cytokine
-1000.0	P01236	D	PRL*	Extracellular Space	cytokine
-1000.0	P01236	D	PRL*	Extracellular Space	cytokine
-1000.0	P01236	D	PRL*	Extracellular Space	cytokine

-6.6	O76038		SCGH	Cytoplasm	other
4.9	Q9UI15		TAGLN3	Extracellular	other
-1000.0	P21980		TGM2	Cytoplasm	enzyme
10.6	Q8IWU9		TPH2	Plasma Membrane	enzyme
-11.4	014530		TXNDC9	Cytoplasm	other
5. 5	P08670		VIM	Cytoplasm	other
-1000.0	P27348		YWHAQ	Cytoplasm	other
7.3	Q14584		ZNF266	Nucleus	other
-8.2	Q00007	unmapped			
-32.5	P01620	unmapped			
-3.1	P18988	unmapped			
-1000.0	gi1066765	unmapped			
-1000.0	P18988	unmapped			
-20.2	P01968	unmapped			
5.0	Q14259	unmapped			

Note: D = duplicate
