# Supplementary material for: Multiomics-Based Signaling Pathway Network Alterations in Human Non-functional Pituitary Adenomas
Source: Front Endocrinol (Lausanne). 2019 Dec 17;10:835. doi: 10.3389/fendo.2019.00835 (PMC6928143; doi:10.3389/fendo.2019.00835)
Supplement: Supplementary file 3 [file Presentation_3.zip › Supplemental materials 4.1.pdf]

Supplemental materials 4.1 Nitroproteins and nitroproein-binding proteins from a NFPA for IPA analysis (Datatset 4)

ID	Notes	Molecules	Location	Function
Q15027		ACAP1	Plasaa Membrane	other
Q13017		ARHGAP5	Cytoplasa	enzyme
Q9C0E4		GRIP2	Plasaa Meabrane	other
Q9UHA7		IL36A	Extracellular Space	cytokine
O43187		IRAK2	Plasaa Membrane	kinase
P59901		LILRA4	Plasaa Membrane	other
P31321		PRKAR1B	Cytoplasa	kinase
P25787		PSMA2	Cytoplasa	peptidase
Q8IUC4		RHPN2	Cytoplasm	other
O95470		SGPL1	Cytoplasm	enzyme
UBB		UBB	Cytoplasa	enzyme
UBC		UBC	Cytoplasa	enzyme
O94892		ZNF432	Hucleus	other
