# Supplementary material for: Multiomics-Based Signaling Pathway Network Alterations in Human Non-functional Pituitary Adenomas
Source: Front Endocrinol (Lausanne). 2019 Dec 17;10:835. doi: 10.3389/fendo.2019.00835 (PMC6928143; doi:10.3389/fendo.2019.00835)

Supplemental materials 4.3
Statistically significant canonical pathways derived from nitroproteins and nitroproein-binding proteins from a NFPA for IPA analysis (Datatset 4)

1. Toll-like Receptor Signaling
2. Hepatic Cholestasis
3. Protein Ubiquitination Pathway
4. Renal Cell Carcinoma Signaling
5. GABA Receptor Signaling
6. IL-1 Signaling
7. Hereditary Breast Cancer Signaling
8. p38 MAPK Signaling
9. RhoA Signaling
10. Wnt/ β -catenin Signaling
11. Clathrin-mediated Endocytosis Signaling
12. Huntington’s Disease Signaling
13. Role of Macrophage, Fibroblasts and Endothelial Cells in Rheumatoid Arthritis
14. Sonic Hedgehog Signaling
15. Netrin Signaling
16. Role of Hypercytokinemia/hyperchemokinemia in the Pathogenesis of Influenza
17. Neuroprotective Role of THOP1 in Alzheimer’s Disease
18. iNOS Signaling
19. Gaft-versus-Host Disease Signaling
20. Amyloid Processing
21. Role of Cytokines in Mediating Communication between Immune Cells
22. Phototransduction Pathway
23. PXR_RXR activation
24. IL-10 signaling
25. Melatonin signaling
26. BMP signaling pathway
27. Leptin signaling in obesity
28. Melanocyte development and pigmentation signaling
29. Altered T cell and B cell signaling in Rheumatoid arthritis

Dataset 4-Canonical Pathway Chart

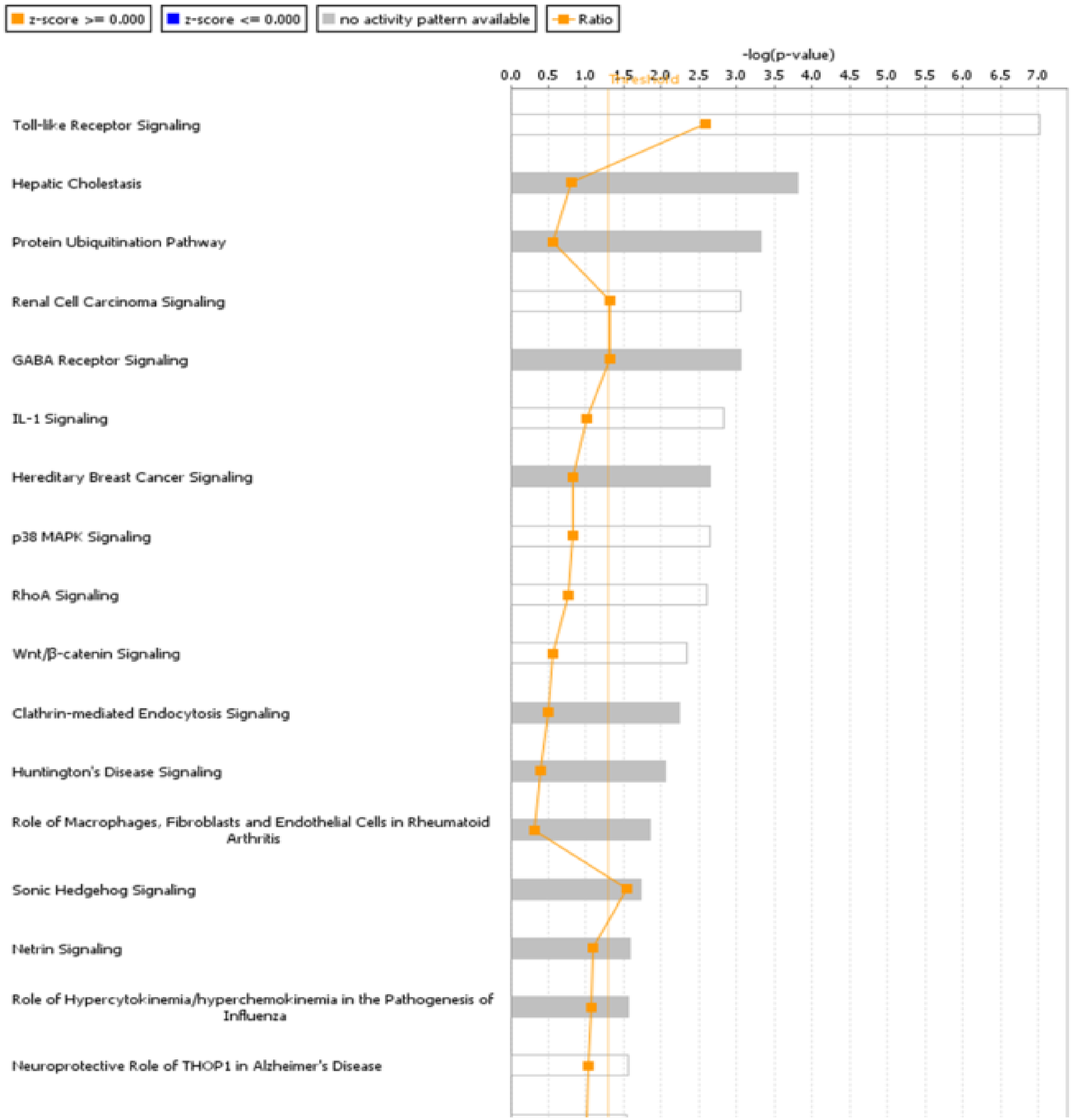

1-Toll-like Receptor Signaling

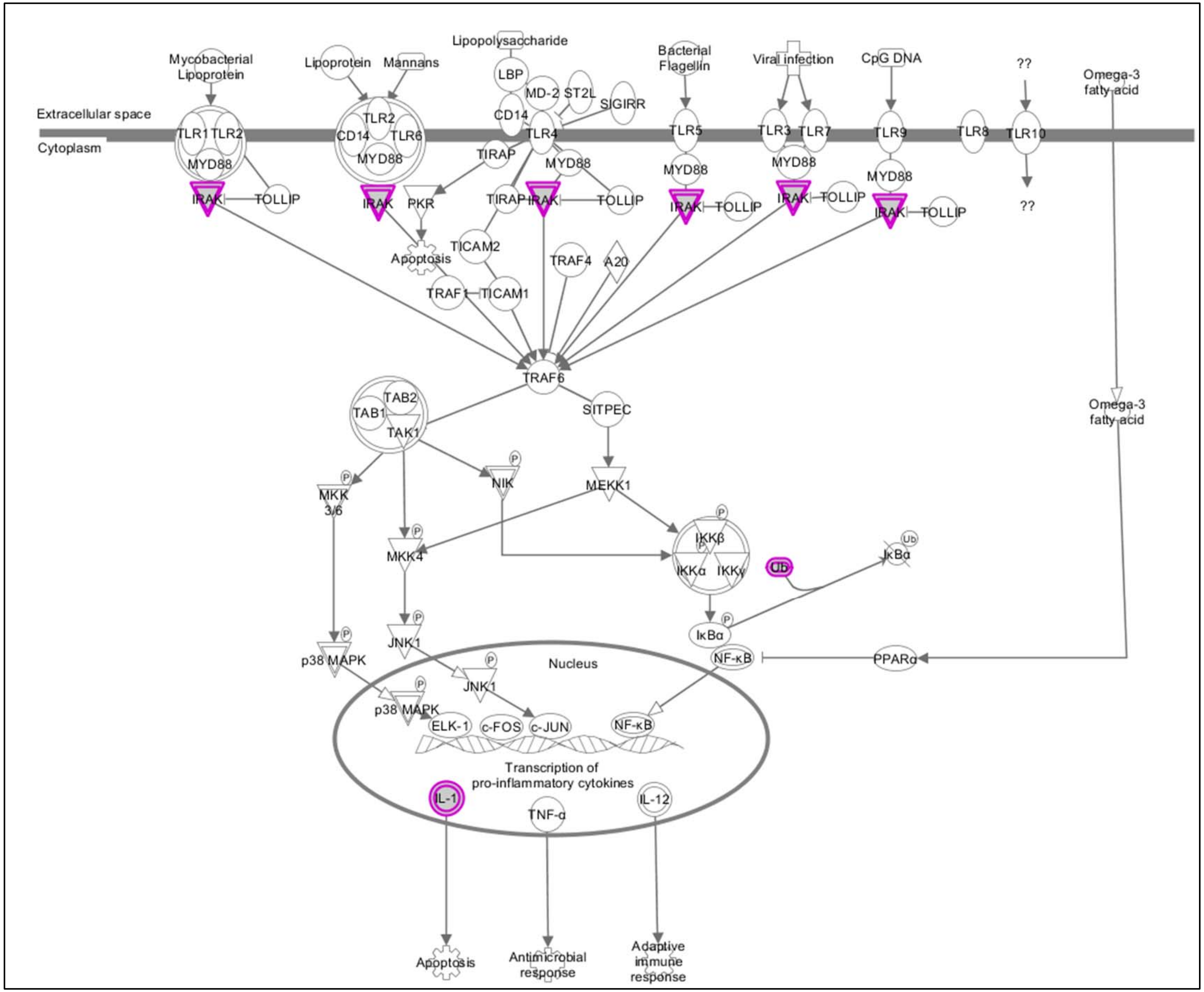

2-Hepatic Cholestasis

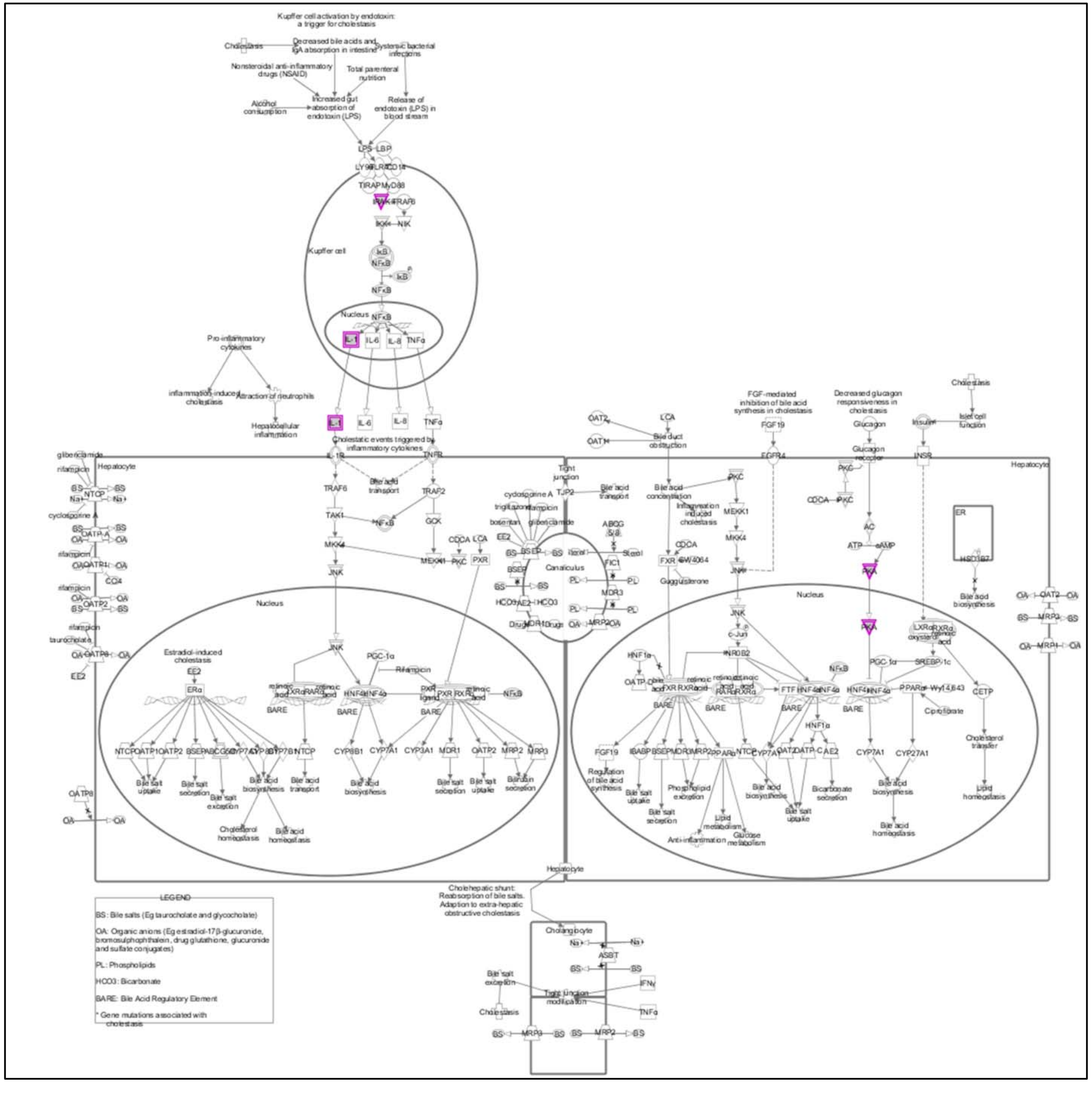

3-Protein Ubiquitination Pathway

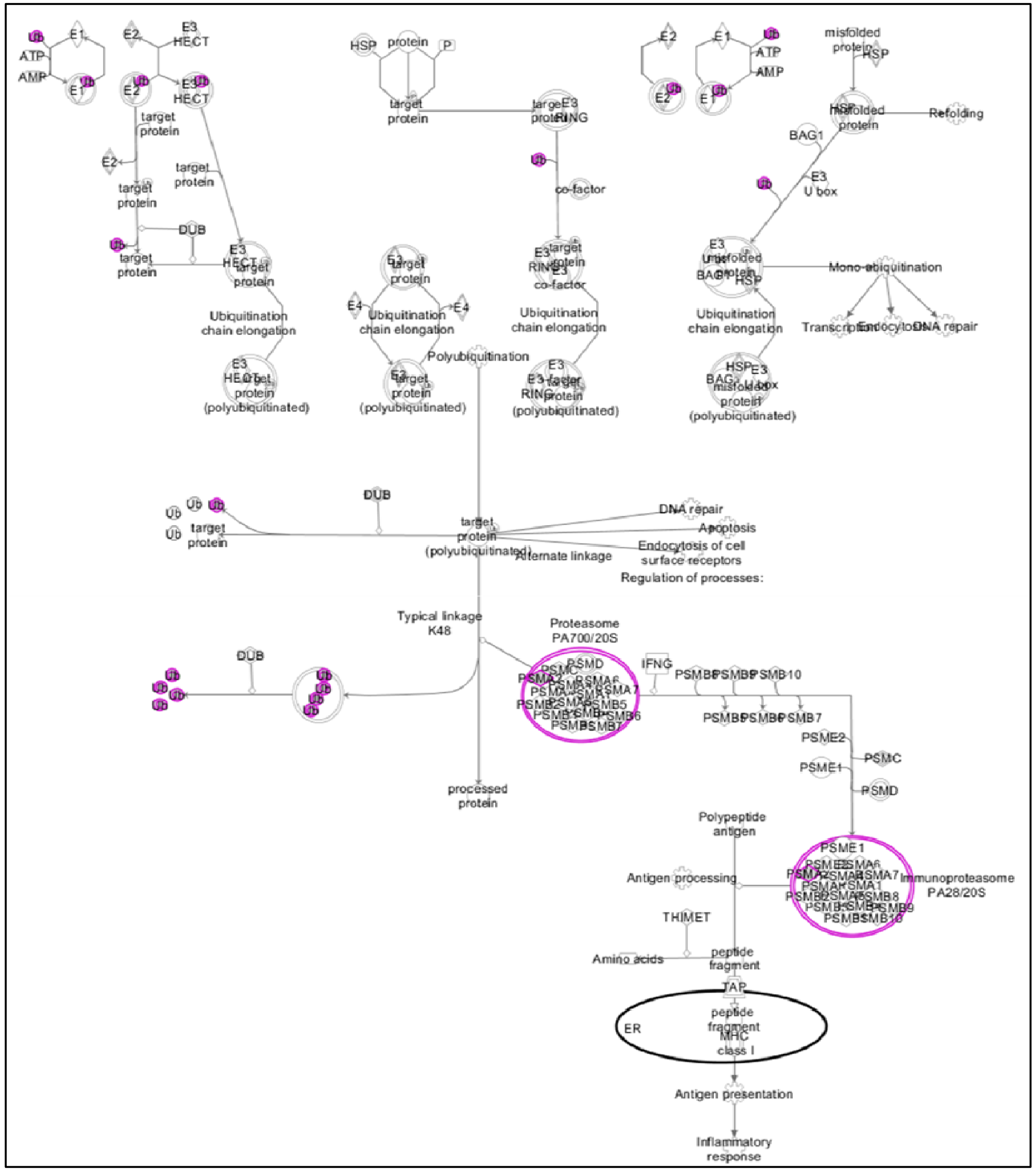

4-Renal Cell Carcinoma Signaling

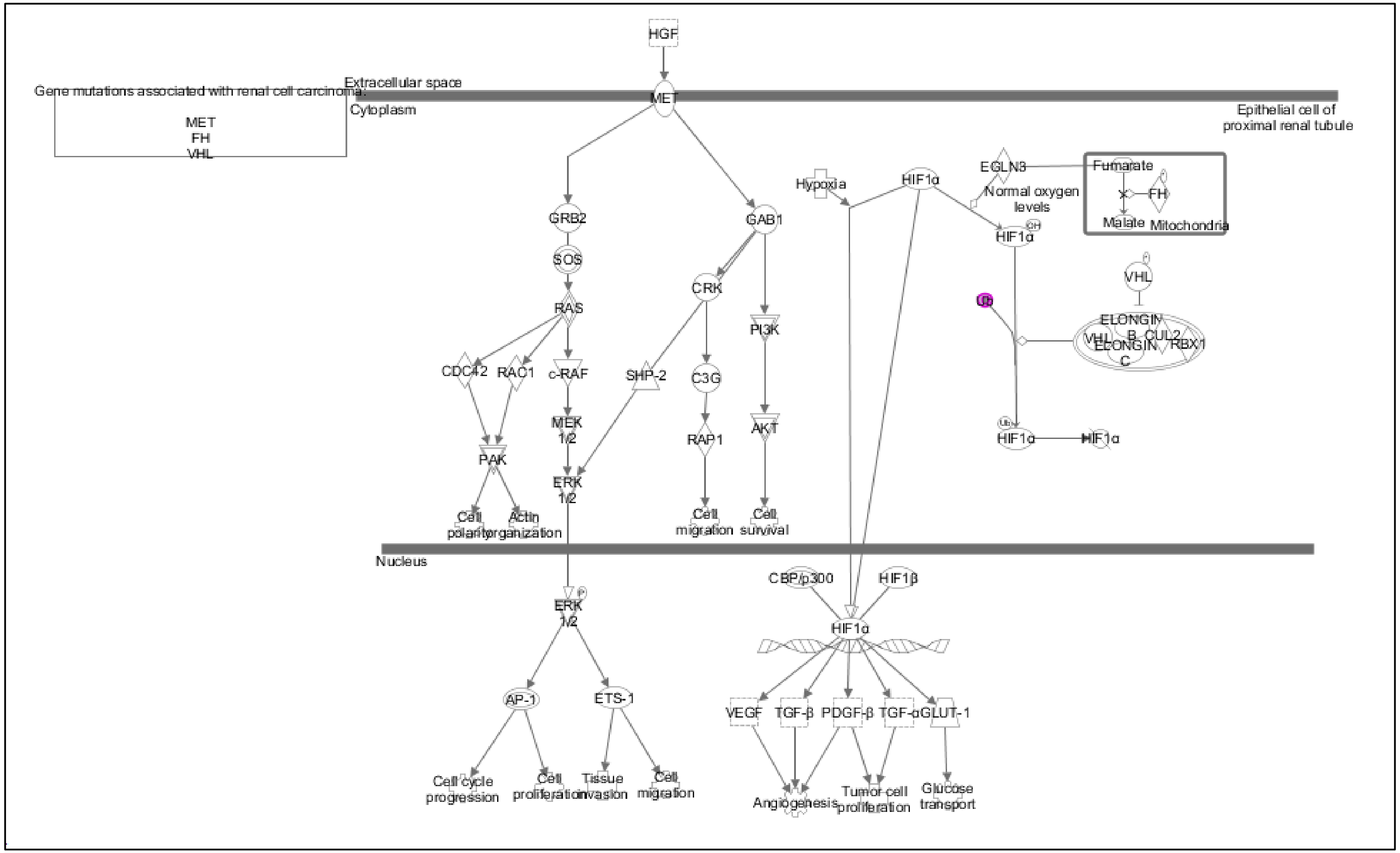

5-GABA Receptor Signaling

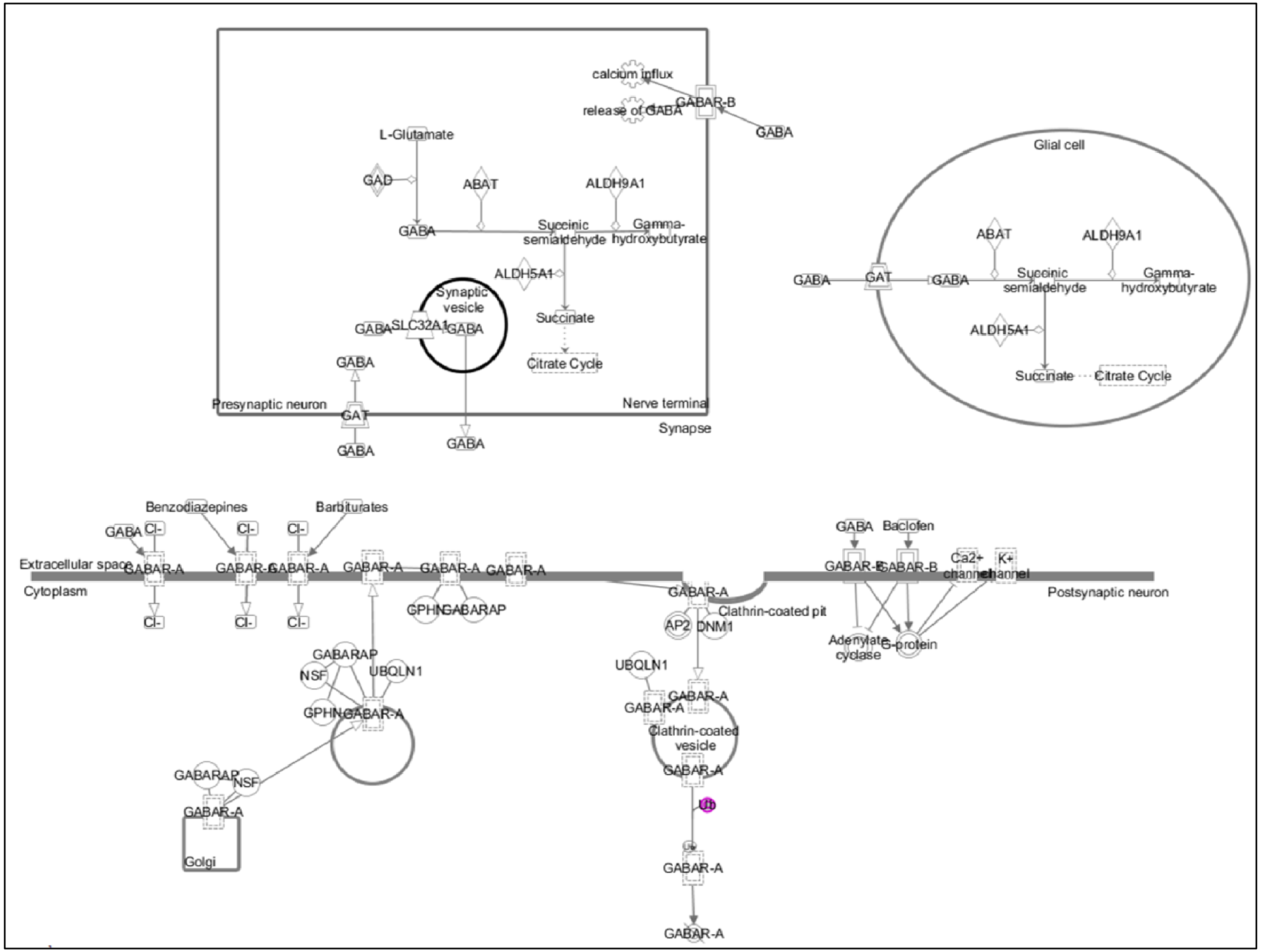

6-IL-1 Signaling

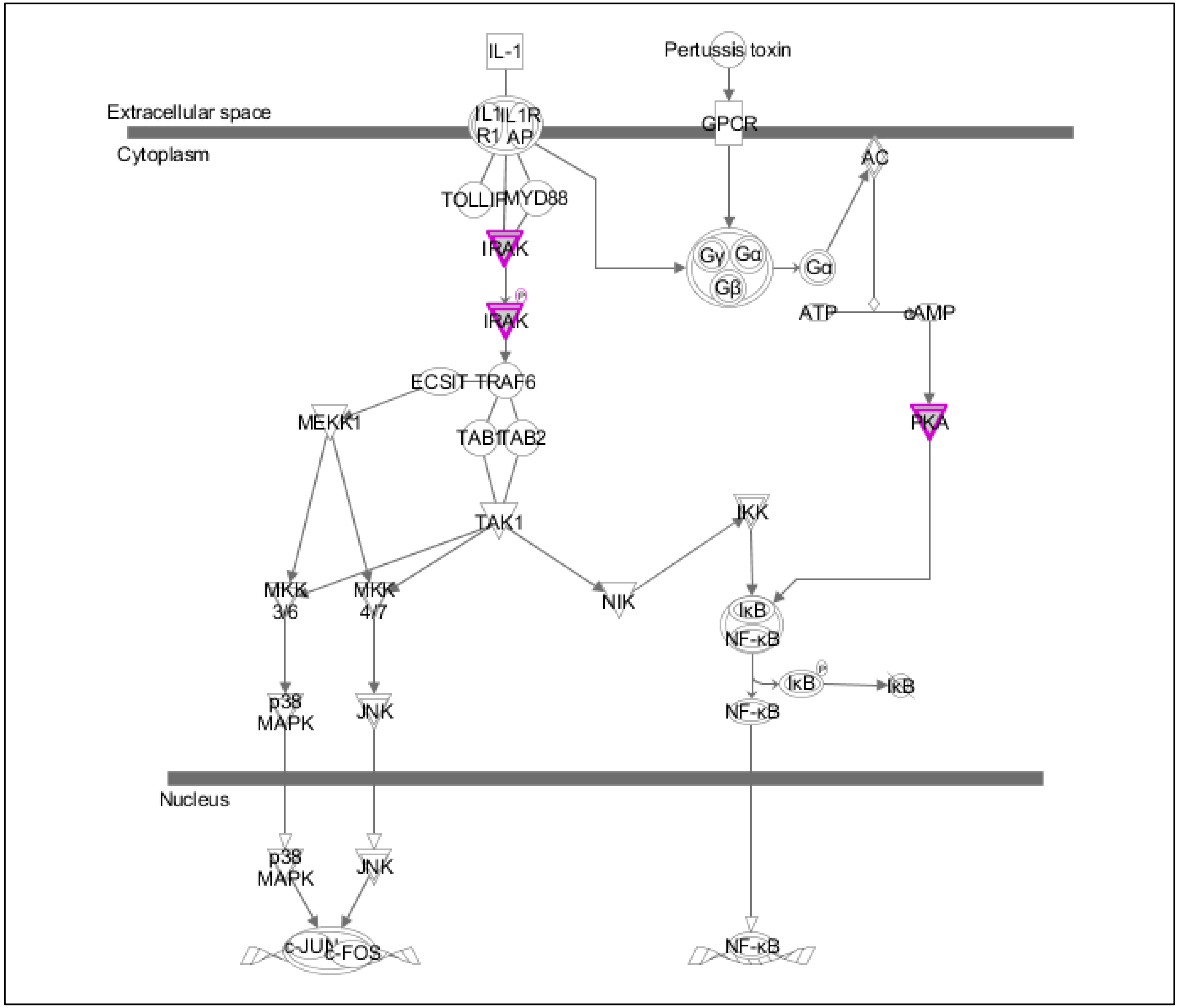

7-Hereditary Breast Cancer Signaling

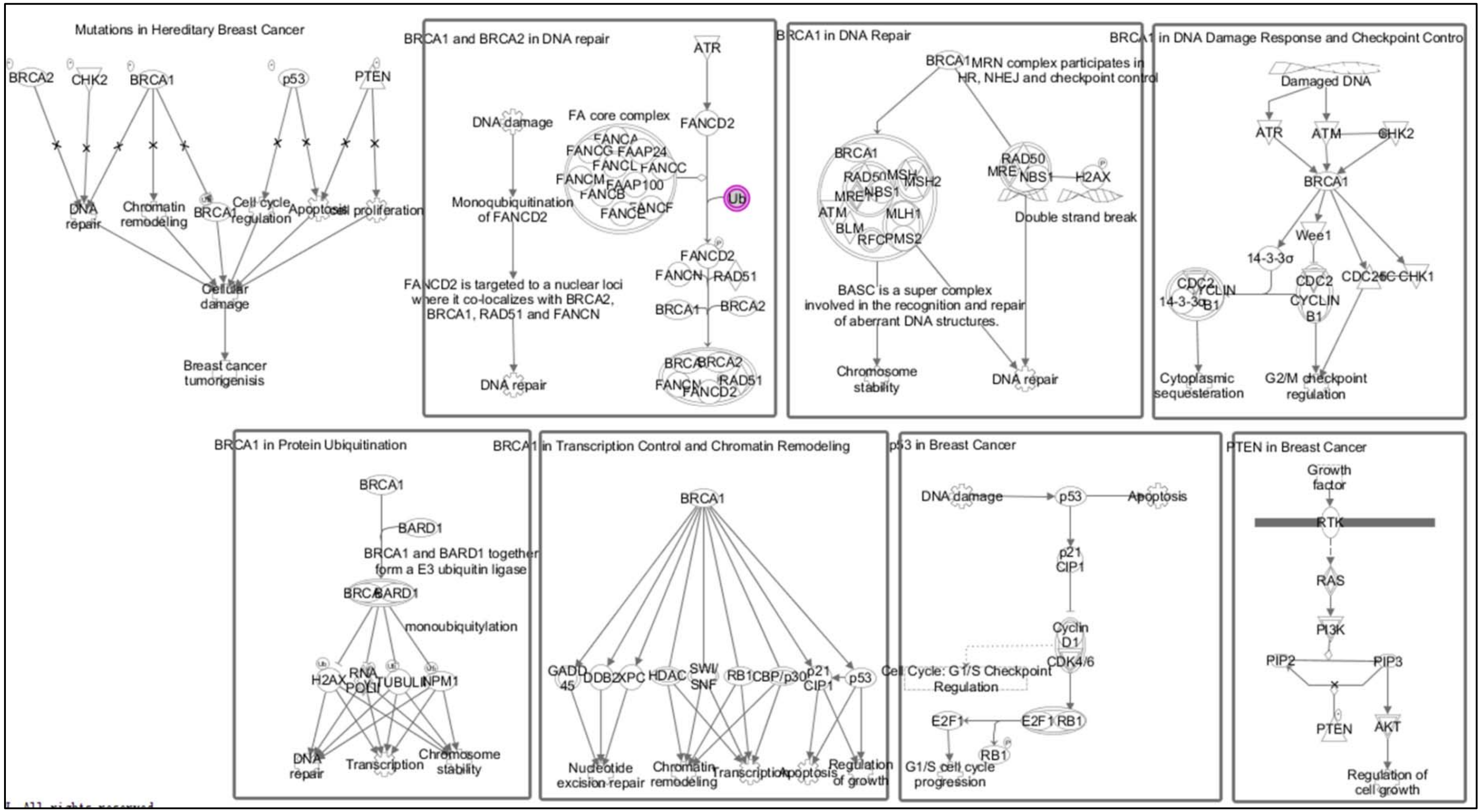

8-p38 MAPK Signaling

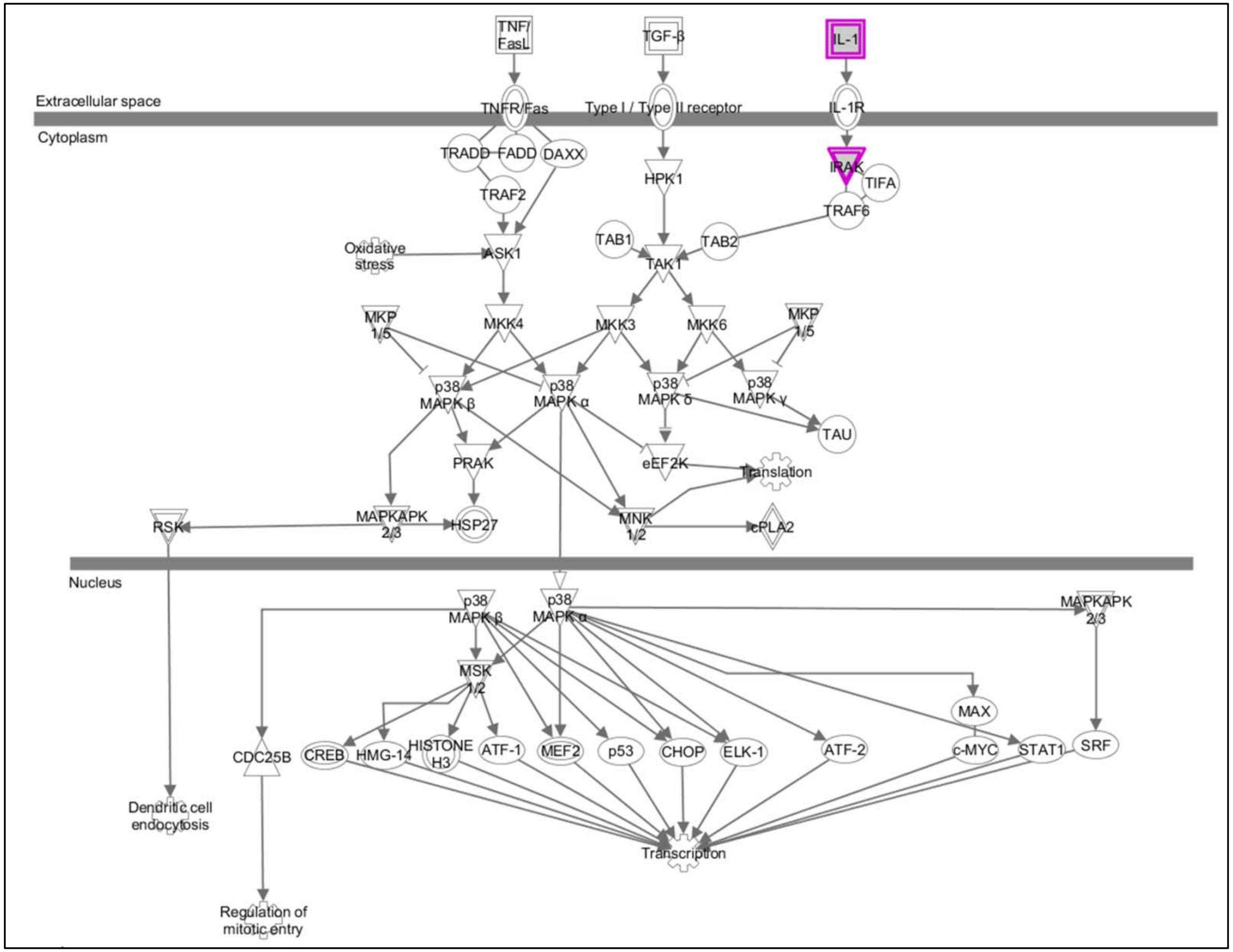

9-RhoA Signaling

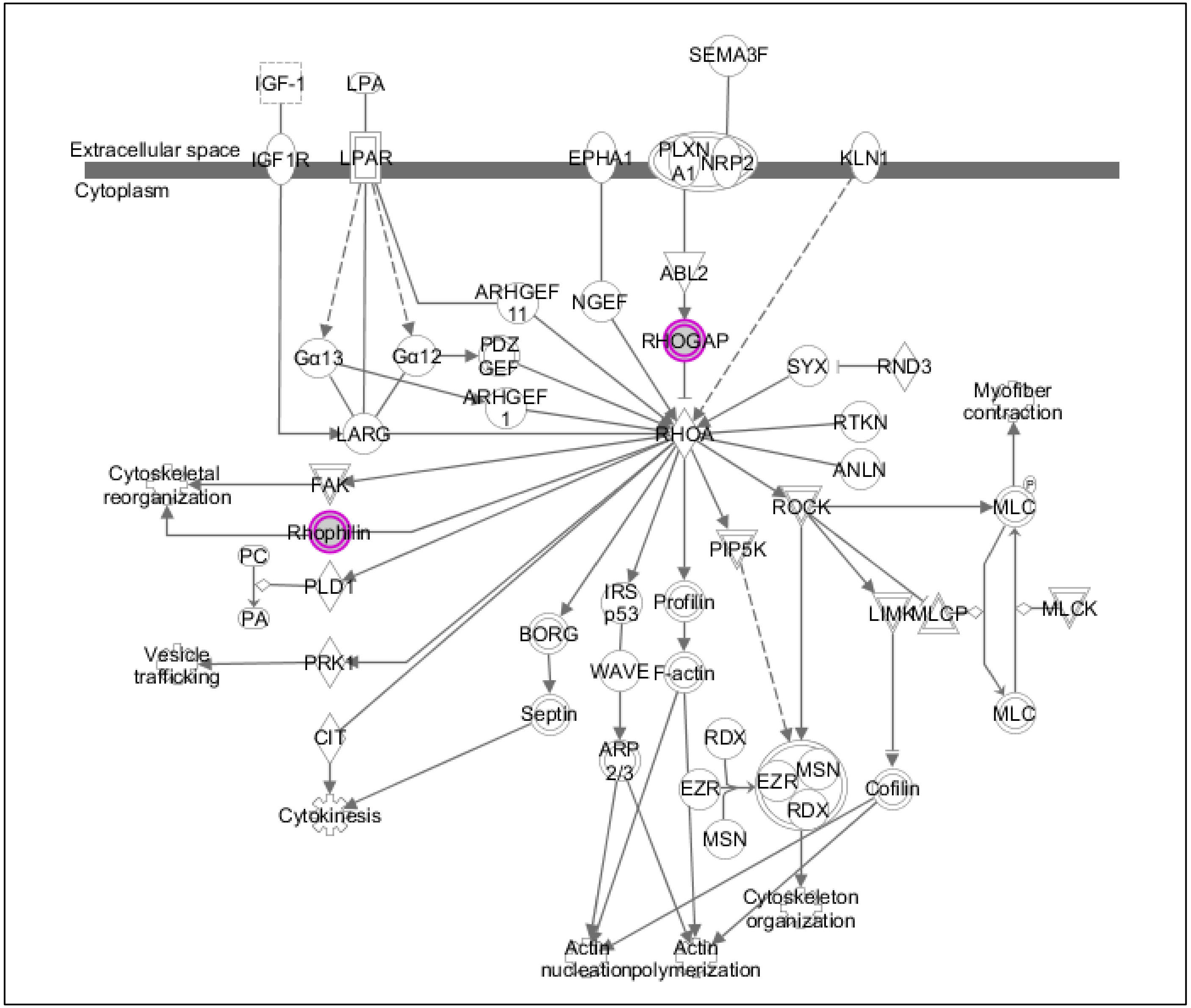

10-Wnt/ β -catenin Signaling

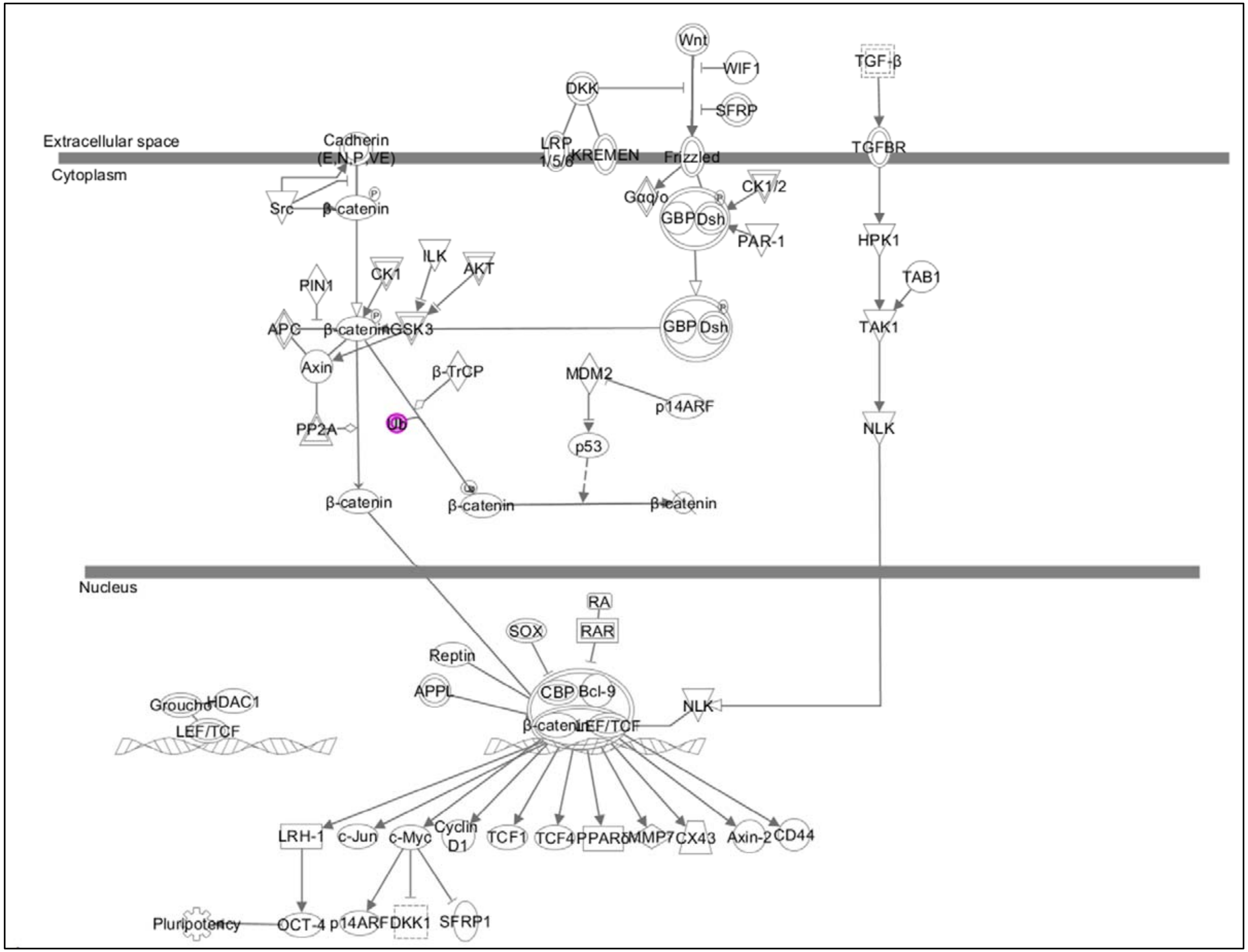

11-Clathrin-mediated Endocytosis Signaling

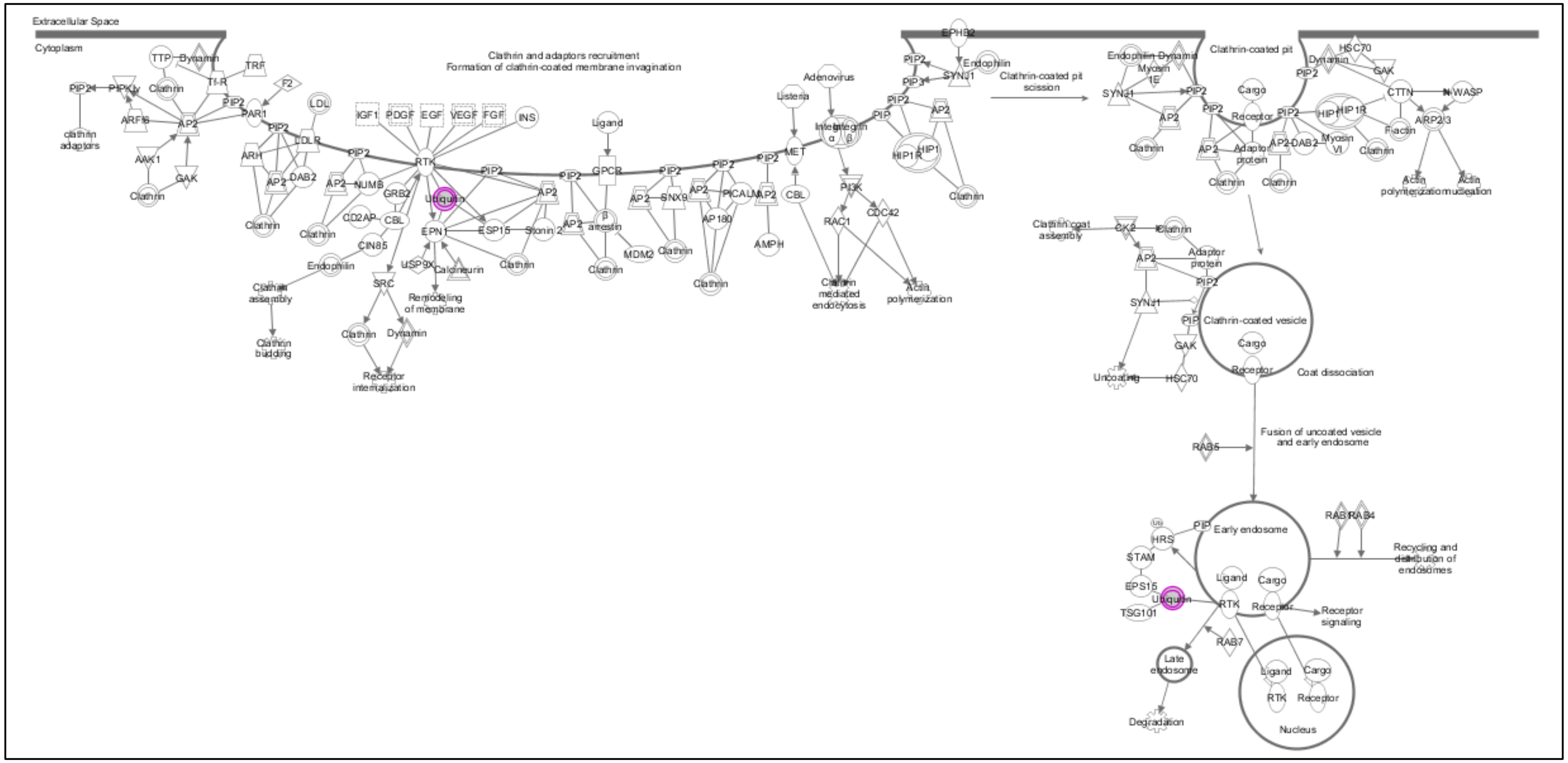

12-Huntington's Disease Signaling

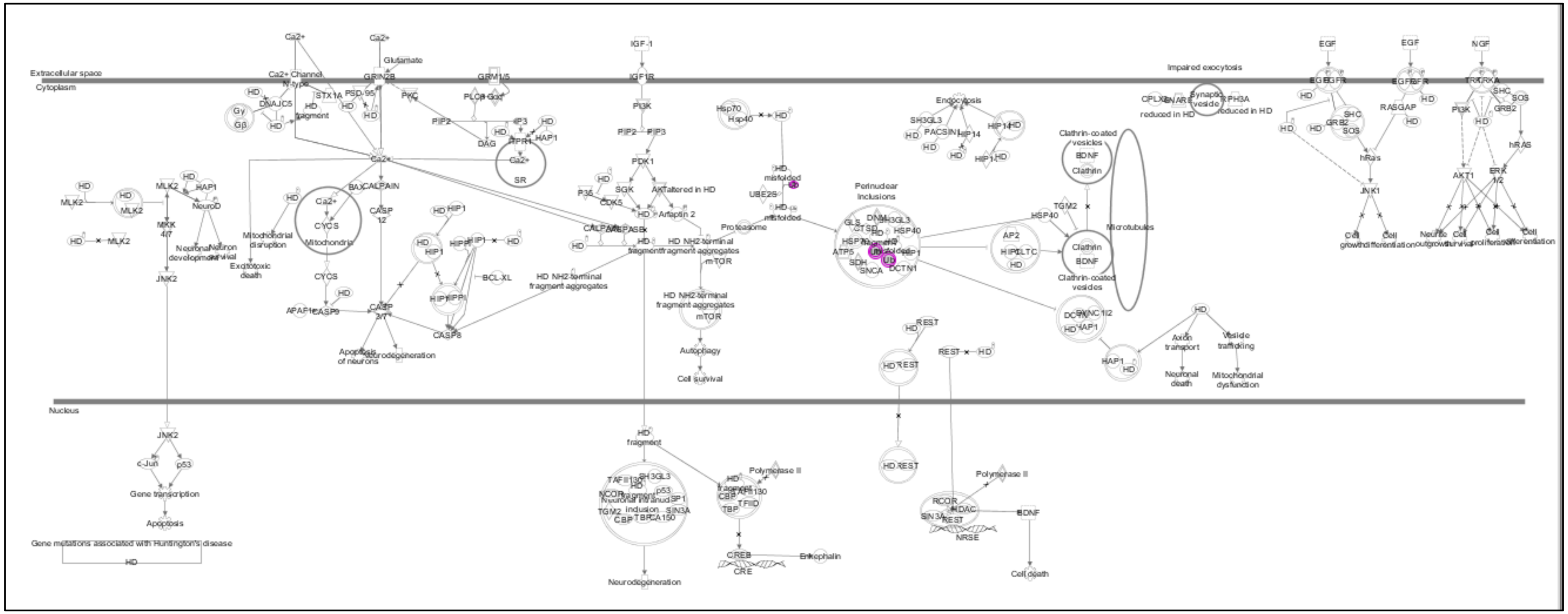

13-Role of Macrophage, Fibroblasts and Endothelial Cells inRheumatoid Arthritis

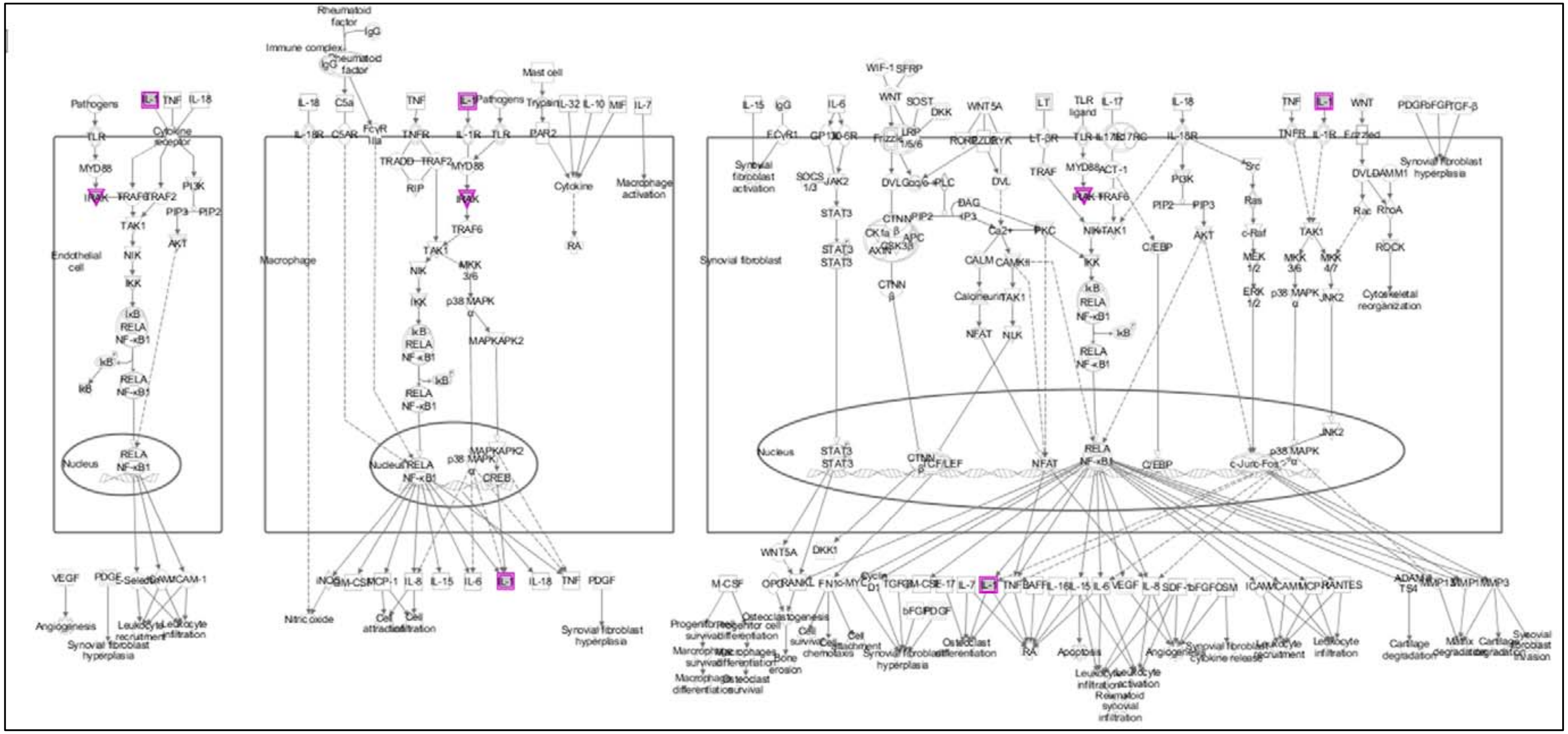

14-Sonic Hedgehog Signaling

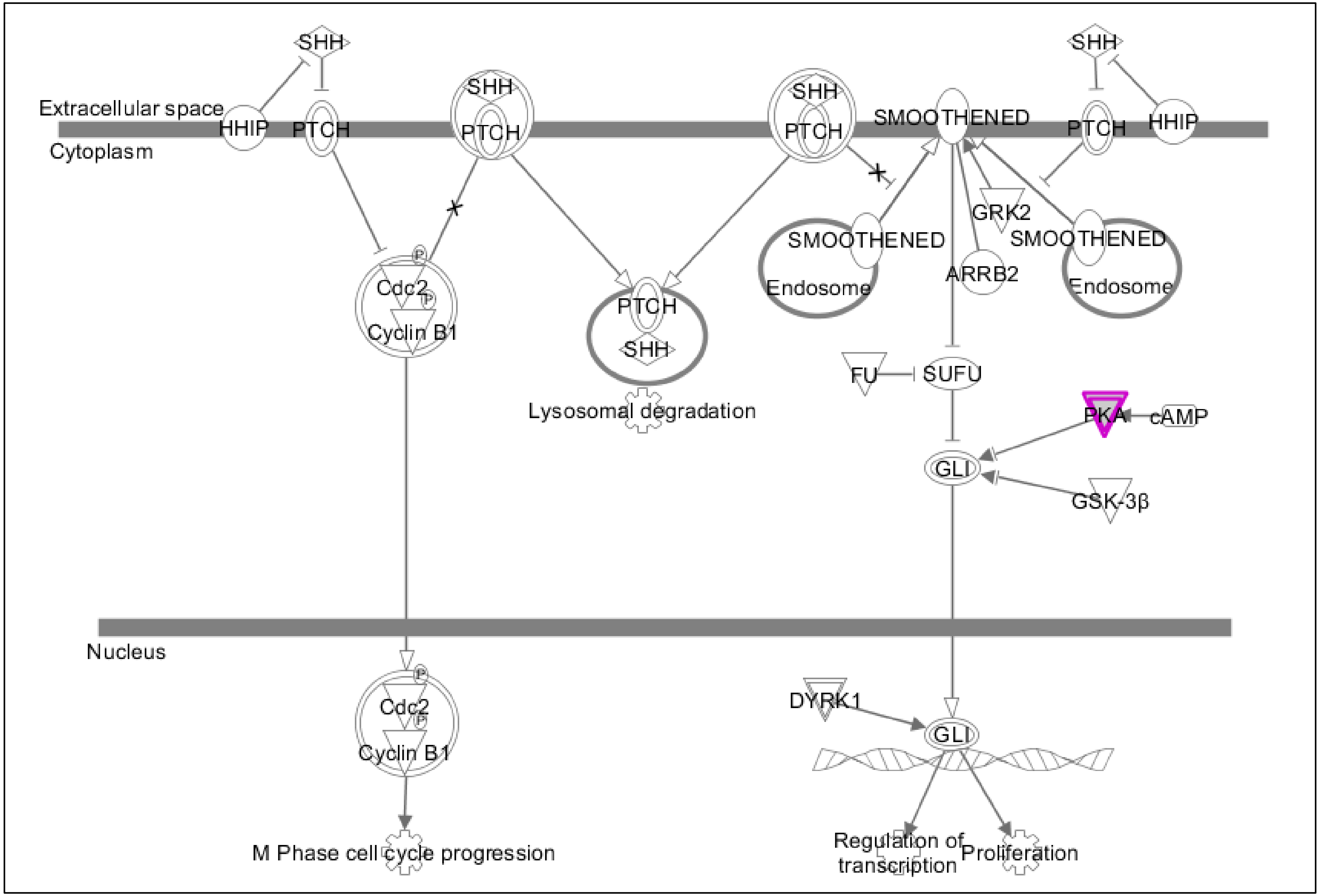

15-Netrin Signaling

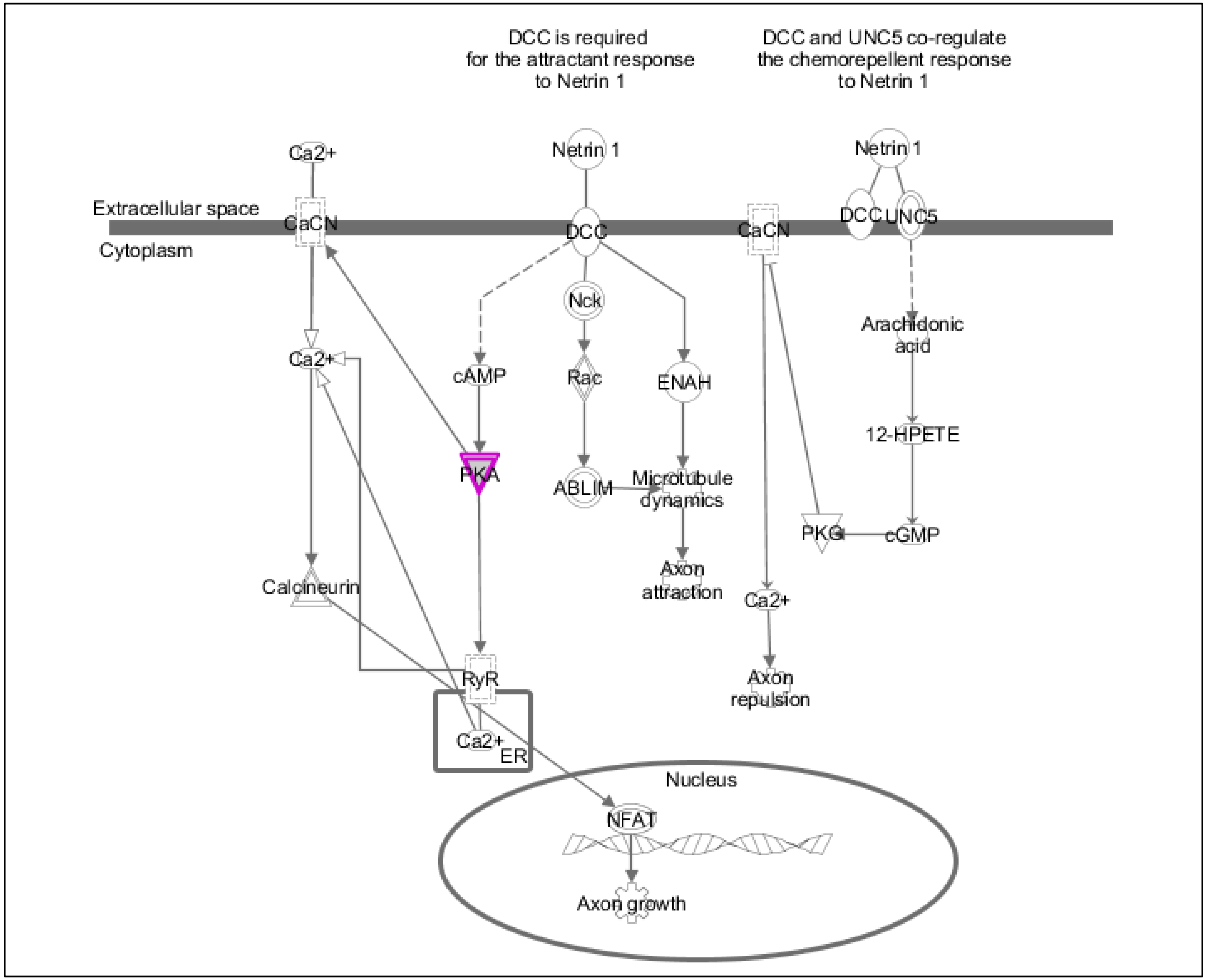

17-Neuroprotective Role of THOP1 in Alzheimer's Disease

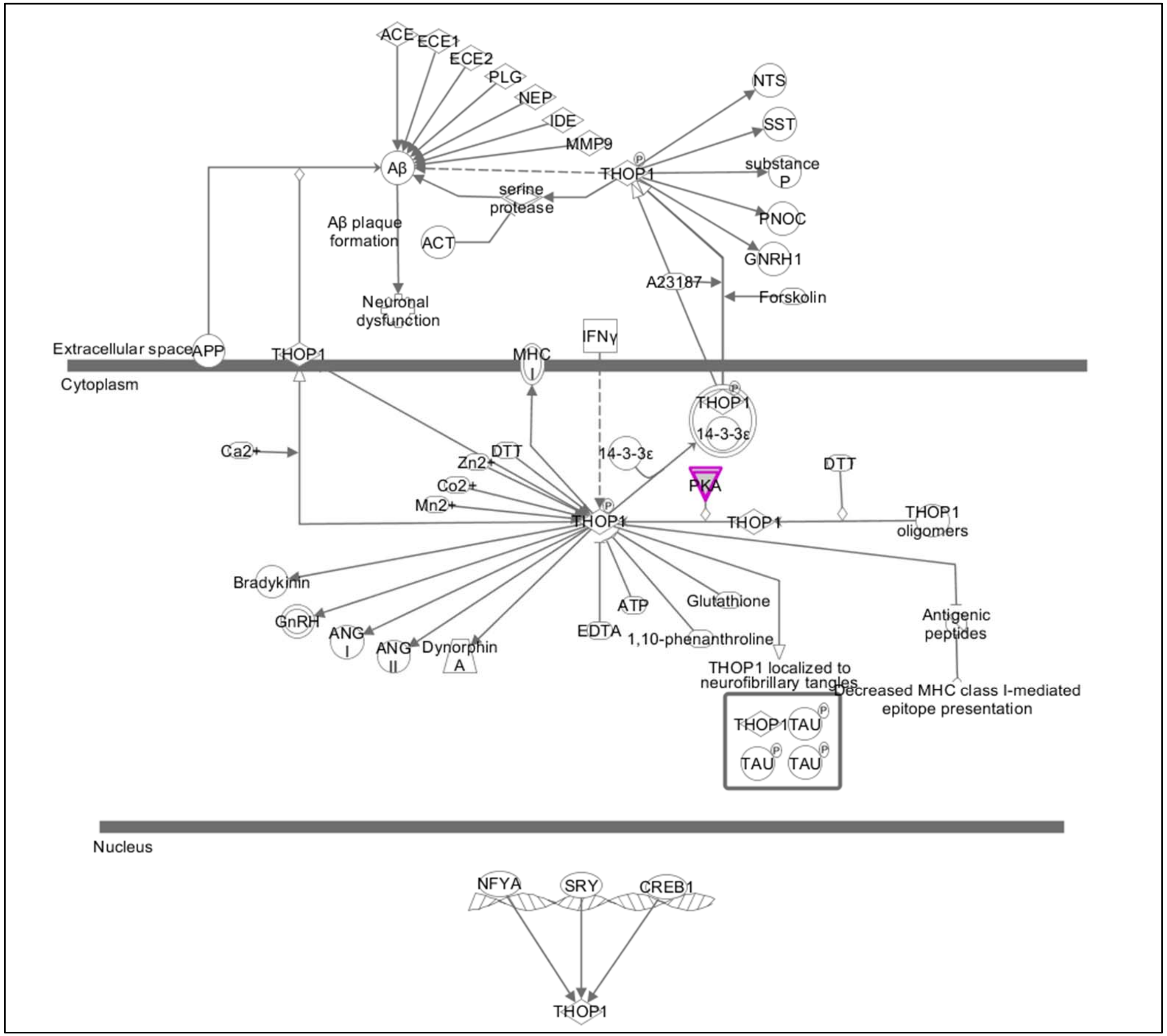

18-iNOS Signaling

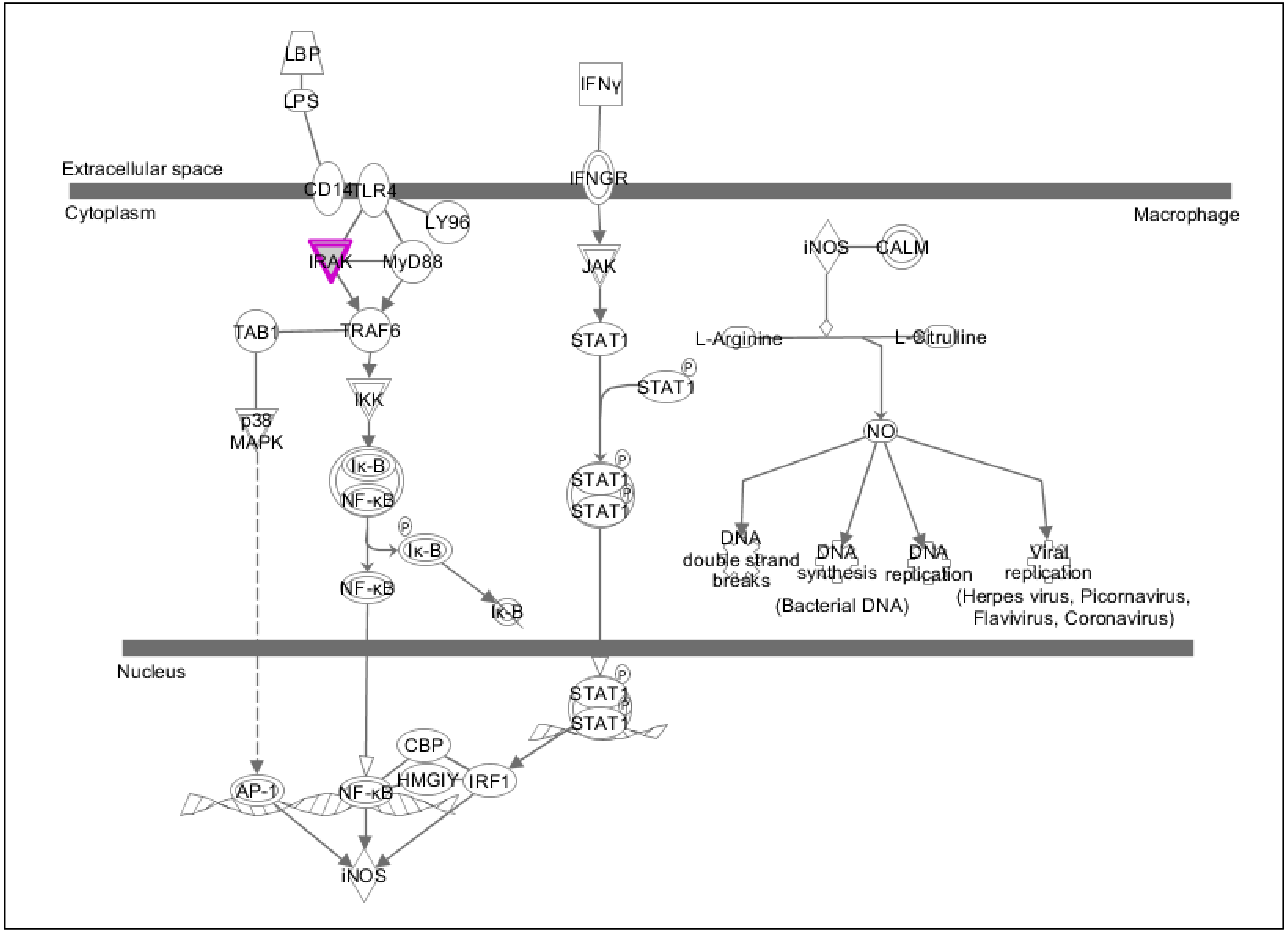

19-Gaft-versus-Host Disease Signaling

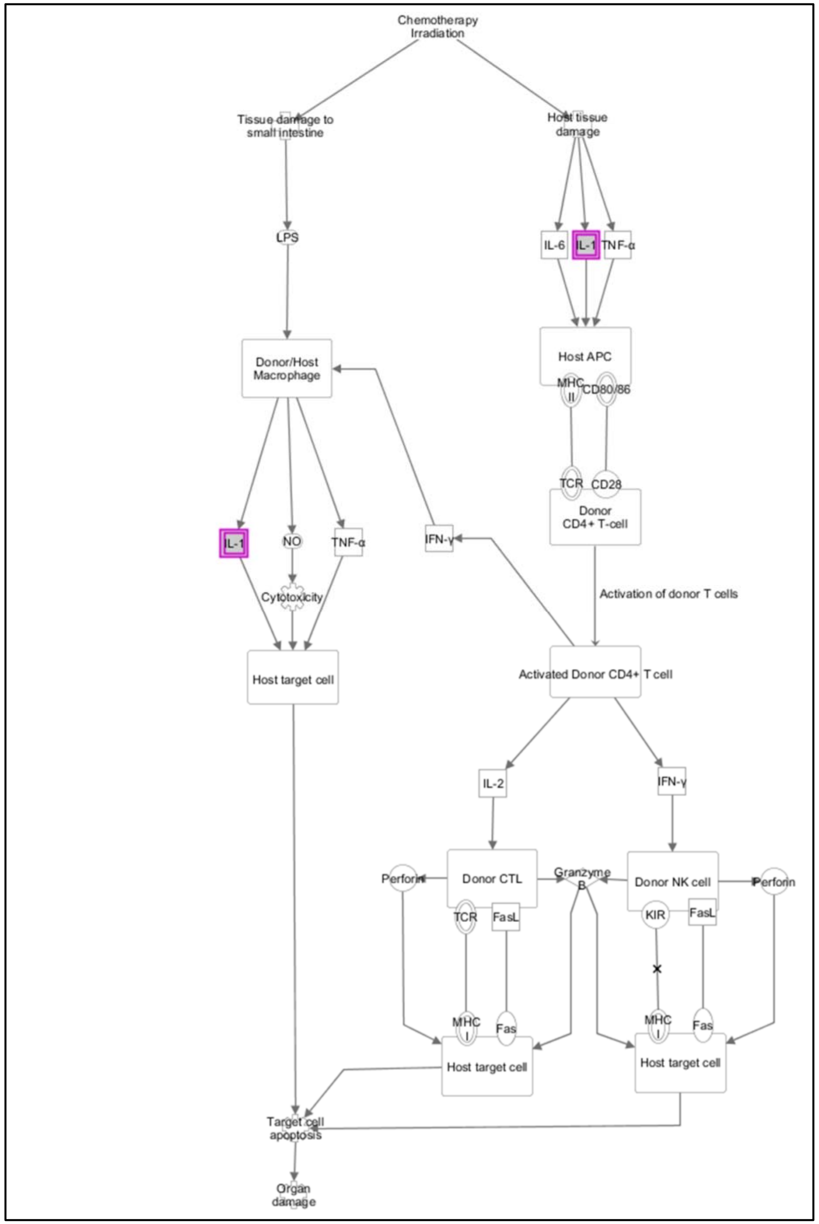

20-Amyloid Processing

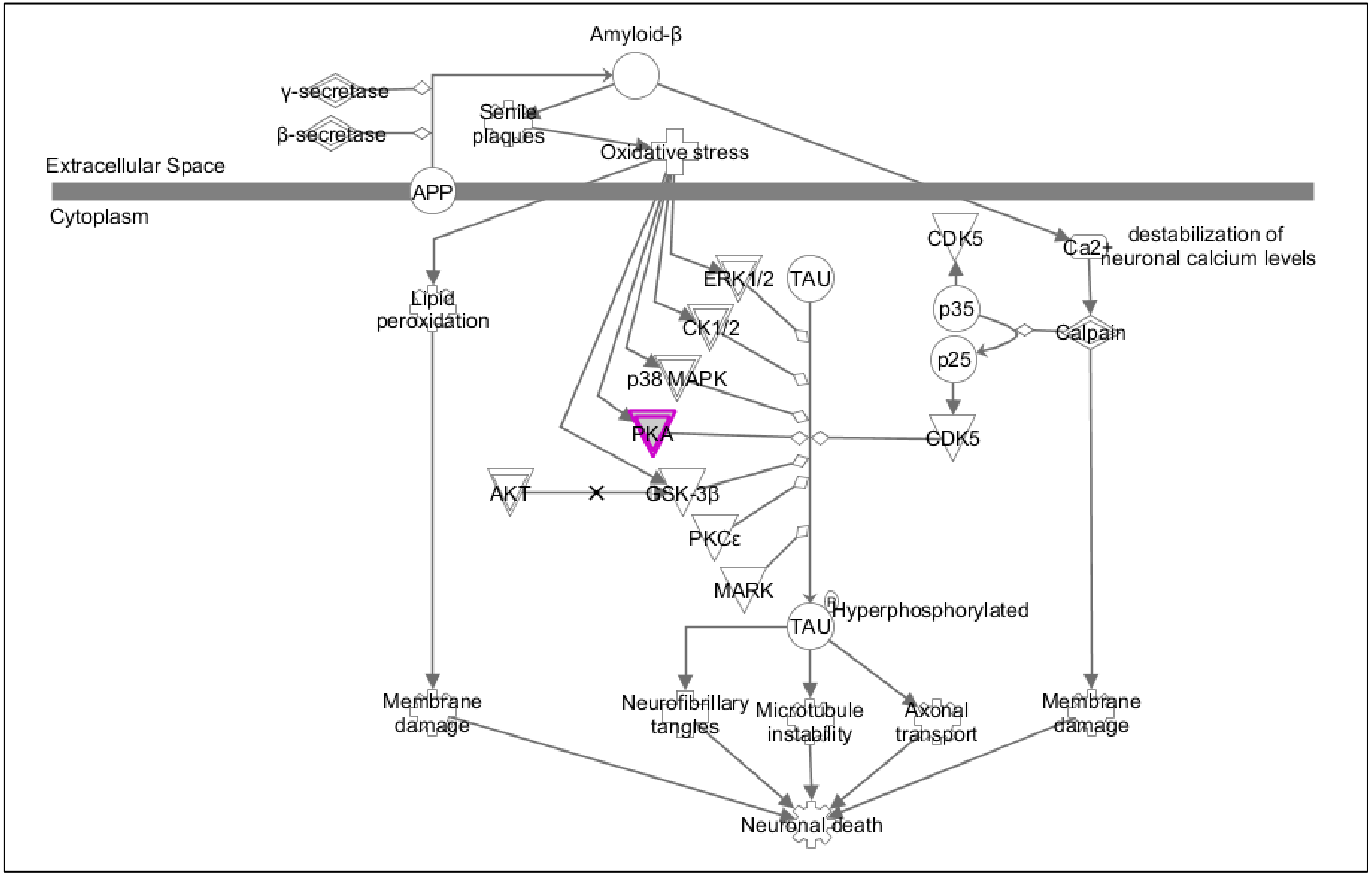

21-Role of Cytokines in Mediating Communication between Immune Cells

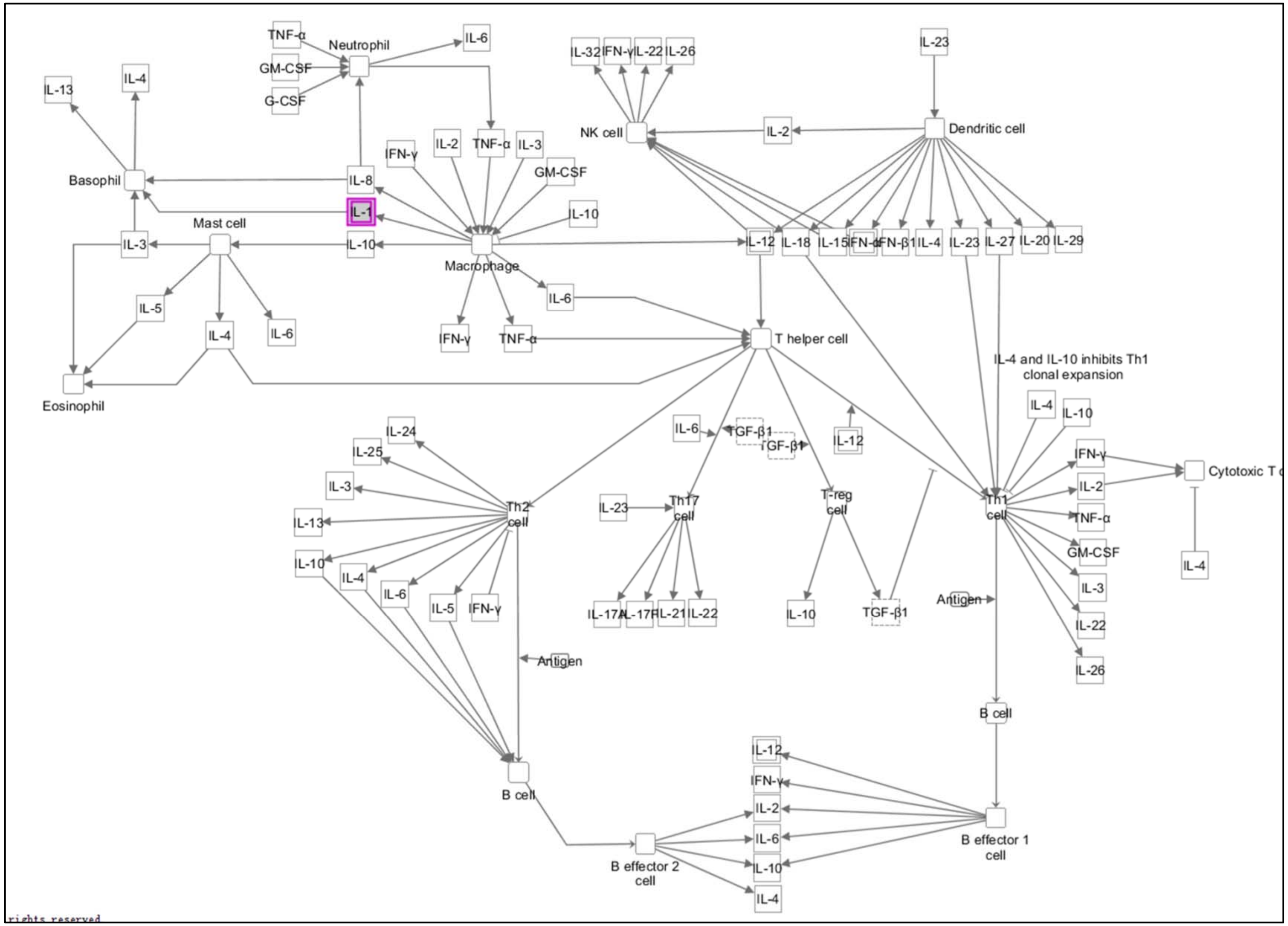

rights reserved

22-Phototransduction pathway

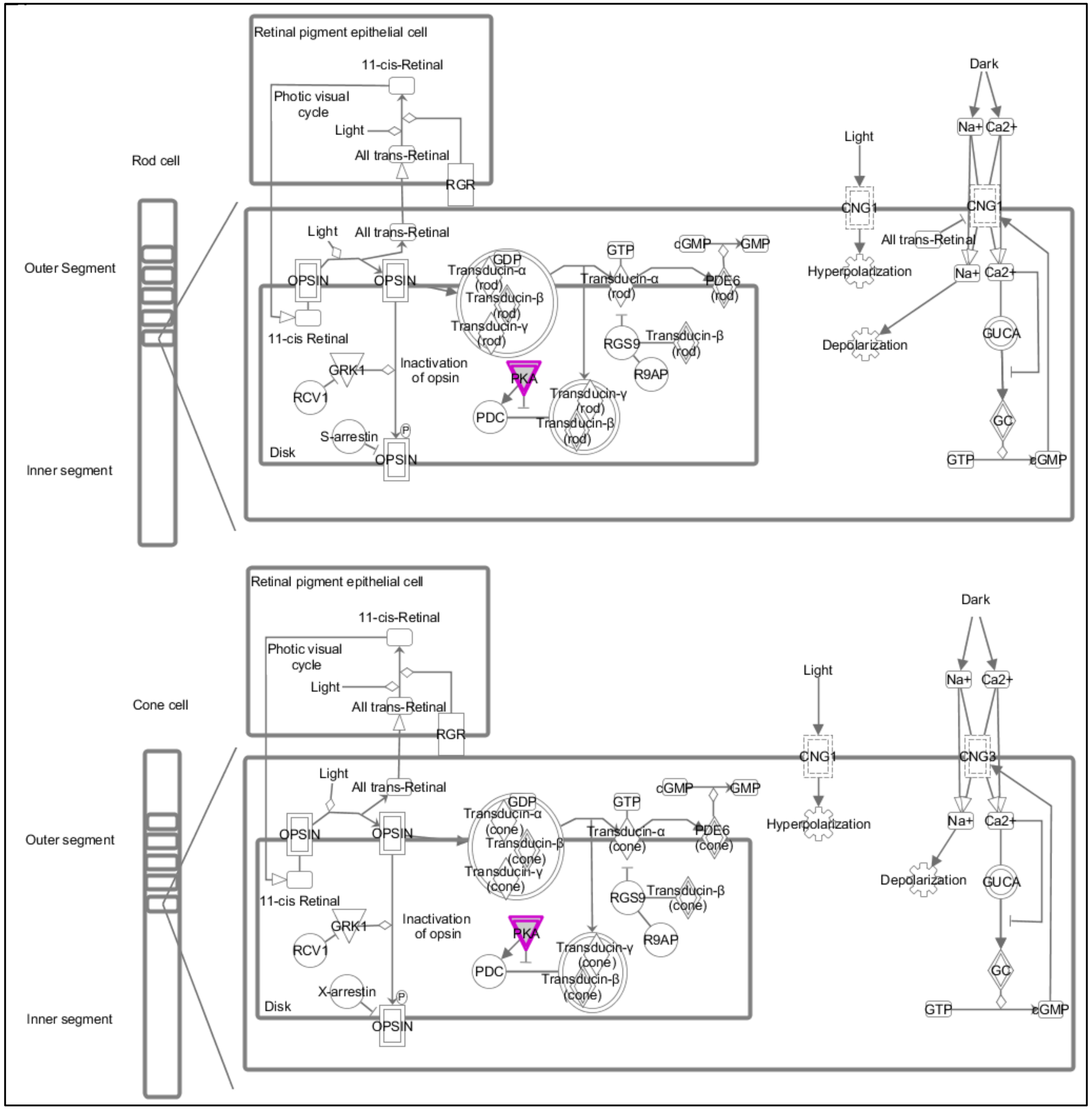

23-PXR_RXR activation

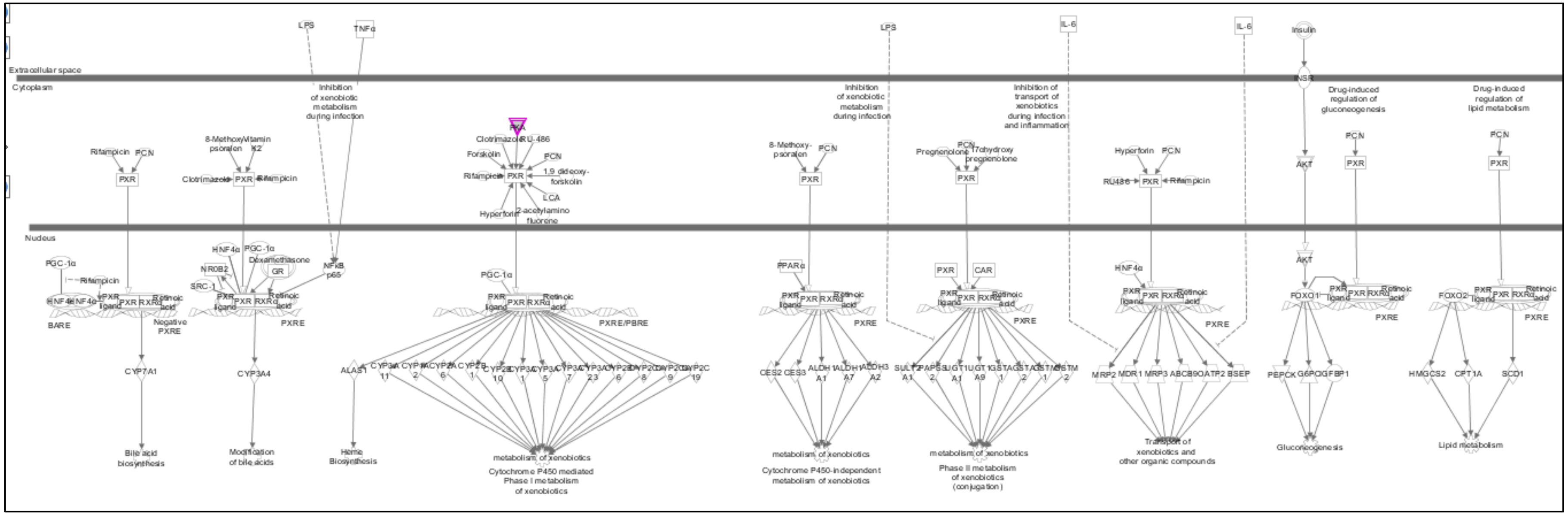

24-IL-10 signaling

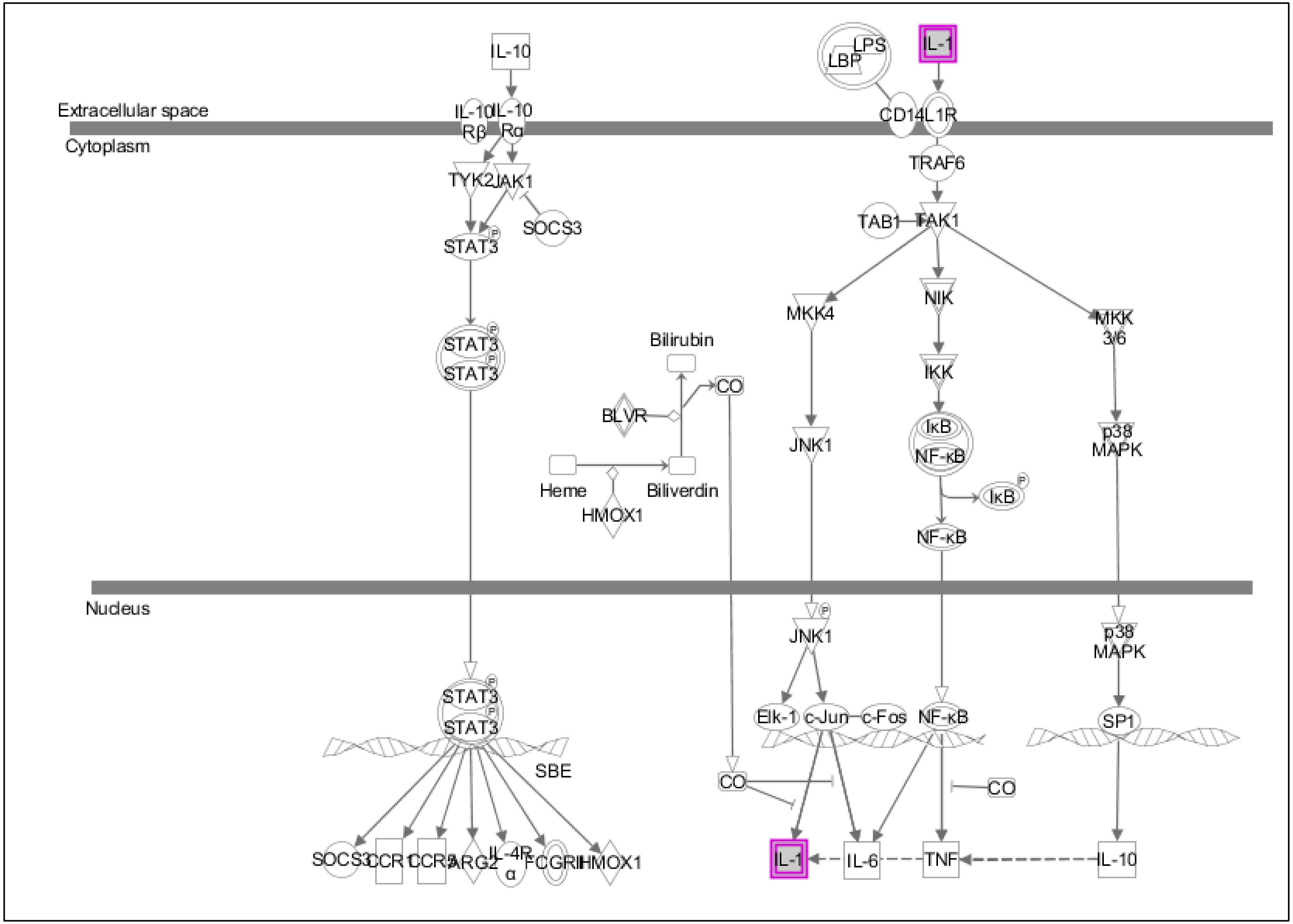

25-Melatonin signaling

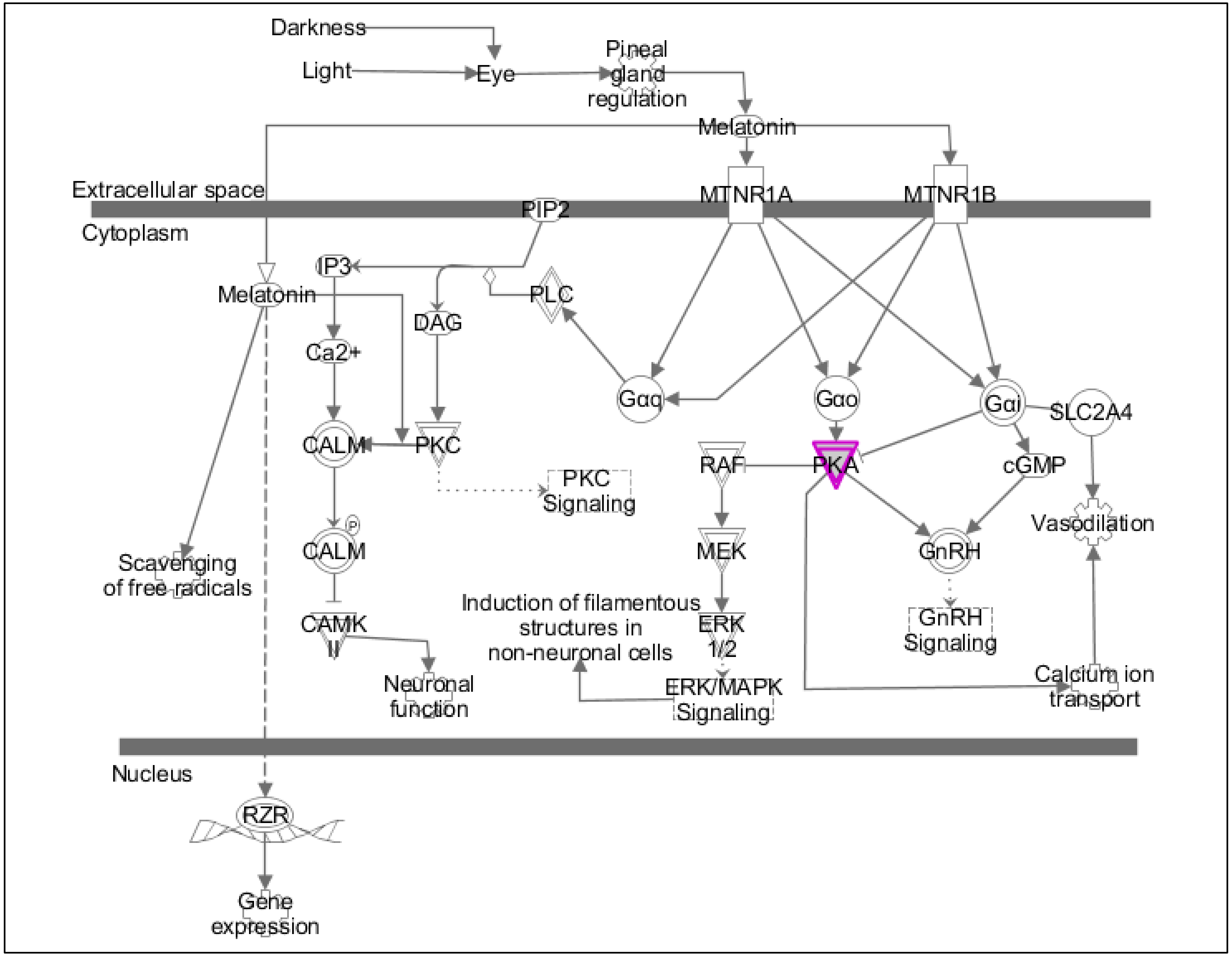

26-BMP signaling pathway

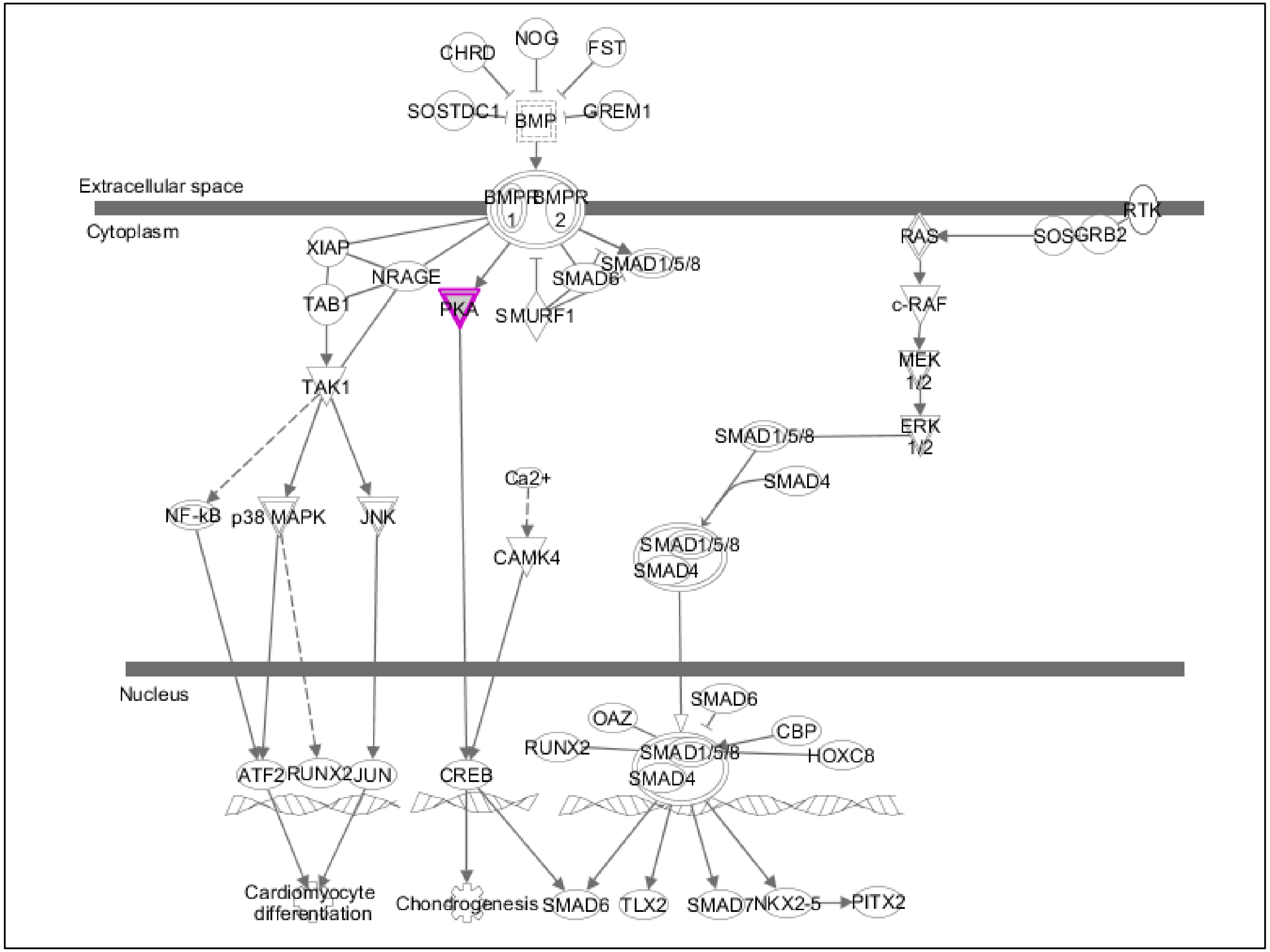

27-Leptin signaling in obesity

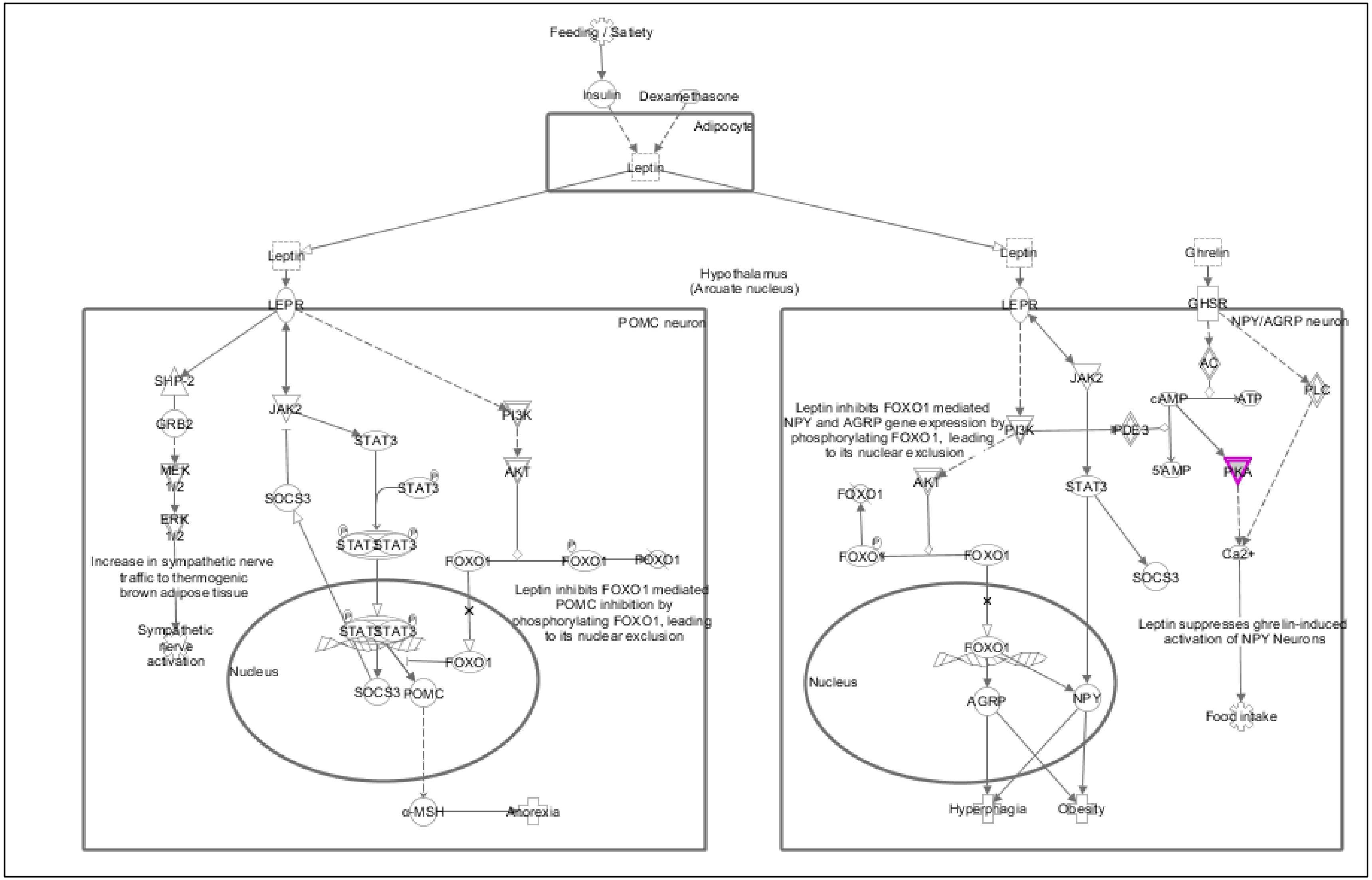

28-Melanocyte development and pigmentation signaling

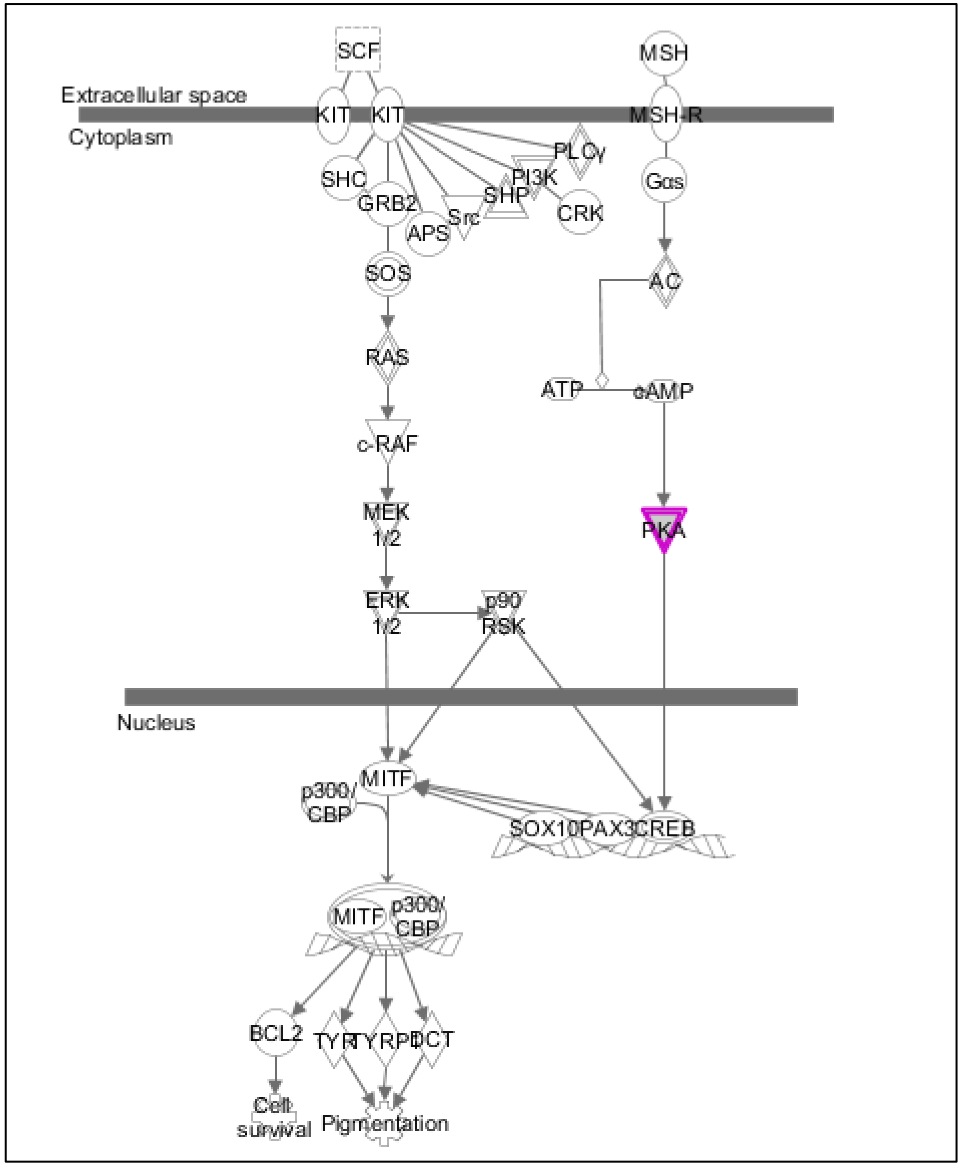

29-Altered T cell and B cell signaling in Rheumatoid arthritis

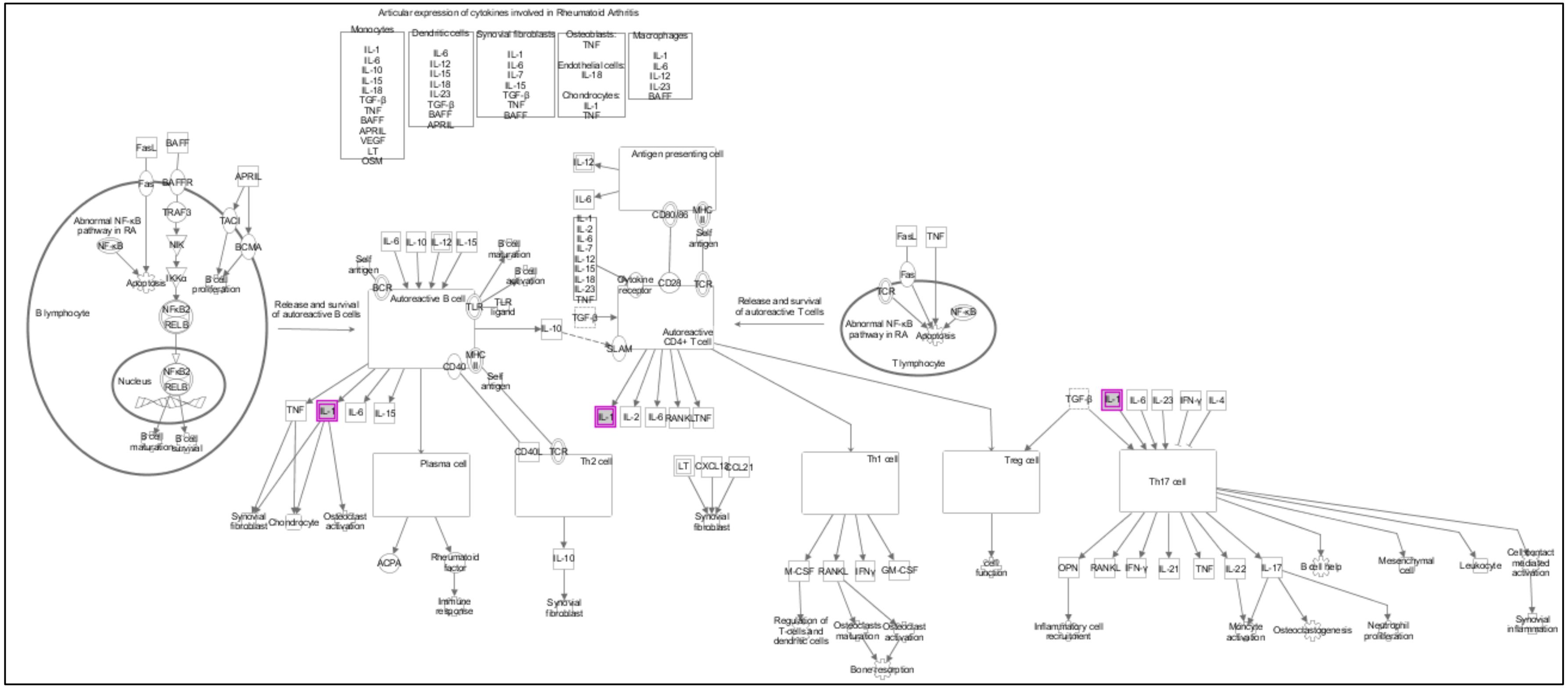

Supplement: Supplementary file 3 [file Presentation_3.zip › Supplemental materials 4.3.pdf]
