# Supplementary material for: Multiomics-Based Signaling Pathway Network Alterations in Human Non-functional Pituitary Adenomas
Source: Front Endocrinol (Lausanne). 2019 Dec 17;10:835. doi: 10.3389/fendo.2019.00835 (PMC6928143; doi:10.3389/fendo.2019.00835)
Supplement: Supplementary file 3 [file Presentation_3.zip › Supplemental materials 5.1.pdf]

Supplemental materials 5.1 Differentially expressed genes between invasive and non-invasive NFPAs for IPA analysis (Dataset 5)

| ID | Notes | Molecules | Description | Location | Function |
|---|---|---|---|---|---|
| SLURP1 | | SLURP1 | secreted LY6/PLAUR domain containing 1 | Extracellular Space | cytokine |
| ALDOA | | ALDOA | aldolase A, fructose-bisphosphate | Cytoplasm | enzyme |
| AW386276 | | ST6GAL1 | ST6 beta-galactosamide alpha-2,6-sialyltranferase 1 | Cytoplasm | enzyme |
| CHAT | | CHAT | choline O-acetyltransferase | Nucleus | enzyme |
| COX6A2 | | COX6A2 | cytochrome c oxidase subunit VIa polypeptide 2 | Cytoplasm | enzyme |
| DICER1 | | DICER1 | dicer 1, ribonuclease type III | Cytoplasm | enzyme |
| DNAJC18 | | DNAJC18 | DnaJ (Hsp40) homolog, subfamily C, member 18 | Other | enzyme |
| FBXO9 | | FBXO9 | F-box protein 9 | Cytoplasm | enzyme |
| GNPTAB | | GNPTAB | N-acetylglucosamine-1-phosphate transferase, alpha and beta subunits | Cytoplasm | enzyme |
| HS2ST1 | | HS2ST1 | heparan sulfate 2-O-sulfotransferase 1 | Cytoplasm | enzyme |
| HSPCA | | HSP90AA1 | heat shock protein 90kDa alpha (cytosolic), class A member 1 | Cytoplasm | enzyme |

| | | | | |
|---|---|---|---|---|
| HSPCB | HSP90AB1 | heat shock protein 90kDa alpha (cytosolic), class B member 1 | Cytoplasm | enzyme |
| IDH3G | IDH3G | isocitrate dehydrogenase 3 (NAD+) gamma | Cytoplasm | enzyme |
| KARS | KARS | lysyl-tRNA synthetase | Cytoplasm | enzyme |
| 391462 | PON3 | paraoxonase 3 | Extracellular Space | enzyme |
| MAN1B1 | MAN1B1 | mannosidase, alpha, class 1B, member 1 | Cytoplasm | enzyme |
| MRPL49 | MRPL49 | mitochondrial ribosomal protein L49 | Cytoplasm | enzyme |
| MTND3 | MT-ND3 | NADH dehydrogenase, subunit 3 (complex I) | Cytoplasm | enzyme |
| MYO5A | MYO5A | myosin VA (heavy chain 12, myoxin) | Cytoplasm | enzyme |
| NAV1 | NAV1 | neuron navigator 1 | Other | enzyme |
| OGDH | OGDH | oxoglutarate (alpha-ketoglutarate) dehydrogenase (lipoamide) | Cytoplasm | enzyme |
| PABPN1 | PABPN1 | poly(A) binding protein, nuclear 1 | Nucleus | enzyme |
| PARP10 | PARP10 | poly (ADP-ribose) polymerase family, member 10 | Cytoplasm | enzyme |
| PRDX2 | PRDX2 | peroxiredoxin 2 | Cytoplasm | enzyme |
| RAB18 | RAB18 | RAB18, member RAS oncogene family | Cytoplasm | enzyme |

| | | | | |
|---|---|---|---|---|
| RAB1A | RAB1A | RAB1A, member RAS oncogene family | Cytoplasm | enzyme |
| RALBP1 | RALBP1 | ralA binding protein 1 | Cytoplasm | enzyme |
| RFNG | RFNG | RFNG O-fucosylpeptide 3-beta-N-acetylglucosaminyltransferase | Cytoplasm | enzyme |
| RHOBTB3 | RHOBTB3 | Rho-related BTB domain containing 3 | Cytoplasm | enzyme |
| TBXAS1 | TBXAS1 | thromboxane A synthase 1 (platelet) | Plasma Membrane | enzyme |
| 2054351 | ARHGEF10 | Rho guanine nucleotide exchange factor (GEF) 10 | Cytoplasm | enzyme |
| UBE2L3 | UBE2L3 | ubiquitin-conjugating enzyme E2L 3 | Nucleus | enzyme |
| UHRF2 | UHRF2 | ubiquitin-like with PHD and ring finger domains 2, E3 ubiquitin protein ligase | Nucleus | enzyme |
| VARS | VARS | valyl-tRNA synthetase | Cytoplasm | enzyme |
| WHSC1L1 | WHSC1L1 | Wolf-Hirschhorn syndrome candidate 1-like 1 | Nucleus | enzyme |
| ZDHHC21 | ZDHHC21 | zinc finger, DHHC-type containing 21 | Plasma Membrane | enzyme |
| GPR12 | GPR12 | G protein-coupled receptor 12 | Plasma Membrane | G-protein coupled receptor |
| Y10152 | CRHR2 | corticotropin releasing hormone receptor 2 | Plasma Membrane | G-protein coupled receptor |
| GDNF | GDNF | glial cell derived neurotrophic factor | Extracellular Space | growth factor |

| | | | | |
|---|---|---|---|---|
| CACNA1E | CACNA1E | calcium channel, voltage-dependent, R type, alpha 1E subunit | Plasma Membrane | ion channel |
| SCN3A | SCN3A | sodium channel, voltage-gated, type III, alpha subunit | Plasma Membrane | ion channel |
| AF130080 | NUCKS1 | nuclear casein kinase and cyclin-dependent kinase substrate 1 | Nucleus | kinase |
| BRD3 | BRD3 | bromodomain containing 3 | Nucleus | kinase |
| CDC2L2 | CDK11A | cyclin-dependent kinase 11A | Nucleus | kinase |
| CDK3 | CDK3 | cyclin-dependent kinase 3 | Other | kinase |
| CDKL5 | CDKL5 | cyclin-dependent kinase-like 5 | Nucleus | kinase |
| CKB | CKB | creatine kinase, brain | Cytoplasm | kinase |
| FLT3 | FLT3 | fms-related tyrosine kinase 3 | Plasma Membrane | kinase |
| IHPK2 | IP6K2 | inositol hexakisphosphate kinase 2 | Cytoplasm | kinase |
| PDPK1 | PDPK1 | 3-phosphoinositide dependent protein kinase 1 | Cytoplasm | kinase |
| PFKL | PFKL | phosphofructokinase, liver | Cytoplasm | kinase |
| PIK3CB | PIK3CB | phosphatidylinositol-4,5-bisphosphate 3-kinase, catalytic subunit beta | Cytoplasm | kinase |
| PRKAR1A | PRKAR1A | protein kinase, cAMP-dependent, regulatory, type I, alpha | Cytoplasm | kinase |

| | | | | |
|---|---|---|---|---|
| RPS6KA5 | RPS6KA5 | ribosomal protein S6 kinase, 90kDa, polypeptide 5 | Nucleus | kinase |
| WNK3 | WNK3 | WNK lysine deficient protein kinase 3 | Plasma Membrane | kinase |
| ESR1 | ESR1 | estrogen receptor 1 | Nucleus | ligand-dependent nuclear receptor |
| ESR2 | ESR2 | estrogen receptor 2 (ER beta) | Nucleus | ligand-dependent nuclear receptor |
| ACRBP | ACRBP | acrosin binding protein | Extracellular Space | other |
| AI878825 | FAM193B | family with sequence similarity 193, member B | Other | other |
| AK091904 | ZBTB20 | zinc finger and BTB domain containing 20 | Nucleus | other |
| AK096229 | LOC100128364 | uncharacterized LOC100128364 | Other | other |
| AKAP10 | AKAP10 | A kinase (PRKA) anchor protein 10 | Cytoplasm | other |
| BAALC | BAALC | brain and acute leukemia, cytoplasmic | Cytoplasm | other |
| BBC3 | BBC3 | BCL2 binding component 3 | Cytoplasm | other |
| BC011940 | LINC01023 | long intergenic non-protein coding RNA 1023 | Other | other |
| BC018035 | MAPT-AS1 | MAPT antisense RNA 1 | Other | other |
| BC028232 | SNORA78 | small nucleolar RNA, H/ACA box 78 | Other | other |

| | | | | |
|---|---|---|---|---|
| BC034002 | LOC648987 | uncharacterized LOC648987 | Other | other |
| BC035731 | SYCE1L | synaptonemal complex central element protein 1-like | Other | other |
| BC039450 | HHIP-AS1 | HHIP antisense RNA 1 | Other | other |
| BTN3A2 | BTN3A2 | butyrophilin, subfamily 3, member A2 | Plasma Membrane | other |
| C14orf129 | GSKIP | GSK3B interacting protein | Other | other |
| C19orf24 | C19orf24 | chromosome 19 open reading frame 24 | Other | other |
| C1orf113 | SH3D21 | SH3 domain containing 21 | Cytoplasm | other |
| C1orf128 | PITHD1 | PITH (C-terminal proteasome-interacting domain of thioredoxin-like) domain containing 1 | Nucleus | other |
| C1orf149 | MEAF6 | MYST/Esa1-associated factor 6 | Nucleus | other |
| C1orf173 | ERICH3 | glutamate-rich 3 | Other | other |
| C1orf43 | C1orf43 | chromosome 1 open reading frame 43 | Other | other |
| C20orf86 | ANKRD60 | ankyrin repeat domain 60 | Other | other |
| C3orf10 | BRK1 | BRICK1, SCAR/WAVE actin-nucleating complex subunit | Cytoplasm | other |
| C9orf10 | FAM120A | family with sequence similarity 120A | Cytoplasm | other |

| | | | | |
|---|---|---|---|---|
| C9orf62 | C9orf62 | chromosome 9 open reading frame 62 | Other | other |
| CAPG | CAPG | capping protein (actin filament), gelsolin-like | Nucleus | other |
| CASC4 | CASC4 | cancer susceptibility candidate 4 | Cytoplasm | other |
| CD200 | CD200 | CD200 molecule | Plasma Membrane | other |
| CDH1 | CDH1 | cadherin 1, type 1, E-cadherin (epithelial) | Plasma Membrane | other |
| CEP2 | CEP250 | centrosomal protein 250kDa | Nucleus | other |
| CHMP5 | CHMP5 | charged multivesicular body protein 5 | Cytoplasm | other |
| CPLX2 | CPLX2 | complexin 2 | Cytoplasm | other |
| DCUN1D1 | DCUN1D1 | DCN1, defective in cullin neddylation 1, domain containing 1 | Nucleus | other |
| DKFZP434I0714 | DKFZP434I0714 | uncharacterized protein DKFZP434I0714 | Other | other |
| DLGAP3 | DLGAP3 | discs, large (Drosophila) homolog-associated protein 3 | Cytoplasm | other |
| DST | DST | dystonin | Plasma Membrane | other |
| ENST00000325814 | C17orf96 | chromosome 17 open reading frame 96 | Other | other |
| FAM79A | TPRG1L | tumor protein p63 regulated 1-like | Cytoplasm | other |

| | | | | |
|---|---|---|---|---|
| FAM98A | FAM98A | family with sequence similarity 98, member A | Other | other |
| FBXO38 | FBXO38 | F-box protein 38 | Other | other |
| FCRL2 | FCRL2 | Fc receptor-like 2 | Other | other |
| FLJ22184 | FLJ22184 | putative uncharacterized protein FLJ22184 | Other | other |
| FLJ38379 | FLJ38379 | uncharacterized FLJ38379 | Other | other |
| FLNA | FLNA | filamin A, alpha | Cytoplasm | other |
| FMN2 | FMN2 | formin 2 | Cytoplasm | other |
| FTS | AKTIP | AKT interacting protein | Cytoplasm | other |
| GPIAP1 | CAPRIN1 | cell cycle associated protein 1 | Plasma Membrane | other |
| H41 | H41 | histocompatibility 41 | Other | other |
| HNRPA0 | HNRNPA0 | heterogeneous nuclear ribonucleoprotein A0 | Nucleus | other |
| IER5 | IER5 | immediate early response 5 | Other | other |
| IGFBP5 | IGFBP5 | insulin-like growth factor binding protein 5 | Extracellular Space | other |
| IMMT | IMMT | inner membrane protein, mitochondrial | Cytoplasm | other |

| | | | | |
|---|---|---|---|---|
| KIAA0182 | GSE1 | Gse1 coiled-coil protein | Extracellular Space | other |
| KIAA0319L | KIAA0319L | KIAA0319-like | Cytoplasm | other |
| KIAA0476 | DENND4B | DENN/MADD domain containing 4B | Extracellular Space | other |
| KIAA1407 | KIAA1407 | KIAA1407 | Other | other |
| KIAA1671 | KIAA1671 | KIAA1671 | Other | other |
| L07392 | C7orf50 | chromosome 7 open reading frame 50 | Other | other |
| LCE1D | LCE1D | late cornified envelope 1D | Cytoplasm | other |
| LIN7C | LIN7C | lin-7 homolog C (C. elegans) | Cytoplasm | other |
| 144363 | LYRM5 | LYR motif containing 5 | Cytoplasm | other |
| 146325 | PRR35 | proline rich 35 | Other | other |
| 196752 | ZSWIM8 | zinc finger, SWIM-type containing 8 | Extracellular Space | other |
| 201175 | ARHGAP27 | Rho GTPase activating protein 27 | Cytoplasm | other |
| 255783 | INAFM1 | InaF-motif containing 1 | Other | other |
| 283345 | RPL13P5 | ribosomal protein L13 pseudogene 5 | Other | other |

| | | | | |
|---|---|---|---|---|
| 283849 | EXOC3L1 | exocyst complex component 3-like 1 | Cytoplasm | other |
| 284889 | LOC284889 | uncharacterized LOC284889 | Other | other |
| 339210 | C17orf67 | chromosome 17 open reading frame 67 | Other | other |
| 346355 | ELFN1 | extracellular leucine-rich repeat and fibronectin type III domain containing 1 | Plasma Membrane | other |
| 375010 | ANKRD20A12P | | Other | other |
| 387680 | FAM21A/FAM21C | family with sequence similarity 21, member C | Cytoplasm | other |
| 389199 | ACTR1A | ARP1 actin-related protein 1 homolog A, centractin alpha (yeast) | Cytoplasm | other |
| 389607 | CCDC125 | coiled-coil domain containing 125 | Other | other |
| 391014 | HNRNPA2B1 | heterogeneous nuclear ribonucleoprotein A2/B1 | Nucleus | other |
| 399491 | FGB | fibrinogen beta chain | Extracellular Space | other |
| 399959 | MIR100HG | mir-100-let-7a-2 cluster host gene (non-protein coding) | Other | other |
| 439951 | 3-indoleglycolaldehyde | | Other | chemical - endogenous non-[illegible] |
| 51328 | HLA-A | major histocompatibility complex, class I, A | Plasma Membrane | other |
| 92482 | BBIP1 | BBSome interacting protein 1 | Cytoplasm | other |

| | | | | |
|---|---|---|---|---|
| LRRTM2 | LRRTM2 | leucine rich repeat transmembrane neuronal 2 | Plasma Membrane | other |
| MAG | MAG | myelin associated glycoprotein | Plasma Membrane | other |
| MCP | CD46 | CD46 molecule, complement regulatory protein | Plasma Membrane | other |
| MED8 | MED8 | mediator complex subunit 8 | Nucleus | other |
| MON1B | MON1B | MON1 secretory trafficking family member B | Other | other |
| MORF4L1 | MORF4L1 | mortality factor 4 like 1 | Nucleus | other |
| MYCNOS | MYCNOS | MYCN opposite strand | Other | other |
| NPAS3 | NPAS3 | neuronal PAS domain protein 3 | Nucleus | other |
| NPIP | NPIPA1 (includes others) | nuclear pore complex interacting protein family, member A1 | Nucleus | other |
| OSBP | OSBP | oxysterol binding protein | Cytoplasm | other |
| PARD6G | PARD6G | par-6 family cell polarity regulator gamma | Plasma Membrane | other |
| PKIG | PKIG | protein kinase (cAMP-dependent, catalytic) inhibitor gamma | Other | other |
| PPP1R11 | PPP1R11 | protein phosphatase 1, regulatory (inhibitor) subunit 11 | Cytoplasm | other |
| PRR5 | PRR5 | proline rich 5 (renal) | Other | other |

| | | | | |
|---|---|---|---|---|
| PRR7 | PRR7 | proline rich 7 (synaptic) | Other | other |
| PRTG | PRTG | protogenin | Extracellular Space | other |
| PTPLAD1 | PTPLAD1 | protein tyrosine phosphatase-like A domain containing 1 | Cytoplasm | other |
| PYY2 | PYY2 | peptide YY, 2 (pseudogene) | Other | other |
| RAI17 | ZMIZ1 | zinc finger, MIZ-type containing 1 | Nucleus | other |
| RASA4 | RASA4 | RAS p21 protein activator 4 | Cytoplasm | other |
| RGS3 | RGS3 | regulator of G-protein signaling 3 | Nucleus | other |
| RKHD1 | MEX3D | mex-3 RNA binding family member D | Nucleus | other |
| RNPS1 | RNPS1 | RNA binding protein S1, serine-rich domain | Nucleus | other |
| RPL10 | RPL10 | ribosomal protein L10 | Cytoplasm | other |
| RPL18A | RPL18A | ribosomal protein L18a | Cytoplasm | other |
| RPS2 | RPS2 | ribosomal protein S2 | Cytoplasm | other |
| RPS5 | RPS5 | ribosomal protein S5 | Cytoplasm | other |
| RSBN1 | RSBN1 | round spermatid basic protein 1 | Nucleus | other |

| | | | | |
|---|---|---|---|---|
| S100A5 | S100A5 | S100 calcium binding protein A5 | Nucleus | other |
| SAFB | SAFB | scaffold attachment factor B | Nucleus | other |
| SCRT2 | SCRT2 | scratch family zinc finger 2 | Nucleus | other |
| SEL1L | SEL1L | sel-1 suppressor of lin-12-like (C. elegans) | Cytoplasm | other |
| SELM | SELM | selenoprotein M | Cytoplasm | other |
| SELT | SELT | selenoprotein T | Cytoplasm | other |
| SESN1 | SESN1 | sestrin 1 | Nucleus | other |
| SFT2D3 | SFT2D3 | SFT2 domain containing 3 | Other | other |
| SH3KBP1 | SH3KBP1 | SH3-domain kinase binding protein 1 | Cytoplasm | other |
| SON | SON | SON DNA binding protein | Nucleus | other |
| SRP9 | SRP9 | signal recognition particle 9kDa | Cytoplasm | other |
| TCP1 | TCP1 | t-complex 1 | Cytoplasm | other |
| TFG | TFG | TRK-fused gene | Cytoplasm | other |
| THAP5 | THAP5 | THAP domain containing 5 | Nucleus | other |

| | | | | |
|---|---|---|---|---|
| 2016168 | SPOCK1 | sparc/osteonectin, cwcv and kazal-like domains proteoglycan (testican) 1 | Extracellular Space | other |
| 2074889 | E330034G19Rik | RIKEN cDNA E330034G19 gene | Other | other |
| 2164098 | NUCB1 | nucleobindin 1 | Cytoplasm | other |
| 2165212 | ATG13 | autophagy related 13 | Cytoplasm | other |
| 2210612 | TMEM167A | transmembrane protein 167A | Other | other |
| TNXB | TNXB | tenascin XB | Extracellular Space | other |
| TPM3 | TPM3 | tropomyosin 3 | Cytoplasm | other |
| TPM4 | TPM4 | tropomyosin 4 | Cytoplasm | other |
| TRIM35 | TRIM35 | tripartite motif containing 35 | Cytoplasm | other |
| UNC13C | UNC13C | unc-13 homolog C (C. elegans) | Cytoplasm | other |
| YWHAG | YWHAG | tyrosine 3-monooxygenase/tryptophan 5-monooxygenase activation protein, gamma | Cytoplasm | other |
| ZBTB33 | ZBTB33 | zinc finger and BTB domain containing 33 | Nucleus | other |
| ZNF341 | ZNF341 | zinc finger protein 341 | Nucleus | other |
| ZNF347 | ZNF347 | zinc finger protein 347 | Nucleus | other |

| | | | | |
|---|---|---|---|---|
| ZNF44 | ZNF44 | zinc finger protein 44 | Nucleus | other |
| ZNF575 | ZNF575 | zinc finger protein 575 | Nucleus | other |
| ZNF579 | ZNF579 | zinc finger protein 579 | Nucleus | other |
| ADAMTS7 | ADAMTS7 | ADAM metallopeptidase with thrombospondin type 1 motif, 7 | Extracellular Space | peptidase |
| PRSS27 | PRSS27 | protease, serine 27 | Extracellular Space | peptidase |
| TPSG1 | TPSG1 | tryptase gamma 1 | Extracellular Space | peptidase |
| PPM1A | PPM1A | protein phosphatase, Mg2+/Mn2+ dependent, 1A | Cytoplasm | phosphatase |
| PPM1K | PPM1K | protein phosphatase, Mg2+/Mn2+ dependent, 1K | Cytoplasm | phosphatase |
| AA219130 | ETV5 | ets variant 5 | Nucleus | transcription regulator |
| CBX1 | CBX1 | chromobox homolog 1 | Nucleus | transcription regulator |
| CBX5 | CBX5 | chromobox homolog 5 | Nucleus | transcription regulator |
| CEBPA | CEBPA | CCAAT/enhancer binding protein (C/EBP), alpha | Nucleus | transcription regulator |
| CITED1 | CITED1 | Cbp/p300-interacting transactivator, with Glu/Asp-rich carboxy-terminal domain, 1 | Nucleus | transcription regulator |
| CLOCK | CLOCK | clock circadian regulator | Nucleus | transcription regulator |

| | | | | |
|---|---|---|---|---|
| DNAJB6 | DNAJB6 | DnaJ (Hsp40) homolog, subfamily B, member 6 | Nucleus | transcription regulator |
| ENST00000315091 | TARDBP | TAR DNA binding protein | Nucleus | transcription regulator |
| FKHL18 | FOXS1 | forkhead box S1 | Nucleus | transcription regulator |
| FOXQ1 | FOXQ1 | forkhead box Q1 | Nucleus | transcription regulator |
| HUWE1 | HUWE1 | HECT, UBA and WWE domain containing 1, E3 ubiquitin protein ligase | Nucleus | transcription regulator |
| LBH | LBH | limb bud and heart development | Nucleus | transcription regulator |
| NEUROG3 | NEUROG3 | neurogenin 3 | Nucleus | transcription regulator |
| NFE2L1 | NFE2L1 | nuclear factor, erythroid 2-like 1 | Nucleus | transcription regulator |
| POLR2J2 | POLR2J2/POLR2J3 | polymerase (RNA) II (DNA directed) polypeptide J3 | Other | transcription regulator |
| SKI | SKI | SKI proto-oncogene | Nucleus | transcription regulator |
| SNAI2 | SNAI2 | snail family zinc finger 2 | Nucleus | transcription regulator |
| SOX1 | SOX1 | SRY (sex determining region Y)-box 1 | Nucleus | transcription regulator |
| SOX3 | SOX3 | SRY (sex determining region Y)-box 3 | Nucleus | transcription regulator |
| SUPT16H | SUPT16H | suppressor of Ty 16 homolog (S. cerevisiae) | Nucleus | transcription regulator |

| | | | | |
|---|---|---|---|---|
| TBX3 | TBX3 | T-box 3 | Nucleus | transcription regulator |
| TCF12 | TCF12 | transcription factor 12 | Nucleus | transcription regulator |
| TLE4 | TLE4 | transducin-like enhancer of split 4 | Nucleus | transcription regulator |
| ZFPM1 | ZFPM1 | zinc finger protein, FOG family member 1 | Nucleus | transcription regulator |
| ZKSCAN1 | ZKSCAN1 | zinc finger with KRAB and SCAN domains 1 | Nucleus | transcription regulator |
| ZNF206 | ZSCAN10 | zinc finger and SCAN domain containing 10 | Nucleus | transcription regulator |
| CPEB3 | CPEB3 | cytoplasmic polyadenylation element binding protein 3 | Cytoplasm | translation regulator |
| EEF1A1 | EEF1A1 | eukaryotic translation elongation factor 1 alpha 1 | Cytoplasm | translation regulator |
| CHRNB2 | CHRNB2 | cholinergic receptor, nicotinic, beta 2 (neuronal) | Plasma Membrane | transmembrane receptor |
| CXADR | CXADR | coxsackie virus and adenovirus receptor | Plasma Membrane | transmembrane receptor |
| LGR4 | LGR4 | leucine-rich repeat containing G protein-coupled receptor 4 | Plasma Membrane | transmembrane receptor |
| SRCRB4D | SSC4D | scavenger receptor cysteine rich family, 4 domains | Plasma Membrane | transmembrane receptor |
| AK055669 | TNPO1 | transportin 1 | Nucleus | transporter |
| BAX | BAX | BCL2-associated X protein | Cytoplasm | transporter |

| | | | | | |
|---|---|---|---|---|---|
| GOLGA3 | | GOLGA3 | golgin A3 | Cytoplasm | transporter |
| HNRPU | | HNRNPU | heterogeneous nuclear ribonucleoprotein U (scaffold attachment factor A) | Nucleus | transporter |
| KIF3B | | KIF3B | kinesin family member 3B | Cytoplasm | transporter |
| NRXN1 | | NRXN1 | neurexin 1 | Plasma Membrane | transporter |
| SLC8A1 | | SLC8A1 | solute carrier family 8 (sodium/calcium exchanger), member 1 | Plasma Membrane | transporter |
| SNAP25 | | SNAP25 | synaptosomal-associated protein, 25kDa | Plasma Membrane | transporter |
| SNX3 | | SNX3 | sorting nexin 3 | Cytoplasm | transporter |
| 2142362 | | ZFYVE16 | zinc finger, FYVE domain containing 16 | Nucleus | transporter |
| UCP3 | | UCP3 | uncoupling protein 3 (mitochondrial, proton carrier) | Cytoplasm | transporter |
| VPS13B | | VPS13B | vacuolar protein sorting 13 homolog B (yeast) | Nucleus | transporter |
| P185089 | unmapped | | | | |
| P280953 | unmapped | | | | |
| P210297 | unmapped | | | | |
| P144275 | unmapped | | | | |

| | |
|---|---|
| P152278 | unmapped |
| P164815 | unmapped |
| P195510 | unmapped |
| P24142 | unmapped |
| P264004 | unmapped |
| P264597 | unmapped |
| P307424 | unmapped |
| P324214 | unmapped |
| P324644 | unmapped |
| P358305 | unmapped |
| P375076 | unmapped |
| P384422 | unmapped |
| P417996 | unmapped |
| P461664 | unmapped |

| | |
|---|---|
| P641742 | unmapped |
| P714316 | unmapped |
| P7785 | unmapped |
| P780709 | unmapped |
| P853302 | unmapped |
| P922101 | unmapped |
| P927245 | unmapped |
| P928250 | unmapped |
| P101073 | unmapped |
| P112100 | unmapped |
| P114268 | unmapped |
| P1291 | unmapped |
| P132422 | unmapped |
| P171984 | unmapped |

| | |
|---|---|
| P230059 | unmapped |
| P231880 | unmapped |
| P233211 | unmapped |
| P61074 | unmapped |
| P65793 | unmapped |
| P71864 | unmapped |
| P77759 | unmapped |
| AF159295 | unmapped |
| AF293339 | unmapped |
| AK021866 | unmapped |
| AK022038 | unmapped |
| AK057820 | unmapped |
| AY029066 | unmapped |
| BM547196 | unmapped |

| | |
|---|---|
| C18orf23 | unmapped |
| C19orf31 | unmapped |
| CACNA11 | unmapped |
| CR607811 | unmapped |
| CR611094 | unmapped |
| CR616772 | unmapped |
| CR618720 | unmapped |
| DKFZp313P036 | unmapped |
| DKFZp547E087 | unmapped |
| ENST00000331458 | unmapped |
| ENST00000334083 | unmapped |
| ENST00000339054 | unmapped |
| ENST00000340049 | unmapped |
| ENST00000355411 | unmapped |

| | |
|---|---|
| ENST00000360262 | unmapped |
| FLJ00038 | unmapped |
| FLJ10490 | unmapped |
| FLJ13111 | unmapped |
| FLJ31413 | unmapped |
| FLJ36701 | unmapped |
| FLJ43692 | unmapped |
| HNRPA3 | unmapped |
| KIAA1327 | unmapped |
| KIAA1545 | unmapped |
| KIAA1856 | unmapped |
| 284072 | unmapped |
| 340947 | unmapped |
| 399839 | unmapped |

| | |
|---|---|
| 440504 | unmapped |
| 441421 | unmapped |
| M14087 | unmapped |
| MGC10334 | unmapped |
| MGC13098 | unmapped |
| MGC3121 | unmapped |
| NP111687 | unmapped |
| PRO1855 | unmapped |
| S81524 | unmapped |
| 2053095 | unmapped |
| 2057861 | unmapped |
| 2071427 | unmapped |
| 2080894 | unmapped |
| 2087262 | unmapped |

| | |
|---|---|
| 2097260 | unmapped |
| 2098338 | unmapped |
| 2099668 | unmapped |
| 2105947 | unmapped |
| 2122491 | unmapped |
| 2134185 | unmapped |
| 2145096 | unmapped |
| 2172359 | unmapped |
| 2174081 | unmapped |
| 2175925 | unmapped |
| 2182662 | unmapped |
| 2199258 | unmapped |
| 2206574 | unmapped |
