# Supplementary material for: Multiomics-Based Signaling Pathway Network Alterations in Human Non-functional Pituitary Adenomas
Source: Front Endocrinol (Lausanne). 2019 Dec 17;10:835. doi: 10.3389/fendo.2019.00835 (PMC6928143; doi:10.3389/fendo.2019.00835)

Supplemental materials 5.3
Statistically significant canonical pathways derived from differentially expressed genes between invasive and non-invasive NFPAs for IPA analysis (Dataset 5)

1. eNOS signaling
2. Cardiac beta-adrenergic signaling
3. IGF-1 signaling
4. mTOR signaling
5. Nitric oxide signaling in cardiavascular system
6. Aldosterone signaling in epithelial cells
7. Growth hormone signaling
8. FLT3 signaling hematopoietic progenitor cells
9. PI3K-AKT signaling
10. Docosahexaenoic acid (DHA) signaling
11. Prostate cancer signaling
12. ILK signaling
13. EIF2 signaling
14. ERK-MAPK signaling
15. Virus entry via endocytic pathways
16. Aryl hydrocarbon receptor signaling
17. Endometrial cancer signaling
18. p53 signaling
19. AMPK signaling
20. Telomerase signaling
21. Myc mediated apoptosis signaling
22. Amyotrophic lateral sclerosis signaling
23. Estrogen-mediate S-phase entry
24. NGF signaling
25. Hypoxia signaling in the cardiavascular system
26. Huntington's disease signaling
27. PEDF signaling
28. Calcium signaling
29. Acute myeloid leukemia signaling
30. Protein ubiquitination pathway

Dataset 5-Canonical pathway Chart

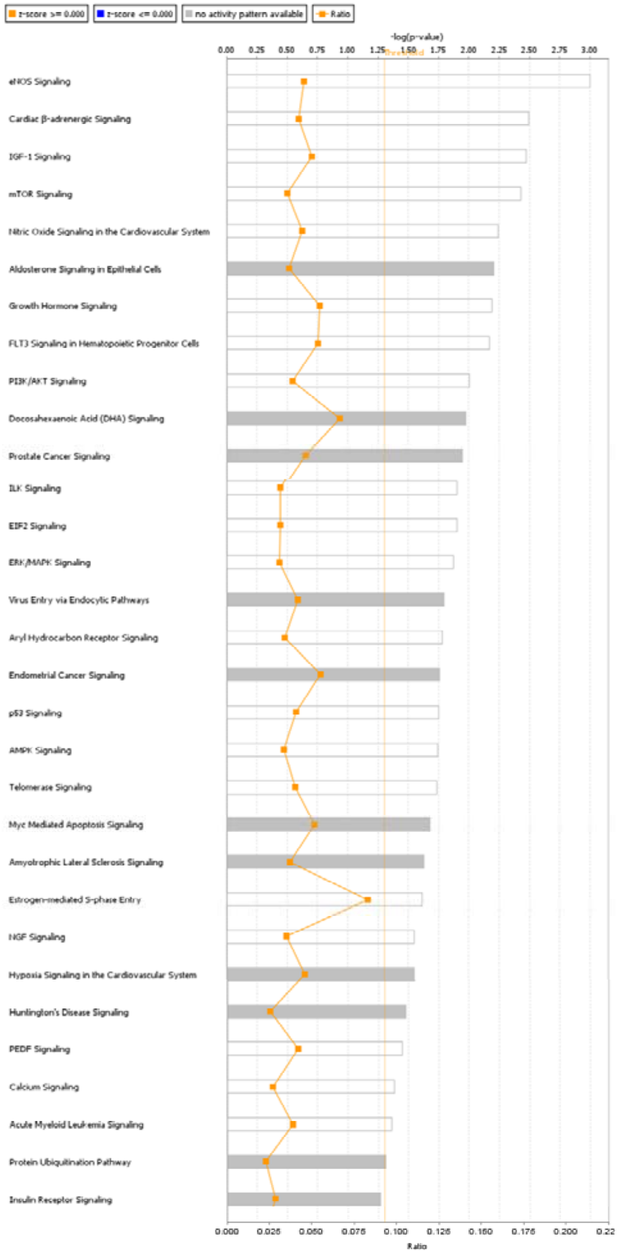

1-eNOS signaling

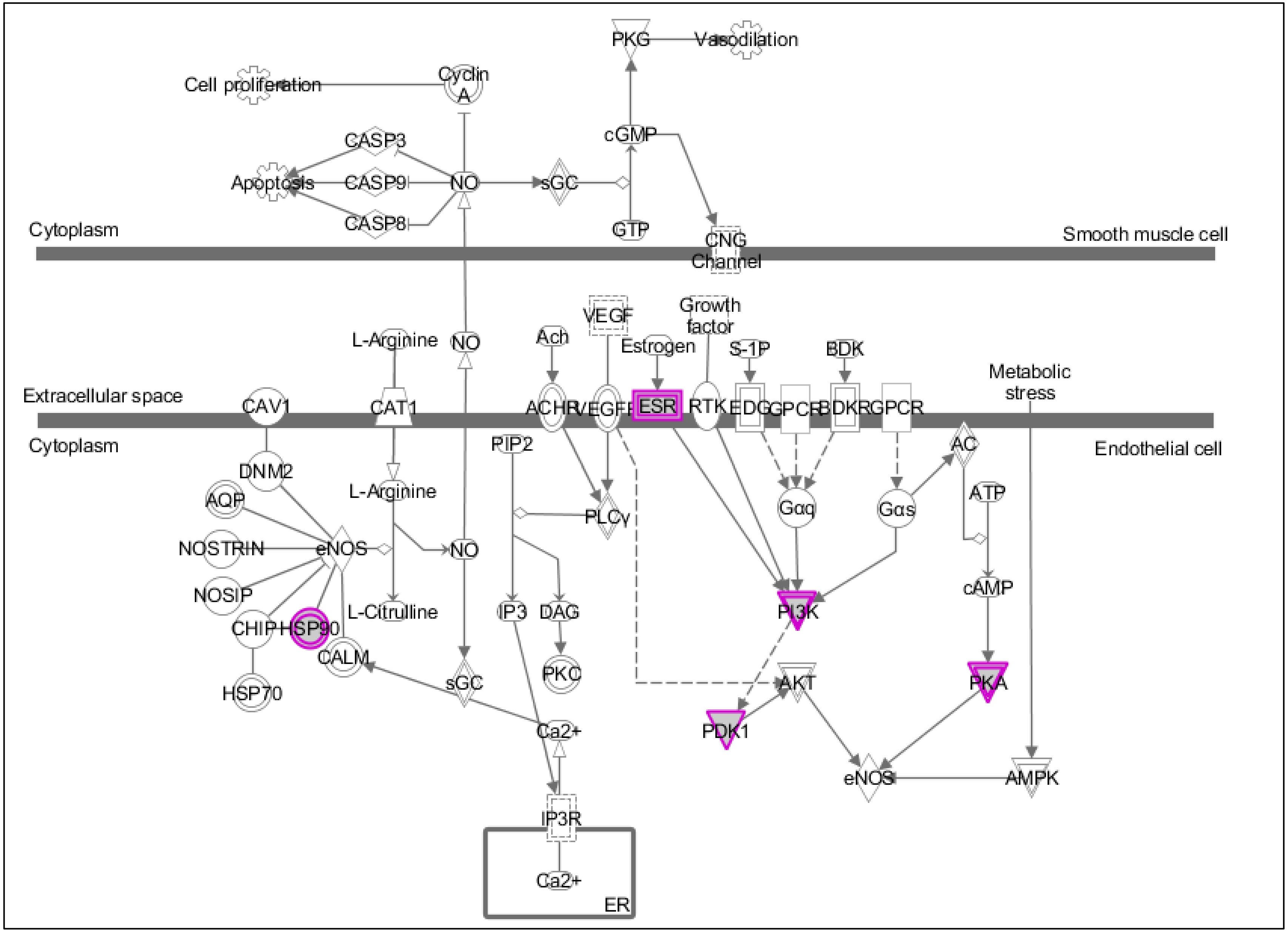

2-Cardiac beta-adrenergic signaling

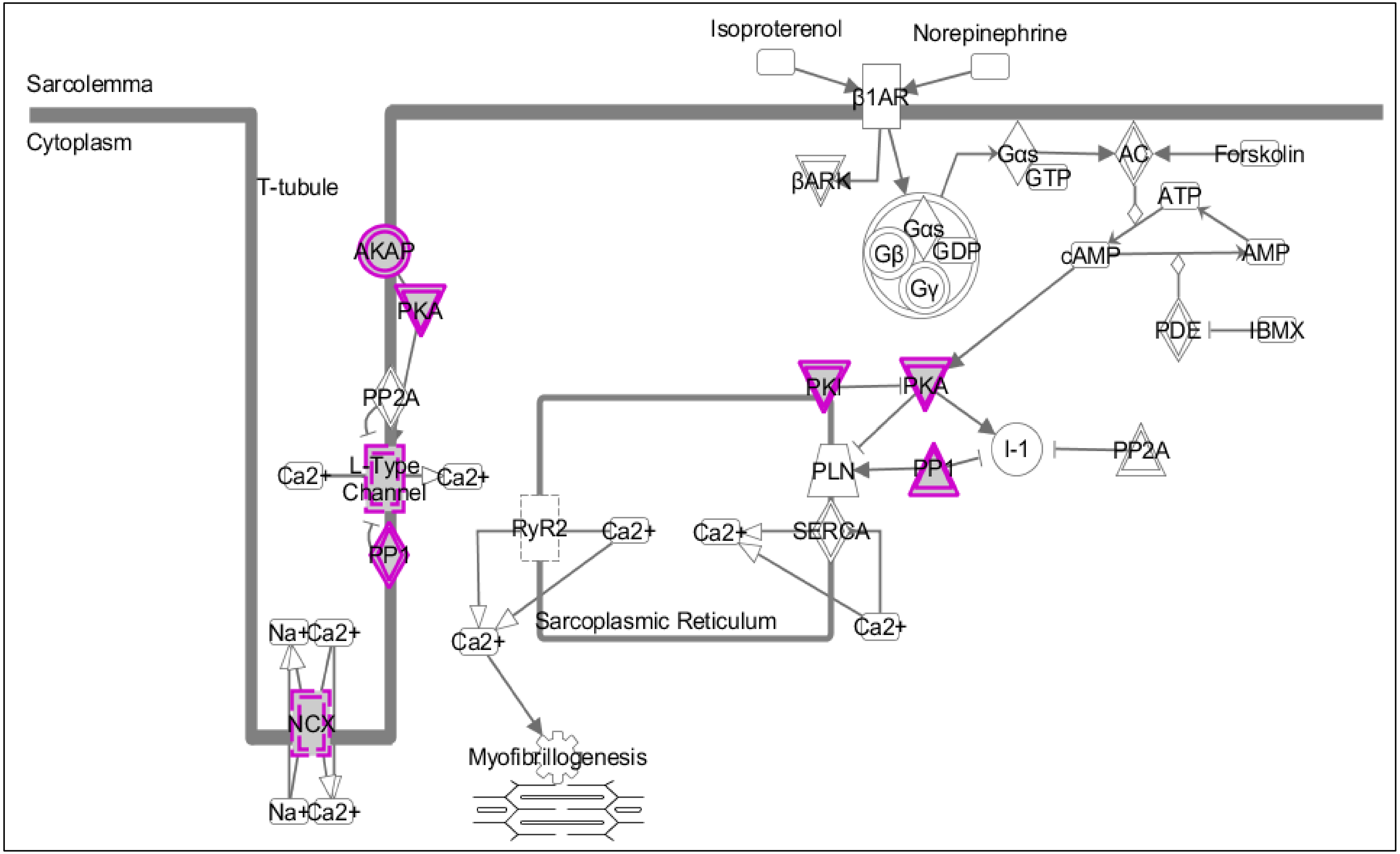

3-IGF-1 signaling

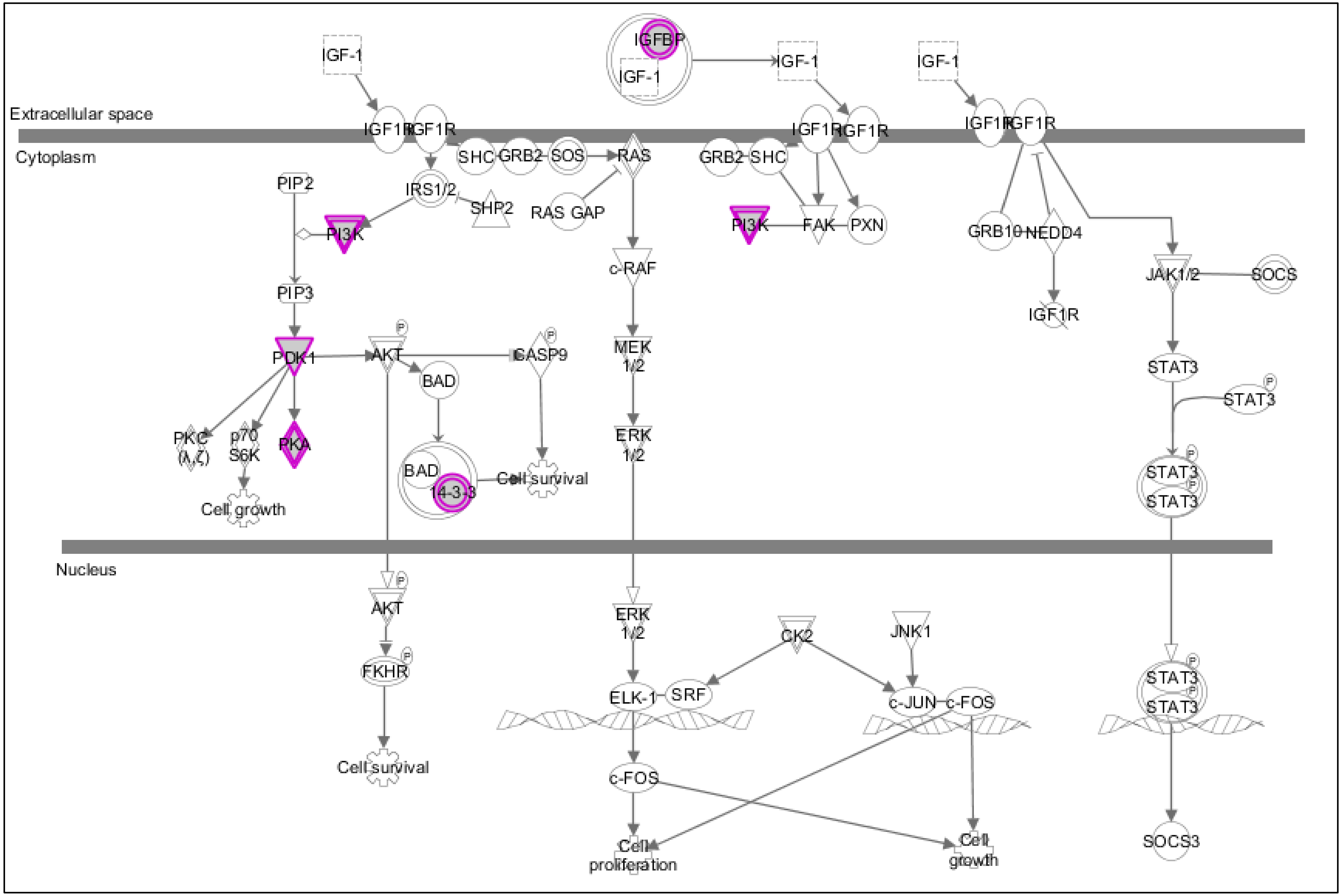

4-mTOR signaling

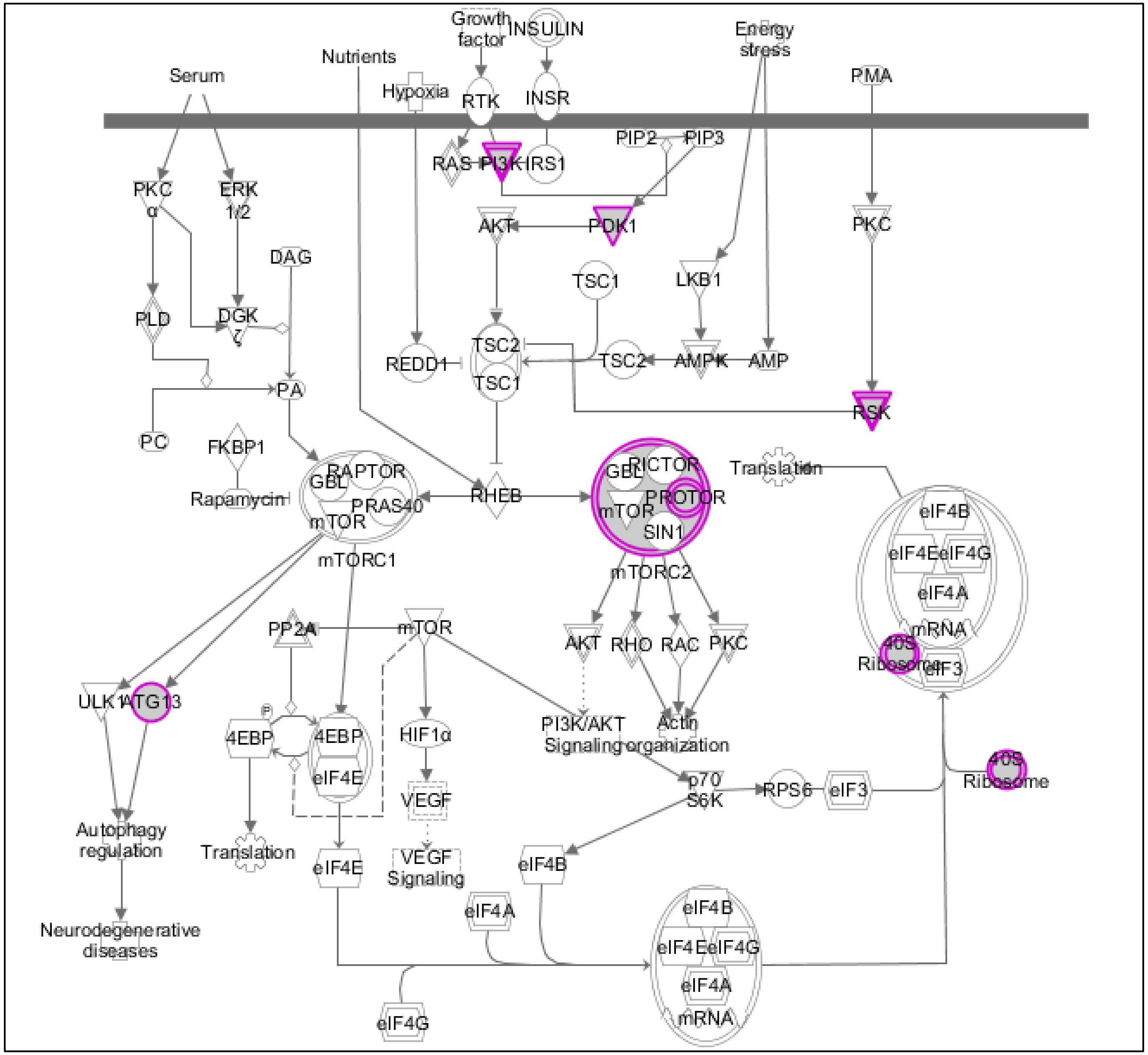

5-Nitric oxide signaling in cardiavascular system

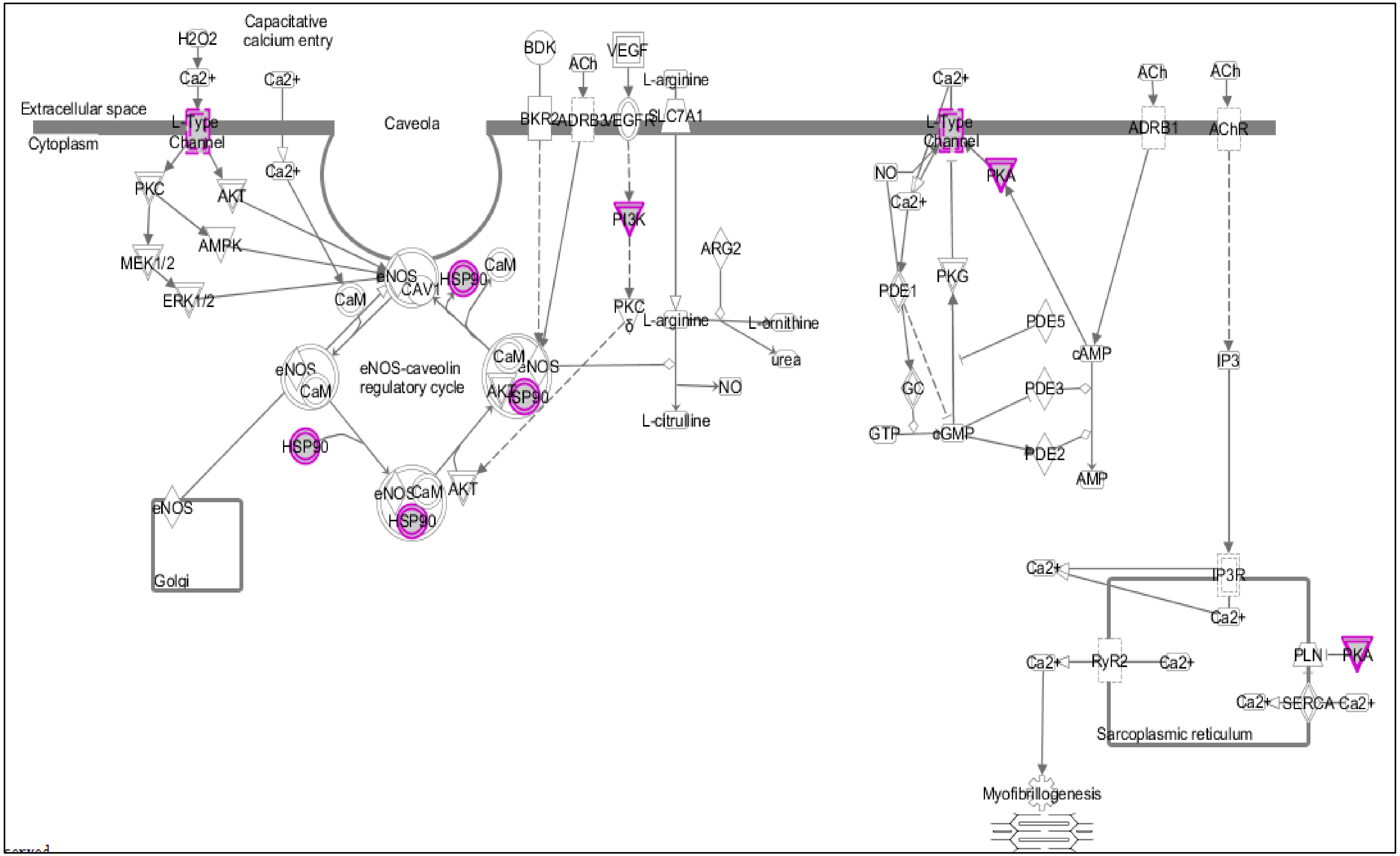

6-Aldosterone signaling in epithelial cells

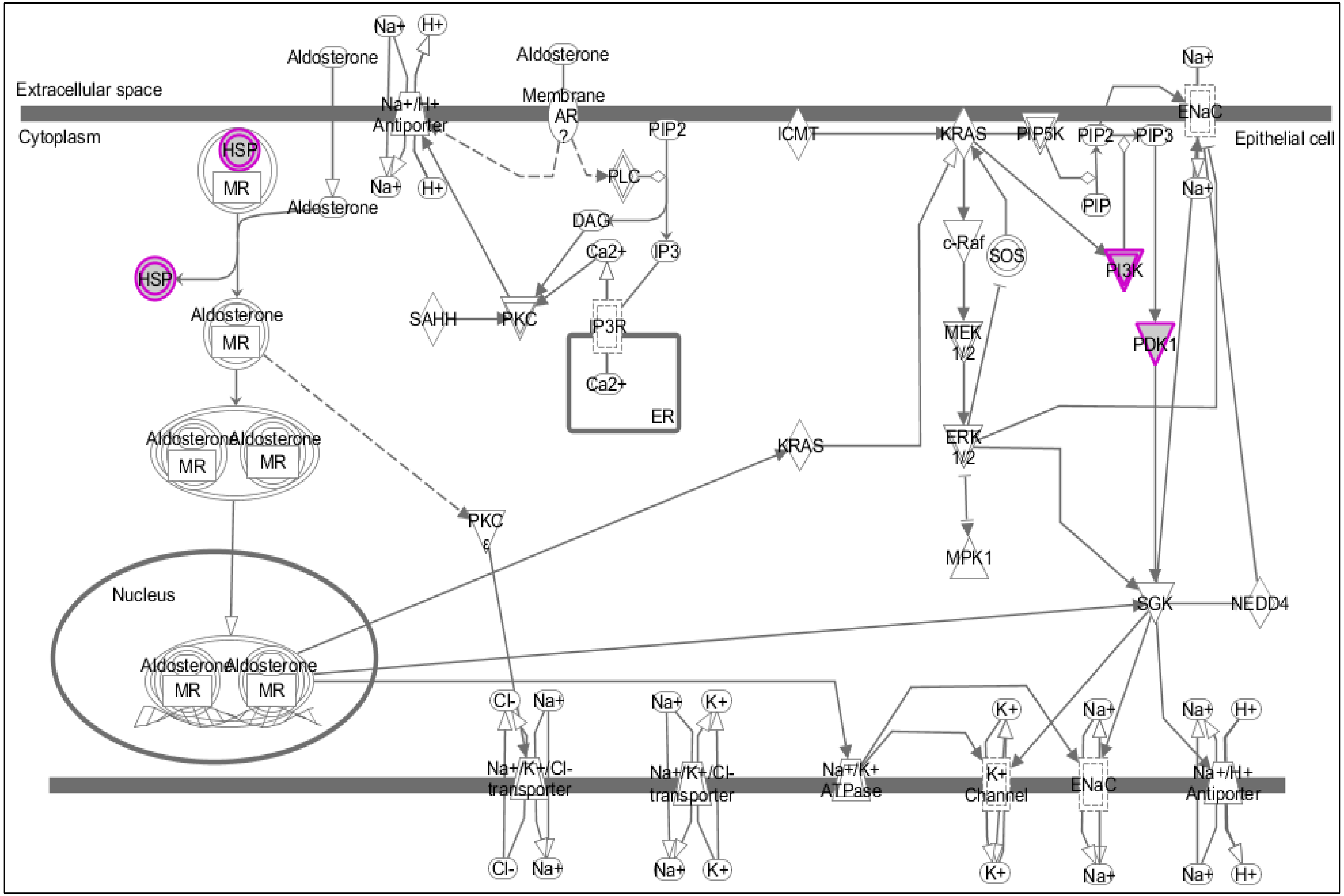

7-Growth hormone signaling

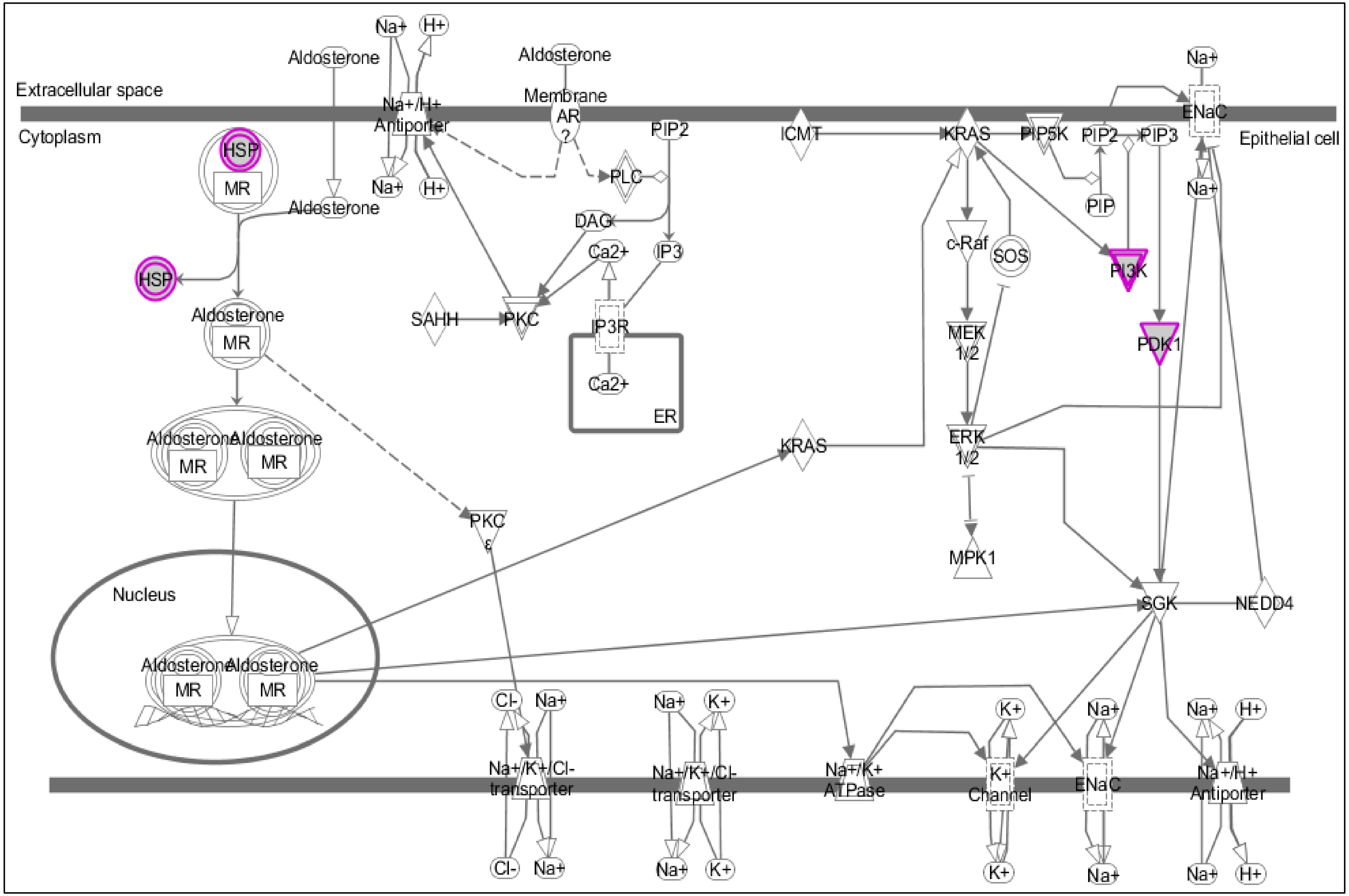

8-FLT3 signaling hematopoietic progenitor cells

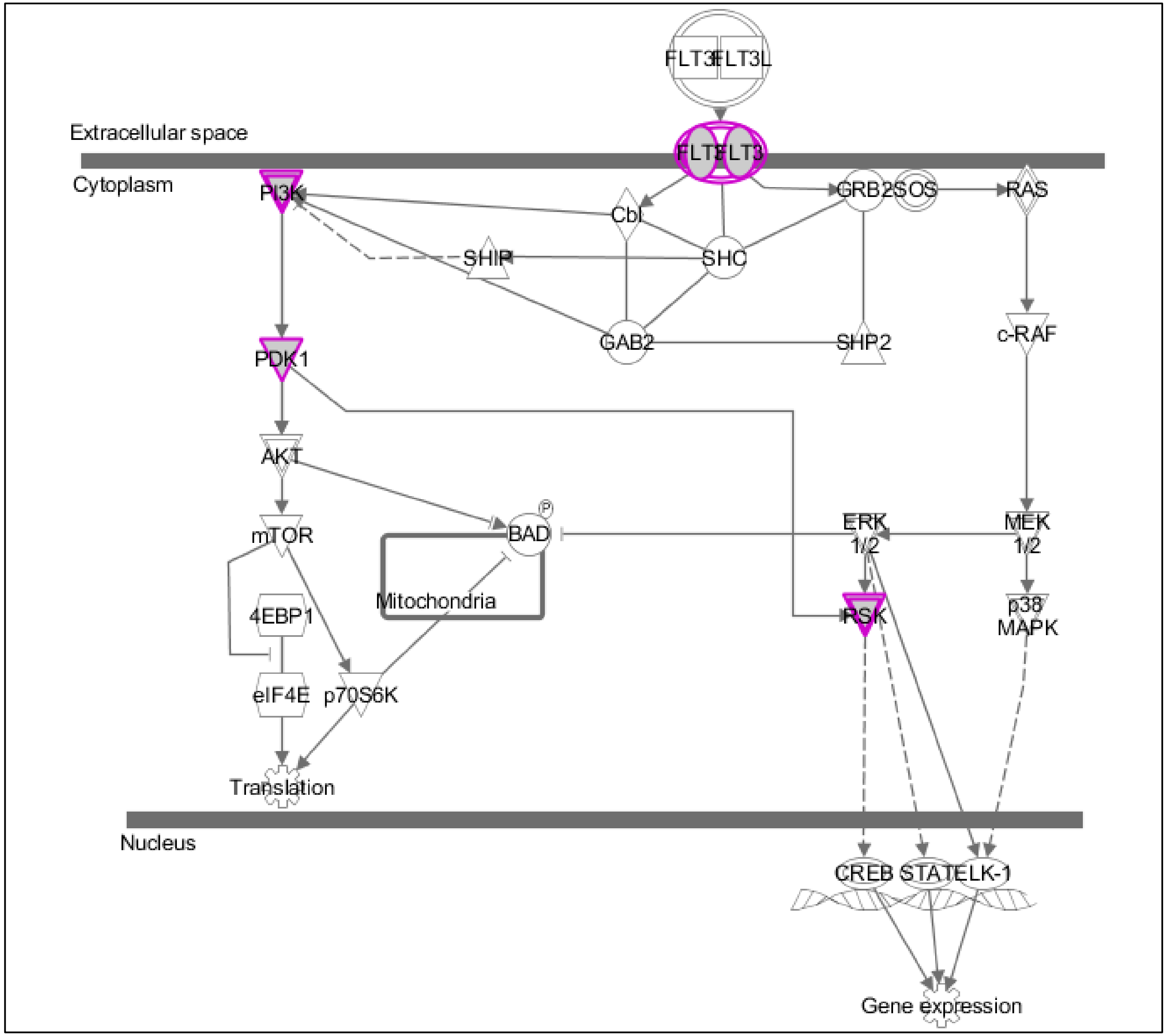

9-PI3K-AKT signaling

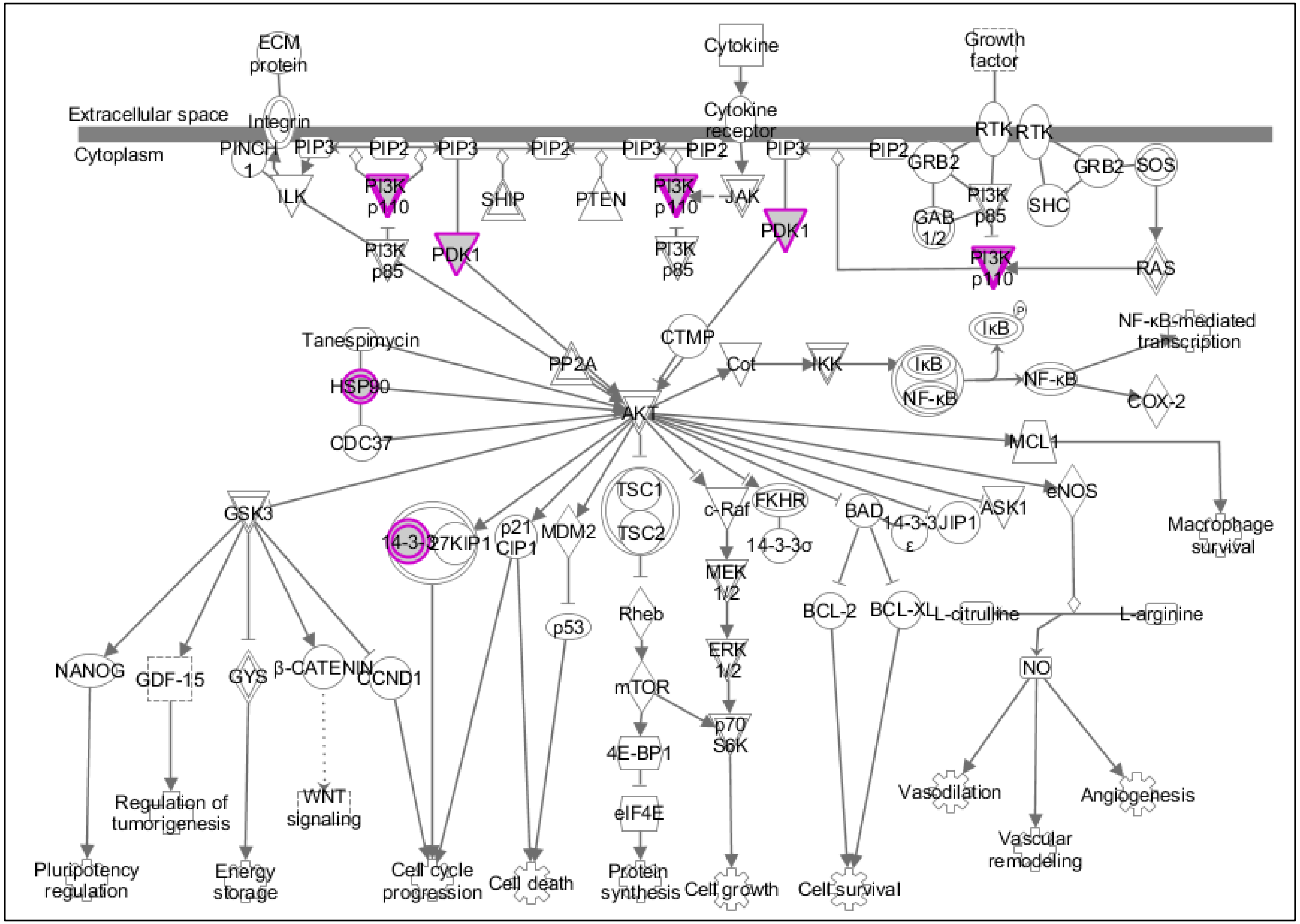

10-Docosahexaenoic acid (DHA) signaling

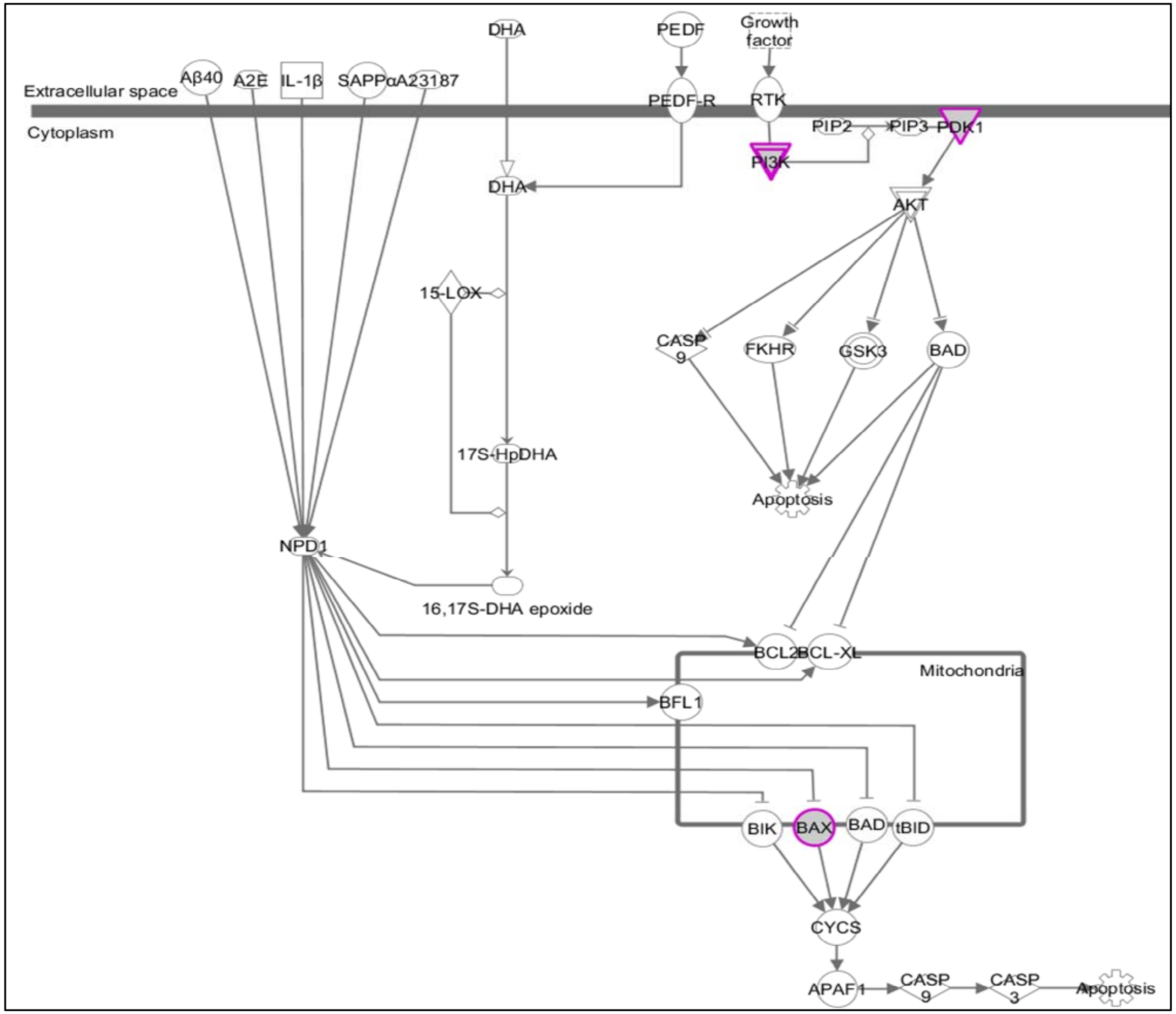

11-Prostate cancer signaling

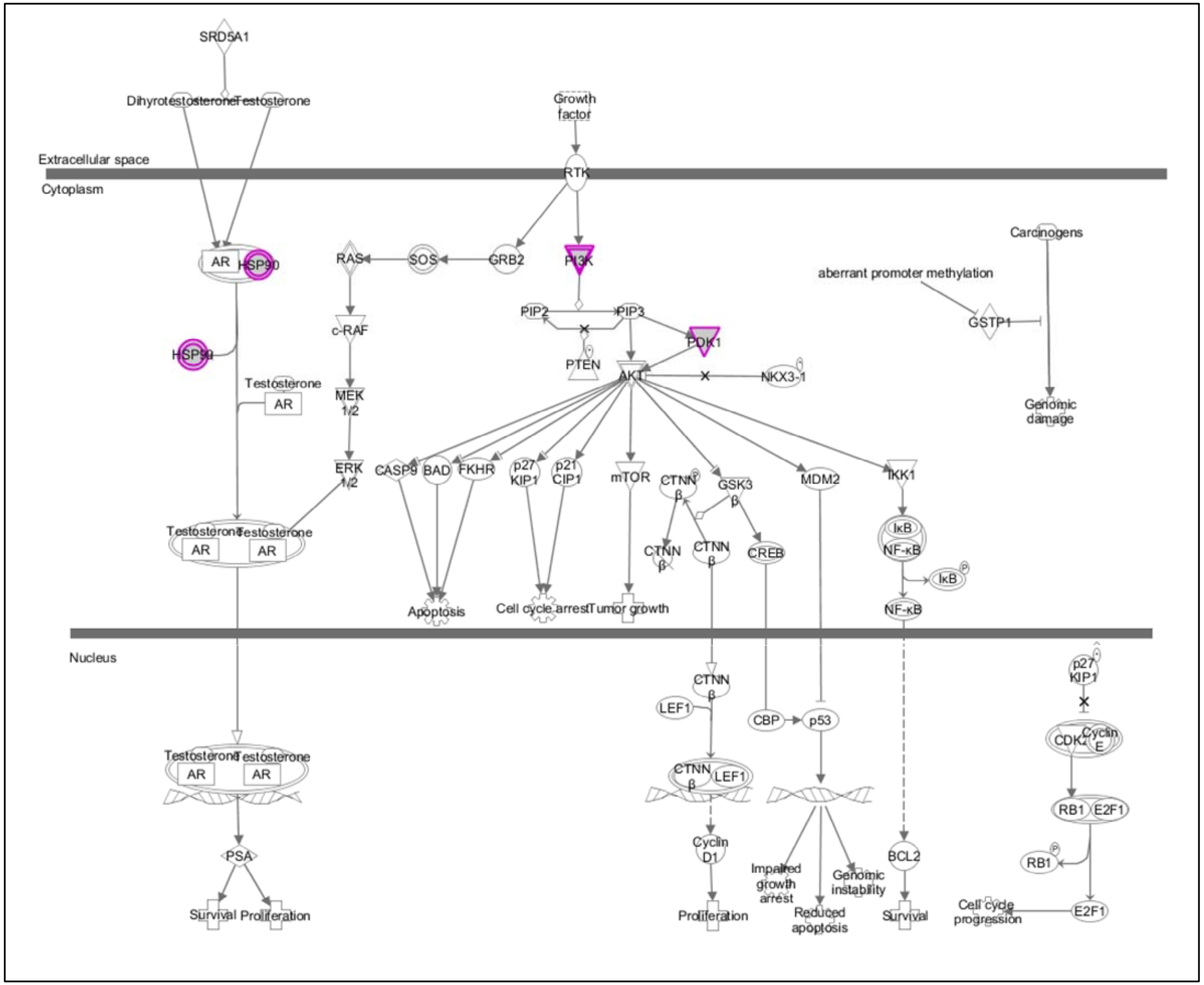

12-ILK signaling

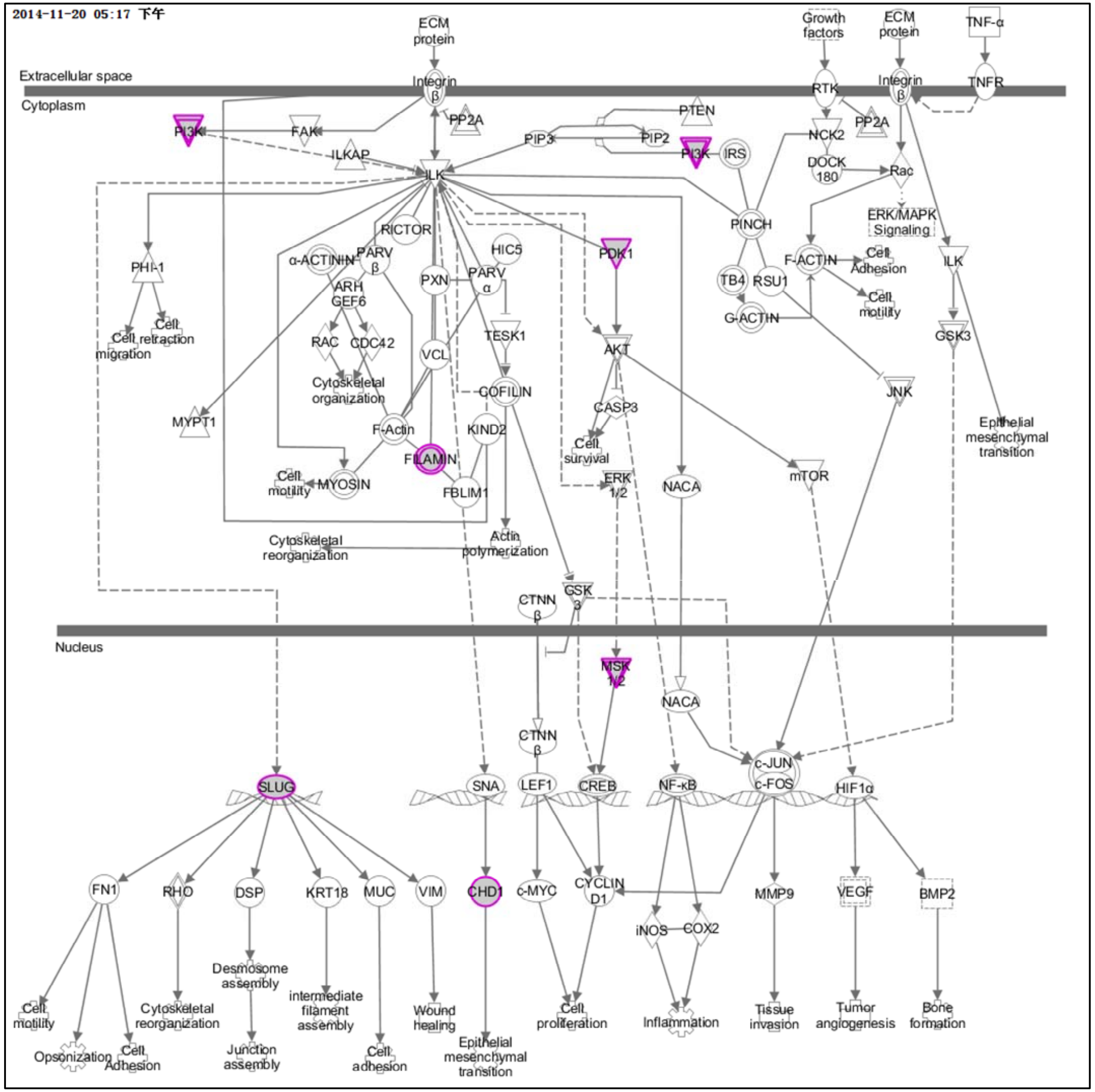

13-EIF2 signaling

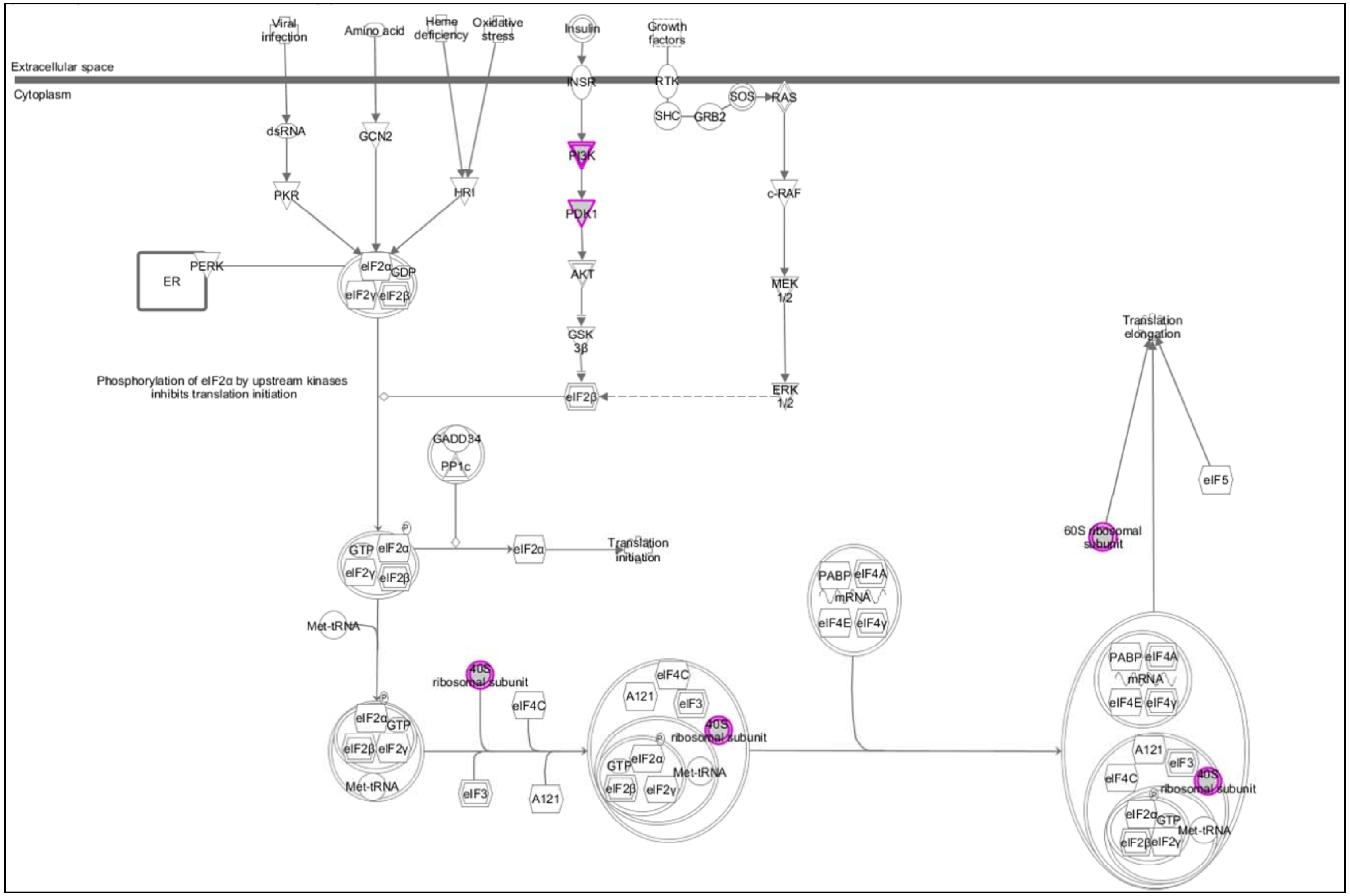

14-ERK-MAPK signaling

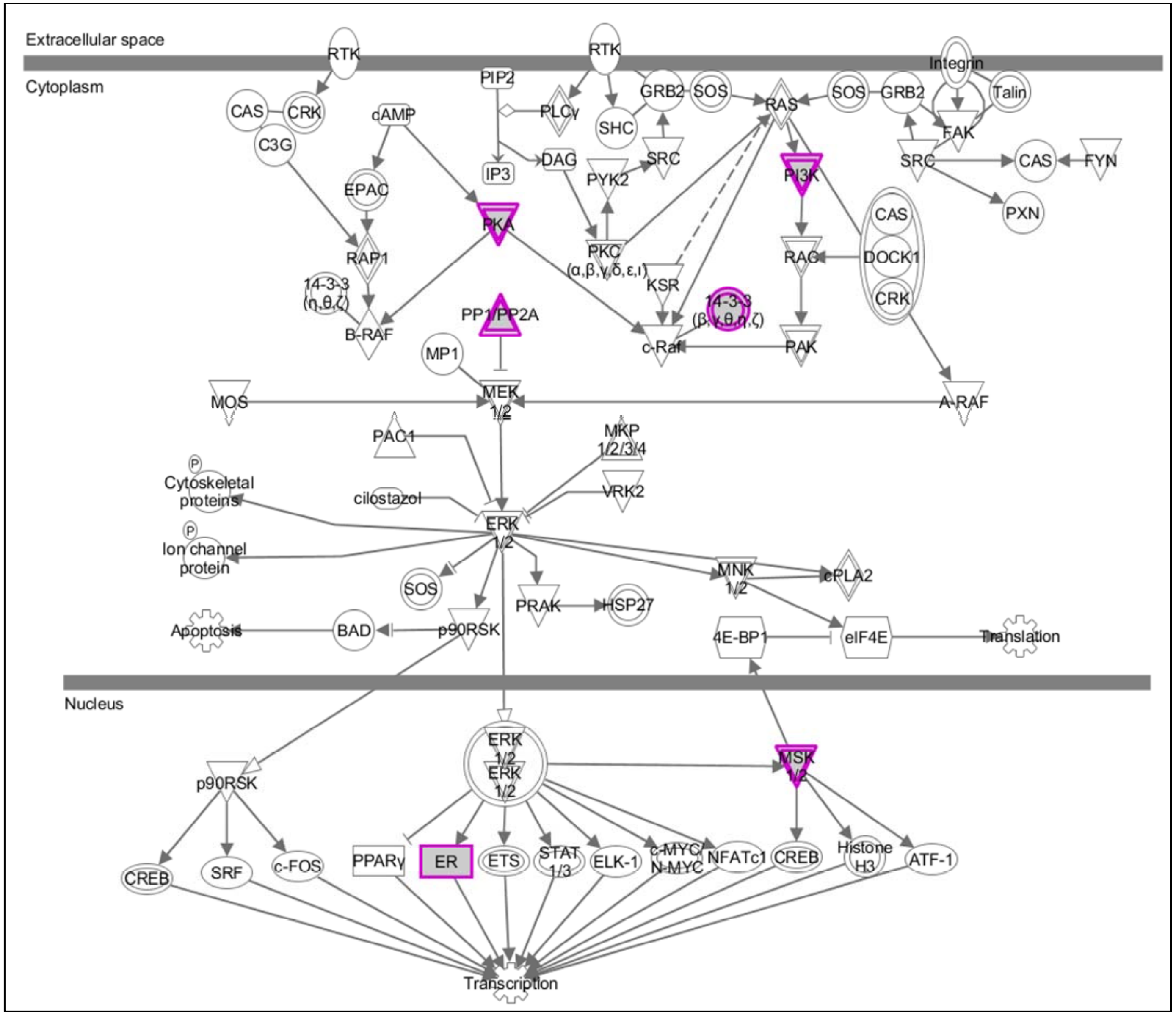

15-Virus entry via endocytic pathways

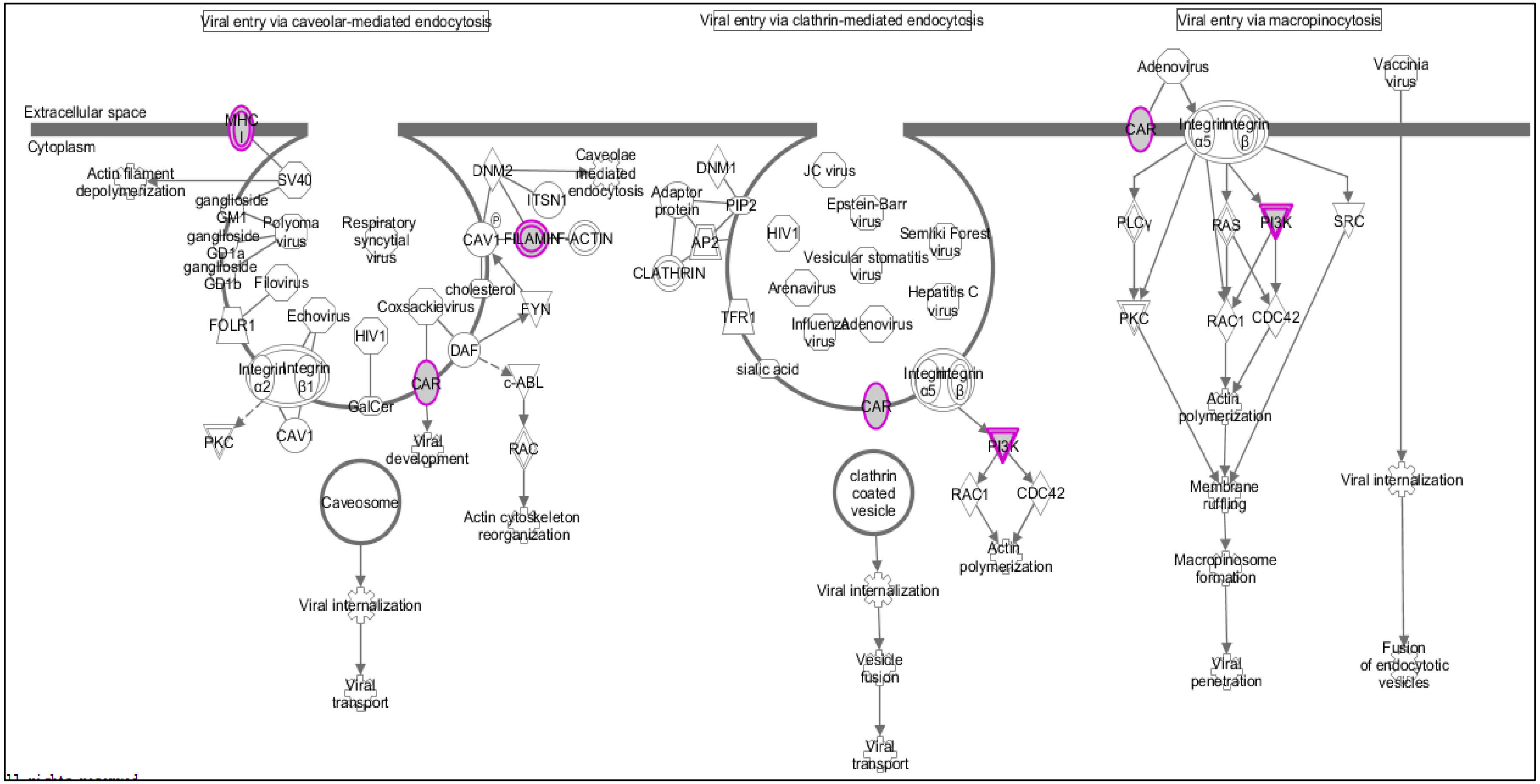

16-Aryl hydrocarbon receptor signaling

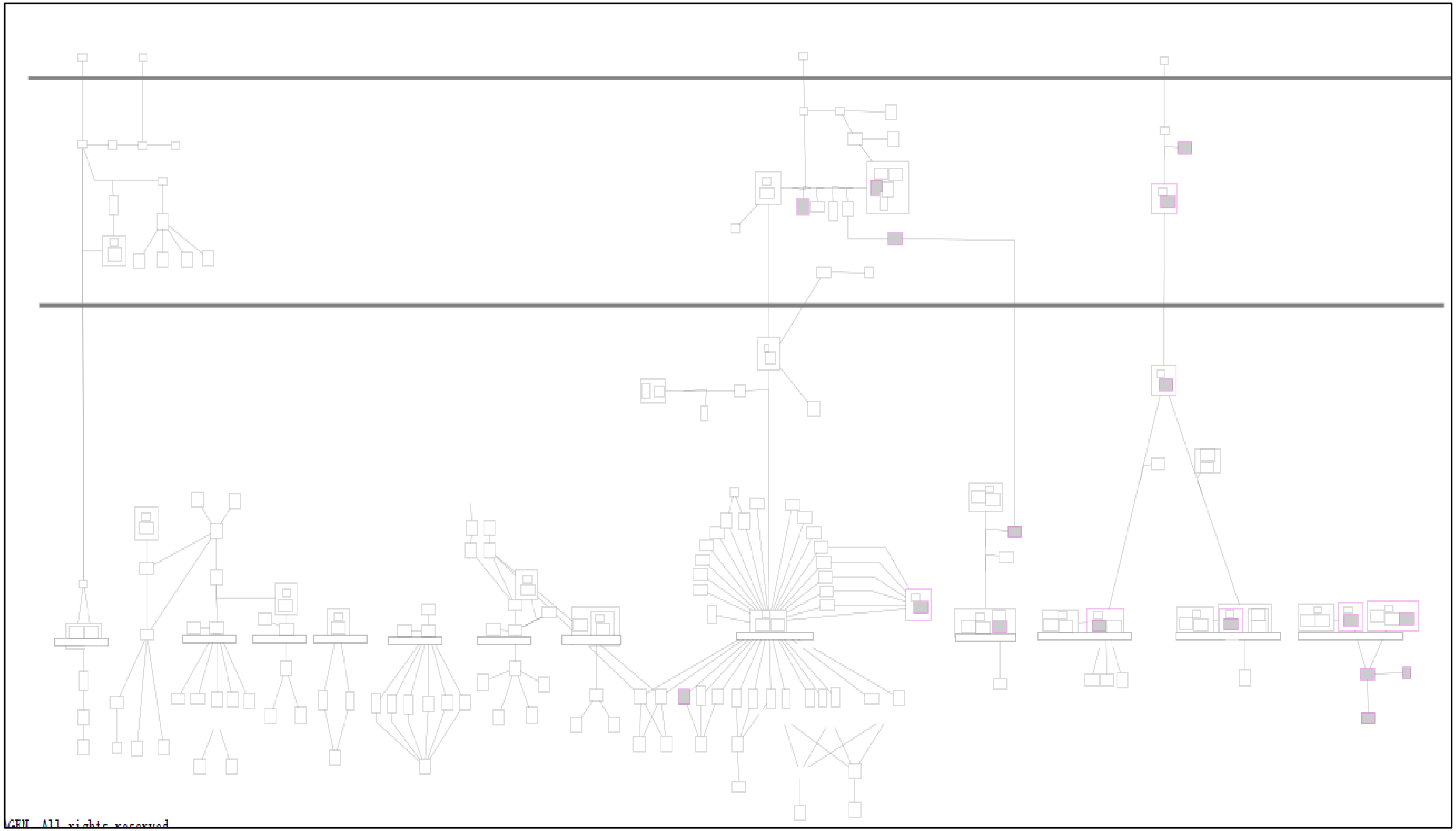

17-Endometrial cancer signaling

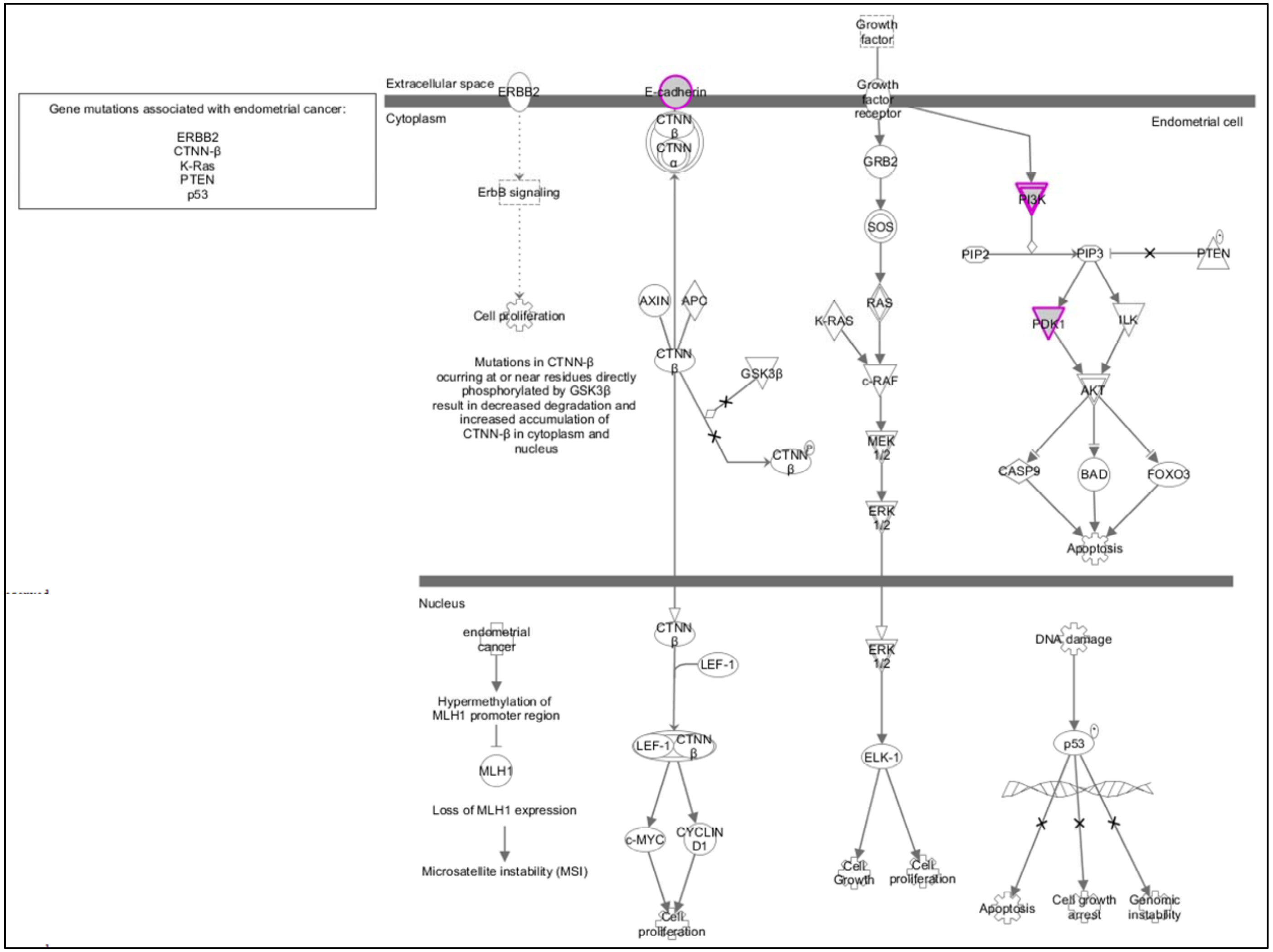

18-p53 signaling

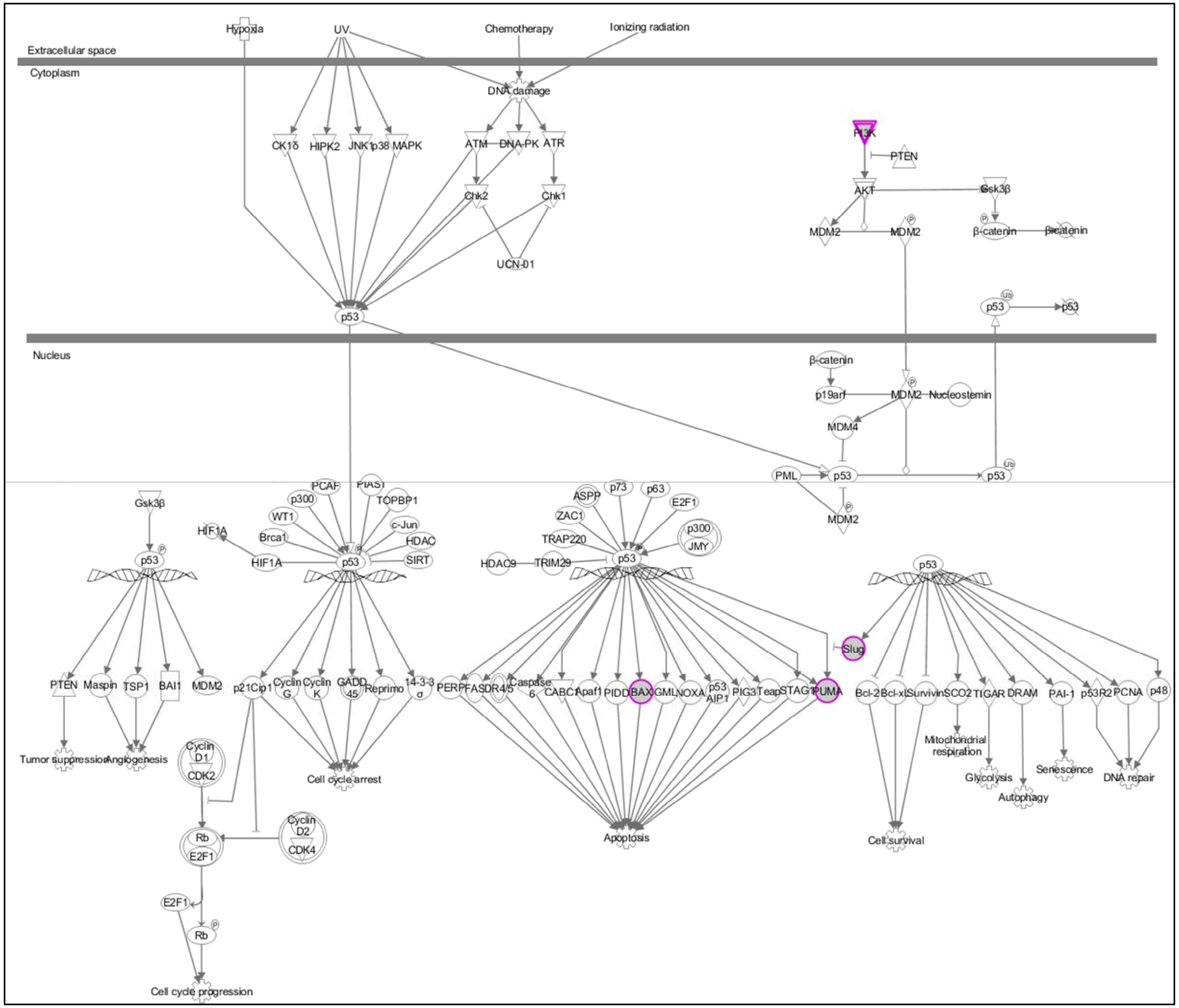

19-AMPK signaling

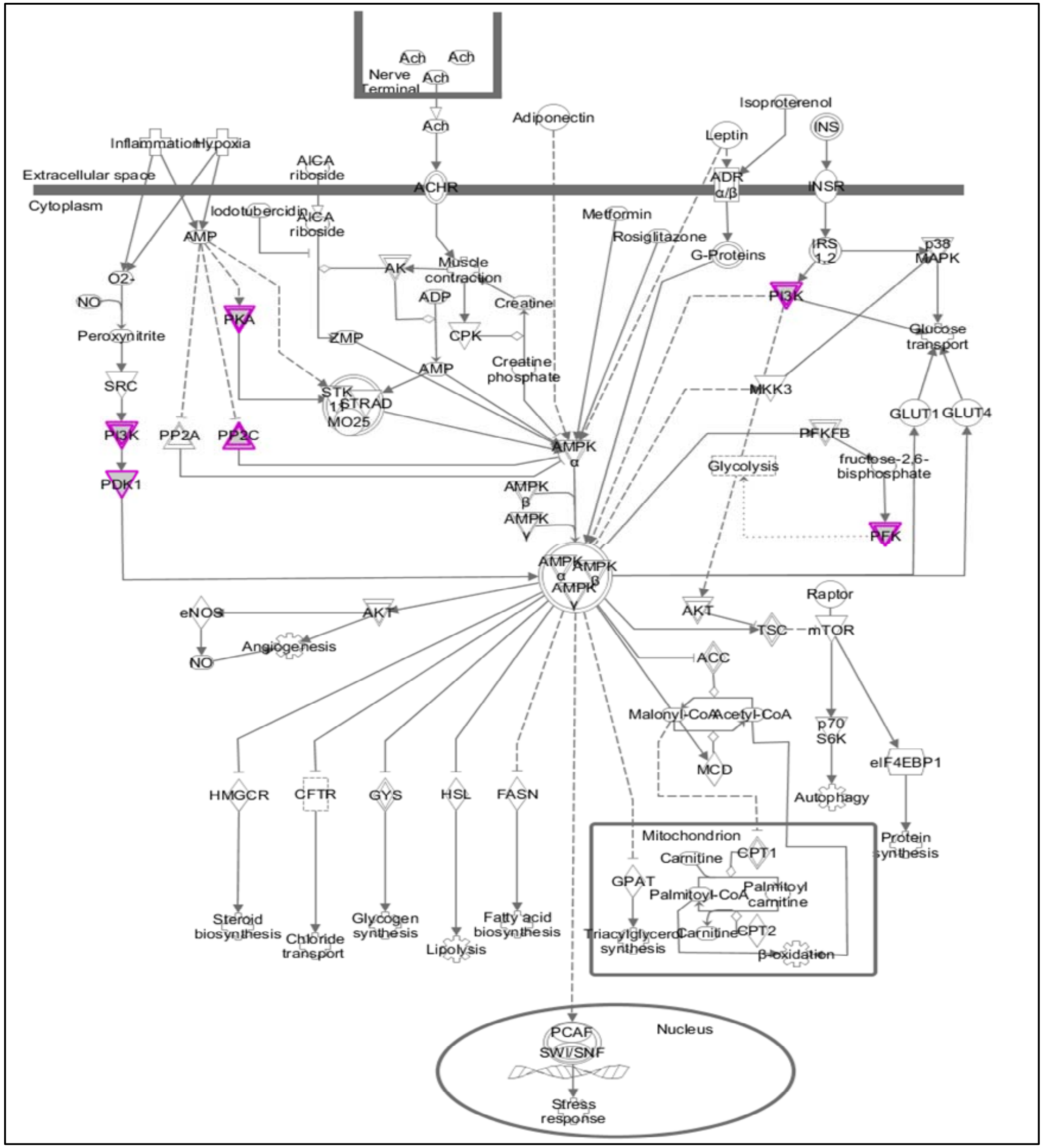

20-Telomerase signaling

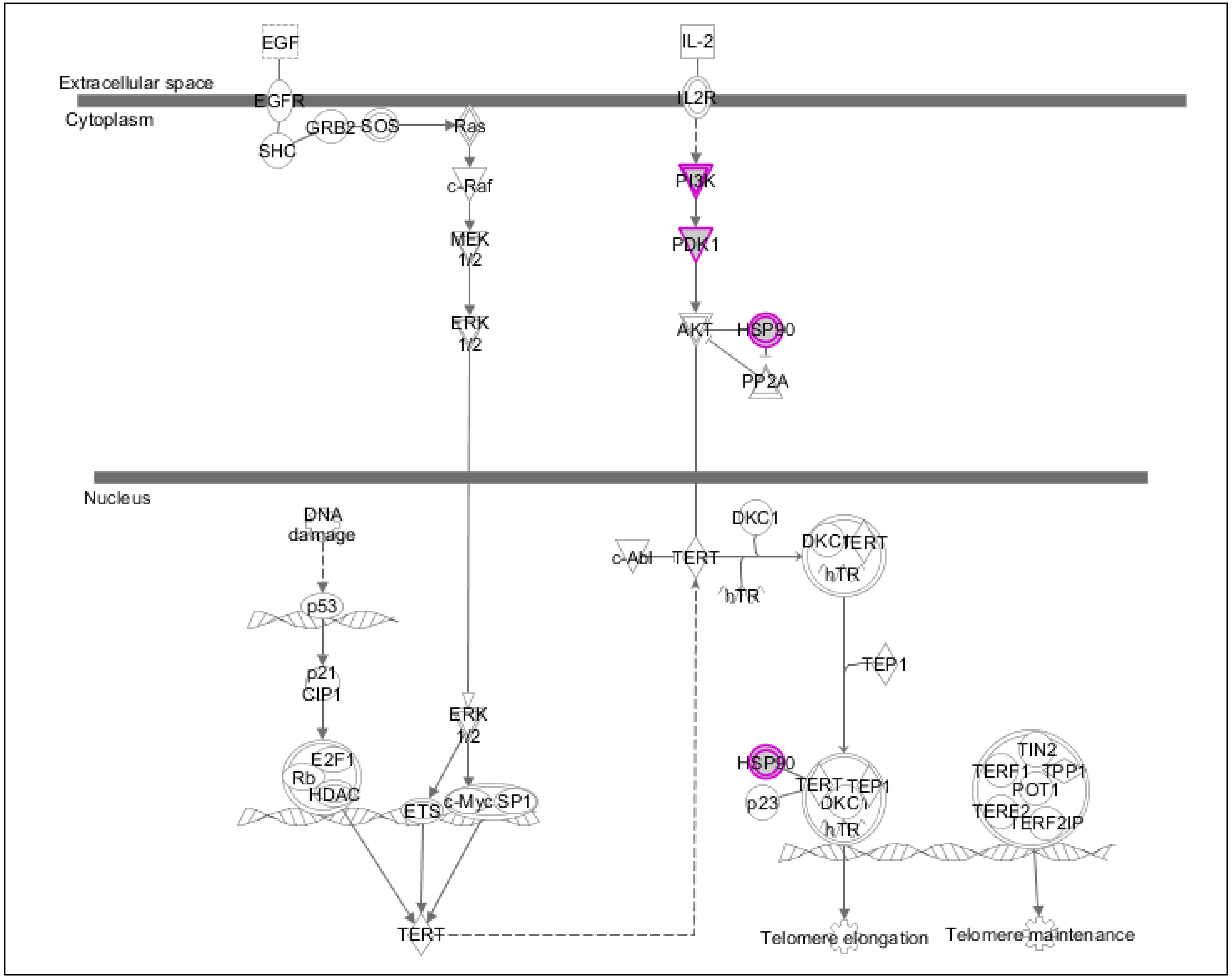

21-Myc mediated apoptosis signaling

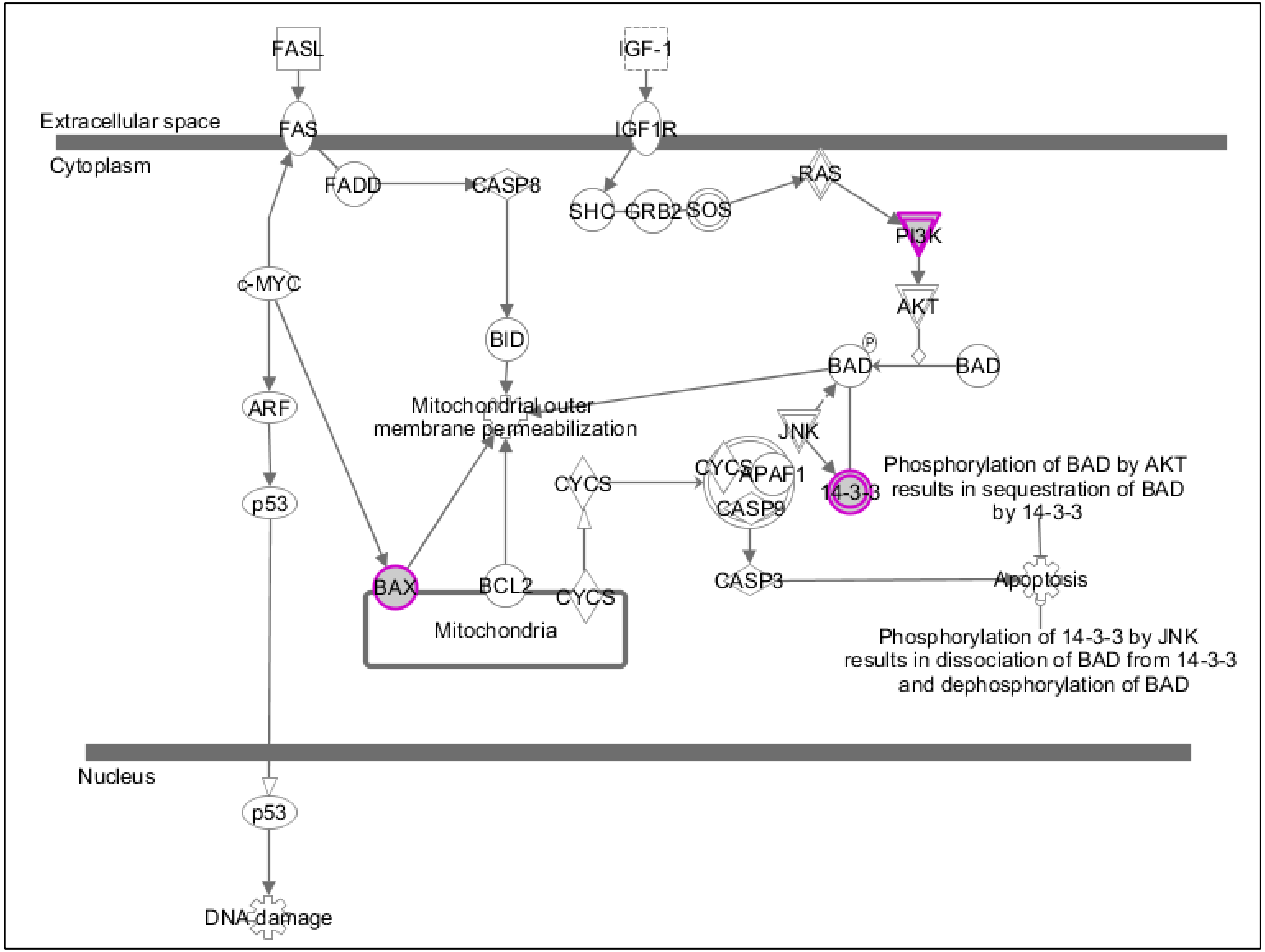

22-Amyotrophic lateral sclerosis signaling

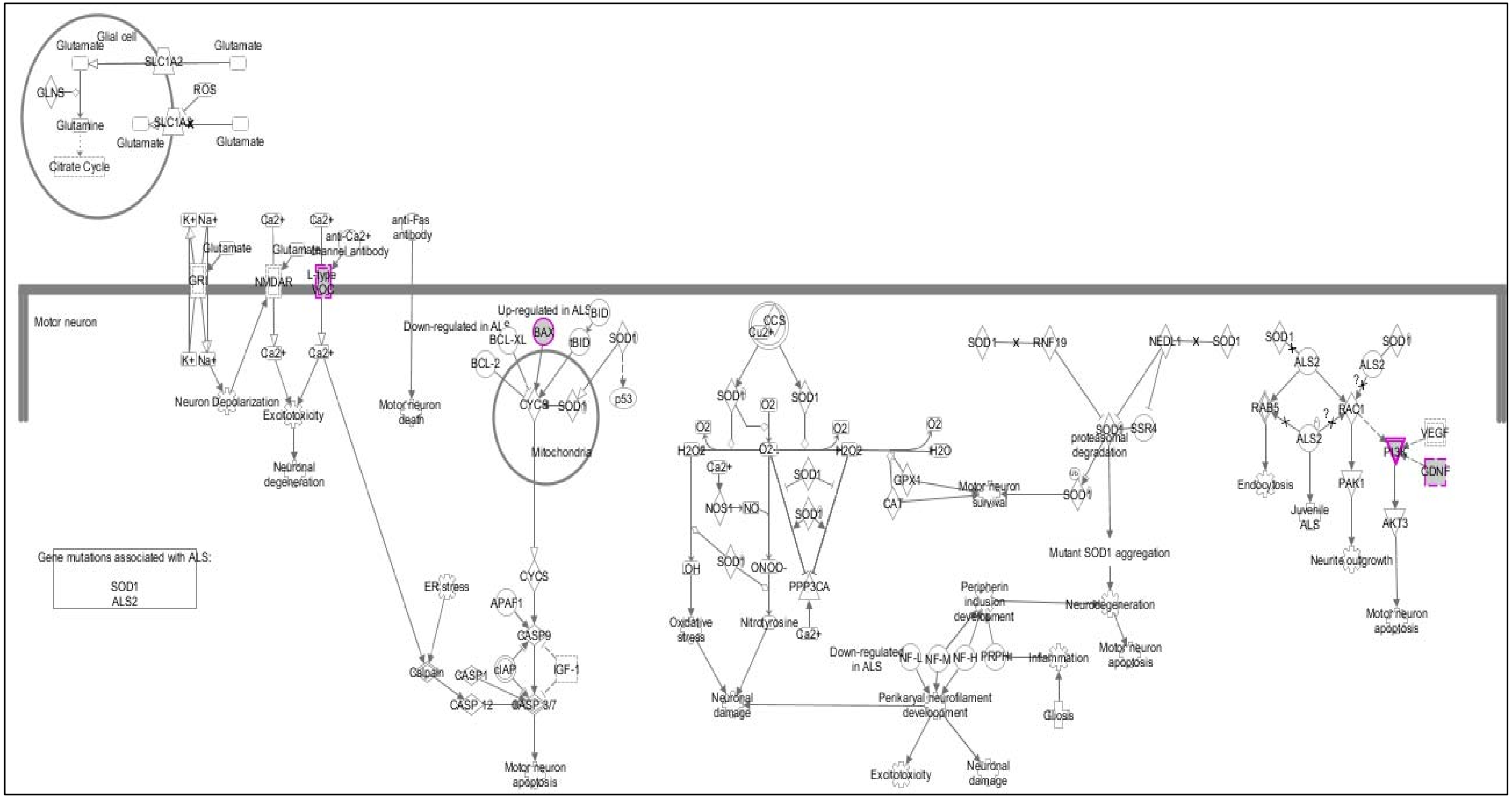

23-Estrogen-mediate S-phase entry

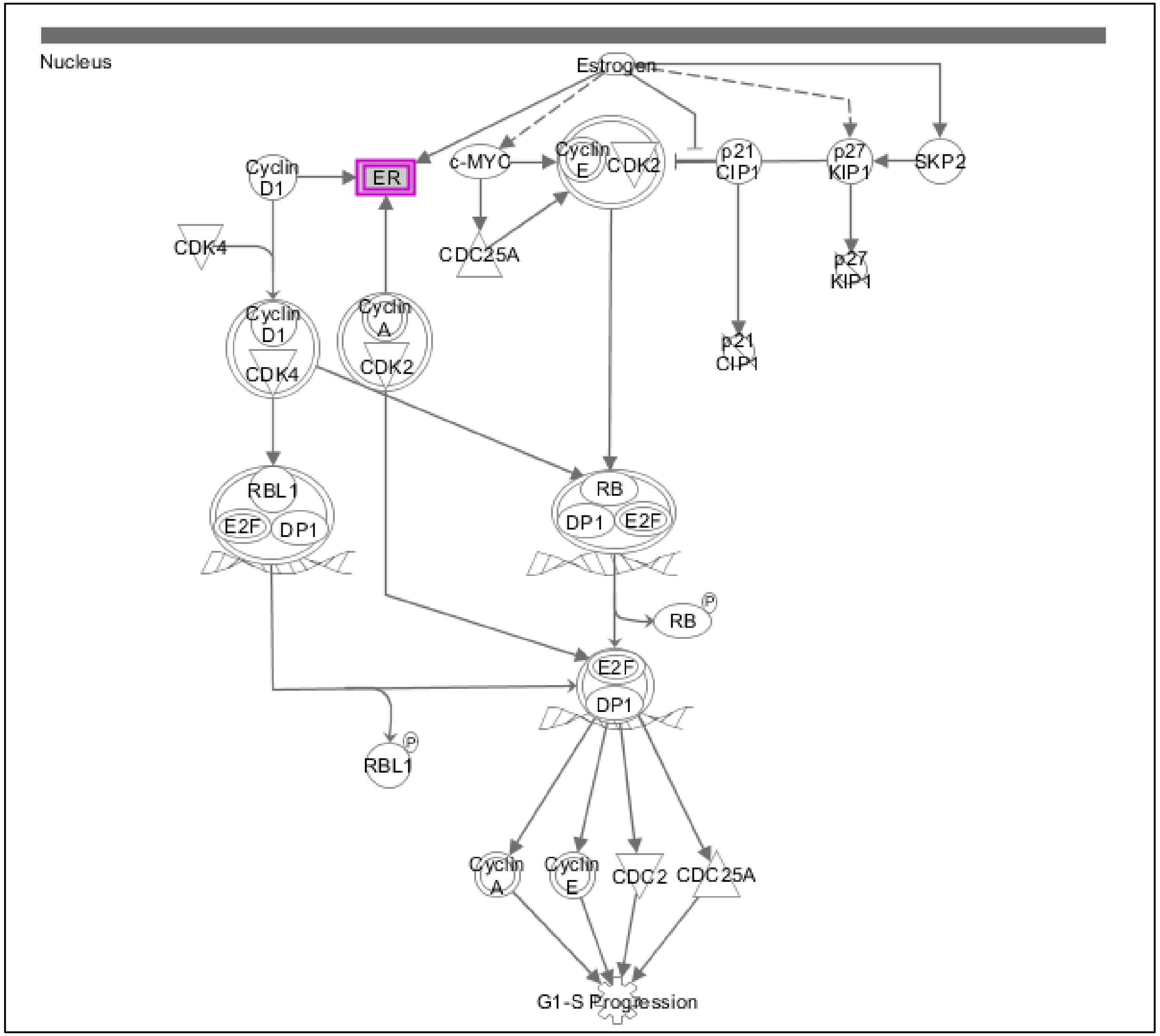

24-NGF signaling

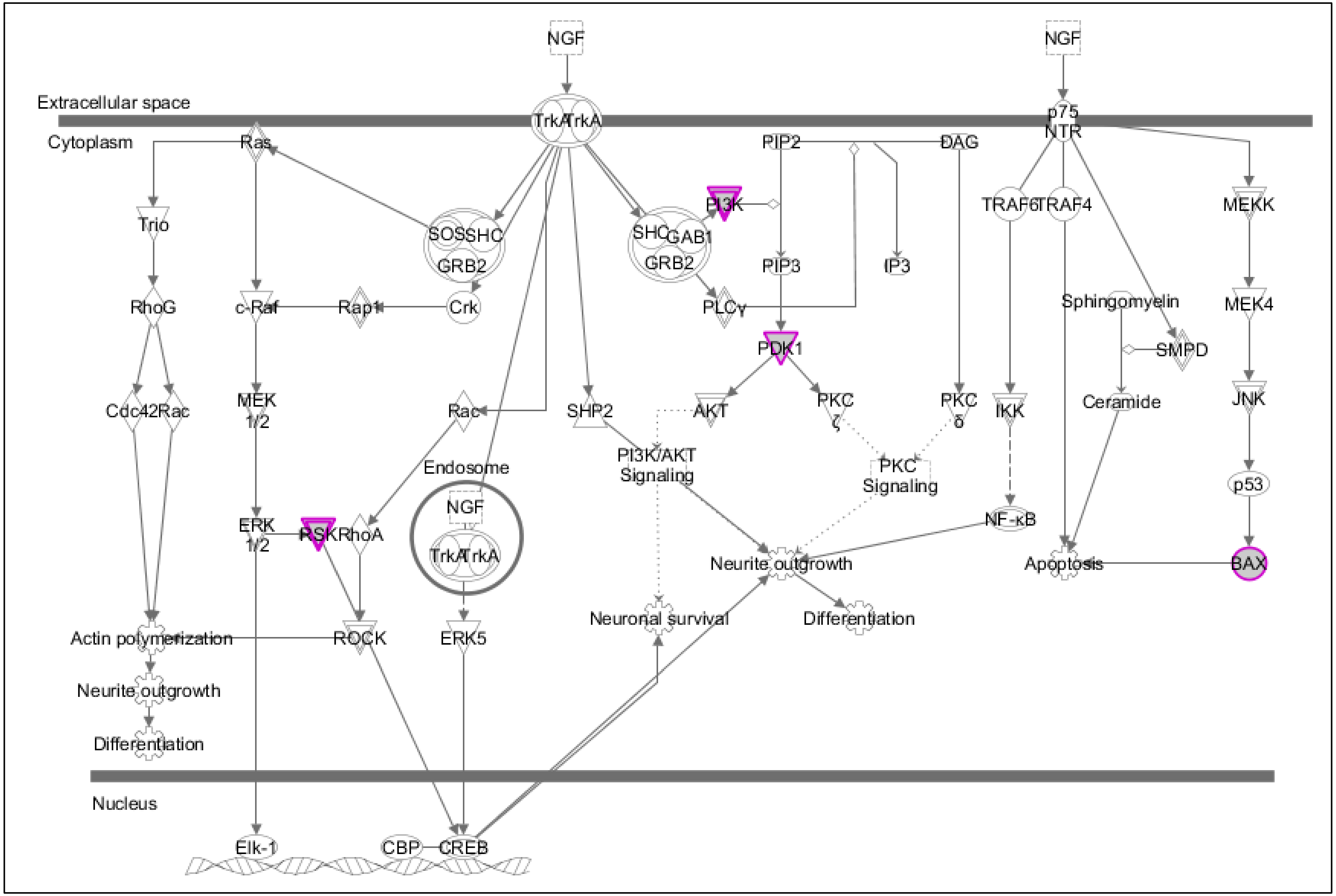

25-Hypoxia signaling in the cardiavascular system

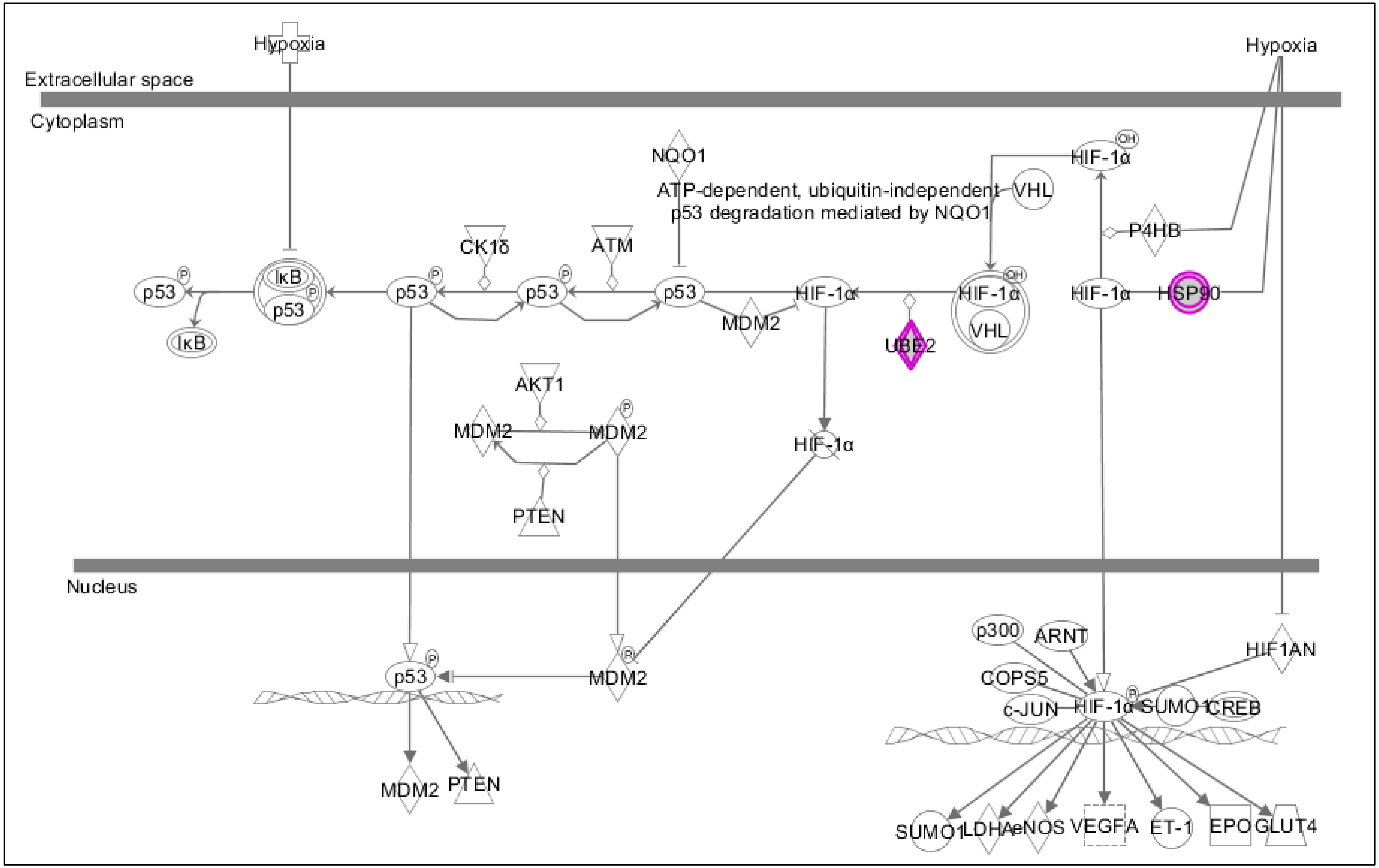

26-Huntington's disease signaling

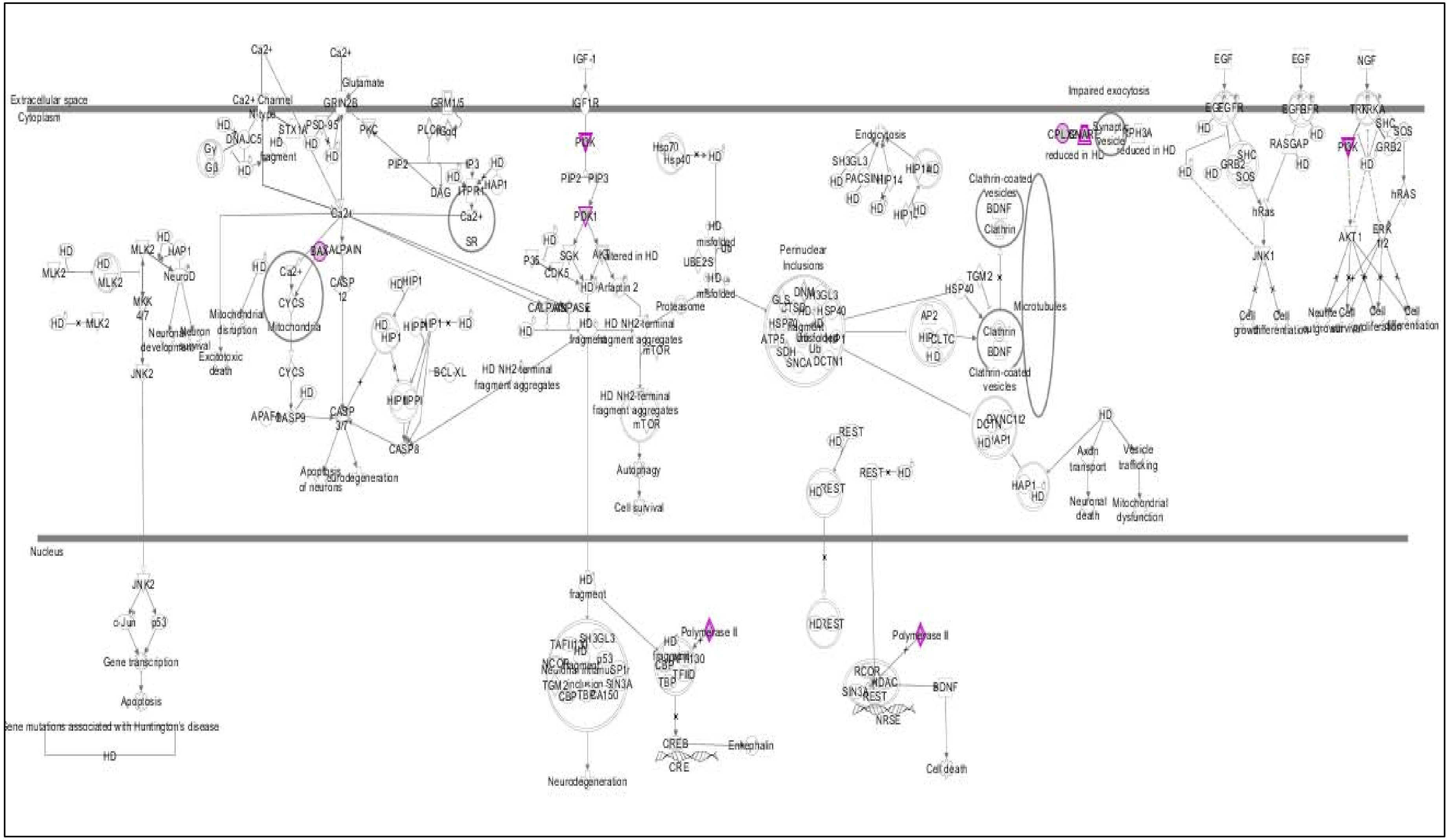

27-PEDF signaling

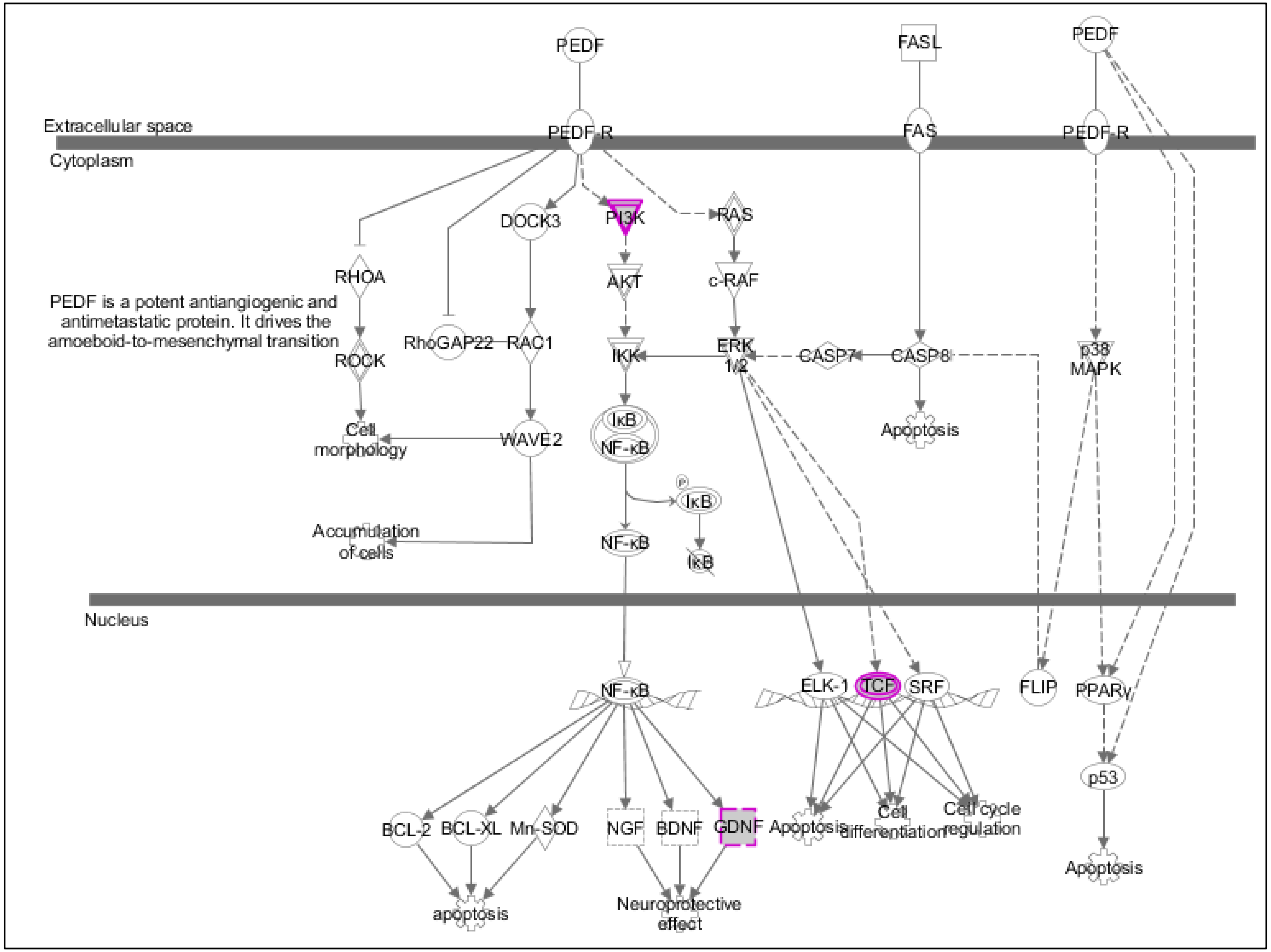

28-Calcium signaling

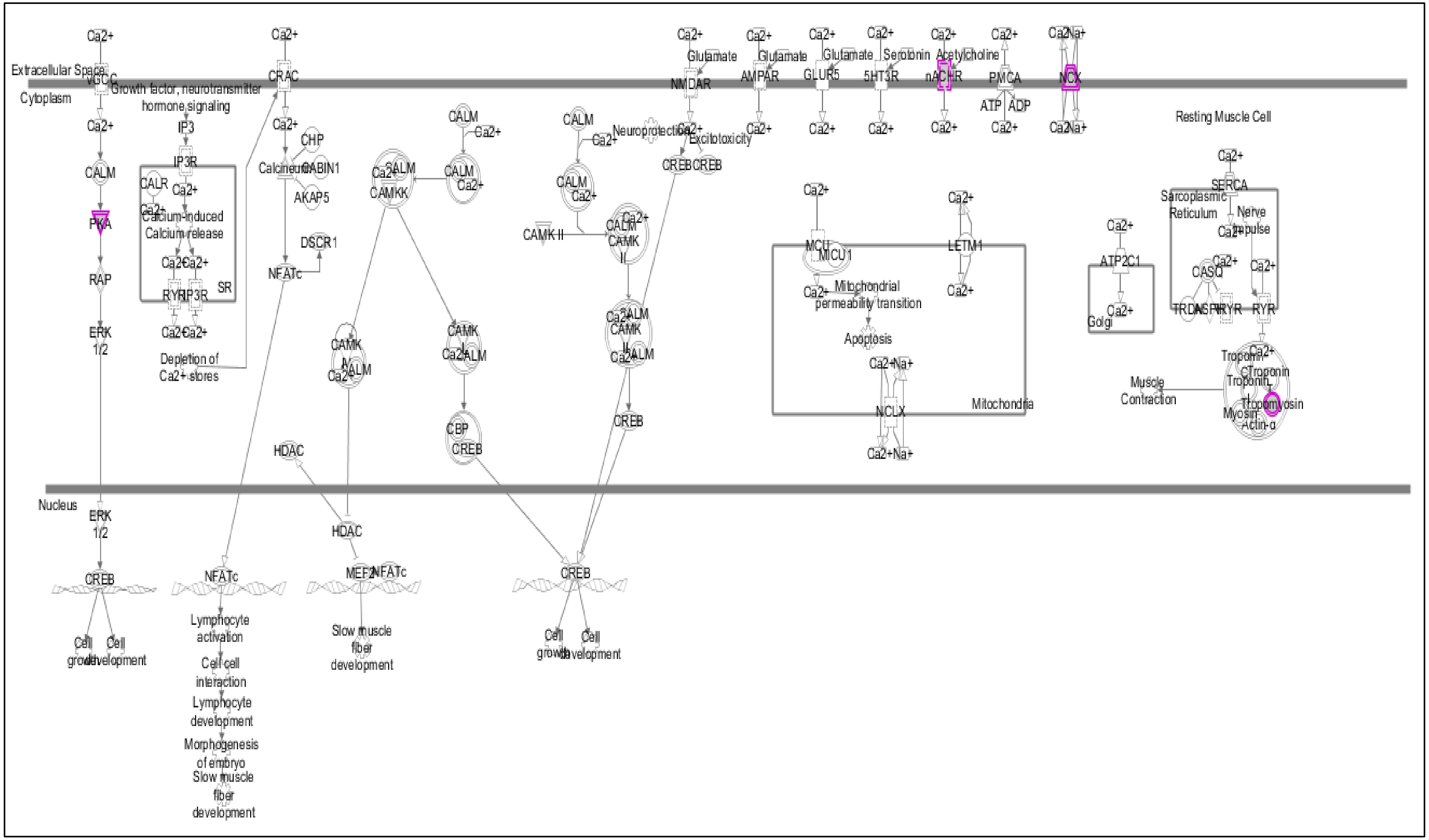

29-Acute myeloid leukemia signaling

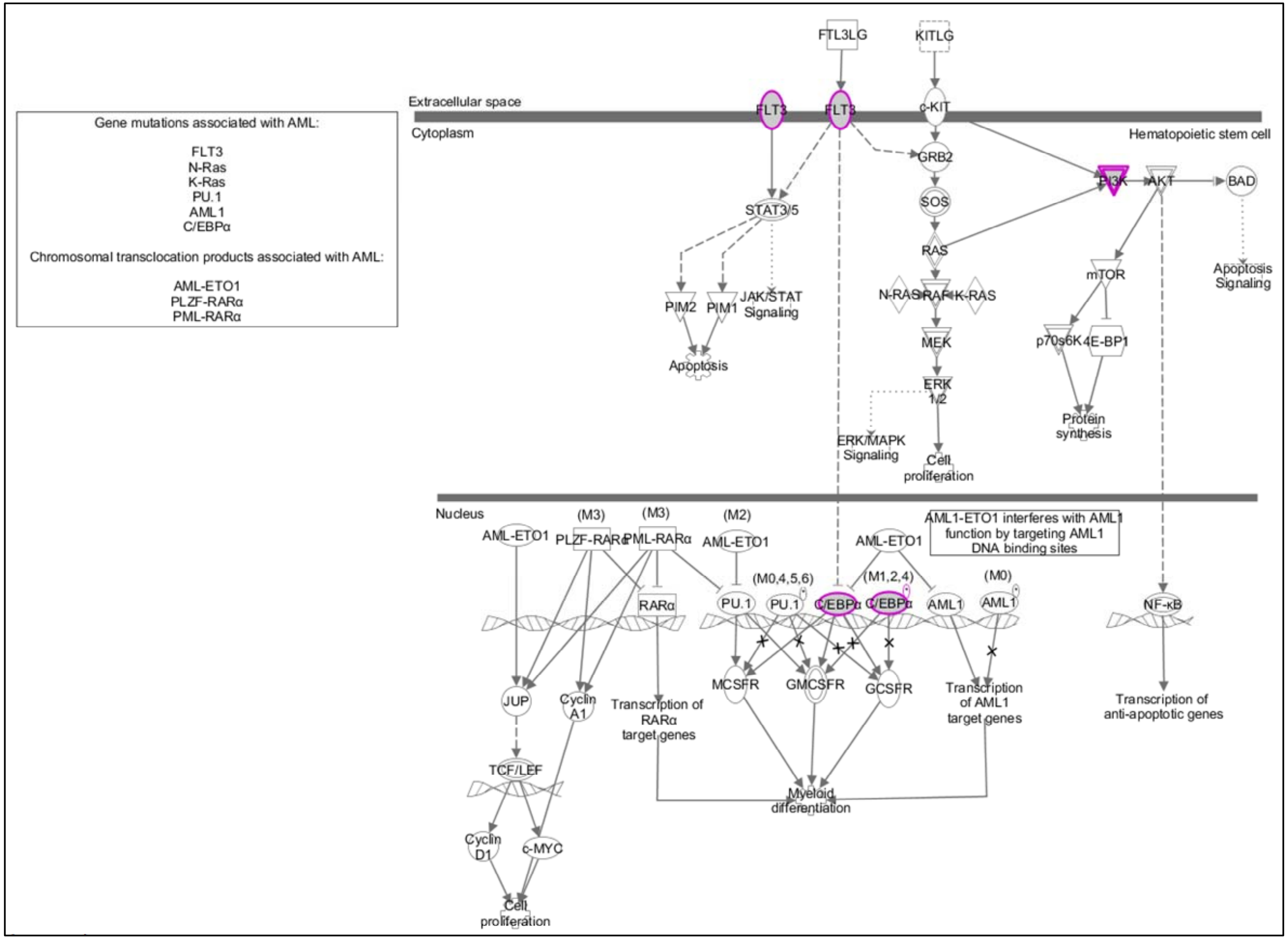

30-Protein ubiquitination pathway

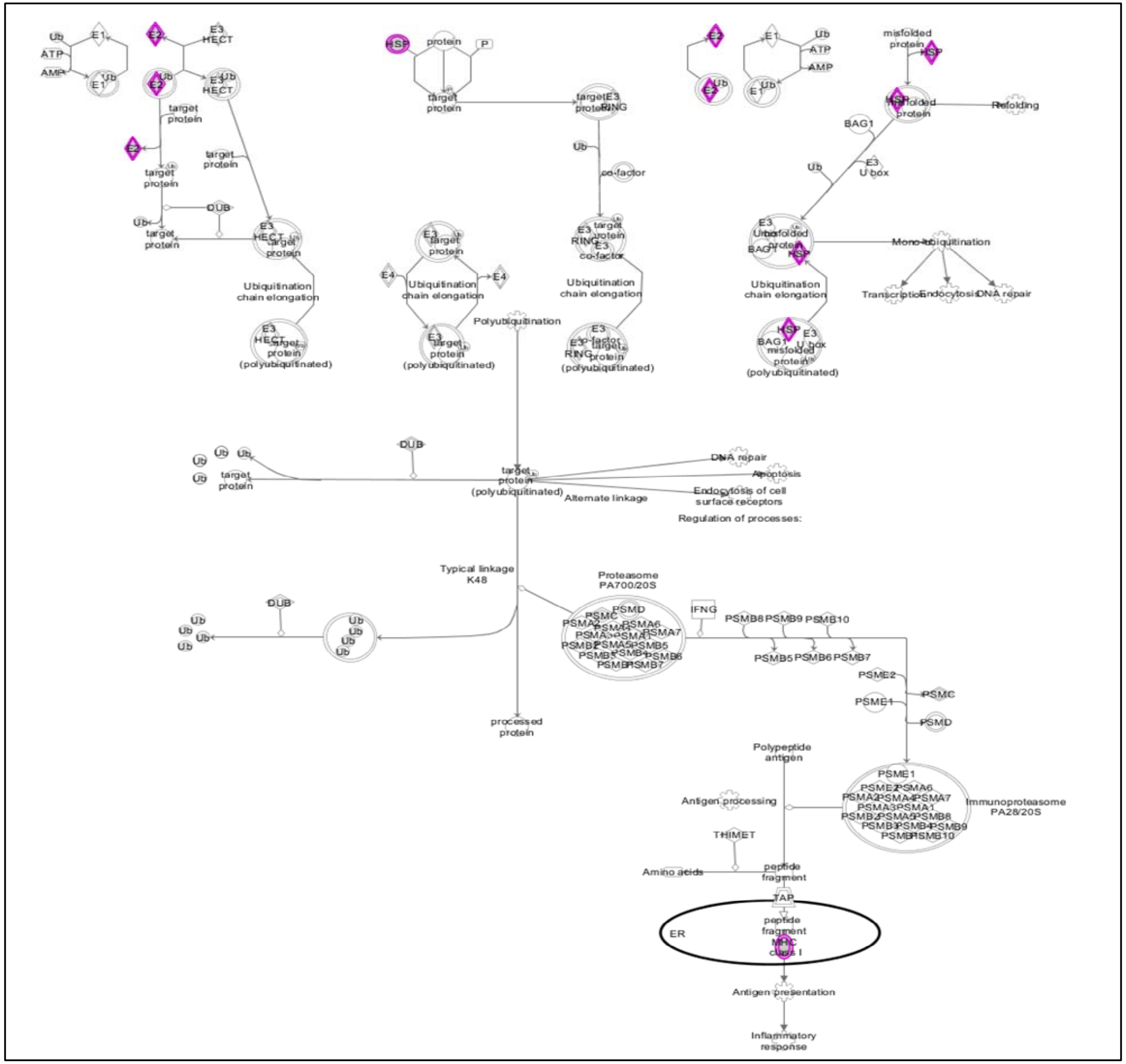

Supplement: Supplementary file 3 [file Presentation_3.zip › Supplemental materials 5.3.pdf]
