# Supplementary material for: Multiomics-Based Signaling Pathway Network Alterations in Human Non-functional Pituitary Adenomas
Source: Front Endocrinol (Lausanne). 2019 Dec 17;10:835. doi: 10.3389/fendo.2019.00835 (PMC6928143; doi:10.3389/fendo.2019.00835)
Supplement: Supplementary file 3 [file Presentation_3.zip › Supplemental materials 6.1.pdf]

Suppemental materials 6.1 Differentially expressed proteins between invasive and non-invasive NFPAs for IPA analysis (Dataset 6)

Fold Change	ID	Notes	Molecules	Description	Location	Function	Drug
3.5	P78536		ADAM17	ADAM metallopeptidase domain 17	Plasma Membrane	peptidase	
-17.9	Q9UPQ3		AGAP1	ArfGAP with GTPase domain, ankyrin repeat and PH domain 1	Cytoplasm	enzyme	
3.4	Q96BJ3		AIDA	axin interactor, dorsalization associated	Cytoplasm	other	
-94.3	P25705		ATP5A1	ATP synthase, H+ transporting, mitochondrial F1 complex, alpha subunit 1, cardiac muscle	Cytoplasm	transporter	
4.2	P06576		ATP5B	ATP synthase, H+ transporting, mitochondrial F1 complex, beta polypeptide	Cytoplasm	transporter	
8.5	P56817		BACE1	beta-site APP-cleaving enzyme 1	Cytoplasm	peptidase	
-4.9	Q02338		BDH1	3-hydroxybutyrate dehydrogenase, type 1	Cytoplasm	enzyme	
4	P29466		CASP1	caspase 1, apoptosis-related cysteine peptidase	Cytoplasm	peptidase	
16	P04040		CAT	catalase	Cytoplasm	enzyme	fomepizole
3.2	Q8NA31		CCDC79	coiled-coil domain containing 79	Nucleus	peptidase	

13.6	Q00535	CDK5	cyclin-dependent kinase 5	Nucleus	kinase	flavopiridol
3.2	Q9Y6N3	CLCA3P	chloride channel accessory 3, pseudogene	Plasma Membrane	ion channel	
4.6	P01040	CSTA	cystatin A (stefin A)	Cytoplasm	other	
3.5	P07108	DBI	diazepam binding inhibitor (GABA receptor modulator, acyl-CoA binding protein)	Cytoplasm	other	
-4.6	Q96QD5	DEPDC7	DEP domain containing 7	Cytoplasm	other	
-7.3	Q9Y6G9	DYNC1LI1	Dynein, cytoplasmic 1, light chain	Cytoplasm	other	
-7.6	P25101	EDNRA	Endothelin receptor type A	Plasma Membrane	Transmembrane receptor	Bosentan, Avosentan
-16.3	Q96KP1	EXOC2	exocyst complex component 2	Cytoplasm	transporter	
3	P00742	F10	coagulation factor X	Extracellular Space	peptidase	dalteparin, heparin, enoxaparin, rivaroxaban, deligoparin, idraparinux, tifacogin, RPR 120844, RPR 208566, DPC 423, nematode anticoagulant protein c2, apixaban, activated recombinant human factor VII, F8, F9, fondaparinux

-8	Q9NPI8		FANCF	Fanconi anemia, complementation group F	Nucleus	other
-7.6	P07332		FES	feline sarcoma oncogene	Cytoplasm	kinase
-5.9	P02792		FTL	ferritin, light polypeptide	Cytoplasm	enzyme
14.4	A2VDF0		FUOM	fucose mutarotase	Other	enzyme
-11.1	P01241	D	GH1	growth hormone 1	Extracellular Space	growth factor
-5.9	P01241	D	GH1	growth hormone 1	Extracellular Space	growth factor
-5	P60983		GMFB	glia maturation factor, beta	Cytoplasm	growth factor
7.7	Q14344		GNA13	guanine nucleotide binding protein (G protein), alpha 13	Plasma Membrane	enzyme
-4.1	P38405		GNAL	guanine nucleotide binding protein (G protein), alpha activating activity polypeptide, olfactory type	Cytoplasm	enzyme
-4.6	P11021		HSPA5	heat shock 70kDa protein 5 (glucose-regulated protein, 78kDa)	Cytoplasm	enzyme
-25.6	P17066		HSPA6	heat shock 70kDa protein 6 (HSP70B')	Other	other
-33	P01859		IGHG2	immunoglobulin heavy constant gamma 2 (G2m marker)	Plasma Membrane	other

7.8	P01834		IGKC	immunoglobulin kappa constant	Extracellular Space	other	
10.6	Q16891		IMMT	inner membrane protein, mitochondrial	Cytoplasm	other	
-3.9	Q96CN7		ISOC1	isochorismatase domain containing 1	Cytoplasm	enzyme	
-11.6	P37285		KLC1	kinesin light chain 1	Cytoplasm	other	
5.4	P04264		KRT1	keratin 1	Cytoplasm	other	
16	P08779		KRT16	keratin 16	Cytoplasm	other	
-5.8	P42704		LRPPRC	leucine-rich pentatricopeptide repeat containing	Cytoplasm	other	
6.1	Q9P267	D	MBD5	methyl-CpG binding domain protein 5	Other	other	
7.8	Q9P267	D	MBD5	methyl-CpG binding domain protein 5	Other	other	
4.6	Q5JXM2		METTL24	methyltransferase like 24	Other	other	
12.4	Q99797		MIPEP	mitochondrial intermediate peptidase	Cytoplasm	peptidase	
-7	Q99542		MMP19	matrix metallopeptidase 19	Extracellular Space	peptidase	marimastat
3.2	Q8N3R9		MPP5	membrane protein, palmitoylated 5 (MAGUK p55 subfamily member 5)	Plasma Membrane	kinase	

-10.3	Q9UL42	PNMA2	paraneoplastic Ma antigen 2	Nucleus	transporter
3	Q8N4Z0	RAB42	RAB42, member RAS oncogene family	Other	other
6.5	Q9BYM8	RBCK1	RanBP-type and C3HC4-type zinc finger containing 1	Cytoplasm	transcription regulator
3.6	Q9Y3B9	RRP15	ribosomal RNA processing 15 homolog (S. cerevisiae)	Nucleus	other
24.3	Q9UIY3	RWDD2A	RWD domain containing 2A	Other	other
18.6	P23297	S100A1	S100 calcium binding protein A1	Cytoplasm	other
-7.4	A4FU49	SH3D21	SH3 domain containing 21	Other	other
-22.5	Q9HD45	TM9SF3	transmembrane 9 superfamily member 3	Cytoplasm	transporter
-10.7	A6NHL2	TUBAL3	tubulin, alpha-like 3	Other	enzyme
-5.6	Q8TB05	UBALD1	UBA-like domain containing 1	Other	other
-7.3	Q7Z3I7	ZNF572	zinc finger protein 572	Nucleus	other
37.4	Q8N823	ZNF611	zinc finger protein 611	Other	other
3.4	P18988				

10.2	P04434
-13.8	P02080
