# Supplementary material for: Multiomics-Based Signaling Pathway Network Alterations in Human Non-functional Pituitary Adenomas
Source: Front Endocrinol (Lausanne). 2019 Dec 17;10:835. doi: 10.3389/fendo.2019.00835 (PMC6928143; doi:10.3389/fendo.2019.00835)

Supplemental materials 6.3
Statistically significant canonical pathways derived from differentially expressed proteins between invasive and non-invasive NFPAs for IPA analysis (Dataset 6)

1. Huntington's Disease Signaling
2. Mitochondrial Dysfunction
3. Axonal Guidance Signaling
4. Inhibition of Matrix Metalloproteases
5. Hematopoiesis from Pluripotent Stem Cells
6. Semaphorin signaling in Neurons
7. Primary Immunodeficiency Signaling
8. Amyloid Processing
9. Endothelin-1 Signaling
10. Superoxide Radicals Degradation
11. TR-RXR Activation
12. CDK5 Signaling
13. Neuregulin Signaling
14. Amyotrophic Lateral Sclerosis Signaling
15. Extrinsic Prothrombin Activation Pathway
16. Endoplasmic Reticulum Stress Pathway
17. Ketogenesis
18. Ketolysis
19. eNOS Signaling

Dataset 6-Canonical pathway Chart

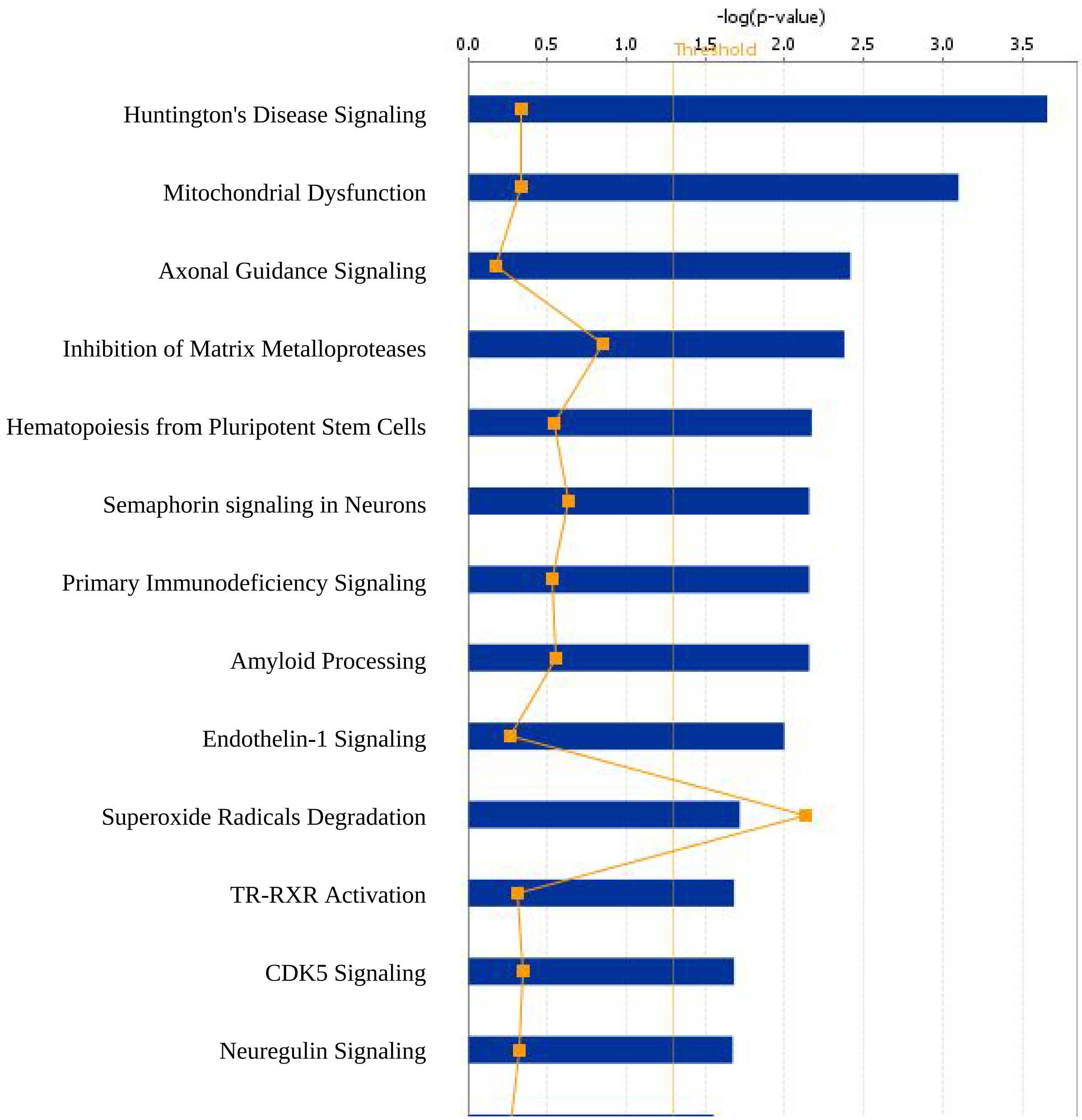

1-Huntington's disease signaling

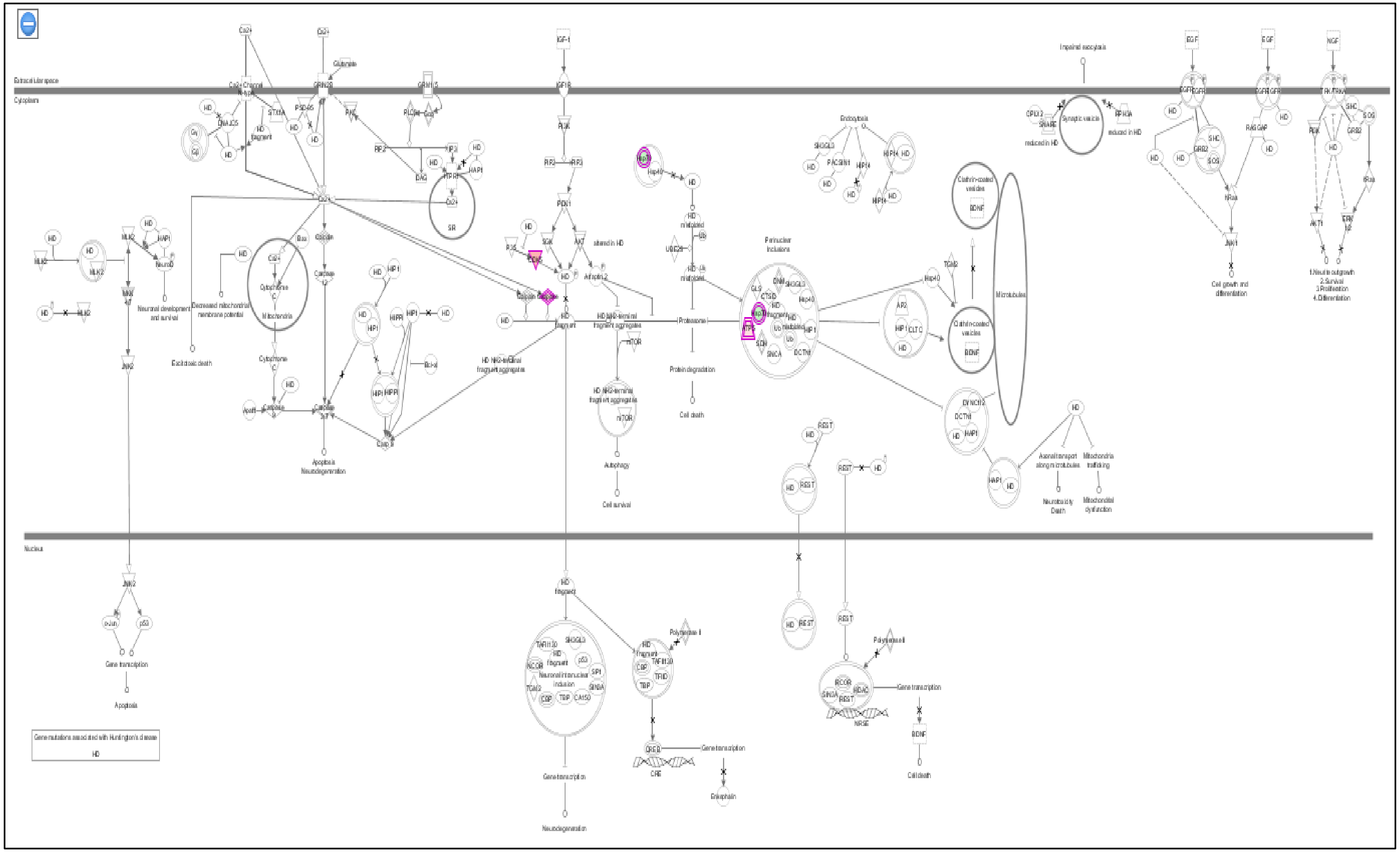

2-Mitochondrial Dysfunction

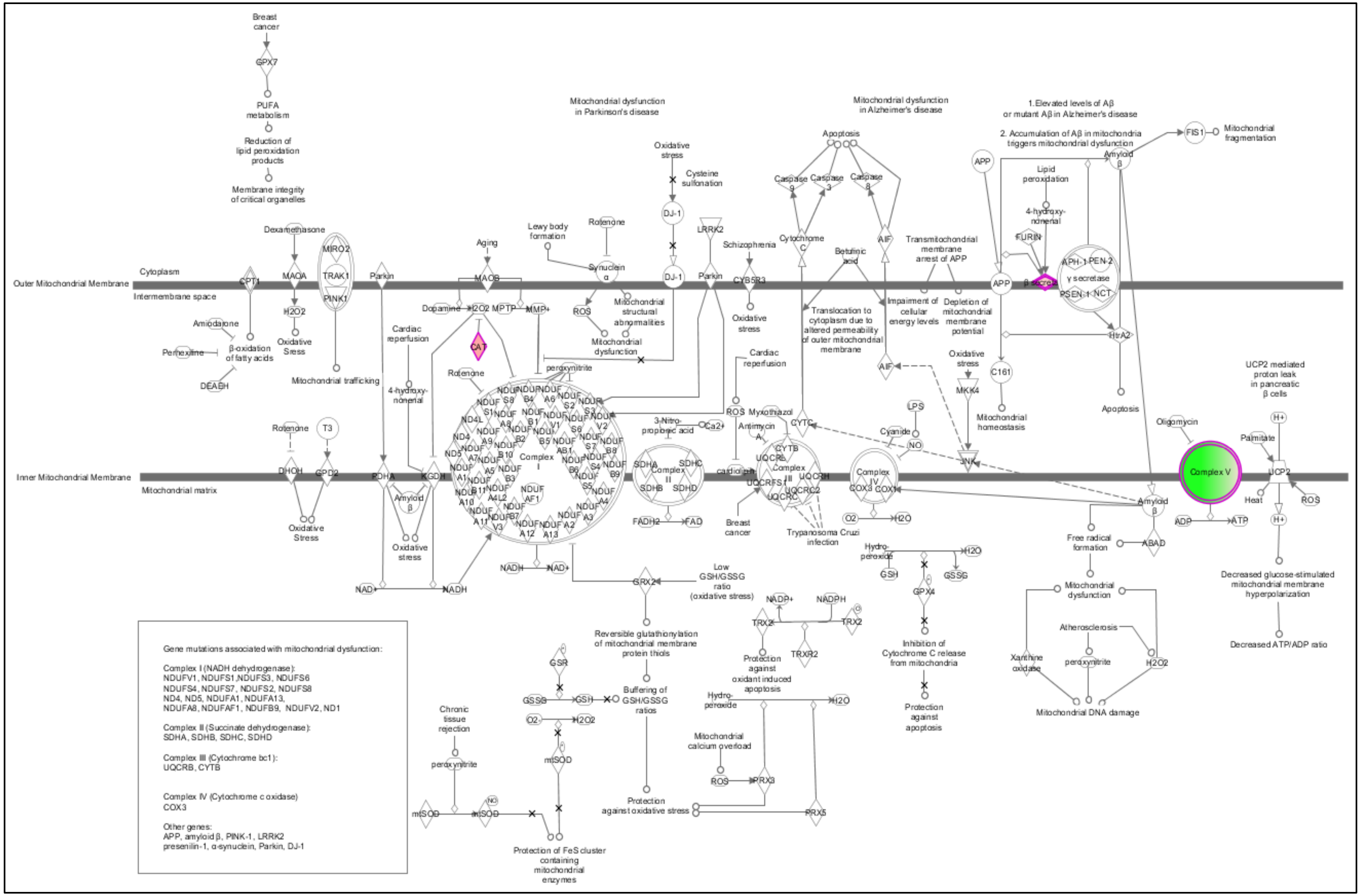

3-Axonal guidance signaling

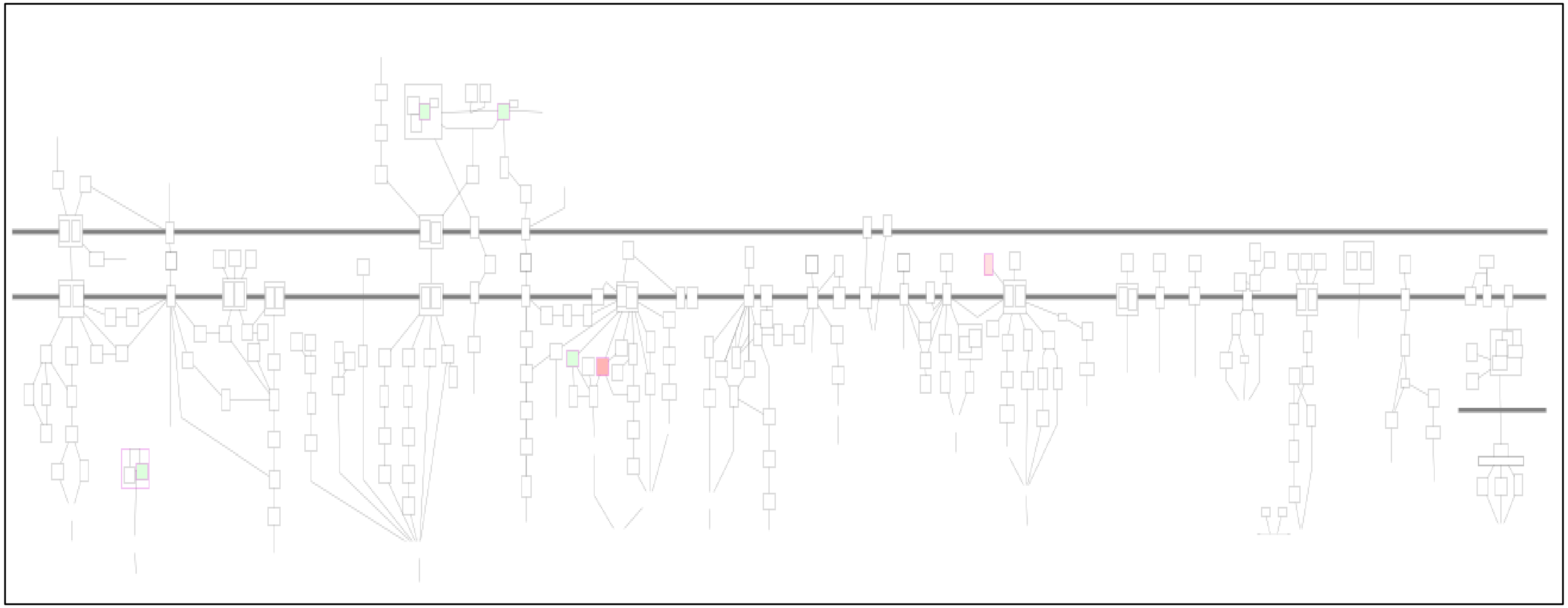

4-Inhibition of matrix metalloproteases

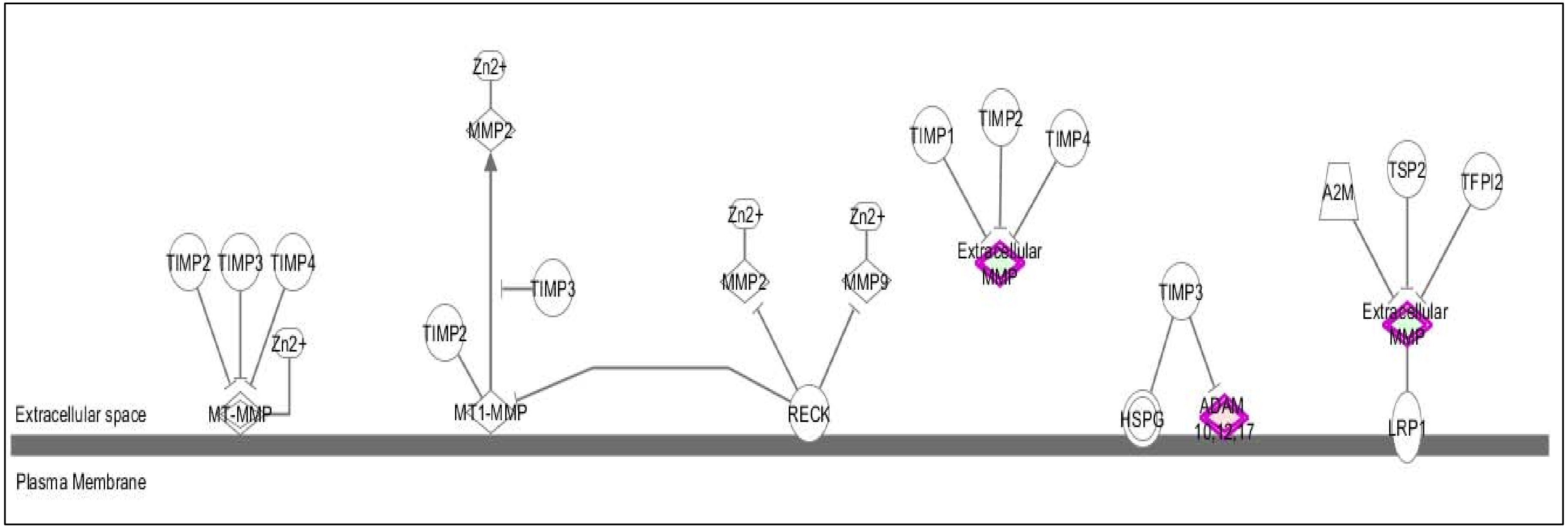

5-Hematopoiesis from pluripotent stem cells

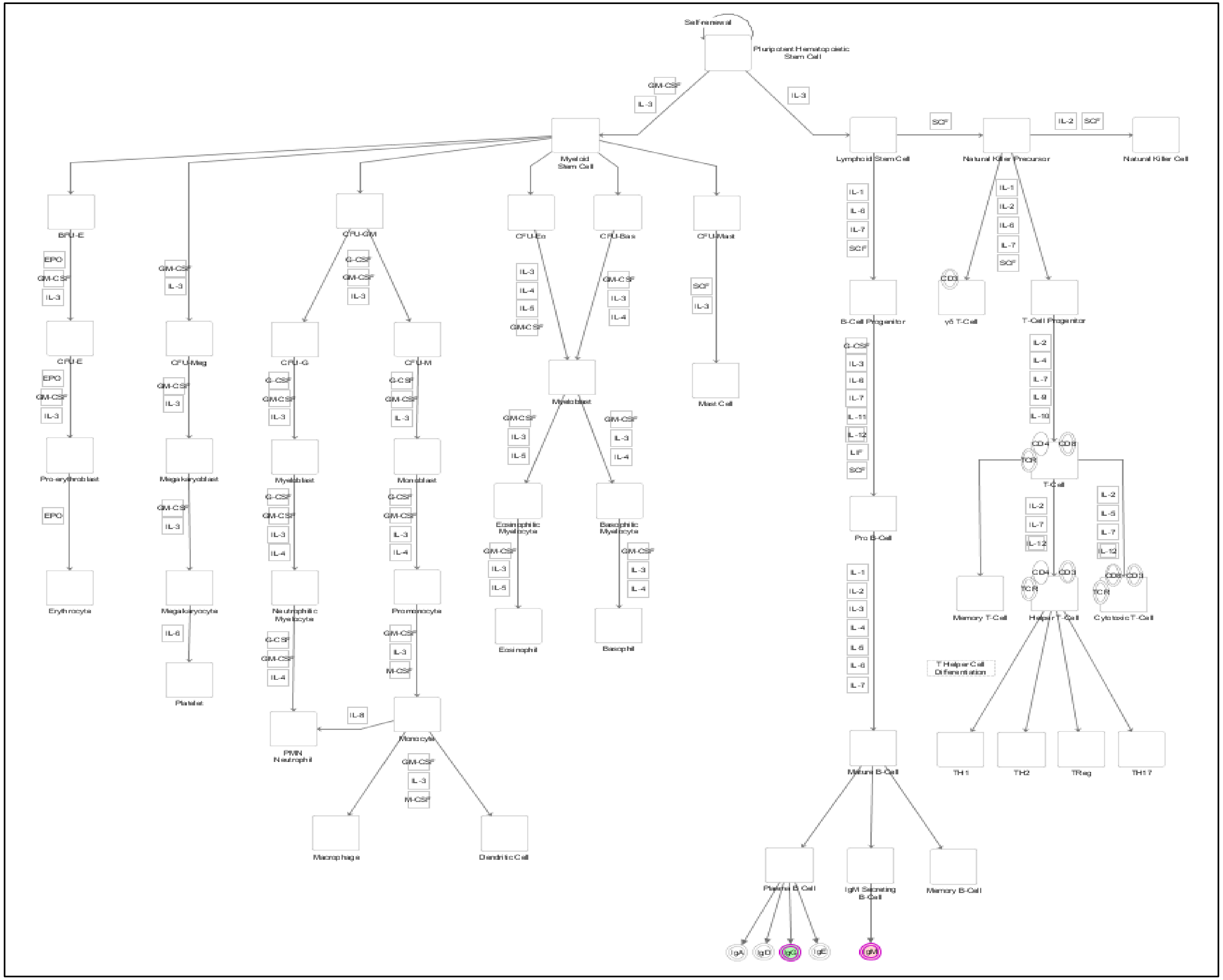

6-Semaphorin signaling in neurons

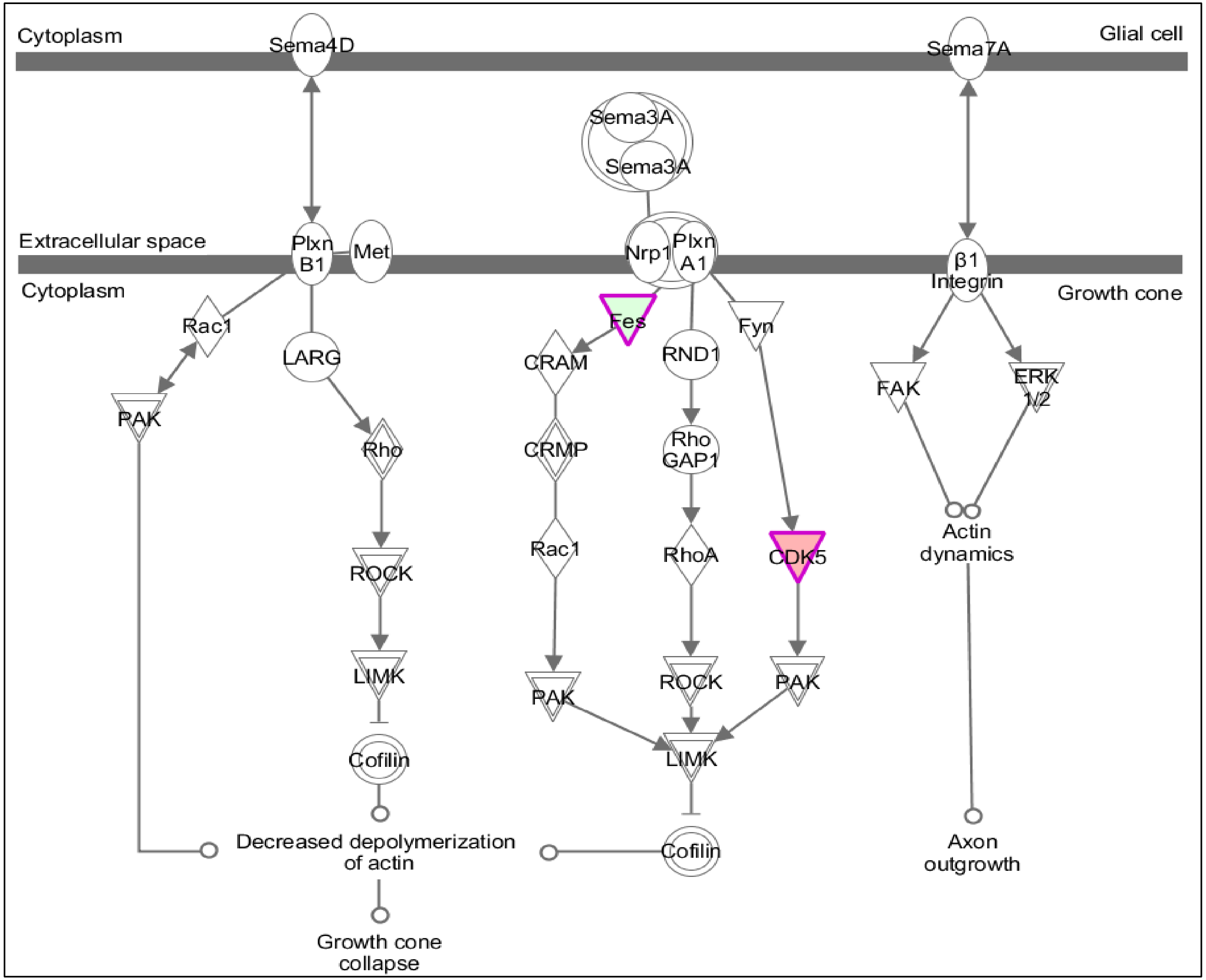

7-Primary immunodeficiency signaling

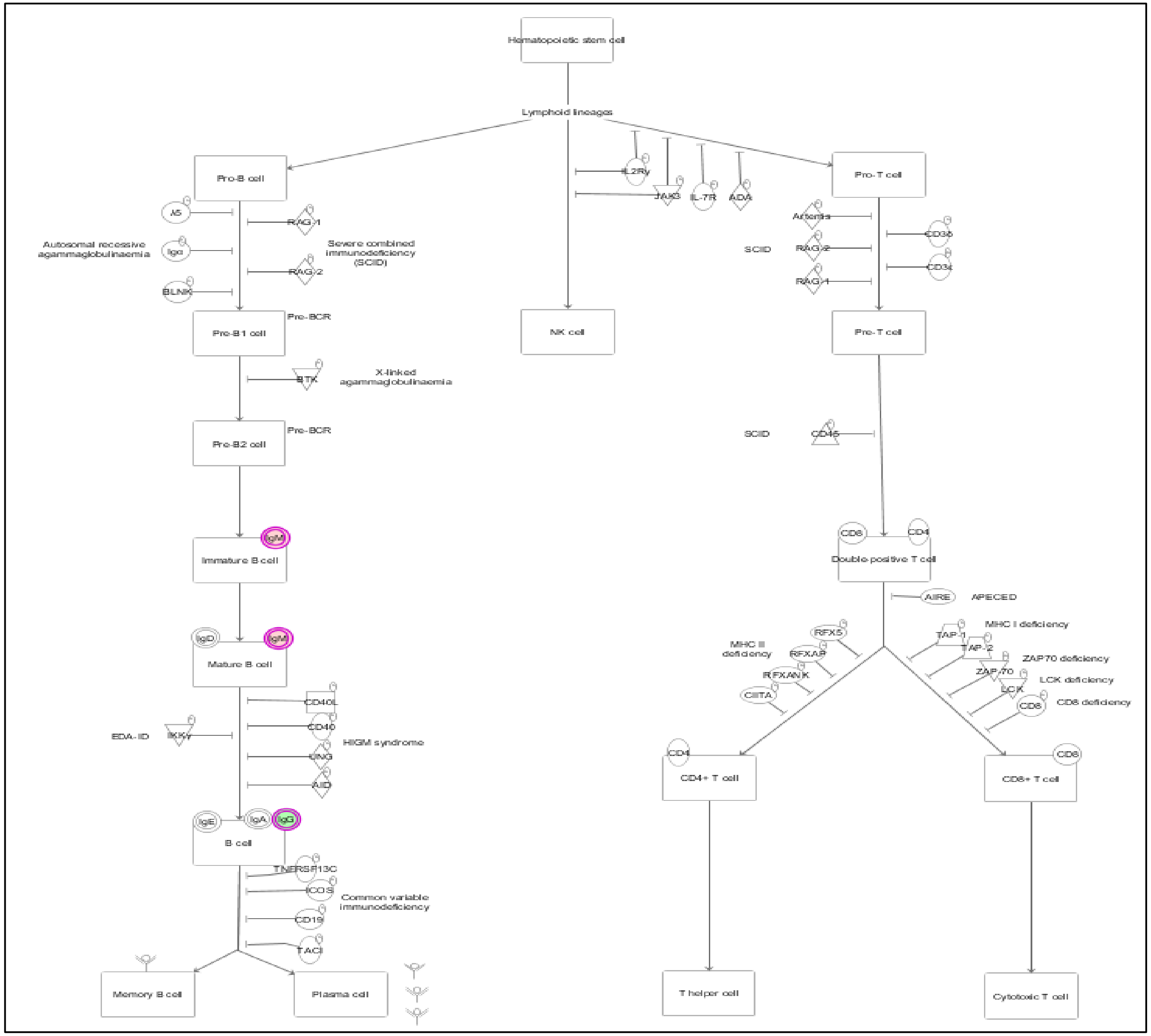

8-Amyloid processing

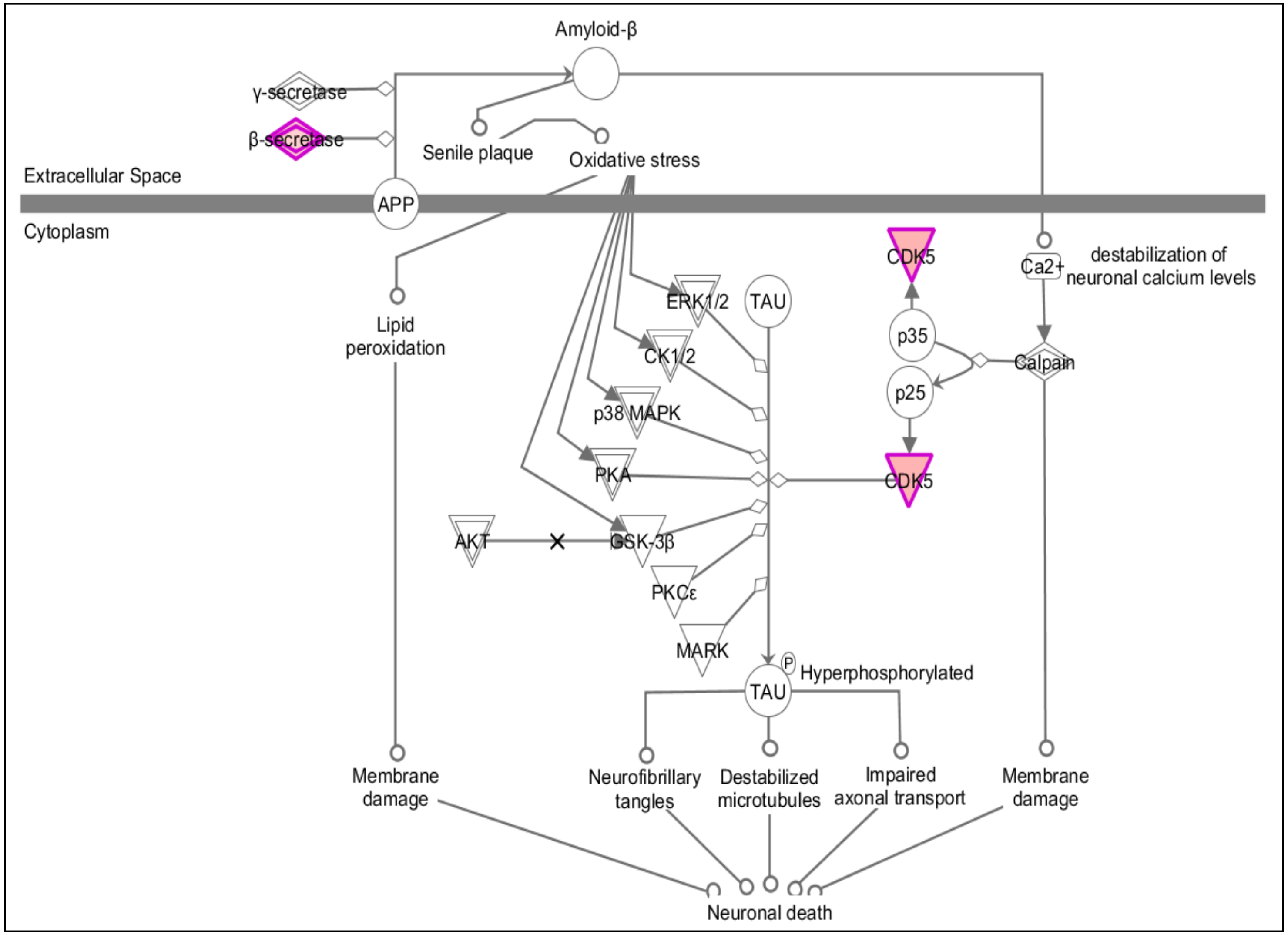

9-Endothelin-1

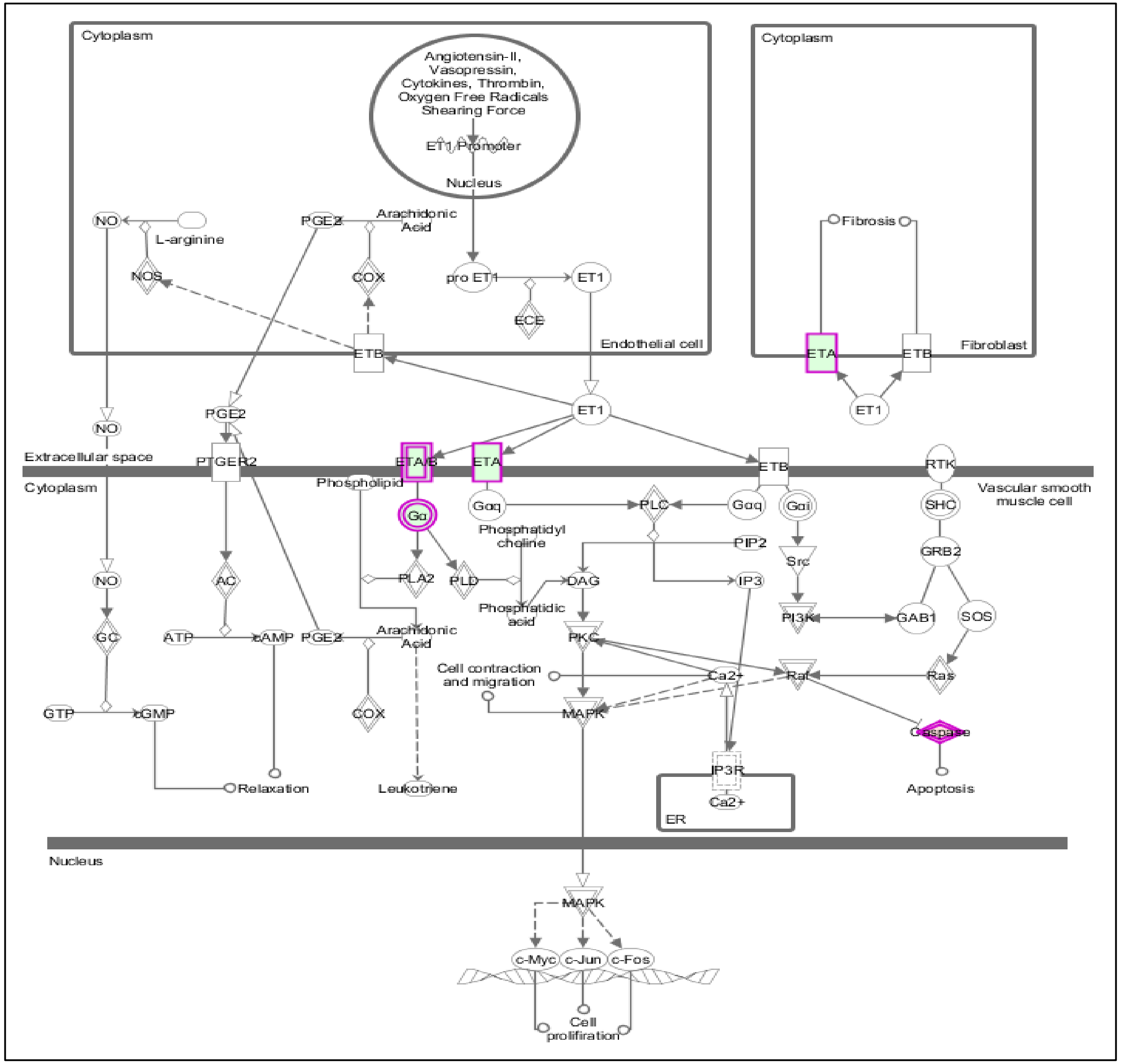

10-Superoxide radicals degradation

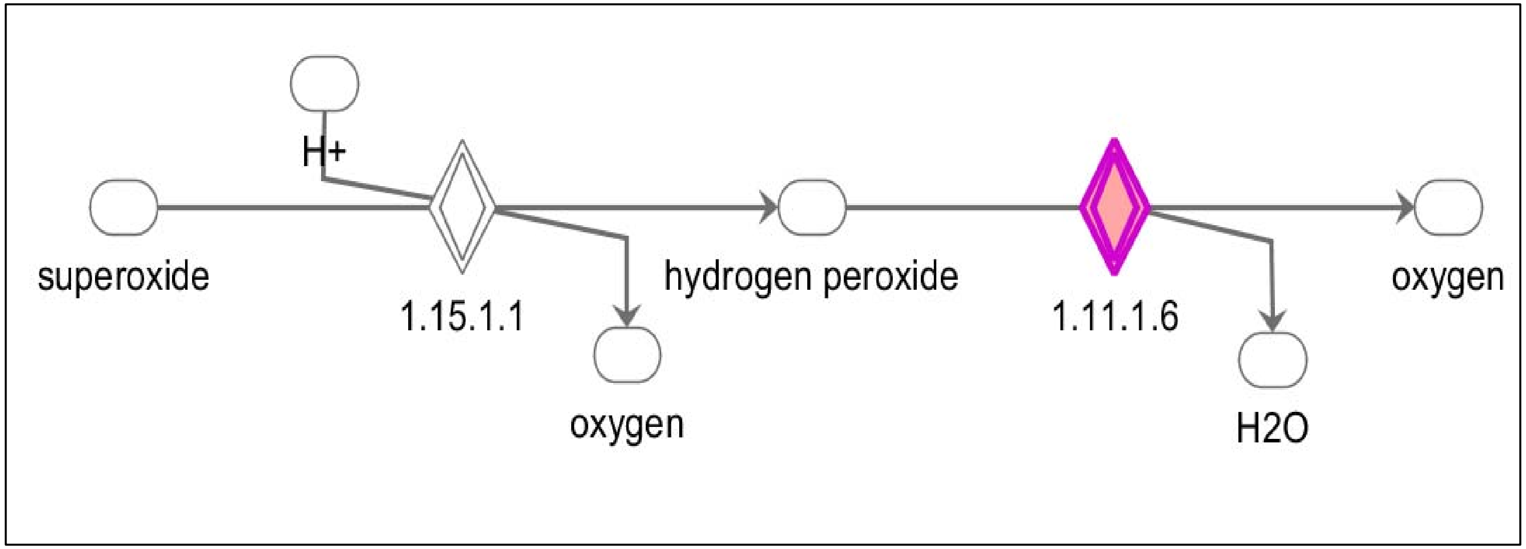

11-TR/RXR activation

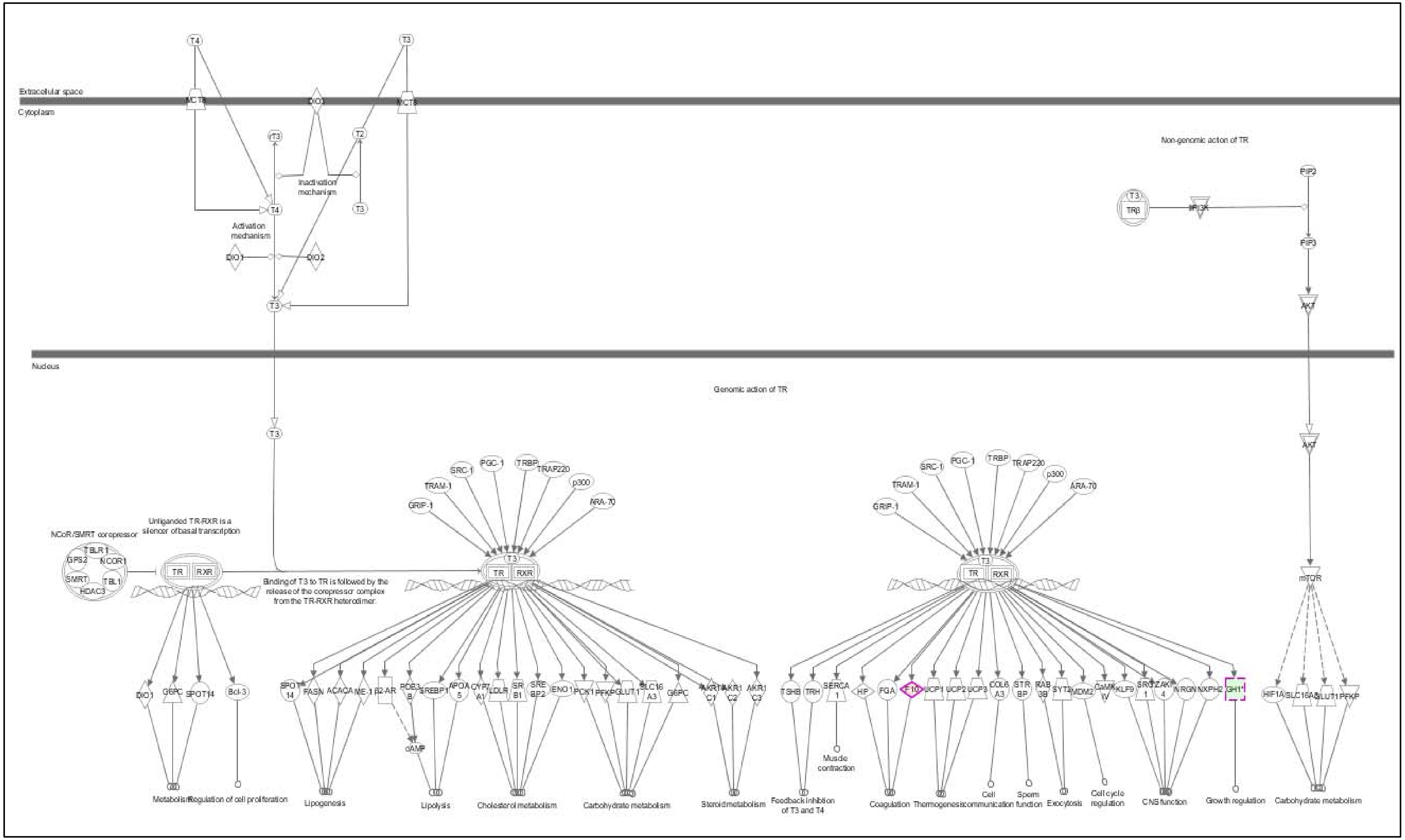

12-CDK5 signaling

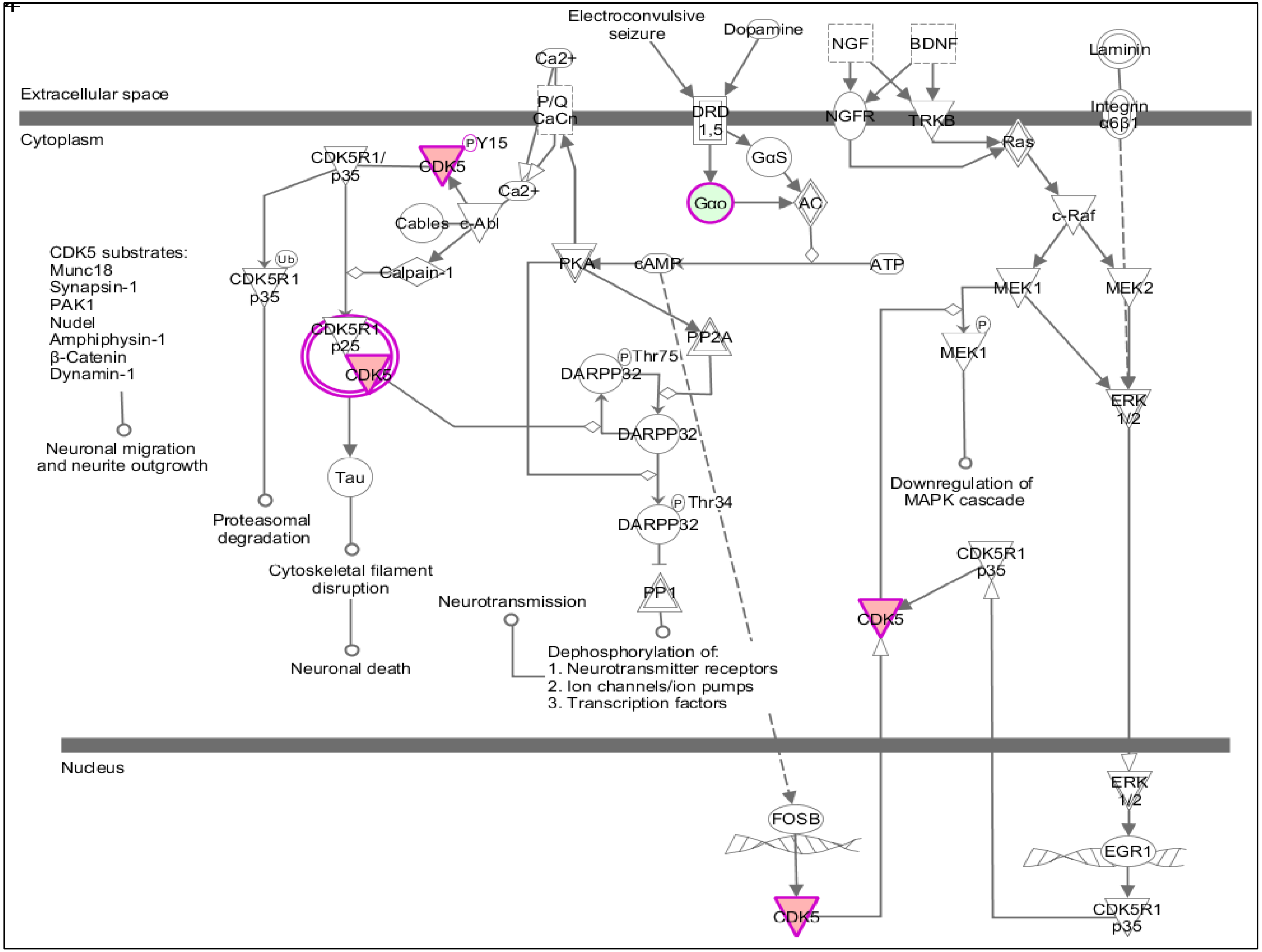

13-Neuregulin signaling

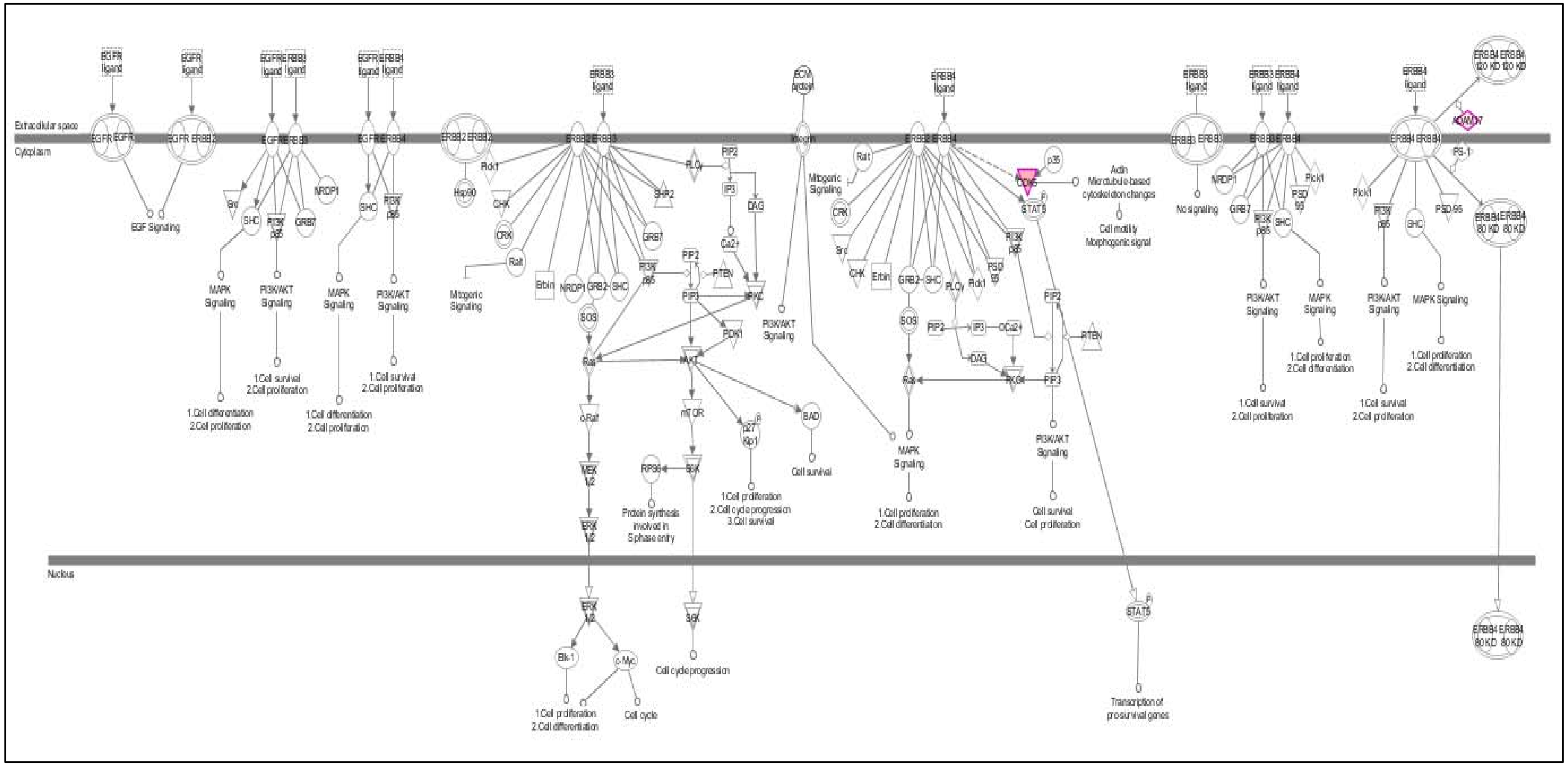

14-Amyotrophic lateral sclerosis signaling

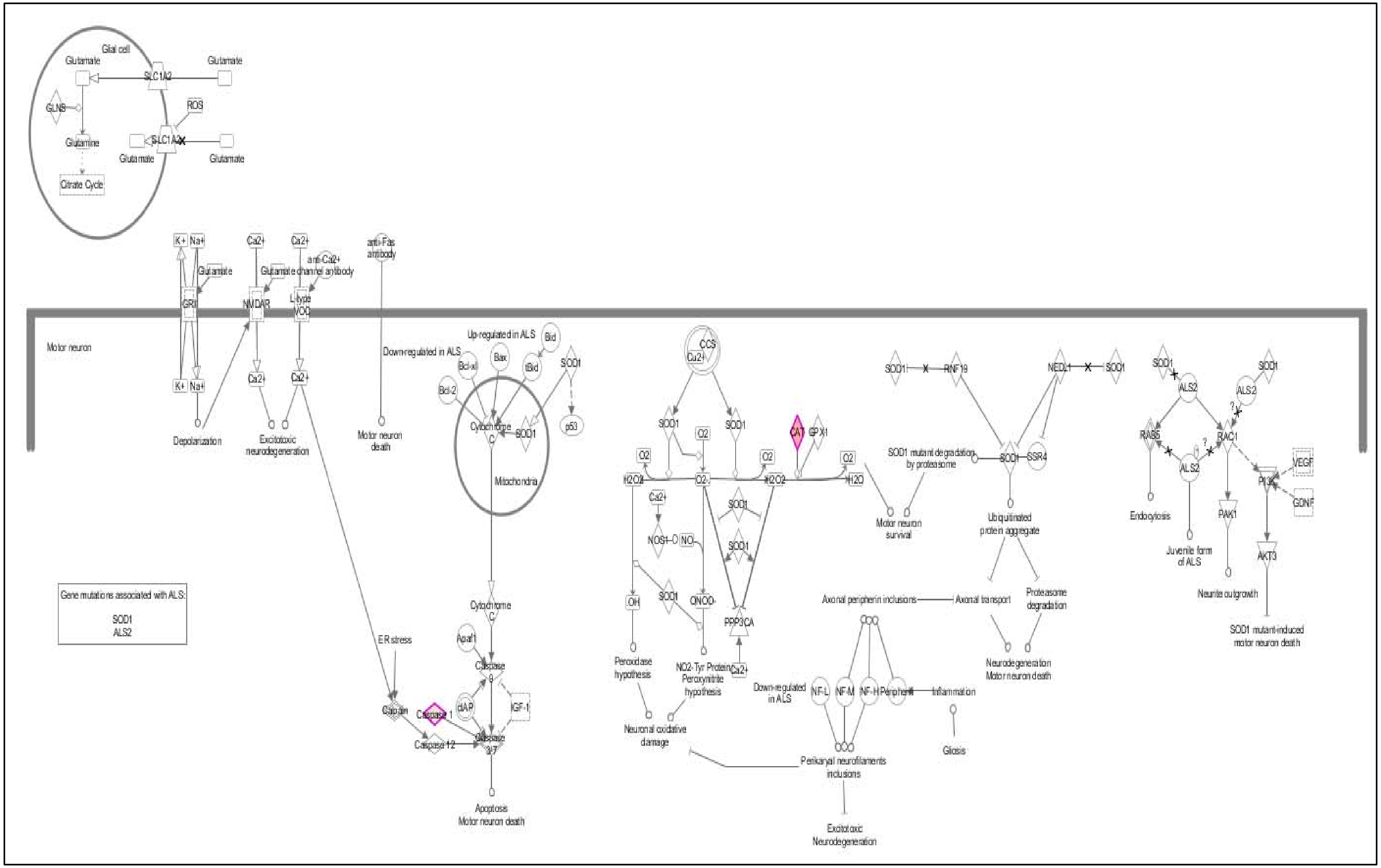

15-Extrinsic prothrombin activation pathway

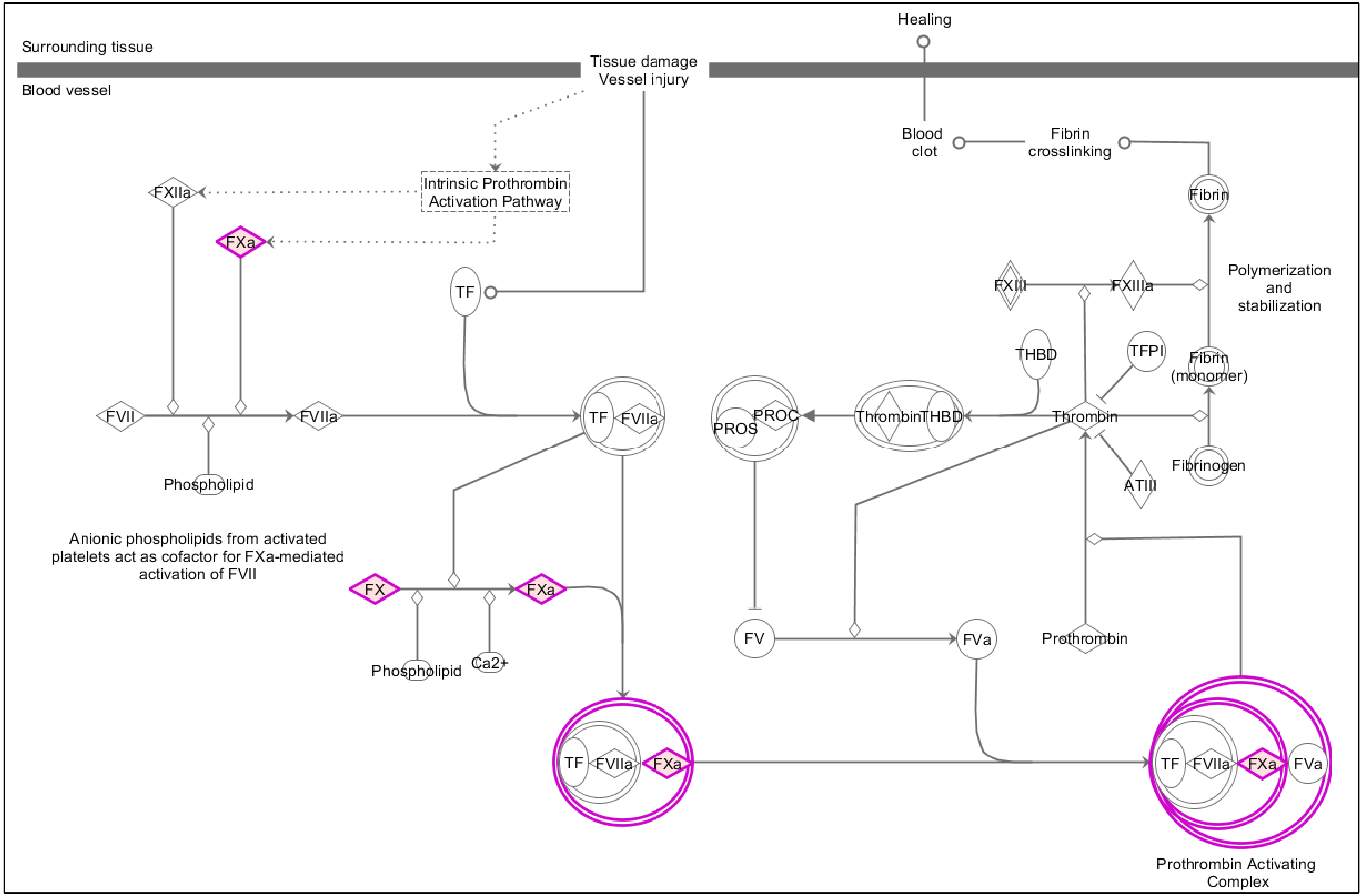

16-Endoplasmic reticulum stress pathway

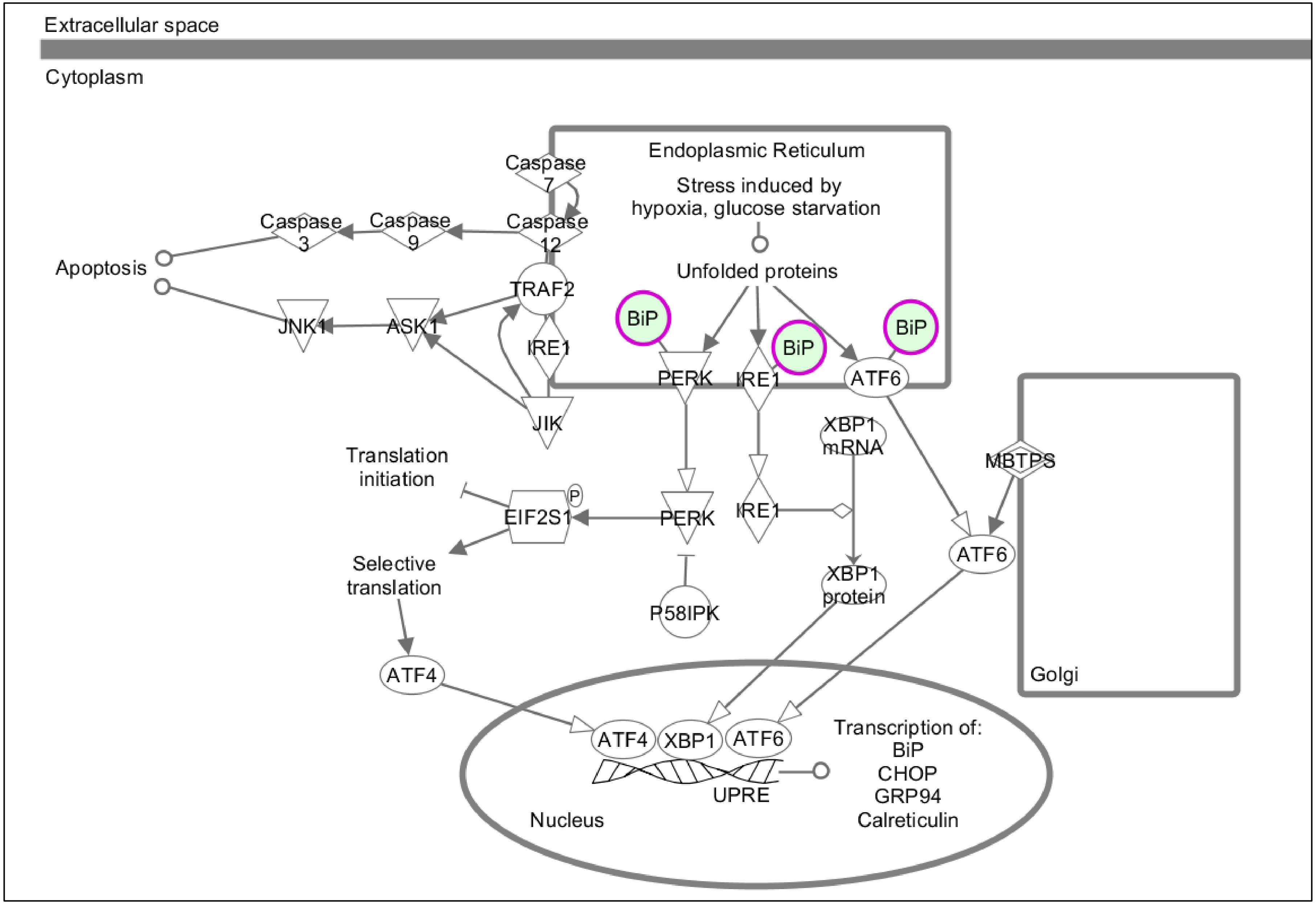

17-Ketogenesis

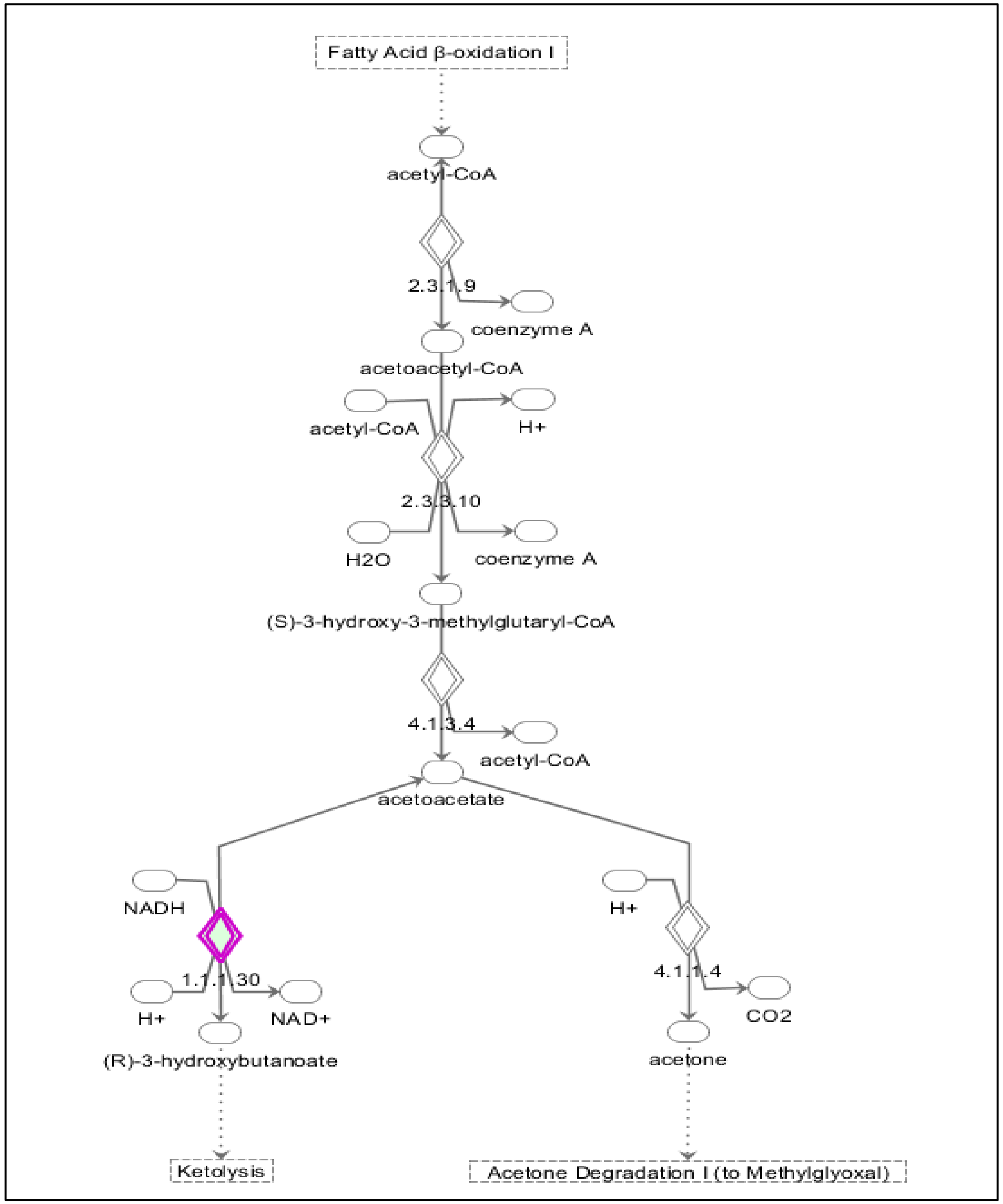

18-Ketolysis

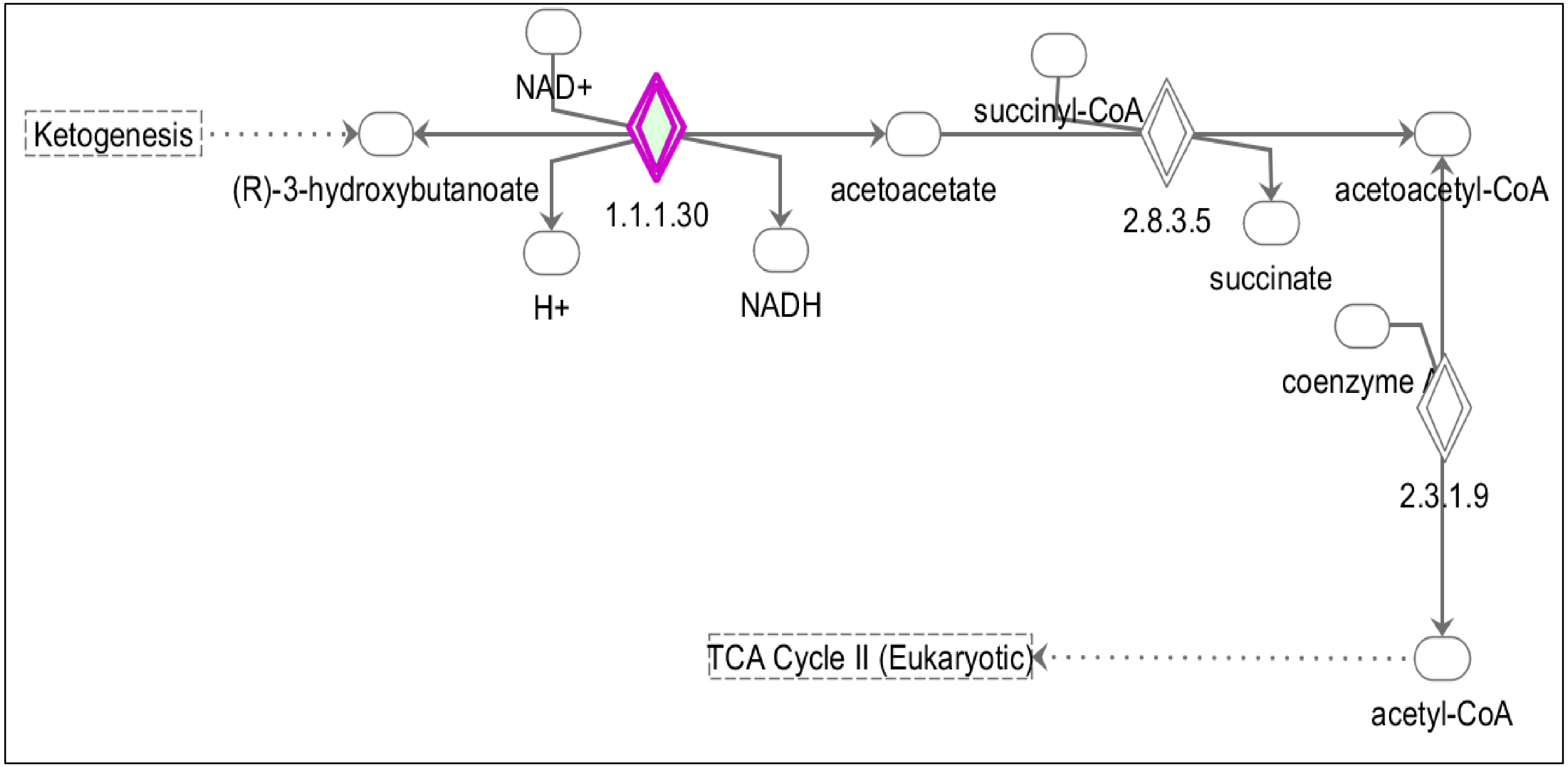

19-eNOS signaling

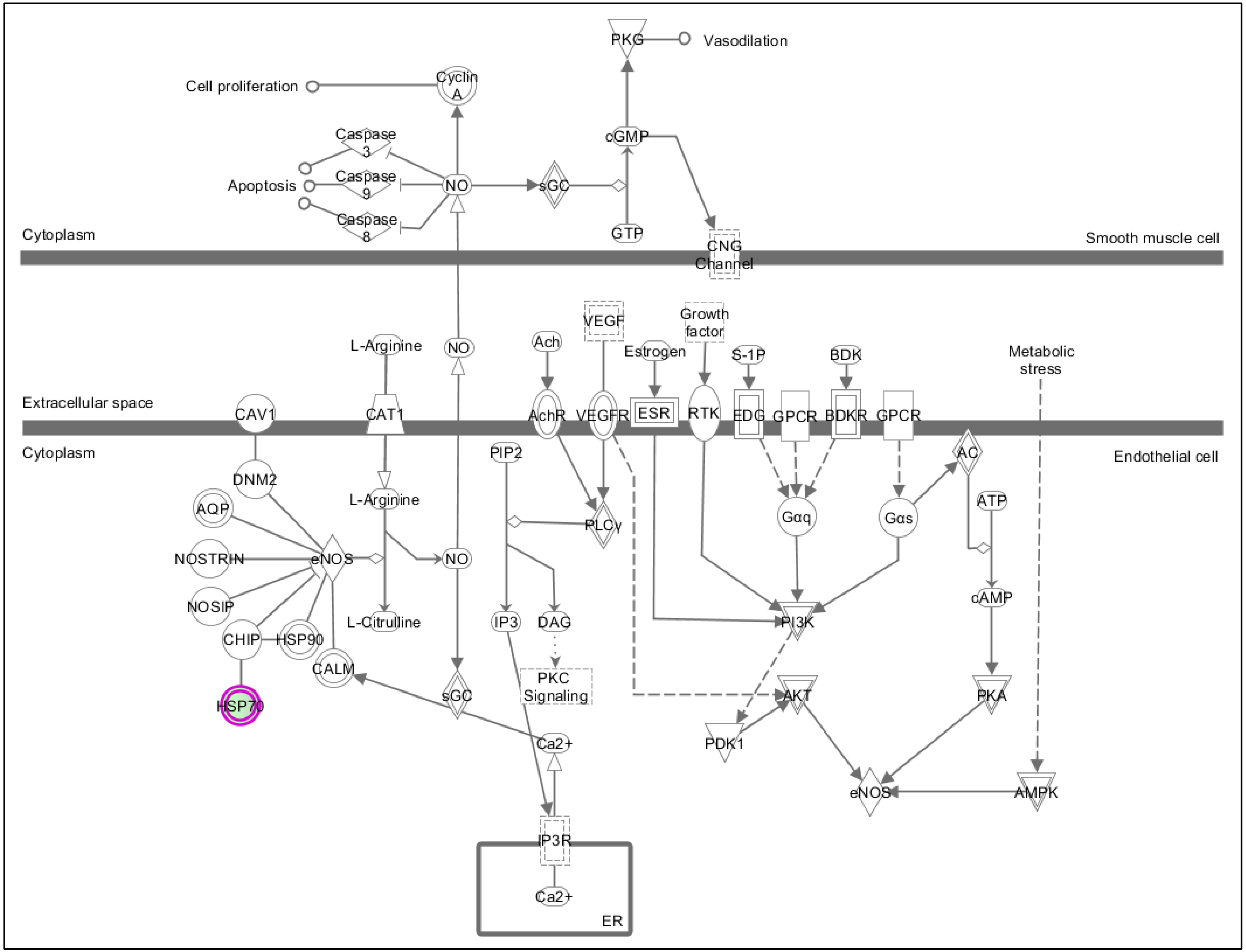

Supplement: Supplementary file 3 [file Presentation_3.zip › Supplemental materials 6.3.pdf]
