# Supplementary material for: Multiomics-Based Signaling Pathway Network Alterations in Human Non-functional Pituitary Adenomas
Source: Front Endocrinol (Lausanne). 2019 Dec 17;10:835. doi: 10.3389/fendo.2019.00835 (PMC6928143; doi:10.3389/fendo.2019.00835)

Supplemental materials 7.3
Statistically significant canonical pathways derived from mapping proteins from pituitary controls for IPA analysis (Dataset 7)

1. EIF2 Signaling
2. Protein Ubiquitination Pathway
3. Mitochondrial Dysfunction
4. Regulation of eIF4 and p70S6K Signaling
5. Oxidative Phosphorylation
6. NRF2-mediated Oxidative Stress Response
7. Remodeling of Epithelial Adherences Junctions
8. Glycolysis Ⅰ
9. Unfolded protein response
10. Clathrin-mediated Endocytosis Signling
11. Fatty Acid β-oxidation Ⅰ
12. Gluconeogenesis Ⅰ
13. Aldosterone Signaling in Epithelial Cells
14. Granzyme A Signling
15. Caveolar-mediated Endocytosis Signaling
16. Actin Cytoskeleton Signaling
17. Epithelial Adherens Junction Signaling
18. mTOR Signaling
19. TCA Cycle Ⅱ(Eukaryotic)
20. Integrin Signaling
21. LXR/RXR Activation
22. Huntington’s Disease Signaling
23. ILK Signaling
24. RhoGDI Signaling
25. RhoA Signaling
26. Acute Phase Response Signaling
27. Regulation of Actin-based Motility by Rho
28. FXR/RXR Activation
29. Signaling by Rho Family GTPases
30. Ethanol Degradation Ⅱ
31. Aryl Hydrocarbon Receptor Signaling
32. Xenobiotic Metabolism Signaling
33. Virus Entry via Endocytic Pathways
34. Valine Degradation Ⅰ
35. CDK5 Signaling
36. Axonal Guidance Signaling
37. Noradrenaline and Adrenaline Degradation
38. Ovarian Cancer Signaling
39. Germ Cell-Sertoli Cell Junction Signaling
40. Protein Kinase A Signaling
41. TR/RXR Actiation
42. Endoplasmic Reticulum Stress Pathway
43. Ephrin B Signaling
44. Tryptophan Degradation X (Mammalian, via Tryptamine)
45. Atherosclerosis Signaling
46. Glutaryl-CoA Degradation
47. GNRH Signaling
48. Colanic Acid Building Blocks Bisynthesis
49. Ephrin Receptor Signaling
50. Isoleucine Degradation Ⅰ
51. Ethanol Degradation Ⅳ
52. VEGF Signaling
53. CREB Signaling in Neurons
54. G Beta Gamma Signaling
55. PI3K/AKT Signaling
56. Telomerase Signaling
57. PPARα/RXRα Activation
58. Sertoli Cell-Sertoli Cell Junction Signaling
59. Cardiac Hypertrophy Signaling
60. IL-1 Signaling
61. Androgen Signaling
62. Tight Junction Signaling
63. Fatty Acid α-oxidation
64. p70S6K Signaling
65. Thyroid Cancer Signaling
66. Regulaion of Cellular Mechanics by Calpain Protease
67. Gap Junction Signaling
68. Lipid Antigen Presentation by CD1
69. ERK/MAPK Signaling
70. Serotonin Degradation
71. Leukocyte Extravasation Signaling
72. DNA Double-Strand Break Repair by Non-Homologous End Joining
73. Agrin Interactions at Neuromuscular Junction
74. Molecular Mechanisms of Cancer
75. Oxidative Ethanol Degradation Ⅲ
76. eNOS Signaling
77. Telomere Extension by Telomerase
78. tRNA Charging
79. Putrescine Degradation Ⅲ
80. Dopamine Receptor Signaling
81. Relaxin Signaling
82. Neuregulin Signaling
83. Dopamine Degradation
84. Intrinsic Prothrombin Activation Pathway
85. Actin Nucleation by ARP-WASP Complex
86. AMPK Signaling
87. Sonic Hedgehog Signaling
88. Rac Signaling

89. Breast Cancer Regulation by Stathmin1
90. 14-3-3-mediated Signaling
91. Leptin Signaling in Obesity
92. Spliceosomal Cycle
93. FAK Signaling
94. Role of Tissue Factor in Cancer
95. Paxillin Signaling
96. Acetyl-CoA Biosynthesis Ⅰ(Pyruvate Dehydrogenase Complex)
97. RAN Signaling
98. RAR Activation
99. CXCR4 Signaling
100. Histamine Degradation
101. IL-12 Signaling and Production in Macrophages
102. Production of Nitric Oxide and Reactive Oxygen Species in Macrophages
103. DNA Methylation and Transcriptional Repression Signaling
104. BER Pathway
105. LPS/IL-1 Mediated Inhibition of RXR Function
106. Cardiac β-adrenergic Signaling
107. Melanocyte Development and Pigmentation Signaling
108. Prostate Cancer Signaling
109. α-Adrenergic Signaling
110. Melatonin Signaling
111. Mitotic Roles of Polo-like Kinase
112. Hepatic Fibrosis/Hepatic Stellate Cell Activation
113. Corticotropin Releasing Hormone Signaling
114. IGF-1 Signaling
115. Aspartate Degradation Ⅱ
116. Glutathione-mediated Detoxification
117. Superoxide Radicals Degradation
118. Inhibition of Matrix Metalloproteases
119. Pentose Phosphate Pathway
120. Calcium Signaling
121. PXR/RXR Activation
122. Amyloid Processing
123. Granzyme B Signaling
124. P2Y Purigenic Receptor Signaling Pahway
125. 2-ketoglutarate Dehydrogenase Complex
126. Amyotrophic Lateral Sclerosis Signaling
127. Complement System
128. Glutathione Redox Reactions Ⅰ
129. Endometrial Cancer Signaling
130. Nucleotide Excision Repair Pathway
131. Tryptophan Degradation Ⅲ (Eukaryotic)
132. Phospholipase C Signaling
133. Ketolysis
134. Thrombin Signaling
135. Nitric Oxide Signaling in the Cardiovascular System
136. Neuroprotective Role of THOP1 in Alzheimer’s Disease
137. Antigen Presentation Pathway
138. Synaptic Long Term Depression
139. PAK Signaling
140. Pentose Phosphate Pathway (Oxidative Branch)
141. L-cysteine Degradation Ⅰ
142. Sucrose Degradation Ⅴ(Mammalian)
143. fMLP Signaling in Neutrophils
144. Pyrimidine Ribonucleotides Interconversion
145. Coloretal Cancer Metastasis Signaling
146. Glucocorticoid Receptor Signaling
147. Cell Cycle: G2/M DNA Damage Checkpoint Regulation
148. Cellular Effects of Sildenafil (Viagra)
149. Fatty Acid β-oxidation Ⅲ(Unsaturated, Odd Number)
150. HGF Signaling
151. GABA Receptor Signaling
152. Pyrimidine Ribonucleotides De Novo Biosynthesis
153. Mechanisms of Viral Exit from Host Cells
154. PCP Pathway
155. Primary Immunodeficiency Signaling
156. Systemic Lupus Erythematosus Signaling
157. GDP-mannose Biosynthesis
158. Gai Signaling
159. Methylglyoxal Degradation Ⅲ
160. Purine Nucleotides De Novo Biosynthesis Ⅱ
161. HIPPO Signaling
162. ERK5 Signaling
163. Phenylalanine Degradation Ⅳ(Mammalian, via Side Chain)
164. CTLA4 Signaling in Cytotoxic T Lymphocytes
165. Lserine Degradation
166. Hypoxia Signaling in the Cardiovascular System
167. Gliblastma Multiforme Signaling
168. Galactose Degradation Ⅰ(Leloir Pathway)
169. Agranulocyte Adhesion and Diapedesis
170. Synaptic Long Term Potentiation
171. UDP-N-actyl-D-galactosamine Biosynthesis Ⅱ
172. 3-phosphoinositide Biosynthesis
173. Coagulation System
174. Aspartate Biosynthesis

Dataset 7-Canonical pathway Chart

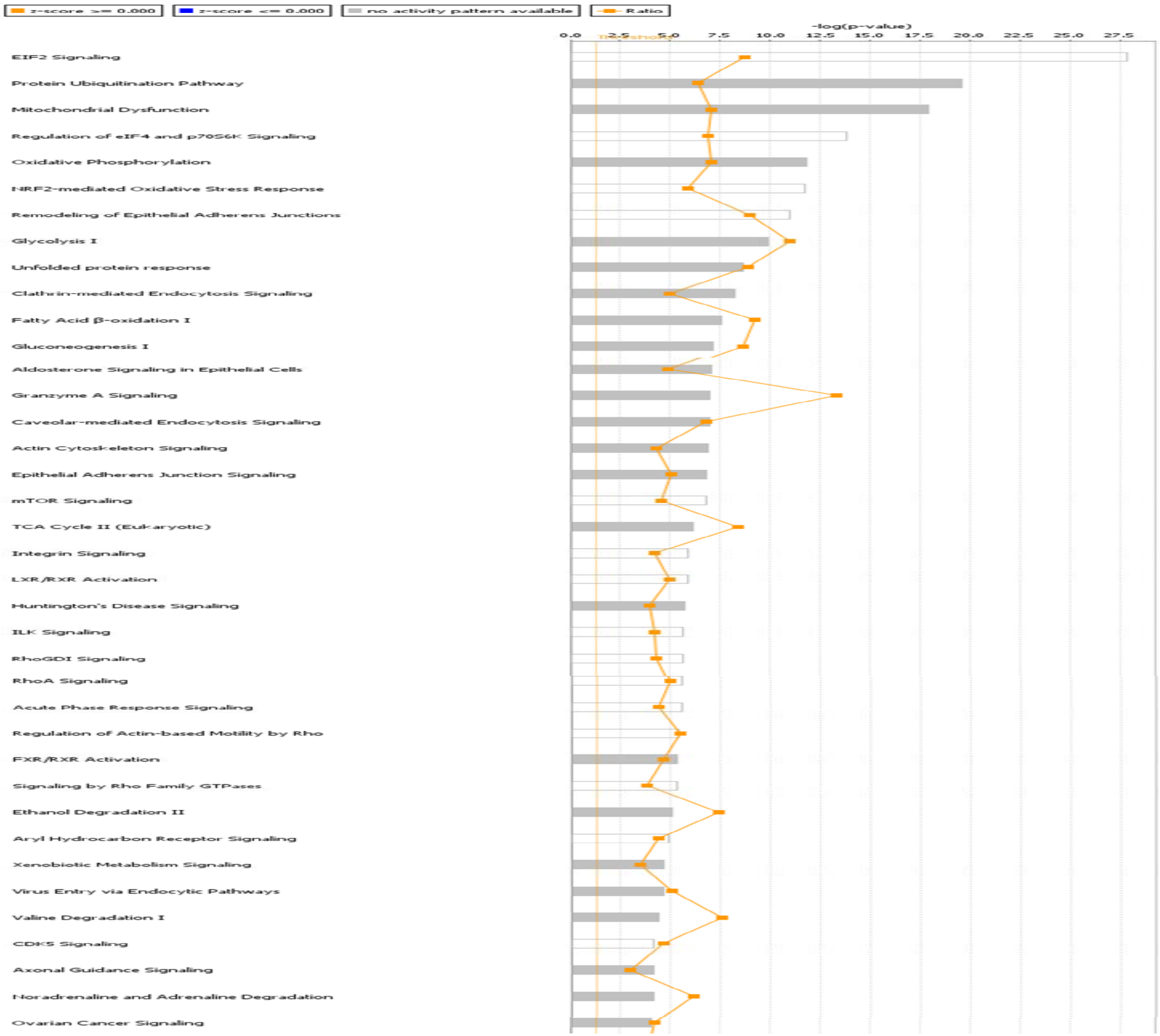

1-EIF2 Signaling

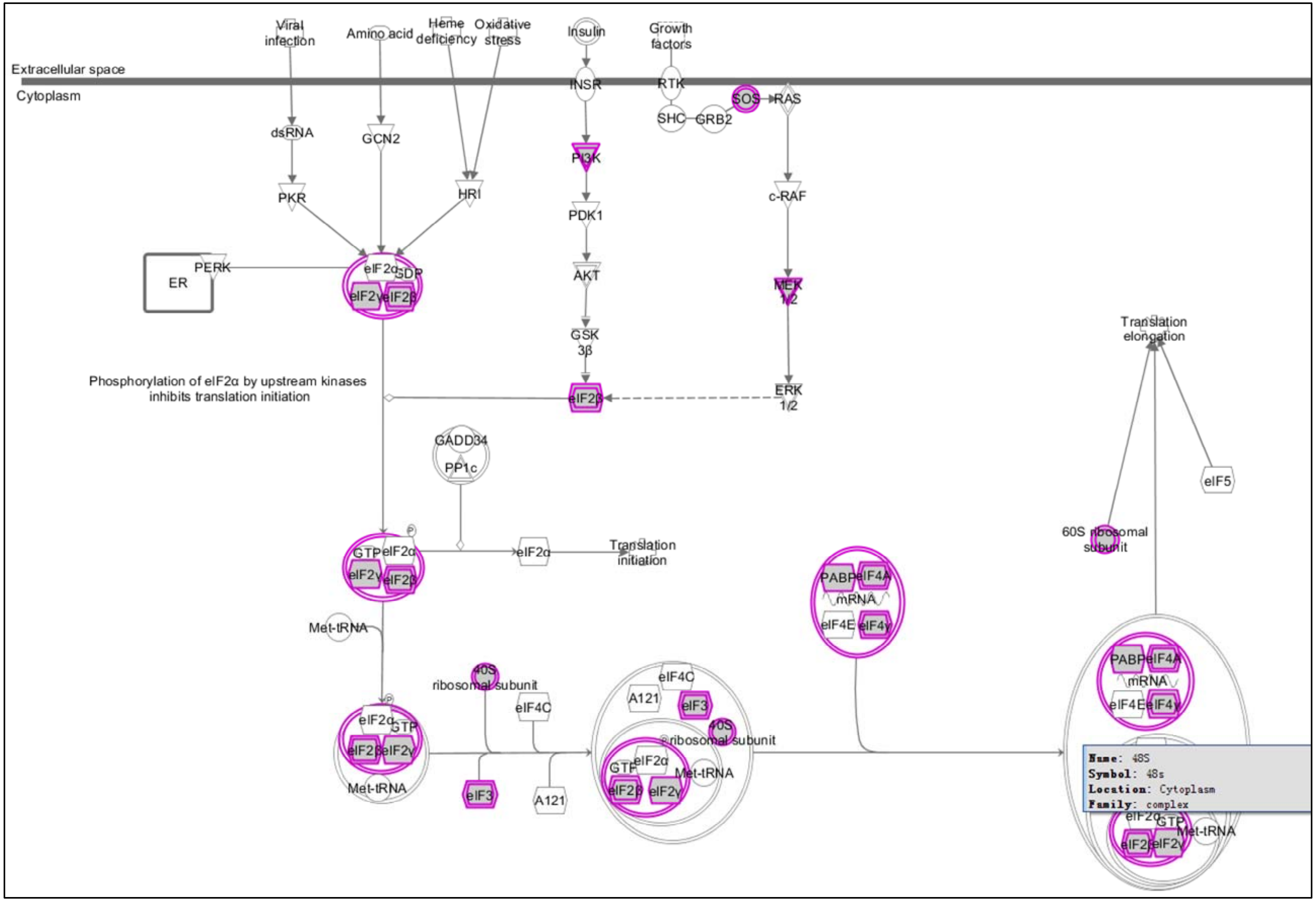

2-Protein Ubiquitination Pathway

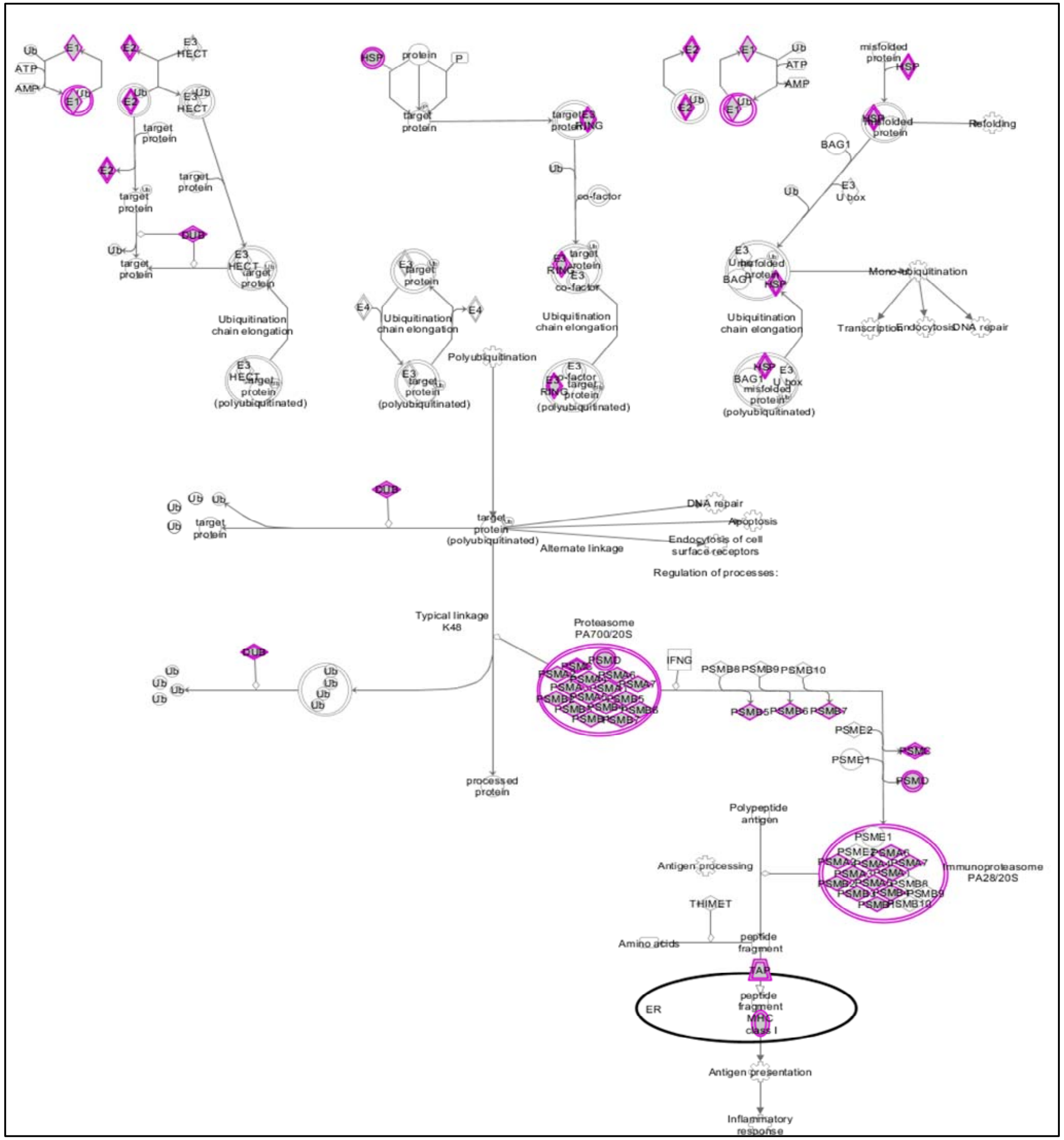

3-Mitochondrial Dysfunction

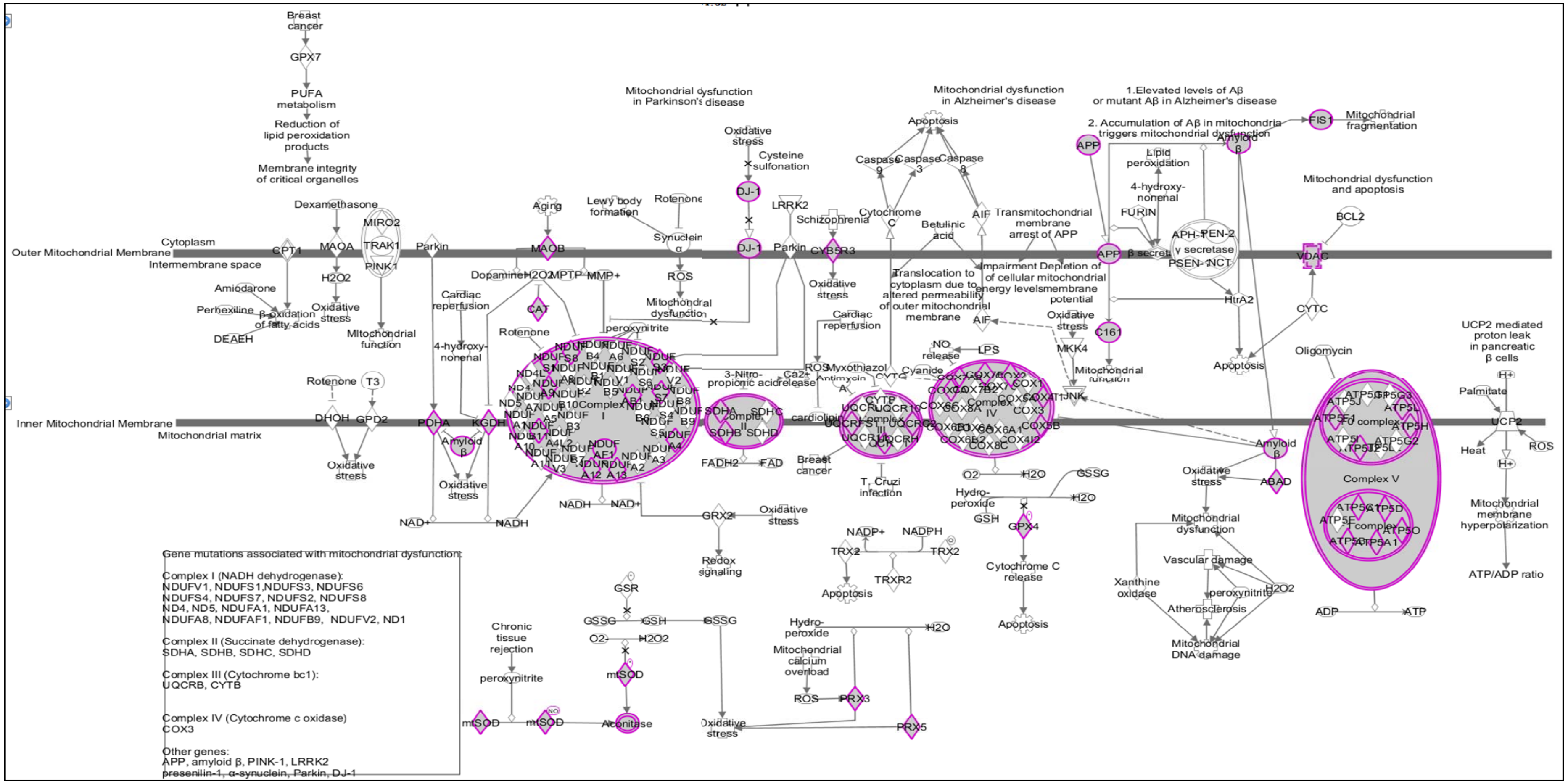

4-Regulation of eIF4 and p70S6K Signaling

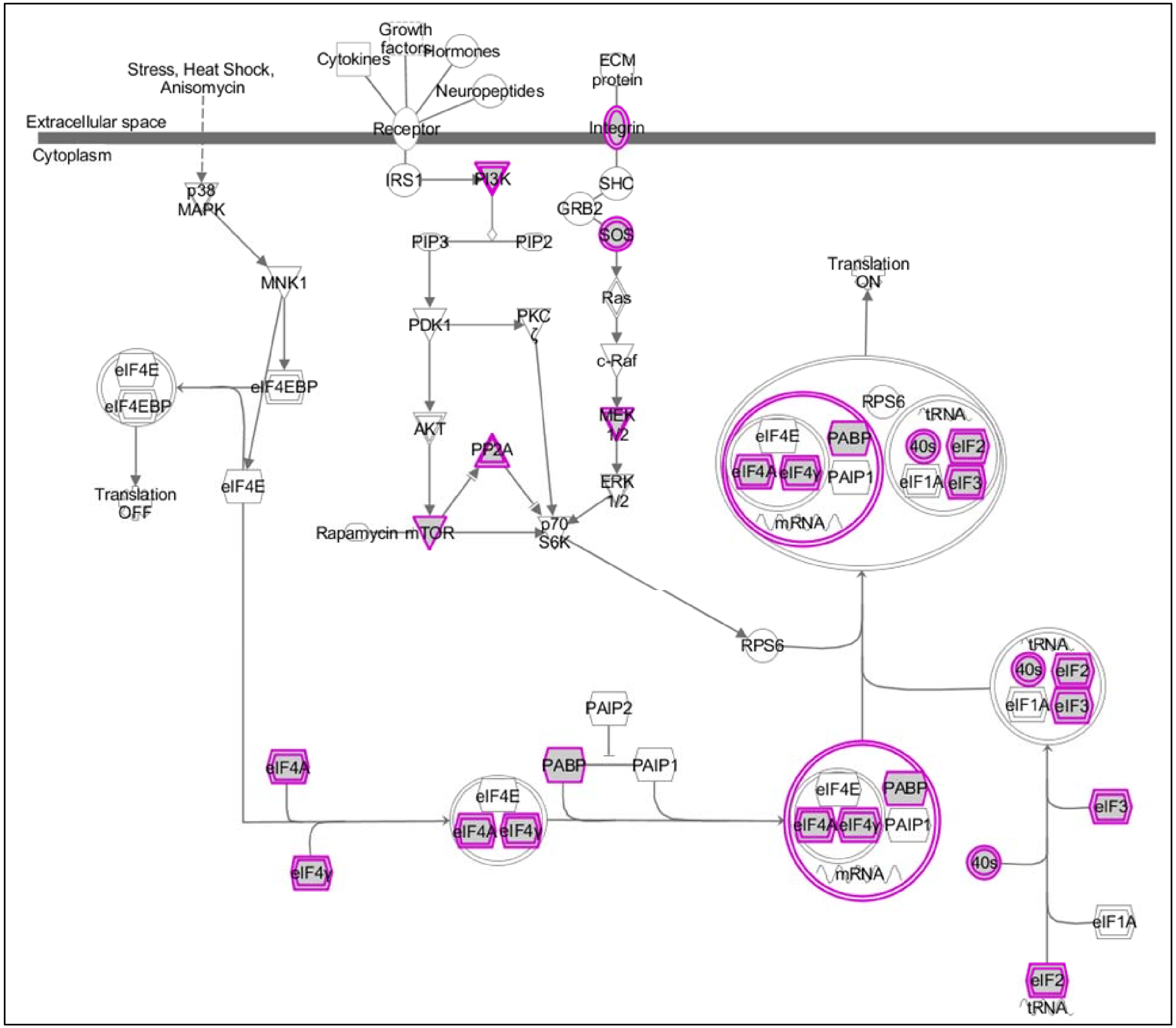

5-Oxidative Phosphorylation

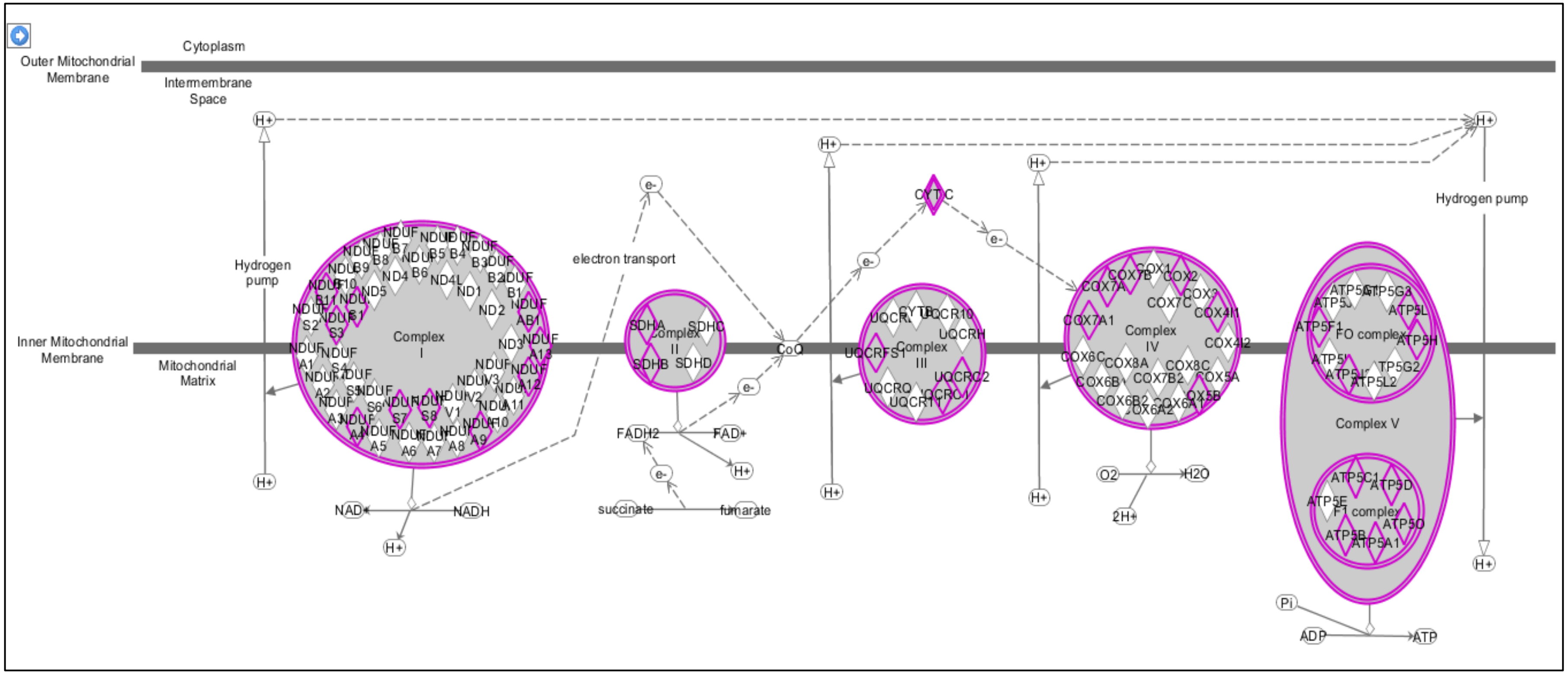

6-NRF2-mediated Oxidative Stress Response

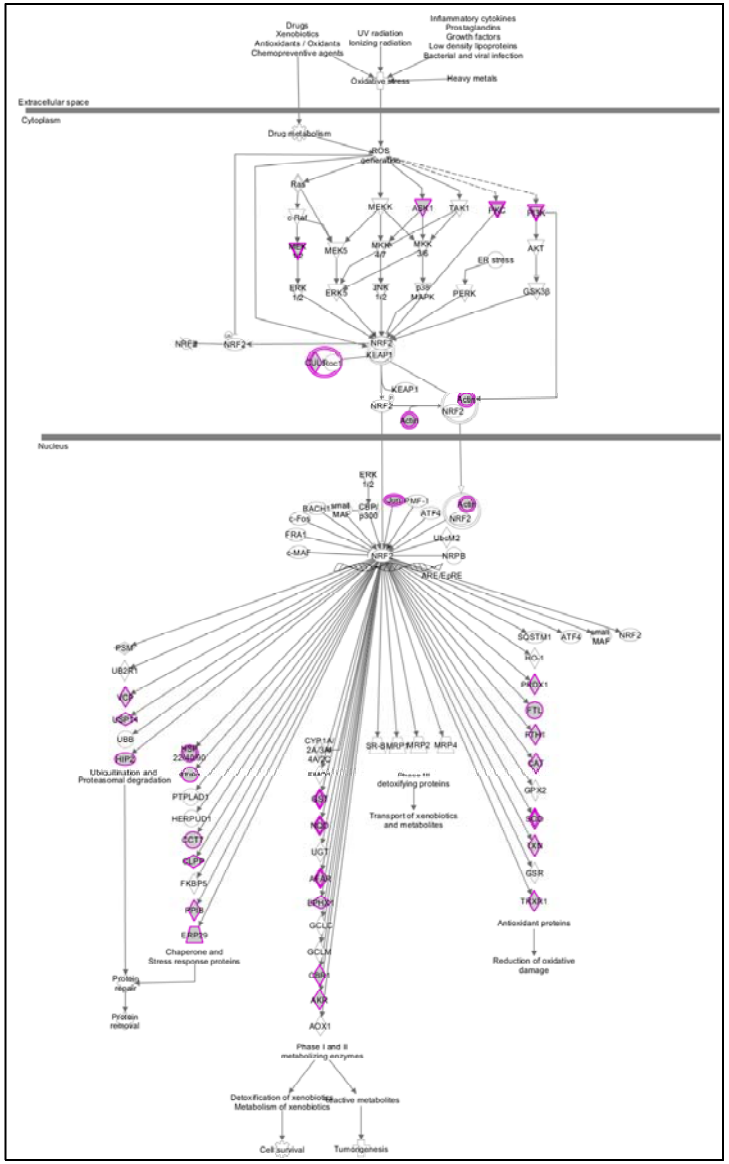

7-Remodeling of Epithelial Adherences Junction

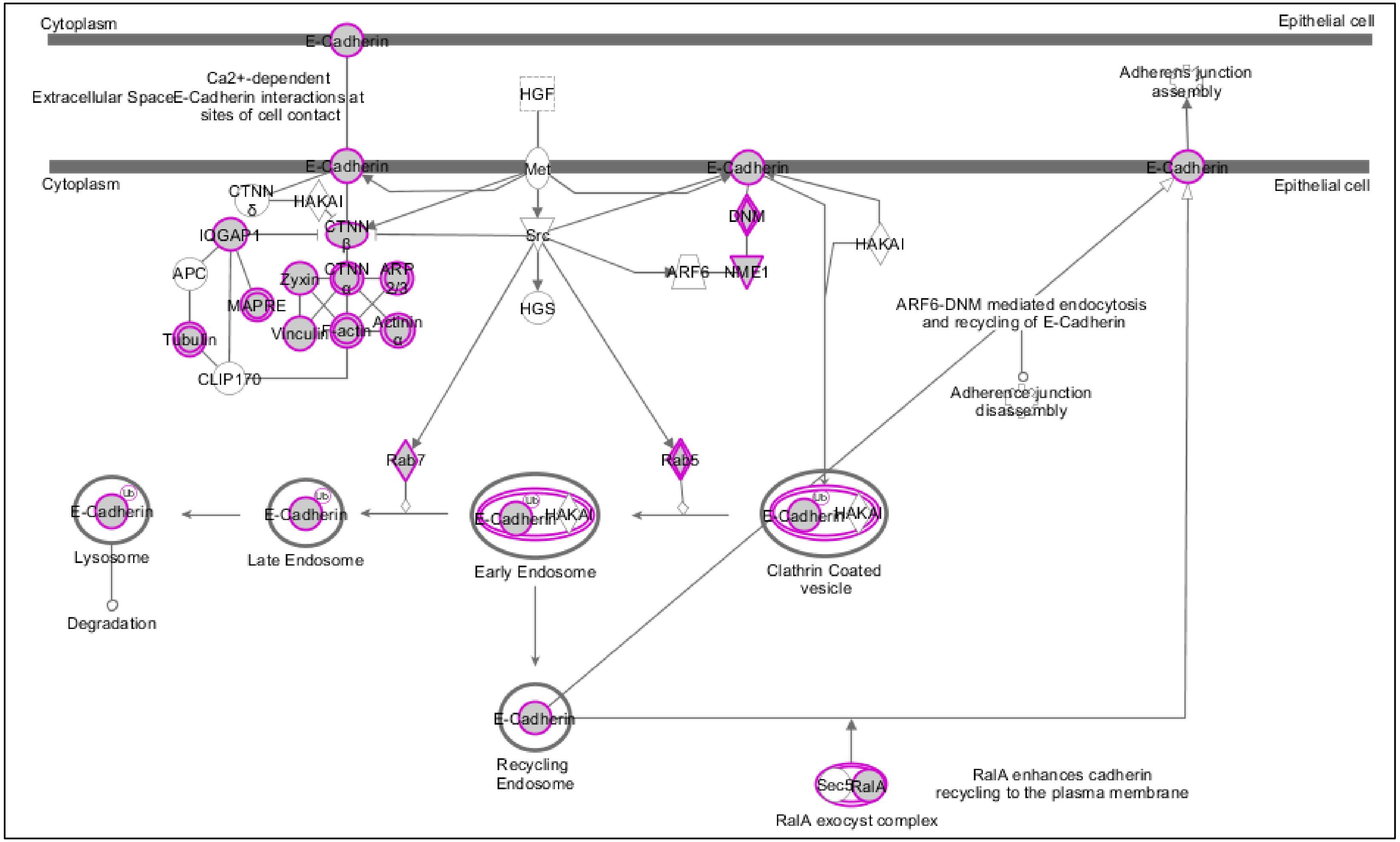

8-Glycolysis Ⅰ

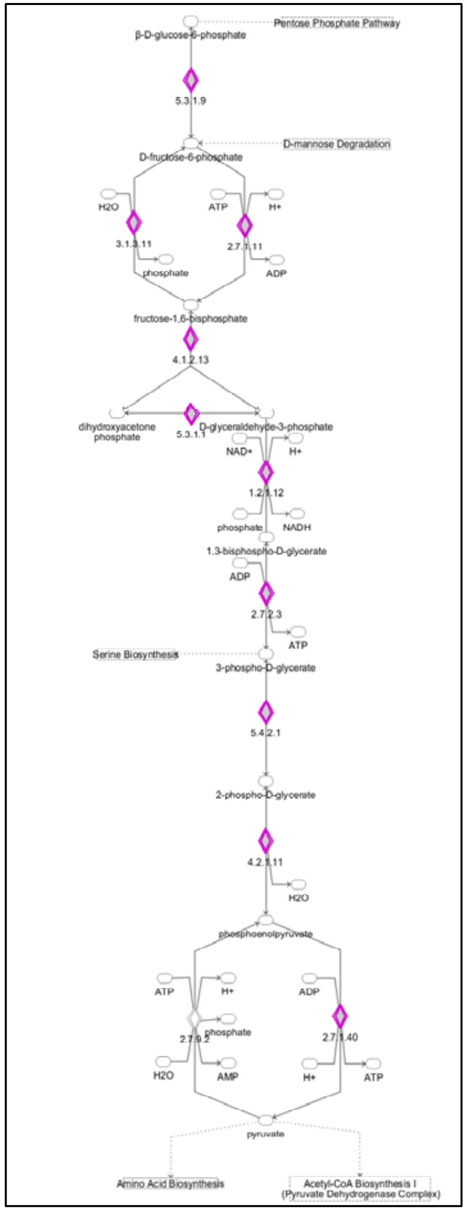

9-Unfolded protein response

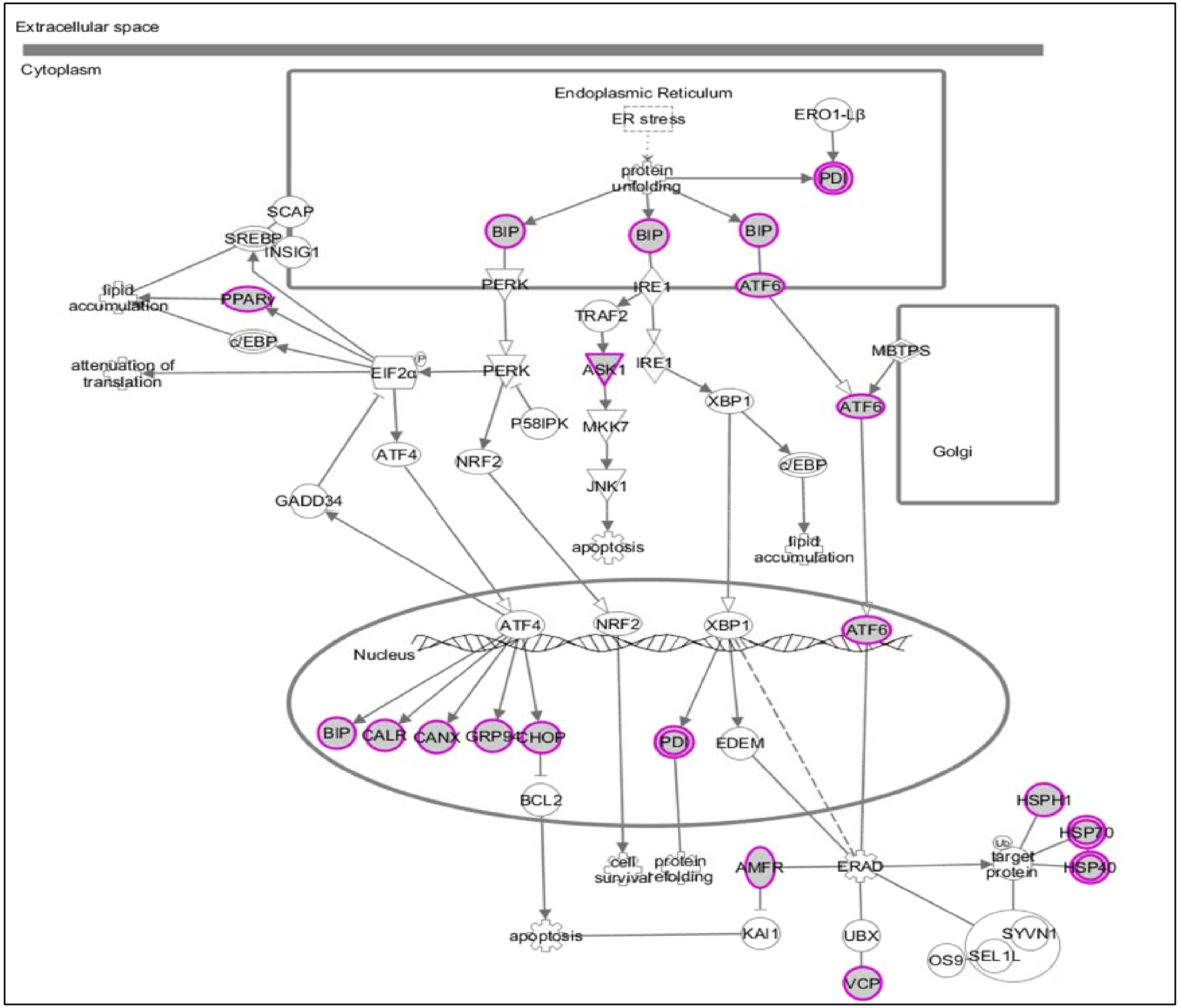

10-Clathrin-mediated Endocytosis Signling

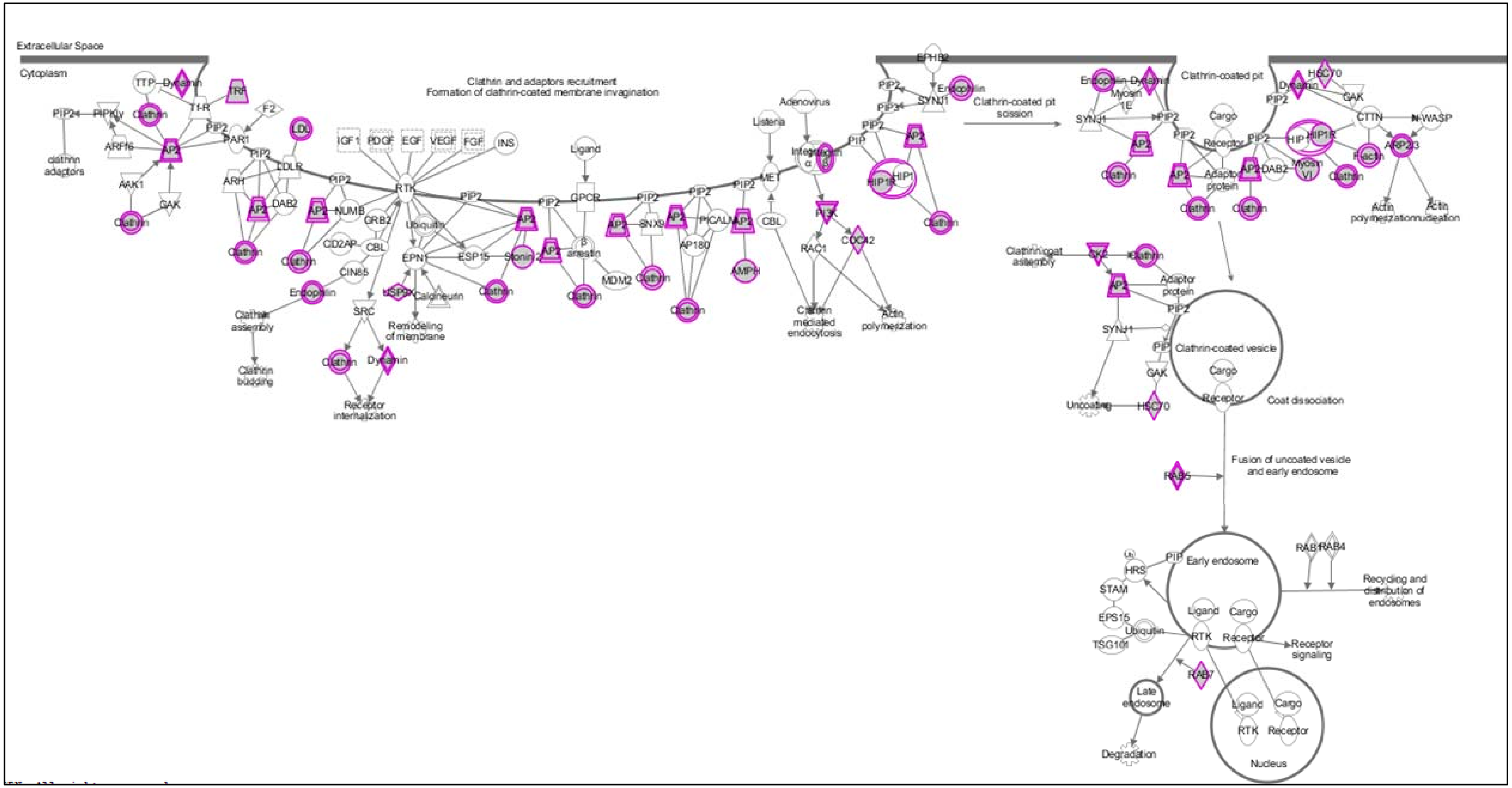

11-Fatty Acid β -oxidation Ⅰ

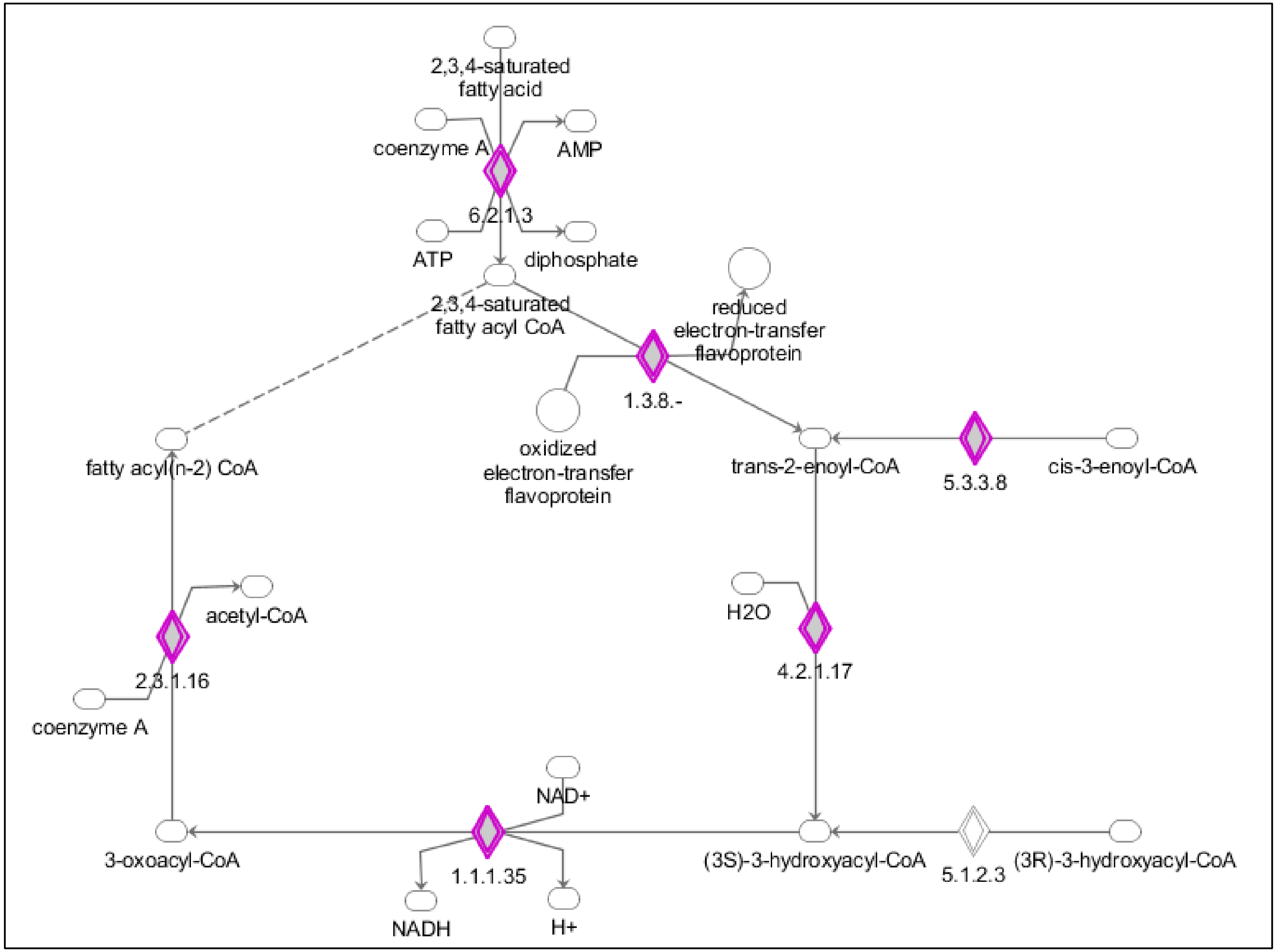

12-Gluconeogenesis Ⅰ

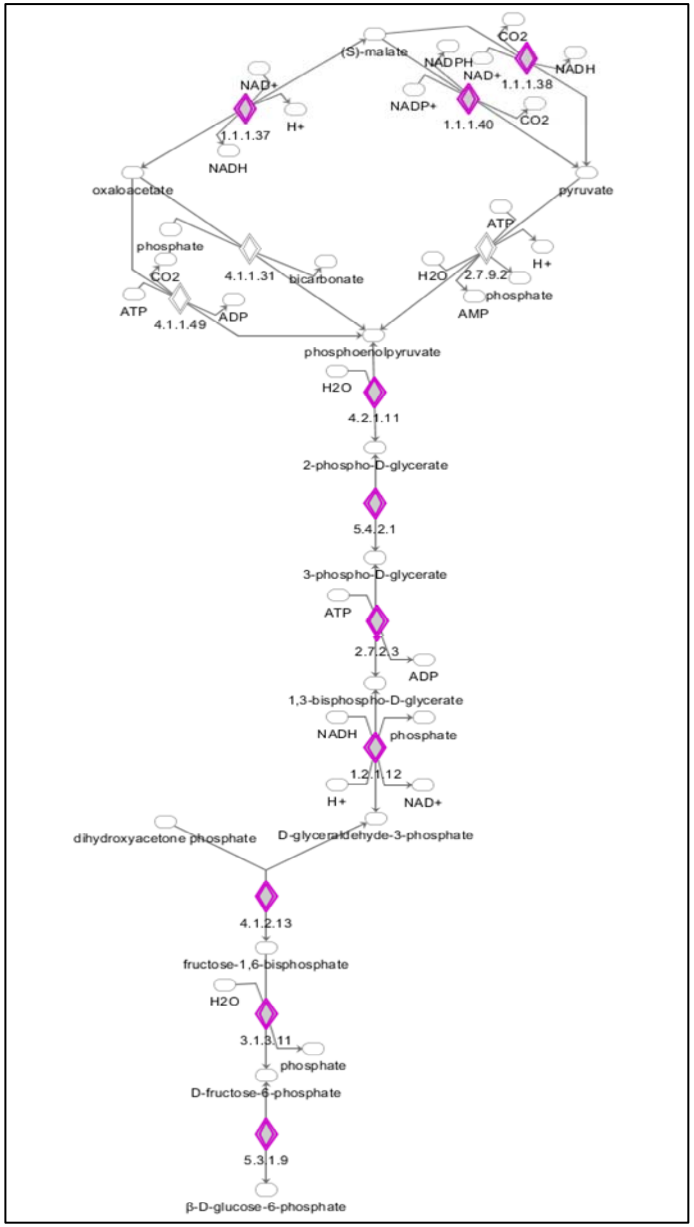

13-Aldosterone Signaling in Epithelial Cells

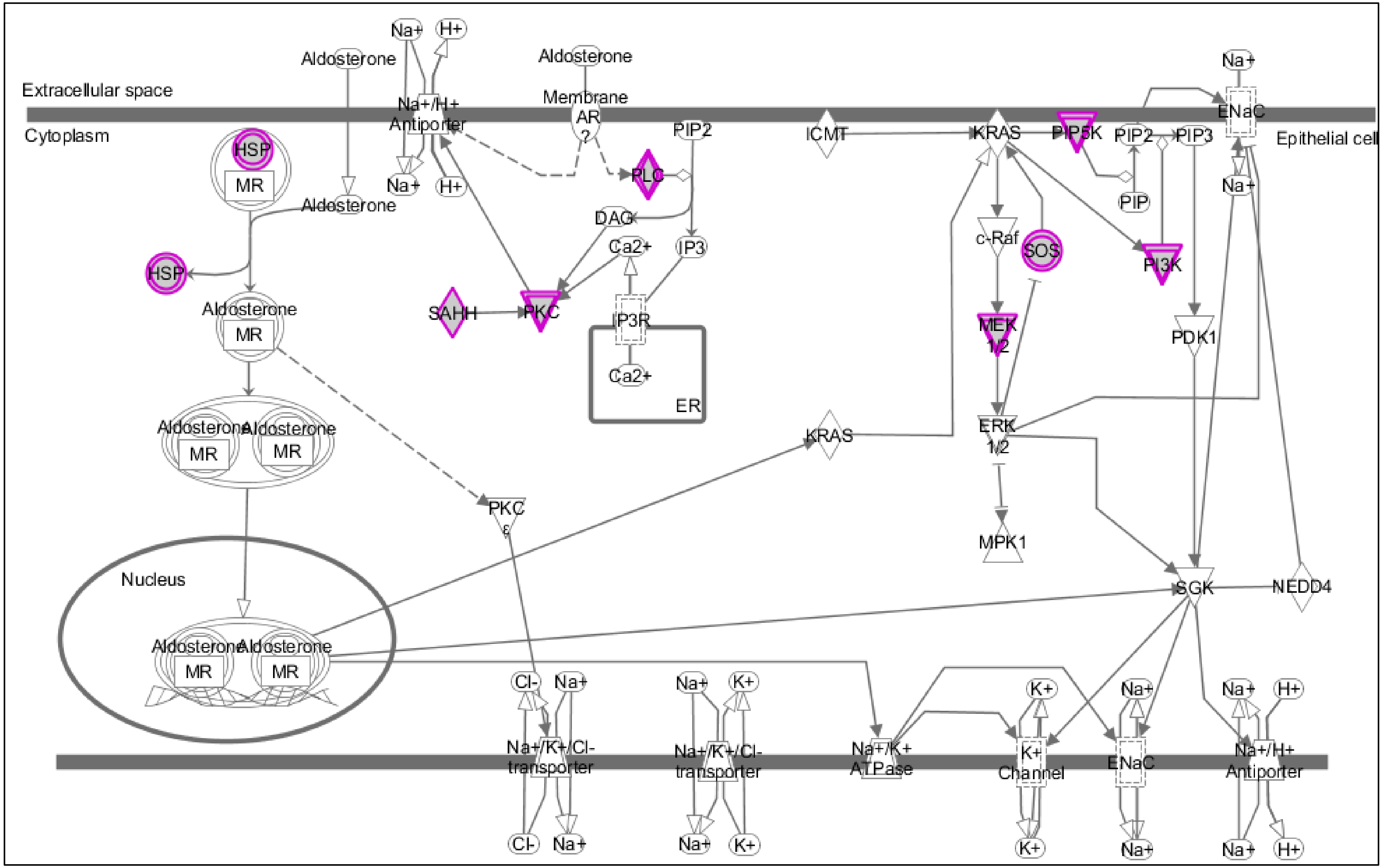

14-Granzyme A Signling

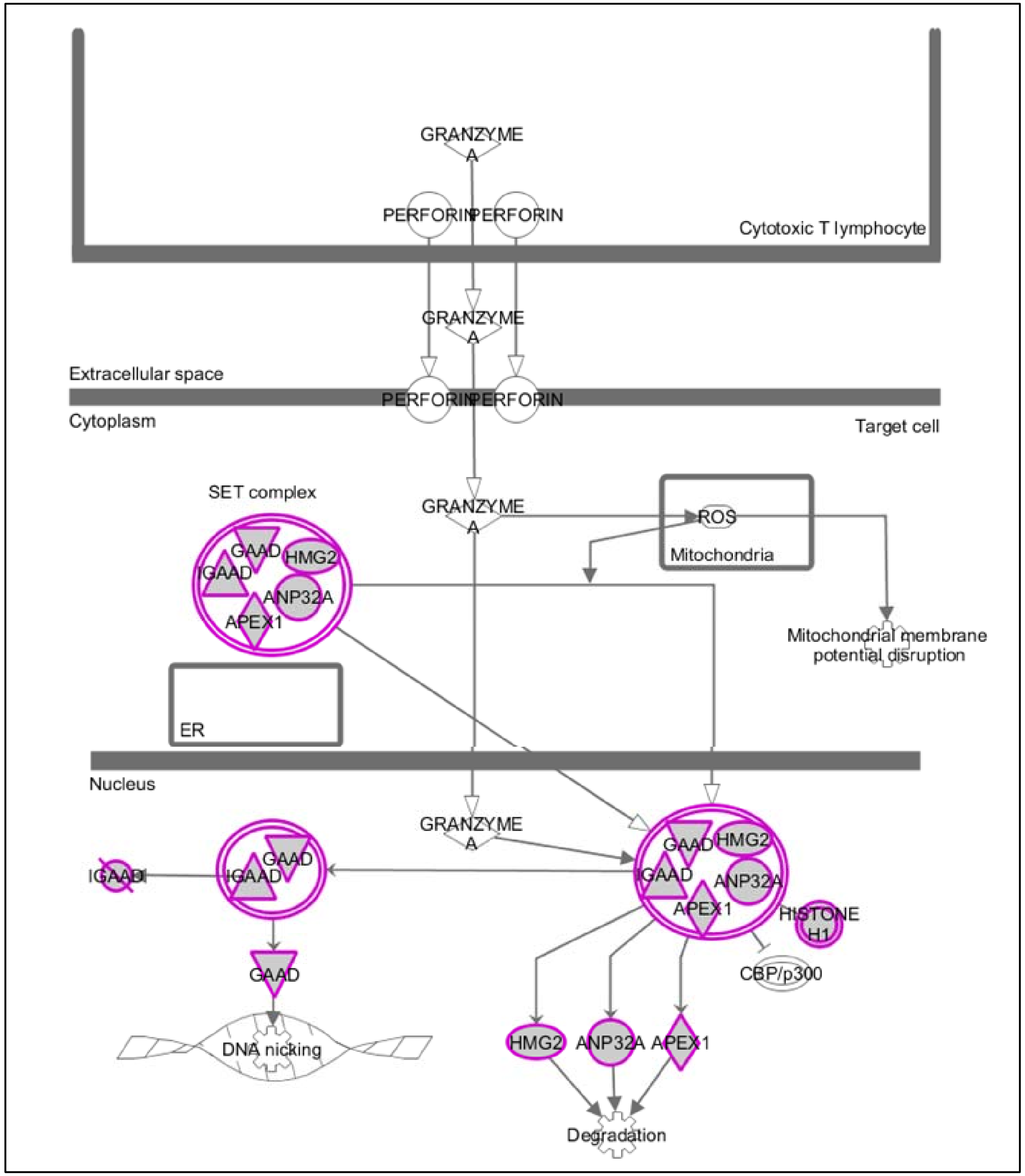

15-Caveolar-mediated Endocytosis Signaling

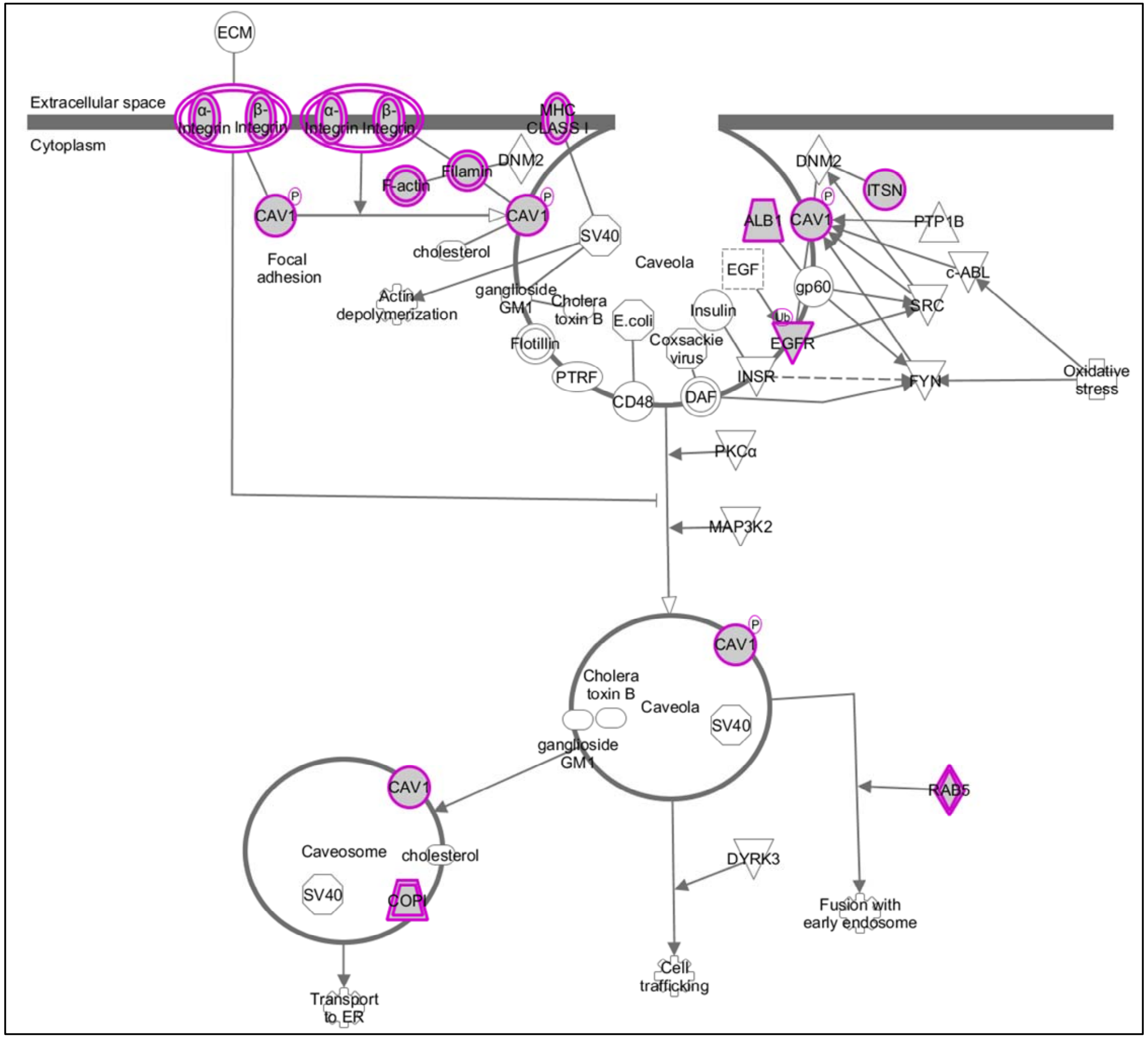

16-Actin Cytoskeleton Signaling

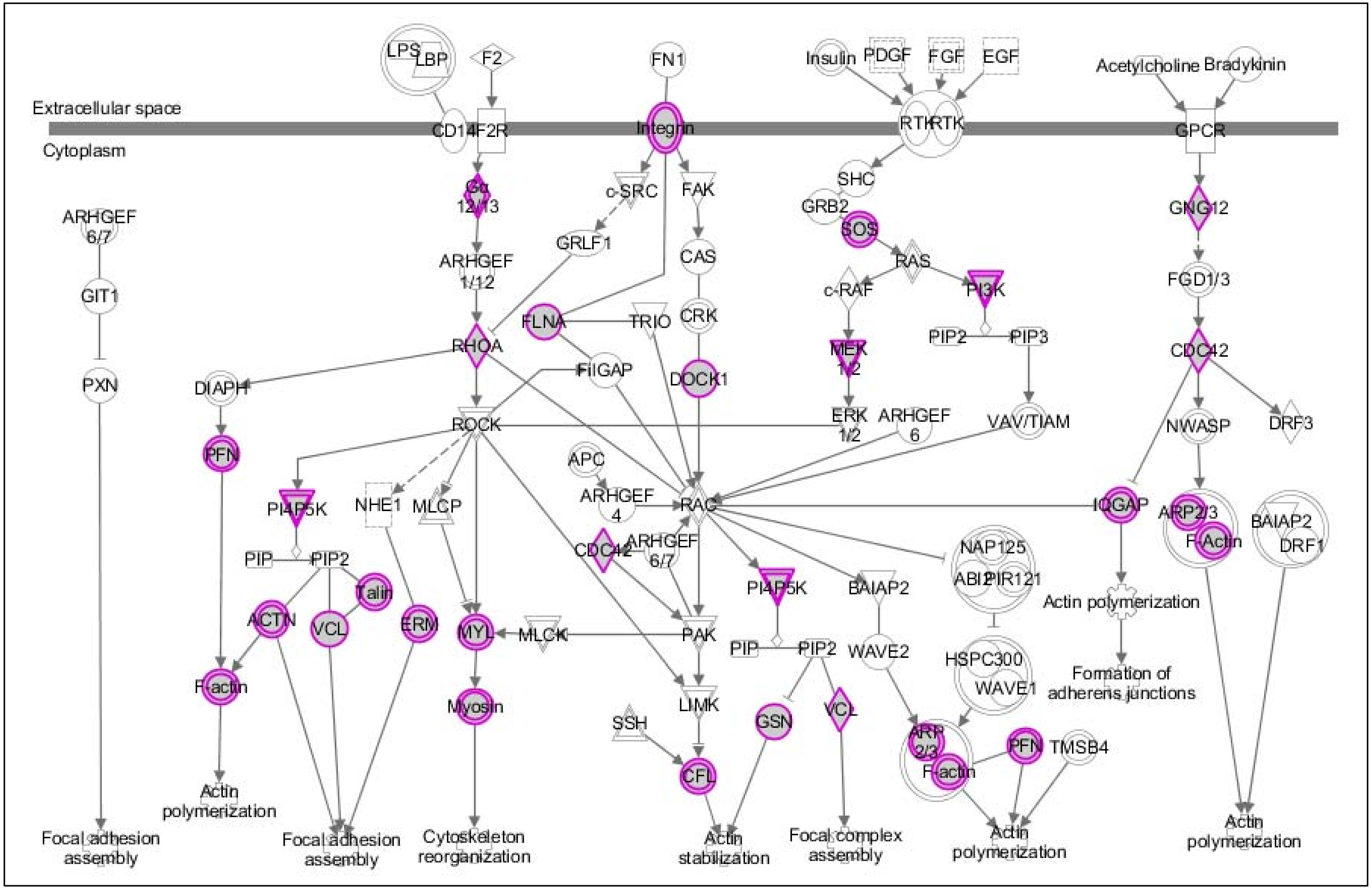

17-Epithelial Adherens Junction Signaling

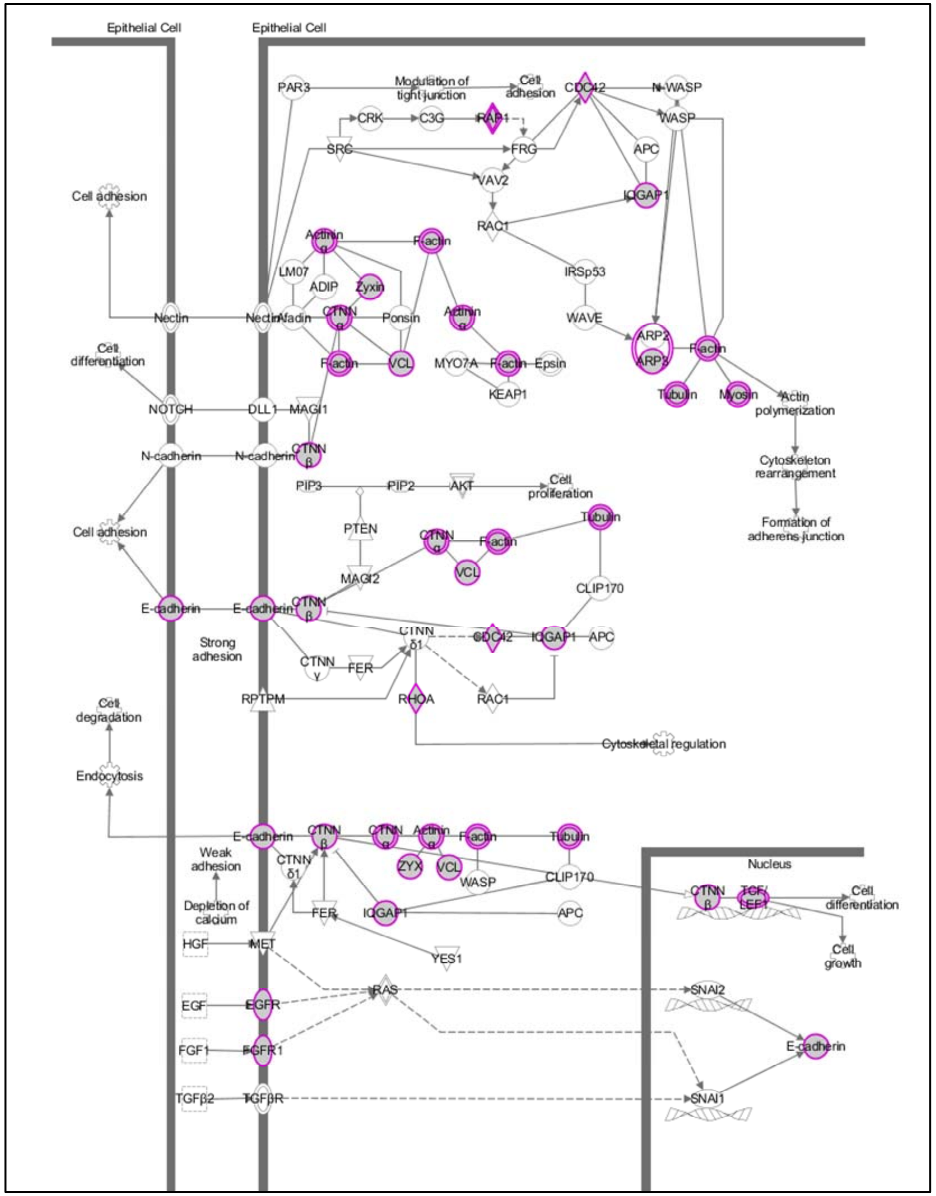

18-mTOR Signaling

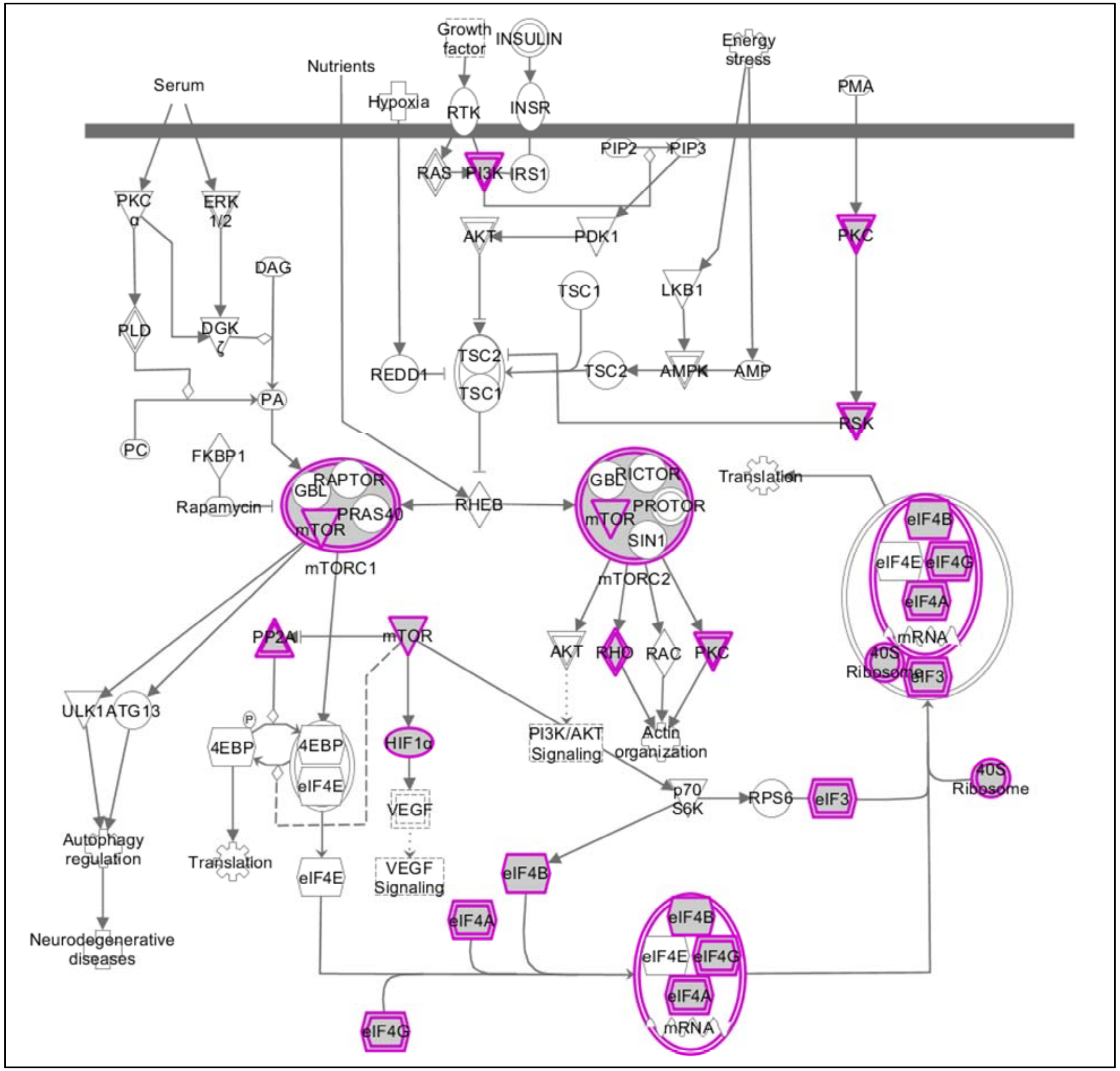

19-TCA Cycle Ⅱ(Eukaryotic)

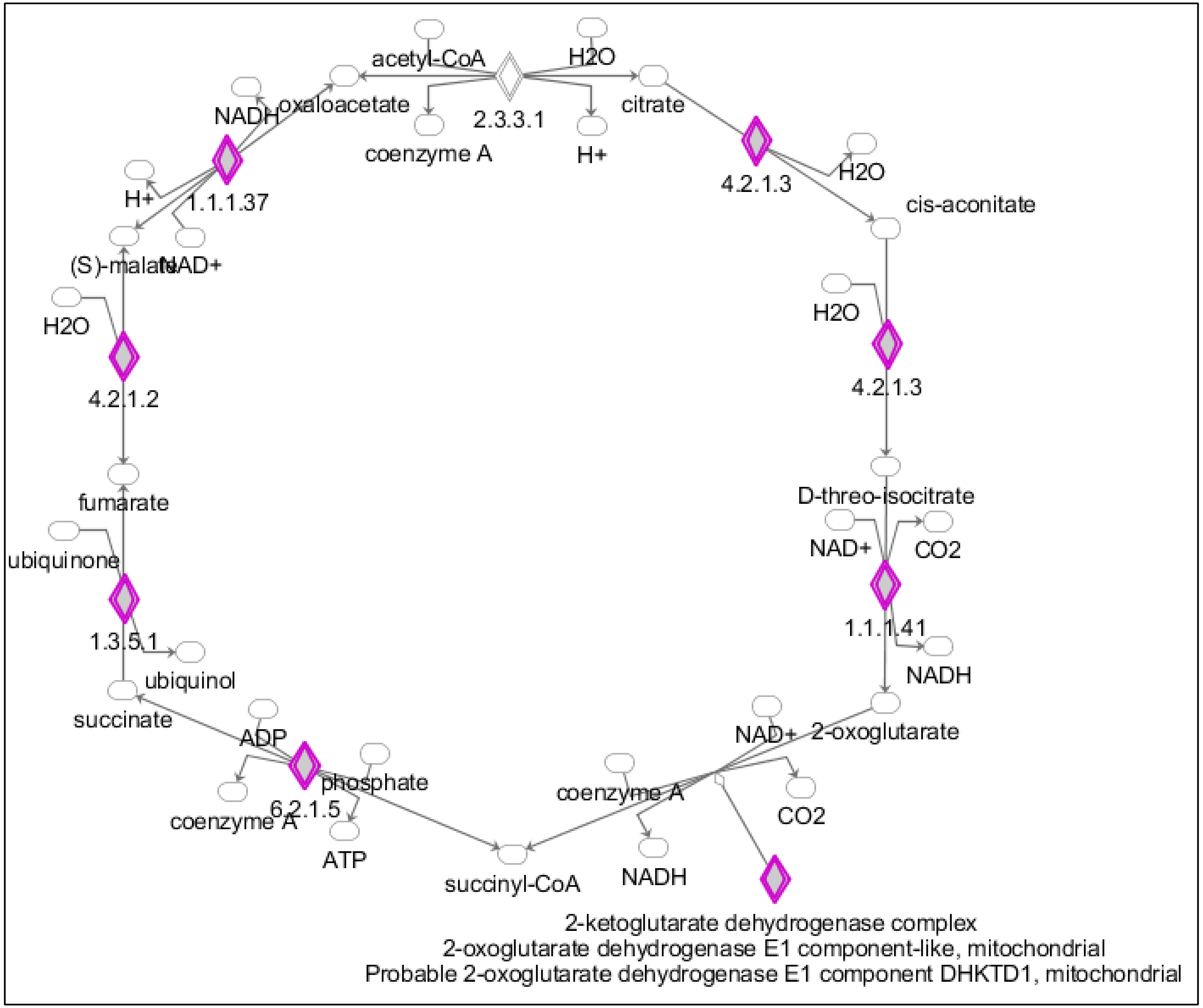

20-Integrin Signaling

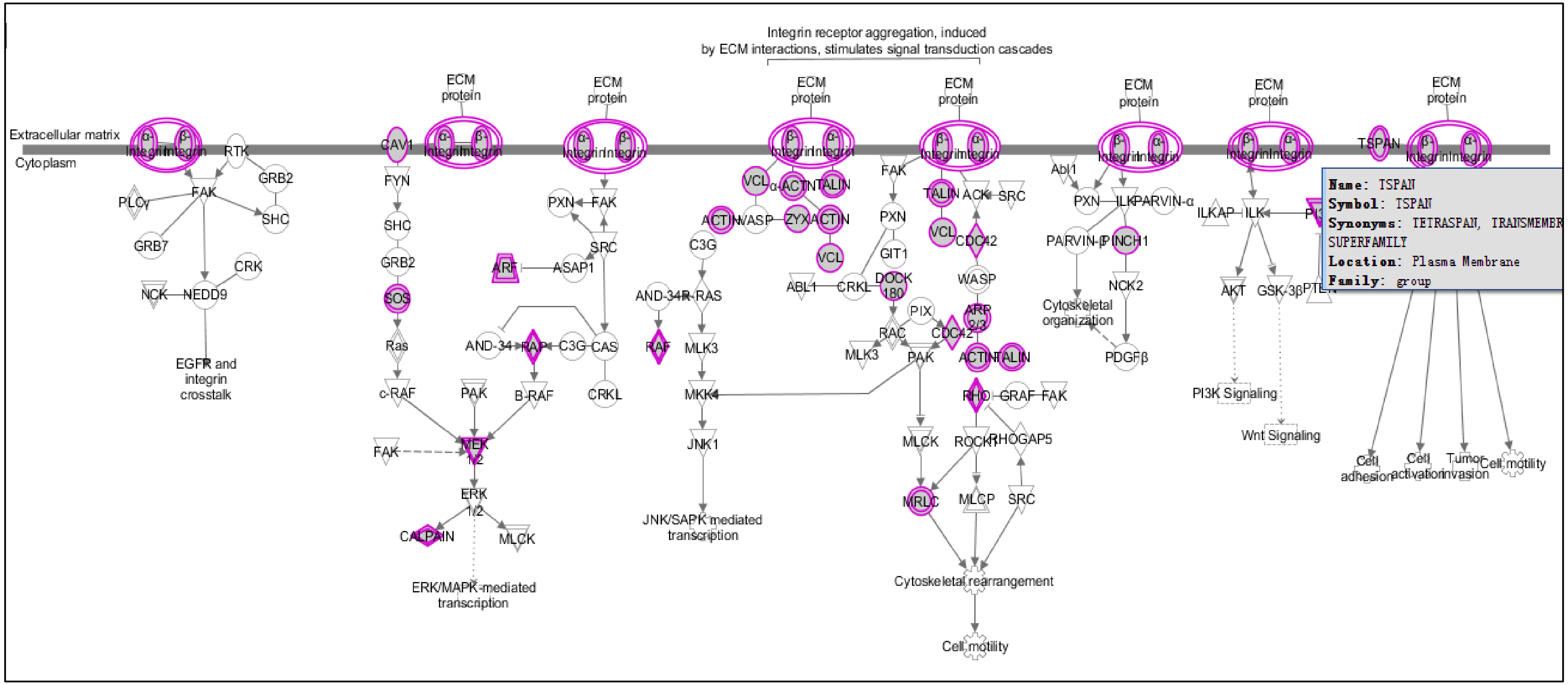

21-LXR-RXR Activation

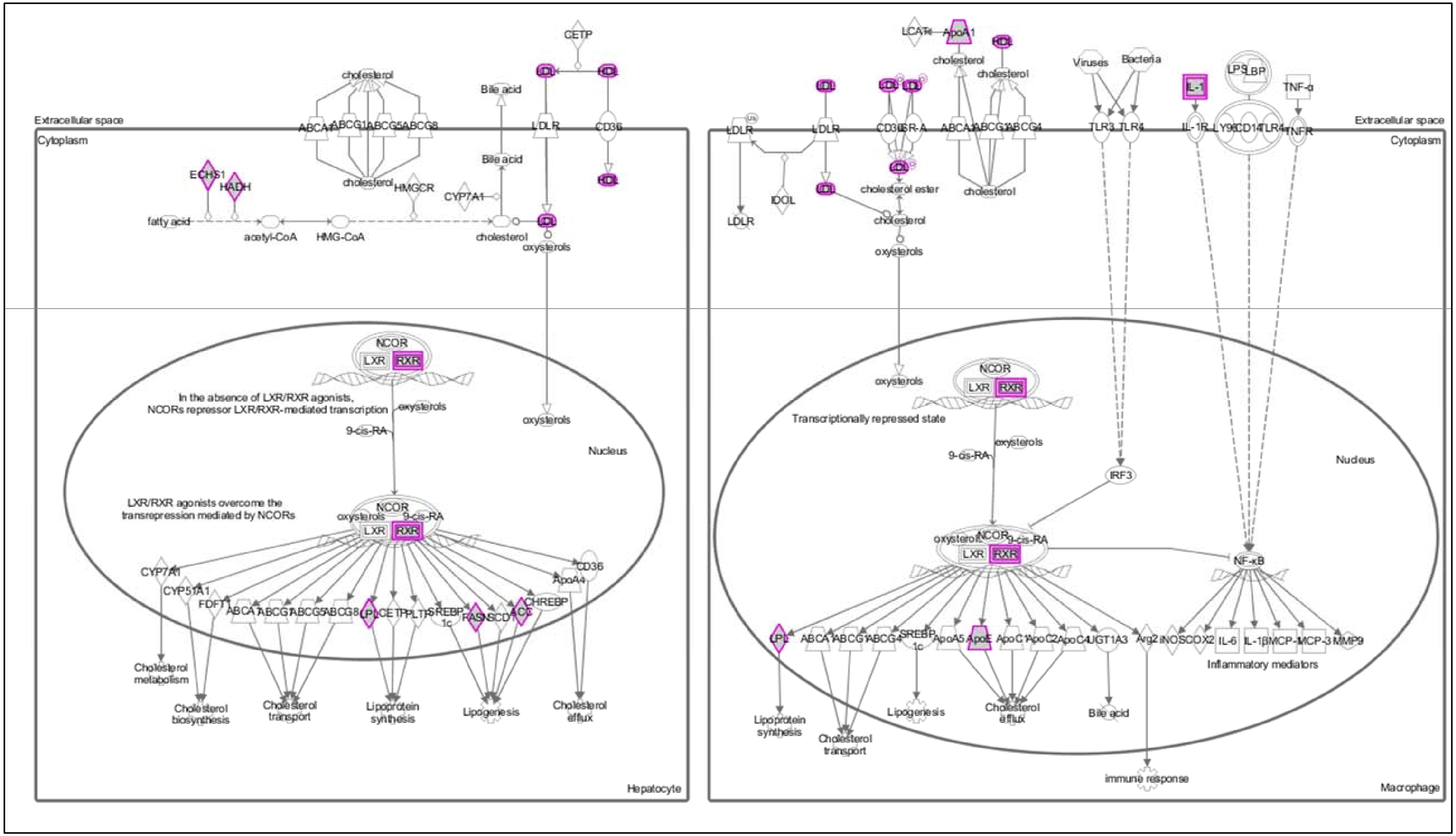

22-Huntington's Disease Signaling

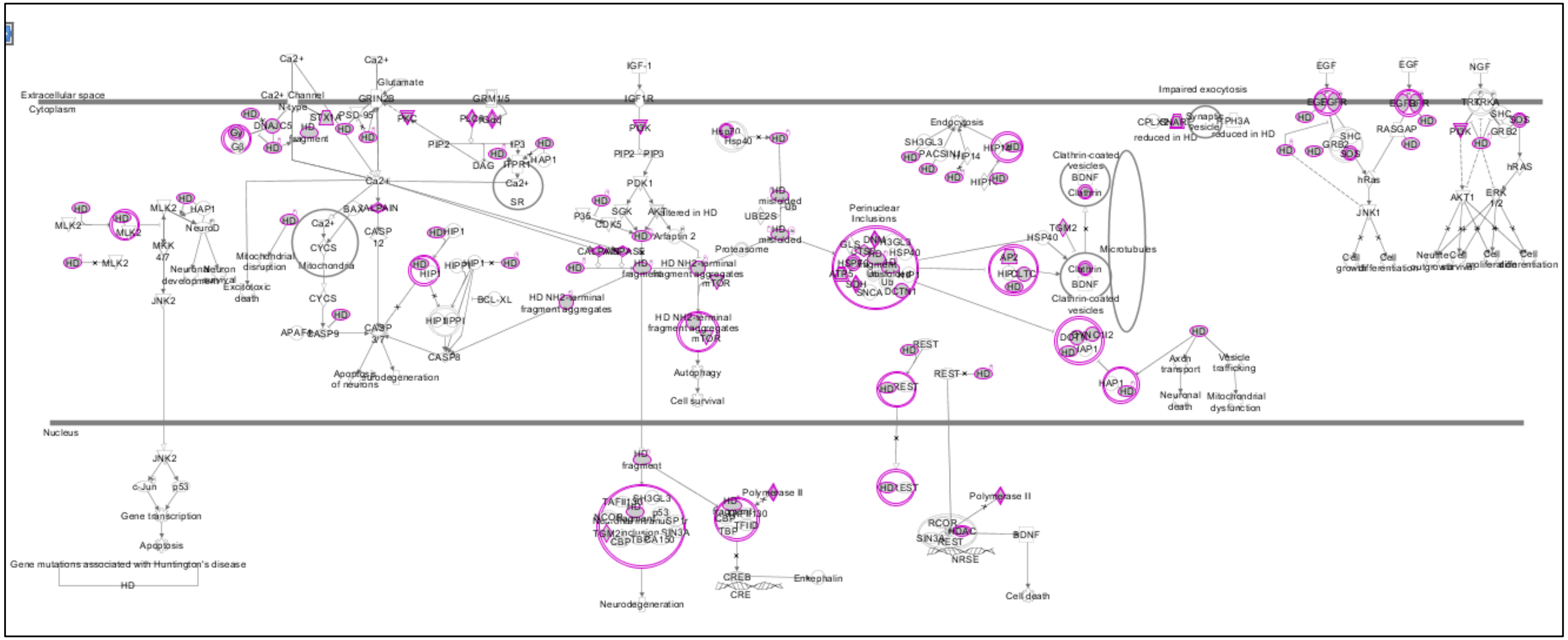

23-ILK Signaling

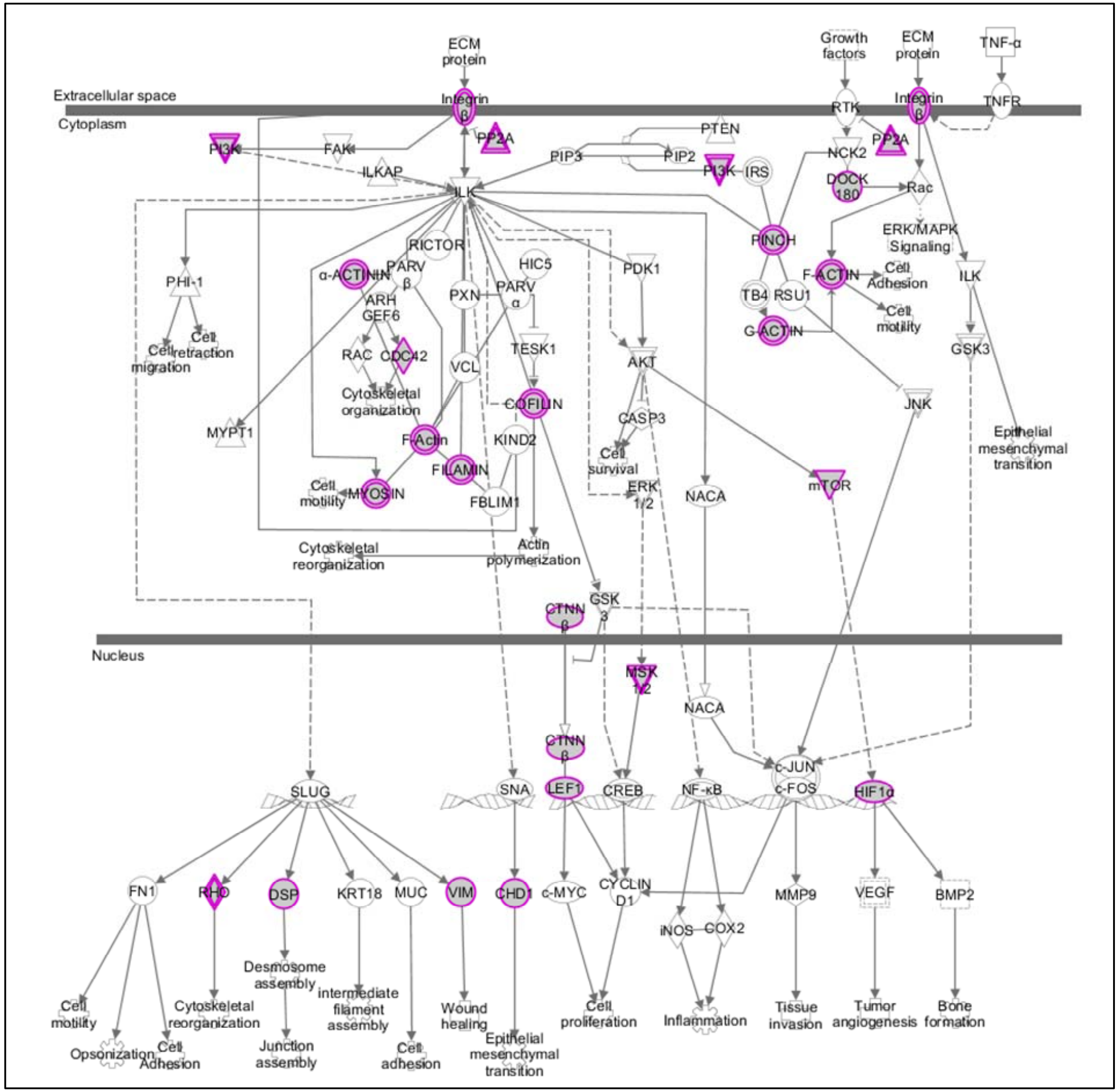

24-RhoGDI Signaling

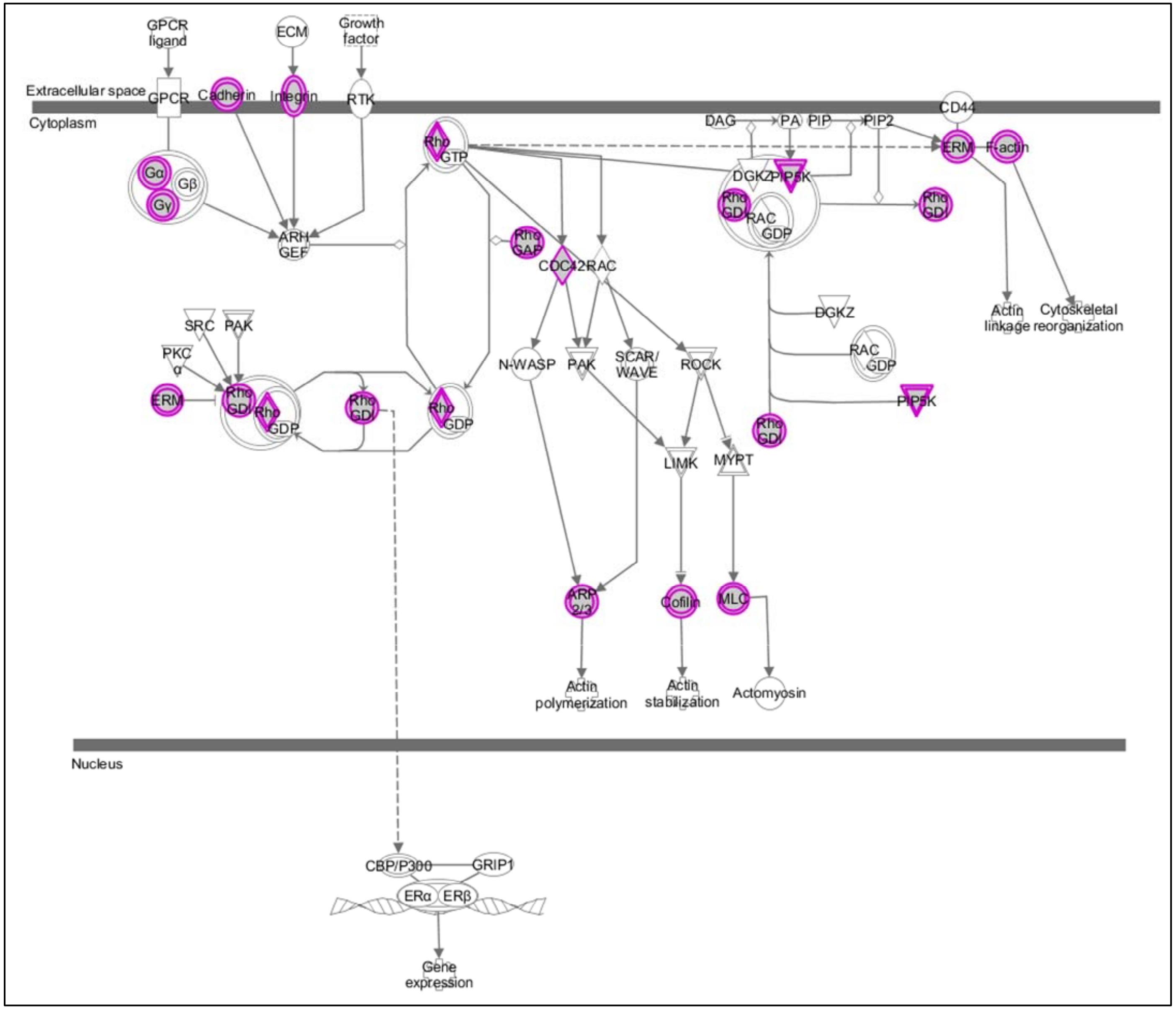

25-RhoA Signaling

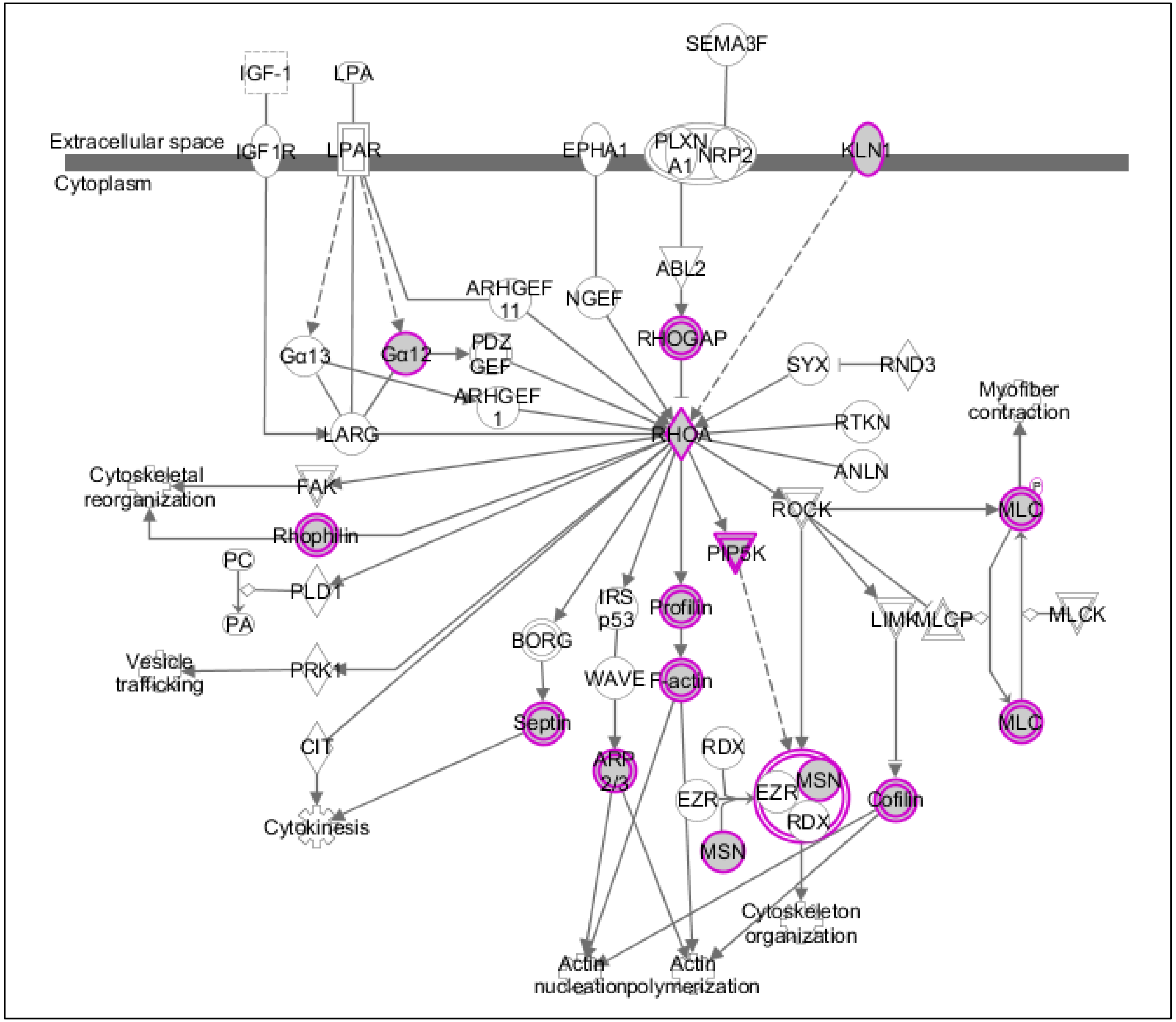

26-Acute Phase Response Signaling

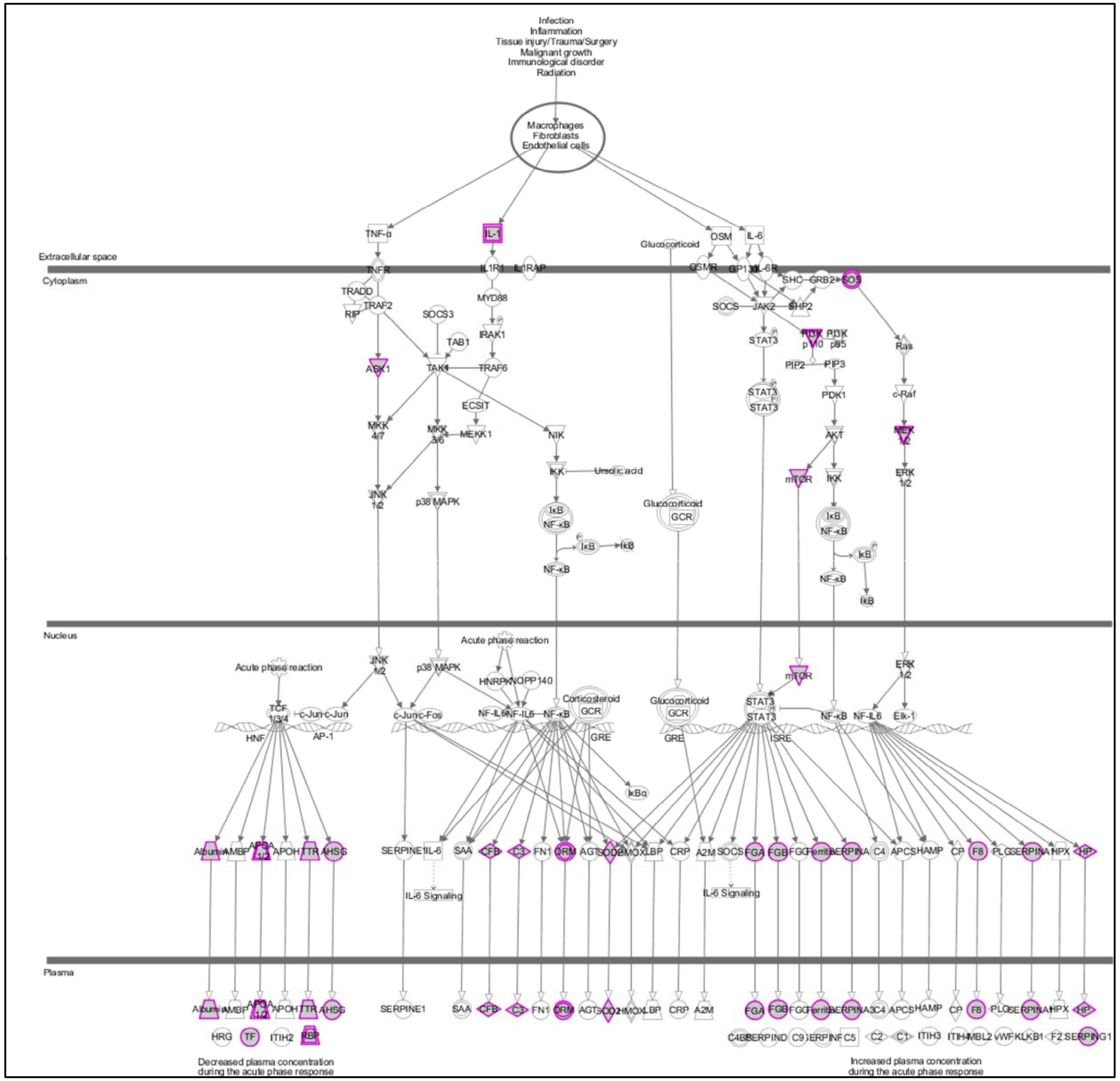

27-Regulation of Actin-based Motility by Rho

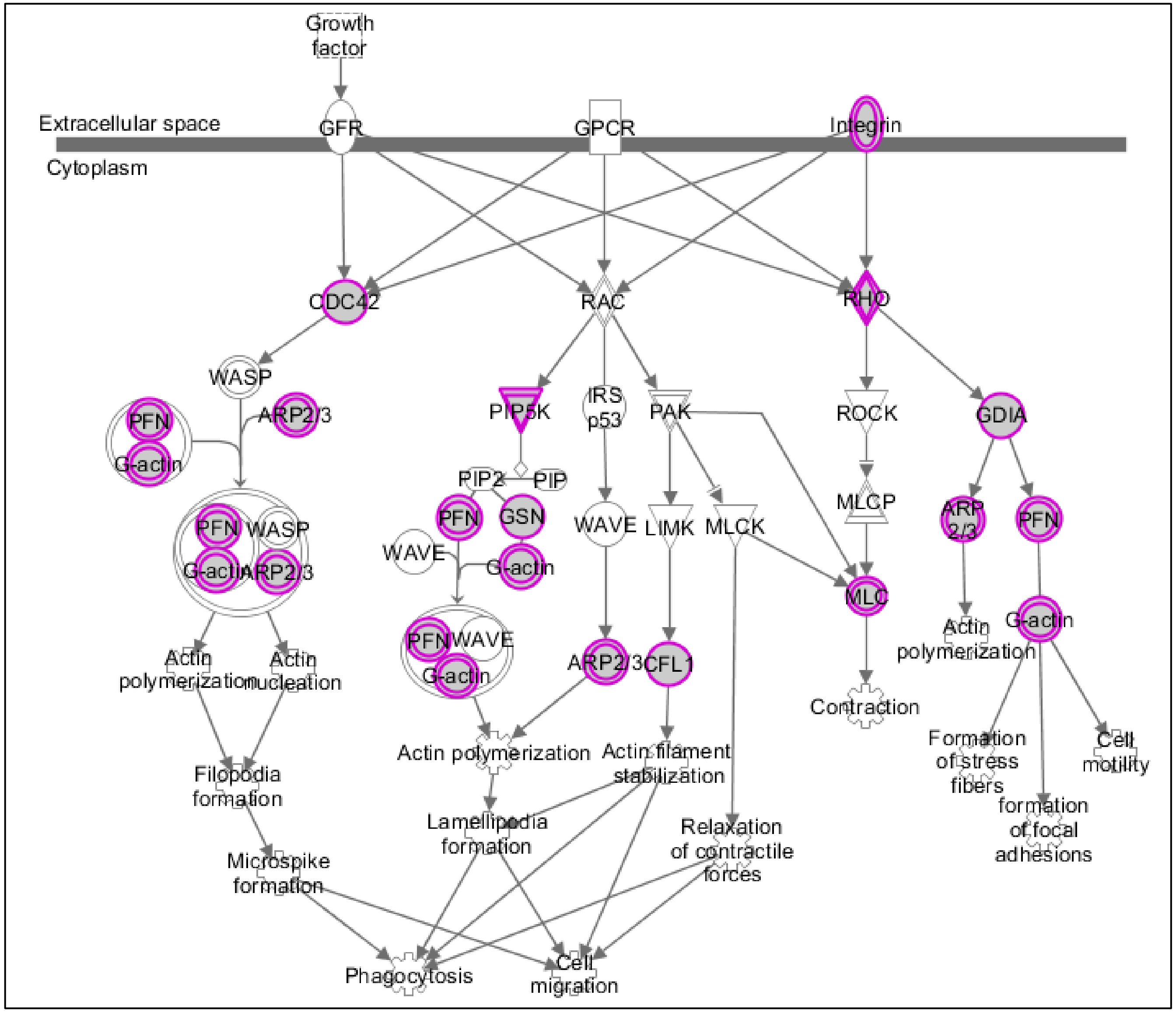

28-FXR-RXR Activation

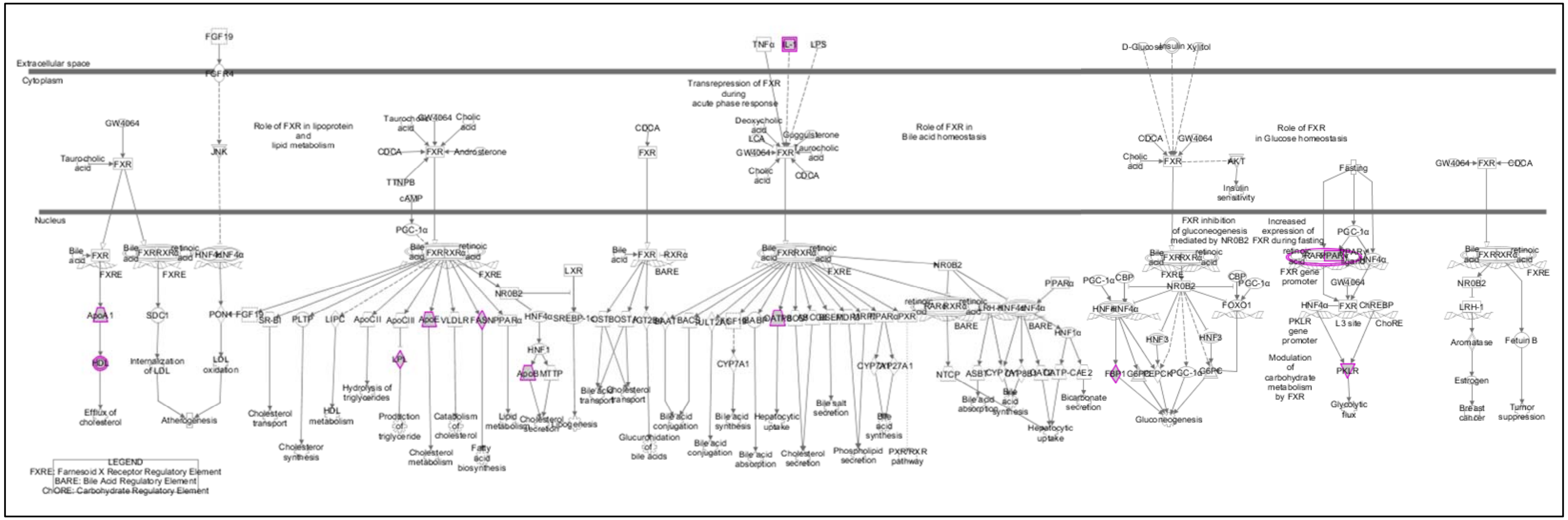

29-Signaling by Rho Family GTPases

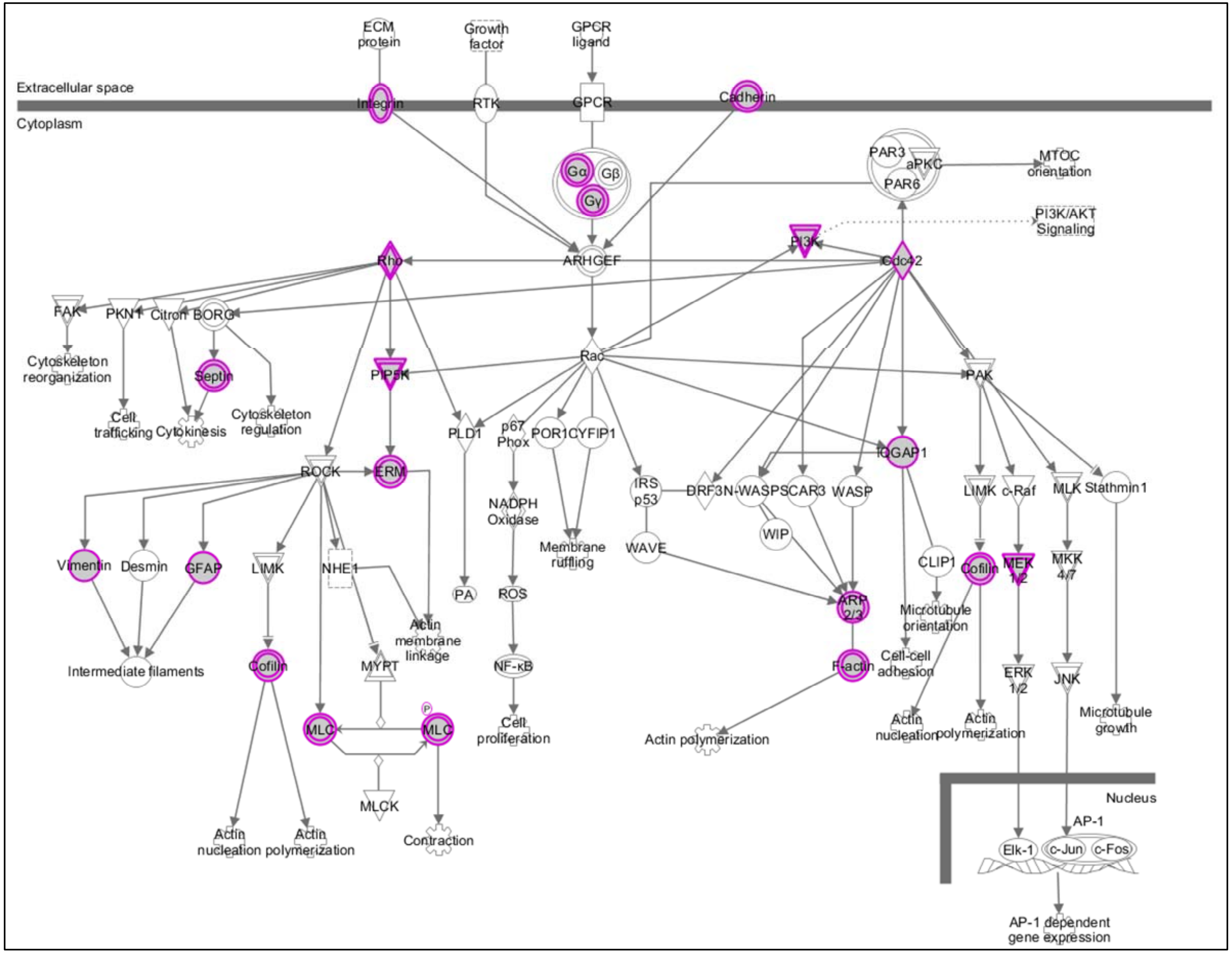

30-Ethanol Degradation Ⅱ

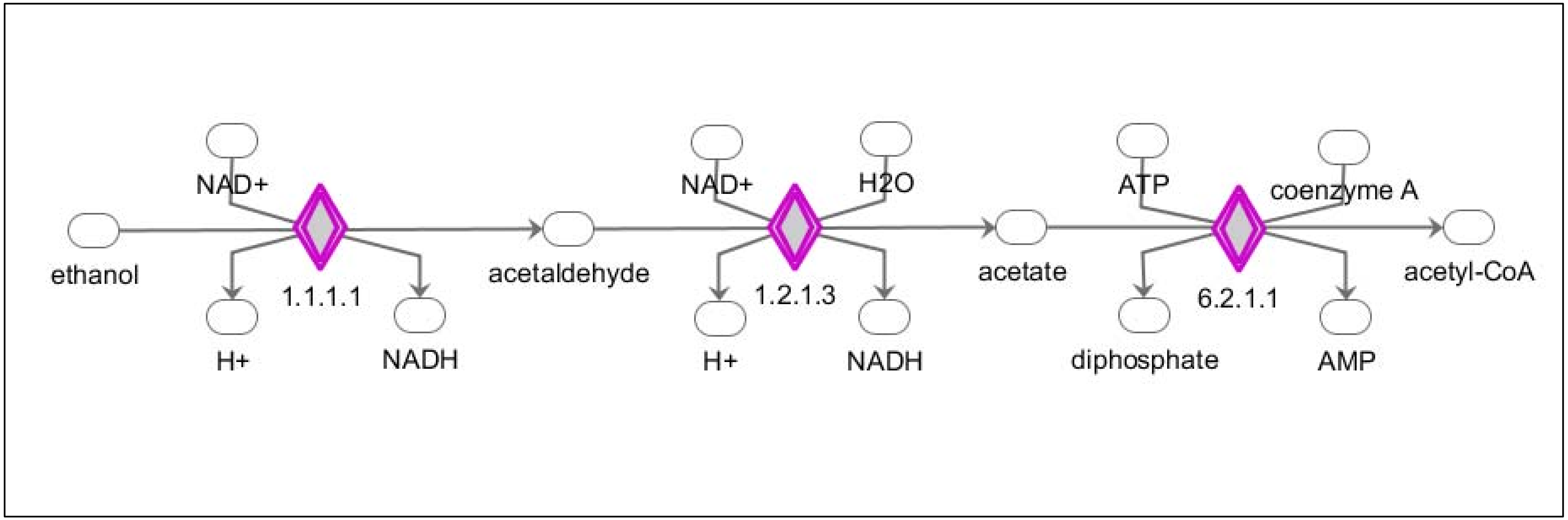

31-AryI Hydrocarbon Receptor Signaling

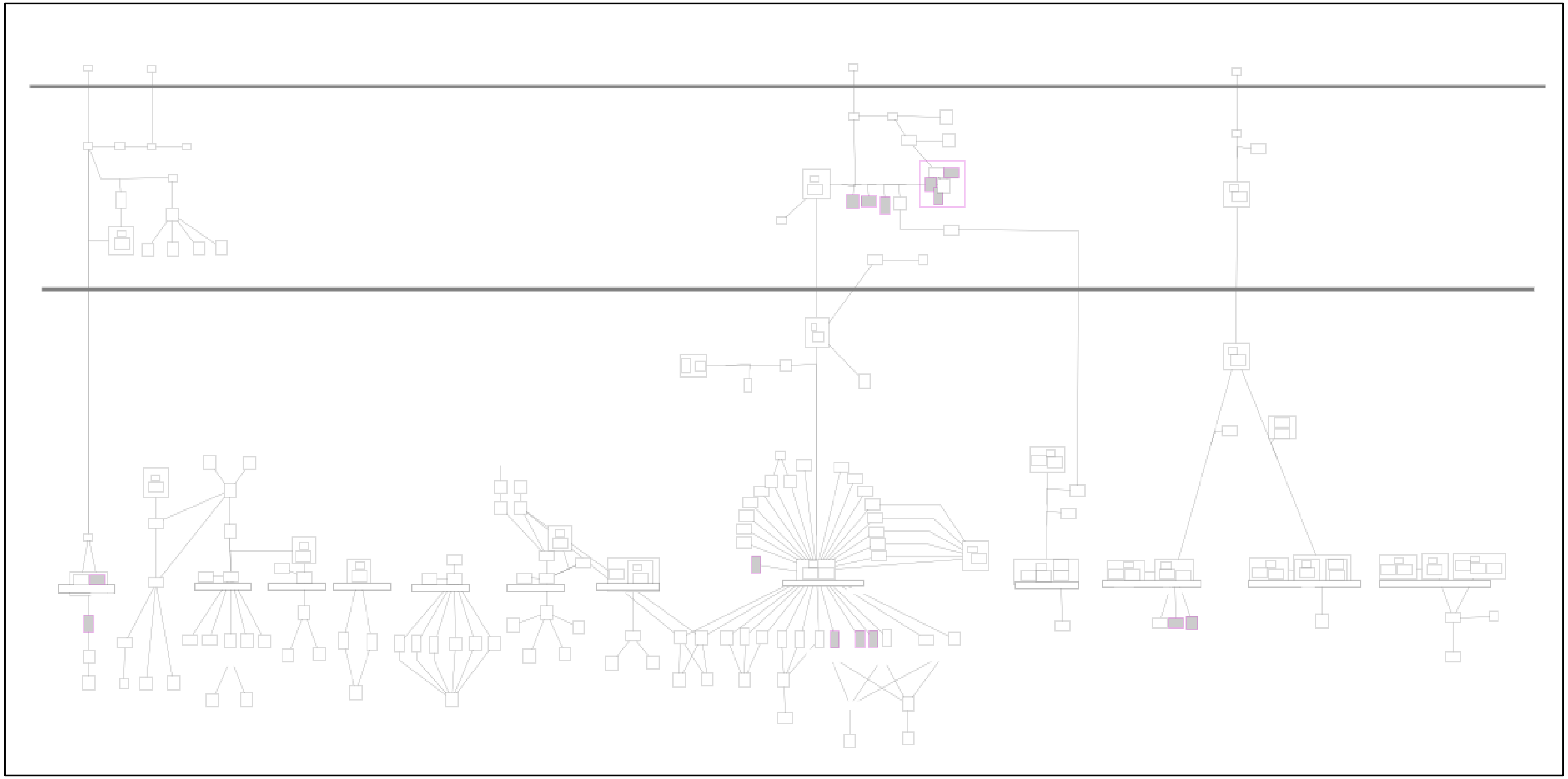

32-Xenobiotic Metabolism Signaling

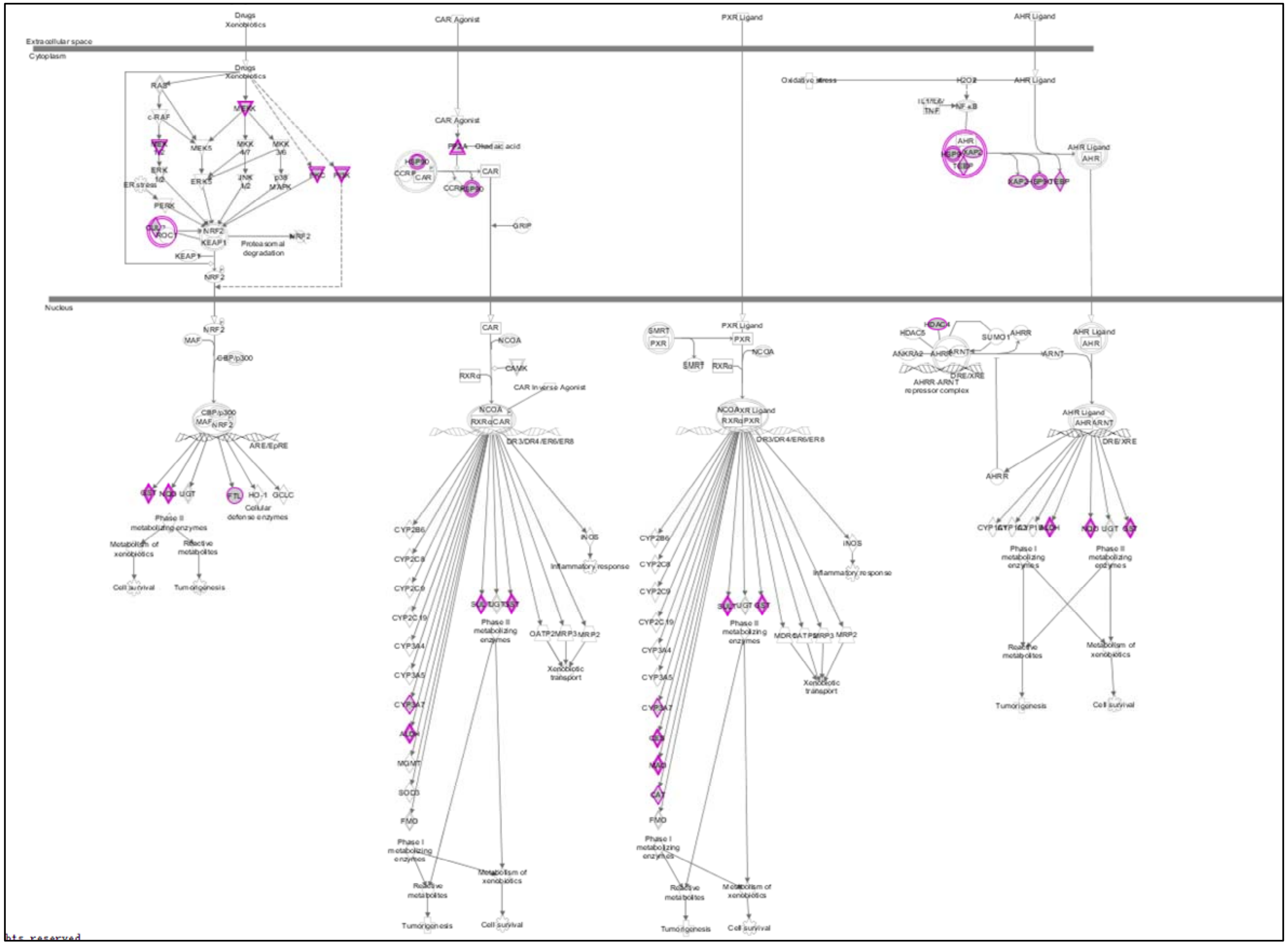

33-Virus Entry via Endocytic Pathways

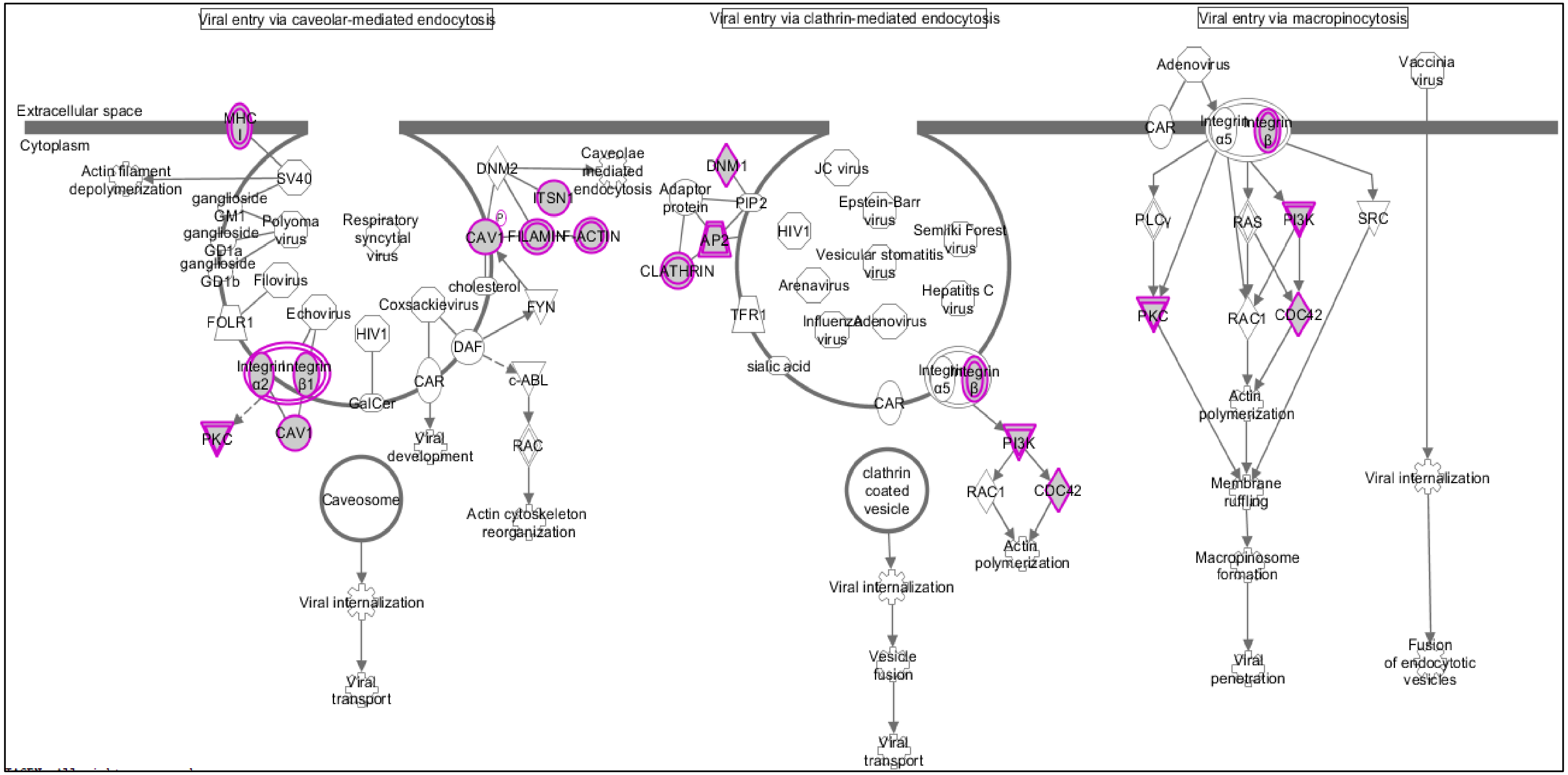

34-Valine Degradation Ⅰ

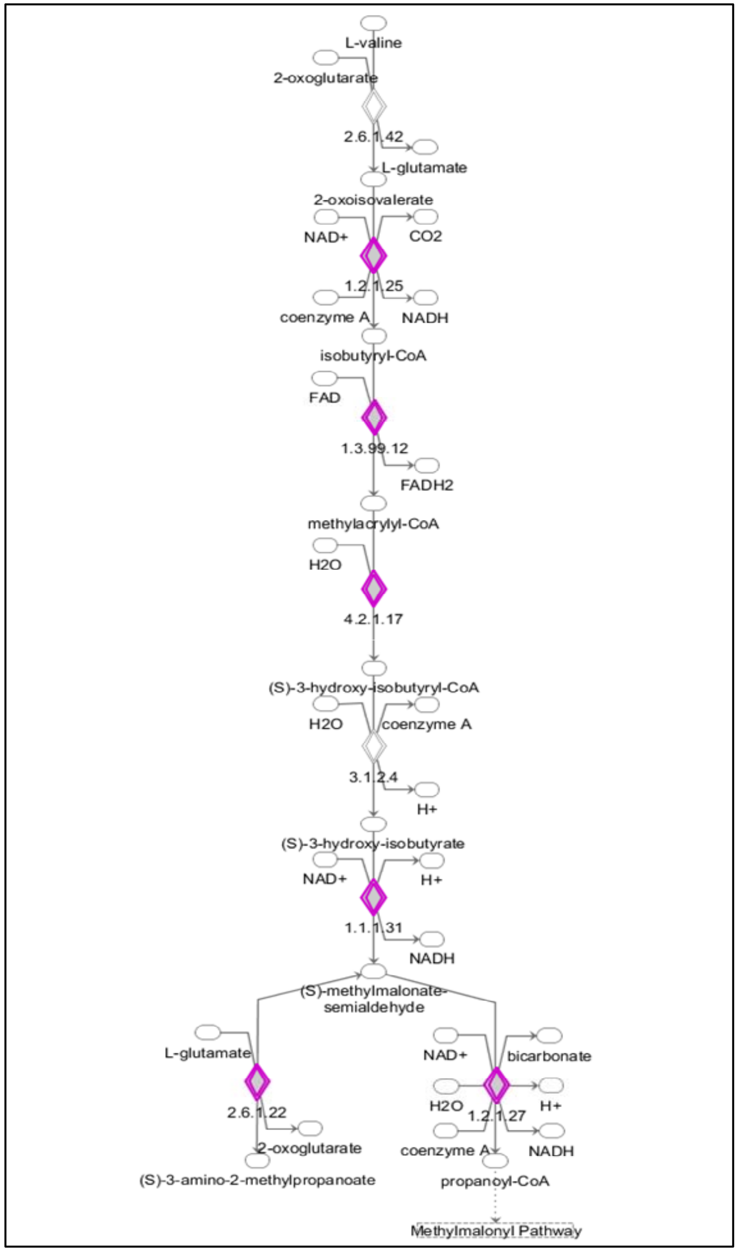

35-CDK5 Signaling

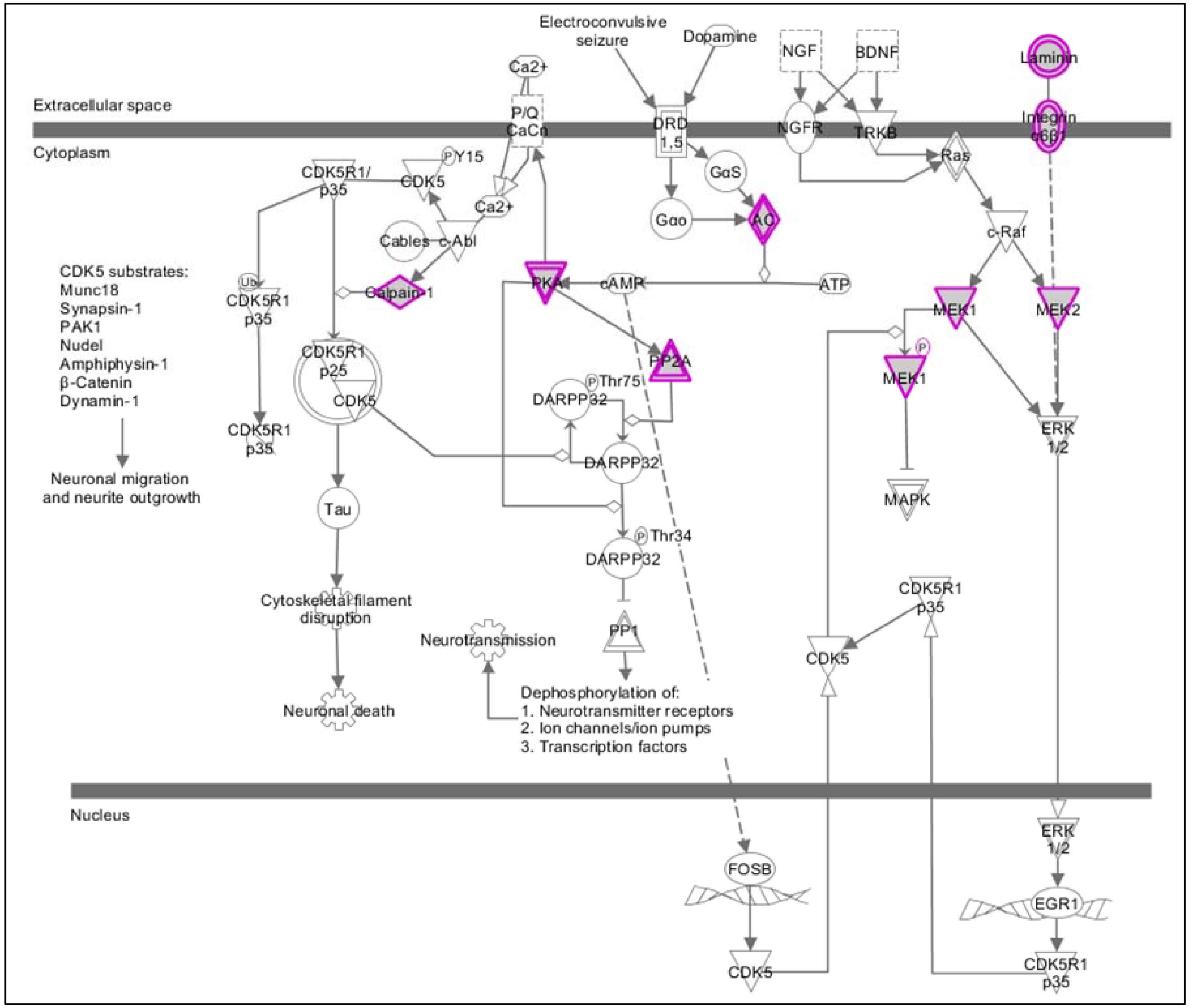

36-Axonal Guidance Signaling

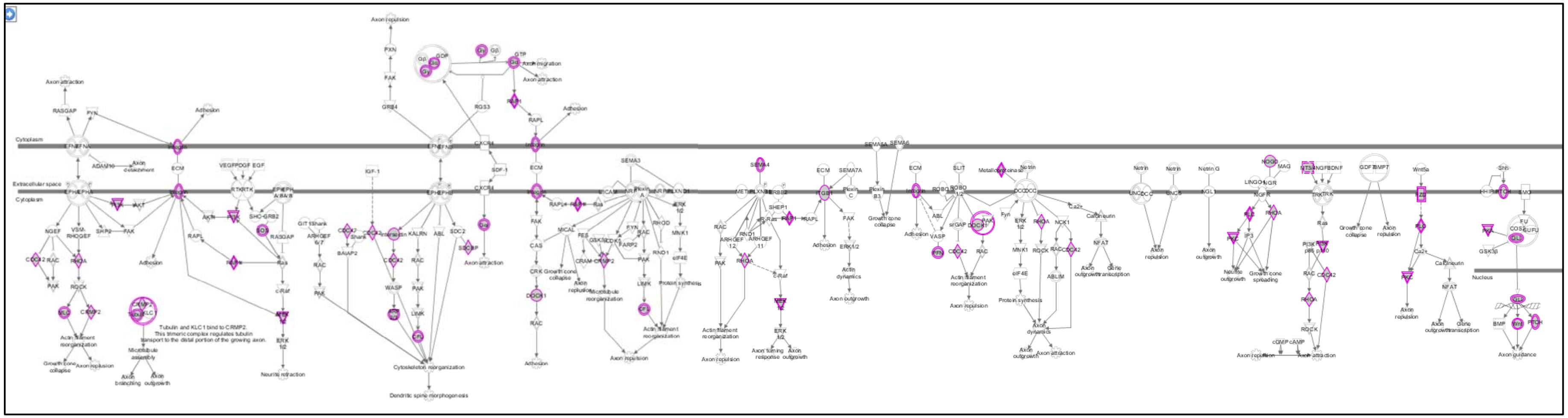

37-Noradrenaline and Adrenaline Degradation

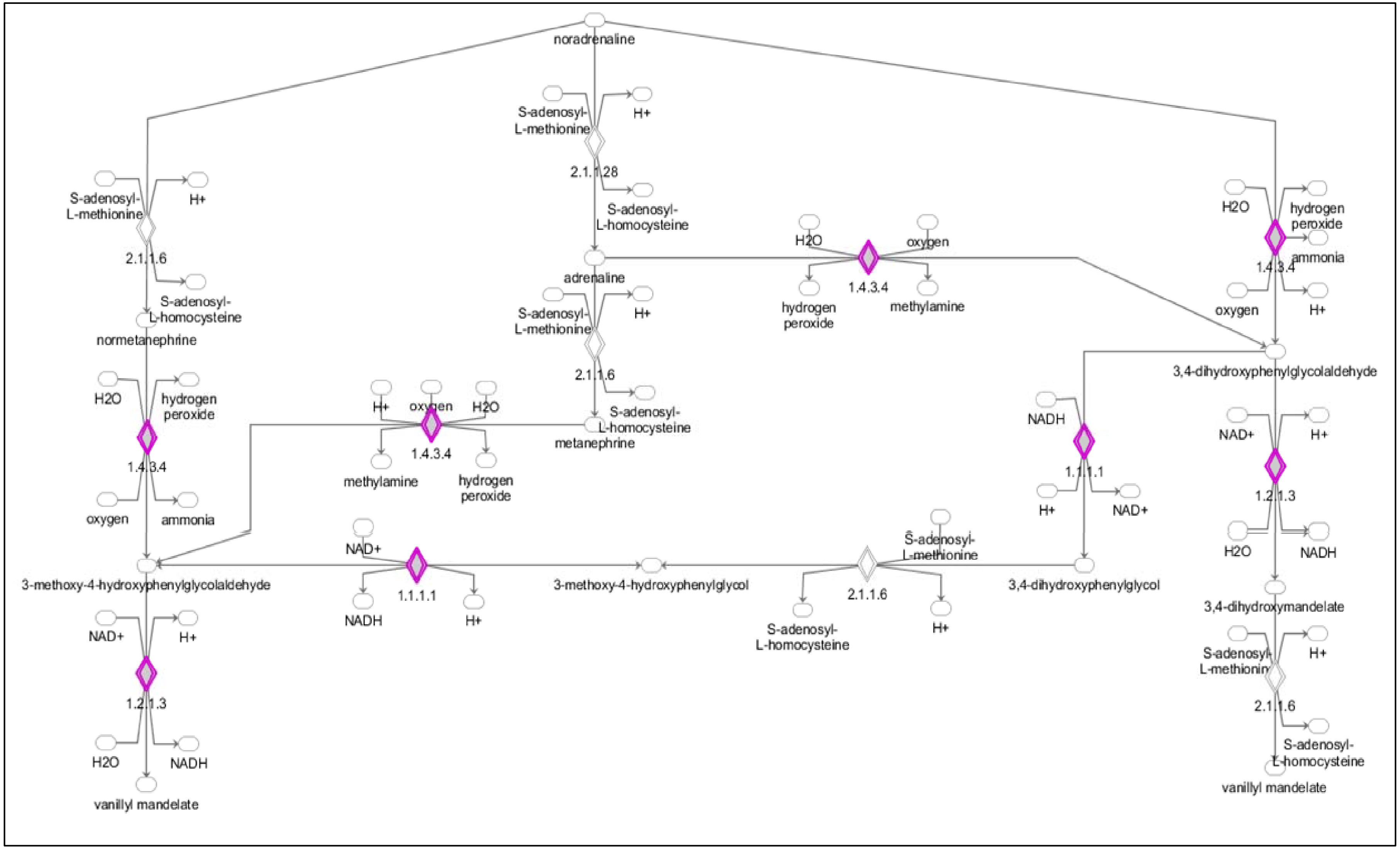

38-Ovarian Cancer Signaling

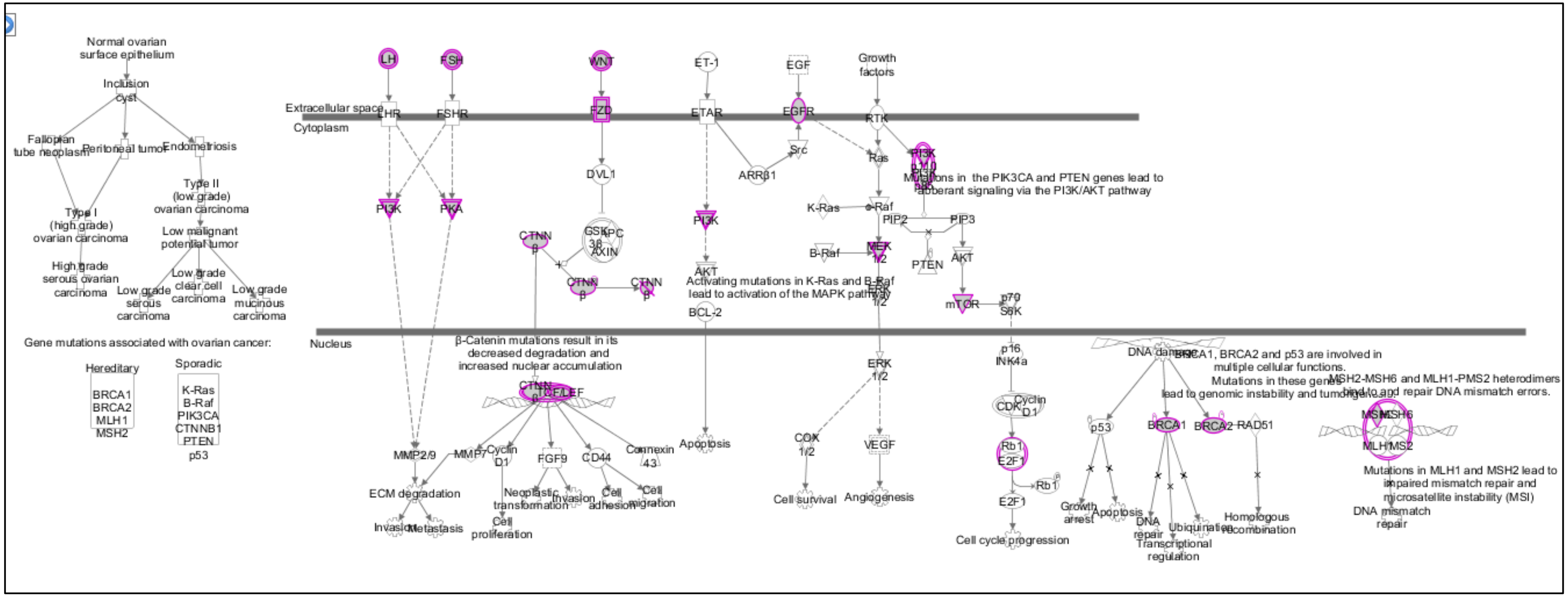

39-Germ Cell-Sertoli Cell Junction Signaling

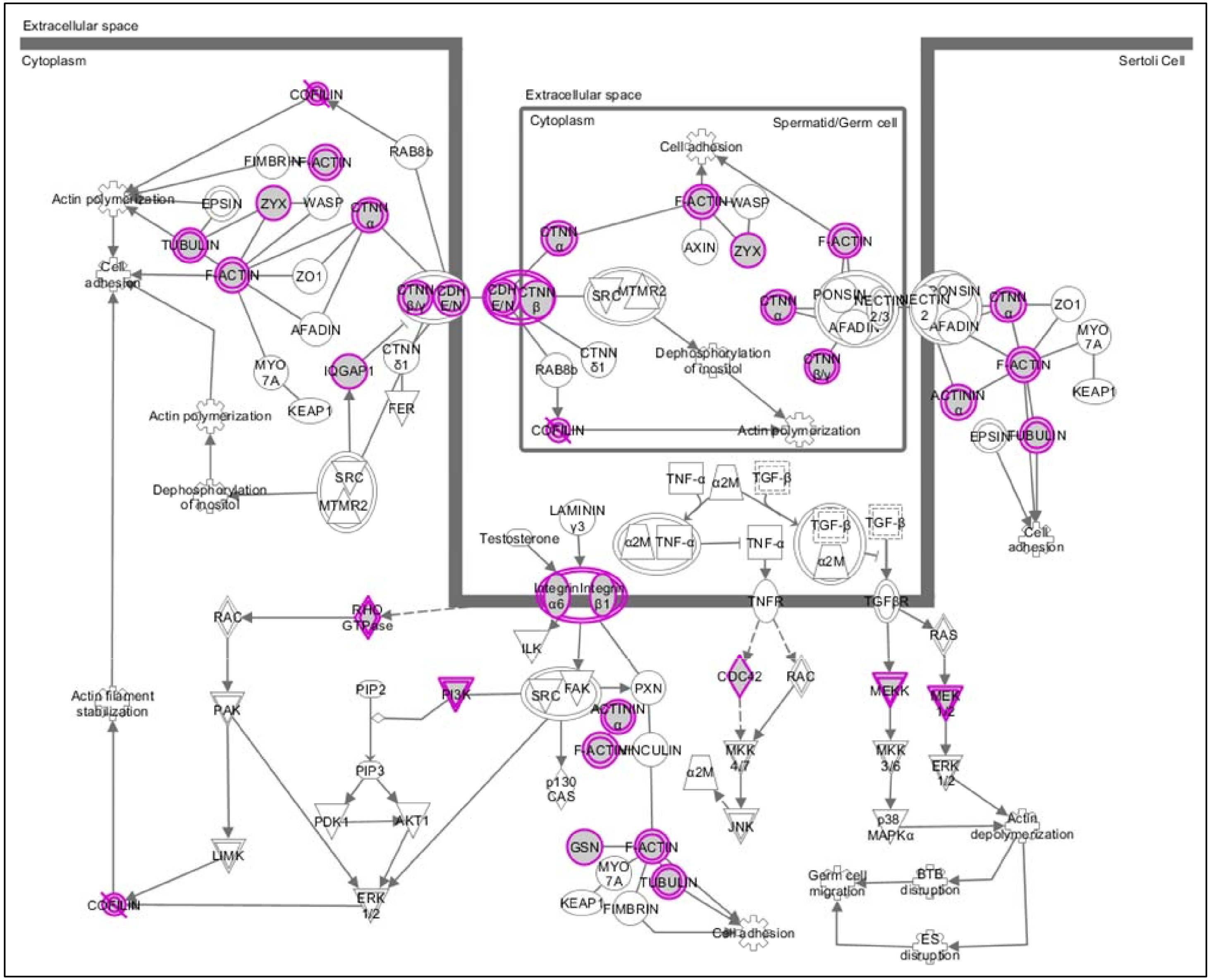

40-Protein Kinase A Signaling

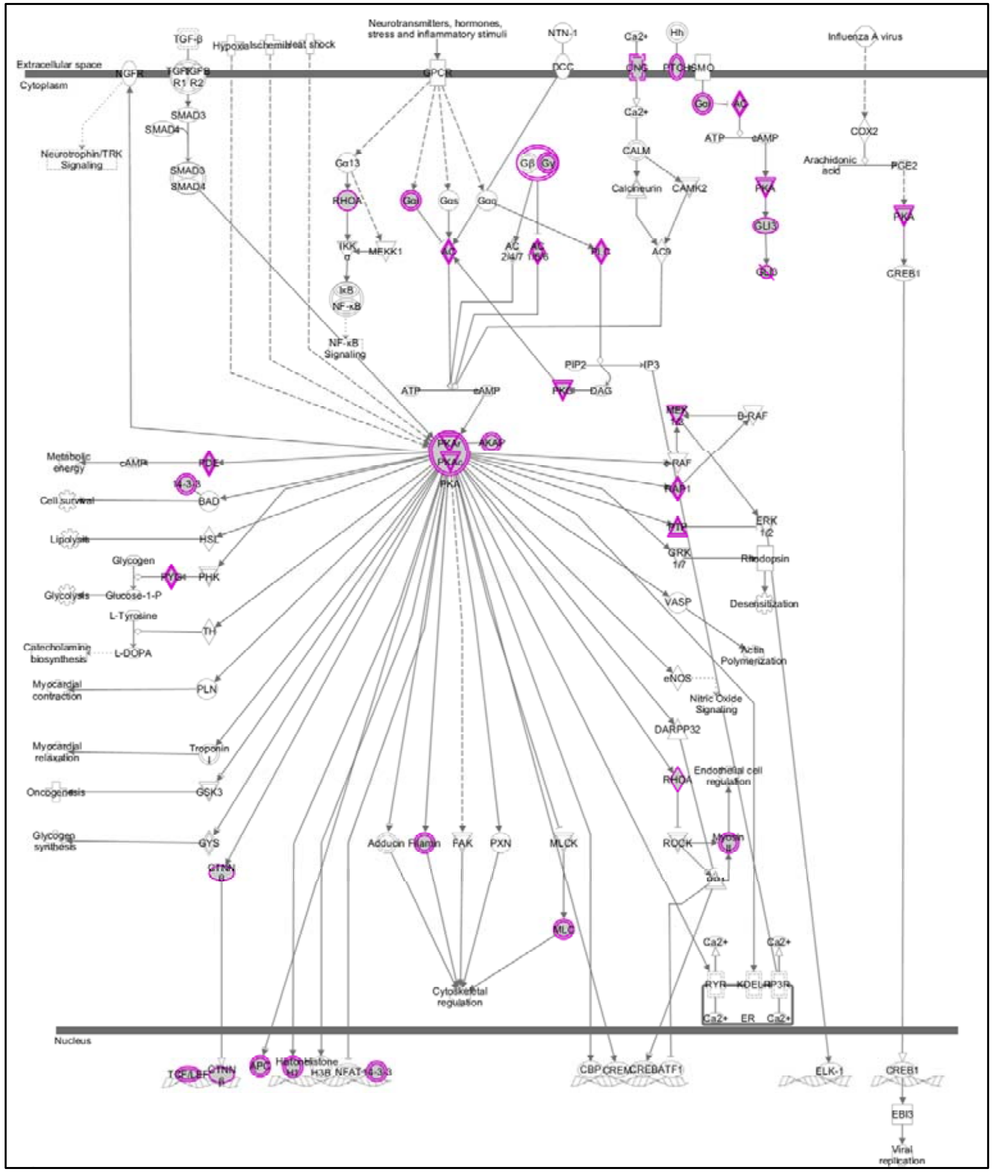

41-TR-RXR Actiation

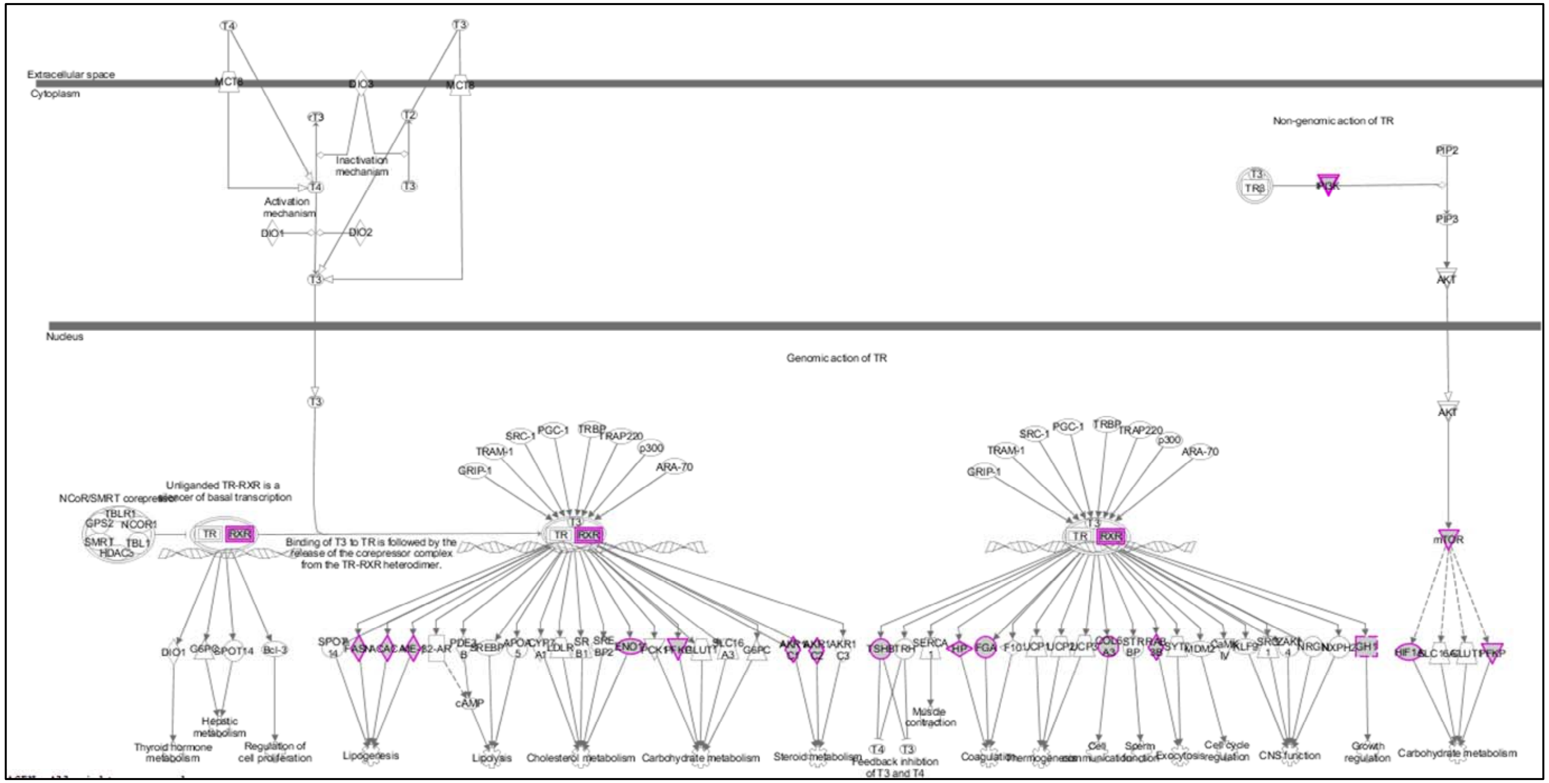

42-Endoplasmic Reticulum Stress Pathway

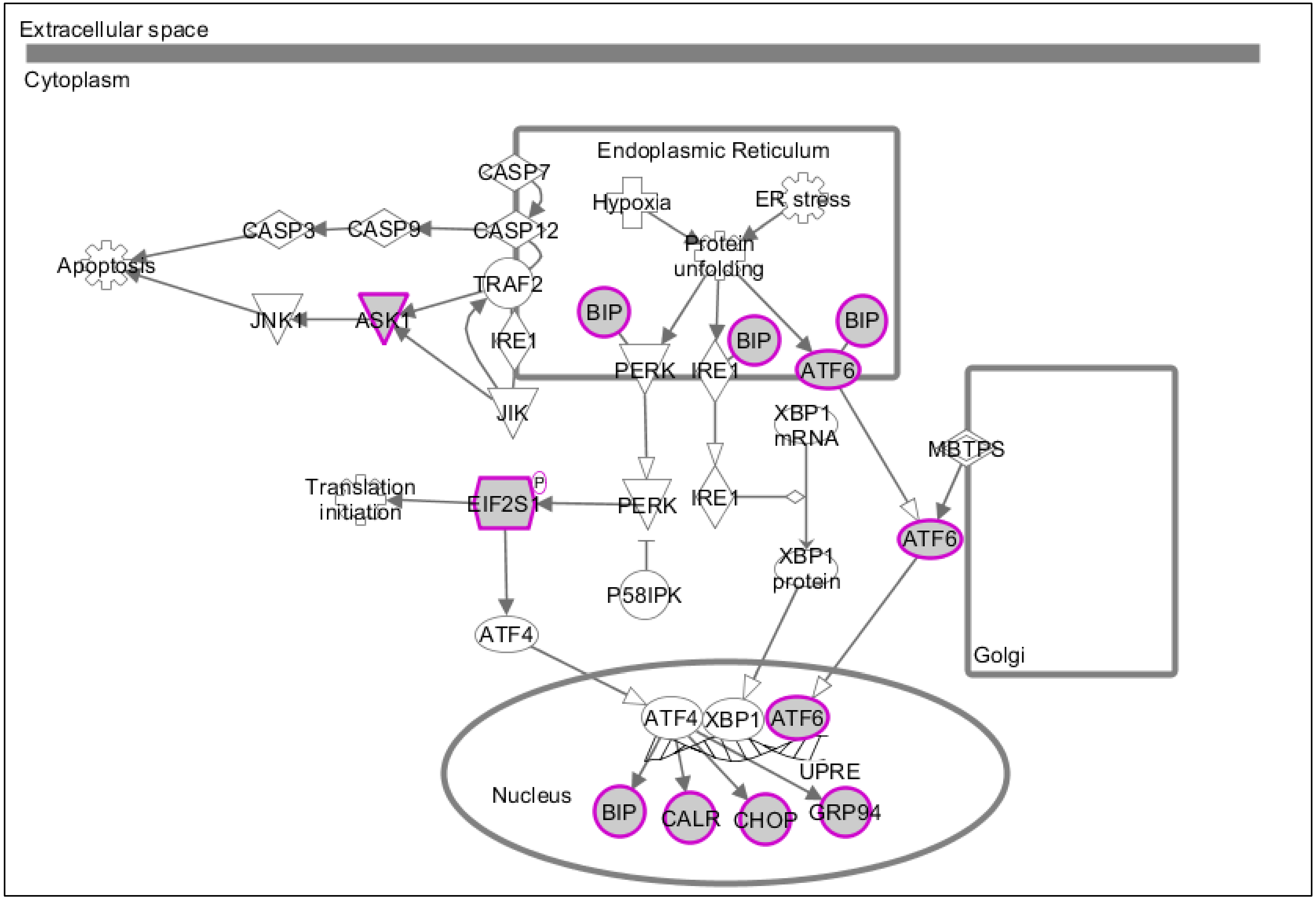

43-Ephrin B Signaling

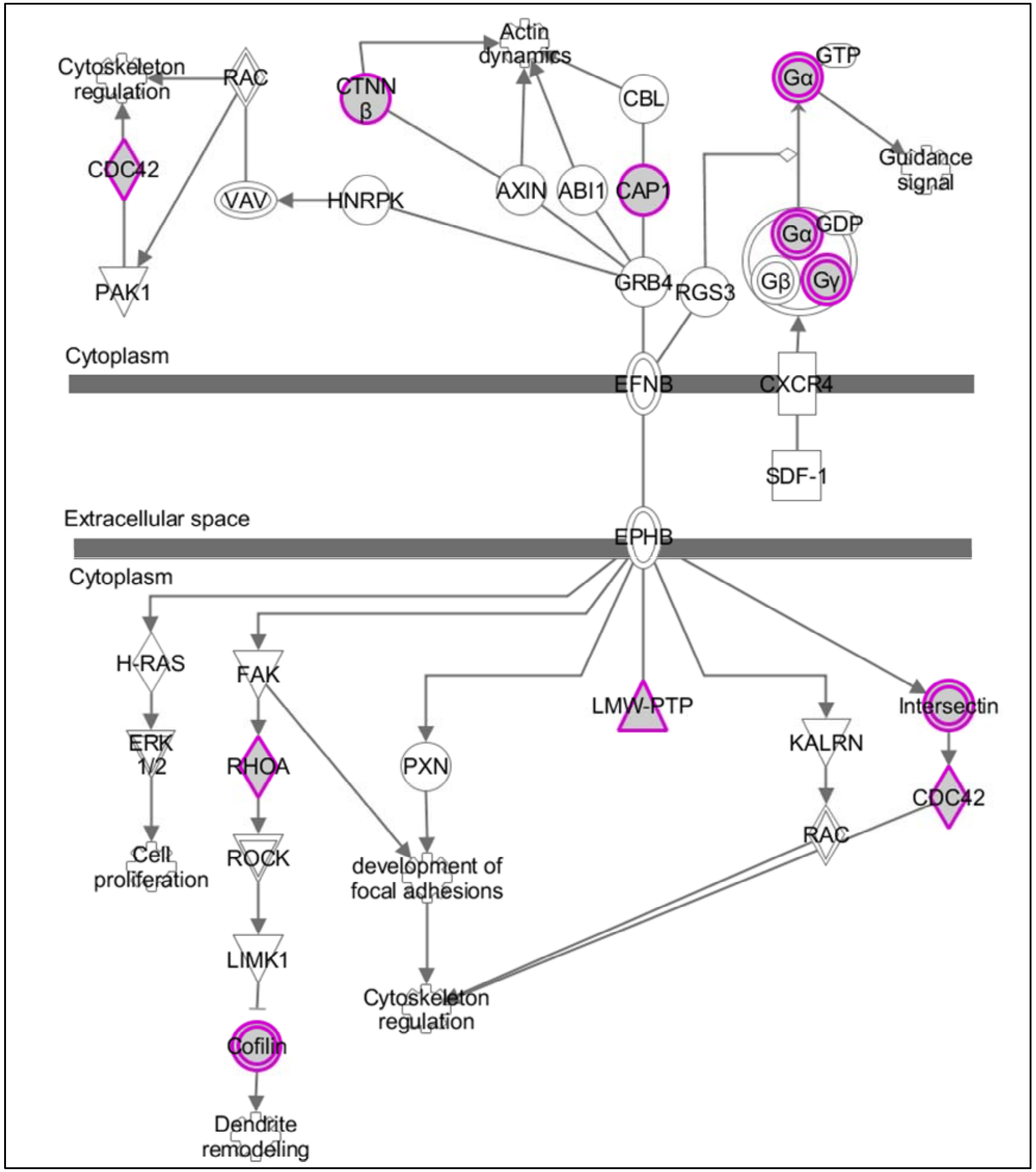

44-Tryptophan Degradation X(Mammalian, via Tryptamine)

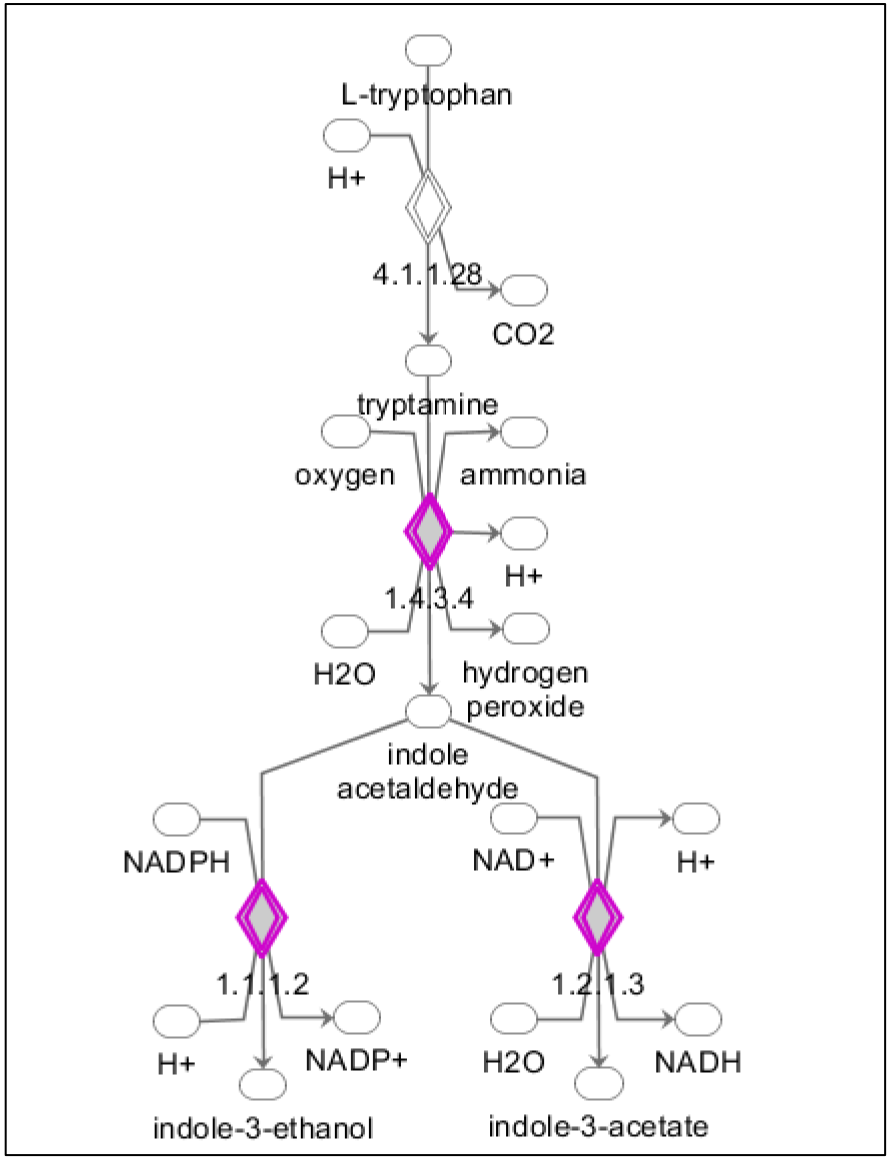

45-Atherosclerosis Signaling

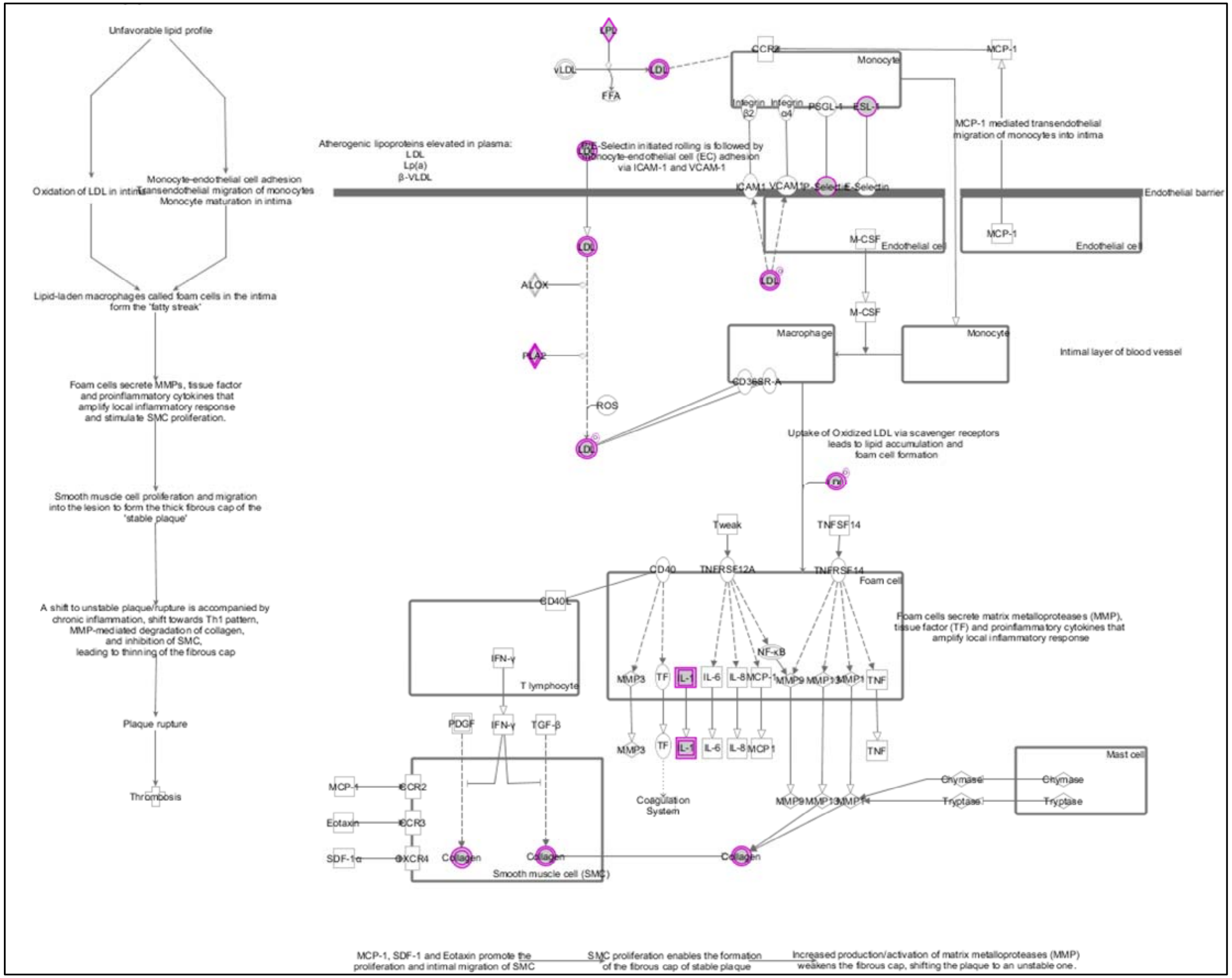

46-Glutaryl-CoA Degradation

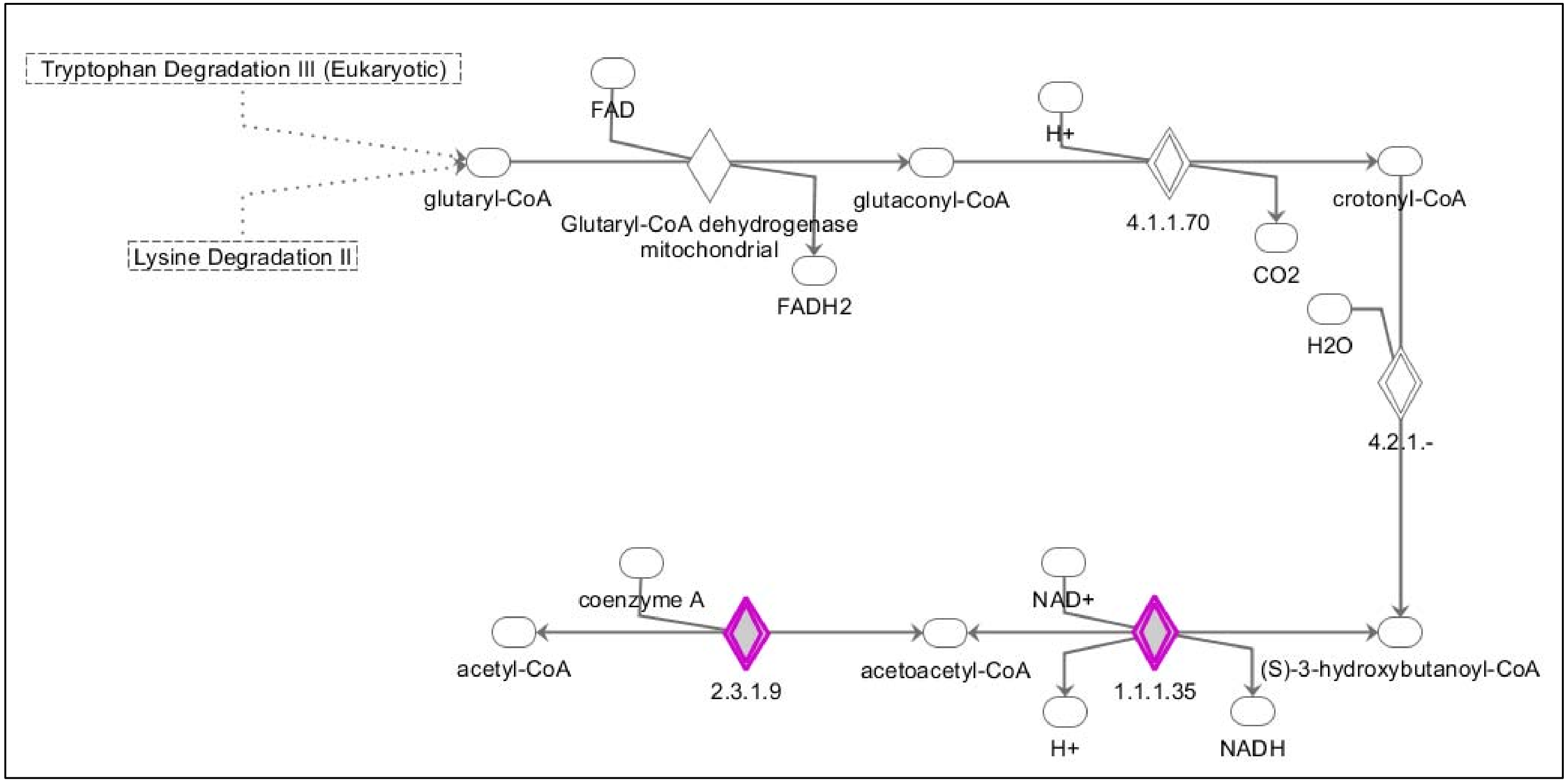

47-GNRH Signaling

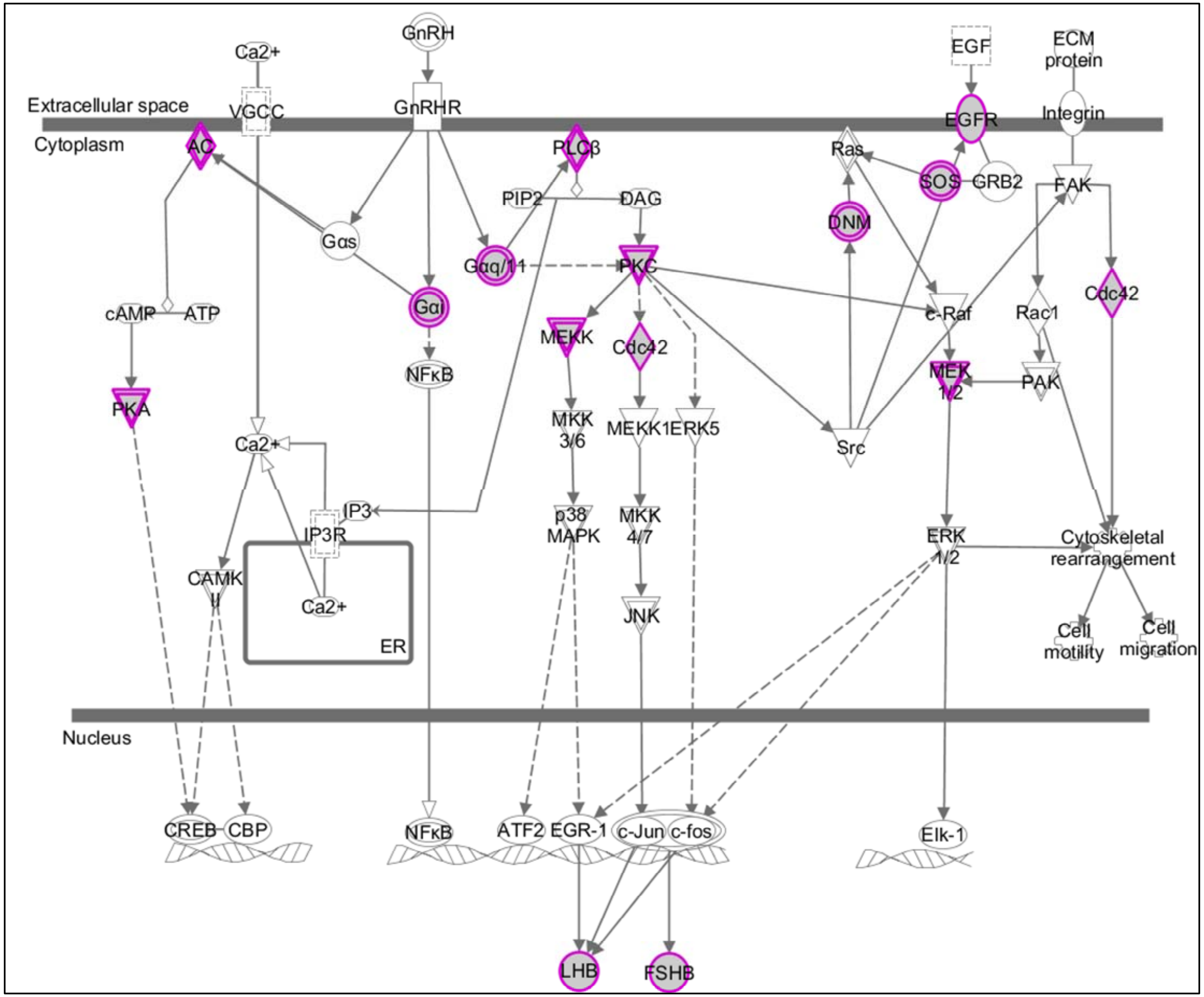

48-Colanic Acid Building Blocks Bisynthesis

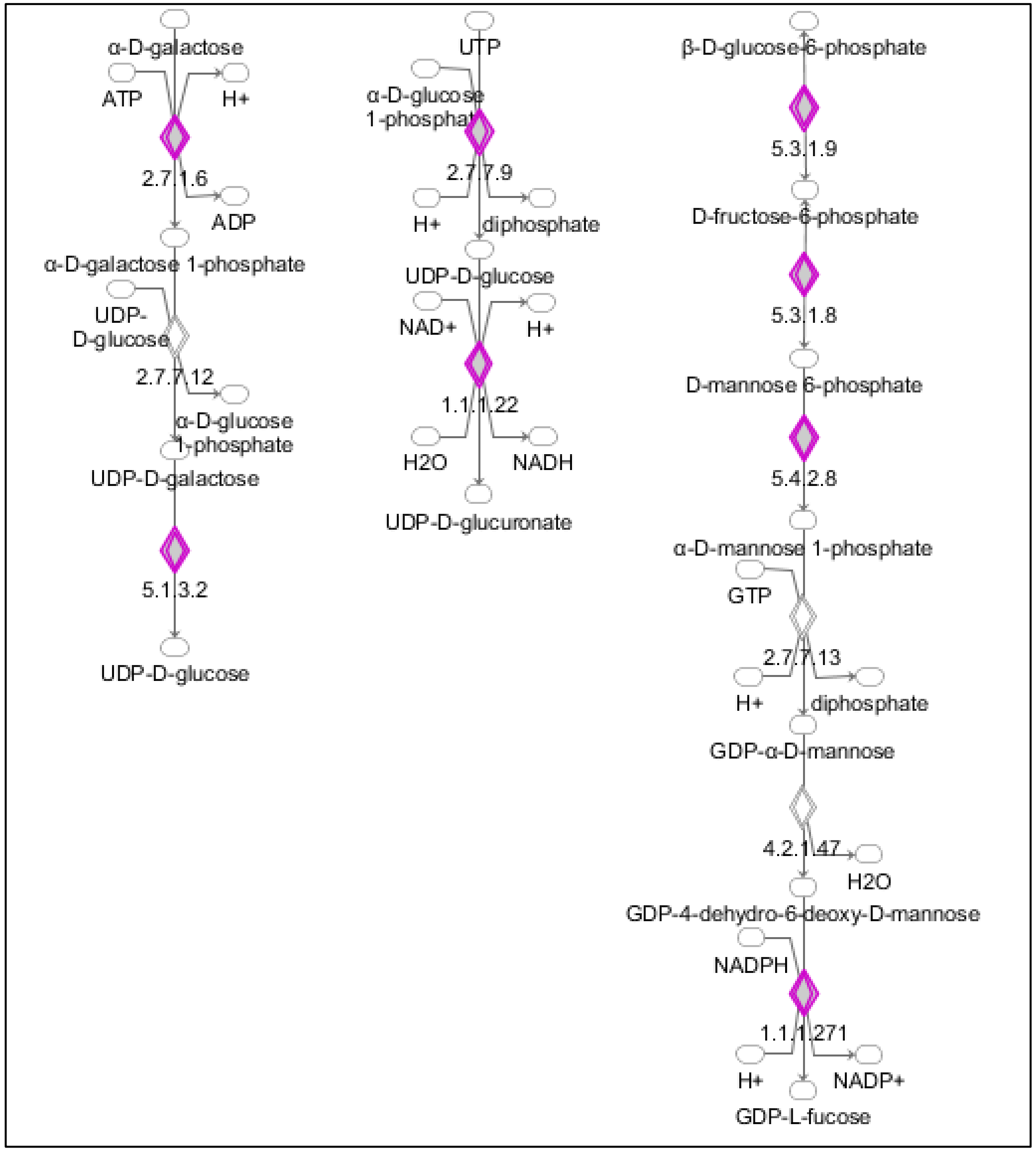

49-Ephrin Receptor Signaling

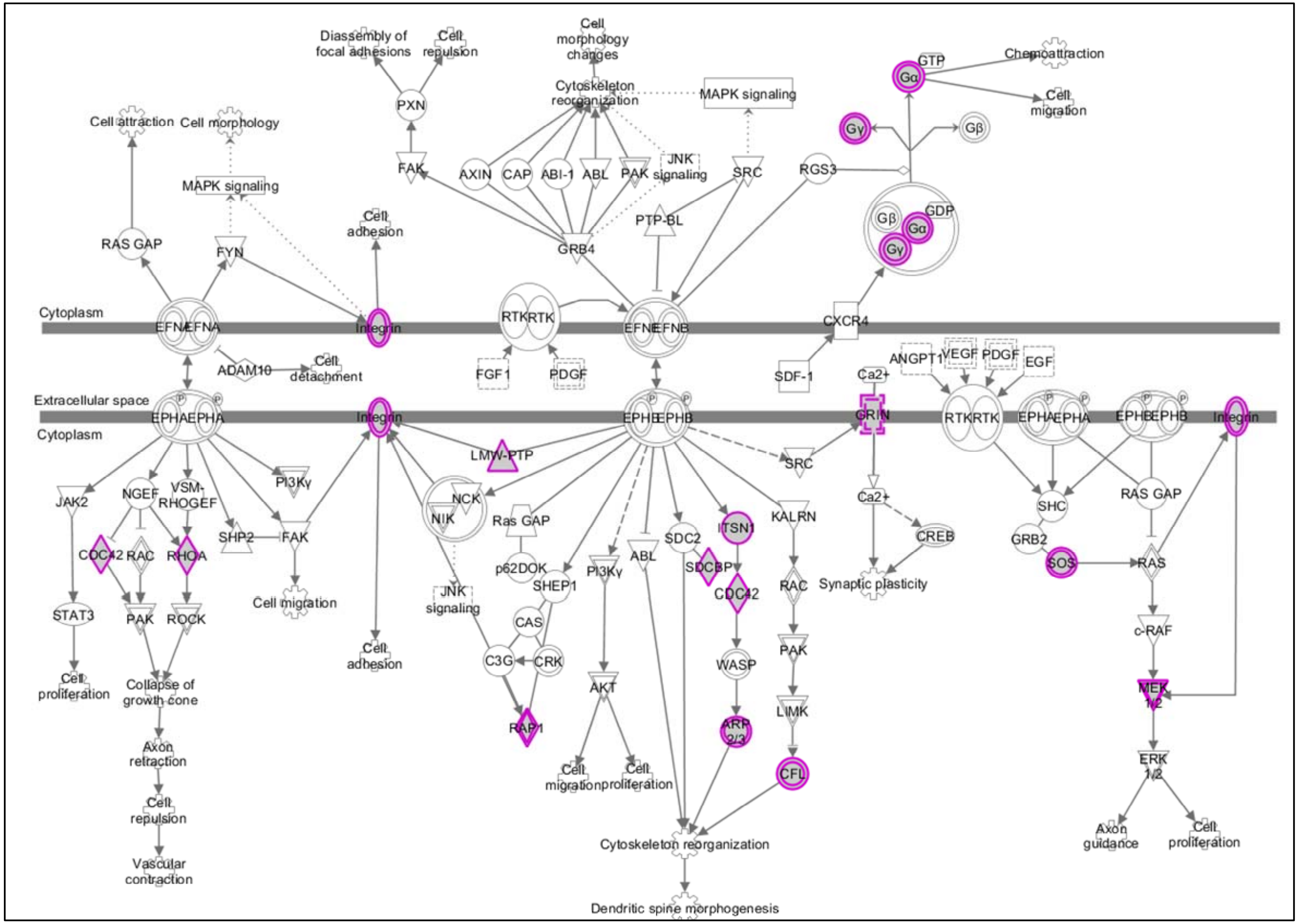

50-Isoleucine Degradation Ⅰ

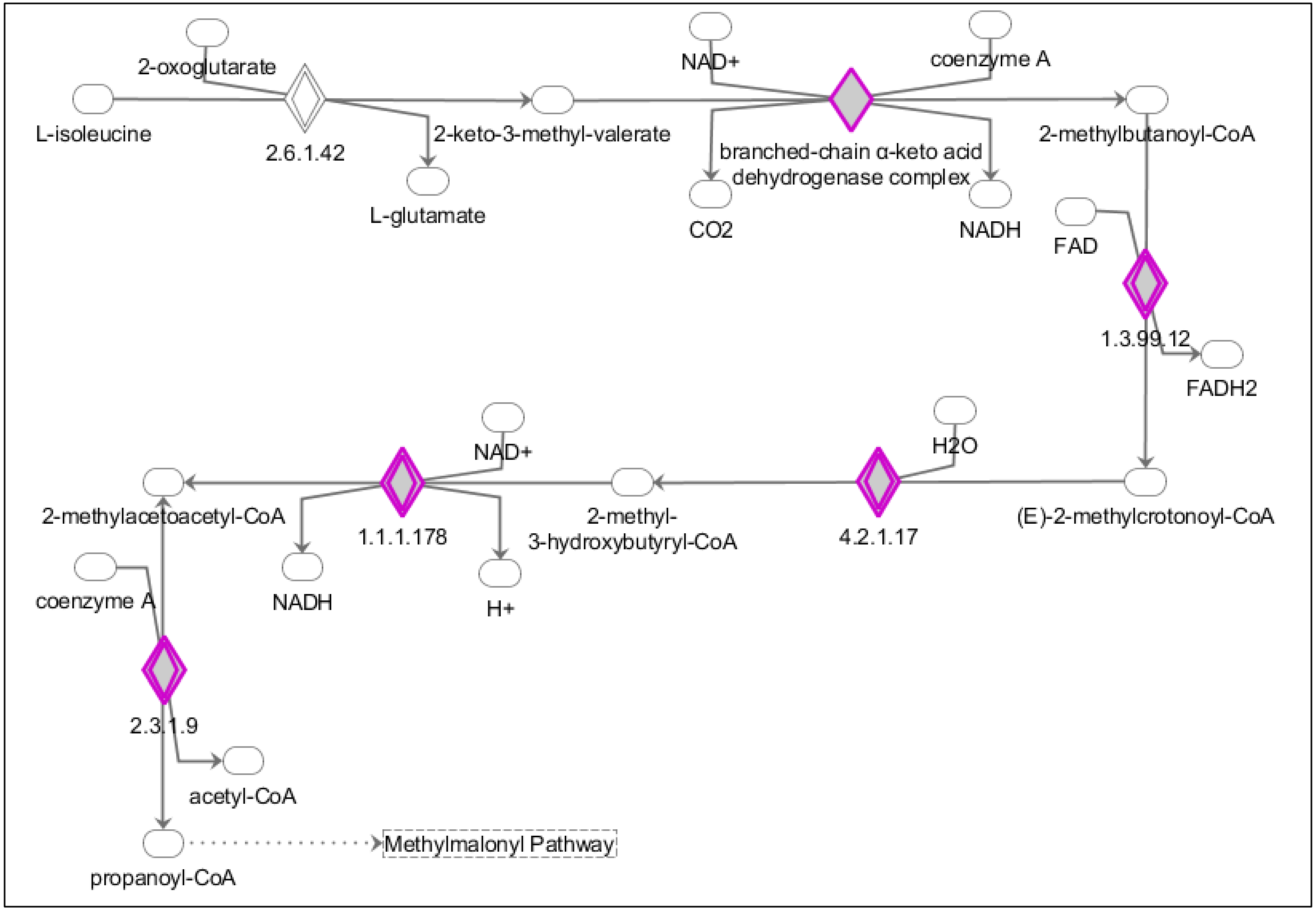

51-Ethanol Degradation Ⅳ

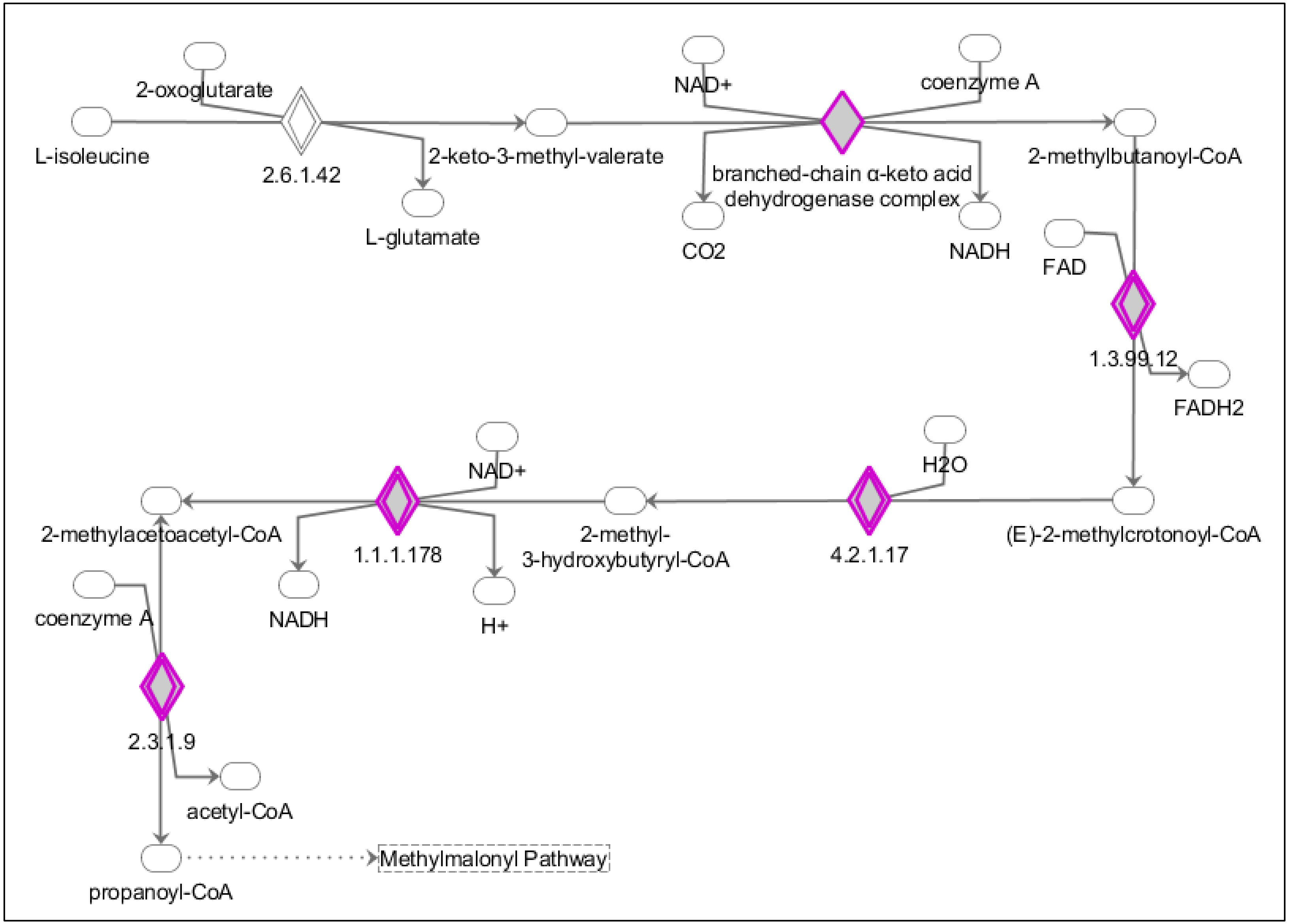

52-VEGF Signaling

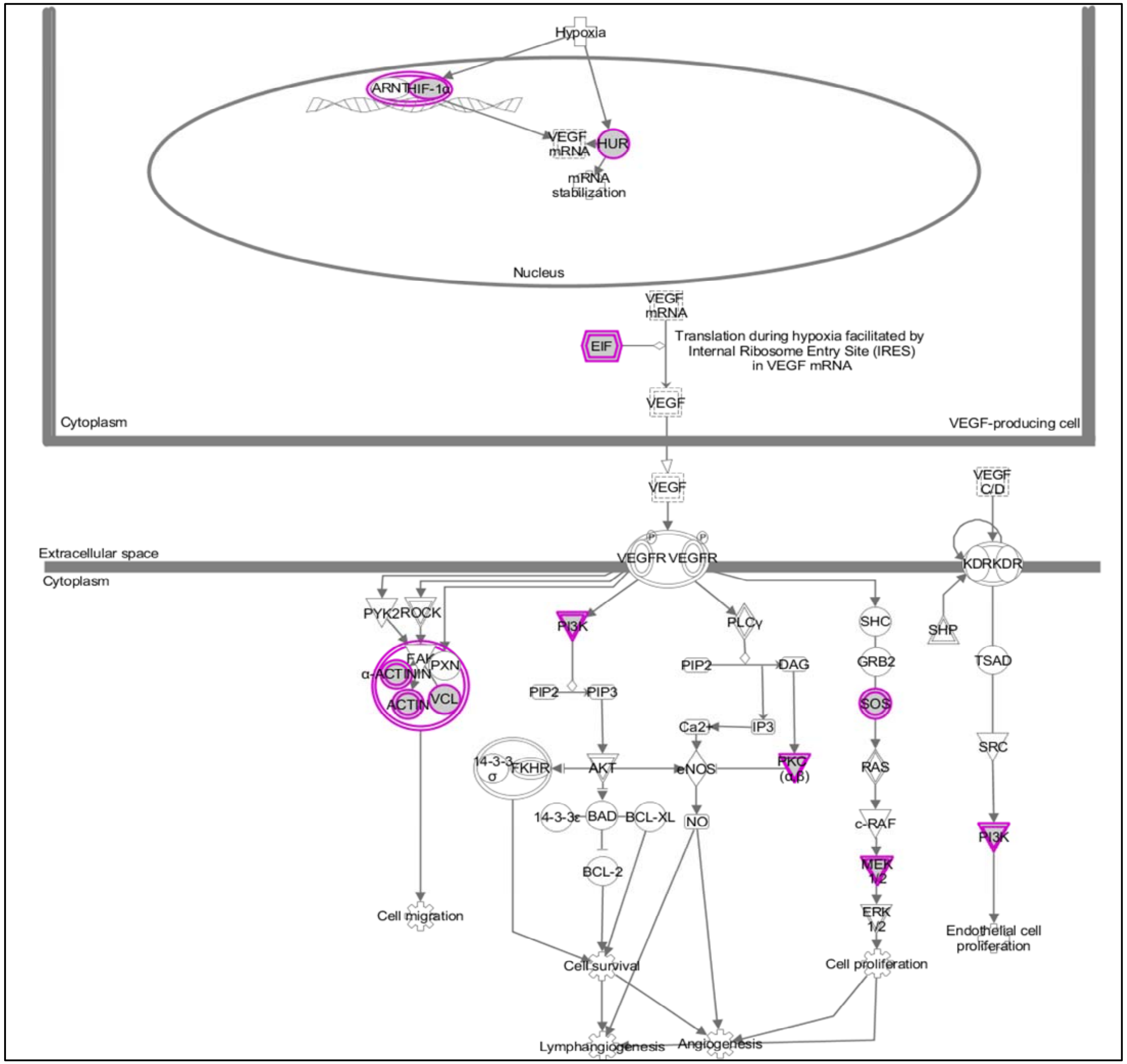

53-CREB Signaling in Neurons

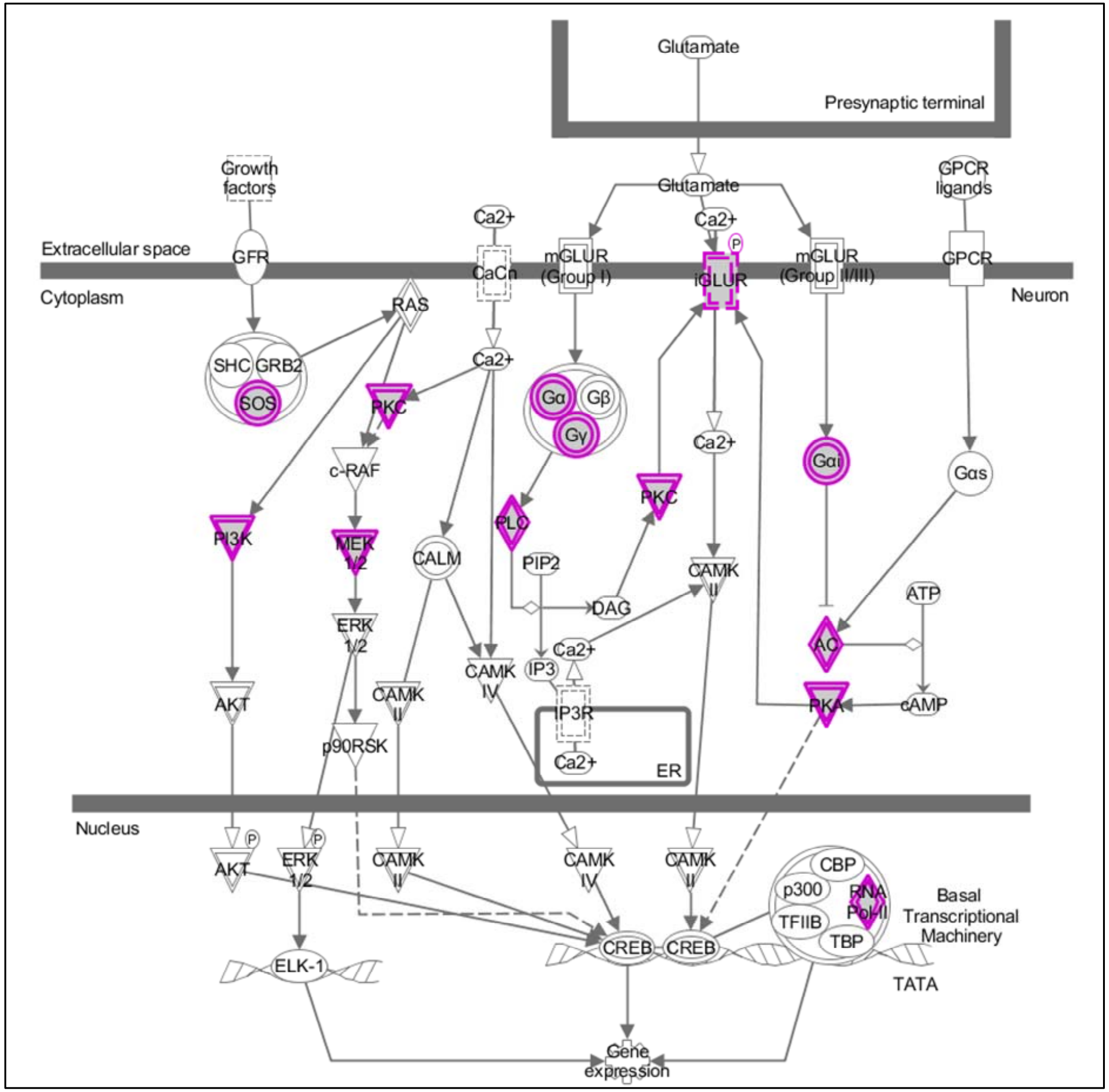

54-G Beta Gamma Signaling

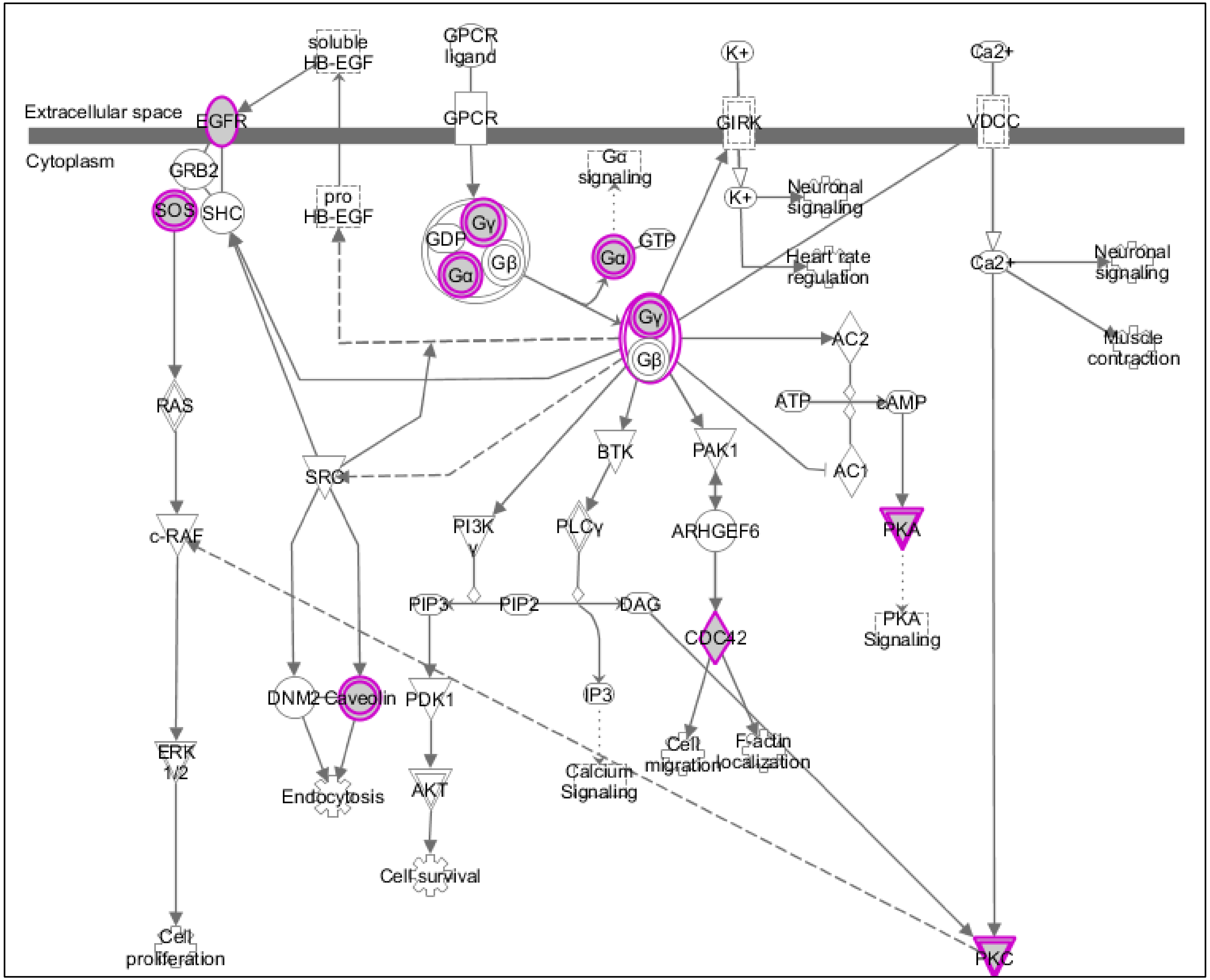

55-PI3K-AKT Signaling

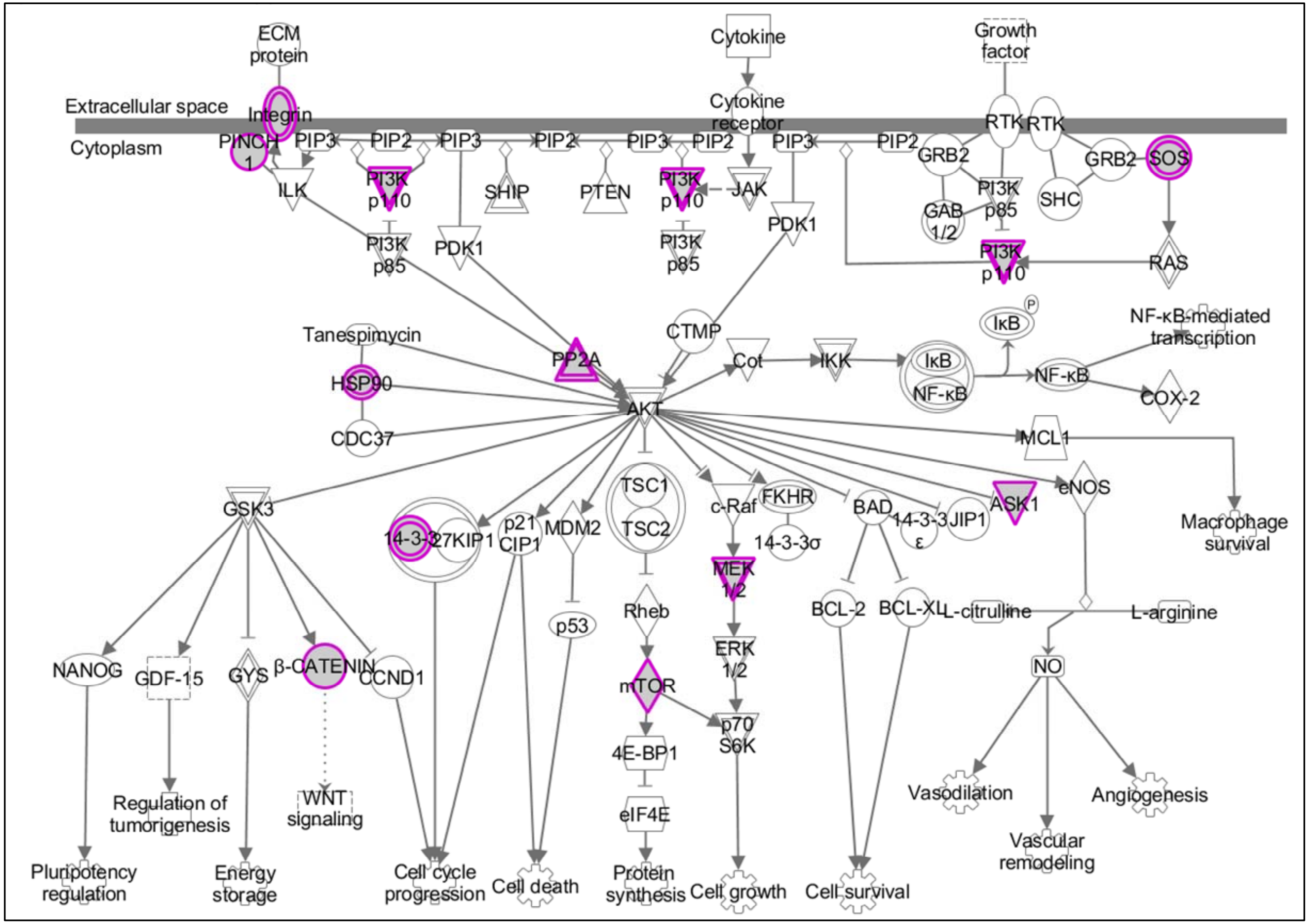

56-Telomerase Signaling

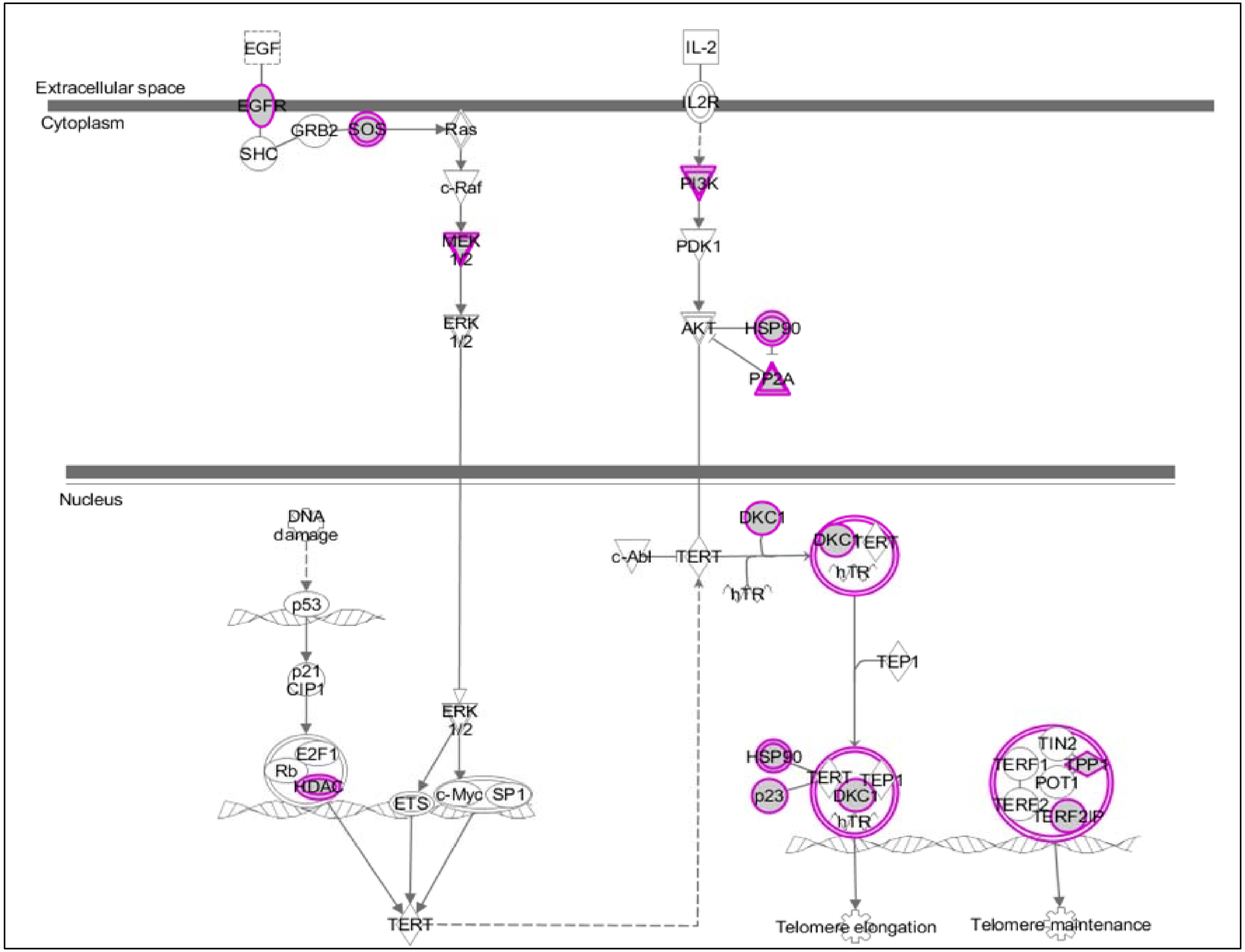

57-PPARα-RXRα Activation

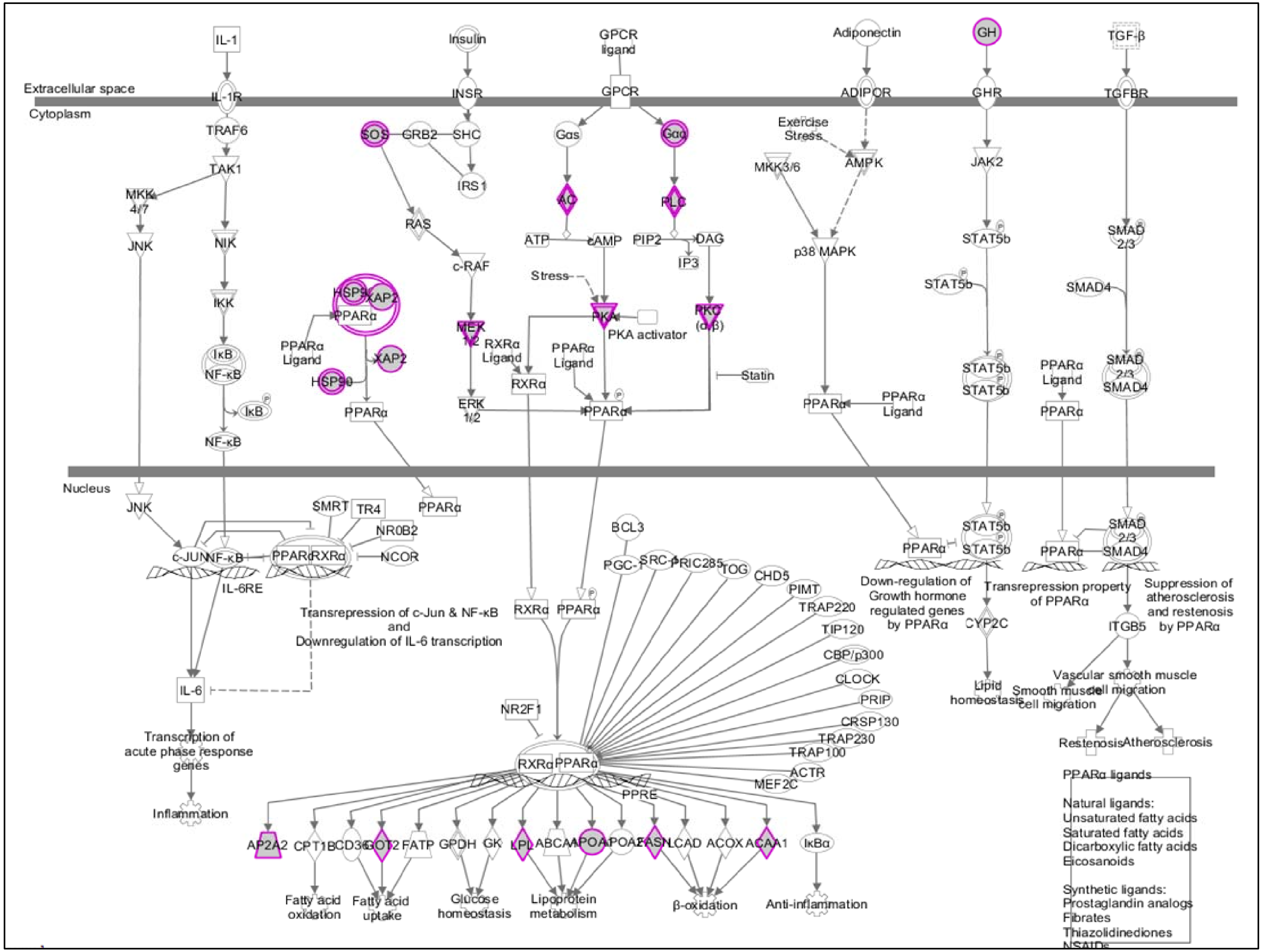

58-Sertoli Cell-Sertoli Cell Junction Signaling

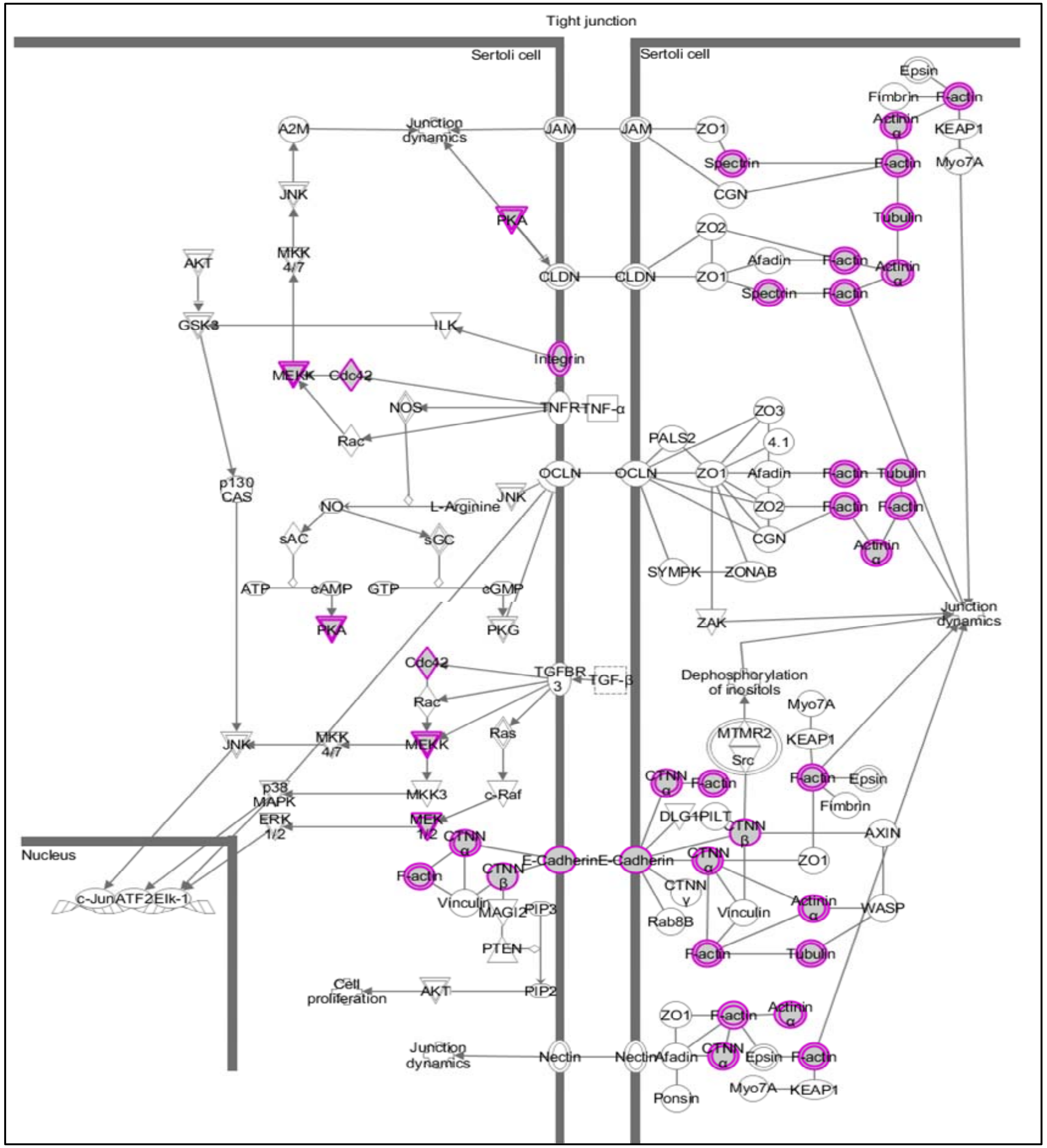

59-Cardiac Hypertrophy Signaling

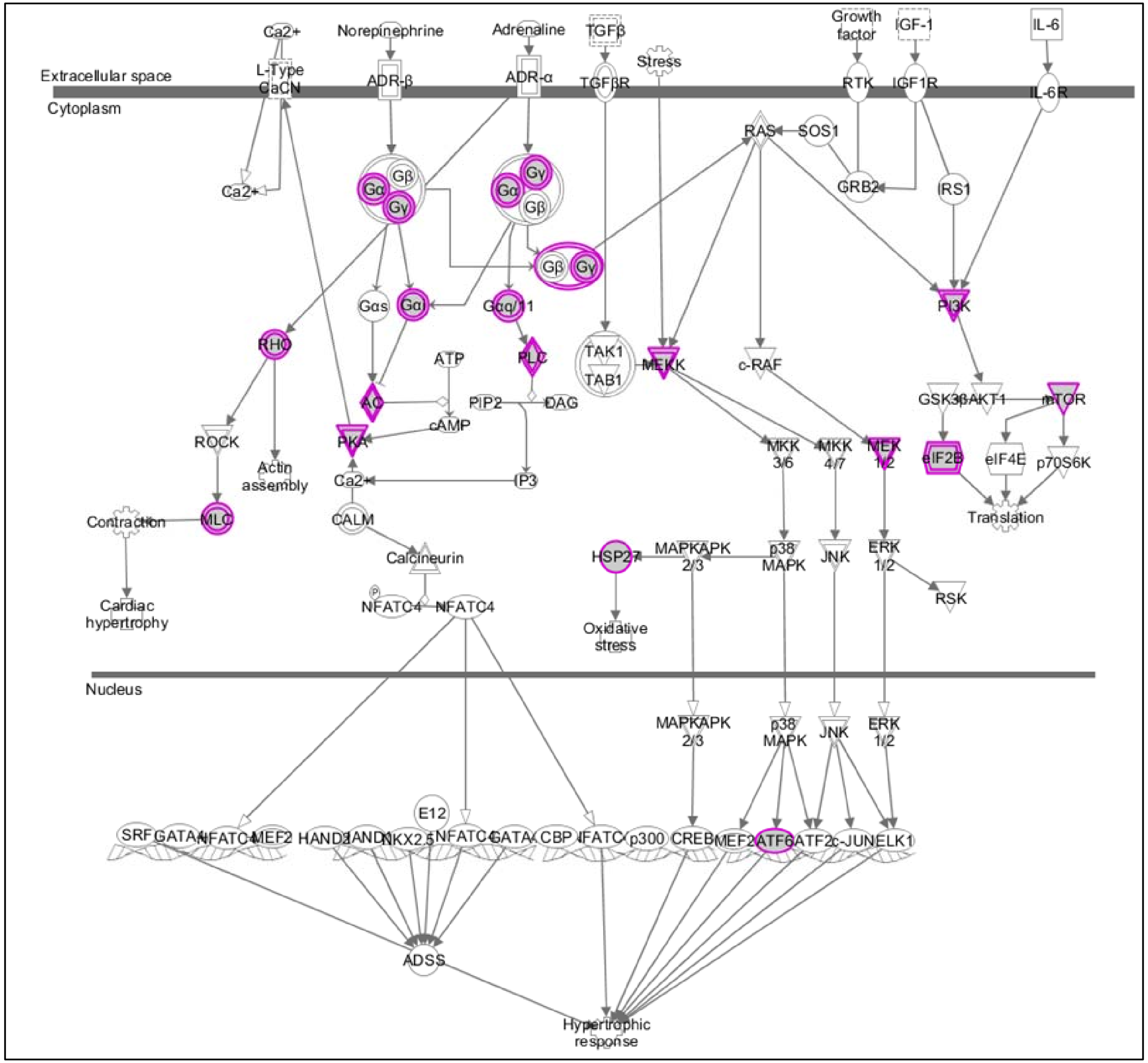

60-IL-1 Signaling

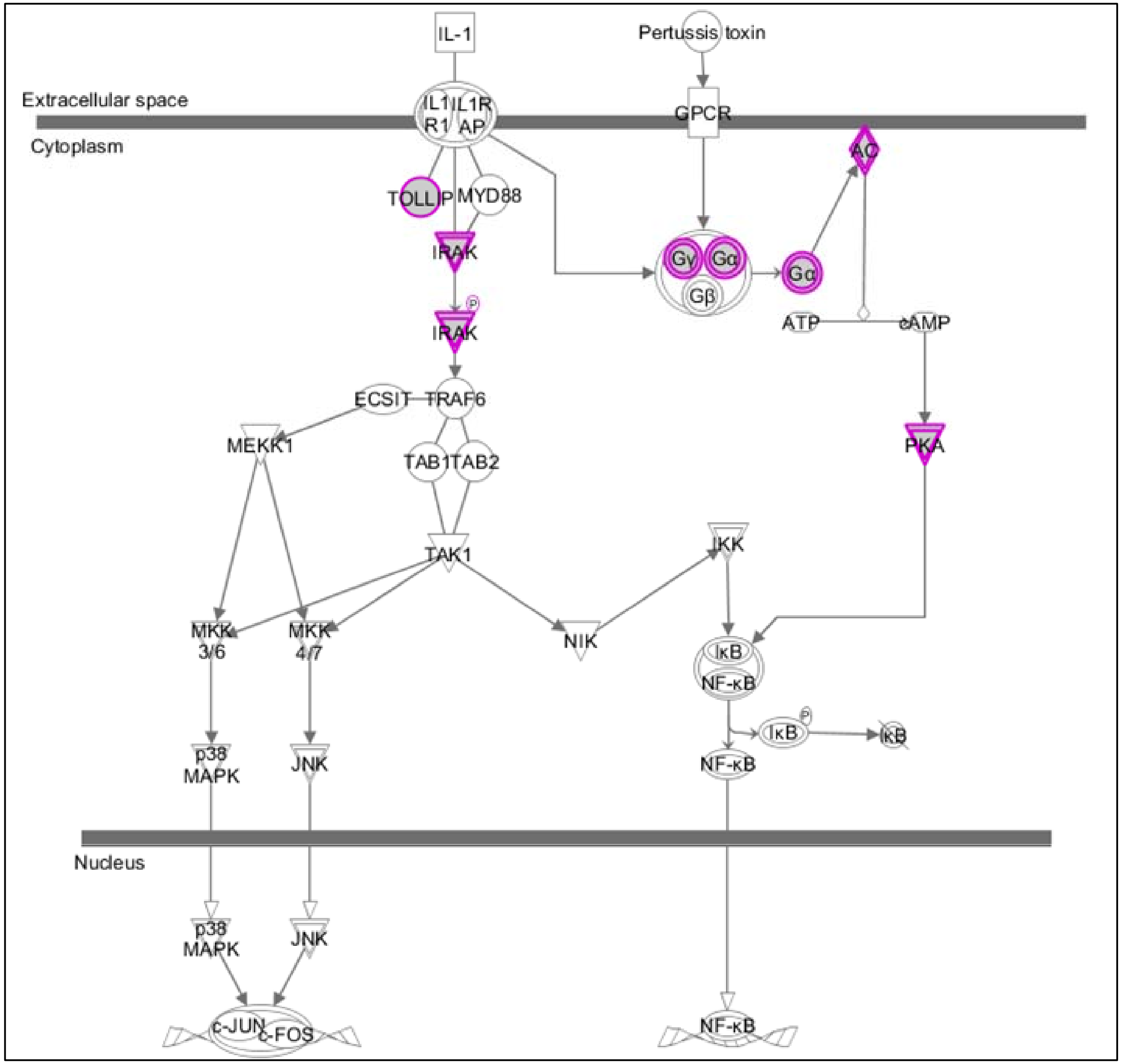

61-Androgen Signaling

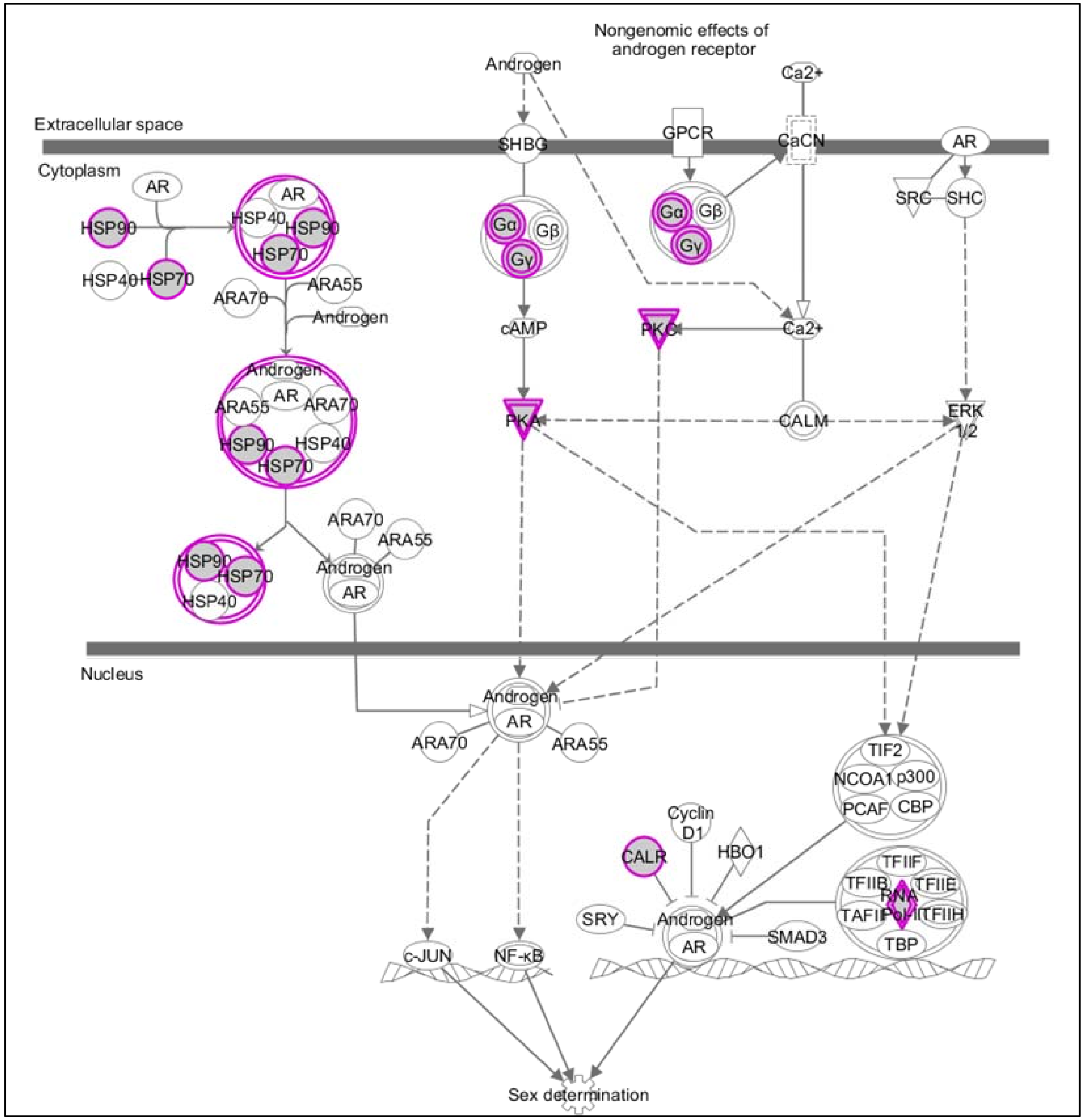

62-Tight Junction Signaling

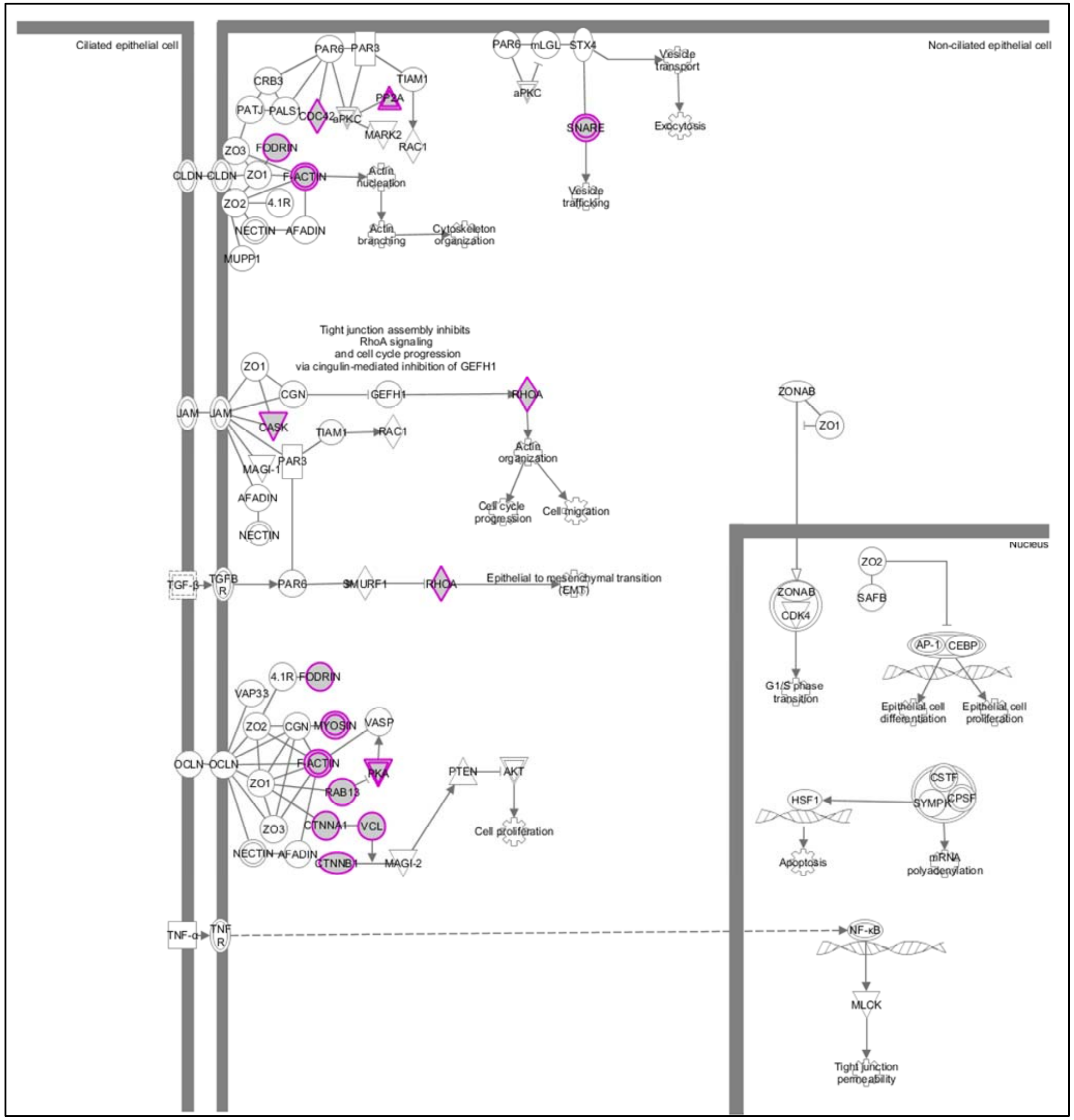

63-Fatty Acid α -oxidation

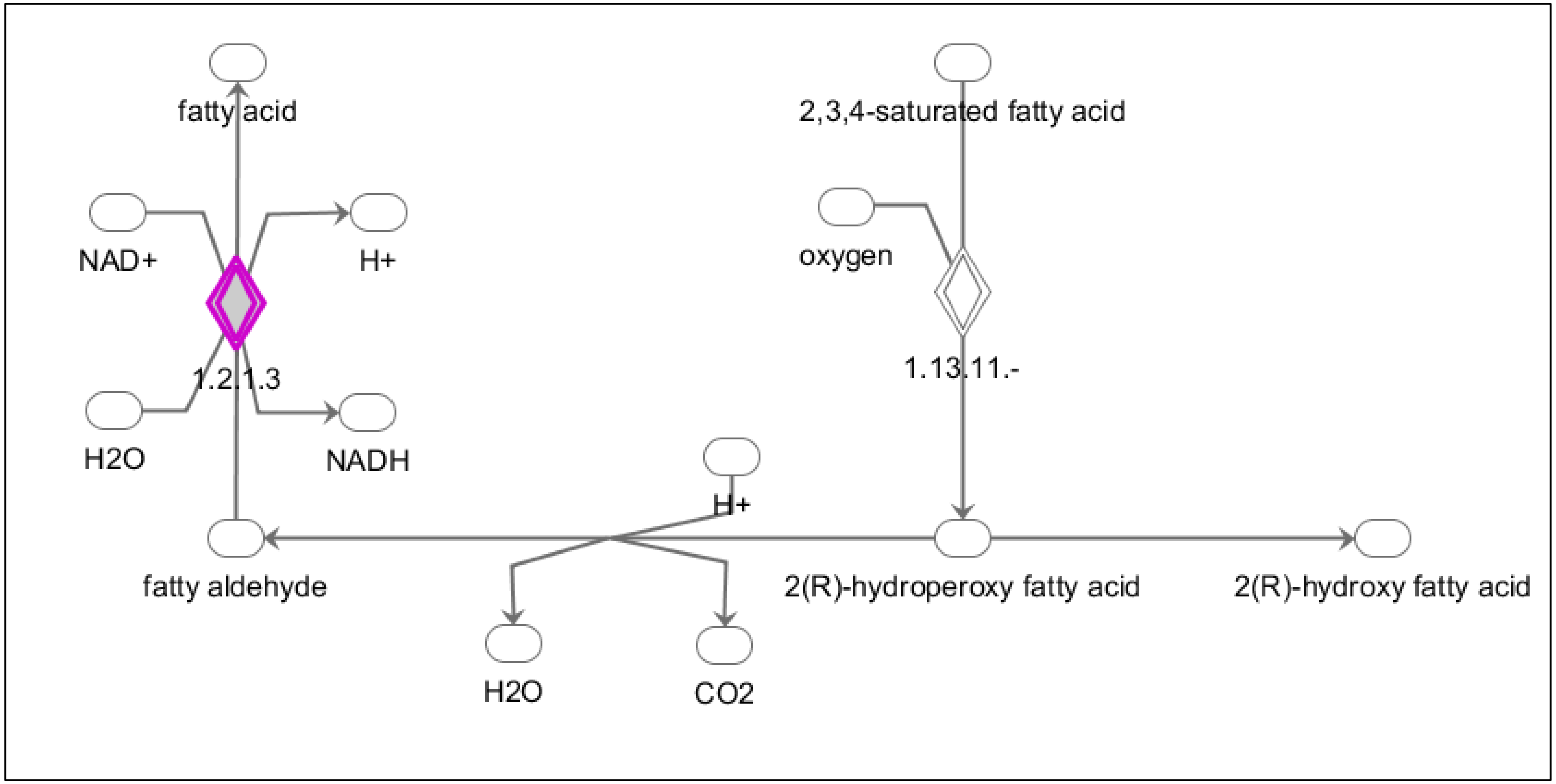

64-p70S6K Signaling

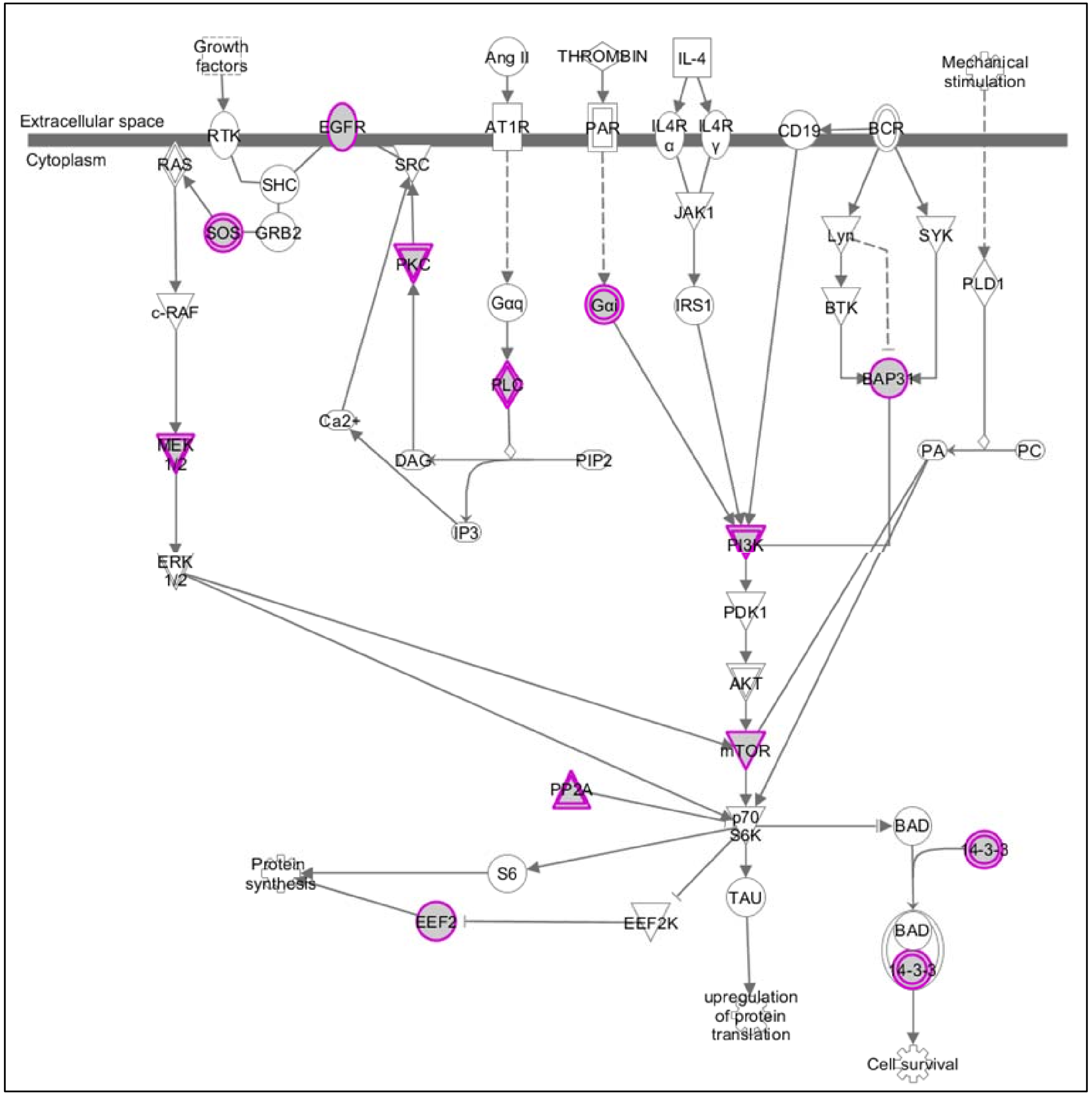

65-Thyroid Cancer Signaling

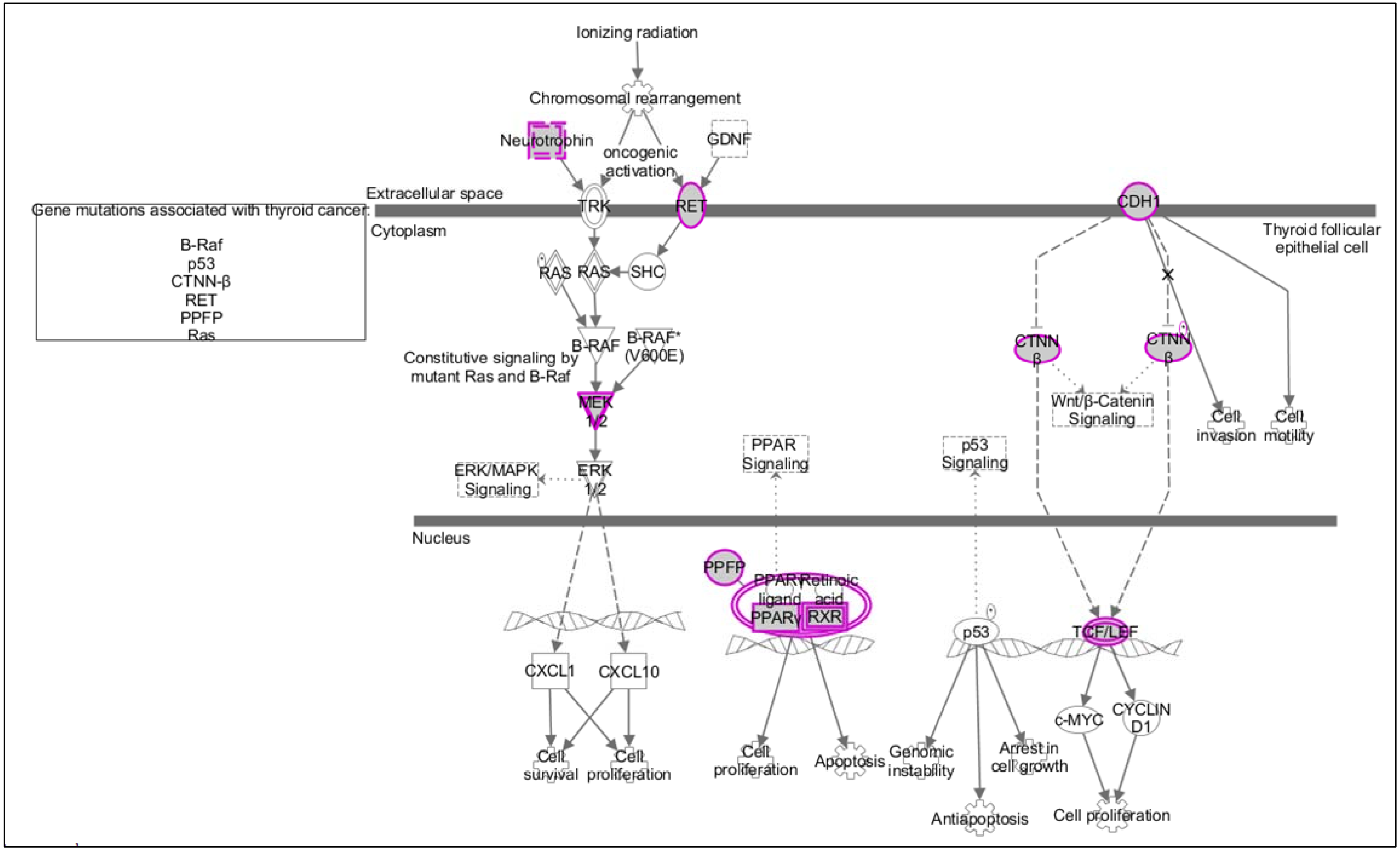

66-Regulaion of Cellular Mechanics by Calpain Protease

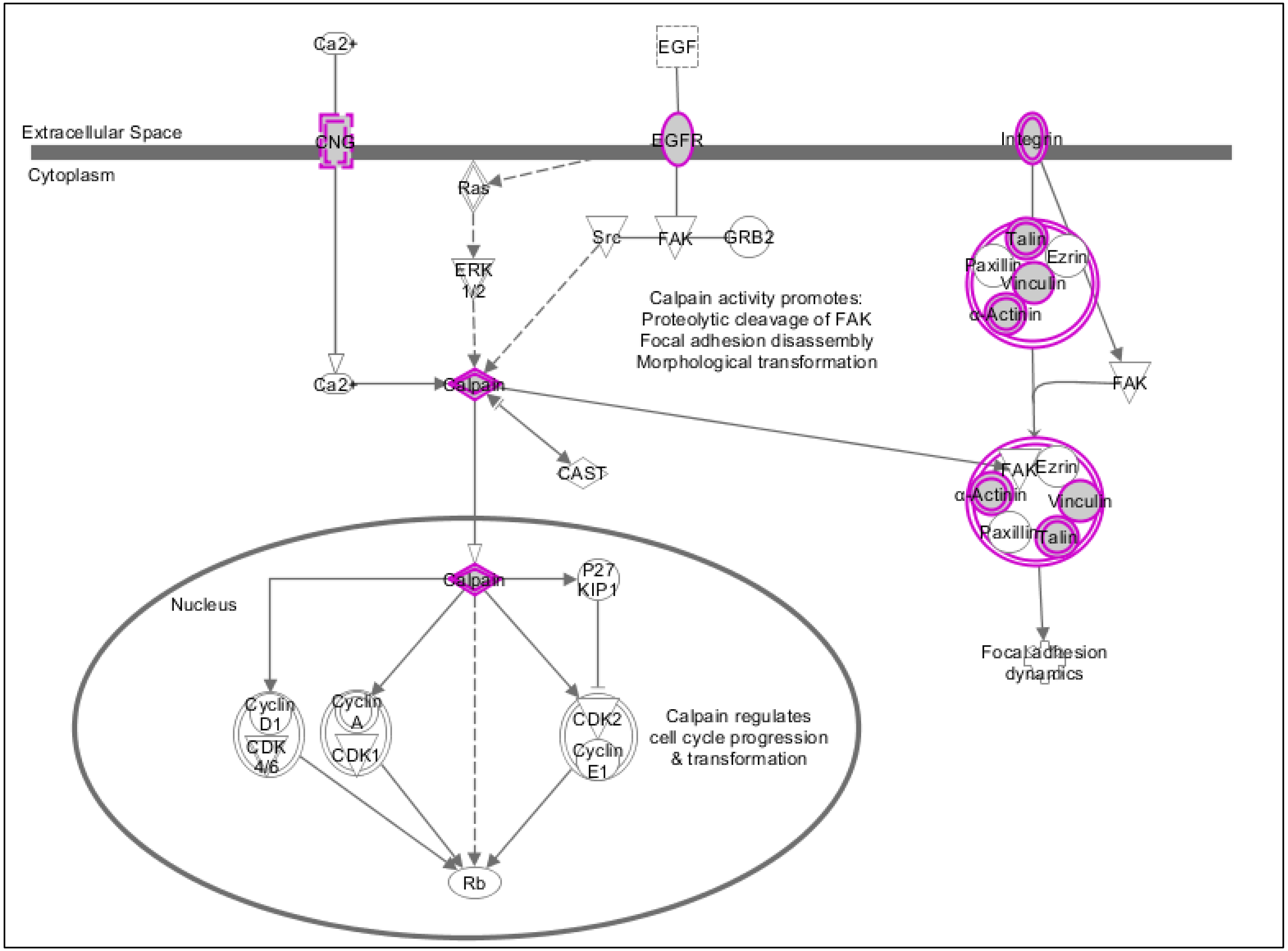

67-Gap Junction Signaling

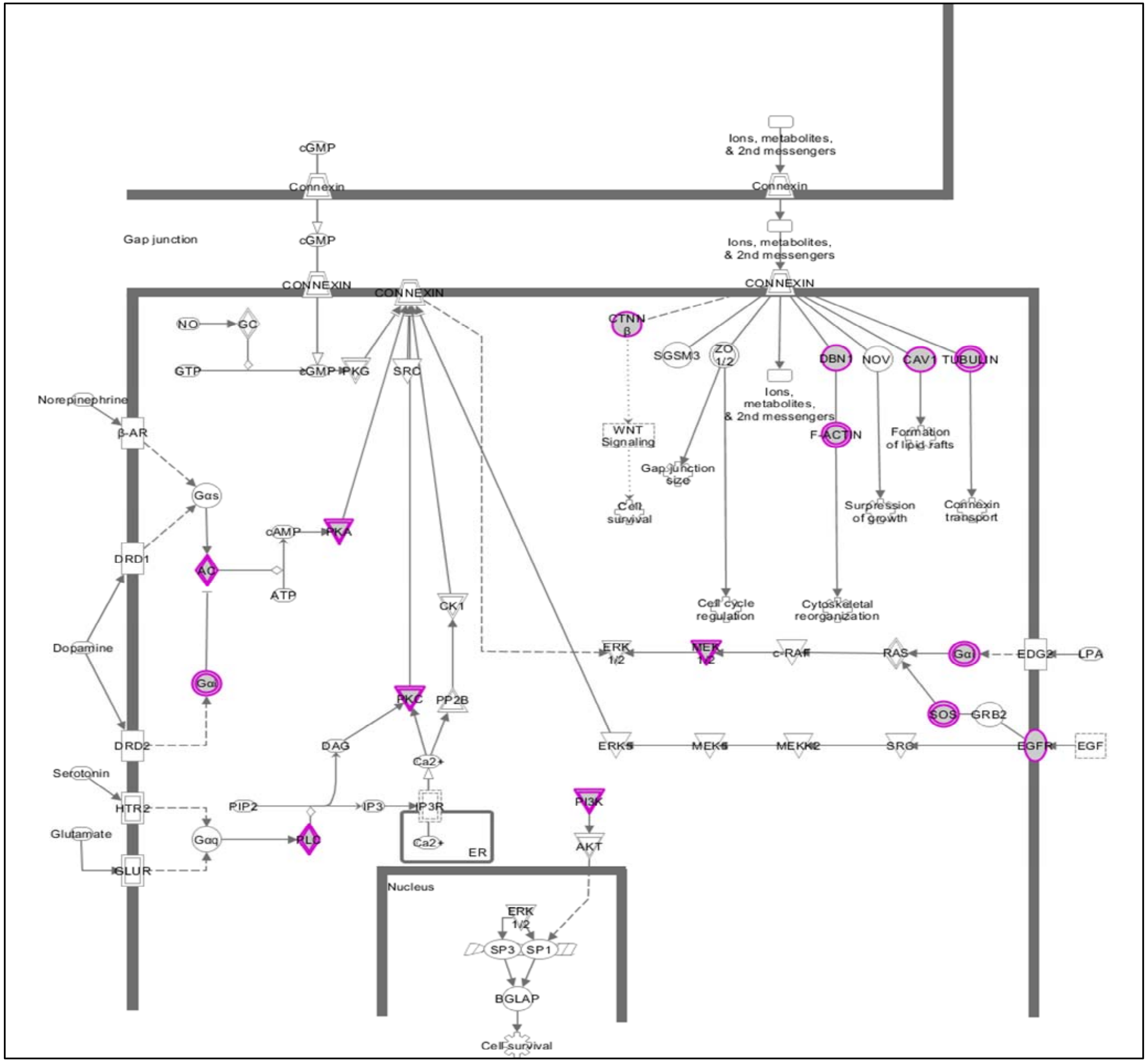

68-Lipid Antigen Presentation by CD1

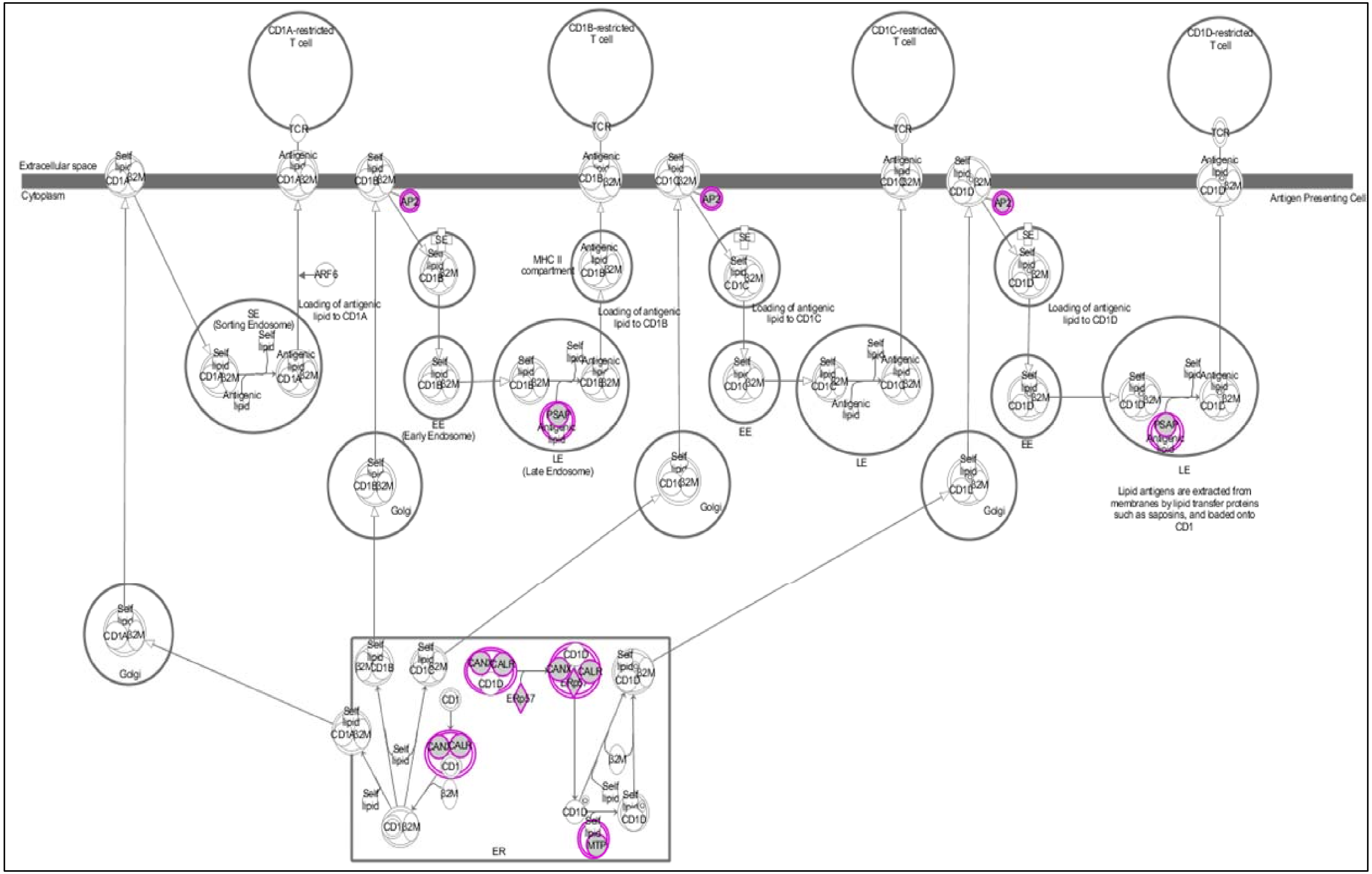

69-ERK-MAPK Signaling

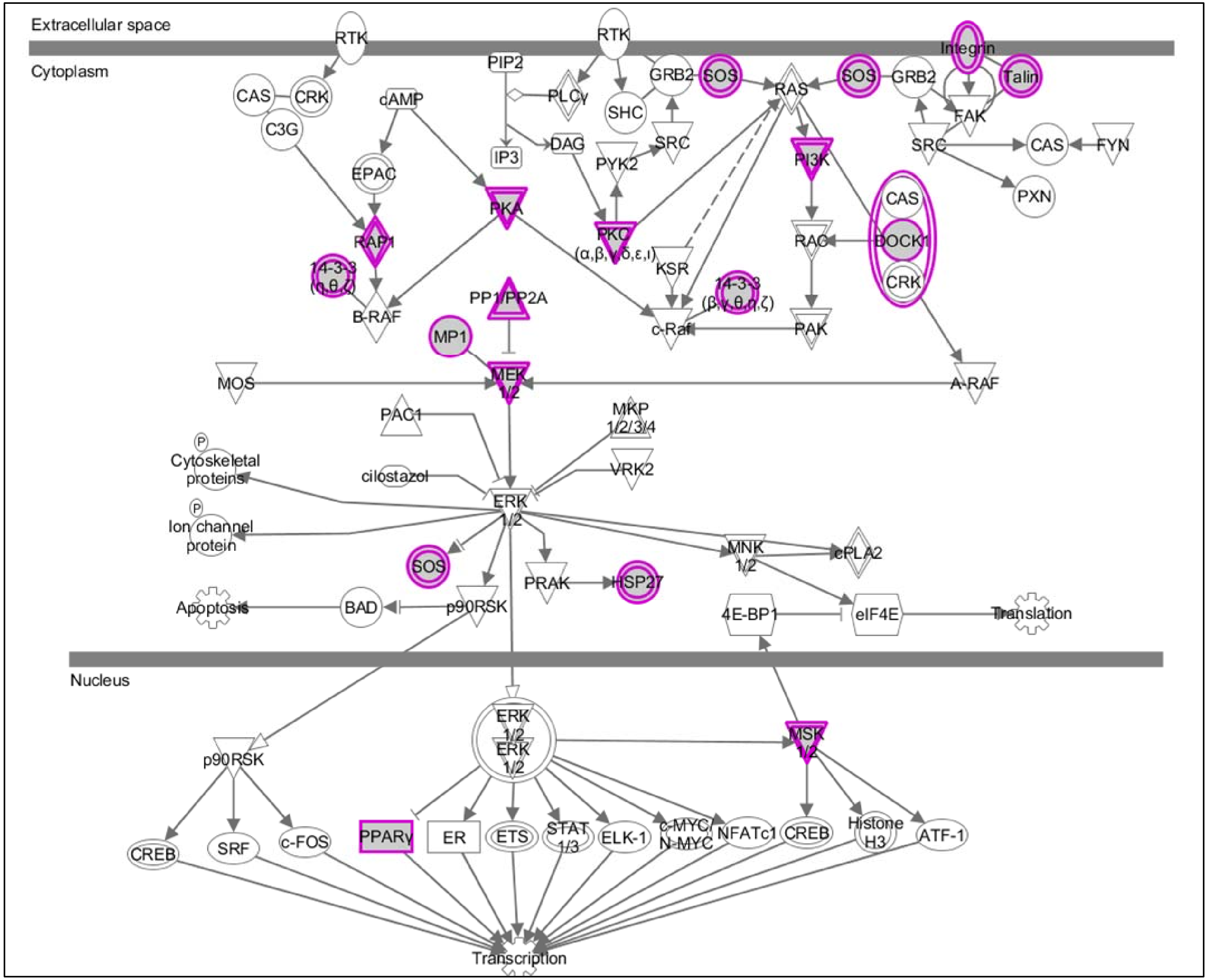

70-Serotonin Degradation

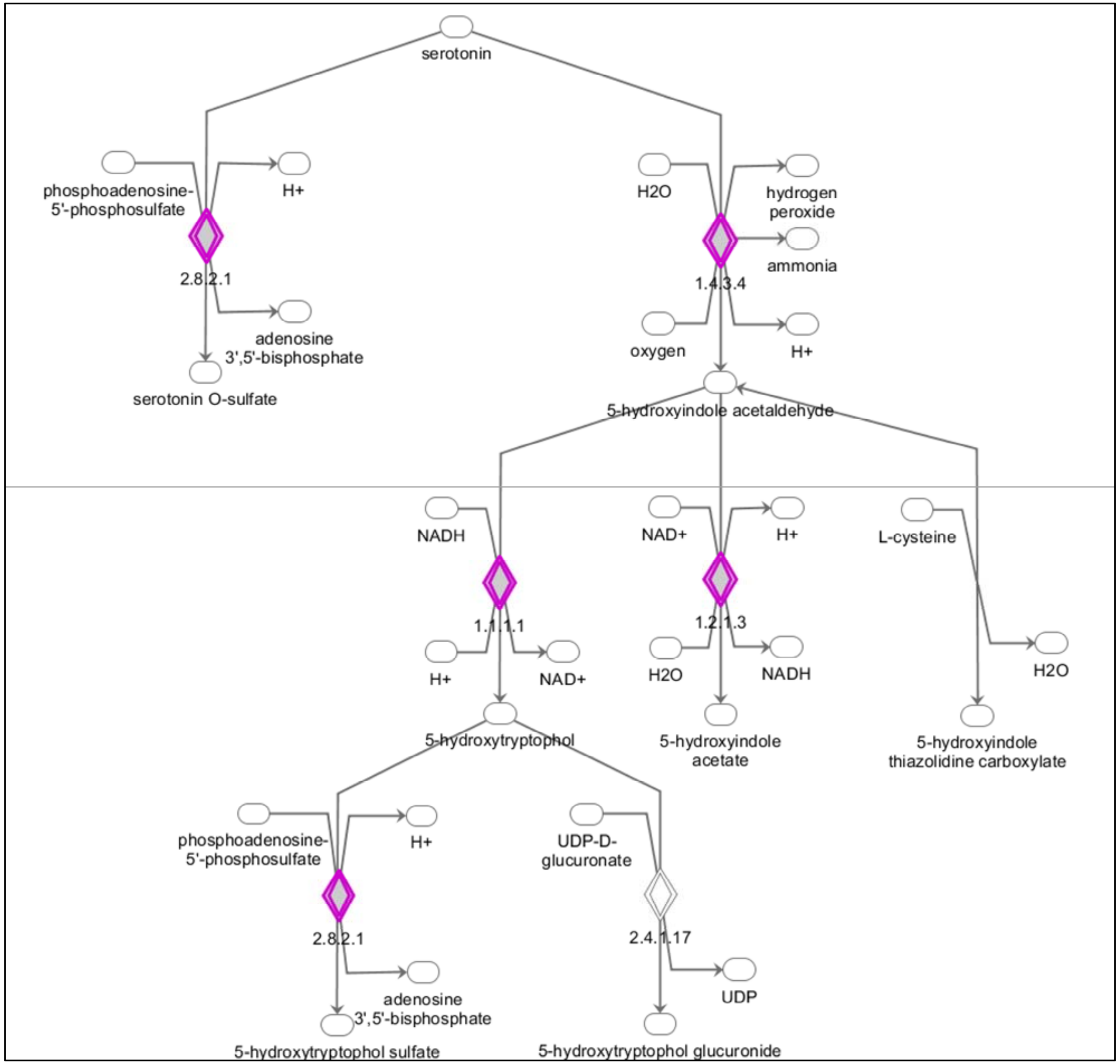

71-Leukocyte Extravasation Signaling

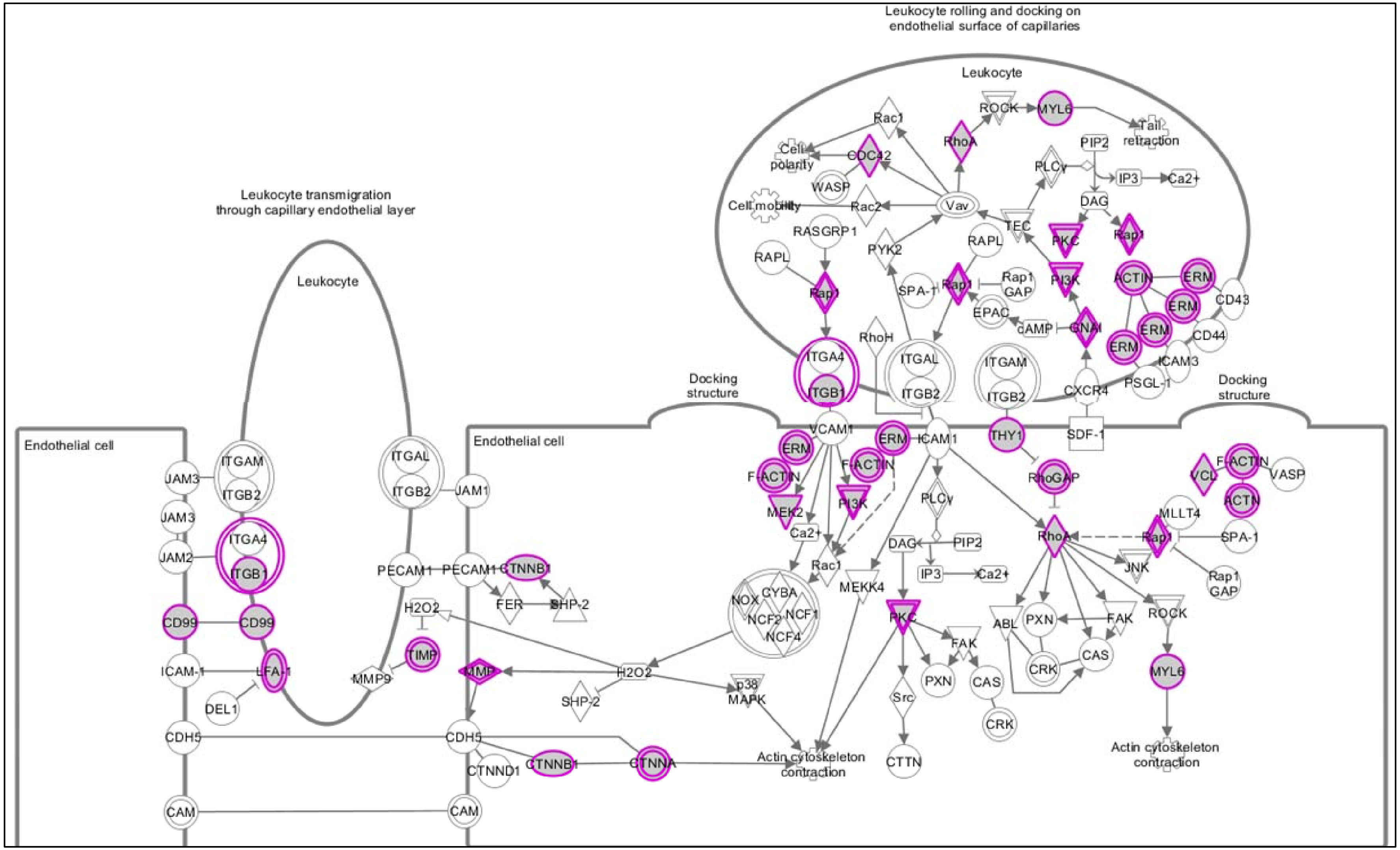

72-DNA Double-Strand Break Repair by Non-Homologous End Joining

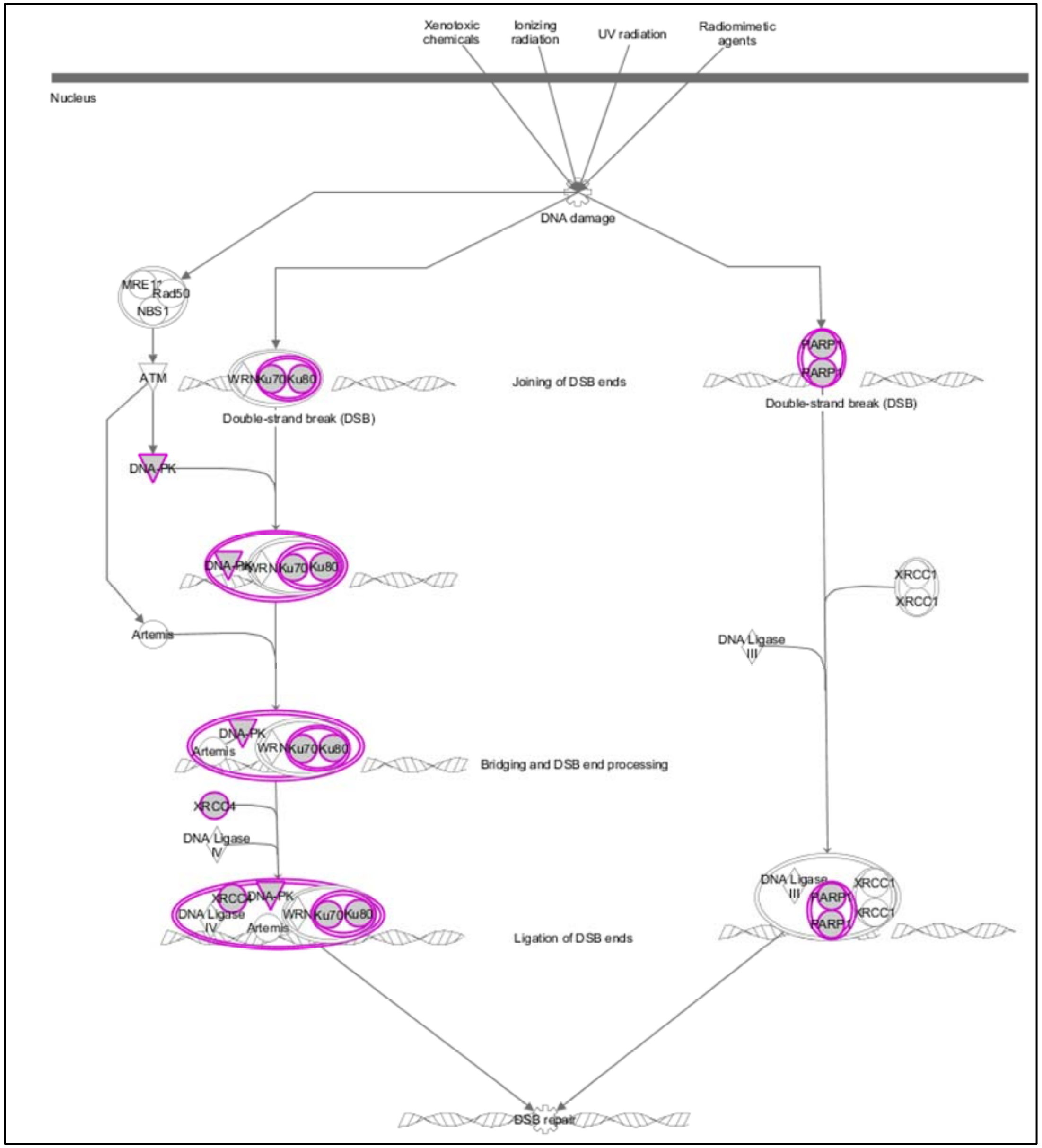

73-Agrin Interactions at Neuromuscular Junction

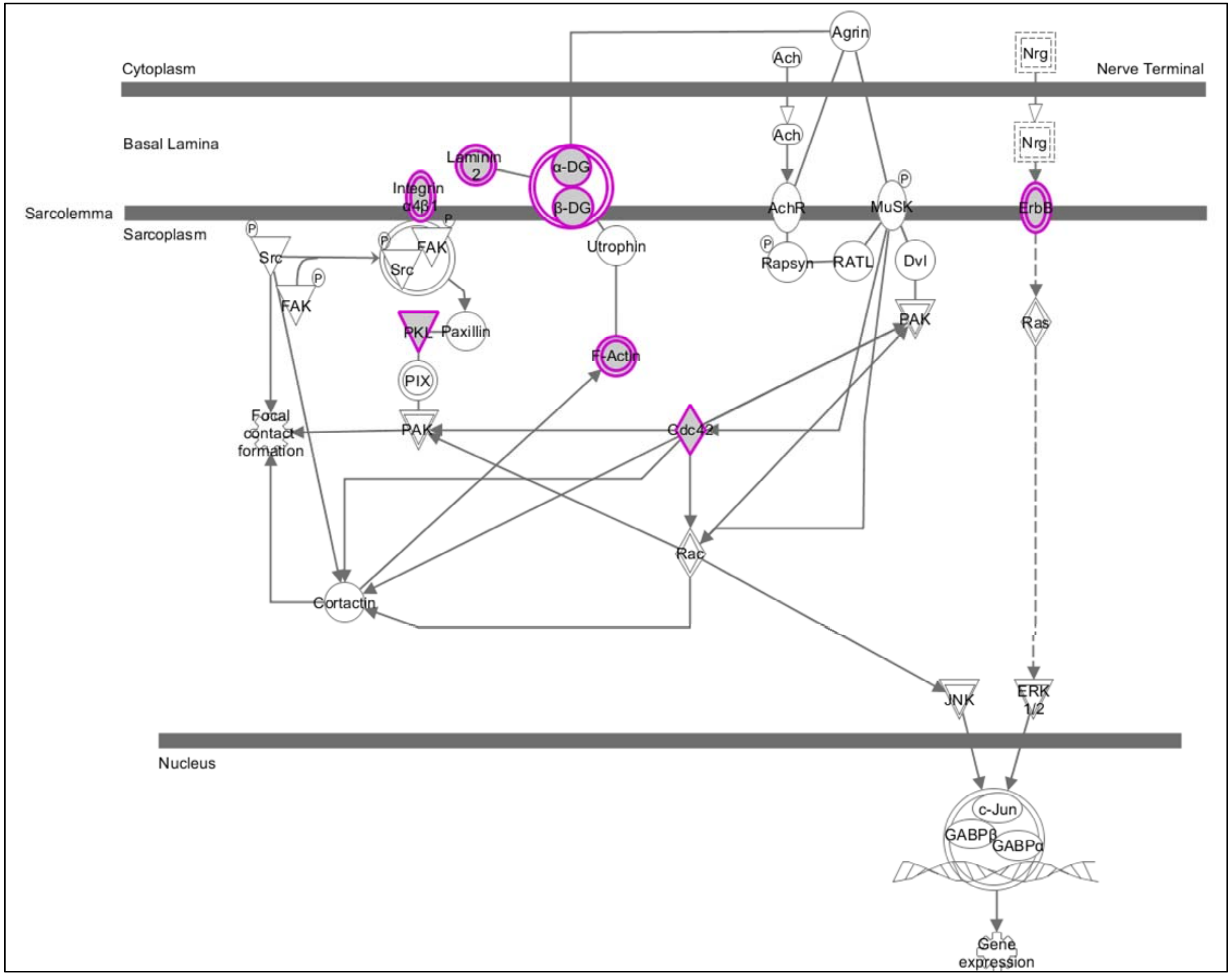

74-Molecular Mechanisms of Cancer

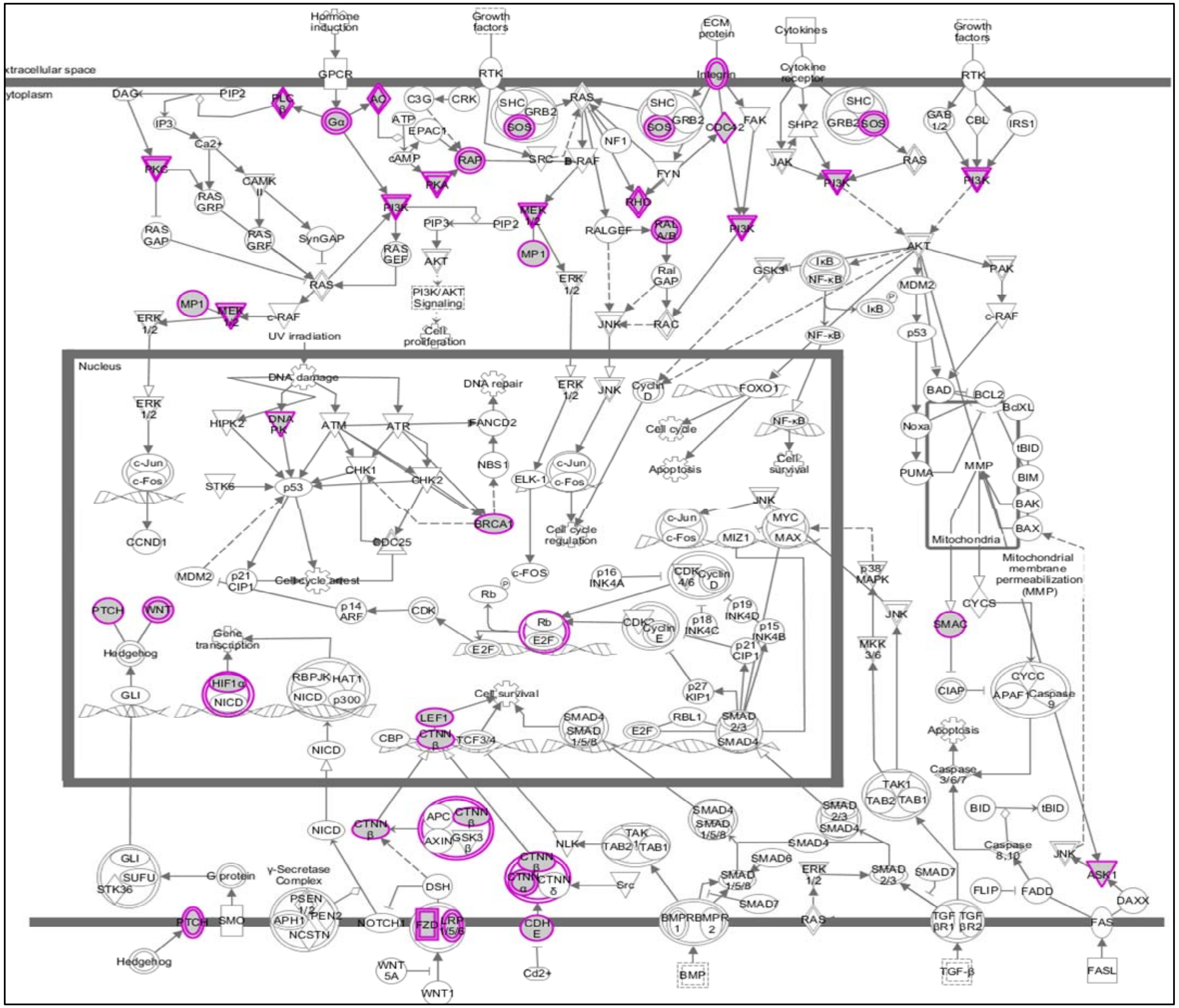

75-Oxidative Ethanol Degradation Ⅲ

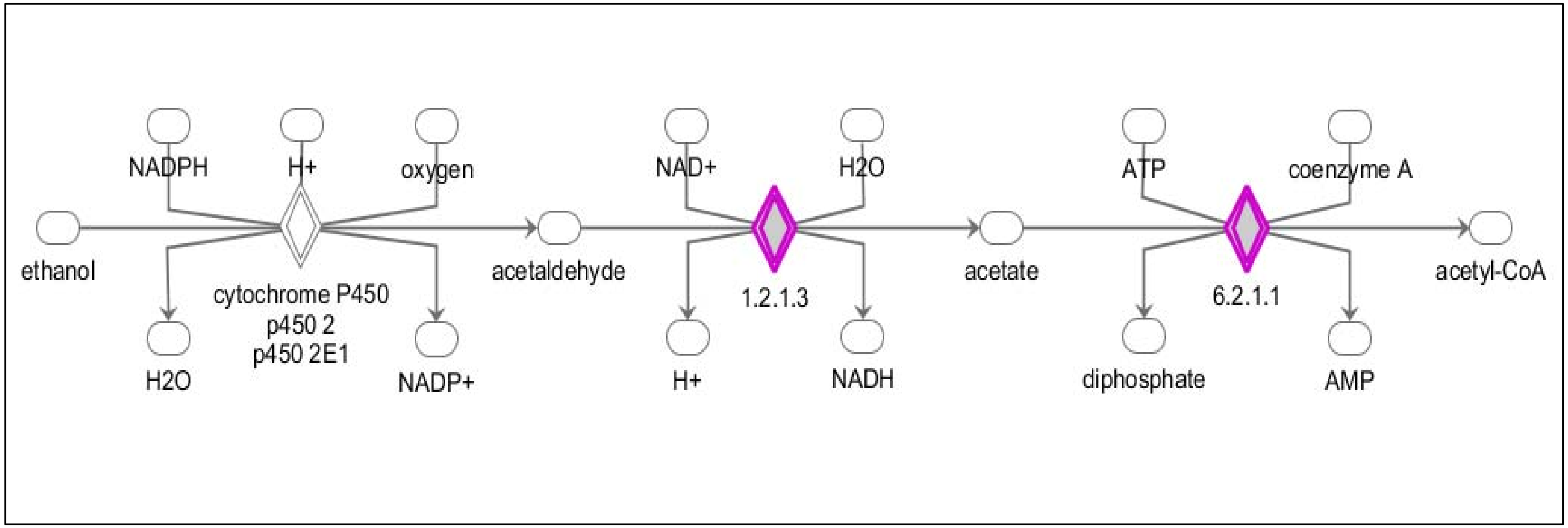

76-eNOS Signaling

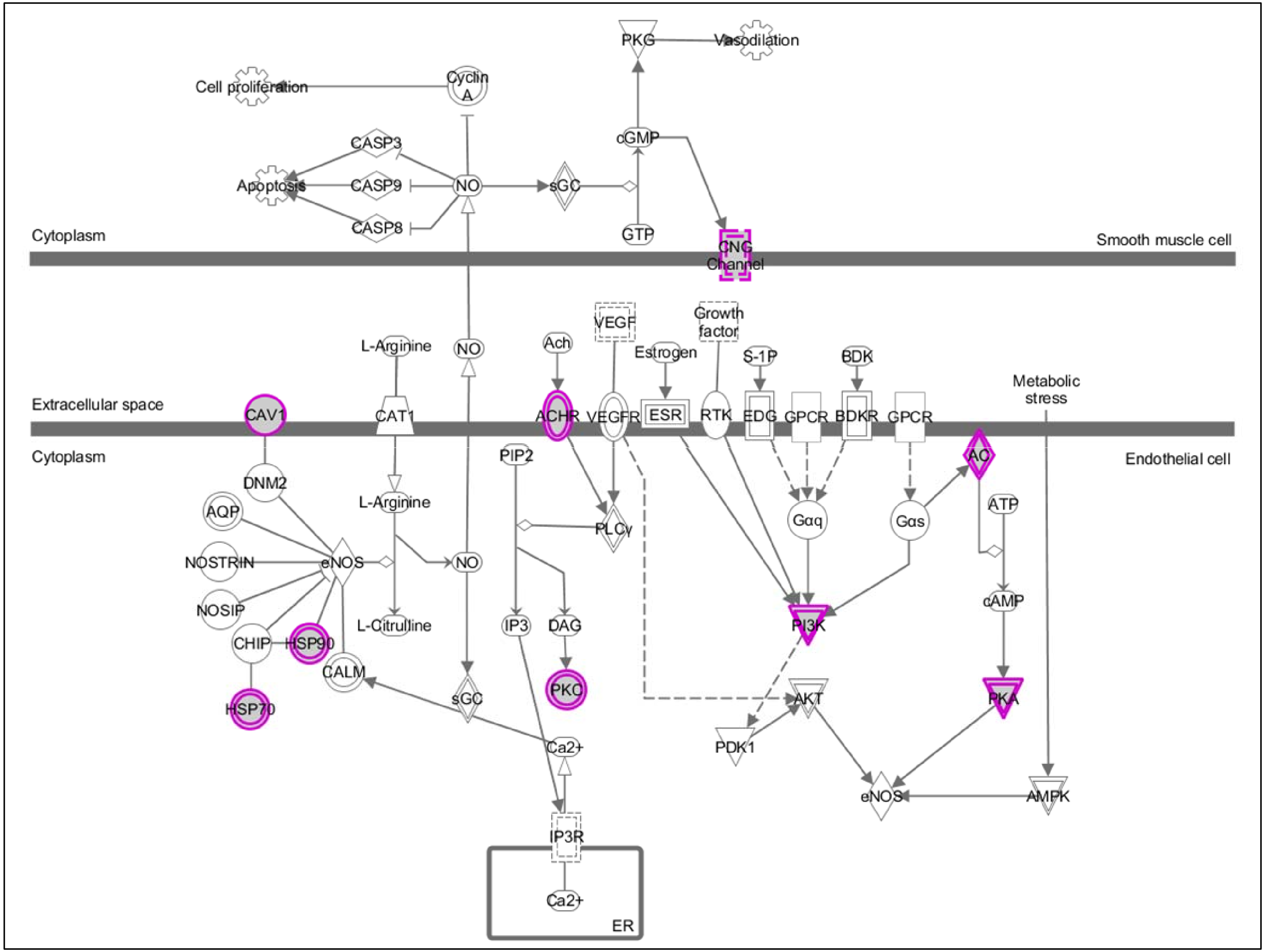

77-Telomere Extension by Telomerase

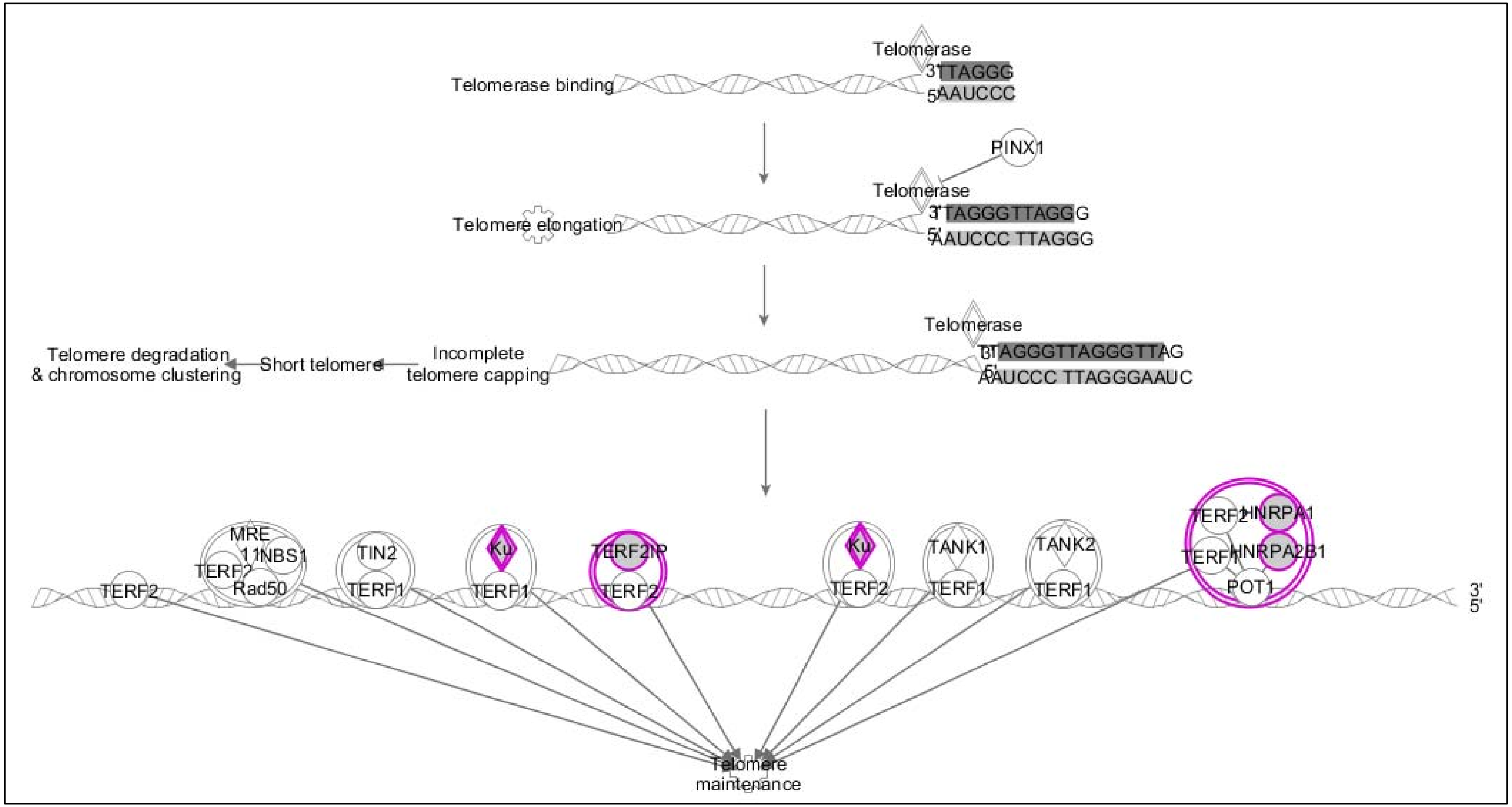

78-tRNA Charging

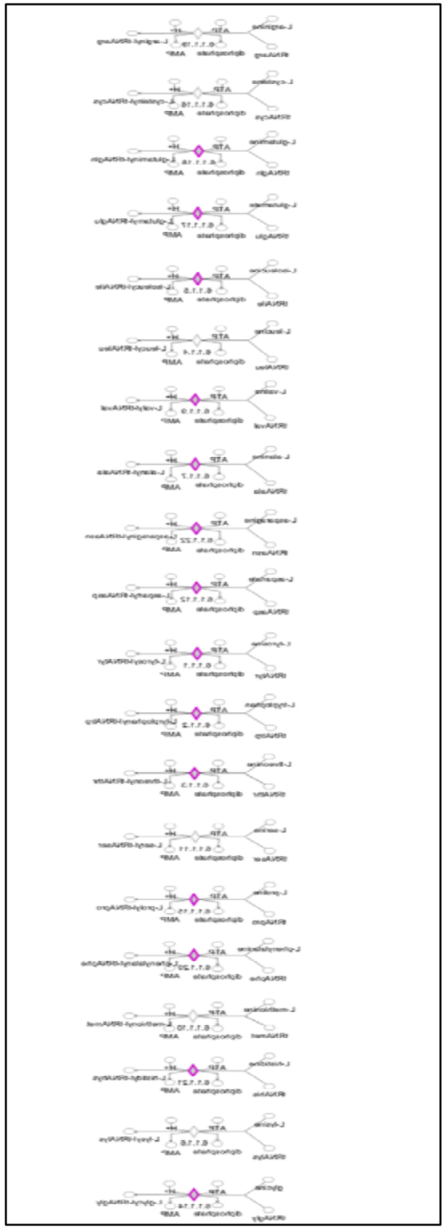

79-Putrescine Degradation III

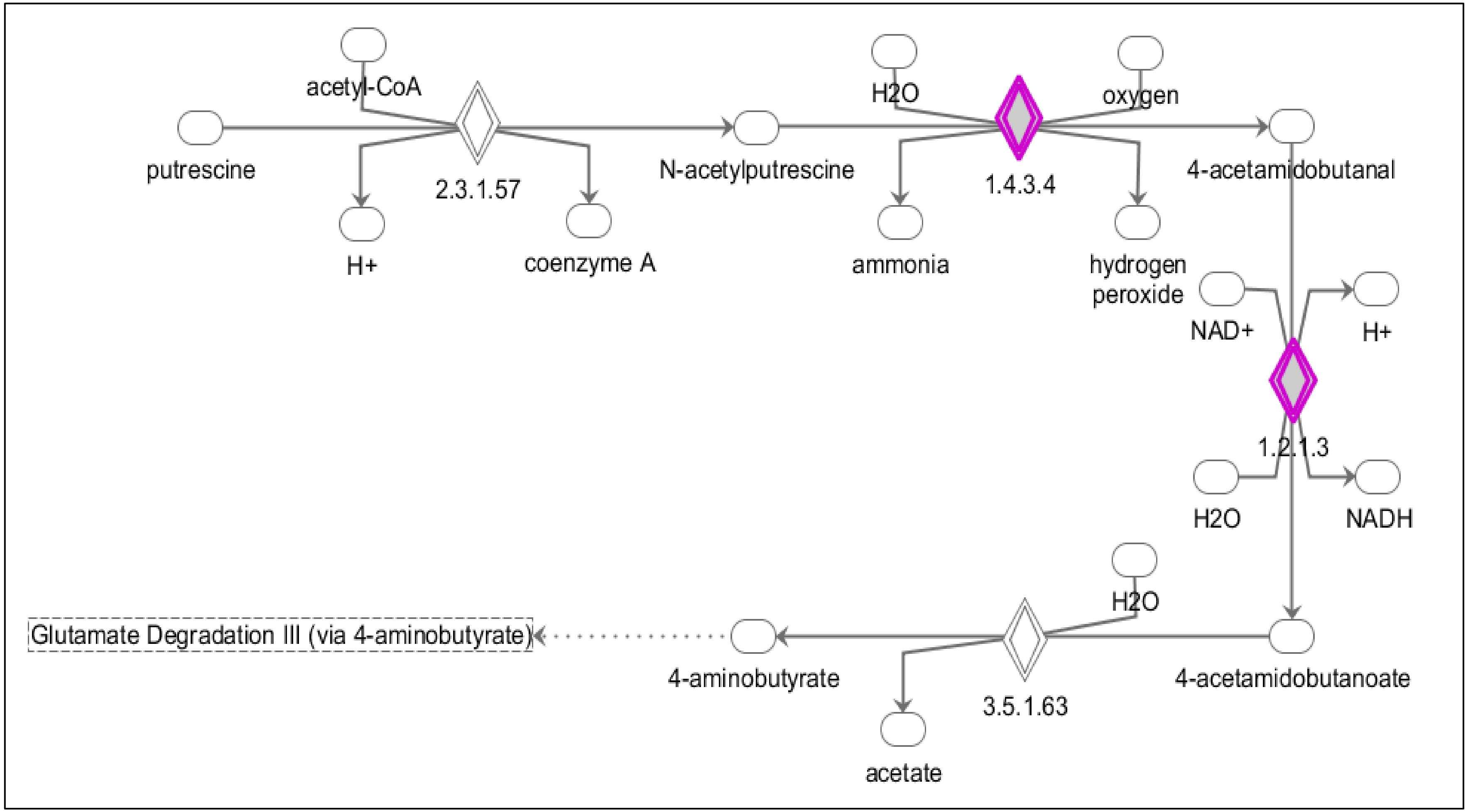

80-Dopamine Receptor Signaling

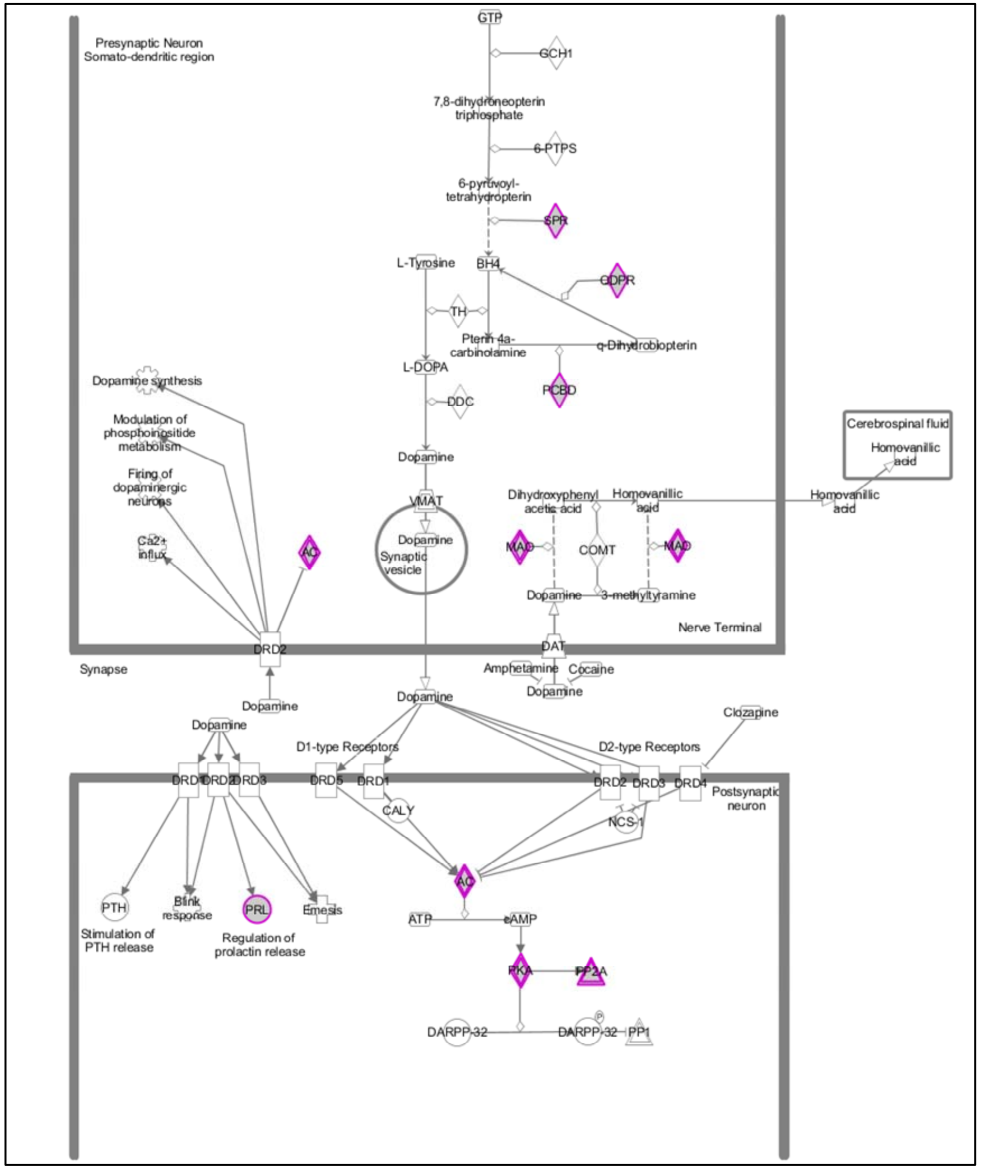

81-Relaxin Signaling

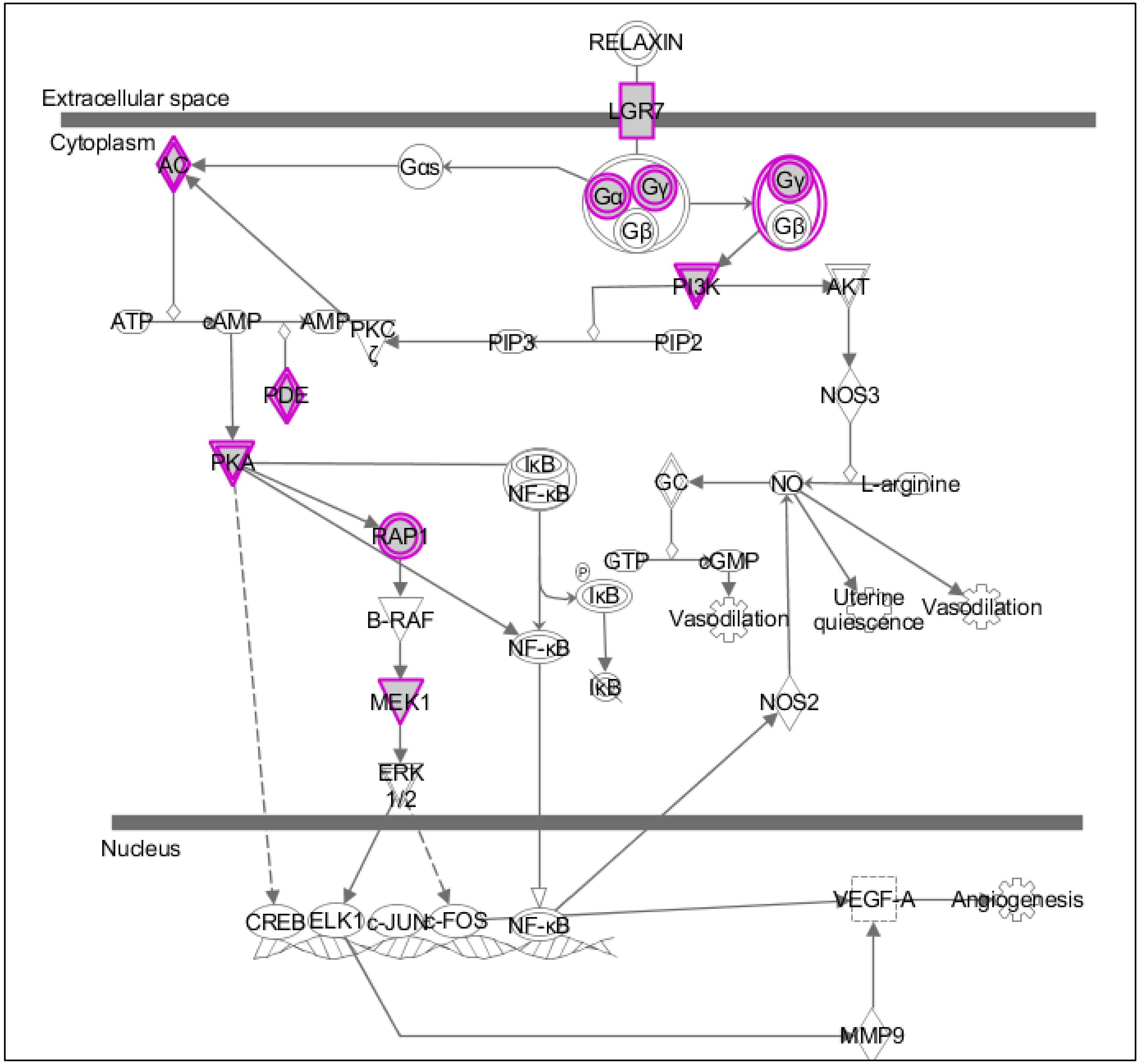

82-Neuregulin Signaling

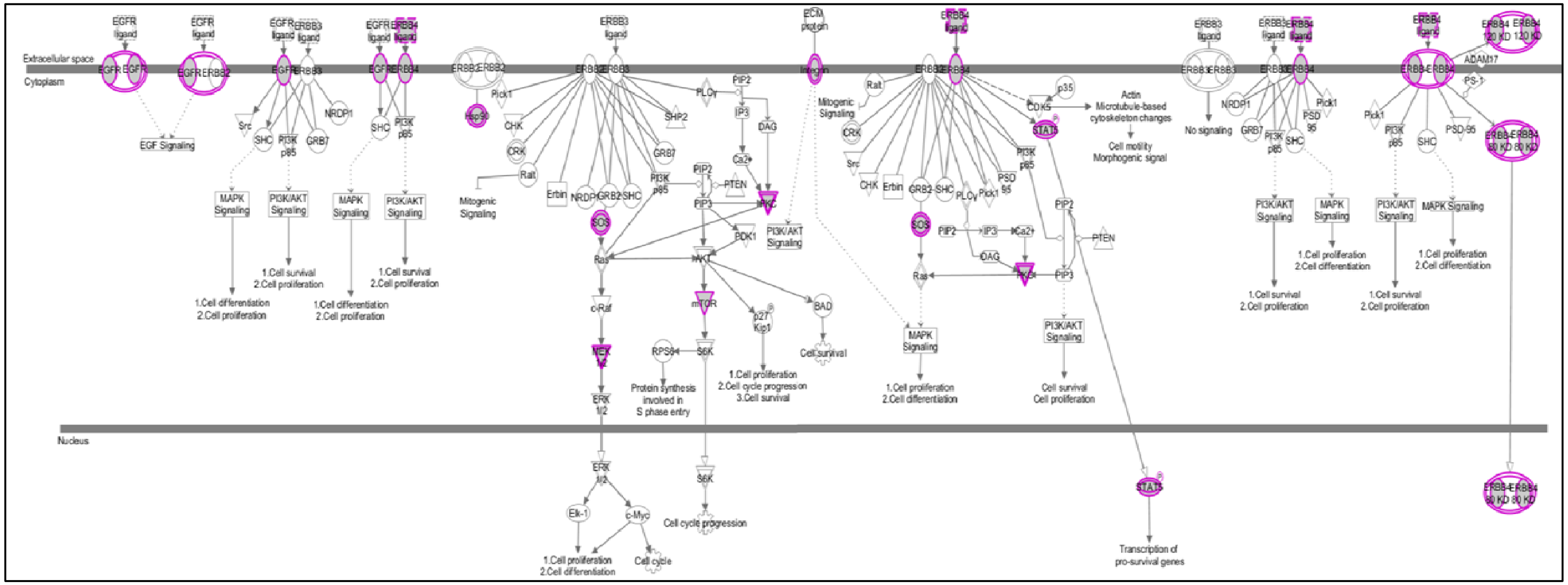

83-Dopamine Degradation

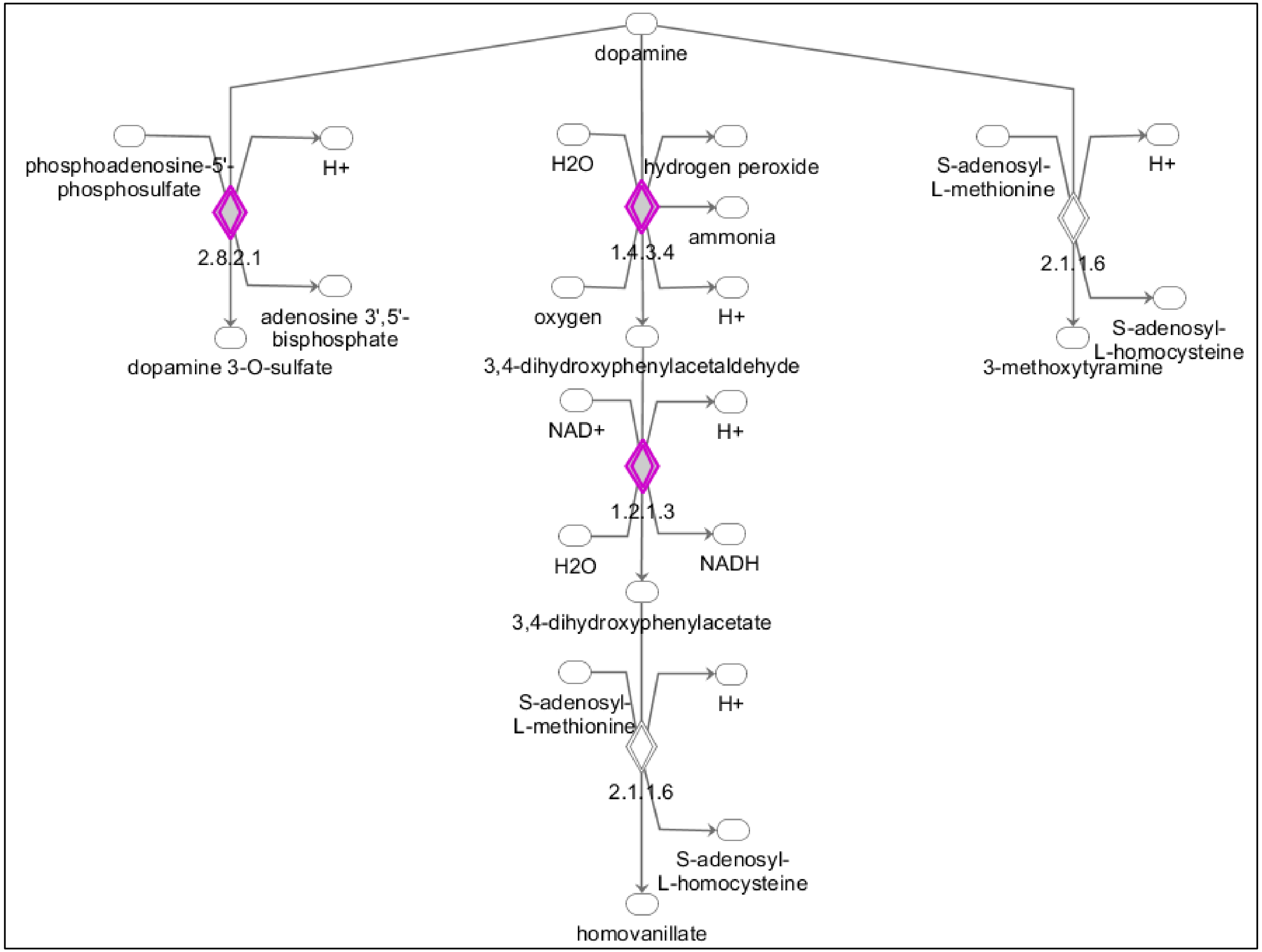

84-Intrinsic Prothrombin Activation Pathway

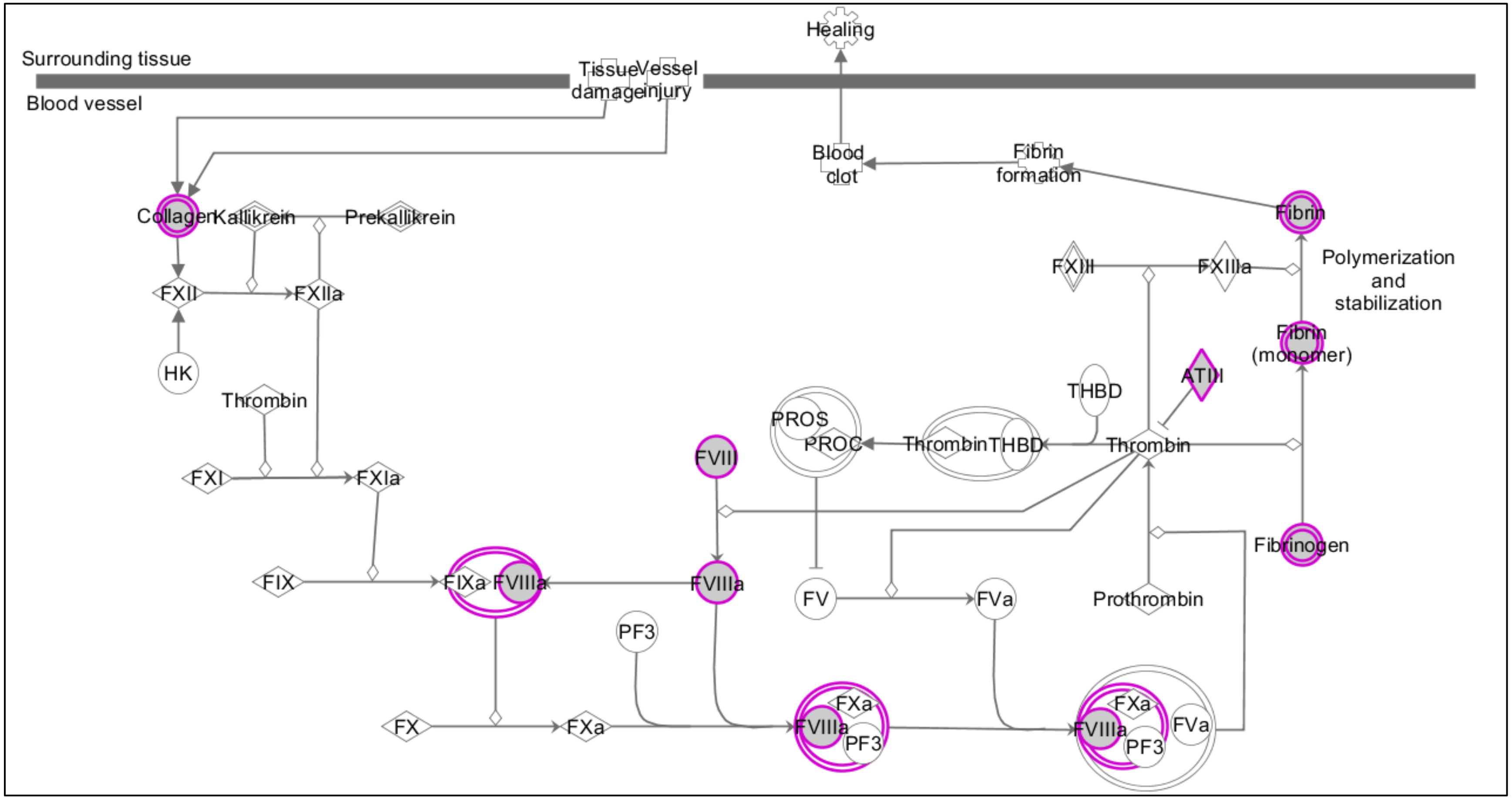

85-Actin Nucleation by ARP-WASP Complex

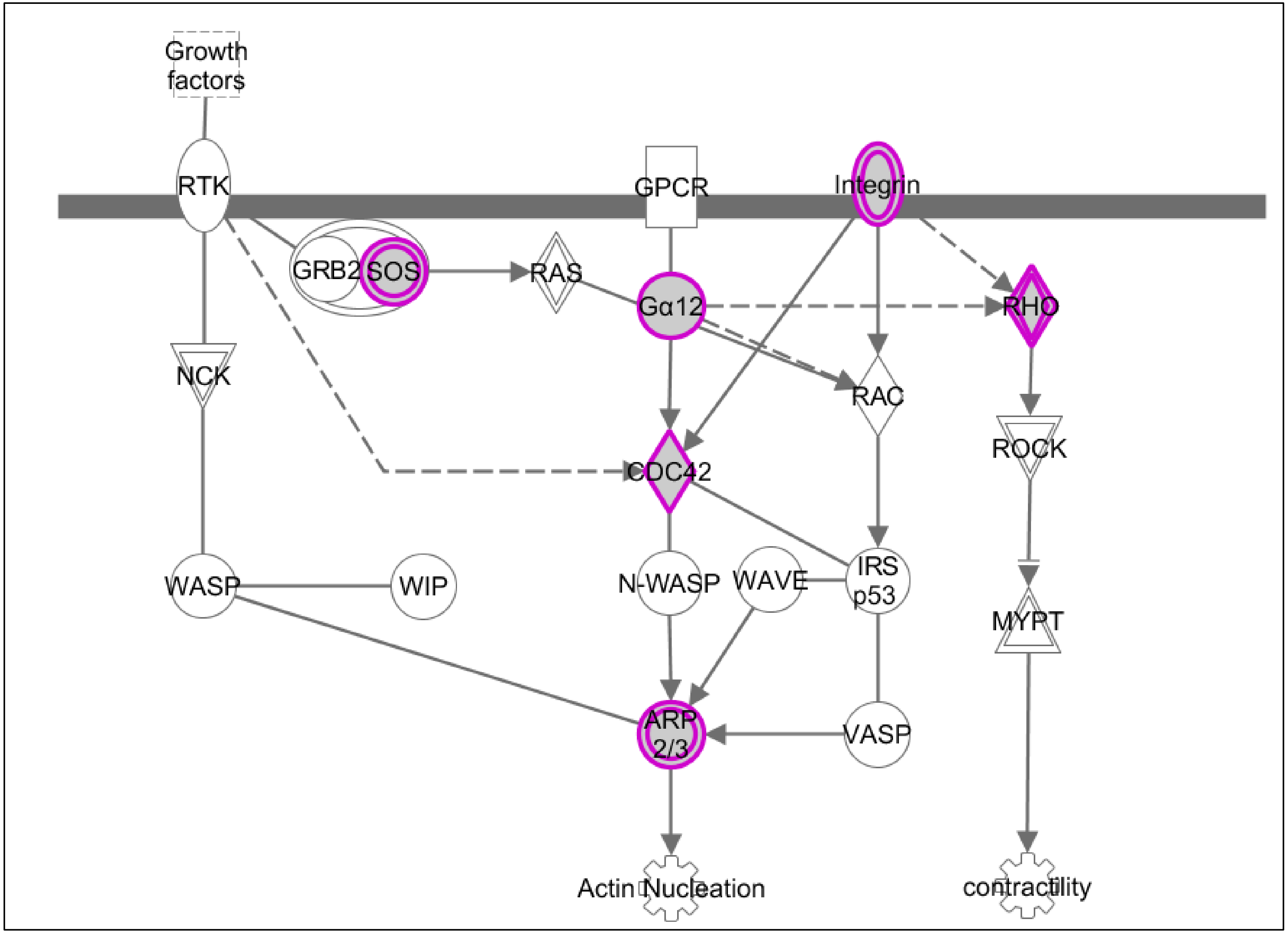

86-AMPK Signaling

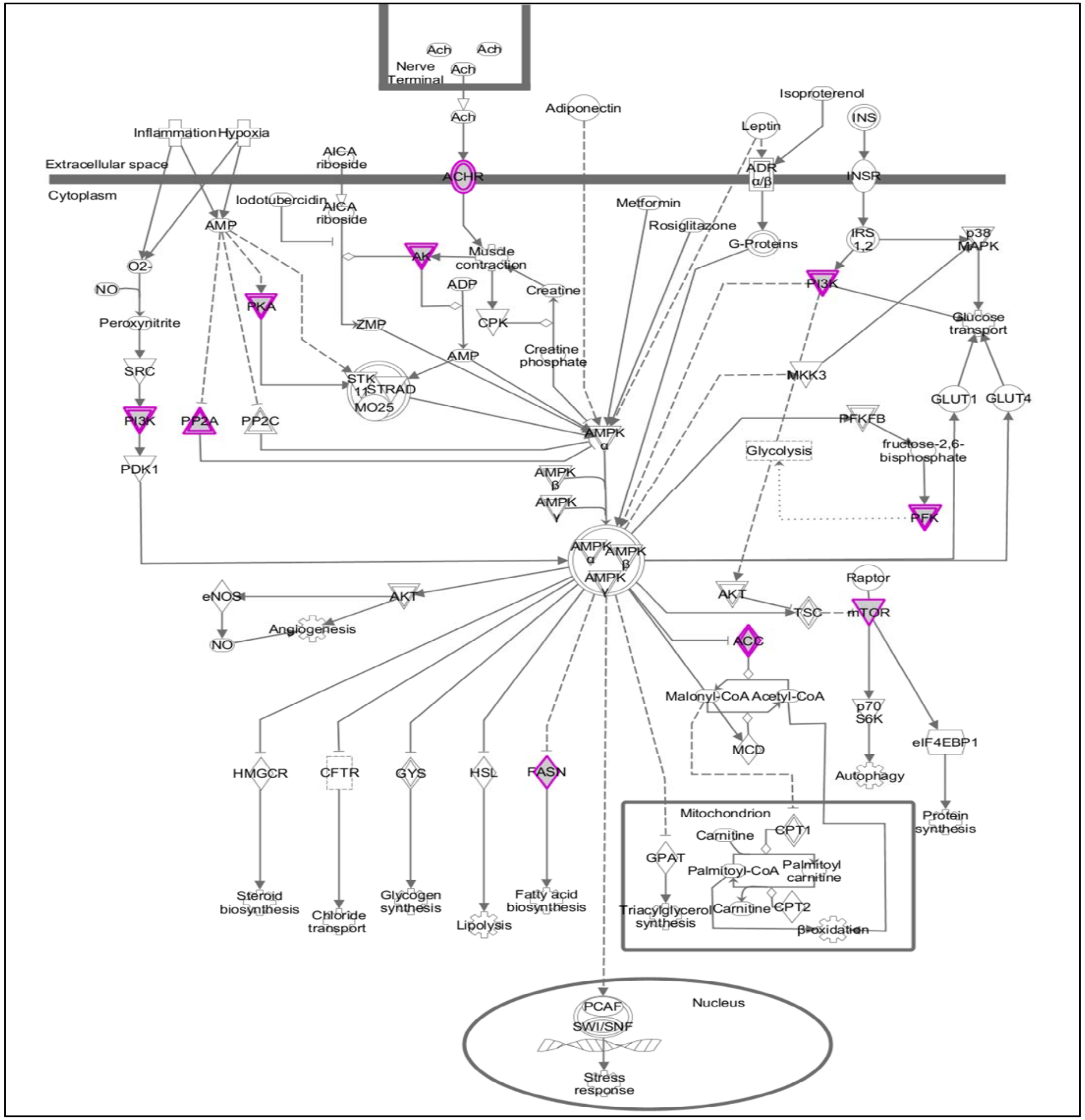

87-Sonic Hedgehog Signaling

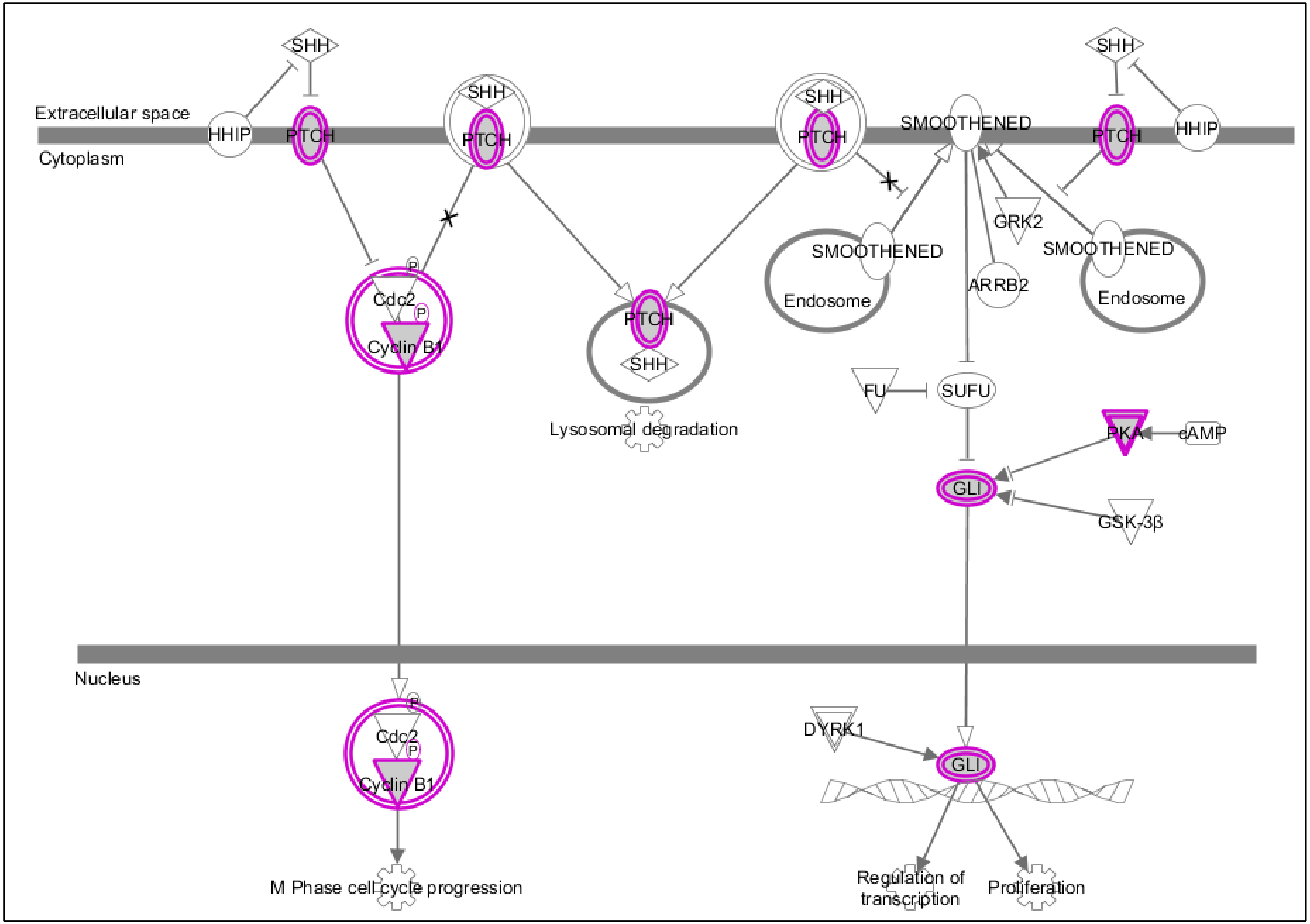

88-Rac Signaling

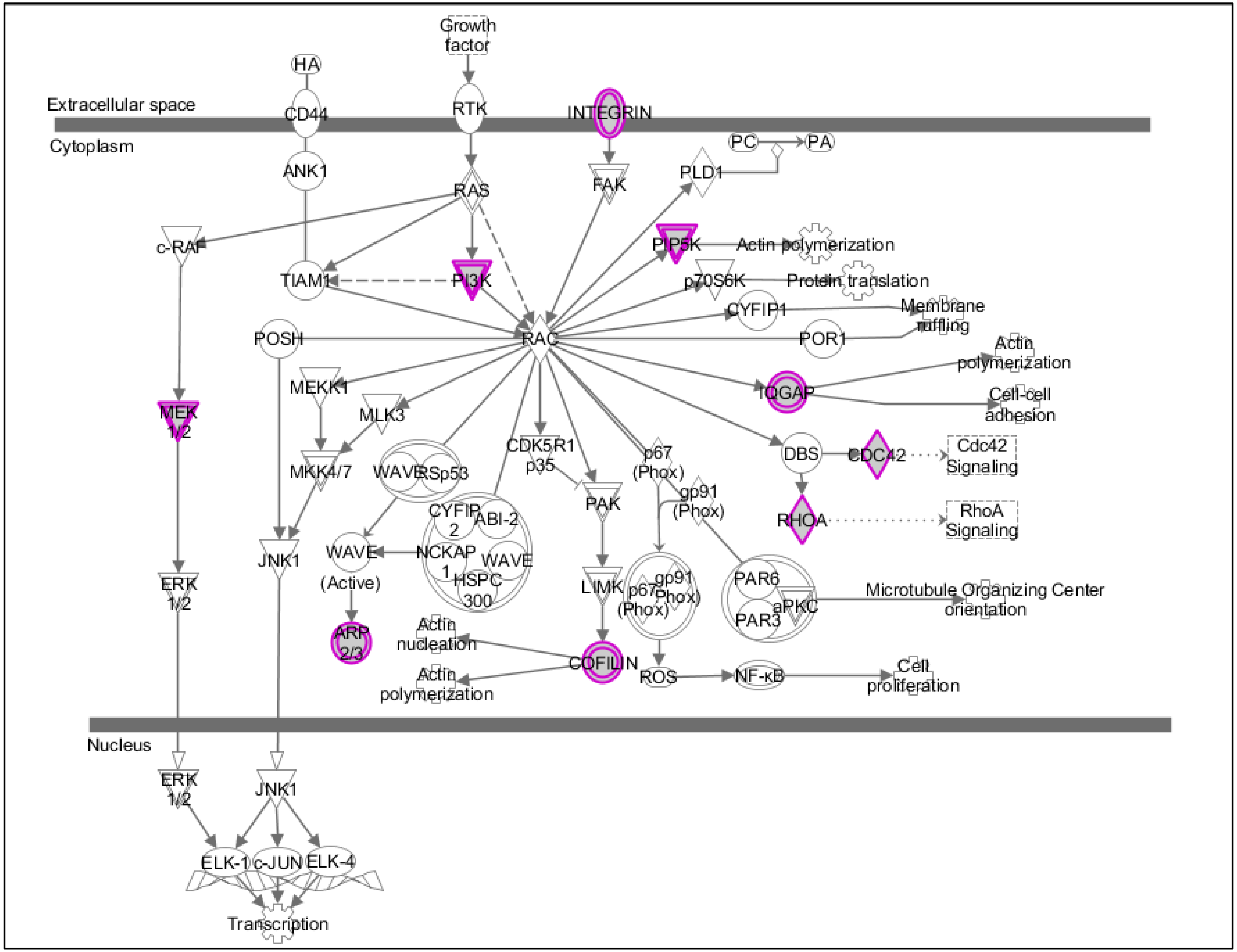

89-Breast Cancer Regulation by Stathmin1

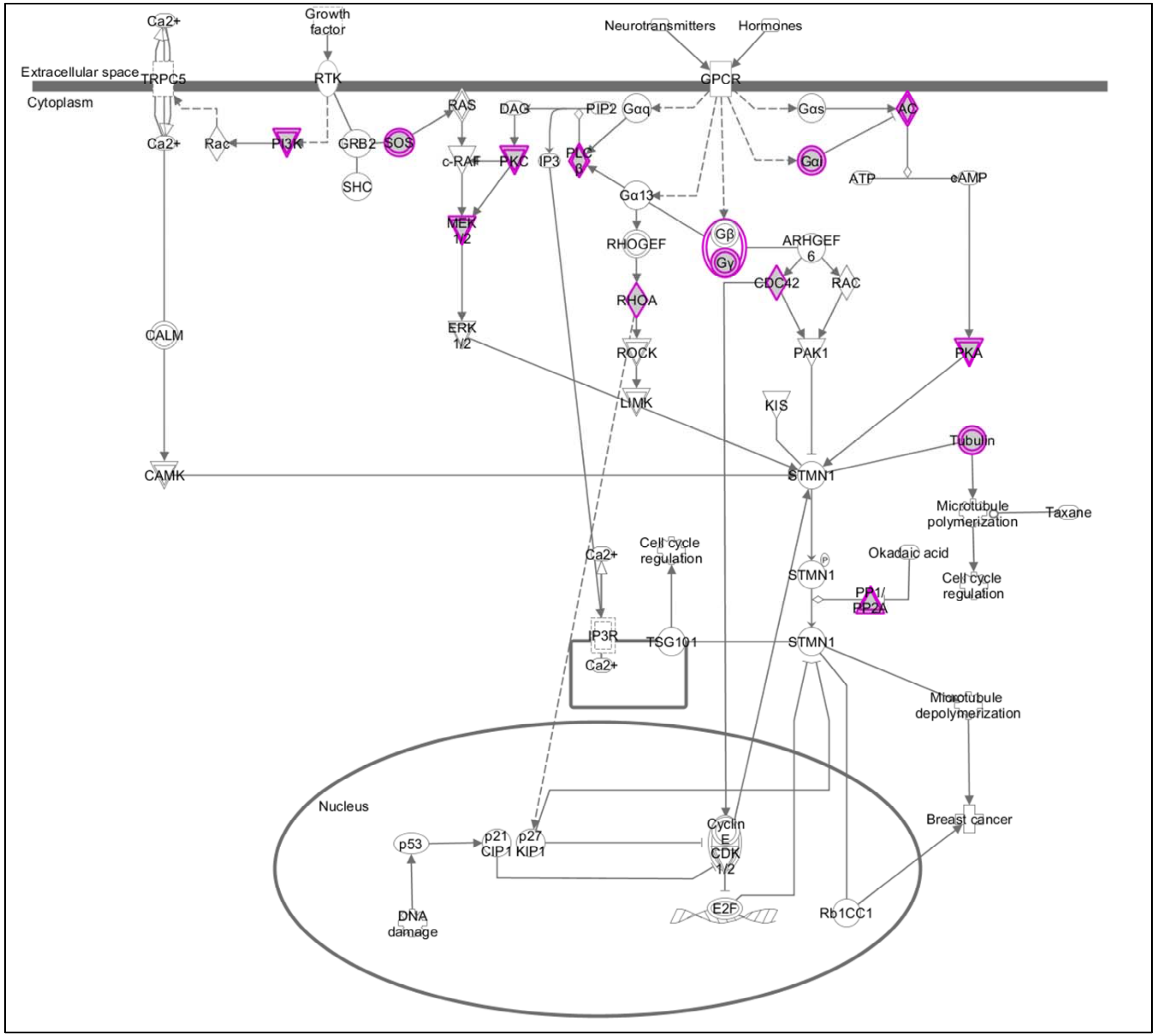

90-14-3-3-mediated Signaling

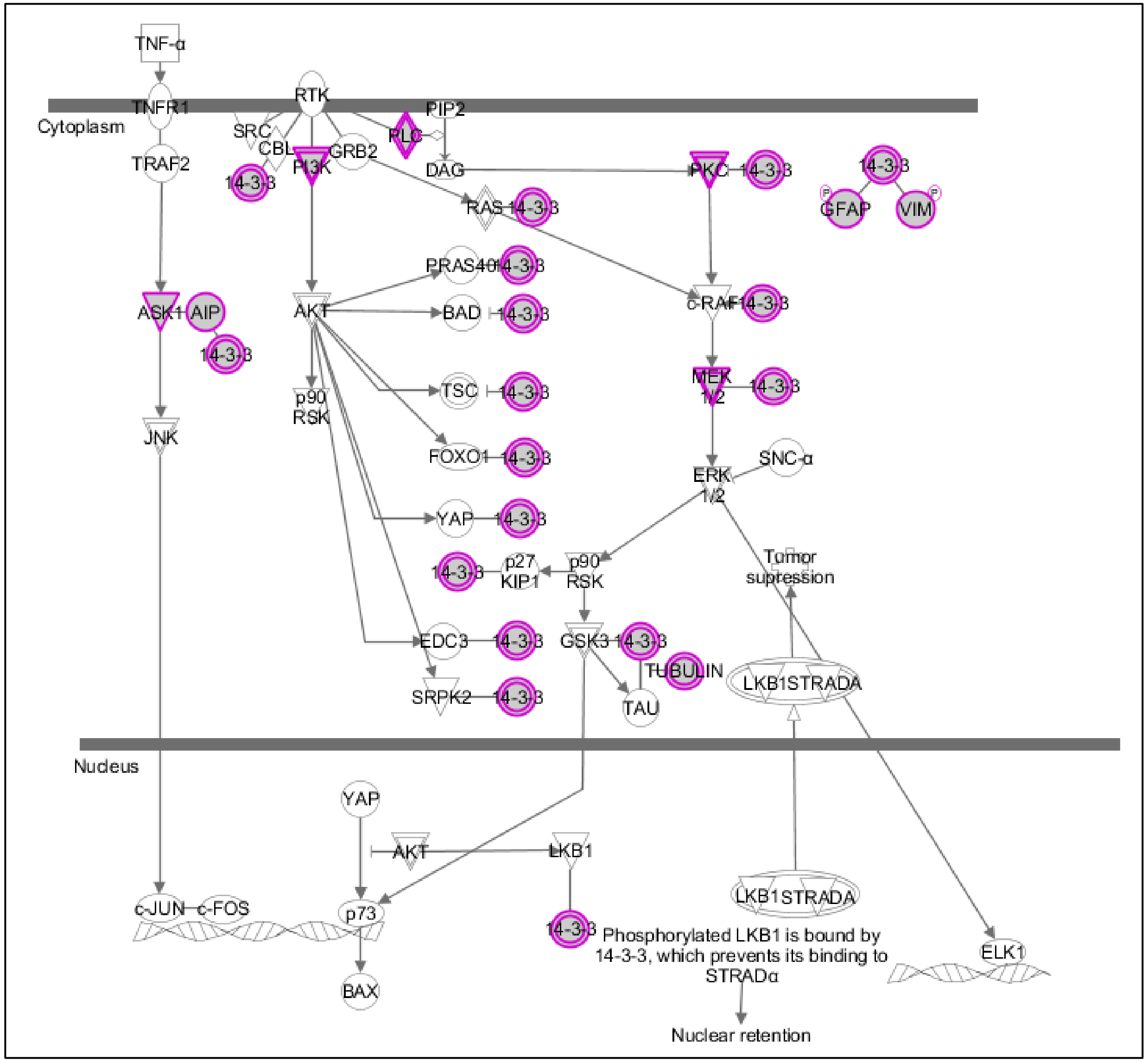

91-Leptin Signaling Obesity

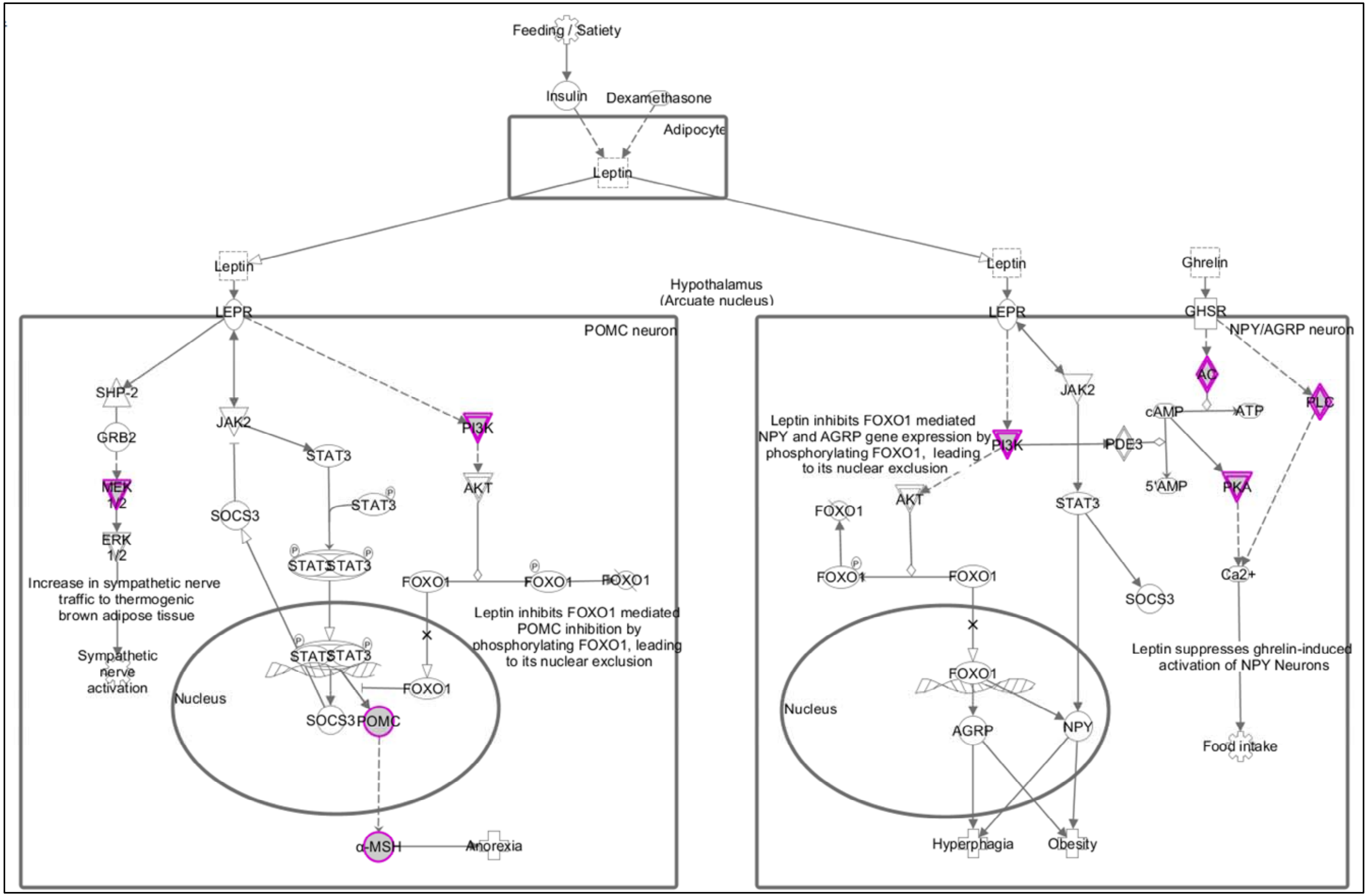

92-Spliceosomal Cycle

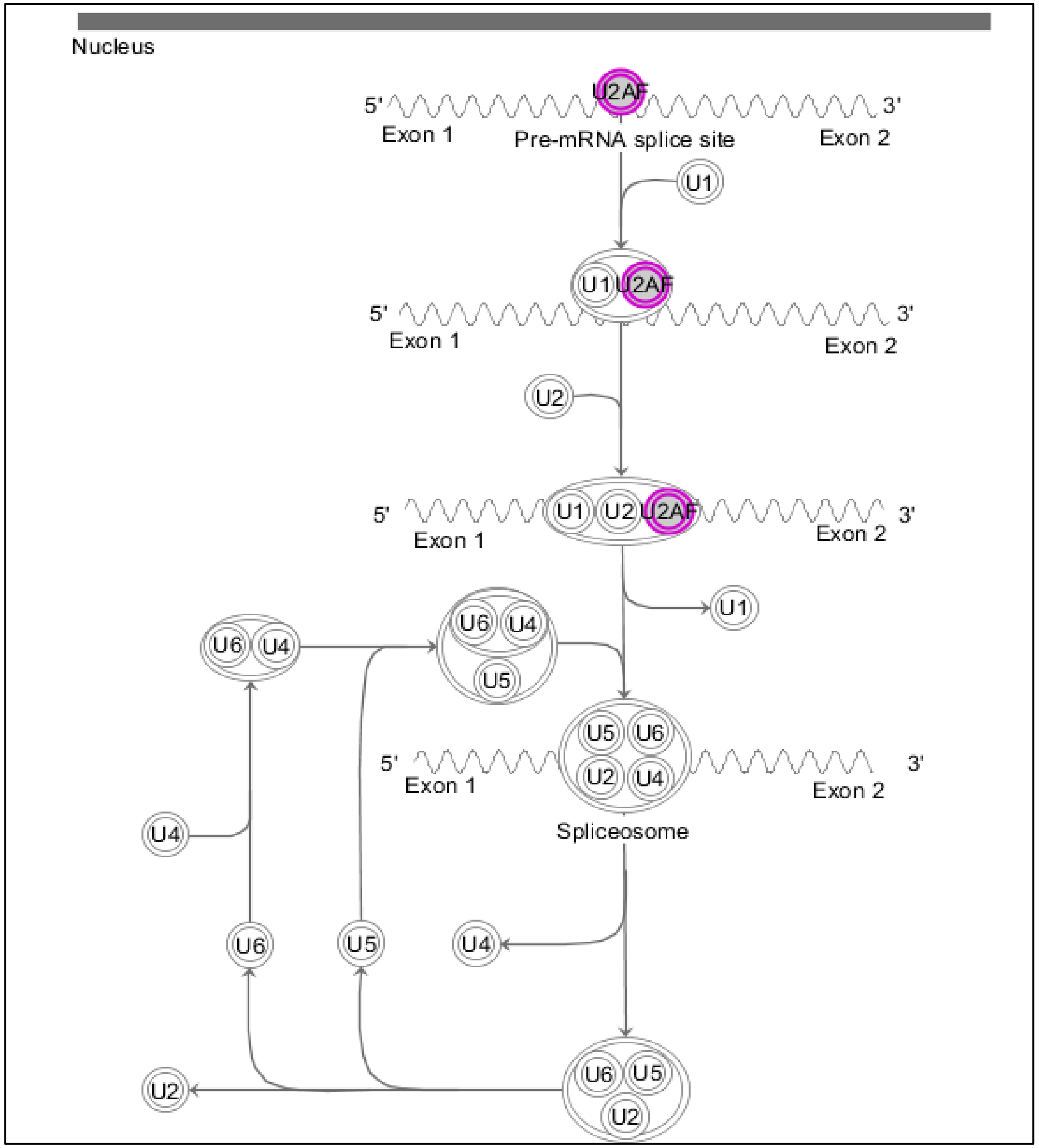

93-FAK Signaling

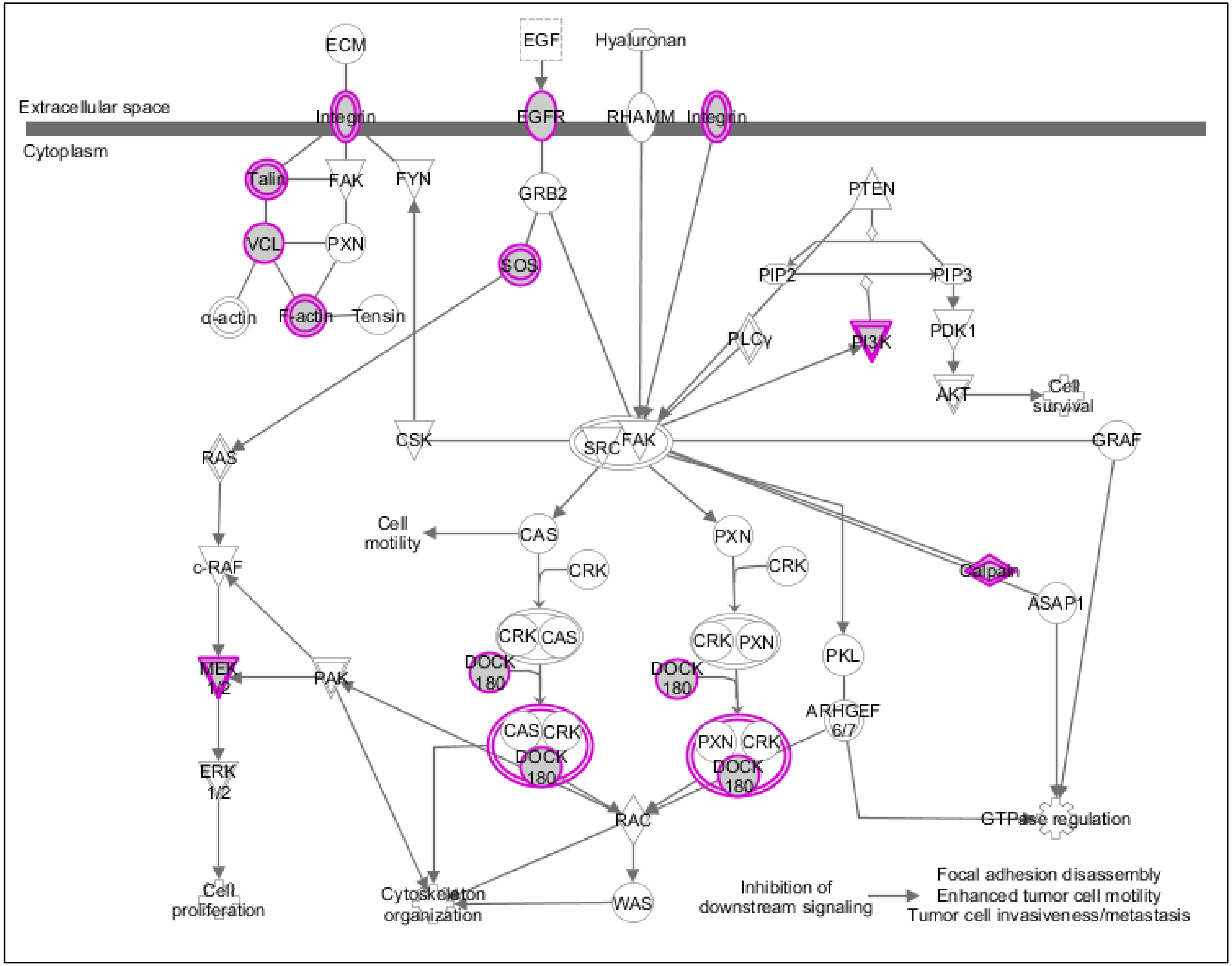

94-Role of Tissue Factor in Cancer

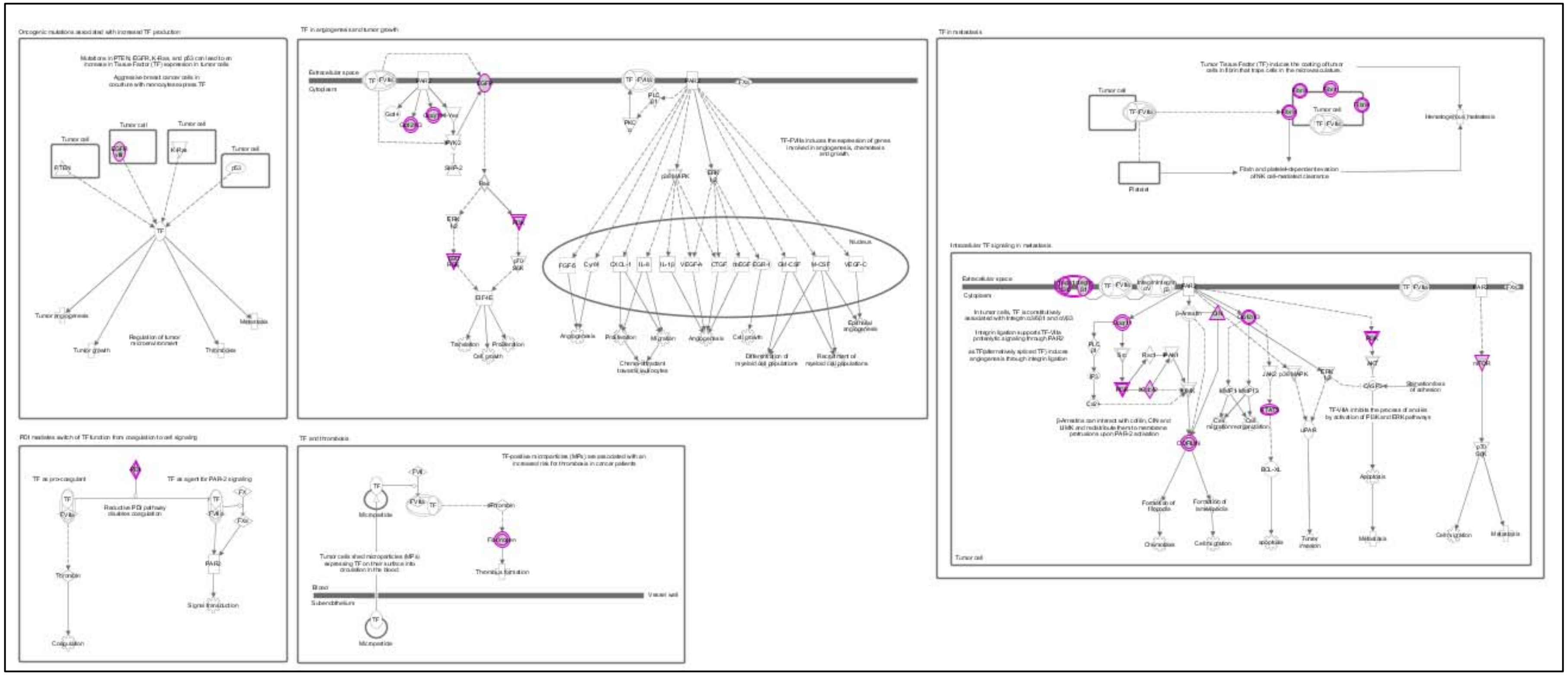

95-Paxillin Signaling

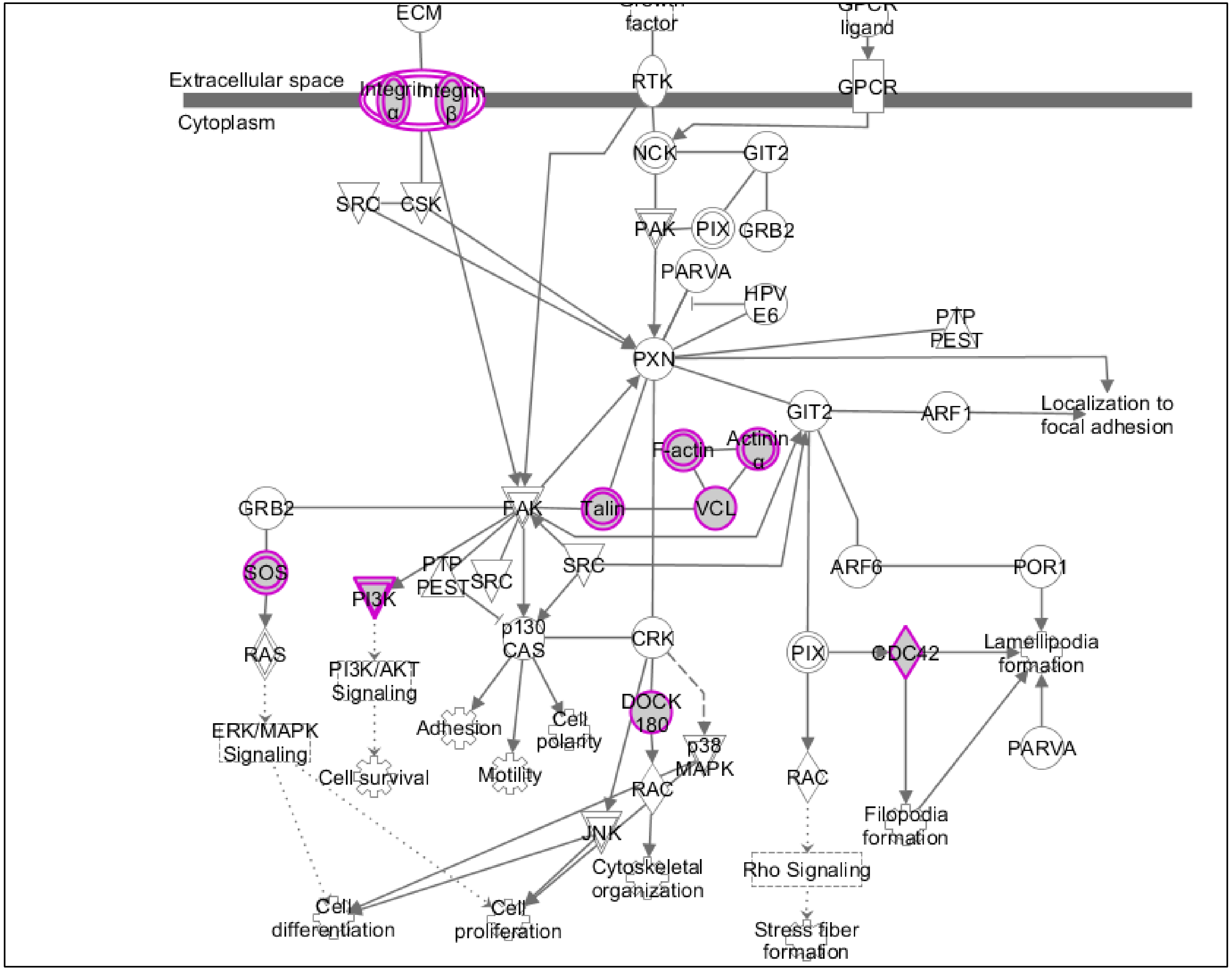

96-Acetyl-CoA Biosynthesis Ⅰ(Pyruvate Dehydrogenase Complex)

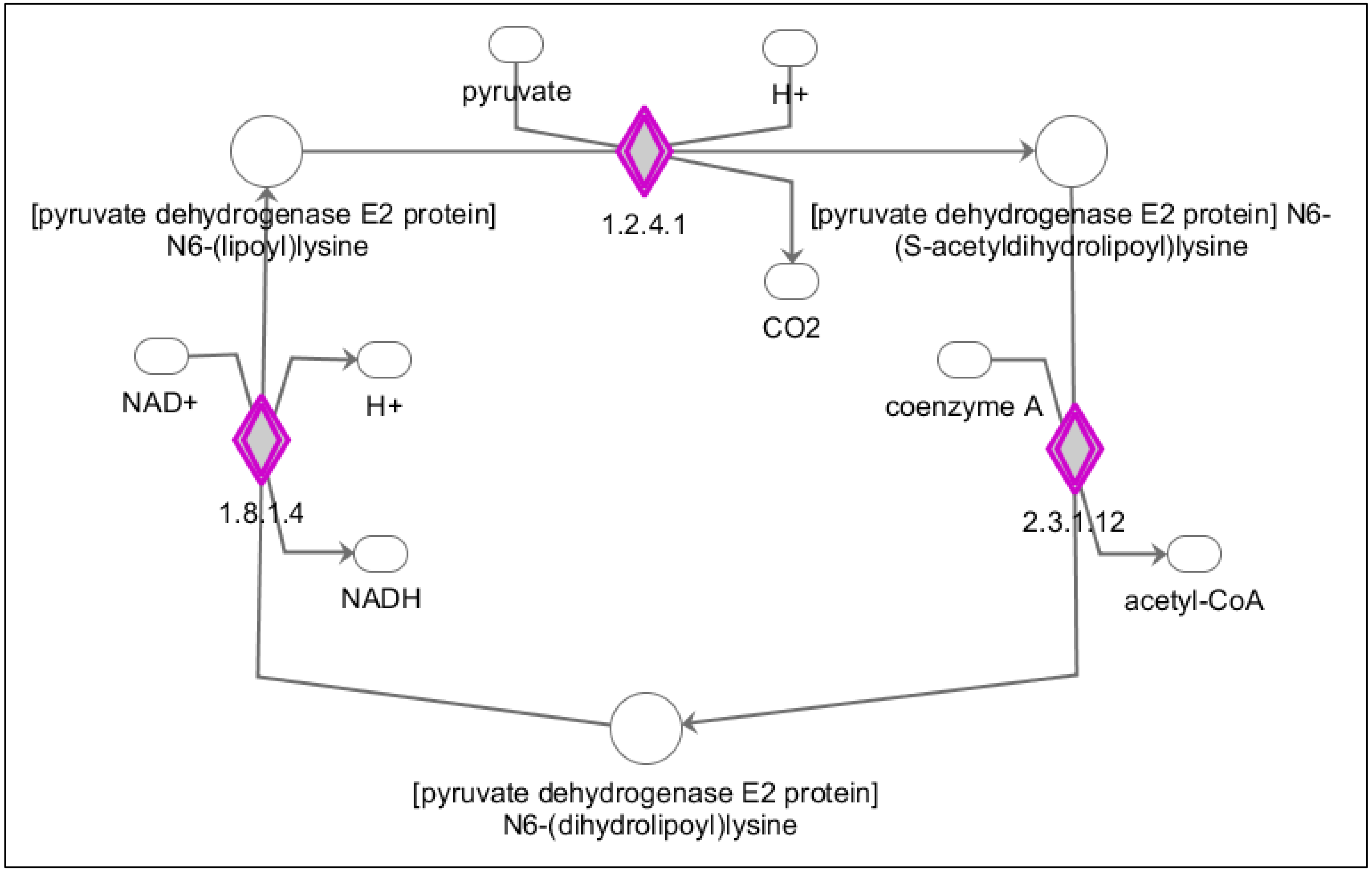

97-RAN Signaling

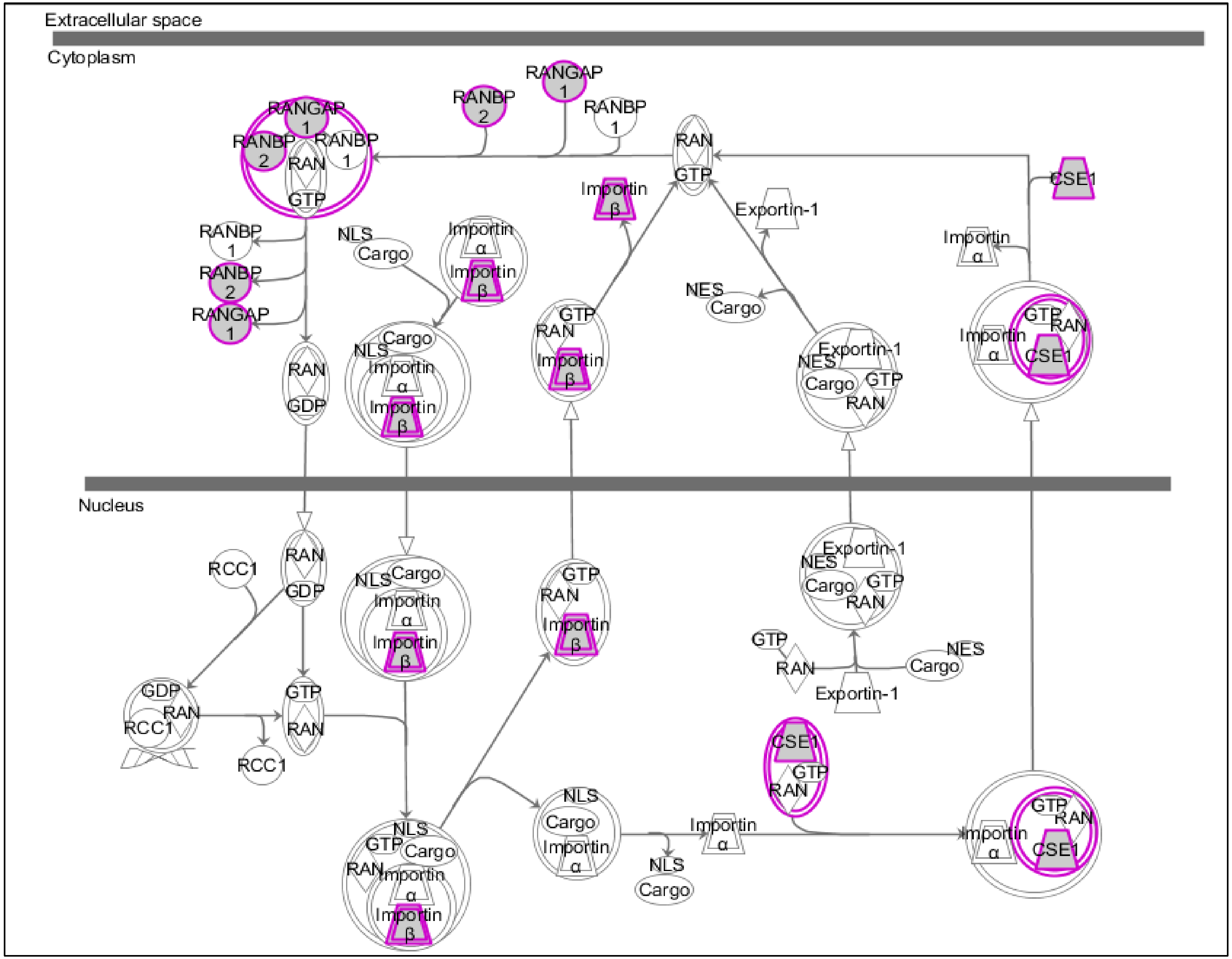

98-RAR Activation

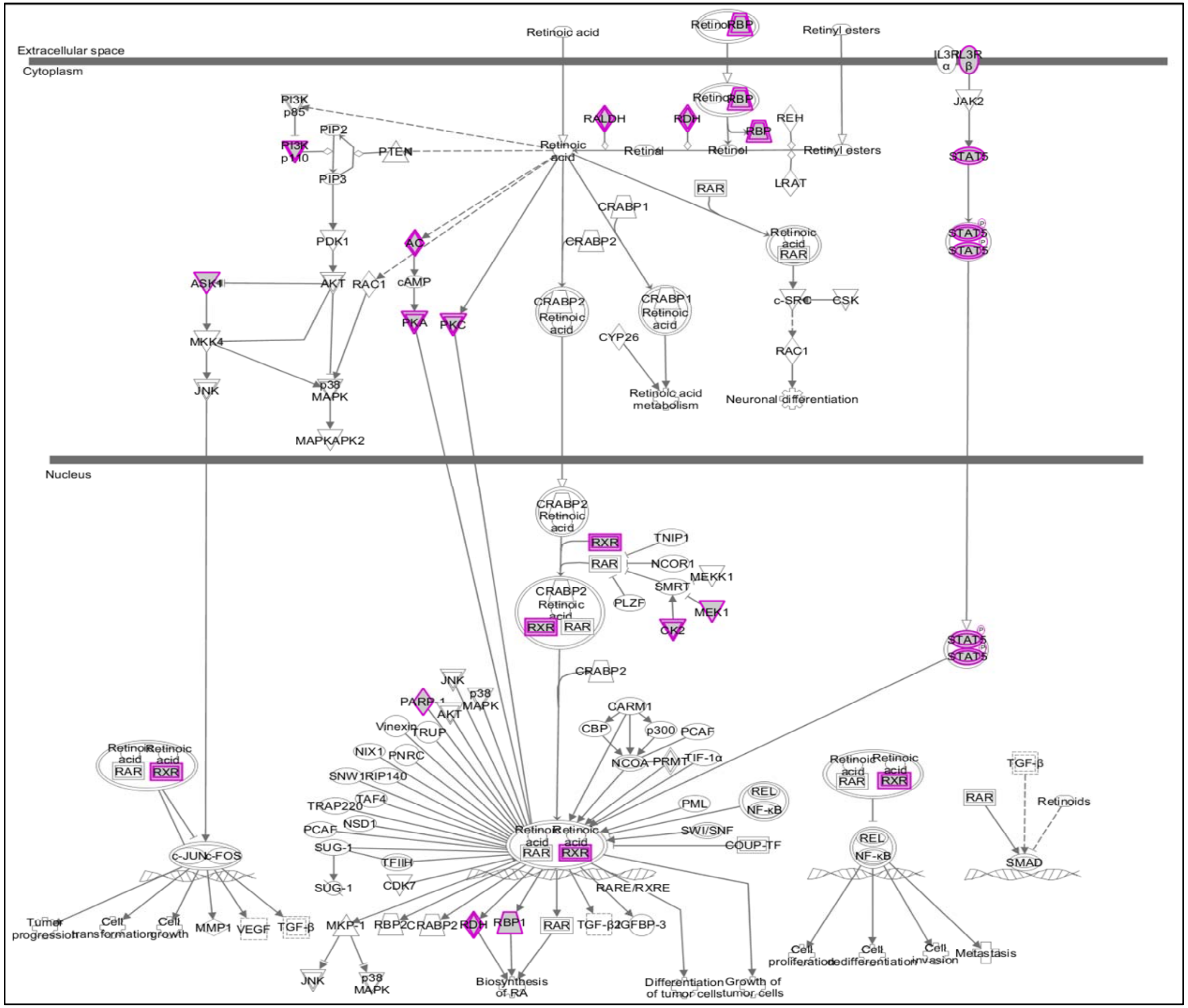

99-CXCR4 Signaling

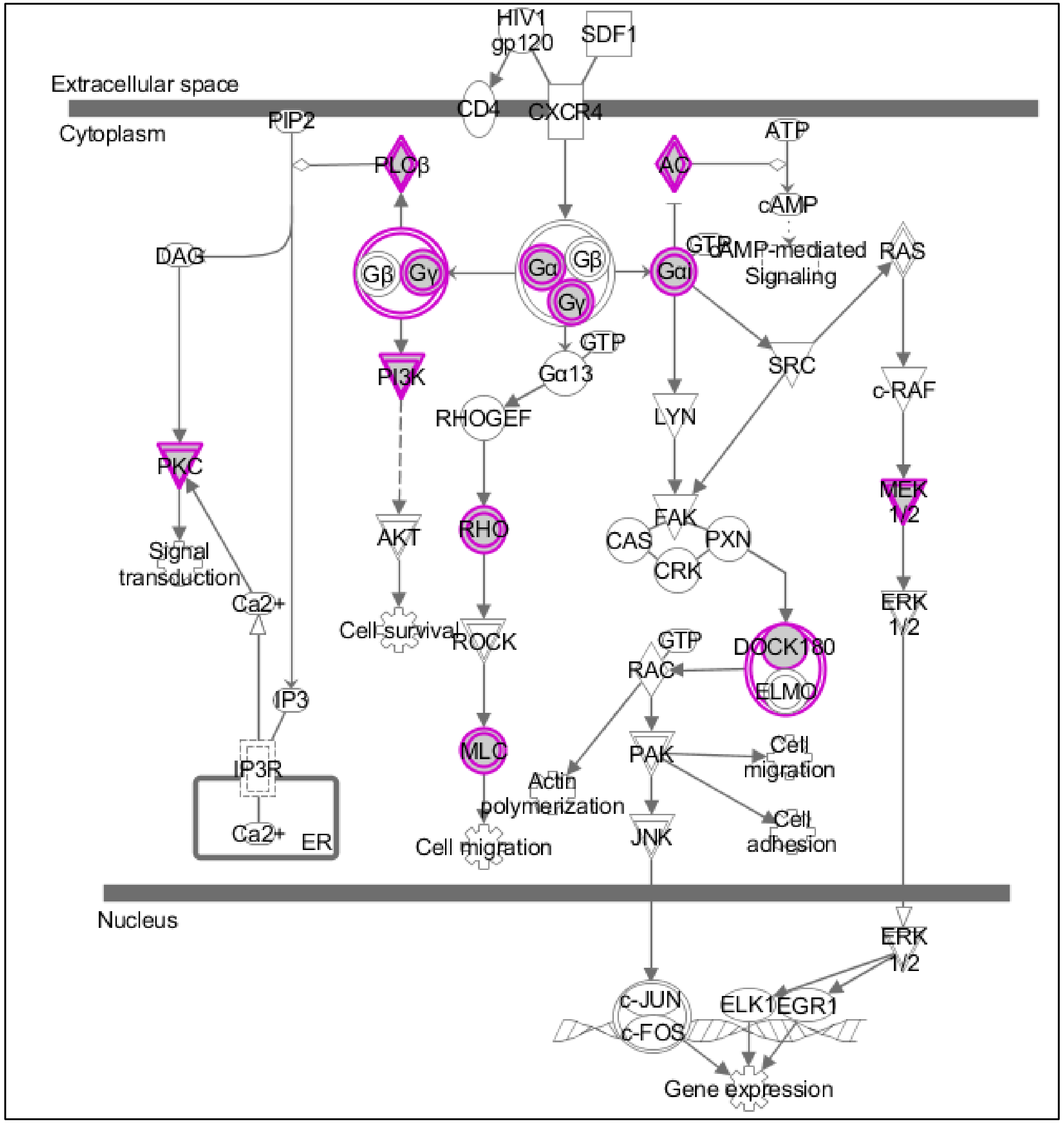

100-Histamine Degradation

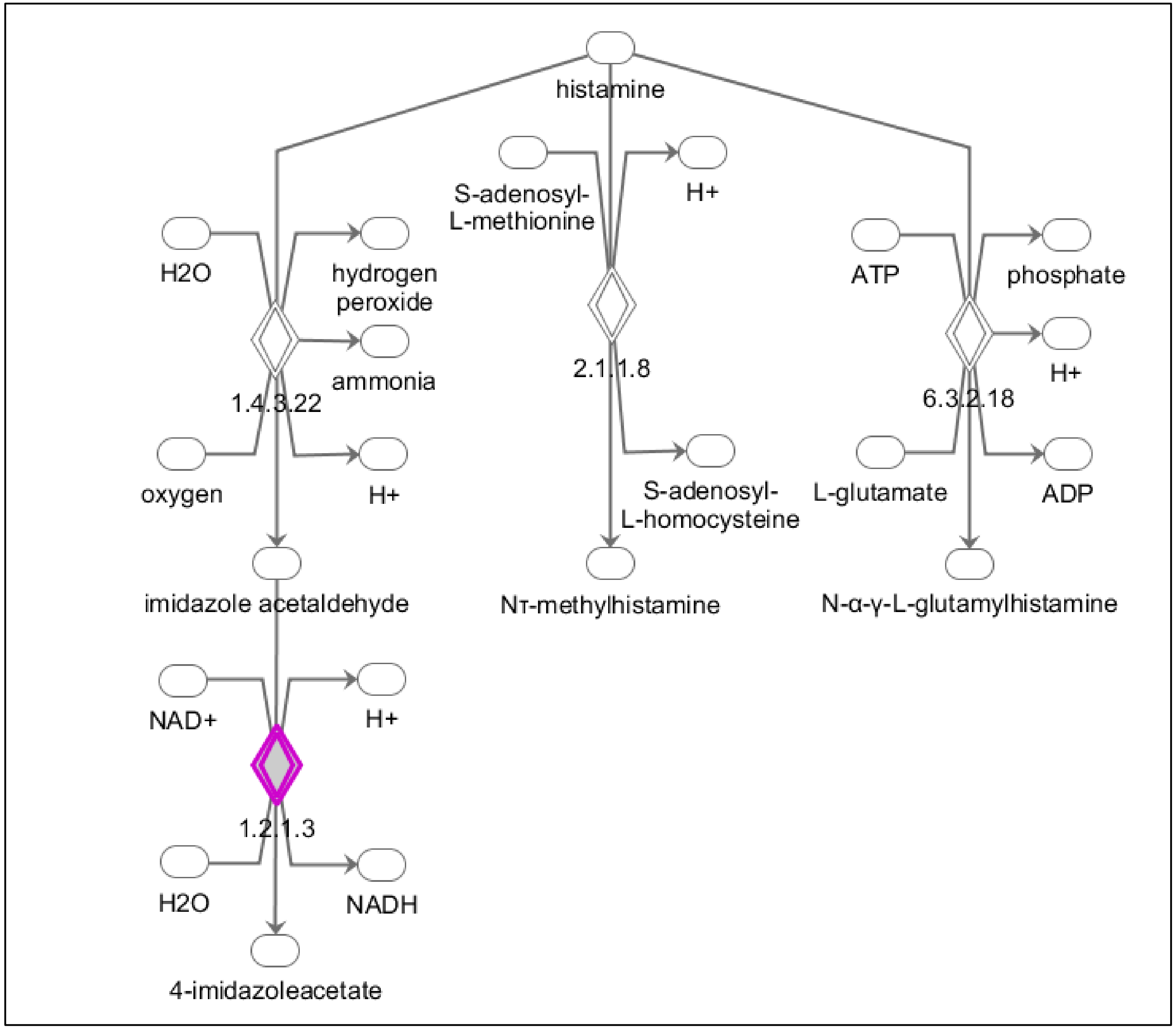

101-IL-12 Signaling and Production in Macrophages

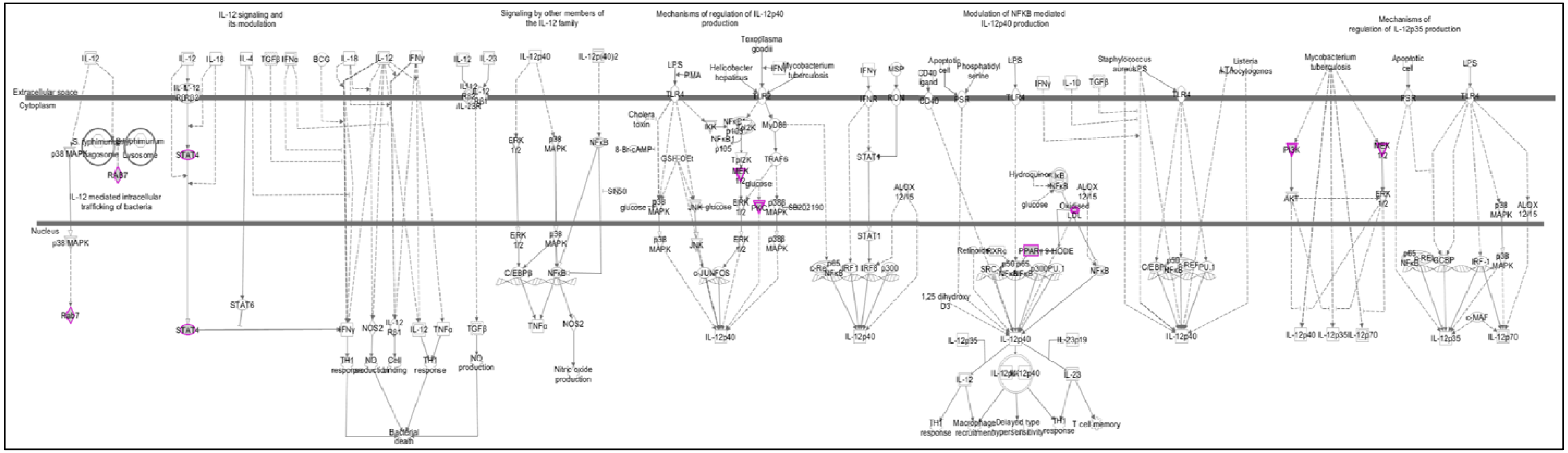

102-Production of Nitric Oxide and Reactive Oxygen Species in Macrophages

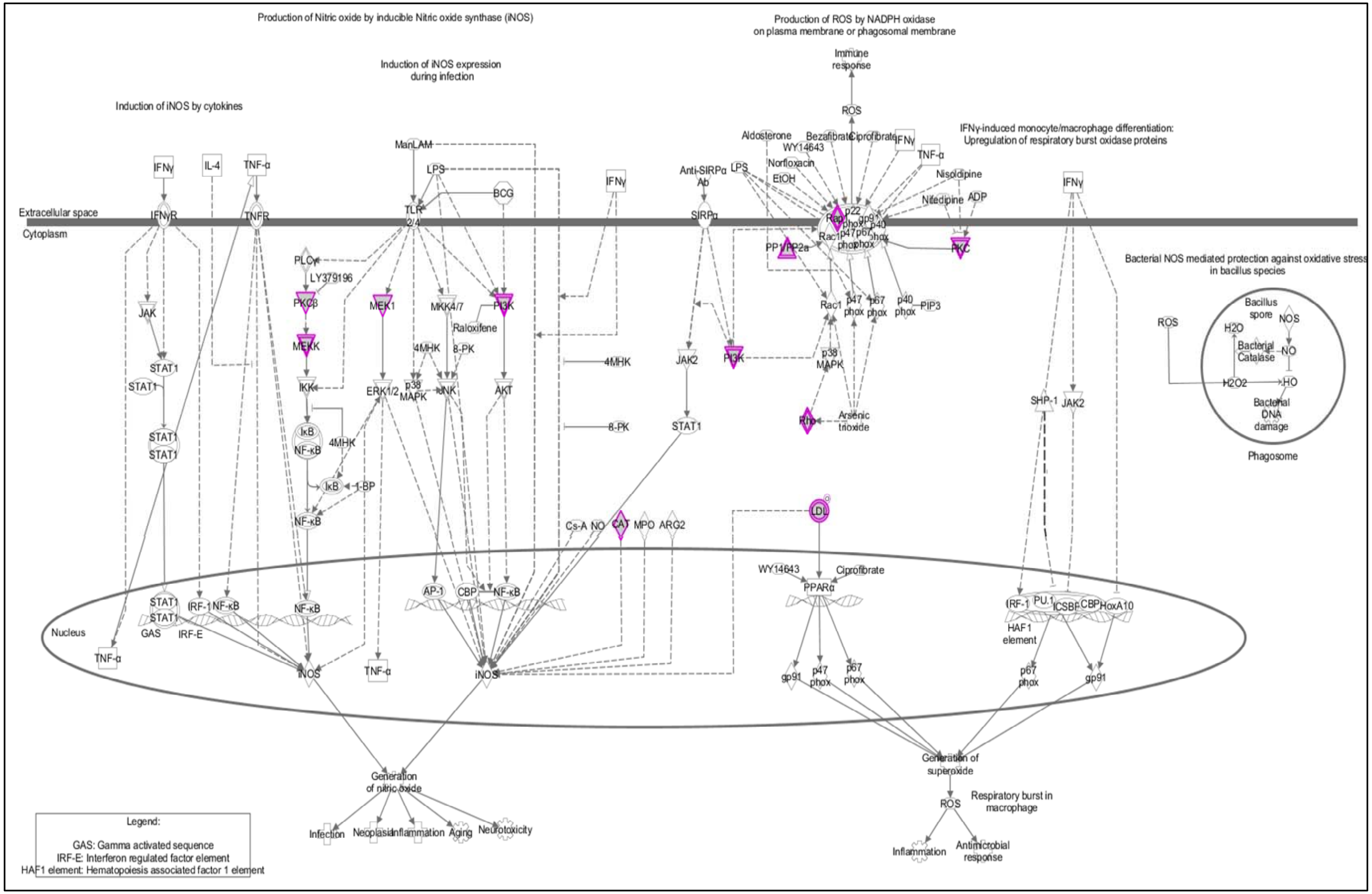

103-DNA Methylation and Transcriptional Repression Signaling

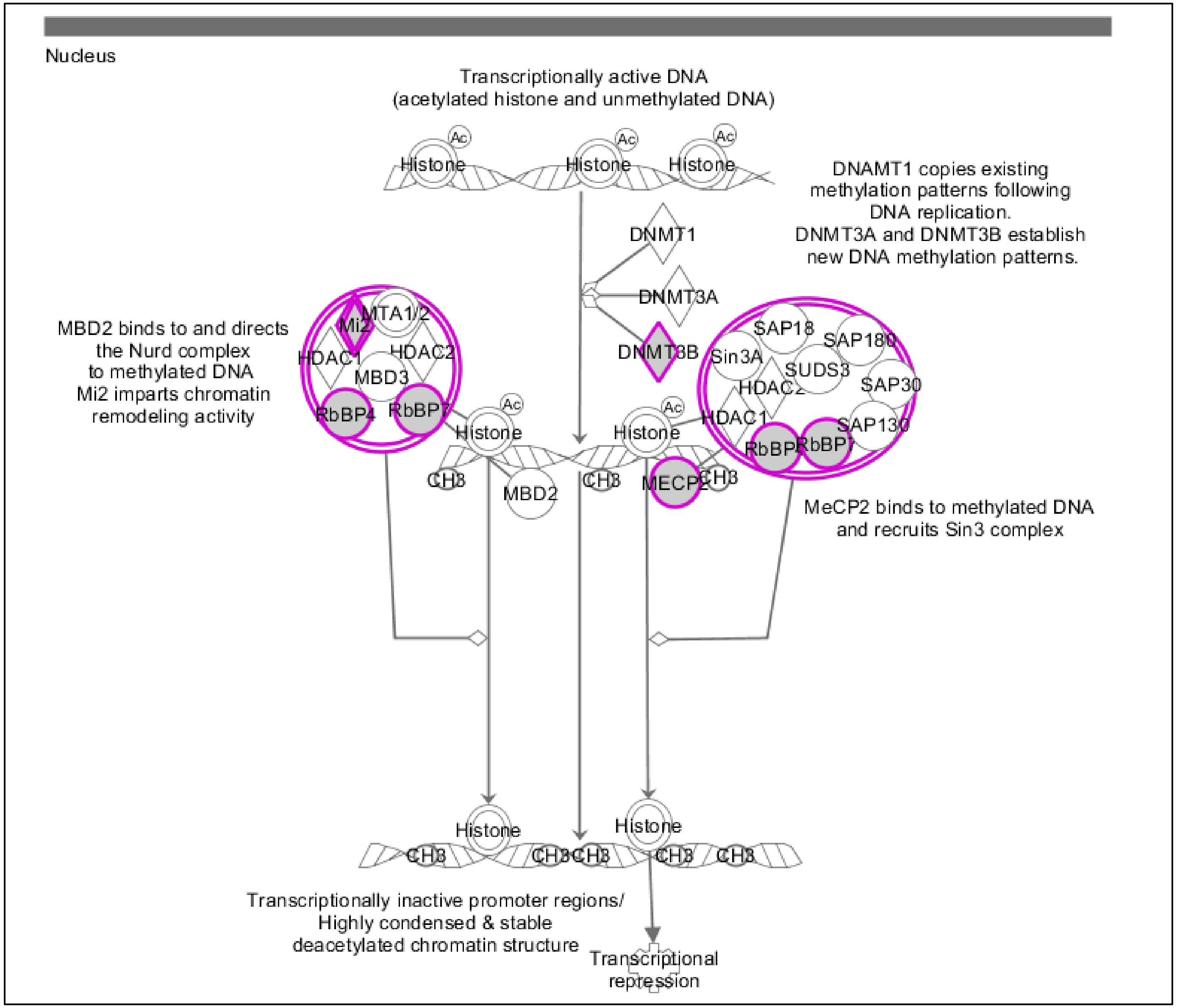

104-BER Pathway

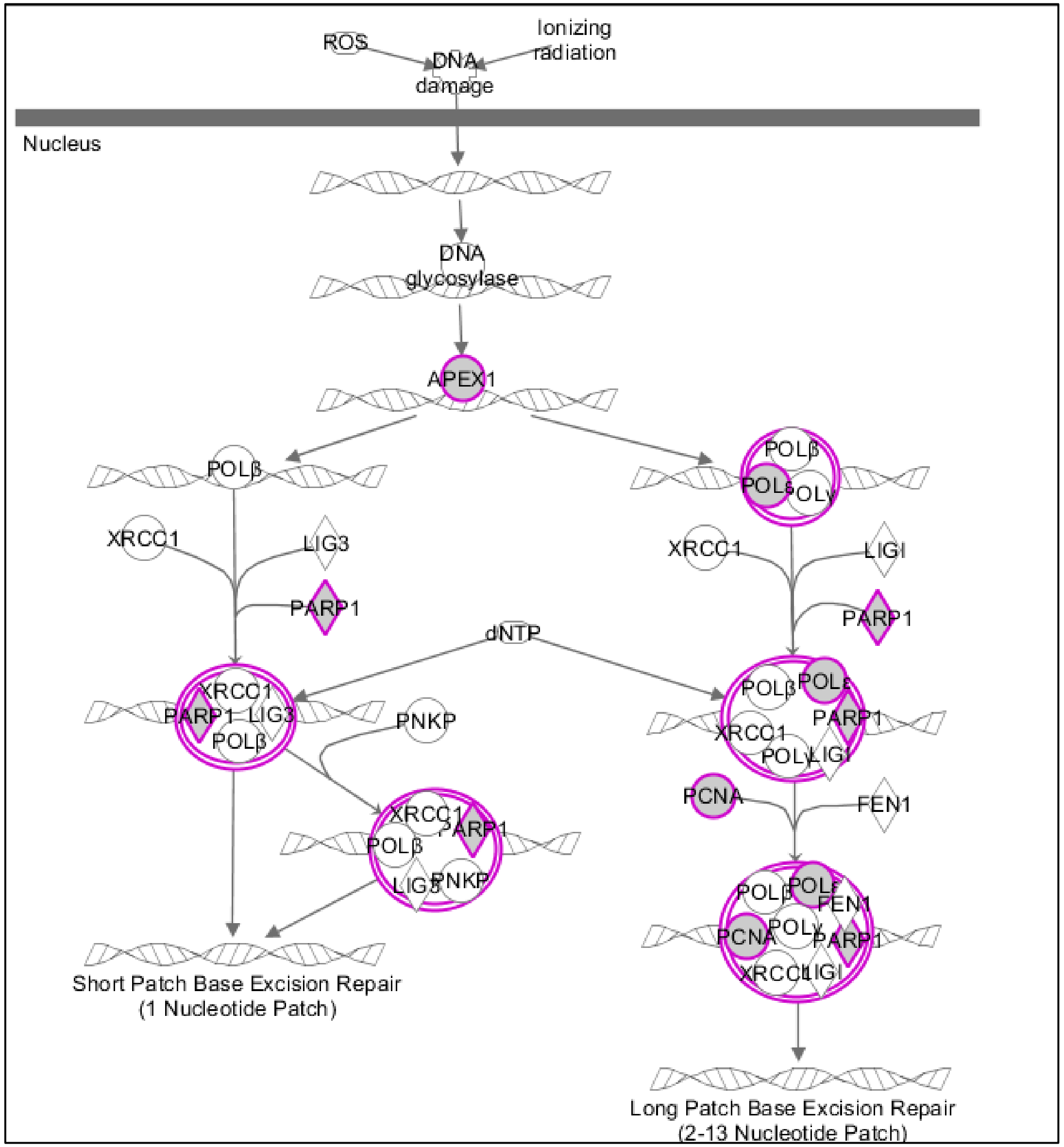

105-LPS-IL-1 Mediated Inhibition of RXR Function

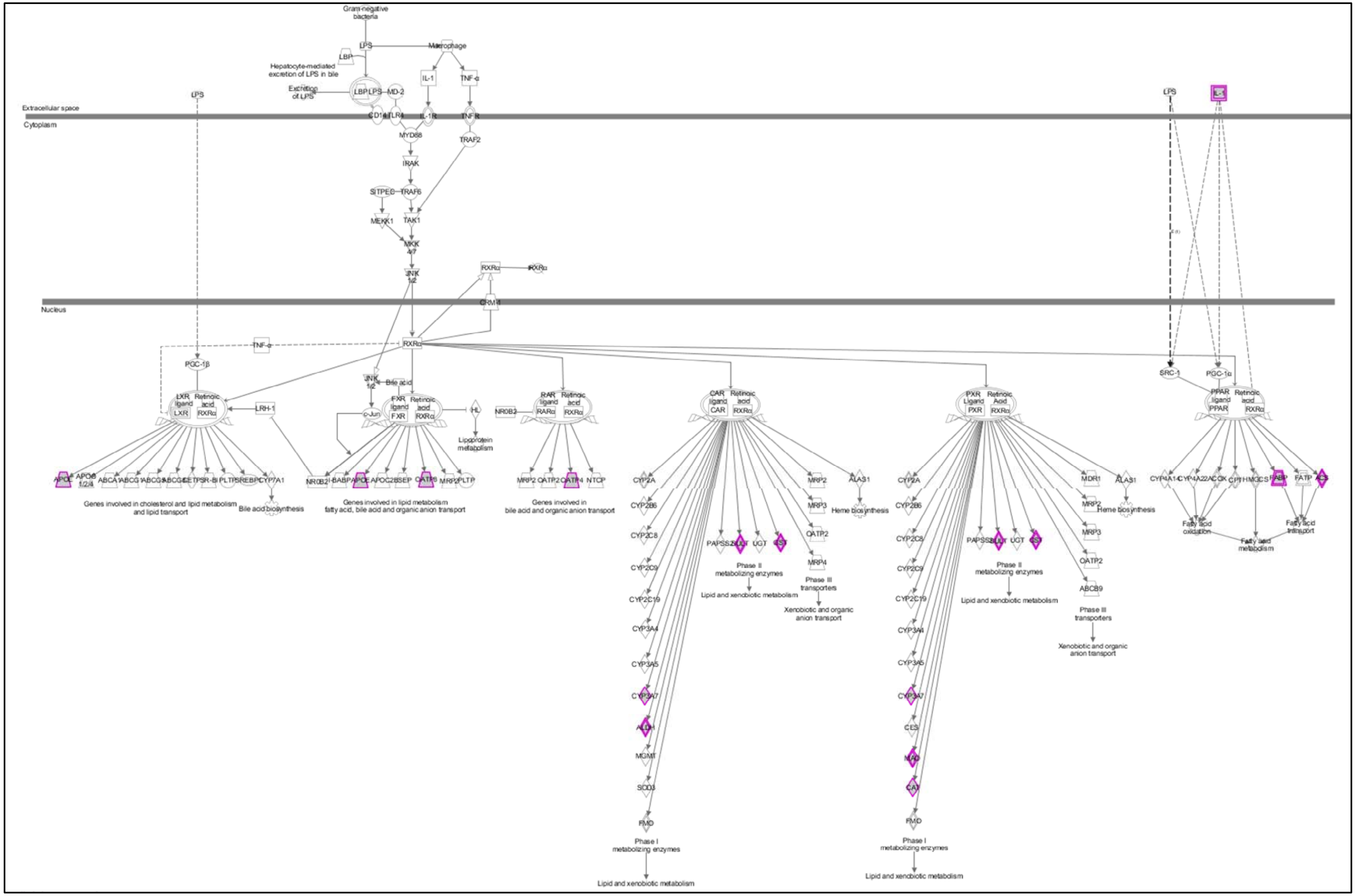

106-Cardiac β -adrenergic Signaling

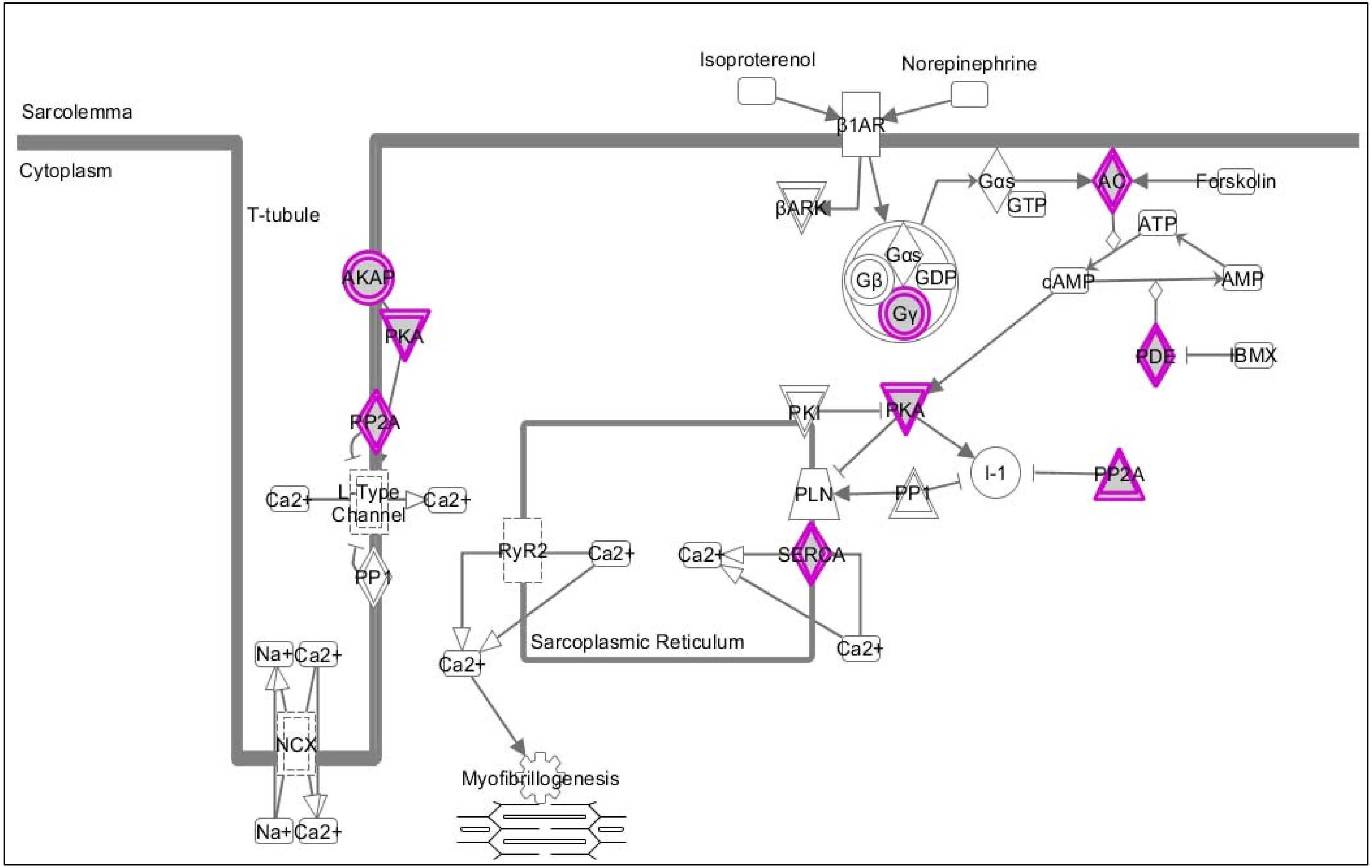

107-Melanocyte Development and Pigmentation Signaling

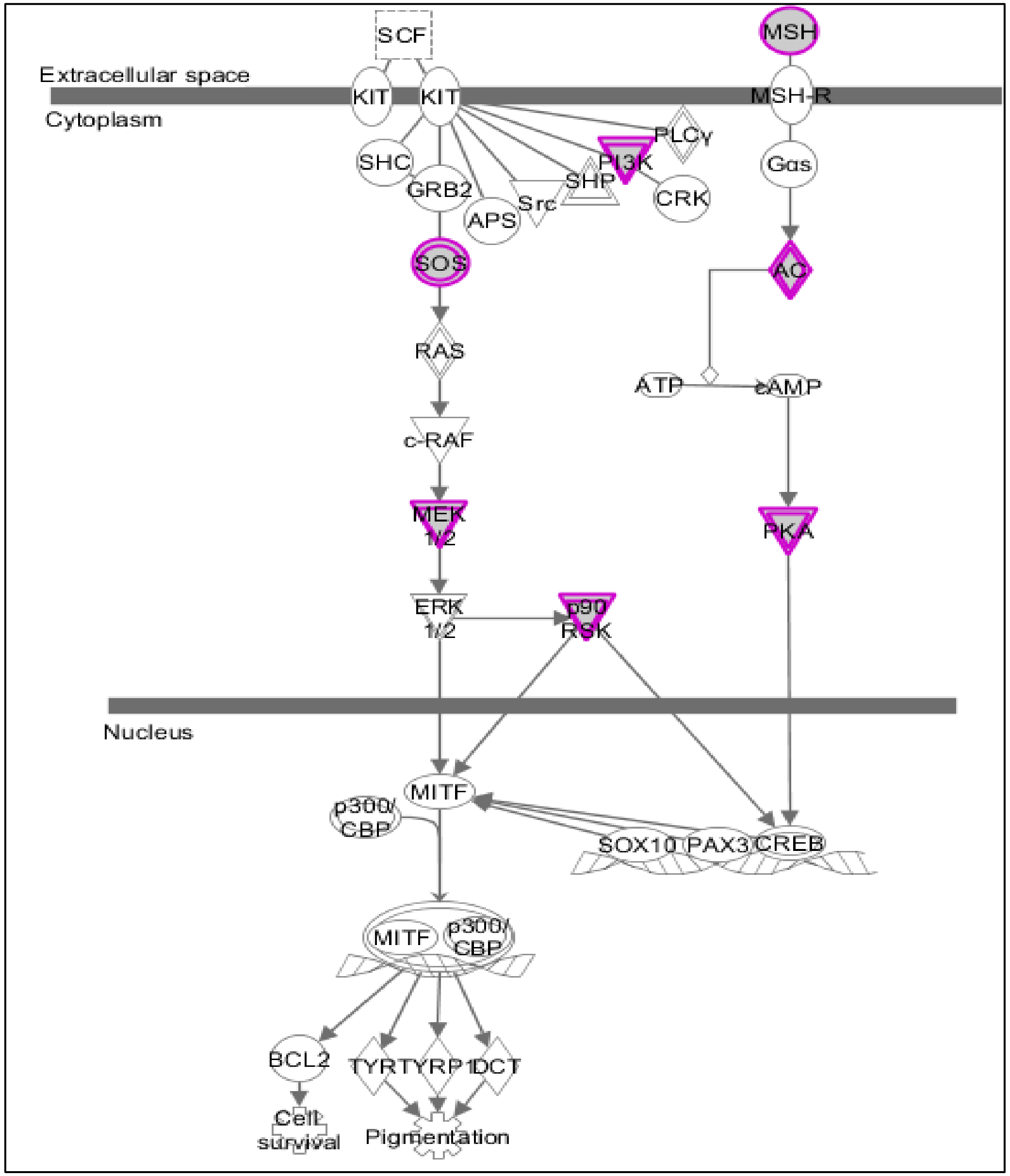

108-Prostate Cancer Signaling

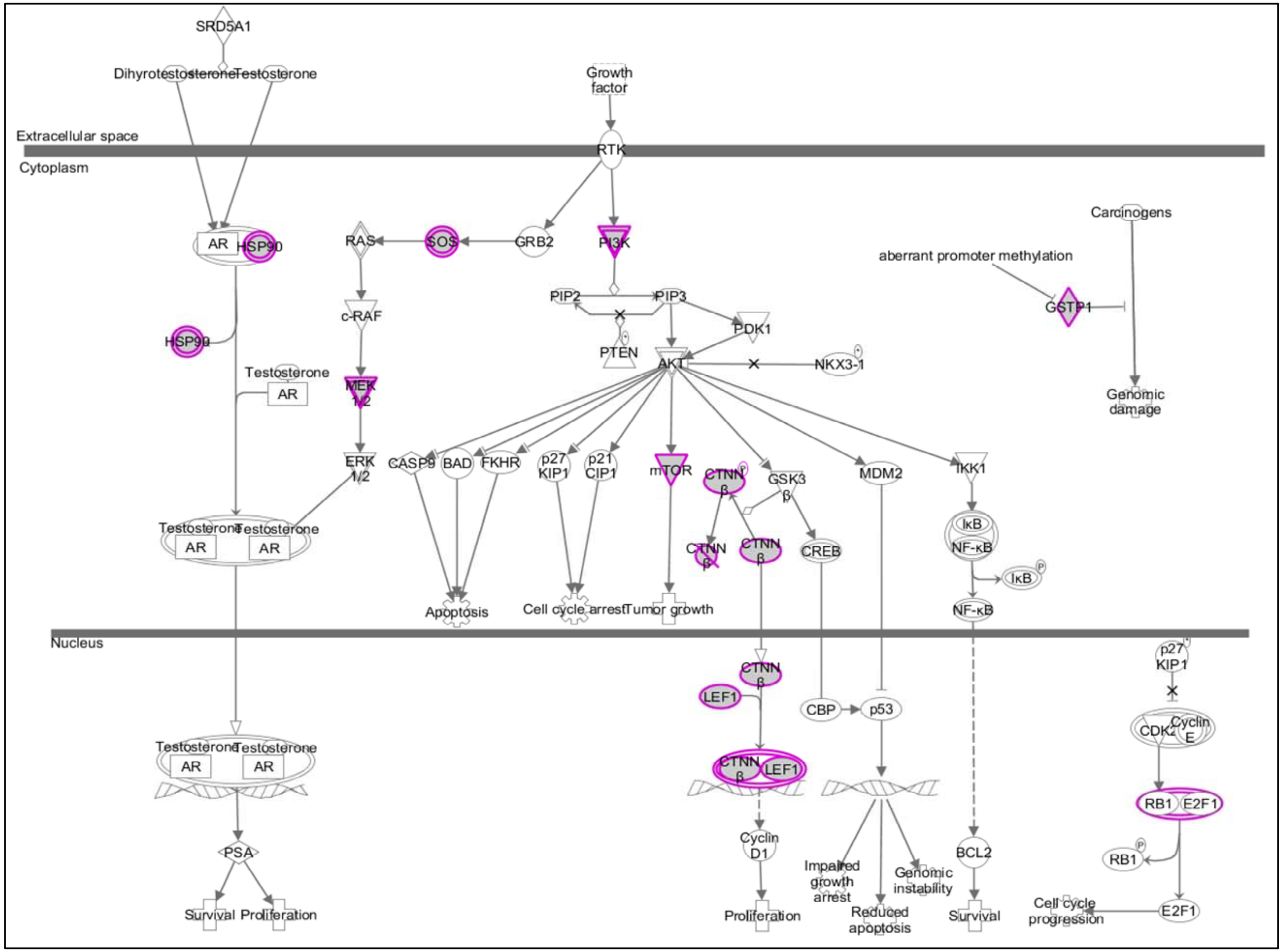

109- α -Adrenergic Signaling

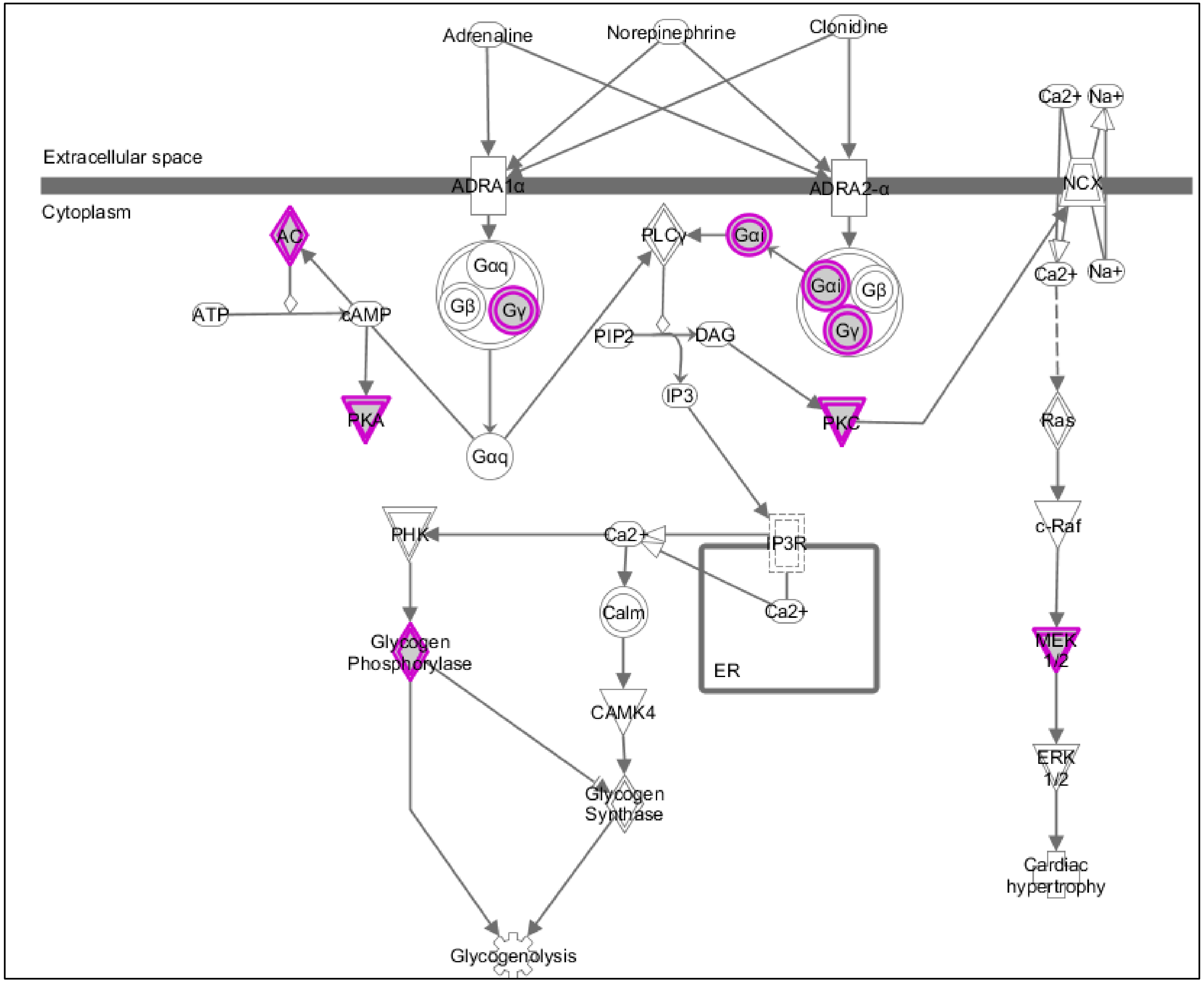

110-Melatonin Signaling

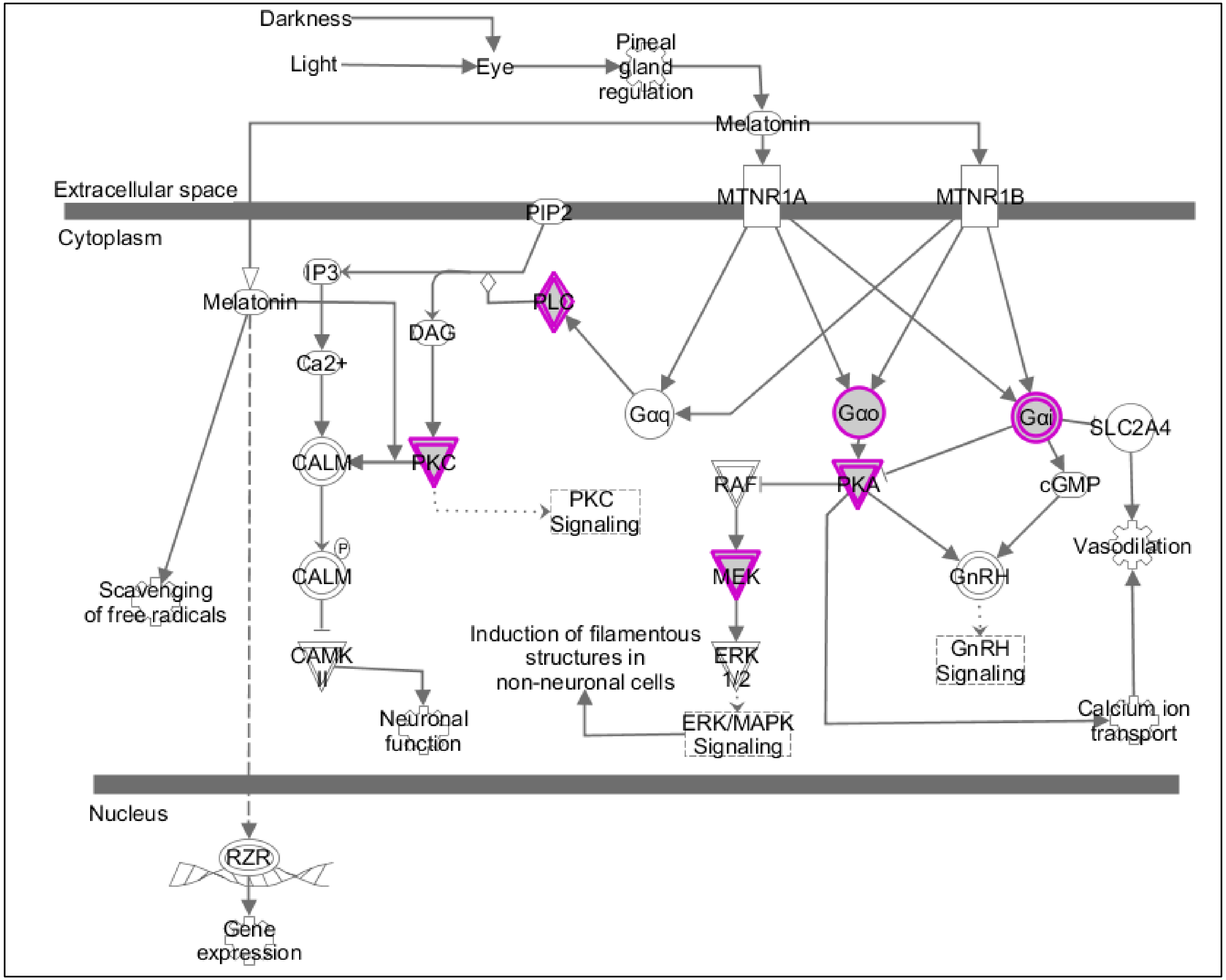

111-Mitotic Roles of Polo-like Kinase

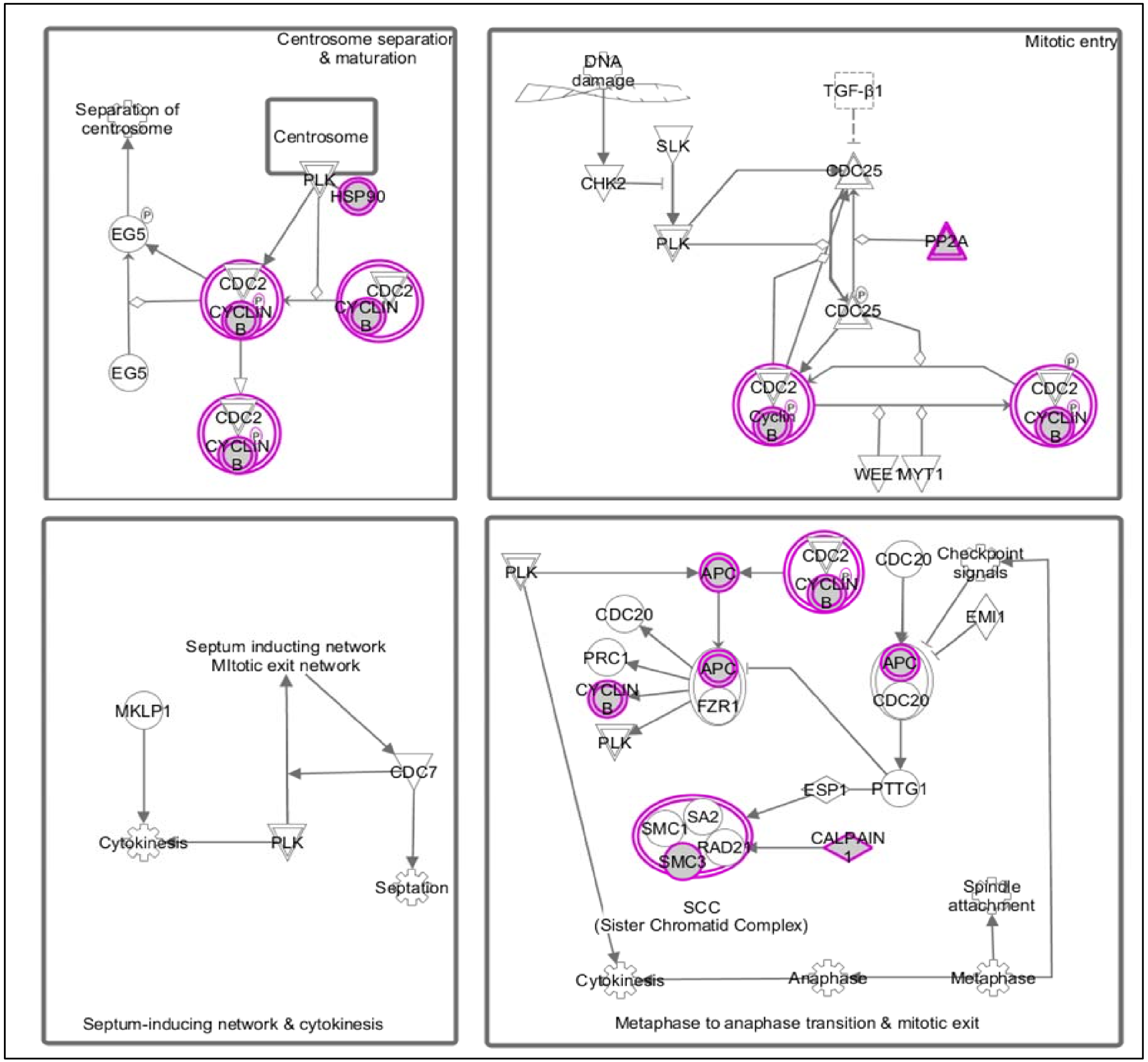

112-Hepatic Fibrosis-Hepatic Stellate Cell Activation

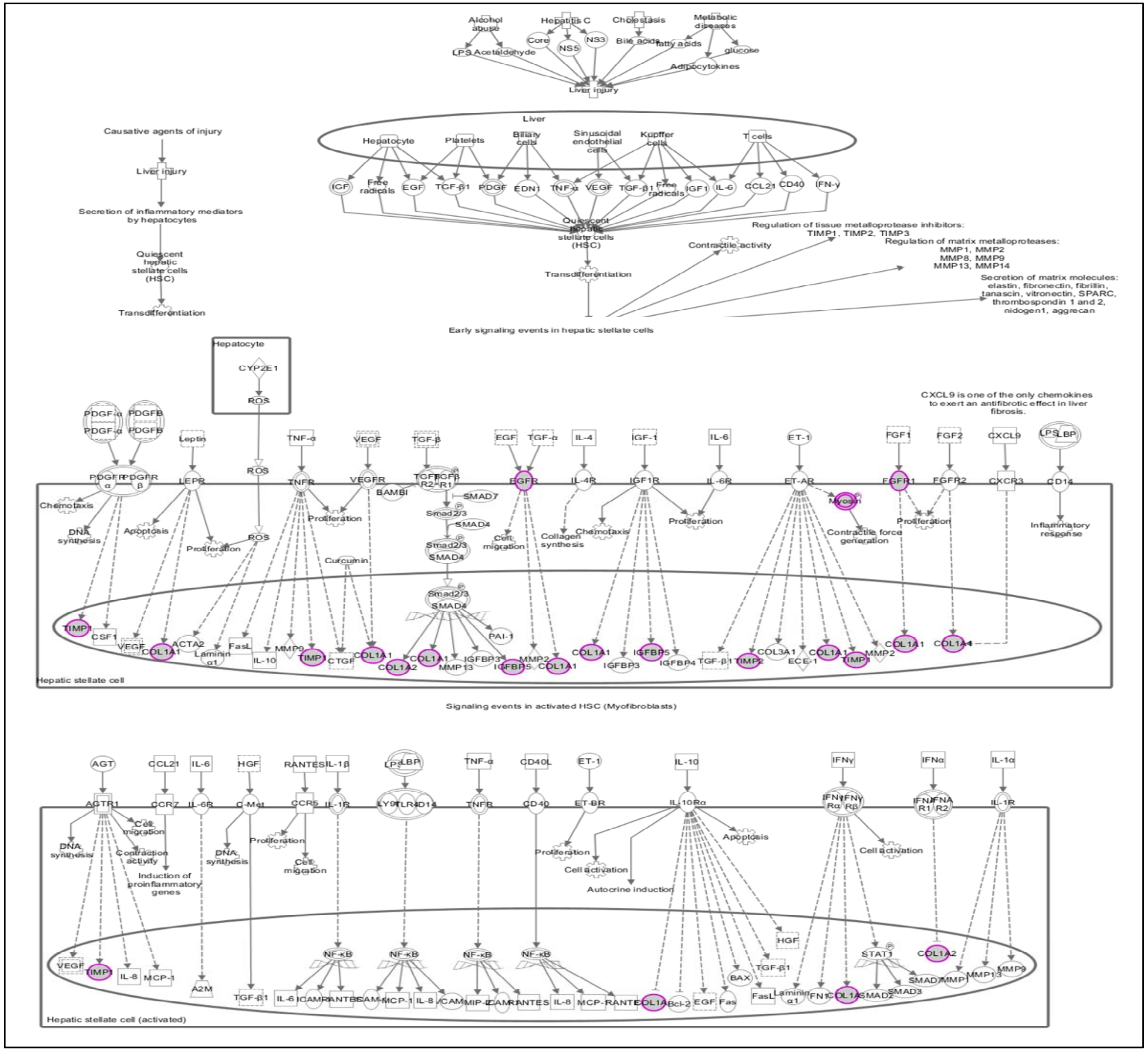

113-Corticotropin Releasing Hormone Signaling

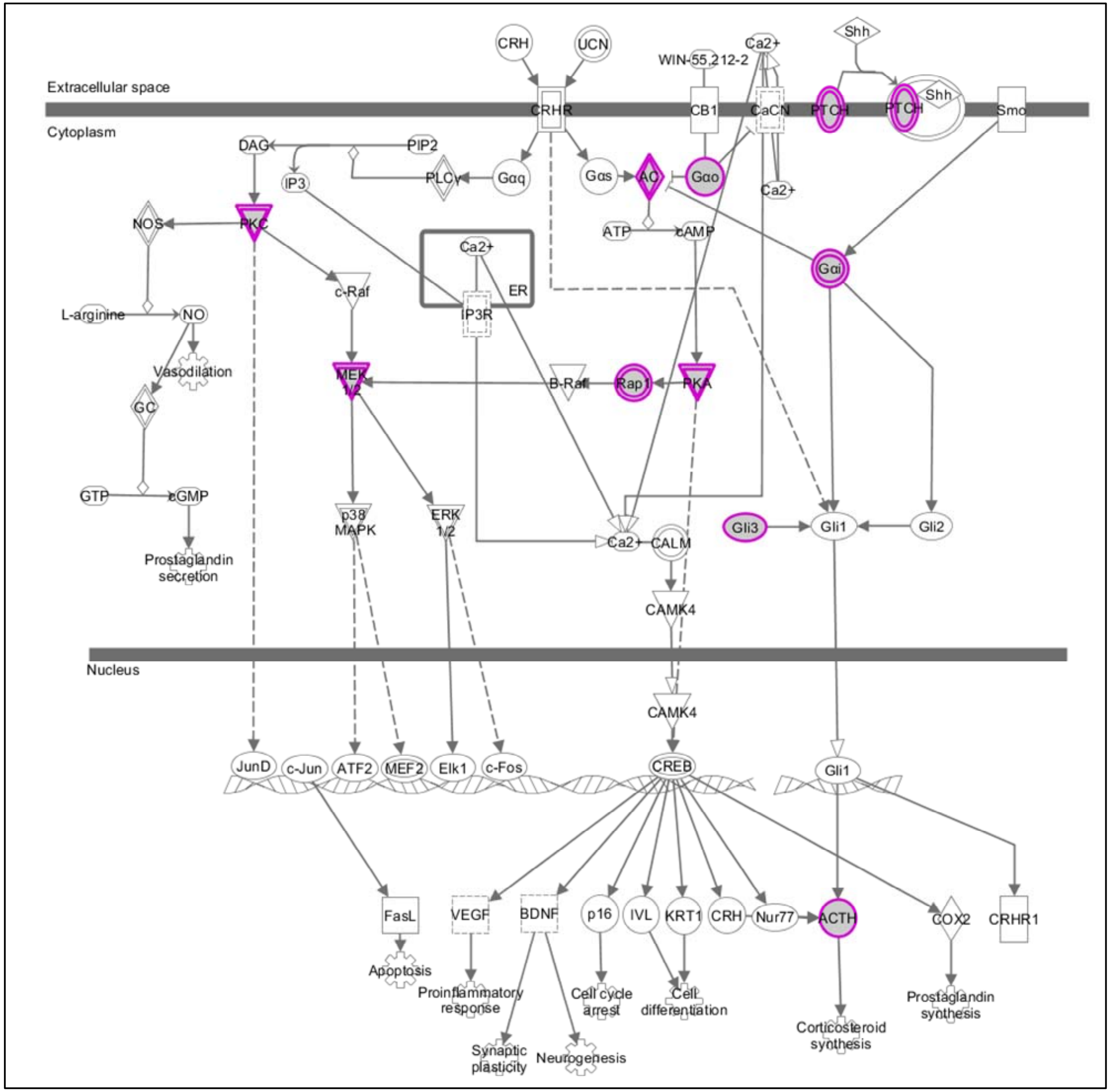

114-IGF-1-Signaling

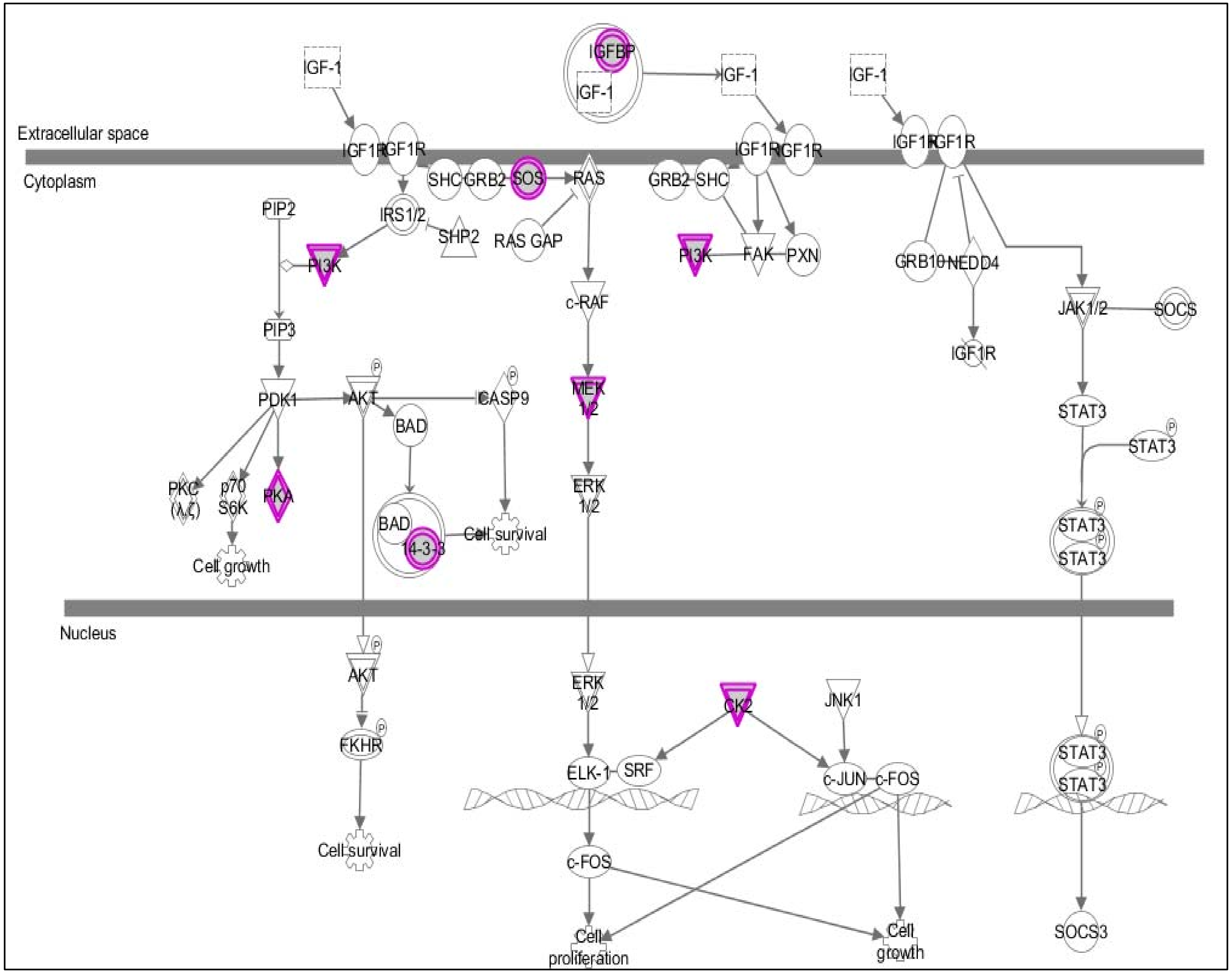

115-Aspartate Degradation Ⅱ

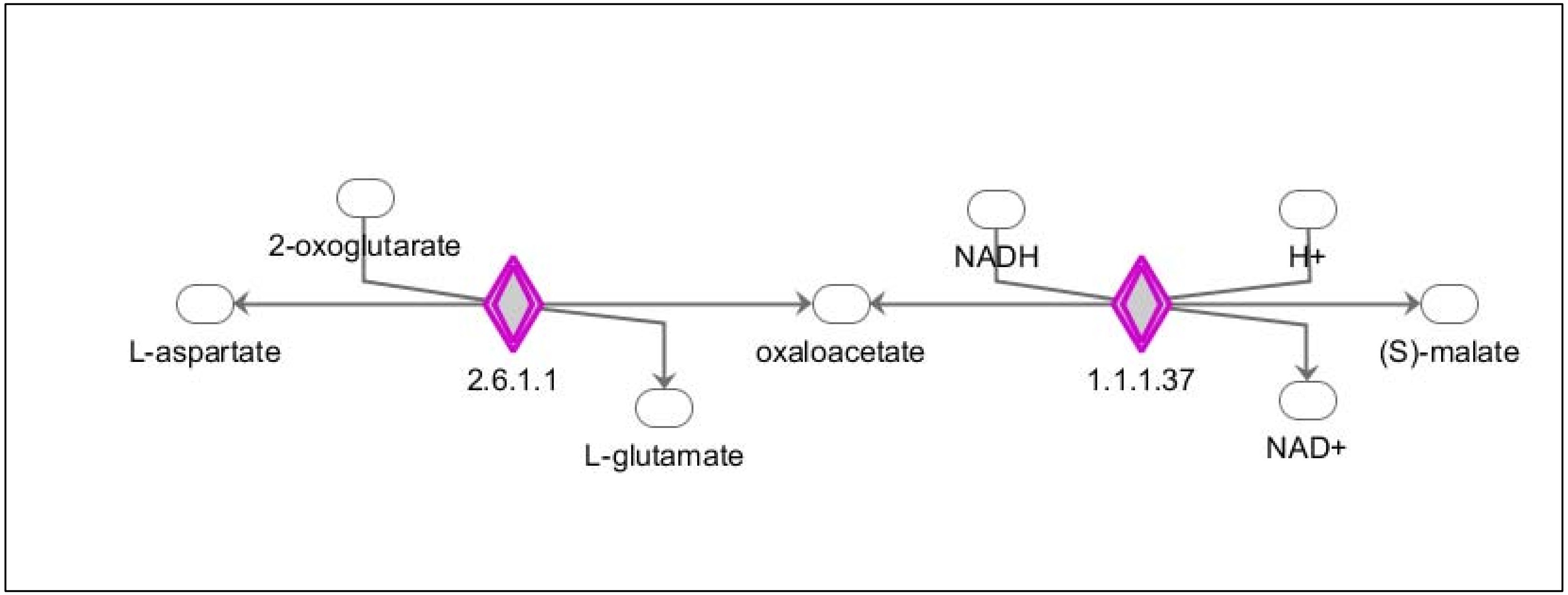

116-Glutathione-mediated Detoxification

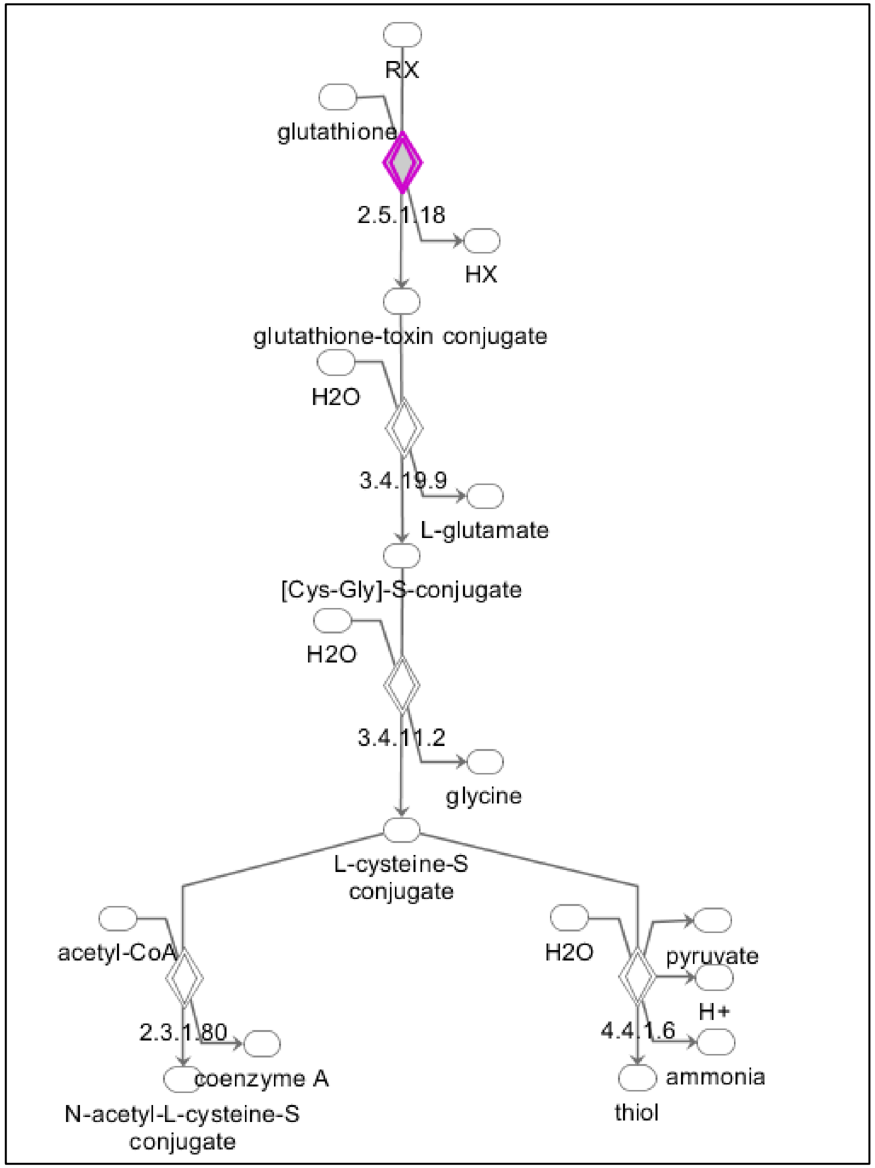

117-Superoxide Radicals Degradation

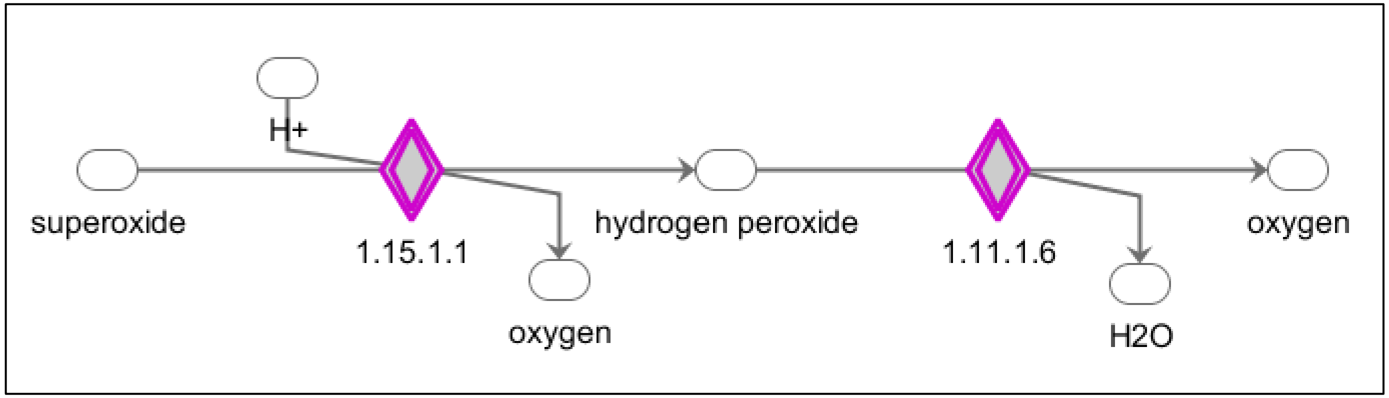

118-Inhibition of Matrix Metalloproteases

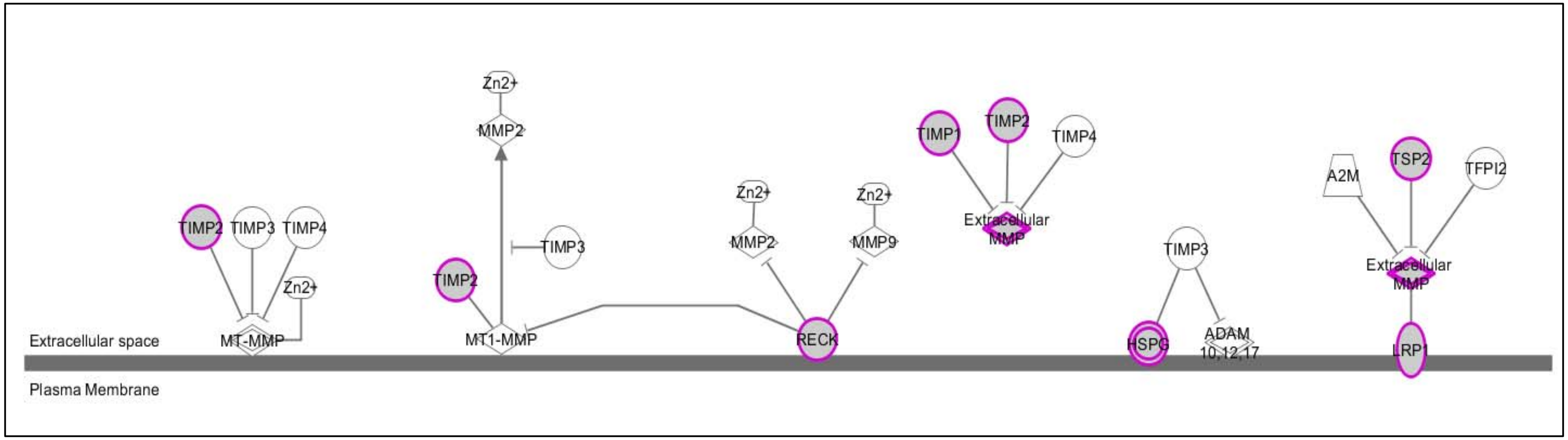

119-Pentose Phosphate Pathway

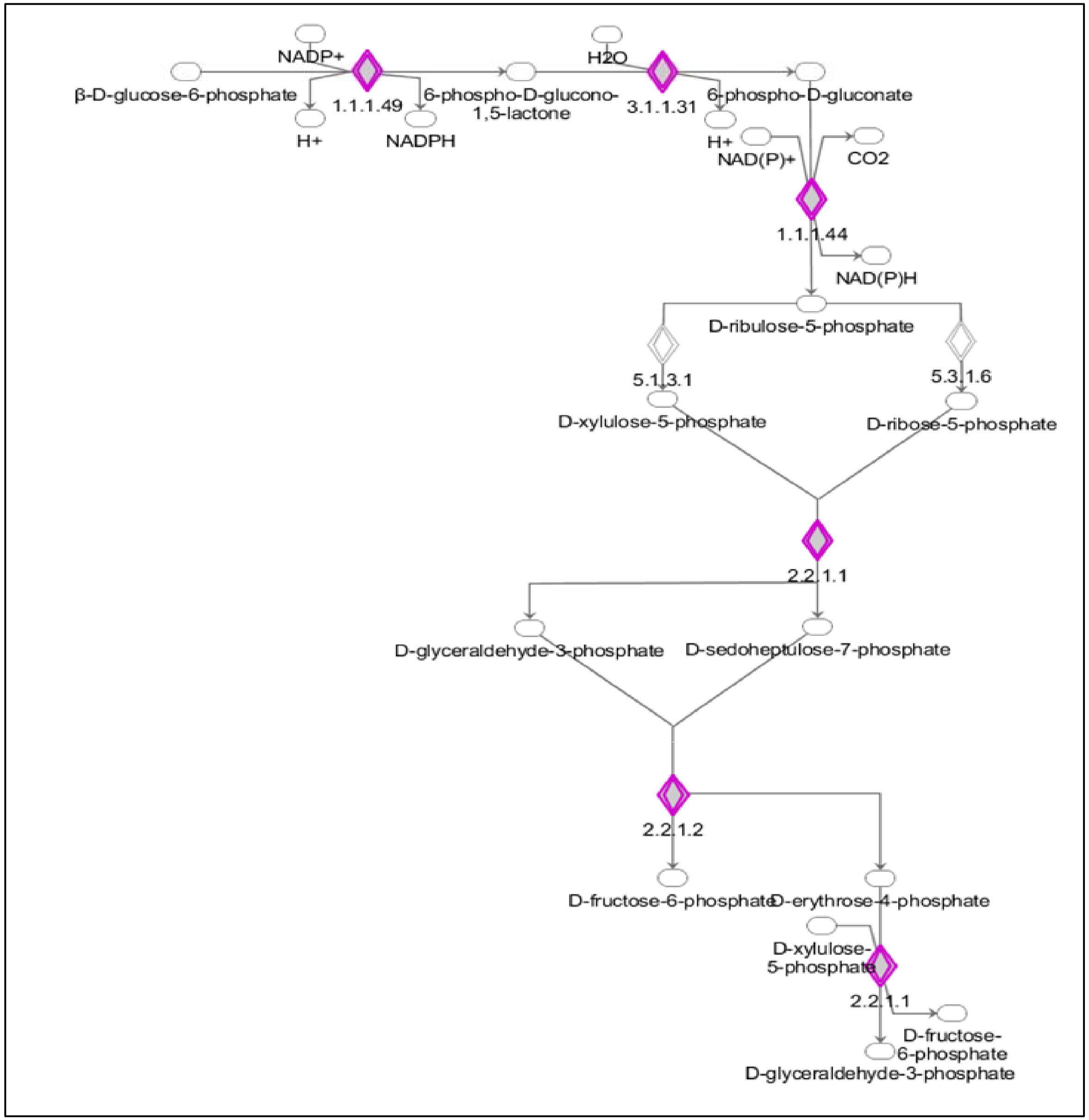

120-Calcium Signaling

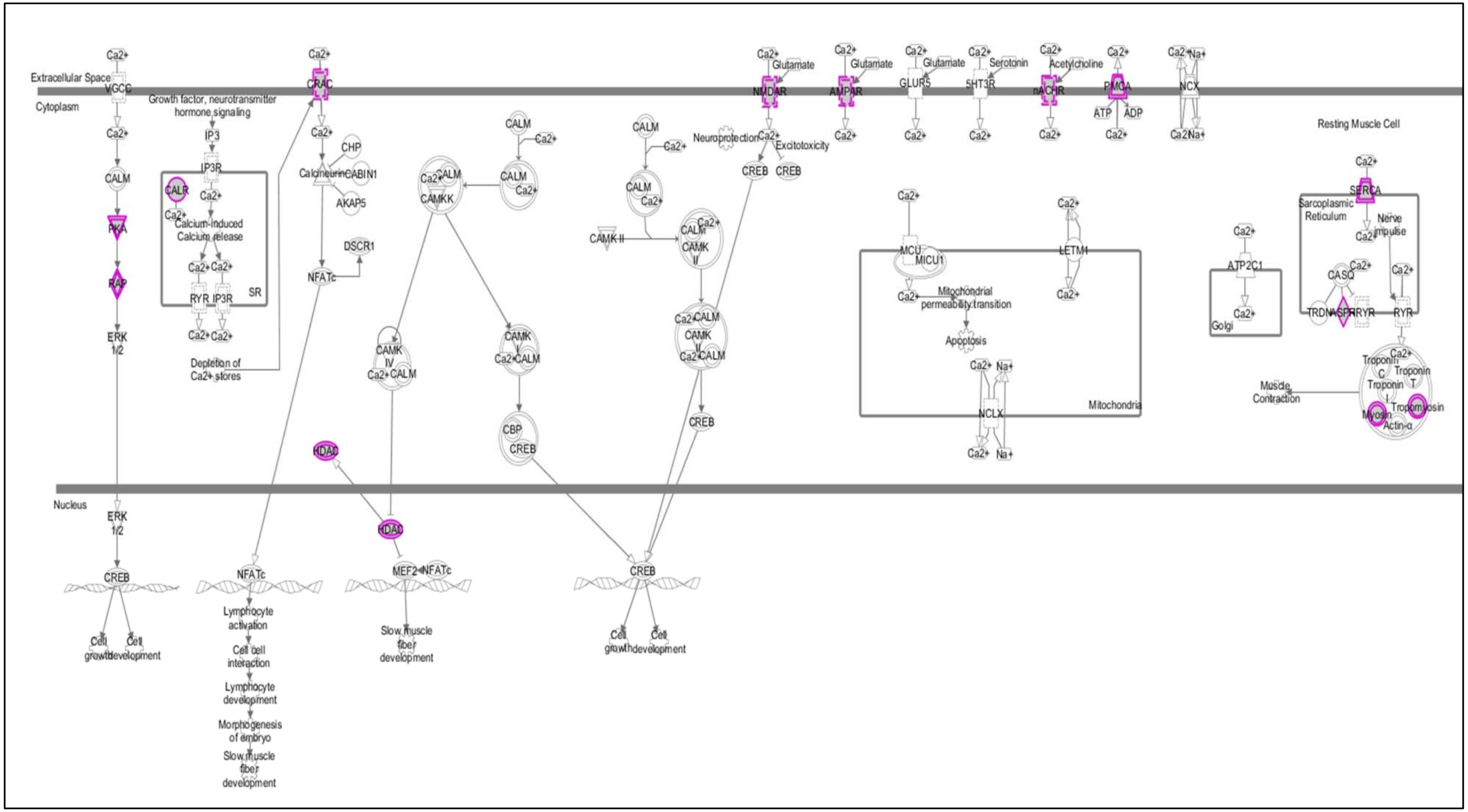

121-PXR/RXR Activation

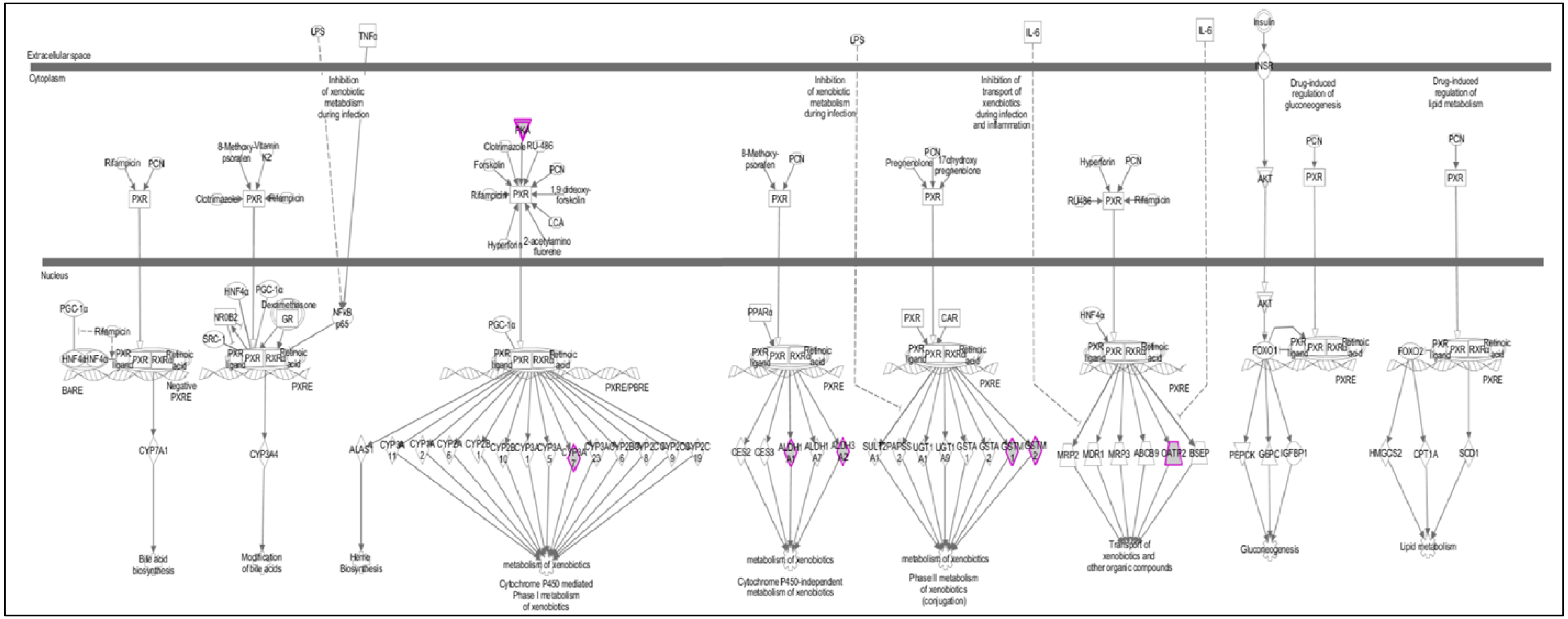

122-Amyloid Processing

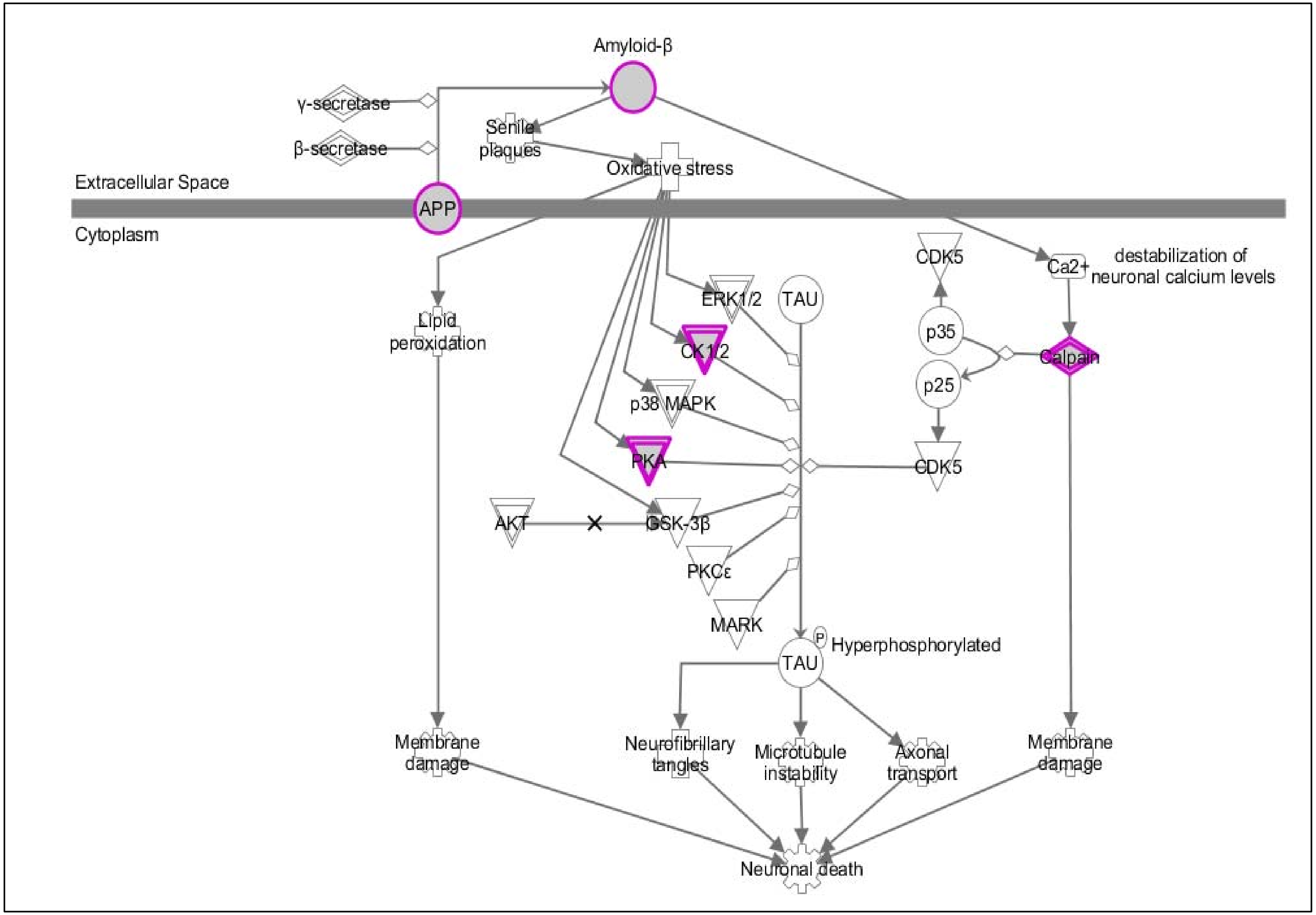

123-Granzyme B Signaling

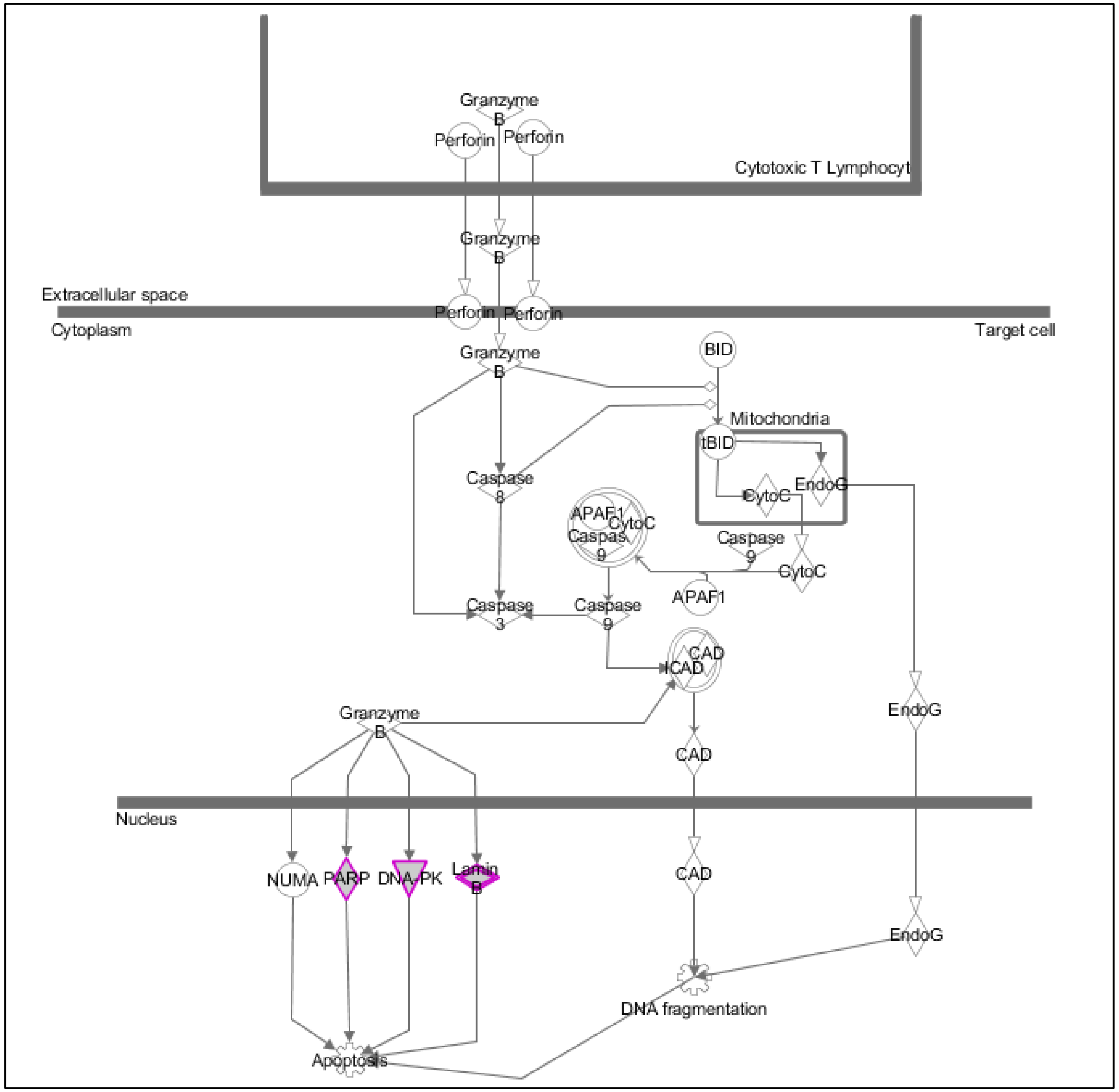

124-P2Y Purigenic Receptor Signaling Pahway

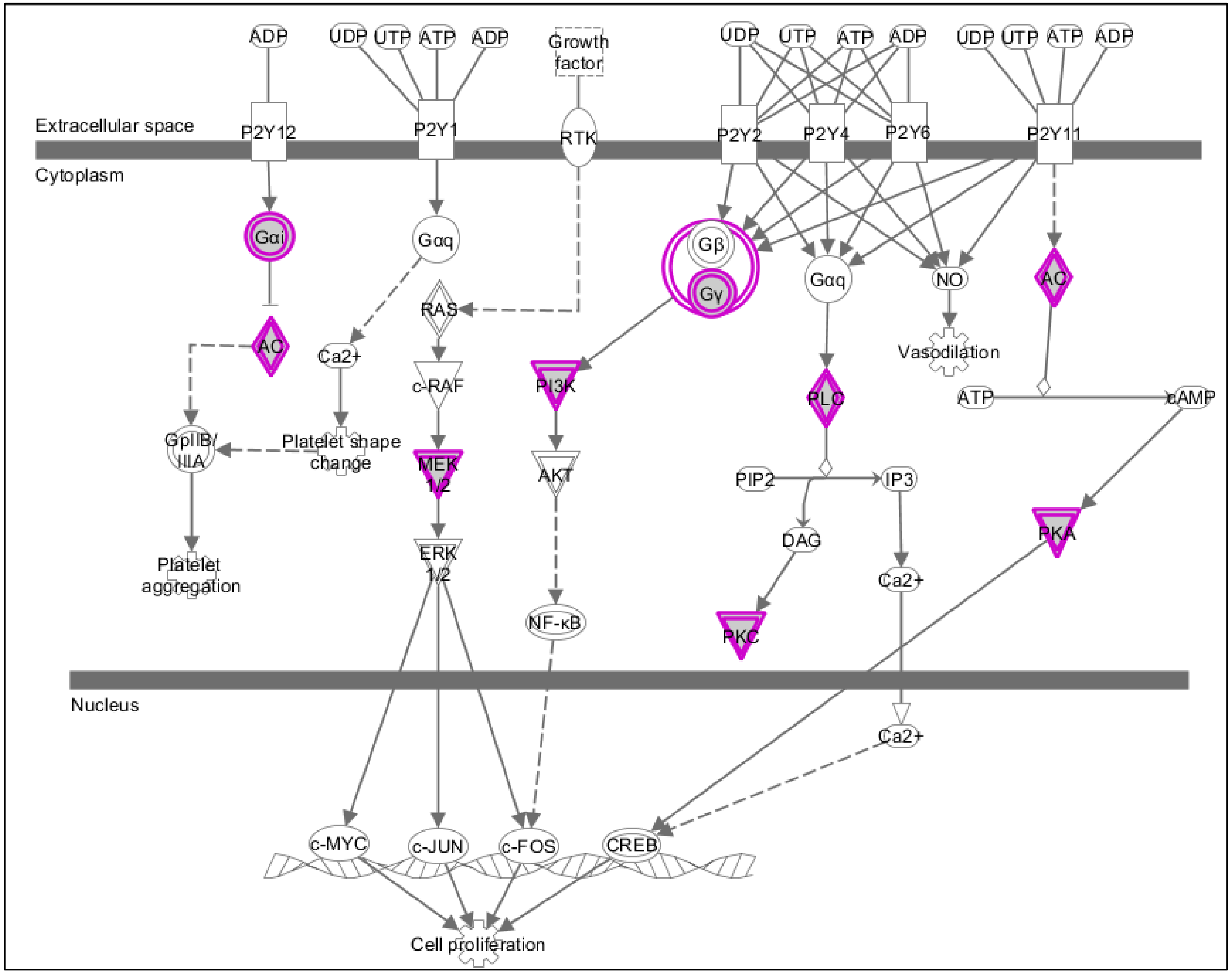

125-2-ketoglutarate Dehydrogenase Complex

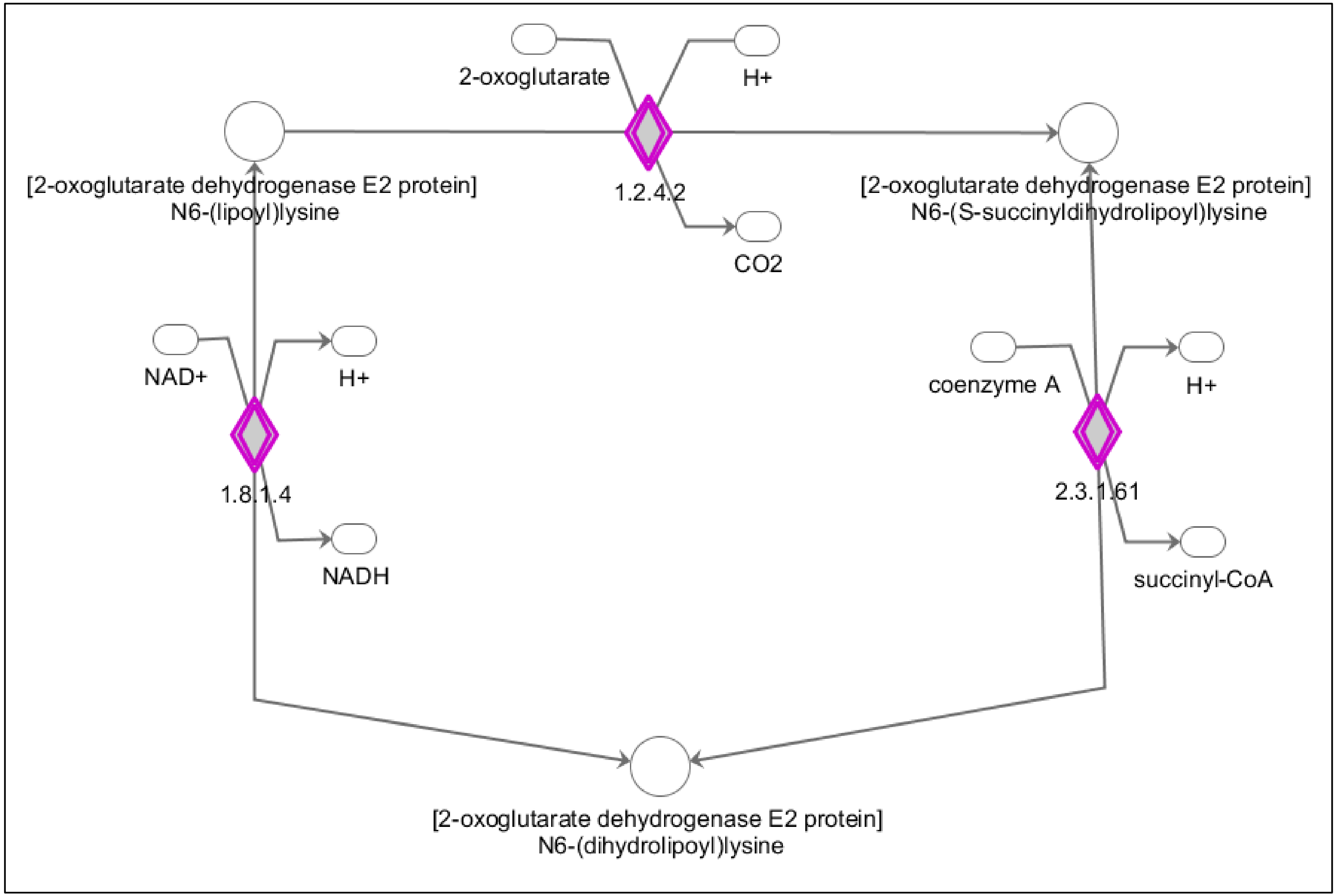

126-Amyotrophic Lateral Sclerosis Signaling

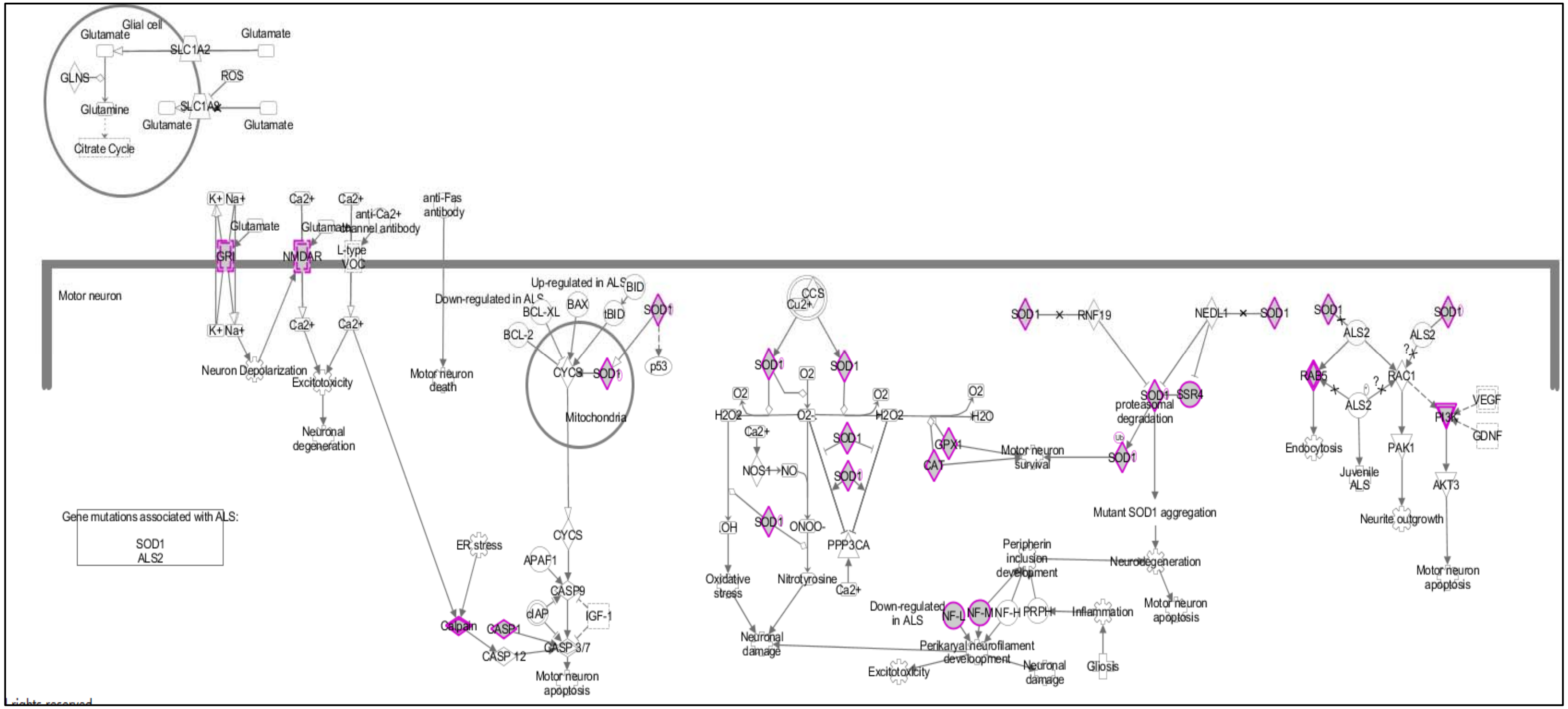

127-Complement System

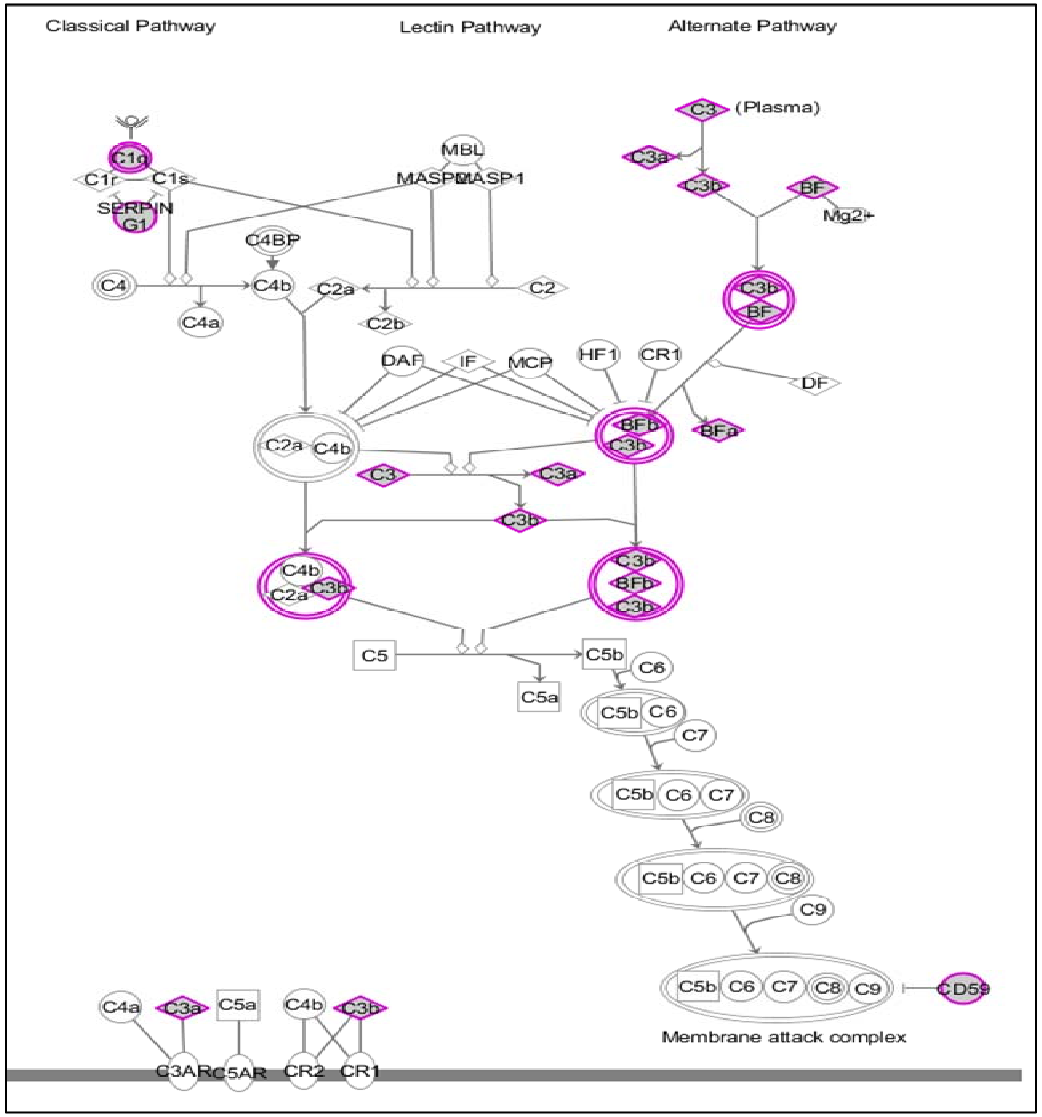

128-Glutathione Redox Reactions Ⅰ

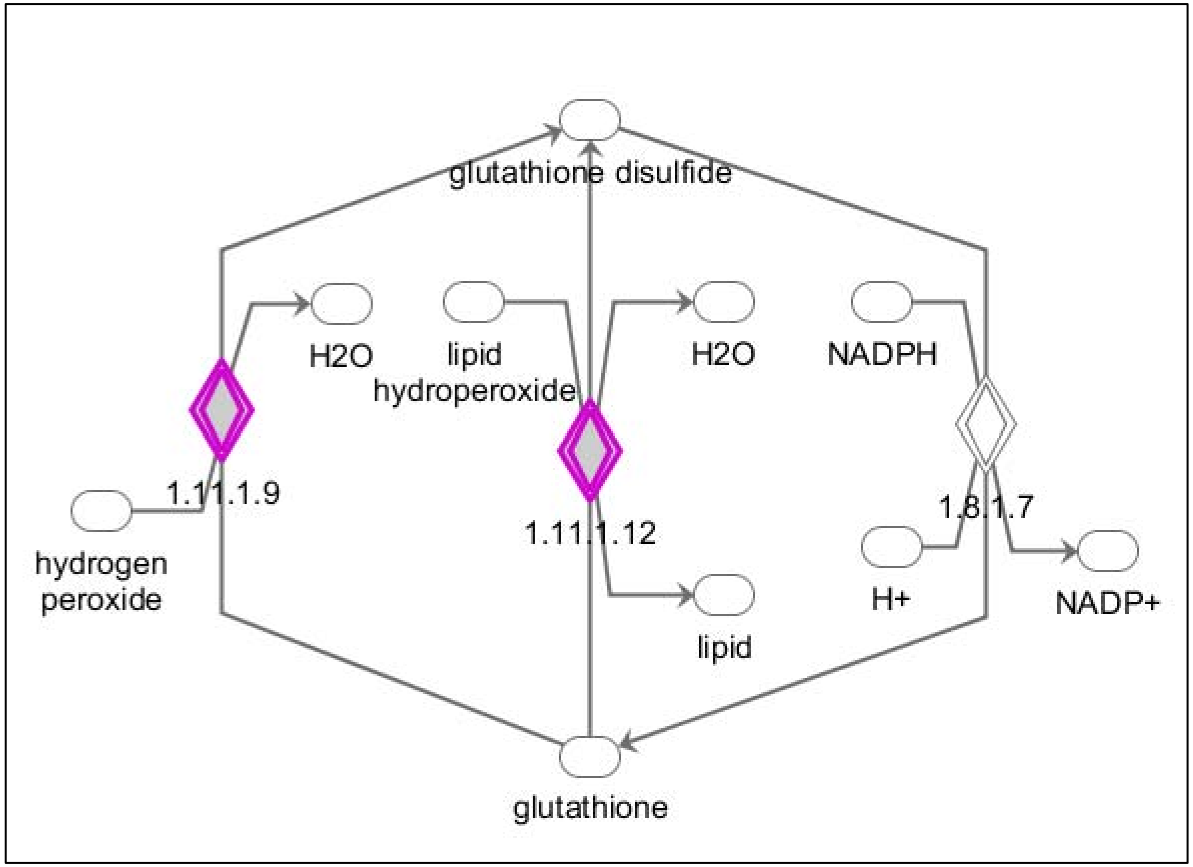

129-Endometrial Cancer Signaling

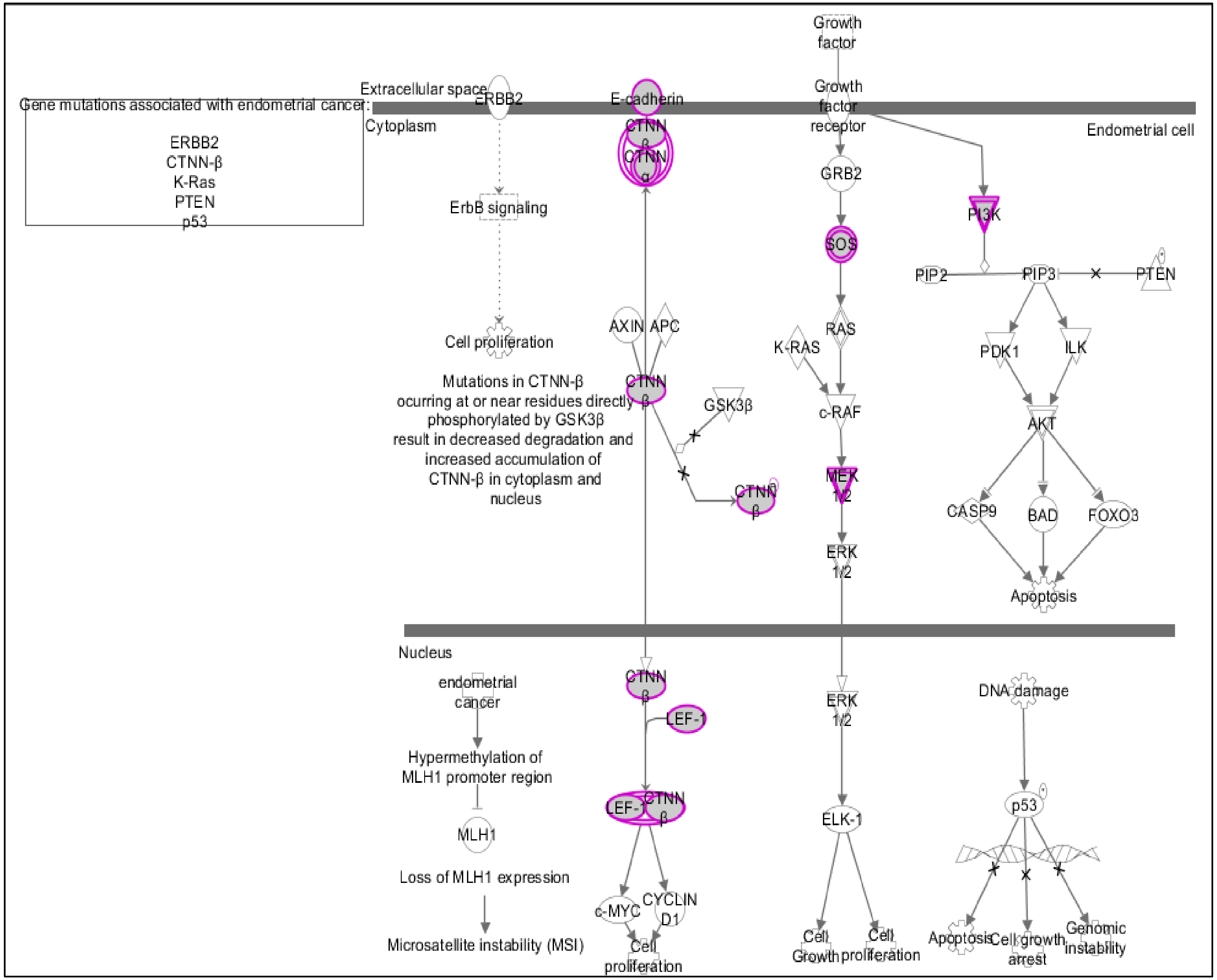

130-Nucleotide Excision Repair Pathway

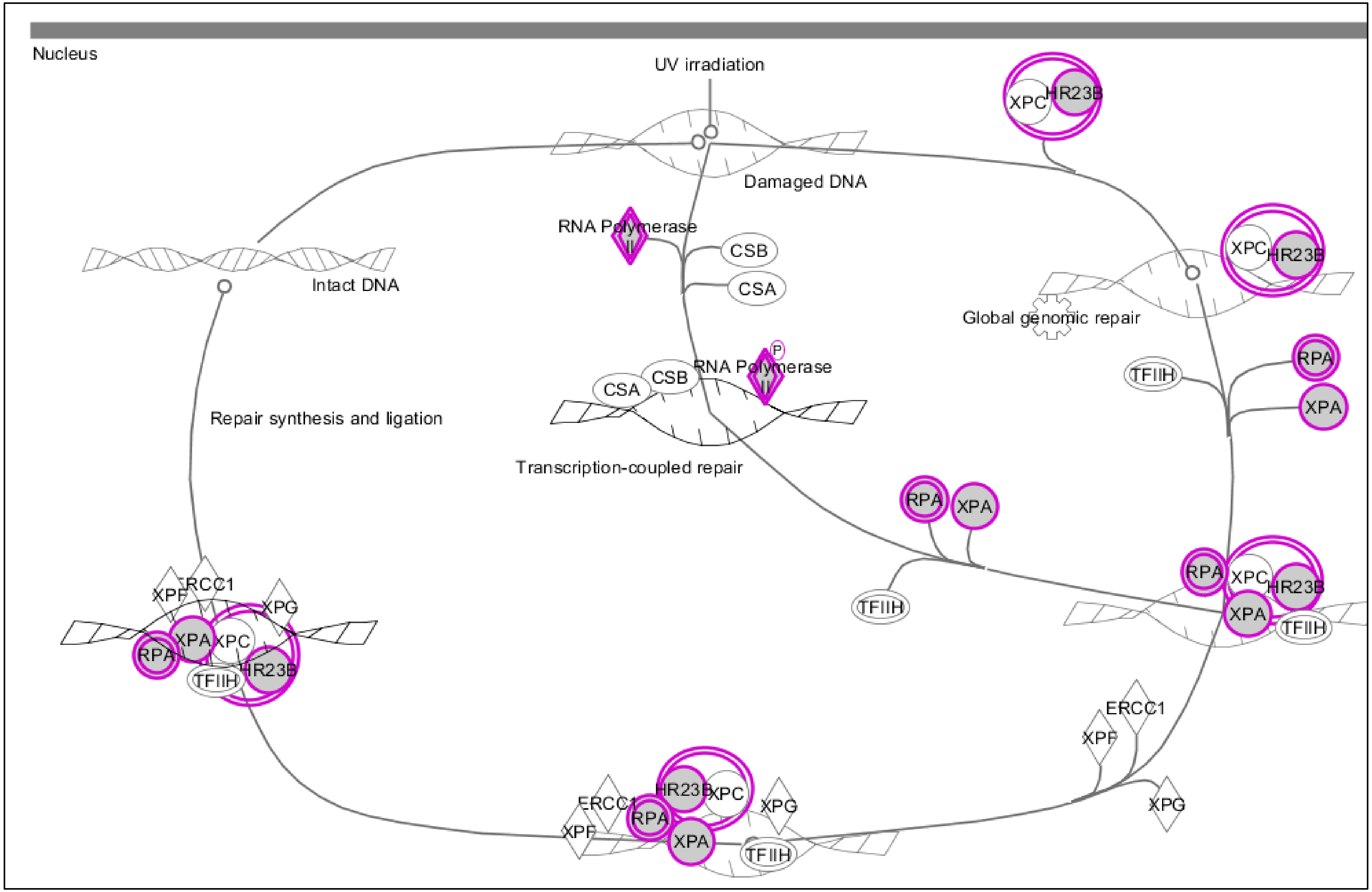

131-Tryptophan Degradation III (Eukaryotic)

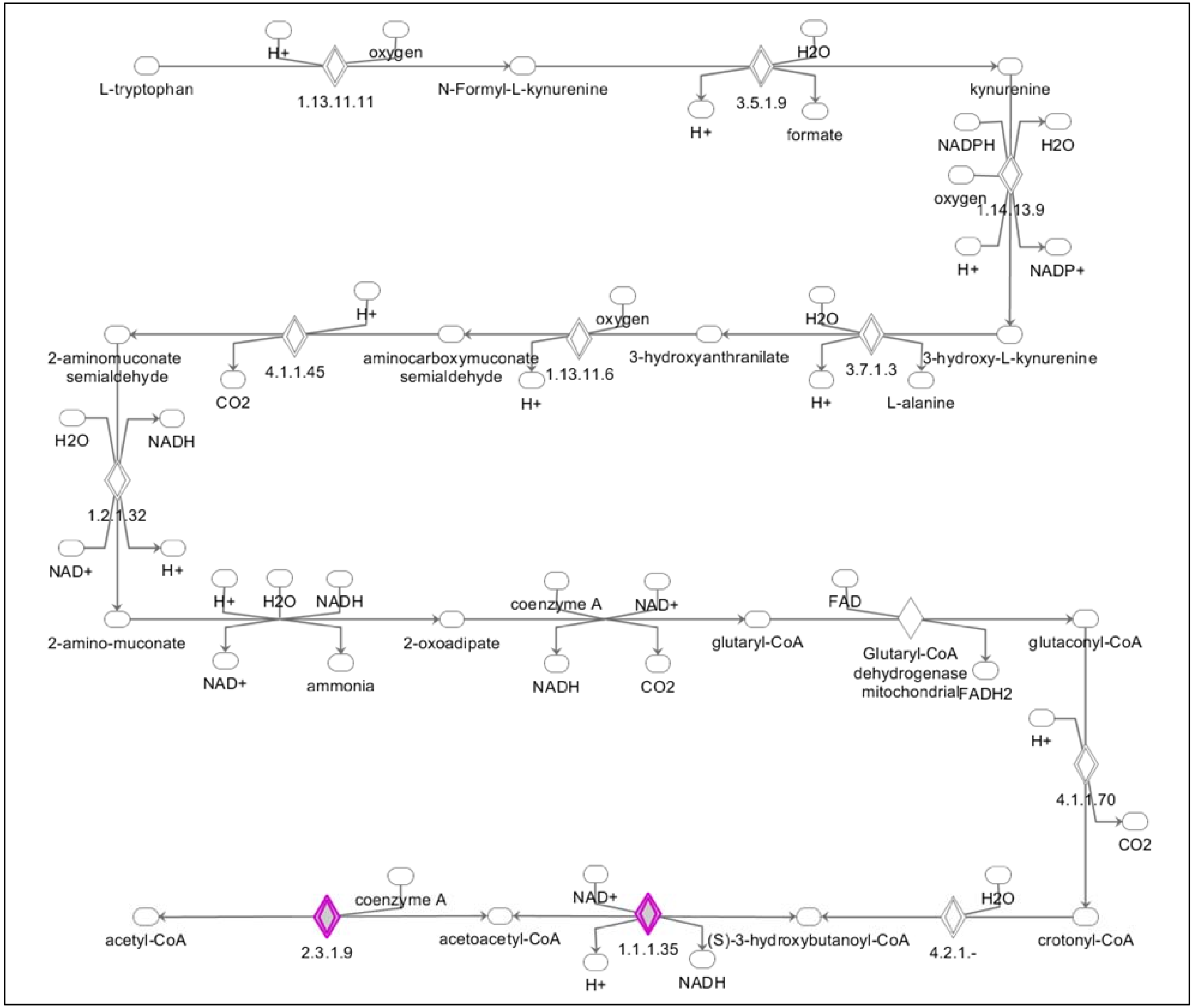

132-Phospholipase C Signaling

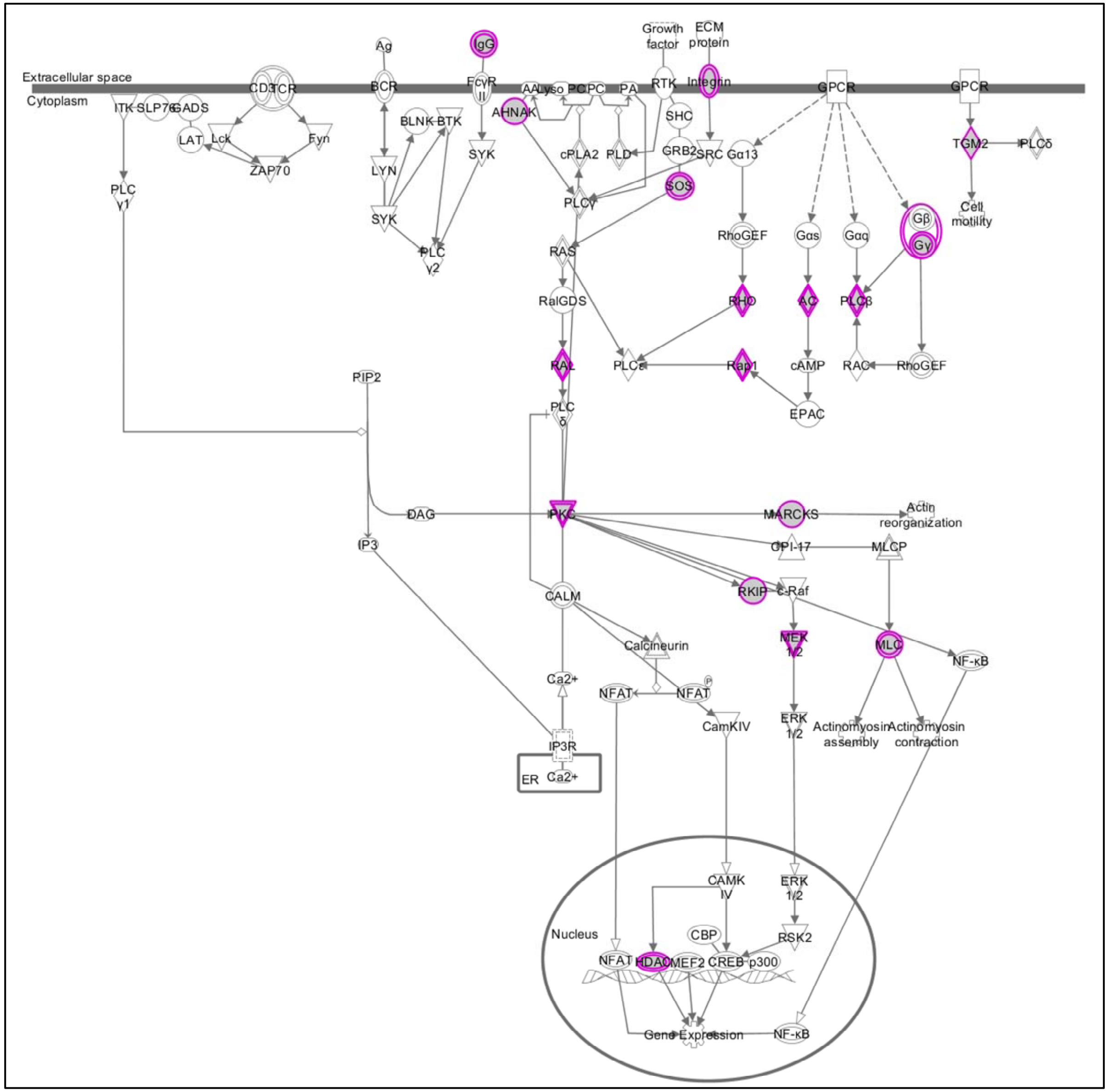

133-Ketolysis

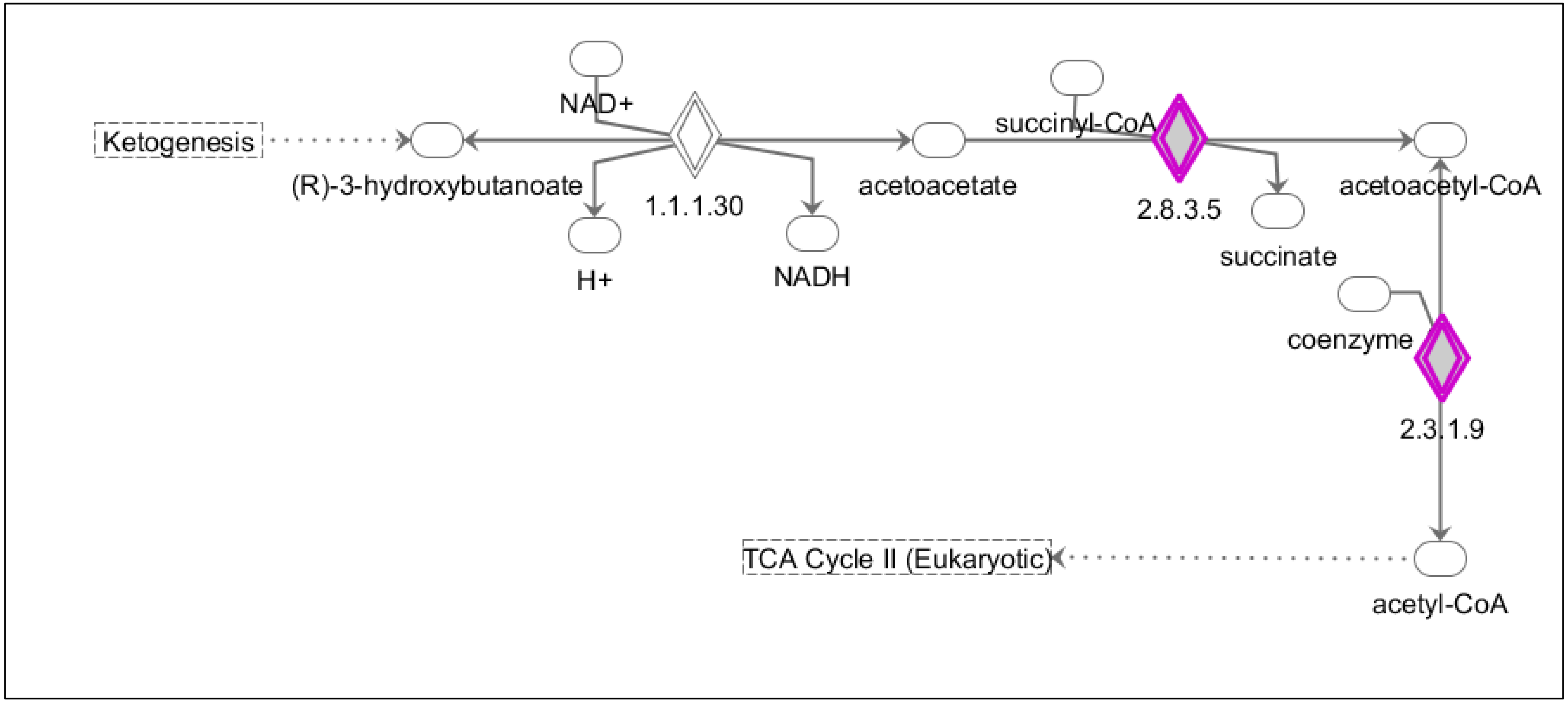

134-Thrombin Signaling

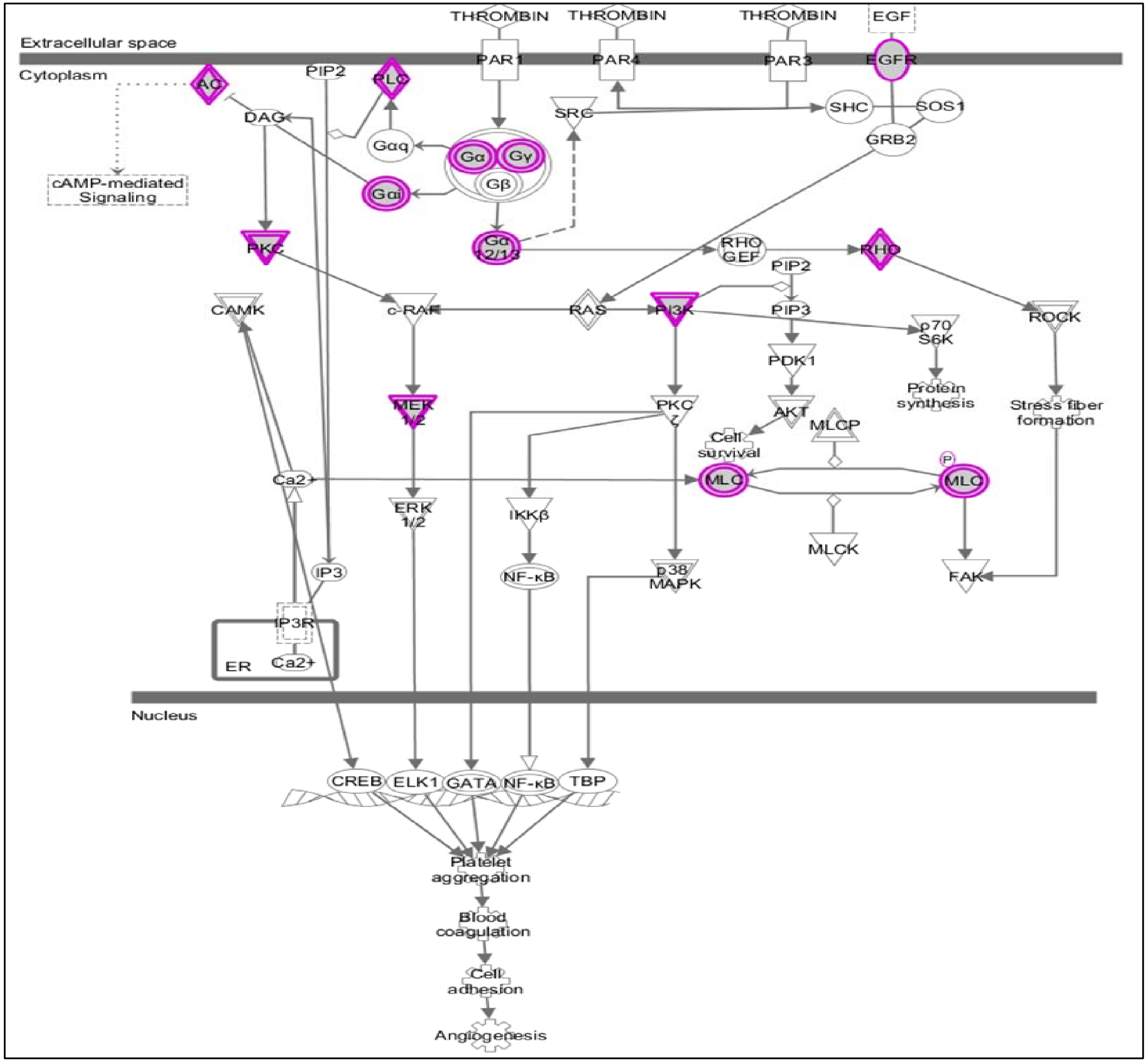

135-Nitric Oxide Signaling in the Cardiovascular System

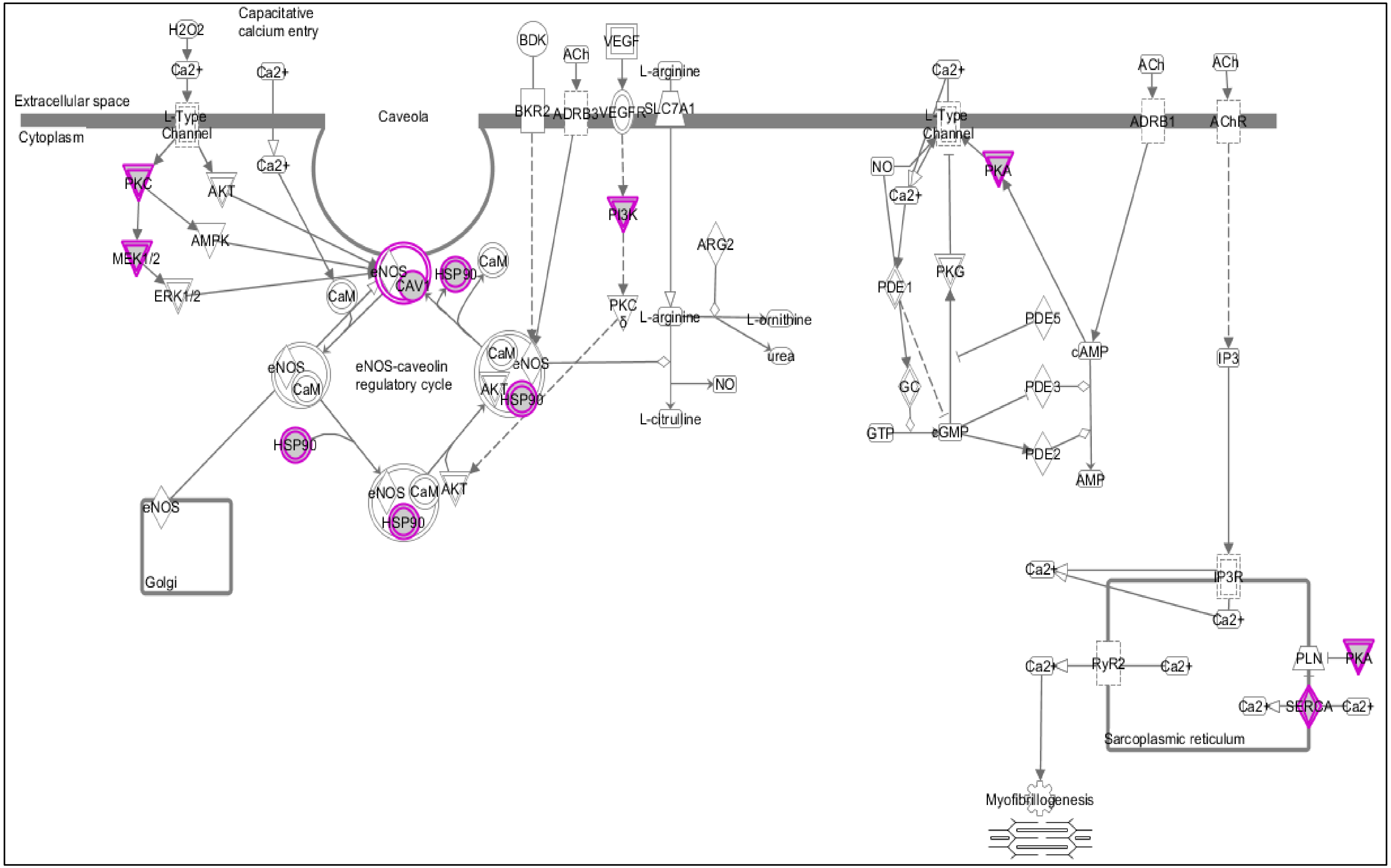

136-Neuroprotective Role of THOP1 in Alzheimer's Disease

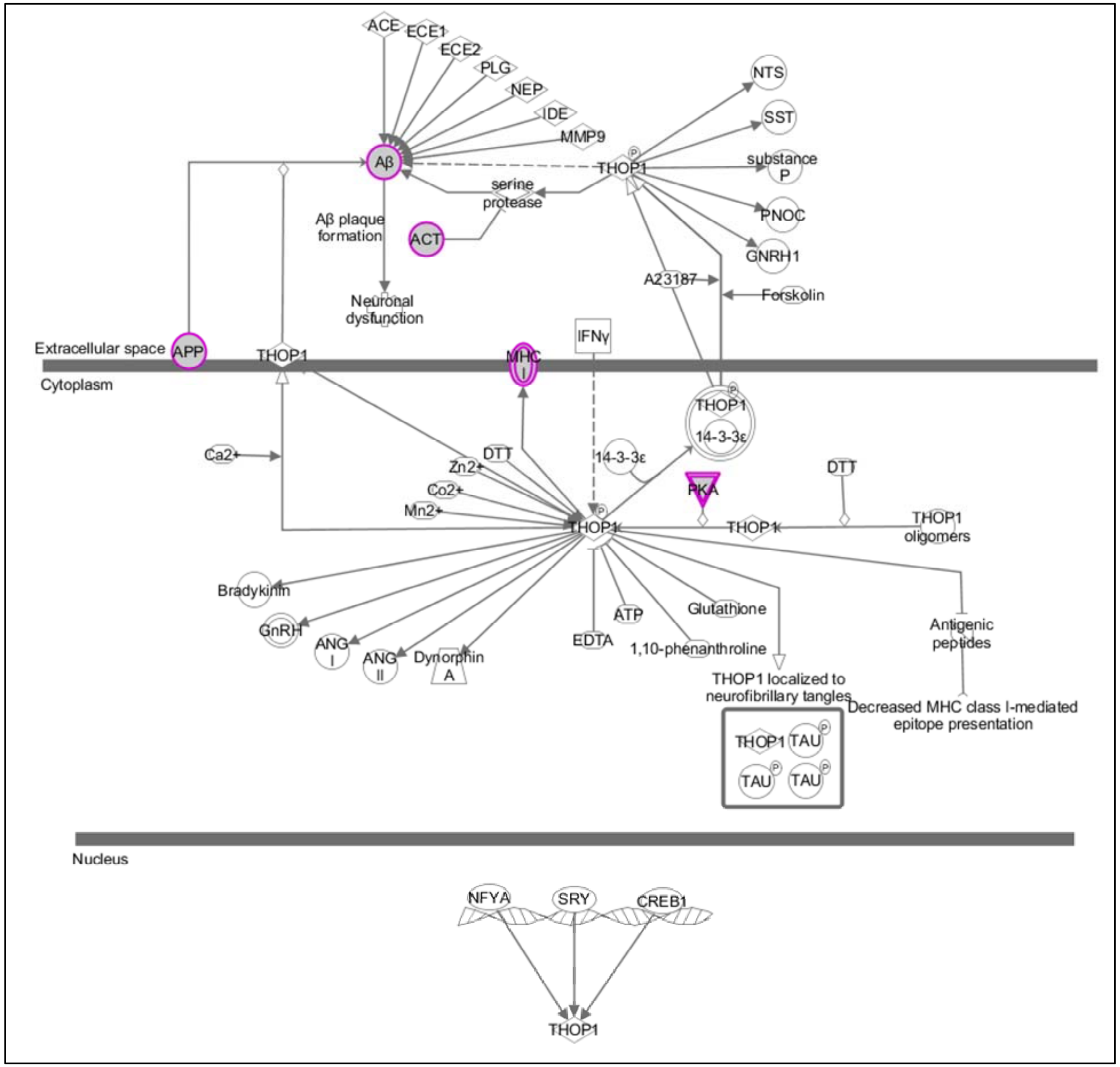

137-Antigen Presentation Pathway

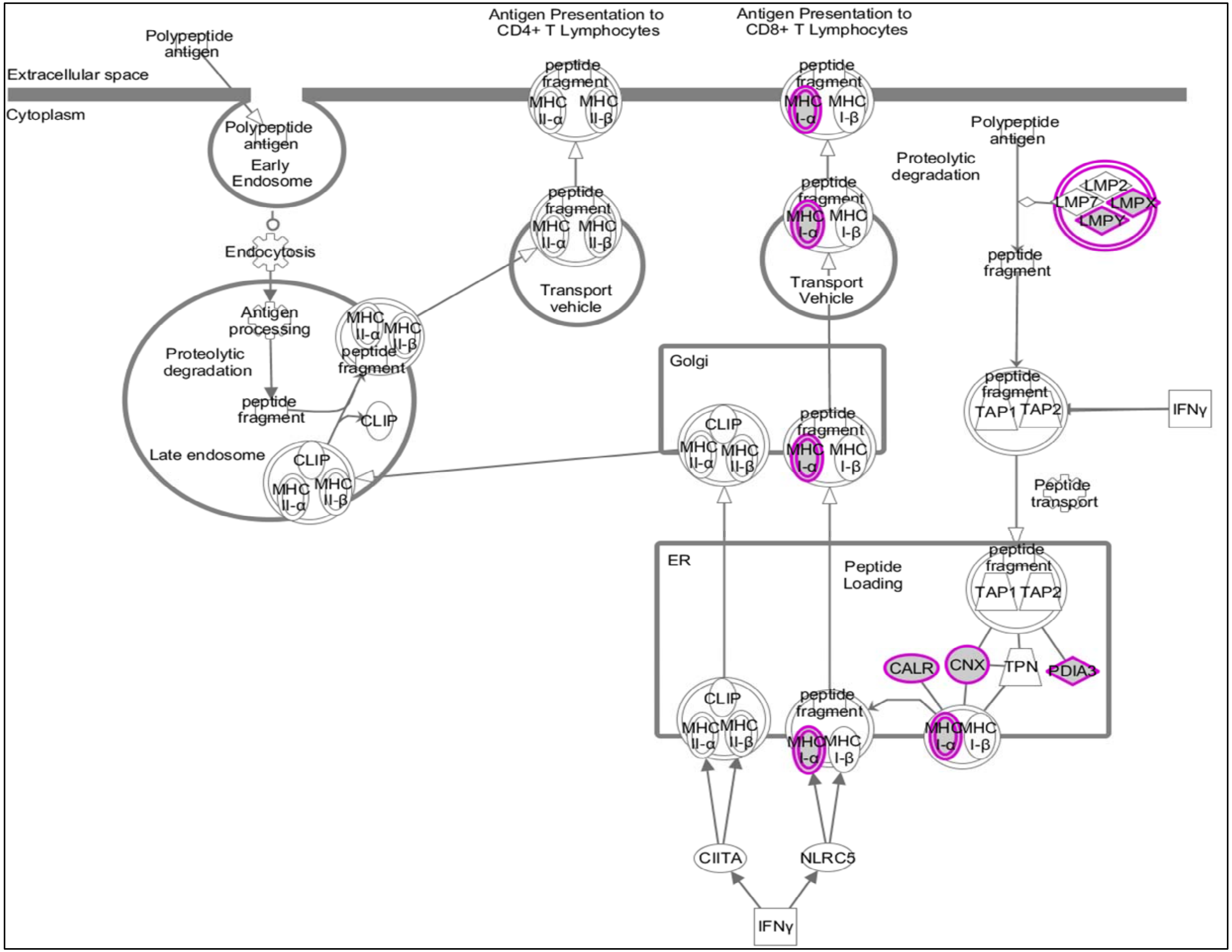

138-Synaptic Long Term Depression

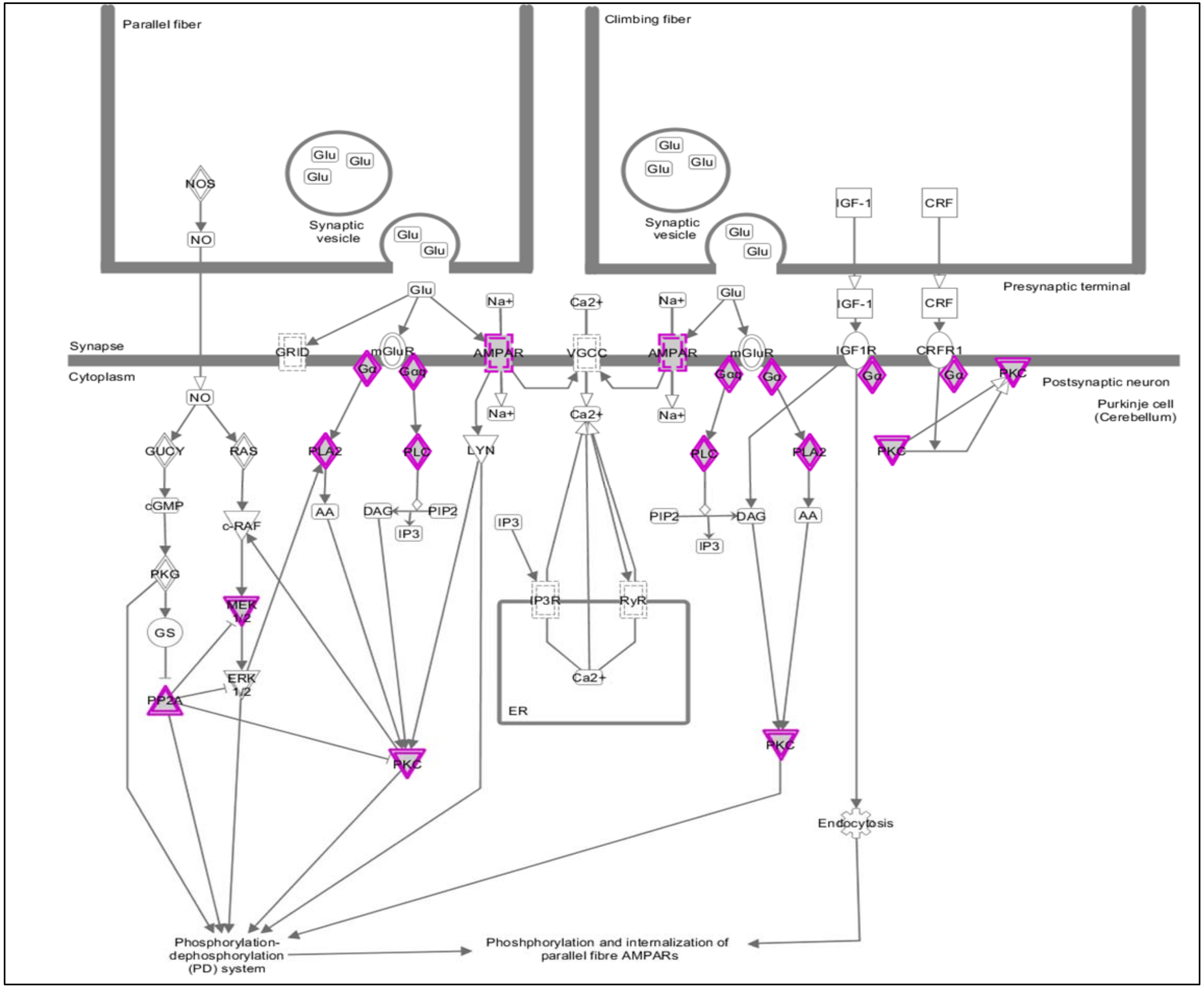

139-PAK Signaling

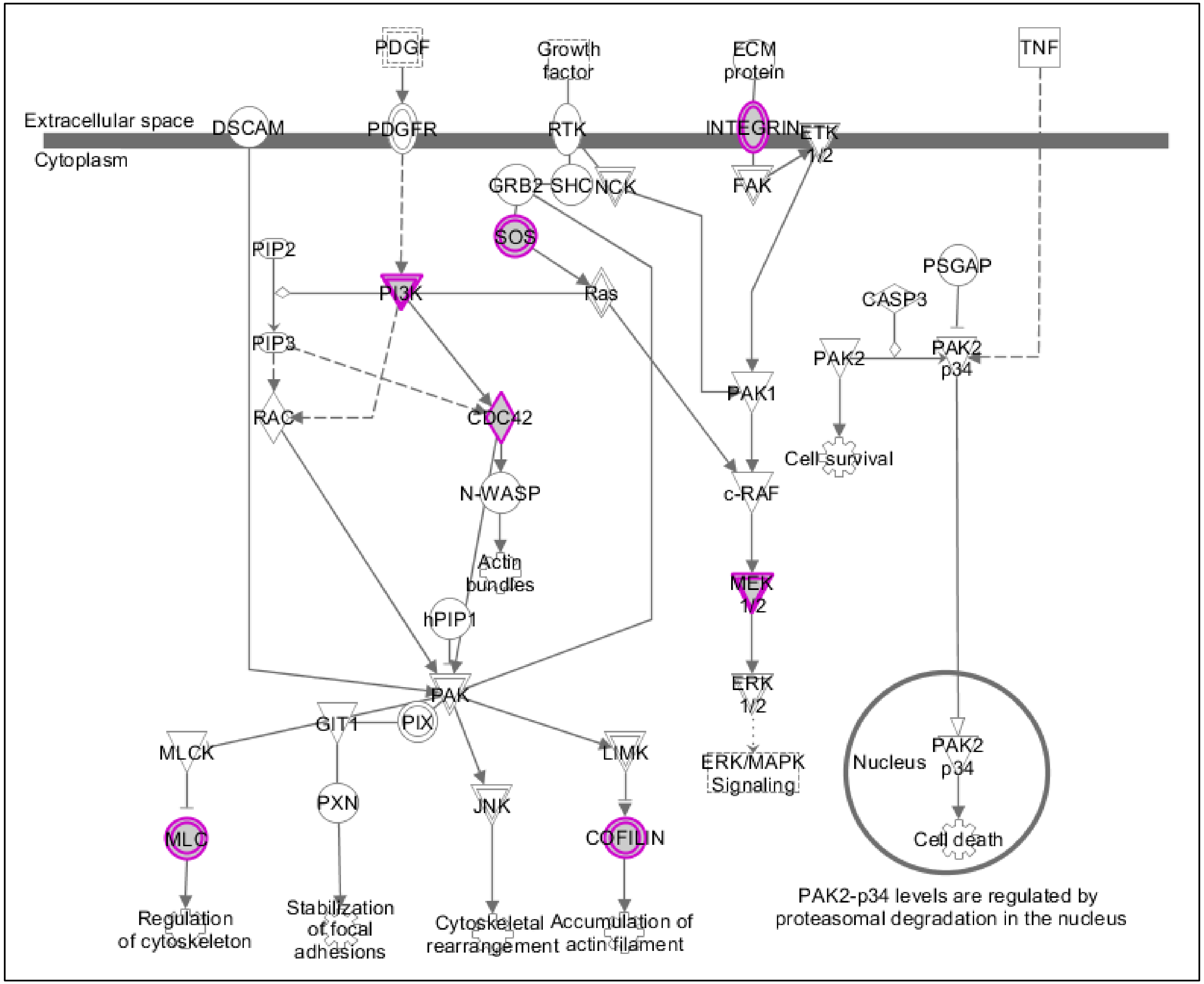

140-Pentose Phosphate Pathway (Oxidative Branch)

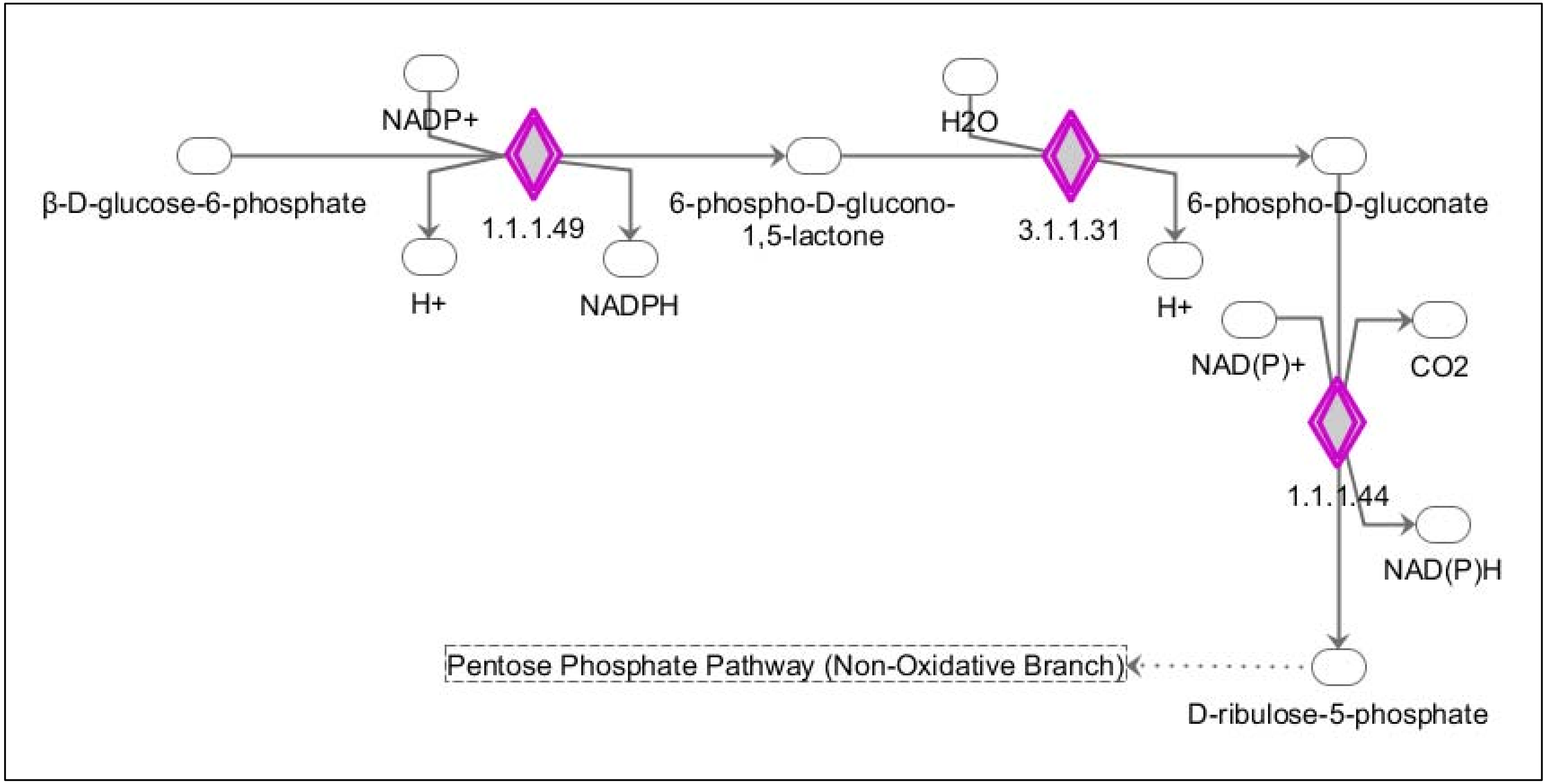

141-L-cysteine Degradation Ⅰ

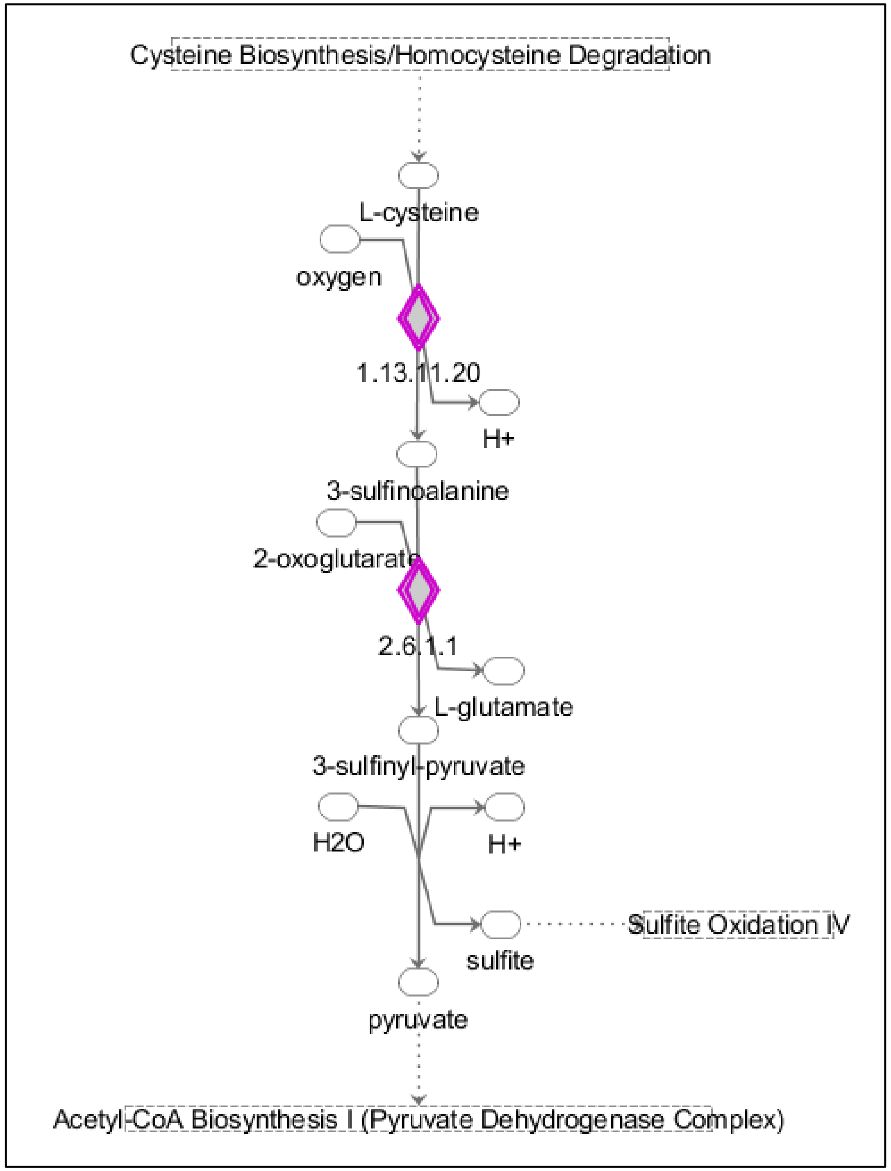

142-Sucrose Degradation Ⅴ(Mammalian)

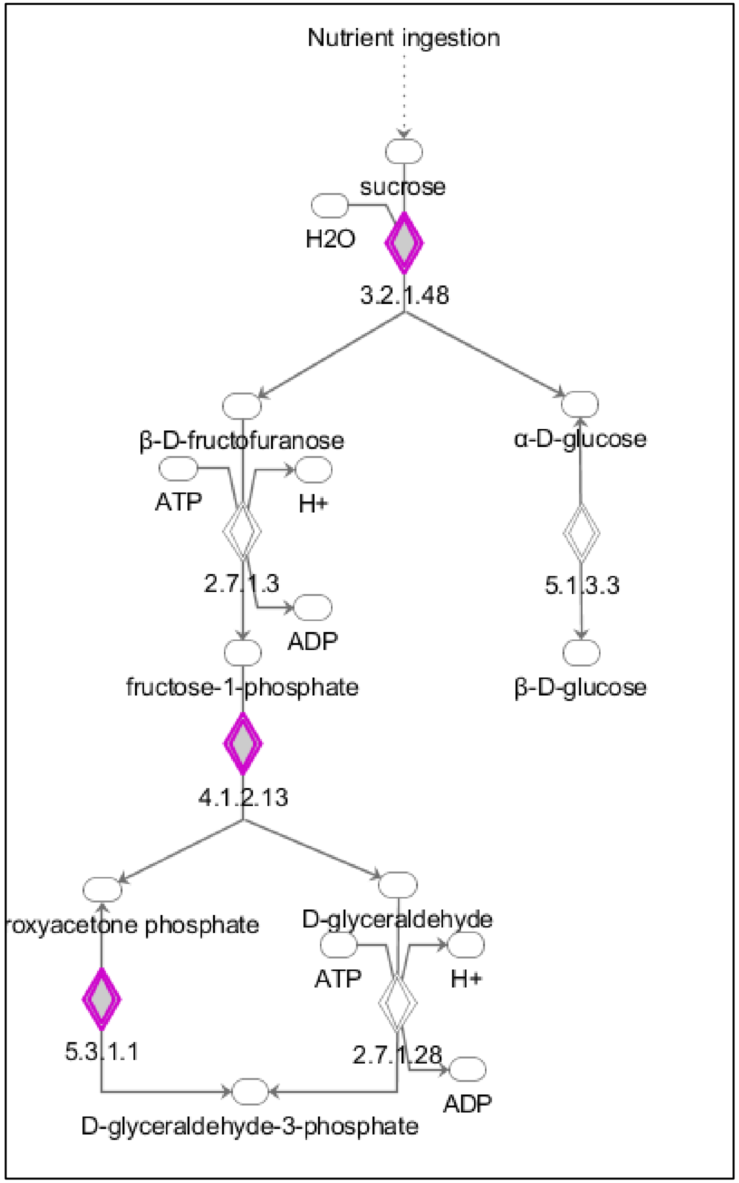

143-fMLP Signaling in Neutrophils

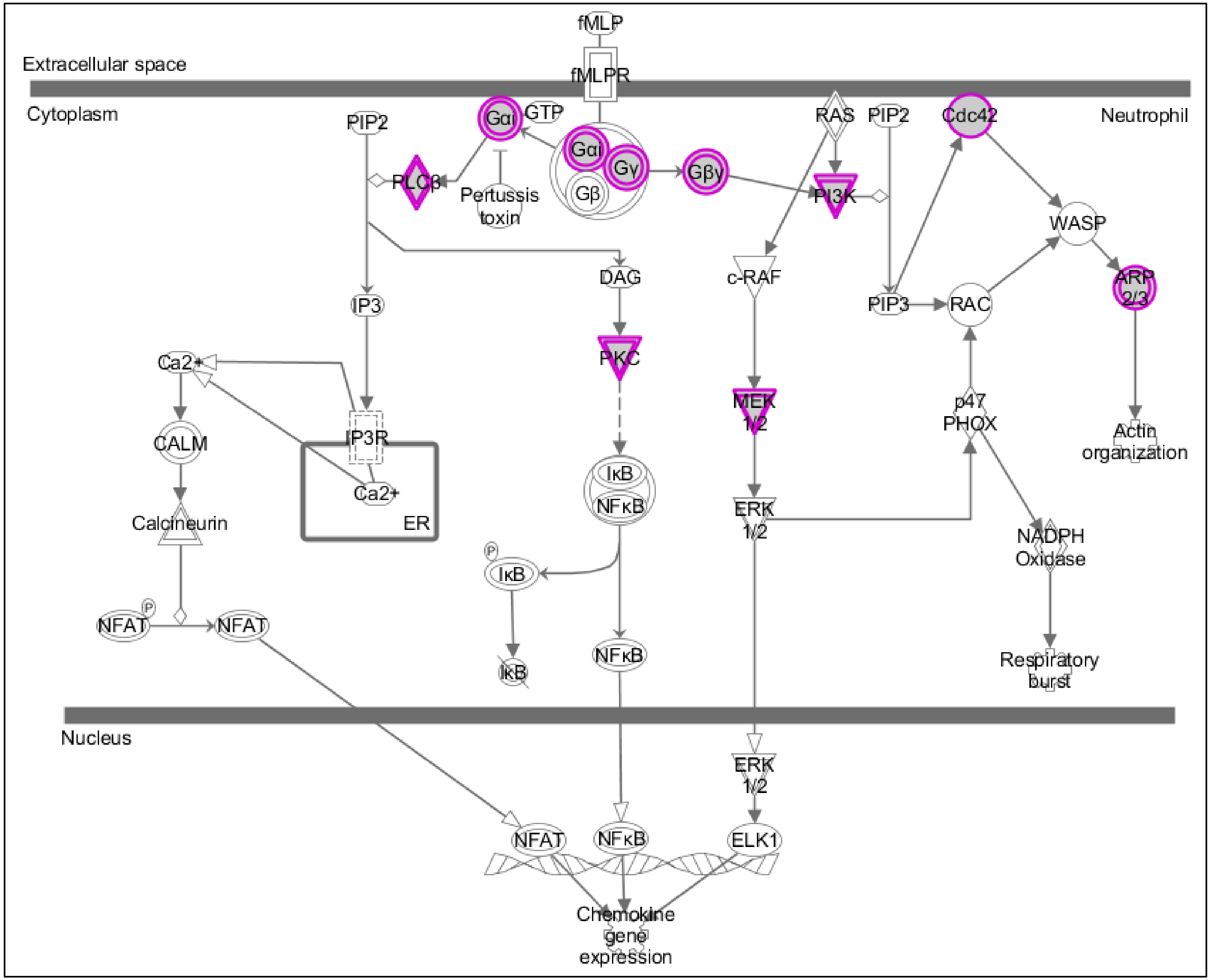

144-Pyrimidine Ribonucleotides Interconversion

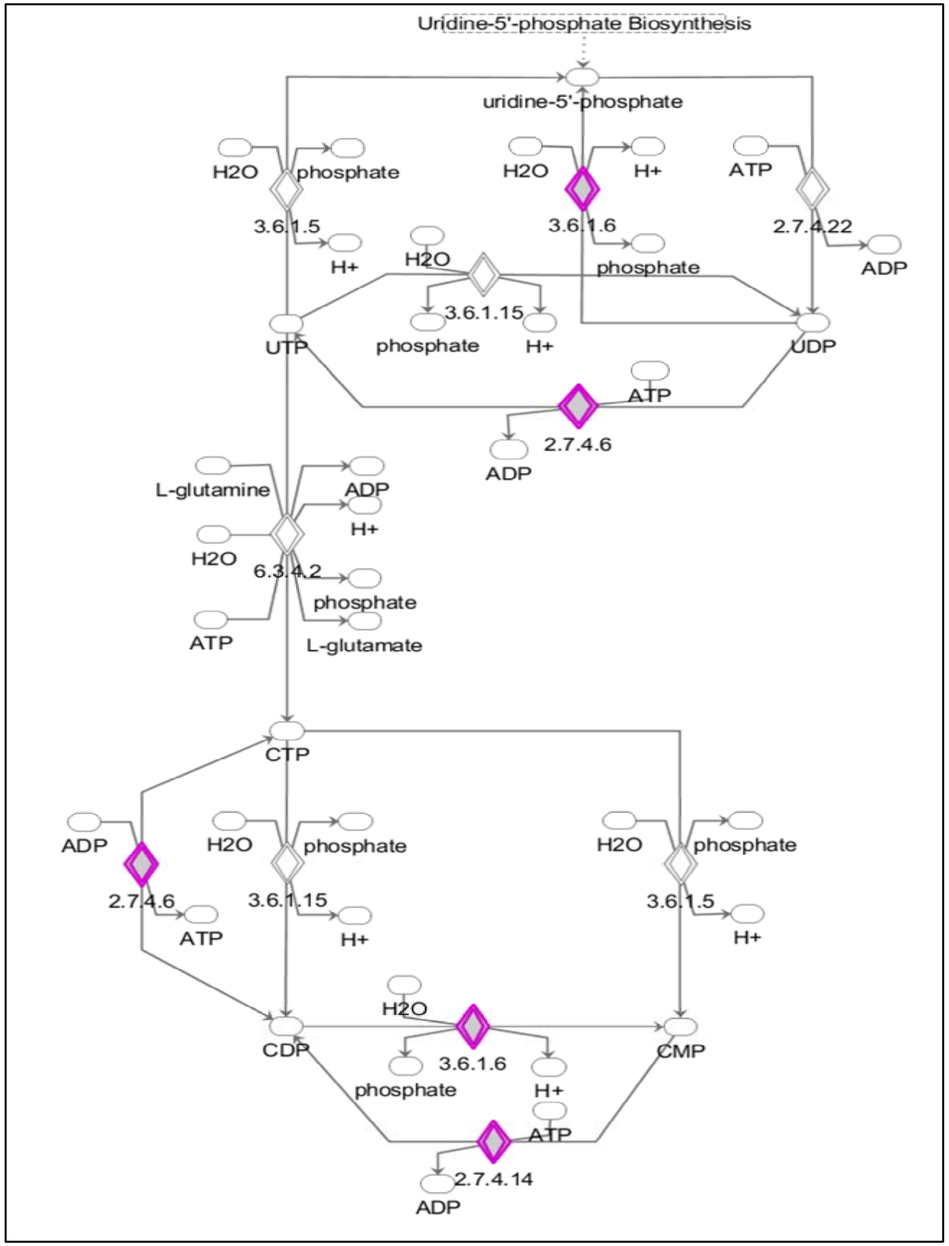

145-Coloretal Cancer Metastasis Signaling

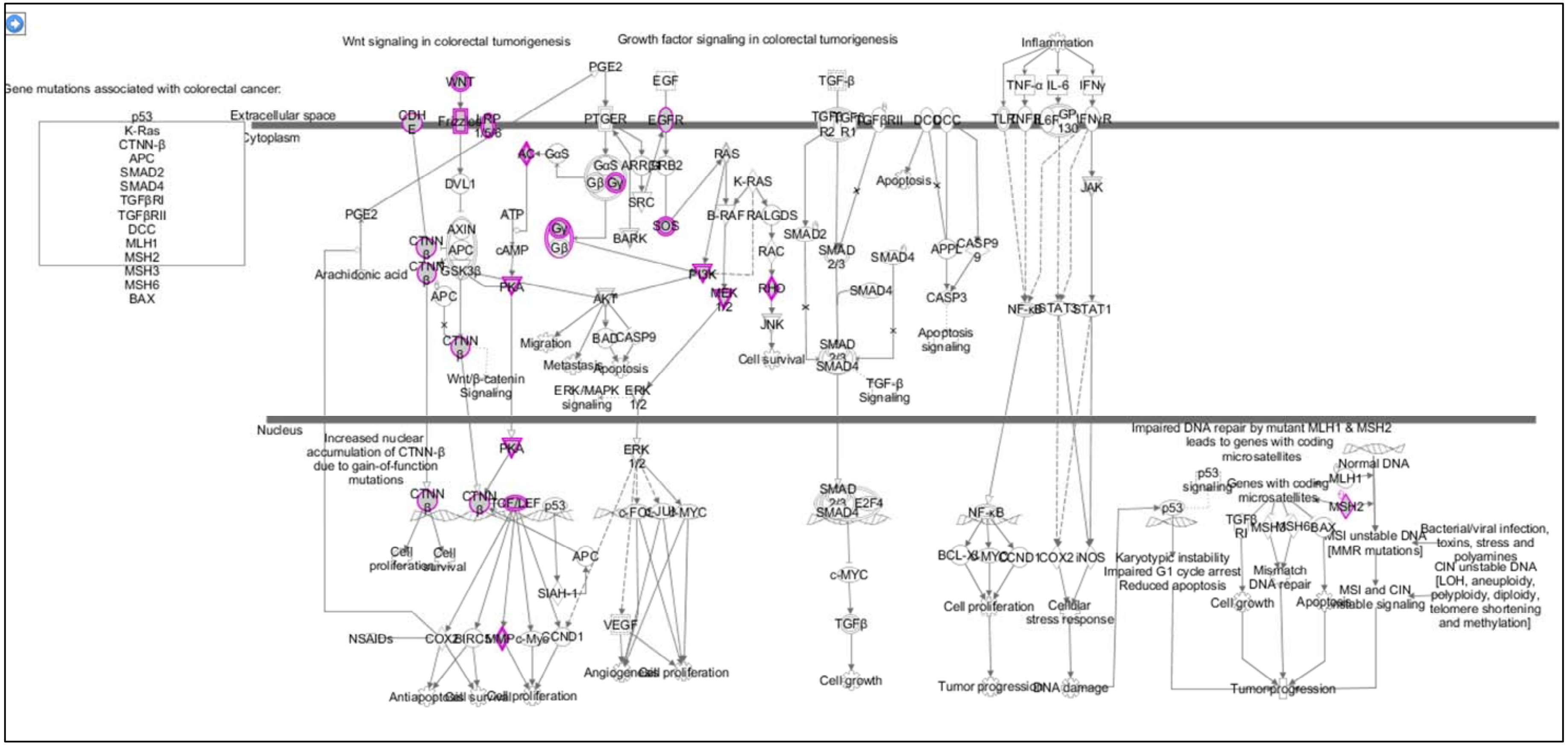

146-Glucocorticoid Receptor Signaling

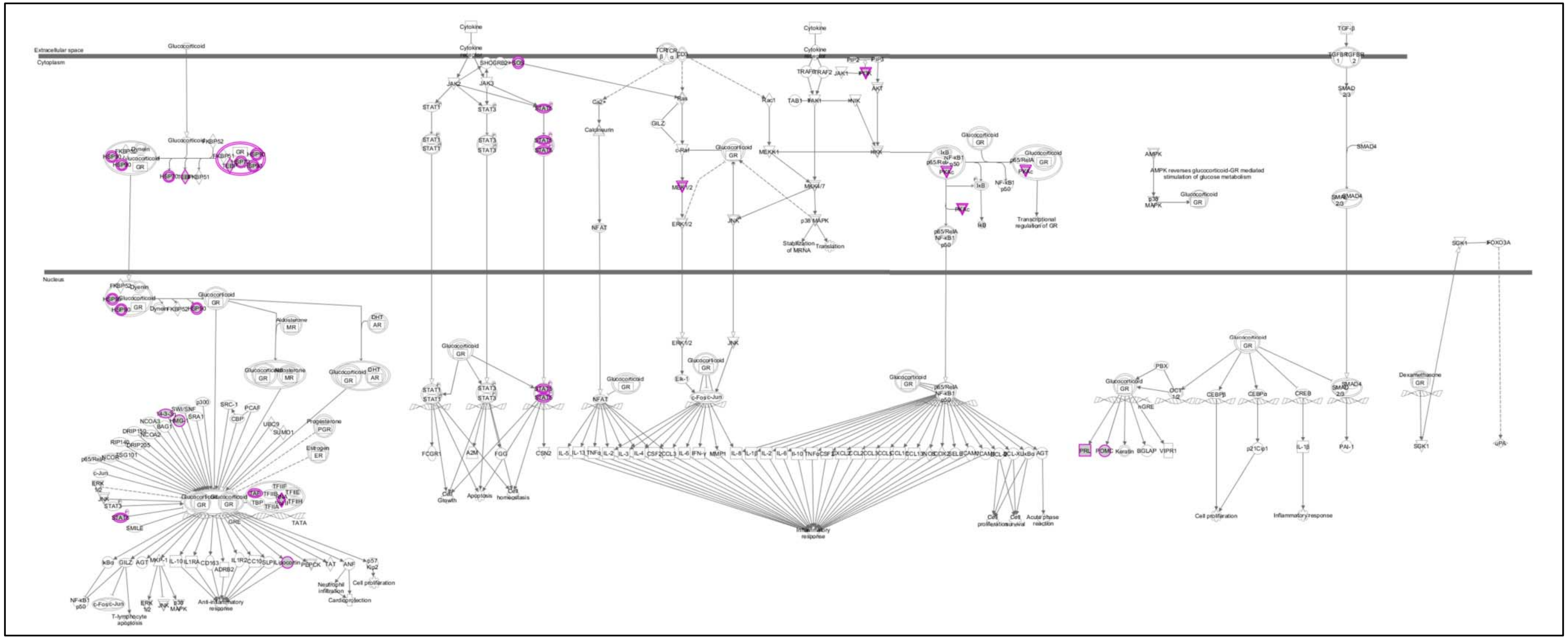

147-Cell Cycle: G2/M DNA Damage Checkpoint Regulation

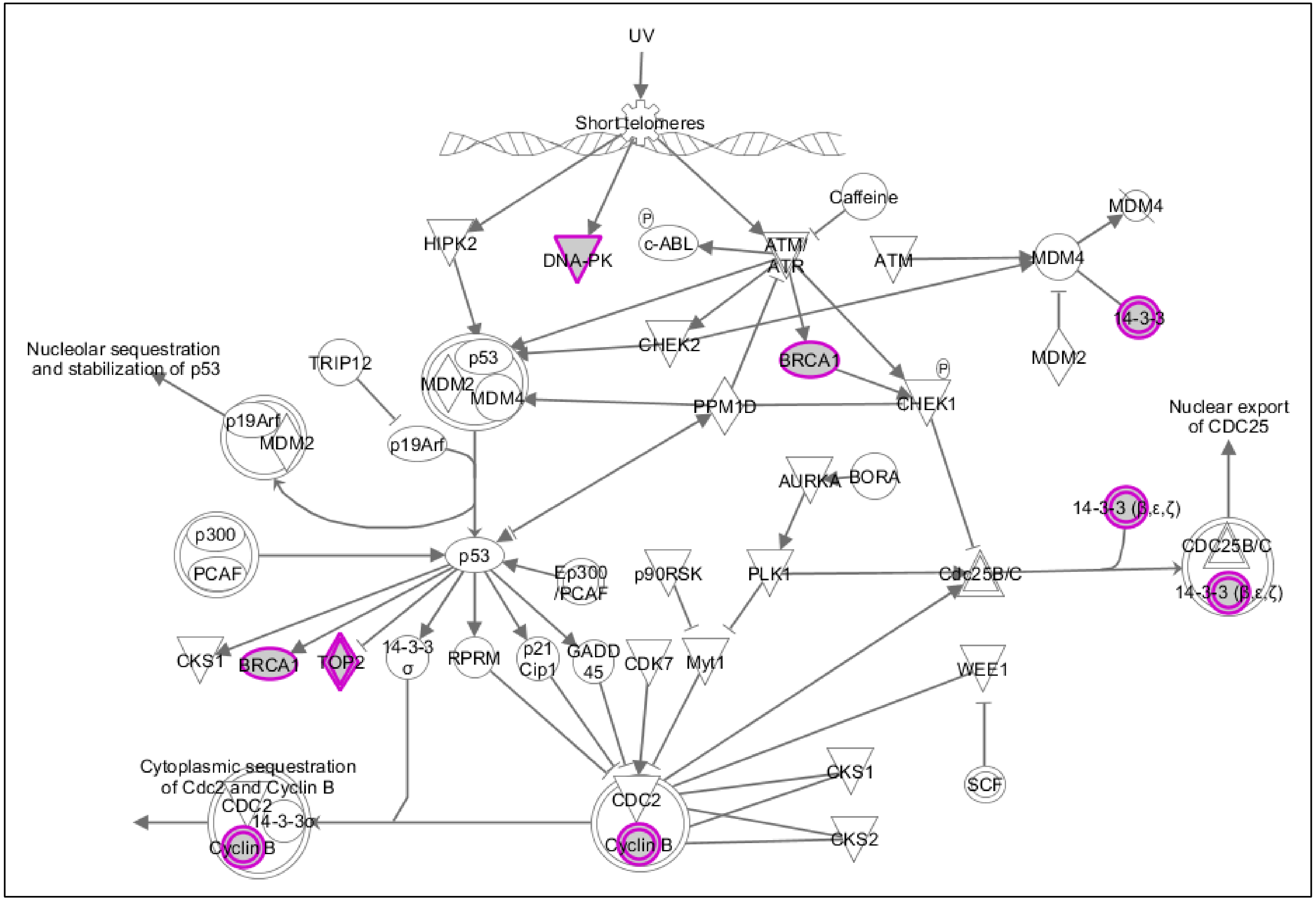

148-Cellular Effects of Sildenafil (Viagra)

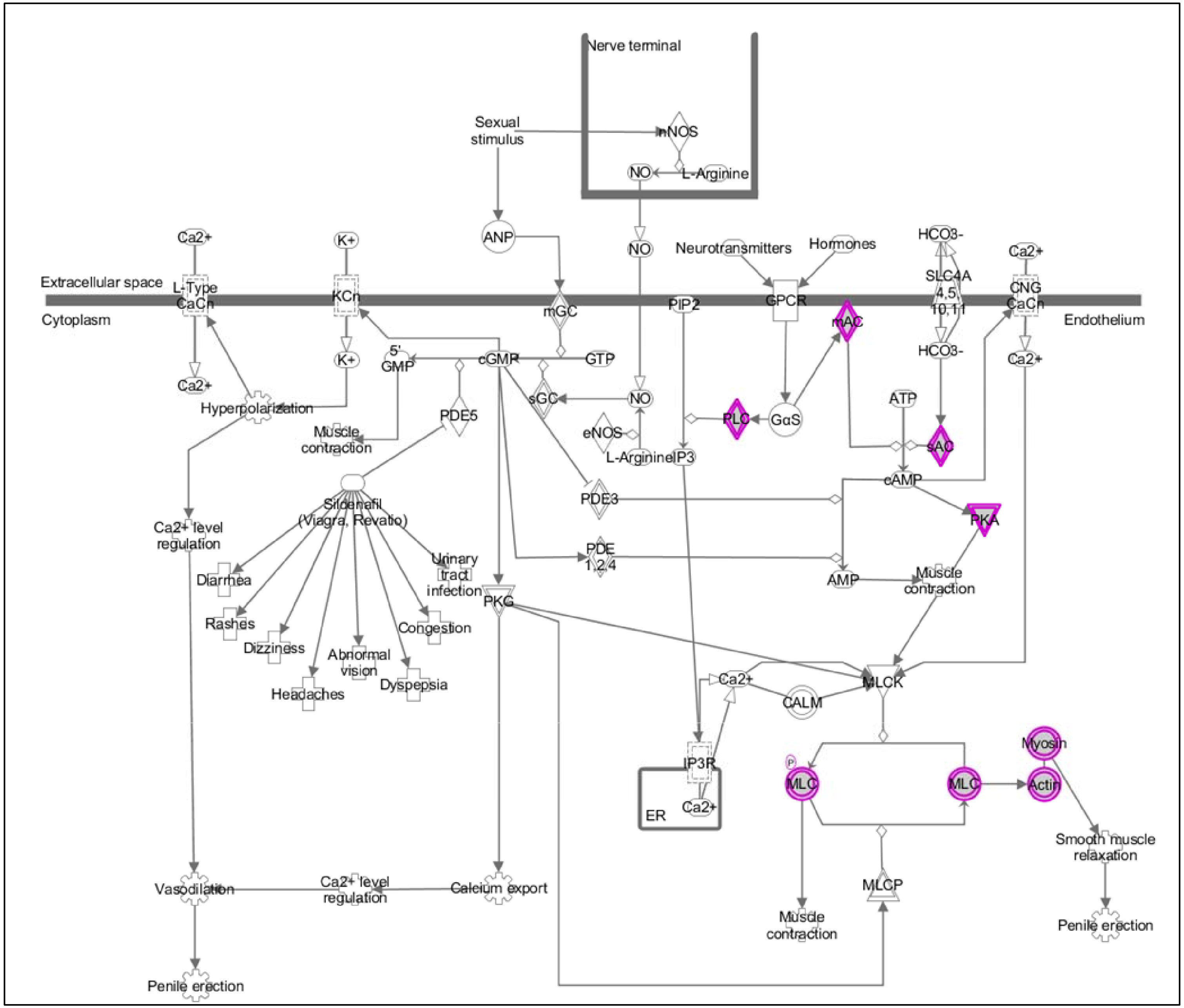

149-Fatty Acid β-oxidation Ⅲ(Unsaturated, Odd Number)

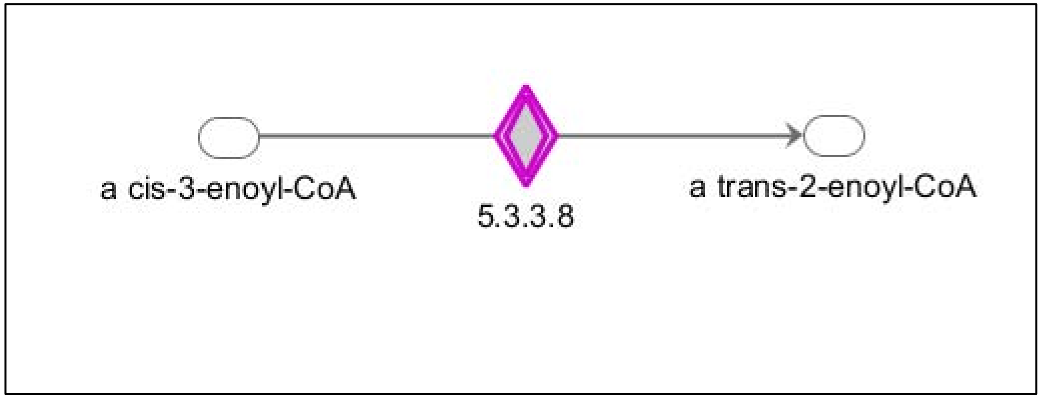

150-HGF Signaling

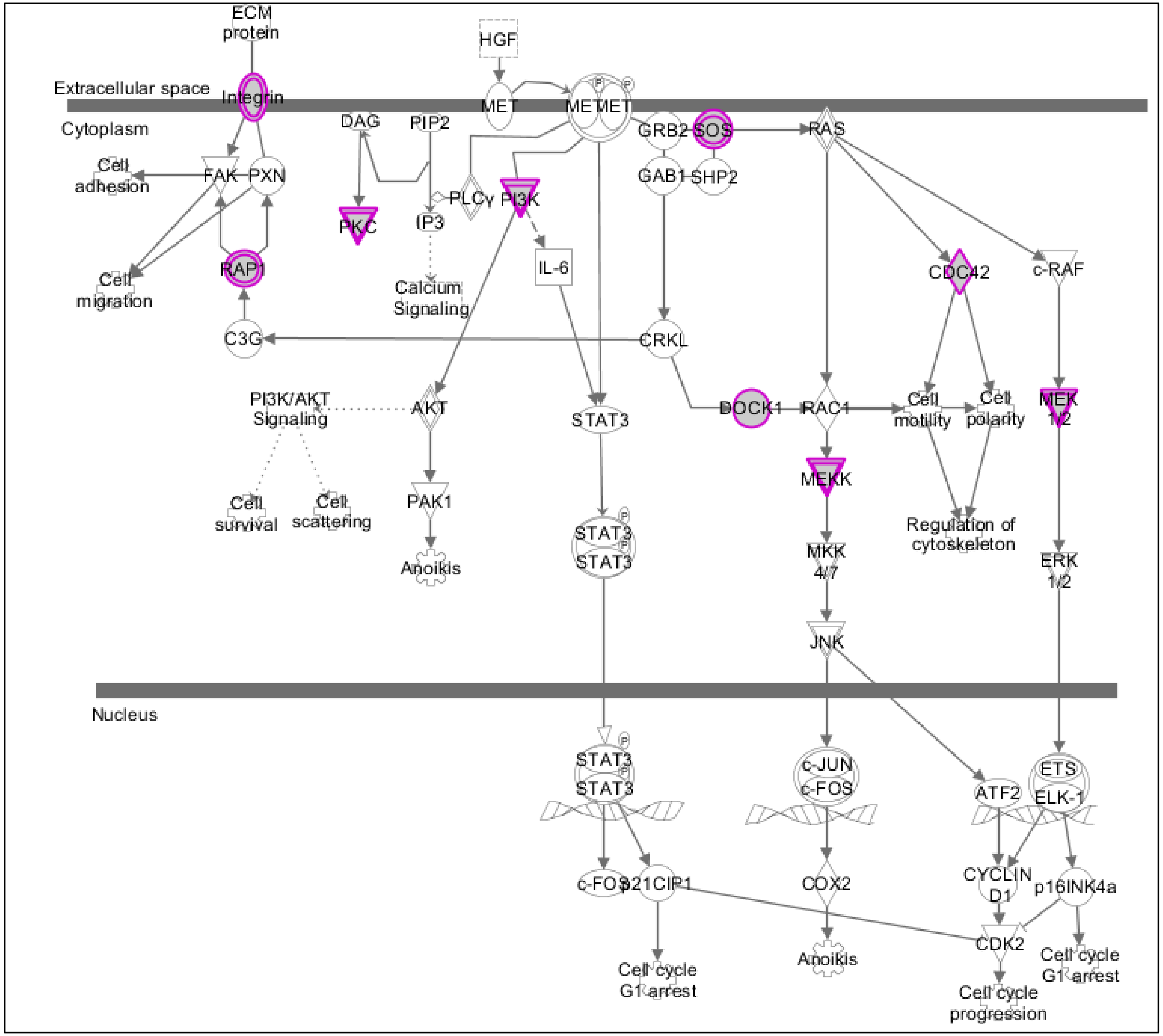

151-GABA Receptor Signaling

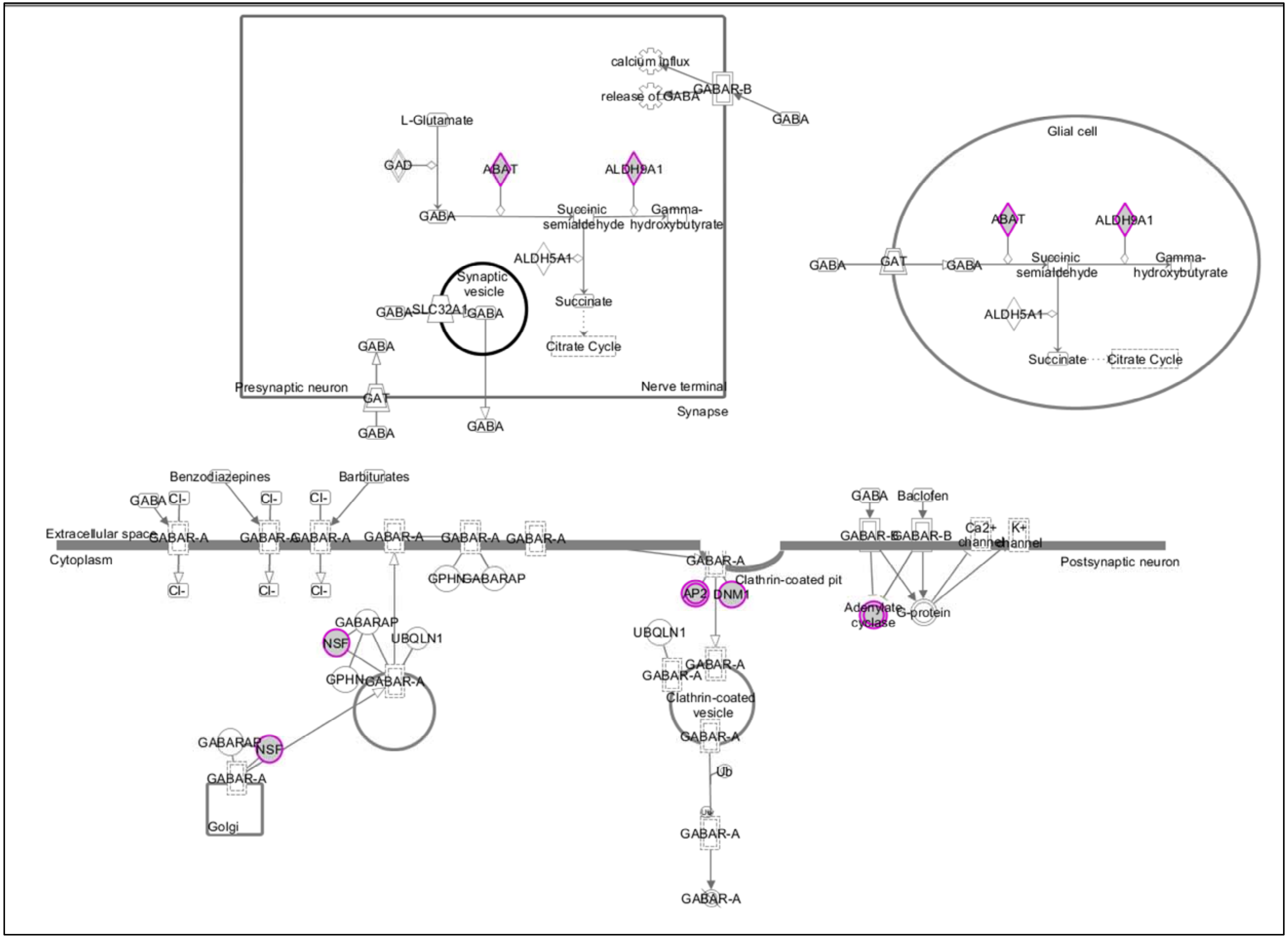

152-Pyrimidine Ribonucleotides De Novo Biosynthesis

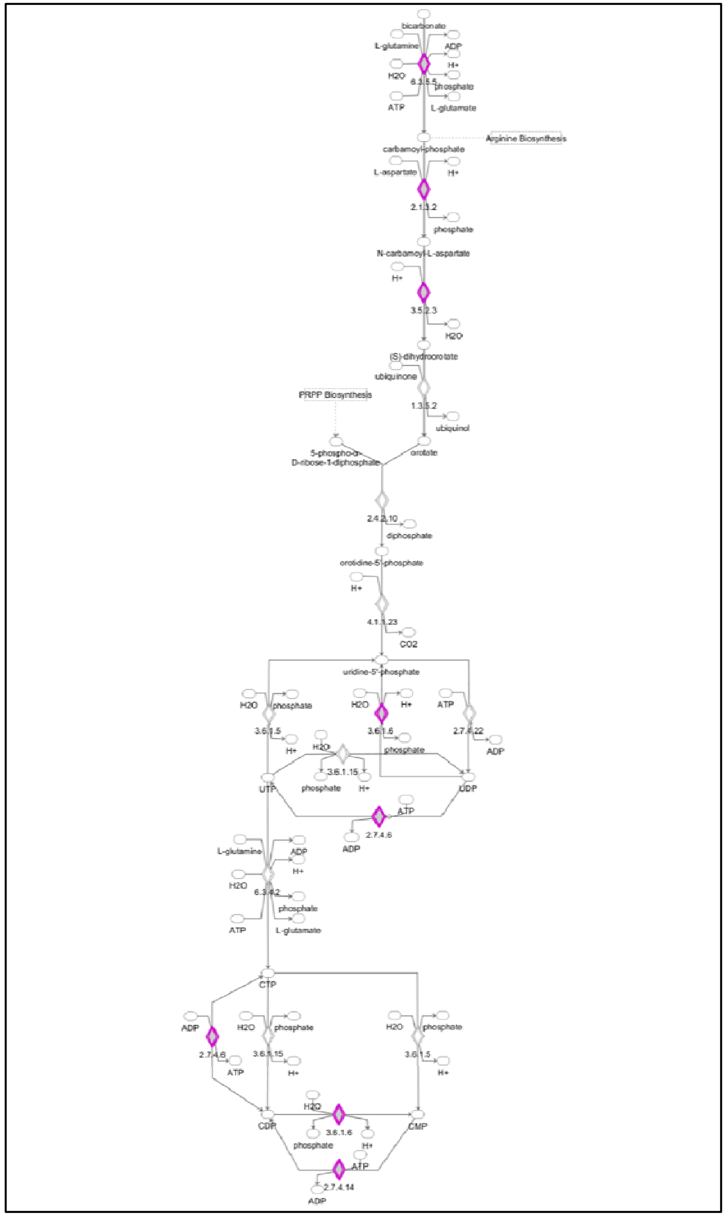

153-Mechanisms of Viral Exit from Host Cells

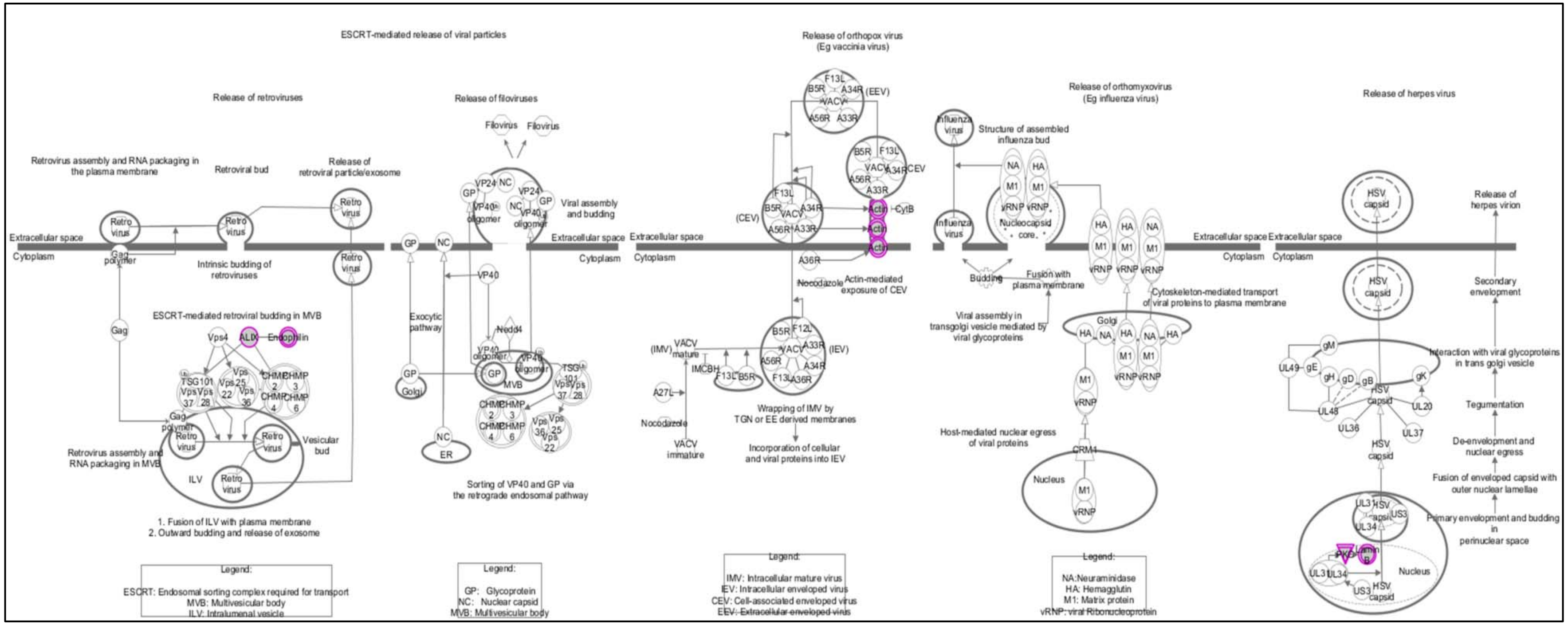

154-PCP Pathway

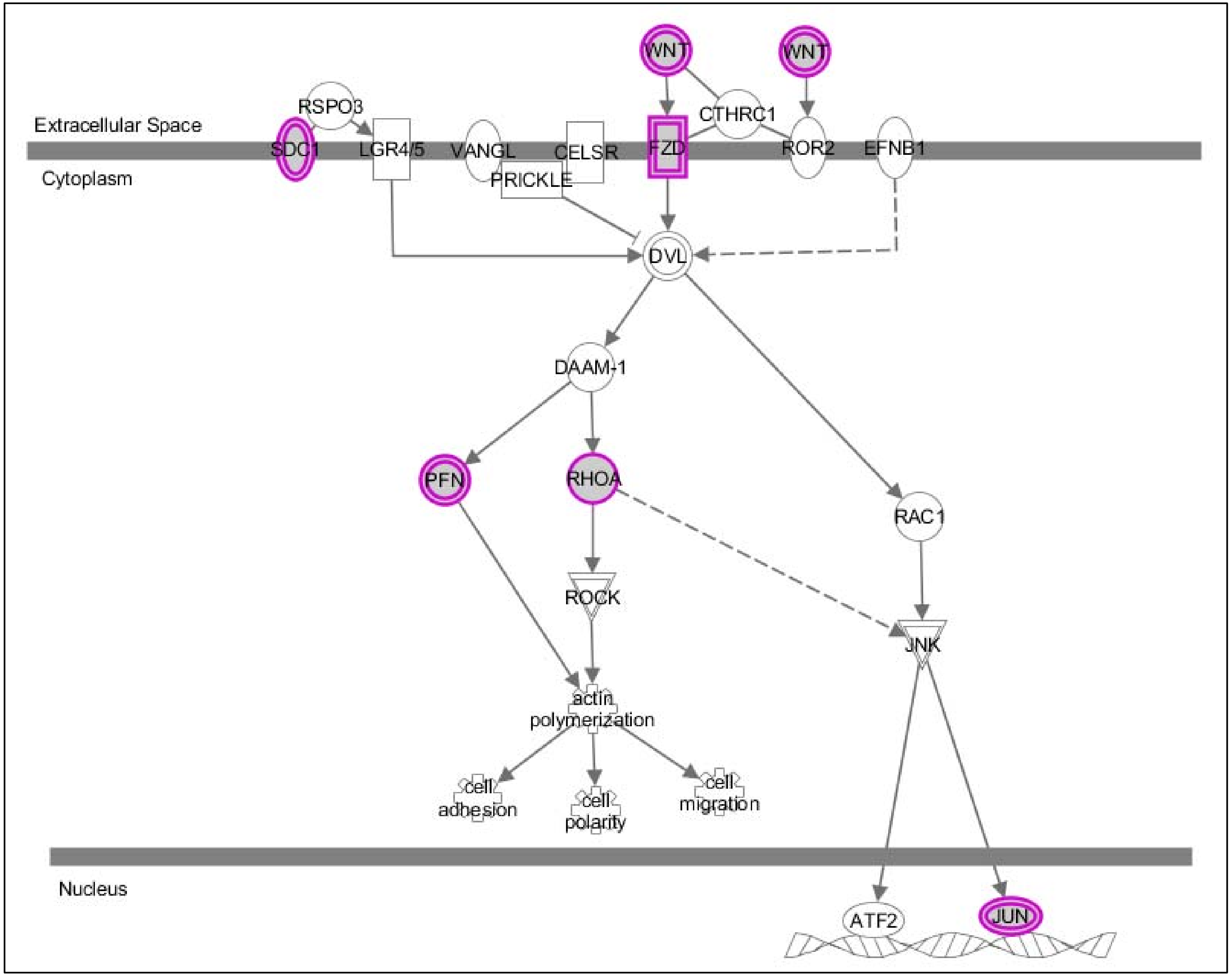

155-Primary Immunodeficiency Signaling

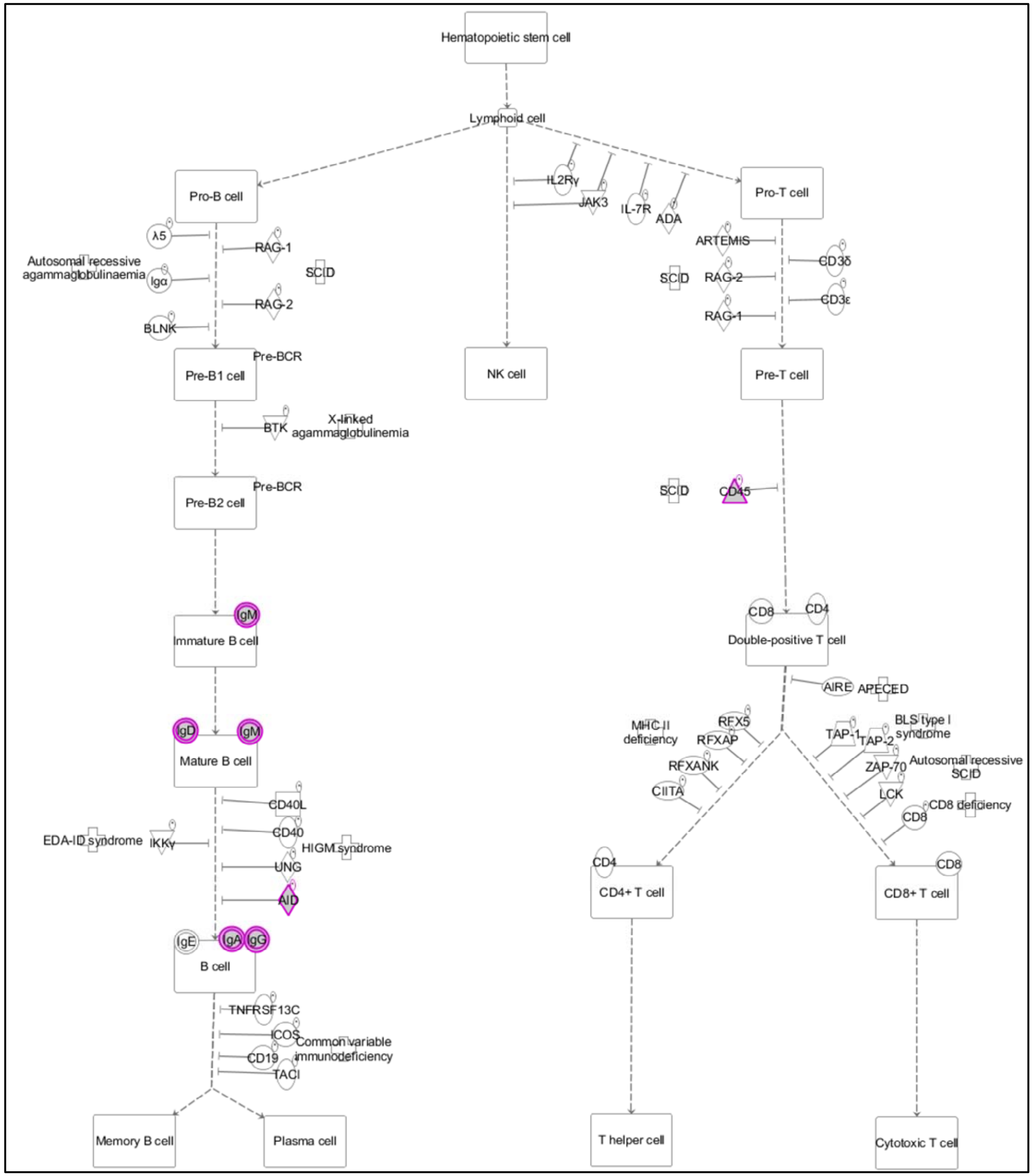

156-Systemic Lupus Erythematosus Signaling

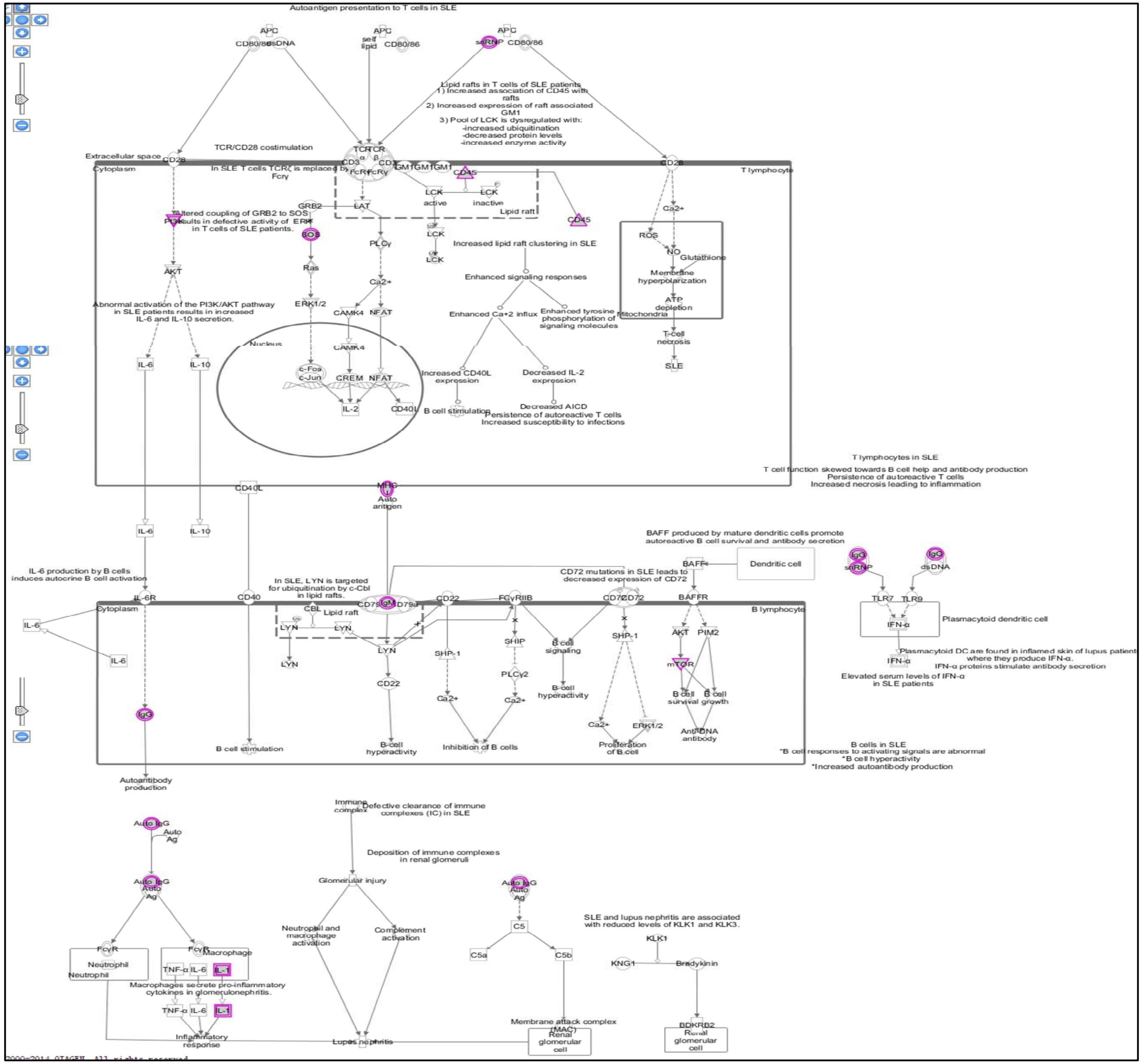

2000-2014 QIAGEN. All rights reserved.

157-GDP-mannose Biosynthesis

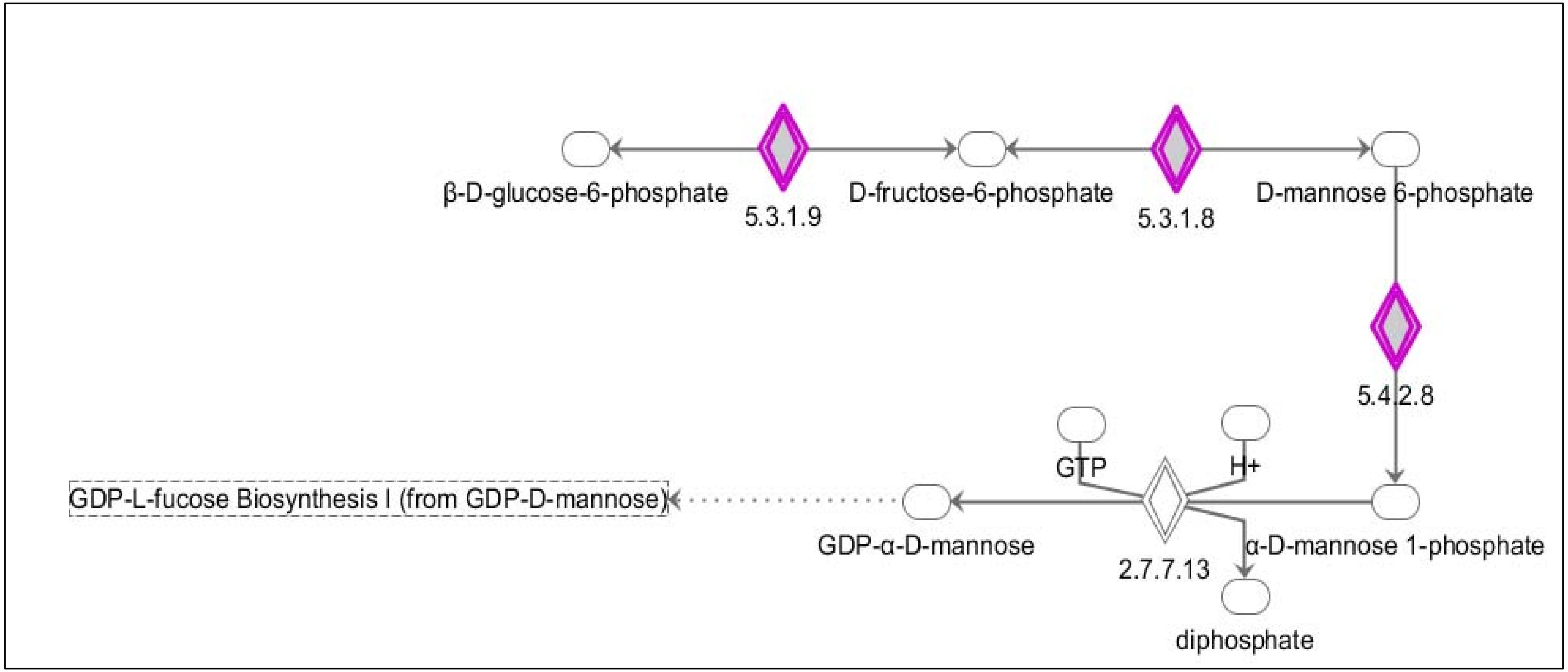

158-G α i Signaling

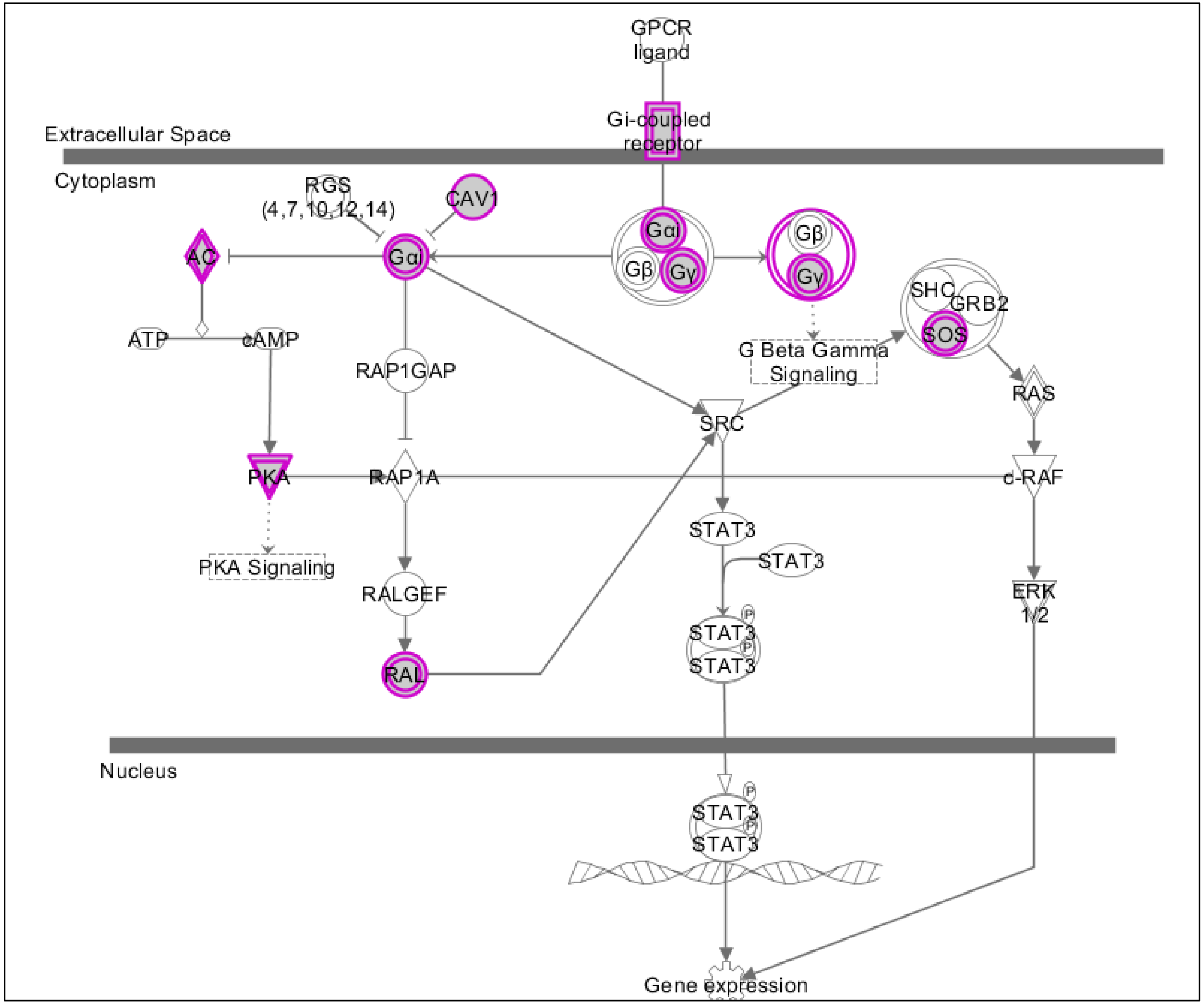

159-Methylglyoxal Degradation Ⅲ

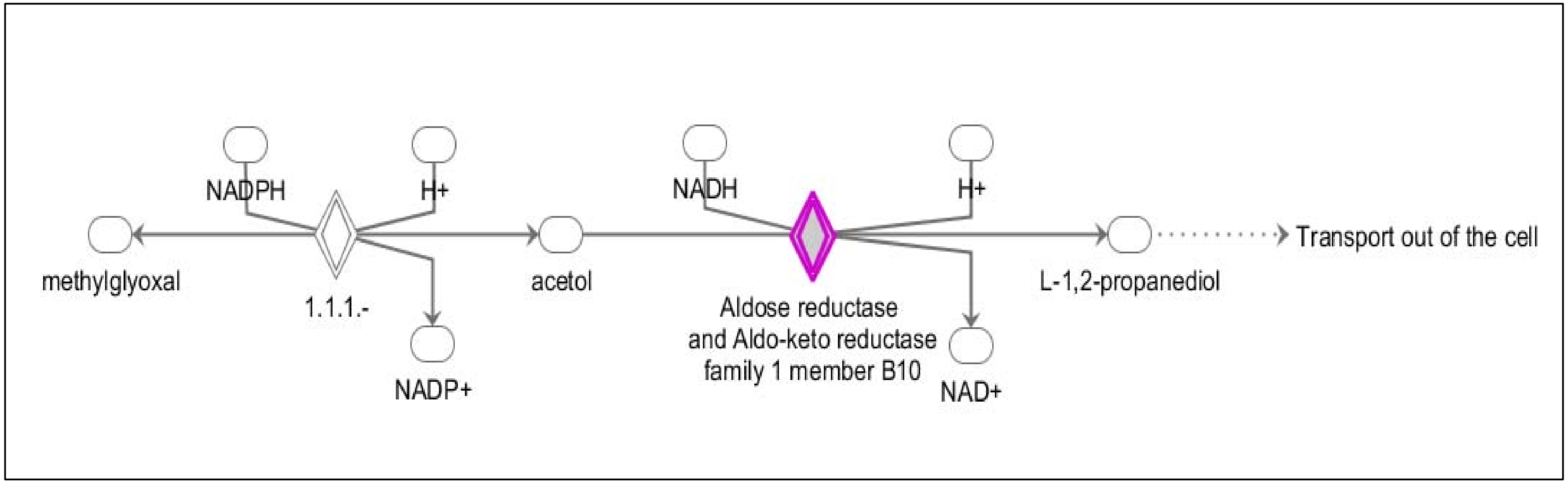

160-Purine Nucleotides De Novo Biosynthesis Ⅱ

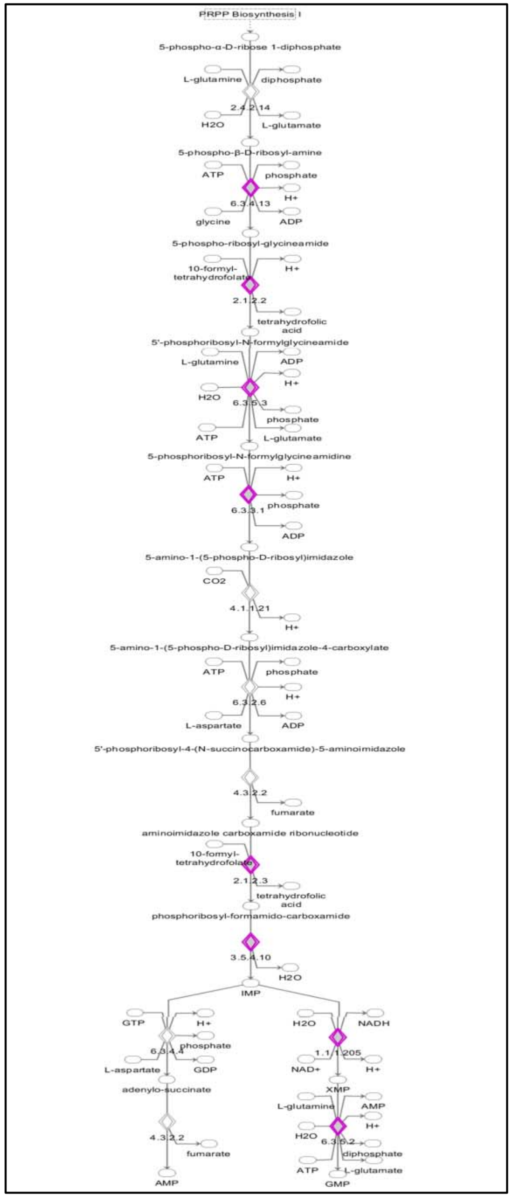

161-HIPPO Signaling

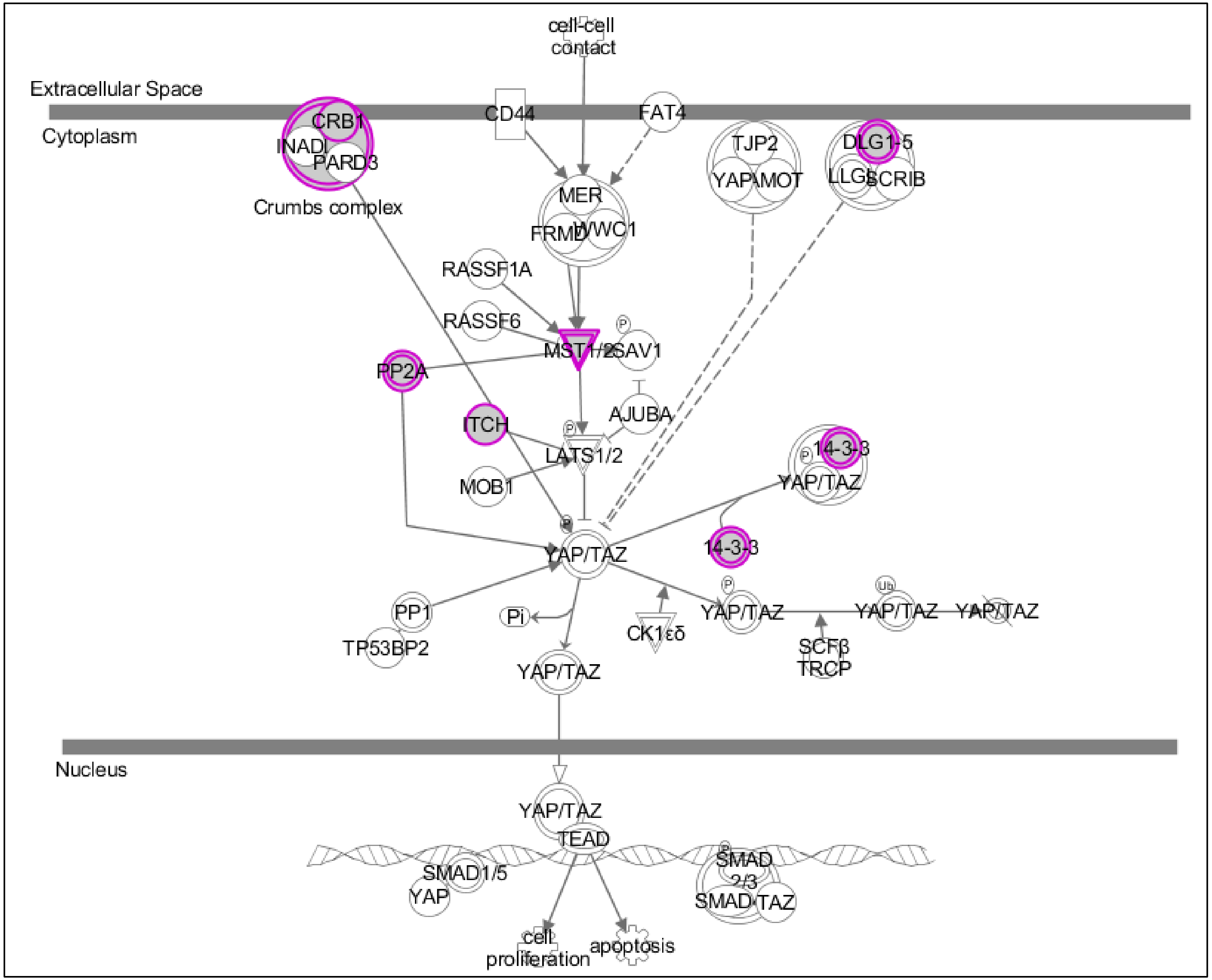

162-ERK5 Signaling

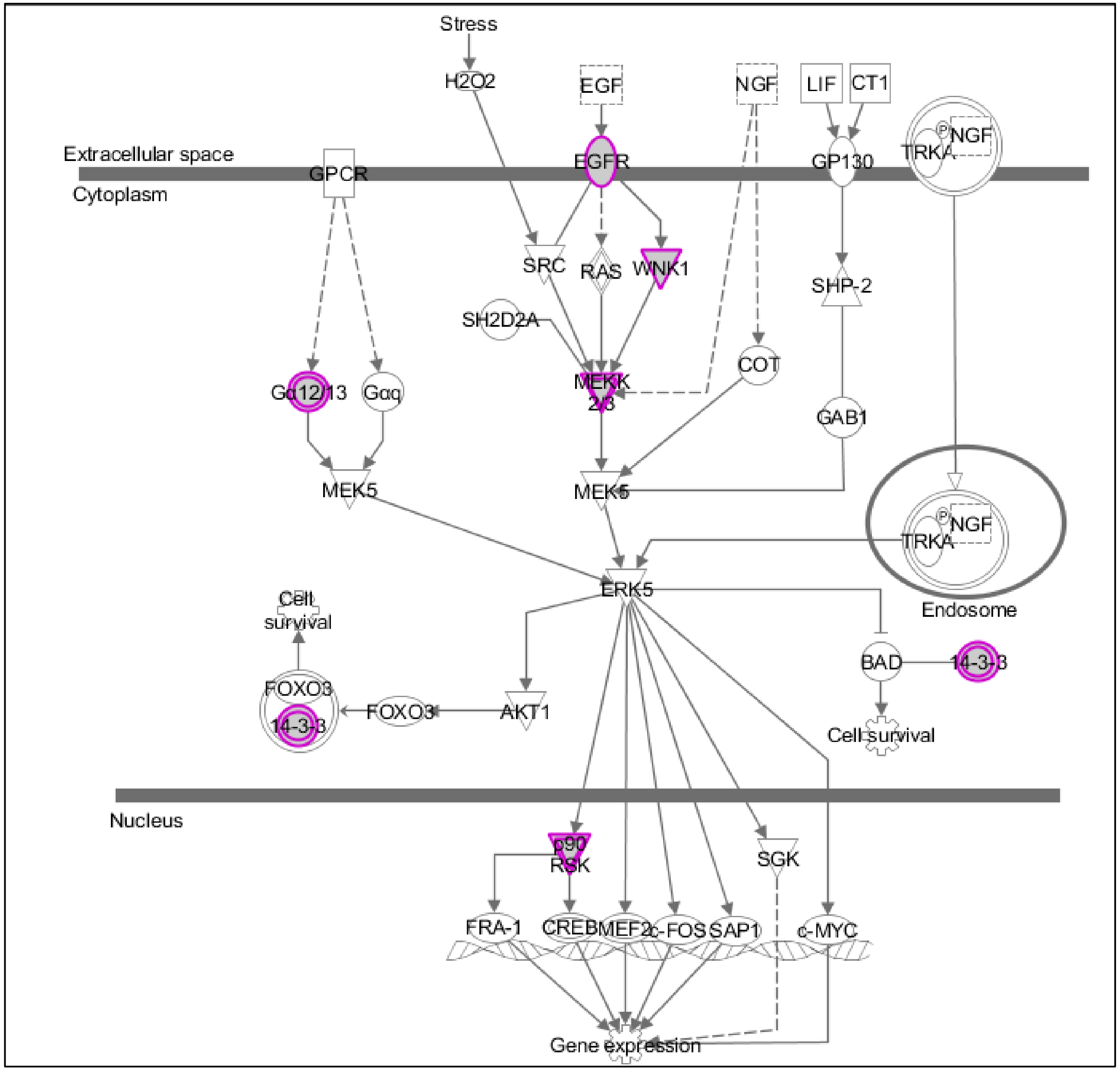

163-Phenylalanine Degradation Ⅳ(Mammalian, via Side Chain)

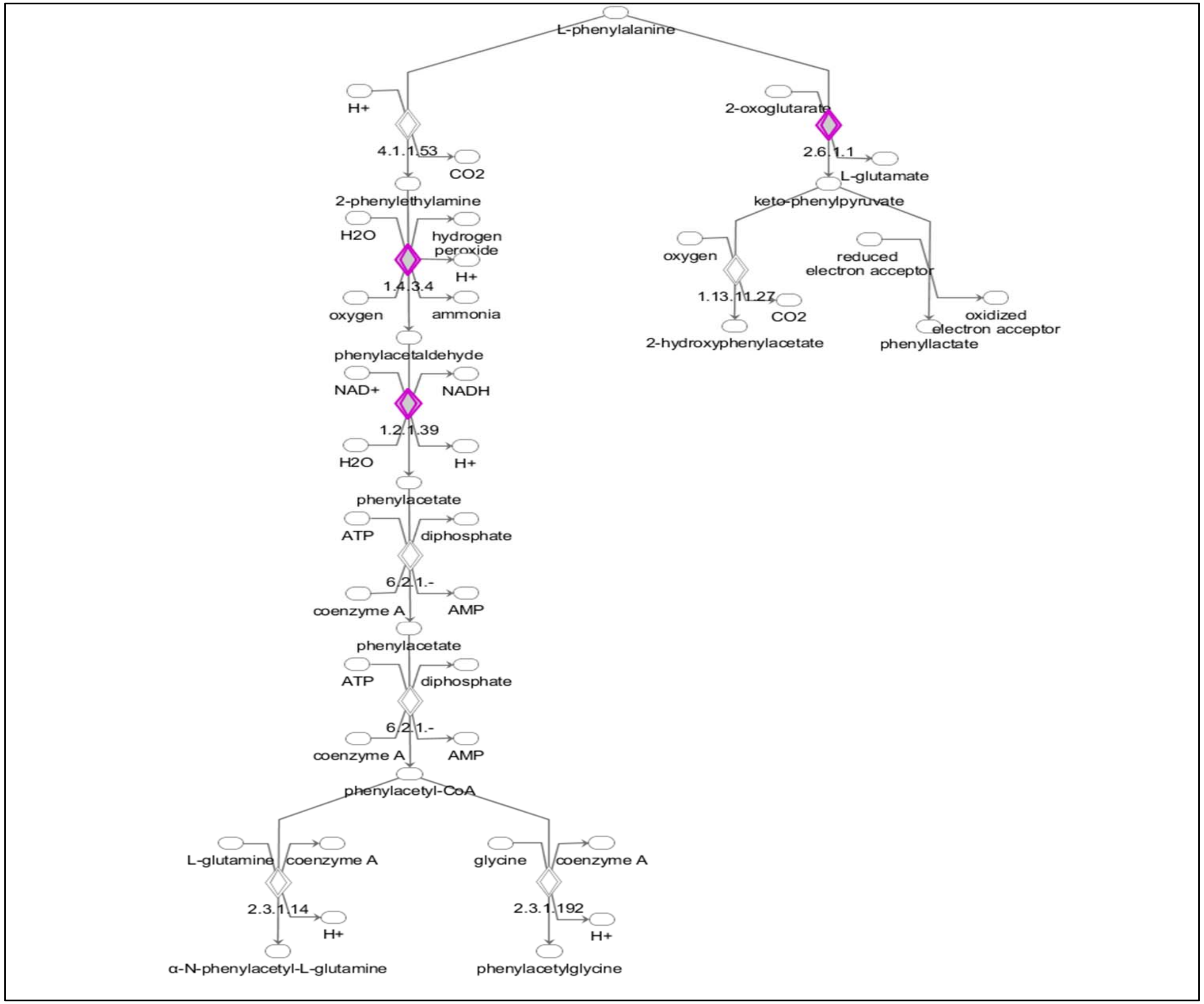

164-CTLA4 Signaling in Cytotoxic T Lymphocytes

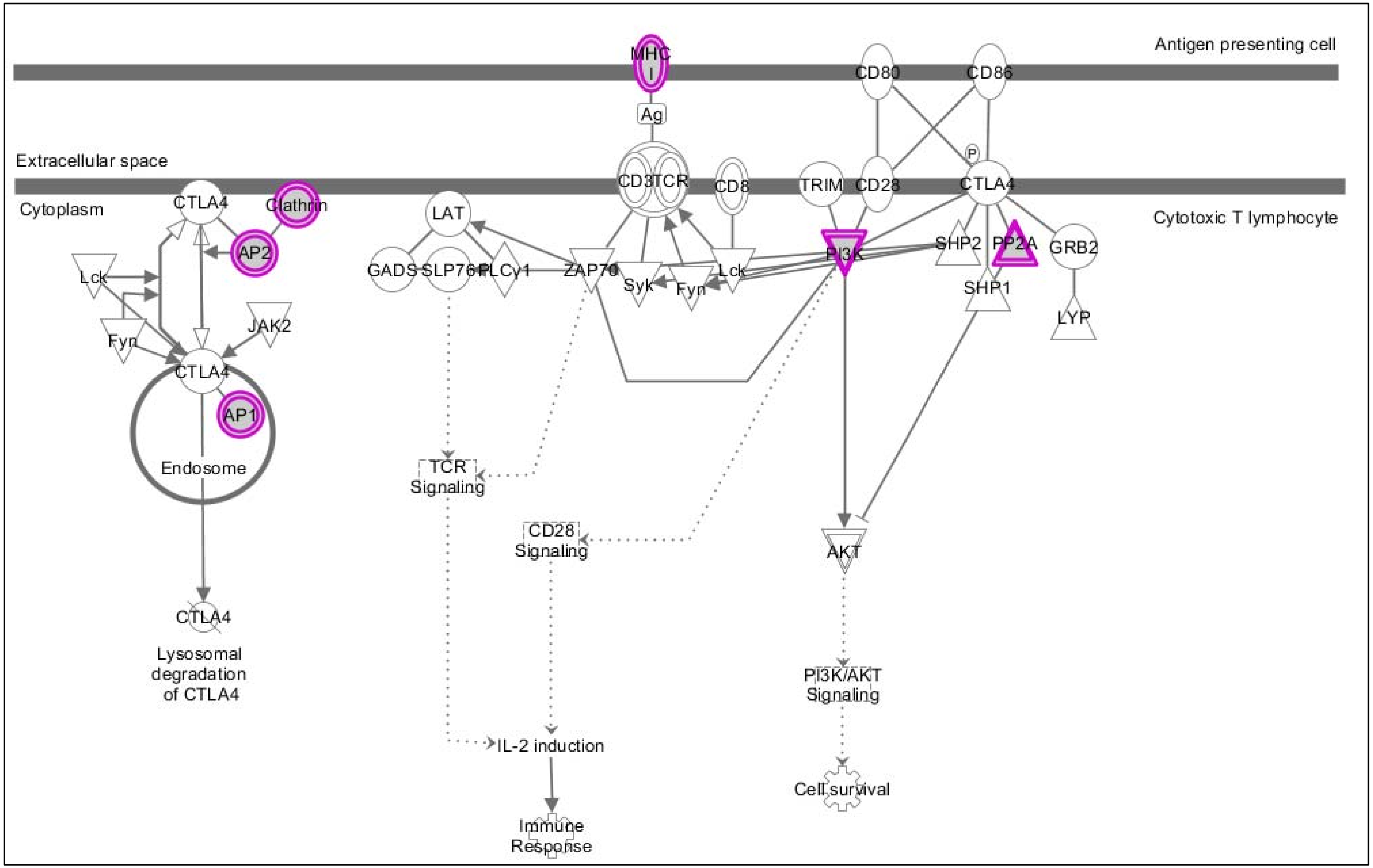

165-Lserine Degradation

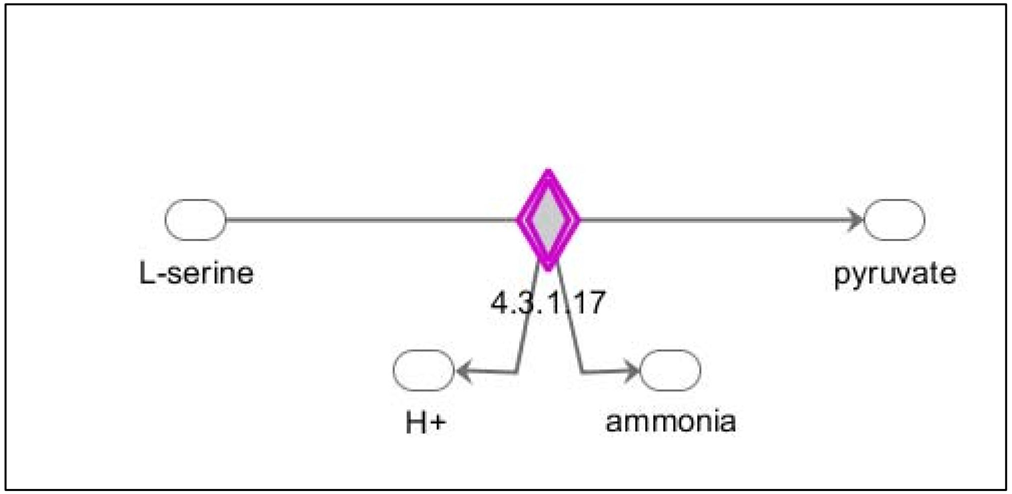

166-Hypoxia Signaling in the Cardiovascular System

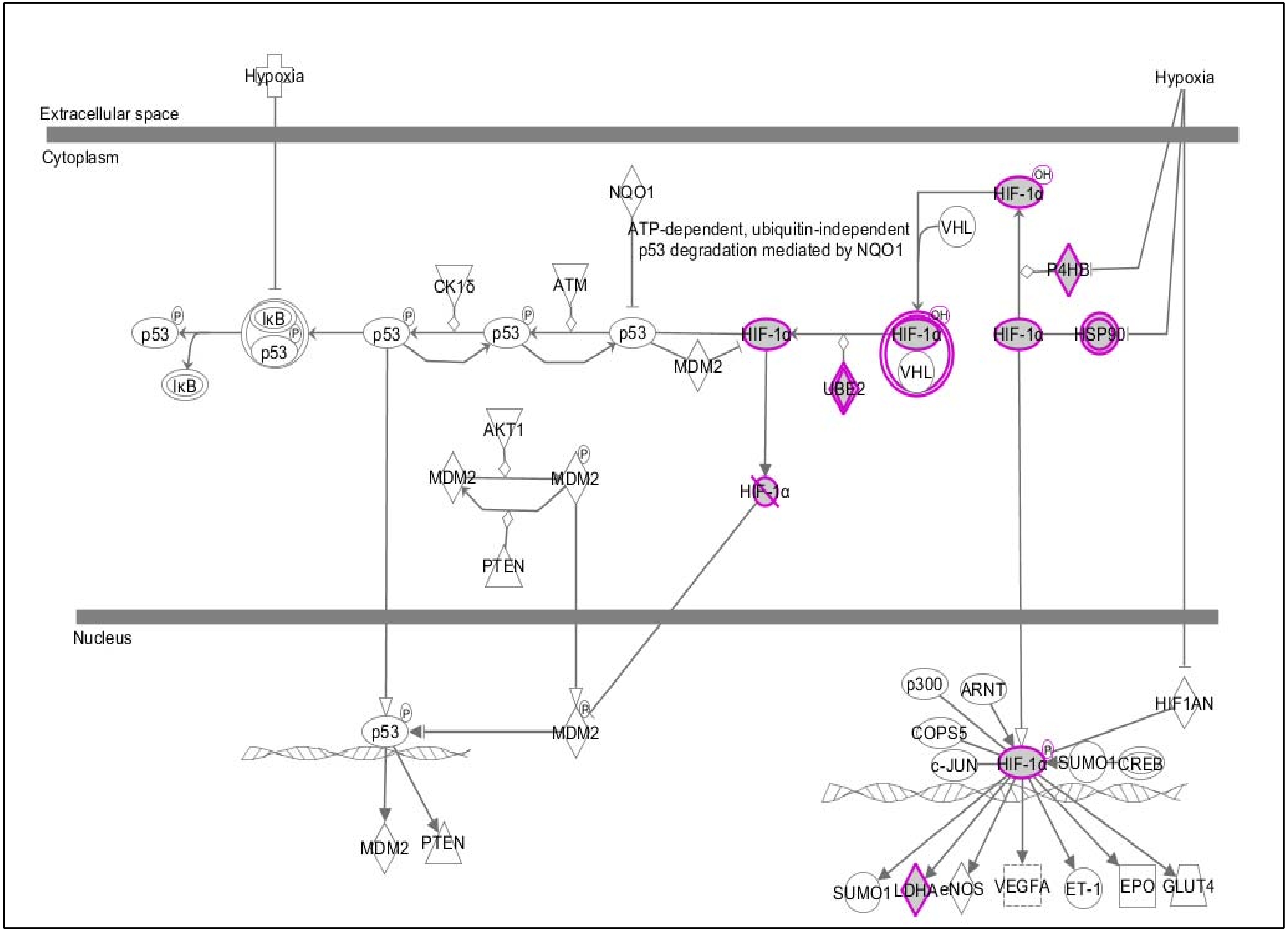

167-Gliblastma Multiforme Signaling

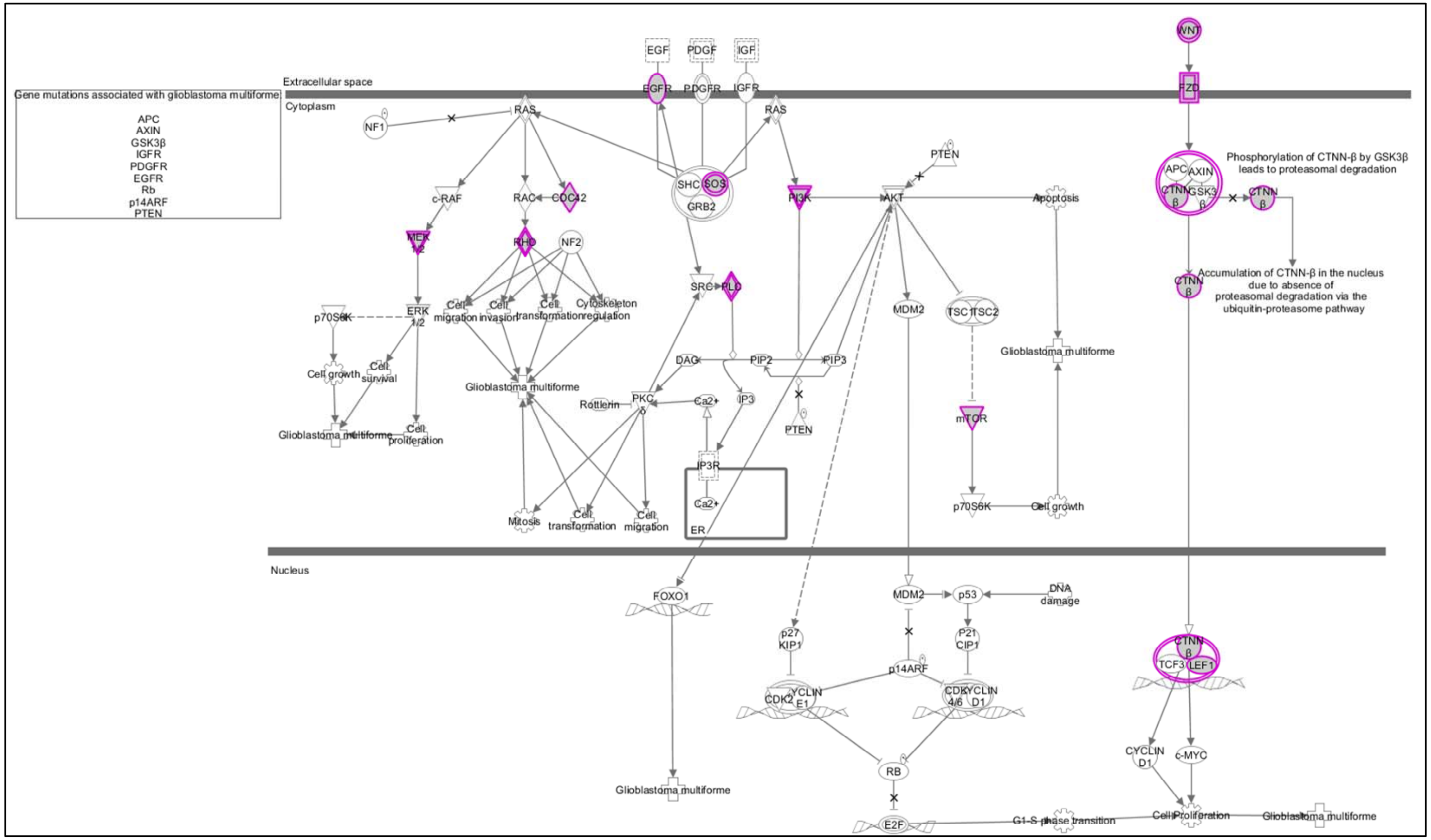

168-Galactose Degradation Ⅰ (Leloir Pathway)

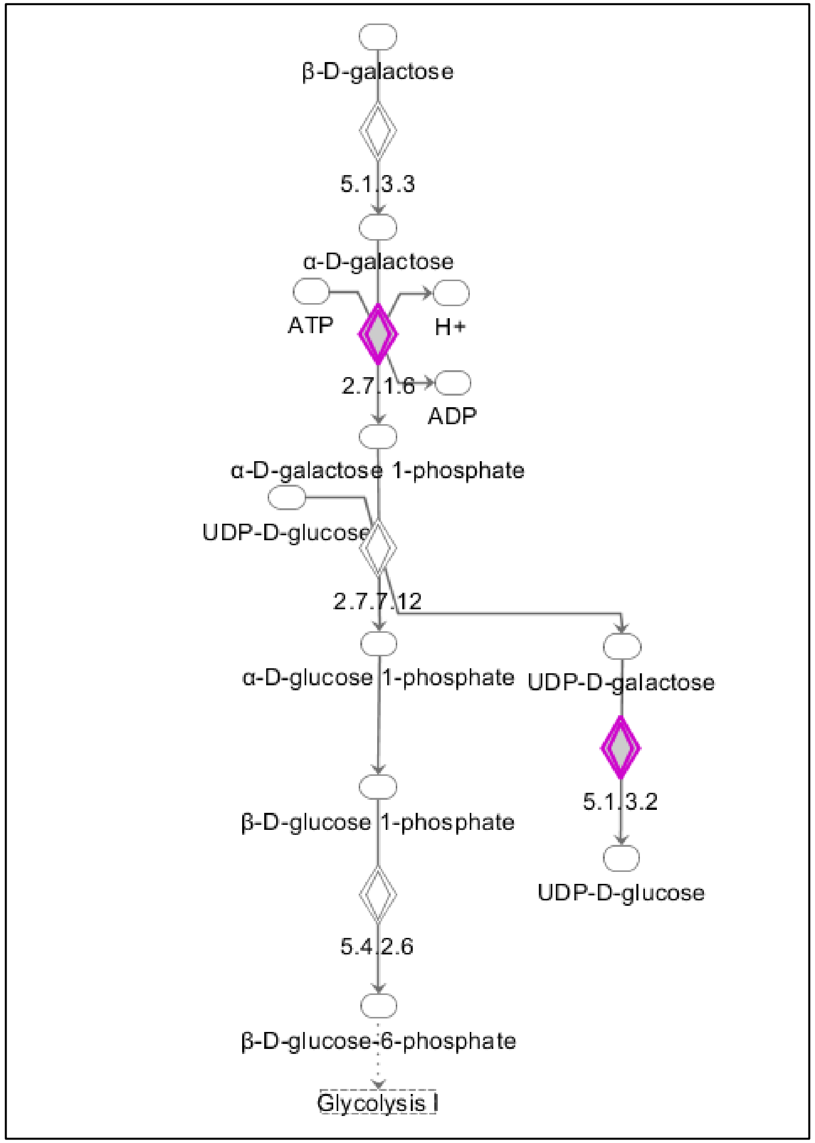

169-Agranulocyte Adhesion and Diapedesis

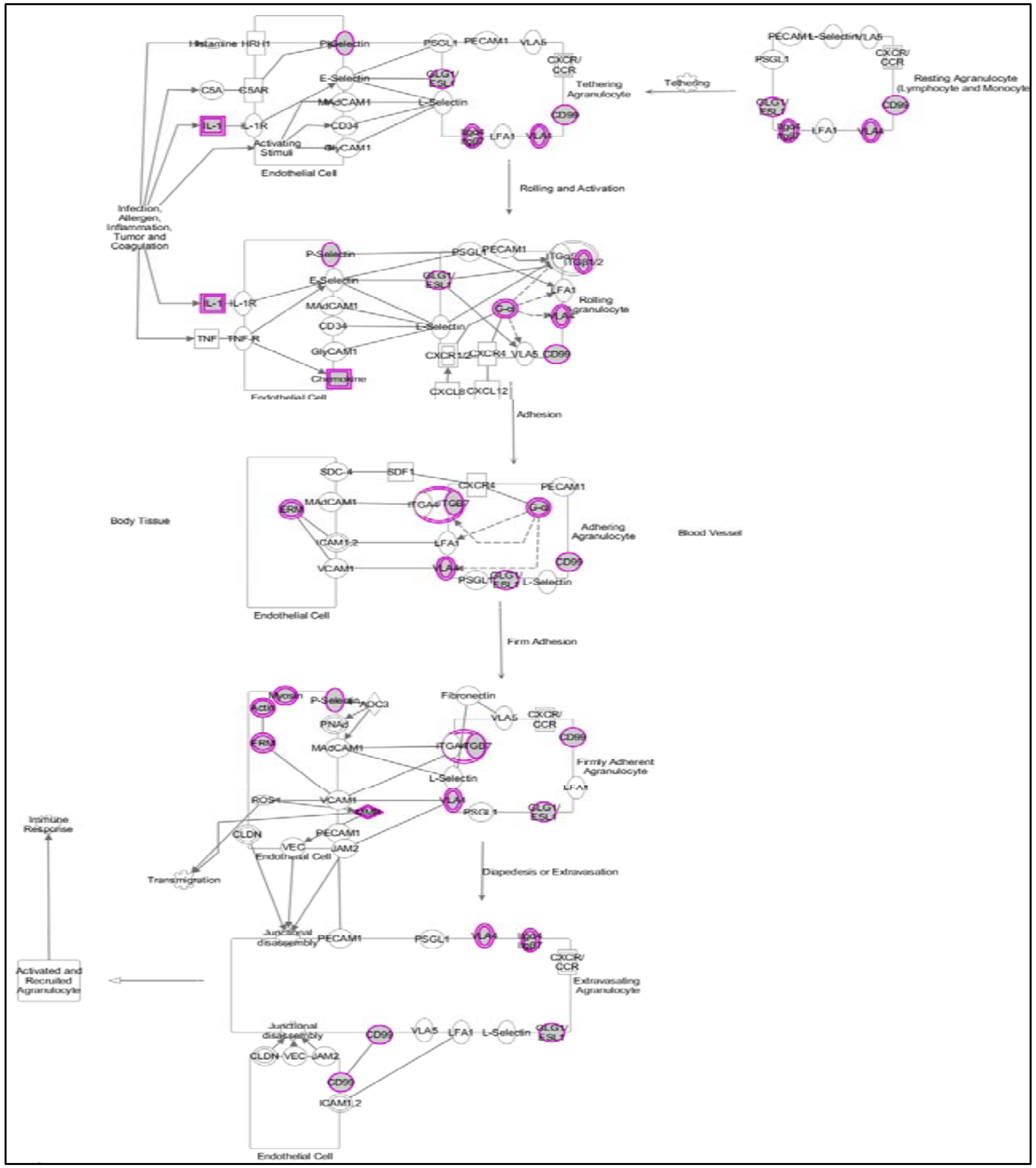

170-Synaptic Long Term Potentiation

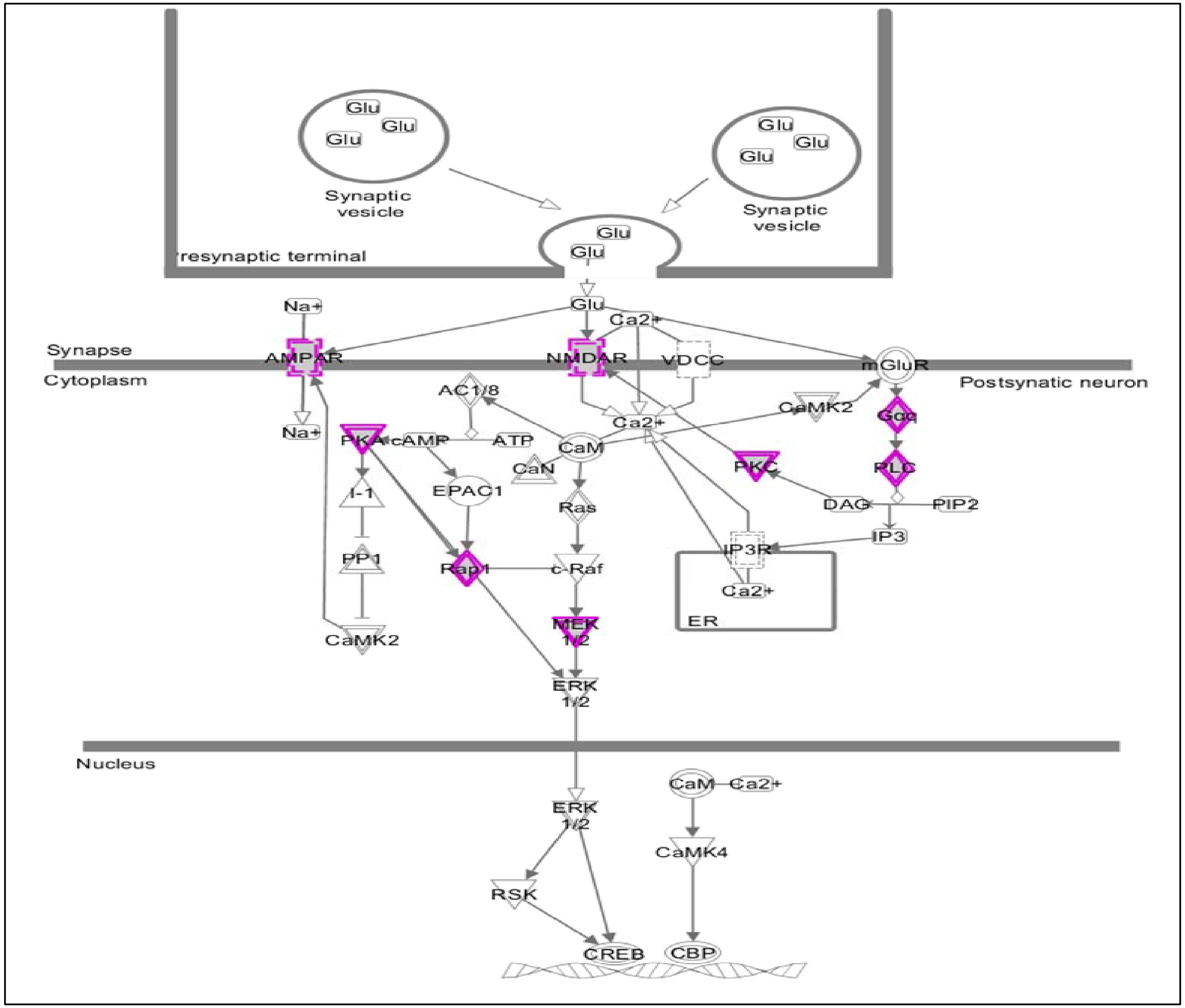

171-UDP-N-actyl-D-galactosamine Biosynthesis Ⅱ

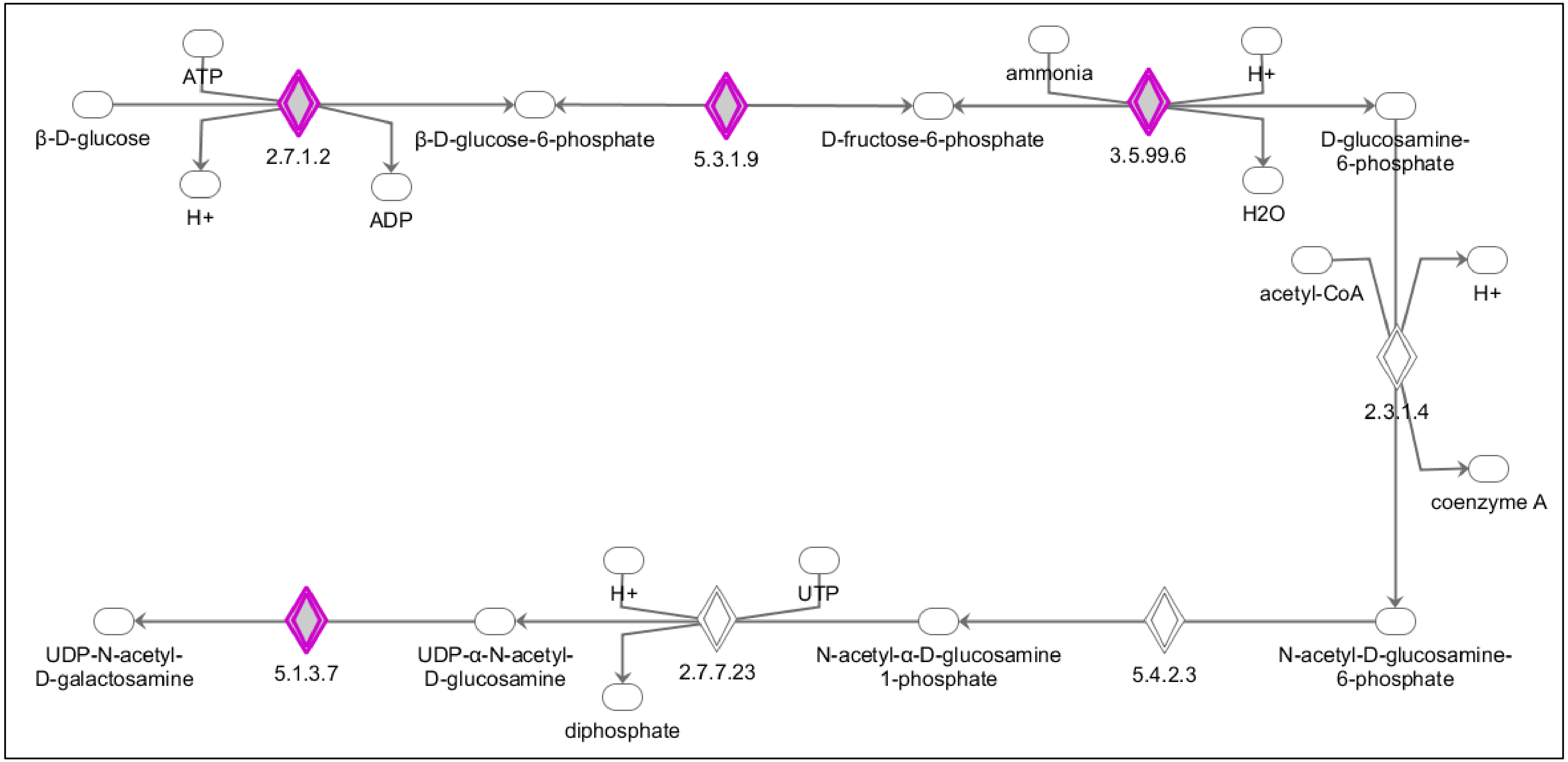

172-3-phosphoinositide Biosynthesis

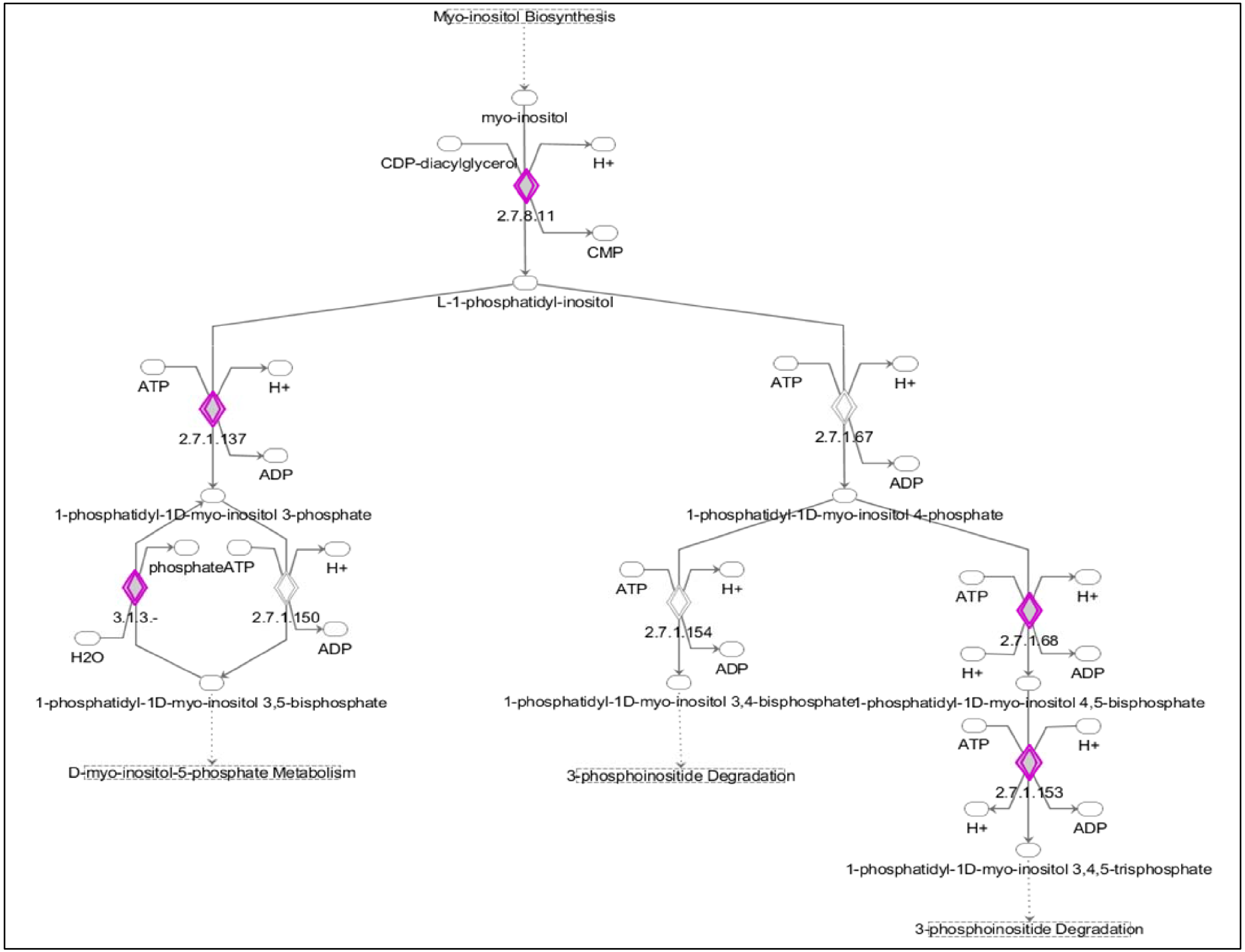

173-Coagulation System

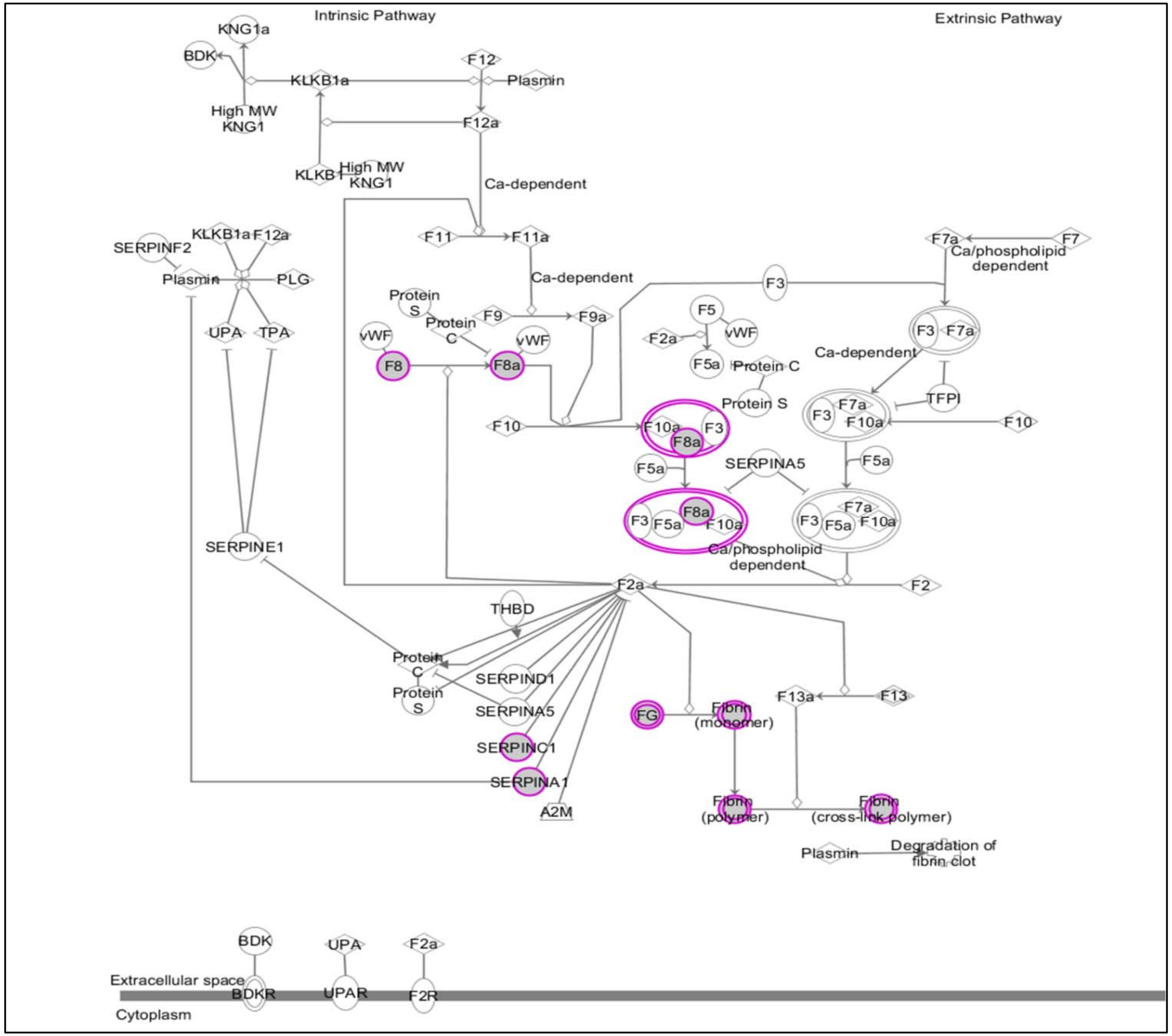

174-Aspartate Biosynthesis

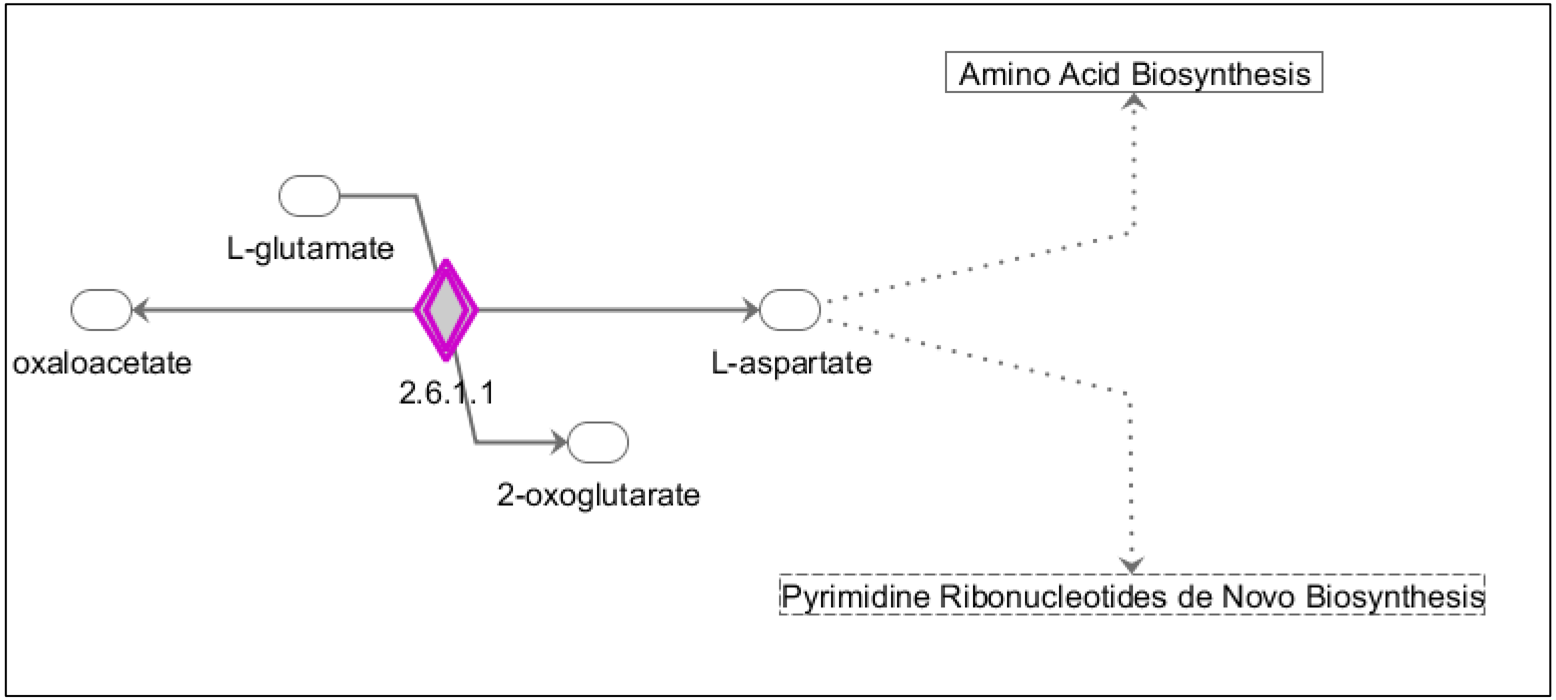

Supplement: Supplementary file 4 [file Presentation_4.zip › Supplemental materials 7.3.pdf]
