# Supplementary material for: Multiomics-Based Signaling Pathway Network Alterations in Human Non-functional Pituitary Adenomas
Source: Front Endocrinol (Lausanne). 2019 Dec 17;10:835. doi: 10.3389/fendo.2019.00835 (PMC6928143; doi:10.3389/fendo.2019.00835)
Supplement: Supplementary file 4 [file Presentation_4.zip › Supplemental materials 7.1.pdf]

Supplemental materials 7.1 Mapping proteins from pituitary controls for IPA analysis (Dataset 7)

ID	Notes	Molecules	Description	Location	Function	Drug
P01033		TIMP1	TIMP metallopeptidase inhibitor 1	Extracellular Space	cytokine	
P01236		PRL	prolactin	Extracellular Space	cytokine	
P13521		SCG2	secretogranin II	Extracellular Space	cytokine	
P14174		MIF	macrophage migration inhibitory factor (glycosylation-inhibiting factor)	Extracellular Space	cytokine	
P42830		CXCL5	chemokine (C-X-C motif) ligand 5	Extracellular Space	cytokine	
P56704		WNT3A	wingless-type MMTV integration site family, member 3A	Extracellular Space	cytokine	
Q92520		FAM3C	family with sequence similarity 3, member C	Extracellular Space	cytokine	
Q9UBP4		DKK3	dickkopf WNT signaling pathway inhibitor 3	Extracellular Space	cytokine	
Q9UHA7		IL36A	interleukin 36, alpha	Extracellular Space	cytokine	
NP_009193		PARK7	parkinson protein 7	Nucleus	enzyme	

O00116	AGPS	alkylglycerone phosphate synthase	Cytoplasm	enzyme
O00154	ACOT7	acyl-CoA thioesterase 7	Cytoplasm	enzyme
O00217	NDUFS8	NADH dehydrogenase (ubiquinone) Fe-S protein 8, 23kDa (NADH-coenzyme Q reductase)	Cytoplasm	enzyme
O00330	PDHX	pyruvate dehydrogenase complex, component X	Cytoplasm	enzyme
O00469	PLOD2	procollagen-lysine, 2-oxoglutarate 5-dioxygenase 2	Cytoplasm	enzyme
O00483	NDUFA4	NDUFA4, mitochondrial complex associated	Cytoplasm	enzyme
O00560	SDCBP	syndecan binding protein (syntenin)	Plasma Membrane	enzyme
O00571	DDX3X	DEAD (Asp-Glu-Ala-Asp) box helicase 3, X-linked	Cytoplasm	enzyme
O00763	ACACB	acetyl-CoA carboxylase beta	Cytoplasm	enzyme
O14561	NDUFAB1	NADH dehydrogenase (ubiquinone) 1, alpha/beta subcomplex, 1, 8kDa	Cytoplasm	enzyme
O14735	CDIPT	CDP-diacylglycerol--inositol 3-phosphatidyltransferase	Cytoplasm	enzyme
O14880	MGST3	microsomal glutathione S-transferase 3	Cytoplasm	enzyme

O15067	PFAS	phosphoribosylformylglycinamidine synthase	Cytoplasm	enzyme
O15270	SPTLC2	serine palmitoyltransferase, long chain base subunit 2	Cytoplasm	enzyme
O15305	PMM2	phosphomannomutase 2	Cytoplasm	enzyme
O43143	DHX15	DEAH (Asp-Glu-Ala-His) box helicase 15	Nucleus	enzyme
O43169	CYB5B	cytochrome b5 type B (outer mitochondrial membrane)	Cytoplasm	enzyme
O43175	PHGDH	phosphoglycerate dehydrogenase	Cytoplasm	enzyme
O43306	ADCY6	adenylate cyclase 6	Plasma Membrane	enzyme
O43396	TXNL1	thioredoxin-like 1	Cytoplasm	enzyme
O43488	AKR7A2	aldo-keto reductase family 7, member A2 (aflatoxin aldehyde reductase)	Cytoplasm	enzyme
O43505	B3GNT1	UDP-GlcNAc:betaGal beta-1,3-N-acetylglucosaminyltransferase 1	Cytoplasm	enzyme
O43776	NARS	asparaginyl-tRNA synthetase	Cytoplasm	enzyme
O43837	IDH3B	isocitrate dehydrogenase 3 (NAD+) beta	Cytoplasm	enzyme

O43865	AHCYL1	adenosylhomocysteinase-like 1	Cytoplasm	enzyme
O60266	ADCY3	adenylate cyclase 3	Plasma Membrane	enzyme
O60313	OPA1	optic atrophy 1 (autosomal dominant)	Cytoplasm	enzyme
O60701	UGDH	UDP-glucose 6-dehydrogenase	Nucleus	enzyme
O60762	DPM1	dolichyl-phosphate mannosyltransferase polypeptide 1, catalytic subunit	Cytoplasm	enzyme
O60832	DKC1	dyskeratosis congenita 1, dyskerin	Nucleus	enzyme
O75223	GGCT	gamma-glutamylcyclotransferase	Cytoplasm	enzyme
O75251	NDUFS7	NADH dehydrogenase (ubiquinone) Fe-S protein 7, 20kDa (NADH-coenzyme Q reductase)	Cytoplasm	enzyme
O75489	NDUFS3	NADH dehydrogenase (ubiquinone) Fe-S protein 3, 30kDa (NADH-coenzyme Q reductase)	Cytoplasm	enzyme
O75521	ECI2	enoyl-CoA delta isomerase 2	Cytoplasm	enzyme
O75608	LYPLA1	lysophospholipase I	Cytoplasm	enzyme
O75643	SNRNP200	small nuclear ribonucleoprotein 200kDa (U5)	Nucleus	enzyme

O75828	CBR3	carbonyl reductase 3	Cytoplasm	enzyme
O75874	IDH1	isocitrate dehydrogenase 1 (NADP+), soluble	Cytoplasm	enzyme
O75916	RGS9	regulator of G-protein signaling 9	Cytoplasm	enzyme
O75947	ATP5H	ATP synthase, H+ transporting, mitochondrial Fo complex, subunit d	Cytoplasm	enzyme
O94760	DDAH1	dimethylarginine dimethylaminohydrolase 1	Cytoplasm	enzyme
O94788	ALDH1A2	aldehyde dehydrogenase 1 family, member A2	Cytoplasm	enzyme
O94851	MICAL2	microtubule associated monooxygenase, calponin and LIM domain containing 2	Cytoplasm	enzyme
O95140	MFN2	mitofusin 2	Cytoplasm	enzyme
O95336	PGLS	6-phosphogluconolactonase	Cytoplasm	enzyme
O95396	MOCS3	molybdenum cofactor synthesis 3	Cytoplasm	enzyme
O95573	ACSL3	acyl-CoA synthetase long-chain family member 3	Cytoplasm	enzyme
O95674	CDS2	CDP-diacylglycerol synthase (phosphatidate cytidylyltransferase) 2	Cytoplasm	enzyme

O95865	DDAH2	dimethylarginine dimethylaminohydrolase 2	Cytoplasm	enzyme	
P00167	CYB5A	cytochrome b5 type A (microsomal)	Cytoplasm	enzyme	
P00325	ADH1B	alcohol dehydrogenase 1B (class I), beta polypeptide	Cytoplasm	enzyme	caffeine/ethanol, fomepizole, ethanol
P00338	LDHA	lactate dehydrogenase A	Cytoplasm	enzyme	
P00352	ALDH1A1	aldehyde dehydrogenase 1 family, member A1	Cytoplasm	enzyme	disulfiram, chlorpropamide
P00354	GAPDH*	glyceraldehyde-3-phosphate dehydrogenase	Cytoplasm	enzyme	
P00367	GLUD1	glutamate dehydrogenase 1	Cytoplasm	enzyme	
P00387	CYB5R3	cytochrome b5 reductase 3	Cytoplasm	enzyme	
P00403	MT-CO2	cytochrome c oxidase subunit II	Cytoplasm	enzyme	
P00441	SOD1	superoxide dismutase 1, soluble	Cytoplasm	enzyme	
P00491	PNP	purine nucleoside phosphorylase	Nucleus	enzyme	forodesine, 9-deaza-9-(3-thienylmethyl)guanine

P00492	HPRT1	hypoxanthine phosphoribosyltransferase 1	Cytoplasm	enzyme	6-mercaptopurine, thioguanine, azathioprine, 6-mercaptopurine/methotrexate/prednisone/vincristine, cyclophosphamide/cytarabine/6-mercaptopurine
P00505	GOT2	glutamic-oxaloacetic transaminase 2, mitochondrial	Cytoplasm	enzyme	
P00915	CA1	carbonic anhydrase I	Cytoplasm	enzyme	ethoxyzolamide, dichlorphenamide, brimonidine/brinzolamide, methazolamide, diazoxide, hydrochlorothiazide, acetazolamide, trichloromethiazide, dorzolamide, chlorothiazide, dorzolamide/timolol, brinzolamide, chlorothiazide/reserpine, quinethazone, chlorthalidone, benzthiazide, sulfacetamide, topiramate
P00918	CA2	carbonic anhydrase II	Cytoplasm	enzyme	ethoxyzolamide, dichlorphenamide, phentermine/topiramate, brimonidine/brinzolamide, methazolamide, diazoxide, hydrochlorothiazide, acetazolamide, trichloromethiazide, dorzolamide, chlorothiazide, dorzolamide/timolol, brinzolamide, chlorothiazide/reserpine, quinethazone, chlorthalidone, benzthiazide, sulfacetamide, topiramate
P01008	SERPINC1	serpin peptidase inhibitor, clade C (antithrombin), member 1	Extracellular Space	enzyme	heparin, enoxaparin, ardeparin, SR-123781A, glucuronyl glucosamine glycan sulfate, fondaparinux, nadroparin
P02792	FTL	ferritin, light polypeptide	Cytoplasm	enzyme	

P02794	FTH1	ferritin, heavy polypeptide 1	Cytoplasm	enzyme	
P04040	CAT	catalase	Cytoplasm	enzyme	fomepizole
P04075	ALDOA	aldolase A, fructose-bisphosphate	Cytoplasm	enzyme	
P04083	ANXA1	annexin A1	Plasma Membrane	enzyme	hydrocortisone, hydrocortisone/prednisone, hydrocortisone/mitoxantrone
P04179	SOD2	superoxide dismutase 2, mitochondrial	Cytoplasm	enzyme	
P04181	OAT	ornithine aminotransferase	Cytoplasm	enzyme	
P04406	GAPDH*	glyceraldehyde-3-phosphate dehydrogenase	Cytoplasm	enzyme	
P04424	ASL	argininosuccinate lyase	Cytoplasm	enzyme	
P04843	RPN1	ribophorin I	Cytoplasm	enzyme	
P04844	RPN2	ribophorin II	Cytoplasm	enzyme	
P05091	ALDH2	aldehyde dehydrogenase 2 family (mitochondrial)	Cytoplasm	enzyme	disulfiram, chlorpropamide
P05455	SSB	Sjogren syndrome antigen B (autoantigen La)	Nucleus	enzyme	

P06132	UROD	uroporphyrinogen decarboxylase	Cytoplasm	enzyme	
P06276	BCHE	butyrylcholinesterase	Plasma Membrane	enzyme	dipivefrin, malathion, atropine/edrophonium, echothiophate, tacrine, edrophonium, isoflurophate, pyridostigmine, pralidoxime, rivastigmine
P06733	ENO1*	enolase 1, (alpha)	Cytoplasm	enzyme	
P06744	GPI	glucose-6-phosphate isomerase	Extracellular Space	enzyme	
P06858	LPL	lipoprotein lipase	Cytoplasm	enzyme	atorvastatin/niacin, nicotinic acid/pioglitazone, nicotinic acid, tyloxapol, lovastatin/niacin
P06865	HEXA	hexosaminidase A (alpha polypeptide)	Cytoplasm	enzyme	
P07195	LDHB	lactate dehydrogenase B	Cytoplasm	enzyme	
P07203	GPX1	glutathione peroxidase 1	Cytoplasm	enzyme	
P07237	P4HB	prolyl 4-hydroxylase, beta polypeptide	Cytoplasm	enzyme	
P07686	HEXB	hexosaminidase B (beta polypeptide)	Cytoplasm	enzyme	
P07741	APRT	adenine phosphoribosyltransferase	Cytoplasm	enzyme	

P07814	EPRS	glutamyl-prolyl-tRNA synthetase	Cytoplasm	enzyme	
P07900	HSP90AA1	heat shock protein 90kDa alpha (cytosolic), class A member 1	Cytoplasm	enzyme	alvespimycin, retaspimycin, luminespib, cisplatin
P07954	FH	fumarate hydratase	Cytoplasm	enzyme	
P08236	GUSB	glucuronidase, beta	Cytoplasm	enzyme	
P08238	HSP90AB1	heat shock protein 90kDa alpha (cytosolic), class B member 1	Cytoplasm	enzyme	alvespimycin, retaspimycin, cisplatin
P08559	PDHA1	pyruvate dehydrogenase (lipoamide) alpha 1	Cytoplasm	enzyme	
P08574	CYC1	cytochrome c-1	Cytoplasm	enzyme	
P09104	ENO2	enolase 2 (gamma, neuronal)	Cytoplasm	enzyme	
P09110	ACAA1	acetyl-CoA acyltransferase 1	Cytoplasm	enzyme	
P09211	GSTP1	glutathione S-transferase pi 1	Cytoplasm	enzyme	
P09417	QDPR	quinoid dihydropteridine reductase	Cytoplasm	enzyme	
P09471	GNAO1	guanine nucleotide binding protein (G protein), alpha activating activity polypeptide O	Plasma Membrane	enzyme	

P09488	GSTM1	glutathione S-transferase mu 1	Cytoplasm	enzyme	
P09543	CNP	2',3'-cyclic nucleotide 3' phosphodiesterase	Cytoplasm	enzyme	
P09622	DLD	dihydrolipoamide dehydrogenase	Cytoplasm	enzyme	hexachlorophene
P09874	PARP1	poly (ADP-ribose) polymerase 1	Nucleus	enzyme	veliparib, olaparib, INO-1001
P09960	LTA4H	leukotriene A4 hydrolase	Cytoplasm	enzyme	
P09972	ALDOC	aldolase C, fructose-bisphosphate	Cytoplasm	enzyme	
P10253	GAA	glucosidase, alpha; acid	Cytoplasm	enzyme	miglitol, acarbose
P10515	DLAT	dihydrolipoamide S-acetyltransferase	Cytoplasm	enzyme	
P10599	TXN	thioredoxin	Cytoplasm	enzyme	
P10606	COX5B	cytochrome c oxidase subunit Vb	Cytoplasm	enzyme	
P10768	ESD	esterase D	Cytoplasm	enzyme	
P10809	HSPD1	heat shock 60kDa protein 1 (chaperonin)	Cytoplasm	enzyme	

P11021	HSPA5	heat shock 70kDa protein 5 (glucose-regulated protein, 78kDa)	Cytoplasm	enzyme
P11142	HSPA8	heat shock 70kDa protein 8	Cytoplasm	enzyme
P11177	PDHB	pyruvate dehydrogenase (lipoamide) beta	Cytoplasm	enzyme
P11216	PYGB	phosphorylase, glycogen; brain	Cytoplasm	enzyme
P11233	RALA	v-ral simian leukemia viral oncogene homolog A (ras related)	Cytoplasm	enzyme
P11310	ACADM	acyl-CoA dehydrogenase, C-4 to C-12 straight chain	Cytoplasm	enzyme

P11387	TOP1	topoisomerase (DNA) I	Nucleus	enzyme	elsamitrucin, T 0128, polyglutamate camptothecin, elomotecan, tafluposide, cetuximab/irinotecan, capecitabine/irinotecan, 5-fluorouracil/irinotecan, cisplatin/topotecan, cyclophosphamide/topotecan, cyclophosphamide/topotecan/vincristine, irinotecan/temozolomide, irinotecan/vincristine, cyclophosphamide/irinotecan/temozolomide, irinotecan/temozolomide/vincristine, bevacizumab/irinotecan, bevacizumab/irinotecan/oxaliplatin, aflibercept/irinotecan, irinotecan/oxaliplatin, cyclophosphamide/temozolomide/topotecan, docetaxel/irinotecan, capecitabine/irinotecan/oxaliplatin, bevacizumab/capecitabine/irinotecan/oxaliplatin, ifosfamide/topotecan, 5-fluorouracil/irinotecan/oxaliplatin, gemcitabine/irinotecan, TAS-103, beta-lapachone, irinotecan, 7-ethyl-10-hydroxy-camptothecin, topotecan, 9-amino-20-camptothecin, stibogluconic acid, rubitecan, gimatecan, karenitecin
P11413	G6PD	glucose-6-phosphate dehydrogenase	Cytoplasm	enzyme	
P11498	PC	pyruvate carboxylase	Cytoplasm	enzyme	
P11766	ADH5	alcohol dehydrogenase 5 (class III), chi polypeptide	Cytoplasm	enzyme	

P12004	PCNA	proliferating cell nuclear antigen	Nucleus	enzyme	
P12081	HARS	histidyl-tRNA synthetase	Cytoplasm	enzyme	
P12268	IMPDH2	IMP (inosine 5'-monophosphate) dehydrogenase 2	Cytoplasm	enzyme	thioguanine, VX-944, pegintron/ribavirin, interferon alfa-2b/ribavirin, mycophenolate mofetil, mycophenolic acid, ribavirin
P12956	XRCC6	X-ray repair complementing defective repair in Chinese hamster cells 6	Nucleus	enzyme	
P13010	XRCC5	X-ray repair complementing defective repair in Chinese hamster cells 5 (double-strand-break rejoining)	Nucleus	enzyme	
P13073	COX4I1	cytochrome c oxidase subunit IV isoform 1	Cytoplasm	enzyme	
P13667	PDIA4	protein disulfide isomerase family A, member 4	Cytoplasm	enzyme	
P13716	ALAD	aminolevulinate dehydratase	Cytoplasm	enzyme	delta-aminolevulinic acid
P14314	PRKCSH	protein kinase C substrate 80K-H	Cytoplasm	enzyme	
P14406	COX7A2	cytochrome c oxidase subunit VIIa polypeptide 2 (liver)	Cytoplasm	enzyme	
P14410	SI	sucrase-isomaltase (alpha-glucosidase)	Cytoplasm	enzyme	acarbose
P14550	AKR1A1	aldo-keto reductase family 1, member A1 (aldehyde reductase)	Cytoplasm	enzyme	

P14621	ACYP2	acylphosphatase 2, muscle type	Cytoplasm	enzyme	
P14868	DARS	aspartyl-tRNA synthetase	Cytoplasm	enzyme	
P15121	AKR1B1	aldo-keto reductase family 1, member B1 (aldose reductase)	Cytoplasm	enzyme	sorbinil
P16083	NQO2	NAD(P)H dehydrogenase, quinone 2	Cytoplasm	enzyme	
P16152	CBR1	carbonyl reductase 1	Cytoplasm	enzyme	
P16435	POR	P450 (cytochrome) oxidoreductase	Cytoplasm	enzyme	daunorubicin/tretinoin, cisplatin/doxorubicin/methotrexate, cisplatin/doxorubicin/ifosfamide/methotrexate, cisplatin/doxorubicin, carboplatin/doxorubicin, dacarbazine/doxorubicin, doxorubicin/ifosfamide, doxorubicin/streptozocin, dacarbazine/doxorubicin/ifosfamide, doxorubicin/tretinoin, nitazoxanide, doxorubicin, daunorubicin
P16930	FAH	fumarylacetoacetate hydrolase (fumarylacetoacetase)	Cytoplasm	enzyme	
P17066	HSPA6	heat shock 70kDa protein 6 (HSP70B')	Nucleus	enzyme	
P17174	GOT1	glutamic-oxaloacetic transaminase 1, soluble	Cytoplasm	enzyme	

P18206	VCL	vinculin	Plasma Membrane	enzyme	
P19086	GNAZ	guanine nucleotide binding protein (G protein), alpha z polypeptide	Plasma Membrane	enzyme	
P19388	POLR2E	polymerase (RNA) II (DNA directed) polypeptide E, 25kDa	Nucleus	enzyme	
P19835	CEL	carboxyl ester lipase	Extracellular Space	enzyme	
P20132	SDS	serine dehydratase	Cytoplasm	enzyme	
P20336	RAB3A	RAB3A, member RAS oncogene family	Cytoplasm	enzyme	
P20337	RAB3B	RAB3B, member RAS oncogene family	Cytoplasm	enzyme	
P20839	IMPDH1	IMP (inosine 5'-monophosphate) dehydrogenase 1	Cytoplasm	enzyme	thioguanine, VX-944, pegintron/ribavirin, PEG-interferon alfa-2a/ribavirin, interferon alfa-2b/ribavirin, mycophenolate mofetil, mycophenolic acid, ribavirin
P20933	AGA	aspartylglucosaminidase	Cytoplasm	enzyme	
P21266	GSTM3	glutathione S-transferase mu 3 (brain)	Cytoplasm	enzyme	
P21580	TNFAIP3	tumor necrosis factor, alpha-induced protein 3	Nucleus	enzyme	

P21912	SDHB	succinate dehydrogenase complex, subunit B, iron sulfur (Ip)	Cytoplasm	enzyme	
P21953	BCKDHB	branched chain keto acid dehydrogenase E1, beta polypeptide	Cytoplasm	enzyme	
P21980	TGM2	transglutaminase 2	Cytoplasm	enzyme	
P22061	PCMT1	protein-L-isoaspartate (D-aspartate) O-methyltransferase	Cytoplasm	enzyme	
P22102	GART	phosphoribosylglycinamide formyltransferase, phosphoribosylglycinamide synthetase, phosphoribosylaminoimidazole synthetase	Cytoplasm	enzyme	bevacizumab/pemetrexed, pemetrexed
P22314	UBA1	ubiquitin-like modifier activating enzyme 1	Cytoplasm	enzyme	
P22352	GPX3	glutathione peroxidase 3 (plasma)	Extracellular Space	enzyme	
P22695	UQCRC2	ubiquinol-cytochrome c reductase core protein II	Cytoplasm	enzyme	
P22712	ENO1*	enolase 1, (alpha)	Cytoplasm	enzyme	
P22748	CA4	carbonic anhydrase IV	Plasma Membrane	enzyme	ethoxyzolamide, dichlorphenamide, phentermine/topiramate, methazolamide, hydrochlorothiazide, acetazolamide, trichloromethiazide, chlorothiazide, chlorothiazide/reserpine, chlorthalidone, benzthiazide, sulfacetamide, topiramate
P23284	PPIB	peptidylprolyl isomerase B (cyclophilin B)	Cytoplasm	enzyme	

P23368	ME2	malic enzyme 2, NAD(+)-dependent, mitochondrial	Cytoplasm	enzyme	
P23381	WARS	tryptophanyl-tRNA synthetase	Cytoplasm	enzyme	
P23396	RPS3	ribosomal protein S3	Cytoplasm	enzyme	
P23434	GCSH	glycine cleavage system protein H (aminomethyl carrier)	Cytoplasm	enzyme	
P23526	AHCY	adenosylhomocysteinase	Cytoplasm	enzyme	9-(2',3'-dihydroxycyclopent-4'-enyl)adenine, 3-deazaneplanocin, neplanocin A, 3-deazaaristeromycin, cyanovirin-N, 6'-C-methylneplanocin A
P24310	COX7A1	cytochrome c oxidase subunit VIIa polypeptide 1 (muscle)	Cytoplasm	enzyme	
P24311	COX7B	cytochrome c oxidase subunit VIIb	Cytoplasm	enzyme	
P24462	CYP3A7	cytochrome P450, family 3, subfamily A, polypeptide 7	Cytoplasm	enzyme	cobicistat, cobicistat/elvitegravir/ emtricitabine/tenofovir disoproxil
P24752	ACAT1	acetyl-CoA acetyltransferase 1	Cytoplasm	enzyme	
P24855	DNASE1	deoxyribonuclease I	Extracellular Space	enzyme	
P25325	MPST	mercaptopyruvate sulfurtransferase	Cytoplasm	enzyme	

P26440	IVD	isovaleryl-CoA dehydrogenase	Cytoplasm	enzyme	
P26599	PTBP1	polypyrimidine tract binding protein 1	Nucleus	enzyme	
P26639	TARS	threonyl-tRNA synthetase	Nucleus	enzyme	
P26640	VARS	valyl-tRNA synthetase	Cytoplasm	enzyme	
P26885	FKBP2	FK506 binding protein 2, 13kDa	Cytoplasm	enzyme	
P27338	MAOB	monoamine oxidase B	Cytoplasm	enzyme	safinamide, ladostigil, rasagiline, phentermine/topiramate, fenfluramine/phentermine, pargyline, methamphetamine, selegiline, dextroamphetamine, procainamide, tranylcypromine, phenelzine, isocarboxazid, phentermine, benzphetamine
P27695	APEX1	APEX nuclease (multifunctional DNA repair enzyme) 1	Nucleus	enzyme	
P27708	CAD	carbamoyl-phosphate synthetase 2, aspartate transcarbamylase, and dihydroorotase	Cytoplasm	enzyme	
P28161	GSTM2	glutathione S-transferase mu 2 (muscle)	Cytoplasm	enzyme	
P28331	NDUFS1	NADH dehydrogenase (ubiquinone) Fe-S protein 1, 75kDa (NADH-coenzyme Q reductase)	Cytoplasm	enzyme	

P28845	HSD11B1	hydroxysteroid (11-beta) dehydrogenase 1	Cytoplasm	enzyme	
P29401	TKT	transketolase	Cytoplasm	enzyme	
P29992	GNA11	guanine nucleotide binding protein (G protein), alpha 11 (Gq class)	Plasma Membrane	enzyme	
P30041	PRDX6	peroxiredoxin 6	Cytoplasm	enzyme	
P30043	BLVRB	biliverdin reductase B (flavin reductase (NADPH))	Cytoplasm	enzyme	
P30044	PRDX5	peroxiredoxin 5	Cytoplasm	enzyme	auranofin
P30046	DDT	D-dopachrome tautomerase	Cytoplasm	enzyme	
P30048	PRDX3	peroxiredoxin 3	Cytoplasm	enzyme	
P30084	ECHS1	enoyl CoA hydratase, short chain, 1, mitochondrial	Cytoplasm	enzyme	
P30876	POLR2B	polymerase (RNA) II (DNA directed) polypeptide B, 140kDa	Nucleus	enzyme	
P31040	SDHA	succinate dehydrogenase complex, subunit A, flavoprotein (Fp)	Cytoplasm	enzyme	
P31930	UQCRC1	ubiquinol-cytochrome c reductase core protein I	Cytoplasm	enzyme	

P31937	HIBADH	3-hydroxyisobutyrate dehydrogenase	Cytoplasm	enzyme	
P31939	ATIC	5-aminoimidazole-4-carboxamide ribonucleotide formyltransferase/IMP cyclohydrolase	Cytoplasm	enzyme	pemetrexed
P32119	PRDX2	peroxiredoxin 2	Cytoplasm	enzyme	
P34949	MPI	mannose phosphate isomerase	Cytoplasm	enzyme	
P35270	SPR	sepiapterin reductase (7,8-dihydrobiopterin:NADP+ oxidoreductase)	Cytoplasm	enzyme	
P35579	MYH9	myosin, heavy chain 9, non-muscle	Cytoplasm	enzyme	
P36269	GGT5	gamma-glutamyltransferase 5	Plasma Membrane	enzyme	
P36404	ARL2	ADP-ribosylation factor-like 2	Cytoplasm	enzyme	
P36405	ARL3	ADP-ribosylation factor-like 3	Cytoplasm	enzyme	
P36578	RPL4	ribosomal protein L4	Cytoplasm	enzyme	
P36871	PGM1	phosphoglucomutase 1	Cytoplasm	enzyme	
P36957	DLST	dihydrolipoamide S-succinyltransferase (E2 component of 2-oxo-glutarate complex)	Cytoplasm	enzyme	

P36969	GPX4	glutathione peroxidase 4	Cytoplasm	enzyme
P37287	PIGA	phosphatidylinositol glycan anchor biosynthesis, class A	Cytoplasm	enzyme
P37837	TALDO1	transaldolase 1	Cytoplasm	enzyme
P38919	EIF4A3	eukaryotic translation initiation factor 4A3	Nucleus	enzyme
P39656	DDOST	dolichyl-diphosphooligosaccharide--protein glycosyltransferase subunit (non-catalytic)	Cytoplasm	enzyme
P40617	ARL4A	ADP-ribosylation factor-like 4A	Nucleus	enzyme
P40925	MDH1	malate dehydrogenase 1, NAD (soluble)	Cytoplasm	enzyme
P40926	MDH2	malate dehydrogenase 2, NAD (mitochondrial)	Cytoplasm	enzyme
P40939	HADHA	hydroxyacyl-CoA dehydrogenase/3-ketoacyl-CoA thiolase/enoyl-CoA hydratase (trifunctional protein), alpha subunit	Cytoplasm	enzyme
P41250	GARS	glycyl-tRNA synthetase	Cytoplasm	enzyme
P41252	IARS	isoleucyl-tRNA synthetase	Cytoplasm	enzyme
P42126	ECI1	enoyl-CoA delta isomerase 1	Cytoplasm	enzyme

P42765	ACAA2	acetyl-CoA acyltransferase 2	Cytoplasm	enzyme	
P43034	PAFAH1B1	platelet-activating factor acetylhydrolase 1b, regulatory subunit 1 (45kDa)	Cytoplasm	enzyme	
P43246	MSH2	mutS homolog 2	Nucleus	enzyme	
P45954	ACADSB	acyl-CoA dehydrogenase, short/branched chain	Cytoplasm	enzyme	
P46439	GSTM5	glutathione S-transferase mu 5	Cytoplasm	enzyme	
P46926	GNPDA1	glucosamine-6-phosphate deaminase 1	Cytoplasm	enzyme	
P47897	QARS	glutaminyl-tRNA synthetase	Cytoplasm	enzyme	
P47985	UQCRFS1	ubiquinol-cytochrome c reductase, Rieske iron-sulfur polypeptide 1	Cytoplasm	enzyme	
P48163	ME1	malic enzyme 1, NADP(+)-dependent, cytosolic	Cytoplasm	enzyme	
P48735	IDH2	isocitrate dehydrogenase 2 (NADP+), mitochondrial	Cytoplasm	enzyme	
P49189	ALDH9A1	aldehyde dehydrogenase 9 family, member A1	Cytoplasm	enzyme	
P49327	FASN	fatty acid synthase	Cytoplasm	enzyme	orlistat, cerulenin

P49419	ALDH7A1	aldehyde dehydrogenase 7 family, member A1	Cytoplasm	enzyme
P49588	AARS	alanyl-tRNA synthetase	Cytoplasm	enzyme
P49748	ACADVL	acyl-CoA dehydrogenase, very long chain	Cytoplasm	enzyme
P49773	HINT1	histidine triad nucleotide binding protein 1	Nucleus	enzyme
P49789	FHIT	fragile histidine triad	Cytoplasm	enzyme
P49792	RANBP2	RAN binding protein 2	Nucleus	enzyme
P49915	GMPS	guanine monphosphate synthase	Nucleus	enzyme
P50213	IDH3A	isocitrate dehydrogenase 3 (NAD+) alpha	Cytoplasm	enzyme
P50225	SULT1A1	sulfotransferase family, cytosolic, 1A, phenol-preferring, member 1	Cytoplasm	enzyme
P50897	PPT1	palmitoyl-protein thioesterase 1	Cytoplasm	enzyme
P50990	CCT8	chaperonin containing TCP1, subunit 8 (theta)	Cytoplasm	enzyme
P51148	RAB5C	RAB5C, member RAS oncogene family	Cytoplasm	enzyme

P51149	RAB7A	RAB7A, member RAS oncogene family	Cytoplasm	enzyme	
P51153	RAB13	RAB13, member RAS oncogene family	Plasma Membrane	enzyme	
P51159	RAB27A	RAB27A, member RAS oncogene family	Cytoplasm	enzyme	
P51160	PDE6C	phosphodiesterase 6C, cGMP-specific, cone, alpha prime	Cytoplasm	enzyme	
P51580	TPMT	thiopurine S-methyltransferase	Cytoplasm	enzyme	olsalazine
P51648	ALDH3A2	aldehyde dehydrogenase 3 family, member A2	Cytoplasm	enzyme	
P51659	HSD17B4	hydroxysteroid (17-beta) dehydrogenase 4	Cytoplasm	enzyme	
P52209	PGD	phosphogluconate dehydrogenase	Cytoplasm	enzyme	
P53004	BLVRA	biliverdin reductase A	Cytoplasm	enzyme	
P53396	ACLY	ATP citrate lyase	Cytoplasm	enzyme	
P53597	SUCLG1	succinate-CoA ligase, alpha subunit	Cytoplasm	enzyme	
P54577	YARS	tyrosyl-tRNA synthetase	Cytoplasm	enzyme	

P55072	VCP	valosin containing protein	Cytoplasm	enzyme
P55084	HADHB	hydroxyacyl-CoA dehydrogenase/3-ketoacyl-CoA thiolase/enoyl-CoA hydratase (trifunctional protein), beta subunit	Cytoplasm	enzyme
P55265	ADAR	adenosine deaminase, RNA-specific	Nucleus	enzyme
P55268	LAMB2	laminin, beta 2 (laminin S)	Extracellular Space	enzyme
P55809	OXCT1	3-oxoacid CoA transferase 1	Cytoplasm	enzyme
P59768	GNG2	guanine nucleotide binding protein (G protein), gamma 2	Plasma Membrane	enzyme
P60174	TPI1	triosephosphate isomerase 1	Cytoplasm	enzyme
P60953	CDC42	cell division cycle 42	Cytoplasm	enzyme
P61019	RAB2A	RAB2A, member RAS oncogene family	Cytoplasm	enzyme
P61026	RAB10	RAB10, member RAS oncogene family	Cytoplasm	enzyme
P61081	UBE2M	ubiquitin-conjugating enzyme E2M	Cytoplasm	enzyme
P61088	UBE2N	ubiquitin-conjugating enzyme E2N	Cytoplasm	enzyme

P61106	RAB14	RAB14, member RAS oncogene family	Cytoplasm	enzyme	
P61224	RAP1B	RAP1B, member of RAS oncogene family	Cytoplasm	enzyme	
P61586	RHOA	ras homolog family member A	Cytoplasm	enzyme	
P61604	HSPE1	heat shock 10kDa protein 1	Cytoplasm	enzyme	
P78345	RPP38	ribonuclease P/MRP 38kDa subunit	Nucleus	enzyme	
P78417	GSTO1	glutathione S-transferase omega 1	Cytoplasm	enzyme	
P80404	ABAT	4-aminobutyrate aminotransferase	Cytoplasm	enzyme	valproic acid, vigabatrin
P83876	TXNL4A	thioredoxin-like 4A	Nucleus	enzyme	
P98160	HSPG2	heparan sulfate proteoglycan 2	Extracellular Space	enzyme	
Q00688	FKBP3	FK506 binding protein 3, 25kDa	Nucleus	enzyme	
Q01433	AMPD2	adenosine monophosphate deaminase 2	Cytoplasm	enzyme	
Q01970	PLCB3	phospholipase C, beta 3 (phosphatidylinositol-specific)	Cytoplasm	enzyme	

Q02218	OGDH	oxoglutarate (alpha-ketoglutarate) dehydrogenase (lipoamide)	Cytoplasm	enzyme	
Q02252	ALDH6A1	aldehyde dehydrogenase 6 family, member A1	Cytoplasm	enzyme	
Q02880	TOP2B	topoisomerase (DNA) II beta 180kDa	Nucleus	enzyme	novobiocin, etoposide, CPI-0004Na, becatecarin, elsamitrucin, AQ4N, elomotecan, tafluposide, cyclophosphamide/epirubicin/5-fluorouracil, cytarabine/daunorubicin, hydrocortisone/mitoxantrone, mitoxantrone/prednisone, cladribine/cytarabine/daunorubicin, cytarabine/daunorubicin/tretinoin, arsenic trioxide/daunorubicin/tretinoin, cytarabine/etoposide, cytarabine/etoposide/mitoxantrone, cytarabine/mitoxantrone, daunorubicin/tretinoin, cisplatin/doxorubicin/methotrexate, cyclophosphamide/epirubicin, cyclophosphamide/etoposide, cisplatin/doxorubicin/ifosfamide/methotrexate, cisplatin/doxorubicin, cyclophosphamide/docetaxel/epirubicin/5-fluorouracil/trastuzumab, clofarabine/cyclophosphamide/etoposide, cyclophosphamide/epirubicin/vincristine, carboplatin/doxorubicin, cladribine/cytarabine/filgrastim/mitoxantrone, epirubicin/5-fluorouracil/oxaliplatin, dacarbazine/doxorubicin, capecitabine/epirubicin/oxaliplatin, doxorubicin/ifosfamide, doxorubicin/streptozocin, dacarbazine/doxorubicin/ifosfamide, dexamethasone/fludarabine phosphate/mitoxantrone, cyclophosphamide/etoposi

Q03113	GNA12	guanine nucleotide binding protein (G protein) alpha 12	Plasma Membrane	enzyme
Q04446	GBE1	glucan (1,4-alpha-), branching enzyme 1	Cytoplasm	enzyme
Q04828	AKR1C1/AKR1C2	aldo-keto reductase family 1, member C2	Cytoplasm	enzyme
Q05193	DNM1	dynamin 1	Cytoplasm	enzyme
Q06210	GFPT1	glutamine--fructose-6-phosphate transaminase 1	Cytoplasm	enzyme
Q06830	PRDX1	peroxiredoxin 1	Cytoplasm	enzyme
Q07131	UGP2	UDP-glucose pyrophosphorylase 2	Cytoplasm	enzyme

Q07864	POLE	polymerase (DNA directed), epsilon, catalytic subunit	Nucleus	enzyme	nelarabine, bortezomib/cladribine/rituximab, gemcitabine/paclitaxel, cladribine/cytarabine/daunorubicin, gemcitabine/oxaliplatin, gemcitabine/vinorelbine, docetaxel/gemcitabine, docetaxel/gemcitabine/vincristine, cladribine/cytarabine/filgrastim, clofarabine/cyclophosphamide/etoposide, capecitabine/gemcitabine, gemcitabine/oxaliplatin/paclitaxel, cladribine/cytarabine/filgrastim/mitoxantrone, clofarabine/cytarabine/filgrastim, cladribine/rituximab, capecitabine/docetaxel/gemcitabine, cladribine/cytarabine/filgrastim/idarubicin, bevacizumab/gemcitabine, gemcitabine/oxaliplatin/rituximab, L-asparaginase/gemcitabine/oxaliplatin, cladribine/cytarabine/filgrastim/idarubicin/plerixafor, gemcitabine/irinotecan, 5-fluorouracil/gemcitabine, clofarabine/filgrastim, gemcitabine, clofarabine, trifluridine, cladribine
Q07973	CYP24A1	cytochrome P450, family 24, subfamily A, polypeptide 1	Cytoplasm	enzyme	
Q08211	DHX9	DEAH (Asp-Glu-Ala-His) box helicase 9	Nucleus	enzyme	
Q08257	CRYZ	crystallin, zeta (quinone reductase)	Cytoplasm	enzyme	

Q09028	RBBP4	retinoblastoma binding protein 4	Nucleus	enzyme	
Q12797	ASPH	aspartate beta-hydroxylase	Cytoplasm	enzyme	
Q12873	CHD3	chromodomain helicase DNA binding protein 3	Nucleus	enzyme	
Q13011	ECH1	enoyl CoA hydratase 1, peroxisomal	Cytoplasm	enzyme	
Q13085	ACACA	acetyl-CoA carboxylase alpha	Cytoplasm	enzyme	
Q13093	PLA2G7	phospholipase A2, group VII (platelet-activating factor acetylhydrolase, plasma)	Extracellular Space	enzyme	darapladib
Q13126	MTAP	methylthioadenosine phosphorylase	Nucleus	enzyme	
Q13162	PRDX4	peroxiredoxin 4	Cytoplasm	enzyme	
Q13371	PDCL	phosducin-like	Cytoplasm	enzyme	
Q13472	TOP3A	topoisomerase (DNA) III alpha	Nucleus	enzyme	
Q13510	ASAH1	N-acylsphingosine amidohydrolase (acid ceramidase) 1	Cytoplasm	enzyme	
Q13618	CUL3	cullin 3	Nucleus	enzyme	

Q13630	TSTA3	tissue specific transplantation antigen P35B	Plasma Membrane	enzyme
Q13724	MOGS	mannosyl-oligosaccharide glucosidase	Cytoplasm	enzyme
Q13838	DDX39B	DEAD (Asp-Glu-Ala-Asp) box polypeptide 39B	Nucleus	enzyme
Q14194	CRMP1	collapsin response mediator protein 1	Cytoplasm	enzyme
Q14353	GAMT	guanidinoacetate N-methyltransferase	Cytoplasm	enzyme
Q14376	GALE	UDP-galactose-4-epimerase	Cytoplasm	enzyme
Q14554	PDIA5	protein disulfide isomerase family A, member 5	Cytoplasm	enzyme
Q14894	CRYM	crystallin, mu	Cytoplasm	enzyme
Q14914	PTGR1	prostaglandin reductase 1	Cytoplasm	enzyme
Q15008	PSMD6	proteasome (prosome, macropain) 26S subunit, non-ATPase, 6	Cytoplasm	enzyme
Q15019	SEPT2	septin 2	Cytoplasm	enzyme
Q15029	EFTUD2	elongation factor Tu GTP binding domain containing 2	Nucleus	enzyme

Q15084	PDIA6	protein disulfide isomerase family A, member 6	Cytoplasm	enzyme
Q15102	PAFAH1B3	platelet-activating factor acetylhydrolase 1b, catalytic subunit 3 (29kDa)	Cytoplasm	enzyme
Q15181	PPA1	pyrophosphatase (inorganic) 1	Cytoplasm	enzyme
Q15185	PTGES3	prostaglandin E synthase 3 (cytosolic)	Cytoplasm	enzyme
Q15738	NSDHL	NAD(P) dependent steroid dehydrogenase-like	Cytoplasm	enzyme
Q15843	NEDD8	neural precursor cell expressed, developmentally down-regulated 8	Nucleus	enzyme
Q16555	DPYSL2	dihydropyrimidinase-like 2	Cytoplasm	enzyme
Q16698	DECR1	2,4-dienoyl CoA reductase 1, mitochondrial	Cytoplasm	enzyme
Q16769	QPCT	glutaminyl-peptide cyclotransferase	Cytoplasm	enzyme
Q16795	NDUFA9	NADH dehydrogenase (ubiquinone) 1 alpha subcomplex, 9, 39kDa	Cytoplasm	enzyme
Q16836	HADH	hydroxyacyl-CoA dehydrogenase	Cytoplasm	enzyme
Q16878	CDO1	cysteine dioxygenase type 1	Cytoplasm	enzyme

Q16881	TXNRD1	thioredoxin reductase 1	Cytoplasm	enzyme	arsenic trioxide/tretinoin, arsenic trioxide/daunorubicin/tretinoin, arsenic trioxide/gemtuzumab ozogamicin/tretinoin, arsenic trioxide/idarubicin/tretinoin, arsenic trioxide
Q86V15	CASZ1	castor zinc finger 1	Nucleus	enzyme	
Q8IY37	DHX37	DEAH (Asp-Glu-Ala-His) box polypeptide 37	Other	enzyme	
Q8NCC3	PLA2G15	phospholipase A2, group XV	Cytoplasm	enzyme	
Q8TEA8	DTD1	D-tyrosyl-tRNA deacylase 1	Cytoplasm	enzyme	
Q8WUD1	RAB2B	RAB2B, member RAS oncogene family	Plasma Membrane	enzyme	
Q8WYQ5	DGCR8	DGCR8 microprocessor complex subunit	Nucleus	enzyme	
Q92499	DDX1	DEAD (Asp-Glu-Ala-Asp) box helicase 1	Nucleus	enzyme	
Q92506	HSD17B8	hydroxysteroid (17-beta) dehydrogenase 8	Cytoplasm	enzyme	
Q92562	FIG4	FIG4 phosphoinositide 5-phosphatase	Cytoplasm	enzyme	
Q92830	KAT2A	K(lysine) acetyltransferase 2A	Cytoplasm	enzyme	

Q92841	DDX17	DEAD (Asp-Glu-Ala-Asp) box helicase 17	Nucleus	enzyme
Q92900	UPF1	UPF1 regulator of nonsense transcripts homolog (yeast)	Nucleus	enzyme
Q92903	CDS1	CDP-diacylglycerol synthase (phosphatidate cytidylyltransferase) 1	Cytoplasm	enzyme
Q92945	KHSRP	KH-type splicing regulatory protein	Nucleus	enzyme
Q96E17	RAB3C	RAB3C, member RAS oncogene family	Cytoplasm	enzyme
Q96FW1	OTUB1	OTU deubiquitinase, ubiquitin aldehyde binding 1	Cytoplasm	enzyme
Q96IU4	ABHD14B	abhydrolase domain containing 14B	Cytoplasm	enzyme
Q96J02	ITCH	itchy E3 ubiquitin protein ligase	Nucleus	enzyme
Q96MG8	PCMTD1	protein-L-isoaspartate (D-aspartate) O-methyltransferase domain containing 1	Cytoplasm	enzyme
Q99714	HSD17B10	hydroxysteroid (17-beta) dehydrogenase 10	Cytoplasm	enzyme
Q99798	ACO2	aconitase 2, mitochondrial	Cytoplasm	enzyme
Q9BPW8	NIPSNAP1	nipsnap homolog 1 (C. elegans)	Cytoplasm	enzyme

Q9BS26	ERP44	endoplasmic reticulum protein 44	Cytoplasm	enzyme
Q9BY32	ITPA	inosine triphosphatase (nucleoside triphosphate pyrophosphatase)	Cytoplasm	enzyme
Q9GZR7	DDX24	DEAD (Asp-Glu-Ala-Asp) box helicase 24	Nucleus	enzyme
Q9GZT4	SRR	serine racemase	Cytoplasm	enzyme
Q9GZX7	AICDA	activation-induced cytidine deaminase	Cytoplasm	enzyme
Q9H1E5	TMX4	thioredoxin-related transmembrane protein 4	Cytoplasm	enzyme
Q9H3N1	TMX1	thioredoxin-related transmembrane protein 1	Cytoplasm	enzyme
Q9H4K7	MTG2	mitochondrial ribosome-associated GTPase 2	Cytoplasm	enzyme
Q9H867	VCPKMT	valosin containing protein lysine (K) methyltransferase	Other	enzyme
Q9HAQ2	KIF9	kinesin family member 9	Cytoplasm	enzyme
Q9HBH5	RDH14	retinol dehydrogenase 14 (all-trans/9-cis/11-cis)	Other	enzyme
Q9HCE1	MOV10	Mov10 RISC complex RNA helicase	Nucleus	enzyme

Q9HCK8	CHD8	chromodomain helicase DNA binding protein 8	Nucleus	enzyme
Q9HDC9	APMAP	adipocyte plasma membrane associated protein	Plasma Membrane	enzyme
Q9NP90	RAB9B	RAB9B, member RAS oncogene family	Plasma Membrane	enzyme
Q9NPC3	CCNB1IP1	cyclin B1 interacting protein 1, E3 ubiquitin protein ligase	Nucleus	enzyme
Q9NPD3	EXOSC4	exosome component 4	Nucleus	enzyme
Q9NPJ3	ACOT13	acyl-CoA thioesterase 13	Cytoplasm	enzyme
Q9NR30	DDX21	DEAD (Asp-Glu-Ala-Asp) box helicase 21	Nucleus	enzyme
Q9NR31	SAR1A	secretion associated, Ras related GTPase 1A	Cytoplasm	enzyme
Q9NR45	NANS	N-acetylneuraminic acid synthase	Cytoplasm	enzyme
Q9NTG7	SIRT3	sirtuin 3	Cytoplasm	enzyme
Q9NVS9	PNPO	pyridoxamine 5'-phosphate oxidase	Cytoplasm	enzyme
Q9NX14	NDUFB11	NADH dehydrogenase (ubiquinone) 1 beta subcomplex, 11, 17.3kDa	Cytoplasm	enzyme

Q9NYL4	FKBP11	FK506 binding protein 11, 19 kDa	Cytoplasm	enzyme
Q9NZ01	TECR	trans-2,3-enoyl-CoA reductase	Plasma Membrane	enzyme
Q9NZ71	RTEL1	regulator of telomere elongation helicase 1	Nucleus	enzyme
Q9P0J0	NDUFA13	NADH dehydrogenase (ubiquinone) 1 alpha subcomplex, 13	Cytoplasm	enzyme
Q9P2D1	CHD7	chromodomain helicase DNA binding protein 7	Nucleus	enzyme
Q9P2R7	SUCLA2	succinate-CoA ligase, ADP-forming, beta subunit	Cytoplasm	enzyme
Q9P2T1	GMPR2	guanosine monophosphate reductase 2	Cytoplasm	enzyme
Q9UBC3	DNMT3B	DNA (cytosine-5-)-methyltransferase 3 beta	Nucleus	enzyme
Q9UBI6	GNG12	guanine nucleotide binding protein (G protein), gamma 12	Plasma Membrane	enzyme
Q9UBQ7	GRHPR	glyoxylate reductase/hydroxypyruvate reductase	Cytoplasm	enzyme
Q9UBT2	UBA2	ubiquitin-like modifier activating enzyme 2	Cytoplasm	enzyme
Q9UHG3	PCYOX1	prenylcysteine oxidase 1	Cytoplasm	enzyme

Q9UI09	NDUFA12	NADH dehydrogenase (ubiquinone) 1 alpha subcomplex, 12	Cytoplasm	enzyme
Q9UK22	FBXO2	F-box protein 2	Cytoplasm	enzyme
Q9UL25	RAB21	RAB21, member RAS oncogene family	Cytoplasm	enzyme
Q9UPY8	MAPRE3	microtubule-associated protein, RP/EB family, member 3	Cytoplasm	enzyme
Q9Y285	FARSA	phenylalanyl-tRNA synthetase, alpha subunit	Cytoplasm	enzyme
Q9Y2E5	MAN2B2	mannosidase, alpha, class 2B, member 2	Cytoplasm	enzyme
Q9Y2P8	RCL1	RNA terminal phosphate cyclase-like 1	Nucleus	enzyme
Q9Y2S2	CRYL1	crystallin, lambda 1	Cytoplasm	enzyme
Q9Y2X3	NOP58	NOP58 ribonucleoprotein	Nucleus	enzyme
Q9Y570	PPME1	protein phosphatase methylesterase 1	Other	enzyme
Q9Y617	PSAT1	phosphoserine aminotransferase 1	Cytoplasm	enzyme
O00155	GPR25	G protein-coupled receptor 25	Plasma Membrane	G-protein coupled receptor

O00270	GPR31	G protein-coupled receptor 31	Plasma Membrane	G-protein coupled receptor	
P33765	ADORA3	adenosine A3 receptor	Plasma Membrane	G-protein coupled receptor	adenosine, dyphylline, aminophylline, clofarabine, theophylline, caffeine
P48546	GIPR	gastric inhibitory polypeptide receptor	Plasma Membrane	G-protein coupled receptor	
Q13467	FZD5	frizzled class receptor 5	Plasma Membrane	G-protein coupled receptor	
Q8NFJ5	GPRC5A	G protein-coupled receptor, class C, group 5, member A	Plasma Membrane	G-protein coupled receptor	
Q8NGR1	OR13A1	olfactory receptor, family 13, subfamily A, member 1	Plasma Membrane	G-protein coupled receptor	
Q8NH51	OR8K3	olfactory receptor, family 8, subfamily K, member 3	Plasma Membrane	G-protein coupled receptor	
Q9BY15	EMR3	egf-like module containing, mucin-like, hormone receptor-like 3	Plasma Membrane	G-protein coupled receptor	
Q9H3N8	HRH4	histamine receptor H4	Plasma Membrane	G-protein coupled receptor	tesmilifene, triprolidine, buclizine
Q9HBX9	RXFP1	relaxin/insulin-like family peptide receptor 1	Plasma Membrane	G-protein coupled receptor	relaxin
Q9Y2T5	GPR52	G protein-coupled receptor 52	Plasma Membrane	G-protein coupled receptor	
P01241	GH1	growth hormone 1	Extracellular Space	growth factor	

P20774	OGN	osteoglycin	Extracellular Space	growth factor	
P20783	NTF3	neurotrophin 3	Extracellular Space	growth factor	
O00299	CLIC1	chloride intracellular channel 1	Nucleus	ion channel	
O96008	TOMM40	translocase of outer mitochondrial membrane 40 homolog (yeast)	Cytoplasm	ion channel	
P08133	ANXA6	annexin A6	Plasma Membrane	ion channel	
P20073	ANXA7	annexin A7	Plasma Membrane	ion channel	
P21796	VDAC1	voltage-dependent anion channel 1	Cytoplasm	ion channel	
P42261	GRIA1	glutamate receptor, ionotropic, AMPA 1	Plasma Membrane	ion channel	talampanel, farampator, LY451395, tezampanel, perampanel, sevoflurane, isoflurane, desflurane, methoxyflurane, enflurane
P45880	VDAC2	voltage-dependent anion channel 2	Cytoplasm	ion channel	
P48995	TRPC1	transient receptor potential cation channel, subfamily C, member 1	Plasma Membrane	ion channel	
P54105	CLNS1A	chloride channel, nucleotide-sensitive, 1A	Plasma Membrane	ion channel	

P54289	CACNA2D1	calcium channel, voltage-dependent, alpha 2/delta subunit 1	Plasma Membrane	ion channel	amlodipine/hydrochlorothiazide/valsartan, amlodipine/telmisartan, aliskiren/amlodipine, amlodipine/hydrochlorothiazide/olmesartan medoxomil, aliskiren/amlodipine/hydrochlorothiazide, nilvadipine, amlodipine/valsartan, amlodipine/benazepril, gabapentin, bepridil, enalapril/felodipine, amlodipine/atorvastatin, nisoldipine, isradipine, felodipine, nitrendipine, amlodipine, nicardipine, nifedipine, pregabalin
Q12879	GRIN2A	glutamate receptor, ionotropic, N-methyl D-aspartate 2A	Plasma Membrane	ion channel	dextromethorphan/morphine, neramexane, bicifadine, delucemine, nebostinel, besonprodil, UK-240455, tenocyclidine, dextromethorphan/quinidine, ketamine, felbamate, memantine, orphenadrine, cycloserine, N-(2-indanyl)glycinamide, dextromethorphan, brompheniramine/dextromethorphan/pseudoephedrine, chlorpheniramine/dextromethorphan/phenylephrine, carbinoxamine/dextromethorphan/pseudoephedrine, dextromethorphan/promethazine, 1-aminocyclopropane-1-carboxylic acid
Q14028	CNGB1	cyclic nucleotide gated channel beta 1	Plasma Membrane	ion channel	
Q99712	KCNJ15	potassium inwardly-rectifying channel, subfamily J, member 15	Plasma Membrane	ion channel	

Q9NPC2	KCNK9	potassium channel, subfamily K, member 9	Plasma Membrane	ion channel	doxapram, halothane
Q9NR82	KCNQ5	potassium voltage-gated channel, KQT-like subfamily, member 5	Plasma Membrane	ion channel	ezogabine
Q9P0X4	CACNA1I	calcium channel, voltage-dependent, T type, alpha 1I subunit	Plasma Membrane	ion channel	cinnarizine, flunarizine, mibefradil, zonisamide
Q9Y277	VDAC3	voltage-dependent anion channel 3	Cytoplasm	ion channel	
O00764	PDXK	pyridoxal (pyridoxine, vitamin B6) kinase	Cytoplasm	kinase	
O14936	CASK	calcium/calmodulin-dependent serine protein kinase (MAGUK family)	Plasma Membrane	kinase	
O43187	IRAK2	interleukin-1 receptor-associated kinase 2	Plasma Membrane	kinase	
O75582	RPS6KA5	ribosomal protein S6 kinase, 90kDa, polypeptide 5	Nucleus	kinase	
O75838	CIB2	calcium and integrin binding family member 2	Other	kinase	
O76094	SRP72	signal recognition particle 72kDa	Nucleus	kinase	

P00533	EGFR	epidermal growth factor receptor	Plasma Membrane	kinase	cetuximab, AEE 788, panitumumab, BMS-599626, varlitinib, XL647, bevacizumab/erlotinib, afatinib, sapitinib, cetuximab/irinotecan, lapatinib/pazopanib, irinotecan/panitumumab, erlotinib/vismodegib, erlotinib/gemcitabine, lapatinib/letrozole, capecitabine/lapatinib, bevacizumab/panitumumab, bevacizumab/cetuximab, capecitabine/erlotinib, lapatinib/paclitaxel, cabozantinib/erlotinib, lapatinib/trastuzumab, canertinib, gefitinib, neratinib, PD 153035, lapatinib, vandetanib, erlotinib
P00558	PGK1	phosphoglycerate kinase 1	Cytoplasm	kinase	
P00568	AK1	adenylate kinase 1	Cytoplasm	kinase	
P05771	PRKCB	protein kinase C, beta	Cytoplasm	kinase	enzastaurin, ruboxistaurin, ingenol mebutate
P07949	RET	ret proto-oncogene	Plasma Membrane	kinase	sunitinib, motesanib, cabozantinib, regorafenib, ponatinib, bortezomib/sorafenib, dexamethasone/lenalidomide/sorafenib, bevacizumab/sorafenib, imatinib/sirolimus, cabozantinib/erlotinib, imatinib, sorafenib, vandetanib
P08237	PFKM	phosphofructokinase, muscle	Cytoplasm	kinase	
P10644	PRKAR1A	protein kinase, cAMP-dependent, regulatory, type I, alpha	Cytoplasm	kinase	

P11362	FGFR1	fibroblast growth factor receptor 1	Plasma Membrane	kinase	pazopanib, regorafenib, bortezomib/sorafenib, dexamethasone/lenalidomide/sorafenib, sorafenib
P12277	CKB	creatine kinase, brain	Cytoplasm	kinase	
P13861	PRKAR2A	protein kinase, cAMP-dependent, regulatory, type II, alpha	Cytoplasm	kinase	
P14618	PKM	pyruvate kinase, muscle	Cytoplasm	kinase	
P14635	CCNB1	cyclin B1	Cytoplasm	kinase	
P15531	NME1	NME/NM23 nucleoside diphosphate kinase 1	Cytoplasm	kinase	
P17612	PRKACA	protein kinase, cAMP-dependent, catalytic, alpha	Cytoplasm	kinase	
P17858	PFKL	phosphofructokinase, liver	Cytoplasm	kinase	
P19367	HK1	hexokinase 1	Cytoplasm	kinase	
P19784	CSNK2A2	casein kinase 2, alpha prime polypeptide	Cytoplasm	kinase	
P22392	NME2	NME/NM23 nucleoside diphosphate kinase 2	Nucleus	kinase	
P30085	CMPK1	cytidine monophosphate (UMP-CMP) kinase 1, cytosolic	Nucleus	kinase	

P30613	PKLR	pyruvate kinase, liver and RBC	Cytoplasm	kinase	
P31323	PRKAR2B	protein kinase, cAMP-dependent, regulatory, type II, beta	Cytoplasm	kinase	
P35590	TIE1	tyrosine kinase with immunoglobulin-like and EGF-like domains 1	Plasma Membrane	kinase	
P36507	MAP2K2	mitogen-activated protein kinase kinase 2	Cytoplasm	kinase	selumetinib, trametinib, dabrafenib/trametinib
P37023	ACVRL1	activin A receptor type II-like 1	Plasma Membrane	kinase	
P42336	PIK3CA	phosphatidylinositol-4,5-bisphosphate 3-kinase, catalytic subunit alpha	Cytoplasm	kinase	SF 1126, PX-866, dactolisib, 2-(1H-indazol-4-yl)-6-(4-methanesulfonylpiperazin-1-ylmethyl)-4-morpholin-4-ylthieno(3,2-d)pyrimidine, buparlisib, XL147
P42345	MTOR	mechanistic target of rapamycin (serine/threonine kinase)	Nucleus	kinase	ridaforolimus, OSI-027, methotrexate/sirolimus/tacrolimus, dactolisib, everolimus/exemestane, lenalidomide/temsirolimus, imatinib/sirolimus, cyclophosphamide/sirolimus, cyclosporine A/sirolimus/tacrolimus, sirolimus, temsirolimus, tacrolimus, everolimus, pimecrolimus
P48426	PIP4K2A	phosphatidylinositol-5-phosphate 4-kinase, type II, alpha	Cytoplasm	kinase	
P51570	GALK1	galactokinase 1	Cytoplasm	kinase	

P54819	AK2	adenylate kinase 2	Cytoplasm	kinase	
P55263	ADK	adenosine kinase	Nucleus	kinase	pegintron/ribavirin, PEG-interferon alfa-2a/ribavirin, interferon alfa-2b/ribavirin, ribavirin
P60891	PRPS1	phosphoribosyl pyrophosphate synthetase 1	Cytoplasm	kinase	
P78356	PIP4K2B	phosphatidylinositol-5-phosphate 4-kinase, type II, beta	Cytoplasm	kinase	
P78371	CCT2	chaperonin containing TCP1, subunit 2 (beta)	Cytoplasm	kinase	
P78527	PRKDC	protein kinase, DNA-activated, catalytic polypeptide	Nucleus	kinase	
Q01415	GALK2	galactokinase 2	Cytoplasm	kinase	
Q01813	PFKP	phosphofructokinase, platelet	Cytoplasm	kinase	
Q02750	MAP2K1	mitogen-activated protein kinase kinase 1	Cytoplasm	kinase	selumetinib, trametinib, dabrafenib/trametinib
Q07002	CDK18	cyclin-dependent kinase 18	Cytoplasm	kinase	
Q12792	TWF1	twinfilin actin-binding protein 1	Cytoplasm	kinase	
Q12851	MAP4K2	mitogen-activated protein kinase kinase kinase kinase 2	Cytoplasm	kinase	

Q13188	STK3	serine/threonine kinase 3	Cytoplasm	kinase	
Q13232	NME3	NME/NM23 nucleoside diphosphate kinase 3	Cytoplasm	kinase	
Q15126	PMVK	phosphomevalonate kinase	Cytoplasm	kinase	
Q15303	ERBB4	v-erb-b2 avian erythroblastic leukemia viral oncogene homolog 4	Plasma Membrane	kinase	BMS-599626, afatinib
Q15700	DLG2	discs, large homolog 2 (Drosophila)	Plasma Membrane	kinase	
Q16760	DGKD	diacylglycerol kinase, delta 130kDa	Cytoplasm	kinase	
Q16774	GUK1	guanylate kinase 1	Cytoplasm	kinase	
Q92597	NDRG1	N-myc downstream regulated 1	Nucleus	kinase	
Q96S44	TP53RK	TP53 regulating kinase	Nucleus	kinase	
Q99683	MAP3K5	mitogen-activated protein kinase kinase kinase 5	Cytoplasm	kinase	
Q99759	MAP3K3	mitogen-activated protein kinase kinase kinase 3	Cytoplasm	kinase	
Q9H1E3	NUCKS1	nuclear casein kinase and cyclin-dependent kinase substrate 1	Nucleus	kinase	

Q9H479	FN3K	fructosamine 3 kinase	Cytoplasm	kinase	
Q9H4A3	WNK1	WNK lysine deficient protein kinase 1	Cytoplasm	kinase	
Q9NYV4	CDK12	cyclin-dependent kinase 12	Nucleus	kinase	
Q9UIJ7	AK3	adenylate kinase 3	Cytoplasm	kinase	
Q9UJ70	NAGK	N-acetylglucosamine kinase	Cytoplasm	kinase	
P37231	PPARG	peroxisome proliferator-activated receptor gamma	Nucleus	ligand-dependent nuclear receptor	icosapent, amlodipine/telmisartan, aleglitazar, aspirin/dipyridamole/telmisartan, clopidogrel/telmisartan, glimepiride/rosiglitazone, nicotinic acid/pioglitazone, alogliptin/pioglitazone, rosiglitazone, farglitazar, sulfasalazine, pioglitazone, telmisartan, tesaglitazar, troglitazone, hydrochlorothiazide/telmisartan, balsalazide, mesalamine, bezafibrate
P48443	RXRG	retinoid X receptor, gamma	Nucleus	ligand-dependent nuclear receptor	etretinate, cytarabine/daunorubicin/tretinoin, idarubicin/mitoxantrone/tretinoin, arsenic trioxide/tretinoin, arsenic trioxide/daunorubicin/tretinoin, arsenic trioxide/gemtuzumab ozogamicin/tretinoin, daunorubicin/tretinoin, idarubicin/tretinoin, doxorubicin/tretinoin, arsenic trioxide/idarubicin/tretinoin, bexarotene, adapalene, acitretin, tretinoin, alitretinoin

O00159	MYO1C	myosin IC	Cytoplasm	other
O00160	MYO1F	myosin IF	Cytoplasm	other
O00192	ARVCF	armadillo repeat gene deleted in velocardiofacial syndrome	Plasma Membrane	other
O00231	PSMD11	proteasome (prosome, macropain) 26S subunit, non-ATPase, 11	Cytoplasm	other
O00232	PSMD12	proteasome (prosome, macropain) 26S subunit, non-ATPase, 12	Cytoplasm	other
O00567	NOP56	NOP56 ribonucleoprotein	Nucleus	other
O14940	MOCS1	molybdenum cofactor synthesis 1	Cytoplasm	other
O14994	SYN3	synapsin III	Plasma Membrane	other
O15143	ARPC1B	actin related protein 2/3 complex, subunit 1B, 41kDa	Cytoplasm	other
O15144	ARPC2	actin related protein 2/3 complex, subunit 2, 34kDa	Cytoplasm	other
O15173	PGRMC2	progesterone receptor membrane component 2	Nucleus	other
O15230	LAMA5	laminin, alpha 5	Extracellular Space	other

O15258	RER1	retention in endoplasmic reticulum sorting receptor 1	Cytoplasm	other
O15371	EIF3D	eukaryotic translation initiation factor 3, subunit D	Cytoplasm	other
O15372	EIF3H	eukaryotic translation initiation factor 3, subunit H	Cytoplasm	other
O15381	NVL	nuclear VCP-like	Nucleus	other
O43237	DYNC1LI2	dynein, cytoplasmic 1, light intermediate chain 2	Cytoplasm	other
O43242	PSMD3	proteasome (prosome, macropain) 26S subunit, non-ATPase, 3	Cytoplasm	other
O43264	ZW10	zw10 kinetochore protein	Nucleus	other
O43390	HNRNPR	heterogeneous nuclear ribonucleoprotein R	Nucleus	other
O43583	DENR	density-regulated protein	Other	other
O43707	ACTN4	actinin, alpha 4	Cytoplasm	other
O43823	AKAP8	A kinase (PRKA) anchor protein 8	Nucleus	other
O43852	CALU	calumenin	Cytoplasm	other

O60506	SYNCRIP	synaptotagmin binding, cytoplasmic RNA interacting protein	Nucleus	other
O60774	FMO6P	flavin containing monooxygenase 6 pseudogene	Cytoplasm	other
O60890	OPHN1	oligophrenin 1	Cytoplasm	other
O75083	WDR1	WD repeat domain 1	Extracellular Space	other
O75096	LRP4	low density lipoprotein receptor-related protein 4	Extracellular Space	other
O75113	N4BP1	NEDD4 binding protein 1	Cytoplasm	other
O75146	HIP1R	huntingtin interacting protein 1 related	Cytoplasm	other
O75154	RAB11FIP3	RAB11 family interacting protein 3 (class II)	Cytoplasm	other
O75175	CNOT3	CCR4-NOT transcription complex, subunit 3	Cytoplasm	other
O75323	GBAS	glioblastoma amplified sequence	Plasma Membrane	other
O75340	PDCD6	programmed cell death 6	Cytoplasm	other
O75367	H2AFY	H2A histone family, member Y	Nucleus	other

O75368	SH3BGRL	SH3 domain binding glutamate-rich protein like	Cytoplasm	other
O75369	FLNB	filamin B, beta	Cytoplasm	other
O75396	SEC22B	SEC22 vesicle trafficking protein homolog B (S. cerevisiae) (gene/pseudogene)	Cytoplasm	other
O75400	PRPF40A	PRP40 pre-mRNA processing factor 40 homolog A (S. cerevisiae)	Nucleus	other
O75494	SRSF10	serine/arginine-rich splicing factor 10	Nucleus	other
O75533	SF3B1	splicing factor 3b, subunit 1, 155kDa	Nucleus	other
O75884	RBBP9	retinoblastoma binding protein 9	Nucleus	other
O76038	SCGN	secretagogin, EF-hand calcium binding protein	Cytoplasm	other
O76041	NEBL	nebulette	Plasma Membrane	other
O94811	TPPP	tubulin polymerization promoting protein	Cytoplasm	other
O94833	DST*	dystonin	Plasma Membrane	other
O94905	ERLIN2	ER lipid raft associated 2	Plasma Membrane	other

O94913	PCF11	PCF11 cleavage and polyadenylation factor subunit	Nucleus	other
O95197	RTN3	reticulon 3	Cytoplasm	other
O95198	KLHL2	kelch-like family member 2	Cytoplasm	other
O95292	VAPB	VAMP (vesicle-associated membrane protein)-associated protein B and C	Plasma Membrane	other
O95447	LCA5L	Leber congenital amaurosis 5-like	Extracellular Space	other
O95757	HSPA4L	heat shock 70kDa protein 4-like	Cytoplasm	other
O95777	LSM8	LSM8 homolog, U6 small nuclear RNA associated (S. cerevisiae)	Nucleus	other
O95980	RECK	reversion-inducing-cysteine-rich protein with kazal motifs	Plasma Membrane	other
P01009	SERPINA1	serpin peptidase inhibitor, clade A (alpha-1 antiproteinase, antitrypsin), member 1	Extracellular Space	other
P01011	SERPINA3	serpin peptidase inhibitor, clade A (alpha-1 antiproteinase, antitrypsin), member 3	Extracellular Space	other
P01034	CST3	cystatin C	Extracellular Space	other
P01185	AVP	arginine vasopressin	Extracellular Space	other

P01189	POMC	proopiomelanocortin	Extracellular Space	other	
P01215	CGA	glycoprotein hormones, alpha polypeptide	Extracellular Space	other	
P01222	TSHB	thyroid stimulating hormone, beta	Extracellular Space	other	
P01225	FSHB	follicle stimulating hormone, beta polypeptide	Extracellular Space	other	
P01229	LHB	luteinizing hormone beta polypeptide	Extracellular Space	other	
P01242	GH2	growth hormone 2	Extracellular Space	other	
P01834	IGKC	immunoglobulin kappa constant	Extracellular Space	other	
P01842	IGLC1	immunoglobulin lambda constant 1 (Mcg marker)	Cytoplasm	other	
P01857	IGHG1	immunoglobulin heavy constant gamma 1 (G1m marker)	Extracellular Space	other	
P01861	IGHG4	immunoglobulin heavy constant gamma 4 (G4m marker)	Extracellular Space	other	
P01876	IGHA1	immunoglobulin heavy constant alpha 1	Extracellular Space	other	
P02452	COL1A1	collagen, type I, alpha 1	Extracellular Space	other	collagenase clostridium histolyticum

P02462	COL4A1	collagen, type IV, alpha 1	Extracellular Space	other	collagenase clostridium histolyticum
P02511	CRYAB	crystallin, alpha B	Nucleus	other	
P02545	LMNA	lamin A/C	Nucleus	other	
P02671	FGA	fibrinogen alpha chain	Extracellular Space	other	F2
P02675	FGB	fibrinogen beta chain	Extracellular Space	other	F2
P02746	C1QB	complement component 1, q subcomponent, B chain	Extracellular Space	other	
P02747	C1QC	complement component 1, q subcomponent, C chain	Extracellular Space	other	
P02763	ORM1	orosomucoid 1	Extracellular Space	other	
P02765	AHSG	alpha-2-HS-glycoprotein	Extracellular Space	other	
P04004	VTN	vitronectin	Extracellular Space	other	
P04156	PRNP	prion protein	Plasma Membrane	other	
P04216	THY1	Thy-1 cell surface antigen	Plasma Membrane	other	

P04350	TUBB4A	tubulin, beta 4A class IVa	Cytoplasm	other	epothilone B, colchicine/probenecid, larotaxel, ABT-751, eribulin, simotaxel, davunetide, vintafolide, milataxel, cevipabulin, brentuximab vedotin, cabazitaxel, gemcitabine/paclitaxel, docetaxel/prednisone, capecitabine/docetaxel, paclitaxel/trastuzumab, cyclophosphamide/prednisone/vincristine, docetaxel/hydrocortisone, cyclophosphamide/docetaxel, gemcitabine/vinorelbine, cyclophosphamide/daunorubicin/imatinib/prednisone/vincristine, cyclophosphamide/topotecan/vincristine, docetaxel/gemcitabine, docetaxel/gemcitabine/vincristine, irinotecan/vincristine, irinotecan/temozolomide/vincristine, bevacizumab/paclitaxel, cyclophosphamide/docetaxel/epirubicin/5-fluorouracil/trastuzumab, docetaxel/trastuzumab, trastuzumab/vinorelbine, gemcitabine/oxaliplatin/paclitaxel, cyclophosphamide/epirubicin/vincristine, docetaxel/irinotecan, docetaxel/5-fluorouracil/oxaliplatin, capecitabine/docetaxel/gemcitabine, L-asparaginase/prednisone/vincristine, cyclophosphamide/etoposide/prednisone/rituximab/vincristine, cyclophosphamide/vinorelbine, cyclophosphamide/mitoxantrone/prednisone/vincris
P04792	HSPB1	heat shock 27kDa protein 1	Cytoplasm	other	

P04899	GNAI2	guanine nucleotide binding protein (G protein), alpha inhibiting activity polypeptide 2	Plasma Membrane	other	
P05060	CHGB	chromogranin B (secretogranin 1)	Extracellular Space	other	
P05067	APP	amyloid beta (A4) precursor protein	Plasma Membrane	other	bapineuzumab, florbetapir F18, florbetaben F
P05155	SERPING1	serpin peptidase inhibitor, clade G (C1 inhibitor), member 1	Extracellular Space	other	
P05386	RPLP1	ribosomal protein, large, P1	Cytoplasm	other	
P05388	RPLP0	ribosomal protein, large, P0	Cytoplasm	other	
P05408	SCG5	secretogranin V (7B2 protein)	Extracellular Space	other	
P06396	GSN	gelsolin	Extracellular Space	other	
P07196	NEFL	neurofilament, light polypeptide	Cytoplasm	other	
P07197	NEFM	neurofilament, medium polypeptide	Plasma Membrane	other	
P07305	H1F0	H1 histone family, member 0	Nucleus	other	
P07355	ANXA2	annexin A2	Plasma Membrane	other	

P07437	TUBB	tubulin, beta class I	Cytoplasm	other	ixabepilone, colchicine/probenecid, brentuximab vedotin, cabazitaxel, 6-mercaptopurine/methotrexate/prednisone/vincristine, dexamethasone/doxorubicin/vincristine, gemcitabine/vinorelbine, irinotecan/vincristine, trastuzumab/vinorelbine, methotrexate/vinorelbine, dasatinib/dexamethasone/vincristine, vinorelbine, vincristine, podophyllotoxin, colchicine
P07585	DCN	decorin	Extracellular Space	other	
P07602	PSAP	prosaposin	Extracellular Space	other	
P07737	PFN1	profilin 1	Cytoplasm	other	
P07910	HNRNPC	heterogeneous nuclear ribonucleoprotein C (C1/C2)	Nucleus	other	
P07942	LAMB1	laminin, beta 1	Extracellular Space	other	
P07951	TPM2	tropomyosin 2 (beta)	Other	other	
P08123	COL1A2	collagen, type I, alpha 2	Extracellular Space	other	collagenase clostridium histolyticum

P08572	COL4A2	collagen, type IV, alpha 2	Extracellular Space	other	collagenase clostridium histolyticum
P08621	SNRNP70	small nuclear ribonucleoprotein 70kDa (U1)	Nucleus	other	
P08670	VIM	vimentin	Cytoplasm	other	
P08708	RPS17	ribosomal protein S17	Cytoplasm	other	
P08758	ANXA5	annexin A5	Plasma Membrane	other	
P08962	CD63	CD63 molecule	Plasma Membrane	other	
P09012	SNRPA	small nuclear ribonucleoprotein polypeptide A	Nucleus	other	
P09105	HBQ1	hemoglobin, theta 1	Other	other	
P09132	SRP19	signal recognition particle 19kDa	Cytoplasm	other	
P09382	LGALS1	lectin, galactoside-binding, soluble, 1	Extracellular Space	other	
P09486	SPARC	secreted protein, acidic, cysteine-rich (osteonectin)	Extracellular Space	other	
P09525	ANXA4	annexin A4	Plasma Membrane	other	

P09651	HNRNPA1	heterogeneous nuclear ribonucleoprotein A1	Nucleus	other	
P10645	CHGA	chromogranin A (parathyroid secretory protein 1)	Cytoplasm	other	
P10909	CLU	clusterin	Cytoplasm	other	
P11047	LAMC1	laminin, gamma 1 (formerly LAMB2)	Extracellular Space	other	
P11277	SPTB	spectrin, beta, erythrocytic	Plasma Membrane	other	
P11279	LAMP1	lysosomal-associated membrane protein 1	Plasma Membrane	other	
P11532	DMD	dystrophin	Plasma Membrane	other	
P12109	COL6A1	collagen, type VI, alpha 1	Extracellular Space	other	collagenase clostridium histolyticum
P12110	COL6A2	collagen, type VI, alpha 2	Extracellular Space	other	collagenase clostridium histolyticum
P12111	COL6A3	collagen, type VI, alpha 3	Extracellular Space	other	collagenase clostridium histolyticum
P12814	ACTN1	actinin, alpha 1	Cytoplasm	other	
P12830	CDH1	cadherin 1, type 1, E-cadherin (epithelial)	Plasma Membrane	other	

P13489	RNH1	ribonuclease/angiogenin inhibitor 1	Cytoplasm	other	
P13591	NCAM1	neural cell adhesion molecule 1	Plasma Membrane	other	BB-10901
P13693	TPT1	tumor protein, translationally-controlled 1	Cytoplasm	other	
P13987	CD59	CD59 molecule, complement regulatory protein	Plasma Membrane	other	
P14136	GFAP	glial fibrillary acidic protein	Cytoplasm	other	
P14209	CD99	CD99 molecule	Plasma Membrane	other	
P14543	NID1	nidogen 1	Extracellular Space	other	
P14625	HSP90B1	heat shock protein 90kDa beta (Grp94), member 1	Cytoplasm	other	alvespimycin, retaspimycin, cisplatin
P14678	SNRPB	small nuclear ribonucleoprotein polypeptides B and B1	Nucleus	other	
P14866	HNRNPL	heterogeneous nuclear ribonucleoprotein L	Nucleus	other	
P15880	RPS2	ribosomal protein S2	Cytoplasm	other	
P15924	DSP	desmoplakin	Plasma Membrane	other	

P15927	RPA2	replication protein A2, 32kDa	Nucleus	other	
P16035	TIMP2	TIMP metallopeptidase inhibitor 2	Extracellular Space	other	
P16401	HIST1H1B	histone cluster 1, H1b	Nucleus	other	
P16403	HIST1H1C	histone cluster 1, H1c	Nucleus	other	
P16422	EPCAM	epithelial cell adhesion molecule	Plasma Membrane	other	tucotuzumab celmoleukin, catumaxomab, adecatumumab
P17987	TCP1*	t-complex 1	Cytoplasm	other	
P17987	TCP1*	t-complex 1	Cytoplasm	other	
P18077	RPL35A	ribosomal protein L35a	Cytoplasm	other	
P18085	ARF4	ADP-ribosylation factor 4	Cytoplasm	other	
P18583	SON	SON DNA binding protein	Nucleus	other	
P18621	RPL17	ribosomal protein L17	Cytoplasm	other	
P19105	MYL12A	myosin, light chain 12A, regulatory, non-sarcomeric	Cytoplasm	other	

P19338	NCL	nucleolin	Nucleus	other	
P19652	ORM2	orosomucoid 2	Extracellular Space	other	
P20700	LMNB1	lamin B1	Nucleus	other	
P20848	SERPINA2	serpin peptidase inhibitor, clade A (alpha-1 antiproteinase, antitrypsin), member 2 (gene/pseudogene)	Extracellular Space	other	
P20908	COL5A1	collagen, type V, alpha 1	Extracellular Space	other	collagenase clostridium histolyticum
P20929	NEB	nebulin	Cytoplasm	other	
P21291	CSRP1	cysteine and glycine-rich protein 1	Nucleus	other	
P21333	FLNA	filamin A, alpha	Cytoplasm	other	
P21810	BGN	biglycan	Extracellular Space	other	
P21926	CD9	CD9 molecule	Plasma Membrane	other	
P22087	FBL	fibrillarin	Nucleus	other	
P22105	TNXB	tenascin XB	Extracellular Space	other	

P22626	HNRNPA2B1	heterogeneous nuclear ribonucleoprotein A2/B1	Nucleus	other
P23025	XPA	xeroderma pigmentosum, complementation group A	Nucleus	other
P23246	SFPQ	splicing factor proline/glutamine-rich	Nucleus	other
P23327	HRC	histidine rich calcium binding protein	Cytoplasm	other
P23528	CFL1	cofilin 1 (non-muscle)	Nucleus	other
P24043	LAMA2	laminin, alpha 2	Extracellular Space	other
P24593	IGFBP5	insulin-like growth factor binding protein 5	Extracellular Space	other
P24821	TNC	tenascin C	Extracellular Space	other
P25398	RPS12	ribosomal protein S12	Cytoplasm	other
P26038	MSN	moesin	Plasma Membrane	other
P26368	U2AF2	U2 small nuclear RNA auxiliary factor 2	Nucleus	other
P26373	RPL13	ribosomal protein L13	Cytoplasm	other

P27105	STOM	stomatin	Plasma Membrane	other
P27348	YWHAQ	tyrosine 3-monooxygenase/tryptophan 5-monooxygenase activation protein, theta	Cytoplasm	other
P27635	RPL10	ribosomal protein L10	Cytoplasm	other
P27824	CANX	calnexin	Cytoplasm	other
P29508	SERPINB3	serpin peptidase inhibitor, clade B (ovalbumin), member 3	Cytoplasm	other
P29622	SERPINA4	serpin peptidase inhibitor, clade A (alpha-1 antiproteinase, antitrypsin), member 4	Extracellular Space	other
P29966	MARCKS	myristoylated alanine-rich protein kinase C substrate	Plasma Membrane	other
P30042	C21orf33/LOC102724023	chromosome 21 open reading frame 33	Cytoplasm	other
P30050	RPL12	ribosomal protein L12	Nucleus	other
P30086	PEBP1	phosphatidylethanolamine binding protein 1	Cytoplasm	other
P30414	NKTR	natural killer cell triggering receptor	Plasma Membrane	other
P30443	HLA-A	major histocompatibility complex, class I, A	Plasma Membrane	other

P30740	SERPINB1	serpin peptidase inhibitor, clade B (ovalbumin), member 1	Cytoplasm	other
P31146	CORO1A	coronin, actin binding protein, 1A	Cytoplasm	other
P31150	GDI1	GDP dissociation inhibitor 1	Cytoplasm	other
P31689	DNAJA1	DnaJ (Hsp40) homolog, subfamily A, member 1	Nucleus	other
P31942	HNRNPH3	heterogeneous nuclear ribonucleoprotein H3 (2H9)	Nucleus	other
P31943	HNRNPH1	heterogeneous nuclear ribonucleoprotein H1 (H)	Nucleus	other
P31948	STIP1	stress-induced phosphoprotein 1	Cytoplasm	other
P31949	S100A11	S100 calcium binding protein A11	Cytoplasm	other
P32969	RPL9	ribosomal protein L9	Cytoplasm	other
P33176	KIF5B	kinesin family member 5B	Cytoplasm	other
P33778	HIST1H2BB	histone cluster 1, H2bb	Nucleus	other
P34931	HSPA1L	heat shock 70kDa protein 1-like	Cytoplasm	other

P34932	HSPA4	heat shock 70kDa protein 4	Cytoplasm	other
P35080	PFN2	profilin 2	Cytoplasm	other
P35221	CTNNA1	catenin (cadherin-associated protein), alpha 1, 102kDa	Plasma Membrane	other
P35237	SERPINB6	serpin peptidase inhibitor, clade B (ovalbumin), member 6	Cytoplasm	other
P35268	RPL22	ribosomal protein L22	Nucleus	other
P35442	THBS2	thrombospondin 2	Extracellular Space	other
P35443	THBS4	thrombospondin 4	Extracellular Space	other
P35555	FBN1	fibrillin 1	Extracellular Space	other
P35580	MYH10	myosin, heavy chain 10, non-muscle	Cytoplasm	other
P35749	MYH11	myosin, heavy chain 11, smooth muscle	Cytoplasm	other
P37108	SRP14	signal recognition particle 14kDa (homologous Alu RNA binding protein)	Cytoplasm	other
P38159	RBMX	RNA binding motif protein, X-linked	Nucleus	other

P38646	HSPA9	heat shock 70kDa protein 9 (mortalin)	Cytoplasm	other	
P39019	RPS19	ribosomal protein S19	Cytoplasm	other	
P39023	RPL3	ribosomal protein L3	Cytoplasm	other	omacetaxine mepesuccinate
P39059	COL15A1	collagen, type XV, alpha 1	Extracellular Space	other	collagenase clostridium histolyticum
P39060	COL18A1	collagen, type XVIII, alpha 1	Extracellular Space	other	collagenase clostridium histolyticum
P39687	ANP32A	acidic (leucine-rich) nuclear phosphoprotein 32 family, member A	Other	other	
P40121	CAPG	capping protein (actin filament), gelsolin-like	Nucleus	other	
P40227	CCT6A	chaperonin containing TCP1, subunit 6A (zeta 1)	Cytoplasm	other	
P40429	RPL13A	ribosomal protein L13a	Cytoplasm	other	
P41732	TSPAN7	tetraspanin 7	Plasma Membrane	other	
P42025	ACTR1B	ARP1 actin-related protein 1 homolog B, centractin beta (yeast)	Cytoplasm	other	
P42166	TMPO	thymopoietin	Nucleus	other	

P42704	LRPPRC	leucine-rich pentatricopeptide repeat containing	Cytoplasm	other
P42766	RPL35	ribosomal protein L35	Cytoplasm	other
P43243	MATR3	matrin 3	Nucleus	other
P46013	MKI67	marker of proliferation Ki-67	Nucleus	other
P46060	RANGAP1	Ran GTPase activating protein 1	Nucleus	other
P46776	RPL27A	ribosomal protein L27a	Nucleus	other
P46777	RPL5	ribosomal protein L5	Cytoplasm	other
P46778	RPL21	ribosomal protein L21	Cytoplasm	other
P46779	RPL28	ribosomal protein L28	Cytoplasm	other
P46782	RPS5	ribosomal protein S5	Cytoplasm	other
P46783	RPS10	ribosomal protein S10	Cytoplasm	other
P46821	MAP1B	microtubule-associated protein 1B	Cytoplasm	other

P46940	IQGAP1	IQ motif containing GTPase activating protein 1	Cytoplasm	other
P47755	CAPZA2	capping protein (actin filament) muscle Z-line, alpha 2	Cytoplasm	other
P47756	CAPZB	capping protein (actin filament) muscle Z-line, beta	Cytoplasm	other
P47914	RPL29	ribosomal protein L29	Cytoplasm	other
P48059	LIMS1	LIM and senescent cell antigen-like domains 1	Plasma Membrane	other
P48444	ARCN1	archain 1	Cytoplasm	other
P48556	PSMD8	proteasome (prosome, macropain) 26S subunit, non-ATPase, 8	Cytoplasm	other
P48643	CCT5	chaperonin containing TCP1, subunit 5 (epsilon)	Cytoplasm	other
P48723	HSPA13	heat shock protein 70kDa family, member 13	Cytoplasm	other
P49006	MARCKSL1	MARCKS-like 1	Cytoplasm	other
P49207	RPL34	ribosomal protein L34	Cytoplasm	other
P49257	LMAN1	lectin, mannose-binding, 1	Cytoplasm	other

P49321	NASP	nuclear autoantigenic sperm protein (histone-binding)	Nucleus	other
P49368	CCT3	chaperonin containing TCP1, subunit 3 (gamma)	Cytoplasm	other
P49418	AMPH	amphiphysin	Plasma Membrane	other
P49454	CENPF	centromere protein F, 350/400kDa	Nucleus	other
P50395	GDI2	GDP dissociation inhibitor 2	Cytoplasm	other
P50402	EMD	emerin	Nucleus	other
P50453	SERPINB9	serpin peptidase inhibitor, clade B (ovalbumin), member 9	Cytoplasm	other
P50502	ST13	suppression of tumorigenicity 13 (colon carcinoma) (Hsp70 interacting protein)	Cytoplasm	other
P50851	LRBA	LPS-responsive vesicle trafficking, beach and anchor containing	Cytoplasm	other
P50914	RPL14	ribosomal protein L14	Cytoplasm	other
P50991	CCT4	chaperonin containing TCP1, subunit 4 (delta)	Cytoplasm	other
P50995	ANXA11	annexin A11	Nucleus	other

P51571	SSR4	signal sequence receptor, delta	Cytoplasm	other
P51884	LUM	lumican	Extracellular Space	other
P51888	PRELP	proline/arginine-rich end leucine-rich repeat protein	Extracellular Space	other
P51991	HNRNPA3	heterogeneous nuclear ribonucleoprotein A3	Nucleus	other
P52272	HNRNPM	heterogeneous nuclear ribonucleoprotein M	Nucleus	other
P52565	ARHGDIA	Rho GDP dissociation inhibitor (GDI) alpha	Cytoplasm	other
P52566	ARHGDIB	Rho GDP dissociation inhibitor (GDI) beta	Cytoplasm	other
P52943	CRIP2	cysteine-rich protein 2	Other	other
P54107	CRISP1	cysteine-rich secretory protein 1	Extracellular Space	other
P54727	RAD23B	RAD23 homolog B (S. cerevisiae)	Nucleus	other
P55036	PSMD4	proteasome (prosome, macropain) 26S subunit, non-ATPase, 4	Cytoplasm	other
P55209	NAP1L1	nucleosome assembly protein 1-like 1	Nucleus	other

P55795	HNRNPH2	heterogeneous nuclear ribonucleoprotein H2 (H')	Nucleus	other
P56182	RRP1	ribosomal RNA processing 1	Nucleus	other
P56703	WNT3	wingless-type MMTV integration site family, member 3	Extracellular Space	other
P57088	TMEM33	transmembrane protein 33	Cytoplasm	other
P57678	GEMIN4	gem (nuclear organelle) associated protein 4	Nucleus	other
P58417	NXPH1	neurexophilin 1	Extracellular Space	other
P59045	NLRP11	NLR family, pyrin domain containing 11	Extracellular Space	other
P59190	RAB15	RAB15, member RAS oncogene family	Cytoplasm	other
P59998	ARPC4	actin related protein 2/3 complex, subunit 4, 20kDa	Cytoplasm	other
P60033	CD81	CD81 molecule	Plasma Membrane	other
P60228	EIF3E	eukaryotic translation initiation factor 3, subunit E	Cytoplasm	other
P60520	GABARAPL2	GABA(A) receptor-associated protein-like 2	Cytoplasm	other

P60660	MYL6	myosin, light chain 6, alkali, smooth muscle and non-muscle	Cytoplasm	other
P60709	ACTB	actin, beta	Cytoplasm	other
P60866	RPS20	ribosomal protein S20	Cytoplasm	other
P60981	DSTN	destrin (actin depolymerizing factor)	Cytoplasm	other
P61011	SRP54	signal recognition particle 54kDa	Cytoplasm	other
P61158	ACTR3	ARP3 actin-related protein 3 homolog (yeast)	Plasma Membrane	other
P61163	ACTR1A	ARP1 actin-related protein 1 homolog A, centractin alpha (yeast)	Cytoplasm	other
P61247	RPS3A	ribosomal protein S3A	Nucleus	other
P61254	RPL26	ribosomal protein L26	Cytoplasm	other
P61313	RPL15	ribosomal protein L15	Cytoplasm	other
P61326	MAGOH	mago-nashi homolog, proliferation-associated (Drosophila)	Nucleus	other
P61353	RPL27	ribosomal protein L27	Cytoplasm	other

P78333	GPC5	glypican 5	Plasma Membrane	other	
P78559	MAP1A	microtubule-associated protein 1A	Cytoplasm	other	estramustine
P82279	CRB1	crumbs family member 1, photoreceptor morphogenesis associated	Plasma Membrane	other	
P82914	MRPS15	mitochondrial ribosomal protein S15	Cytoplasm	other	
P82979	SARNP	SAP domain containing ribonucleoprotein	Nucleus	other	
P83731	RPL24	ribosomal protein L24	Cytoplasm	other	
Q00610	CLTC	clathrin, heavy chain (Hc)	Plasma Membrane	other	
Q01081	LOC102724594/U2AF1	U2 small nuclear RNA auxiliary factor 1	Nucleus	other	
Q01082	SPTBN1	spectrin, beta, non-erythrocytic 1	Plasma Membrane	other	
Q01518	CAP1	CAP, adenylate cyclase-associated protein 1 (yeast)	Plasma Membrane	other	
Q01844	EWSR1	EWS RNA-binding protein 1	Nucleus	other	
Q01995	TAGLN	transgelin	Cytoplasm	other	

Q02045	MYL5	myosin, light chain 5, regulatory	Cytoplasm	other
Q02224	CENPE	centromere protein E, 312kDa	Nucleus	other
Q02487	DSC2	desmocollin 2	Plasma Membrane	other
Q02539	HIST1H1A	histone cluster 1, H1a	Nucleus	other
Q02543	RPL18A	ribosomal protein L18a	Cytoplasm	other
Q02878	RPL6	ribosomal protein L6	Cytoplasm	other
Q03001	DST*	dystonin	Plasma Membrane	other
Q03252	LMNB2	lamin B2	Nucleus	other
Q03701	CEBPZ	CCAAT/enhancer binding protein (C/EBP), zeta	Nucleus	other
Q04837	SSBP1	single-stranded DNA binding protein 1, mitochondrial	Cytoplasm	other
Q05682	CALD1	caldesmon 1	Cytoplasm	other
Q06828	FMOD	fibromodulin	Extracellular Space	other

Q07020	RPL18	ribosomal protein L18	Cytoplasm	other
Q07507	DPT	dermatopontin	Extracellular Space	other
Q07890	SOS2	son of sevenless homolog 2 (Drosophila)	Cytoplasm	other
Q07960	ARHGAP1	Rho GTPase activating protein 1	Cytoplasm	other
Q09161	NCBP1	nuclear cap binding protein subunit 1, 80kDa	Nucleus	other
Q09666	AHNAK	AHNAK nucleoprotein	Nucleus	other
Q12765	SCRN1	secernin 1	Cytoplasm	other
Q12766	HMGXB3	HMG box domain containing 3	Nucleus	other
Q12955	ANK3	ankyrin 3, node of Ranvier (ankyrin G)	Plasma Membrane	other
Q13151	HNRNPA0	heterogeneous nuclear ribonucleoprotein A0	Nucleus	other
Q13156	RPA4	replication protein A4, 30kDa	Nucleus	other

Q13200	PSMD2	proteasome (prosome, macropain) 26S subunit, non-ATPase, 2	Cytoplasm	other	bortezomib/cladribine/rituximab, bortezomib/sorafenib, bortezomib/paclitaxel, bortezomib/dexamethasone, bortezomib/dexamethasone/doxorubicin, bortezomib/dexamethasone/lenalidomide, bortezomib/dexamethasone/thalidomide, bortezomib/prednisone, bortezomib/doxorubicin, bortezomib/vorinostat, bortezomib/thalidomide, bortezomib/rituximab, bortezomib/dexamethasone/rituximab, bortezomib
Q13228	SELENBP1	selenium binding protein 1	Cytoplasm	other	
Q13326	SGCG	sarcoglycan, gamma (35kDa dystrophin-associated glycoprotein)	Plasma Membrane	other	
Q13409	DYNC1I2	dynein, cytoplasmic 1, intermediate chain 2	Cytoplasm	other	
Q13426	XRCC4	X-ray repair complementing defective repair in Chinese hamster cells 4	Nucleus	other	
Q13442	PDAP1	PDGFA associated protein 1	Cytoplasm	other	

Q13509	TUBB3	tubulin, beta 3 class III	Cytoplasm	other	epothilone B, ixabepilone, colchicine/probenecid, larotaxel, ABT-751, eribulin, simotaxel, davunetide, vintafolide, milataxel, cevipabulin, brentuximab vedotin, cabazitaxel, gemcitabine/paclitaxel, docetaxel/prednisone, capecitabine/docetaxel, paclitaxel/trastuzumab, capecitabine/ixabepilone, cyclophosphamide/prednisone/vincristine, docetaxel/hydrocortisone, cyclophosphamide/docetaxel, gemcitabine/vinorelbine, cyclophosphamide/daunorubicin/imatinib/prednisone/vincristine, cyclophosphamide/topotecan/vincristine, docetaxel/gemcitabine, docetaxel/gemcitabine/vincristine, irinotecan/vincristine, irinotecan/temozolomide/vincristine, bevacizumab/paclitaxel, cyclophosphamide/docetaxel/epirubicin/5-fluorouracil/trastuzumab, docetaxel/trastuzumab, trastuzumab/vinorelbine, gemcitabine/oxaliplatin/paclitaxel, cyclophosphamide/epirubicin/vincristine, docetaxel/irinotecan, docetaxel/5-fluorouracil/oxaliplatin, capecitabine/docetaxel/gemcitabine, L-asparaginase/prednisone/vincristine, cyclophosphamide/etoposide/prednisone/rituximab/vincristine, cyclophosphamide/vinorelbine, cyclopl
Q13561	DCTN2	dynactin 2 (p50)	Cytoplasm	other	

Q13595	TRA2A	transformer 2 alpha homolog (Drosophila)	Nucleus	other	
Q13620	CUL4B	cullin 4B	Nucleus	other	
Q13642	FHL1	four and a half LIM domains 1	Cytoplasm	other	
Q13685	AAMP	angio-associated, migratory cell protein	Plasma Membrane	other	
Q13740	ALCAM	activated leukocyte cell adhesion molecule	Plasma Membrane	other	
Q13813	SPTAN1	spectrin, alpha, non-erythrocytic 1	Plasma Membrane	other	
Q13938	CAPS	calcyphosine	Cytoplasm	other	
Q14031	COL4A6	collagen, type IV, alpha 6	Extracellular Space	other	collagenase clostridium histolyticum
Q14108	SCARB2	scavenger receptor class B, member 2	Plasma Membrane	other	
Q14112	NID2	nidogen 2 (osteonidogen)	Extracellular Space	other	
Q14141	SEPT6	septin 6	Cytoplasm	other	
Q14165	MLEC	malectin	Plasma Membrane	other	

Q14185	DOCK1	dedicator of cytokinesis 1	Cytoplasm	other
Q14203	DCTN1	dynactin 1	Cytoplasm	other
Q14257	RCN2	reticulocalbin 2, EF-hand calcium binding domain	Cytoplasm	other
Q14392	LRRC32	leucine rich repeat containing 32	Plasma Membrane	other
Q14515	SPARCL1	SPARC-like 1 (hevin)	Extracellular Space	other
Q14690	PDCD11	programmed cell death 11	Nucleus	other
Q14696	MESDC2	mesoderm development candidate 2	Extracellular Space	other
Q14767	LTBP2	latent transforming growth factor beta binding protein 2	Extracellular Space	other
Q14789	GOLGB1	golgin B1	Cytoplasm	other
Q14807	KIF22	kinesin family member 22	Nucleus	other
Q15005	SPCS2	signal peptidase complex subunit 2 homolog (S. cerevisiae)	Cytoplasm	other
Q15006	EMC2	ER membrane protein complex subunit 2	Nucleus	other

Q15021	NCAPD2	non-SMC condensin I complex, subunit D2	Nucleus	other
Q15050	RRS1	RRS1 ribosome biogenesis regulator homolog (S. cerevisiae)	Nucleus	other
Q15075	EEA1	early endosome antigen 1	Cytoplasm	other
Q15113	PCOLCE	procollagen C-endopeptidase enhancer	Extracellular Space	other
Q15149	PLEC	plectin	Cytoplasm	other
Q15233	NONO	non-POU domain containing, octamer-binding	Nucleus	other
Q15293	RCN1	reticulocalbin 1, EF-hand calcium binding domain	Cytoplasm	other
Q15366	PCBP2	poly(rC) binding protein 2	Nucleus	other
Q15393	SF3B3	splicing factor 3b, subunit 3, 130kDa	Nucleus	other
Q15582	TGFBI	transforming growth factor, beta-induced, 68kDa	Extracellular Space	other
Q15631	TSN	translin	Nucleus	other
Q15717	ELAVL1	ELAV like RNA binding protein 1	Cytoplasm	other

Q15811	ITSN1	intersectin 1 (SH3 domain protein)	Cytoplasm	other
Q15818	NPTX1	neuronal pentraxin I	Extracellular Space	other
Q15942	ZYX	zyxin	Plasma Membrane	other
Q16181	SEPT7	septin 7	Cytoplasm	other
Q16186	ADRM1	adhesion regulating molecule 1	Plasma Membrane	other
Q16352	INA	internexin neuronal intermediate filament protein, alpha	Cytoplasm	other
Q16531	DDB1	damage-specific DNA binding protein 1, 127kDa	Nucleus	other
Q16600	ZNF239	zinc finger protein 239	Nucleus	other
Q16643	DBN1	drebrin 1	Cytoplasm	other
Q16658	FSCN1	fascin actin-bundling protein 1	Cytoplasm	other
Q16799	RTN1	reticulon 1	Cytoplasm	other
Q16891	IMMT	inner membrane protein, mitochondrial	Cytoplasm	other

Q86TI0	TBC1D1	TBC1 (tre-2/USP6, BUB2, cdc16) domain family, member 1	Nucleus	other
Q86UR5	RIMS1	regulating synaptic membrane exocytosis 1	Plasma Membrane	other
Q86W92	PPFIBP1	PTPRF interacting protein, binding protein 1 (liprin beta 1)	Plasma Membrane	other
Q86X10	RALGAPB	Ral GTPase activating protein, beta subunit (non-catalytic)	Other	other
Q8IUG5	MYO18B	myosin XVIIIB	Cytoplasm	other
Q8IZP1	TBC1D3 (includes others)	TBC1 domain family, member 3H	Extracellular Space	other
Q8N111	CEND1	cell cycle exit and neuronal differentiation 1	Other	other
Q8N1F7	NUP93	nucleoporin 93kDa	Nucleus	other
Q8N2C9	C21orf128	chromosome 21 open reading frame 128	Other	other
Q8NBJ7	SUMF2	sulfatase modifying factor 2	Cytoplasm	other
Q8NF91	SYNE1	spectrin repeat containing, nuclear envelope 1	Nucleus	other
Q8TB36	GDAP1	ganglioside induced differentiation associated protein 1	Cytoplasm	other

Q8TCX5	RHPN1	rhophilin, Rho GTPase binding protein 1	Cytoplasm	other
Q8TD10	MIPOL1	mirror-image polydactyly 1	Nucleus	other
Q8WUM4	PDCD6IP	programmed cell death 6 interacting protein	Cytoplasm	other
Q8WXE9	STON2	stonin 2	Cytoplasm	other
Q92522	H1FX	H1 histone family, member X	Nucleus	other
Q92538	GBF1	golgi brefeldin A resistant guanine nucleotide exchange factor 1	Cytoplasm	other
Q92563	SPOCK2	sparc/osteonectin, cwcv and kazal-like domains proteoglycan (testican) 2	Extracellular Space	other
Q92598	HSPH1	heat shock 105kDa/110kDa protein 1	Cytoplasm	other
Q92599	SEPT8	septin 8	Extracellular Space	other
Q92608	DOCK2	dedicator of cytokinesis 2	Cytoplasm	other
Q92621	NUP205	nucleoporin 205kDa	Nucleus	other
Q92688	ANP32B	acidic (leucine-rich) nuclear phosphoprotein 32 family, member B	Nucleus	other

Q92896	GLG1	golgi glycoprotein 1	Cytoplasm	other
Q96AA8	JAKMIP2	janus kinase and microtubule interacting protein 2	Extracellular Space	other
Q96BY6	DOCK10	dedicator of cytokinesis 10	Cytoplasm	other
Q96CN9	GCC1	GRIP and coiled-coil domain containing 1	Cytoplasm	other
Q96DT7	ZBTB10	zinc finger and BTB domain containing 10	Nucleus	other
Q96FX2	DPH3	diphthamide biosynthesis 3	Cytoplasm	other
Q96G25	MED8	mediator complex subunit 8	Nucleus	other
Q96JQ0	DCHS1	dachsous cadherin-related 1	Plasma Membrane	other
Q96MG7	NDNL2	necdin-like 2	Nucleus	other
Q96MY7	FAM161B	family with sequence similarity 161, member B	Other	other
Q96N46	TTC14	tetratricopeptide repeat domain 14	Other	other
Q96P20	NLRP3	NLR family, pyrin domain containing 3	Cytoplasm	other

Q96R05	RBP7	retinol binding protein 7, cellular	Cytoplasm	other	
Q96SN8	CDK5RAP2	CDK5 regulatory subunit associated protein 2	Cytoplasm	other	
Q99426	TBCB	tubulin folding cofactor B	Other	other	
Q99574	SERPINI1	serpin peptidase inhibitor, clade I (neuroserpin), member 1	Extracellular Space	other	
Q99715	COL12A1	collagen, type XII, alpha 1	Extracellular Space	other	collagenase clostridium histolyticum
Q99733	NAP1L4	nucleosome assembly protein 1-like 4	Other	other	
Q99832	CCT7	chaperonin containing TCP1, subunit 7 (eta)	Cytoplasm	other	
Q9BQE5	APOL2	apolipoprotein L, 2	Cytoplasm	other	
Q9BRH9	ZNF251	zinc finger protein 251	Nucleus	other	
Q9BTT0	ANP32E	acidic (leucine-rich) nuclear phosphoprotein 32 family, member E	Nucleus	other	
Q9BTX7	TTPAL	tocopherol (alpha) transfer protein-like	Other	other	
Q9BXI6	TBC1D10A	TBC1 domain family, member 10A	Plasma Membrane	other	

Q9BY12	SCAPER	S-phase cyclin A-associated protein in the ER	Extracellular Space	other
Q9C030	TRIM6	tripartite motif containing 6	Cytoplasm	other
Q9C0B9	ZCCHC2	zinc finger, CCHC domain containing 2	Cytoplasm	other
Q9C0C4	SEMA4C	sema domain, immunoglobulin domain (Ig), transmembrane domain (TM) and short cytoplasmic domain, (semaphorin) 4C	Plasma Membrane	other
Q9C0K0	BCL11B	B-cell CLL/lymphoma 11B (zinc finger protein)	Nucleus	other
Q9H0E2	TOLLIP	toll interacting protein	Cytoplasm	other
Q9H0U4	RAB1B	RAB1B, member RAS oncogene family	Cytoplasm	other
Q9H0V9	LMAN2L	lectin, mannose-binding 2-like	Cytoplasm	other
Q9H0W7	THAP2	THAP domain containing, apoptosis associated protein 2	Nucleus	other
Q9H1A4	ANAPC1	anaphase promoting complex subunit 1	Nucleus	other
Q9H3K6	BOLA2/BOLA2B	bolA family member 2	Cytoplasm	other
Q9H3P2	NELFA	negative elongation factor complex member A	Nucleus	other

Q9H3Z4	DNAJC5	DnaJ (Hsp40) homolog, subfamily C, member 5	Plasma Membrane	other
Q9H3Z7	ABHD16B	abhydrolase domain containing 16B	Other	other
Q9H4M7	PLEKHA4	pleckstrin homology domain containing, family A (phosphoinositide binding specific) member 4	Cytoplasm	other
Q9H5X1	FAM96A	family with sequence similarity 96, member A	Extracellular Space	other
Q9HB58	SP110	SP110 nuclear body protein	Nucleus	other
Q9HB71	CACYBP	calcyclin binding protein	Nucleus	other
Q9HBI0	PARVG	parvin, gamma	Cytoplasm	other
Q9HCD5	NCOA5	nuclear receptor coactivator 5	Nucleus	other
Q9HCK0	ZBTB26	zinc finger and BTB domain containing 26	Nucleus	other
Q9HCN8	SDF2L1	stromal cell-derived factor 2-like 1	Cytoplasm	other
Q9HD67	MYO10	myosin X	Cytoplasm	other
Q9NPP4	NLRC4	NLR family, CARD domain containing 4	Cytoplasm	other

Q9NQC3	RTN4	reticulon 4	Cytoplasm	other
Q9NR28	DIABLO	diablo, IAP-binding mitochondrial protein	Cytoplasm	other
Q9NR46	SH3GLB2	SH3-domain GRB2-like endophilin B2	Cytoplasm	other
Q9NR50	EIF2B3	eukaryotic translation initiation factor 2B, subunit 3 gamma, 58kDa	Cytoplasm	other
Q9NS15	LTBP3	latent transforming growth factor beta binding protein 3	Extracellular Space	other
Q9NS86	LANCL2	LanC lantibiotic synthetase component C-like 2 (bacterial)	Plasma Membrane	other
Q9NTK5	OLA1	Obg-like ATPase 1	Cytoplasm	other
Q9NU22	MDN1	MDN1, midasin homolog (yeast)	Nucleus	other
Q9NUD5	ZCCHC3	zinc finger, CCHC domain containing 3	Other	other
Q9NYB0	TERF2IP	telomeric repeat binding factor 2, interacting protein	Nucleus	other
Q9NYC9	DNAH9	dynein, axonemal, heavy chain 9	Cytoplasm	other
Q9NYF5	FAM13B	family with sequence similarity 13, member B	Cytoplasm	other

Q9NYP3	DONSON	downstream neighbor of SON	Extracellular Space	other
Q9NZ45	CISD1	CDGSH iron sulfur domain 1	Cytoplasm	other
Q9NZE8	MRPL35	mitochondrial ribosomal protein L35	Cytoplasm	other
Q9P0M6	H2AFY2	H2A histone family, member Y2	Nucleus	other
Q9P2L0	WDR35	WD repeat domain 35	Cytoplasm	other
Q9P2S6	ANKMY1	ankyrin repeat and MYND domain containing 1	Other	other
Q9UBS3	DNAJB9	DnaJ (Hsp40) homolog, subfamily B, member 9	Nucleus	other
Q9UBS4	DNAJB11	DnaJ (Hsp40) homolog, subfamily B, member 11	Cytoplasm	other
Q9UFN0	NIPSNAP3A	nipsnap homolog 3A (C. elegans)	Nucleus	other
Q9UHA4	LAMTOR3	late endosomal/lysosomal adaptor, MAPK and MTOR activator 3	Cytoplasm	other
Q9UHP9	SMPX	small muscle protein, X-linked	Cytoplasm	other
Q9UJ99	CDH22	cadherin 22, type 2	Plasma Membrane	other

Q9UJV3	MID2	midline 2	Cytoplasm	other
Q9UKA4	AKAP11	A kinase (PRKA) anchor protein 11	Cytoplasm	other
Q9UKM9	RALY	RALY heterogeneous nuclear ribonucleoprotein	Nucleus	other
Q9UL16	CFAP45	cilia and flagella associated protein 45	Other	other
Q9ULP0	NDRG4	NDRG family member 4	Other	other
Q9ULX6	AKAP8L	A kinase (PRKA) anchor protein 8-like	Nucleus	other
Q9UM54	MYO6	myosin VI	Cytoplasm	other
Q9UNL2	SSR3	signal sequence receptor, gamma (translocon-associated protein gamma)	Cytoplasm	other
Q9UNX3	RPL26L1	ribosomal protein L26-like 1	Cytoplasm	other
Q9UNX4	WDR3	WD repeat domain 3	Cytoplasm	other
Q9UQE7	SMC3	structural maintenance of chromosomes 3	Nucleus	other
Q9Y262	EIF3L	eukaryotic translation initiation factor 3, subunit L	Cytoplasm	other

Q9Y295	DRG1	developmentally regulated GTP binding protein 1	Cytoplasm	other
Q9Y296	TRAPPC4	trafficking protein particle complex 4	Cytoplasm	other
Q9Y2Q5	LAMTOR2	late endosomal/lysosomal adaptor, MAPK and MTOR activator 2	Cytoplasm	other
Q9Y333	LSM2	LSM2 homolog, U6 small nuclear RNA associated (S. cerevisiae)	Nucleus	other
Q9Y375	NDUFAF1	NADH dehydrogenase (ubiquinone) complex I, assembly factor 1	Cytoplasm	other
Q9Y383	LUC7L2	LUC7-like 2 (S. cerevisiae)	Other	other
Q9Y3A4	RRP7A	ribosomal RNA processing 7 homolog A (S. cerevisiae)	Cytoplasm	other
Q9Y3A5	SBDS	Shwachman-Bodian-Diamond syndrome	Nucleus	other
Q9Y3D6	FIS1	fission 1 (mitochondrial outer membrane) homolog (S. cerevisiae)	Cytoplasm	other
Q9Y3F4	STRAP	serine/threonine kinase receptor associated protein	Plasma Membrane	other
Q9Y3R5	DOPEY2	dopey family member 2	Cytoplasm	other
Q9Y490	TLN1	talin 1	Plasma Membrane	other

Q9Y4K1	AIM1	absent in melanoma 1	Extracellular Space	other	
Q9Y4L1	HYOU1	hypoxia up-regulated 1	Cytoplasm	other	
Q9Y4P3	TBL2	transducin (beta)-like 2	Plasma Membrane	other	
Q9Y5M8	SRPRB	signal recognition particle receptor, B subunit	Cytoplasm	other	
Q9Y5S9	RBM8A	RNA binding motif protein 8A	Nucleus	other	
Q9Y6C2	EMILIN1	elastin microfibril interfacer 1	Extracellular Space	other	
O14773	TPP1	tripeptidyl peptidase I	Cytoplasm	peptidase	
O14818	PSMA7	proteasome (prosome, macropain) subunit, alpha type, 7	Cytoplasm	peptidase	
O14967	CLGN	calmegin	Cytoplasm	peptidase	
O60882	MMP20	matrix metallopeptidase 20	Extracellular Space	peptidase	marimastat
O75439	PMPCB	peptidase (mitochondrial processing) beta	Cytoplasm	peptidase	
P00451	F8	coagulation factor VIII, procoagulant component	Extracellular Space	peptidase	drotrecogin alfa, F9

P00738	HP	haptoglobin	Extracellular Space	peptidase	
P00751	CFB	complement factor B	Extracellular Space	peptidase	
P01024	C3	complement component 3	Extracellular Space	peptidase	IgG
P02788	LTF	lactotransferrin	Extracellular Space	peptidase	
P04080	CSTB	cystatin B (stefin B)	Cytoplasm	peptidase	
P04632	CAPNS1	calpain, small subunit 1	Cytoplasm	peptidase	
P07099	EPHX1	epoxide hydrolase 1, microsomal (xenobiotic)	Cytoplasm	peptidase	
P07339	CTSD	cathepsin D	Cytoplasm	peptidase	
P07384	CAPN1	calpain 1, (mu/I) large subunit	Cytoplasm	peptidase	
P07711	CTSL	cathepsin L	Cytoplasm	peptidase	
P07858	CTSB	cathepsin B	Cytoplasm	peptidase	
P09668	CTSH	cathepsin H	Cytoplasm	peptidase	

P09936	UCHL1	ubiquitin carboxyl-terminal esterase L1 (ubiquitin thiolesterase)	Cytoplasm	peptidase	
P15088	CPA3	carboxypeptidase A3 (mast cell)	Extracellular Space	peptidase	
P15374	UCHL3	ubiquitin carboxyl-terminal esterase L3 (ubiquitin thiolesterase)	Cytoplasm	peptidase	
P16870	CPE	carboxypeptidase E	Cytoplasm	peptidase	
P20618	PSMB1	proteasome (prosome, macropain) subunit, beta type, 1	Cytoplasm	peptidase	carfilzomib, bortezomib/cladribine/rituximab, bortezomib/sorafenib, bortezomib/paclitaxel, bortezomib/dexamethasone, bortezomib/dexamethasone/doxorubicin, bortezomib/dexamethasone/lenalidomide, bortezomib/dexamethasone/thalidomide, carfilzomib/dexamethasone/lenalidomide, bortezomib/prednisone, bortezomib/doxorubicin, bortezomib/vorinostat, bortezomib/thalidomide, bortezomib/rituximab, bortezomib/dexamethasone/rituximab, bortezomib
P25774	CTSS	cathepsin S	Cytoplasm	peptidase	
P25786	PSMA1	proteasome (prosome, macropain) subunit, alpha type, 1	Cytoplasm	peptidase	
P25787	PSMA2	proteasome (prosome, macropain) subunit, alpha type, 2	Cytoplasm	peptidase	

P25788	PSMA3	proteasome (prosome, macropain) subunit, alpha type, 3	Cytoplasm	peptidase	
P25789	PSMA4	proteasome (prosome, macropain) subunit, alpha type, 4	Cytoplasm	peptidase	
P28066	PSMA5	proteasome (prosome, macropain) subunit, alpha type, 5	Cytoplasm	peptidase	
P28070	PSMB4	proteasome (prosome, macropain) subunit, beta type, 4	Cytoplasm	peptidase	
P28072	PSMB6	proteasome (prosome, macropain) subunit, beta type, 6	Other	peptidase	
P28074	PSMB5	proteasome (prosome, macropain) subunit, beta type, 5	Cytoplasm	peptidase	carfilzomib, bortezomib/cladribine/rituximab, bortezomib/sorafenib, bortezomib/paclitaxel, bortezomib/melphalan, bortezomib/dexamethasone, bortezomib/dexamethasone/doxorubicin, bortezomib/dexamethasone/lenalidomide, bortezomib/dexamethasone/thalidomide, carfilzomib/dexamethasone/lenalidomide, bortezomib/prednisone, bortezomib/doxorubicin, bortezomib/vorinostat, bortezomib/thalidomide, bortezomib/rituximab, bortezomib/dexamethasone/rituximab, belinostat/bortezomib, bortezomib
P28838	LAP3	leucine aminopeptidase 3	Cytoplasm	peptidase	
P29144	TPP2	tripeptidyl peptidase II	Cytoplasm	peptidase	

P29466	CASP1	caspase 1, apoptosis-related cysteine peptidase	Cytoplasm	peptidase	
P30101	PDIA3	protein disulfide isomerase family A, member 3	Cytoplasm	peptidase	
P35998	PSMC2	proteasome (prosome, macropain) 26S subunit, ATPase, 2	Nucleus	peptidase	
P43686	PSMC4	proteasome (prosome, macropain) 26S subunit, ATPase, 4	Nucleus	peptidase	
P45974	USP5	ubiquitin specific peptidase 5 (isopeptidase T)	Cytoplasm	peptidase	
P49720	PSMB3	proteasome (prosome, macropain) subunit, beta type, 3	Cytoplasm	peptidase	
P49721	PSMB2	proteasome (prosome, macropain) subunit, beta type, 2	Cytoplasm	peptidase	carfilzomib, bortezomib/cladribine/rituximab, bortezomib/sorafenib, bortezomib/paclitaxel, bortezomib/dexamethasone, bortezomib/dexamethasone/doxorubicin, bortezomib/dexamethasone/lenalidomide, bortezomib/dexamethasone/thalidomide, carfilzomib/dexamethasone/lenalidomide, bortezomib/prednisone, bortezomib/doxorubicin, bortezomib/vorinostat, bortezomib/thalidomide, bortezomib/rituximab, bortezomib/dexamethasone/rituximab, bortezomib
P54578	USP14	ubiquitin specific peptidase 14 (tRNA-guanine transglycosylase)	Cytoplasm	peptidase	

P55786	NPEPPS	aminopeptidase puromycin sensitive	Cytoplasm	peptidase
P60900	PSMA6	proteasome (prosome, macropain) subunit, alpha type, 6	Cytoplasm	peptidase
P61009	SPCS3	signal peptidase complex subunit 3 homolog (S. cerevisiae)	Cytoplasm	peptidase
Q03154	ACY1	aminoacylase 1	Cytoplasm	peptidase
Q13107	USP4	ubiquitin specific peptidase 4 (proto-oncogene)	Nucleus	peptidase
Q14204	DYNC1H1	dynein, cytoplasmic 1, heavy chain 1	Cytoplasm	peptidase
Q16740	CLPP	caseinolytic mitochondrial matrix peptidase proteolytic subunit	Cytoplasm	peptidase
Q8NDH3	NPEPL1	aminopeptidase-like 1	Nucleus	peptidase
Q8TE58	ADAMTS15	ADAM metallopeptidase with thrombospondin type 1 motif, 15	Extracellular Space	peptidase
Q92743	HTRA1	HtrA serine peptidase 1	Extracellular Space	peptidase
Q92890	UFD1L	ubiquitin fusion degradation 1 like (yeast)	Cytoplasm	peptidase
Q93008	USP9X	ubiquitin specific peptidase 9, X-linked	Plasma Membrane	peptidase

Q96KP4	CNDP2	CNDP dipeptidase 2 (metallopeptidase M20 family)	Cytoplasm	peptidase
Q96RU2	USP28	ubiquitin specific peptidase 28	Nucleus	peptidase
Q96SM3	CPXM1	carboxypeptidase X (M14 family), member 1	Extracellular Space	peptidase
Q99436	PSMB7	proteasome (prosome, macropain) subunit, beta type, 7	Cytoplasm	peptidase
Q9BYT8	NLN	neurolysin (metallopeptidase M3 family)	Cytoplasm	peptidase
Q9H9J4	USP42	ubiquitin specific peptidase 42	Other	peptidase
Q9HB40	SCPEP1	serine carboxypeptidase 1	Cytoplasm	peptidase
Q9NPF4	OSGEP	O-sialoglycoprotein endopeptidase	Other	peptidase
Q9NQE7	PRSS16	protease, serine, 16 (thymus)	Extracellular Space	peptidase
Q9NY33	DPP3	dipeptidyl-peptidase 3	Cytoplasm	peptidase
Q9UBC5	MYO1A	myosin IA	Cytoplasm	peptidase
Q9UBR2	CTSZ	cathepsin Z	Cytoplasm	peptidase

Q9UHL4	DPP7	dipeptidyl-peptidase 7	Cytoplasm	peptidase
Q9UKF5	ADAM29	ADAM metallopeptidase domain 29	Plasma Membrane	peptidase
Q9UKQ9	KLK9	kallikrein-related peptidase 9	Extracellular Space	peptidase
Q9ULA0	DNPEP	aspartyl aminopeptidase	Cytoplasm	peptidase
Q9UNM6	PSMD13	proteasome (prosome, macropain) 26S subunit, non-ATPase, 13	Cytoplasm	peptidase
O00743	PPP6C	protein phosphatase 6, catalytic subunit	Nucleus	phosphatase
O00757	FBP2	fructose-1,6-bisphosphatase 2	Cytoplasm	phosphatase
O75145	PPFIA3	protein tyrosine phosphatase, receptor type, f polypeptide (PTPRF), interacting protein (liprin), alpha 3	Plasma Membrane	phosphatase
P07738	BPGM	2,3-bisphosphoglycerate mutase	Extracellular Space	phosphatase
P08575	PTPRC	protein tyrosine phosphatase, receptor type, C	Plasma Membrane	phosphatase
P09467	FBP1	fructose-1,6-bisphosphatase 1	Cytoplasm	phosphatase
P18669	PGAM1	phosphoglycerate mutase 1 (brain)	Cytoplasm	phosphatase

P24666	ACP1	acid phosphatase 1, soluble	Cytoplasm	phosphatase
P29218	IMPA1	inositol(myo)-1(or 4)-monophosphatase 1	Cytoplasm	phosphatase
P30153	PPP2R1A	protein phosphatase 2, regulatory subunit A, alpha	Cytoplasm	phosphatase
Q01105	SET	SET nuclear proto-oncogene	Nucleus	phosphatase
Q06190	PPP2R3A	protein phosphatase 2, regulatory subunit B'', alpha	Nucleus	phosphatase
Q15257	PPP2R4	protein phosphatase 2A activator, regulatory subunit 4	Cytoplasm	phosphatase
Q8TF05	PPP4R1	protein phosphatase 4, regulatory subunit 1	Other	phosphatase
Q96GD0	PDXP	pyridoxal (pyridoxine, vitamin B6) phosphatase	Plasma Membrane	phosphatase
Q9UKK9	NUDT5	nudix (nucleoside diphosphate linked moiety X)-type motif 5	Cytoplasm	phosphatase
Q9Y217	MTMR6	myotubularin related protein 6	Cytoplasm	phosphatase
O00170	AIP	aryl hydrocarbon receptor interacting protein	Nucleus	transcription regulator
O75840	KLF7	Kruppel-like factor 7 (ubiquitous)	Nucleus	transcription regulator

O94763	URI1	URI1, prefoldin-like chaperone	Nucleus	transcription regulator
O94864	SUPT7L	suppressor of Ty 7 (S. cerevisiae)-like	Nucleus	transcription regulator
O94906	PRPF6	pre-mRNA processing factor 6	Nucleus	transcription regulator
O95905	ECD	ecdysoneless homolog (Drosophila)	Nucleus	transcription regulator
O95947	TBX6	T-box 6	Nucleus	transcription regulator
P06748	NPM1	nucleophosmin (nucleolar phosphoprotein B23, numatrin)	Nucleus	transcription regulator
P09429	HMGB1	high mobility group box 1	Nucleus	transcription regulator
P10071	GLI3	GLI family zinc finger 3	Nucleus	transcription regulator
P16104	H2AFX	H2A histone family, member X	Nucleus	transcription regulator
P17017	ZNF14	zinc finger protein 14	Nucleus	transcription regulator
P17275	JUNB	jun B proto-oncogene	Nucleus	transcription regulator
P17481	HOXB8	homeobox B8	Nucleus	transcription regulator

P17483	HOXB4	homeobox B4	Nucleus	transcription regulator
P17980	PSMC3	proteasome (prosome, macropain) 26S subunit, ATPase, 3	Nucleus	transcription regulator
P18124	RPL7	ribosomal protein L7	Nucleus	transcription regulator
P18850	ATF6	activating transcription factor 6	Cytoplasm	transcription regulator
P20719	HOXA5	homeobox A5	Nucleus	transcription regulator
P26583	HMGB2	high mobility group box 2	Nucleus	transcription regulator
P27797	CALR	calreticulin	Cytoplasm	transcription regulator
P28069	POU1F1	POU class 1 homeobox 1	Nucleus	transcription regulator
P29375	KDM5A	lysine (K)-specific demethylase 5A	Nucleus	transcription regulator
P31946	YWHAB	tyrosine 3-monooxygenase/tryptophan 5-monooxygenase activation protein, beta	Cytoplasm	transcription regulator
P35222	CTNNB1	catenin (cadherin-associated protein), beta 1, 88kDa	Nucleus	transcription regulator
P35232	PHB	prohibitin	Nucleus	transcription regulator

P35609	ACTN2	actinin, alpha 2	Nucleus	transcription regulator	
P35638	DDIT3	DNA-damage-inducible transcript 3	Nucleus	transcription regulator	
P35659	DEK	DEK proto-oncogene	Nucleus	transcription regulator	
P38398	BRCA1	breast cancer 1, early onset	Nucleus	transcription regulator	
P42229	STAT5A	signal transducer and activator of transcription 5A	Nucleus	transcription regulator	
P42858	HTT	huntingtin	Cytoplasm	transcription regulator	
P51587	BRCA2	breast cancer 2, early onset	Nucleus	transcription regulator	
P51608	MECP2	methyl CpG binding protein 2	Nucleus	transcription regulator	
P54198	HIRA	histone cell cycle regulator	Nucleus	transcription regulator	
P55198	MLLT6	myeloid/lymphoid or mixed-lineage leukemia (trithorax homolog, Drosophila); translocated to, 6	Nucleus	transcription regulator	
P55316	FOXG1	forkhead box G1	Nucleus	transcription regulator	
P56524	HDAC4	histone deacetylase 4	Nucleus	transcription regulator	tributyrin, belinostat, pyroxamide, romidepsin

P58546	MTPN	myotrophin	Nucleus	transcription regulator
P61086	UBE2K	ubiquitin-conjugating enzyme E2K	Cytoplasm	transcription regulator
P61457	PCBD1	pterin-4 alpha-carbinolamine dehydratase/dimerization cofactor of hepatocyte nuclear factor 1 alpha	Nucleus	transcription regulator
P80723	BASP1	brain abundant, membrane attached signal protein 1	Nucleus	transcription regulator
Q00056	HOXA4	homeobox A4	Nucleus	transcription regulator
Q00059	TFAM	transcription factor A, mitochondrial	Cytoplasm	transcription regulator
Q00577	PURA	purine-rich element binding protein A	Nucleus	transcription regulator
Q01085	TIAL1	TIA1 cytotoxic granule-associated RNA binding protein-like 1	Nucleus	transcription regulator
Q01130	SRSF2	serine/arginine-rich splicing factor 2	Nucleus	transcription regulator
Q01826	SATB1	SATB homeobox 1	Nucleus	transcription regulator
Q04917	YWHAH	tyrosine 3-monooxygenase/tryptophan 5-monooxygenase activation protein, eta	Cytoplasm	transcription regulator
Q07021	C1QBP	complement component 1, q subcomponent binding protein	Cytoplasm	transcription regulator

Q12888	TP53BP1	tumor protein p53 binding protein 1	Nucleus	transcription regulator
Q12906	ILF3	interleukin enhancer binding factor 3, 90kDa	Nucleus	transcription regulator
Q12986	NFX1	nuclear transcription factor, X-box binding 1	Nucleus	transcription regulator
Q13148	TARDBP	TAR DNA binding protein	Nucleus	transcription regulator
Q13263	TRIM28	tripartite motif containing 28	Nucleus	transcription regulator
Q14103	HNRNPD	heterogeneous nuclear ribonucleoprotein D (AU-rich element RNA binding protein 1, 37kDa)	Nucleus	transcription regulator
Q14526	HIC1	hypermethylated in cancer 1	Nucleus	transcription regulator
Q14765	STAT4	signal transducer and activator of transcription 4	Nucleus	transcription regulator
Q15054	POLD3	polymerase (DNA-directed), delta 3, accessory subunit	Nucleus	transcription regulator
Q15369	TCEB1	transcription elongation factor B (SIII), polypeptide 1 (15kDa, elongin C)	Nucleus	transcription regulator
Q15545	TAF7	TAF7 RNA polymerase II, TATA box binding protein (TBP)-associated factor, 55kDa	Nucleus	transcription regulator
Q15560	TCEA2	transcription elongation factor A (SII), 2	Nucleus	transcription regulator

Q15911	ZFHX3	zinc finger homeobox 3	Nucleus	transcription regulator
Q16576	RBBP7	retinoblastoma binding protein 7	Nucleus	transcription regulator
Q16665	HIF1A	hypoxia inducible factor 1, alpha subunit (basic helix-loop-helix transcription factor)	Nucleus	transcription regulator
Q86V81	ALYREF	Aly/REF export factor	Nucleus	transcription regulator
Q8IYF1	TCEB3B	transcription elongation factor B polypeptide 3B (elongin A2)	Nucleus	transcription regulator
Q8TDD1	DDX54	DEAD (Asp-Glu-Ala-Asp) box polypeptide 54	Nucleus	transcription regulator
Q92750	TAF4B	TAF4b RNA polymerase II, TATA box binding protein (TBP)-associated factor, 105kDa	Nucleus	transcription regulator
Q92833	JARID2	jumonji, AT rich interactive domain 2	Nucleus	transcription regulator
Q96GC6	ZNF274	zinc finger protein 274	Nucleus	transcription regulator
Q99551	MTERF1	mitochondrial transcription termination factor 1	Cytoplasm	transcription regulator
Q9H7E2	TDRD3	tudor domain containing 3	Nucleus	transcription regulator
Q9HCU5	PREB	prolactin regulatory element binding	Nucleus	transcription regulator

Q9NVU0	POLR3E	polymerase (RNA) III (DNA directed) polypeptide E (80kD)	Nucleus	transcription regulator
Q9UDV7	ZNF282	zinc finger protein 282	Nucleus	transcription regulator
Q9UJU2	LEF1	lymphoid enhancer-binding factor 1	Nucleus	transcription regulator
Q9UJW8	ZNF180	zinc finger protein 180	Nucleus	transcription regulator
Q9UKY1	ZHX1	zinc fingers and homeoboxes 1	Nucleus	transcription regulator
Q9ULU4	ZMYND8	zinc finger, MYND-type containing 8	Nucleus	transcription regulator
Q9UQ80	PA2G4	proliferation-associated 2G4, 38kDa	Nucleus	transcription regulator
Q9Y230	RUVBL2	RuvB-like AAA ATPase 2	Nucleus	transcription regulator
Q9Y265	RUVBL1	RuvB-like AAA ATPase 1	Nucleus	transcription regulator
Q9Y2Y1	POLR3K	polymerase (RNA) III (DNA directed) polypeptide K, 12.3 kDa	Nucleus	transcription regulator
O00303	EIF3F	eukaryotic translation initiation factor 3, subunit F	Cytoplasm	translation regulator
O75570	MTRF1	mitochondrial translational release factor 1	Cytoplasm	translation regulator

P05198	EIF2S1	eukaryotic translation initiation factor 2, subunit 1 alpha, 35kDa	Cytoplasm	translation regulator
P08865	RPSA	ribosomal protein SA	Cytoplasm	translation regulator
P11940	PABPC1	poly(A) binding protein, cytoplasmic 1	Cytoplasm	translation regulator
P13639	EEF2	eukaryotic translation elongation factor 2	Cytoplasm	translation regulator
P20042	EIF2S2	eukaryotic translation initiation factor 2, subunit 2 beta, 38kDa	Cytoplasm	translation regulator
P23588	EIF4B	eukaryotic translation initiation factor 4B	Cytoplasm	translation regulator
P24534	EEF1B2	eukaryotic translation elongation factor 1 beta 2	Cytoplasm	translation regulator
P26641	EEF1G	eukaryotic translation elongation factor 1 gamma	Cytoplasm	translation regulator
P29692	EEF1D	eukaryotic translation elongation factor 1 delta (guanine nucleotide exchange protein)	Cytoplasm	translation regulator
P41091	EIF2S3	eukaryotic translation initiation factor 2, subunit 3 gamma, 52kDa	Cytoplasm	translation regulator
P46781	RPS9	ribosomal protein S9	Cytoplasm	translation regulator
P49411	TUFM	Tu translation elongation factor, mitochondrial	Cytoplasm	translation regulator

P55884	EIF3B	eukaryotic translation initiation factor 3, subunit B	Cytoplasm	translation regulator
P56537	EIF6	eukaryotic translation initiation factor 6	Cytoplasm	translation regulator
P60842	EIF4A1	eukaryotic translation initiation factor 4A1	Cytoplasm	translation regulator
Q04637	EIF4G1	eukaryotic translation initiation factor 4 gamma, 1	Cytoplasm	translation regulator
Q05639	EEF1A2	eukaryotic translation elongation factor 1 alpha 2	Cytoplasm	translation regulator
Q14011	CIRBP	cold inducible RNA binding protein	Nucleus	translation regulator
Q14240	EIF4A2	eukaryotic translation initiation factor 4A2	Cytoplasm	translation regulator
Q15365	PCBP1	poly(rC) binding protein 1	Nucleus	translation regulator
Q99613	EIF3C	eukaryotic translation initiation factor 3, subunit C	Other	translation regulator
Q9NRA8	EIF4ENIF1	eukaryotic translation initiation factor 4E nuclear import factor 1	Cytoplasm	translation regulator
Q9UBQ5	EIF3K	eukaryotic translation initiation factor 3, subunit K	Cytoplasm	translation regulator
Q9UKV8	AGO2	argonaute RISC catalytic component 2	Cytoplasm	translation regulator

O00264	PGRMC1	progesterone receptor membrane component 1	Plasma Membrane	transmembrane receptor
O60449	LY75	lymphocyte antigen 75	Plasma Membrane	transmembrane receptor
P05556	ITGB1	integrin, beta 1 (fibronectin receptor, beta polypeptide, antigen CD29 includes MDF2, MSK12)	Plasma Membrane	transmembrane receptor
P16109	SELP	selectin P (granule membrane protein 140kDa, antigen CD62)	Plasma Membrane	transmembrane receptor
P17301	ITGA2	integrin, alpha 2 (CD49B, alpha 2 subunit of VLA-2 receptor)	Plasma Membrane	transmembrane receptor
P19256	CD58	CD58 molecule	Plasma Membrane	transmembrane receptor
P23229	ITGA6	integrin, alpha 6	Plasma Membrane	transmembrane receptor
P26010	ITGB7	integrin, beta 7	Plasma Membrane	transmembrane receptor
P26442	AMFR	autocrine motility factor receptor, E3 ubiquitin protein ligase	Plasma Membrane	transmembrane receptor
P30533	LRPAP1	low density lipoprotein receptor-related protein associated protein 1	Plasma Membrane	transmembrane receptor

P32297	CHRNA3	cholinergic receptor, nicotinic, alpha 3 (neuronal)	Plasma Membrane	transmembrane receptor	ABT-089, isoflurane, mecamylamine, succinylcholine, levamisole, rocuronium, doxacurium, amobarbital, mivacurium, pipecuronium, rapacuronium, metocurine, atracurium, cisatracurium, acetylcholine, nicotine, D-tubocurarine, arecoline, ibogaine, enflurane, pancuronium, vecuronium
P32927	CSF2RB	colony stimulating factor 2 receptor, beta, low-affinity (granulocyte-macrophage)	Plasma Membrane	transmembrane receptor	sargramostim, EPO/sargramostim, rituximab/sargramostim
P35052	GPC1	glypican 1	Plasma Membrane	transmembrane receptor	
P50895	BCAM	basal cell adhesion molecule (Lutheran blood group)	Plasma Membrane	transmembrane receptor	
P78552	IL13RA1	interleukin 13 receptor, alpha 1	Plasma Membrane	transmembrane receptor	cintredekin besudotox
Q03135	CAV1	caveolin 1, caveolae protein, 22kDa	Plasma Membrane	transmembrane receptor	
Q07954	LRP1	low density lipoprotein receptor-related protein 1	Plasma Membrane	transmembrane receptor	
Q08380	LGALS3BP	lectin, galactoside-binding, soluble, 3 binding protein	Plasma Membrane	transmembrane receptor	
Q13635	PTCH1	patched 1	Plasma Membrane	transmembrane receptor	
Q14118	DAG1	dystroglycan 1 (dystrophin-associated glycoprotein 1)	Plasma Membrane	transmembrane receptor	

Q86UP2	KTN1	kinectin 1 (kinesin receptor)	Plasma Membrane	transmembrane receptor
Q99969	RARRES2	retinoic acid receptor responder (tazarotene induced) 2	Plasma Membrane	transmembrane receptor
Q9NZR2	LRP1B	low density lipoprotein receptor-related protein 1B	Plasma Membrane	transmembrane receptor
O00410	IPO5	importin 5	Nucleus	transporter
O00445	SYT5	synaptotagmin V	Cytoplasm	transporter
O14579	COPE	coatomer protein complex, subunit epsilon	Cytoplasm	transporter
O14617	AP3D1	adaptor-related protein complex 3, delta 1 subunit	Cytoplasm	transporter
O15540	FABP7	fatty acid binding protein 7, brain	Cytoplasm	transporter
O43681	ASNA1	arsA arsenite transporter, ATP-binding, homolog 1 (bacterial)	Nucleus	transporter
O60312	ATP10A	ATPase, class V, type 10A	Plasma Membrane	transporter
O60333	KIF1B	kinesin family member 1B	Cytoplasm	transporter
O60763	USO1	USO1 vesicle transport factor	Cytoplasm	transporter

O60784	TOM1	target of myb1 (chicken)	Cytoplasm	transporter
O75431	MTX2	metaxin 2	Cytoplasm	transporter
O75436	VPS26A	vacuolar protein sorting 26 homolog A (S. pombe)	Cytoplasm	transporter
O75746	SLC25A12	solute carrier family 25 (aspartate/glutamate carrier), member 12	Cytoplasm	transporter
O75787	ATP6AP2	ATPase, H+ transporting, lysosomal accessory protein 2	Cytoplasm	transporter
O75964	ATP5L	ATP synthase, H+ transporting, mitochondrial Fo complex, subunit G	Cytoplasm	transporter
O94973	AP2A2	adaptor-related protein complex 2, alpha 2 subunit	Cytoplasm	transporter
O95741	CPNE6	copine VI (neuronal)	Plasma Membrane	transporter
P02042	HBD	hemoglobin, delta	Other	transporter
P02647	APOA1	apolipoprotein A-I	Extracellular Space	transporter
P02649	APOE	apolipoprotein E	Extracellular Space	transporter
P02730	SLC4A1	solute carrier family 4 (anion exchanger), member 1 (Diego blood group)	Plasma Membrane	transporter

P02753	RBP4	retinol binding protein 4, plasma	Extracellular Space	transporter	
P02766	TTR	transthyretin	Extracellular Space	transporter	
P02768	ALB	albumin	Extracellular Space	transporter	
P02774	GC	group-specific component (vitamin D binding protein)	Extracellular Space	transporter	
P02787	TF	transferrin	Extracellular Space	transporter	
P04114	APOB	apolipoprotein B	Extracellular Space	transporter	mipomersen
P05023	ATP1A1	ATPase, Na+/K+ transporting, alpha 1 polypeptide	Plasma Membrane	transporter	digoxin, trichloromethiazide, ciclopirox olamine, ethacrynic acid, reserpine/trichloromethiazide, bretylium, perphenazine, ouabain, digitoxin
P05026	ATP1B1	ATPase, Na+/K+ transporting, beta 1 polypeptide	Plasma Membrane	transporter	
P05141	SLC25A5	solute carrier family 25 (mitochondrial carrier; adenine nucleotide translocator), member 5	Cytoplasm	transporter	clodronic acid
P05413	FABP3	fatty acid binding protein 3, muscle and heart (mammary-derived growth inhibitor)	Cytoplasm	transporter	
P06576	ATP5B	ATP synthase, H+ transporting, mitochondrial F1 complex, beta polypeptide	Cytoplasm	transporter	

P09455	RBP1	retinol binding protein 1, cellular	Extracellular Space	transporter	
P12235	SLC25A4	solute carrier family 25 (mitochondrial carrier; adenine nucleotide translocator), member 4	Cytoplasm	transporter	clodronic acid
P12236	SLC25A6	solute carrier family 25 (mitochondrial carrier; adenine nucleotide translocator), member 6	Cytoplasm	transporter	clodronic acid
P13804	ETFA	electron-transfer-flavoprotein, alpha polypeptide	Cytoplasm	transporter	
P16615	ATP2A2	ATPase, Ca++ transporting, cardiac muscle, slow twitch 2	Cytoplasm	transporter	
P20020	ATP2B1	ATPase, Ca++ transporting, plasma membrane 1	Plasma Membrane	transporter	
P21281	ATP6V1B2	ATPase, H+ transporting, lysosomal 56/58kDa, V1 subunit B2	Cytoplasm	transporter	gallium nitrate
P21283	ATP6V1C1	ATPase, H+ transporting, lysosomal 42kDa, V1 subunit C1	Cytoplasm	transporter	
P21579	SYT1	synaptotagmin I	Cytoplasm	transporter	
P22307	SCP2	sterol carrier protein 2	Cytoplasm	transporter	
P24539	ATP5F1	ATP synthase, H+ transporting, mitochondrial Fo complex, subunit B1	Cytoplasm	transporter	
P25705	ATP5A1	ATP synthase, H+ transporting, mitochondrial F1 complex, alpha subunit 1, cardiac muscle	Cytoplasm	transporter	

P27449	ATP6V0C	ATPase, H+ transporting, lysosomal 16kDa, V0 subunit c	Cytoplasm	transporter	
P30040	ERP29	endoplasmic reticulum protein 29	Cytoplasm	transporter	
P30049	ATP5D	ATP synthase, H+ transporting, mitochondrial F1 complex, delta subunit	Cytoplasm	transporter	
P31025	LCN1	lipocalin 1	Extracellular Space	transporter	
P35606	COPB2	coatomer protein complex, subunit beta 2 (beta prime)	Cytoplasm	transporter	
P35658	NUP214	nucleoporin 214kDa	Nucleus	transporter	
P36542	ATP5C1	ATP synthase, H+ transporting, mitochondrial F1 complex, gamma polypeptide 1	Cytoplasm	transporter	
P36543	ATP6V1E1	ATPase, H+ transporting, lysosomal 31kDa, V1 subunit E1	Cytoplasm	transporter	
P38117	ETFB	electron-transfer-flavoprotein, beta polypeptide	Cytoplasm	transporter	
P38606	ATP6V1A	ATPase, H+ transporting, lysosomal 70kDa, V1 subunit A	Plasma Membrane	transporter	bafilomycin A1, bafilomycin b1
P46459	NSF	N-ethylmaleimide-sensitive factor	Cytoplasm	transporter	
P48047	ATP5O	ATP synthase, H+ transporting, mitochondrial F1 complex, O subunit	Cytoplasm	transporter	

P48739	PITPNB	phosphatidylinositol transfer protein, beta	Cytoplasm	transporter
P49755	TMED10	transmembrane emp24-like trafficking protein 10 (yeast)	Cytoplasm	transporter
P51572	BCAP31	B-cell receptor-associated protein 31	Cytoplasm	transporter
P53007	SLC25A1	solute carrier family 25 (mitochondrial carrier; citrate transporter), member 1	Other	transporter
P53618	COPB1	coatomer protein complex, subunit beta 1	Cytoplasm	transporter
P53621	COPA	coatomer protein complex, subunit alpha	Cytoplasm	transporter
P53680	AP2S1	adaptor-related protein complex 2, sigma 1 subunit	Cytoplasm	transporter
P53992	SEC24C	SEC24 family member C	Cytoplasm	transporter
P55060	CSE1L	CSE1 chromosome segregation 1-like (yeast)	Nucleus	transporter
P56134	ATP5J2	ATP synthase, H+ transporting, mitochondrial Fo complex, subunit F2	Cytoplasm	transporter
P57768	SNX16	sorting nexin 16	Other	transporter
P61421	ATP6V0D1	ATPase, H+ transporting, lysosomal 38kDa, V0 subunit d1	Cytoplasm	transporter

P80188	LCN2	lipocalin 2	Extracellular Space	transporter
Q00169	PITPNA	phosphatidylinositol transfer protein, alpha	Cytoplasm	transporter
Q00325	SLC25A3	solute carrier family 25 (mitochondrial carrier; phosphate carrier), member 3	Cytoplasm	transporter
Q00341	HDLBP	high density lipoprotein binding protein	Nucleus	transporter
Q00765	REEP5	receptor accessory protein 5	Extracellular Space	transporter
Q00839	HNRNPU	heterogeneous nuclear ribonucleoprotein U (scaffold attachment factor A)	Nucleus	transporter
Q01469	FABP5	fatty acid binding protein 5 (psoriasis-associated)	Cytoplasm	transporter
Q02952	AKAP12	A kinase (PRKA) anchor protein 12	Cytoplasm	transporter
Q02978	SLC25A11	solute carrier family 25 (mitochondrial carrier; oxoglutarate carrier), member 11	Cytoplasm	transporter
Q04941	PLP2	proteolipid protein 2 (colonic epithelium-enriched)	Cytoplasm	transporter
Q10567	AP1B1	adaptor-related protein complex 1, beta 1 subunit	Cytoplasm	transporter
Q12907	LMAN2	lectin, mannose-binding 2	Cytoplasm	transporter

Q14974	KPNB1	karyopherin (importin) beta 1	Nucleus	transporter
Q15363	TMED2	transmembrane emp24 domain trafficking protein 2	Cytoplasm	transporter
Q15904	ATP6AP1	ATPase, H+ transporting, lysosomal accessory protein 1	Cytoplasm	transporter
Q16623	STX1A	syntaxin 1A (brain)	Cytoplasm	transporter
Q93050	ATP6V0A1	ATPase, H+ transporting, lysosomal V0 subunit a1	Cytoplasm	transporter
Q96C24	SYTL4	synaptotagmin-like 4	Cytoplasm	transporter
Q96JB2	COG3	component of oligomeric golgi complex 3	Cytoplasm	transporter
Q96QK1	VPS35	vacuolar protein sorting 35 homolog (S. cerevisiae)	Cytoplasm	transporter
Q99536	VAT1	vesicle amine transport 1	Plasma Membrane	transporter
Q99598	TSNAX	translin-associated factor X	Nucleus	transporter
Q99829	CPNE1	copine I	Nucleus	transporter
Q9BVK6	TMED9	transmembrane emp24 protein transport domain containing 9	Cytoplasm	transporter

Q9H270	VPS11	vacuolar protein sorting 11 homolog (S. cerevisiae)	Cytoplasm	transporter
Q9H329	EPB41L4B	erythrocyte membrane protein band 4.1 like 4B	Other	transporter
Q9H9B4	SFXN1	sideroflexin 1	Cytoplasm	transporter
Q9H9E3	COG4	component of oligomeric golgi complex 4	Cytoplasm	transporter
Q9HD45	TM9SF3	transmembrane 9 superfamily member 3	Cytoplasm	transporter
Q9NPD5	SLCO1B3	solute carrier organic anion transporter family, member 1B3	Plasma Membrane	transporter
Q9NS69	TOMM22	translocase of outer mitochondrial membrane 22 homolog (yeast)	Cytoplasm	transporter
Q9NV70	EXOC1	exocyst complex component 1	Cytoplasm	transporter
Q9P241	ATP10D	ATPase, class V, type 10D	Cytoplasm	transporter
Q9UBQ0	VPS29	vacuolar protein sorting 29 homolog (S. cerevisiae)	Cytoplasm	transporter
Q9UG63	ABCF2	ATP-binding cassette, sub-family F (GCN20), member 2	Cytoplasm	transporter
Q9UNF0	PACSIN2	protein kinase C and casein kinase substrate in neurons 2	Cytoplasm	transporter

Q9UNH7		SNX6	sorting nexin 6	Cytoplasm	transporter
Q9UNQ0		ABCG2	ATP-binding cassette, sub-family G (WHITE), member 2	Plasma Membrane	transporter
Q9Y2L5		TRAPPC8	trafficking protein particle complex 8	Cytoplasm	transporter
Q9Y3B3		TMED7	transmembrane emp24 protein transport domain containing 7	Cytoplasm	transporter
Q9Y5K8		ATP6V1D	ATPase, H+ transporting, lysosomal 34kDa, V1 subunit D	Cytoplasm	transporter
Q9Y678		COPG1	coatomer protein complex, subunit gamma 1	Cytoplasm	transporter
Q9Y6Q5		AP1M2	adaptor-related protein complex 1, mu 2 subunit	Cytoplasm	transporter
O75642	unmapped				
P00938	unmapped				
P01028	unmapped				
P01243	unmapped				
P01597	unmapped				

P01614	unmapped
P01737	unmapped
P01884	unmapped
P01922	unmapped
P02023	unmapped
P02096	unmapped
P02248	unmapped
P02261	unmapped
P02278	unmapped
P02304	unmapped
P02383	unmapped
P02570	unmapped

P02570	unmapped
P02571	unmapped
P02593	unmapped
P03996	unmapped
P04206	unmapped
P04270	unmapped
P04643	unmapped
P04645	unmapped
P04720	unmapped
P04895	unmapped
P04901	unmapped
P05092	unmapped

P05209	unmapped
P05215	unmapped
P05217	unmapped
P05218	unmapped
P05323	unmapped
P06366	unmapped
P07226	unmapped
P08107	unmapped
P08578	unmapped
P09058	unmapped
P10113	unmapped
P10159	unmapped

P10660	unmapped
P11174	unmapped
P11476	unmapped
P11518	unmapped
P12750	unmapped
P12947	unmapped
P13641	unmapped
P13662	unmapped
P14118	unmapped
P15154	unmapped
P16106	unmapped
P16475	unmapped

P16632	unmapped
P17008	unmapped
P17080	unmapped
P17317	unmapped
P18282	unmapped
P19065	unmapped
P19138	unmapped
P20071	unmapped
P20172	unmapped
P21378	unmapped
P23131	unmapped
P23152	unmapped

P23411	unmapped
P23821	unmapped
P24410	unmapped
P25111	unmapped
P25112	unmapped
P25120	unmapped
P25232	unmapped
P25388	unmapped
P28001	unmapped
P29312	unmapped
P29316	unmapped
P29354	unmapped

P30054	unmapped
P30712	unmapped
P31327)	unmapped
P32889	unmapped
P34062	unmapped
P34991	unmapped
P35214	unmapped
P37140	unmapped
P39026	unmapped
P39027	unmapped
P39028	unmapped
P42024	unmapped

P42655	unmapped
P43330	unmapped
P43331	unmapped
P46966	unmapped
P47210	unmapped
P53025	unmapped
Q00382	unmapped
Q02546	unmapped
Q03527	unmapped
Q04984	unmapped
Q07244	unmapped
Q13748	unmapped

Q14259	unmapped
Q15356	unmapped
Q15668	unmapped
Q15701	unmapped
Q15763	unmapped
Q15815	unmapped
Q64320	unmapped
Q92524	unmapped
Q93080	unmapped
Q9BTC0)	unmapped
Q9Y3C3	unmapped
Q9Y4Y8	unmapped
