# Supplementary material for: Multiomics-Based Signaling Pathway Network Alterations in Human Non-functional Pituitary Adenomas
Source: Front Endocrinol (Lausanne). 2019 Dec 17;10:835. doi: 10.3389/fendo.2019.00835 (PMC6928143; doi:10.3389/fendo.2019.00835)

Supplemental materials 8.3
Statistically significant canonical pathways derived from nitroproteins from a pituitary control for IPA analysis (Dataset 8)

1. Cellular effects of sildenafil (Viagra)
2. Gap junction signaling
3. Mechanisms of viral exit from host cells
4. Sertoli cell-sertoli cell junction signaling
5. Clathrin-mediated endocytosis signaling
6. MSP-RON signaling pathway
7. Remodeling of epthelial adherens junctions
8. Agrin interactions at neuromuscular junction
9. Caveolar-mediated endocytosis signaling
10. Crosstalk between dentritic cells and natrual killer cells
11. FAK signaling
12. Death receptor signaling
13. Regulation of actin-based motility by Rho
14. Virus entry via endocytic pathways
15. VEGF signaling
16. Fcy receptor-mediated phagocytosis in macrophages and monocytes
17. Paxillin signaling
18. RhoA signaling
19. Epthelial adherens junction signaling
20. Tec kinase signaling
21. Germ cell-sertoli cell junction signaling
22. Tight junction signaling
23. RhoGDI signaling
24. NRF2-mediated oxidative stress response
25. ILK signaling
26. Agranulocyte adhesion and diapedesis
27. Integrin signaling
28. Leukocyte extravasation signaling
29. Actin cytoskeleton signaling
30. Signaling by Rho family GTPases
31. Calcium signaling
32. Protein ubiquitination pathway
33. Nitric oxide signaling in the cardiovascular system

Dataset 8-Canonical Pathway Chart

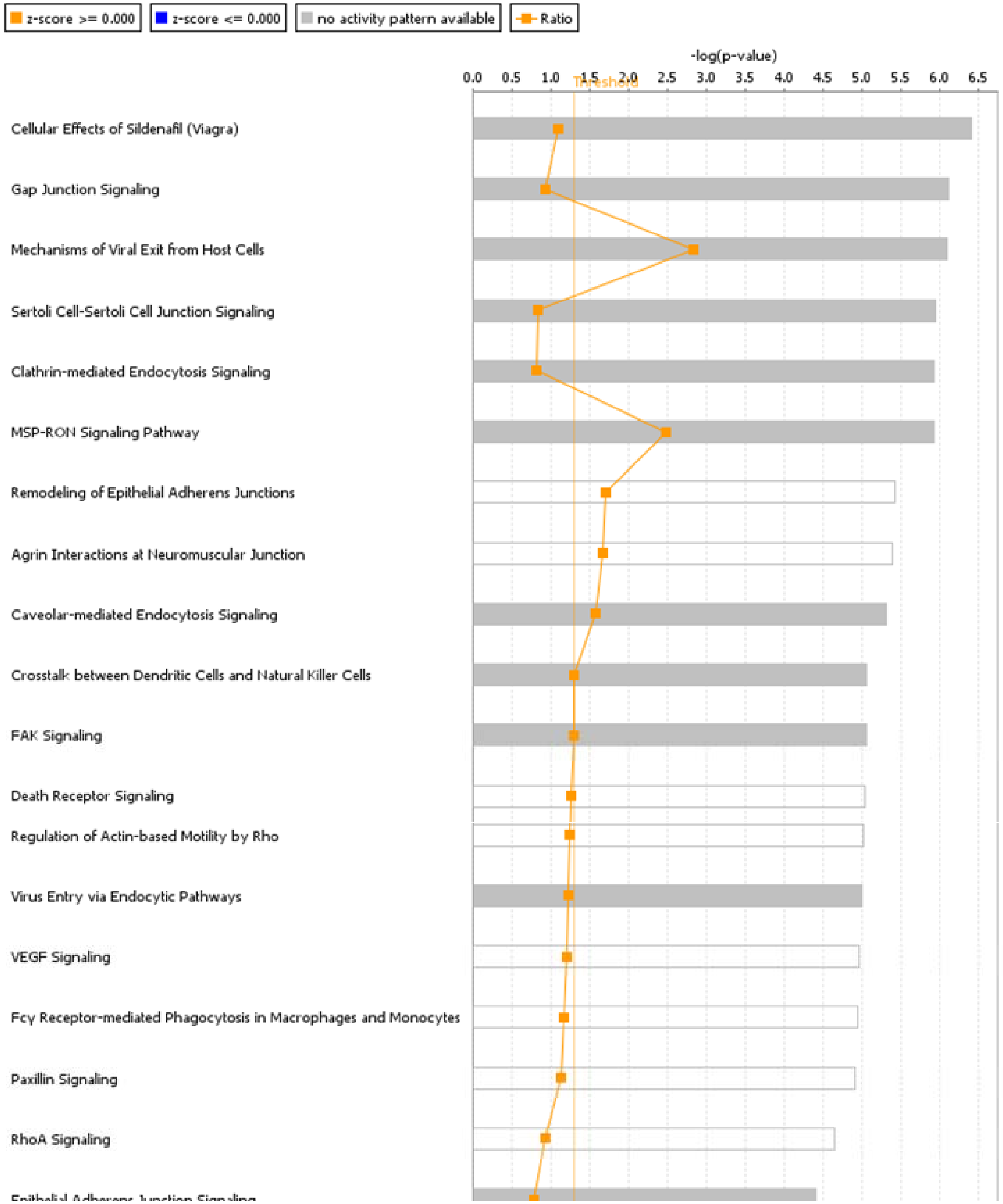

1-Cellular effects of sildenafil (Viagra)

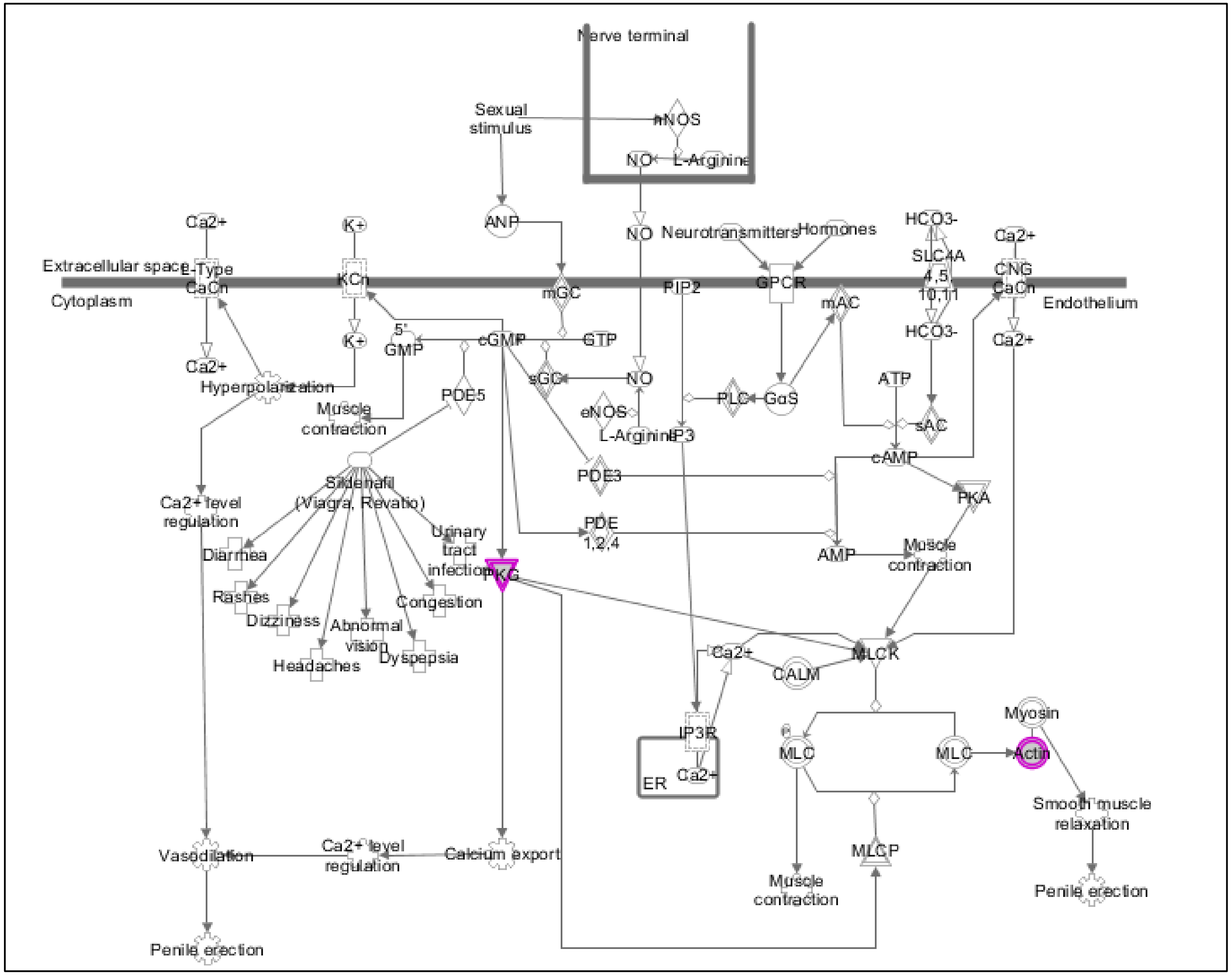

2-Gap junction signaling

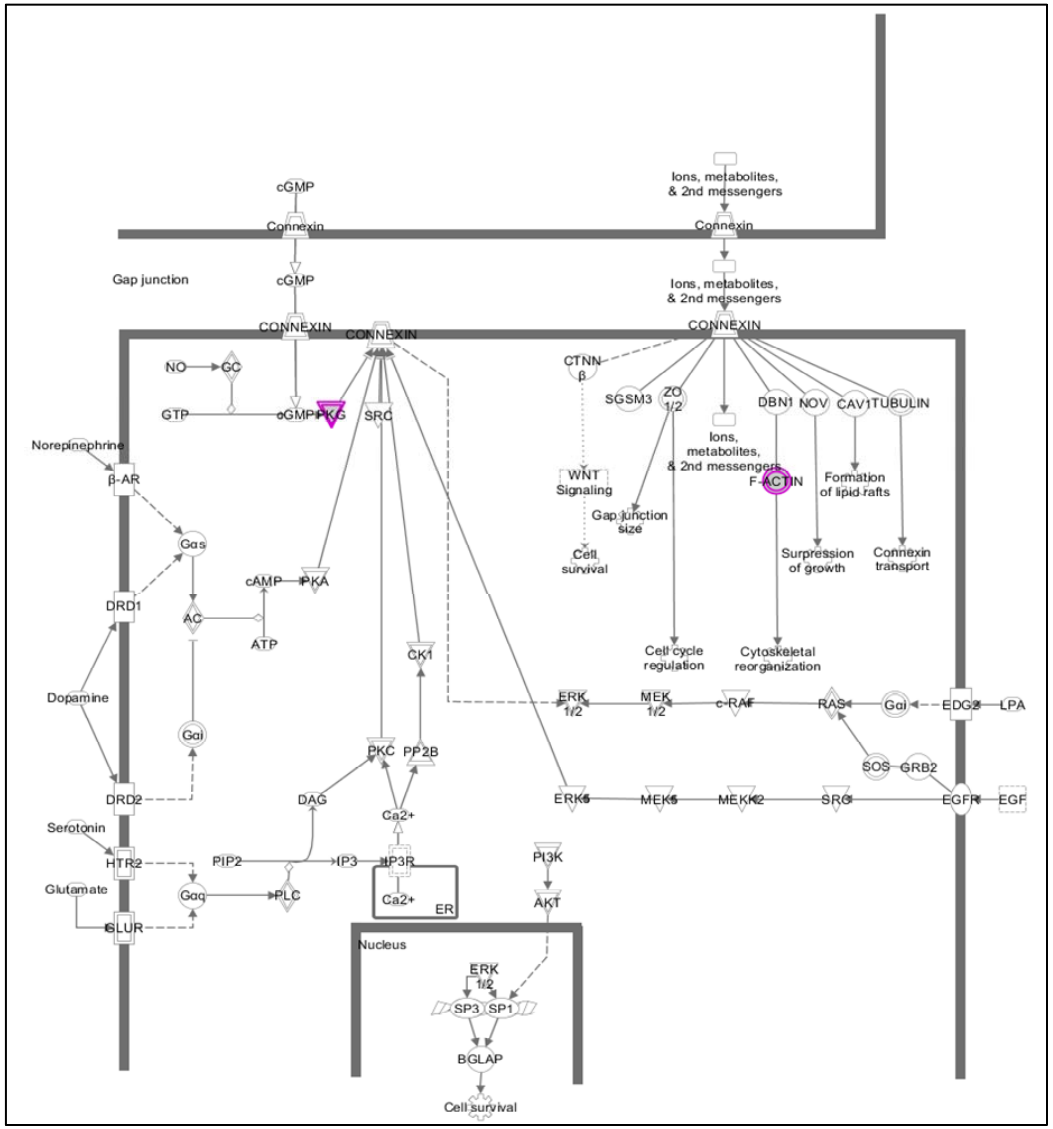

3-Mechanisms of viral exit from host cells

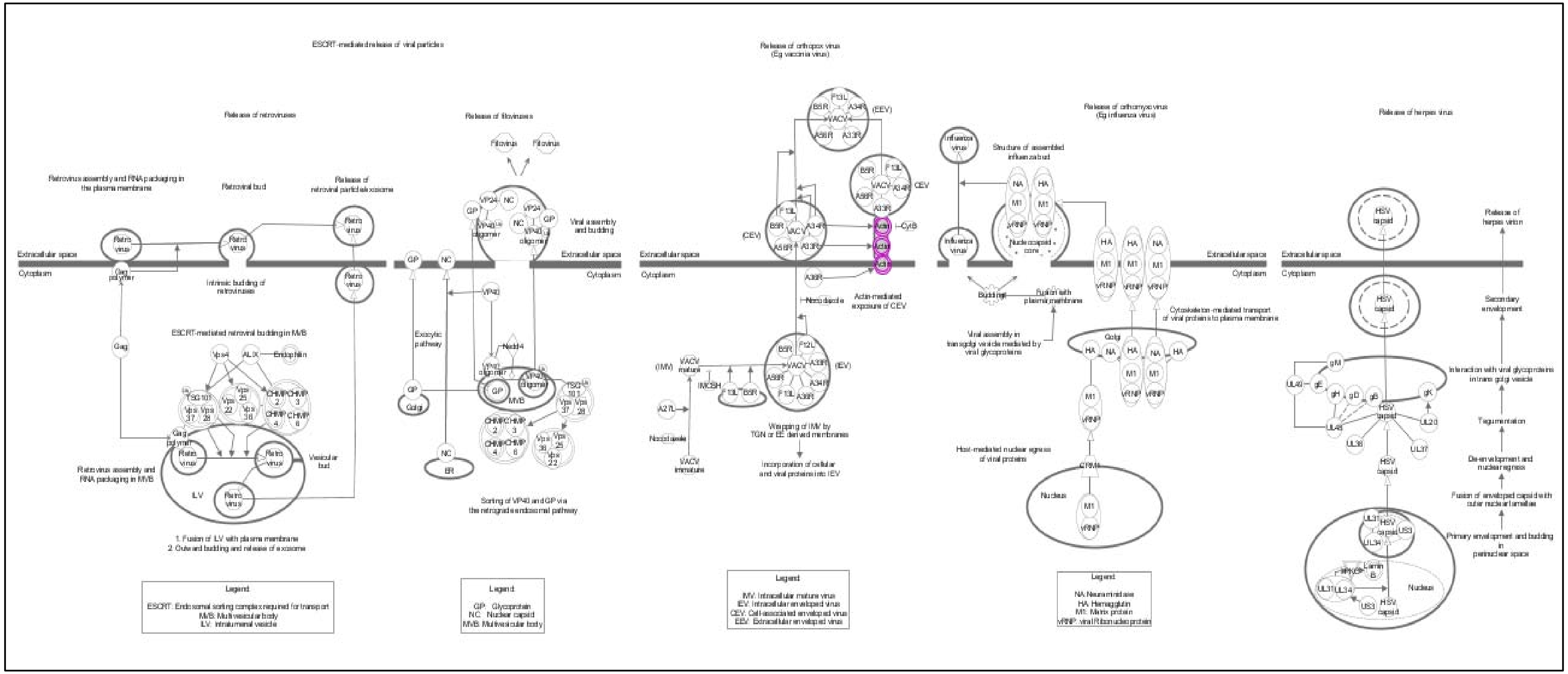

4-Sertoli cell-sertoli cell junction signaling

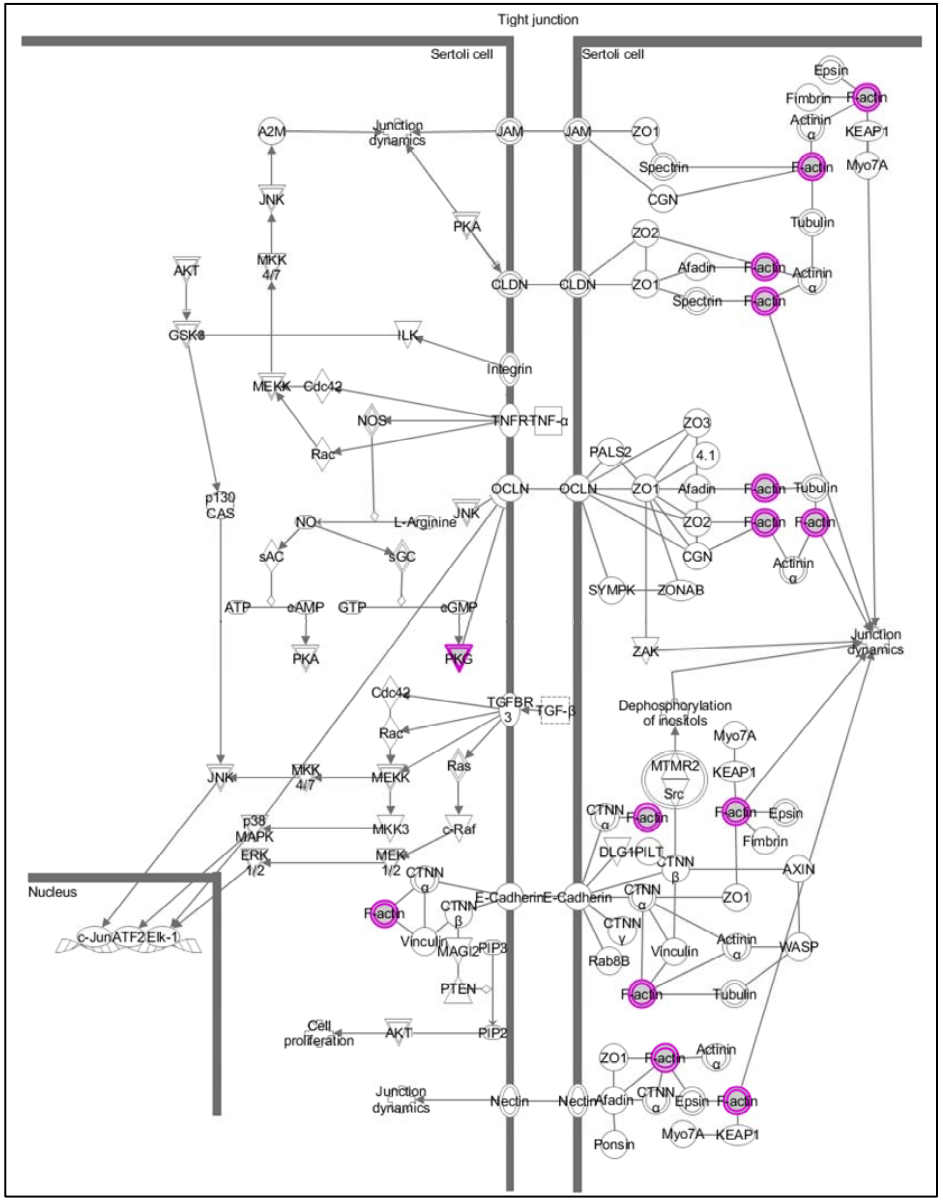

5-Clathrin-mediated endocytosis signaling

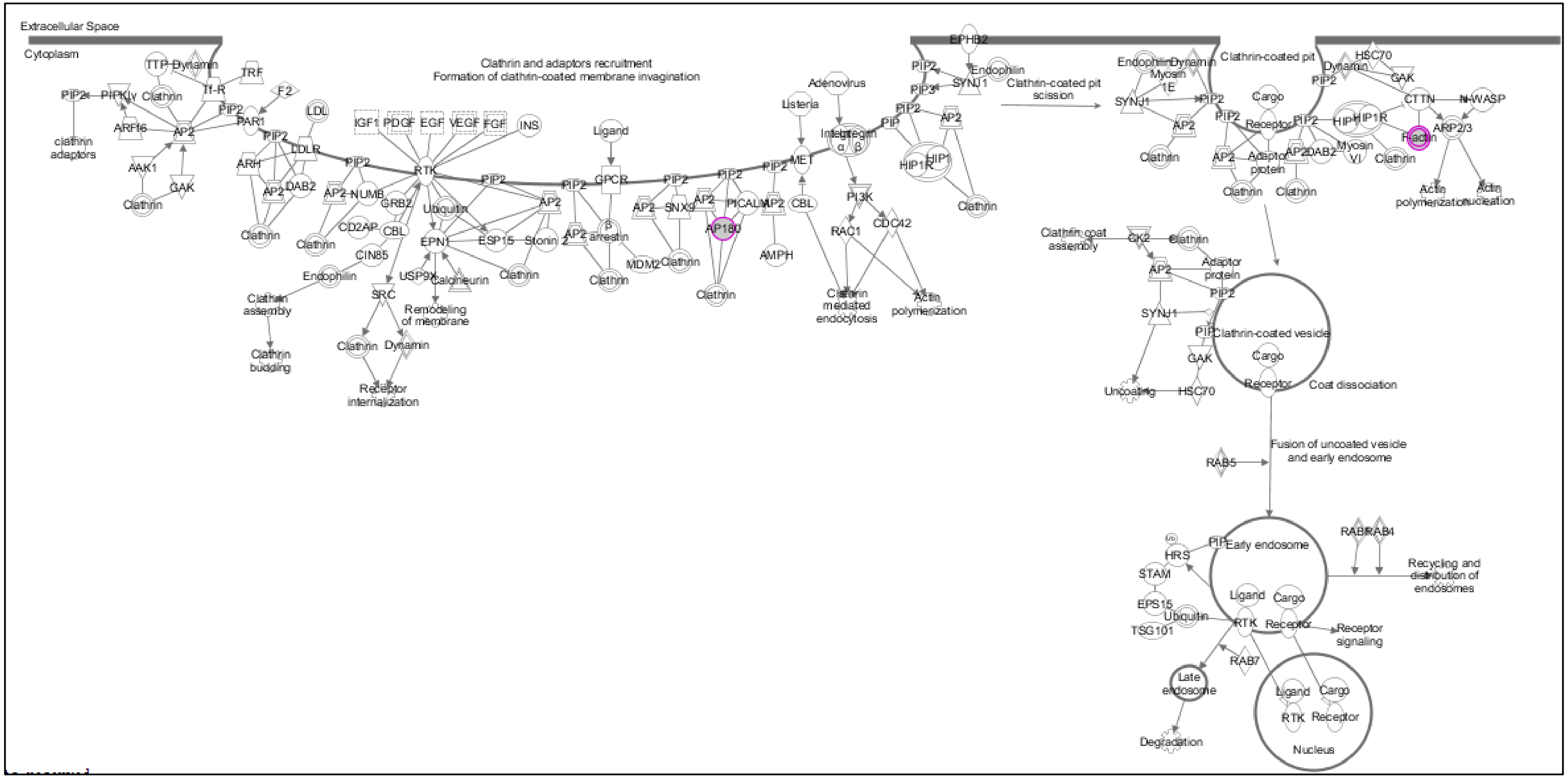

6-MSP-RON signaling pathway

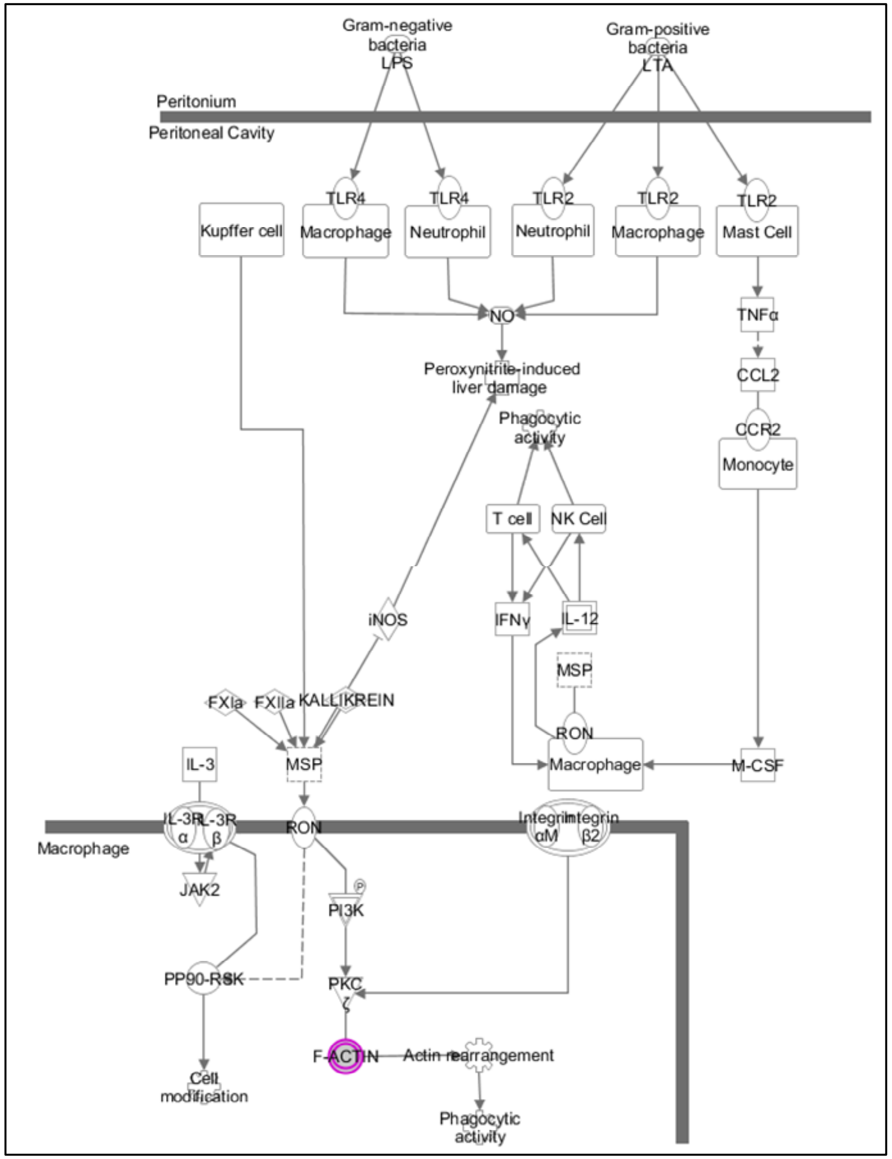

7-Remodeling of epthelial adherens junctions

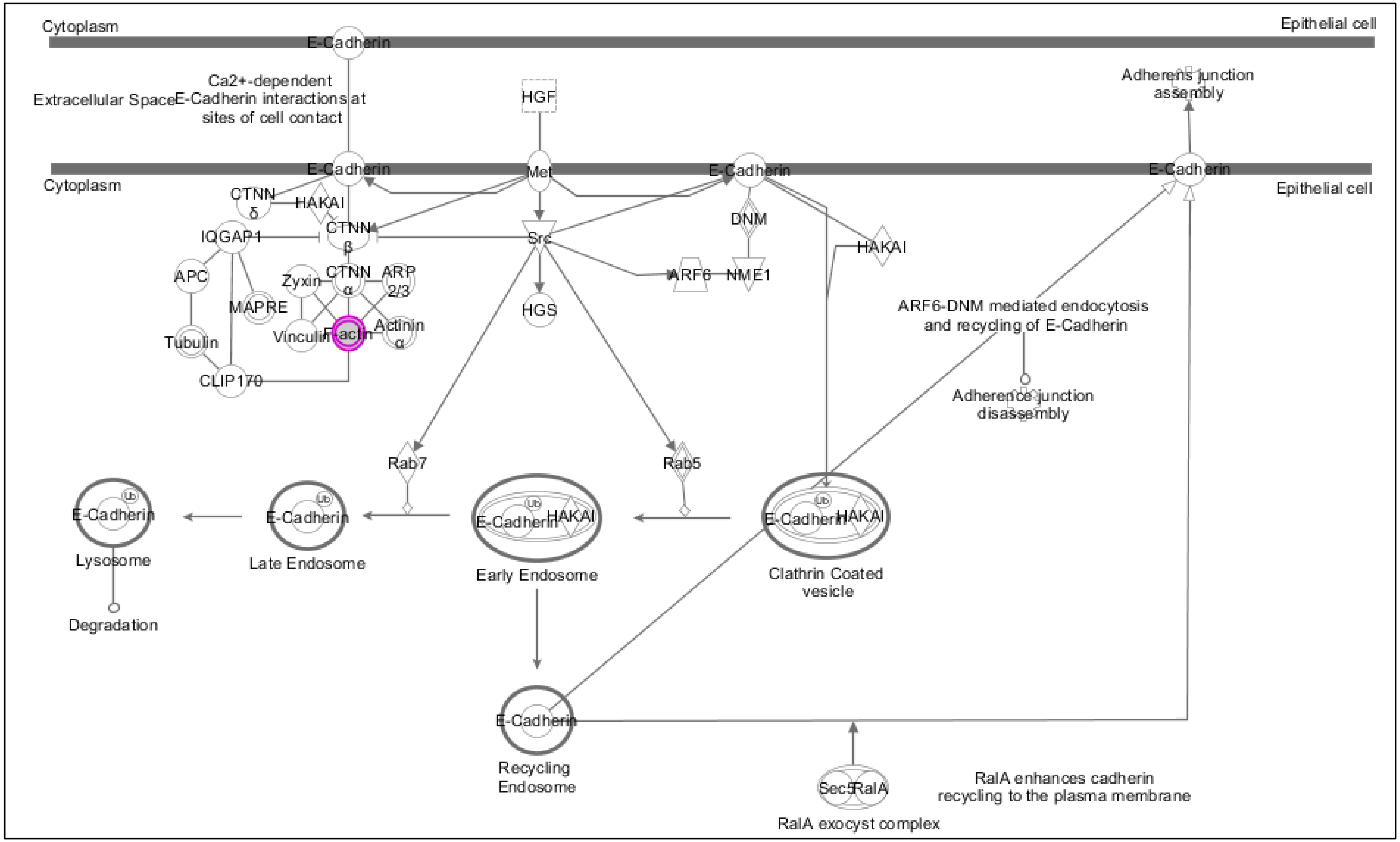

8-Agrin interactions at neuromuscular junction

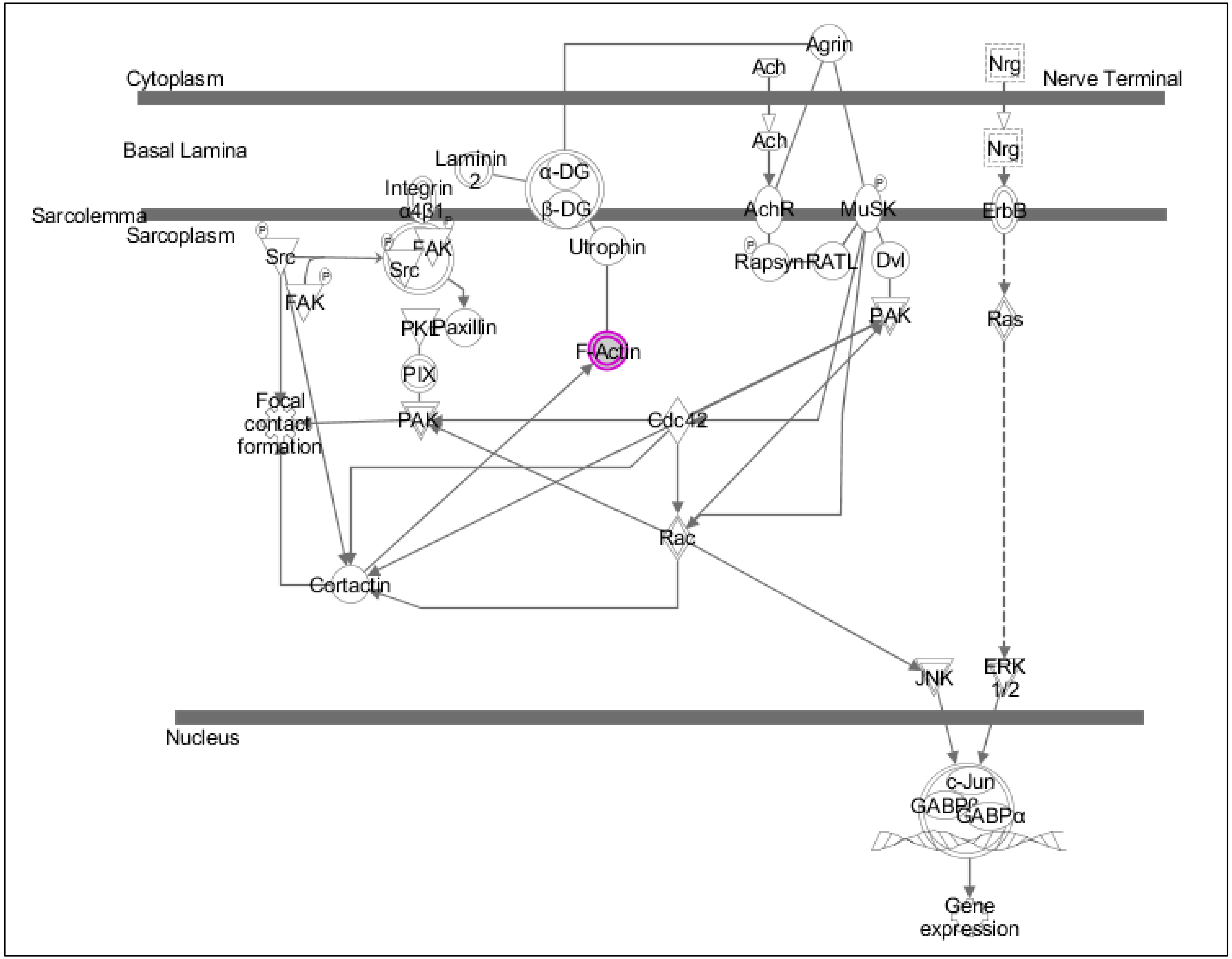

9-Caveolar-mediated endocytosis signaling

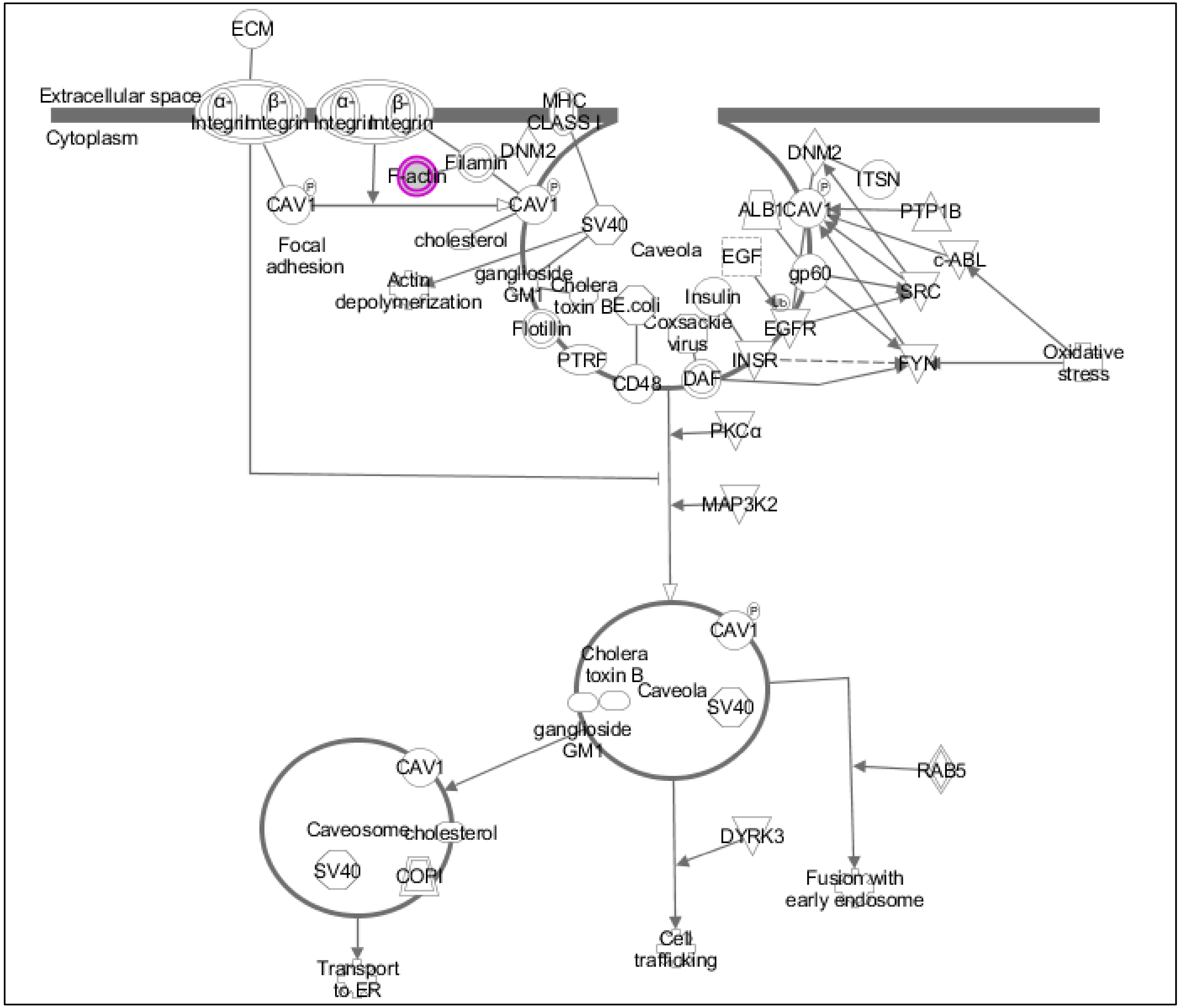

10-Crosstalk between dentritic cells and natrual killer cells

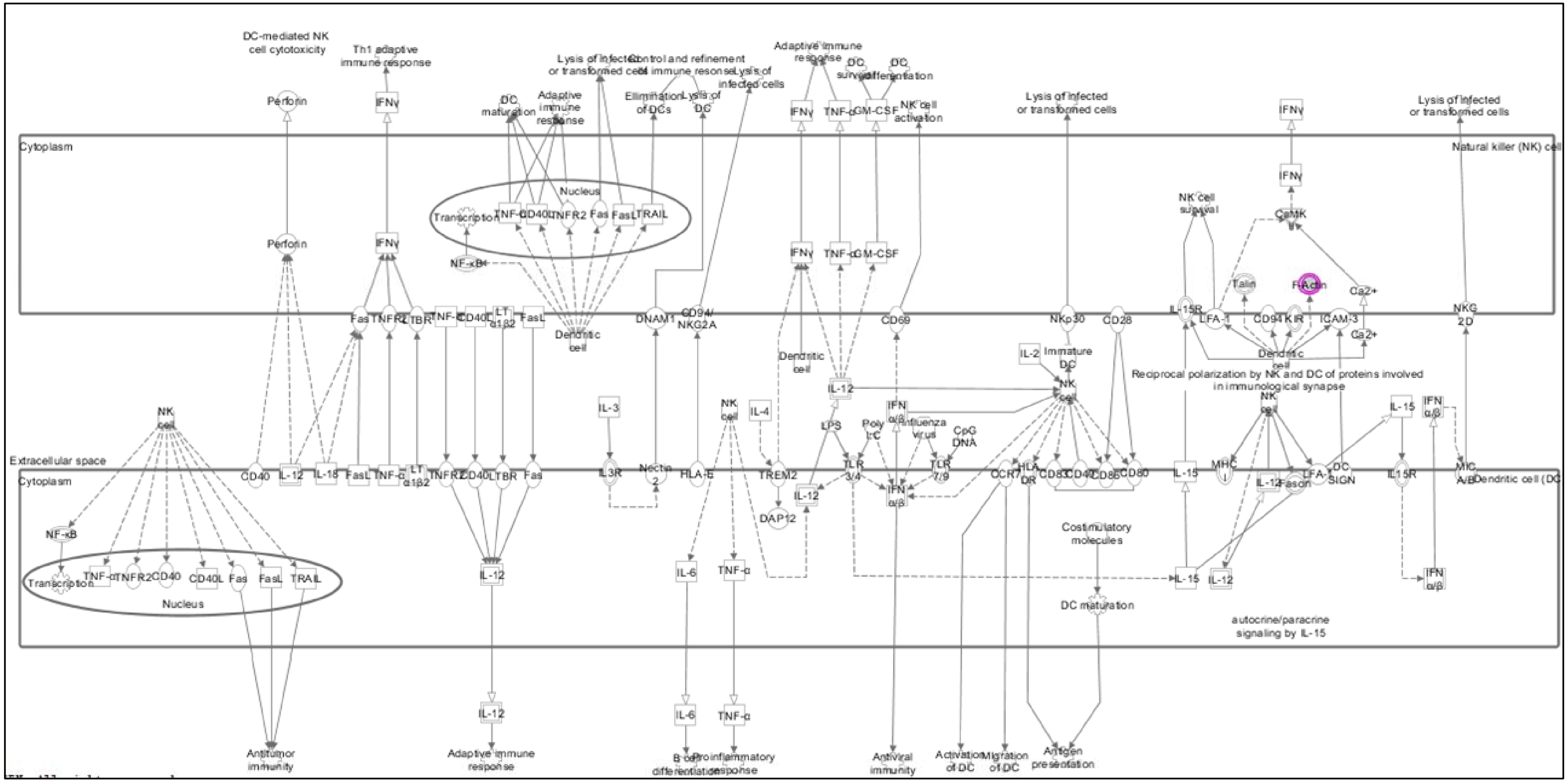

11-FAK signaling

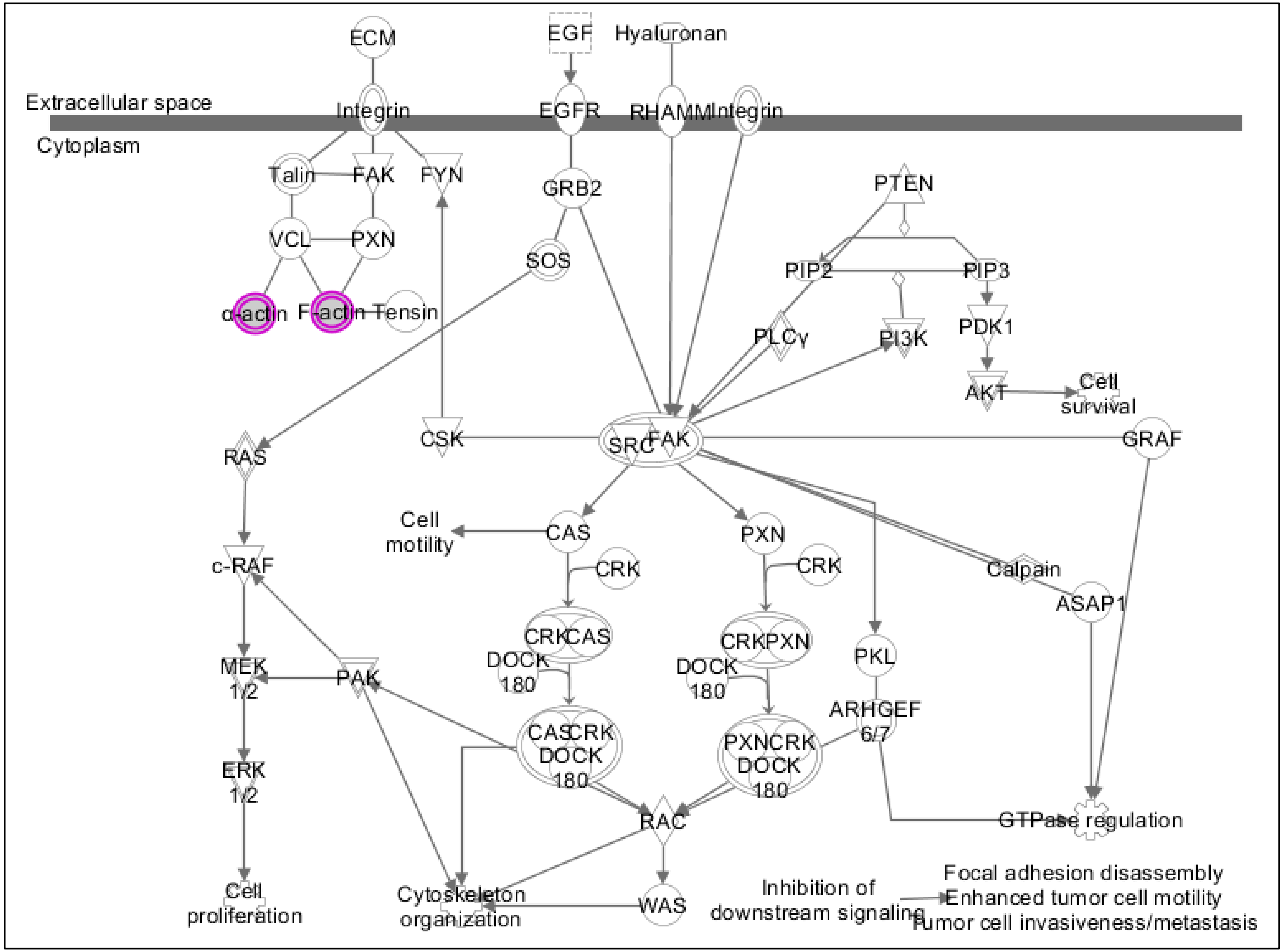

12-Death receptor signaling

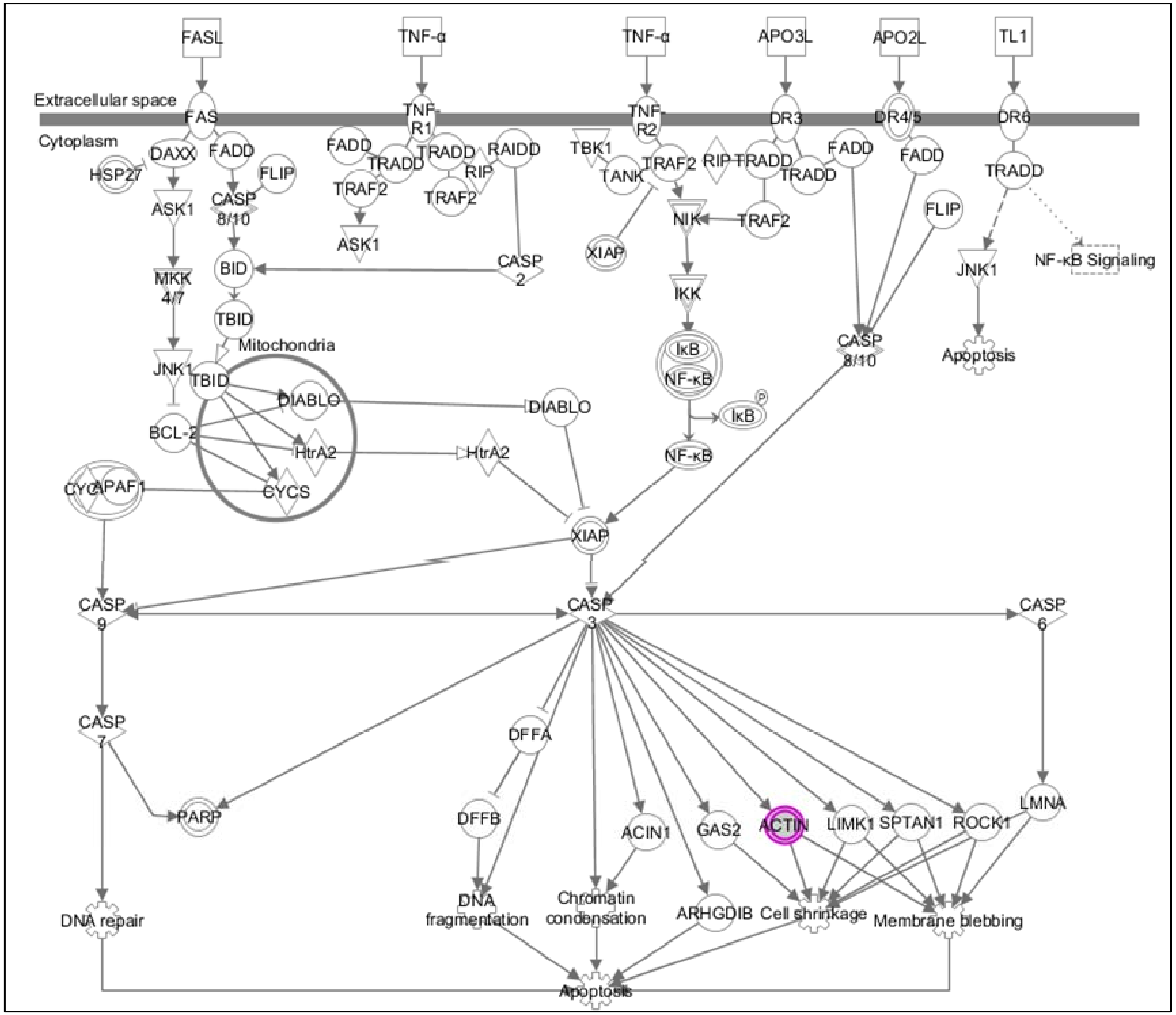

13-Regulation of actin-based motility by Rho

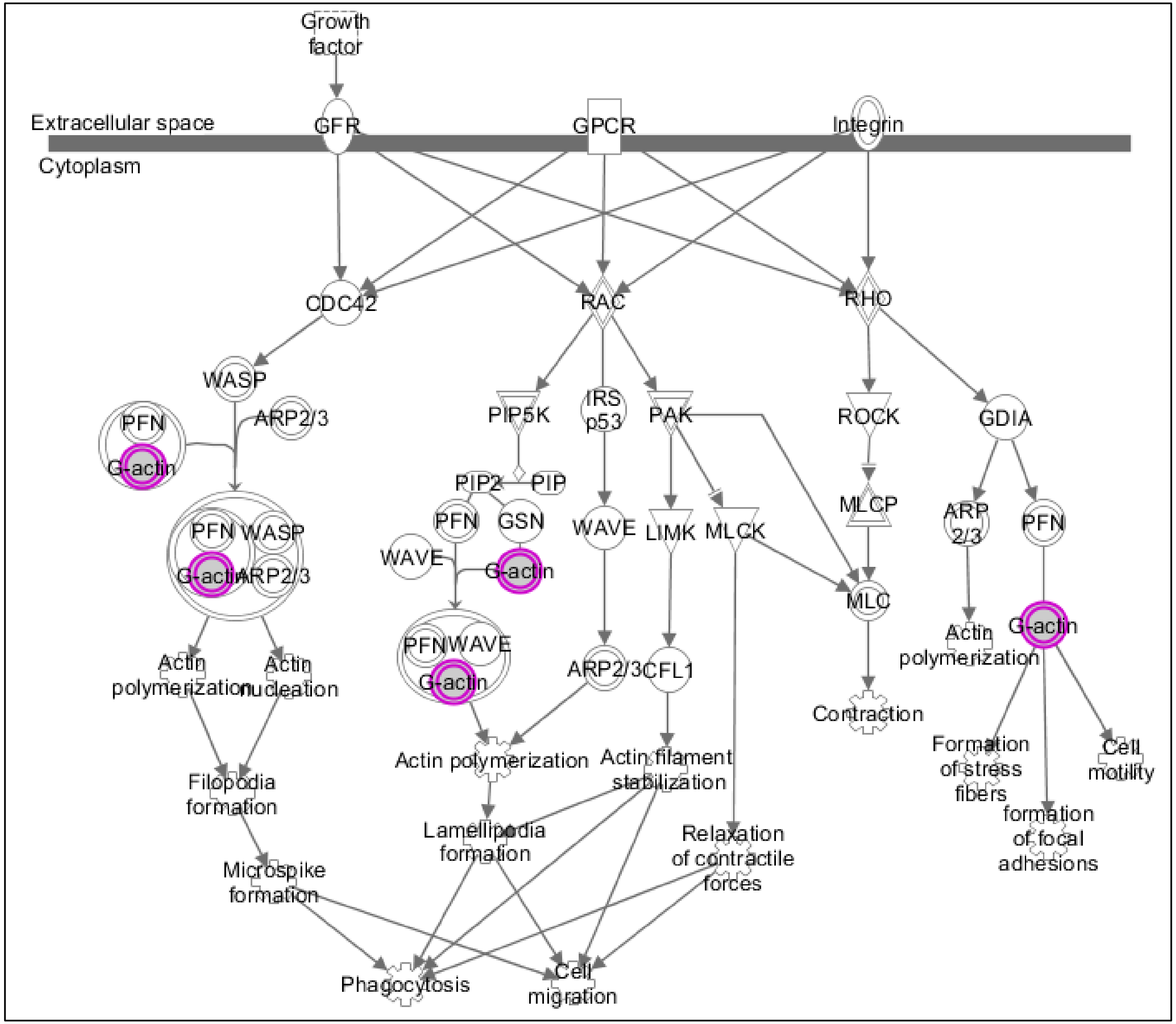

14-Virus entry via endocytic pathways

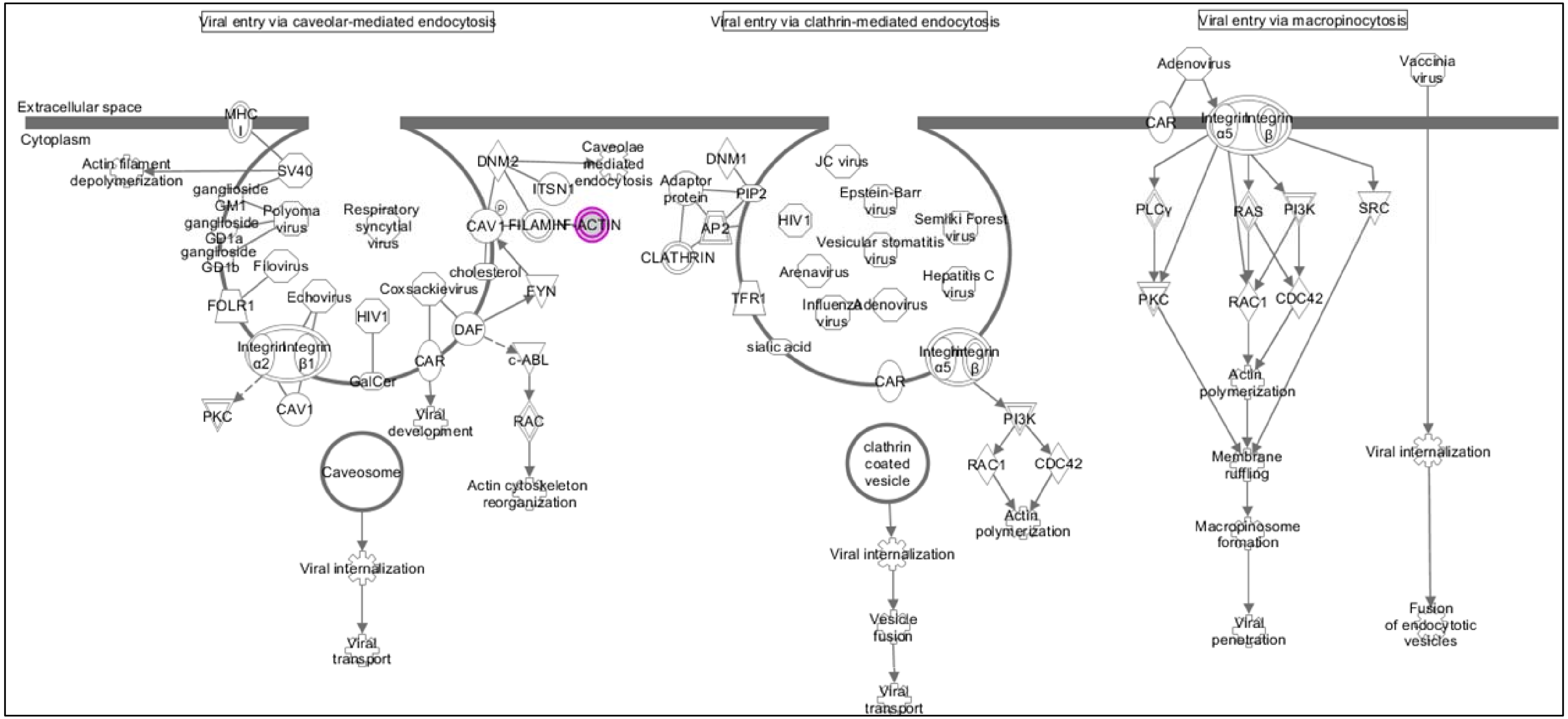

15-VEGF signaling

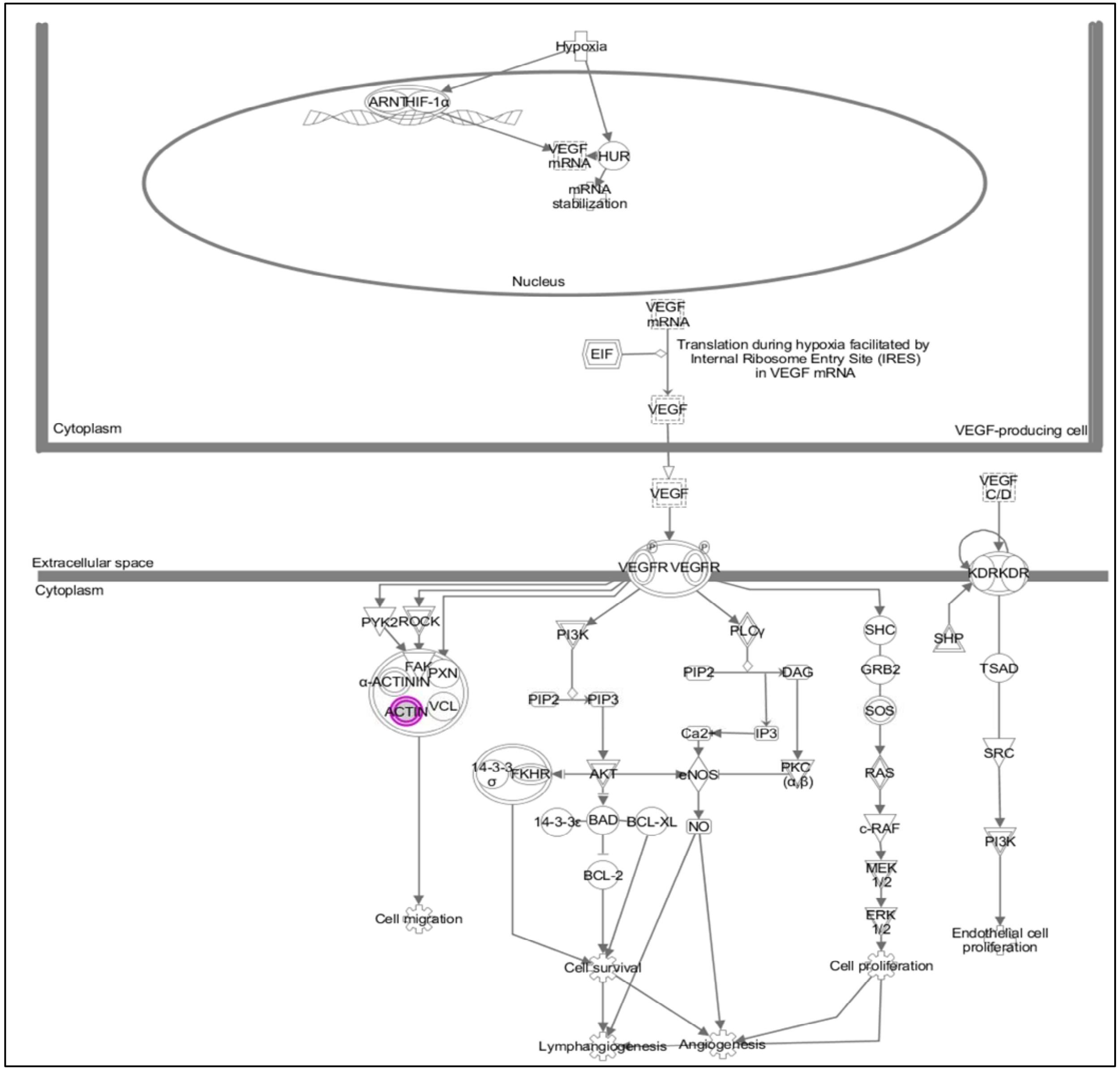

16-Fcy receptor-mediated phagocytosis in macrophages and monocytes

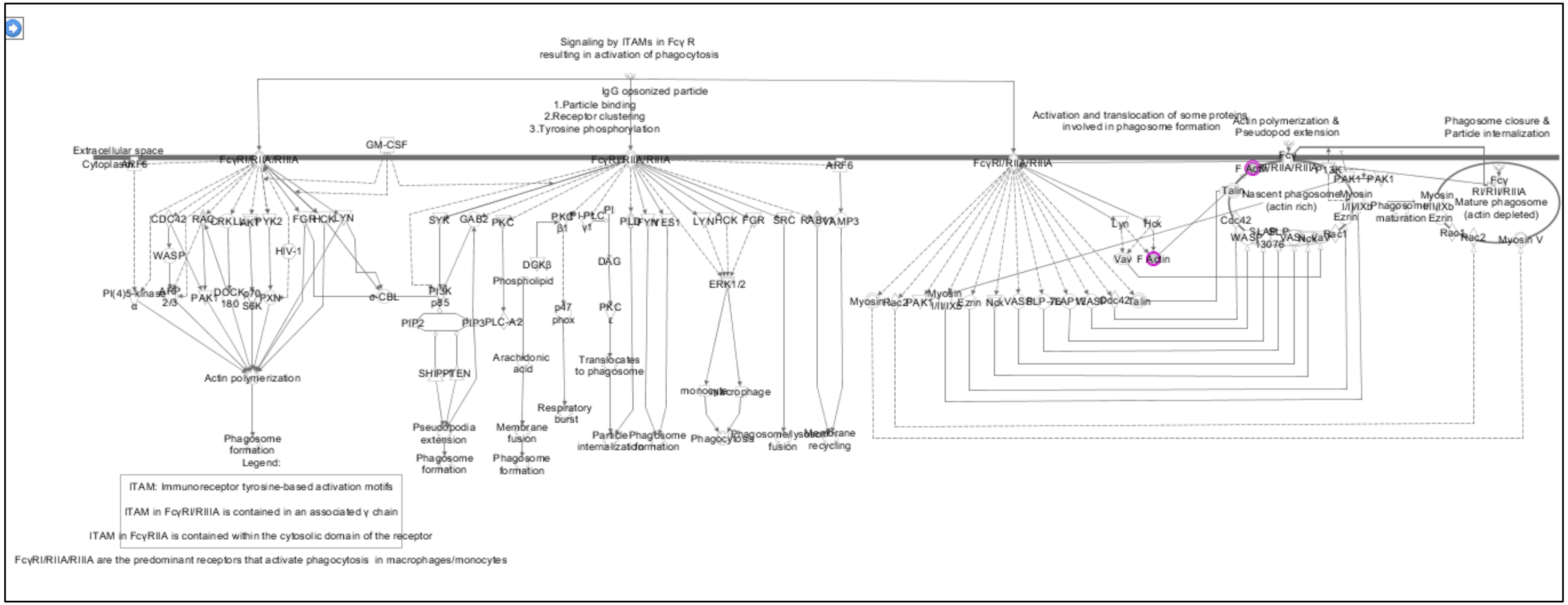

17-Paxillin signaling

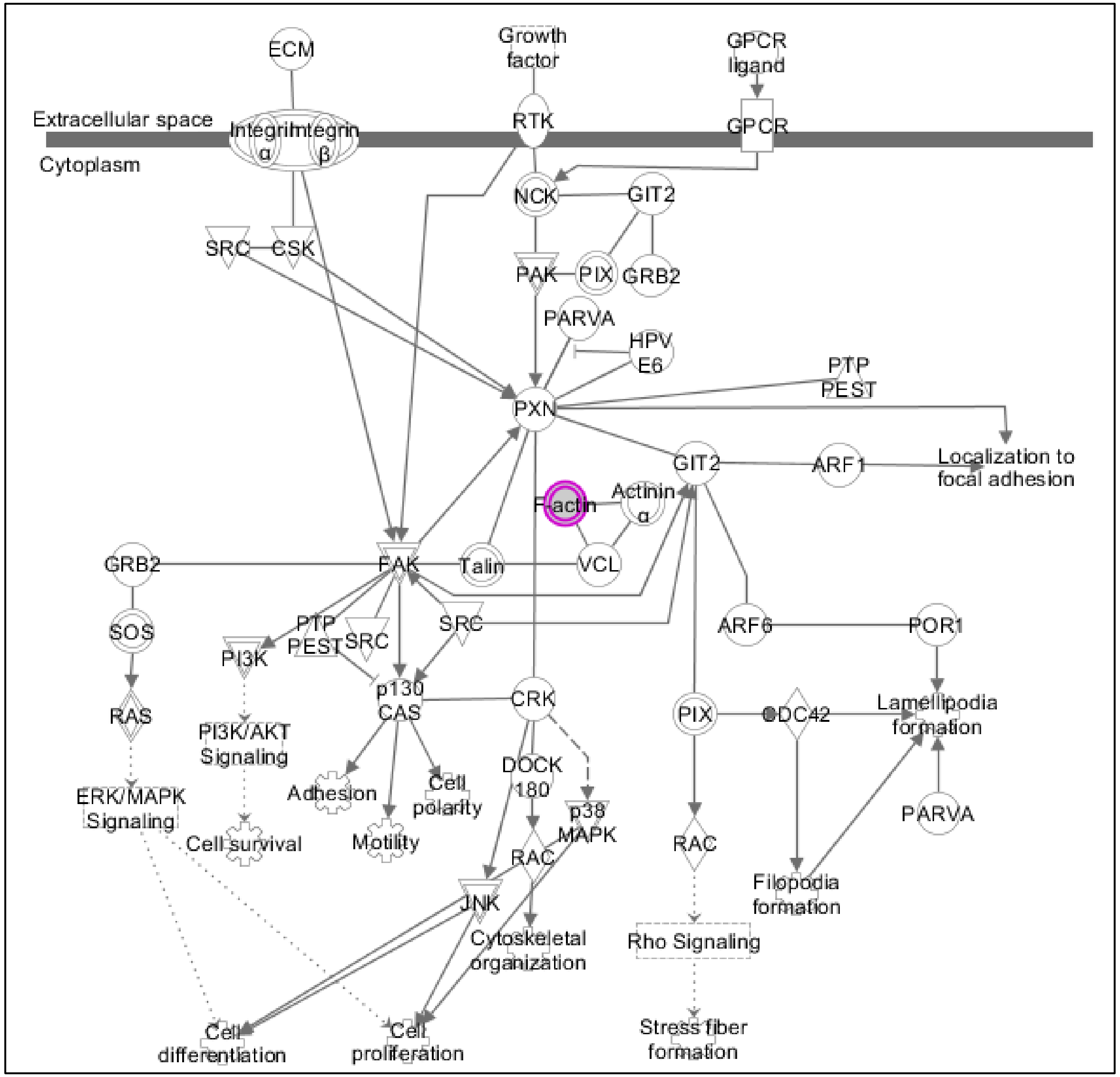

18-RhoA signaling

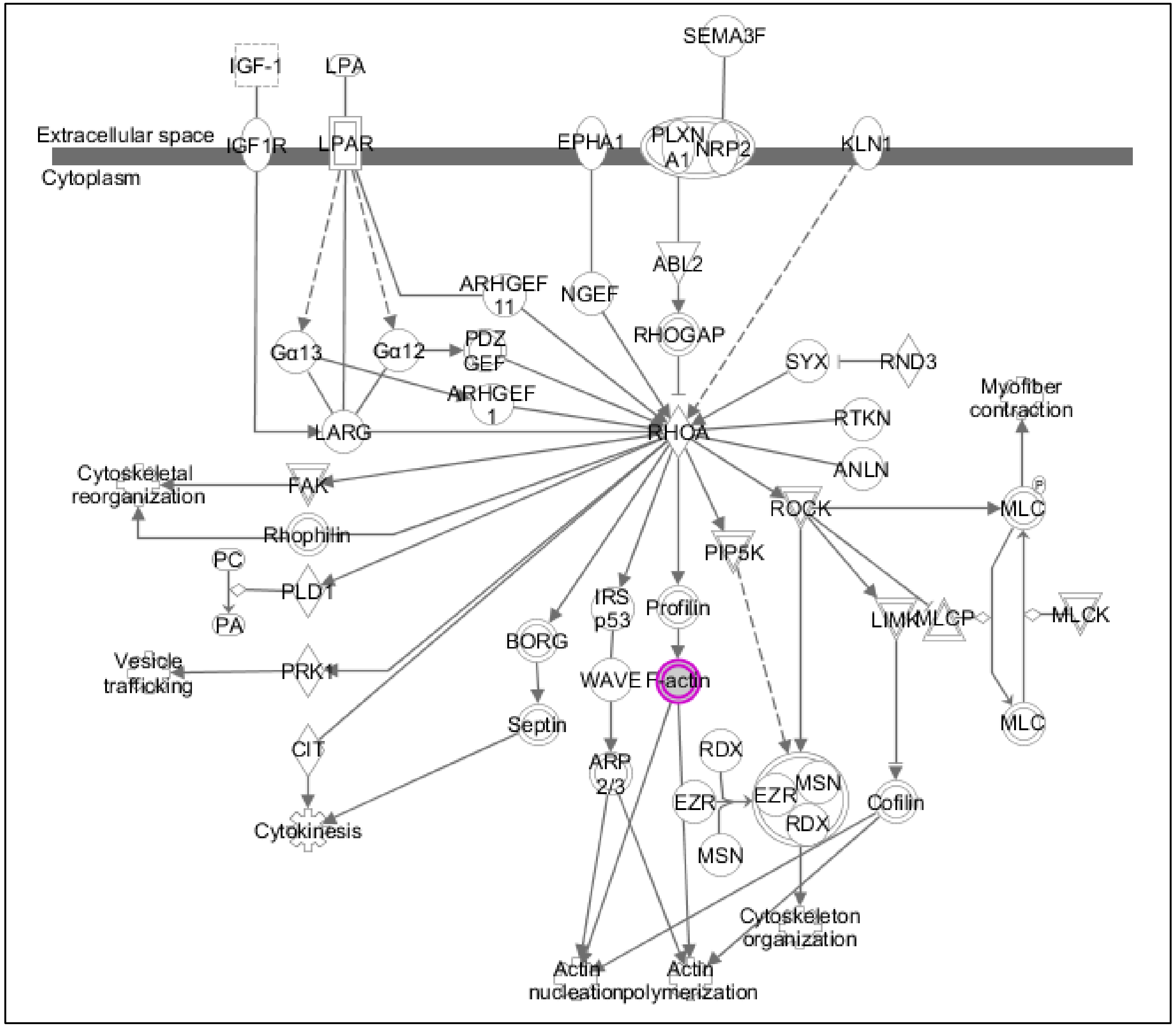

19-Epthelial adherens junction signaling

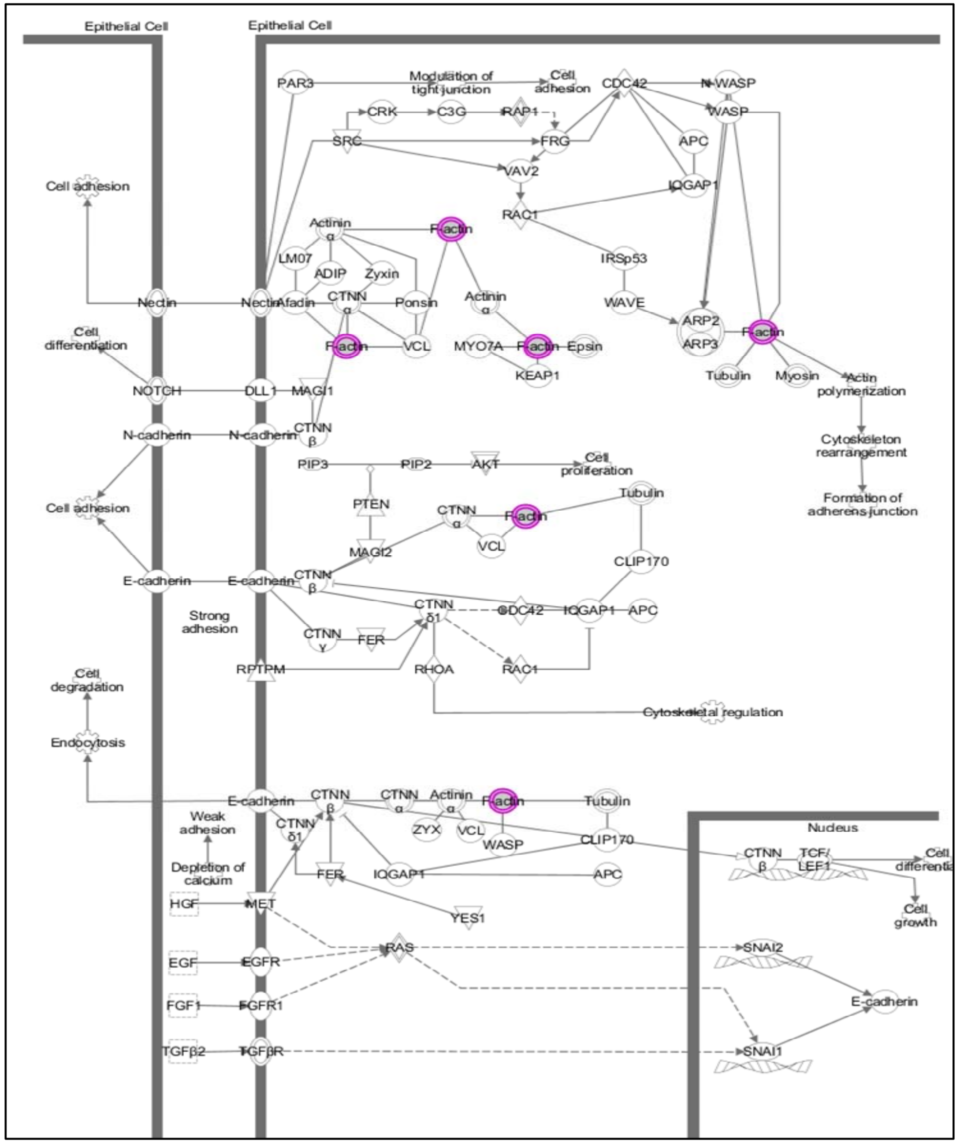

20-Tec kinase signaling

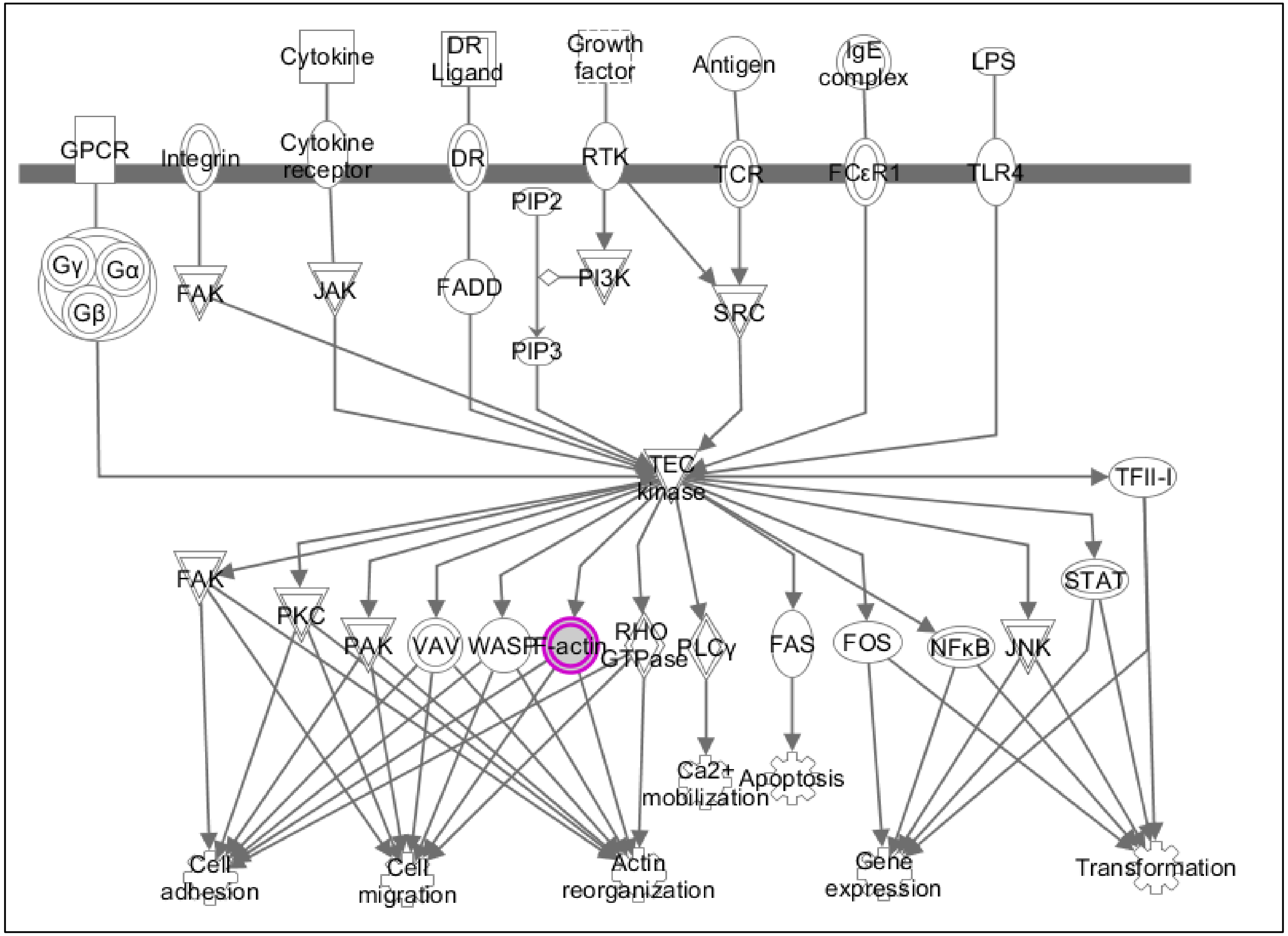

21-Germ cell-sertoli cell junction signaling

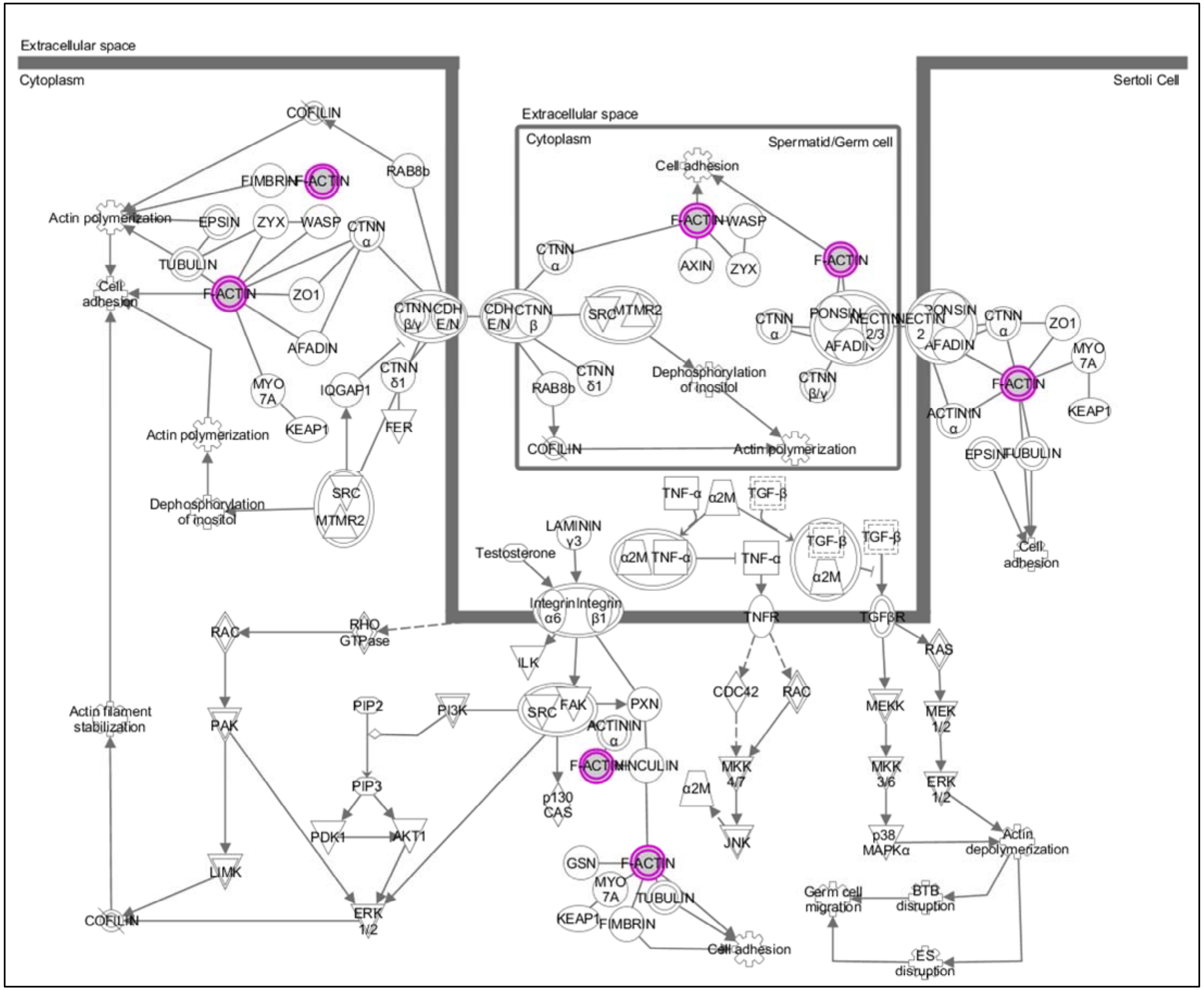

22-Tight junction signaling

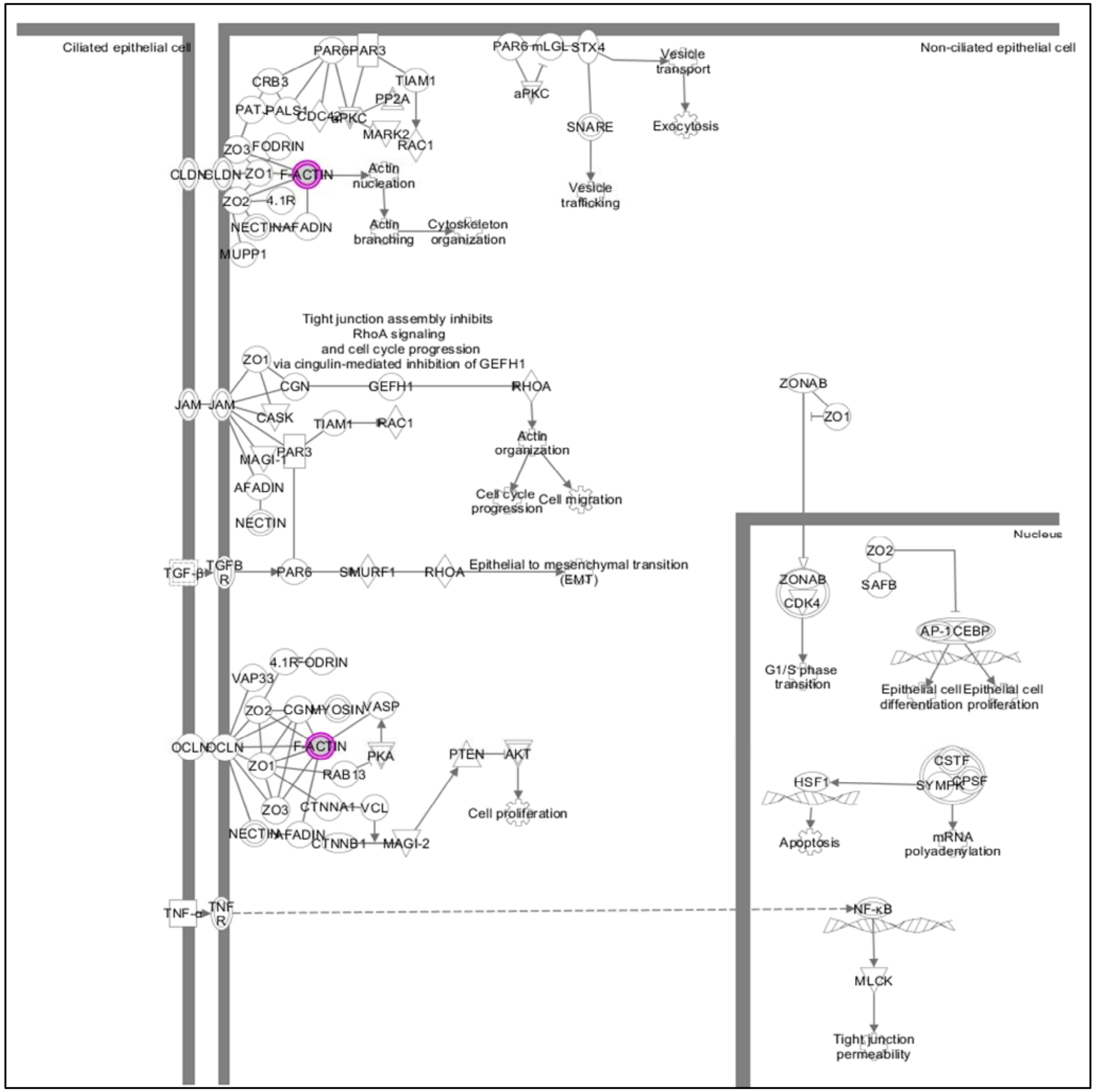

23-RhoGDI signaling

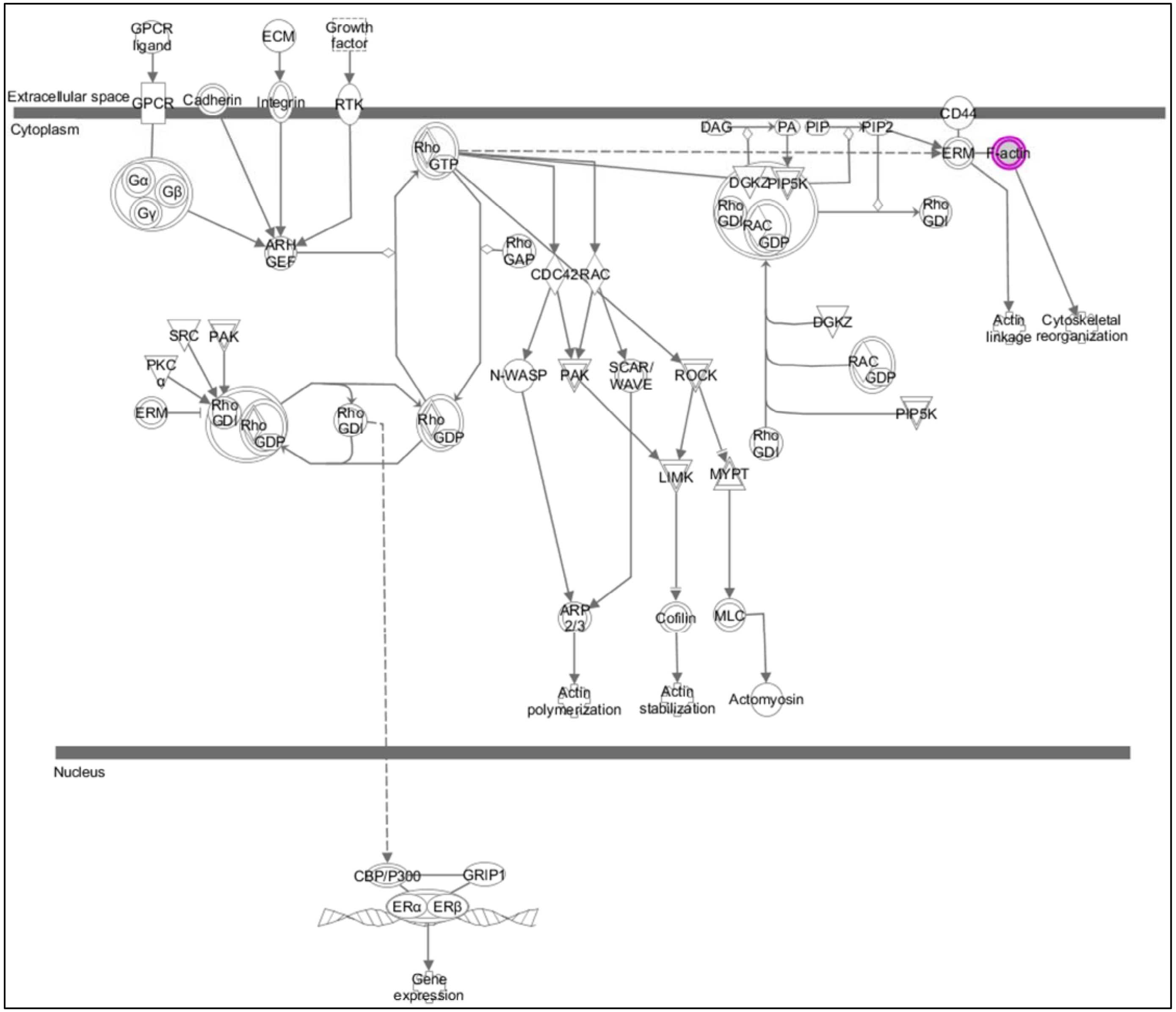

24-NRF2-mediated oxidative stress response

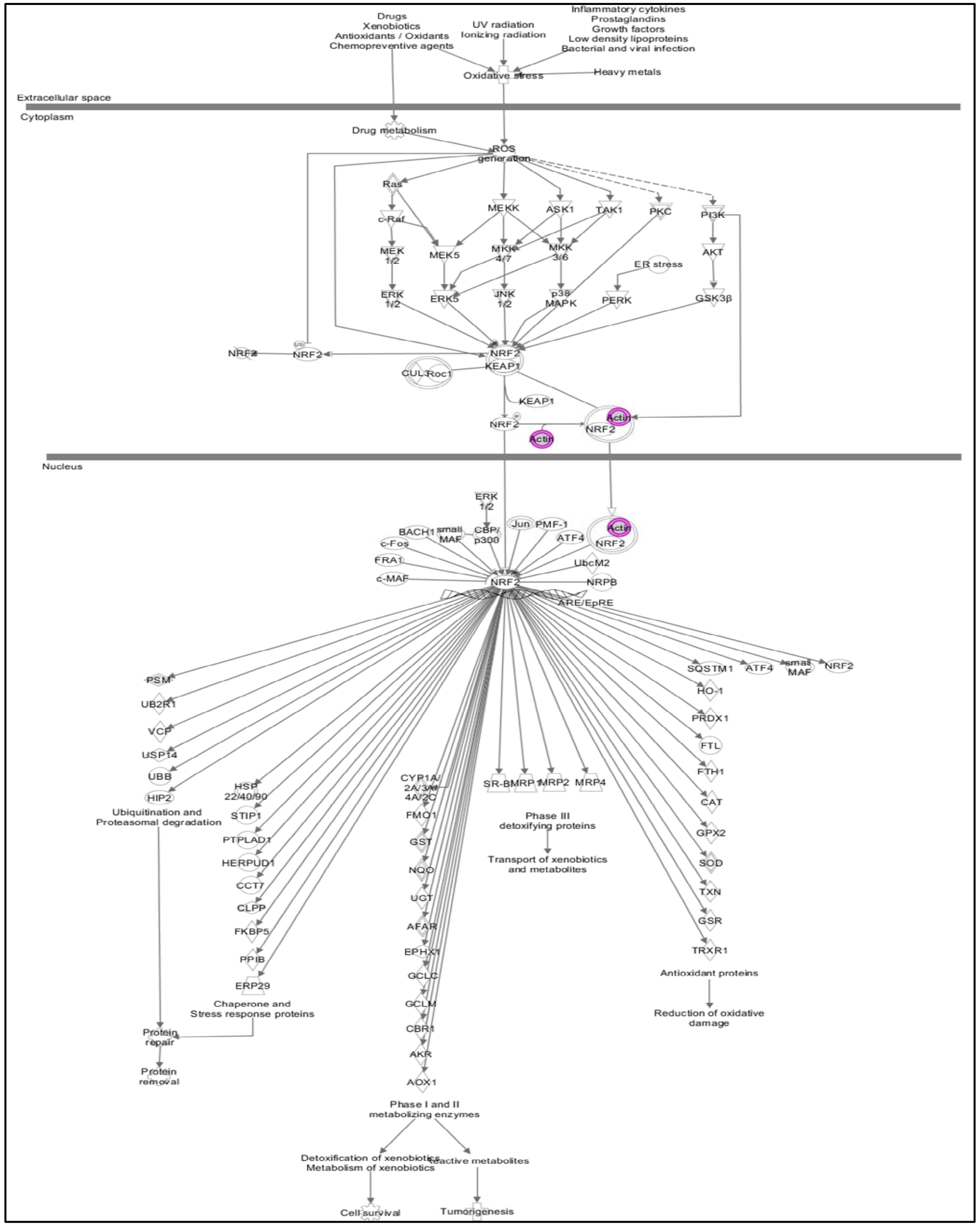

25-ILK signaling

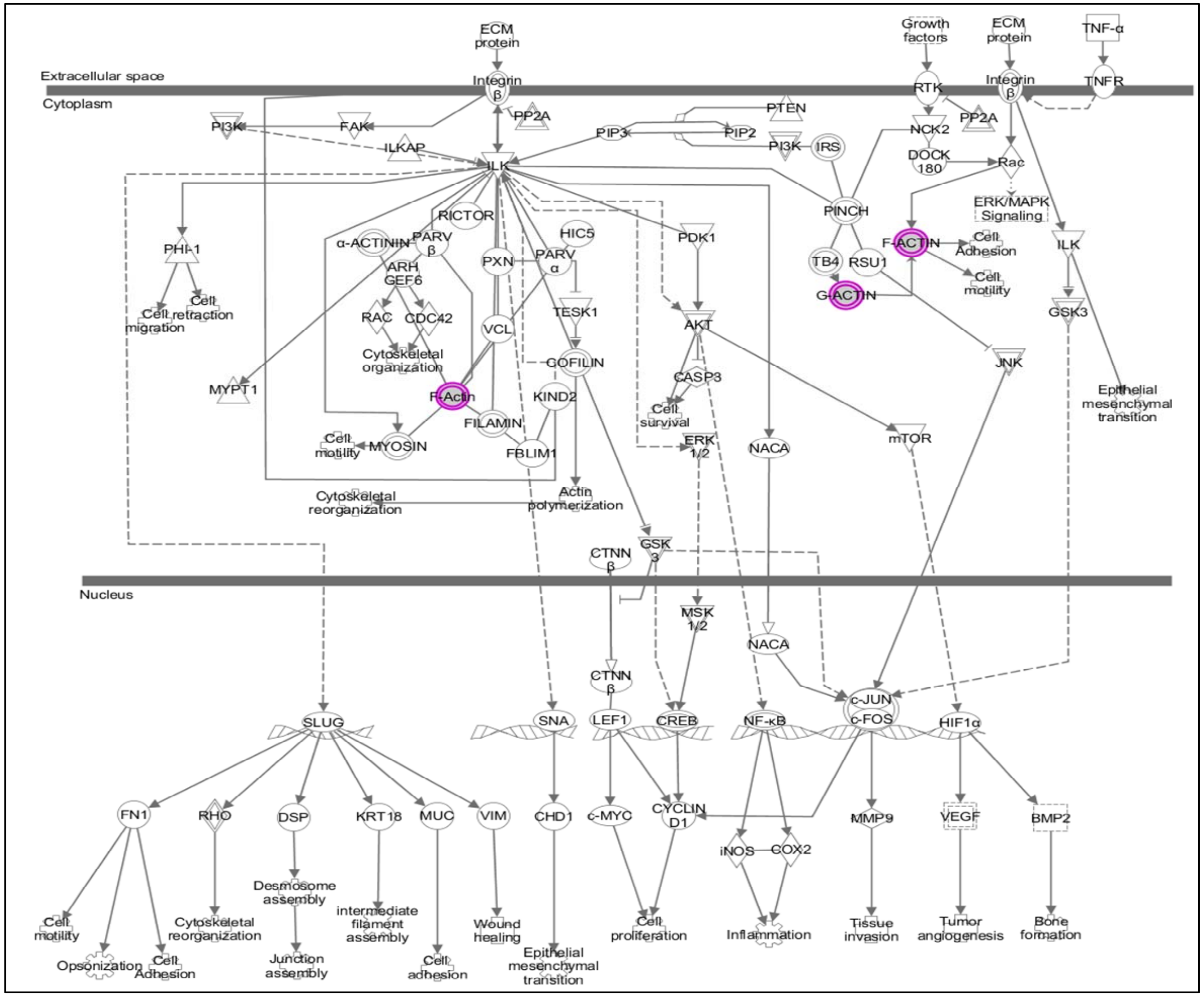

26-Agranulocyte adhesion and diapedesis

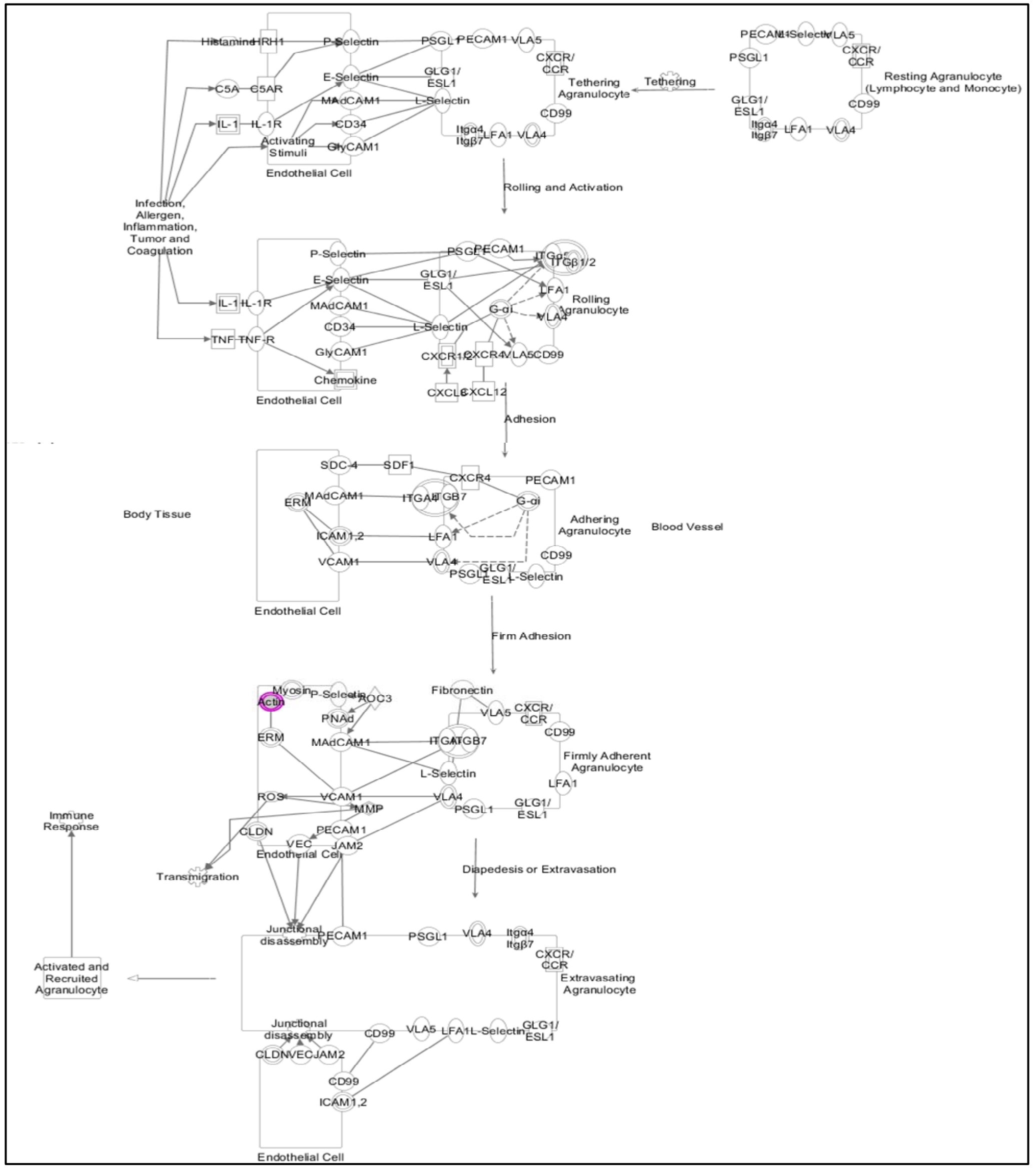

27-Integrin signaling

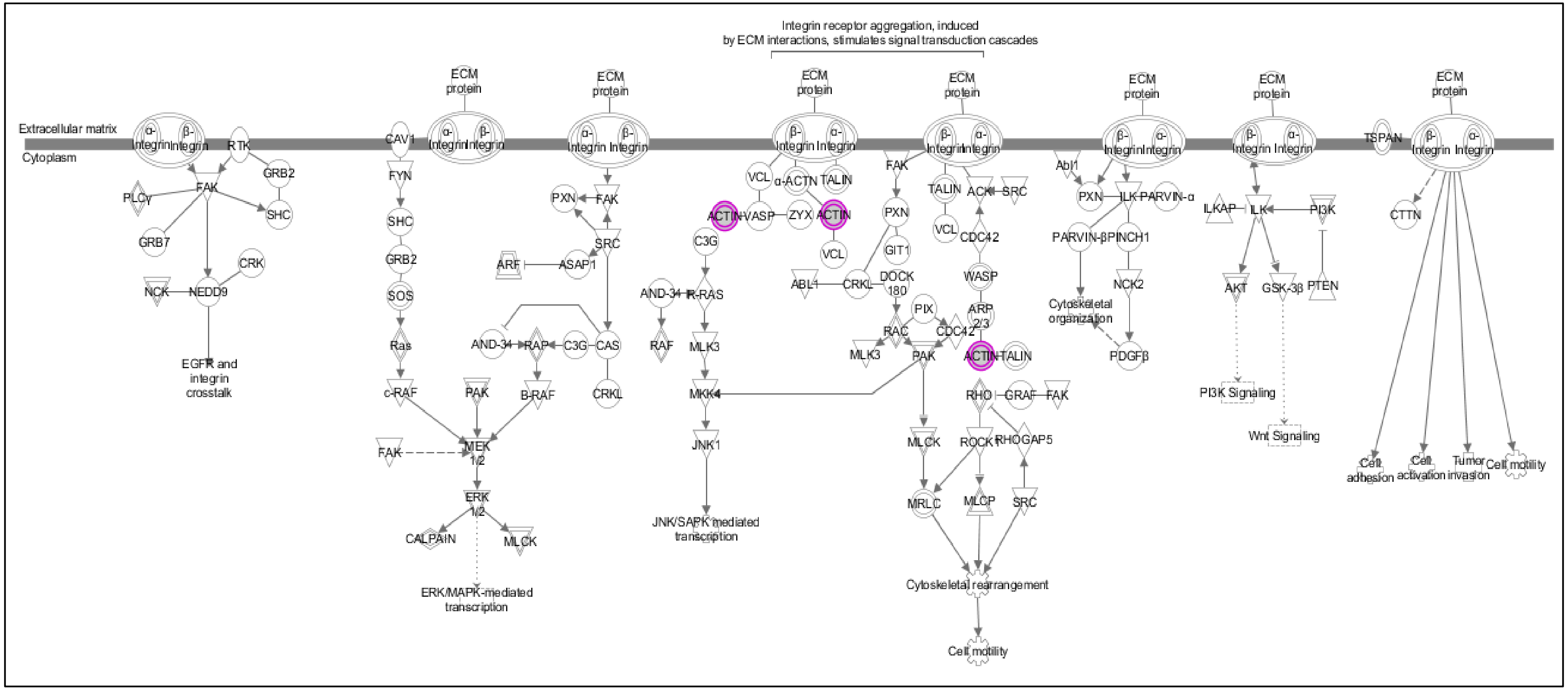

28-Leukocyte extravasation signaling

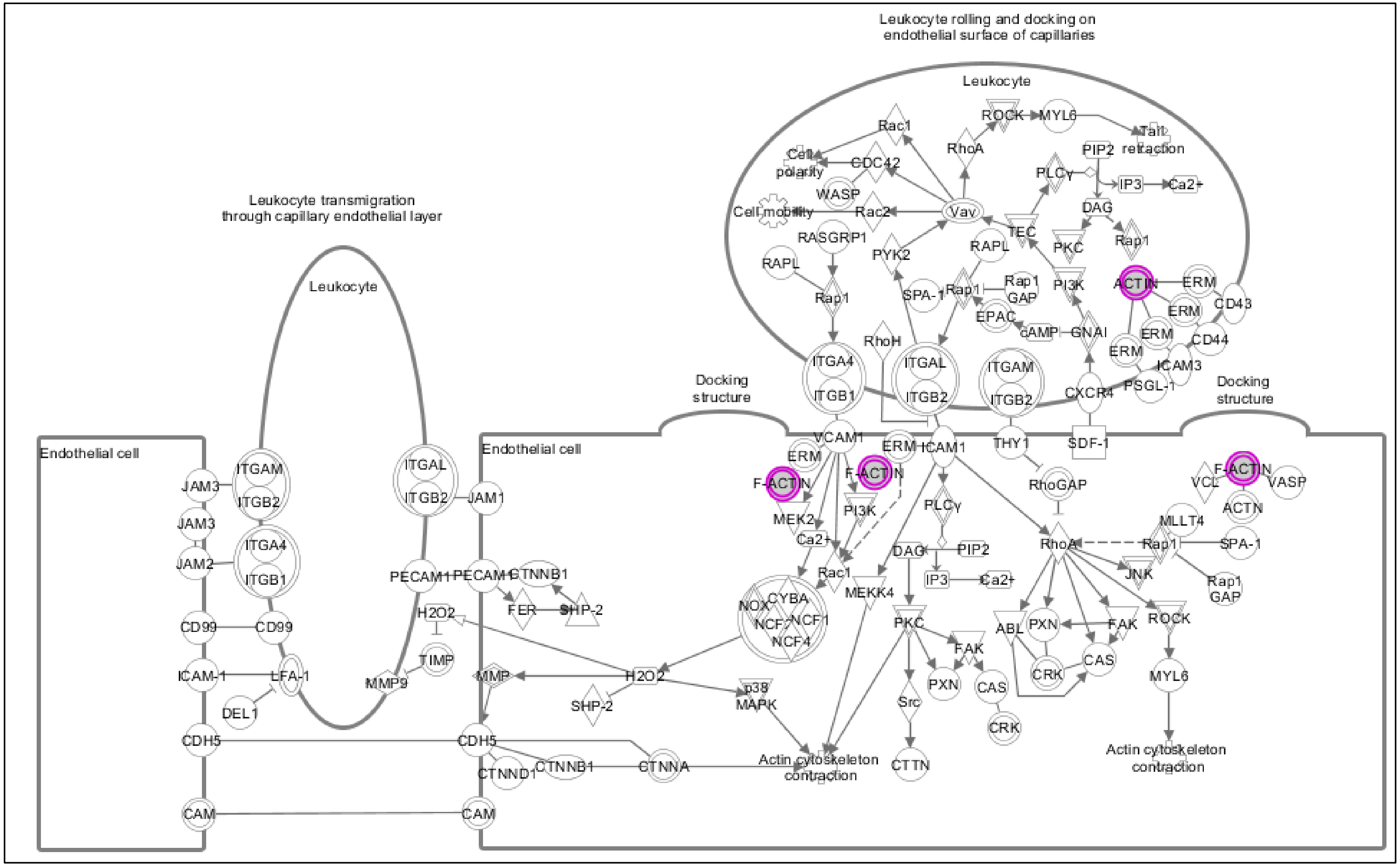

29-Actin cytoskeleton signaling

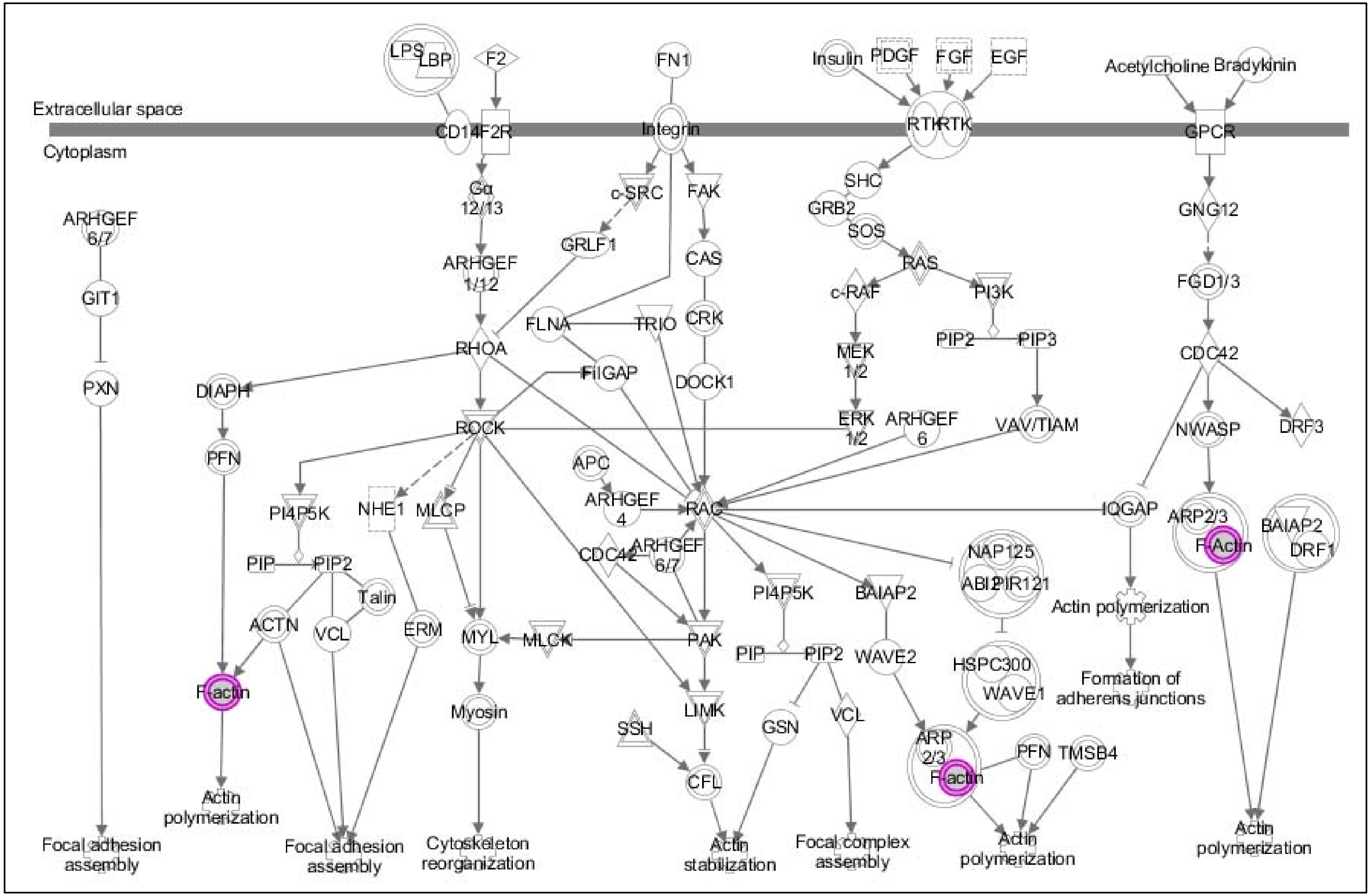

30-Signaling by Rho family GTPases

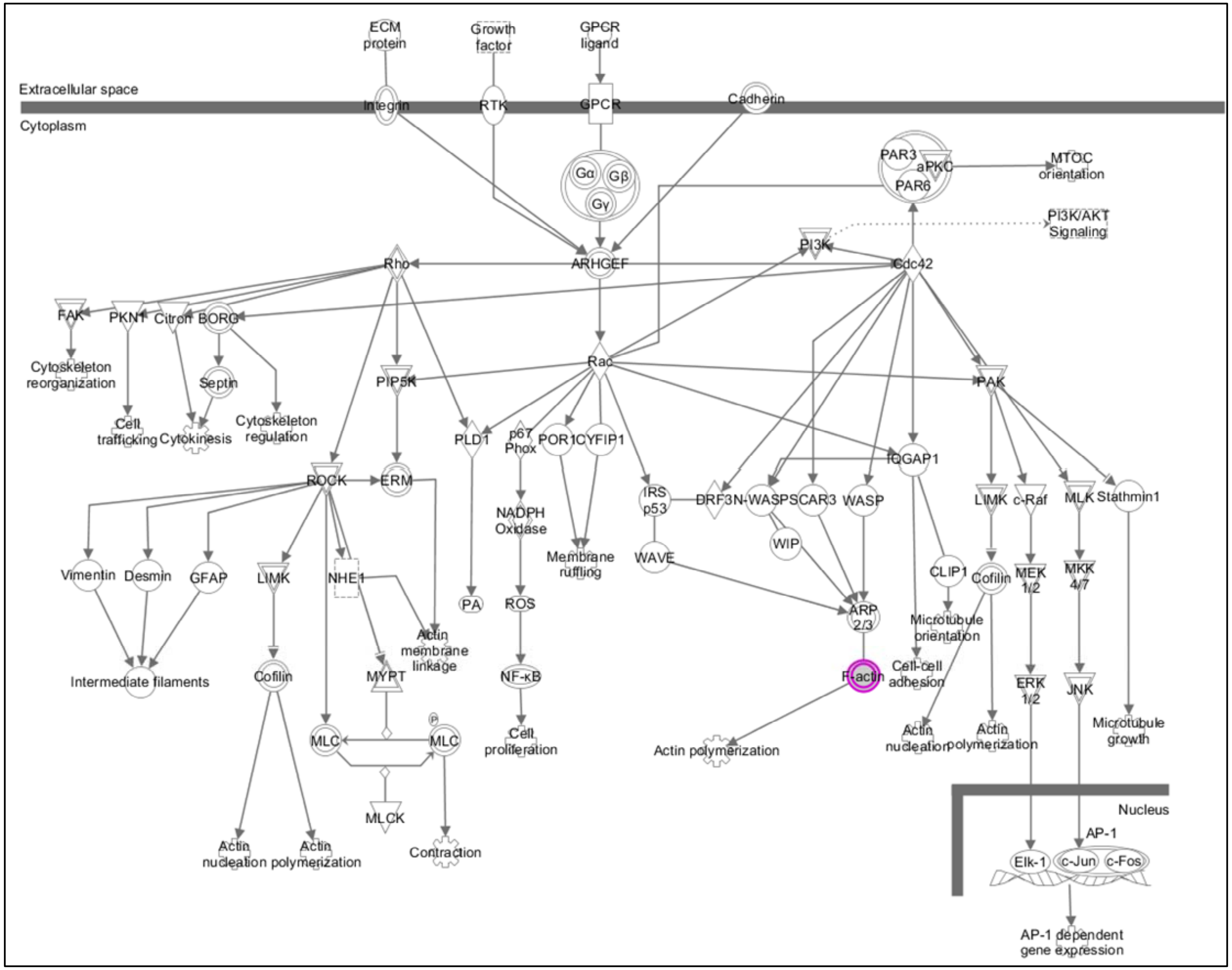

31-Calcium signaling

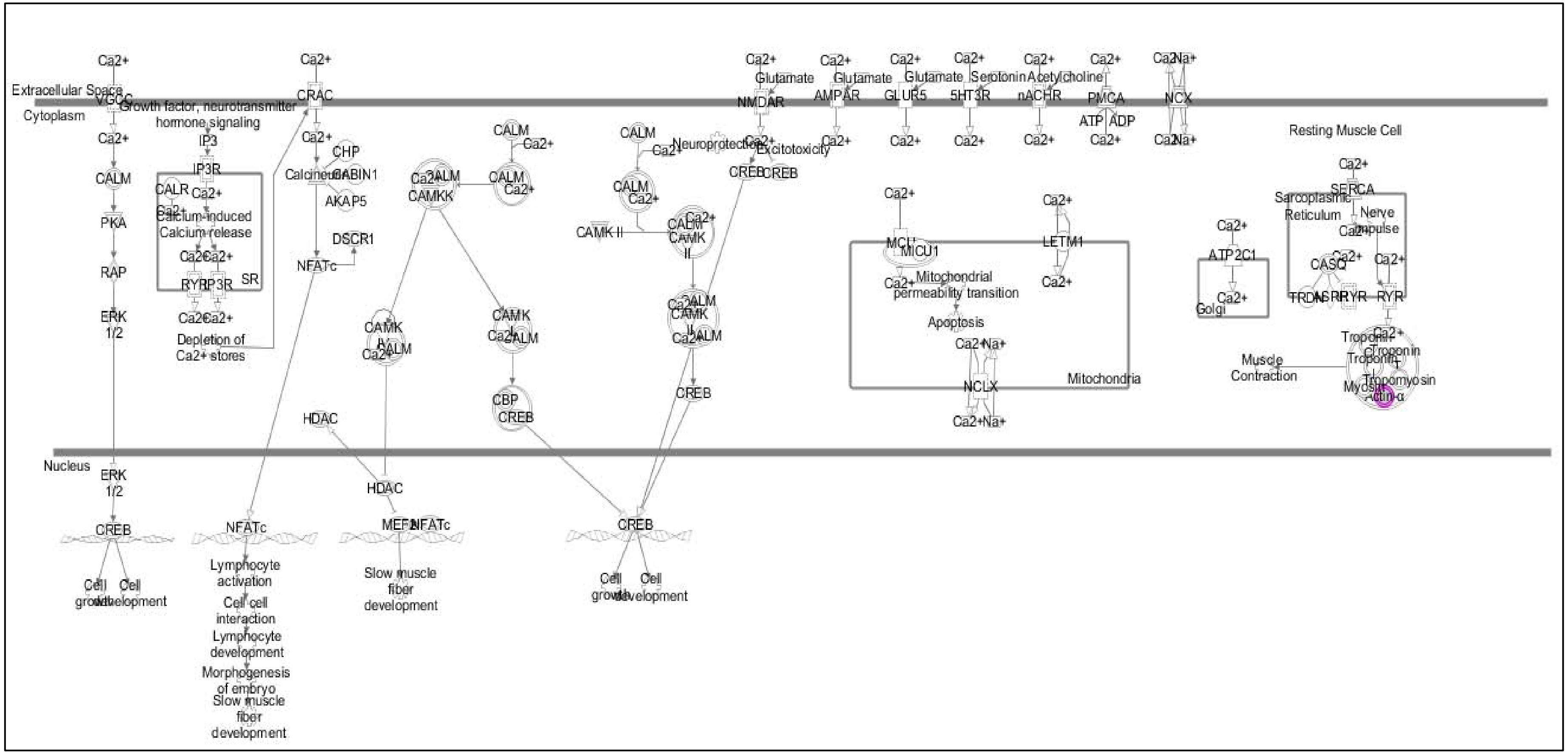

32-protein ubiquitination pathway

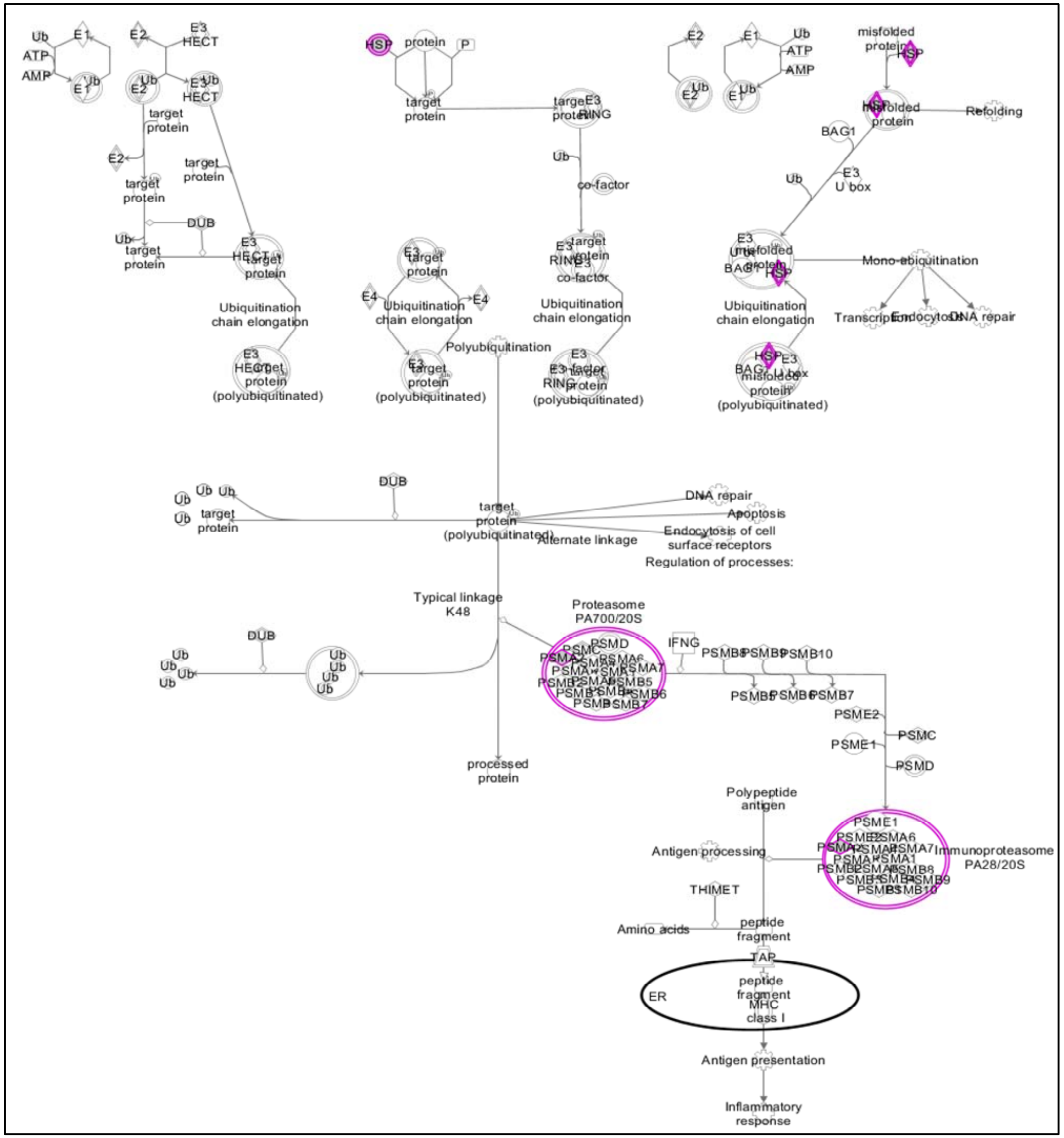

33-Nitric oxide signaling in the cardiovascular system

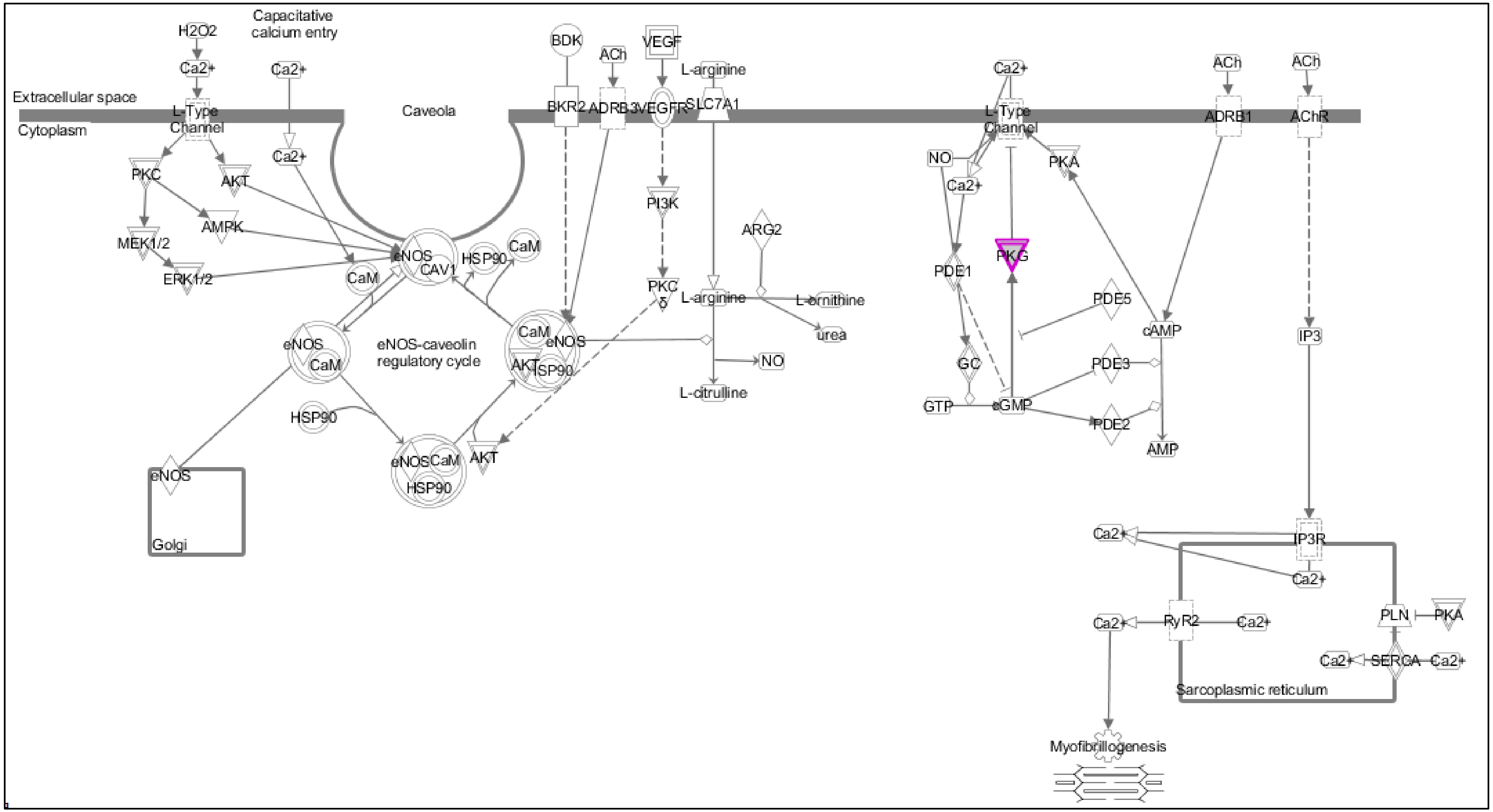

Supplement: Supplementary file 5 [file Presentation_5.zip › Supplemental materials 8.3.pdf]
