# Supplementary material for: Multiomics-Based Signaling Pathway Network Alterations in Human Non-functional Pituitary Adenomas
Source: Front Endocrinol (Lausanne). 2019 Dec 17;10:835. doi: 10.3389/fendo.2019.00835 (PMC6928143; doi:10.3389/fendo.2019.00835)
Supplement: Supplementary file 5 [file Presentation_5.zip › Supplemental materials 9.1.pdf]

Supplemental materials 9.1 Phosphoproteins from pituitary controls for IPA analysis (Dataset 9)

ID	Notes	Molecules	Description	Location	Function	Drug
O15530		PDPK1	3-phosphoinositide dependent protein kinase 1	Cytoplasm	kinase	
P01189		POMC	proopiomelanocortin	Extracellular Space	other	
P01241		GH1	growth hormone 1	Extracellular Space	growth factor	
P02671		FGA	fibrinogen alpha chain	Extracellular Space	other	F2
P04004		VTN	vitronectin	Extracellular Space	other	
P05060		CHGB	chromogranin B (secretogranin 1)	Extracellular Space	other	
P05386		RPLP1	ribosomal protein, large, P1	Cytoplasm	other	
P05387	D	RPLP2*	ribosomal protein, large, P2	Cytoplasm	other	
P05387	D	RPLP2*	ribosomal protein, large, P2	Cytoplasm	other	
P05388		RPLP0	ribosomal protein, large, P0	Cytoplasm	other	

P07900	HSP90AA1	heat shock protein 90kDa alpha (cytosolic), class A member 1	Cytoplasm	enzyme	alvespimycin, retaspimycin, luminespib, cisplatin
P07910	HNRNPC	heterogeneous nuclear ribonucleoprotein C (C1/C2)	Nucleus	other	
P10451	SPP1	secreted phosphoprotein 1	Extracellular Space	cytokine	
P10645	CHGA	chromogranin A (parathyroid secretory protein 1)	Cytoplasm	other	
P13521	SCG2	secretogranin II	Extracellular Space	cytokine	
P13861	PRKAR2A	protein kinase, cAMP-dependent, regulatory, type II, alpha	Cytoplasm	kinase	
P22466	GAL	galanin/GMAP prepropeptide	Extracellular Space	other	
P23396	RPS3	ribosomal protein S3	Cytoplasm	enzyme	
P29692	EEF1D	eukaryotic translation elongation factor 1 delta (guanine nucleotide exchange protein)	Cytoplasm	translation regulator	
P35221	CTNNA1	catenin (cadherin-associated protein), alpha 1, 102kDa	Plasma Membrane	other	
P50502	ST13	suppression of tumorigenicity 13 (colon carcinoma) (Hsp70 interacting protein)	Cytoplasm	other	
P51858	HDGF	hepatoma-derived growth factor	Extracellular Space	growth factor	

P60174		TPI1	triosephosphate isomerase 1	Cytoplasm	enzyme
Q01518		CAP1	CAP, adenylate cyclase-associated protein 1 (yeast)	Plasma Membrane	other
Q15019		Septin2	septin 2	Cytoplasm	enzyme
Q15185		PTGES3	prostaglandin E synthase 3 (cytosolic)	Cytoplasm	enzyme
Q9H3Z4		DNAJC5	DnaJ (Hsp40) homolog, subfamily C, member 5	Plasma Membrane	other
Q9UKM9		RALY	RALY heterogeneous nuclear ribonucleoprotein	Nucleus	other
P62805	unmapped				
