# Supplementary material for: Multiomics-Based Signaling Pathway Network Alterations in Human Non-functional Pituitary Adenomas
Source: Front Endocrinol (Lausanne). 2019 Dec 17;10:835. doi: 10.3389/fendo.2019.00835 (PMC6928143; doi:10.3389/fendo.2019.00835)

Supplemental materials 9.3
Statistically significant canonical pathways derived from phosphoproteins from pituitary controls for IPA analysis (Dataset 9)

1. EIF2 signaling
2. Telomerase signaling
3. eNOS signaling
4. Aldosterone signaling in epithelial cells
5. PPARa_RXRa activation
6. Endometrial cancer signaling
7. Growth hormone signaling
8. Leptin signaling in obesity
9. Glucocorticoid receptor signaling
10. Melanocyte development and pigmentation signaling
11. Prostate cancer signaling
12. TR_RXR activation
13. Neurigulin signaling
14. G beta gamma signaling
15. IGF-1 signaling
16. Nitric oxide signaling in cardiovascular system
17. Androgen signaling
18. Corticotropin releasing hormone signaling
19. LXR_RXR activation
20. PI3K_AKT signaling
21. FXR_RXR activation
22. Insulin receptor signaling
23. Aryl hydrocarbon receptor signaling
24. AMPK signaling
25. Regulation of eIF4 and p70S6K signaling
26. Germ cell-sertoli cell junction signaling
27. Prostanoid biosynthesis
28. Tight junction signaling
29. Acute phase response signaling
30. RAR activation
31. Extrinsic prothrombin activation pathway
32. Sertoli cell-sertoli cell junction signaling
33. Sucrose degradation V (mammalian)
34. mTOR signaling
35. Huntington's disease signaling
36. Intrinsic prothrombin activation pathway
37. Sonic hedgehog signaling
38. Protein ubiquitination pathway
39. Coagulation system
40. Role of JAK2 in hormone-like cytokine signaling
41. G-protein coupled receptor signaling
42. Xenobiotic metabolism signaling
43. Glycolysis

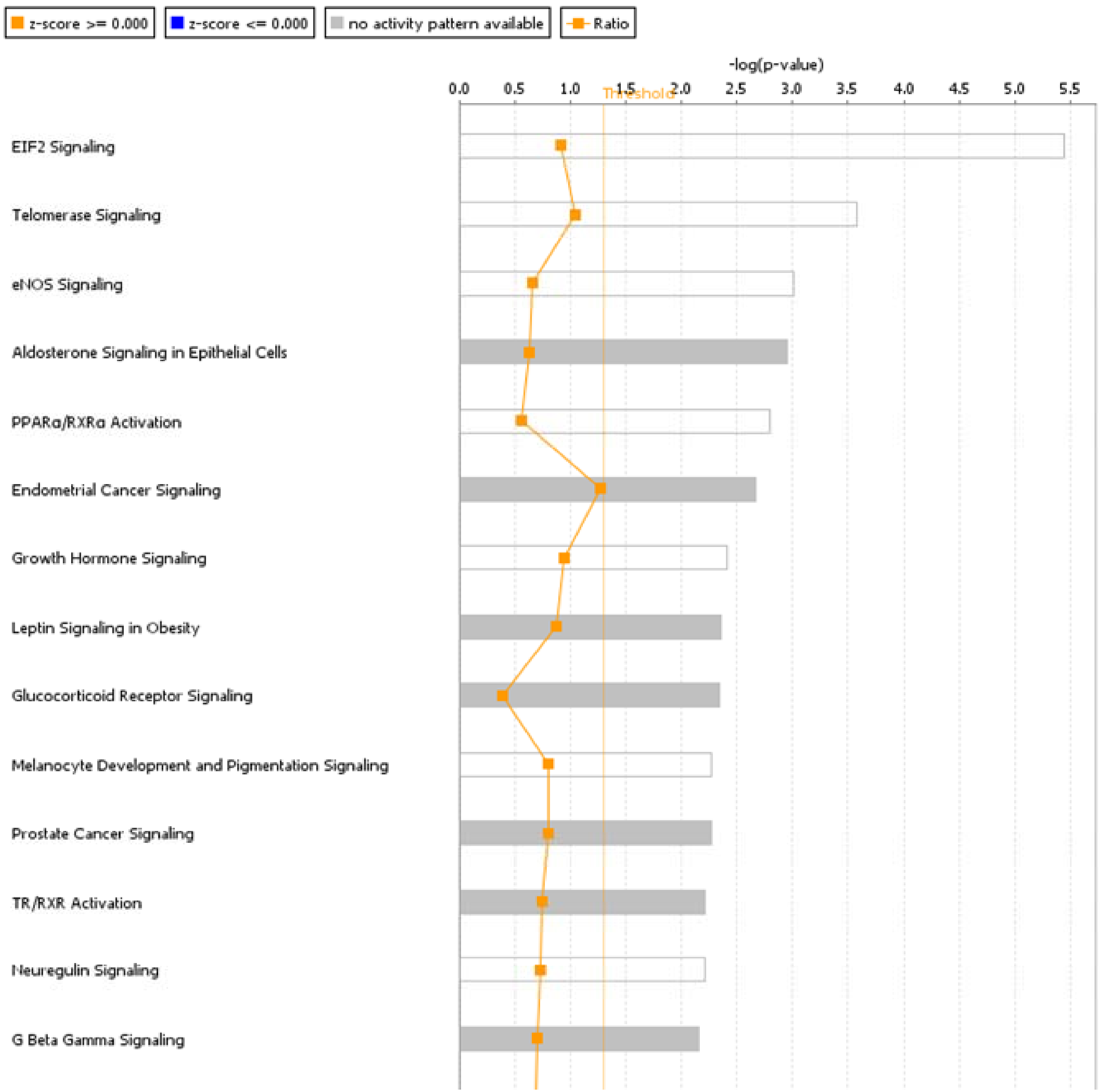
z-score >= 0.000
z-score <= 0.000
no activity pattern available
Ratio
-log(p-value)
0.0
0.5
1.0
1.5
2.0
2.5
3.0
3.5
4.0
4.5
5.0
5.5
Threshold
EIF2 Signaling
Telomerase Signaling
eNOS Signaling
Aldosterone Signaling in Epithelial Cells
PPARα/RXRα Activation
Endometrial Cancer Signaling
Growth Hormone Signaling
Leptin Signaling in Obesity
Glucocorticoid Receptor Signaling
Melanocyte Development and Pigmentation Signaling
Prostate Cancer Signaling
TR/RXR Activation
Neuregulin Signaling
G Beta Gamma Signaling

1-EIF2 signaling

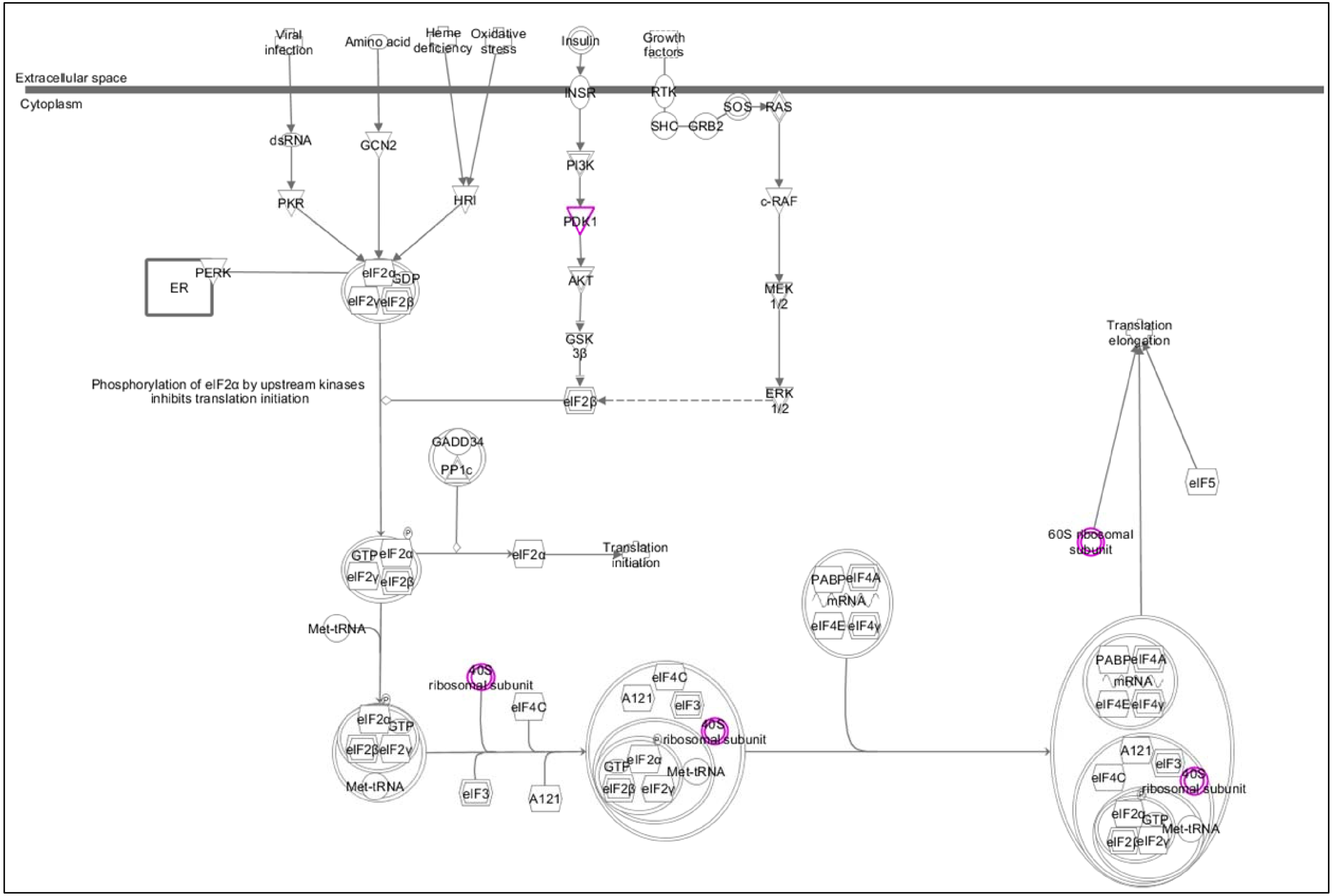

2-Telomerase signaling

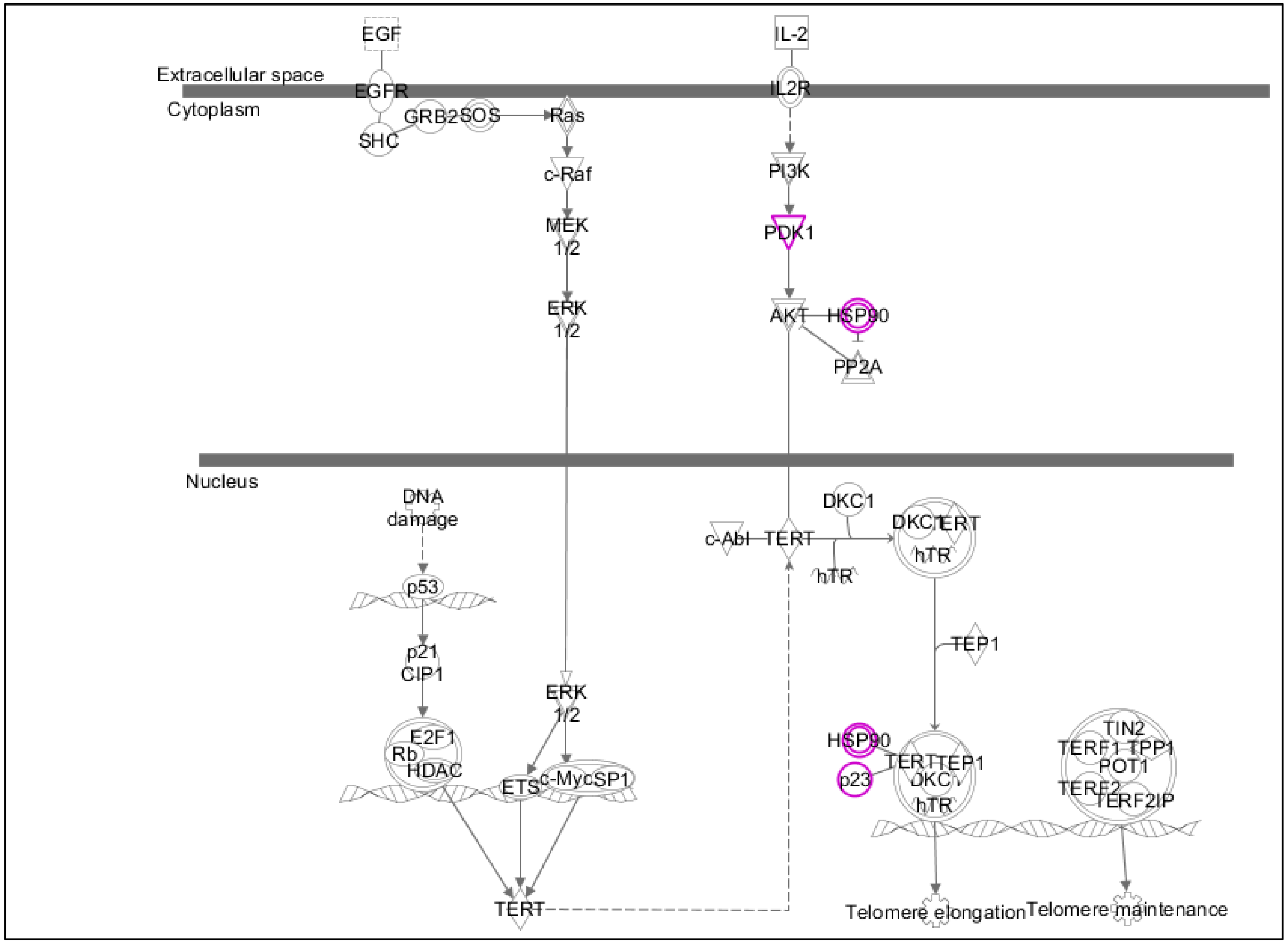

3-eNOS signaling

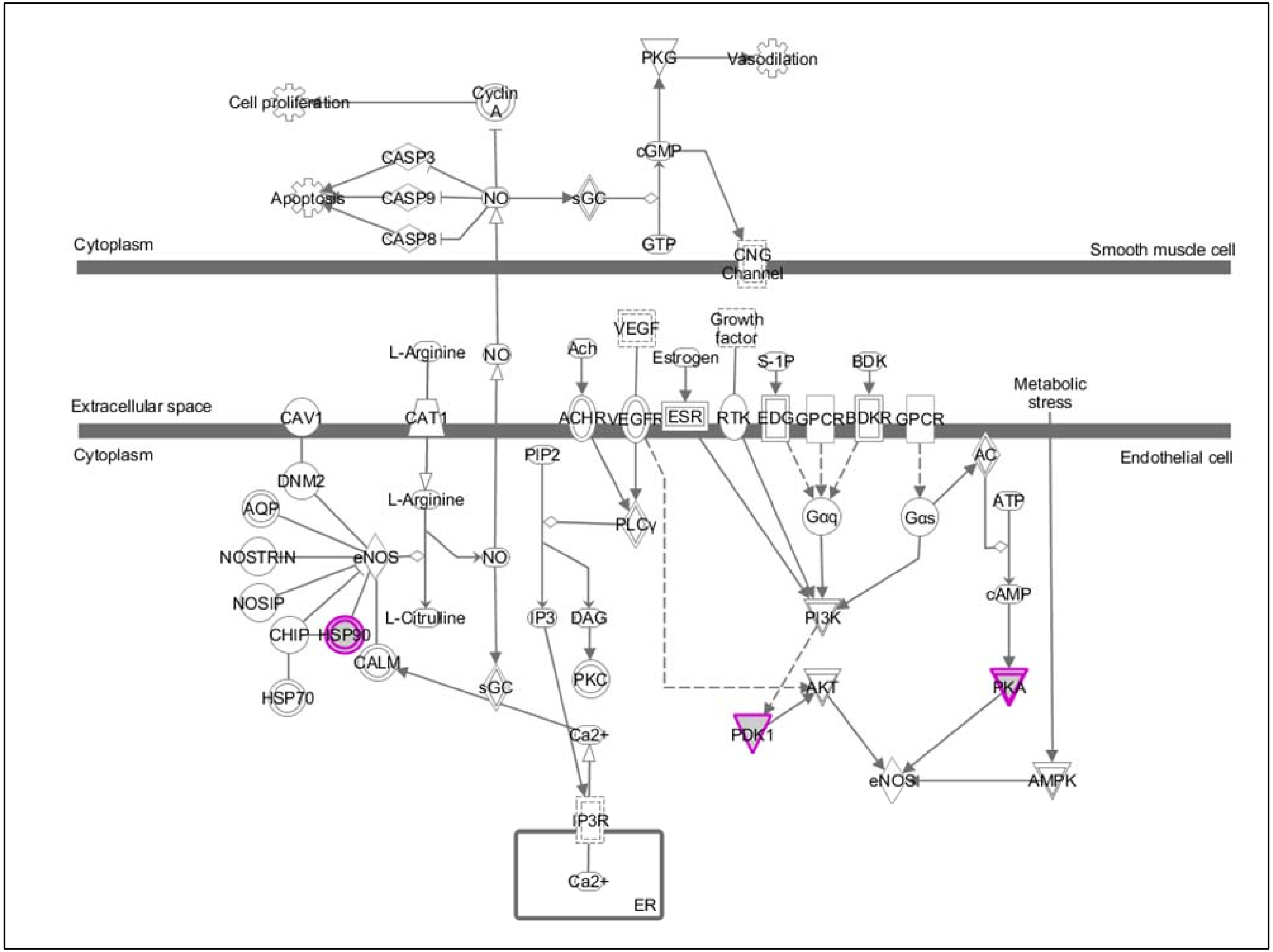

4-Aldosterone signaling in epithelial cells

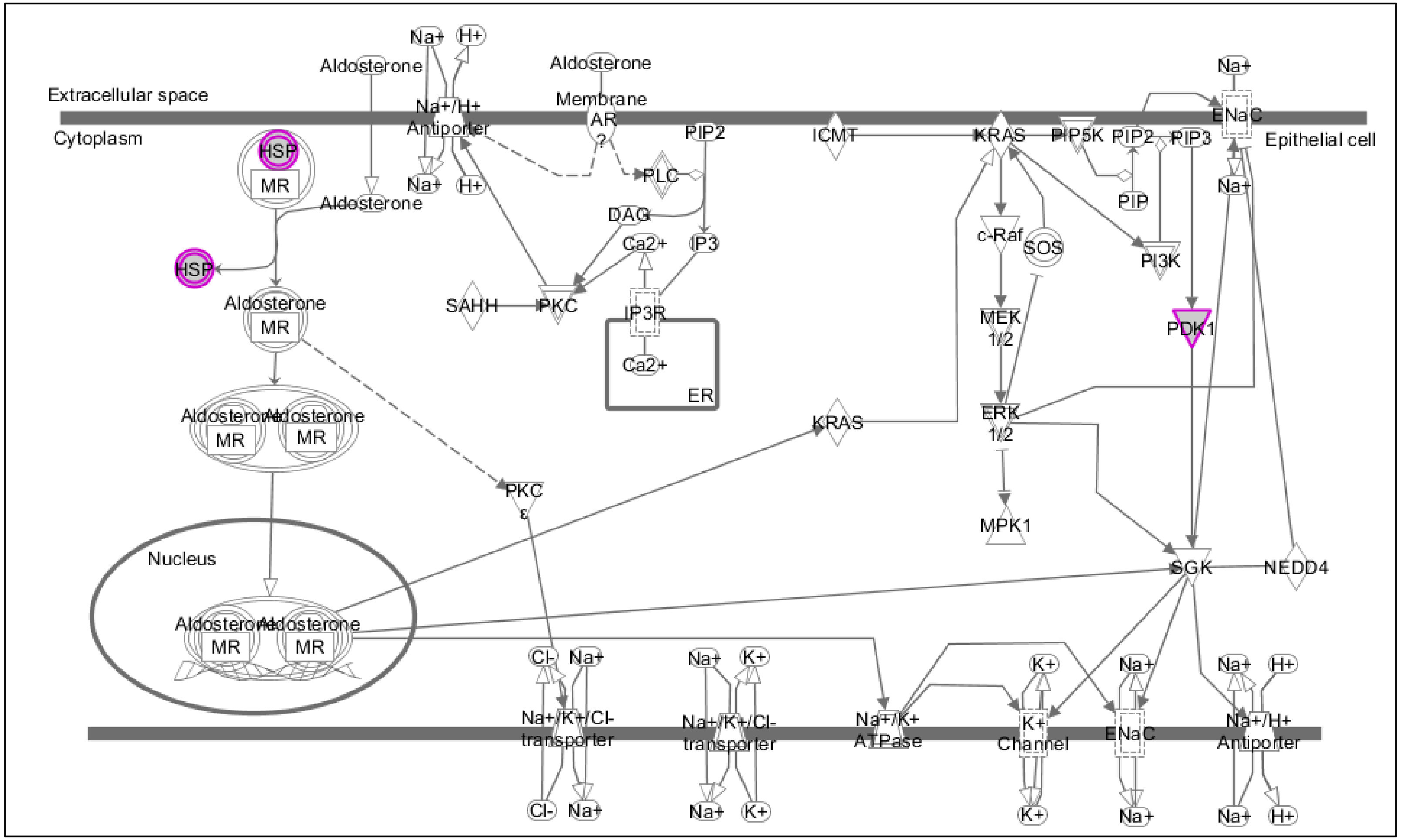

5-PPARa_RXRa activation

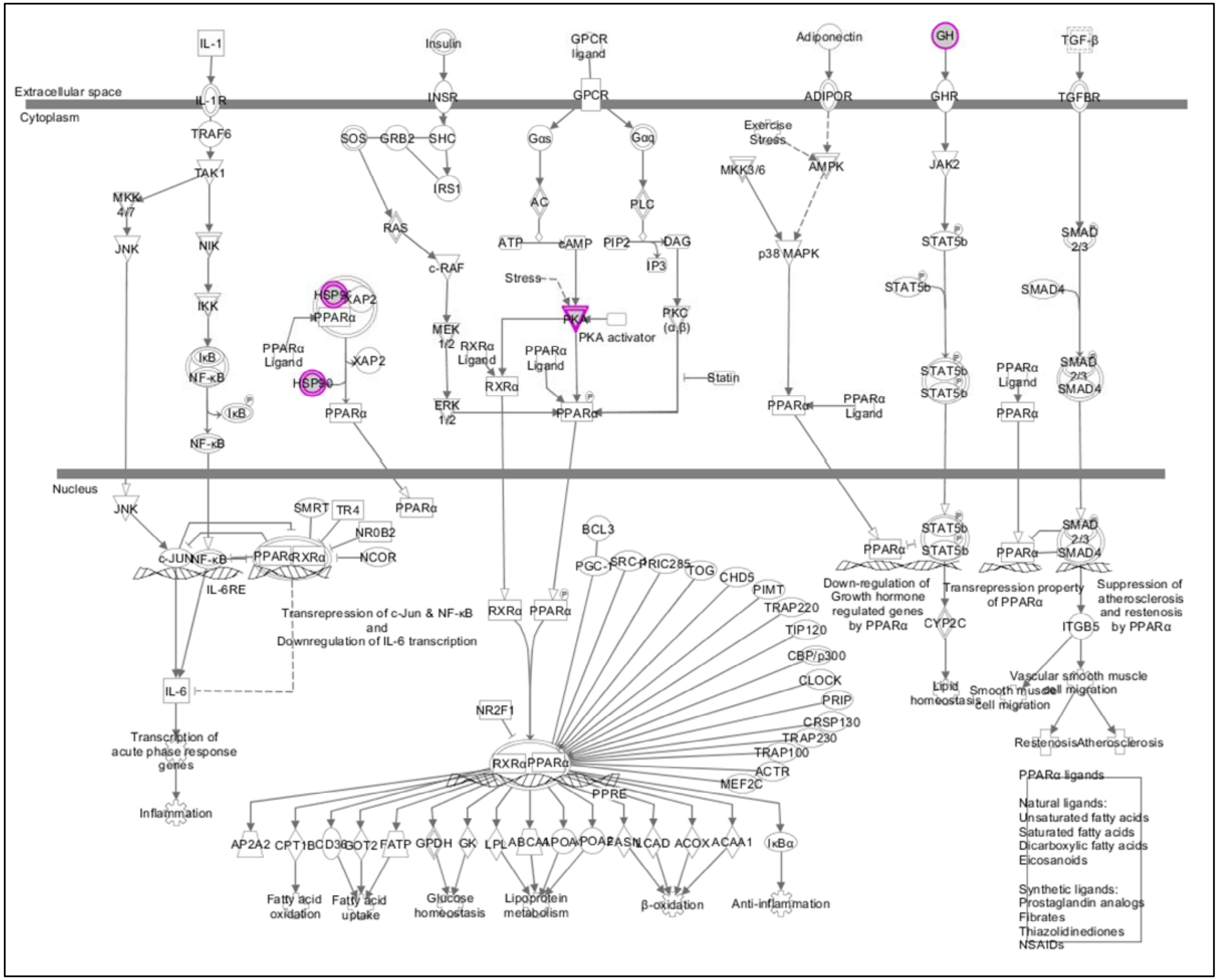

6-Endometrial cancer signaling

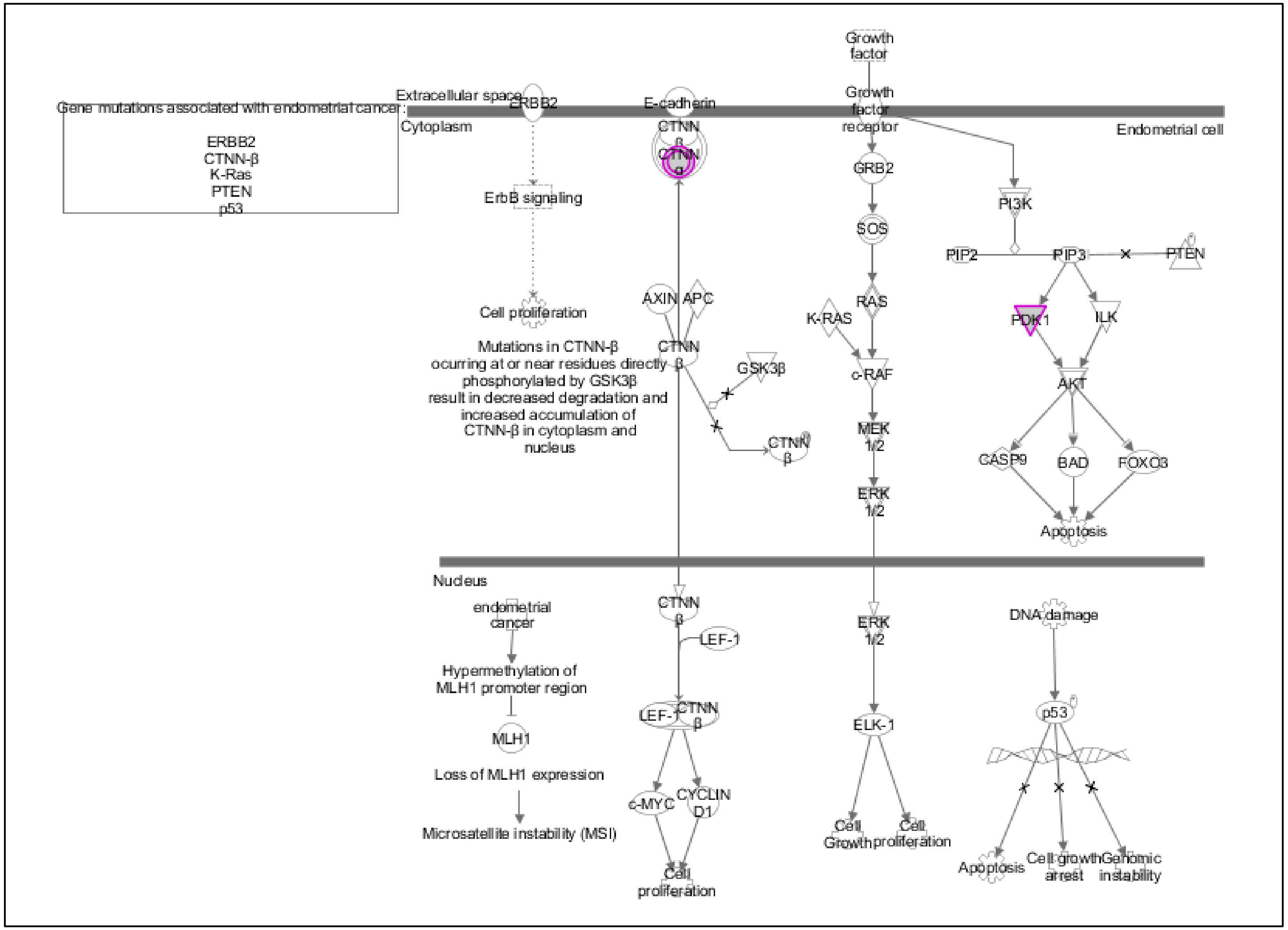

7-Growth hormone signaling

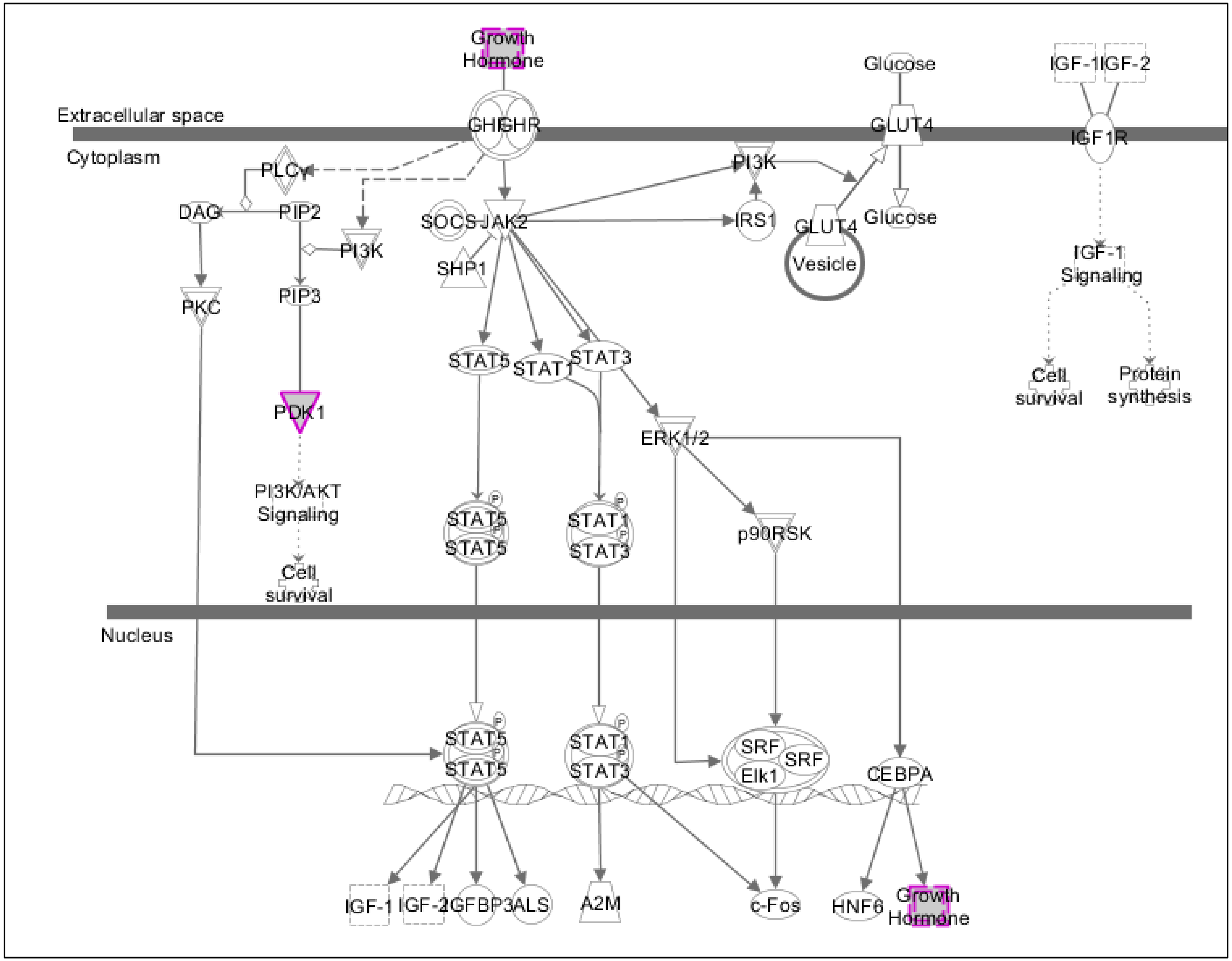

8-Leptin signaling in obestiy

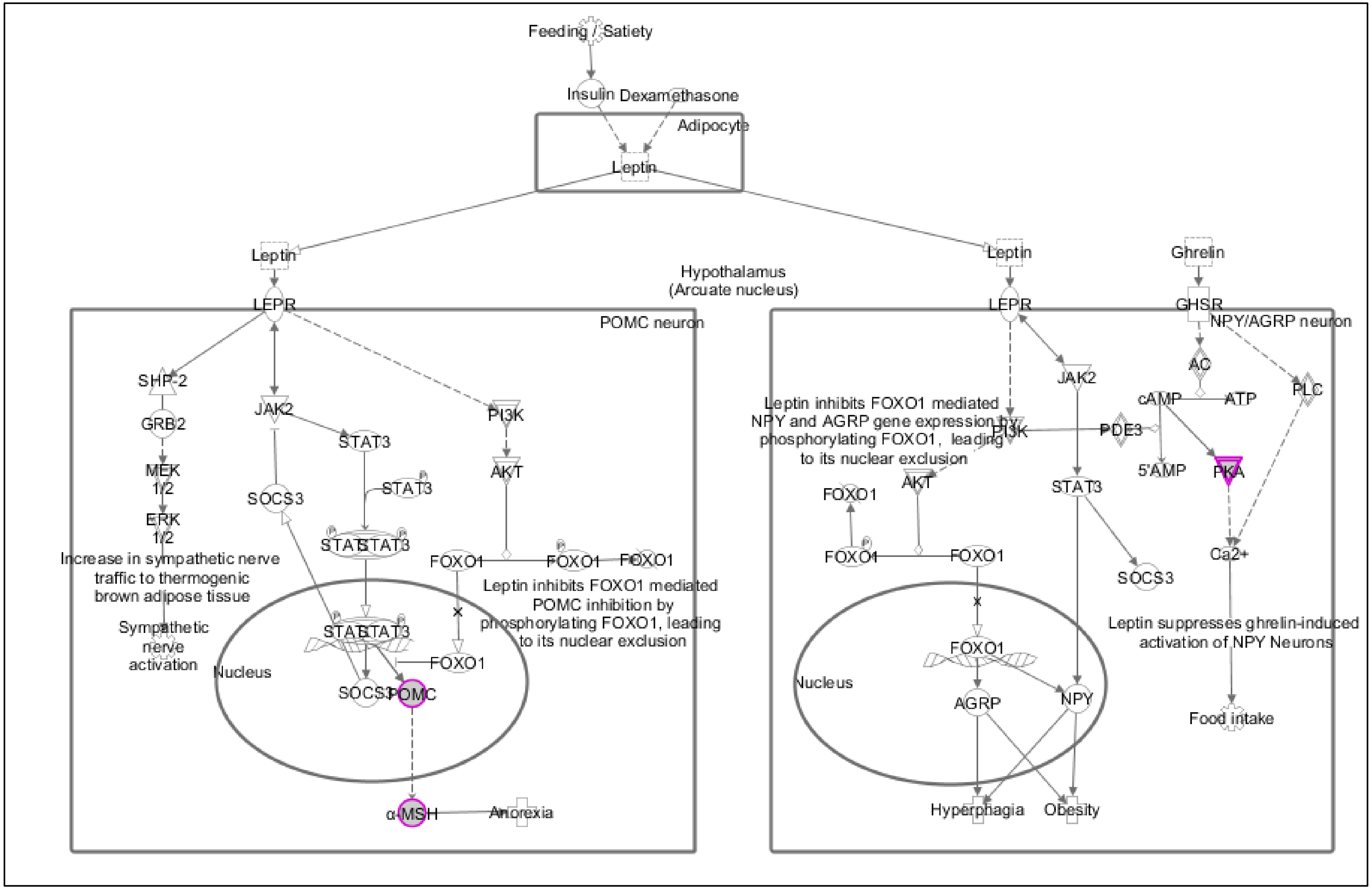

9-Glucocorticoid receptor signaling

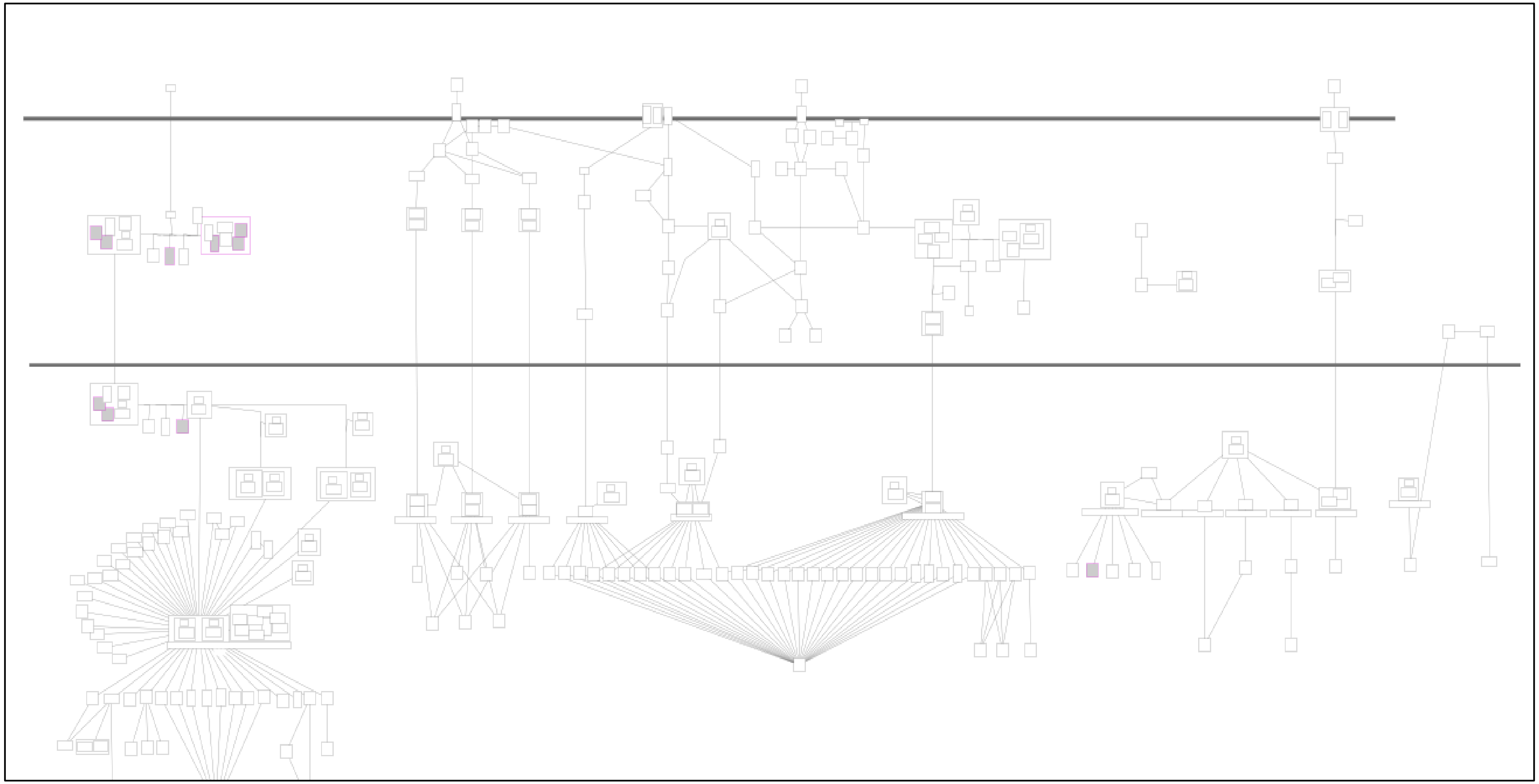

10-Melanocyte development and pigmentation signaling

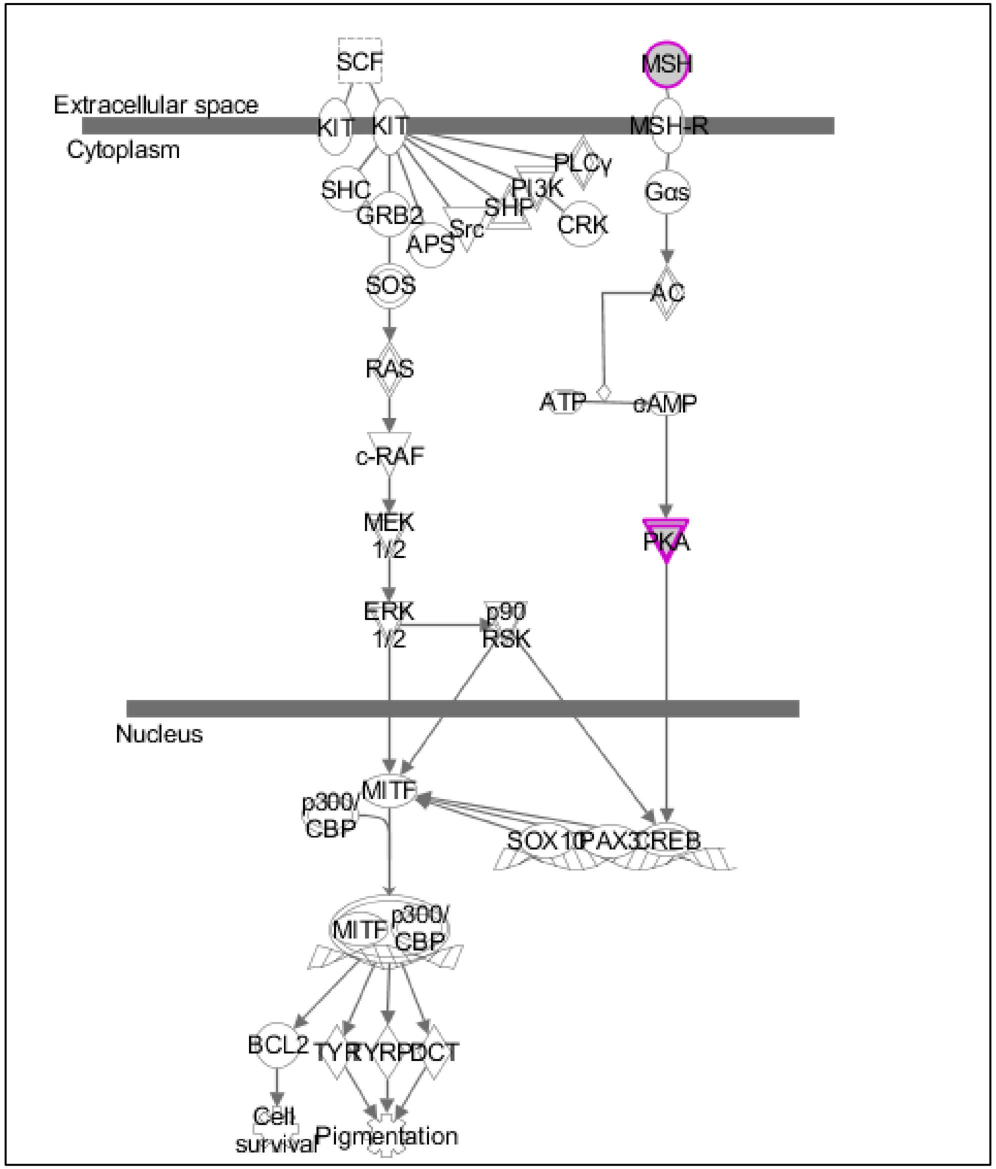

11-Prostate cancer signaling

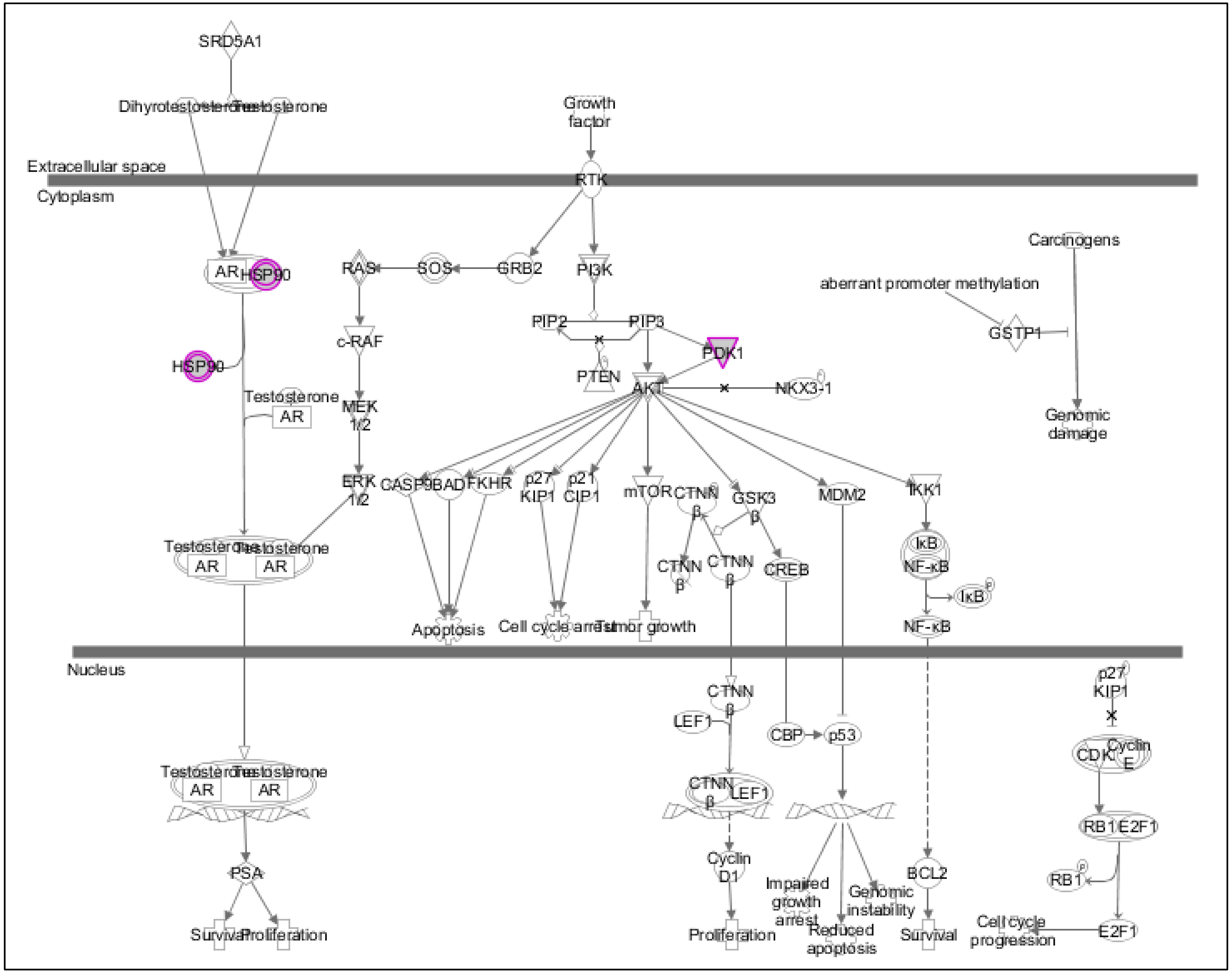

12-TR_RXR activation

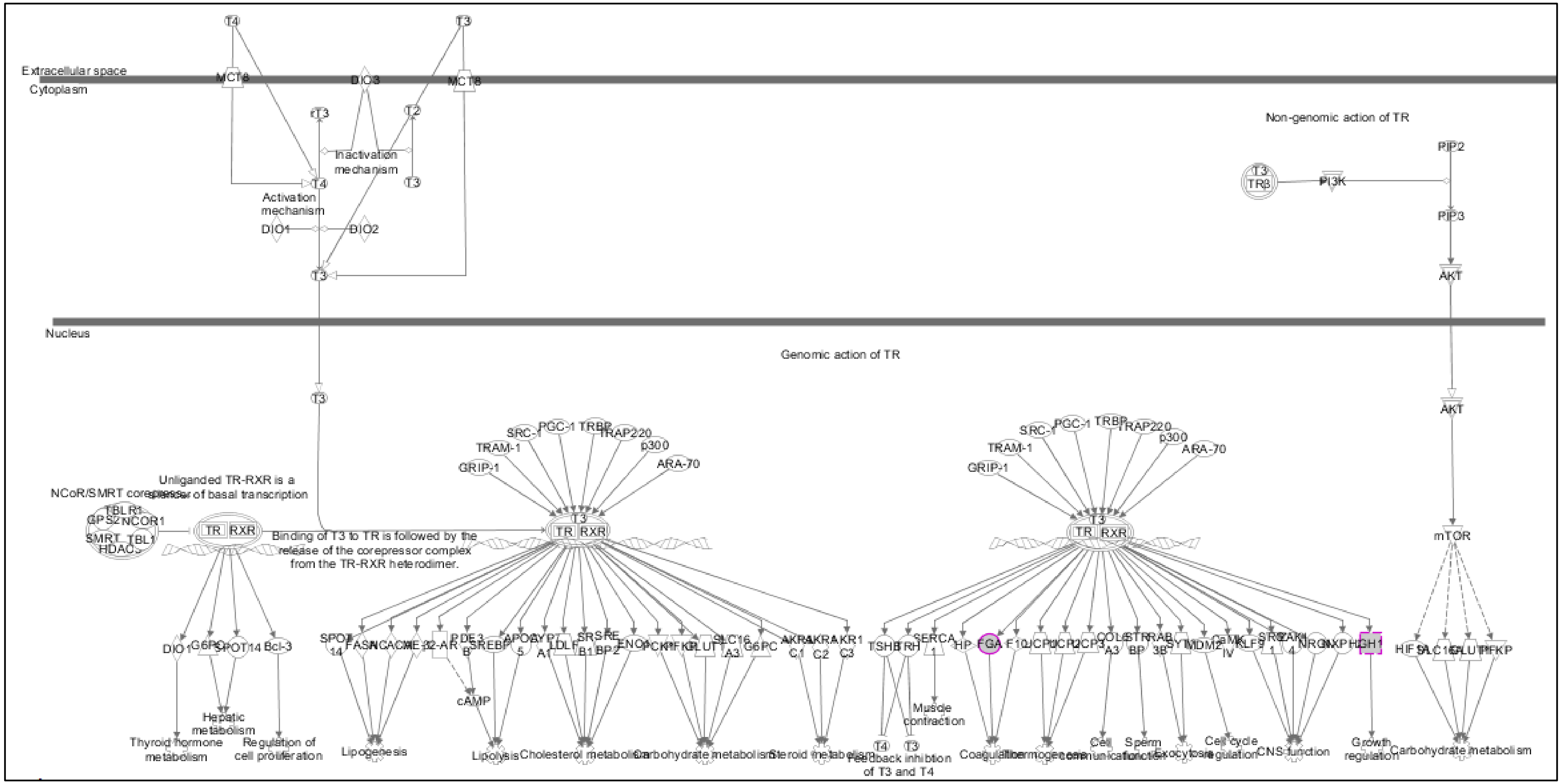

13-Neurigulin signaling

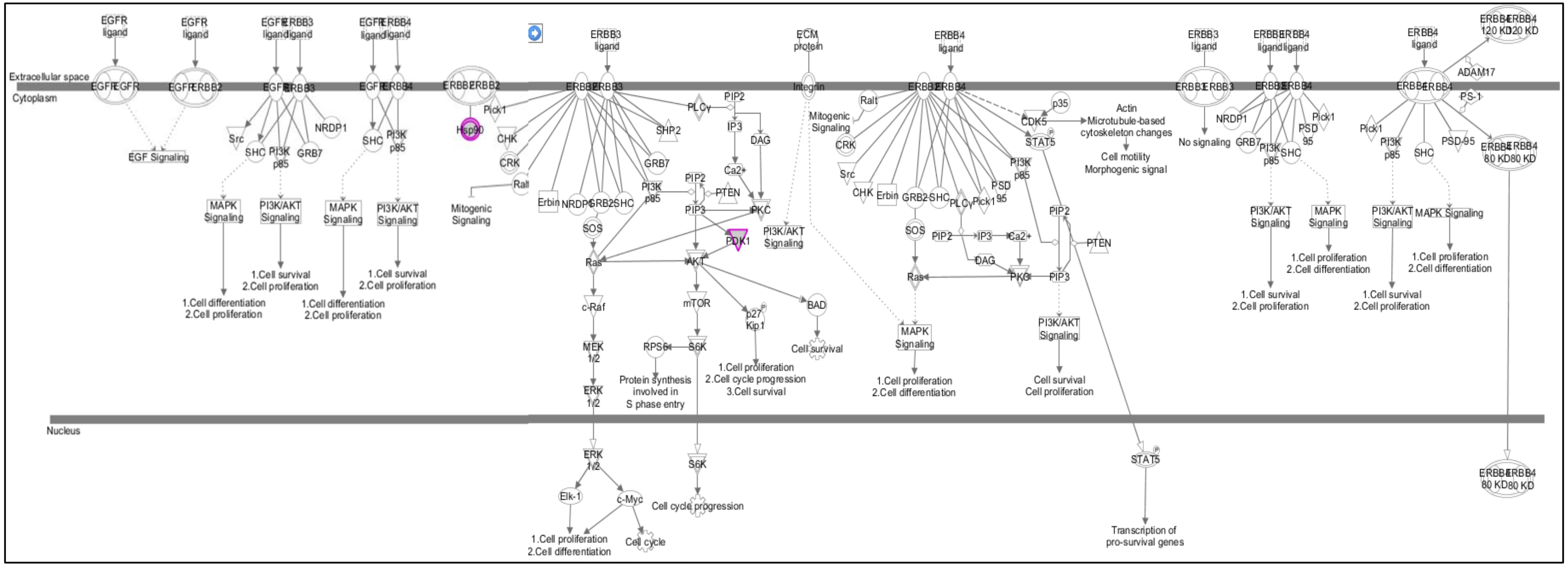

14-G beta gamma signaling

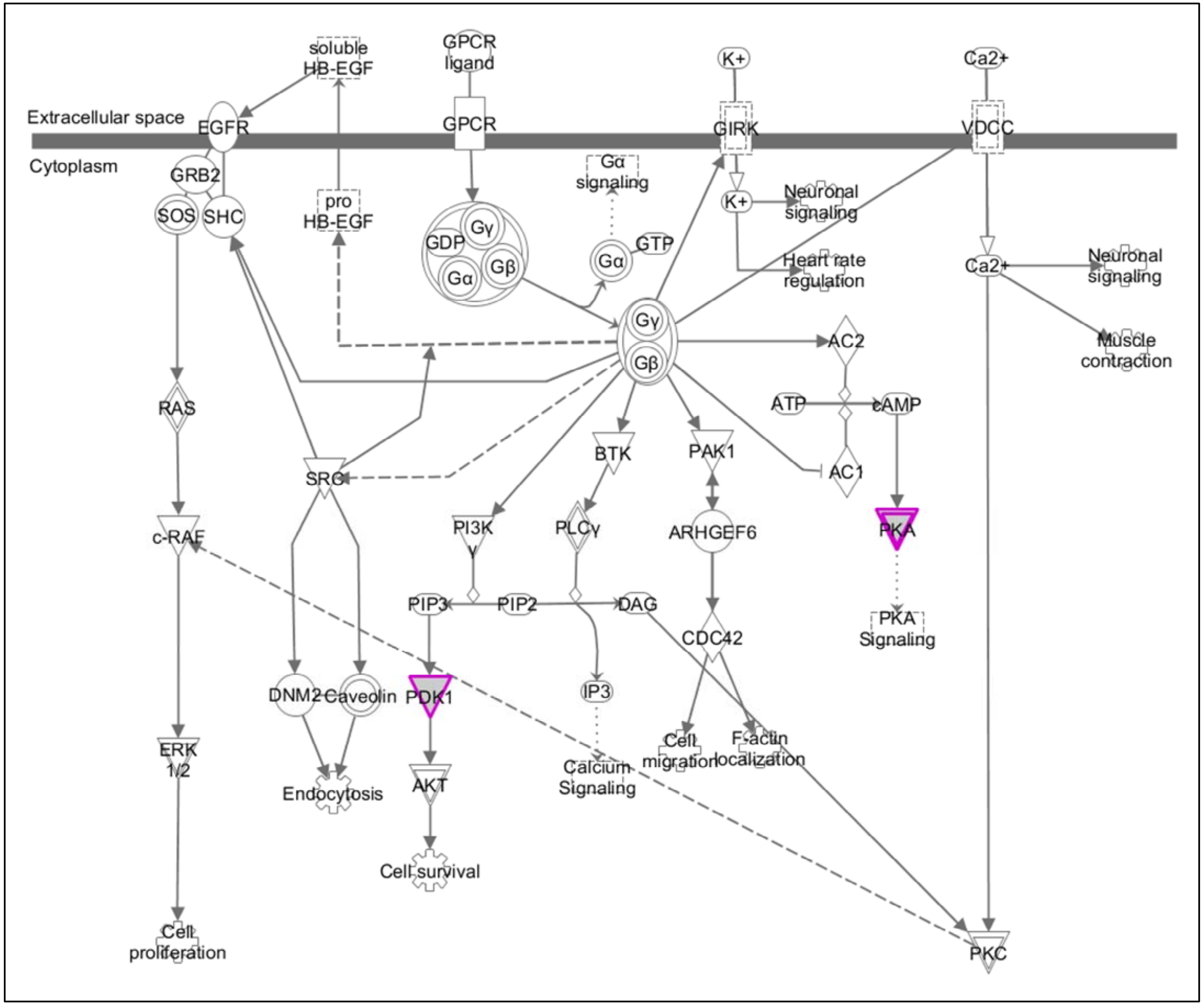

15-IGF-1 signaling

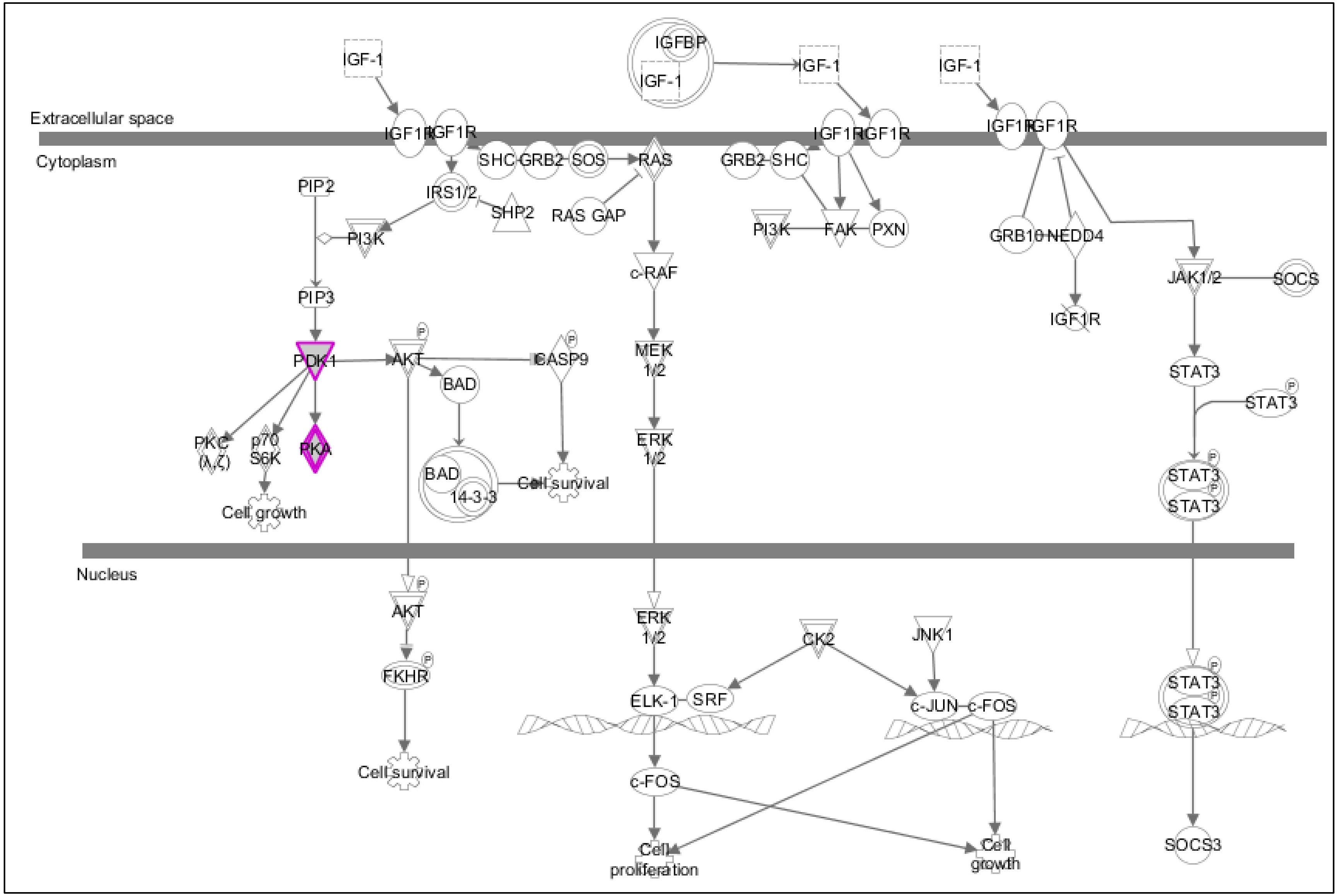

16-Nitric oxide signaling in cardiovascular system

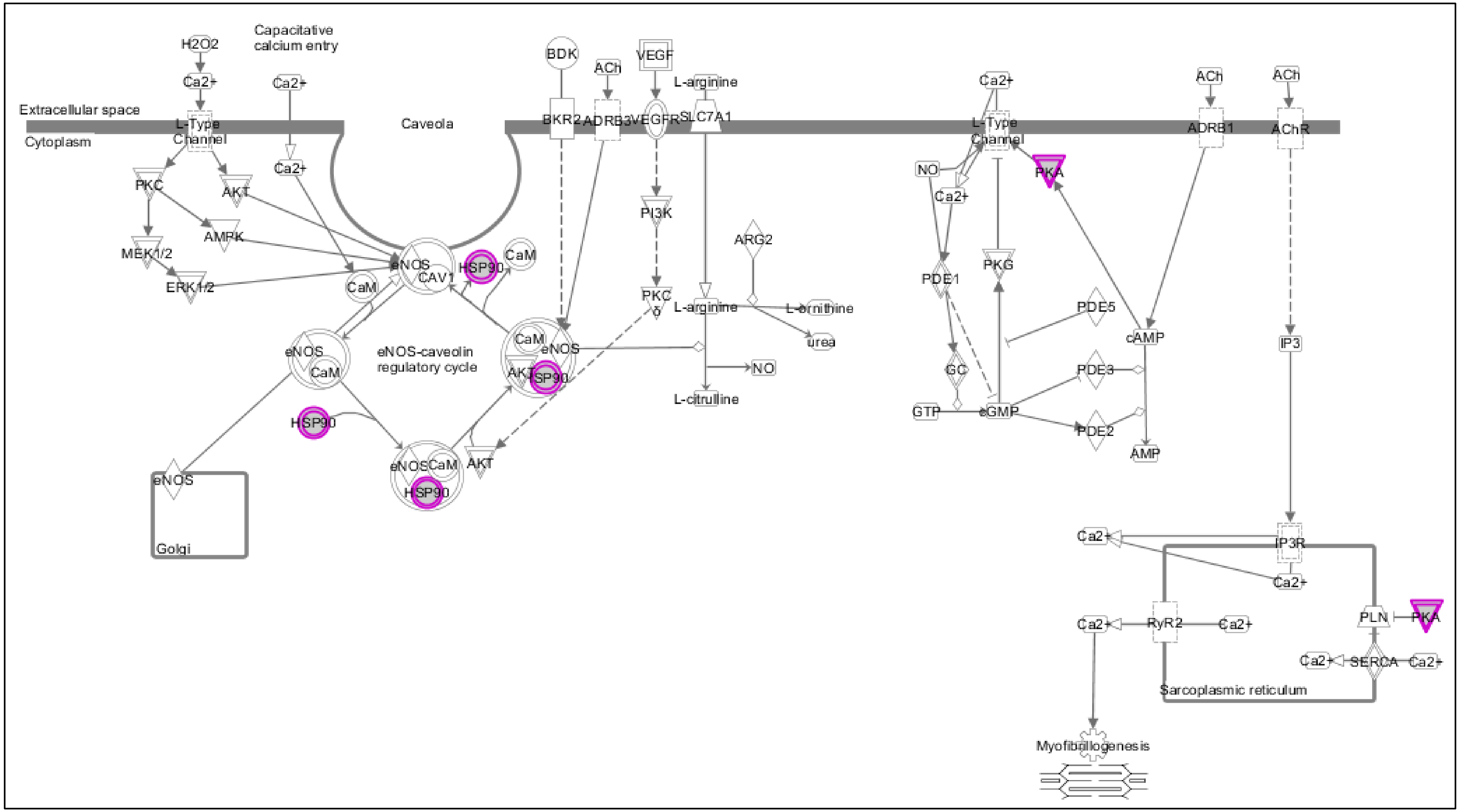

17-Androgen signaling

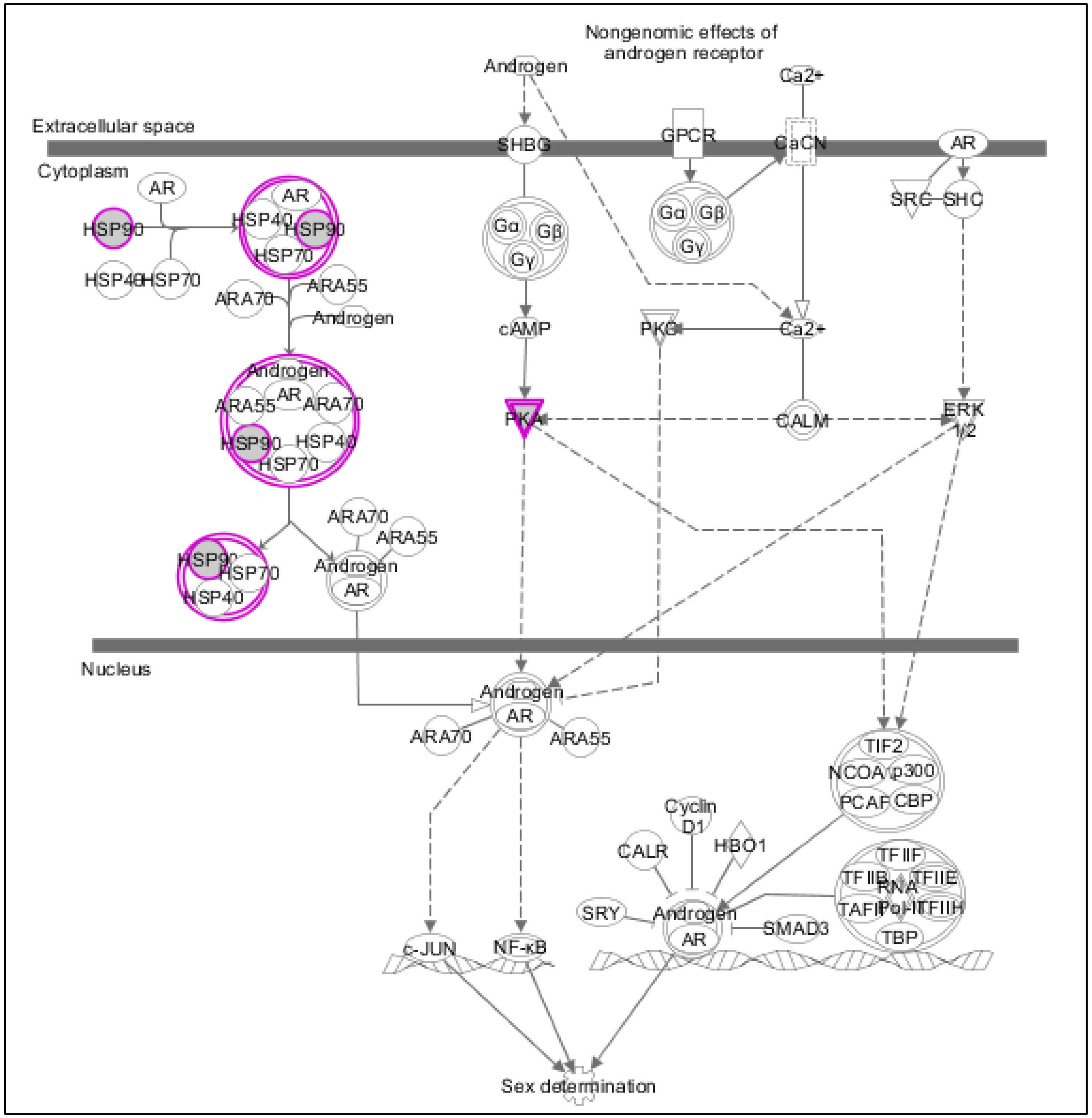

18-Corticotropin releasing hormone signaling

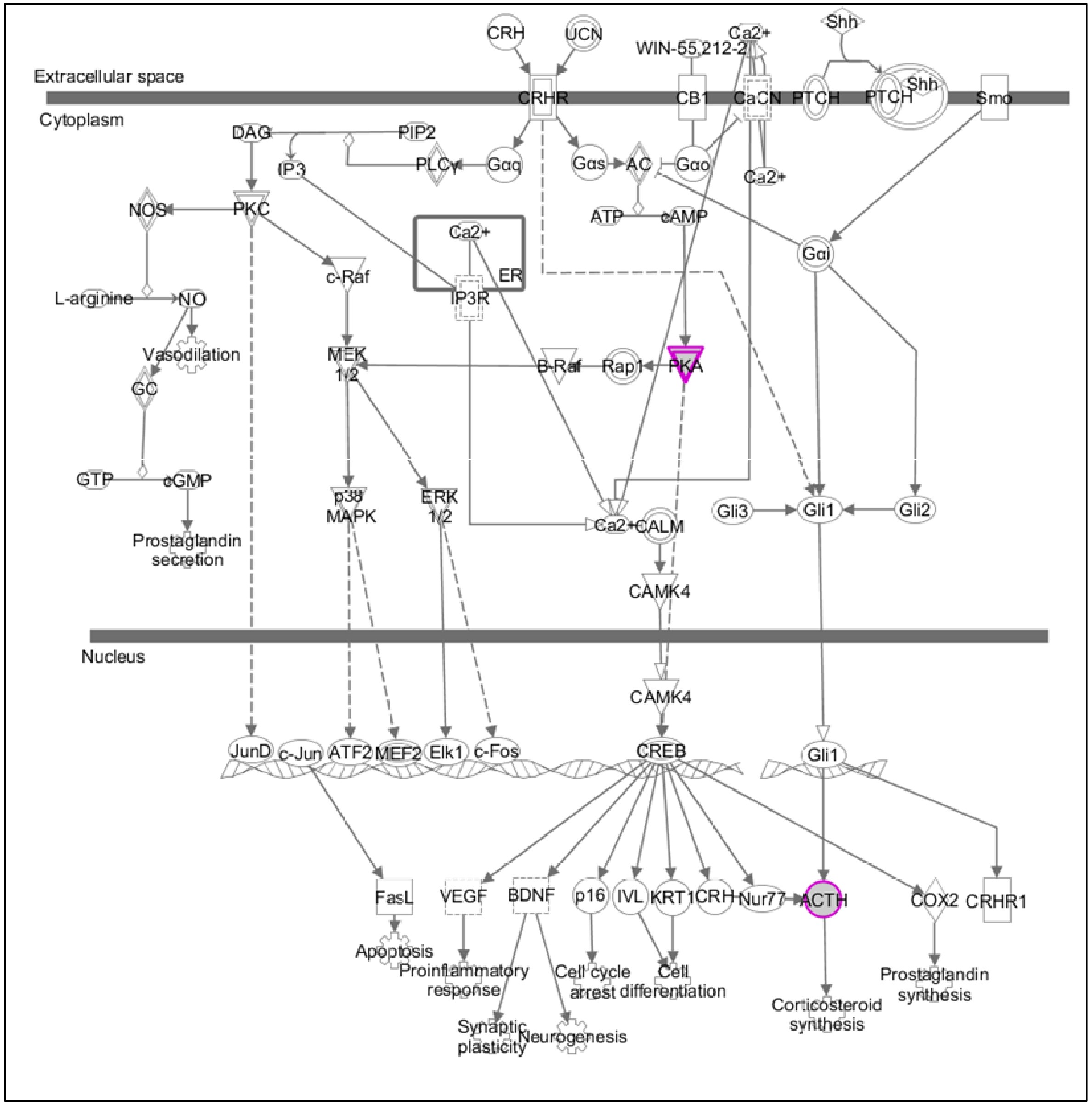

19-LXR_RXR activation

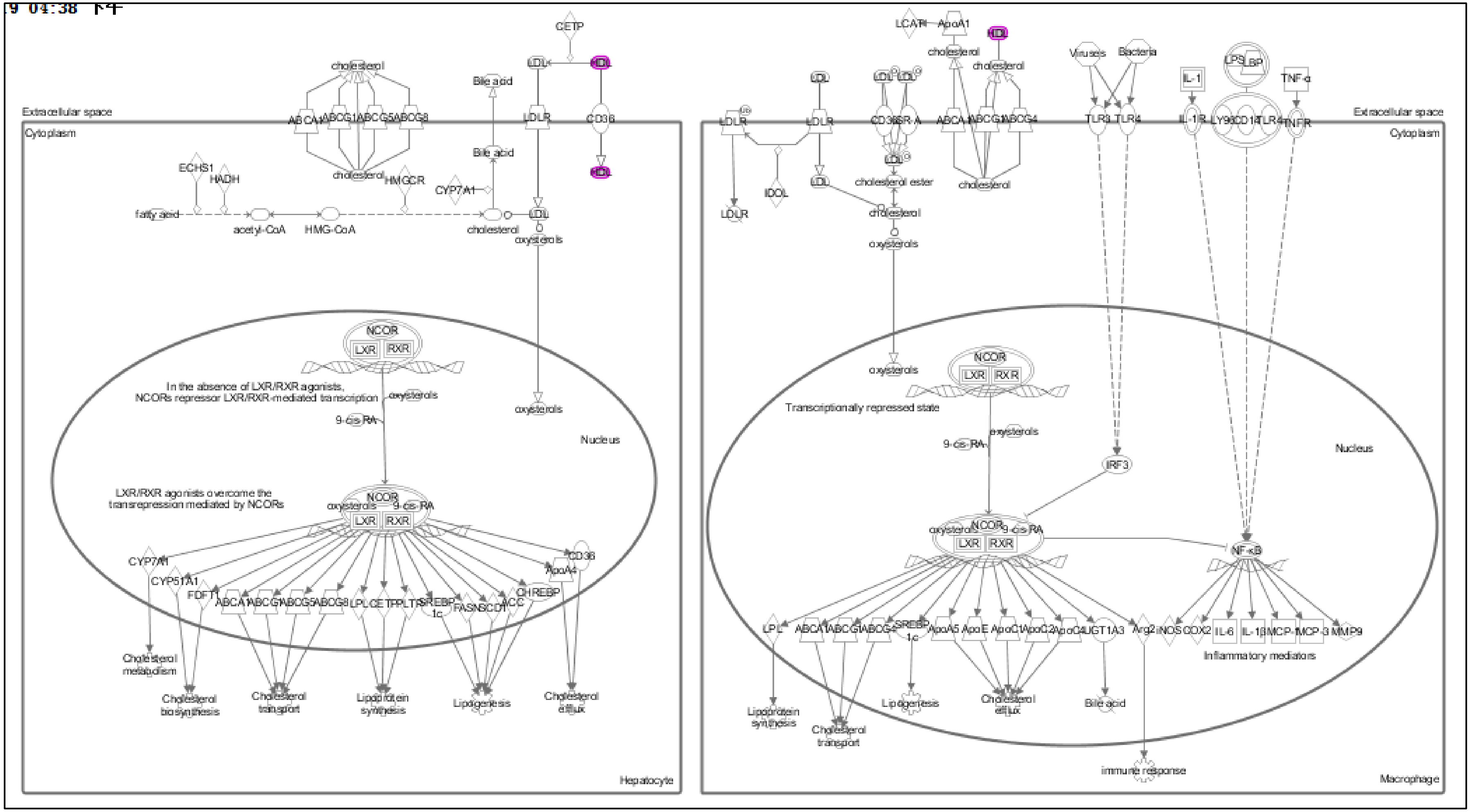

20-PI3K_AKT signaling

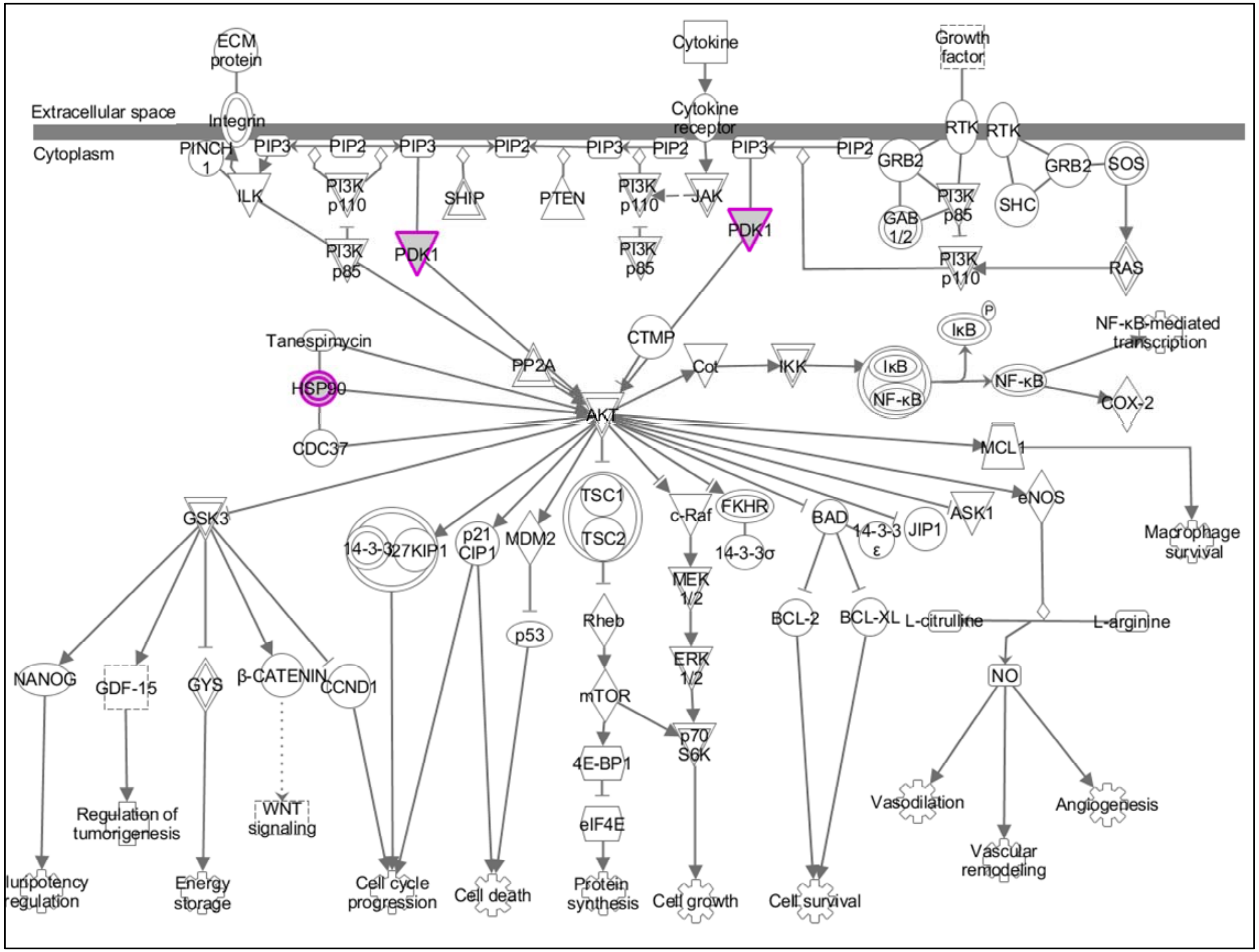

21-FXR_RXR activation

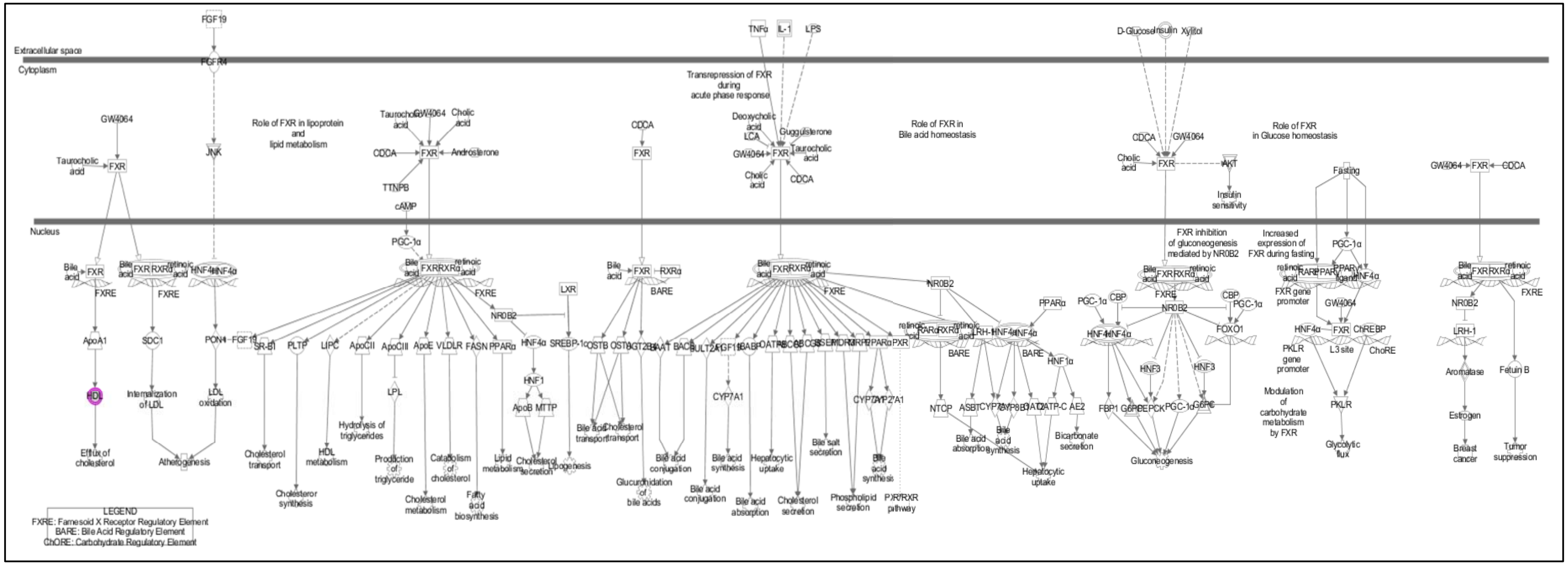

22-Insulin receptor signaling

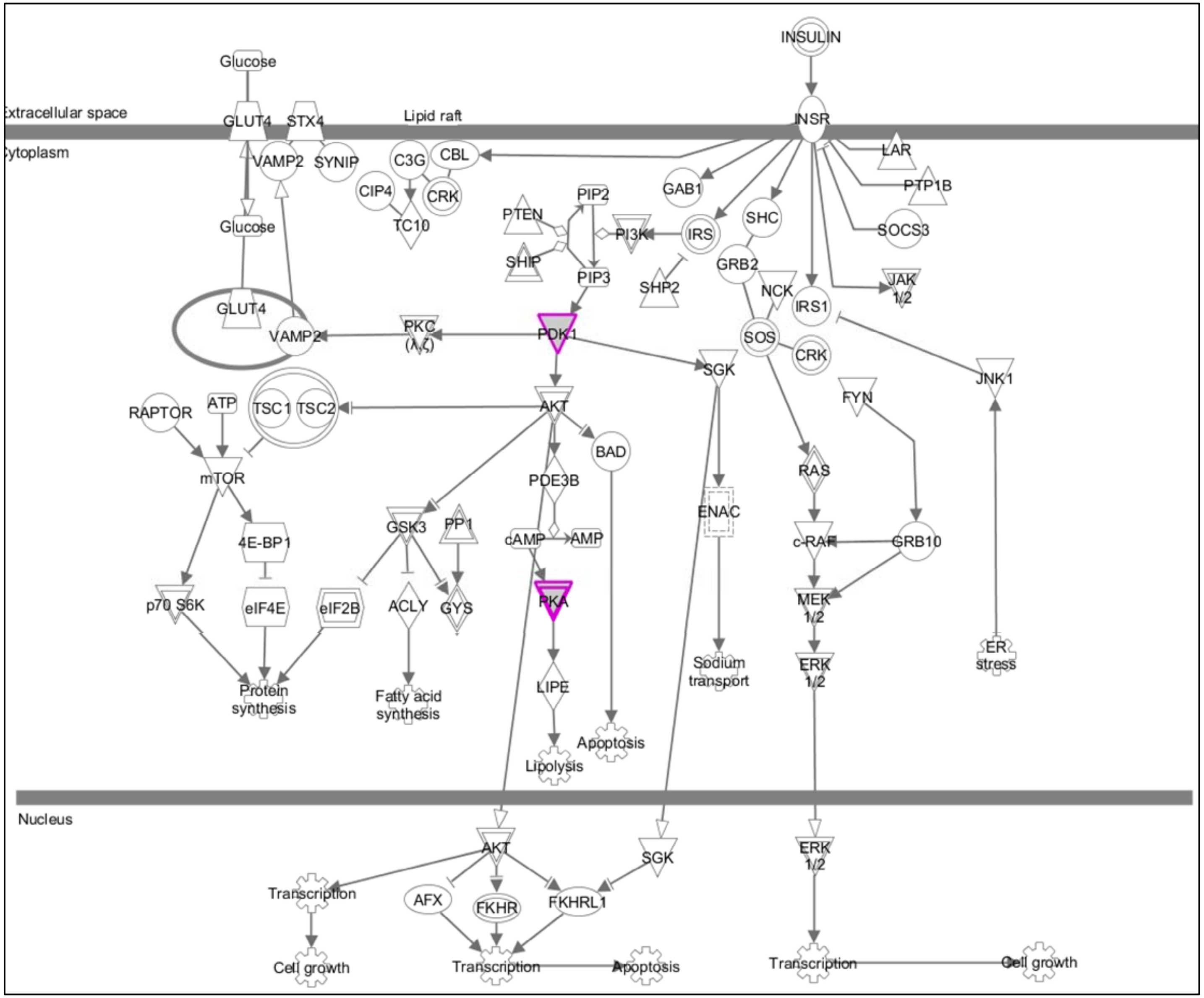

23-Aryl hydrocarbon receptor signaling

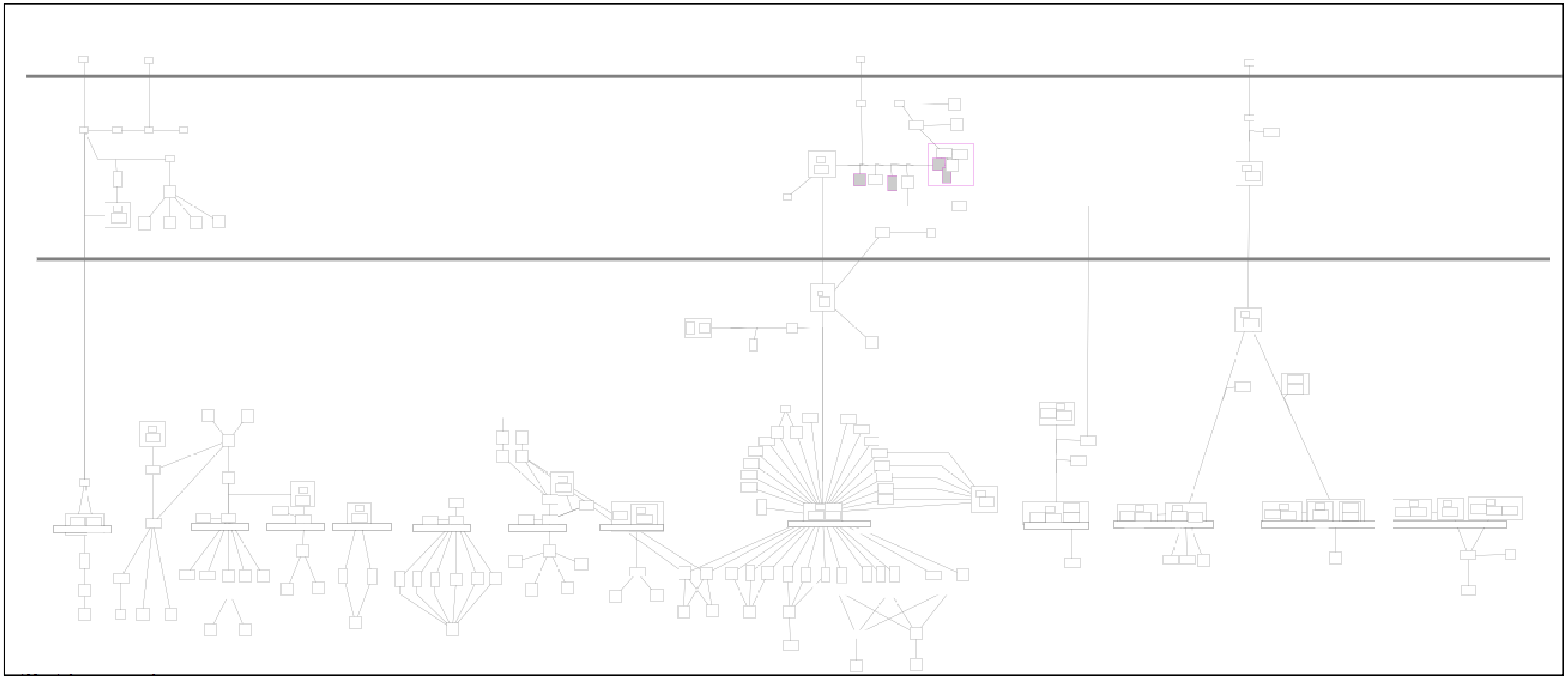

24-AMPK signaling

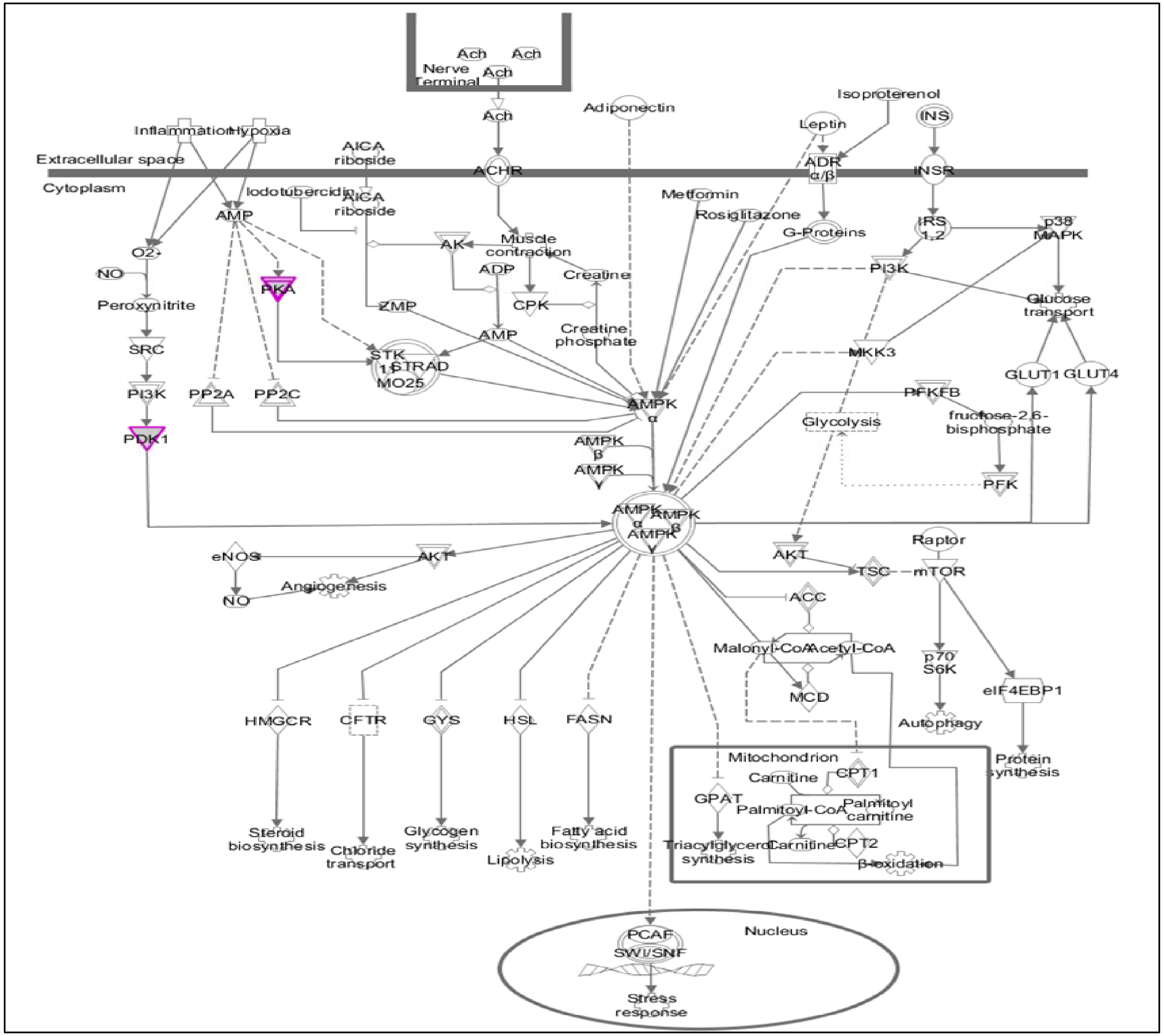

25-Regulation of eIF4 and p70S6K signaling

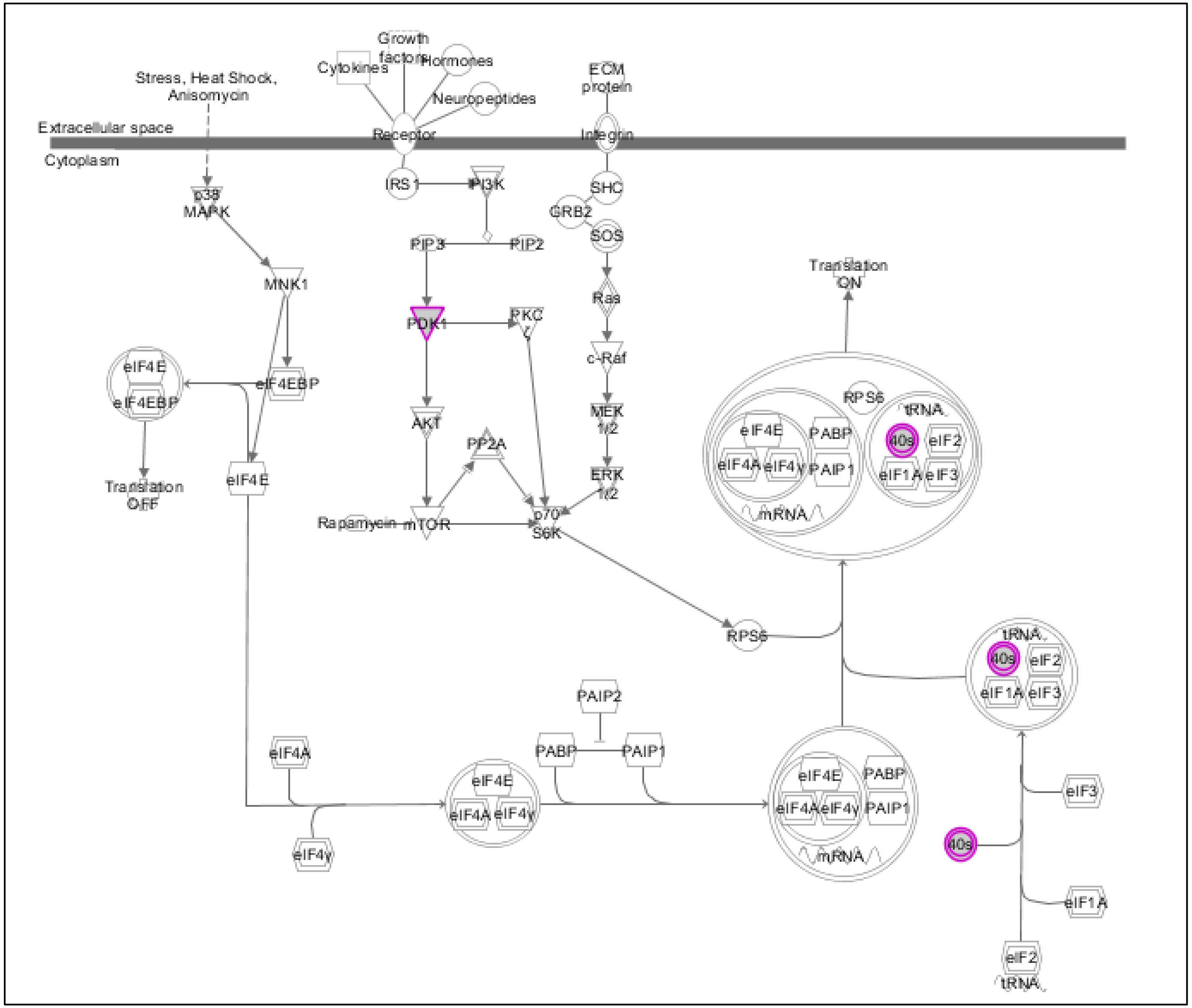

26-Germ cell-sertoli cell junction signaling

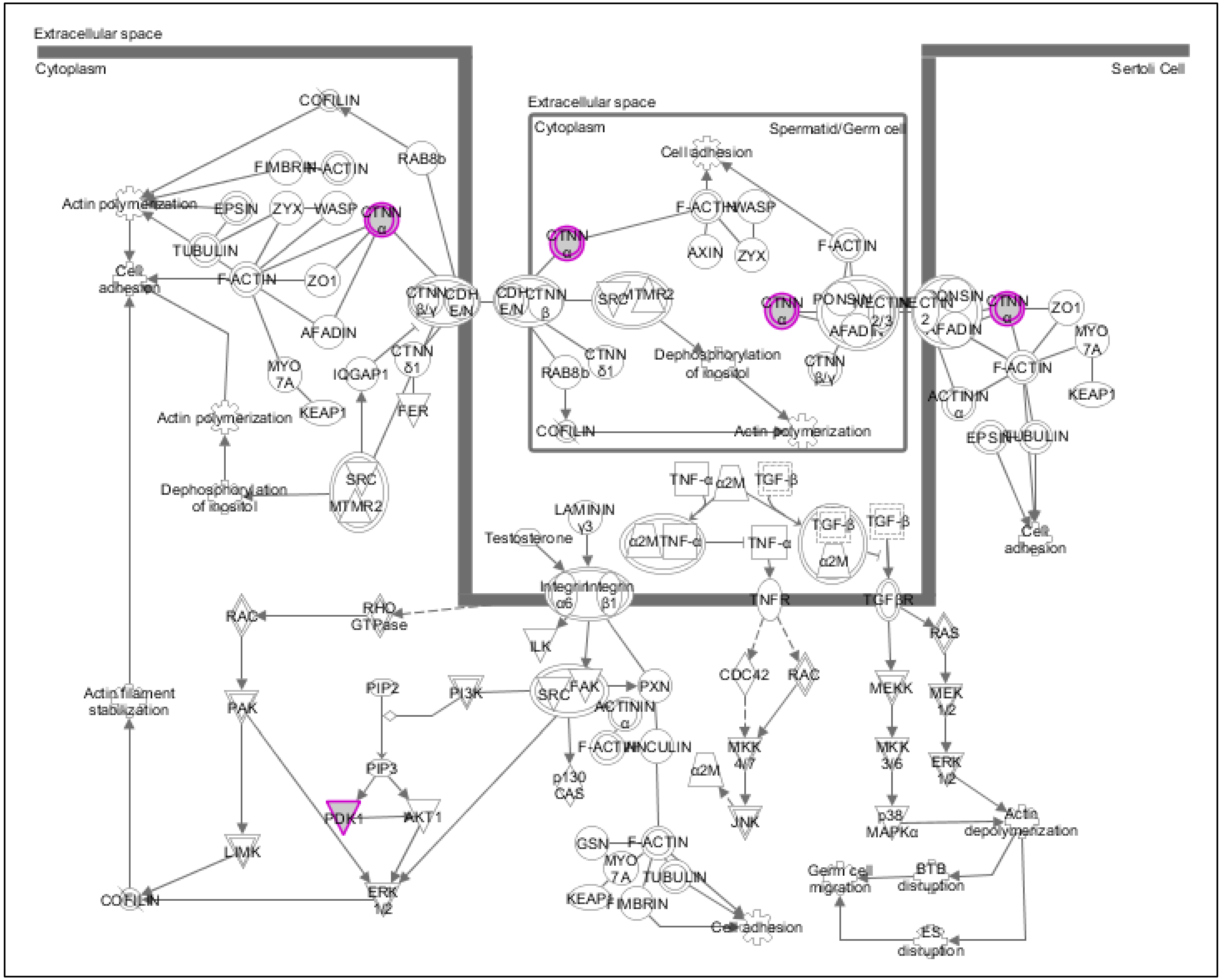

27-Prostanoid biosynthesis

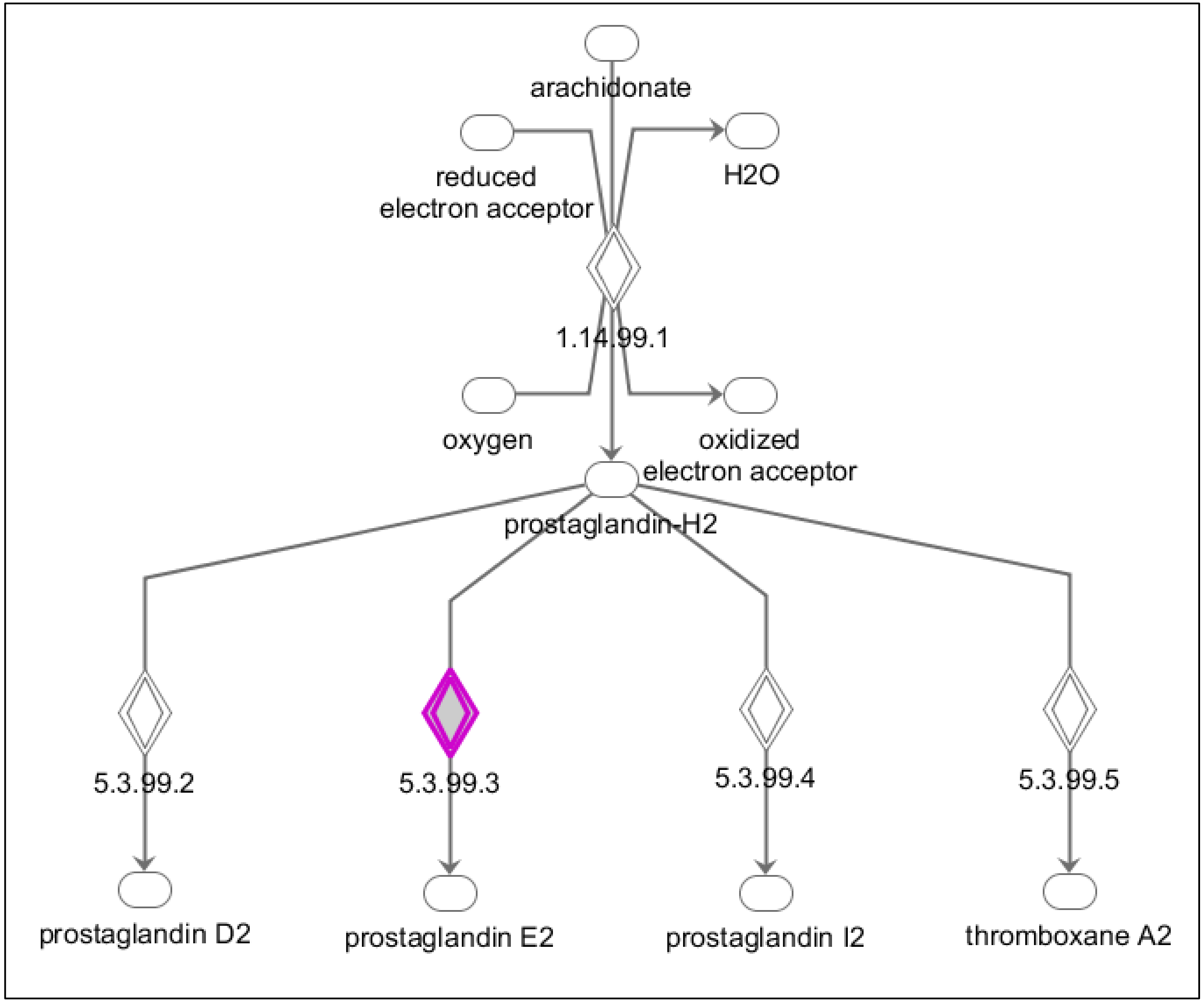

28-Tight junction signaling

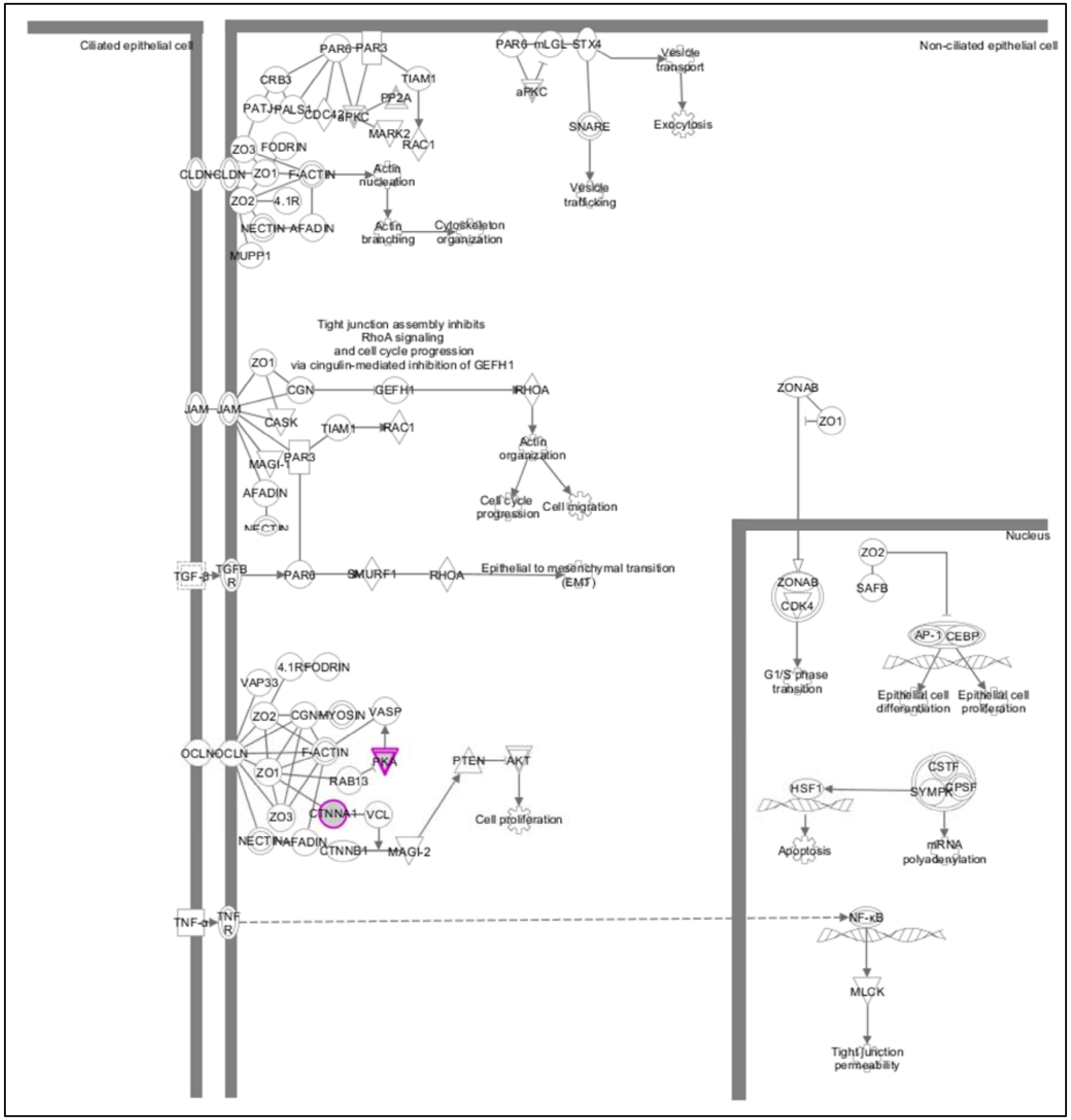

29-Acute phase response signaling

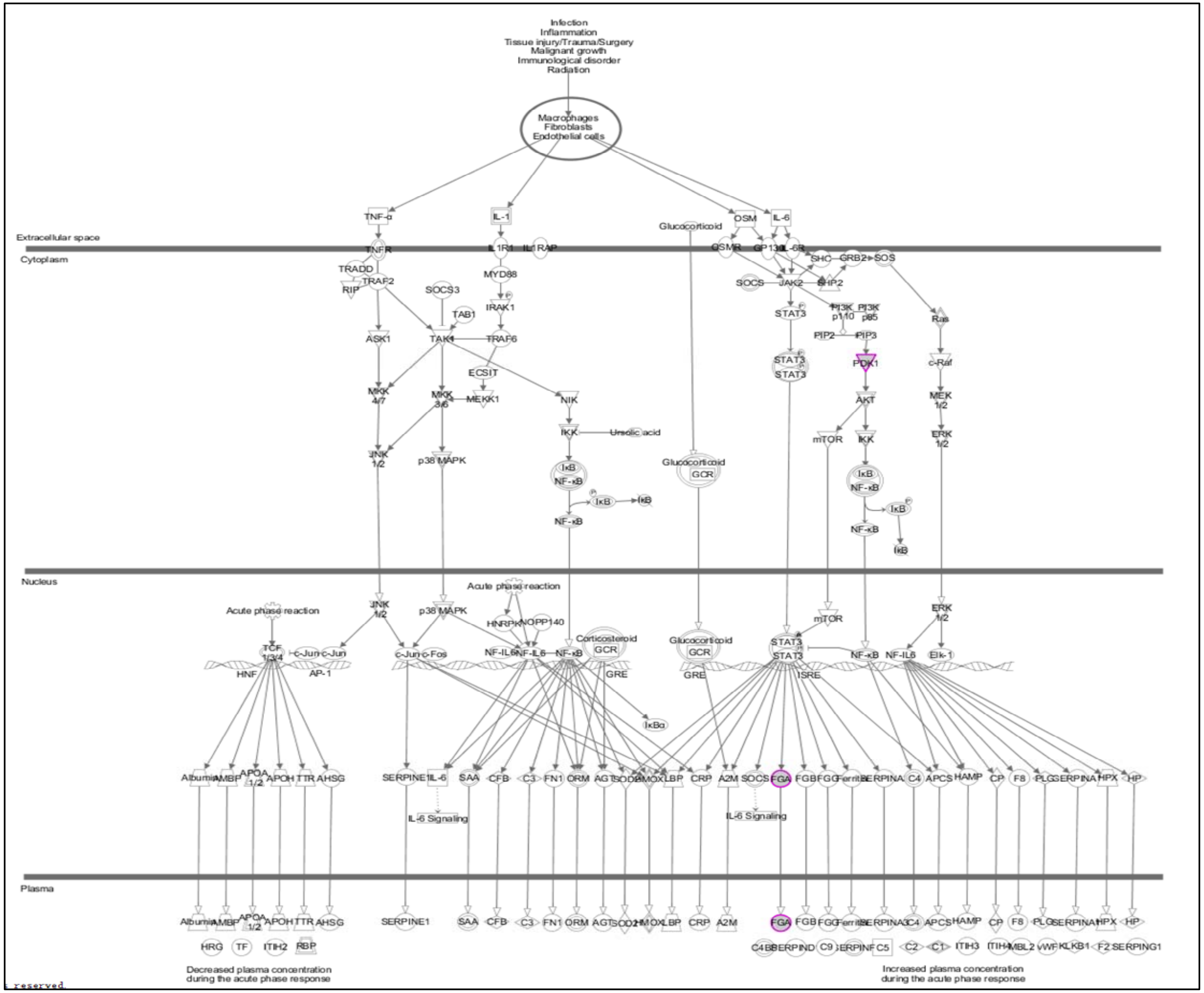

30-RAR activation

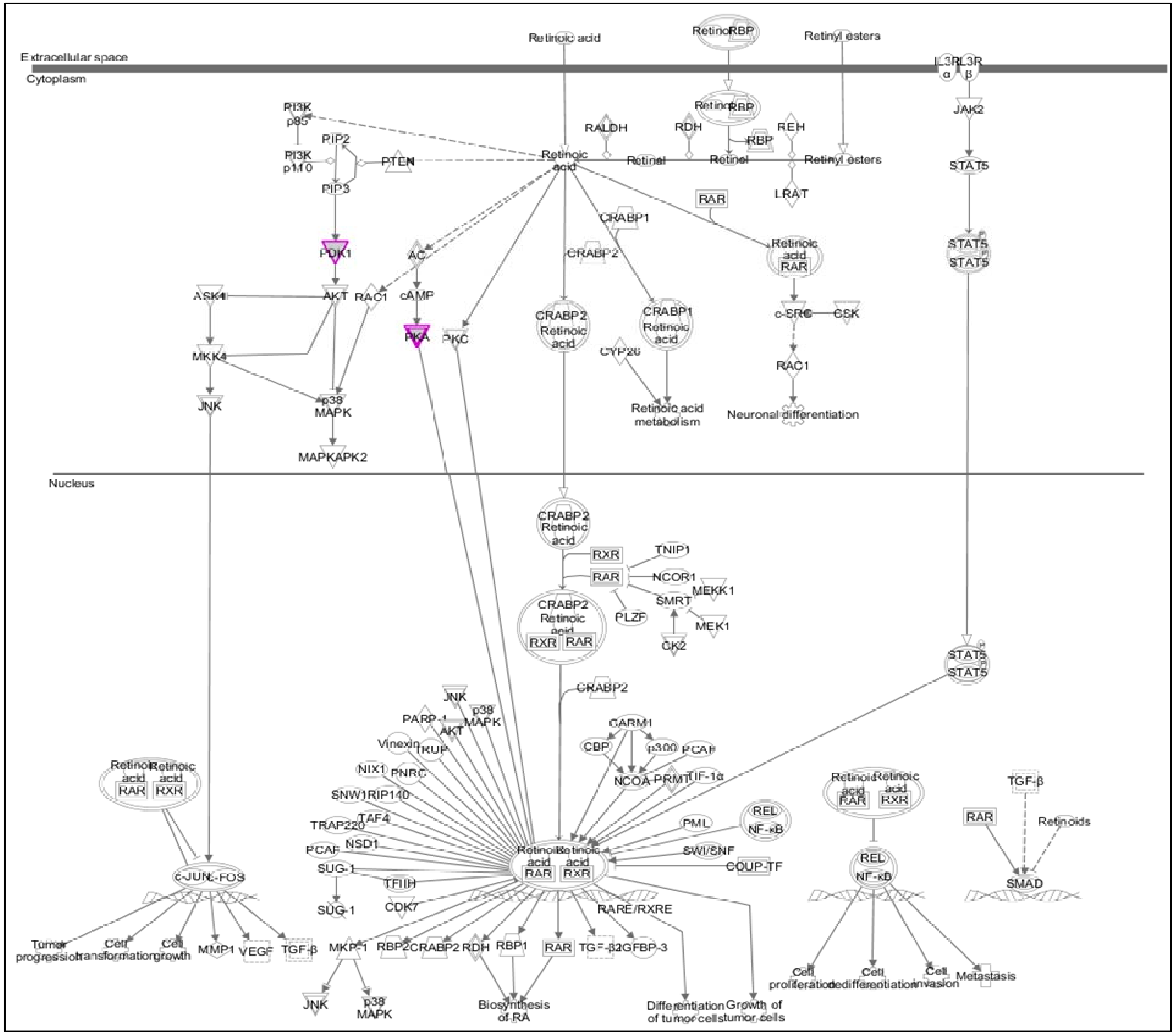

31-Extrinsic prothrombin activation pathway

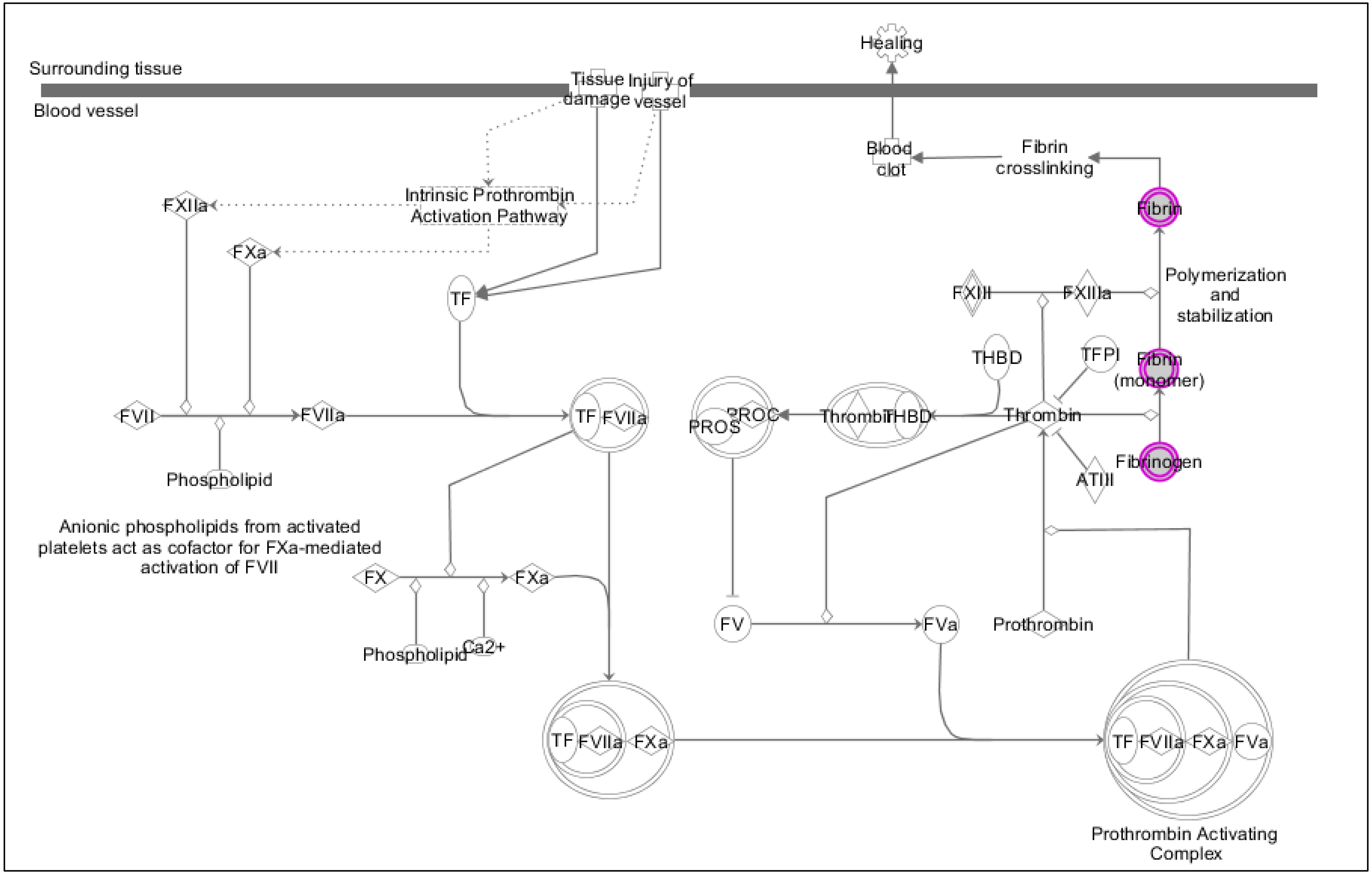

32-Sertoli cell-sertoli cell junction signaling

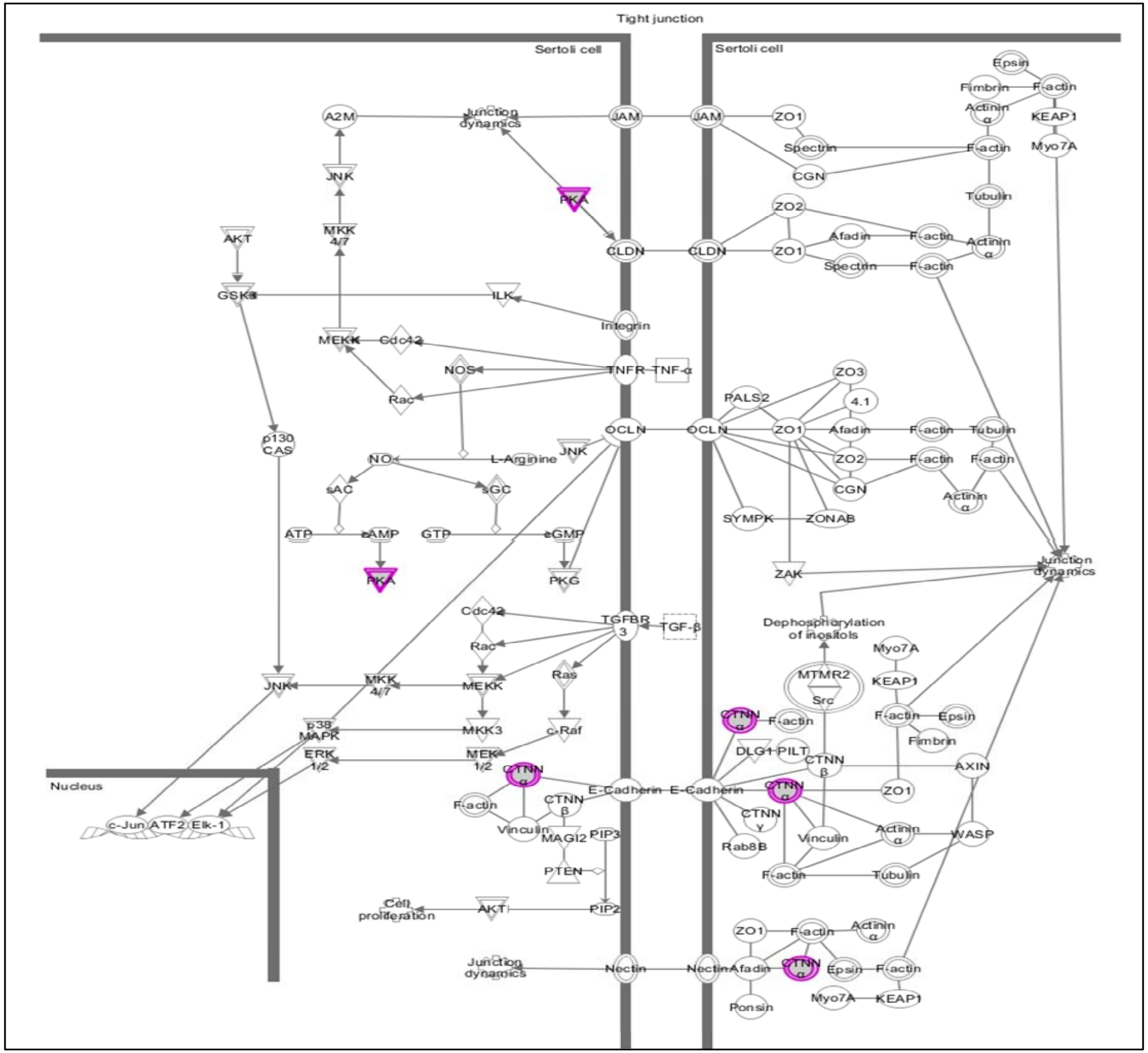

33-sucrose degradation V (mammalian)

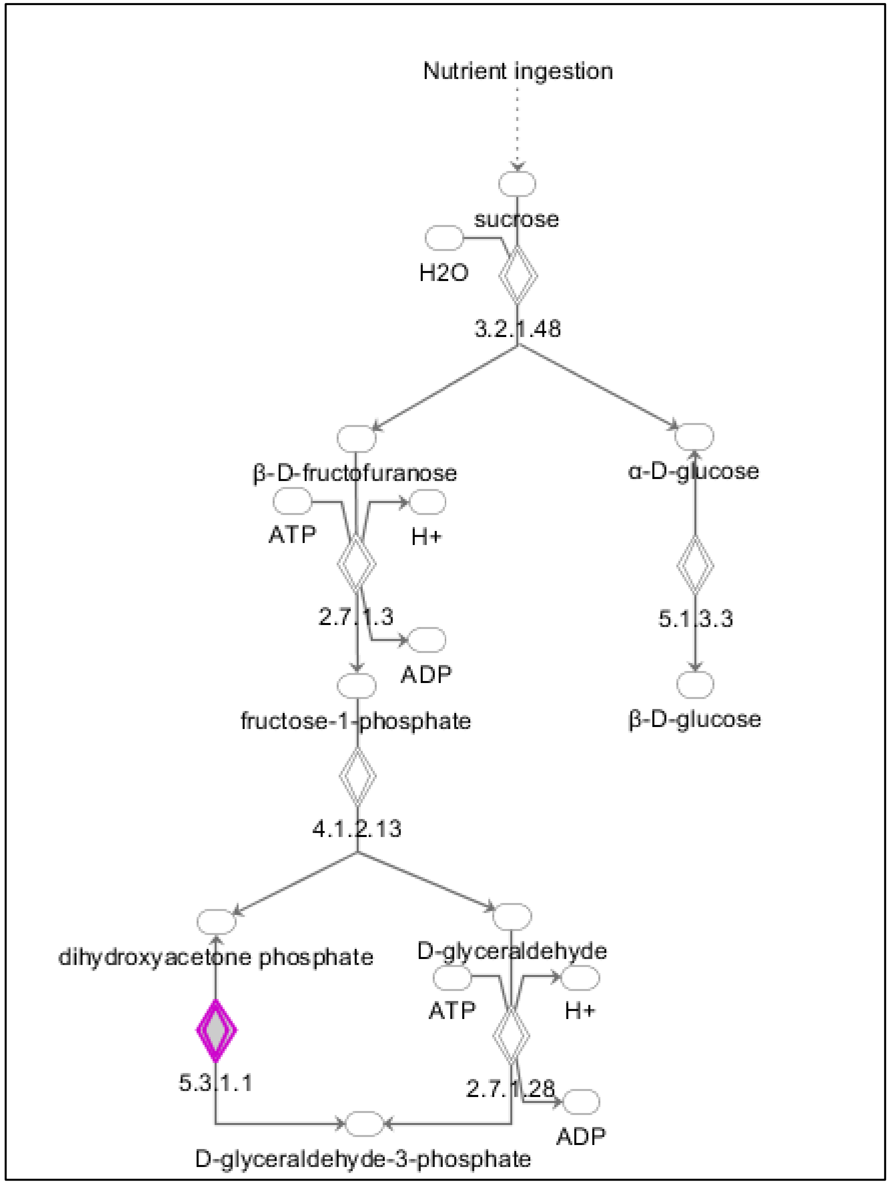

34-mTOR signaling

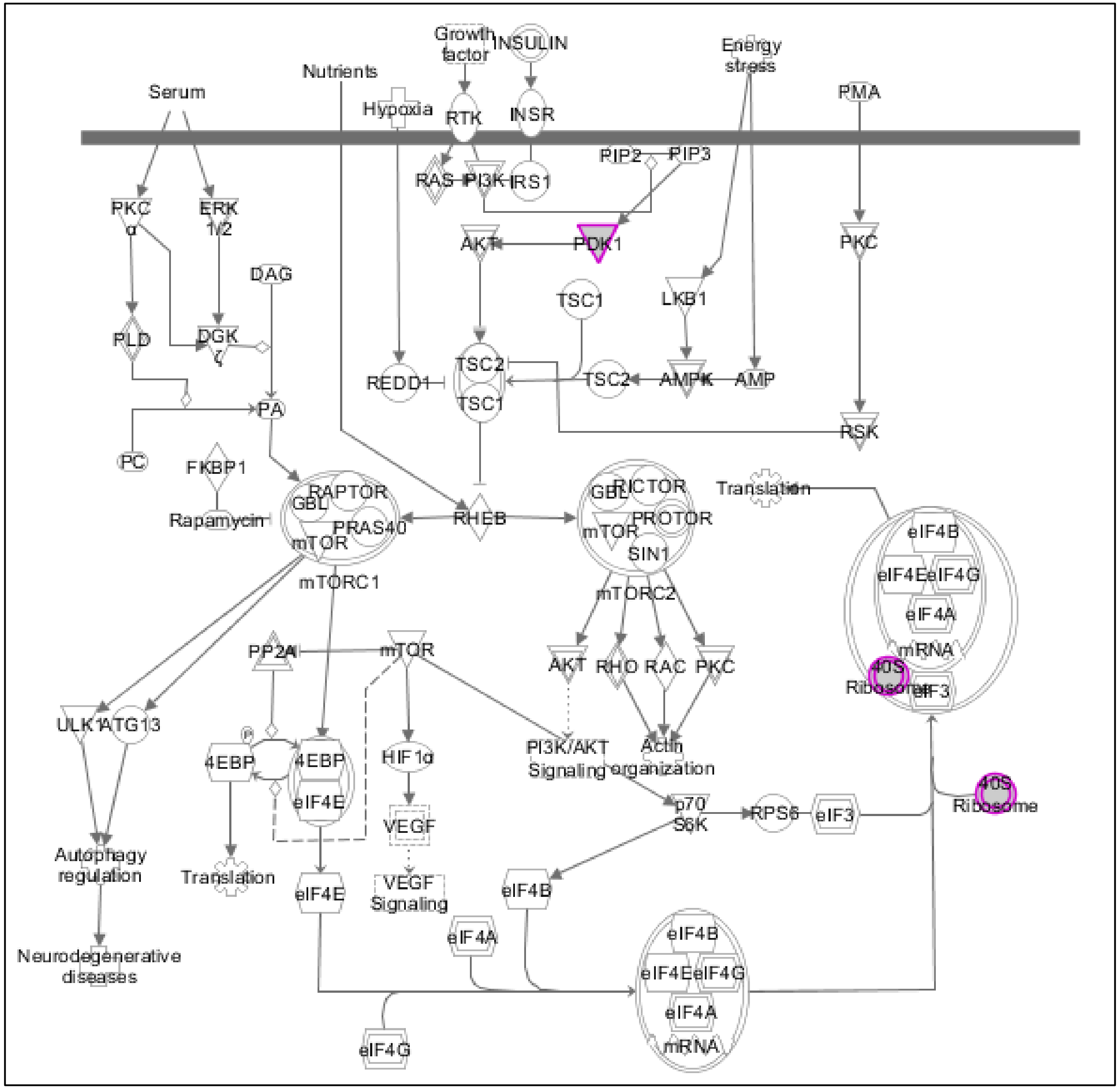

35-Huntington's disease signaling

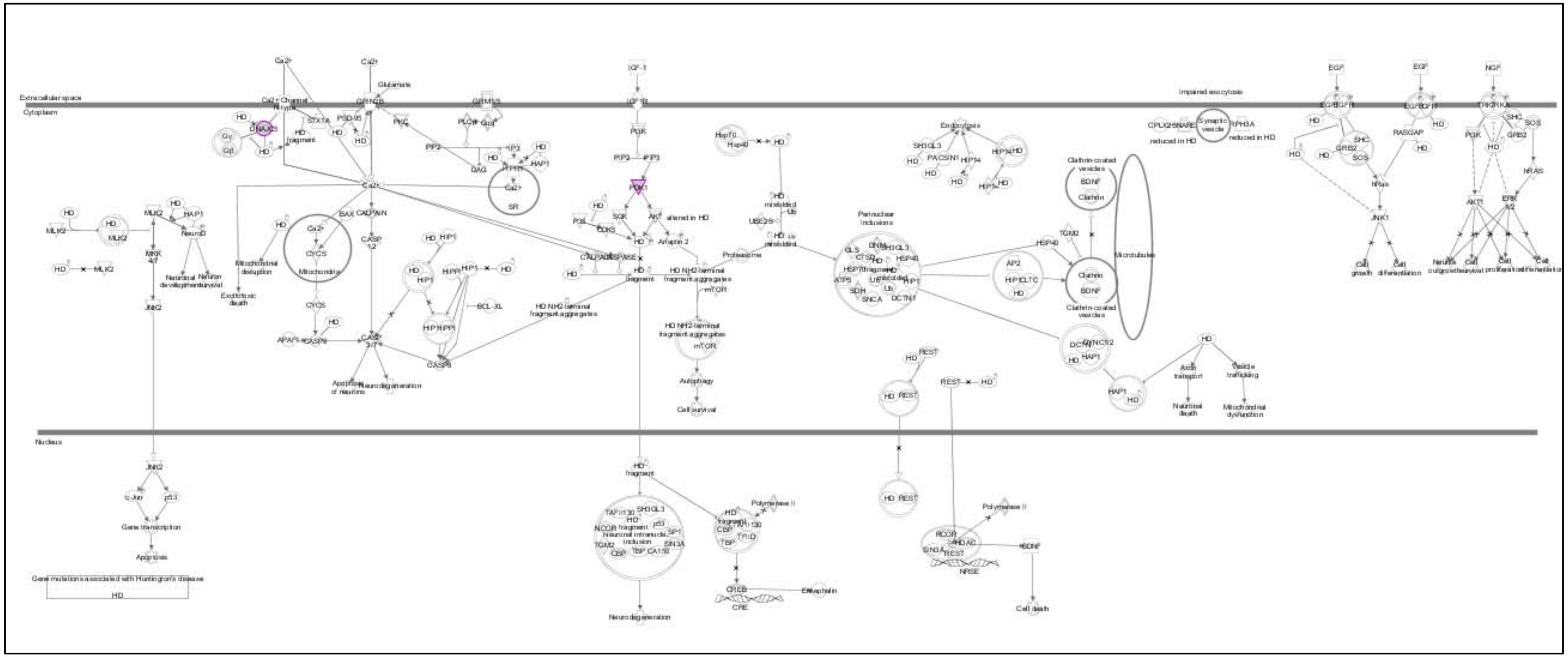

36-Intrinsic prothrombin activation pathway

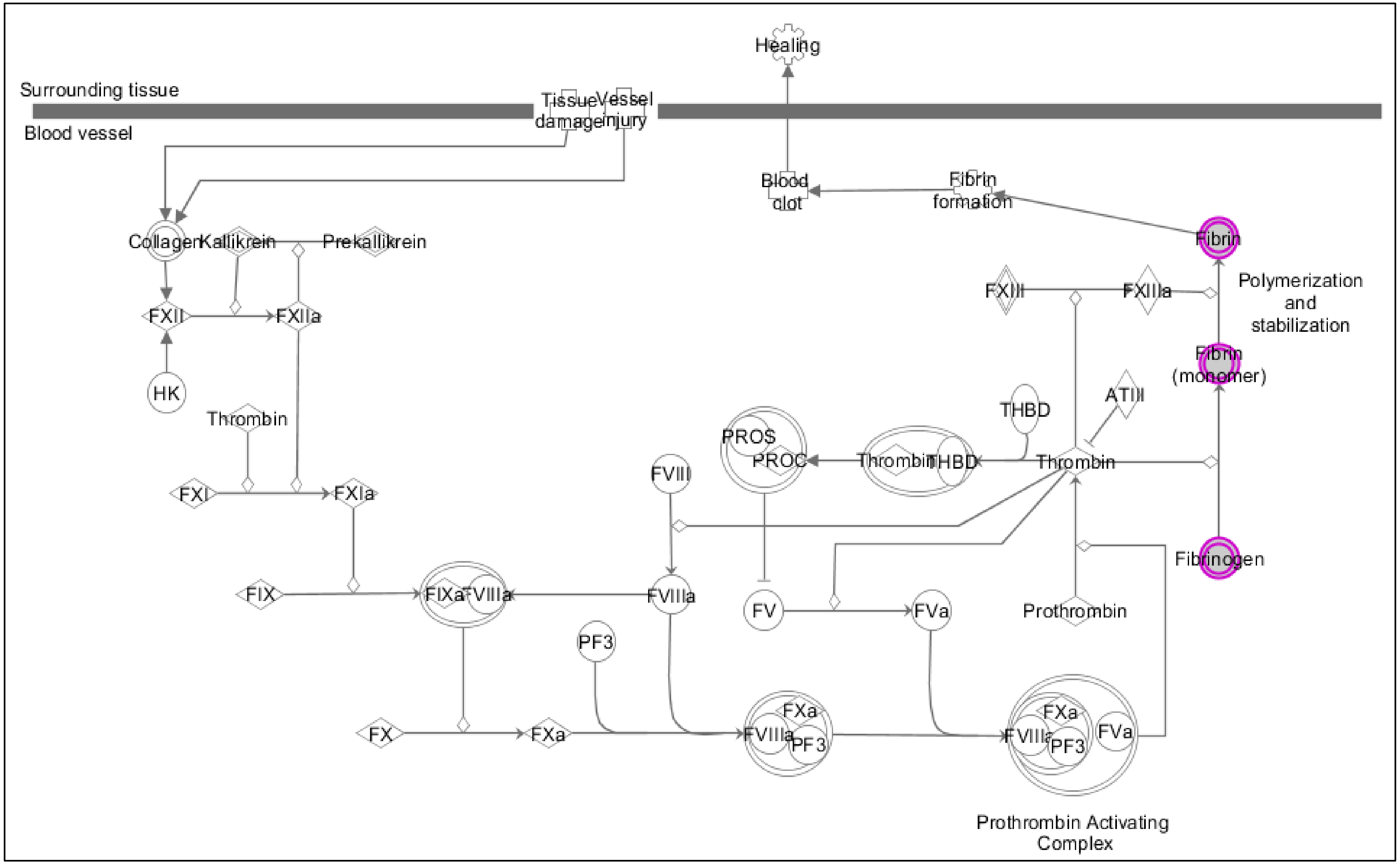

37-Sonic hedgehog signaling

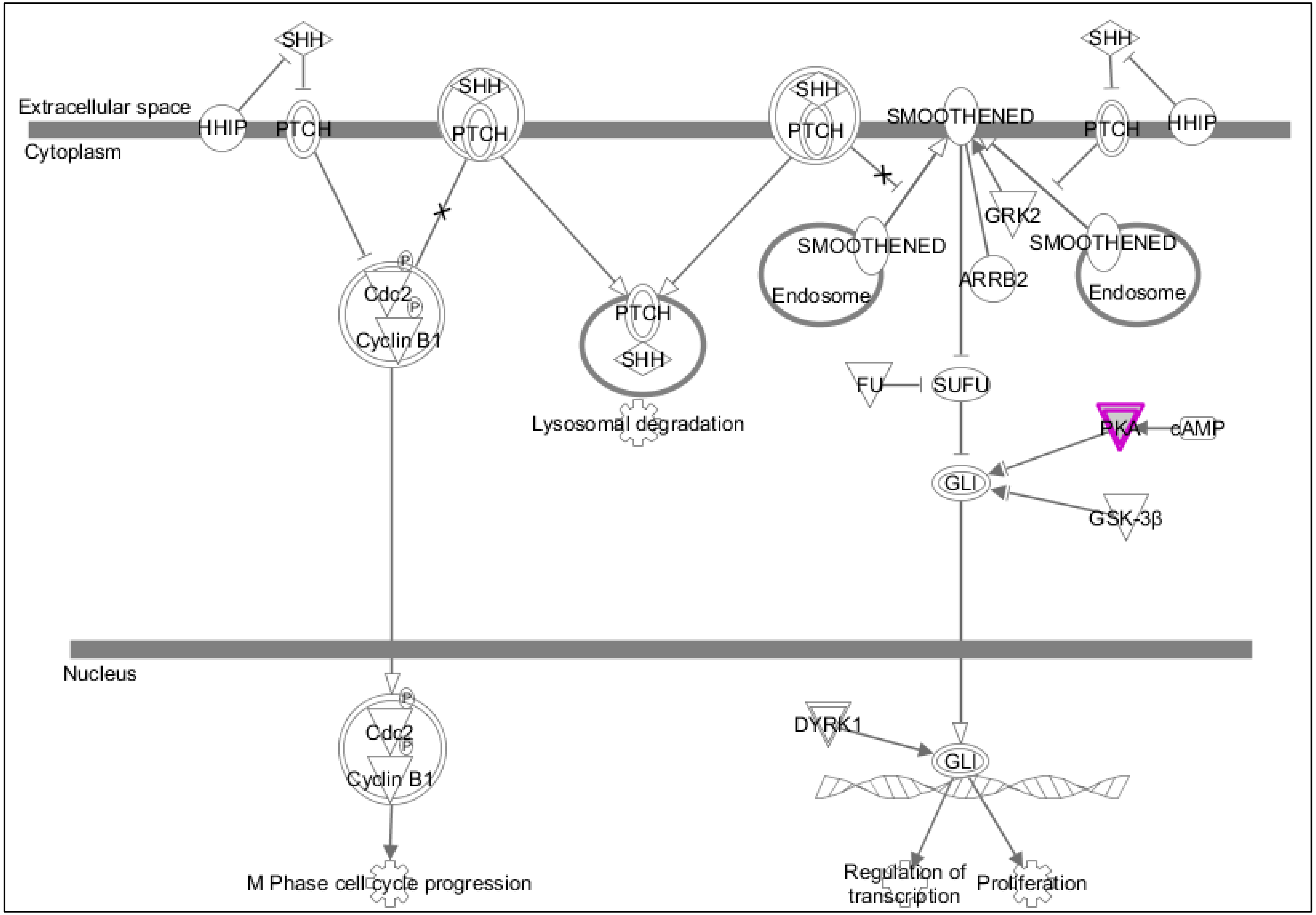

38-Protein ubiquitination pathway

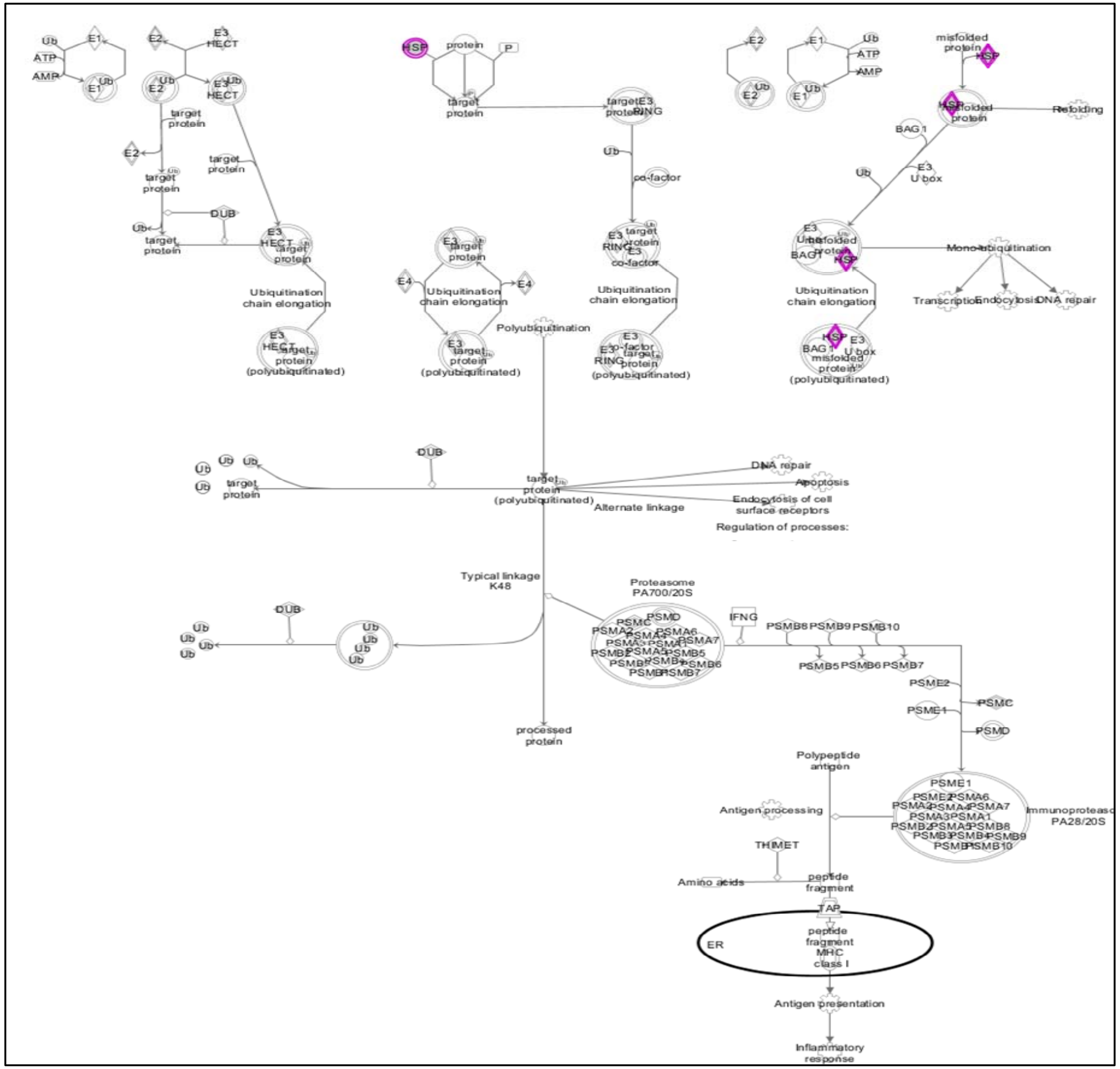

39-Coagulation system

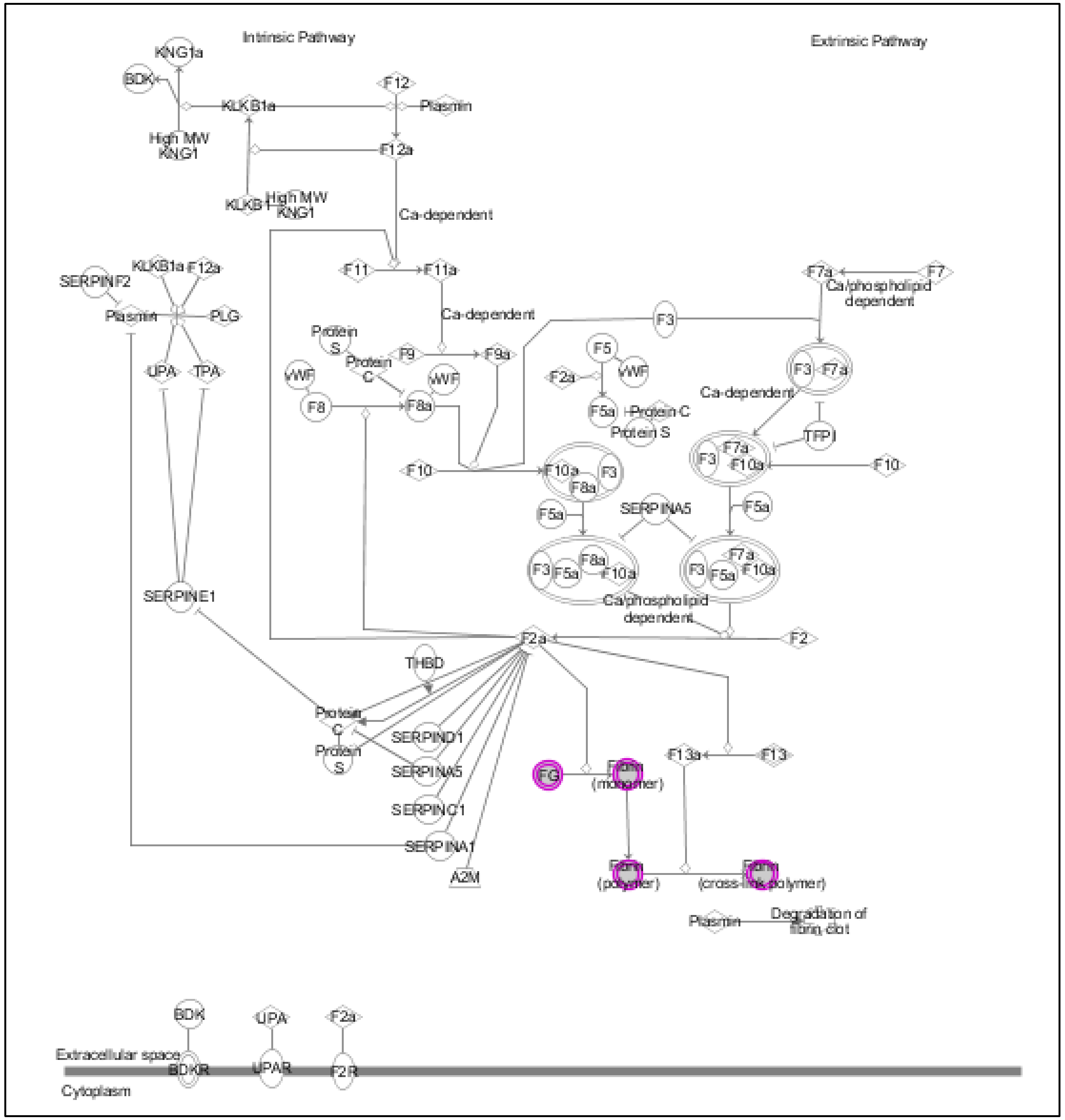

40-Role of JAK2 in hormone-like cytokine signaling

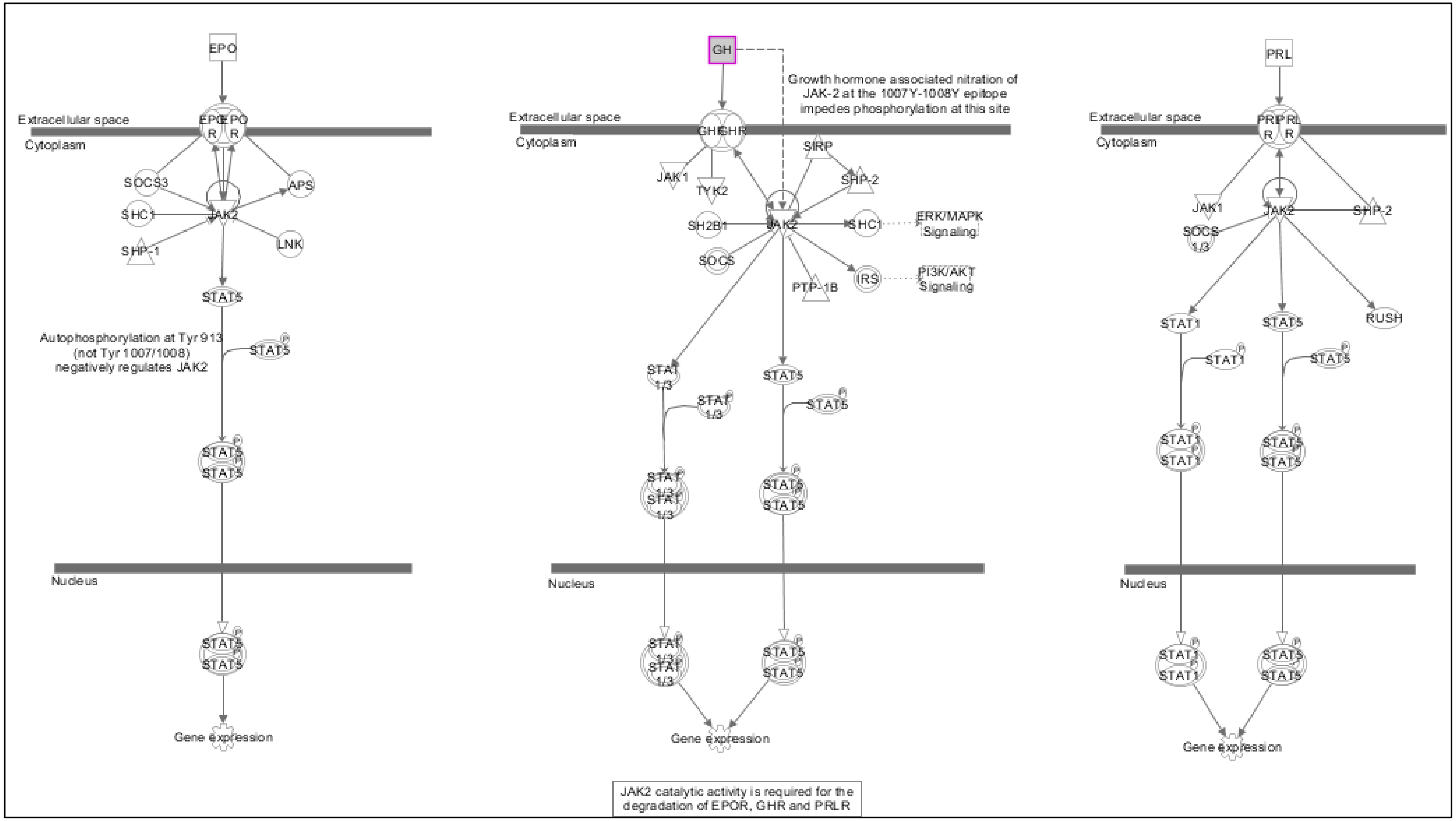

41-G-protein coupled receptor signaling

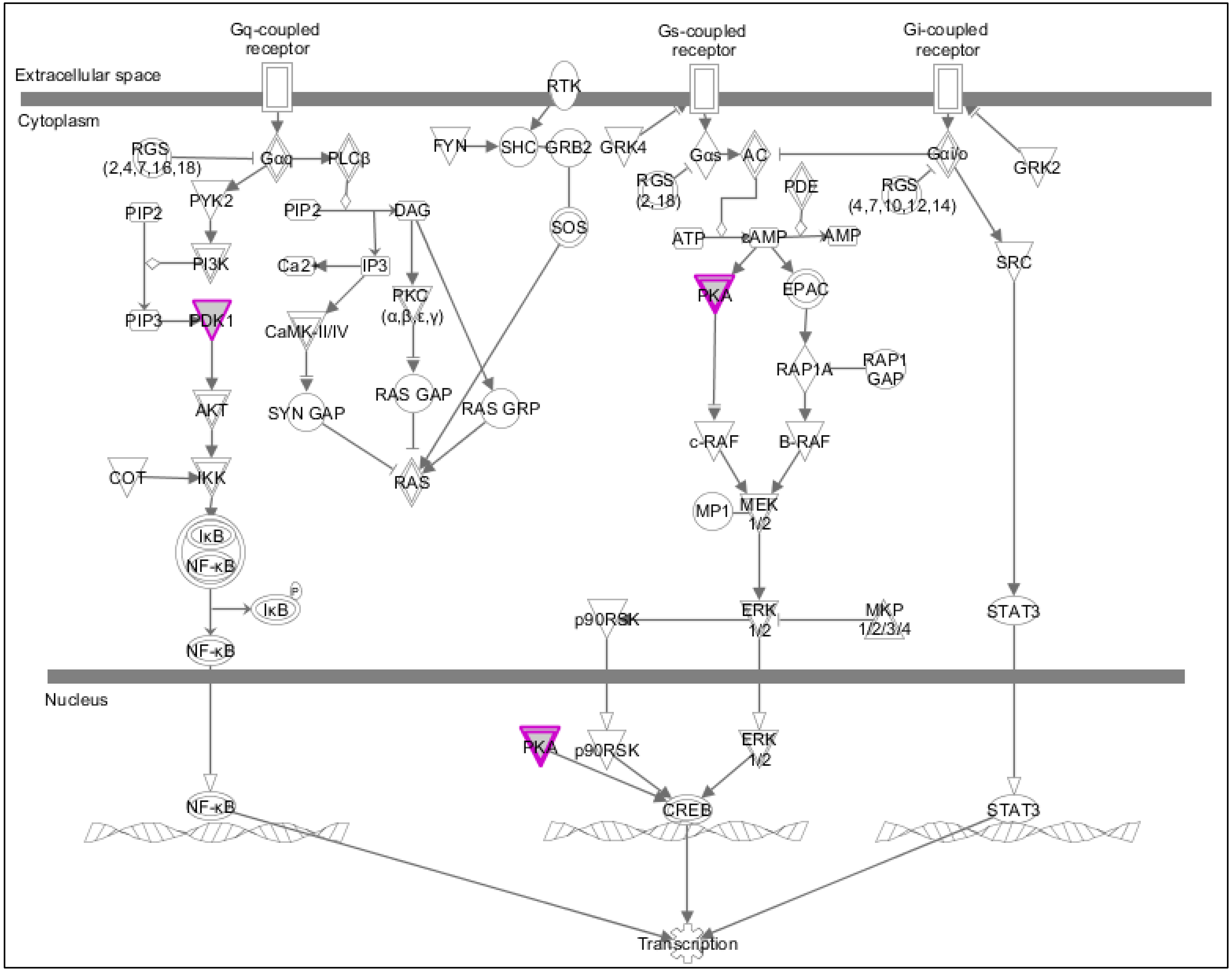

42-Xenobiotic metabolism signaling

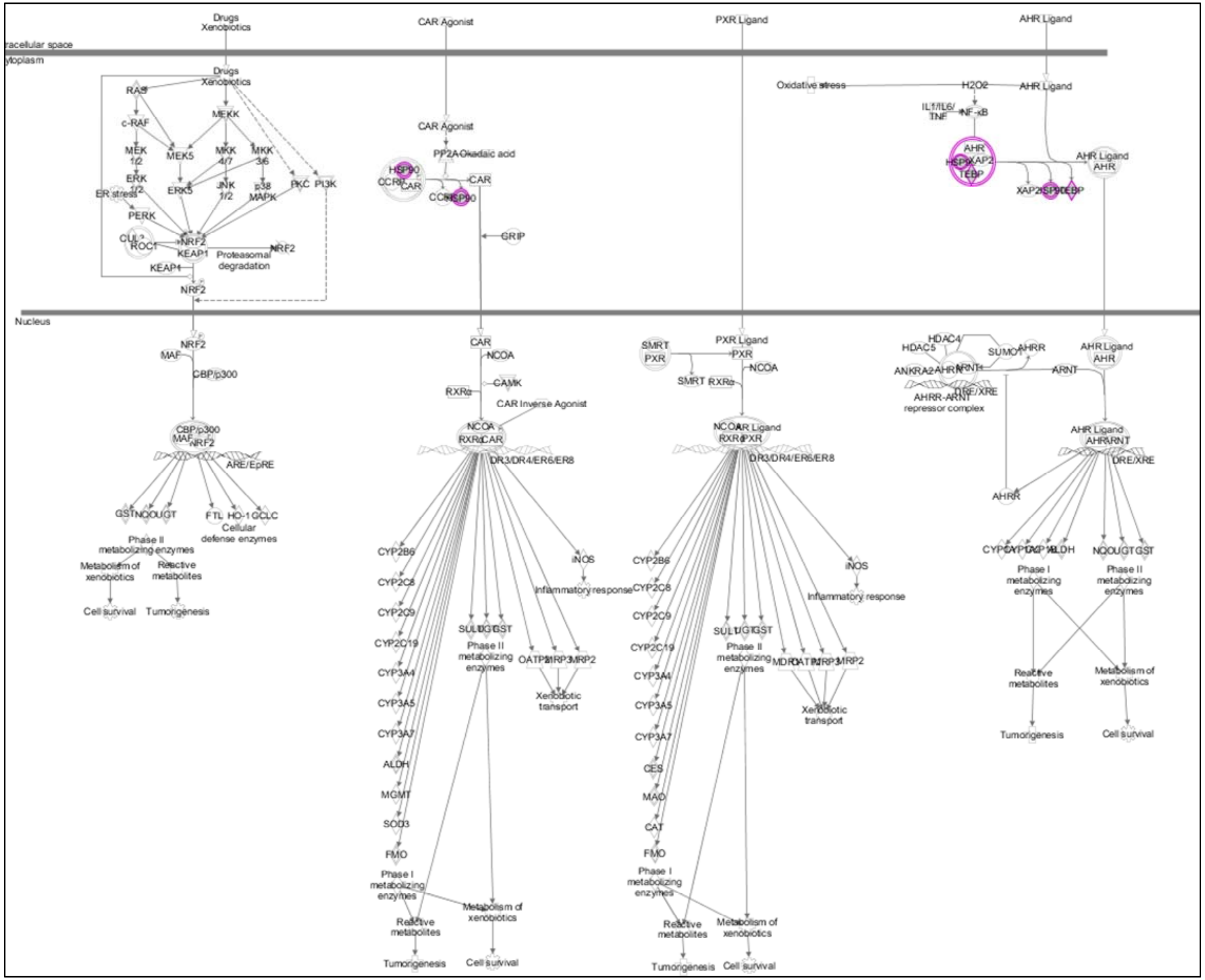

43-Glycolysis I

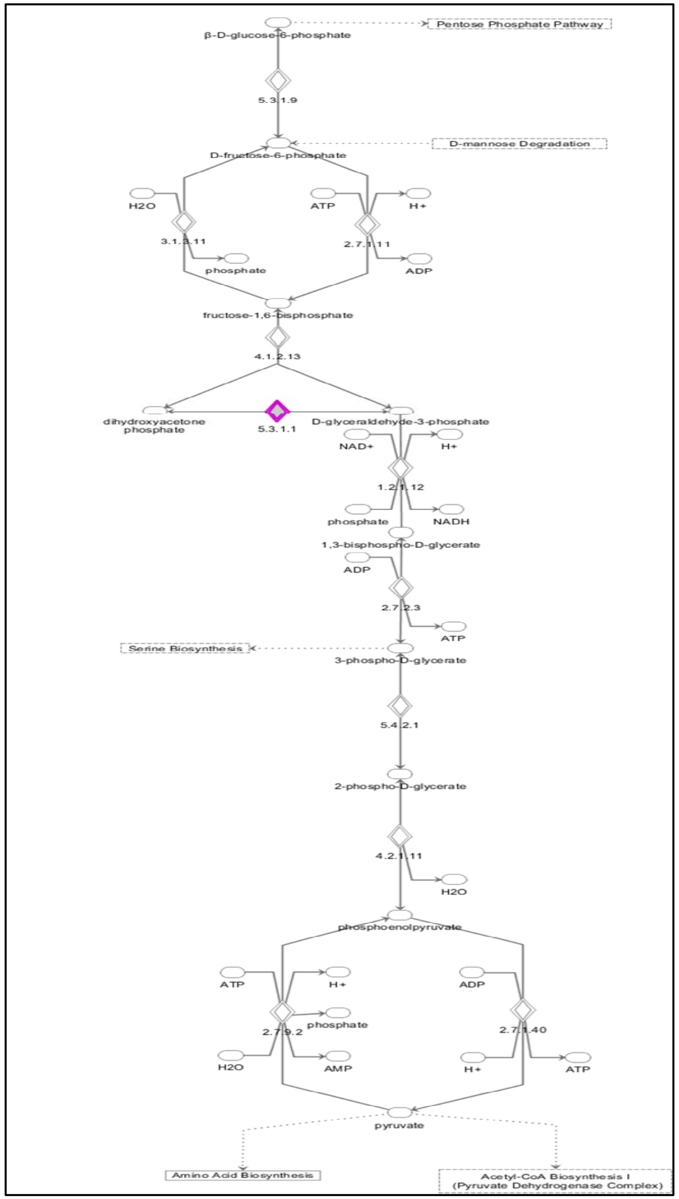

Supplement: Supplementary file 5 [file Presentation_5.zip › Supplemental materials 9.3.pdf]
