# Supplementary material for: Multiomics-Based Signaling Pathway Network Alterations in Human Non-functional Pituitary Adenomas
Source: Front Endocrinol (Lausanne). 2019 Dec 17;10:835. doi: 10.3389/fendo.2019.00835 (PMC6928143; doi:10.3389/fendo.2019.00835)
Supplement: Supplementary file 5 [file Presentation_5.zip › Supplemental materials 8.1.pdf]

Supplemental materials 8.1 Nitroproteins from a pituitary control for IPA analysis (Dataset 8)

ID	Notes	Molecules	Description	Location	Familiy	Drug
O60641		SNAP91	synaptosomal-associated protein, 91kDa	Plasma Membrane	other	
P24071		FCAR	Fc fragment of IgA, receptor for	Plasma Membrane	other	
ACTA2	D	ACTA2	actin, alpha 2, smooth muscle, aorta	Cytoplasm	other	
Q13237		PRKG2	protein kinase, cGMP-dependent, type II	Cytoplasm	kinase	
Q8IWL3		HSCB	HscB mitochondrial iron-sulfur cluster co-chaperone	Cytoplasm	other	
P52823		STC1	stanniocalcin 1	Extracellular Space	kinase	
P25787		PSMA2	proteasome (prosome, macropain) subunit, alpha type, 2	Cytoplasm	peptidase	
Q6TCH7		PAQR3	progestin and adipoQ receptor family member III	Cytoplasm	other	
ACTG2	D	ACTG2	actin, gamma 2, smooth muscle, enteric	Cytoplasm	other	
ACTC1		ACTC1	actin, alpha, cardiac muscle 1	Cytoplasm	enzyme	
